# Re-analysis of single-cell transcriptomics reveals a critical role of macrophage-like smooth muscle cells in advanced atherosclerotic plaque

**DOI:** 10.7150/thno.87201

**Published:** 2024-01-27

**Authors:** Xue Gong, Yunchang Liu, Huiying Liu, Nian Cao, Liping Zeng, Miao Tian, Chunyu Zeng, Yijie Hu, Runjun Zhang, Yundai Chen, Gengze Wu

**Affiliations:** 1 Department of Cardiology, Daping Hospital, The Third Military Medical University (Army Medical University), P.R. China.; 2 Department of Cardiology, the Sixth Medical Centre, Chinese PLA General Hospital, Beijing, P.R. China.; 3 Key Laboratory of Geriatric Cardiovascular and Cerebrovascular Disease Research, Ministry of Education of China; Chongqing Key Laboratory for Hypertension Research, Chongqing Cardiovascular Clinical Research Center, Chongqing Institute of Cardiology, Chongqing, P. R. China.; 4 College of Pulmonary and Critical Care Medicine, The 8th Medical Centre, Chinese PLA General Hospital, Beijing, P.R. China.; 5 Department of Cardiac Surgery, Daping Hospital, The Third Military Medical University (Army Medical University), P.R. China.; 6 Department of Cardiology, No. 926 Hospital, Joint Logistics Support Force of PLA, P.R. China.

**Keywords:** atherosclerosis, single-cell transcriptome, smooth muscle cell, macrophage, phenotypic transition

## Abstract

**Aims:** Smooth muscle cell (SMC) remodeling poses a critical feature in the development and progression of atherosclerosis. Although fate mapping and in silicon approaches have expanded SMC phenotypes in atherosclerosis, it still remains elusive about the contributions of individual SMC phenotypes and molecular dynamics to advanced atherosclerotic plaque.

**Methods:** Using single-cell transcriptome, we investigated cellular compositions of human carotid plaque laden with atherosclerotic core, followed by in vivo experiments utilizing SMC-lineage tracing technology, bulk RNA sequencing (RNA-seq) and both in vivo and in vitro validation of the underlying molecular mechanism.

**Results:** 5 functionally distinct SMC subtypes were uncovered based on transcriptional features (described as contractile, fibroblast-like, osteogenic, synthetic and macrophage-like) within the niche. A proinflammatory, macrophage-like SMC subtype displaying an intermediary phenotype between SMC and macrophage, exhibits prominent potential in destabilizing plaque. At the molecular level, we explored cluster-specific master regulons by algorithm, and identified interferon regulatory factor-8 (IRF8) as a potential stimulator of SMC-to-macrophage transdifferentiation via activating nuclear factor-κB (NF-κB) signaling.

**Conclusions:** Our study illustrates a comprehensive cell atlas and molecular landscape of advanced atherosclerotic lesion, which might renovate current understanding of SMC biology in atherosclerosis.

## Introduction

Atherosclerosis is the major cause of cardiovascular disease (CVD) with rising morbidity and mortality annually and globally 1. Major pathological manifestations of atherosclerosis include arterial wall thickening and plaque buildup, which can turn into blockage eventually. Morphological features of atherosclerotic plaque vary heavily on its contents, involving cellularity, extracellular matrix (ECM), collagen fibrils and inflammation infiltration. The interplay of these factors associates with microenvironment remodeling within the atherosclerotic niche, shaping plaque vulnerability and deciding clinical outcomes 2.

Smooth muscle cells (SMCs), the dominant vascular cell type, provide mechanical support and vasoactive responses in vascular homeostasis, whose impairment leads to various vasculopathies 3-5. Contractile SMCs underlie cellular basis of vascular biology in contrast to remodeled SMCs with declining intrinsic properties and altered cellular behavior (e.g., proliferation, migration and differentiation). From the molecular perspective, characteristic gene profile shift is recognized from contractile genes like α-smooth muscle actin 2 (ACTA2), transgelin (TAGLN) and smooth muscle myosin heavy chain (MYH) to ECM or inflammatory genes, generating synthetic and proinflammatory phenotypes, respectively 6. Apart from these most investigated phenotypes during atherogenesis, recent single-cell transcriptomics together with fate mapping methods have further expanded SMC phenotypes by taking advantage of refining transcriptional feature at high resolution, extending SMCs from contractile, synthetic, fibroblast, proinflammatory and osteogenic phenotypes to subtle types 7. For another, however, these findings complicate the understanding of atherogenesis. It still remains controversial over whether the leading atheroprone role is played by fibroblast-like, osteogenic, intermediary or other clusters in human atherosclerosis 8, 9.

To solve these uncertainties, we referred to publicly available single-cell transcriptome data of human advanced carotid plaque, and identified 5 functionally distinct SMC subtypes from the atherosclerotic core. External validation distinguished that macrophage-like SMCs were a prominent cellular feature of unstable plaque. Via prediction and verification, we investigated cluster-specific molecular dynamics during atherosclerotic remodeling and highlighted interferon regulatory factor-8 (IRF8) as a stimulator for SMC acquiring macrophage-like profile upon remodeling. Further functional enrichment analysis, high throughput transcriptome sequencing and experimental validation revealed that IRF8 activated nuclear factor-κB (NF-κB) signaling in ox-LDL stimulated SMC transdifferentiation.

## Methods

### Data collection

For single-cell RNA sequencing (scRNA-seq) data using Chromium instrument (10x Genomics), carotid samples from 3 patients undergoing carotid endarterectomy were dissected into atherosclerotic core and proximal adjacent portions. Raw data were downloaded from GSE159677 in the Gene Expression Omnibus (GEO) database. ScRNA-seq data of non-atherosclerotic arteries were derived from 6 normal human ascending aorta (AA) samples (GSE216860) and 3 AA samples of control group (GSE213740). Additional scRNA-seq data of atherosclerotic arteries were obtained from carotid (GSE155514) and right coronary arteries (GSE131778). Bulk transcriptome data using RNA sequencing or microarray were derived from human unstable atherosclerotic plaques and deposited in GSE28829, GSE120521 and GSE163154.

### Data processing, clustering and annotation

Following the Seurat (version 4.2) pipeline in the R environment, each sample was filtered out the cells containing > 5% mitochondrial genes and those with gene counts < 200 or > 6,000. Top 2,000 variable genes and scaled data were obtained by the *FindVariableFeatures* and *ScaleData* functions, respectively. Data integration was performed based on computed anchor gene set, and principal components (PCs) were calculated on previously identified variable genes using the *RunPCA* function. A shared nearest neighbor (SNN) graph was constructed with the *FindNeighbors* function using the top 30 PCs, followed by identifying clusters using the *Findclusters* function with a resolution parameter of 0.6. The first 10 PCs were selected for dimension reduction using the Uniform Manifold Approximation and Projection (UMAP) algorithm. Cluster-specific marker genes were identified with the *FindAllMarkers* function setting log-fold threshold of 0.25 (avg_log2FC > 0.25), and at least 25% of cells expressing the detected gene in the target cluster (pct.1 > 25%). Meanwhile, the percentage difference of gene-expressing cells in the target cluster compared to others should be over 20% (pct.1-pct.2 > 20%). Adjusted p-value < 0.05 were considered statistically significant. Finally, major cell types of arterial wall were manually annotated according to the canonical cluster-defining genes. AA and additional atherosclerotic scRNA-seq datasets were analyzed using the *Findclusters* function with modified resolution parameters of 0.3 and 0.5, respectively.

SMC clusters identified in alternative atherosclerotic scRNA-seq datasets were scored using the *AddModuleScore* function by calculating the average expression of the top 100 cluster-specific genes of SMC1-5 subtypes. An equivalent number of randomly selected genes were set as control. Visualization of scores was overlaid in UMAP using the *Nebulosa* package (version 1.10).

### Human coronary artery sample collection

Human coronary arteries were collected from 2 male patients undergoing coronary artery bypass grafting surgery. Informed consents were obtained and the study was approved by the Third Military Medical University Ethics Board. Experiments were performed in accordance with the principles of Declaration of Helsinki. Briefly, left anterior descending coronary arteries were excised, removed of perivascular connective tissues in cold phosphate buffer solution (PBS) and separated of atherosclerotic lesions and adjacent tissues. All samples were fixed in formalin, dehydrated and embedded in paraffin before section (4 μm).

### Animal research

Animal studies were approved by the Animal Care and Use Committees of the Third Military Medical University and performed following the guidelines for animal care and use of the Third Military Medical University. 3-month-old Apolipoprotein E-null (ApoE-/-) mice on a C57BL/6J background were purchased from Vital River (Beijing, China). SMC-lineage tracing mice (Myh11-CreERT2;tdTomato;ApoE-/-) were obtained by crossing Myh11-CreERT2 (Cyagen Co., Guangzhou, Beijing), B-CAG-Upar-tdTomato (Biocytogen Co., Beijing, China) and ApoE-/- mice. Myh11-CreERT2 (Y-linked) transgene served as an indicator of SMC-specific expression. For lineage labeling, male transgenic mice have undergone Cre recombinase activation by intraperitoneal injection of tamoxifen (75mg/kg, Sigma) for 5 consecutive days at the age of 2 months. Adeno-associated virus (AAV) 8 targeting IRF8 (shRNA: 5'-GCCATATAAAGTTTACCGAAT-3') was constructed based on the pHBAAV-SM22A vector (HANBIO, Beijing, China) and injected into vein tail at the age of 3 months. The above mice were either treated with high-fat diet (HFD, 40%Kcal from fat containing 1.25% cholesterol) or regular chow diet for 3 months. All mice were randomly allocated into experimental groups.

### Statistics

Cluster-specific marker genes were identified using Wilcoxon rank-sum test implemented in the *FindAllMarkers* function of Seurat package with adjusted P value < 0.05 and avglog2FC > 0.25. Differentially expressed genes (DEGs) between groups were explored by the *FindMarkers* function using embedded Wilcoxon rank-sum test with adjusted p-value < 0.05 and |avglog2FC| > 0.15. Statistical differences were compared using two-tailed *t* test between two groups and one-way ANOVA within three or more groups with Dunnett's T3 post test. GraphPad Prism (version 8.0) was used to perform statistical analyses of in vitro experiments. P value < 0.05 was considered statistically significant.

An Expanded Methods section is available in the Supplemental Material.

## Results

### The landscape of cellular composition of human carotid atherosclerosis

**Figure 1:**
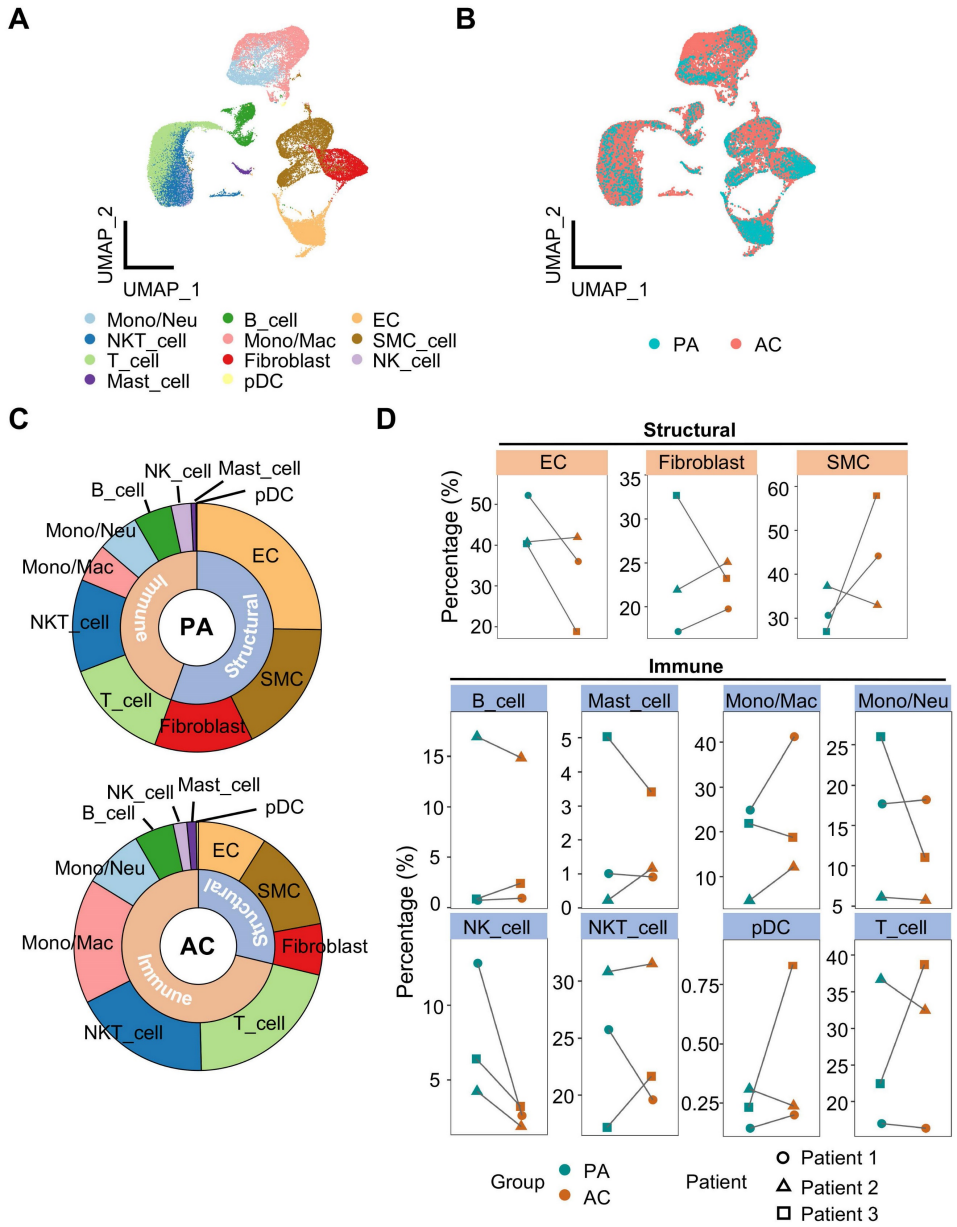
** Overview of calcified atherosclerotic niche in human carotid arteries.** (A-B) Uniform manifold approximation and projection (UMAP) of single-cell RNA sequencing (scRNA-seq) data of human carotid arteries exhibiting cell types (A) and groups (B). PA, proximal adjacent tissue. AC, atherosclerotic core. (C) Sunburst plot of cellular composition in PA and AC. (D) Proportions of cell types against structural or immune cells between PA (green) and AC (red). SMC, smooth muscle cell. Mono/Neu, monocyte/neutrocyte. Mono/Mac, monocyte/macrophage. EC, endothelial cell. pDC, plasmacytoid DC.

To gain insight into the cellularity during atherosclerotic remodeling, we analyzed the publicly available scRNA-seq data of human carotid arteries. A total of 51,720 cells were contained in the dataset from 3 patients undergoing endarterectomies. For each specimen, atherosclerotic core (39,225 cells) and proximal adjacent tissue (12,477 cells) were separated for sequencing. Following the Seurat platform workflow including quality control, dimension reduction, clustering and manual annotation, 11 cell types were identified consequently (Figure 1A, S1A-S1C and Table S1). Batch effect was eliminated as attested by overlapped samples after integration (Figure 1A, S1D). Discrepancies of cellularity were evident between groups and among individuals (Figure 1B, S1D). Then, we calculated relative cellular abundance by classifying the resulting cell types into structural or immune subsets, and revealed increased proportion of immune subset in contrast to decreased structural fraction in the lesion (Figure 1C, S1E). By pairwise comparison, however, an inconsistent shift in cellular abundance between PA and AC group was observed in within-group samples for most cell types, suggesting heterogenous cellularity of plaque lesions. In light of this, we focused on the trends displayed by at least 2 within-group samples, and found that SMC and Mono/Mac, both pivotal players in atherosclerosis, were differentially deposited (percentage difference ≥10% in at least 2 paired samples) in the atherosclerotic core compared to adjacent control (Figure 1D). Moreover, active ligand-receptor interactions were exhibited between SMC and other structural cells, and this complicated intercellular communication network suggested a pivotal role of SMC in vascular biology (Figure S1F).

### SMC exhibits phenotypic variety in human atherosclerosis

**Figure 2:**
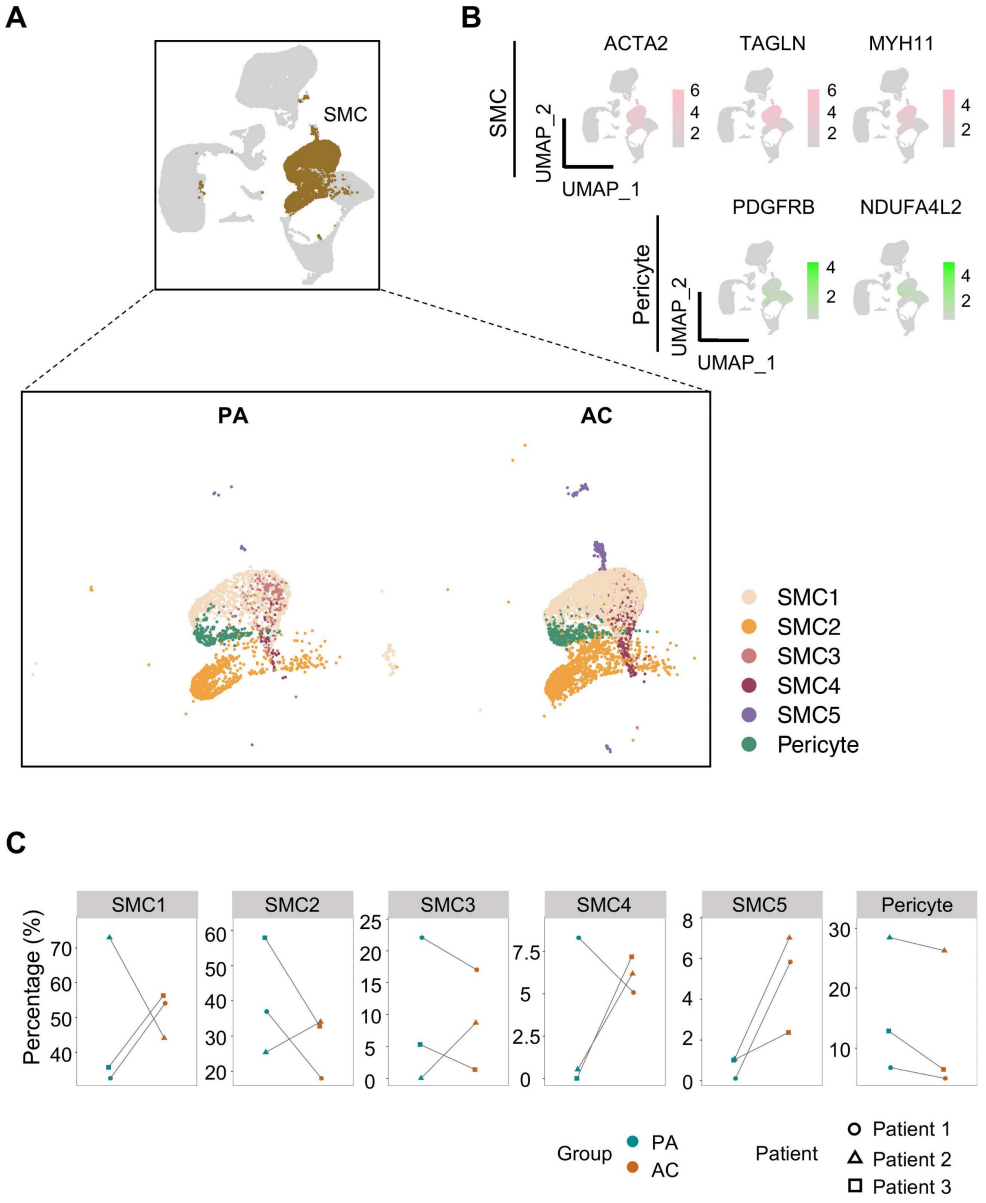
** Reclustering ACTA2-positive cells identifies SMC subtypes and pericyte.** (A) Uniform manifold approximation and projection (UMAP) presentation of human carotid single-cell RNA sequencing (scRNA-seq) data highlighting smooth muscle cells (SMCs, brown, upper panel) and subdivided pericyte and SMC clusters (lower panel). (B) Expression of SMC (ACTA2, TAGLN, MYH11) and pericyte (PDGFRB, NDUFA4L2) markers. Color darkness indicates average gene expression. (C) Proportions of SMC subtypes and pericyte in proximal adjacent tissue (PA) and atherosclerotic core (AC).

Since transcriptomic perturbations trigger cellular identity shift 10, we focused on SMC, whose phenotypic heterogeneity intimately associates with atherogenesis, for closer examination. According to the discrepancies of cluster-specific expression profiles, 5 SMC subtypes and pericyte were distinguished consequently (Figure 2A, Table S2). As reported, pericyte, featured by PDGFRB and NDUFA4L2 expression, is a rare vascular cell type bearing highly transcriptomic resemblance to SMC 11 (Figure 2B, S2A). By comparison, SMC1 and SMC2 constituted the bulk of SMCs. And in contrast to the conflicting shifts in cellular abundance for SMC1-4 between PA and AC group discerned by pairwise comparison, SMC5, though unprivileged in proportion, underwent consistent and marked expansion within the atherosclerotic lesion (Figure 2C, S2B-S2C).

**Figure 3:**
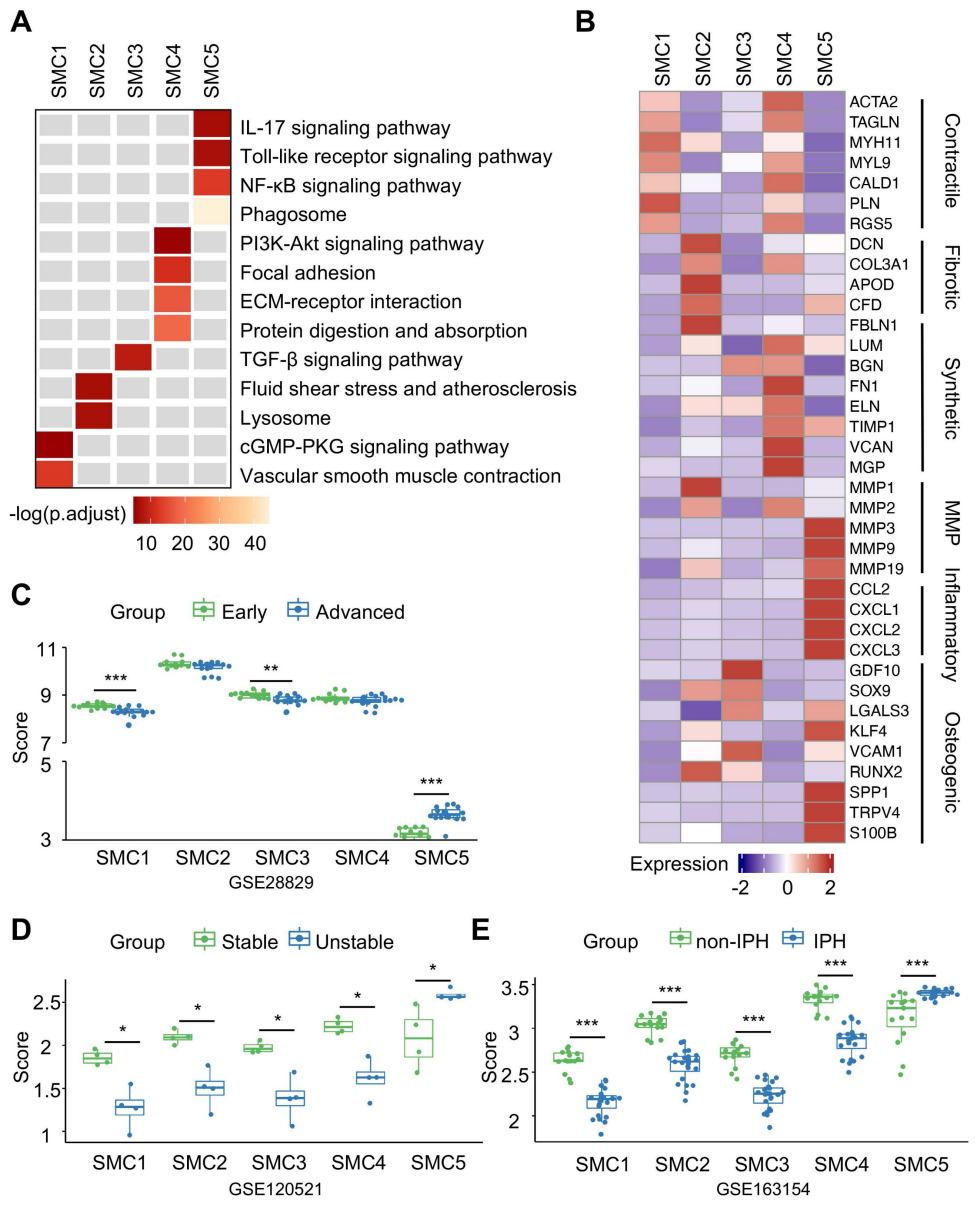
** Characterization of smooth muscle cells (SMCs) reveals functional variety within clusters.** (A) Kyoto Encyclopedia of Genes and Genomes (KEGG) analysis of SMC subtypes using cluster-specific upregulated genes. (B) Heatmap showing average expression of functional genes in each SMC subtype. (C-E) The signature scores of each SMC subtype in bulk transcriptomes of advanced (C), unstable (D) and intraplaque hemorrhaged (IPH, E) plaque. Cluster-specific activated genes underlie scoring set. *P<0.05, **P<0.01, ***P<0.001. P.ajust, adjusted P value.

To discern phenotypic disparities of SMC subtypes, we performed KEGG analysis of DEGs presented in each cluster, and revealed cluster-specific activated signaling (Figure 3A). This result was also in consistent with characteristic gene profiles, defining 5 SMC subtypes by functional annotation: SMC1 represented the original, biological type of SMC for enriched contractile function and genes. SMC2 exhibited dual properties of SMC1 and fibroblast, as suggested by molecular characteristics and its close proximity to both cell types in the UMAP projection. SMC3 and SMC4 were enriched in osteogenic and synthetic genes, respectively. And SMC5 displayed a proinflammatory signature featured by phagosome-involved function and inflammation-related signaling (Figure 3B, S2D). Accordingly, we described SMC1-5 subtypes as contractile, fibroblast-like, osteogenic, synthetic and macrophage-like. Likewise, intercellular communications among them were active as evidenced by ligand-receptor interactions (Figure S3A-S3B). Given the genomic disparities between atherosclerotic and non-atherosclerotic arteries, we further investigated SMC phenotypic heterogeneity in normal ascending aortae. SMC clustering resulted in 4 SMC clusters retaining functional signatures of SMC 1, 2 and 4 subtypes as revealed by KEGG analysis (Figure S4, Table S3-S6). This finding implied that SMC has undergone comprehensive dynamic transcriptional reprogramming upon atherosclerotic remodeling.

In the next step, we aimed to measure pathogenic contributions by each SMC subtype to advanced atherosclerosis using 3 sets of bulk transcriptomes derived from human unstable plaques (Figure S5). Aided by ssGSEA, we extracted cluster-specific feature genes from single transcriptome data as scoring reference. In different sets of bulk transcriptomes of vulnerable plaques, however, we found SMC5-defining scores increased significantly and consistently in advanced, unstable and intraplaque haemorrhage featured groups (Figure 3C-3E). Taken together, these findings suggested that SMC5 might pose the leading pathogenic potential in aggravating plaque vulnerability.

To further evaluate the conservation of SMC phenotypes in different atherosclerotic conditions, we extended SMC1-5 subtype features to SMC clusters identified in additional atherosclerotic scRNA-seq datasets. Specifically, signature scores of SMC1-5 subtypes were calculated using top 100 cluster-specific makers and we found the presence of all SMC subtypes in both carotid and coronary plaques (Figure S6, Table S7).

### Transcriptional features underlie SMC phenotypic variety

**Figure 4:**
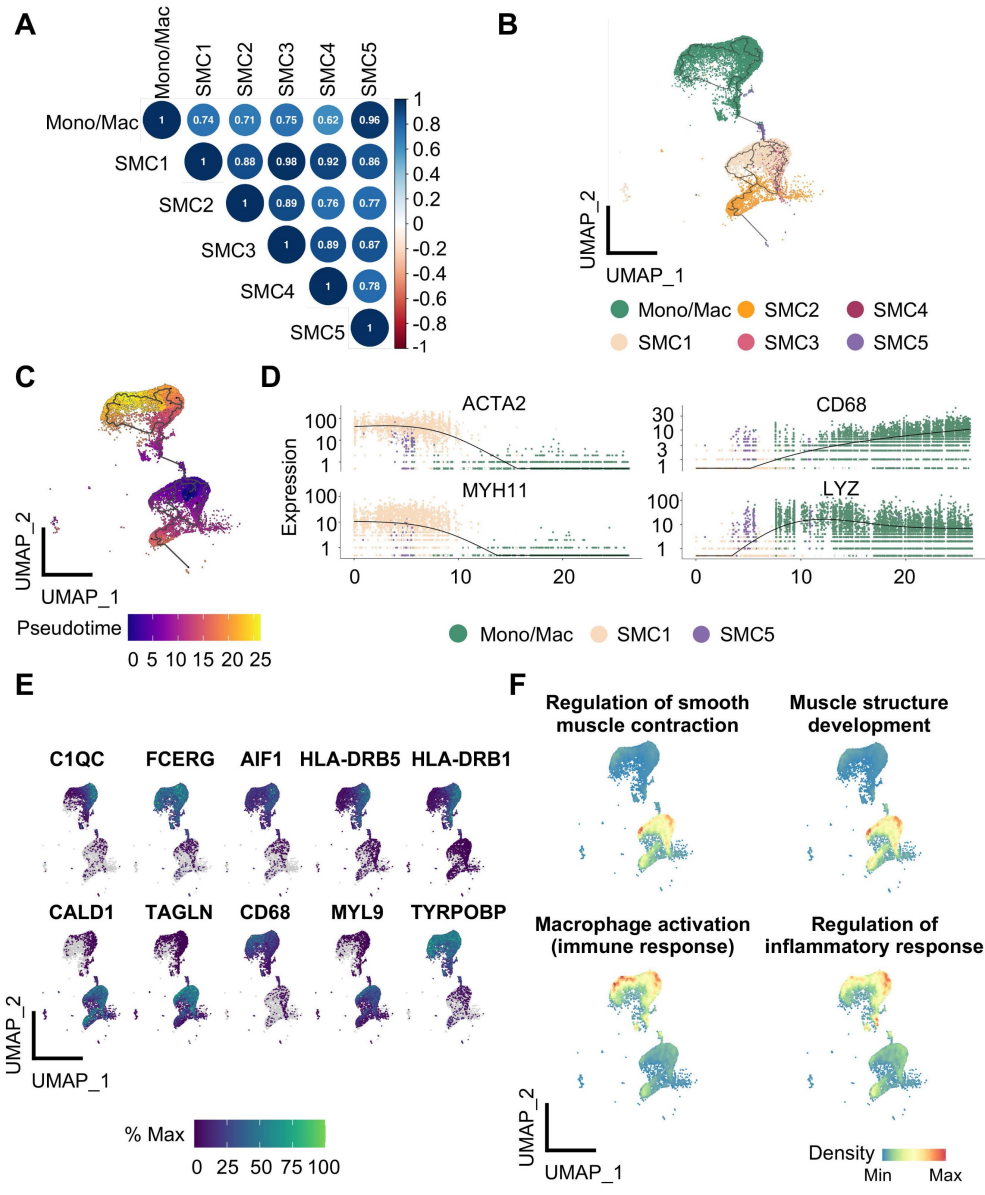
** Identification of the differentiation trajectory from smooth muscle cell (SMC) to macrophage in human carotid arteries.** (A) Correlation plot of SMC subtypes and monocyte/macrophage (Mono/Mac) calculated by top 1000 highly variable genes. (B) Trajectory inference between SMC and Mono/Mac predicted by Monocle 3. (C) Pseudotime projections from SMC1 to Mono/Mac. (D) Transcription of SMC (ACTA2, MYH11) and Mono/Mac (CD68, LYZ) markers ordered by pseudotime trajectory. (E) Expression of top 10 track genes. Color lightness indicates expression abundance. (F) Enrichment analysis of selected biological processes based on top 50 track genes calculated by UCell. Density indicates enrichment level.

Given prominent significance of SMC5 in atherosclerosis progression, we put focus on it for further exploration. We first noticed a remarkable similarity of highly variable gene profiles between SMC5 and Mono/Mac, suggesting their intimate molecular correlation (Figure 4A). Using the unbiased trajectory inference and pseudotime calculation, we identified a potential differentiation trajectory with SMC1 on the one end and Mono/Mac on the other leaving SMC5 located in the middle (Figure 4B). Pseudotime scores of marker genes (ACTA2 and MYH11 for SMC, CD68 and LYZ for Mono/Mac) and top track genes both distinguished dual molecular characteristics captured by SMC5 (Figure 4C, 4E). Similarly, skewed functional associations with SMC and macrophage were implicated in SMC5 as attested by functional enrichment of significant track genes (Figure 4F, Table S8).

**Figure 5:**
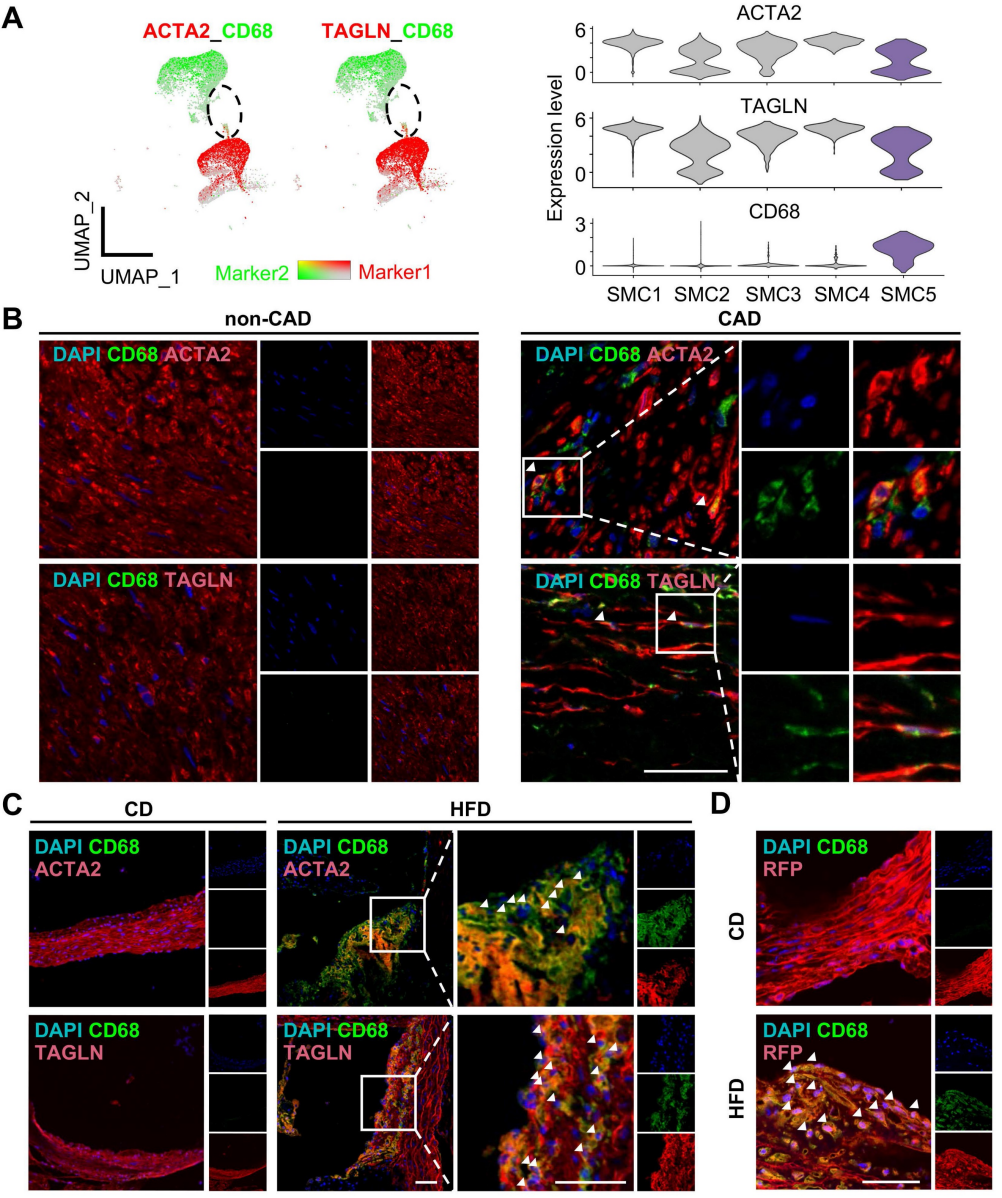
** Identification of macrophage-like smooth muscle cell (SMC) in atherosclerosis.** (A) Expression of markers ACTA2 or TAGLN (red) and CD68 (green) in SMC and monocyte/macrophage (Mono/Mac) clusters visualized in Uniform Manifold Approximation and Projection (UMAP, left panel) and violin plot (right panel). Color darkness indicates average gene expression. (B) Immunofluorescent staining of ACTA2 or TAGLN (red) and CD68 (green) in human left coronary arteries with or without coronary artery disease (CAD). DAPI, blue. Scale bar, 50 µm. (C) Immunofluorescent staining of ACTA2 or TAGLN (red) and CD68 (green) in aortae of ApoE-/- mice following 3-month high fat diet (HFD) treatment. Chow diet (CD), control. Scale bar, 100 µm. (D) Immunofluorescent staining of RFP (Red) and CD68 (green) in aortae of Myh11-CreERT2;tdTomato;ApoE-/- mice as treated in (C). Scale bar, 50 µm. The white arrows showed CD68 positive SMC (SMC5).

Given SMC5 co-expressed SMC and macrophage marker genes, we conducted multi-immunofluorescence in human coronary artery and mouse aortic or carotid sections to testify SMC5 in vivo (Figure 5A-D, Figure S7). Expectedly, SMC5 identified by cells expressing both ACTA2 (or TAGLN) and CD68 were evident in the atherosclerotic plaque (Figure 5B-C, Figure S7). Given the loss of SMC lineage markers during atherosclerotic remodeling confused SMC identity, we further took advantage of a SMC-lineage tracing model (Myh11-CreERT2;tdTomato;ApoE-/-) to definitely track SMC5 origin. Tamoxifen induction activated SMC-specific recombination, leading to stable and robust red fluorescent protein expression. Following a 3-month pro-atherosclerotic high fat diet treatment, CD68+ and tdTomato+ SMCs were heavily deposited within the aortic atherosclerotic plaque compared to the regular chow diet fed control group without showing visible plaque (Figure 5D). These findings indicated that SMC could transdifferentiate towards macrophage under atherosclerotic conditions.

**Figure 6:**
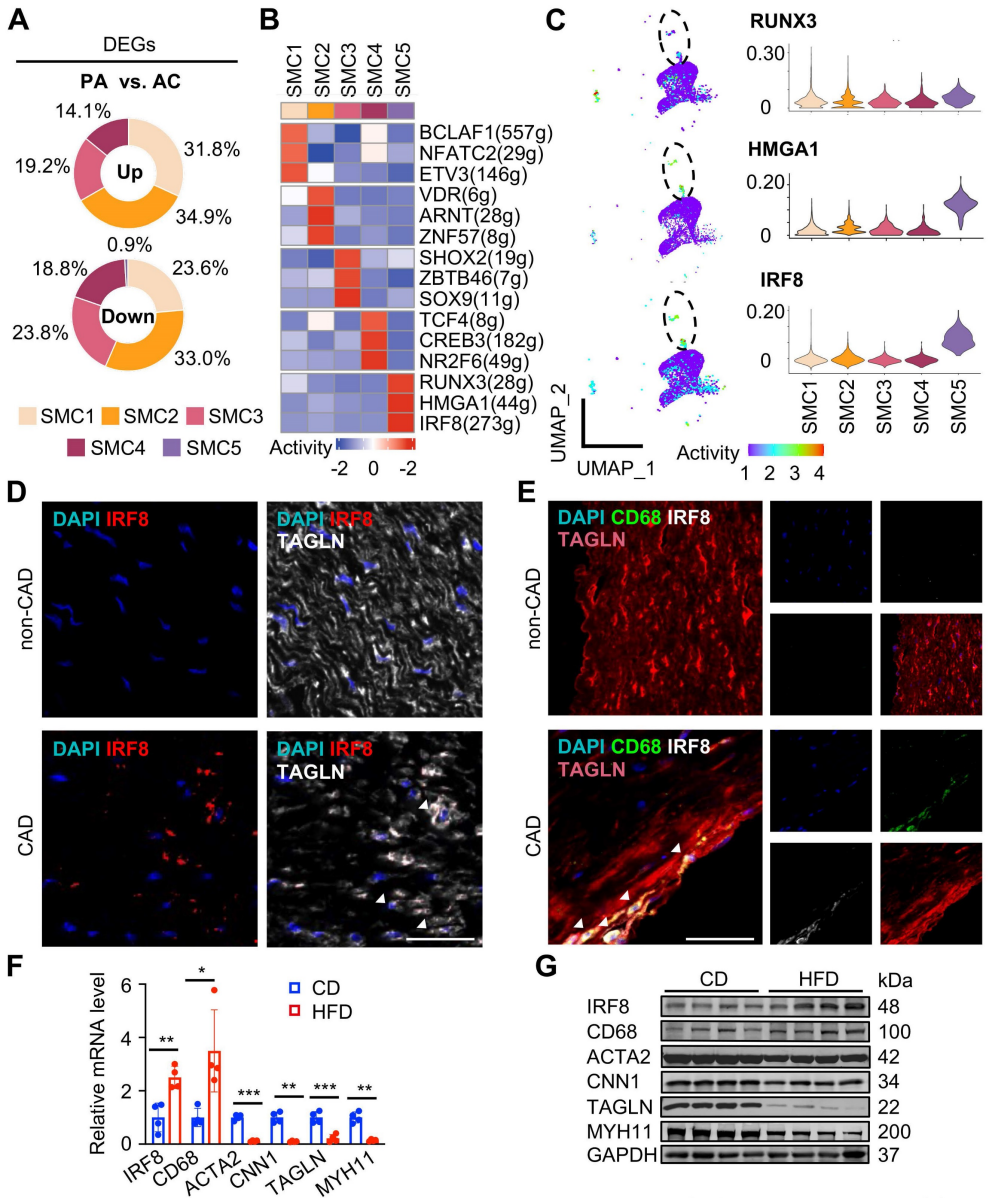
** IRF8 activation is a transcriptional feature in macrophage-like SMC during remodeling.** (A) Proportions of up- and down-regulated differentially expressed genes (DEGs) in each SMC subtype upon atherosclerotic remodeling. DEGs are defined with adjusted P value < 0.05 and |log2(fold change)| > 0.25. (B) Heatmap of top 3 active transcriptional factors (TF) predicted by pySCENIC. (C) UMAP of regulon activities (left) and violin plot of scores (right) for predicted SMC5 TFs. (D-E) IRF8 expression detected by RNAscope (D) and immunofluorescence (E) in human coronary arteries. CAD, coronary artery disease. Scale bar, 50 µm. (F-G) IRF8 mRNA (F) and protein (G) levels in aortae of ApoE-/- mice following 3-month high fat diet (HFD) treatment. Chow diet (CD), control. Data are presented as mean ± SD, *P<0.05, **P<0.01, ***P<0.001. The white arrows showed IRF8 positive SMC (SMC5).

Next, we investigated molecular dynamics underlying each SMC subtype. By counting cluster-specific DEGs, we found that DEGs were dominantly derived from SMC1-4 subtypes upon atherosclerotic remodeling compared to SMC5, suggesting it a fully remodeled phenotype corresponding to a “quiet” transcriptome (Figure 6A, Table S9). Consistent with previous results, GO terms of DEGs revealed inflammatory differentiation and vascular function in SMC1 remodeling, suggested ECM, collagen and filament related descriptions in SMC2, SMC3 and SMC4, and highlighted immune annotations in SMC5 (Figure S8A).

To explore the driving mechanisms underlying transcriptomic signatures, we applied unbiased, non-redundant pySCENIC algorithm to identify activated TFs within SMCs (Figure 6B). Specifically, SMC phenotype-modulating TFs such as BCL2-associated transcription factor 1 (BCLAF1) 12 and the nuclear factor of activated T cells (NFAT) family member, NFATC2^13^ were identified from contractile SMC1. Sex-determining region Y-box 9 (Sox9) 14 and transcription factor 4 (TCF4) 15 were distinguished in SMC3 and SMC4, known for osteochondrogenic and pro-mitogenic effect, respectively. Characteristic regulons in SMC5 were RUNX Family Transcription Factor 3 (RUNX3), High Mobility Group AT-Hook 1 (HMGA1) and IRF8, all involved in inflammatory regulation 16-18. Given the central role of SMC5 in pathogenesis, we compared transcriptional activities of these potential TFs within SMCs, and found both HMGA1 and IRF8 were overwhelmingly activated in SMC5 (Figure 6C, Table S10). Specifically, we found a large overlap of IRF8 targets and SMC5 feature genes, and functional terms of those overlapped genes were mostly suggestive of macrophage differentiation (e.g., myeloid leukocyte differentiation) and characteristics (e.g., endocytic vesicle). In contrast, HMGA1 or RUNX3 targets were less activated in SMC5, and HMGA1 was functionally implicated in leukocyte mediated adaptive immune processes (Figure S8B-C). Through RNAscope and immunofluorescent assays, we confirmed that IRF8 was activated both at the transcription and translation level within SMC upon atherosclerotic remodeling (Figure 6D-E). And this result was also supported by in vivo studies of high-fat diet induced ApoE-/- mice revealing IRF8 activation, concomitant with increased macrophage while decreased SMC markers (Figure 6F-G). Therefore, we inferred that IRF8 might act as a master regulator of SMC acquiring an atherogenic, macrophage-like signature in advanced atherosclerotic lesion.

### IRF8 facilitates SMC-to-macrophage transition via activating NF-κB signaling upon atherosclerotic remodeling

**Figure 7:**
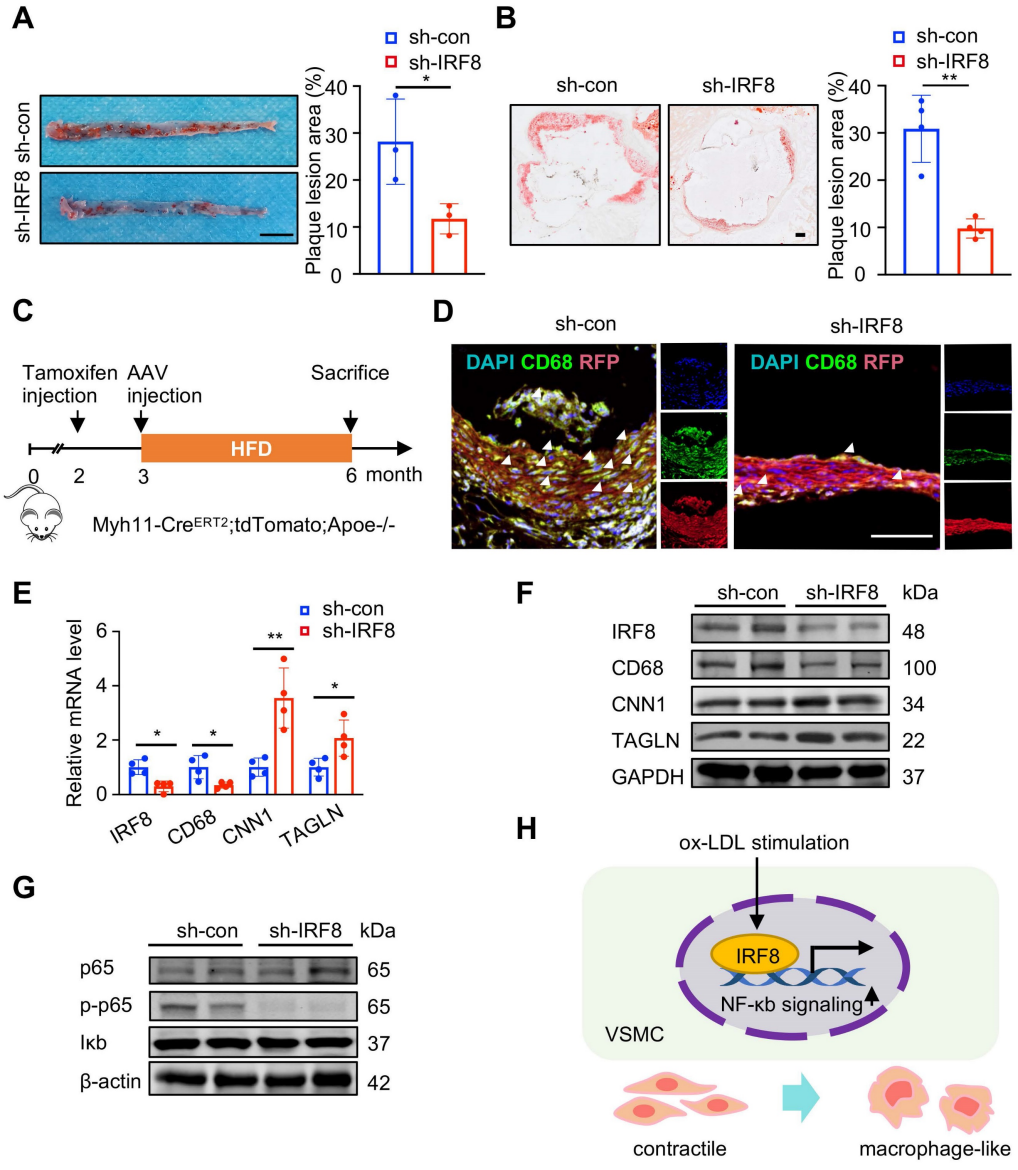
** IRF8 promotes SMC transdifferentiation towards macrophage via NF-κb signaling.** (A) Oil red O-stained aortae of ApoE-/- mice upon AAV8-mediated IRF8 silencing followed by 3-month high fat diet (HFD) treatment. Scale bar, 200 mm. (B) Oil red O-stained aortic roots as treated in (A). Scale bar, 200 µm. (C) Schematic illustration of experimental design using SMC-lineage tracing mice (Myh11-CreERT2;tdTomato;ApoE-/-). (D) Immunofluorescent staining of CD68 (green) in aortae of Myh11-CreERT2;tdTomato;ApoE-/- mice as treated in (A). Scale bar, 100 µm. (E-F) The mRNA (E) and protein (F) levels of IRF8, SMC and macrophage markers in aortae of ApoE-/- mice as treated in (A). (G) The protein levels of p65, phosphorylated p65 (p-p65) and Iκb in vivo. (H) Schematic review showing that IRF8 promotes vascular SMC (VSMC) phenotypic transition via activating NF-κb during atherosclerosis. Data are presented as mean ± SD, *P<0.05, **P<0.01. The white arrows showed CD68 positive SMC (SMC5).

To inspect the effect of IRF8 on SMC5 formation, we administered AAV8-mediated IRF8 silencing in vivo, and found decreased plaque formation in the aortae of ApoE-/- mice by IRF8 depletion (Figure 7A-B). Further immunofluorescent analysis of SMC-lineage-labeled mice revealed reduced SMC5 abundance following IRF8 silencing (Figure 7C-D). Consistently, increased SMC while reduced macrophage markers were confirmed in the IRF8-silencing aortae of ApoE-/- mice (Figure 7E-F). As prior analysis suggested (Figure 3A), NF-κb signaling activation posed a functional feature of SMC5, which was consolidated to promote a pro-inflammatory signature of SMC 19. Here, by silencing IRF8 in vivo, we showed that phosphorylated p65, an indicator of activated NF-κb signaling, was blocked by IRF8 suppression (Figure 7G). For in vitro studies, we further investigated whether IRF8 could regulate early events in SMC pro-inflammatory response by incubating mouse vascular SMCs with ox-LDL for indicated time. Results showed significantly increased level of IRF8 at 4 and 8 hours post ox-LDL treatment (Figure S9A-B). And to ascertain the regulation of IRF8 in SMC remodeling, we silenced IRF8 in vitro upon ox-LDL stimulation, followed by bulk RNA sequencing (Figure S9C-H). Via functional annotation of each sample via gene set variation analysis (GSVA), we showed that SMC associated intrinsic properties were heavily lost by ox-LDL treatment but regained upon IRF8 silencing. By contrast, macrophage or immune associated processes or genes were highlighted in the ox-LDL treated group but inactivated in IRF8-silenced SMCs (Figure S9G-H). Consistent with bioinformatics, the mRNA and protein assays both demonstrated that ox-LDL induced suppression of SMC and promotion of macrophage markers was prevented by IRF8 silencing (Figure S9I-J). And ox-LDL activated, proinflammatory NF-κb signaling was concomitantly inhibited following IRF8 inhibition (Figure S9K). Moreover, these in vitro experiments were replicated in human aortic SMCs (HASMCs), which further consolidated the role of IRF8 in SMC remodeling (Figure S10). Together, these results demonstrated that IRF8-NF-κb axis conferred a positive role in SMC transdifferentiation towards macrophage during atherosclerosis (Figure 7H).

## Discussion

Atherosclerosis poses a great risk to public health with annually increasing mortality and morbidity 1. SMC remodeling underlies a fundamental pathological basis which determines plaque initiation, progression and stabilization 20. In recent years, since molecular features are increasingly recognized to be the ultimate definition of cell type, SMC phenotypes have undergone rapid expansion by fate mapping approaches 10, 21. Although synthetic and inflammatory SMCs have long been identified as two major remodeled phenotypes with pathological significance, the complexity of SMC phenotypical heterogeneity in atherosclerosis is being revealed by single-cell transcriptome technology 22. Thus, utilizing publicly available scRNA datasets, we analyzed the atherosclerotic landscape of human advanced carotid plaque, presented phenotypic panorama and molecular dynamics of SMCs in atherosclerotic niche, and identified a macrophage-like SMC subtype with pathogenic potential in destabilizing lesions. Mechanistically, we searched fate-defining master regulators within SMCs by computational prediction and demonstrated that IRF8 facilitated SMC transdifferentiation towards macrophage via activating NF-κb signaling.

Conventional view holds that macrophage-like (“foam cell”) and fibrotic SMCs contribute differently to plaque stability; the former precipitates plaque vulnerability via inflammation while the latter thickens the protective fibrous cap 6, 23. However, the pathogenic contribution of each SMC phenotype to atherogenesis has invited much controversy these days even with the aid of lineage-tracing method combined with scRNA technique. The growing discoveries of SMC phenotypes upon remodeling are complicating their atheroprone effects in atherosclerotic plaques generated or reshaped by heterogenous conditions. For example, Wirka RC et al. claimed that murine plaques were dominated by fibroblast-like SMCs 8 and absent in reported atherogenic, proinflammatory SMCs 24, 25. However, Pan H et al. detected both macrophage-like and fibrochondrocyte-like SMCs in atherosclerotic lesions; in the meantime, he addressed the pathogenetic significance of an intermediate SMC cluster with pluripotency 7. Lately, the driving role in atherosclerosis progression was re-claimed by macrophage-like SMCs via interaction with macrophages 26. Other minor SMC phenotypes such as osteogenic, mesenchymal, adipocyte and oxygen sensing clusters were reported in human carotid, coronary and pulmonary arteries with elusive effects in pathogenesis 11, 26. Indeed, disease status, individual difference and genetic background complicate plaque pathology in vivo and underlie the source of analytical bias. Here, we explored the atherosclerotic niche in advanced atherosclerosis based on scRNA data of human carotid plaque. Notably, our de novo analysis avoided down-sampling prior to clustering as adopted by the original data provider aiming to alleviate the gap of cell counts in AC and PA tissue (~13,000 AC cells/patient and ~5000 PA cells/ patient in average), leaving only 3 SMC clusters discovered in the original study 27. For our analysis attempting to attain a detailed, complete atherosclerotic landscape, the bulk of cells were incorporated and integrated for clustering to improve the resolution power of minor clusters. Consequently, we distinguished 5 functionally distinct SMC subtypes depicted as contractile, fibroblast-like, osteogenic, synthetic and macrophage-like according to their molecular traits, of which SMC5 is a minor type of SMC. In particular, however, the highest pathogenic potential is exhibited by macrophage-like SMC5, as supported by bulk transcriptomes of unstable plaque. At the molecular level, SMC5 is featured by enrichment of atherogenic genes, including inflammatory genes (e.g., CCL2, CXCL1-3), matrix metalloproteinases (MMPs, e.g., MMP3, 9 and 19) and osteogenic genes (e.g., LGALS3, KLF4) 28-31. Such skewed profiles are also exhibited in synthetic and fibroblast-like SMCs. However, since their abundance was not significantly altered in the lesion, we infer that they may not be decisive in human plaque vulnerability 31. For another, SMC heterogeneity analysis of non-atherosclerotic arteries reveals a limited number of clusters, whose signatures corresponded to SMC1, 2 and 4 subtypes. In contrast, SMC1-5 subtypes were all highlighted in additional atherosclerotic scRNA-datasets despite different vascular beds. The differed distribution of phenotypes further supports the pathogenic potential of SMC5 in atherosclerotic remodeling.

As trajectory analysis and pseudotime calculation suggests, co-expression of SMC and macrophage lineage markers present a molecular feature of SMC5. By multi-immunofluorescence, we identified ACTA2 and CD68 double positive cells around atherosclerotic core of human coronary arteries, indicating SMC5 infiltration marks a cellular trait of unstable plaque. Previous reports found that certain master regulators (e.g., KLF4, TCF21 and OCT4) and pathways (e.g., Perk 32) are potent stimulators of SMC pluripotency. Thus, it raises the question of how a specific cell fate is ultimately decided during transdifferentiation 31. As recent findings began to shed light on it, some TFs have been revealed as essential or synergistical factors in deciding SMC outcomes upon remodeling. For instance, KLF4-induced activation of RUNX2 and SOX9 generates an osteogenic-like phenotype whereas C/EBP and PPARy are prerequisites for developing adipocyte-like SMC 33. For another, TFs such as LGALS3 34 and BCLAF1 12 are positively associated with macrophage-like SMC development. In our study, through transcriptional analysis and literature retrieval, we predicted IRF8, a TF of the interferon regulatory factor family, as a potential regulator of the pro-inflammatory signature acquired by macrophage-like SMC5 35, 36. Previous studies showed that IRF8 stimulates M1 macrophage-associated genes, and is essential for myeloid cell development and monocyte/macrophage differentiation 37, 38. Moreover, IRF8 also stimulates SMC phenotypic transition by promoting synthetic SMCs during neointimal remodeling upon vascular injury 39. By high throughput RNA sequencing, loss-of-function experiment and SMC-lineage tracing technology, we identified SMC as the origin of SMC5 and demonstrated a regulating role of IRF8 in SMC transdifferentiation towards macrophage during atherosclerosis. And for first time, we revealed that IRF8 promoted ox-LDL mediated SMC-to-macrophage transdifferentiation via NF-κb signaling, a recognized pathway in initiating inflammatory response and facilitating SMC-derived foam cells 40, 41. Since cell therapy poses a promising therapeutic approach available in anti-cancer treatment, our findings highlighted SMC phenotype plasticity in atherogenesis and illustrated cell fate deciding mechanism, which might reshape anti-atherosclerosis strategy through targeting potential harmful phenotype.

In conclusion, we gain new insights into SMC biology in advanced atherosclerosis, and reveal the pathogenic potential of macrophage-like SMC in plaque instability. Our findings expand the knowledge of SMC phenotypic remodeling in atherosclerosis and provide potential molecular targets for developing novel therapeutic methods.

## Supplementary Material

Supplementary methods, figures and tables.

## Abbreviations

ACTA2: α-smooth muscle actin 2
BCLAF1: BCL2-associated transcription factor1
CVD: cardiovascular disease
DAPI: 4′,6-diamidino-2-phenylindole
DAVID: Database for Annotation, Visualization and Integrated Discovery
ECM: extracellular matrix
FBS: fetal bovine serum
GEO: Gene Expression Omnibus
GO: Gene Ontology
KEGG: Kyoto Encyclopedia of Genes and Genomes
ssGSEA: single-sample gene set enrichment analysis
GSVA: gene set variation analysis
SMC: smooth muscle cell
HMGA1: High Mobility Group AT-Hook 1
IRF8: interferon regulatory factor-8
KEGG: Kyoto Encyclopedia of Genes and Genomes
MMP: matrix metalloproteinase
Mono/Mac: monocyte/macrophage
MSigDB: Molecular Signatures Database
MYH: myosin heavy chain
NFAT: nuclear factor of activated T cells
NF-κB: nuclear factor-κB
ox-LDL: oxidized lipoprotein
PBS: phosphate buffer solution
PC: principal component
RUNX3: RUNX Family Transcription Factor 3
scRNA-seq: single-cell RNA sequencing
SNN: shared nearest neighbor
Sox9: sex-determining region Y-box 9
TAGLN: transgelin
TCF4: transcription factor 4
TF: transcription factor
TSS: transcription start site
UMAP: Uniform Manifold Approximation and Projection
