# Supplementary material for: Re-analysis of single-cell transcriptomics reveals a critical role of macrophage-like smooth muscle cells in advanced atherosclerotic plaque
Source: Theranostics. 2024 Jan 27;14(4):1450–63. doi: 10.7150/thno.87201 (PMC10879858; doi:10.7150/thno.87201)
Supplement: Supplementary file 1 — Supplementary methods, figures and tables. [file thnov14p1450s1.pdf]

Expanded Methods

Cell culture, treatment and siRNA transfection

Mouse vascular SMCs were purchased from PriCells and cultured in smooth muscle cell medium (MED-0003, PriCells, China) with 10% fetal bovine serum (FBS) and supplemented growth factors. Human aortic SMCs (HASMCs) were obtained from Sciencell and cultured in Smooth Muscle Cell Medium (#1101, Sciencell, USA) containing 2 % FBS and SMC growth supplement. Cells between passage 3-8 were used for experiments.

For time-dependent oxidized lipoprotein (ox-LDL) treatment, cells were incubated with ox-LDL (100 μg/ml, Yiyuan Biotechnology Co., China) for indicated time. Naïve LDL (SAE0053, Sigma, USA) was used as control. Before ox-LDL treatment, cells were transfected with 50 nM IRF8-targeting siRNA mixture or scramble siRNA (Tsingke Biotechnology Co., China) for 24-48 h by using the HiPerFect Transfection Reagent (301705, Qiagen, Germany) following the manufacturer's guidelines. The sequences of IRF8-targeting siRNAs were listed as follows:

(For mouse)

siRNA-1: GAGAGCUGCAGCAAUUCUA,

siRNA-2: GGUUACGCUGUGCUCUGAA,

siRNA-3: GCAAGGGCGUGUUCGUGAA.

(For human)

siRNA-1: CCAUACAAAGUUUACCGAA,

siRNA-2: GCAGCUGUAUGUCCGGCAA,

siRNA-3: GCGUGUUCUGCAGCGGCAA.

Cell–cell communication analysis

Intercellular communication networks were built on predicted ligand-receptor interactions using the *CellChat* package (version 1.5) embedded database, CellChatDB.human. The major signaling inputs and outputs were visualized using the *netVisual_circle* function. Bubble plots of ligand-receptor interactions were computed by the *netVisual_bubble* function.

Functional Enrichment Analysis

Gene Ontology (GO) and Kyoto Encyclopedia of Genes and Genomes (KEGG) analyses were performed using the *clusterProfiler* (version 4.4) package. Adjusted p-value < 0.05 was set as the redundancy cutoff. To evaluate single-cell biological processes, *irGSEA* (version 1.1) package was applied on the top 50 significant tack genes using the UCell method and referring to the Molecular Signatures Database (MSigDB) embedded C5 ontology datasets.

Correlation analysis

Average expressions of top 1000 variable genes within monocyte/macrophage (Mono/Mac) and SMC subtypes were used for computing intercellular correlation using the *Corrplot* package. Statistical significance was calculated using the *cor.mtest* function setting the confidential level at 0.95.

Pseudotime trajectory analysis

Cell trajectories and pseudotime calculation were analyzed by the *Monocle* 3 package following standard guidelines. Seurat-embedded clustering settings were transferred to Monocle 3, followed by cell trajectory prediction using the *orderCells* function setting the starting pseudotime state from SMC1. Significant track genes were calculated using the *graph_test* function, and ranked by the morans_I index.

SMC phenotype signature analysis using bulk RNA datasets

Molecular signatures of SMC1-5 subtypes were acquired by extracting cluster-specific marker genes (avg_log2FC>0.15 and adjusted p-value<0.05) identified in the clustering step of scRNA-seq analysis. Next, single-sample gene set enrichment analysis (*ssGSEA*) was performed with the GSVA package to calculate the proportions of 5 SMC subtypes in 3 bulk transcriptomes of human vulnerable plaques. SMC1-5 signature scores were depicted as box plots using the *ggplot2* package.

Transcription factor (TF) analysis

Active TFs in each SMC subtype were predicated by pySCENIC software in Python environment. Transcription start site (TSS) and regulatory network were computed based on motif-rankings using hg38-tss-centered-10 kb and hg38-500 bp-upstream reference sets. The loom format file was then processed in R environment using SCENIC, SCopeLoomR and AUCell packages to identify regulons and assess TF activities.

Oil red O staining

En-face aortae were carefully isolated from the aortic arch to the iliac artery and were cut along the longitudinal axis. Following fixing in 4% formalin for 24 h, aortae were washed in PBS for three times before oil red O staining using Modified Oil Red O Staining Kit (Beyotime, China) according to the manufacturer's instructions. For aortic root sections, fresh frozen samples were sectioned at 8 μm and incubated in modified oil red O solution for 20 min following the manufacturer's instructions. All analyses were assessed by Image J software (NIH, USA).

Multi-immunofluorescence

Deparaffinization and rehydration were performed on paraffin embedded artery sections in dimethylbenzene and ethanol of decreasing concentrations. Heat-induced antigen retrieval was conducted in Citrate-EDTA Antigen Retrieval Solution (Beyotime, China) followed by blockade of endogenous peroxidase using 3% H_2O_2 and then blocked in QuickBlock™ Blocking Buffer for Immunol Staining (Beyotime, China). Multi-immunofluorescence was performed using four-color mIHC Fluorescence kit (Hunan Aifang Biotechnology Co., Ltd., Changsha, China) based on the tyramide signal amplification (TSA) technology according to the manufacturer's instruction. Primary antibodies of anti-rabbit ACTA2 (1:1000, 14395-1-AP, Proteintech, China), anti-rabbit IRF8 (1:800, 18977-1-AP, Proteintech, China), anti-mouse TAGLN (1:1000, 60213-1-Ig, Proteintech, China), anti-rabbit RFP (1:1000, ab62341, Abcam, USA) and anti-mouse CD68 (1:1000, 66231-2-Ig, Proteintech, China) or anti-rabbit CD68 (1:1000, 28058-1-AP, Proteintech, China) were mounted to sections at 4°C overnight before

incubation with anti-rat/mouse horseradish peroxidase-conjugated (HRP) secondary antibody for 15 min. TSA 520, 570 and 620 were used for immunofluorescent labeling for 10 min in the dark. Nucleus was labeled by 4′,6-diamidino-2-phenylindole (DAPI). Images were taken by confocal microscopy (Olympus, Japan).

RNAscope in situ hybridization

RNAscope assay (ACD Biosystems, USA) was performed following the manufacturer's protocol. Paraffin-embedded human coronary sections were hybridized with IRF8 mRNA probe. Amplification of probe signals were based on TSA technology and labeled with Alexa 555. Probe signals were visualized by confocal microscopy (Olympus, Japan).

Bulk RNA sequencing (RNA-seq) and bioinformatics analysis

Total RNA was extracted from mouse aortic SMCs using Trizol reagent kit (Invitrogen, Carlsbad, CA, USA) according to the manufacturer's protocol. The NEBNext Ultra RNA Library Prep Kit for Illumina (NEB, New England Biolabs, Ipswich, MA, USA) was used to construct the sequencing library using 1 μg of purified RNA following the manufacturer's guidelines. Then, the cDNA library was sequenced using Illumina Novaseq6000 by Gene Denovo Biotechnology Co. (Guangzhou, China).

Raw reads were filtered by fastp (version 0.18.0) and removed of ribosome RNA mapped ones using Bowtie2 (version 2.2.8). Then, paired-end clean reads were aligned with an in-build reference genome using HISAT2. 2.4, and mapped reads

were assembled by StringTie v1.3.1. TPM (Transcripts Per Kilobase of exon model per Million mapped reads) value was calculated by using RSEM software. Raw data were deposited in the China National Center for Bioinformation (CNCB) under the accession number PRJCA022581.

Activities of biological processes in each sample were assessed by gene set variation analysis (GSVA) using the R package GSVA. Heatmap of gene expression was conducted using the R package pheatmap.

Real-time PCR

RNA was extracted from cells with RNAiso Plus (Takara, Japan) and reverse transcription of purified DNA was generated by using PrimeScript™ RT Master Mix (Takara, Japan). Next, qRT-PCR was performed on 1 μg of cDNA using TB Green® Premix Ex Taq™ II (Takara, Japan) on the Bio-Rad CFX96 system. $2^{-\Delta\Delta CT}$ method was used to calculate relative gene expression. Primers (Tsingke Biotechnology Co.) used in this study were listed as follows:

(For mouse)

GADPH forward primer: AACGACCCCTTCATTGACCT, reverse primer: TGGAAGATGGTGATGGGCTT;

IRF8 forward primer: GGAACCTTCTGTGGATGAGTACA, reverse primer: AGCTGAATGGTGTGTGTCATAGG;

CD68 forward primer: TGGACAGCTTACCTTTGGATTCA, reverse primer: TAGAGAGAGCAGGTCAAGGTGAA.

ACTA2 forward primer: AATGGCTCTGGGCTCTGTAA, reverse primer: GGTGATGATGCCGTCTTCTA.

CNN1 forward primer: AAAAACGTGTGAGGAGGGAAGA, reverse primer: GTATTTCTGGGCCAGCTTGTTC.

TAGLN forward primer: GGTGAGCCAAGCAGACTTCC, reverse primer: TGTTGAGGCAGAGAAGGCTTG.

MYH11 forward primer: CACTCCTCAATGCCTCCTCT, reverse primer: TTTCCCCAACTGCTCTTTG.

(For human)

GADPH forward primer: AACGACCCCTTCATTGACCT, reverse primer: TGGAAGATGGTGATGGGCTT;

IRF8 forward primer: GTCTTCGACACCAGCCAGTT, reverse primer: AGCTCTTCCCAGCCTCTTCT;

ACTA2 forward primer: GTTCCGCTCCTCTCTCCAAC, reverse primer: GTGCGGACAGGAATTGAAGC.

CNN1 forward primer: GTTAAGAACAAGCTGGCCCAGAAG, reverse primer: CACCCCATACTTGGTGATGGC.

MYH11 forward primer: GCAAGAAGAGGCACGAGATG, reverse primer: TTTTCCAGCTCTCCCGTGAT

Western blot

Whole cells were lysed in RIPA lysis buffer (Beyotime, China) with protease

inhibitors (MCE, USA). Proteins were separated by 10% SDS–PAGE, transferred to nitrocellulose blotting membranes, and incubated overnight at 4 °C with antibodies involving anti-IRF8 (1:1000, 18977-1-AP, Proteintech, China), anti-CD68 for mouse (1:300, 28058-1-AP, Proteintech, China), anti-CD68 for human (1:500, 86985, Cell Signaling Technology, USA), anti-ACTA2 (1:1000, 14395-1-AP, Proteintech, China), anti-CNN1 (1:500, 24855-1-AP, Proteintech, China), anti-TAGLN (1:500, 60213-1-Ig, Proteintech, China) anti-MYH11 (1:300, 21404-1-AP, Proteintech, China), anti-β-actin (1:1000, 66009-1-Ig, Proteintech, China), anti-GAPDH (1:5000, 60004-1-Ig, Proteintech, China), anti-p65 (1:500, 8242, Cell Signaling Technology, USA), anti-phosphorylated p65 (1:200, 3033, Cell Signaling Technology, USA) and anti-IκB (1:500, 10268-1-AP, Proteintech, China). Then membranes were incubated with IRDye 800CW donkey anti-rabbit (926-32213, LI-COR, USA) or anti-mouse (926-32212, LI-COR, USA) antibody in the dark at the room temperature for 1 h and visualized using the Odyssey Infrared Imaging System (LI-COR, USA).

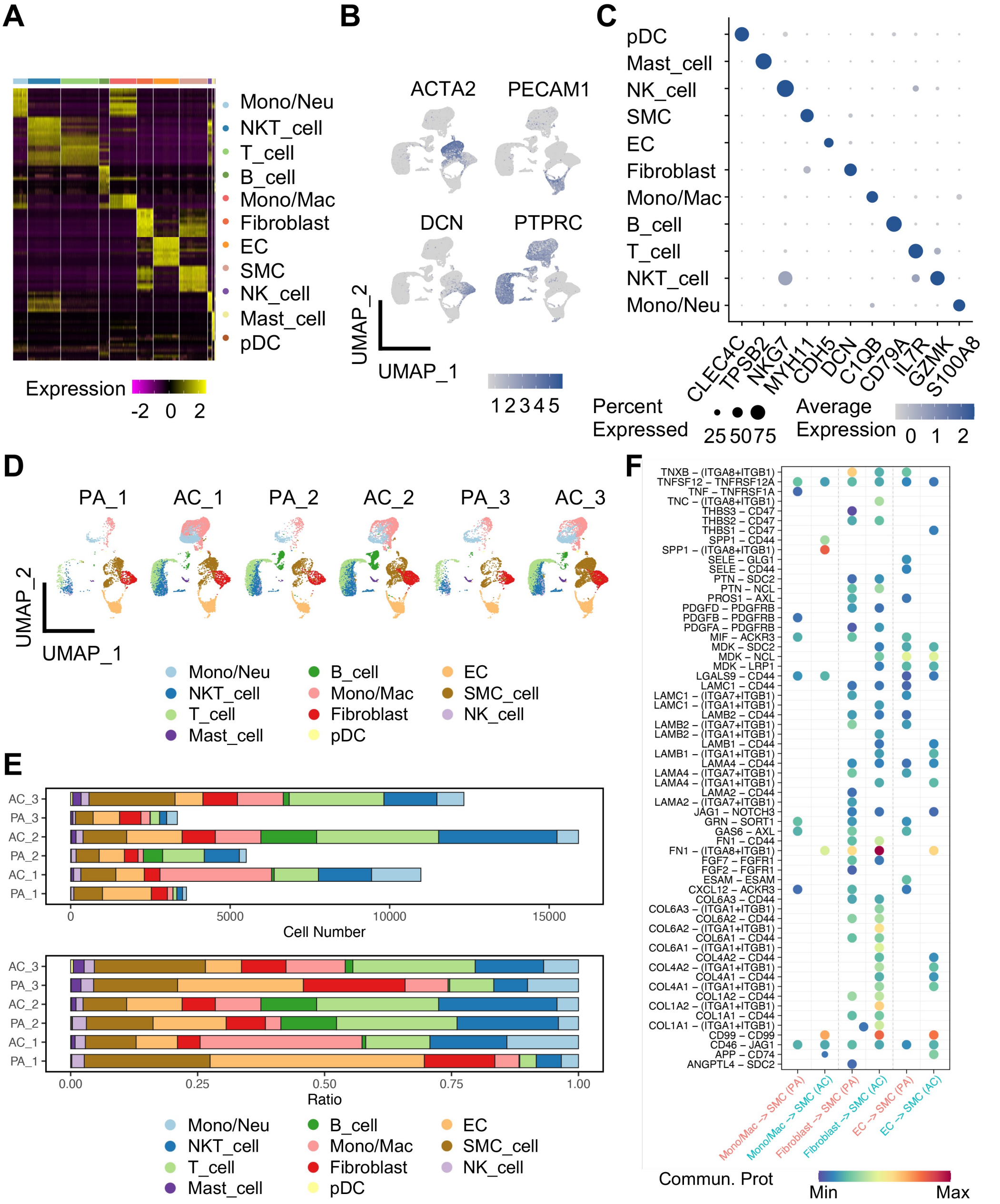

Figure S1. Characterization of single-cell RNA sequencing data of human carotid arteries. (A) Heatmap of top 10 feature genes for each cell type. (B) Uniform Manifold Approximation and Projection (UMAP) of markers ACTA2, PECAM1, DCN and PTPRC for identifying smooth muscle cell (SMC), endothelial cell (EC), fibroblast and immune cell. Color darkness indicates average gene expression. (C) Dot plot of marker gene expressions. Dot size and color darkness indicate the percentage of gene-expressing cells and average expression, respectively. (D) UMAP of cell types in each sample. PA, proximal adjacent tissue. AC, atherosclerotic core. (E) Bar plot of cell numbers and proportions of cell types within each sample. (F) Ligand–receptor interactions among monocyte/macrophage (Mono/Mac), fibroblast, EC and SMC. Dot size represents statistical significance. Communication probability (Commun. Prot) is calculated by Cellchat.

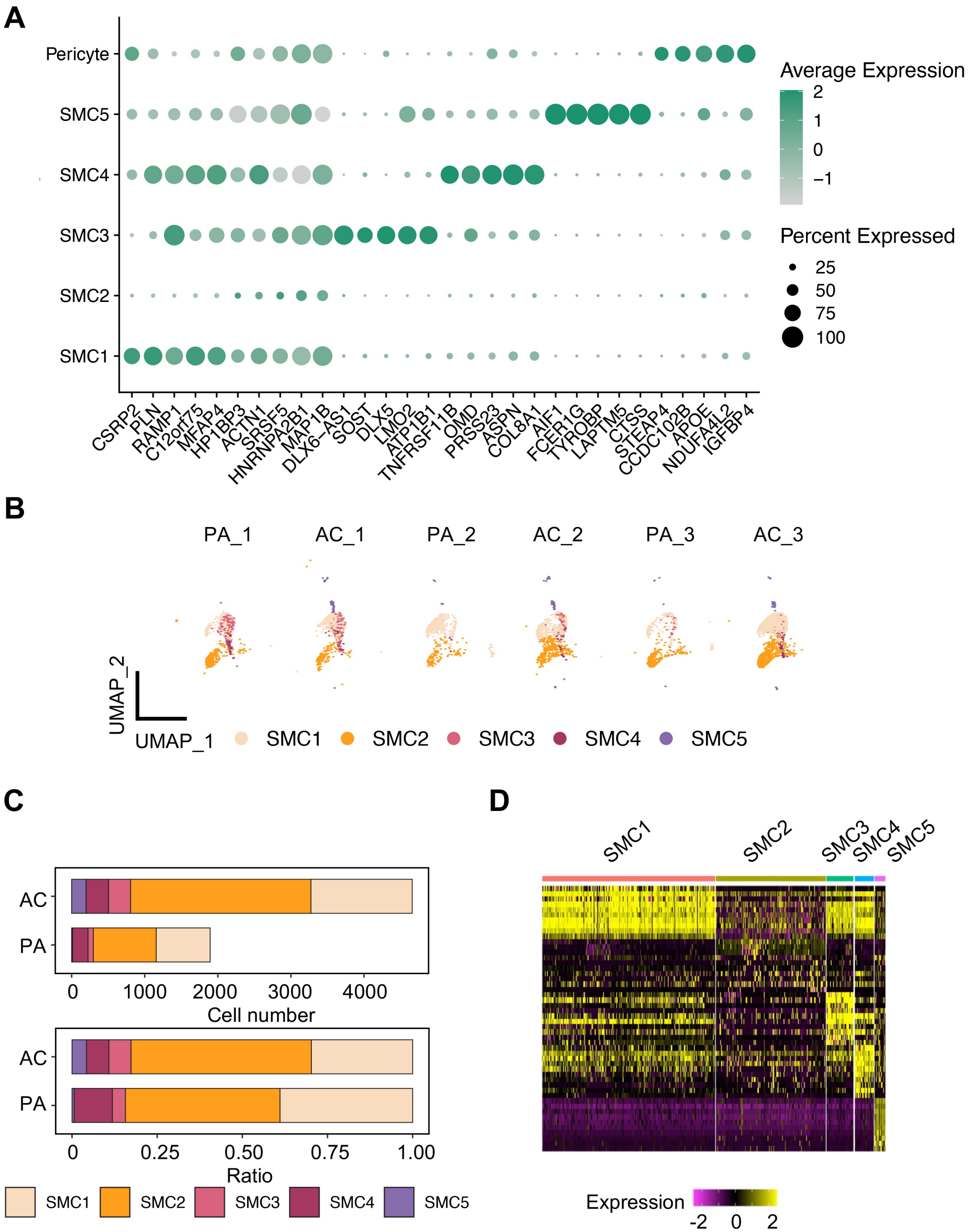

Figure S2. Annotation of pericyte and smooth muscle cell (SMC) subtypes. (A) Dot plot of genes distinguishing pericyte from each SMC cluster. Dot size and color darkness indicate the percentage of cells expressing the gene and average expression, respectively. (B) Distribution of SMC subtypes in each sample. PA, proximal adjacent tissue. AC, atherosclerotic core. (C) Bar plot of cell number and proportions of SMC subtypes in PA and AC group. (D) Heatmap of top 10 genes enriched in each SMC subtype.

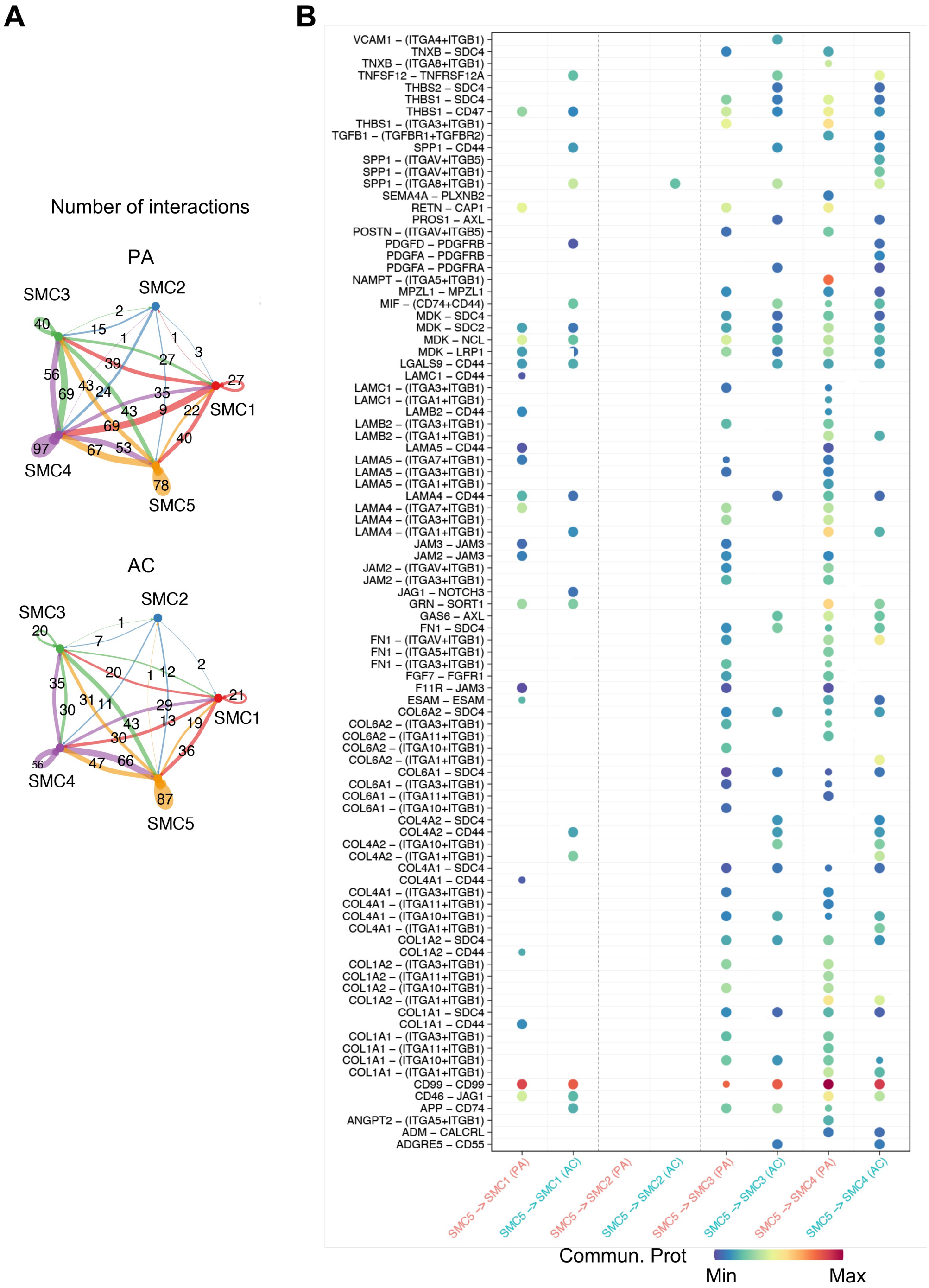

Figure S3. Interactions within smooth muscle cell (SMC) subtypes in atherosclerosis. (A) Cell communication analysis of SMC subtypes in proximal adjacent tissue (PA) and atherosclerotic core (AC). (B) Ligand–receptor interactions of SMC5 with other SMC clusters. Commun. Prot, communication probability.

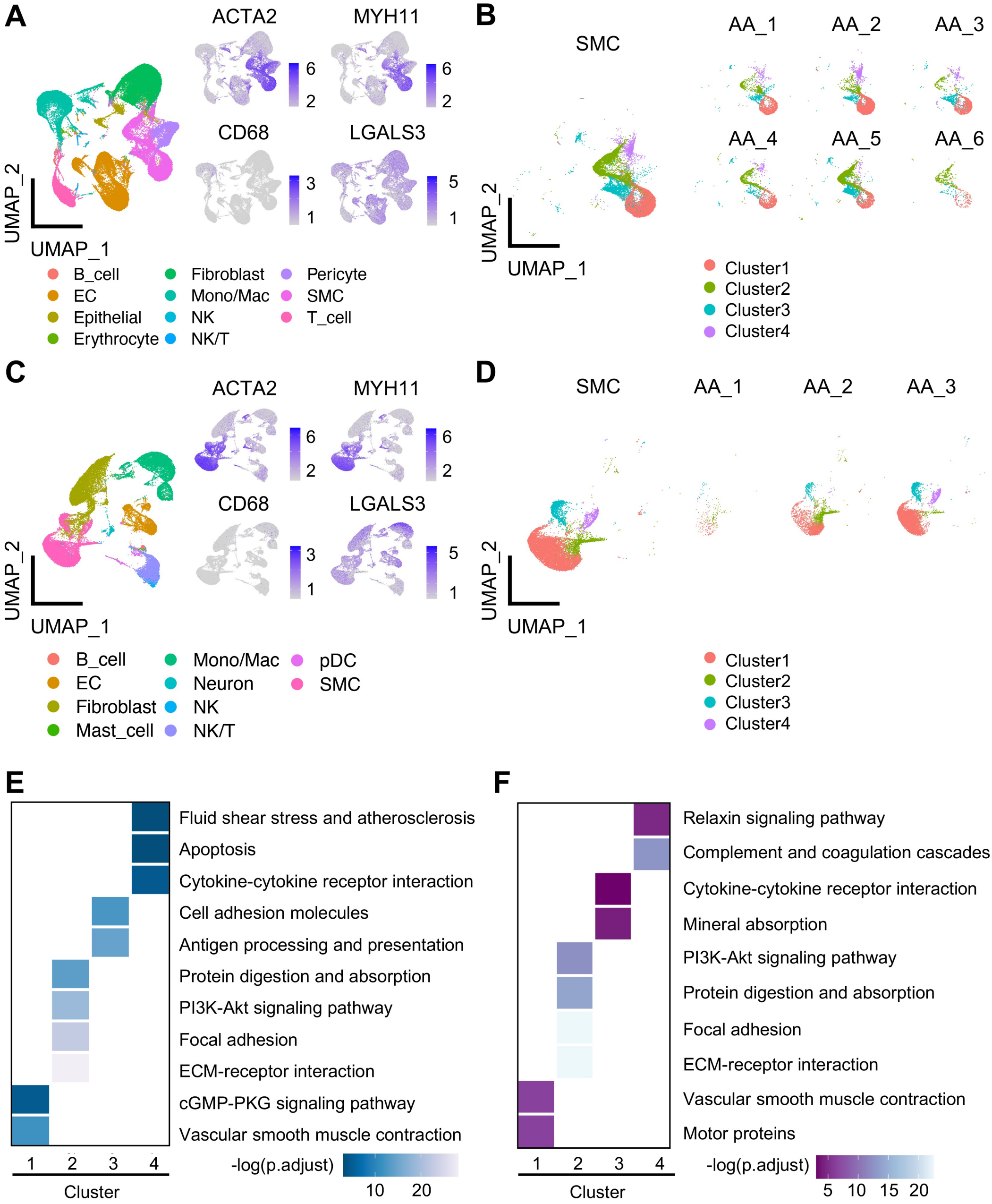

Figure S4. Identification of smooth muscle cell (SMC) clusters in non-atherosclerotic arteries. (A) Uniform Manifold Approximation and Projection (UMAP) of single-cell RNA sequencing (scRNA-seq) data (GSE216860) from 6 normal human ascending aortae (AA) and expression plots of ACTA2, MYH11, CD68 and LGALS3. (B) Visualization of SMC clusters in AA samples of GSE216860. (C) UMAP of scRNA-seq data (GSE213740) from 3 normal human AA samples and expression plots of ACTA2, MYH11, CD68 and LGALS3. (D) Visualization of SMC clusters in AA samples of GSE213740. (E-F) Kyoto Encyclopedia of Genes and Genomes (KEGG) analysis of SMC clusters in GSE216860 (E) and GSE213740 (F) using cluster-specific upregulated genes. P.adjust, adjusted P value.

GEO accession	Data type	Sample description
GSE28829	Affymetrix GeneChip	13 early (intimal thickening and xanthoma), 16 advanced (fibrous cap atheroma) atherosclerotic carotid artery segments
GSE120521	High-throughput sequencing	4 stable and 4 unstable sections of human atherosclerotic plaque
GSE163154	Illumina array	16 low- and 27 high-risk atherosclerotic lesion carotid segments with intraplaque haemorrhage (IPH)

Figure S5. Bulk RNA transcriptome datasets used for evaluating molecular features characterized by each SMC subtype.

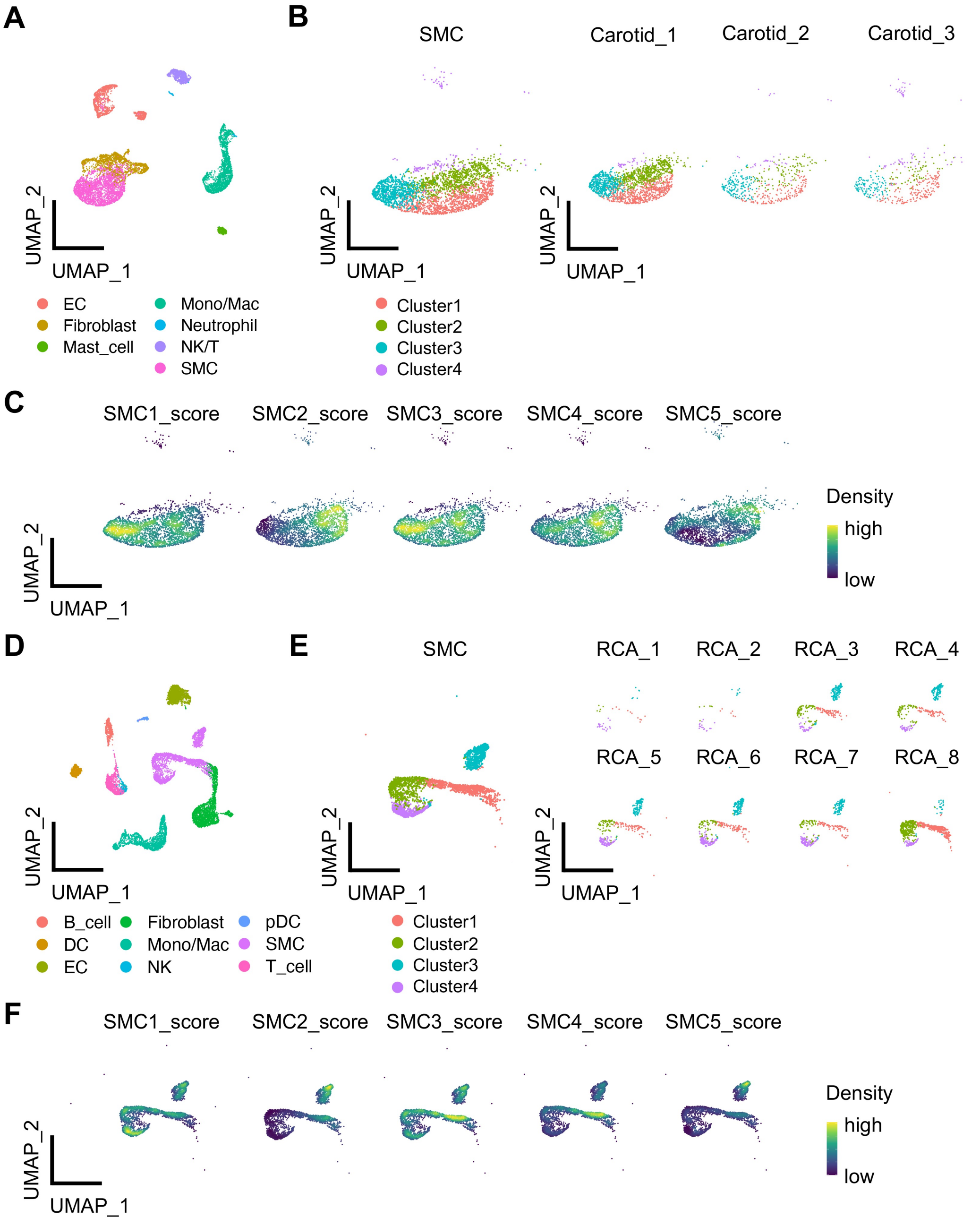

Figure S6. Conservation of smooth muscle cell (SMC) subtype features in different datasets of atherosclerotic arteries. (A) Uniform Manifold Approximation and Projection (UMAP) of single-cell RNA sequencing (scRNA-seq) data (GSE155514) from 3 plaque-laden human carotid samples. (B) Visualization of SMC clusters in carotid samples of GSE155514. (C) Density plot of of the SMC cluster scores in 1-4 SMC clusters identified in GSE155514. (D) UMAP of scRNA-seq data (GSE131778) from 8 atherosclerotic human right coronary arteries (RCA). (E) Visualization of SMC clusters in RCA samples of GSE131778. (F) Density plot of of the SMC cluster scores in 1-4 SMC clusters identified in GSE131778.

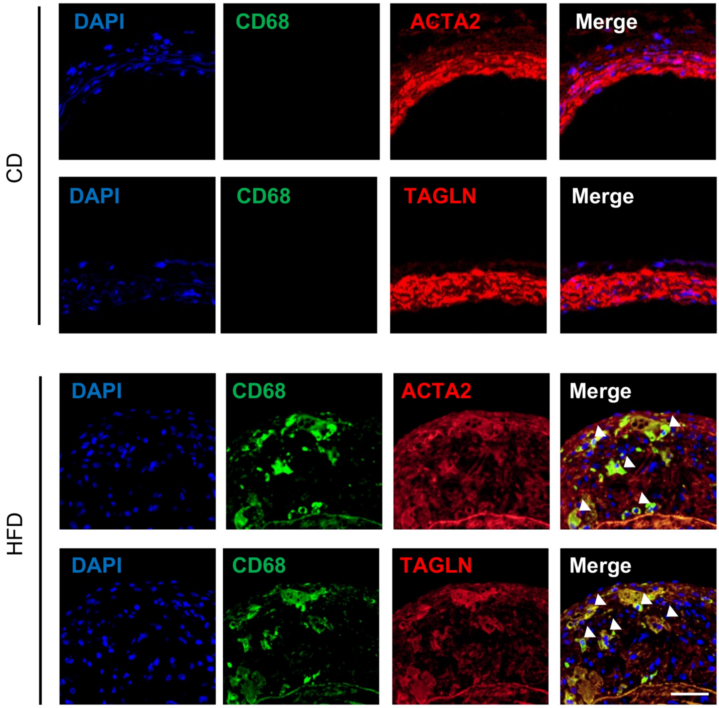

Figure S7. Immunofluorescent staining of ACTA2 or TAGLN (red) and CD68 (green) in the carotid arteries of ApoE-/- mice following 3-month high fat diet (HFD) treatment. Chow diet (CD), control. Scale bar, 50 µm. The white arrow indicated CD68 positive SMCs (SMC5).

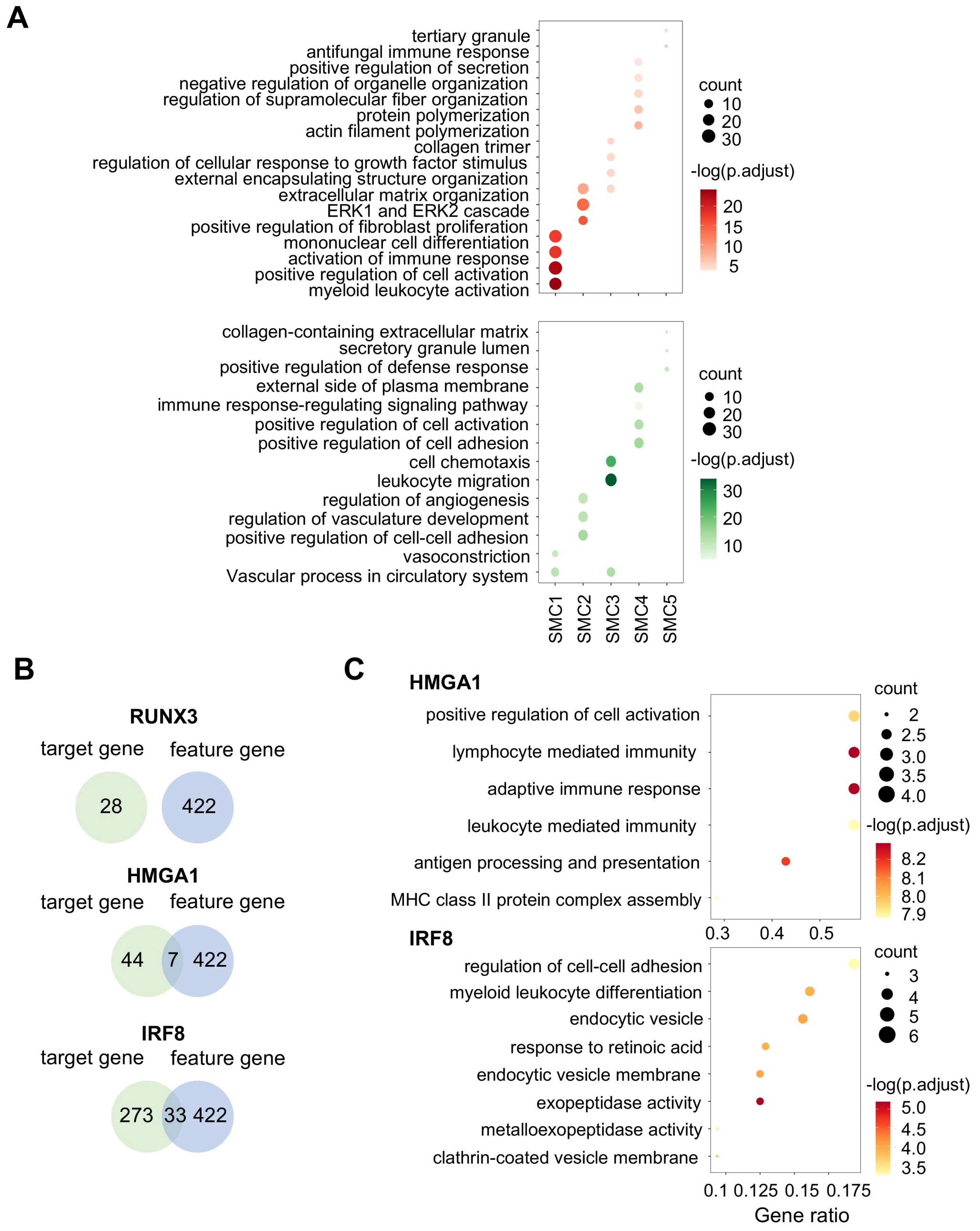

Figure S8. Transcriptional features of smooth muscle cell (SMC) subtypes upon remodeling. (A) Gene ontology (GO) analysis of significantly up- (left) and down-regulated (right) genes of SMC subtypes in atherosclerotic core (AC) compared to proximal adjacent (PA) tissue. Dot size and color darkness indicate the number of enriched GO terms and statistical significance, respectively. (B) Venn plot of predicted SMC5 regulon targets and its feature genes (adjusted P value < 0.05, avg_log2FC>0.15). (C) GO analysis of the overlapped genes as indicated in (B). P.adjust, adjusted P value.

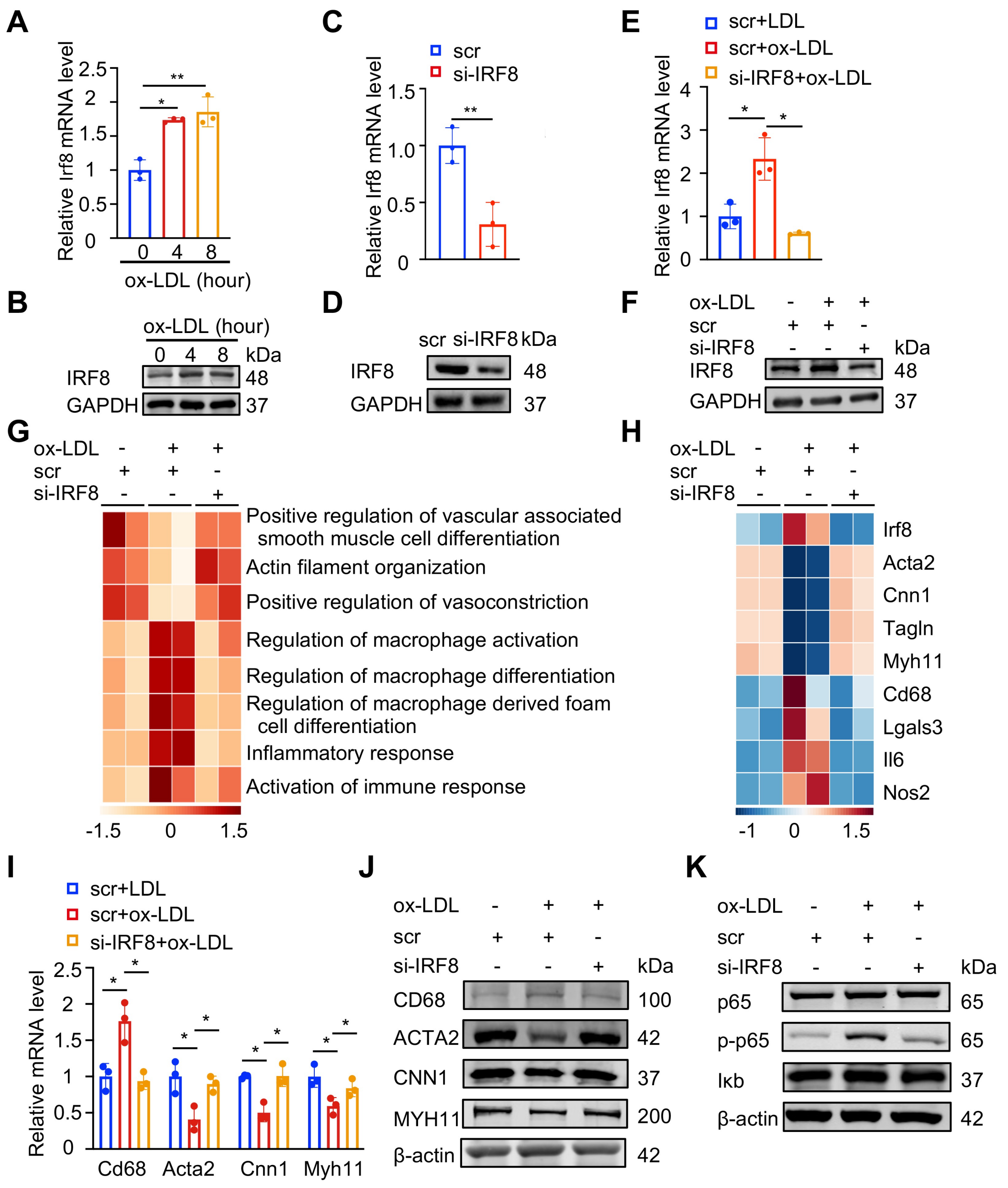

Figure S9. IRF8 silencing inhibits SMC-to-macrophage transition in mouse aortic smooth muscle cells (SMCs). (A-B) Real-time PCR (A) and western blot (B) analysis of IRF8 expression following oxidized low density lipoprotein (ox-LDL) treatment at indicated time. (C-D) The mRNA (C) and protein (D) level of IRF8 following IRF8 silencing in vitro. Scr, control siRNA. (E-F) Real-time PCR (E) and western blot (F) analysis of IRF8 expression following IRF8 silencing and 8-hour ox-LDL treatment. (G) Significant biological processes revealed by gene set variation analysis (GSVA) in the transcriptome of murine aortic SMCs. (H) Heatmap showing expression of SMC and macrophage feature genes. (I-J) IRF8 expression detected by real-time PCR (I) and western blot (J) analysis following IRF8 silencing and 72-hour ox-LDL treatment. (K) Western blot analysis of p65, phosphorylated p65 (p-p65) and Iκb expressions in vitro. Data are presented as mean ± SD, *P<0.05, **P<0.01.

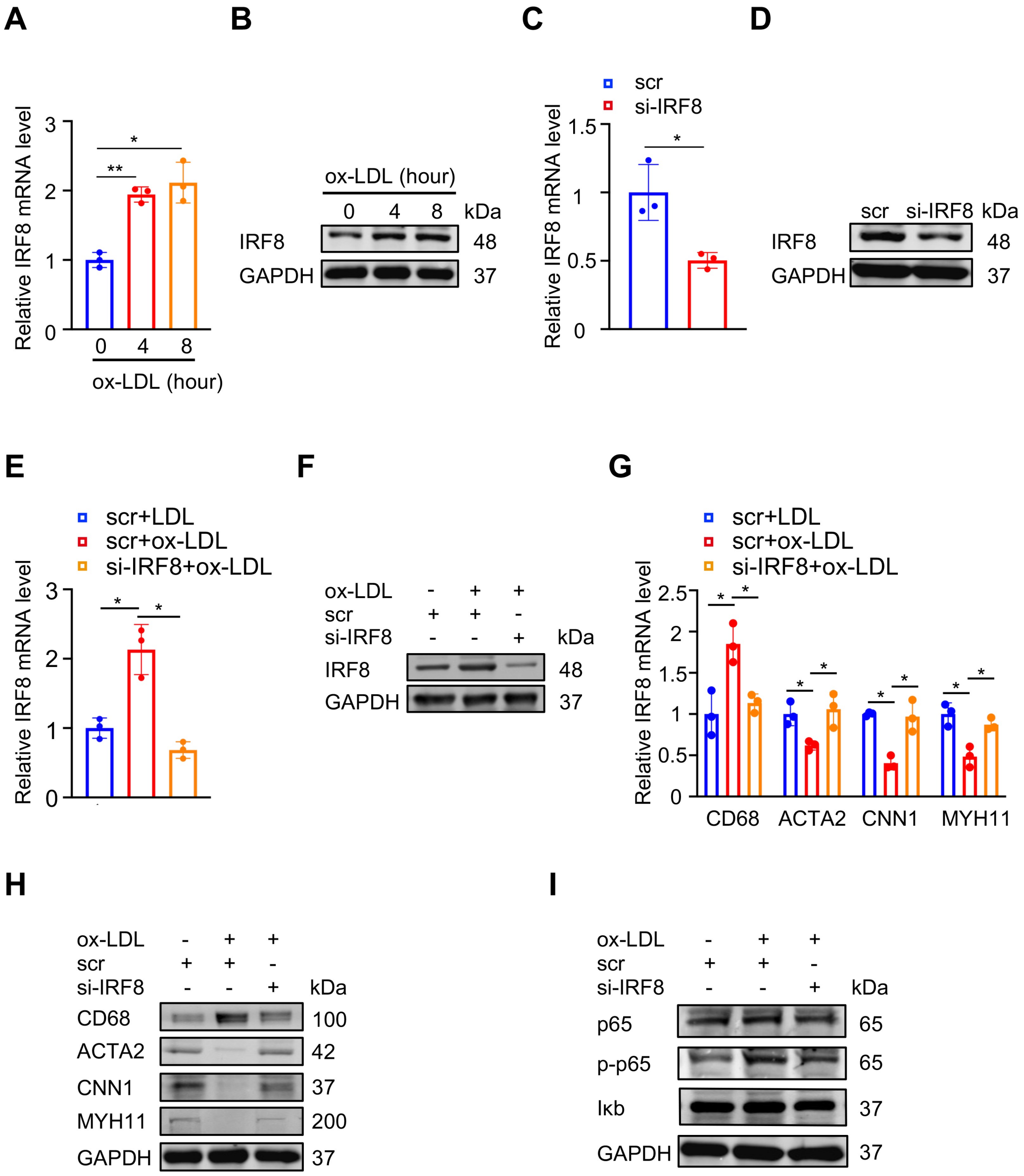

Figure S10. IRF8 silencing inhibits SMC-to-macrophage transition in human aortic smooth muscle cells (HASMCs). (A-B) Real-time PCR (A) and western blot (B) analysis of IRF8 expression following oxidized low density lipoprotein (ox-LDL) treatment at indicated time. (C-D) The mRNA (C) and protein (D) level of IRF8 following IRF8 silencing in vitro. Scr, control siRNA. (E-F) Real-time PCR (E) and western blot (F) analysis of IRF8 expression following IRF8 silencing and 8-hour ox-LDL treatment. (G-H) IRF8 expression detected by real-time PCR (G) and western blot (H) analysis following IRF8 silencing and 72-hour ox-LDL treatment. (I) Western blot analysis of p65, phosphorylated p65 (p-p65) and Iκb expressions in vitro. Data are presented as mean ± SD, *P<0.05, **P<0.01.

Table S1. Markers for cluster annotation in human carotid artery

gene	p_val	avg_log2FC	pct.1	pct.2	p_val_adj	cluster
LYZ	0	1.532953391	0.939	0.539	0	Neutrophil
S100A9	0	1.516613439	0.905	0.454	0	Neutrophil
S100A8	0	1.383692746	0.764	0.379	0	Neutrophil
AIF1	0	1.365295521	0.935	0.527	0	Neutrophil
FCN1	0	1.244657135	0.802	0.328	0	Neutrophil
TYROBP	0	1.231492304	0.943	0.555	0	Neutrophil
FCER1G	0	1.158949051	0.928	0.54	0	Neutrophil
MNDA	0	1.102463666	0.851	0.467	0	Neutrophil
CD14	0	1.098721514	0.92	0.529	0	Neutrophil
IL1B	0	1.07992051	0.825	0.371	0	Neutrophil
MS4A6A	0	1.057181471	0.913	0.524	0	Neutrophil
CPVL	0	1.035167976	0.893	0.476	0	Neutrophil
PLAUR	0	1.024930558	0.88	0.599	0	Neutrophil
SERPINA1	0	1.01986572	0.862	0.414	0	Neutrophil
CTSS	0	1.018091274	0.933	0.664	0	Neutrophil
CYBB	0	1.008012738	0.902	0.5	0	Neutrophil
NAMPT	0	0.996367226	0.907	0.626	0	Neutrophil
LST1	0	0.993081045	0.836	0.491	0	Neutrophil
CXCL8	0	0.964190734	0.712	0.367	0	Neutrophil
C5AR1	0	0.951656891	0.814	0.463	0	Neutrophil
IGSF6	0	0.940324774	0.879	0.512	0	Neutrophil
HLA-DRA	0	0.931193581	0.982	0.803	0	Neutrophil
SAT1	0	0.924544762	0.978	0.824	0	Neutrophil
PLEK	0	0.91984526	0.833	0.514	0	Neutrophil
TYMP	0	0.919425259	0.91	0.647	0	Neutrophil
AREG	0	0.899925933	0.775	0.556	0	Neutrophil
OLR1	0	0.898714137	0.848	0.472	0	Neutrophil
CEBPB	0	0.892765662	0.912	0.599	0	Neutrophil
MAFB	0	0.875607086	0.89	0.558	0	Neutrophil
BCL2A1	0	0.861482948	0.819	0.491	0	Neutrophil
CD68	0	0.857483334	0.891	0.475	0	Neutrophil
CFD	0	0.847176044	0.873	0.443	0	Neutrophil
HLA-DRB5	0	0.844269348	0.967	0.784	0	Neutrophil
TMEM176B	0	0.830821898	0.893	0.467	0	Neutrophil
FGL2	0	0.827259301	0.863	0.495	0	Neutrophil
CXCL2	0	0.824734455	0.738	0.435	0	Neutrophil
RGS2	0	0.820998501	0.857	0.639	0	Neutrophil
CLEC7A	0	0.820837514	0.856	0.494	0	Neutrophil
IFI30	0	0.820260874	0.81	0.499	0	Neutrophil
PTPRE	0	0.818220001	0.832	0.53	0	Neutrophil
HLA-DPA1	0	0.796261791	0.977	0.79	0	Neutrophil
CEBPD	0	0.795002415	0.937	0.68	0	Neutrophil
HLA-DPB1	0	0.789737155	0.959	0.776	0	Neutrophil
CTSB	0	0.784819095	0.906	0.568	0	Neutrophil
HLA-DQB1	0	0.77585935	0.941	0.666	0	Neutrophil
PSAP	0	0.773502064	0.901	0.655	0	Neutrophil
HLA-DQA1	0	0.773155984	0.921	0.642	0	Neutrophil
SPI1	0	0.771572033	0.806	0.47	0	Neutrophil
GLUL	0	0.769703245	0.848	0.548	0	Neutrophil
IER3	0	0.76176829	0.902	0.659	0	Neutrophil
GK	0	0.760244659	0.786	0.442	0	Neutrophil
HLA-DRB1	0	0.755782248	0.96	0.778	0	Neutrophil
FCGR2A	0	0.748168966	0.862	0.465	0	Neutrophil
C1orf162	0	0.74015484	0.824	0.535	0	Neutrophil

PHACTR1	0	0.726321301	0.747	0.458	0	Neutrophil
FPR1	0	0.723234066	0.732	0.361	0	Neutrophil
NCF2	0	0.721359475	0.777	0.341	0	Neutrophil
HLA-DMA	0	0.716703907	0.925	0.632	0	Neutrophil
AC020656.1	0	0.714607465	0.665	0.295	0	Neutrophil
SOD2	0	0.714225355	0.871	0.581	0	Neutrophil
THBS1	0	0.7103667	0.784	0.516	0	Neutrophil
THBD	0	0.706344407	0.822	0.534	0	Neutrophil
GPR183	0	0.699225428	0.897	0.687	0	Neutrophil
HMOX1	0	0.696277489	0.777	0.518	0	Neutrophil
CST3	0	0.682623149	0.95	0.721	0	Neutrophil
CTSH	0	0.677345149	0.9	0.589	0	Neutrophil
FCGR3A	0	0.673857683	0.834	0.425	0	Neutrophil
HLA-DQA2	0	0.672564399	0.935	0.694	0	Neutrophil
CD83	0	0.671019565	0.839	0.599	0	Neutrophil
VAMP8	0	0.666556211	0.907	0.673	0	Neutrophil
SLC11A1	0	0.644893931	0.659	0.425	0	Neutrophil
ITGB2	0	0.64484497	0.922	0.706	0	Neutrophil
GRN	0	0.640941769	0.854	0.637	0	Neutrophil
PYCARD	0	0.635979901	0.878	0.668	0	Neutrophil
MS4A7	0	0.632541457	0.802	0.449	0	Neutrophil
COTL1	0	0.631483078	0.923	0.751	0	Neutrophil
TKT	0	0.630817391	0.904	0.64	0	Neutrophil
CSTA	0	0.624355752	0.714	0.35	0	Neutrophil
PPIF	0	0.618127658	0.754	0.449	0	Neutrophil
JAML	0	0.617207504	0.796	0.43	0	Neutrophil
CD74	0	0.616646149	0.981	0.891	0	Neutrophil
HLA-DMB	0	0.612464416	0.853	0.531	0	Neutrophil
FCGRT	0	0.609354405	0.884	0.609	0	Neutrophil
CHMP1B	0	0.607208292	0.845	0.526	0	Neutrophil
FCGR1A	0	0.604650296	0.86	0.457	0	Neutrophil
ITGAX	0	0.604480269	0.704	0.39	0	Neutrophil
F13A1	0	0.60371747	0.868	0.529	0	Neutrophil
PLIN2	0	0.594997776	0.856	0.665	0	Neutrophil
EFHD2	0	0.593282837	0.825	0.609	0	Neutrophil
MXD1	0	0.593177486	0.719	0.458	0	Neutrophil
CLEC10A	0	0.589151939	0.795	0.354	0	Neutrophil
IL1RN	0	0.583186063	0.661	0.334	0	Neutrophil
PLBD1	0	0.581400961	0.762	0.395	0	Neutrophil
TNFSF13B	0	0.580629673	0.815	0.572	0	Neutrophil
SGK1	0	0.571091275	0.834	0.598	0	Neutrophil
KLF4	0	0.567738583	0.89	0.577	0	Neutrophil
AP1S2	0	0.567297453	0.847	0.541	0	Neutrophil
ATP1B3	0	0.565767698	0.882	0.673	0	Neutrophil
INSIG1	0	0.56269317	0.768	0.553	0	Neutrophil
S100A12	0	0.562634109	0.609	0.229	0	Neutrophil
TLR2	0	0.555990939	0.718	0.395	0	Neutrophil
LYN	0	0.553087934	0.776	0.493	0	Neutrophil
HBEGF	0	0.549521355	0.709	0.466	0	Neutrophil
CD36	0	0.548720309	0.733	0.446	0	Neutrophil
C15orf48	0	0.546449192	0.713	0.401	0	Neutrophil
RNASET2	0	0.542173999	0.87	0.673	0	Neutrophil
VEGFA	0	0.539428973	0.785	0.462	0	Neutrophil
CSF1R	0	0.538403753	0.855	0.402	0	Neutrophil
TREM1	0	0.53583977	0.712	0.421	0	Neutrophil
ASAH1	0	0.530879945	0.806	0.577	0	Neutrophil

TMEM176A	0	0.529502111	0.865	0.459	0	Neutrophil
NPC2	0	0.525089801	0.915	0.708	0	Neutrophil
LILRB2	0	0.522727323	0.754	0.377	0	Neutrophil
CCDC88A	0	0.518840031	0.831	0.515	0	Neutrophil
PPT1	0	0.516103625	0.847	0.509	0	Neutrophil
SLC8A1	0	0.516098773	0.794	0.48	0	Neutrophil
DSE	0	0.515168776	0.819	0.45	0	Neutrophil
EMILIN2	0	0.513794151	0.781	0.449	0	Neutrophil
LY86	0	0.511733037	0.811	0.529	0	Neutrophil
GCA	0	0.508696382	0.718	0.496	0	Neutrophil
ZNF331	0	0.507483764	0.834	0.658	0	Neutrophil
APOBEC3A	0	0.507052044	0.631	0.35	0	Neutrophil
NR4A3	0	0.506319735	0.809	0.51	0	Neutrophil
FCGR2B	0	0.504515767	0.809	0.555	0	Neutrophil
LAPTM5	0	0.503015477	0.845	0.71	0	Neutrophil
CD163	0	0.492646511	0.75	0.477	0	Neutrophil
CTSZ	0	0.491780762	0.879	0.684	0	Neutrophil
SAMSN1	0	0.487082518	0.846	0.702	0	Neutrophil
FBP1	0	0.486049635	0.748	0.477	0	Neutrophil
LAP3	0	0.482750361	0.874	0.603	0	Neutrophil
ATP6V1B2	0	0.482727356	0.758	0.458	0	Neutrophil
CYBA	0	0.482346047	0.967	0.903	0	Neutrophil
CD86	0	0.474227477	0.724	0.467	0	Neutrophil
FTL	0	0.473153378	0.996	0.98	0	Neutrophil
TGFBI	0	0.471778422	0.806	0.508	0	Neutrophil
RASGEF1B	0	0.463702144	0.809	0.517	0	Neutrophil
MX2	0	0.4625227	0.746	0.533	0	Neutrophil
CFP	0	0.456288163	0.707	0.425	0	Neutrophil
NCF4	0	0.454158761	0.82	0.58	0	Neutrophil
AOAH	0	0.448328884	0.769	0.492	0	Neutrophil
LILRB3	0	0.442409418	0.657	0.324	0	Neutrophil
LILRB1	0	0.44234033	0.753	0.396	0	Neutrophil
NLRP3	0	0.442335174	0.778	0.483	0	Neutrophil
FYB1	0	0.441924583	0.891	0.705	0	Neutrophil
LAIR1	0	0.44090717	0.745	0.423	0	Neutrophil
MRC1	0	0.436544042	0.813	0.554	0	Neutrophil
RAB31	0	0.433834005	0.773	0.552	0	Neutrophil
MARCO	0	0.433263198	0.67	0.409	0	Neutrophil
CAPG	0	0.431781348	0.79	0.507	0	Neutrophil
SRGN	0	0.43159318	0.944	0.892	0	Neutrophil
CLEC12A	0	0.431199317	0.692	0.338	0	Neutrophil
RAB32	0	0.430337468	0.794	0.507	0	Neutrophil
SLC7A7	0	0.425045086	0.766	0.355	0	Neutrophil
RASSF4	0	0.421966529	0.793	0.43	0	Neutrophil
ADAP2	0	0.420374276	0.786	0.497	0	Neutrophil
SERPINB9	0	0.419416916	0.802	0.472	0	Neutrophil
ACSL1	0	0.416139926	0.694	0.413	0	Neutrophil
CD300E	0	0.413127341	0.706	0.283	0	Neutrophil
HAVCR2	0	0.410714219	0.718	0.412	0	Neutrophil
FTH1	0	0.406248347	0.992	0.975	0	Neutrophil
TBXAS1	0	0.404071915	0.725	0.494	0	Neutrophil
PDXK	0	0.40356293	0.757	0.526	0	Neutrophil
RNF144B	0	0.401232618	0.738	0.445	0	Neutrophil
ALOX5	0	0.399956679	0.685	0.409	0	Neutrophil
EPB41L3	0	0.398949661	0.754	0.381	0	Neutrophil
MFSD1	0	0.397336383	0.825	0.49	0	Neutrophil

IL18	0	0.392598604	0.753	0.435	0	Neutrophil
SNX10	0	0.386638302	0.718	0.479	0	Neutrophil
OTUD1	0	0.385311897	0.737	0.45	0	Neutrophil
JDP2	0	0.383572992	0.773	0.497	0	Neutrophil
C1QA	0	0.37950037	0.836	0.598	0	Neutrophil
MIR3945HG	0	0.377267054	0.804	0.311	0	Neutrophil
ELL2	0	0.369671551	0.754	0.5	0	Neutrophil
LAT2	0	0.368424652	0.768	0.403	0	Neutrophil
SHTN1	0	0.364447826	0.726	0.428	0	Neutrophil
P2RY13	0	0.363428829	0.722	0.434	0	Neutrophil
IRF8	0	0.360699572	0.76	0.538	0	Neutrophil
PAK1	0	0.360233303	0.752	0.466	0	Neutrophil
LILRB4	0	0.356932933	0.717	0.408	0	Neutrophil
MARCH1	0	0.355548429	0.698	0.472	0	Neutrophil
TFRC	0	0.349132352	0.759	0.479	0	Neutrophil
CD300A	0	0.348945748	0.659	0.297	0	Neutrophil
RIPK2	0	0.342299503	0.737	0.482	0	Neutrophil
GAPT	0	0.341572663	0.728	0.374	0	Neutrophil
KCTD12	0	0.340048157	0.782	0.47	0	Neutrophil
VSIG4	0	0.331693684	0.74	0.45	0	Neutrophil
BMP2K	0	0.325134567	0.785	0.492	0	Neutrophil
LILRA5	0	0.324714888	0.607	0.172	0	Neutrophil
LRRC25	0	0.323980335	0.669	0.284	0	Neutrophil
MS4A4A	0	0.322074271	0.763	0.462	0	Neutrophil
GNA15	0	0.319386527	0.808	0.375	0	Neutrophil
TFEC	0	0.316367325	0.752	0.408	0	Neutrophil
CREB5	0	0.313622396	0.655	0.39	0	Neutrophil
FCGR1B	0	0.31254704	0.716	0.34	0	Neutrophil
CD33	0	0.308192189	0.733	0.394	0	Neutrophil
DSC2	0	0.302552504	0.784	0.331	0	Neutrophil
SAP30	0	0.297552537	0.828	0.594	0	Neutrophil
B3GNT5	0	0.295516356	0.745	0.449	0	Neutrophil
GRASP	0	0.294388676	0.813	0.558	0	Neutrophil
CD84	0	0.291408162	0.738	0.493	0	Neutrophil
SLC16A10	0	0.287620045	0.703	0.401	0	Neutrophil
C1QC	0	0.280112337	0.835	0.525	0	Neutrophil
CD4	0	0.270709284	0.732	0.435	0	Neutrophil
MIR181A1HG	0	0.268046027	0.734	0.486	0	Neutrophil
FPR3	0	0.264791964	0.802	0.313	0	Neutrophil
NRG1	0	0.259932792	0.77	0.22	0	Neutrophil
ASGR2	0	0.258885968	0.687	0.145	0	Neutrophil
CXorf21	4.80E-307	0.270822412	0.687	0.464	9.59E-304	Neutrophil
RNASE6	5.55E-307	0.37443175	0.713	0.484	1.11E-303	Neutrophil
GLRX	2.61E-305	0.411899945	0.84	0.644	5.23E-302	Neutrophil
SDS	5.30E-303	0.336773709	0.63	0.288	1.06E-299	Neutrophil
RETN	1.14E-300	0.323836552	0.588	0.181	2.29E-297	Neutrophil
POU2F2	1.34E-297	0.346932277	0.726	0.532	2.68E-294	Neutrophil
ATP2B1-AS1	5.32E-296	0.513321785	0.711	0.511	1.06E-292	Neutrophil
BLVRB	3.42E-291	0.475186375	0.777	0.599	6.85E-288	Neutrophil
RGS10	1.81E-288	0.340862233	0.884	0.615	3.62E-285	Neutrophil
CKS2	6.56E-286	0.264876004	0.812	0.662	1.31E-282	Neutrophil
LCP1	3.37E-283	0.435213262	0.872	0.74	6.74E-280	Neutrophil
CXCL16	1.05E-282	0.39458258	0.723	0.496	2.10E-279	Neutrophil
OGFRL1	4.00E-277	0.378862583	0.704	0.493	8.01E-274	Neutrophil
FABP5	8.28E-270	0.374453229	0.843	0.642	1.66E-266	Neutrophil
ACP5	1.71E-266	0.252320898	0.699	0.493	3.42E-263	Neutrophil

UCP2	1.83E-265	0.325073391	0.796	0.565	3.67E-262	Neutrophil
ABHD5	3.89E-258	0.377658705	0.731	0.533	7.79E-255	Neutrophil
VCAN	7.79E-257	0.536396439	0.753	0.516	1.56E-253	Neutrophil
EREG	1.61E-255	0.529522335	0.594	0.249	3.21E-252	Neutrophil
CTSL	3.36E-253	0.37644942	0.805	0.606	6.73E-250	Neutrophil
EIF4A3	9.00E-250	0.350013867	0.86	0.69	1.80E-246	Neutrophil
PILRA	1.19E-246	0.505625906	0.653	0.393	2.37E-243	Neutrophil
NR4A2	1.70E-244	0.392938359	0.835	0.619	3.40E-241	Neutrophil
OSM	2.29E-242	0.383932756	0.637	0.394	4.57E-239	Neutrophil
PLEKHO1	5.49E-242	0.2960186	0.848	0.638	1.10E-238	Neutrophil
CXCL3	9.79E-241	0.588981321	0.648	0.388	1.96E-237	Neutrophil
NFIL3	6.73E-230	0.444752127	0.717	0.563	1.35E-226	Neutrophil
NINJ1	1.54E-226	0.353551417	0.729	0.497	3.08E-223	Neutrophil
CDKN1A	2.97E-222	0.290507756	0.837	0.646	5.94E-219	Neutrophil
STX11	2.53E-221	0.303854005	0.706	0.489	5.06E-218	Neutrophil
SYAP1	3.29E-211	0.369662911	0.765	0.545	6.58E-208	Neutrophil
HNMT	7.74E-211	0.333401241	0.729	0.547	1.55E-207	Neutrophil
SCIMP	1.05E-206	0.268904687	0.608	0.355	2.11E-203	Neutrophil
ARHGAP18	4.58E-206	0.291158547	0.804	0.64	9.17E-203	Neutrophil
SLC31A2	2.82E-205	0.359089554	0.653	0.459	5.64E-202	Neutrophil
PTX3	3.30E-203	0.270319915	0.584	0.303	6.59E-200	Neutrophil
HCK	1.16E-202	0.424623567	0.642	0.459	2.33E-199	Neutrophil
RBM47	4.21E-201	0.346489756	0.631	0.361	8.42E-198	Neutrophil
CD55	7.44E-201	0.390413843	0.77	0.627	1.49E-197	Neutrophil
CLEC4A	1.60E-200	0.321681213	0.629	0.426	3.21E-197	Neutrophil
ASGR1	3.05E-197	0.436298808	0.593	0.279	6.10E-194	Neutrophil
TIMP1	3.00E-192	0.33593216	0.916	0.79	5.99E-189	Neutrophil
MARCKS	3.77E-188	0.377651543	0.812	0.704	7.55E-185	Neutrophil
BST1	4.27E-184	0.287571561	0.607	0.377	8.54E-181	Neutrophil
RAB20	5.07E-182	0.4388886	0.645	0.481	1.01E-178	Neutrophil
NFKBIA	3.45E-179	0.266860223	0.953	0.907	6.90E-176	Neutrophil
PFKFB3	7.64E-176	0.272480483	0.703	0.543	1.53E-172	Neutrophil
S100A4	8.12E-176	0.258328583	0.95	0.89	1.62E-172	Neutrophil
C3AR1	6.54E-168	0.268013381	0.646	0.422	1.31E-164	Neutrophil
LRP1	1.38E-163	0.261524452	0.674	0.453	2.76E-160	Neutrophil
UPP1	1.32E-162	0.320298892	0.739	0.568	2.63E-159	Neutrophil
ATF3	3.55E-161	0.331588241	0.801	0.62	7.10E-158	Neutrophil
BASP1	7.84E-159	0.446168354	0.666	0.527	1.57E-155	Neutrophil
GADD45B	7.79E-158	0.304335158	0.875	0.738	1.56E-154	Neutrophil
MPEG1	1.83E-157	0.3836369	0.606	0.355	3.65E-154	Neutrophil
RAB11FIP1	6.76E-153	0.339585855	0.722	0.558	1.35E-149	Neutrophil
HERPUD1	1.21E-151	0.27826242	0.929	0.832	2.42E-148	Neutrophil
CSTB	1.05E-146	0.254998406	0.886	0.719	2.09E-143	Neutrophil
LGALS9	1.71E-135	0.333276871	0.672	0.553	3.41E-132	Neutrophil
CCL3	8.31E-135	0.440756563	0.718	0.614	1.66E-131	Neutrophil
CSF3R	2.08E-133	0.413905748	0.559	0.29	4.16E-130	Neutrophil
HCST	1.83E-126	0.272928259	0.88	0.77	3.66E-123	Neutrophil
CYCS	1.47E-119	0.268519951	0.872	0.753	2.95E-116	Neutrophil
CTSD	2.10E-117	0.251137953	0.835	0.667	4.20E-114	Neutrophil
SLC16A3	6.68E-104	0.285197371	0.647	0.525	1.34E-100	Neutrophil
EVI2B	4.87E-101	0.296323313	0.728	0.649	9.75E-98	Neutrophil
STXBP2	1.63E-100	0.377664043	0.596	0.446	3.26E-97	Neutrophil
HSPA1A	4.12E-99	0.26185407	0.901	0.746	8.23E-96	Neutrophil
ANPEP	1.61E-93	0.352729771	0.583	0.464	3.22E-90	Neutrophil
FGR	7.42E-90	0.341485342	0.553	0.372	1.48E-86	Neutrophil
CSF2RA	2.76E-89	0.250223187	0.545	0.259	5.52E-86	Neutrophil

TNFAIP2	7.39E-88	0.320768415	0.607	0.462	1.48E-84	Neutrophil
BID	8.91E-79	0.305160867	0.653	0.573	1.78E-75	Neutrophil
KYNU	1.69E-69	0.275296451	0.573	0.43	3.39E-66	Neutrophil
THEMIS2	7.73E-68	0.321226489	0.56	0.409	1.55E-64	Neutrophil
CCL3L1	7.16E-65	0.342700254	0.61	0.521	1.43E-61	Neutrophil
LILRA2	2.53E-57	0.298765929	0.524	0.339	5.06E-54	Neutrophil
C19orf38	9.76E-49	0.270365666	0.535	0.426	1.95E-45	Neutrophil
G0S2	2.36E-35	0.586216525	0.509	0.402	4.73E-32	Neutrophil
AQP9	9.02E-34	0.275989055	0.48	0.244	1.80E-30	Neutrophil
MCEMP1	5.59E-24	0.283990369	0.45	0.098	1.12E-20	Neutrophil
DMXL2	7.43E-23	0.270938565	0.506	0.367	1.49E-19	Neutrophil
SMIM25	9.70E-21	0.29792704	0.485	0.278	1.94E-17	Neutrophil
GZMK	0	1.500403246	0.943	0.703	0	NK/T_cell
CCL5	0	1.476657901	0.978	0.765	0	NK/T_cell
NKG7	0	1.401458245	0.89	0.603	0	NK/T_cell
GZMA	0	1.32900695	0.956	0.741	0	NK/T_cell
CCL4	0	1.221520366	0.949	0.803	0	NK/T_cell
CST7	0	1.19624023	0.921	0.71	0	NK/T_cell
DUSP2	0	0.971743191	0.925	0.783	0	NK/T_cell
IL32	0	0.957845673	0.954	0.79	0	NK/T_cell
TRBC2	0	0.922912701	0.883	0.764	0	NK/T_cell
CD69	0	0.895813405	0.916	0.765	0	NK/T_cell
GZMH	0	0.884482485	0.657	0.596	0	NK/T_cell
CTSW	0	0.850395749	0.647	0.48	0	NK/T_cell
CCL4L2	0	0.83938489	0.798	0.721	0	NK/T_cell
TRAC	0	0.838899611	0.871	0.772	0	NK/T_cell
CD8A	0	0.831228443	0.662	0.597	0	NK/T_cell
XCL2	0	0.825726758	0.616	0.511	0	NK/T_cell
CD2	0	0.80633297	0.83	0.745	0	NK/T_cell
GZMM	0	0.802770435	0.749	0.632	0	NK/T_cell
CD3D	0	0.800380721	0.851	0.746	0	NK/T_cell
CD8B	0	0.799749138	0.696	0.538	0	NK/T_cell
CXCR4	0	0.797632677	0.958	0.841	0	NK/T_cell
CD3G	0	0.742826264	0.797	0.7	0	NK/T_cell
HCST	0	0.726297786	0.897	0.755	0	NK/T_cell
CD3E	0	0.689664228	0.728	0.695	0	NK/T_cell
CORO1A	0	0.684041614	0.883	0.753	0	NK/T_cell
CRTAM	0	0.646905743	0.66	0.523	0	NK/T_cell
CD52	0	0.621972223	0.861	0.791	0	NK/T_cell
TRBC1	0	0.618489048	0.683	0.678	0	NK/T_cell
CD48	0	0.567239913	0.818	0.767	0	NK/T_cell
RGCC	0	0.546057013	0.853	0.794	0	NK/T_cell
ITM2C	0	0.517041821	0.6	0.562	0	NK/T_cell
LCP1	0	0.497185906	0.795	0.741	0	NK/T_cell
EZR	0	0.495005913	0.845	0.774	0	NK/T_cell
CYTIP	0	0.486965767	0.794	0.781	0	NK/T_cell
ISG20	0	0.485548842	0.742	0.712	0	NK/T_cell
IRF1	0	0.483451792	0.822	0.769	0	NK/T_cell
DDIT4	0	0.474609174	0.854	0.794	0	NK/T_cell
ALOX5AP	0	0.473892192	0.707	0.676	0	NK/T_cell
TNFSF9	0	0.459054708	0.582	0.514	0	NK/T_cell
ITM2A	0	0.444796184	0.731	0.732	0	NK/T_cell
TRGC2	0	0.444788095	0.579	0.593	0	NK/T_cell
SRGN	0	0.424197073	0.969	0.881	0	NK/T_cell
STMN1	0	0.36471483	0.694	0.602	0	NK/T_cell
FYB1	0	0.363808156	0.754	0.712	0	NK/T_cell

TNFRSF9	0	0.255392391	0.539	0.513	0	NK/T_cell
MATK	1.54E-288	0.465238291	0.517	0.45	3.08E-285	NK/T_cell
SOCS1	9.74E-285	0.442858501	0.698	0.719	1.95E-281	NK/T_cell
GZMB	3.03E-270	0.479706856	0.567	0.603	6.06E-267	NK/T_cell
GNLY	5.73E-241	0.567378163	0.519	0.495	1.15E-237	NK/T_cell
TNF	7.74E-235	0.512649605	0.611	0.631	1.55E-231	NK/T_cell
GBP5	3.15E-229	0.406103092	0.575	0.573	6.29E-226	NK/T_cell
ITGB2	1.29E-223	0.311724283	0.742	0.718	2.58E-220	NK/T_cell
IFNG	4.86E-223	0.593474794	0.555	0.605	9.71E-220	NK/T_cell
KLRD1	4.42E-219	0.554662072	0.514	0.5	8.83E-216	NK/T_cell
PRF1	6.96E-209	0.466513415	0.506	0.471	1.39E-205	NK/T_cell
XCL1	6.95E-206	0.634005352	0.482	0.46	1.39E-202	NK/T_cell
RHOH	7.23E-189	0.399954439	0.646	0.703	1.45E-185	NK/T_cell
CD37	4.35E-183	0.303976877	0.728	0.771	8.70E-180	NK/T_cell
RGS1	1.27E-176	0.355653845	0.717	0.755	2.54E-173	NK/T_cell
DNAJB1	1.68E-167	0.293052607	0.758	0.779	3.37E-164	NK/T_cell
CD247	2.47E-159	0.491125467	0.559	0.605	4.93E-156	NK/T_cell
PRDM1	6.73E-158	0.35395899	0.625	0.669	1.35E-154	NK/T_cell
DUSP4	1.71E-157	0.379772937	0.55	0.567	3.41E-154	NK/T_cell
CD27	4.46E-143	0.370202779	0.551	0.616	8.92E-140	NK/T_cell
TNFAIP3	2.79E-128	0.31014159	0.667	0.698	5.59E-125	NK/T_cell
C12orf75	1.18E-127	0.269241889	0.585	0.571	2.37E-124	NK/T_cell
TRDC	3.90E-116	0.328150136	0.434	0.414	7.80E-113	NK/T_cell
IL2RB	2.47E-76	0.317940613	0.452	0.502	4.95E-73	NK/T_cell
TNFSF14	6.31E-64	0.253147691	0.438	0.481	1.26E-60	NK/T_cell
CXCR6	7.01E-64	0.323498743	0.458	0.5	1.40E-60	NK/T_cell
TENT5C	1.36E-61	0.374144388	0.532	0.621	2.73E-58	NK/T_cell
KLRC1	2.63E-60	0.294048866	0.44	0.482	5.27E-57	NK/T_cell
TIGIT	1.20E-55	0.352595442	0.508	0.618	2.40E-52	NK/T_cell
HOPX	2.29E-51	0.40189562	0.525	0.616	4.58E-48	NK/T_cell
CXCR3	2.29E-44	0.359816833	0.487	0.592	4.58E-41	NK/T_cell
CD7	9.42E-26	0.496922051	0.48	0.588	1.88E-22	NK/T_cell
PTTG1	6.42E-24	0.277604099	0.483	0.572	1.28E-20	NK/T_cell
OASL	2.16E-22	0.326523453	0.455	0.54	4.33E-19	NK/T_cell
SPOCK2	1.01E-15	0.278887249	0.503	0.621	2.01E-12	NK/T_cell
IL7R	0	1.343007577	0.889	0.682	0	T_cell
LTB	0	1.041623497	0.874	0.708	0	T_cell
CD2	0	0.990294658	0.879	0.731	0	T_cell
IL32	0	0.891943309	0.903	0.797	0	T_cell
TRAC	0	0.858134475	0.873	0.768	0	T_cell
TRBC2	0	0.8210102	0.855	0.767	0	T_cell
CD3D	0	0.819675972	0.84	0.745	0	T_cell
CD69	0	0.817023989	0.884	0.767	0	T_cell
CD52	0	0.747867123	0.879	0.785	0	T_cell
TRBC1	0	0.747548045	0.718	0.67	0	T_cell
CD3E	0	0.747399313	0.753	0.688	0	T_cell
KLRB1	0	0.721289276	0.714	0.598	0	T_cell
CXCR4	0	0.712322173	0.922	0.846	0	T_cell
CD3G	0	0.702176266	0.754	0.707	0	T_cell
CYTIP	0	0.676431654	0.852	0.767	0	T_cell
SPOCK2	0	0.65501576	0.664	0.587	0	T_cell
CCR7	0	0.653008128	0.625	0.624	0	T_cell
SOCS1	0	0.600002667	0.787	0.699	0	T_cell
BATF	0	0.574729365	0.701	0.55	0	T_cell
RHOH	0	0.574723259	0.719	0.688	0	T_cell
DDIT4	0	0.549911455	0.871	0.788	0	T_cell

TNFAIP3	0	0.545469426	0.749	0.68	0	T_cell
CD48	0	0.534955265	0.786	0.772	0	T_cell
BIRC3	0	0.515710352	0.713	0.649	0	T_cell
SAMSN1	0	0.507355946	0.77	0.698	0	T_cell
CD40LG	0	0.507267752	0.565	0.519	0	T_cell
ISG20	0	0.500218875	0.768	0.705	0	T_cell
FYB1	0	0.485351849	0.771	0.706	0	T_cell
CORO1A	0	0.467784241	0.812	0.766	0	T_cell
EZR	0	0.449073701	0.825	0.776	0	T_cell
RGCC	0	0.438691544	0.848	0.794	0	T_cell
PRDM1	0	0.438123653	0.674	0.659	0	T_cell
DUSP2	0	0.411262573	0.849	0.796	0	T_cell
ITM2A	0	0.3960472	0.767	0.723	0	T_cell
GZMA	0	0.390810761	0.78	0.775	0	T_cell
TENT5C	0	0.374947339	0.626	0.601	0	T_cell
IRF1	0	0.373795757	0.805	0.772	0	T_cell
ALOX5AP	0	0.372823427	0.731	0.669	0	T_cell
CD37	0	0.372265732	0.764	0.764	0	T_cell
MAL	0	0.367281894	0.511	0.437	0	T_cell
GBP5	0	0.34703957	0.624	0.562	0	T_cell
SRGN	0	0.32861975	0.922	0.889	0	T_cell
HIST1H1D	0	0.302612147	0.595	0.52	0	T_cell
TNFRSF18	0	0.273740522	0.634	0.603	0	T_cell
CCR6	0	0.262957868	0.571	0.564	0	T_cell
TNFRSF4	1.59E-305	0.290897464	0.614	0.63	3.19E-302	T_cell
HCST	5.14E-295	0.31020969	0.82	0.769	1.03E-291	T_cell
PIM2	3.00E-267	0.339311783	0.543	0.477	6.01E-264	T_cell
DUSP5	2.93E-199	0.254869583	0.662	0.631	5.87E-196	T_cell
RGS1	3.60E-197	0.320488849	0.74	0.751	7.20E-194	T_cell
CD247	4.16E-172	0.374342644	0.574	0.603	8.32E-169	T_cell
SLAMF1	7.86E-146	0.301639717	0.529	0.594	1.57E-142	T_cell
GPR183	3.77E-145	0.279282615	0.708	0.7	7.53E-142	T_cell
CXCR3	2.08E-114	0.270634526	0.536	0.584	4.16E-111	T_cell
TNF	1.78E-79	0.301162229	0.595	0.635	3.57E-76	T_cell
SELL	6.13E-73	0.280889681	0.436	0.427	1.23E-69	T_cell
CD7	1.46E-69	0.395777195	0.522	0.582	2.92E-66	T_cell
EVI2B	1.92E-66	0.250143716	0.607	0.666	3.83E-63	T_cell
PDE4B	8.29E-62	0.260311663	0.549	0.569	1.66E-58	T_cell
ICOS	8.81E-56	0.391766678	0.468	0.5	1.76E-52	T_cell
AQP3	2.67E-33	0.330289642	0.492	0.576	5.33E-30	T_cell
CD79A	0	1.55589209	0.928	0.571	0	B_cell
MS4A1	0	1.261628362	0.756	0.613	0	B_cell
BANK1	0	0.992152775	0.623	0.516	0	B_cell
IGHA1	0	0.892083991	0.851	0.831	0	B_cell
CD83	0	0.831354041	0.739	0.609	0	B_cell
IGKC	0	0.715967583	0.983	0.979	0	B_cell
CD37	0	0.70067129	0.862	0.759	0	B_cell
CD74	0	0.561132954	0.989	0.893	0	B_cell
EZR	0	0.526613639	0.886	0.78	0	B_cell
CYBA	0	0.413428524	0.975	0.904	0	B_cell
HLA-DQA1	2.34E-291	0.689746758	0.794	0.655	4.67E-288	B_cell
HLA-DQB1	4.01E-280	0.575361724	0.826	0.678	8.02E-277	B_cell
HLA-DRA	4.25E-276	0.689692211	0.902	0.811	8.49E-273	B_cell
BASP1	4.56E-266	0.574417575	0.668	0.53	9.13E-263	B_cell
HERPUD1	6.22E-262	0.449478721	0.861	0.838	1.24E-258	B_cell
HLA-DPA1	6.90E-246	0.509834996	0.915	0.798	1.38E-242	B_cell

ISG20	5.50E-236	0.484899442	0.791	0.713	1.10E-232	B_cell
IGLC2	2.61E-229	0.68443726	0.9	0.916	5.22E-226	B_cell
TNFRSF13C	1.23E-203	0.747558289	0.575	0.545	2.46E-200	B_cell
HLA-DPB1	2.76E-191	0.506525707	0.846	0.787	5.53E-188	B_cell
HLA-DOB	1.15E-189	0.390843881	0.481	0.381	2.31E-186	B_cell
FCRL5	4.16E-179	0.381029471	0.511	0.452	8.32E-176	B_cell
FAM30A	1.89E-178	0.60484323	0.536	0.513	3.78E-175	B_cell
LTB	2.05E-176	0.642483857	0.746	0.739	4.10E-173	B_cell
POU2F2	5.64E-176	0.476753786	0.585	0.544	1.13E-172	B_cell
CD55	7.40E-173	0.43168928	0.716	0.633	1.48E-169	B_cell
HLA-DRB1	5.24E-170	0.384736273	0.89	0.786	1.05E-166	B_cell
FCRLA	3.20E-164	0.469880095	0.486	0.388	6.40E-161	B_cell
PDE4B	9.37E-151	0.356379404	0.652	0.561	1.87E-147	B_cell
CTSH	2.19E-145	0.334207935	0.699	0.607	4.37E-142	B_cell
BTG2	2.71E-143	0.329945429	0.822	0.782	5.43E-140	B_cell
VPREB3	4.23E-141	0.713049253	0.509	0.461	8.46E-138	B_cell
HLA-DMA	4.75E-136	0.327788198	0.751	0.648	9.50E-133	B_cell
CD24	1.16E-123	0.52575317	0.461	0.447	2.32E-120	B_cell
EAF2	4.74E-123	0.318175261	0.516	0.501	9.49E-120	B_cell
CD52	2.40E-122	0.416992262	0.809	0.802	4.80E-119	B_cell
HLA-DQA2	6.35E-115	0.494939735	0.697	0.712	1.27E-111	B_cell
RHOH	6.42E-112	0.371092819	0.688	0.694	1.28E-108	B_cell
HLA-DRB5	1.33E-110	0.381712849	0.824	0.796	2.66E-107	B_cell
MARCKS	2.29E-108	0.326511617	0.78	0.708	4.58E-105	B_cell
PNOC	2.91E-102	0.277806684	0.444	0.379	5.82E-99	B_cell
CD27	5.23E-99	0.359690021	0.577	0.607	1.05E-95	B_cell
LINC01857	2.95E-97	0.40093314	0.393	0.371	5.89E-94	B_cell
SEC11C	1.53E-95	0.406833769	0.624	0.64	3.07E-92	B_cell
POU2AF1	2.63E-95	0.333683235	0.444	0.413	5.26E-92	B_cell
CXCR4	7.36E-95	0.293765388	0.913	0.858	1.47E-91	B_cell
EVI2B	2.50E-94	0.299672799	0.687	0.653	5.00E-91	B_cell
IRF8	1.03E-88	0.50111708	0.534	0.556	2.07E-85	B_cell
FCRL2	2.74E-88	0.280404248	0.402	0.369	5.49E-85	B_cell
CD48	8.72E-88	0.326918483	0.759	0.776	1.74E-84	B_cell
SPIB	1.34E-87	0.576911137	0.44	0.43	2.69E-84	B_cell
RNASET2	1.95E-86	0.253739818	0.751	0.684	3.90E-83	B_cell
ODC1	2.43E-82	0.329247458	0.627	0.636	4.86E-79	B_cell
TNFSF9	2.03E-73	0.350162271	0.491	0.527	4.06E-70	B_cell
CD70	9.58E-64	0.440510225	0.457	0.511	1.92E-60	B_cell
CD40	1.20E-62	0.316956357	0.47	0.482	2.40E-59	B_cell
LY86	2.19E-61	0.386213762	0.52	0.551	4.38E-58	B_cell
NCF1	1.30E-59	0.30109612	0.563	0.589	2.60E-56	B_cell
MZB1	3.14E-59	0.891484079	0.531	0.651	6.28E-56	B_cell
BIRC3	1.31E-58	0.274564141	0.652	0.662	2.62E-55	B_cell
BCL11A	6.22E-58	0.422033468	0.402	0.419	1.24E-54	B_cell
TNFRSF17	6.67E-56	0.27898779	0.451	0.497	1.33E-52	B_cell
RAB11FIP1	3.10E-52	0.288152459	0.55	0.571	6.19E-49	B_cell
SCIMP	1.06E-51	0.312610839	0.359	0.374	2.12E-48	B_cell
SELL	6.84E-46	0.28011451	0.403	0.43	1.37E-42	B_cell
DERL3	2.49E-38	0.631855364	0.443	0.509	4.98E-35	B_cell
BCL2A1	7.25E-34	0.333862157	0.463	0.518	1.45E-30	B_cell
CCR7	3.25E-30	0.548929002	0.521	0.63	6.50E-27	B_cell
PIM2	1.30E-24	0.264510204	0.434	0.492	2.61E-21	B_cell
IGHM	2.38E-24	0.941729645	0.462	0.611	4.75E-21	B_cell
TNFRSF13B	4.14E-23	0.519443691	0.439	0.543	8.27E-20	B_cell
MARCH1	4.93E-21	0.308022498	0.392	0.493	9.85E-18	B_cell

IGLC3	4.21E-17	0.621093803	0.589	0.746	8.42E-14	B_cell
JCHAIN	4.07E-14	0.852978453	0.545	0.726	8.14E-11	B_cell
IGHG4	1.09E-12	0.512777474	0.282	0.496	2.17E-09	B_cell
IGHG3	1.17E-11	0.724280883	0.533	0.72	2.33E-08	B_cell
LINC01781	2.70E-08	0.443111711	0.396	0.473	5.39E-05	B_cell
IGHG1	4.81E-08	0.806920098	0.516	0.692	9.63E-05	B_cell
IGLV3-1	9.54E-07	0.327823307	0.392	0.542	0.001907841	B_cell
TOR3A	4.75E-06	0.260615968	0.394	0.522	0.009502471	B_cell
C1QA	0	1.642090111	0.967	0.56	0	Monocyte/Macrophage
C1QB	0	1.604876555	0.943	0.481	0	Monocyte/Macrophage
C1QC	0	1.578789773	0.954	0.484	0	Monocyte/Macrophage
AIF1	0	1.49996324	0.991	0.489	0	Monocyte/Macrophage
TYROBP	0	1.48865002	0.991	0.52	0	Monocyte/Macrophage
CD14	0	1.472112797	0.965	0.494	0	Monocyte/Macrophage
FCER1G	0	1.461131539	0.986	0.502	0	Monocyte/Macrophage
HLA-DQA1	0	1.36088716	0.987	0.611	0	Monocyte/Macrophage
HLA-DRA	0	1.310191919	0.999	0.787	0	Monocyte/Macrophage
LYZ	0	1.298737422	0.968	0.506	0	Monocyte/Macrophage
CCL3	0	1.262074145	0.909	0.576	0	Monocyte/Macrophage
MS4A6A	0	1.255581313	0.964	0.487	0	Monocyte/Macrophage
HLA-DQB1	0	1.24145236	0.991	0.638	0	Monocyte/Macrophage
HLA-DRB5	0	1.237237921	0.998	0.766	0	Monocyte/Macrophage
CD68	0	1.236239863	0.967	0.433	0	Monocyte/Macrophage
CTSB	0	1.226159154	0.971	0.534	0	Monocyte/Macrophage
HLA-DQA2	0	1.201037186	0.982	0.669	0	Monocyte/Macrophage
HLA-DPA1	0	1.198108108	0.997	0.774	0	Monocyte/Macrophage
HLA-DPB1	0	1.173654676	0.996	0.757	0	Monocyte/Macrophage
SPP1	0	1.168442818	0.811	0.598	0	Monocyte/Macrophage
MS4A7	0	1.149311252	0.874	0.412	0	Monocyte/Macrophage
IGSF6	0	1.140883369	0.953	0.474	0	Monocyte/Macrophage
FCGR2A	0	1.128998167	0.957	0.421	0	Monocyte/Macrophage
HLA-DRB1	0	1.124258385	0.999	0.759	0	Monocyte/Macrophage
HLA-DMA	0	1.123796845	0.985	0.601	0	Monocyte/Macrophage
FCGR3A	0	1.094769771	0.894	0.386	0	Monocyte/Macrophage
SGK1	0	1.093111188	0.969	0.559	0	Monocyte/Macrophage
TMEM176B	0	1.081649985	0.945	0.428	0	Monocyte/Macrophage
FOLR2	0	1.080975486	0.903	0.504	0	Monocyte/Macrophage
C5AR1	0	1.074252848	0.923	0.421	0	Monocyte/Macrophage
CTSZ	0	1.073019883	0.992	0.652	0	Monocyte/Macrophage
PLAUR	0	1.052024907	0.952	0.568	0	Monocyte/Macrophage
F13A1	0	1.040014843	0.927	0.495	0	Monocyte/Macrophage
CPVL	0	1.031397436	0.93	0.44	0	Monocyte/Macrophage
MAFB	0	1.023391335	0.946	0.525	0	Monocyte/Macrophage
SPI1	0	1.022872999	0.938	0.425	0	Monocyte/Macrophage
CTSS	0	1.0110082	0.985	0.636	0	Monocyte/Macrophage
CFD	0	1.006016042	0.897	0.408	0	Monocyte/Macrophage
PLTP	0	1.005272588	0.886	0.477	0	Monocyte/Macrophage
CYBB	0	1.002967273	0.944	0.464	0	Monocyte/Macrophage
PLEK	0	1.00150718	0.942	0.473	0	Monocyte/Macrophage
PSAP	0	0.977352071	0.988	0.623	0	Monocyte/Macrophage
CD163	0	0.975609966	0.884	0.437	0	Monocyte/Macrophage
MS4A4A	0	0.970914767	0.866	0.424	0	Monocyte/Macrophage
CTSL	0	0.970235443	0.916	0.574	0	Monocyte/Macrophage
CD83	0	0.964851708	0.943	0.565	0	Monocyte/Macrophage
HLA-DMB	0	0.955880729	0.959	0.491	0	Monocyte/Macrophage
CD74	0	0.947109482	0.999	0.882	0	Monocyte/Macrophage

GRN	0	0.939839977	0.966	0.604	0	Monocyte/Macrophage
VSIG4	0	0.938899148	0.9	0.404	0	Monocyte/Macrophage
CEBPD	0	0.929683563	0.98	0.655	0	Monocyte/Macrophage
SELENOP	0	0.929248747	0.891	0.63	0	Monocyte/Macrophage
FCGR1A	0	0.929192582	0.896	0.422	0	Monocyte/Macrophage
MRC1	0	0.923597666	0.903	0.521	0	Monocyte/Macrophage
HMOX1	0	0.92225567	0.872	0.484	0	Monocyte/Macrophage
MNDA	0	0.922056901	0.907	0.43	0	Monocyte/Macrophage
FABP5	0	0.909162378	0.928	0.614	0	Monocyte/Macrophage
FCGRT	0	0.905444916	0.961	0.577	0	Monocyte/Macrophage
SAT1	0	0.900398203	0.998	0.81	0	Monocyte/Macrophage
CCL3L1	0	0.894627192	0.794	0.486	0	Monocyte/Macrophage
GLUL	0	0.890835369	0.948	0.511	0	Monocyte/Macrophage
NPC2	0	0.890041121	0.99	0.682	0	Monocyte/Macrophage
MSR1	0	0.869497441	0.8	0.26	0	Monocyte/Macrophage
CXCL8	0	0.867144605	0.699	0.344	0	Monocyte/Macrophage
CST3	0	0.866606063	0.998	0.696	0	Monocyte/Macrophage
VAMP8	0	0.86322146	0.968	0.646	0	Monocyte/Macrophage
CEBPB	0	0.859598984	0.961	0.569	0	Monocyte/Macrophage
TYMP	0	0.859011757	0.969	0.619	0	Monocyte/Macrophage
RNASE1	0	0.856706858	0.82	0.589	0	Monocyte/Macrophage
C3AR1	0	0.856694999	0.86	0.372	0	Monocyte/Macrophage
CXCL3	0	0.85567206	0.798	0.345	0	Monocyte/Macrophage
APOE	0	0.848502595	0.681	0.449	0	Monocyte/Macrophage
LST1	0	0.837197402	0.907	0.455	0	Monocyte/Macrophage
CXCL16	0	0.830948403	0.869	0.456	0	Monocyte/Macrophage
NAMPT	0	0.824823436	0.97	0.596	0	Monocyte/Macrophage
CXCL2	0	0.823216683	0.822	0.4	0	Monocyte/Macrophage
S100A9	0	0.819522966	0.878	0.426	0	Monocyte/Macrophage
APOC1	0	0.815826919	0.633	0.354	0	Monocyte/Macrophage
LGMN	0	0.814097332	0.843	0.489	0	Monocyte/Macrophage
LILRB4	0	0.807451724	0.846	0.366	0	Monocyte/Macrophage
MFSD1	0	0.803454465	0.929	0.45	0	Monocyte/Macrophage
CTSD	0	0.79249471	0.93	0.64	0	Monocyte/Macrophage
CSF1R	0	0.792171817	0.905	0.361	0	Monocyte/Macrophage
TMEM176A	0	0.787993252	0.89	0.426	0	Monocyte/Macrophage
BLVRB	0	0.779664906	0.922	0.563	0	Monocyte/Macrophage
CTSH	0	0.772187172	0.937	0.561	0	Monocyte/Macrophage
CLEC7A	0	0.769490278	0.899	0.461	0	Monocyte/Macrophage
ADAP2	0	0.766634182	0.858	0.464	0	Monocyte/Macrophage
CSTB	0	0.761467881	0.963	0.695	0	Monocyte/Macrophage
RNASET2	0	0.757190369	0.962	0.644	0	Monocyte/Macrophage
IER3	0	0.756208834	0.942	0.635	0	Monocyte/Macrophage
LAPTM5	0	0.748681571	0.984	0.678	0	Monocyte/Macrophage
ITGB2	0	0.748139324	0.982	0.681	0	Monocyte/Macrophage
CAPG	0	0.747118988	0.851	0.477	0	Monocyte/Macrophage
DBI	0	0.741670257	0.979	0.723	0	Monocyte/Macrophage
KLF4	0	0.741249144	0.943	0.545	0	Monocyte/Macrophage
GPR183	0	0.741019299	0.941	0.664	0	Monocyte/Macrophage
GPR34	0	0.738454761	0.872	0.418	0	Monocyte/Macrophage
SERPINA1	0	0.733107958	0.876	0.379	0	Monocyte/Macrophage
RGS10	0	0.719454802	0.969	0.583	0	Monocyte/Macrophage
CCL4L2	0	0.715423392	0.892	0.709	0	Monocyte/Macrophage
FCGR2B	0	0.71467875	0.835	0.533	0	Monocyte/Macrophage
ARHGAP18	0	0.713115165	0.936	0.608	0	Monocyte/Macrophage
INSIG1	0	0.710225906	0.843	0.526	0	Monocyte/Macrophage

MARCKS	0	0.710159583	0.961	0.673	0	Monocyte/Macrophage
RGS2	0	0.708782786	0.9	0.616	0	Monocyte/Macrophage
PYCARD	0	0.70848954	0.951	0.642	0	Monocyte/Macrophage
RASSF4	0	0.704595278	0.87	0.392	0	Monocyte/Macrophage
C15orf48	0	0.700102076	0.751	0.372	0	Monocyte/Macrophage
RNASE6	0	0.70002572	0.83	0.449	0	Monocyte/Macrophage
ASAH1	0	0.688097978	0.892	0.547	0	Monocyte/Macrophage
CD86	0	0.674773063	0.823	0.433	0	Monocyte/Macrophage
DAB2	0	0.673253111	0.852	0.565	0	Monocyte/Macrophage
CCDC88A	0	0.672541161	0.916	0.479	0	Monocyte/Macrophage
GPNMB	0	0.665551757	0.719	0.405	0	Monocyte/Macrophage
IFI30	0	0.664290747	0.823	0.474	0	Monocyte/Macrophage
SOD2	0	0.662963398	0.932	0.55	0	Monocyte/Macrophage
CH25H	0	0.662223767	0.84	0.528	0	Monocyte/Macrophage
RASGEF1B	0	0.660576689	0.882	0.484	0	Monocyte/Macrophage
FBP1	0	0.655723598	0.752	0.456	0	Monocyte/Macrophage
PTPRE	0	0.654976002	0.87	0.502	0	Monocyte/Macrophage
TREM2	0	0.651080757	0.816	0.456	0	Monocyte/Macrophage
FGL2	0	0.6482068	0.889	0.464	0	Monocyte/Macrophage
LAIR1	0	0.645902732	0.827	0.387	0	Monocyte/Macrophage
LY86	0	0.643486156	0.859	0.501	0	Monocyte/Macrophage
PPT1	0	0.643273117	0.923	0.472	0	Monocyte/Macrophage
FTL	0	0.641043866	1	0.978	0	Monocyte/Macrophage
IL1B	0	0.633385623	0.697	0.358	0	Monocyte/Macrophage
VMO1	0	0.632139273	0.698	0.435	0	Monocyte/Macrophage
RAB32	0	0.629459717	0.853	0.477	0	Monocyte/Macrophage
TNFSF13B	0	0.628049991	0.892	0.542	0	Monocyte/Macrophage
IL18	0	0.623510456	0.857	0.396	0	Monocyte/Macrophage
CYBA	0	0.621685735	0.999	0.893	0	Monocyte/Macrophage
PLIN2	0	0.616173637	0.866	0.649	0	Monocyte/Macrophage
RGS1	0	0.615149006	0.945	0.718	0	Monocyte/Macrophage
PILRA	0	0.614191242	0.757	0.358	0	Monocyte/Macrophage
ATF3	0	0.613121543	0.899	0.591	0	Monocyte/Macrophage
OTUD1	0	0.608892919	0.811	0.418	0	Monocyte/Macrophage
NCF4	0	0.605401712	0.872	0.554	0	Monocyte/Macrophage
HNMT	0	0.60037337	0.837	0.517	0	Monocyte/Macrophage
CCL2	0	0.596343993	0.749	0.503	0	Monocyte/Macrophage
GK	0	0.594398814	0.775	0.419	0	Monocyte/Macrophage
EFHD2	0	0.585430043	0.912	0.58	0	Monocyte/Macrophage
PLD3	0	0.584566548	0.758	0.46	0	Monocyte/Macrophage
THBD	0	0.582747356	0.838	0.511	0	Monocyte/Macrophage
CHMP1B	0	0.579914532	0.875	0.498	0	Monocyte/Macrophage
CD84	0	0.57822683	0.838	0.46	0	Monocyte/Macrophage
TFRC	0	0.577026437	0.833	0.447	0	Monocyte/Macrophage
TKT	0	0.570467382	0.957	0.612	0	Monocyte/Macrophage
BCL2A1	0	0.569568565	0.807	0.469	0	Monocyte/Macrophage
ATP6V1B2	0	0.56361801	0.839	0.423	0	Monocyte/Macrophage
MARCO	0	0.561207551	0.692	0.387	0	Monocyte/Macrophage
LYN	0	0.560801603	0.846	0.461	0	Monocyte/Macrophage
CD36	0	0.560187596	0.775	0.419	0	Monocyte/Macrophage
CTSC	0	0.555736397	0.969	0.753	0	Monocyte/Macrophage
PHACTR1	0	0.555440435	0.735	0.438	0	Monocyte/Macrophage
C1orf162	0	0.553957373	0.799	0.518	0	Monocyte/Macrophage
TGFBI	0	0.551882245	0.807	0.486	0	Monocyte/Macrophage
OLR1	0	0.551432008	0.763	0.458	0	Monocyte/Macrophage
SLC1A3	0	0.550624605	0.837	0.269	0	Monocyte/Macrophage

FPR3	0	0.550527837	0.845	0.27	0	Monocyte/Macrophage
BMP2K	0	0.543743136	0.867	0.458	0	Monocyte/Macrophage
HAVCR2	0	0.542002828	0.775	0.381	0	Monocyte/Macrophage
NR4A3	0	0.538495973	0.847	0.483	0	Monocyte/Macrophage
ACP5	0	0.536866125	0.707	0.476	0	Monocyte/Macrophage
SHTN1	0	0.536323081	0.796	0.395	0	Monocyte/Macrophage
SLCO2B1	0	0.534855037	0.812	0.476	0	Monocyte/Macrophage
NPL	0	0.533584521	0.728	0.417	0	Monocyte/Macrophage
SLC16A10	0	0.532991102	0.819	0.361	0	Monocyte/Macrophage
NR4A2	0	0.531510402	0.936	0.588	0	Monocyte/Macrophage
HBEGF	0	0.530638614	0.753	0.442	0	Monocyte/Macrophage
TLR2	0	0.530520242	0.834	0.353	0	Monocyte/Macrophage
NINJ1	0	0.52612315	0.824	0.465	0	Monocyte/Macrophage
CPM	0	0.525002391	0.826	0.474	0	Monocyte/Macrophage
ZNF331	0	0.521715897	0.832	0.646	0	Monocyte/Macrophage
SRGN	0	0.514154033	0.998	0.879	0	Monocyte/Macrophage
TBXAS1	0	0.513176154	0.819	0.463	0	Monocyte/Macrophage
PDXK	0	0.508078679	0.824	0.499	0	Monocyte/Macrophage
EMILIN2	0	0.507748191	0.781	0.425	0	Monocyte/Macrophage
RHOB	0	0.507383269	0.885	0.526	0	Monocyte/Macrophage
IL1RN	0	0.506713023	0.638	0.314	0	Monocyte/Macrophage
ITGAX	0	0.505197306	0.748	0.36	0	Monocyte/Macrophage
FPR1	0	0.504272943	0.775	0.327	0	Monocyte/Macrophage
STAB1	0	0.502997858	0.822	0.507	0	Monocyte/Macrophage
PLEKHO1	0	0.497114956	0.902	0.615	0	Monocyte/Macrophage
DSE	0	0.497020133	0.785	0.429	0	Monocyte/Macrophage
FTH1	0	0.495874911	1	0.972	0	Monocyte/Macrophage
MARCH1	0	0.495033215	0.818	0.437	0	Monocyte/Macrophage
TREM1	0	0.493998609	0.719	0.399	0	Monocyte/Macrophage
RAB31	0	0.493845063	0.871	0.521	0	Monocyte/Macrophage
LGALS9	0	0.492393254	0.784	0.527	0	Monocyte/Macrophage
KCNMA1	0	0.491045357	0.816	0.366	0	Monocyte/Macrophage
NRP2	0	0.490387597	0.841	0.485	0	Monocyte/Macrophage
LAP3	0	0.490127905	0.897	0.58	0	Monocyte/Macrophage
PLBD1	0	0.489171435	0.799	0.362	0	Monocyte/Macrophage
EPB41L3	0	0.485891728	0.798	0.347	0	Monocyte/Macrophage
RBM47	0	0.4846968	0.8	0.314	0	Monocyte/Macrophage
RAB20	0	0.484653947	0.706	0.46	0	Monocyte/Macrophage
CCL8	0	0.48311026	0.775	0.437	0	Monocyte/Macrophage
CD4	0	0.481520367	0.846	0.395	0	Monocyte/Macrophage
OGFRL1	0	0.479568874	0.793	0.464	0	Monocyte/Macrophage
UCP2	0	0.47895577	0.845	0.54	0	Monocyte/Macrophage
SLAMF8	0	0.478142271	0.804	0.463	0	Monocyte/Macrophage
AP1S2	0	0.474644208	0.861	0.517	0	Monocyte/Macrophage
FCGR1B	0	0.472719985	0.81	0.299	0	Monocyte/Macrophage
GADD45B	0	0.47249083	0.952	0.716	0	Monocyte/Macrophage
NCF2	0	0.470763679	0.722	0.318	0	Monocyte/Macrophage
VEGFA	0	0.470070504	0.804	0.435	0	Monocyte/Macrophage
P2RY13	0	0.469091494	0.781	0.404	0	Monocyte/Macrophage
ATP1B3	0	0.468823552	0.929	0.65	0	Monocyte/Macrophage
CLEC10A	0	0.467995787	0.759	0.328	0	Monocyte/Macrophage
HCK	0	0.467715438	0.763	0.427	0	Monocyte/Macrophage
BASP1	0	0.461988395	0.731	0.507	0	Monocyte/Macrophage
KCTD12	0	0.45783297	0.833	0.44	0	Monocyte/Macrophage
CCL4	0	0.452613745	0.924	0.812	0	Monocyte/Macrophage
MPEG1	0	0.45257058	0.766	0.312	0	Monocyte/Macrophage

SLC31A2	0	0.452159403	0.712	0.436	0	Monocyte/Macrophage
MTSS1	0	0.448485822	0.766	0.445	0	Monocyte/Macrophage
CXorf21	0	0.447171268	0.756	0.437	0	Monocyte/Macrophage
SLC16A3	0	0.443597091	0.755	0.499	0	Monocyte/Macrophage
GCA	0	0.438261535	0.795	0.468	0	Monocyte/Macrophage
PLAU	0	0.437995769	0.789	0.509	0	Monocyte/Macrophage
LGALS3	0	0.436300718	0.917	0.723	0	Monocyte/Macrophage
DSC2	0	0.435621843	0.807	0.294	0	Monocyte/Macrophage
BHLHE41	0	0.430624614	0.839	0.378	0	Monocyte/Macrophage
HSPA1A	0	0.43026643	0.939	0.729	0	Monocyte/Macrophage
COTL1	0	0.429225447	0.938	0.736	0	Monocyte/Macrophage
TUBA1C	0	0.427966901	0.881	0.679	0	Monocyte/Macrophage
SLC8A1	0	0.427457993	0.799	0.456	0	Monocyte/Macrophage
LPCAT2	0	0.42523911	0.797	0.402	0	Monocyte/Macrophage
LILRB2	0	0.424194529	0.716	0.355	0	Monocyte/Macrophage
BID	0	0.423429158	0.799	0.544	0	Monocyte/Macrophage
LIPA	0	0.421990497	0.668	0.417	0	Monocyte/Macrophage
LINC01094	0	0.41965972	0.795	0.329	0	Monocyte/Macrophage
EIF4A3	0	0.417141091	0.919	0.668	0	Monocyte/Macrophage
NLRP3	0	0.417126704	0.824	0.454	0	Monocyte/Macrophage
MMP19	0	0.41513647	0.754	0.433	0	Monocyte/Macrophage
HSPH1	0	0.413886897	0.892	0.587	0	Monocyte/Macrophage
TFEC	0	0.409733917	0.778	0.379	0	Monocyte/Macrophage
TNFAIP2	0	0.409682672	0.776	0.426	0	Monocyte/Macrophage
MMP9	0	0.408360966	0.744	0.424	0	Monocyte/Macrophage
IRF8	0	0.408240819	0.799	0.516	0	Monocyte/Macrophage
RNF144B	0	0.407912935	0.829	0.409	0	Monocyte/Macrophage
PTAFR	0	0.406976448	0.634	0.331	0	Monocyte/Macrophage
TUBA1B	0	0.403893353	0.987	0.872	0	Monocyte/Macrophage
NRP1	0	0.402469095	0.87	0.506	0	Monocyte/Macrophage
AOAH	0	0.398685379	0.834	0.462	0	Monocyte/Macrophage
ATP1B1	0	0.3966628	0.784	0.47	0	Monocyte/Macrophage
TMEM70	0	0.396137371	0.801	0.583	0	Monocyte/Macrophage
GLRX	0	0.395635699	0.865	0.625	0	Monocyte/Macrophage
TSPAN33	0	0.394803135	0.843	0.425	0	Monocyte/Macrophage
CD300A	0	0.394609651	0.644	0.273	0	Monocyte/Macrophage
B3GNT5	0	0.392043543	0.787	0.421	0	Monocyte/Macrophage
AC020656.1	0	0.391282652	0.633	0.274	0	Monocyte/Macrophage
ELL2	0	0.385000485	0.819	0.471	0	Monocyte/Macrophage
FILIP1L	0	0.384878592	0.87	0.505	0	Monocyte/Macrophage
BCAT1	0	0.380780216	0.704	0.411	0	Monocyte/Macrophage
HERPUD1	0	0.380697767	0.965	0.819	0	Monocyte/Macrophage
CCL18	0	0.371796794	0.633	0.368	0	Monocyte/Macrophage
SLC11A1	0	0.371365948	0.617	0.415	0	Monocyte/Macrophage
FYB1	0	0.370681884	0.92	0.687	0	Monocyte/Macrophage
LCP1	0	0.369682701	0.928	0.722	0	Monocyte/Macrophage
LTC4S	0	0.369178097	0.865	0.507	0	Monocyte/Macrophage
IGSF21	0	0.366523	0.754	0.314	0	Monocyte/Macrophage
LILRB3	0	0.365095424	0.687	0.295	0	Monocyte/Macrophage
CDKN1A	0	0.364842552	0.904	0.621	0	Monocyte/Macrophage
JDP2	0	0.362248308	0.794	0.474	0	Monocyte/Macrophage
NCF1	0	0.361327944	0.852	0.546	0	Monocyte/Macrophage
PAK1	0	0.360198091	0.786	0.44	0	Monocyte/Macrophage
CD33	0	0.359742001	0.711	0.372	0	Monocyte/Macrophage
THEMIS2	0	0.359495643	0.638	0.385	0	Monocyte/Macrophage
CD72	0	0.359147824	0.7	0.421	0	Monocyte/Macrophage

PDK4	0	0.359081716	0.76	0.463	0	Monocyte/Macrophage
SLC40A1	0	0.356836435	0.773	0.472	0	Monocyte/Macrophage
ADM	0	0.355889386	0.805	0.51	0	Monocyte/Macrophage
C2	0	0.355519361	0.74	0.424	0	Monocyte/Macrophage
PPIF	0	0.353436317	0.661	0.441	0	Monocyte/Macrophage
LAT2	0	0.352737897	0.781	0.374	0	Monocyte/Macrophage
SLC7A7	0	0.349355312	0.716	0.333	0	Monocyte/Macrophage
TMEM37	0	0.348952807	0.773	0.41	0	Monocyte/Macrophage
ANXA2	0	0.348773828	0.957	0.795	0	Monocyte/Macrophage
FUCA1	0	0.346582958	0.729	0.459	0	Monocyte/Macrophage
IGF1	0	0.346239815	0.775	0.354	0	Monocyte/Macrophage
SPINT2	0	0.345477422	0.839	0.538	0	Monocyte/Macrophage
ACSL1	0	0.344363008	0.699	0.392	0	Monocyte/Macrophage
JAML	0	0.342975306	0.833	0.397	0	Monocyte/Macrophage
EIF4E	0	0.337985504	0.824	0.518	0	Monocyte/Macrophage
SAP30	0	0.334801741	0.849	0.573	0	Monocyte/Macrophage
GLA	0	0.329211641	0.666	0.445	0	Monocyte/Macrophage
LRRC25	0	0.32425116	0.595	0.268	0	Monocyte/Macrophage
CSF2RA	0	0.322244506	0.602	0.229	0	Monocyte/Macrophage
CCR1	0	0.321847956	0.606	0.362	0	Monocyte/Macrophage
ALOX5	0	0.319923127	0.615	0.4	0	Monocyte/Macrophage
POU2F2	0	0.31967925	0.785	0.509	0	Monocyte/Macrophage
FCER1A	0	0.319557464	0.701	0.375	0	Monocyte/Macrophage
ANPEP	0	0.314513604	0.647	0.445	0	Monocyte/Macrophage
GNA15	0	0.313835513	0.732	0.356	0	Monocyte/Macrophage
STX11	0	0.311837501	0.714	0.473	0	Monocyte/Macrophage
CXCL1	0	0.311676463	0.731	0.422	0	Monocyte/Macrophage
NFIL3	0	0.311137222	0.793	0.54	0	Monocyte/Macrophage
CX3CR1	0	0.308702229	0.787	0.298	0	Monocyte/Macrophage
CKS2	0	0.307662682	0.855	0.644	0	Monocyte/Macrophage
EVI2B	0	0.307302175	0.827	0.628	0	Monocyte/Macrophage
HMGA1	0	0.304055384	0.742	0.508	0	Monocyte/Macrophage
DMXL2	0	0.302423198	0.675	0.33	0	Monocyte/Macrophage
SYAP1	0	0.302316059	0.836	0.518	0	Monocyte/Macrophage
HSPA1B	0	0.30106528	0.866	0.634	0	Monocyte/Macrophage
RIPK2	0	0.299812044	0.76	0.46	0	Monocyte/Macrophage
MIR181A1HG	0	0.299454844	0.753	0.465	0	Monocyte/Macrophage
CLEC5A	0	0.29657256	0.589	0.292	0	Monocyte/Macrophage
EGR2	0	0.29599527	0.672	0.449	0	Monocyte/Macrophage
RAB11FIP1	0	0.293556297	0.76	0.54	0	Monocyte/Macrophage
SNX10	0	0.293047022	0.676	0.468	0	Monocyte/Macrophage
PAPSS2	0	0.292794159	0.717	0.371	0	Monocyte/Macrophage
LMO2	0	0.29265593	0.768	0.459	0	Monocyte/Macrophage
LRP1	0	0.292496805	0.683	0.436	0	Monocyte/Macrophage
GATM	0	0.289299575	0.582	0.26	0	Monocyte/Macrophage
IL4I1	0	0.287954394	0.618	0.382	0	Monocyte/Macrophage
LILRB1	0	0.285698767	0.703	0.378	0	Monocyte/Macrophage
CLEC4A	0	0.284776918	0.695	0.401	0	Monocyte/Macrophage
IER5	0	0.283899797	0.841	0.594	0	Monocyte/Macrophage
MXD1	0	0.28024401	0.687	0.444	0	Monocyte/Macrophage
COLEC12	0	0.279779263	0.785	0.41	0	Monocyte/Macrophage
AREG	0	0.273570959	0.761	0.542	0	Monocyte/Macrophage
KYNU	0	0.271590213	0.657	0.407	0	Monocyte/Macrophage
MGST2	0	0.270616765	0.78	0.51	0	Monocyte/Macrophage
C1orf54	0	0.270070076	0.723	0.498	0	Monocyte/Macrophage
FKBP2	0	0.265392741	0.899	0.702	0	Monocyte/Macrophage

C3	0	0.265202708	0.723	0.381	0	Monocyte/Macrophage
LILRB5	0	0.262257579	0.66	0.19	0	Monocyte/Macrophage
GRASP	0	0.262160525	0.832	0.536	0	Monocyte/Macrophage
GZF1	0	0.252571131	0.652	0.446	0	Monocyte/Macrophage
OLFML2B	6.99E-304	0.25081998	0.619	0.395	1.40E-300	Monocyte/Macrophage
SDS	1.16E-283	0.382384973	0.568	0.273	2.32E-280	Monocyte/Macrophage
CSTA	4.40E-266	0.292955662	0.589	0.343	8.80E-263	Monocyte/Macrophage
ATF5	1.07E-247	0.276404374	0.602	0.404	2.15E-244	Monocyte/Macrophage
LGALS1	1.00E-246	0.279969843	0.936	0.819	2.01E-243	Monocyte/Macrophage
HCST	1.89E-244	0.266934713	0.923	0.756	3.78E-241	Monocyte/Macrophage
PLA2G7	3.54E-112	0.297855374	0.527	0.318	7.08E-109	Monocyte/Macrophage
LUM	0	1.813092554	0.976	0.554	0	Fibroblast
SFRP2	0	1.667576186	0.919	0.492	0	Fibroblast
DCN	0	1.616485648	0.948	0.413	0	Fibroblast
COL1A2	0	1.592662336	0.99	0.488	0	Fibroblast
CFH	0	1.502515858	0.994	0.518	0	Fibroblast
C1R	0	1.500637313	0.991	0.463	0	Fibroblast
BGN	0	1.49457196	0.995	0.508	0	Fibroblast
CTGF	0	1.461689389	0.987	0.552	0	Fibroblast
MGP	0	1.436415726	0.999	0.708	0	Fibroblast
CYR61	0	1.42857646	0.983	0.533	0	Fibroblast
COL3A1	0	1.417277313	0.957	0.477	0	Fibroblast
AEBP1	0	1.407114668	0.99	0.482	0	Fibroblast
COL14A1	0	1.361456766	0.983	0.43	0	Fibroblast
CCDC80	0	1.326890499	0.977	0.524	0	Fibroblast
FN1	0	1.311506102	0.991	0.54	0	Fibroblast
TAGLN	0	1.310789211	0.993	0.509	0	Fibroblast
PCOLCE	0	1.291025736	0.975	0.489	0	Fibroblast
VCAN	0	1.272083285	0.99	0.492	0	Fibroblast
C1S	0	1.269422471	0.97	0.385	0	Fibroblast
SOD3	0	1.243771013	0.984	0.451	0	Fibroblast
COL1A1	0	1.22883111	0.919	0.427	0	Fibroblast
IGFBP7	0	1.191182243	0.998	0.747	0	Fibroblast
CPE	0	1.184178453	0.973	0.485	0	Fibroblast
CALD1	0	1.182904229	0.997	0.552	0	Fibroblast
NNMT	0	1.181980174	0.987	0.574	0	Fibroblast
COL6A2	0	1.1767685	0.968	0.539	0	Fibroblast
C2orf40	0	1.176575272	0.949	0.556	0	Fibroblast
IGFBP2	0	1.159907506	0.969	0.532	0	Fibroblast
IGFBP6	0	1.158327957	0.893	0.426	0	Fibroblast
PLAC9	0	1.130634415	0.962	0.494	0	Fibroblast
IGFBP5	0	1.128218183	0.942	0.529	0	Fibroblast
SERPING1	0	1.12110701	0.987	0.526	0	Fibroblast
TIMP1	0	1.119000004	1	0.782	0	Fibroblast
EFEMP1	0	1.101957746	0.927	0.417	0	Fibroblast
RARRES2	0	1.068372352	0.905	0.488	0	Fibroblast
OGN	0	1.066829748	0.911	0.376	0	Fibroblast
CRYAB	0	1.06514285	0.941	0.468	0	Fibroblast
SERPINF1	0	1.061344971	0.876	0.521	0	Fibroblast
SPARC	0	1.039019542	0.973	0.602	0	Fibroblast
CTSK	0	1.036732617	0.932	0.454	0	Fibroblast
PRSS23	0	1.034965952	0.918	0.533	0	Fibroblast
DPT	0	1.011266383	0.893	0.46	0	Fibroblast
MYL9	0	1.010492476	0.981	0.437	0	Fibroblast
COL6A1	0	1.008518049	0.911	0.441	0	Fibroblast
COL6A3	0	1.007321362	0.914	0.458	0	Fibroblast

CCL19	0	1.005199672	0.83	0.656	0	Fibroblast
HTRA1	0	1.004438202	0.959	0.555	0	Fibroblast
TPM2	0	1.003023626	0.985	0.469	0	Fibroblast
CP	0	0.992627789	0.915	0.358	0	Fibroblast
SELENOM	0	0.986369463	0.985	0.622	0	Fibroblast
THY1	0	0.985775279	0.909	0.555	0	Fibroblast
ADIRF	0	0.981808919	0.986	0.538	0	Fibroblast
CXCL12	0	0.972488521	0.955	0.616	0	Fibroblast
TPM1	0	0.971568859	0.971	0.539	0	Fibroblast
MFGE8	0	0.968950377	0.951	0.454	0	Fibroblast
SFRP4	0	0.957509069	0.799	0.375	0	Fibroblast
VCAM1	0	0.95511194	0.93	0.502	0	Fibroblast
FMO2	0	0.952621606	0.9	0.426	0	Fibroblast
LTBP2	0	0.951563622	0.9	0.441	0	Fibroblast
PRRX1	0	0.94827167	0.905	0.347	0	Fibroblast
TFPI	0	0.938790972	0.941	0.534	0	Fibroblast
PRELP	0	0.936103591	0.854	0.333	0	Fibroblast
PPIC	0	0.921910757	0.94	0.516	0	Fibroblast
LGALS3BP	0	0.915115467	0.95	0.529	0	Fibroblast
DKK3	0	0.91024827	0.934	0.42	0	Fibroblast
TNC	0	0.907609725	0.917	0.474	0	Fibroblast
FGF7	0	0.903664388	0.868	0.505	0	Fibroblast
THBS2	0	0.903606987	0.917	0.426	0	Fibroblast
CD9	0	0.898775325	0.982	0.639	0	Fibroblast
NBL1	0	0.897652132	0.927	0.547	0	Fibroblast
CLU	0	0.889871979	0.903	0.489	0	Fibroblast
CCL2	0	0.886198815	0.914	0.503	0	Fibroblast
LTBP1	0	0.884567865	0.908	0.338	0	Fibroblast
SULF1	0	0.882096003	0.866	0.323	0	Fibroblast
GSN	0	0.877617199	0.981	0.62	0	Fibroblast
IFITM3	0	0.866767302	0.999	0.713	0	Fibroblast
MXRA8	0	0.855371428	0.832	0.404	0	Fibroblast
SSPN	0	0.849138034	0.906	0.446	0	Fibroblast
RCN3	0	0.847318956	0.895	0.381	0	Fibroblast
OMD	0	0.846838197	0.778	0.285	0	Fibroblast
NUPR1	0	0.844912525	0.917	0.448	0	Fibroblast
SPARCL1	0	0.832890964	0.969	0.586	0	Fibroblast
EDIL3	0	0.832821391	0.93	0.49	0	Fibroblast
FHL1	0	0.832109833	0.914	0.468	0	Fibroblast
PMP22	0	0.830134389	0.938	0.533	0	Fibroblast
CRTAC1	0	0.827893318	0.795	0.357	0	Fibroblast
NOV	0	0.825498054	0.878	0.461	0	Fibroblast
EGR1	0	0.824088116	0.93	0.559	0	Fibroblast
LHFPL6	0	0.823919272	0.891	0.431	0	Fibroblast
CDH11	0	0.82282387	0.925	0.466	0	Fibroblast
CAVIN3	0	0.814946535	0.965	0.561	0	Fibroblast
PDLIM3	0	0.808808245	0.918	0.433	0	Fibroblast
ASPN	0	0.805404913	0.839	0.345	0	Fibroblast
S100A13	0	0.803715857	0.913	0.511	0	Fibroblast
TGM2	0	0.799884287	0.902	0.393	0	Fibroblast
ITGBL1	0	0.796384099	0.808	0.323	0	Fibroblast
COL8A1	0	0.792702483	0.84	0.348	0	Fibroblast
PTGDS	0	0.789482452	0.778	0.457	0	Fibroblast
EFEMP2	0	0.788585471	0.894	0.412	0	Fibroblast
CST3	0	0.78533978	0.998	0.714	0	Fibroblast
SUGCT	0	0.771979849	0.891	0.414	0	Fibroblast

FAP	0	0.768336644	0.873	0.445	0	Fibroblast
MMP2	0	0.767781639	0.823	0.456	0	Fibroblast
POSTN	0	0.767289349	0.819	0.526	0	Fibroblast
C11orf96	0	0.764884449	0.855	0.473	0	Fibroblast
FRZB	0	0.763518927	0.908	0.445	0	Fibroblast
WISP2	0	0.755275497	0.75	0.347	0	Fibroblast
PDGFRL	0	0.753639845	0.837	0.38	0	Fibroblast
CNN3	0	0.749286092	0.94	0.534	0	Fibroblast
COPZ2	0	0.748939307	0.854	0.407	0	Fibroblast
MT1E	0	0.745145457	0.871	0.558	0	Fibroblast
FXYD1	0	0.74392226	0.842	0.442	0	Fibroblast
TIMP3	0	0.742388322	0.819	0.49	0	Fibroblast
DSTN	0	0.741861762	0.991	0.676	0	Fibroblast
SLPI	0	0.737509448	0.727	0.443	0	Fibroblast
ISLR	0	0.736622986	0.801	0.309	0	Fibroblast
ID4	0	0.735351174	0.876	0.439	0	Fibroblast
STEAP4	0	0.727453946	0.879	0.531	0	Fibroblast
SERPINH1	0	0.724290855	0.911	0.544	0	Fibroblast
FHL2	0	0.721919267	0.853	0.475	0	Fibroblast
FAM114A1	0	0.720106355	0.872	0.476	0	Fibroblast
UACA	0	0.714361291	0.922	0.554	0	Fibroblast
SMOC2	0	0.712278901	0.896	0.393	0	Fibroblast
GUCY1A1	0	0.711684574	0.888	0.435	0	Fibroblast
PTN	0	0.708319576	0.843	0.345	0	Fibroblast
LGALS1	0	0.707030115	0.997	0.821	0	Fibroblast
FBLN1	0	0.706052074	0.701	0.323	0	Fibroblast
LRRC17	0	0.705907767	0.793	0.352	0	Fibroblast
TSC22D1	0	0.705459677	0.932	0.609	0	Fibroblast
TNFRSF11B	0	0.700878033	0.778	0.441	0	Fibroblast
PLS3	0	0.696916986	0.911	0.527	0	Fibroblast
NR2F2	0	0.696212133	0.916	0.585	0	Fibroblast
AGT	0	0.695971204	0.827	0.369	0	Fibroblast
CD151	0	0.688115527	0.942	0.578	0	Fibroblast
CTSF	0	0.685502291	0.838	0.418	0	Fibroblast
COX7A1	0	0.684887997	0.854	0.471	0	Fibroblast
GGT5	0	0.681795514	0.88	0.5	0	Fibroblast
GEM	0	0.679651855	0.863	0.566	0	Fibroblast
PMEPA1	0	0.671625561	0.919	0.566	0	Fibroblast
MAP1B	0	0.671166735	0.902	0.436	0	Fibroblast
COL5A2	0	0.666953676	0.856	0.426	0	Fibroblast
GAS6	0	0.664951898	0.891	0.599	0	Fibroblast
HCFC1R1	0	0.66454136	0.928	0.53	0	Fibroblast
PDGFRA	0	0.65010669	0.775	0.313	0	Fibroblast
PRDX4	0	0.645174142	0.933	0.628	0	Fibroblast
LOXL1	0	0.644029441	0.872	0.377	0	Fibroblast
ACTA2	0	0.642177147	0.878	0.469	0	Fibroblast
TM4SF1	0	0.63689728	0.915	0.594	0	Fibroblast
OLFML3	0	0.636556447	0.82	0.333	0	Fibroblast
CAV1	0	0.635626505	0.939	0.592	0	Fibroblast
SVIL	0	0.635472784	0.87	0.459	0	Fibroblast
ID3	0	0.635121666	0.909	0.619	0	Fibroblast
ANGPT2	0	0.628701466	0.87	0.506	0	Fibroblast
MFAP4	0	0.623128861	0.735	0.341	0	Fibroblast
GPX3	0	0.621747237	0.772	0.424	0	Fibroblast
FSTL1	0	0.621093748	0.751	0.411	0	Fibroblast
APOE	0	0.615984487	0.766	0.455	0	Fibroblast

LRP1	0	0.613375581	0.799	0.44	0	Fibroblast
NUCB2	0	0.613237675	0.924	0.644	0	Fibroblast
GPX8	0	0.611301576	0.8	0.408	0	Fibroblast
FBN1	0	0.610844616	0.754	0.431	0	Fibroblast
RND3	0	0.61046479	0.822	0.439	0	Fibroblast
MARCKS	0	0.609250603	0.965	0.689	0	Fibroblast
SGCA	0	0.608122583	0.892	0.401	0	Fibroblast
EPB41L2	0	0.605721386	0.835	0.507	0	Fibroblast
SPTBN1	0	0.599800014	0.857	0.479	0	Fibroblast
CD59	0	0.597085415	0.911	0.553	0	Fibroblast
COL4A2	0	0.596722944	0.891	0.499	0	Fibroblast
PDGFD	0	0.58961155	0.786	0.372	0	Fibroblast
S100A16	0	0.589302175	0.886	0.486	0	Fibroblast
MT1M	0	0.587475363	0.718	0.339	0	Fibroblast
KDELR3	0	0.586605536	0.815	0.387	0	Fibroblast
IGFBP4	0	0.584439345	0.861	0.584	0	Fibroblast
COL15A1	0	0.582879998	0.858	0.483	0	Fibroblast
CTHRC1	0	0.579320311	0.816	0.526	0	Fibroblast
ABI3BP	0	0.57746535	0.682	0.358	0	Fibroblast
ANGPT1	0	0.571547058	0.806	0.399	0	Fibroblast
PALLD	0	0.568576666	0.832	0.429	0	Fibroblast
MMP23B	0	0.565266181	0.703	0.422	0	Fibroblast
MT2A	0	0.555707334	0.962	0.776	0	Fibroblast
PDGFRB	0	0.554146416	0.766	0.36	0	Fibroblast
NDUFA4L2	0	0.553588571	0.703	0.422	0	Fibroblast
MDK	0	0.551798628	0.854	0.52	0	Fibroblast
CAVIN1	0	0.550127407	0.864	0.52	0	Fibroblast
GAP43	0	0.549674013	0.79	0.408	0	Fibroblast
ADH1B	0	0.549028674	0.648	0.278	0	Fibroblast
TGFB1I1	0	0.547265525	0.843	0.392	0	Fibroblast
SPRY1	0	0.545105658	0.886	0.57	0	Fibroblast
TWIST1	0	0.544703949	0.805	0.362	0	Fibroblast
FEZ1	0	0.543502676	0.769	0.39	0	Fibroblast
RGS16	0	0.543494455	0.897	0.624	0	Fibroblast
NPC2	0	0.541688961	0.984	0.7	0	Fibroblast
COL21A1	0	0.54145991	0.841	0.451	0	Fibroblast
CCL21	0	0.535889108	0.796	0.526	0	Fibroblast
CERCAM	0	0.531280954	0.781	0.316	0	Fibroblast
BACE2	0	0.527601106	0.832	0.425	0	Fibroblast
SERPINE1	0	0.526696362	0.78	0.386	0	Fibroblast
TMEM98	0	0.522787283	0.728	0.334	0	Fibroblast
COL4A1	0	0.52247359	0.89	0.517	0	Fibroblast
CCDC102B	0	0.522410426	0.852	0.462	0	Fibroblast
FKBP10	0	0.522063438	0.762	0.38	0	Fibroblast
JUN	0	0.520132739	0.978	0.799	0	Fibroblast
CRIP2	0	0.518667537	0.896	0.592	0	Fibroblast
IL34	0	0.51799222	0.848	0.54	0	Fibroblast
C9orf3	0	0.516234169	0.807	0.463	0	Fibroblast
SGCE	0	0.516145502	0.763	0.378	0	Fibroblast
LGALS3	0	0.515993378	0.975	0.729	0	Fibroblast
TNFRSF12A	0	0.5145872	0.859	0.538	0	Fibroblast
BICC1	0	0.512850197	0.725	0.303	0	Fibroblast
ANTXR1	0	0.510393755	0.735	0.379	0	Fibroblast
SDC2	0	0.509789246	0.75	0.394	0	Fibroblast
PRRX2	0	0.508080001	0.777	0.354	0	Fibroblast
STEAP1	0	0.506239572	0.798	0.388	0	Fibroblast

PDGFA	0	0.506138288	0.838	0.51	0	Fibroblast
IFI6	0	0.504626175	0.869	0.569	0	Fibroblast
SNCG	0	0.503008954	0.876	0.565	0	Fibroblast
NEXN	0	0.501633527	0.807	0.388	0	Fibroblast
CYBRD1	0	0.500667548	0.76	0.423	0	Fibroblast
HSP90B1	0	0.500496826	0.989	0.814	0	Fibroblast
SLC40A1	0	0.50048095	0.869	0.481	0	Fibroblast
CRISPLD1	0	0.497876949	0.825	0.43	0	Fibroblast
AKR1C3	0	0.496173522	0.853	0.509	0	Fibroblast
F3	0	0.493931396	0.723	0.503	0	Fibroblast
IGFBP3	0	0.493270866	0.673	0.356	0	Fibroblast
AQP1	0	0.490146758	0.839	0.512	0	Fibroblast
GSTM3	0	0.490057278	0.844	0.599	0	Fibroblast
S100A6	0	0.487829784	0.999	0.931	0	Fibroblast
NFIB	0	0.485908453	0.777	0.39	0	Fibroblast
INHBA	0	0.485877859	0.745	0.327	0	Fibroblast
LIMCH1	0	0.483396937	0.774	0.376	0	Fibroblast
C7	0	0.483246446	0.675	0.538	0	Fibroblast
EMILIN1	0	0.481424067	0.717	0.364	0	Fibroblast
ERLEC1	0	0.480997847	0.873	0.595	0	Fibroblast
ARHGAP29	0	0.480821996	0.826	0.553	0	Fibroblast
ANXA2	0	0.480443387	0.986	0.801	0	Fibroblast
LTBP4	0	0.479625612	0.756	0.429	0	Fibroblast
P4HA2	0	0.478467604	0.784	0.436	0	Fibroblast
CCDC3	0	0.474406752	0.79	0.413	0	Fibroblast
IL1R1	0	0.47363534	0.783	0.41	0	Fibroblast
DAAM1	0	0.471159224	0.766	0.477	0	Fibroblast
GADD45A	0	0.471094601	0.849	0.568	0	Fibroblast
A2M	0	0.470117742	0.941	0.63	0	Fibroblast
HSPA1A	0	0.468437266	0.95	0.741	0	Fibroblast
C1QTNF1	0	0.468118328	0.759	0.331	0	Fibroblast
CCND1	0	0.467313911	0.835	0.591	0	Fibroblast
AKR1C2	0	0.460670728	0.611	0.302	0	Fibroblast
NTRK2	0	0.452150776	0.718	0.402	0	Fibroblast
S100A4	0	0.451859353	0.996	0.885	0	Fibroblast
ENAH	0	0.450377642	0.809	0.411	0	Fibroblast
LMCD1	0	0.447962115	0.809	0.533	0	Fibroblast
MRC2	0	0.447599919	0.638	0.413	0	Fibroblast
ITGA8	0	0.441589569	0.781	0.372	0	Fibroblast
ECM1	0	0.437600541	0.701	0.489	0	Fibroblast
AKAP12	0	0.436888411	0.661	0.406	0	Fibroblast
ANKH	0	0.436868396	0.746	0.501	0	Fibroblast
LAMA4	0	0.434614072	0.811	0.554	0	Fibroblast
PDLIM1	0	0.434516734	0.902	0.594	0	Fibroblast
GMDS	0	0.4339922	0.773	0.478	0	Fibroblast
FOXC1	0	0.433779227	0.766	0.454	0	Fibroblast
FBLIM1	0	0.43238678	0.776	0.399	0	Fibroblast
FBLN5	0	0.430298654	0.637	0.29	0	Fibroblast
RBP1	0	0.43019557	0.803	0.387	0	Fibroblast
GPNMB	0	0.426350775	0.775	0.419	0	Fibroblast
CD200	0	0.425463986	0.814	0.529	0	Fibroblast
MFAP2	0	0.425303564	0.672	0.289	0	Fibroblast
MSRB3	0	0.416974096	0.771	0.379	0	Fibroblast
SEPT4	0	0.41682524	0.839	0.481	0	Fibroblast
WWTR1	0	0.416638803	0.789	0.425	0	Fibroblast
MXRA5	0	0.416161346	0.826	0.33	0	Fibroblast

CAV2	0	0.414970174	0.792	0.449	0	Fibroblast
PLPP1	0	0.412372602	0.737	0.544	0	Fibroblast
TINAGL1	0	0.406613444	0.851	0.475	0	Fibroblast
RARRES1	0	0.404193087	0.603	0.334	0	Fibroblast
SERPINE2	0	0.401007447	0.626	0.376	0	Fibroblast
SRPX	0	0.400980402	0.683	0.371	0	Fibroblast
RAB31	0	0.399099747	0.843	0.544	0	Fibroblast
F2R	0	0.398931218	0.758	0.522	0	Fibroblast
CSRP1	0	0.389871145	0.819	0.51	0	Fibroblast
HSPA1B	0	0.389088124	0.874	0.647	0	Fibroblast
DPYSL3	0	0.388539216	0.691	0.377	0	Fibroblast
COL12A1	0	0.384445933	0.733	0.4	0	Fibroblast
PRKG1	0	0.381093519	0.78	0.313	0	Fibroblast
CPXM2	0	0.37878785	0.66	0.408	0	Fibroblast
GAS1	0	0.377623579	0.661	0.371	0	Fibroblast
PDLIM4	0	0.376947561	0.759	0.477	0	Fibroblast
CDO1	0	0.370196989	0.64	0.331	0	Fibroblast
PLD3	0	0.369197001	0.788	0.475	0	Fibroblast
GPM6B	0	0.366536637	0.745	0.442	0	Fibroblast
COL5A1	0	0.36648131	0.703	0.309	0	Fibroblast
MYLK	0	0.364746402	0.806	0.401	0	Fibroblast
ATF3	0	0.357955191	0.879	0.611	0	Fibroblast
CTSZ	0	0.355908561	0.938	0.677	0	Fibroblast
SMOC1	0	0.355804352	0.744	0.192	0	Fibroblast
SELENOP	0	0.354326453	0.864	0.648	0	Fibroblast
HGF	0	0.353792898	0.768	0.428	0	Fibroblast
FST	0	0.351600292	0.737	0.342	0	Fibroblast
CRABP2	0	0.348601929	0.656	0.366	0	Fibroblast
HNMT	0	0.348514387	0.848	0.534	0	Fibroblast
RGS3	0	0.347851662	0.735	0.494	0	Fibroblast
HES4	0	0.345368996	0.787	0.501	0	Fibroblast
TNFSF13B	0	0.345035543	0.818	0.569	0	Fibroblast
HAPLN1	0	0.344446482	0.797	0.419	0	Fibroblast
PPA1	0	0.343357886	0.873	0.645	0	Fibroblast
TMEM47	0	0.342760077	0.714	0.352	0	Fibroblast
SPINT2	0	0.339944249	0.861	0.554	0	Fibroblast
FKBP2	0	0.338384672	0.906	0.713	0	Fibroblast
PAWR	0	0.336583119	0.797	0.469	0	Fibroblast
MAP9	0	0.335485666	0.648	0.353	0	Fibroblast
MYH10	0	0.335419959	0.697	0.39	0	Fibroblast
ENG	0	0.335101044	0.803	0.508	0	Fibroblast
PFN2	0	0.331035336	0.672	0.364	0	Fibroblast
SOX4	0	0.328526947	0.798	0.6	0	Fibroblast
DNAJB4	0	0.325490111	0.802	0.517	0	Fibroblast
CSF1	0	0.323255903	0.663	0.469	0	Fibroblast
LMO7	0	0.319596213	0.685	0.387	0	Fibroblast
ENPP1	0	0.317523514	0.775	0.415	0	Fibroblast
SLC7A2	0	0.31685338	0.79	0.283	0	Fibroblast
TNFAIP6	0	0.311751125	0.725	0.38	0	Fibroblast
NET1	0	0.311721631	0.771	0.412	0	Fibroblast
PCDH10	0	0.310910683	0.71	0.374	0	Fibroblast
IBSP	0	0.310519897	0.696	0.316	0	Fibroblast
KCNE4	0	0.310282896	0.713	0.433	0	Fibroblast
TMEM204	0	0.30934735	0.747	0.537	0	Fibroblast
PLOD2	0	0.308736803	0.695	0.248	0	Fibroblast
PCDH7	0	0.307763551	0.669	0.307	0	Fibroblast

GJA1	0	0.30595937	0.735	0.334	0	Fibroblast
PGF	0	0.305537814	0.718	0.498	0	Fibroblast
GXYLT2	0	0.304413667	0.641	0.232	0	Fibroblast
BASP1	0	0.301076298	0.82	0.512	0	Fibroblast
PHLDA2	0	0.298586236	0.831	0.606	0	Fibroblast
RCAN2	0	0.293569639	0.771	0.544	0	Fibroblast
MMP11	0	0.292139324	0.789	0.426	0	Fibroblast
CXCL9	0	0.292134086	0.746	0.528	0	Fibroblast
LAMB1	0	0.289685613	0.751	0.467	0	Fibroblast
NOTCH3	0	0.288252302	0.729	0.357	0	Fibroblast
KCNK17	0	0.285378982	0.732	0.265	0	Fibroblast
FOXC2	0	0.281391607	0.618	0.271	0	Fibroblast
KCNMB1	0	0.280967908	0.757	0.366	0	Fibroblast
CDKN2A	0	0.280619196	0.752	0.581	0	Fibroblast
PTHLH	0	0.274493755	0.63	0.281	0	Fibroblast
COL18A1	0	0.259222954	0.76	0.506	0	Fibroblast
IFIT1	0	0.258078133	0.705	0.537	0	Fibroblast
NQO1	0	0.257852997	0.683	0.5	0	Fibroblast
KRT17	0	0.257786032	0.696	0.418	0	Fibroblast
LINC01615	0	0.256382194	0.774	0.432	0	Fibroblast
CDH13	0	0.255103644	0.643	0.327	0	Fibroblast
EGFLAM	0	0.254196853	0.763	0.241	0	Fibroblast
C12orf75	0	0.25069782	0.835	0.55	0	Fibroblast
CTSD	9.76E-305	0.326549442	0.935	0.657	1.95E-301	Fibroblast
LOX	3.50E-299	0.262062339	0.578	0.276	7.00E-296	Fibroblast
CDKN1C	5.68E-299	0.310404261	0.763	0.589	1.14E-295	Fibroblast
HES1	2.19E-293	0.291722457	0.731	0.507	4.38E-290	Fibroblast
CLEC11A	1.50E-292	0.300397333	0.644	0.448	2.99E-289	Fibroblast
PSAP	4.57E-291	0.341276632	0.938	0.649	9.15E-288	Fibroblast
LMOD1	1.75E-290	0.265515247	0.651	0.292	3.50E-287	Fibroblast
ITGA1	5.07E-286	0.293017615	0.669	0.47	1.01E-282	Fibroblast
S100A10	9.72E-282	0.285702437	0.986	0.907	1.94E-278	Fibroblast
AXL	1.32E-281	0.35095809	0.627	0.324	2.63E-278	Fibroblast
PCOLCE2	3.04E-281	0.432861406	0.565	0.232	6.07E-278	Fibroblast
NPDC1	2.45E-274	0.302004181	0.751	0.539	4.89E-271	Fibroblast
CHPF	1.20E-268	0.355974654	0.621	0.447	2.40E-265	Fibroblast
DAB2	1.31E-264	0.251033459	0.782	0.588	2.62E-261	Fibroblast
DIO2	1.88E-263	0.289479253	0.583	0.346	3.76E-260	Fibroblast
ECM2	2.33E-260	0.389556678	0.591	0.303	4.67E-257	Fibroblast
CEBPD	1.09E-242	0.346937406	0.93	0.678	2.17E-239	Fibroblast
FIBIN	1.15E-232	0.322152635	0.547	0.225	2.30E-229	Fibroblast
VASN	9.49E-232	0.314608831	0.607	0.403	1.90E-228	Fibroblast
FAT1	4.24E-231	0.254619807	0.582	0.333	8.48E-228	Fibroblast
TUBB6	1.32E-227	0.281177671	0.701	0.47	2.65E-224	Fibroblast
FILIP1L	3.23E-223	0.254840808	0.774	0.535	6.45E-220	Fibroblast
PDLIM7	1.54E-215	0.2725528	0.66	0.449	3.09E-212	Fibroblast
PTGIS	2.24E-213	0.380843104	0.574	0.311	4.49E-210	Fibroblast
PHLDA3	5.94E-211	0.304820862	0.671	0.542	1.19E-207	Fibroblast
MAP1A	1.62E-210	0.270815176	0.591	0.424	3.24E-207	Fibroblast
FMOD	5.46E-202	0.354690027	0.536	0.219	1.09E-198	Fibroblast
FGL2	4.50E-199	0.319174426	0.711	0.505	9.00E-196	Fibroblast
FBLN2	2.68E-176	0.286301471	0.562	0.358	5.36E-173	Fibroblast
PDE5A	9.88E-175	0.287529423	0.609	0.394	1.98E-171	Fibroblast
DEPP1	8.57E-174	0.321867505	0.58	0.359	1.71E-170	Fibroblast
ACKR3	3.47E-150	0.278204325	0.538	0.365	6.95E-147	Fibroblast
OLFML2B	2.56E-146	0.272691261	0.585	0.411	5.11E-143	Fibroblast

MT1X	1.98E-136	0.314562072	0.669	0.532	3.95E-133	Fibroblast
MEDAG	2.30E-130	0.3144585	0.525	0.287	4.59E-127	Fibroblast
ZNF503	2.75E-126	0.262248477	0.612	0.474	5.51E-123	Fibroblast
APOD	3.15E-126	0.325418941	0.541	0.396	6.30E-123	Fibroblast
ERRFI1	9.02E-106	0.261694352	0.562	0.446	1.80E-102	Fibroblast
TNXB	2.45E-103	0.255275777	0.515	0.319	4.91E-100	Fibroblast
ELN	8.11E-101	0.307491428	0.507	0.297	1.62E-97	Fibroblast
PROCR	1.78E-51	0.25409672	0.541	0.45	3.56E-48	Fibroblast
RAMP2	0	1.654672663	0.943	0.517	0	Endothelial cell
IFI27	0	1.622385433	0.972	0.603	0	Endothelial cell
GNG11	0	1.450740922	0.959	0.575	0	Endothelial cell
CALCRL	0	1.420642889	0.968	0.557	0	Endothelial cell
SPARCL1	0	1.383597624	0.97	0.565	0	Endothelial cell
PECAM1	0	1.322924437	0.927	0.428	0	Endothelial cell
ID1	0	1.321421491	0.934	0.486	0	Endothelial cell
PLVAP	0	1.317195334	0.905	0.504	0	Endothelial cell
CLEC14A	0	1.30770245	0.92	0.438	0	Endothelial cell
SOX18	0	1.306826371	0.913	0.506	0	Endothelial cell
ACKR1	0	1.305617702	0.804	0.417	0	Endothelial cell
FABP4	0	1.293770827	0.879	0.491	0	Endothelial cell
TM4SF1	0	1.285899811	0.941	0.573	0	Endothelial cell
ECSCR	0	1.263454305	0.896	0.43	0	Endothelial cell
RNASE1	0	1.219396559	0.95	0.572	0	Endothelial cell
PALMD	0	1.194757778	0.889	0.407	0	Endothelial cell
EMCN	0	1.191929652	0.918	0.564	0	Endothelial cell
EGFL7	0	1.180944895	0.898	0.397	0	Endothelial cell
VWF	0	1.172467269	0.817	0.373	0	Endothelial cell
HYAL2	0	1.162447909	0.909	0.47	0	Endothelial cell
ADGRL4	0	1.151101696	0.918	0.501	0	Endothelial cell
NPDC1	0	1.143669632	0.91	0.504	0	Endothelial cell
RBP7	0	1.130547009	0.902	0.474	0	Endothelial cell
RAMP3	0	1.129272998	0.856	0.472	0	Endothelial cell
IGFBP4	0	1.129269302	0.922	0.56	0	Endothelial cell
VWA1	0	1.112493248	0.898	0.461	0	Endothelial cell
HSPG2	0	1.109369548	0.904	0.421	0	Endothelial cell
CAV1	0	1.096083788	0.932	0.574	0	Endothelial cell
PRCP	0	1.076673894	0.903	0.495	0	Endothelial cell
SPRY1	0	1.033180622	0.9	0.55	0	Endothelial cell
ID3	0	0.991809705	0.926	0.601	0	Endothelial cell
SLC9A3R2	0	0.987397796	0.833	0.481	0	Endothelial cell
CRIP2	0	0.986032292	0.928	0.57	0	Endothelial cell
IGFBP7	0	0.972263842	0.981	0.736	0	Endothelial cell
ENG	0	0.963003024	0.922	0.474	0	Endothelial cell
CD59	0	0.952934014	0.935	0.53	0	Endothelial cell
AQP1	0	0.944309589	0.866	0.49	0	Endothelial cell
FKBP1A	0	0.929763117	0.936	0.599	0	Endothelial cell
IFITM3	0	0.917322335	0.975	0.701	0	Endothelial cell
EMP1	0	0.900309855	0.856	0.502	0	Endothelial cell
ESAM	0	0.883695186	0.859	0.437	0	Endothelial cell
PDLIM1	0	0.883437973	0.879	0.581	0	Endothelial cell
A2M	0	0.876303374	0.94	0.613	0	Endothelial cell
ARHGAP29	0	0.86952836	0.894	0.528	0	Endothelial cell
S100A16	0	0.864977249	0.894	0.463	0	Endothelial cell
POSTN	0	0.859706601	0.78	0.515	0	Endothelial cell
MARCKSL1	0	0.853590879	0.902	0.58	0	Endothelial cell
RDX	0	0.853301412	0.889	0.51	0	Endothelial cell

ITGA6	0	0.839833796	0.851	0.381	0	Endothelial cell
TM4SF18	0	0.837284492	0.86	0.53	0	Endothelial cell
LDB2	0	0.833399325	0.827	0.496	0	Endothelial cell
DNASE1L3	0	0.83212298	0.816	0.453	0	Endothelial cell
COL4A1	0	0.830063281	0.906	0.495	0	Endothelial cell
CLDN5	0	0.815561124	0.72	0.388	0	Endothelial cell
CYYR1	0	0.814556969	0.803	0.431	0	Endothelial cell
COL15A1	0	0.811549138	0.862	0.462	0	Endothelial cell
CAVIN2	0	0.79944382	0.739	0.41	0	Endothelial cell
MTUS1	0	0.794989272	0.786	0.438	0	Endothelial cell
S100A13	0	0.793660281	0.867	0.496	0	Endothelial cell
FLT1	0	0.788016341	0.814	0.382	0	Endothelial cell
JAM2	0	0.786201564	0.777	0.454	0	Endothelial cell
GSN	0	0.783590071	0.947	0.606	0	Endothelial cell
SPARC	0	0.782139414	0.921	0.589	0	Endothelial cell
CDH5	0	0.772305812	0.853	0.473	0	Endothelial cell
FAM110D	0	0.765015622	0.821	0.504	0	Endothelial cell
ADAM15	0	0.763865056	0.811	0.44	0	Endothelial cell
CD9	0	0.753410867	0.947	0.625	0	Endothelial cell
SNCG	0	0.752101819	0.829	0.555	0	Endothelial cell
MGST2	0	0.751381546	0.837	0.503	0	Endothelial cell
SPTBN1	0	0.750118721	0.858	0.458	0	Endothelial cell
ADIRF	0	0.7383046	0.885	0.528	0	Endothelial cell
CXorf36	0	0.736849492	0.809	0.449	0	Endothelial cell
CAVIN1	0	0.736091809	0.865	0.501	0	Endothelial cell
DUSP23	0	0.734365325	0.83	0.569	0	Endothelial cell
PLAT	0	0.733782427	0.734	0.471	0	Endothelial cell
MMRN1	0	0.72247698	0.718	0.41	0	Endothelial cell
CD93	0	0.716486666	0.827	0.401	0	Endothelial cell
CD34	0	0.711479983	0.735	0.387	0	Endothelial cell
KANK3	0	0.710118189	0.803	0.417	0	Endothelial cell
FAM167B	0	0.695175641	0.837	0.388	0	Endothelial cell
ICAM2	0	0.687331915	0.864	0.498	0	Endothelial cell
TSPAN7	0	0.686809969	0.717	0.382	0	Endothelial cell
CCL14	0	0.6821656	0.727	0.345	0	Endothelial cell
RGS16	0	0.679314026	0.821	0.621	0	Endothelial cell
IL3RA	0	0.677797568	0.784	0.46	0	Endothelial cell
ADAMTS1	0	0.67572991	0.736	0.449	0	Endothelial cell
SOX4	0	0.671160803	0.86	0.58	0	Endothelial cell
MMRN2	0	0.670725382	0.735	0.34	0	Endothelial cell
PODXL	0	0.667978218	0.793	0.384	0	Endothelial cell
NUAK1	0	0.663354931	0.782	0.372	0	Endothelial cell
COL18A1	0	0.662137931	0.879	0.475	0	Endothelial cell
NFIB	0	0.658238393	0.765	0.371	0	Endothelial cell
WWTR1	0	0.653747682	0.771	0.408	0	Endothelial cell
COL4A2	0	0.652363156	0.877	0.48	0	Endothelial cell
ZNF385D	0	0.645501522	0.766	0.433	0	Endothelial cell
ANXA2	0	0.642440635	0.966	0.794	0	Endothelial cell
MYCT1	0	0.63229707	0.737	0.346	0	Endothelial cell
LMO2	0	0.62836279	0.776	0.46	0	Endothelial cell
BCAM	0	0.627380389	0.743	0.429	0	Endothelial cell
HEG1	0	0.624649953	0.741	0.409	0	Endothelial cell
NR2F2	0	0.623466304	0.854	0.576	0	Endothelial cell
NNMT	0	0.622307393	0.858	0.57	0	Endothelial cell
NOSTRIN	0	0.621987237	0.718	0.387	0	Endothelial cell
MCTP1	0	0.618775045	0.775	0.46	0	Endothelial cell

CLU	0	0.618241414	0.779	0.484	0	Endothelial cell
CAVIN3	0	0.617105268	0.911	0.547	0	Endothelial cell
PRSS23	0	0.616997425	0.861	0.521	0	Endothelial cell
LAMA4	0	0.616817048	0.865	0.532	0	Endothelial cell
OLFM1	0	0.608956846	0.697	0.351	0	Endothelial cell
EFNA1	0	0.60147652	0.737	0.416	0	Endothelial cell
SOX17	0	0.597102768	0.781	0.282	0	Endothelial cell
TCIM	0	0.596137118	0.812	0.383	0	Endothelial cell
SLCO2A1	0	0.595020401	0.739	0.223	0	Endothelial cell
MALL	0	0.59353238	0.727	0.332	0	Endothelial cell
EFNB2	0	0.592261508	0.754	0.424	0	Endothelial cell
RASIP1	0	0.589083651	0.746	0.313	0	Endothelial cell
TIMP3	0	0.586727307	0.792	0.476	0	Endothelial cell
PLPP1	0	0.582174547	0.809	0.523	0	Endothelial cell
APLNR	0	0.579867591	0.799	0.377	0	Endothelial cell
BMPR2	0	0.575725643	0.75	0.437	0	Endothelial cell
THBD	0	0.563614083	0.803	0.518	0	Endothelial cell
HES1	0	0.560459244	0.714	0.497	0	Endothelial cell
TNFSF10	0	0.559447568	0.852	0.644	0	Endothelial cell
CNN3	0	0.555317988	0.811	0.531	0	Endothelial cell
PCDH17	0	0.554913948	0.787	0.391	0	Endothelial cell
ADGRF5	0	0.544381804	0.835	0.505	0	Endothelial cell
MGLL	0	0.54375375	0.788	0.468	0	Endothelial cell
SERPINH1	0	0.535201125	0.865	0.531	0	Endothelial cell
SELE	0	0.517213547	0.615	0.39	0	Endothelial cell
LGALS3	0	0.515118915	0.933	0.722	0	Endothelial cell
PTPRB	0	0.514852356	0.605	0.322	0	Endothelial cell
TUBB6	0	0.514140365	0.809	0.441	0	Endothelial cell
CD200	0	0.509360719	0.846	0.508	0	Endothelial cell
CCND1	0	0.506495039	0.817	0.58	0	Endothelial cell
NRP1	0	0.499501176	0.817	0.516	0	Endothelial cell
CAV2	0	0.499194297	0.738	0.438	0	Endothelial cell
LAMB1	0	0.49879849	0.773	0.448	0	Endothelial cell
IL33	0	0.495317429	0.64	0.388	0	Endothelial cell
RAPGEF5	0	0.495310744	0.768	0.405	0	Endothelial cell
KDR	0	0.487251053	0.785	0.427	0	Endothelial cell
ADAMTS9	0	0.48693511	0.674	0.322	0	Endothelial cell
CXCL12	0	0.484884499	0.756	0.627	0	Endothelial cell
TSC22D1	0	0.484000899	0.857	0.603	0	Endothelial cell
DUSP6	0	0.477626497	0.852	0.625	0	Endothelial cell
LMCD1	0	0.477503729	0.696	0.534	0	Endothelial cell
FOXC1	0	0.477218744	0.689	0.449	0	Endothelial cell
SPNS2	0	0.472600902	0.731	0.379	0	Endothelial cell
RASSF9	0	0.470903969	0.648	0.211	0	Endothelial cell
STC1	0	0.468565581	0.599	0.331	0	Endothelial cell
CD151	0	0.468207807	0.848	0.572	0	Endothelial cell
MDK	0	0.467832205	0.653	0.532	0	Endothelial cell
NES	0	0.46535284	0.648	0.391	0	Endothelial cell
NRN1	0	0.464744077	0.607	0.34	0	Endothelial cell
TMEM204	0	0.463462212	0.784	0.52	0	Endothelial cell
AKR1C3	0	0.46039428	0.682	0.515	0	Endothelial cell
C2CD4B	0	0.455516886	0.58	0.3	0	Endothelial cell
GRASP	0	0.454469518	0.742	0.552	0	Endothelial cell
FSCN1	0	0.451433078	0.805	0.52	0	Endothelial cell
RHOJ	0	0.450104107	0.728	0.398	0	Endothelial cell
FILIP1	0	0.449598651	0.768	0.376	0	Endothelial cell

KLF4	0	0.447329283	0.761	0.575	0	Endothelial cell
PDK4	0	0.439527551	0.694	0.475	0	Endothelial cell
PLK2	0	0.435877068	0.781	0.482	0	Endothelial cell
TINAGL1	0	0.429545086	0.765	0.467	0	Endothelial cell
ROBO4	0	0.428796419	0.652	0.409	0	Endothelial cell
C1orf54	0	0.426447669	0.814	0.486	0	Endothelial cell
DSTN	0	0.425560812	0.914	0.671	0	Endothelial cell
SELP	0	0.424945462	0.597	0.293	0	Endothelial cell
NRP2	0	0.41894825	0.799	0.494	0	Endothelial cell
KRT18	0	0.417273867	0.771	0.534	0	Endothelial cell
CTHRC1	0	0.416885576	0.775	0.516	0	Endothelial cell
APOLD1	0	0.412328446	0.671	0.406	0	Endothelial cell
IER3	0	0.409217577	0.84	0.652	0	Endothelial cell
ITM2A	0	0.407766496	0.856	0.713	0	Endothelial cell
CFH	0	0.407614931	0.847	0.514	0	Endothelial cell
PMP22	0	0.406448037	0.826	0.528	0	Endothelial cell
THSD7A	0	0.400493996	0.746	0.399	0	Endothelial cell
NRGN	0	0.399452622	0.807	0.415	0	Endothelial cell
FAM241A	0	0.395533902	0.67	0.432	0	Endothelial cell
DNAJB4	0	0.393905912	0.687	0.519	0	Endothelial cell
FCGRT	0	0.392225891	0.853	0.596	0	Endothelial cell
PPIC	0	0.391179008	0.7	0.529	0	Endothelial cell
HTRA1	0	0.391086812	0.836	0.551	0	Endothelial cell
ITGB4	0	0.386308865	0.555	0.341	0	Endothelial cell
TIMP1	0	0.386158242	0.969	0.774	0	Endothelial cell
FBN1	0	0.38455589	0.784	0.408	0	Endothelial cell
TFPI	0	0.384151067	0.722	0.544	0	Endothelial cell
BACE2	0	0.382341326	0.647	0.43	0	Endothelial cell
FXYD6	0	0.379158546	0.691	0.393	0	Endothelial cell
S100A10	0	0.36861724	0.981	0.904	0	Endothelial cell
PROCR	0	0.368570619	0.689	0.423	0	Endothelial cell
NRARP	0	0.367587134	0.696	0.501	0	Endothelial cell
UACA	0	0.366766131	0.777	0.555	0	Endothelial cell
LINC01235	0	0.363995181	0.778	0.246	0	Endothelial cell
CD40	0	0.362684005	0.68	0.452	0	Endothelial cell
MGP	0	0.361548721	0.926	0.703	0	Endothelial cell
ASS1	0	0.359276684	0.598	0.396	0	Endothelial cell
ADM5	0	0.355348552	0.615	0.337	0	Endothelial cell
COX7A1	0	0.35382449	0.688	0.474	0	Endothelial cell
IFI6	0	0.341877452	0.763	0.568	0	Endothelial cell
MEOX1	0	0.341327108	0.663	0.363	0	Endothelial cell
ITGA10	0	0.34075525	0.635	0.349	0	Endothelial cell
RHOB	0	0.338526469	0.788	0.543	0	Endothelial cell
CTNNAL1	0	0.336181403	0.608	0.468	0	Endothelial cell
SYNPO	0	0.335446381	0.646	0.412	0	Endothelial cell
C7	0	0.331093694	0.702	0.526	0	Endothelial cell
CDA	0	0.330945939	0.666	0.368	0	Endothelial cell
AL583785.1	0	0.33065792	0.744	0.421	0	Endothelial cell
GJA1	0	0.330240565	0.597	0.332	0	Endothelial cell
APOD	0	0.327799031	0.692	0.366	0	Endothelial cell
PLPP3	0	0.31815319	0.644	0.319	0	Endothelial cell
TSPAN13	0	0.315073451	0.747	0.423	0	Endothelial cell
DAB2	0	0.313827707	0.828	0.571	0	Endothelial cell
INSR	0	0.31277979	0.574	0.302	0	Endothelial cell
TGM2	0	0.312197998	0.681	0.398	0	Endothelial cell
MARCKS	0	0.311343545	0.876	0.687	0	Endothelial cell

RAB3C	0	0.310937505	0.634	0.219	0	Endothelial cell
TNXB	0	0.309638939	0.588	0.297	0	Endothelial cell
F2R	0	0.307356378	0.751	0.51	0	Endothelial cell
TMEM88	0	0.304785266	0.579	0.372	0	Endothelial cell
SELENOP	0	0.303214821	0.831	0.641	0	Endothelial cell
SNAI1	0	0.300819589	0.742	0.413	0	Endothelial cell
MSX1	0	0.299975832	0.723	0.334	0	Endothelial cell
SOX7	0	0.296014849	0.561	0.305	0	Endothelial cell
PHLDA3	0	0.29212602	0.803	0.515	0	Endothelial cell
DPYSL3	0	0.287771348	0.633	0.369	0	Endothelial cell
RGS3	0	0.284995738	0.701	0.486	0	Endothelial cell
PNP	0	0.284659479	0.746	0.562	0	Endothelial cell
CDH13	0	0.284305381	0.685	0.304	0	Endothelial cell
FLRT2	0	0.284029517	0.761	0.224	0	Endothelial cell
MPZL2	0	0.280739944	0.543	0.25	0	Endothelial cell
GATA2	0	0.265948401	0.603	0.331	0	Endothelial cell
IER5L	0	0.264979528	0.822	0.642	0	Endothelial cell
CNKSR3	0	0.262619631	0.594	0.357	0	Endothelial cell
RCAN1	0	0.259129243	0.66	0.469	0	Endothelial cell
STAB1	0	0.259120139	0.705	0.527	0	Endothelial cell
FAM43A	0	0.258196044	0.682	0.462	0	Endothelial cell
ANKRD28	0	0.257557203	0.869	0.649	0	Endothelial cell
IFIT1	0	0.257228742	0.676	0.532	0	Endothelial cell
IGFBP5	0	0.255834969	0.788	0.529	0	Endothelial cell
SELENOM	8.48E-296	0.263592019	0.845	0.623	1.70E-292	Endothelial cell
CDKN1C	3.40E-289	0.257535308	0.72	0.586	6.81E-286	Endothelial cell
FABP5	7.33E-280	0.275485213	0.79	0.637	1.47E-276	Endothelial cell
LTC4S	5.88E-278	0.367587023	0.657	0.541	1.18E-274	Endothelial cell
IFI44L	1.27E-247	0.354534364	0.662	0.599	2.54E-244	Endothelial cell
CYP1B1	2.78E-232	0.252245286	0.526	0.283	5.56E-229	Endothelial cell
GPR146	1.15E-228	0.341854918	0.523	0.322	2.30E-225	Endothelial cell
FBLN2	1.29E-215	0.31587079	0.531	0.351	2.58E-212	Endothelial cell
MCAM	5.41E-211	0.287454732	0.599	0.465	1.08E-207	Endothelial cell
LXN	4.62E-210	0.309617797	0.603	0.504	9.24E-207	Endothelial cell
CNTNAP3B	1.04E-186	0.366010132	0.497	0.234	2.09E-183	Endothelial cell
FAM69B	8.32E-123	0.344432592	0.501	0.321	1.66E-119	Endothelial cell
CST3	2.10E-73	0.252866528	0.947	0.706	4.21E-70	Endothelial cell
EDN1	1.37E-50	0.364925779	0.461	0.359	2.75E-47	Endothelial cell
ACTA2	0	1.721782038	0.917	0.434	0	Smooth muscle cell
TAGLN	0	1.684313347	0.941	0.483	0	Smooth muscle cell
CALD1	0	1.525129862	0.974	0.524	0	Smooth muscle cell
TPM2	0	1.501502084	0.891	0.448	0	Smooth muscle cell
MYL9	0	1.497487635	0.894	0.413	0	Smooth muscle cell
RGS5	0	1.324195651	0.812	0.395	0	Smooth muscle cell
TPM1	0	1.300009096	0.923	0.516	0	Smooth muscle cell
SOD3	0	1.199818928	0.802	0.443	0	Smooth muscle cell
BGN	0	1.173069152	0.863	0.495	0	Smooth muscle cell
MYH11	0	1.169154916	0.782	0.318	0	Smooth muscle cell
C11orf96	0	1.167910658	0.839	0.448	0	Smooth muscle cell
MGP	0	1.160120367	0.981	0.691	0	Smooth muscle cell
IGFBP7	0	1.15236349	0.972	0.734	0	Smooth muscle cell
CTGF	0	1.147428304	0.944	0.528	0	Smooth muscle cell
ADIRF	0	1.143171218	0.861	0.527	0	Smooth muscle cell
PPP1R14A	0	1.084276389	0.756	0.39	0	Smooth muscle cell
FRZB	0	1.060756976	0.801	0.43	0	Smooth muscle cell
MFGE8	0	1.057135439	0.837	0.438	0	Smooth muscle cell

CYR61	0	1.054235852	0.865	0.521	0	Smooth muscle cell
MAP1B	0	1.041787718	0.833	0.415	0	Smooth muscle cell
IGFBP5	0	1.014955725	0.814	0.521	0	Smooth muscle cell
IGFBP2	0	0.96958902	0.82	0.526	0	Smooth muscle cell
LMOD1	0	0.967277918	0.755	0.25	0	Smooth muscle cell
ID4	0	0.965258776	0.825	0.417	0	Smooth muscle cell
CPE	0	0.960482989	0.885	0.466	0	Smooth muscle cell
AEBP1	0	0.95318872	0.849	0.47	0	Smooth muscle cell
DSTN	0	0.947952445	0.897	0.67	0	Smooth muscle cell
CRYAB	0	0.921507082	0.797	0.458	0	Smooth muscle cell
COL14A1	0	0.901794512	0.774	0.426	0	Smooth muscle cell
FHL1	0	0.901724	0.81	0.453	0	Smooth muscle cell
MYH10	0	0.86127687	0.694	0.369	0	Smooth muscle cell
OGN	0	0.853302312	0.729	0.368	0	Smooth muscle cell
FN1	0	0.847753525	0.873	0.528	0	Smooth muscle cell
PLAC9	0	0.843647394	0.811	0.486	0	Smooth muscle cell
C1R	0	0.841774905	0.875	0.445	0	Smooth muscle cell
C2orf40	0	0.832906587	0.87	0.542	0	Smooth muscle cell
LHFPL6	0	0.829988702	0.771	0.419	0	Smooth muscle cell
NR2F2	0	0.822943785	0.91	0.563	0	Smooth muscle cell
VCAN	0	0.816666345	0.883	0.475	0	Smooth muscle cell
NEXN	0	0.81523153	0.686	0.378	0	Smooth muscle cell
SPARCL1	0	0.814246461	0.876	0.574	0	Smooth muscle cell
NOV	0	0.814088851	0.757	0.451	0	Smooth muscle cell
ITGA8	0	0.81194838	0.701	0.356	0	Smooth muscle cell
GUCY1A1	0	0.80994305	0.752	0.425	0	Smooth muscle cell
SERPING1	0	0.802283096	0.859	0.515	0	Smooth muscle cell
A2M	0	0.787854189	0.903	0.614	0	Smooth muscle cell
MFAP4	0	0.784461338	0.611	0.333	0	Smooth muscle cell
CAVIN3	0	0.777772287	0.844	0.553	0	Smooth muscle cell
FILIP1L	0	0.775063645	0.804	0.514	0	Smooth muscle cell
SELENOM	0	0.773392282	0.801	0.627	0	Smooth muscle cell
CSRP1	0	0.772678959	0.773	0.496	0	Smooth muscle cell
TINAGL1	0	0.770532966	0.822	0.453	0	Smooth muscle cell
PDE5A	0	0.76465681	0.702	0.364	0	Smooth muscle cell
PLS3	0	0.754138045	0.778	0.522	0	Smooth muscle cell
CLU	0	0.75289748	0.762	0.483	0	Smooth muscle cell
NNMT	0	0.751560503	0.903	0.558	0	Smooth muscle cell
FXYD1	0	0.744808742	0.677	0.441	0	Smooth muscle cell
EFEMP1	0	0.744808645	0.801	0.402	0	Smooth muscle cell
SMOC2	0	0.743722942	0.84	0.367	0	Smooth muscle cell
PDLIM3	0	0.736873515	0.716	0.432	0	Smooth muscle cell
RARRES2	0	0.736605948	0.781	0.48	0	Smooth muscle cell
MYLK	0	0.736163395	0.752	0.381	0	Smooth muscle cell
GEM	0	0.735875387	0.843	0.549	0	Smooth muscle cell
CD151	0	0.732167055	0.799	0.576	0	Smooth muscle cell
PLN	0	0.711375079	0.654	0.289	0	Smooth muscle cell
EGR1	0	0.710017214	0.864	0.544	0	Smooth muscle cell
COL1A2	0	0.70924985	0.791	0.485	0	Smooth muscle cell
NUPR1	0	0.707518876	0.758	0.442	0	Smooth muscle cell
TGFB1I1	0	0.705579505	0.727	0.38	0	Smooth muscle cell
DKK3	0	0.70511076	0.773	0.41	0	Smooth muscle cell
LTBP1	0	0.70132374	0.701	0.332	0	Smooth muscle cell
RAMP1	0	0.699173826	0.593	0.393	0	Smooth muscle cell
NDUFA4L2	0	0.688386762	0.685	0.405	0	Smooth muscle cell
CAV1	0	0.687464408	0.836	0.585	0	Smooth muscle cell

RCAN2	0	0.681289237	0.714	0.538	0	Smooth muscle cell
PALLD	0	0.680797429	0.715	0.42	0	Smooth muscle cell
HCFC1R1	0	0.663279876	0.822	0.519	0	Smooth muscle cell
CSRP2	0	0.659608324	0.692	0.37	0	Smooth muscle cell
EDIL3	0	0.657986307	0.768	0.486	0	Smooth muscle cell
CAVIN1	0	0.649162151	0.788	0.508	0	Smooth muscle cell
PPP1R12B	0	0.648368008	0.655	0.347	0	Smooth muscle cell
IFITM3	0	0.643197885	0.925	0.705	0	Smooth muscle cell
COL6A2	0	0.623157336	0.791	0.539	0	Smooth muscle cell
CARMN	0	0.612429266	0.654	0.289	0	Smooth muscle cell
SPARC	0	0.607586467	0.848	0.596	0	Smooth muscle cell
PRRX1	0	0.605221742	0.715	0.339	0	Smooth muscle cell
PCOLCE	0	0.598031674	0.837	0.477	0	Smooth muscle cell
SSPN	0	0.596308486	0.738	0.441	0	Smooth muscle cell
PMEPA1	0	0.582951024	0.842	0.554	0	Smooth muscle cell
FMO2	0	0.582121738	0.78	0.412	0	Smooth muscle cell
SEPT4	0	0.579676796	0.87	0.45	0	Smooth muscle cell
MCAM	0	0.579528092	0.65	0.455	0	Smooth muscle cell
FBLIM1	0	0.577352136	0.65	0.394	0	Smooth muscle cell
C9orf3	0	0.574371941	0.706	0.456	0	Smooth muscle cell
CRIP2	0	0.565038083	0.817	0.583	0	Smooth muscle cell
UACA	0	0.564493061	0.812	0.546	0	Smooth muscle cell
COX7A1	0	0.563826249	0.721	0.466	0	Smooth muscle cell
PAWR	0	0.55867042	0.678	0.465	0	Smooth muscle cell
SLC25A4	0	0.557575602	0.702	0.44	0	Smooth muscle cell
ADAMTS1	0	0.556254574	0.749	0.442	0	Smooth muscle cell
C12orf75	0	0.552804619	0.747	0.545	0	Smooth muscle cell
TIMP1	0	0.552412294	0.967	0.772	0	Smooth muscle cell
C1S	0	0.550189759	0.771	0.376	0	Smooth muscle cell
GAS6	0	0.549052253	0.782	0.597	0	Smooth muscle cell
CAV2	0	0.54518989	0.664	0.446	0	Smooth muscle cell
PDGFRB	0	0.540584363	0.69	0.343	0	Smooth muscle cell
MSRB3	0	0.537977108	0.708	0.362	0	Smooth muscle cell
PRKG1	0	0.530161555	0.638	0.303	0	Smooth muscle cell
ASPN	0	0.528660433	0.682	0.336	0	Smooth muscle cell
ID3	0	0.522596285	0.795	0.618	0	Smooth muscle cell
CCDC102B	0	0.503932833	0.869	0.432	0	Smooth muscle cell
NET1	0	0.502469465	0.693	0.399	0	Smooth muscle cell
GSN	0	0.502001154	0.861	0.615	0	Smooth muscle cell
RERGL	0	0.499807695	0.661	0.307	0	Smooth muscle cell
COL18A1	0	0.499745499	0.68	0.502	0	Smooth muscle cell
COL8A1	0	0.499546775	0.672	0.341	0	Smooth muscle cell
NOTCH3	0	0.499388871	0.737	0.329	0	Smooth muscle cell
CD9	0	0.496792279	0.899	0.628	0	Smooth muscle cell
CNN3	0	0.495540136	0.86	0.519	0	Smooth muscle cell
ANGPT1	0	0.49256889	0.68	0.392	0	Smooth muscle cell
ANGPT2	0	0.490422347	0.732	0.503	0	Smooth muscle cell
THBS2	0	0.490233943	0.799	0.411	0	Smooth muscle cell
CCDC3	0	0.487545406	0.691	0.403	0	Smooth muscle cell
PDGFA	0	0.479622618	0.756	0.501	0	Smooth muscle cell
MT1E	0	0.479005268	0.723	0.561	0	Smooth muscle cell
RRAD	0	0.473400694	0.655	0.489	0	Smooth muscle cell
TSC22D1	0	0.464266521	0.826	0.604	0	Smooth muscle cell
TFPI	0	0.461529828	0.876	0.516	0	Smooth muscle cell
ISYNA1	0	0.460631958	0.664	0.445	0	Smooth muscle cell
CNN1	0	0.457952668	0.554	0.22	0	Smooth muscle cell

EFHD1	0	0.457887398	0.578	0.293	0	Smooth muscle cell
SVIL	0	0.455043657	0.68	0.461	0	Smooth muscle cell
CCDC80	0	0.450900435	0.827	0.517	0	Smooth muscle cell
NBL1	0	0.449019647	0.801	0.541	0	Smooth muscle cell
FOXC1	0	0.448582047	0.67	0.448	0	Smooth muscle cell
VASN	0	0.443910422	0.659	0.38	0	Smooth muscle cell
LGALS3BP	0	0.441215144	0.842	0.517	0	Smooth muscle cell
HES4	0	0.439922544	0.712	0.494	0	Smooth muscle cell
CFH	0	0.439453577	0.876	0.504	0	Smooth muscle cell
LTBP4	0	0.438686841	0.672	0.42	0	Smooth muscle cell
STEAP4	0	0.438201785	0.873	0.508	0	Smooth muscle cell
EFEMP2	0	0.434118201	0.769	0.399	0	Smooth muscle cell
ENAH	0	0.432918059	0.67	0.406	0	Smooth muscle cell
C1QTNF1	0	0.429913245	0.683	0.313	0	Smooth muscle cell
SGCA	0	0.429235851	0.746	0.391	0	Smooth muscle cell
MT1M	0	0.427459341	0.617	0.328	0	Smooth muscle cell
GUCY1B1	0	0.425783641	0.702	0.366	0	Smooth muscle cell
SNCG	0	0.424044958	0.713	0.57	0	Smooth muscle cell
ANTXR1	0	0.422018698	0.654	0.367	0	Smooth muscle cell
LGALS1	0	0.420012401	0.856	0.832	0	Smooth muscle cell
LMCD1	0	0.41981718	0.672	0.536	0	Smooth muscle cell
TNFRSF11B	0	0.417128444	0.697	0.431	0	Smooth muscle cell
JUN	0	0.416101778	0.936	0.794	0	Smooth muscle cell
KCNMB1	0	0.413498263	0.626	0.36	0	Smooth muscle cell
PGF	0	0.407299675	0.759	0.475	0	Smooth muscle cell
COL4A2	0	0.405229591	0.721	0.5	0	Smooth muscle cell
NRGN	0	0.401296221	0.679	0.431	0	Smooth muscle cell
COL3A1	0	0.401219237	0.692	0.487	0	Smooth muscle cell
CP	0	0.397134901	0.79	0.34	0	Smooth muscle cell
PCDH7	0	0.396852311	0.585	0.295	0	Smooth muscle cell
KCNE4	0	0.393688249	0.726	0.412	0	Smooth muscle cell
ITGA1	0	0.381121554	0.637	0.461	0	Smooth muscle cell
PRRX2	0	0.370203957	0.695	0.338	0	Smooth muscle cell
GSTM3	0	0.369775509	0.745	0.598	0	Smooth muscle cell
SULF1	0	0.366242581	0.633	0.324	0	Smooth muscle cell
AGT	0	0.36085406	0.775	0.345	0	Smooth muscle cell
LUM	0	0.359496221	0.852	0.545	0	Smooth muscle cell
GPX3	0	0.357174582	0.679	0.415	0	Smooth muscle cell
PTN	0	0.356823034	0.76	0.323	0	Smooth muscle cell
RGS16	0	0.352096517	0.87	0.61	0	Smooth muscle cell
COL1A1	0	0.348882894	0.647	0.438	0	Smooth muscle cell
ARHGAP29	0	0.348406163	0.792	0.54	0	Smooth muscle cell
FGF7	0	0.34649453	0.796	0.491	0	Smooth muscle cell
AKAP12	0	0.346045465	0.643	0.391	0	Smooth muscle cell
TBX2	0	0.34580677	0.623	0.299	0	Smooth muscle cell
CCL19	0	0.343706699	0.84	0.642	0	Smooth muscle cell
APOE	0	0.341113127	0.706	0.443	0	Smooth muscle cell
LTBP2	0	0.339567648	0.709	0.44	0	Smooth muscle cell
HES1	0	0.329604737	0.751	0.488	0	Smooth muscle cell
GPRC5C	0	0.328408437	0.707	0.357	0	Smooth muscle cell
SGCE	0	0.327351402	0.65	0.369	0	Smooth muscle cell
MBNL1-AS1	0	0.324567517	0.601	0.375	0	Smooth muscle cell
RASL11A	0	0.323443887	0.656	0.46	0	Smooth muscle cell
CXCL12	0	0.320409666	0.792	0.619	0	Smooth muscle cell
CDH11	0	0.318036054	0.792	0.456	0	Smooth muscle cell
CYBRD1	0	0.30750837	0.677	0.413	0	Smooth muscle cell

SDC2	0	0.306354415	0.673	0.381	0	Smooth muscle cell
TWIST1	0	0.297982122	0.661	0.355	0	Smooth muscle cell
PDLIM1	0	0.296024292	0.797	0.59	0	Smooth muscle cell
COL4A1	0	0.294389831	0.733	0.517	0	Smooth muscle cell
CCL2	0	0.29092719	0.804	0.492	0	Smooth muscle cell
TMEM98	0	0.289503014	0.602	0.327	0	Smooth muscle cell
GJA4	0	0.283606702	0.667	0.346	0	Smooth muscle cell
SUGCT	0	0.282729384	0.689	0.414	0	Smooth muscle cell
PPIC	0	0.28253351	0.712	0.524	0	Smooth muscle cell
EDNRA	0	0.280868828	0.694	0.334	0	Smooth muscle cell
S100A6	0	0.278606087	0.933	0.937	0	Smooth muscle cell
ELN	0	0.275686216	0.592	0.268	0	Smooth muscle cell
WISP2	0	0.272734416	0.611	0.341	0	Smooth muscle cell
CDO1	0	0.270556217	0.619	0.313	0	Smooth muscle cell
NUDT4	0	0.269071707	0.717	0.458	0	Smooth muscle cell
TM4SF1	0	0.268119743	0.797	0.591	0	Smooth muscle cell
PHLDA3	0	0.267459946	0.69	0.529	0	Smooth muscle cell
S100A4	0	0.266296852	0.916	0.89	0	Smooth muscle cell
TBX2-AS1	0	0.256529232	0.588	0.141	0	Smooth muscle cell
HGF	0	0.255859084	0.739	0.409	0	Smooth muscle cell
DCN	0	0.254662043	0.771	0.405	0	Smooth muscle cell
COX4I2	0	0.252815859	0.617	0.206	0	Smooth muscle cell
SLC7A2	0	0.252301654	0.62	0.276	0	Smooth muscle cell
PRELP	6.05E-304	0.514428966	0.604	0.338	1.21E-300	Smooth muscle cell
HTRA1	8.54E-299	0.389613436	0.725	0.565	1.71E-295	Smooth muscle cell
RND3	5.01E-298	0.253854362	0.659	0.439	1.00E-294	Smooth muscle cell
COL6A1	2.14E-296	0.401817702	0.65	0.451	4.28E-293	Smooth muscle cell
S100A13	3.77E-289	0.251556083	0.73	0.513	7.55E-286	Smooth muscle cell
LBH	7.55E-289	0.288902713	0.792	0.634	1.51E-285	Smooth muscle cell
RBP1	1.59E-288	0.252333281	0.609	0.39	3.19E-285	Smooth muscle cell
OMD	6.62E-284	0.288245139	0.573	0.285	1.32E-280	Smooth muscle cell
IGFBP6	8.96E-280	0.536491281	0.629	0.436	1.79E-276	Smooth muscle cell
EPB41L2	3.18E-277	0.340192769	0.684	0.509	6.36E-274	Smooth muscle cell
TIMP3	1.50E-276	0.387196465	0.666	0.492	3.01E-273	Smooth muscle cell
LMO3	4.28E-275	0.263058197	0.554	0.254	8.55E-272	Smooth muscle cell
CD59	2.80E-273	0.303449173	0.765	0.552	5.60E-270	Smooth muscle cell
SYNPO2	9.90E-260	0.4079308	0.571	0.33	1.98E-256	Smooth muscle cell
INHBA	3.89E-256	0.294503908	0.583	0.324	7.79E-253	Smooth muscle cell
ATF3	6.38E-256	0.31106137	0.793	0.607	1.28E-252	Smooth muscle cell
SORBS2	3.99E-252	0.430616009	0.593	0.389	7.98E-249	Smooth muscle cell
PTP4A3	5.69E-249	0.272702378	0.621	0.468	1.14E-245	Smooth muscle cell
SPINT2	5.47E-242	0.361825936	0.707	0.557	1.09E-238	Smooth muscle cell
LRRC17	4.63E-240	0.252344628	0.588	0.355	9.27E-237	Smooth muscle cell
EMILIN1	1.23E-237	0.301929248	0.578	0.362	2.46E-234	Smooth muscle cell
MT2A	4.06E-235	0.294108845	0.9	0.773	8.12E-232	Smooth muscle cell
ADH1B	1.81E-229	0.508145389	0.548	0.268	3.61E-226	Smooth muscle cell
CST3	1.81E-220	0.348481634	0.914	0.708	3.63E-217	Smooth muscle cell
COPZ2	1.15E-216	0.26432393	0.615	0.415	2.31E-213	Smooth muscle cell
SPTBN1	2.09E-211	0.291260485	0.675	0.483	4.17E-208	Smooth muscle cell
FSTL1	2.28E-198	0.286903208	0.603	0.411	4.56E-195	Smooth muscle cell
WTIP	1.87E-189	0.346510942	0.534	0.27	3.74E-186	Smooth muscle cell
FEZ1	1.00E-185	0.273732653	0.574	0.395	2.00E-182	Smooth muscle cell
SLC40A1	1.06E-184	0.274813652	0.643	0.491	2.12E-181	Smooth muscle cell
RERG	3.80E-180	0.250754921	0.533	0.228	7.60E-177	Smooth muscle cell
TNFRSF12A	5.51E-179	0.299252645	0.665	0.548	1.10E-175	Smooth muscle cell
FOXC2	1.32E-168	0.334505773	0.533	0.261	2.63E-165	Smooth muscle cell

DEPP1	6.76E-168	0.319302099	0.556	0.348	1.35E-164	Smooth muscle cell
MRGPRF	2.62E-147	0.281275851	0.5	0.124	5.23E-144	Smooth muscle cell
FILIP1	1.76E-136	0.32749421	0.573	0.403	3.53E-133	Smooth muscle cell
GMDS	2.36E-135	0.2893735	0.602	0.485	4.72E-132	Smooth muscle cell
TMEM47	9.74E-124	0.338838698	0.543	0.354	1.95E-120	Smooth muscle cell
LIMCH1	1.01E-117	0.357000434	0.558	0.383	2.02E-114	Smooth muscle cell
NFIB	7.18E-110	0.329548841	0.587	0.394	1.44E-106	Smooth muscle cell
PTGIS	2.52E-101	0.298502097	0.508	0.303	5.04E-98	Smooth muscle cell
CRIM1	6.36E-100	0.349876229	0.546	0.42	1.27E-96	Smooth muscle cell
MXRA8	1.70E-98	0.31297425	0.554	0.42	3.40E-95	Smooth muscle cell
FBLN5	5.21E-81	0.332628496	0.491	0.29	1.04E-77	Smooth muscle cell
ACTC1	3.58E-77	0.291657237	0.477	0.285	7.17E-74	Smooth muscle cell
PDLIM7	1.11E-59	0.369286334	0.542	0.454	2.21E-56	Smooth muscle cell
COL21A1	2.53E-56	0.283389491	0.559	0.47	5.05E-53	Smooth muscle cell
LMO7	5.81E-34	0.263806146	0.498	0.397	1.16E-30	Smooth muscle cell
ACTG2	6.61E-28	0.314364974	0.446	0.223	1.32E-24	Smooth muscle cell
PRDM6	1.24E-16	0.257381958	0.431	0.183	2.48E-13	Smooth muscle cell
ITGBL1	1.97E-13	0.331382343	0.47	0.344	3.94E-10	Smooth muscle cell
GNLY	0	2.092920516	0.998	0.489	0	NK_cell
NKG7	0	1.704319283	0.999	0.643	0	NK_cell
FGFBP2	0	1.604327896	0.926	0.31	0	NK_cell
GZMB	0	1.425504489	0.934	0.591	0	NK_cell
GZMH	0	1.400544756	0.938	0.6	0	NK_cell
PRF1	0	1.267323011	0.903	0.469	0	NK_cell
KLRD1	0	1.258897282	0.922	0.494	0	NK_cell
CTSW	0	1.162015735	0.911	0.499	0	NK_cell
CCL5	0	1.160958533	0.988	0.796	0	NK_cell
CST7	0	1.118956791	0.982	0.74	0	NK_cell
HOPX	0	1.042616352	0.895	0.595	0	NK_cell
GZMA	0	1.026964848	0.983	0.772	0	NK_cell
CCL4	0	1.018788586	0.986	0.824	0	NK_cell
GZMM	0	1.00011334	0.947	0.645	0	NK_cell
TRGC2	0	0.866048151	0.865	0.585	0	NK_cell
HCST	1.26E-269	0.711065578	0.976	0.774	2.51E-266	NK_cell
CD247	4.34E-218	0.904103396	0.818	0.593	8.68E-215	NK_cell
CD3D	2.16E-210	0.683760083	0.93	0.76	4.32E-207	NK_cell
CORO1A	1.36E-201	0.635105991	0.954	0.771	2.72E-198	NK_cell
IL32	7.66E-200	0.724772955	0.966	0.815	1.53E-196	NK_cell
ITGB2	1.74E-189	0.634133771	0.929	0.718	3.47E-186	NK_cell
CD52	5.62E-178	0.655022343	0.937	0.8	1.12E-174	NK_cell
C12orf75	4.94E-176	0.631680306	0.853	0.568	9.88E-173	NK_cell
CD3G	2.67E-175	0.702129319	0.864	0.713	5.33E-172	NK_cell
CD48	1.40E-169	0.592989202	0.935	0.772	2.80E-166	NK_cell
FCGR3A	7.90E-158	0.695392635	0.78	0.448	1.58E-154	NK_cell
EFHD2	1.92E-157	0.604335712	0.841	0.621	3.84E-154	NK_cell
SPON2	3.33E-149	0.86138928	0.69	0.397	6.67E-146	NK_cell
TRAC	6.21E-144	0.549014109	0.927	0.785	1.24E-140	NK_cell
RHOH	2.44E-132	0.417797823	0.87	0.69	4.89E-129	NK_cell
CYTIP	2.95E-128	0.516719056	0.913	0.78	5.90E-125	NK_cell
PRDM1	1.13E-127	0.559258697	0.818	0.659	2.26E-124	NK_cell
LCP1	2.79E-120	0.480803699	0.909	0.746	5.58E-117	NK_cell
DDIT4	2.92E-120	0.474469162	0.933	0.801	5.84E-117	NK_cell
SOCS1	5.04E-119	0.425583717	0.872	0.712	1.01E-115	NK_cell
TRBC1	3.85E-102	0.637529748	0.789	0.677	7.70E-99	NK_cell
MATK	2.31E-100	0.593291881	0.687	0.457	4.62E-97	NK_cell
CYBA	9.93E-93	0.358980541	0.985	0.906	1.99E-89	NK_cell

CCL4L2	2.89E-86	0.559169108	0.82	0.732	5.79E-83	NK_cell
CYFIP2	7.53E-86	0.28256438	0.664	0.476	1.51E-82	NK_cell
TRBC2	3.68E-82	0.487190555	0.852	0.782	7.36E-79	NK_cell
S100A4	1.85E-81	0.339732628	0.989	0.892	3.69E-78	NK_cell
PLAC8	6.24E-75	0.637599534	0.638	0.447	1.25E-71	NK_cell
CD3E	2.45E-70	0.512173489	0.773	0.699	4.90E-67	NK_cell
IRF1	2.51E-69	0.370282277	0.885	0.776	5.02E-66	NK_cell
CD160	3.57E-69	0.270781734	0.56	0.272	7.13E-66	NK_cell
GBP5	4.91E-68	0.379418862	0.709	0.571	9.83E-65	NK_cell
CD2	2.19E-66	0.478771611	0.832	0.758	4.38E-63	NK_cell
CD8A	6.87E-66	0.606407226	0.675	0.606	1.37E-62	NK_cell
CXCR4	4.36E-63	0.382735378	0.96	0.859	8.73E-60	NK_cell
UCP2	2.27E-62	0.449811081	0.716	0.579	4.54E-59	NK_cell
XCL2	3.96E-60	0.305975691	0.652	0.526	7.91E-57	NK_cell
CD69	1.14E-59	0.366857527	0.9	0.787	2.28E-56	NK_cell
ISG20	1.42E-58	0.375640281	0.786	0.716	2.83E-55	NK_cell
CD8B	2.60E-57	0.417886505	0.666	0.562	5.20E-54	NK_cell
CX3CR1	2.28E-55	0.389264903	0.6	0.36	4.57E-52	NK_cell
CLIC3	3.77E-55	0.448132506	0.599	0.446	7.53E-52	NK_cell
PTGER2	4.74E-52	0.367219195	0.62	0.446	9.47E-49	NK_cell
KLRB1	3.44E-49	0.519885113	0.681	0.619	6.88E-46	NK_cell
IFNG	4.07E-49	0.300078655	0.655	0.596	8.15E-46	NK_cell
ZNF683	4.62E-48	0.287565341	0.569	0.372	9.24E-45	NK_cell
XBP1	1.37E-47	0.357667513	0.764	0.652	2.74E-44	NK_cell
CD7	3.18E-47	0.562363104	0.649	0.569	6.35E-44	NK_cell
KLRF1	4.82E-45	0.604845009	0.57	0.403	9.64E-42	NK_cell
SRGN	5.66E-44	0.30446169	0.989	0.893	1.13E-40	NK_cell
AC243960.1	1.17E-41	0.309365131	0.618	0.506	2.34E-38	NK_cell
GLRX	4.24E-41	0.26169854	0.793	0.655	8.49E-38	NK_cell
CD37	9.33E-37	0.287529809	0.824	0.763	1.87E-33	NK_cell
TRDC	2.88E-32	0.476936313	0.54	0.415	5.76E-29	NK_cell
DUSP2	4.22E-30	0.298184484	0.818	0.806	8.45E-27	NK_cell
PTGDS	4.60E-23	0.280393589	0.577	0.482	9.20E-20	NK_cell
PLEK	1.87E-21	0.256216693	0.65	0.535	3.74E-18	NK_cell
NCR3	4.61E-16	0.450557488	0.526	0.465	9.23E-13	NK_cell
FKBP11	2.10E-12	0.256564927	0.614	0.582	4.21E-09	NK_cell
CPA3	0	2.16392425	0.975	0.294	0	Mast_cell
TPSAB1	0	2.131952072	0.962	0.299	0	Mast_cell
TPSB2	0	2.011728283	0.95	0.242	0	Mast_cell
HPGD	0	1.744812453	0.946	0.487	0	Mast_cell
MS4A2	5.21E-293	1.473868271	0.865	0.203	1.04E-289	Mast_cell
HPGDS	7.09E-283	1.528394201	0.906	0.375	1.42E-279	Mast_cell
ADCYAP1	1.61E-265	1.143851451	0.817	0.228	3.21E-262	Mast_cell
RAB27B	6.67E-265	1.191441467	0.885	0.456	1.33E-261	Mast_cell
RHEX	1.33E-249	1.173463059	0.815	0.257	2.66E-246	Mast_cell
HDC	2.38E-234	1.103795702	0.797	0.229	4.77E-231	Mast_cell
CTSG	5.96E-225	0.917788201	0.811	0.325	1.19E-221	Mast_cell
FTH1	1.89E-224	0.458996631	1	0.976	3.77E-221	Mast_cell
KIT	5.52E-216	1.118664381	0.808	0.273	1.10E-212	Mast_cell
GATA2	7.78E-215	1.240539196	0.833	0.361	1.56E-211	Mast_cell
VWA5A	4.26E-210	1.160436633	0.835	0.37	8.53E-207	Mast_cell
IL1RL1	1.70E-208	1.06518571	0.804	0.248	3.41E-205	Mast_cell
RGS13	1.74E-203	1.131122854	0.804	0.328	3.48E-200	Mast_cell
SLC18A2	2.55E-173	0.946903162	0.748	0.264	5.09E-170	Mast_cell
TMEM176B	3.53E-166	1.035692672	0.872	0.494	7.07E-163	Mast_cell
LTC4S	2.25E-159	1.092410026	0.827	0.553	4.50E-156	Mast_cell

ARHGAP18	3.72E-148	0.846617055	0.881	0.649	7.44E-145	Mast_cell
BIRC3	9.89E-132	0.946998471	0.876	0.659	1.98E-128	Mast_cell
RAB32	7.52E-129	0.760944756	0.826	0.524	1.50E-125	Mast_cell
FCER1G	2.82E-127	0.971230615	0.914	0.564	5.64E-124	Mast_cell
STXBP6	1.71E-119	0.624458363	0.71	0.389	3.42E-116	Mast_cell
AREG	3.96E-115	1.096800931	0.804	0.57	7.91E-112	Mast_cell
TMEM233	1.96E-111	0.397304641	0.633	0.177	3.92E-108	Mast_cell
NFKBIA	3.56E-107	0.504201838	0.937	0.91	7.12E-104	Mast_cell
TMEM176A	2.90E-105	0.676716463	0.784	0.486	5.80E-102	Mast_cell
MLPH	3.77E-102	0.565095328	0.647	0.219	7.54E-99	Mast_cell
CAPG	1.16E-101	0.694426114	0.815	0.525	2.32E-98	Mast_cell
ALOX5AP	1.45E-97	0.692827916	0.853	0.679	2.90E-94	Mast_cell
PROCR	1.00E-96	0.403112689	0.752	0.454	2.00E-93	Mast_cell
GLUL	7.15E-96	0.720320791	0.84	0.567	1.43E-92	Mast_cell
FCER1A	3.05E-93	0.861286177	0.7	0.416	6.10E-90	Mast_cell
AL157895.1	2.15E-92	0.767443192	0.656	0.286	4.31E-89	Mast_cell
S100A6	2.64E-90	0.344245114	0.991	0.936	5.28E-87	Mast_cell
SRGN	2.89E-86	0.453862666	0.977	0.894	5.77E-83	Mast_cell
CLU	1.86E-75	0.65807885	0.813	0.519	3.71E-72	Mast_cell
SAMSN1	4.00E-73	0.536839403	0.862	0.71	7.99E-70	Mast_cell
TYROBP	5.01E-73	0.673840732	0.896	0.58	1.00E-69	Mast_cell
BATF	1.54E-71	0.54357118	0.755	0.577	3.09E-68	Mast_cell
RGS10	2.76E-71	0.524176244	0.86	0.633	5.53E-68	Mast_cell
PLIN2	4.94E-69	0.570519265	0.84	0.677	9.87E-66	Mast_cell
S100A4	2.87E-66	0.377597364	0.975	0.893	5.73E-63	Mast_cell
CAVIN2	4.14E-63	0.65709514	0.68	0.45	8.27E-60	Mast_cell
CD9	1.77E-54	0.509484079	0.849	0.665	3.54E-51	Mast_cell
PHLDA2	6.14E-54	0.383270384	0.788	0.622	1.23E-50	Mast_cell
CST7	3.06E-53	0.311981056	0.872	0.743	6.12E-50	Mast_cell
STMN1	1.38E-50	0.497801543	0.763	0.616	2.77E-47	Mast_cell
LGALS3	5.83E-50	0.42900824	0.901	0.748	1.17E-46	Mast_cell
DBI	5.34E-49	0.395800352	0.894	0.756	1.07E-45	Mast_cell
ASAH1	2.76E-48	0.517180442	0.737	0.592	5.52E-45	Mast_cell
ALDH1A1	2.65E-47	0.575026425	0.619	0.394	5.30E-44	Mast_cell
LXN	3.04E-46	0.310677707	0.721	0.515	6.07E-43	Mast_cell
ALOX5	3.77E-46	0.604615604	0.629	0.427	7.55E-43	Mast_cell
ANKRD28	2.68E-43	0.538988141	0.763	0.677	5.36E-40	Mast_cell
AKAP12	1.20E-42	0.370928356	0.64	0.424	2.40E-39	Mast_cell
SSR4	1.48E-42	0.27727387	0.917	0.871	2.96E-39	Mast_cell
PLAT	6.88E-39	0.632273151	0.638	0.504	1.38E-35	Mast_cell
ITM2C	7.34E-38	0.444354469	0.691	0.567	1.47E-34	Mast_cell
PYCARD	7.55E-35	0.300996139	0.827	0.682	1.51E-31	Mast_cell
KRT19	3.59E-34	0.519966881	0.563	0.305	7.18E-31	Mast_cell
CALB2	5.04E-34	0.515606387	0.522	0.158	1.01E-30	Mast_cell
CSF1	2.37E-32	0.451298024	0.617	0.483	4.73E-29	Mast_cell
TNFSF10	2.45E-32	0.420634417	0.743	0.67	4.90E-29	Mast_cell
SOCS1	2.24E-29	0.411736121	0.768	0.715	4.47E-26	Mast_cell
BACE2	3.74E-29	0.481589381	0.617	0.456	7.47E-26	Mast_cell
SCIN	2.88E-27	0.349480479	0.545	0.331	5.77E-24	Mast_cell
BTG2	1.70E-26	0.30979801	0.842	0.783	3.40E-23	Mast_cell
VEGFA	1.16E-25	0.463431789	0.61	0.484	2.33E-22	Mast_cell
CD69	1.29E-25	0.39844041	0.808	0.789	2.58E-22	Mast_cell
EGR1	1.68E-25	0.362623843	0.723	0.588	3.35E-22	Mast_cell
SYAP1	5.42E-25	0.452435242	0.662	0.56	1.08E-21	Mast_cell
RGS2	1.45E-23	0.378695648	0.727	0.654	2.91E-20	Mast_cell
PLAUR	5.41E-23	0.336405776	0.728	0.619	1.08E-19	Mast_cell

SOX4	1.52E-21	0.363954056	0.725	0.615	3.04E-18	Mast_cell
LPCAT2	1.37E-20	0.369608876	0.594	0.454	2.73E-17	Mast_cell
GADD45B	1.40E-18	0.263665032	0.779	0.747	2.81E-15	Mast_cell
CTSD	6.62E-18	0.259089936	0.75	0.679	1.32E-14	Mast_cell
PTGS1	7.85E-18	0.340864652	0.531	0.38	1.57E-14	Mast_cell
CCL4L2	8.93E-17	0.288469759	0.75	0.733	1.79E-13	Mast_cell
LAT2	2.77E-15	0.28188172	0.563	0.428	5.54E-12	Mast_cell
BMP2K	3.10E-15	0.389948227	0.583	0.513	6.19E-12	Mast_cell
RHOH	9.15E-15	0.279308471	0.689	0.694	1.83E-11	Mast_cell
DHRS9	2.10E-14	0.444104155	0.5	0.221	4.20E-11	Mast_cell
AP1S2	6.41E-11	0.264972026	0.624	0.563	1.28E-07	Mast_cell
ELL2	2.52E-08	0.327010506	0.54	0.518	5.05E-05	Mast_cell
PTGS2	3.17E-07	0.443876692	0.475	0.311	0.000634996	Mast_cell
CXCL16	4.61E-07	0.256860003	0.57	0.512	0.000921634	Mast_cell
DUSP6	6.27E-07	0.290842606	0.631	0.654	0.001254553	Mast_cell
CLEC4C	3.11E-129	1.264116824	0.984	0.097	6.23E-126	Plasmacytoid_DC
LAMP5	9.45E-92	1.230664656	0.968	0.348	1.89E-88	Plasmacytoid_DC
TCL1A	2.87E-90	1.626169605	0.976	0.44	5.74E-87	Plasmacytoid_DC
PLD4	9.60E-88	1.446553412	0.984	0.329	1.92E-84	Plasmacytoid_DC
GZMB	3.02E-83	1.914586395	1	0.596	6.04E-80	Plasmacytoid_DC
SMIM5	9.08E-81	0.71408057	0.919	0.279	1.82E-77	Plasmacytoid_DC
LILRA4	9.05E-79	1.305983011	0.935	0.226	1.81E-75	Plasmacytoid_DC
JCHAIN	9.59E-79	1.772965809	0.992	0.716	1.92E-75	Plasmacytoid_DC
PLAC8	4.97E-78	1.460089427	0.984	0.45	9.94E-75	Plasmacytoid_DC
IRF7	5.53E-78	1.598505477	0.984	0.539	1.11E-74	Plasmacytoid_DC
MZB1	2.29E-77	1.456936465	0.992	0.644	4.58E-74	Plasmacytoid_DC
DERL3	1.82E-76	1.440497324	0.968	0.505	3.65E-73	Plasmacytoid_DC
SPIB	1.91E-76	1.303805772	0.968	0.43	3.82E-73	Plasmacytoid_DC
ITM2C	1.42E-72	1.289254338	0.992	0.567	2.84E-69	Plasmacytoid_DC
TSPAN13	2.78E-72	1.398174299	0.952	0.464	5.56E-69	Plasmacytoid_DC
IRF8	4.85E-71	1.32684026	0.968	0.553	9.70E-68	Plasmacytoid_DC
SHD	9.03E-71	0.713639705	0.742	0.031	1.81E-67	Plasmacytoid_DC
IL3RA	6.38E-70	1.209710209	0.96	0.501	1.28E-66	Plasmacytoid_DC
IRF4	1.71E-68	1.18772049	0.96	0.496	3.42E-65	Plasmacytoid_DC
HERPUD1	6.17E-68	0.951521287	1	0.839	1.23E-64	Plasmacytoid_DC
HHIP-AS1	1.35E-66	0.405605004	0.839	0.161	2.70E-63	Plasmacytoid_DC
SERPINF1	7.51E-66	1.187413973	0.984	0.549	1.50E-62	Plasmacytoid_DC
ALOX5AP	4.11E-65	1.14816956	0.984	0.68	8.22E-62	Plasmacytoid_DC
BCL11A	9.56E-62	1.09429897	0.911	0.417	1.91E-58	Plasmacytoid_DC
CYBA	1.50E-61	0.694437057	1	0.907	3.01E-58	Plasmacytoid_DC
RNASE6	4.03E-61	1.056978909	0.944	0.499	8.06E-58	Plasmacytoid_DC
HSP90B1	5.61E-61	0.766266776	1	0.828	1.12E-57	Plasmacytoid_DC
CLIC3	1.73E-60	1.458225281	0.919	0.448	3.46E-57	Plasmacytoid_DC
MAP1A	1.10E-58	1.039909068	0.911	0.436	2.21E-55	Plasmacytoid_DC
MYBL2	1.24E-58	0.667597874	0.798	0.212	2.49E-55	Plasmacytoid_DC
LILRB4	3.50E-58	0.974282124	0.944	0.43	7.01E-55	Plasmacytoid_DC
CCDC88A	1.06E-57	0.995028625	0.96	0.537	2.11E-54	Plasmacytoid_DC
SMPD3	8.02E-57	0.982675162	0.879	0.4	1.60E-53	Plasmacytoid_DC
C12orf75	2.48E-54	1.062969874	0.952	0.572	4.96E-51	Plasmacytoid_DC
RAB11FIP1	4.92E-54	1.009138026	0.944	0.569	9.84E-51	Plasmacytoid_DC
EGLN3	5.39E-54	0.90310783	0.887	0.427	1.08E-50	Plasmacytoid_DC
RNASET2	6.81E-52	0.879482188	0.976	0.687	1.36E-48	Plasmacytoid_DC
GPR183	1.34E-51	1.015377113	0.992	0.701	2.67E-48	Plasmacytoid_DC
SSR4	3.98E-50	0.58942088	0.992	0.871	7.95E-47	Plasmacytoid_DC
PTPRE	2.10E-48	0.879254142	0.976	0.551	4.20E-45	Plasmacytoid_DC
EIF4A3	2.62E-48	0.817880153	0.984	0.701	5.24E-45	Plasmacytoid_DC

CYBB	2.62E-48	0.883413622	0.976	0.528	5.25E-45	Plasmacytoid_DC
TNFRSF17	3.92E-48	0.372548538	0.823	0.494	7.84E-45	Plasmacytoid_DC
MPEG1	7.16E-48	0.657126095	0.879	0.372	1.43E-44	Plasmacytoid_DC
LYN	9.99E-48	0.784786163	0.952	0.512	2.00E-44	Plasmacytoid_DC
THEMIS2	2.62E-47	0.865278414	0.871	0.419	5.25E-44	Plasmacytoid_DC
CXCR3	8.05E-46	0.817437139	0.919	0.574	1.61E-42	Plasmacytoid_DC
NUCB2	9.83E-46	0.814786379	0.992	0.666	1.97E-42	Plasmacytoid_DC
GRASP	2.99E-45	0.894463367	0.911	0.576	5.99E-42	Plasmacytoid_DC
TPM2	3.95E-44	0.992355368	0.992	0.51	7.90E-41	Plasmacytoid_DC
MS4A6A	7.00E-44	0.907979297	0.96	0.551	1.40E-40	Plasmacytoid_DC
IGKC	1.03E-43	0.427971012	1	0.98	2.05E-40	Plasmacytoid_DC
SCT	3.46E-43	0.793348815	0.758	0.156	6.92E-40	Plasmacytoid_DC
FAM30A	4.70E-43	0.307534193	0.815	0.514	9.40E-40	Plasmacytoid_DC
PLEK	4.72E-43	0.863676745	0.968	0.536	9.44E-40	Plasmacytoid_DC
HMGB3	5.13E-43	0.291099792	0.815	0.406	1.03E-39	Plasmacytoid_DC
STMN1	3.68E-42	0.835244425	0.935	0.616	7.37E-39	Plasmacytoid_DC
MMP23B	2.12E-41	0.450737015	0.839	0.444	4.23E-38	Plasmacytoid_DC
PMEPA1	3.11E-41	0.793988486	0.944	0.594	6.22E-38	Plasmacytoid_DC
FCER1A	7.10E-41	0.469606569	0.798	0.418	1.42E-37	Plasmacytoid_DC
CHPF	1.63E-39	0.252136463	0.839	0.461	3.25E-36	Plasmacytoid_DC
SHTN1	1.83E-39	0.550533543	0.871	0.448	3.67E-36	Plasmacytoid_DC
CD74	2.00E-39	0.705468134	1	0.898	4.01E-36	Plasmacytoid_DC
HLA-DQA2	3.35E-39	0.759533431	0.992	0.711	6.69E-36	Plasmacytoid_DC
HLA-DMA	2.60E-37	0.766821406	0.992	0.652	5.20E-34	Plasmacytoid_DC
PNOC	2.67E-37	0.440058067	0.75	0.382	5.33E-34	Plasmacytoid_DC
KYNU	3.67E-37	0.363798928	0.823	0.44	7.34E-34	Plasmacytoid_DC
KCNK17	5.88E-37	0.334712757	0.774	0.302	1.18E-33	Plasmacytoid_DC
SELL	5.96E-37	0.884992572	0.855	0.427	1.19E-33	Plasmacytoid_DC
VAMP8	5.60E-36	0.758496306	0.935	0.689	1.12E-32	Plasmacytoid_DC
CKS2	4.32E-35	0.619301126	0.927	0.672	8.63E-32	Plasmacytoid_DC
UCP2	6.12E-35	0.729160674	0.919	0.581	1.22E-31	Plasmacytoid_DC
NPC2	8.26E-35	0.687782788	0.976	0.723	1.65E-31	Plasmacytoid_DC
NCF1	9.46E-35	0.743633097	0.887	0.587	1.89E-31	Plasmacytoid_DC
HLA-DRA	1.60E-34	0.852857385	1	0.816	3.20E-31	Plasmacytoid_DC
DAAM1	3.33E-34	0.580644694	0.887	0.5	6.65E-31	Plasmacytoid_DC
PYCARD	1.29E-33	0.635374644	0.96	0.683	2.57E-30	Plasmacytoid_DC
FKBP2	3.88E-33	0.61182428	0.968	0.728	7.76E-30	Plasmacytoid_DC
DUSP5	1.10E-32	0.845543457	0.855	0.636	2.19E-29	Plasmacytoid_DC
SEC11C	2.03E-32	0.624762474	0.919	0.639	4.07E-29	Plasmacytoid_DC
EAF2	4.76E-32	0.349641819	0.806	0.501	9.51E-29	Plasmacytoid_DC
HLA-DPB1	1.09E-31	0.711802011	0.984	0.789	2.18E-28	Plasmacytoid_DC
DBI	1.27E-30	0.590056724	0.968	0.757	2.55E-27	Plasmacytoid_DC
FCER1G	1.43E-30	0.879067298	0.968	0.567	2.85E-27	Plasmacytoid_DC
CD4	2.67E-30	0.644091533	0.831	0.455	5.35E-27	Plasmacytoid_DC
HLA-DQA1	5.57E-30	0.676350899	0.992	0.661	1.11E-26	Plasmacytoid_DC
P2RY14	6.97E-30	0.804113716	0.758	0.398	1.39E-26	Plasmacytoid_DC
XBP1	8.03E-30	0.589927535	0.935	0.654	1.61E-26	Plasmacytoid_DC
EZR	1.25E-29	0.636195657	0.984	0.785	2.50E-26	Plasmacytoid_DC
SNX10	8.00E-29	0.276177785	0.839	0.495	1.60E-25	Plasmacytoid_DC
ATF5	2.64E-28	0.480930488	0.798	0.43	5.29E-25	Plasmacytoid_DC
HLA-DPA1	2.84E-28	0.612722484	0.992	0.803	5.68E-25	Plasmacytoid_DC
JAML	6.59E-28	0.726593618	0.806	0.455	1.32E-24	Plasmacytoid_DC
HLA-DRB5	1.04E-27	0.644220853	1	0.797	2.08E-24	Plasmacytoid_DC
HLA-DMB	1.19E-27	0.605379939	0.911	0.553	2.39E-24	Plasmacytoid_DC
CTSZ	2.26E-27	0.600397092	0.968	0.697	4.52E-24	Plasmacytoid_DC
DNASE1L3	9.87E-27	0.61473732	0.798	0.499	1.97E-23	Plasmacytoid_DC

LAP3	9.90E-27	0.559278751	0.935	0.622	1.98E-23	Plasmacytoid_DC
CD55	2.46E-26	0.549685064	0.96	0.637	4.92E-23	Plasmacytoid_DC
TNFSF9	3.57E-26	0.274032857	0.831	0.524	7.15E-23	Plasmacytoid_DC
TYROBP	5.93E-25	0.831070228	0.96	0.582	1.19E-21	Plasmacytoid_DC
PTCRA	7.31E-25	0.853911654	0.71	0.26	1.46E-21	Plasmacytoid_DC
CAPG	1.42E-24	0.625854789	0.863	0.527	2.83E-21	Plasmacytoid_DC
MARCH1	2.25E-22	0.306705736	0.823	0.487	4.50E-19	Plasmacytoid_DC
HLA-DQB1	5.13E-22	0.554422026	0.944	0.685	1.03E-18	Plasmacytoid_DC
MGST2	7.39E-22	0.479412852	0.855	0.546	1.48E-18	Plasmacytoid_DC
PIM2	1.21E-21	0.352028068	0.847	0.489	2.42E-18	Plasmacytoid_DC
OAS1	1.79E-21	0.302014467	0.79	0.508	3.59E-18	Plasmacytoid_DC
BTG2	2.28E-21	0.483695865	0.96	0.783	4.57E-18	Plasmacytoid_DC
CD37	2.92E-21	0.545072174	0.919	0.764	5.83E-18	Plasmacytoid_DC
TMEM70	6.11E-21	0.400304183	0.903	0.612	1.22E-17	Plasmacytoid_DC
CTSS	7.15E-21	0.525310445	0.96	0.683	1.43E-17	Plasmacytoid_DC
GAPT	1.03E-20	0.684151388	0.718	0.399	2.07E-17	Plasmacytoid_DC
HLA-DRB1	3.68E-20	0.502195219	0.984	0.791	7.35E-17	Plasmacytoid_DC
LGMN	3.32E-19	0.348292376	0.823	0.536	6.65E-16	Plasmacytoid_DC
GRN	1.95E-18	0.4566695	0.935	0.652	3.89E-15	Plasmacytoid_DC
KPNA2	2.25E-18	0.52146904	0.806	0.626	4.50E-15	Plasmacytoid_DC
NRP1	4.89E-18	0.319491968	0.863	0.554	9.78E-15	Plasmacytoid_DC
CXCR4	5.89E-18	0.542171979	0.976	0.86	1.18E-14	Plasmacytoid_DC
SPINT2	1.63E-17	0.518376773	0.798	0.578	3.26E-14	Plasmacytoid_DC
GAS6	1.01E-16	0.503794839	0.823	0.622	2.03E-13	Plasmacytoid_DC
GNA15	1.64E-16	0.396709968	0.702	0.406	3.27E-13	Plasmacytoid_DC
CYCS	5.42E-16	0.389113225	0.952	0.761	1.08E-12	Plasmacytoid_DC
CTSB	6.49E-16	0.432125744	0.903	0.592	1.30E-12	Plasmacytoid_DC
SDF2L1	2.47E-15	0.320187759	0.879	0.672	4.94E-12	Plasmacytoid_DC
RGS10	2.69E-15	0.367099926	0.935	0.634	5.37E-12	Plasmacytoid_DC
PHACTR1	4.88E-15	0.433793392	0.766	0.478	9.76E-12	Plasmacytoid_DC
AP1S2	6.53E-15	0.458361244	0.798	0.563	1.31E-11	Plasmacytoid_DC
CST3	7.32E-15	0.526414935	0.992	0.737	1.46E-11	Plasmacytoid_DC
TUBB6	1.69E-14	0.279984298	0.839	0.488	3.38E-11	Plasmacytoid_DC
DSTN	1.88E-14	0.407623435	0.968	0.701	3.75E-11	Plasmacytoid_DC
EVI2B	7.28E-14	0.285355864	0.911	0.654	1.46E-10	Plasmacytoid_DC
ZNF331	7.55E-14	0.434586716	0.887	0.67	1.51E-10	Plasmacytoid_DC
ERN1	1.15E-13	0.624498278	0.702	0.485	2.30E-10	Plasmacytoid_DC
SOX4	1.68E-13	0.399668291	0.782	0.615	3.36E-10	Plasmacytoid_DC
ISG20	1.84E-12	0.255470632	0.879	0.717	3.67E-09	Plasmacytoid_DC
ODC1	3.10E-12	0.469615662	0.758	0.635	6.20E-09	Plasmacytoid_DC
CTSC	9.32E-12	0.337288986	0.935	0.782	1.86E-08	Plasmacytoid_DC
CDKN1A	9.88E-12	0.258661768	0.895	0.659	1.98E-08	Plasmacytoid_DC
MX1	6.42E-11	0.372493672	0.839	0.622	1.28E-07	Plasmacytoid_DC
PFKFB3	1.06E-10	0.266562886	0.839	0.554	2.11E-07	Plasmacytoid_DC
LCP1	2.28E-10	0.369191954	0.911	0.749	4.56E-07	Plasmacytoid_DC
TKT	5.05E-10	0.282268338	0.919	0.658	1.01E-06	Plasmacytoid_DC
PLEKHO1	5.77E-10	0.275841299	0.895	0.653	1.15E-06	Plasmacytoid_DC
HCST	6.64E-10	0.323000466	0.976	0.778	1.33E-06	Plasmacytoid_DC
AC097375.1	8.54E-10	0.409442136	0.524	0.083	1.71E-06	Plasmacytoid_DC
SLAMF7	1.12E-09	0.352157821	0.637	0.448	2.25E-06	Plasmacytoid_DC
DDIT4	2.39E-09	0.29264673	0.984	0.803	4.78E-06	Plasmacytoid_DC
CD68	8.05E-09	0.405080986	0.742	0.505	1.61E-05	Plasmacytoid_DC
AREG	2.31E-08	0.627465693	0.694	0.572	4.62E-05	Plasmacytoid_DC
BID	1.03E-07	0.377100323	0.685	0.578	0.000206612	Plasmacytoid_DC
PSAP	1.13E-07	0.283478204	0.935	0.672	0.000225729	Plasmacytoid_DC
TGFBI	2.53E-07	0.32112826	0.677	0.529	0.000506899	Plasmacytoid_DC

SRGN	5.02E-07	0.329832216	0.992	0.895	0.001004944	Plasmacytoid_DC
CHMP1B	6.23E-07	0.273919623	0.718	0.548	0.001245717	Plasmacytoid_DC
IGHM	2.02E-06	0.505853198	0.621	0.604	0.004046935	Plasmacytoid_DC
LAPTM5	5.02E-06	0.270462848	0.911	0.719	0.010047392	Plasmacytoid_DC
LAIR1	8.21E-06	0.309609957	0.629	0.446	0.016428961	Plasmacytoid_DC

Table S2. Markers for smooth muscle cells (SMCs)

gene	p_val	avg_log2FC	pct.1	pct.2	p_val_adj	cluster
CALD1	0	2.21159312	0.919	0.574	0	SMC2
VCAN	0	1.66158269	0.858	0.519	0	SMC2
CCL19	0	1.35443475	0.862	0.662	0	SMC2
CTGF	0	1.27395999	0.901	0.574	0	SMC2
THBS2	0	1.2170724	0.792	0.452	0	SMC2
ENAH	0	1.17088848	0.776	0.429	0	SMC2
CCDC102B	0	1.05879293	0.87	0.478	0	SMC2
MGP	0	0.95374259	0.945	0.723	0	SMC2
CDH11	0	0.86049193	0.805	0.491	0	SMC2
CCL21	0	0.80212056	0.811	0.537	0	SMC2
TNC	0	0.771592	0.854	0.496	0	SMC2
STEAP4	0	0.74395955	0.873	0.546	0	SMC2
ABCA6	0	0.54897064	0.721	0.367	0	SMC2
KCNE4	0	0.54800234	0.785	0.442	0	SMC2
LAMA2	0	0.54304043	0.694	0.347	0	SMC2
TPH1	0	0.34692976	0.759	0.326	0	SMC2
FSTL3	0	0.32741401	0.148	0.374	0	SMC2
MECOM	0	0.31742678	0.715	0.344	0	SMC2
ZNF503	0	0.28885016	0.224	0.496	0	SMC2
SEPT4	0	0.27671594	0.83	0.496	0	SMC2
MAP1A	7.88E-300	0.47258861	0.724	0.425	1.58E-296	SMC2
FGF7	3.35E-282	0.47666443	0.795	0.523	6.69E-279	SMC2
SMOC2	1.22E-280	0.57876344	0.779	0.42	2.44E-277	SMC2
ACTA2	5.61E-280	1.08713132	0.803	0.49	1.12E-276	SMC2
CP	1.37E-272	0.51729123	0.765	0.388	2.75E-269	SMC2
HGF	1.92E-259	0.49789443	0.76	0.443	3.84E-256	SMC2
TPM1	1.29E-249	1.78489689	0.795	0.565	2.58E-246	SMC2
KRT17	2.33E-249	0.31373803	0.747	0.428	4.67E-246	SMC2
SGCA	4.85E-244	0.26346771	0.748	0.428	9.71E-241	SMC2
INSR	1.81E-241	0.28826962	0.196	0.343	3.62E-238	SMC2
RAMP1	3.14E-234	0.49518624	0.245	0.428	6.28E-231	SMC2
TAGLN	9.40E-232	0.6090022	0.829	0.536	1.88E-228	SMC2
AC023157.3	3.64E-230	0.42999736	0.741	0.513	7.28E-227	SMC2
TWIST1	4.94E-229	0.38735248	0.71	0.385	9.89E-226	SMC2
F3	1.56E-220	0.2800471	0.741	0.512	3.13E-217	SMC2
FAP	8.46E-219	0.25420629	0.775	0.467	1.69E-215	SMC2
CDH2	3.15E-218	0.38574196	0.638	0.324	6.29E-215	SMC2
ANGPTL4	1.92E-212	0.35632816	0.685	0.377	3.84E-209	SMC2
INHBA	3.52E-207	0.71498302	0.685	0.347	7.05E-204	SMC2
AEBP1	8.07E-206	1.89230367	0.741	0.515	1.61E-202	SMC2
CDH13	2.09E-202	0.5451855	0.246	0.357	4.19E-199	SMC2
GRIA2	1.57E-196	0.33708039	0.241	0.271	3.13E-193	SMC2
ABCC9	9.46E-196	0.31773286	0.238	0.343	1.89E-192	SMC2
RCAN2	5.93E-194	0.30538102	0.324	0.573	1.19E-190	SMC2
MEDAG	1.30E-193	0.31255724	0.646	0.292	2.59E-190	SMC2
PDLIM7	1.77E-190	0.48857329	0.27	0.475	3.54E-187	SMC2
ADAMTS1	7.68E-187	1.92265607	0.711	0.476	1.54E-183	SMC2
LAMA4	6.04E-186	0.54475633	0.808	0.565	1.21E-182	SMC2
MT1M	1.48E-183	0.35955658	0.263	0.374	2.96E-180	SMC2
RGS4	2.29E-183	0.28088564	0.639	0.32	4.57E-180	SMC2
FMO2	1.74E-182	0.7469736	0.719	0.453	3.48E-179	SMC2

LMO7	1.91E-180	0.41447863	0.259	0.418	3.82E-177	SMC2
DAAM1	1.11E-178	0.50412408	0.282	0.51	2.21E-175	SMC2
TMEM47	3.68E-176	0.35746984	0.254	0.387	7.35E-173	SMC2
DNAJB4	3.39E-169	0.30481835	0.325	0.549	6.78E-166	SMC2
FXYD1	1.69E-168	0.53357992	0.286	0.482	3.39E-165	SMC2
EFEMP2	5.43E-166	0.48231105	0.718	0.44	1.09E-162	SMC2
KCNMA1	4.40E-164	0.45928261	0.28	0.433	8.81E-161	SMC2
HBB	5.37E-164	0.82816458	0.11	0.222	1.07E-160	SMC2
TFPI	6.06E-160	0.60010866	0.817	0.556	1.21E-156	SMC2
MFAP4	2.78E-158	0.39590444	0.27	0.377	5.56E-155	SMC2
ITGBL1	6.87E-158	0.70991768	0.264	0.366	1.37E-154	SMC2
AKAP12	6.47E-157	0.92922145	0.669	0.416	1.29E-153	SMC2
SOX4	2.38E-156	0.85541604	0.832	0.607	4.76E-153	SMC2
CCDC80	1.59E-154	1.25516355	0.755	0.552	3.19E-151	SMC2
NT5DC2	2.09E-153	0.30535368	0.71	0.442	4.19E-150	SMC2
GJA1	1.81E-152	0.50584207	0.667	0.354	3.61E-149	SMC2
OSR1	1.60E-151	0.32142893	0.228	0.225	3.21E-148	SMC2
FN1	2.02E-149	1.39316906	0.747	0.569	4.04E-146	SMC2
FILIP1	1.16E-146	0.58998404	0.301	0.432	2.32E-143	SMC2
PLN	9.43E-146	0.41962812	0.276	0.343	1.89E-142	SMC2
DCN	3.40E-140	0.4316632	0.704	0.446	6.81E-137	SMC2
HSPG2	1.39E-135	0.3491954	0.314	0.491	2.77E-132	SMC2
NR2F2	6.78E-135	0.96467885	0.796	0.604	1.36E-131	SMC2
CPXM2	3.49E-132	0.62264512	0.659	0.418	6.98E-129	SMC2
EFEMP1	4.82E-132	0.86704598	0.708	0.448	9.64E-129	SMC2
PFN2	5.72E-132	0.26146043	0.268	0.394	1.14E-128	SMC2
GPC6	2.11E-130	0.47584975	0.245	0.221	4.22E-127	SMC2
GGT5	1.57E-129	0.33607373	0.756	0.521	3.14E-126	SMC2
IGFBP3	3.78E-127	0.3259373	0.239	0.388	7.55E-124	SMC2
ECM2	4.97E-126	0.355828	0.276	0.328	9.95E-123	SMC2
CFH	4.57E-125	0.71319766	0.795	0.547	9.15E-122	SMC2
MXRA8	5.53E-124	0.46587681	0.311	0.444	1.11E-120	SMC2
MEG3	3.52E-123	0.97534753	0.573	0.259	7.03E-120	SMC2
USP53	8.88E-122	0.77135145	0.278	0.438	1.78E-118	SMC2
GEM	1.24E-117	0.75955465	0.737	0.584	2.47E-114	SMC2
FBLIM1	5.26E-117	0.72895331	0.313	0.435	1.05E-113	SMC2
C7	2.62E-116	0.41494339	0.753	0.54	5.24E-113	SMC2
MBNL1-AS1	5.32E-116	0.26904223	0.627	0.397	1.06E-112	SMC2
RGS16	6.89E-116	0.40918738	0.832	0.639	1.38E-112	SMC2
PTN	7.61E-111	0.26415542	0.688	0.372	1.52E-107	SMC2
NNMT	1.60E-105	0.66730831	0.786	0.6	3.20E-102	SMC2
EDNRA	9.67E-104	0.26425897	0.618	0.375	1.93E-100	SMC2
WISP2	6.06E-103	0.43700224	0.616	0.369	1.21E-99	SMC2
CPE	1.31E-102	0.39954623	0.727	0.516	2.61E-99	SMC2
MSRB3	1.03E-101	0.75876036	0.66	0.401	2.06E-98	SMC2
PAWR	2.68E-97	0.69630511	0.347	0.502	5.35E-94	SMC2
NTRK2	3.79E-96	0.26926616	0.335	0.431	7.58E-93	SMC2
PRELP	4.72E-96	0.78786329	0.312	0.378	9.44E-93	SMC2
C1R	3.32E-94	0.98044871	0.699	0.498	6.65E-91	SMC2
EGR1	3.36E-91	1.44752405	0.718	0.584	6.71E-88	SMC2
MT1E	1.02E-90	0.478573	0.42	0.591	2.05E-87	SMC2
COL5A2	2.50E-89	0.54679056	0.353	0.465	5.00E-86	SMC2
TBX2	5.44E-89	0.52818888	0.325	0.345	1.09E-85	SMC2

CNTN4	6.76E-89	0.2766784	0.525	0.157	1.35E-85	SMC2
LAMB1	3.73E-88	0.35175197	0.375	0.495	7.45E-85	SMC2
ISLR	1.47E-87	0.52183868	0.613	0.338	2.95E-84	SMC2
ALCAM	1.27E-86	0.3855232	0.349	0.444	2.53E-83	SMC2
SYNPO	2.11E-85	0.42441626	0.344	0.447	4.23E-82	SMC2
WTIP	1.10E-83	0.50603782	0.304	0.307	2.20E-80	SMC2
MYO1B	1.34E-82	0.47406459	0.683	0.505	2.67E-79	SMC2
GDF7	7.15E-82	0.27925719	0.277	0.254	1.43E-78	SMC2
HMCN1	1.18E-80	0.74479373	0.298	0.299	2.36E-77	SMC2
PPP1R12B	2.50E-80	1.95202193	0.576	0.383	5.01E-77	SMC2
FOXC2	7.17E-78	0.55599457	0.308	0.299	1.43E-74	SMC2
FBN1	1.69E-77	0.73981543	0.371	0.46	3.37E-74	SMC2
SLPI	3.74E-75	0.43825044	0.648	0.459	7.49E-72	SMC2
PDGFD	2.17E-74	0.61134347	0.615	0.397	4.35E-71	SMC2
CRIM1	2.57E-73	0.77218845	0.329	0.443	5.14E-70	SMC2
NOTCH3	2.57E-73	0.63177084	0.608	0.378	5.14E-70	SMC2
MRC2	1.36E-70	0.8405012	0.595	0.424	2.72E-67	SMC2
SEMA5A	2.27E-69	0.3578377	0.573	0.301	4.54E-66	SMC2
SUGCT	2.68E-69	0.27505413	0.627	0.446	5.35E-66	SMC2
SLIT2	1.36E-68	0.63765176	0.566	0.326	2.71E-65	SMC2
LGALS3BP	4.77E-67	0.2691632	0.754	0.555	9.55E-64	SMC2
UACA	2.14E-66	1.73289685	0.672	0.58	4.28E-63	SMC2
CD151	1.83E-65	0.3004139	0.461	0.614	3.66E-62	SMC2
COX7A1	3.34E-64	0.25585313	0.417	0.505	6.67E-61	SMC2
COL21A1	2.31E-63	1.10585077	0.626	0.477	4.62E-60	SMC2
CARMN	4.03E-63	2.02839856	0.564	0.331	8.05E-60	SMC2
FLRT2	7.73E-61	0.27184507	0.333	0.292	1.55E-57	SMC2
IGFBP6	1.33E-58	0.77626951	0.361	0.468	2.65E-55	SMC2
ABHD5	2.01E-58	0.33138551	0.709	0.54	4.02E-55	SMC2
PDLIM3	3.92E-57	0.76687003	0.385	0.476	7.84E-54	SMC2
SYNPO2	5.83E-56	0.97376339	0.343	0.366	1.17E-52	SMC2
WWTR1	1.14E-54	0.38435355	0.658	0.446	2.29E-51	SMC2
MYL9	8.91E-54	0.61146662	0.665	0.474	1.78E-50	SMC2
PDGFRA	1.96E-53	0.72485637	0.361	0.35	3.91E-50	SMC2
CCL2	3.71E-53	0.28638628	0.75	0.527	7.43E-50	SMC2
ARHGAP29	3.00E-52	0.43638642	0.712	0.57	6.00E-49	SMC2
COL1A1	1.01E-51	1.25924854	0.605	0.462	2.01E-48	SMC2
CD248	1.38E-51	0.28861533	0.349	0.311	2.76E-48	SMC2
COL6A3	5.37E-51	0.90664699	0.618	0.49	1.07E-47	SMC2
ITGA10	6.00E-51	0.79752926	0.359	0.387	1.20E-47	SMC2
MYH11	8.07E-51	1.7162703	0.56	0.376	1.61E-47	SMC2
HES4	6.05E-50	0.36219398	0.451	0.528	1.21E-46	SMC2
NFIB	5.97E-49	0.74890029	0.382	0.423	1.19E-45	SMC2
HTRA1	3.03E-48	0.25504716	0.499	0.592	6.05E-45	SMC2
KCNQ1OT1	1.07E-47	0.81103847	0.619	0.487	2.13E-44	SMC2
LHFPL6	2.14E-47	0.33845056	0.421	0.471	4.28E-44	SMC2
PALLD	2.20E-46	0.8763812	0.388	0.465	4.40E-43	SMC2
TPM2	4.70E-46	0.58894518	0.66	0.505	9.39E-43	SMC2
ANK2	3.12E-45	0.30270183	0.561	0.31	6.23E-42	SMC2
FBLN5	7.38E-45	0.55882512	0.312	0.319	1.48E-41	SMC2
SGCE	5.92E-44	0.30905687	0.378	0.41	1.18E-40	SMC2
FHL1	1.91E-43	1.14998229	0.625	0.499	3.81E-40	SMC2
SVEP1	2.05E-43	0.31593549	0.511	0.238	4.11E-40	SMC2

CSRP2	7.10E-42	0.27820526	0.41	0.416	1.42E-38	SMC2
GPX3	8.13E-42	0.8474256	0.404	0.455	1.63E-38	SMC2
MAP1B	1.74E-39	2.00740195	0.586	0.469	3.48E-36	SMC2
ABCA9	2.99E-37	0.26750863	0.316	0.221	5.97E-34	SMC2
COL5A1	5.06E-37	0.59219771	0.54	0.333	1.01E-33	SMC2
COL14A1	7.54E-37	0.63476644	0.425	0.478	1.51E-33	SMC2
NUDT4	3.53E-36	0.70810444	0.642	0.489	7.05E-33	SMC2
VASN	3.69E-36	0.31399482	0.384	0.421	7.37E-33	SMC2
MFGE8	5.67E-36	1.24661344	0.599	0.49	1.13E-32	SMC2
COL4A1	7.41E-36	0.44051465	0.685	0.542	1.48E-32	SMC2
HSPB7	4.19E-35	0.26609606	0.508	0.312	8.39E-32	SMC2
CRISPLD2	7.53E-34	0.46163959	0.344	0.296	1.51E-30	SMC2
NOV	9.36E-34	0.70351214	0.605	0.49	1.87E-30	SMC2
LIMCH1	1.68E-33	0.5807973	0.393	0.409	3.36E-30	SMC2
PTGDS	3.21E-33	0.53790061	0.653	0.476	6.43E-30	SMC2
ABCA8	9.08E-33	0.61615811	0.491	0.258	1.82E-29	SMC2
SERPINH1	4.31E-32	0.41580961	0.695	0.569	8.63E-29	SMC2
NUPR1	8.79E-32	0.59136262	0.435	0.489	1.76E-28	SMC2
SERPINE1	1.04E-31	0.74163278	0.586	0.411	2.09E-28	SMC2
SYNM	4.35E-31	0.62782495	0.365	0.4	8.71E-28	SMC2
ID4	4.72E-31	0.76396717	0.606	0.469	9.44E-28	SMC2
GPRC5A	6.10E-31	0.52843135	0.502	0.309	1.22E-27	SMC2
AQP1	6.13E-31	0.48664575	0.455	0.542	1.23E-27	SMC2
SORBS2	7.17E-31	0.7192376	0.404	0.418	1.43E-27	SMC2
MYLK	5.52E-30	1.31900948	0.571	0.428	1.10E-26	SMC2
PRDM6	8.12E-30	0.80100369	0.333	0.213	1.62E-26	SMC2
JUN	3.52E-28	1.03995077	0.852	0.812	7.03E-25	SMC2
COL18A1	9.79E-28	0.81802919	0.443	0.531	1.96E-24	SMC2
PI16	1.60E-27	0.41202033	0.328	0.343	3.21E-24	SMC2
CRISPLD1	1.62E-27	0.42178257	0.422	0.464	3.24E-24	SMC2
SCARA5	2.27E-27	0.25641588	0.412	0.24	4.55E-24	SMC2
A2M	1.55E-26	0.75361126	0.742	0.651	3.10E-23	SMC2
LRRC17	2.87E-26	0.61769997	0.549	0.381	5.74E-23	SMC2
FKBP10	5.24E-26	0.3719969	0.576	0.404	1.05E-22	SMC2
DIO2	5.31E-26	0.29168102	0.539	0.358	1.06E-22	SMC2
CCDC144A	1.14E-25	0.40417051	0.359	0.353	2.29E-22	SMC2
NPY1R	1.65E-25	0.35730444	0.311	0.211	3.30E-22	SMC2
LRP1	9.12E-25	0.85724051	0.422	0.471	1.82E-21	SMC2
CYR61	4.22E-24	0.77415372	0.632	0.567	8.43E-21	SMC2
THBS1	8.17E-24	0.36598964	0.636	0.531	1.63E-20	SMC2
SULF1	2.20E-23	1.1504691	0.54	0.36	4.41E-20	SMC2
COL6A1	4.84E-23	1.01720687	0.426	0.481	9.67E-20	SMC2
PCOLCE	4.44E-21	0.3559512	0.63	0.524	8.87E-18	SMC2
PPP1R14A	6.22E-21	0.75336758	0.417	0.443	1.24E-17	SMC2
KCNAB1	1.03E-20	0.3184657	0.478	0.278	2.07E-17	SMC2
TNXB	3.92E-20	0.81238684	0.373	0.333	7.84E-17	SMC2
SFRP1	5.35E-20	0.25985108	0.486	0.232	1.07E-16	SMC2
SDC2	2.15E-19	0.26361584	0.601	0.415	4.31E-16	SMC2
CKB	1.12E-18	0.25044732	0.519	0.399	2.23E-15	SMC2
RERG	2.99E-18	0.28177039	0.495	0.262	5.98E-15	SMC2
OMD	4.32E-18	0.4264524	0.378	0.323	8.65E-15	SMC2
ANTXR1	6.89E-18	0.77045587	0.545	0.402	1.38E-14	SMC2
COL1A2	2.51E-17	1.31302972	0.58	0.526	5.03E-14	SMC2

MCAM	4.23E-17	1.08299367	0.425	0.485	8.46E-14	SMC2
TGFB1I1	4.41E-17	0.35883762	0.438	0.428	8.82E-14	SMC2
ASPN	8.72E-17	0.5040518	0.421	0.383	1.74E-13	SMC2
CRIP2	9.83E-17	0.25871646	0.559	0.619	1.97E-13	SMC2
CX3CL1	1.27E-16	0.34080906	0.494	0.284	2.54E-13	SMC2
FOXC1	1.79E-16	0.83452389	0.583	0.475	3.58E-13	SMC2
FSTL1	4.43E-16	0.80192257	0.556	0.433	8.86E-13	SMC2
SLC40A1	4.98E-16	0.29145955	0.492	0.514	9.95E-13	SMC2
SLIT3	9.56E-16	0.58545118	0.495	0.301	1.91E-12	SMC2
COL8A1	2.07E-15	0.69888036	0.416	0.387	4.15E-12	SMC2
PLAC9	2.67E-15	0.62040424	0.484	0.534	5.34E-12	SMC2
ART4	2.53E-14	0.50997156	0.483	0.324	5.06E-11	SMC2
RARRES2	3.18E-14	0.3932634	0.496	0.523	6.36E-11	SMC2
ATF3	7.63E-14	0.5319538	0.58	0.635	1.53E-10	SMC2
HEG1	2.46E-13	0.2704914	0.433	0.452	4.92E-10	SMC2
ITGA8	4.47E-13	1.46029423	0.515	0.401	8.93E-10	SMC2
NEXN	6.95E-13	0.8855694	0.43	0.421	1.39E-09	SMC2
ANKH	9.55E-13	0.41907877	0.514	0.521	1.91E-09	SMC2
GAS6	7.18E-12	0.71395137	0.579	0.625	1.44E-08	SMC2
COL12A1	1.27E-11	0.54839756	0.434	0.426	2.55E-08	SMC2
ITIH5	1.29E-11	0.29478163	0.411	0.349	2.57E-08	SMC2
FBXO32	1.59E-11	0.40569172	0.482	0.512	3.17E-08	SMC2
PDGFA	1.83E-11	0.34509743	0.605	0.534	3.67E-08	SMC2
FAM114A1	1.88E-11	0.39759891	0.606	0.504	3.75E-08	SMC2
SLC7A2	2.64E-11	0.54752749	0.512	0.317	5.29E-08	SMC2
RGS5	2.70E-11	0.96900519	0.539	0.45	5.40E-08	SMC2
DPYSL3	3.77E-11	0.62613509	0.435	0.401	7.53E-08	SMC2
SVIL	5.03E-11	0.85876452	0.452	0.494	1.01E-07	SMC2
HSPA1A	7.18E-11	1.00539752	0.873	0.753	1.44E-07	SMC2
LMO3	8.29E-11	0.53348532	0.403	0.292	1.66E-07	SMC2
EGR3	2.28E-10	0.27234304	0.437	0.445	4.57E-07	SMC2
CYBRD1	2.34E-10	0.68338203	0.559	0.446	4.68E-07	SMC2
BICC1	4.41E-10	0.41311647	0.408	0.334	8.82E-07	SMC2
INMT	5.02E-10	0.43267453	0.467	0.291	1.00E-06	SMC2
COL3A1	5.28E-10	1.07835153	0.565	0.514	1.06E-06	SMC2
PLK2	5.29E-10	0.28859143	0.623	0.516	1.06E-06	SMC2
DKK3	7.06E-10	0.50684883	0.565	0.457	1.41E-06	SMC2
TFPI2	6.68E-09	0.26106159	0.344	0.262	1.34E-05	SMC2
ANGPT1	8.67E-09	0.75433303	0.532	0.428	1.73E-05	SMC2
TNFRSF11B	1.06E-08	0.55039683	0.54	0.466	2.12E-05	SMC2
TSC22D1	1.14E-08	0.37458698	0.722	0.632	2.28E-05	SMC2
C1S	1.19E-08	0.774467	0.553	0.427	2.38E-05	SMC2
C1QTNF1	1.50E-08	0.67847791	0.519	0.359	3.00E-05	SMC2
SAMD11	2.45E-08	0.26373053	0.339	0.274	4.89E-05	SMC2
LTBP2	4.58E-08	0.8190332	0.565	0.474	9.16E-05	SMC2
PLS3	6.26E-08	0.66197947	0.516	0.56	0.00012513	SMC2
ELN	8.78E-08	0.95253535	0.475	0.307	0.00017564	SMC2
ERRFI1	9.39E-08	0.5804683	0.444	0.456	0.00018775	SMC2
LMCD1	9.90E-08	0.63129383	0.592	0.554	0.00019806	SMC2
PRKG1	1.02E-07	1.27814594	0.424	0.348	0.00020392	SMC2
CSRP1	1.07E-07	0.55939414	0.524	0.536	0.0002133	SMC2
ISYNA1	2.10E-07	0.32348595	0.465	0.477	0.00042023	SMC2
OGN	3.37E-07	0.86088641	0.524	0.415	0.00067331	SMC2

TIMP1	5.16E-07	0.4004709	0.896	0.795	0.0010312	SMC2
COL4A2	6.22E-07	0.64093254	0.611	0.528	0.00124354	SMC2
ZNF385D	1.87E-06	0.35088001	0.552	0.473	0.0037425	SMC2
CCDC3	2.42E-06	0.36818289	0.468	0.443	0.00484127	SMC2
CNKSR3	3.53E-06	0.25277001	0.441	0.385	0.00705868	SMC2
CLU	3.73E-06	0.73414589	0.587	0.519	0.00746583	SMC2
HSPA1B	6.36E-06	0.90799553	0.758	0.661	0.01271927	SMC2
MYH10	1.58E-05	1.79245973	0.486	0.412	0.03155958	SMC2
MMP2	1.74E-05	0.43113516	0.591	0.481	0.03479653	SMC2
BGN	2.01E-05	0.9144979	0.582	0.546	0.0402578	SMC2
ACTA2	0	3.97935575	0.991	0.469	0	SMC1
TAGLN	0	3.80081653	0.998	0.517	0	SMC1
TPM2	0	3.57312844	0.995	0.478	0	SMC1
MYL9	0	3.47752853	0.994	0.446	0	SMC1
DSTN	0	2.81503323	0.994	0.682	0	SMC1
CALD1	0	2.78947068	0.998	0.56	0	SMC1
ADIRF	0	2.73392071	0.988	0.546	0	SMC1
C11orf96	0	2.68810489	0.962	0.472	0	SMC1
SOD3	0	2.68492409	0.979	0.461	0	SMC1
MYH11	0	2.5613465	0.957	0.344	0	SMC1
IGFBP7	0	2.518671	0.999	0.751	0	SMC1
TPM1	0	2.51185188	0.982	0.546	0	SMC1
PPP1R14A	0	2.47385814	0.932	0.408	0	SMC1
RGS5	0	2.3796573	0.949	0.42	0	SMC1
FRZB	0	2.30493274	0.958	0.45	0	SMC1
IGFBP2	0	2.18847844	0.942	0.542	0	SMC1
MFGE8	0	2.17804472	0.957	0.463	0	SMC1
MFAP4	0	2.15653708	0.842	0.341	0	SMC1
IGFBP5	0	2.12272584	0.943	0.536	0	SMC1
LMOD1	0	2.09954312	0.925	0.28	0	SMC1
ID4	0	2.05999235	0.95	0.442	0	SMC1
CSRP1	0	1.92006578	0.91	0.509	0	SMC1
CRYAB	0	1.89668735	0.932	0.477	0	SMC1
PLN	0	1.89321031	0.897	0.302	0	SMC1
SELENOM	0	1.89302934	0.977	0.629	0	SMC1
CYR61	0	1.89071726	0.975	0.542	0	SMC1
MGP	0	1.87330081	0.996	0.714	0	SMC1
NOV	0	1.85294465	0.9	0.467	0	SMC1
BGN	0	1.84072808	0.981	0.518	0	SMC1
NEXN	0	1.83931236	0.836	0.393	0	SMC1
CSRP2	0	1.83083382	0.833	0.387	0	SMC1
CD151	0	1.82452936	0.944	0.585	0	SMC1
RERGL	0	1.78342727	0.824	0.325	0	SMC1
FILIP1L	0	1.76849393	0.905	0.531	0	SMC1
CPE	0	1.74473342	0.962	0.495	0	SMC1
CLU	0	1.74351843	0.908	0.496	0	SMC1
FHL1	0	1.73345924	0.912	0.476	0	SMC1
MYH10	0	1.71424269	0.822	0.387	0	SMC1
CAVIN3	0	1.70572145	0.946	0.57	0	SMC1
MAP1B	0	1.70372995	0.937	0.442	0	SMC1
A2M	0	1.70312983	0.973	0.633	0	SMC1
CTGF	0	1.69377051	0.977	0.561	0	SMC1
FXYD1	0	1.66454798	0.867	0.447	0	SMC1

RCAN2	0	1.6606573	0.932	0.538	0	SMC1
OGN	0	1.6563387	0.864	0.389	0	SMC1
RAMP1	0	1.65283505	0.815	0.394	0	SMC1
C12orf75	0	1.64847133	0.918	0.55	0	SMC1
C2orf40	0	1.64842439	0.932	0.565	0	SMC1
TINAGL1	0	1.64500625	0.943	0.475	0	SMC1
COL14A1	0	1.64439734	0.933	0.444	0	SMC1
GUCY1A1	0	1.64114195	0.861	0.445	0	SMC1
ITGA8	0	1.64097	0.859	0.374	0	SMC1
PLS3	0	1.6385685	0.896	0.535	0	SMC1
PDLIM3	0	1.59380818	0.874	0.445	0	SMC1
LHFPL6	0	1.58790043	0.914	0.438	0	SMC1
SMOC2	0	1.58254467	0.877	0.404	0	SMC1
NR2F2	0	1.57046266	0.966	0.588	0	SMC1
HCFC1R1	0	1.51838455	0.925	0.537	0	SMC1
NUPR1	0	1.5131	0.914	0.457	0	SMC1
SERPING1	0	1.51040968	0.965	0.536	0	SMC1
TGFB1I1	0	1.50572794	0.847	0.4	0	SMC1
EDIL3	0	1.4542775	0.877	0.502	0	SMC1
RARRES2	0	1.42246728	0.906	0.496	0	SMC1
LTBP1	0	1.41331071	0.854	0.352	0	SMC1
NNMT	0	1.40014447	0.964	0.583	0	SMC1
SLC25A4	0	1.39618424	0.789	0.456	0	SMC1
GEM	0	1.38427348	0.909	0.569	0	SMC1
AEBP1	0	1.38169101	0.923	0.496	0	SMC1
MYLK	0	1.37264232	0.861	0.404	0	SMC1
PALLD	0	1.36784929	0.876	0.434	0	SMC1
DKK3	0	1.35068876	0.862	0.434	0	SMC1
NET1	0	1.34560977	0.84	0.414	0	SMC1
PDE5A	0	1.34004211	0.801	0.385	0	SMC1
CAV1	0	1.3360561	0.953	0.597	0	SMC1
CAV2	0	1.31066549	0.82	0.453	0	SMC1
IFITM3	0	1.30922518	0.994	0.718	0	SMC1
SPARCL1	0	1.29314203	0.987	0.591	0	SMC1
CCDC3	0	1.28585365	0.866	0.415	0	SMC1
PLAC9	0	1.28112019	0.944	0.503	0	SMC1
S100A6	0	1.28085296	0.999	0.932	0	SMC1
CAVIN1	0	1.27422618	0.908	0.523	0	SMC1
MCAM	0	1.27114291	0.762	0.463	0	SMC1
RRAD	0	1.2500175	0.809	0.493	0	SMC1
SSPN	0	1.24137159	0.835	0.459	0	SMC1
FBLIM1	0	1.22393803	0.81	0.404	0	SMC1
VCAN	0	1.2071915	0.95	0.504	0	SMC1
GAS6	0	1.20072013	0.876	0.605	0	SMC1
EGR1	0	1.18713647	0.938	0.566	0	SMC1
C9orf3	0	1.18120828	0.776	0.472	0	SMC1
FMO2	0	1.18120183	0.846	0.438	0	SMC1
CRIP2	0	1.1650625	0.939	0.594	0	SMC1
COX7A1	0	1.15428502	0.834	0.479	0	SMC1
MT1E	0	1.15304069	0.869	0.564	0	SMC1
ACTC1	0	1.14856087	0.671	0.288	0	SMC1
ID3	0	1.14591169	0.941	0.622	0	SMC1
SPINT2	0	1.13480454	0.799	0.564	0	SMC1

LGALS1	0	1.12272616	0.99	0.824	0	SMC1
SORBS2	0	1.12098329	0.726	0.397	0	SMC1
PAWR	0	1.11964362	0.831	0.472	0	SMC1
NDUFA4L2	0	1.11083619	0.805	0.42	0	SMC1
PMEPA1	0	1.11057601	0.904	0.574	0	SMC1
CNN1	0	1.10901917	0.68	0.239	0	SMC1
S100A4	0	1.10629314	0.993	0.887	0	SMC1
MSRB3	0	1.10225124	0.757	0.387	0	SMC1
MT1M	0	1.09703959	0.79	0.34	0	SMC1
NRGN	0	1.08759419	0.847	0.44	0	SMC1
EFEMP1	0	1.08544903	0.875	0.43	0	SMC1
TSC22D1	0	1.06525044	0.886	0.618	0	SMC1
C1R	0	1.06492348	0.952	0.475	0	SMC1
EFHD1	0	1.06136926	0.67	0.31	0	SMC1
ASPN	0	1.0399634	0.859	0.352	0	SMC1
COL8A1	0	1.03886355	0.819	0.359	0	SMC1
SEPT4	0	1.02379099	0.912	0.482	0	SMC1
SNCG	0	0.99756141	0.865	0.571	0	SMC1
PRRX1	0	0.9915507	0.805	0.364	0	SMC1
PPP1R12B	0	0.98927628	0.748	0.366	0	SMC1
ISYNA1	0	0.97225661	0.776	0.456	0	SMC1
PRKG1	0	0.96169951	0.727	0.325	0	SMC1
ANGPT1	0	0.95803189	0.751	0.411	0	SMC1
PDGFA	0	0.95305259	0.84	0.516	0	SMC1
ANGPT2	0	0.95193734	0.83	0.516	0	SMC1
CARMN	0	0.94730054	0.703	0.316	0	SMC1
SGCA	0	0.93081355	0.769	0.419	0	SMC1
HES4	0	0.92900565	0.832	0.504	0	SMC1
SVIL	0	0.9280733	0.804	0.471	0	SMC1
CNN3	0	0.92338575	0.928	0.542	0	SMC1
PRELP	0	0.92310826	0.742	0.35	0	SMC1
KCNMB1	0	0.92167284	0.738	0.374	0	SMC1
FN1	0	0.90838007	0.934	0.552	0	SMC1
WTIP	0	0.89909087	0.71	0.279	0	SMC1
NOTCH3	0	0.87212507	0.784	0.36	0	SMC1
CD9	0	0.85446198	0.963	0.646	0	SMC1
LBH	0	0.85100491	0.907	0.639	0	SMC1
VASN	0	0.84894351	0.794	0.394	0	SMC1
ANTXR1	0	0.83995558	0.757	0.384	0	SMC1
SYNPO2	0	0.81350292	0.681	0.343	0	SMC1
FILIP1	0	0.79874229	0.707	0.408	0	SMC1
GSTM3	0	0.79861786	0.896	0.6	0	SMC1
TNFRSF12A	0	0.78597779	0.848	0.545	0	SMC1
GUCY1B1	0	0.7728519	0.742	0.391	0	SMC1
SPARC	0	0.76804798	0.94	0.611	0	SMC1
C1S	0	0.76721079	0.873	0.402	0	SMC1
CP	0	0.76422405	0.87	0.372	0	SMC1
PCDH7	0	0.75733751	0.699	0.311	0	SMC1
NBL1	0	0.7572617	0.85	0.559	0	SMC1
C1QTNF1	0	0.75643005	0.791	0.336	0	SMC1
PCOLCE	0	0.75059889	0.927	0.501	0	SMC1
RASL11A	0	0.74871828	0.771	0.469	0	SMC1
EFEMP2	0	0.74730297	0.81	0.427	0	SMC1

HTRA1	0	0.74302929	0.82	0.572	0	SMC1
ADAMTS1	0	0.72613923	0.789	0.465	0	SMC1
TNFRSF11B	0	0.72049616	0.828	0.444	0	SMC1
LTBP4	0	0.70838457	0.744	0.436	0	SMC1
GPRC5C	0	0.70778901	0.814	0.379	0	SMC1
PHLDA3	0	0.70083344	0.806	0.535	0	SMC1
PTP4A3	0	0.69803105	0.737	0.472	0	SMC1
THBS2	0	0.69367449	0.869	0.438	0	SMC1
PRRX2	0	0.67505913	0.85	0.357	0	SMC1
PDGFRB	0	0.67047349	0.779	0.366	0	SMC1
GSN	0	0.65555719	0.952	0.629	0	SMC1
LGALS3BP	0	0.64985519	0.873	0.542	0	SMC1
BCAM	0	0.64486638	0.749	0.45	0	SMC1
COL6A2	0	0.63323728	0.874	0.554	0	SMC1
TBX2-AS1	0	0.6300909	0.743	0.167	0	SMC1
GPX3	0	0.59182487	0.819	0.427	0	SMC1
EDNRA	0	0.58871916	0.762	0.359	0	SMC1
MT1A	0	0.58520757	0.751	0.34	0	SMC1
SGCE	0	0.57469913	0.768	0.384	0	SMC1
TBX2	0	0.56391793	0.779	0.315	0	SMC1
PGF	0	0.55871402	0.829	0.494	0	SMC1
PDLIM1	0	0.55566775	0.854	0.603	0	SMC1
SUGCT	0	0.55519549	0.736	0.434	0	SMC1
CYBRD1	0	0.55168492	0.749	0.43	0	SMC1
TMEM98	0	0.55020002	0.702	0.343	0	SMC1
AGT	0	0.54846596	0.844	0.376	0	SMC1
HES1	0	0.54143845	0.853	0.502	0	SMC1
LTBP2	0	0.53303212	0.836	0.453	0	SMC1
FGF7	0	0.51875125	0.855	0.512	0	SMC1
PPIC	0	0.51103555	0.835	0.531	0	SMC1
PHLDA2	0	0.50126937	0.86	0.608	0	SMC1
HSPB8	0	0.50097973	0.666	0.321	0	SMC1
CDO1	0	0.50006056	0.761	0.328	0	SMC1
AKR1C3	0	0.49922537	0.815	0.518	0	SMC1
OMD	0	0.4914128	0.699	0.3	0	SMC1
LMO3	0	0.49103684	0.665	0.272	0	SMC1
DPT	0	0.46850829	0.768	0.477	0	SMC1
GJA4	0	0.4541723	0.758	0.367	0	SMC1
NTRK2	0	0.43950898	0.809	0.401	0	SMC1
MAP3K7CL	0	0.43436578	0.719	0.381	0	SMC1
LGALSL	0	0.42942058	0.821	0.447	0	SMC1
S100A13	0	0.42624064	0.876	0.521	0	SMC1
SERPINI1	0	0.42413116	0.734	0.474	0	SMC1
C1QTNF7	0	0.423458	0.698	0.266	0	SMC1
UACA	0	0.41751391	0.86	0.565	0	SMC1
NUDT4	0	0.41237673	0.792	0.474	0	SMC1
HAND2	0	0.41027236	0.671	0.276	0	SMC1
TIMP1	0	0.40980054	0.995	0.786	0	SMC1
PTN	0	0.40570985	0.842	0.354	0	SMC1
ARHGAP29	0	0.40490836	0.806	0.56	0	SMC1
KRT8	0	0.4023421	0.781	0.401	0	SMC1
CST6	0	0.39006099	0.658	0.315	0	SMC1
HMCN1	0	0.3667345	0.688	0.272	0	SMC1

SOST	0	0.36018691	0.687	0.156	0	SMC1
RGS16	0	0.35667576	0.894	0.629	0	SMC1
CDH11	0	0.34615073	0.813	0.482	0	SMC1
CXCL12	0	0.34266142	0.858	0.629	0	SMC1
NT5DC2	0	0.34016049	0.712	0.435	0	SMC1
NPTX2	0	0.33854842	0.637	0.314	0	SMC1
PDLIM4	0	0.33717304	0.777	0.481	0	SMC1
LOXL1	0	0.33102906	0.744	0.394	0	SMC1
TM4SF1	0	0.32973758	0.934	0.599	0	SMC1
ELN	0	0.32898574	0.734	0.285	0	SMC1
TFPI	0	0.30318883	0.904	0.544	0	SMC1
FOXS1	0	0.29414491	0.816	0.343	0	SMC1
COX4I2	0	0.28646391	0.642	0.239	0	SMC1
BACE2	0	0.2857008	0.796	0.434	0	SMC1
PDGFD	0	0.28256961	0.723	0.384	0	SMC1
CCND1	0	0.27969179	0.853	0.594	0	SMC1
DLX5	0	0.27667183	0.747	0.284	0	SMC1
CCDC102B	0	0.2656776	0.879	0.468	0	SMC1
CDH6	0	0.25953087	0.656	0.187	0	SMC1
RCN3	0	0.25816023	0.726	0.402	0	SMC1
LINC01615	0	0.25639751	0.847	0.433	0	SMC1
PDGFRA	0	0.25564372	0.759	0.323	0	SMC1
KCNE4	1.16E-307	0.52144203	0.695	0.44	2.32E-304	SMC1
PMP22	1.86E-307	0.33085856	0.875	0.545	3.72E-304	SMC1
IGFBP6	3.40E-305	0.72021976	0.734	0.445	6.81E-302	SMC1
CD59	3.21E-295	0.46100293	0.859	0.563	6.43E-292	SMC1
FOXC1	1.33E-292	0.85652733	0.72	0.463	2.66E-289	SMC1
NQO1	1.62E-290	0.40308464	0.725	0.5	3.23E-287	SMC1
FOXC2	3.85E-288	0.67406255	0.65	0.276	7.70E-285	SMC1
CAMK2N1	4.53E-288	0.28055686	0.69	0.433	9.06E-285	SMC1
CASQ2	4.82E-288	0.43906138	0.608	0.173	9.64E-285	SMC1
RND3	3.48E-286	0.34163148	0.753	0.451	6.97E-283	SMC1
COL18A1	4.51E-283	0.88801142	0.737	0.513	9.03E-280	SMC1
RBP1	3.70E-282	0.5433605	0.685	0.403	7.41E-279	SMC1
JUN	1.67E-279	1.00629646	0.966	0.804	3.34E-276	SMC1
CDKN1C	5.25E-274	0.4556611	0.824	0.588	1.05E-270	SMC1
SLC7A2	1.70E-271	0.46376575	0.661	0.302	3.41E-268	SMC1
TMEM47	1.87E-269	0.78035094	0.669	0.361	3.74E-266	SMC1
CX3CL1	8.10E-263	0.31229434	0.633	0.269	1.62E-259	SMC1
ALCAM	8.19E-263	0.6309904	0.707	0.422	1.64E-259	SMC1
LMCD1	1.53E-262	0.84940467	0.728	0.543	3.06E-259	SMC1
CRIM1	1.90E-262	0.75449746	0.687	0.421	3.80E-259	SMC1
ATF3	3.33E-261	0.72250331	0.89	0.615	6.67E-258	SMC1
ACTB	1.37E-254	0.37065609	1	0.966	2.74E-251	SMC1
IER5L	7.17E-251	0.33006796	0.872	0.651	1.43E-247	SMC1
SDC2	2.61E-245	0.56876067	0.695	0.404	5.23E-242	SMC1
MT2A	3.49E-245	0.69940104	0.966	0.779	6.97E-242	SMC1
MXRA8	3.47E-239	0.60477492	0.675	0.423	6.94E-236	SMC1
KLHL23	2.11E-237	0.33599322	0.632	0.394	4.23E-234	SMC1
HGF	4.41E-237	0.29978022	0.714	0.438	8.82E-234	SMC1
DNAJB4	2.54E-234	0.43325594	0.75	0.526	5.09E-231	SMC1
RGS4	6.68E-223	0.35300552	0.629	0.312	1.34E-219	SMC1
PDLIM7	2.84E-222	0.92508027	0.674	0.452	5.67E-219	SMC1

LIMCH1	7.21E-222	0.840684	0.664	0.39	1.44E-218	SMC1
DEPP1	8.43E-221	0.70295346	0.655	0.358	1.69E-217	SMC1
SLIT3	1.43E-218	0.35601615	0.621	0.287	2.86E-215	SMC1
SULF1	4.98E-217	0.49932967	0.675	0.347	9.96E-214	SMC1
FHL5	3.02E-212	0.64306	0.594	0.203	6.04E-209	SMC1
ADH1B	1.15E-210	1.41865497	0.628	0.286	2.30E-207	SMC1
GMDS	1.50E-209	0.82703008	0.678	0.49	3.00E-206	SMC1
WISP2	1.91E-206	0.74848939	0.643	0.361	3.82E-203	SMC1
P4HA2	9.44E-201	0.27025402	0.671	0.45	1.89E-197	SMC1
AKAP12	3.88E-198	0.40283741	0.661	0.41	7.76E-195	SMC1
MBNL1-AS1	3.86E-197	0.67610032	0.628	0.392	7.71E-194	SMC1
COL6A1	8.61E-197	0.40122534	0.704	0.463	1.72E-193	SMC1
PLPP1	2.11E-194	0.37481619	0.726	0.549	4.23E-191	SMC1
COL4A2	8.94E-179	0.54350323	0.72	0.518	1.79E-175	SMC1
ITGA1	9.74E-178	0.40483955	0.671	0.474	1.95E-174	SMC1
COPZ2	1.52E-176	0.57602274	0.662	0.428	3.03E-173	SMC1
EPB41L2	1.56E-173	0.44290827	0.731	0.52	3.12E-170	SMC1
PCDH10	4.97E-172	0.46267032	0.62	0.387	9.94E-169	SMC1
CRISPLD1	2.37E-170	0.57502369	0.64	0.45	4.74E-167	SMC1
MGLL	9.89E-167	0.30617112	0.734	0.494	1.98E-163	SMC1
SLC40A1	3.05E-162	0.52321893	0.69	0.501	6.09E-159	SMC1
SYNPO	1.42E-158	0.54848117	0.641	0.429	2.85E-155	SMC1
FBXO32	7.68E-155	0.55171999	0.662	0.501	1.54E-151	SMC1
EMILIN1	7.22E-144	0.53153678	0.613	0.378	1.44E-140	SMC1
GADD45A	1.93E-142	0.58399198	0.732	0.581	3.86E-139	SMC1
NFIB	1.84E-141	0.59735002	0.667	0.405	3.69E-138	SMC1
ACKR3	2.54E-141	0.28162946	0.607	0.363	5.07E-138	SMC1
ENAH	3.05E-141	0.69765062	0.642	0.429	6.09E-138	SMC1
RGS7BP	5.59E-140	0.27519224	0.313	0.28	1.12E-136	SMC1
ERLEC1	2.68E-139	0.33103287	0.814	0.604	5.36E-136	SMC1
MYO1B	2.35E-138	0.25035089	0.691	0.5	4.69E-135	SMC1
TIMP3	8.27E-137	0.49362522	0.679	0.506	1.65E-133	SMC1
FAM114A1	1.40E-134	0.40159248	0.695	0.496	2.80E-131	SMC1
TWIST1	2.54E-133	0.52189445	0.618	0.383	5.07E-130	SMC1
ESAM	2.94E-133	0.31636728	0.663	0.479	5.87E-130	SMC1
TGM2	4.75E-122	0.43686387	0.662	0.419	9.50E-119	SMC1
AQP1	2.21E-115	0.29820943	0.699	0.528	4.43E-112	SMC1
ACTG2	1.10E-111	0.98312711	0.557	0.234	2.21E-108	SMC1
LMO7	1.15E-111	0.5614754	0.593	0.399	2.30E-108	SMC1
FEZ1	6.69E-104	0.56052667	0.604	0.408	1.34E-100	SMC1
RHOB	3.34E-101	0.43392542	0.726	0.564	6.68E-98	SMC1
LRRC17	1.43E-100	0.37801463	0.602	0.373	2.86E-97	SMC1
PDGFRL	4.78E-100	0.3159812	0.607	0.404	9.57E-97	SMC1
FBLN5	9.86E-92	0.66952981	0.564	0.302	1.97E-88	SMC1
WFDC1	6.39E-89	0.52504496	0.537	0.222	1.28E-85	SMC1
ITGBL1	4.54E-88	0.6668207	0.575	0.347	9.07E-85	SMC1
WWTR1	4.39E-87	0.30840163	0.649	0.442	8.78E-84	SMC1
FAM13C	1.06E-84	0.42836757	0.586	0.393	2.13E-81	SMC1
MRGPRF	2.72E-84	0.6440102	0.544	0.152	5.44E-81	SMC1
RAPGEF5	3.28E-84	0.2552319	0.619	0.44	6.55E-81	SMC1
ECM2	1.92E-77	0.50691208	0.555	0.311	3.84E-74	SMC1
LDOC1	1.24E-75	0.63288099	0.563	0.386	2.47E-72	SMC1
PTGIS	2.12E-74	0.43790805	0.557	0.317	4.24E-71	SMC1

MT1X	1.01E-68	0.59480296	0.64	0.536	2.03E-65	SMC1
PRDM6	1.61E-62	0.53258976	0.52	0.197	3.23E-59	SMC1
FMOD	2.93E-60	0.25153188	0.349	0.238	5.87E-57	SMC1
TUBB6	5.60E-60	0.31442422	0.656	0.477	1.12E-56	SMC1
ZNF503	7.93E-60	0.5203313	0.609	0.477	1.59E-56	SMC1
FSTL1	8.49E-60	0.31714027	0.6	0.427	1.70E-56	SMC1
GADD45G	9.05E-59	0.26533276	0.424	0.507	1.81E-55	SMC1
PLK2	8.81E-51	0.32188729	0.633	0.513	1.76E-47	SMC1
INHBA	1.39E-46	0.41394164	0.557	0.347	2.77E-43	SMC1
AXL	2.20E-45	0.3852425	0.554	0.335	4.40E-42	SMC1
MAP9	1.82E-41	0.4717991	0.545	0.365	3.64E-38	SMC1
PFN2	6.64E-41	0.57966057	0.538	0.379	1.33E-37	SMC1
KCNAB1	5.20E-40	0.34813472	0.372	0.28	1.04E-36	SMC1
FBXL22	2.71E-39	0.26332875	0.388	0.412	5.42E-36	SMC1
CDH13	2.40E-38	0.5002807	0.547	0.339	4.80E-35	SMC1
TCEAL2	5.15E-38	0.74795798	0.518	0.366	1.03E-34	SMC1
KCNK15	4.81E-34	0.2798611	0.498	0.307	9.62E-31	SMC1
GADD45B	2.53E-32	0.25403939	0.927	0.735	5.07E-29	SMC1
CTSF	4.63E-32	0.47782364	0.565	0.444	9.26E-29	SMC1
SYNM	5.09E-32	0.57500157	0.525	0.39	1.02E-28	SMC1
PHGDH	1.67E-29	0.4251428	0.499	0.302	3.34E-26	SMC1
ENPP1	1.83E-27	0.43560926	0.538	0.438	3.65E-24	SMC1
TSPAN2	2.76E-27	0.50006534	0.521	0.395	5.53E-24	SMC1
RGS3	1.69E-22	0.35076636	0.581	0.509	3.38E-19	SMC1
INMT	9.26E-21	0.39734253	0.491	0.285	1.85E-17	SMC1
FAT1	7.29E-20	0.26184477	0.512	0.342	1.46E-16	SMC1
IL33	2.00E-19	0.27704187	0.542	0.412	3.99E-16	SMC1
ANGPTL1	9.95E-19	0.27040826	0.379	0.193	1.99E-15	SMC1
SCRG1	1.29E-17	0.58088771	0.496	0.383	2.59E-14	SMC1
ERRFI1	5.09E-17	0.29825569	0.532	0.45	1.02E-13	SMC1
DES	2.32E-16	0.39472173	0.369	0.119	4.63E-13	SMC1
HSPB6	7.81E-14	0.54388878	0.482	0.27	1.56E-10	SMC1
KCNMA1	5.70E-11	0.27994314	0.523	0.42	1.14E-07	SMC1
RASL12	5.17E-09	0.49091372	0.477	0.256	1.03E-05	SMC1
RERG	7.16E-09	0.50520104	0.485	0.257	1.43E-05	SMC1
NOV	2.14E-225	3.41186479	0.992	0.491	4.28E-222	SMC4
PPP1R14A	9.02E-215	2.98204646	0.984	0.438	1.80E-211	SMC4
ACTA2	4.11E-214	3.3463447	1	0.499	8.22E-211	SMC4
OGN	4.12E-214	2.9450976	0.987	0.415	8.25E-211	SMC4
FHL1	1.57E-212	2.69282707	0.992	0.5	3.14E-209	SMC4
TPM2	2.12E-210	3.04920396	1	0.507	4.24E-207	SMC4
TPM1	3.53E-209	2.80102136	0.997	0.571	7.06E-206	SMC4
TAGLN	6.68E-209	3.1489968	1	0.545	1.34E-205	SMC4
MGP	2.64E-208	3.4108604	1	0.73	5.28E-205	SMC4
EFEMP1	5.81E-208	2.80178239	0.984	0.455	1.16E-204	SMC4
IGFBP2	8.26E-204	2.91328045	0.997	0.565	1.65E-200	SMC4
FN1	5.20E-203	3.06042187	1	0.574	1.04E-199	SMC4
IGFBP7	2.78E-202	2.63722793	1	0.766	5.57E-199	SMC4
TNFRSF11B	3.19E-201	3.15767837	0.963	0.465	6.38E-198	SMC4
ASPN	3.42E-201	2.64456592	0.96	0.381	6.83E-198	SMC4
MYH10	1.12E-198	2.42318427	0.976	0.411	2.24E-195	SMC4
MYL9	6.20E-198	2.71357887	0.997	0.478	1.24E-194	SMC4
DSTN	1.27E-197	2.30252496	1	0.7	2.54E-194	SMC4

BGN	6.45E-197	2.62629761	1	0.544	1.29E-193	SMC4
ITGBL1	3.63E-195	2.03433499	0.971	0.358	7.26E-192	SMC4
COL8A1	2.22E-194	2.36733349	0.965	0.384	4.44E-191	SMC4
C5orf46	2.68E-194	0.88423117	0.867	0.333	5.37E-191	SMC4
CALD1	1.43E-193	2.6014025	1	0.585	2.85E-190	SMC4
CTGF	7.15E-193	2.96351701	1	0.584	1.43E-189	SMC4
AEBP1	7.32E-193	2.5906282	0.995	0.52	1.46E-189	SMC4
VCAN	1.41E-191	2.82822112	0.992	0.53	2.83E-188	SMC4
NEXN	1.65E-188	2.31454421	0.957	0.418	3.31E-185	SMC4
LTBP2	2.19E-185	1.91267813	0.976	0.474	4.37E-182	SMC4
THBS2	1.89E-184	1.90941295	0.973	0.462	3.78E-181	SMC4
MYH11	6.86E-182	2.06037944	0.979	0.379	1.37E-178	SMC4
PDLIM3	1.06E-180	1.99296503	0.976	0.469	2.13E-177	SMC4
COL1A2	2.30E-180	2.29143735	0.995	0.525	4.60E-177	SMC4
LTBP1	2.66E-178	2.07100209	0.963	0.38	5.32E-175	SMC4
MFGE8	1.78E-175	2.15308548	0.973	0.491	3.57E-172	SMC4
SGCA	1.36E-174	1.26359111	0.941	0.438	2.71E-171	SMC4
SFRP4	1.54E-174	1.31823776	0.939	0.406	3.08E-171	SMC4
COL14A1	9.49E-174	2.08612701	0.987	0.472	1.90E-170	SMC4
C2orf40	1.30E-170	2.01776834	0.965	0.586	2.61E-167	SMC4
MFAP4	6.17E-169	2.30060072	0.933	0.369	1.23E-165	SMC4
PRELP	2.02E-168	1.8898158	0.947	0.371	4.04E-165	SMC4
CYR61	4.22E-166	2.1118533	0.997	0.566	8.44E-163	SMC4
CD151	6.54E-166	1.85608082	0.979	0.605	1.31E-162	SMC4
ITGA8	2.65E-165	1.76243378	0.944	0.401	5.29E-162	SMC4
CRYAB	2.80E-164	2.26657245	0.957	0.503	5.61E-161	SMC4
LMOD1	3.36E-164	1.54817717	0.952	0.317	6.71E-161	SMC4
SUGCT	3.38E-163	1.7332788	0.925	0.45	6.76E-160	SMC4
PLAC9	2.02E-162	1.90373266	0.973	0.529	4.03E-159	SMC4
SMOC2	3.18E-160	1.48703732	0.952	0.43	6.37E-157	SMC4
COL5A1	1.41E-159	0.6311629	0.877	0.337	2.82E-156	SMC4
RGS5	1.13E-158	1.67536404	0.955	0.45	2.25E-155	SMC4
DKK3	4.53E-158	1.74546929	0.949	0.458	9.07E-155	SMC4
FMO2	4.63E-157	1.7027762	0.933	0.461	9.25E-154	SMC4
EDIL3	1.46E-156	1.81682175	0.955	0.523	2.92E-153	SMC4
PLS3	1.98E-156	1.56729204	0.971	0.555	3.97E-153	SMC4
LINC01615	3.01E-154	0.7860754	0.88	0.457	6.02E-151	SMC4
COL1A1	4.10E-154	1.37797925	0.949	0.464	8.19E-151	SMC4
IGFBP6	2.16E-152	1.86699774	0.955	0.46	4.32E-149	SMC4
GAS6	4.20E-152	1.89163944	0.965	0.62	8.39E-149	SMC4
SCG2	6.07E-152	0.88910255	0.827	0.298	1.21E-148	SMC4
FBLN5	1.20E-151	1.49362021	0.912	0.314	2.40E-148	SMC4
C1R	1.38E-151	1.70657809	0.987	0.502	2.76E-148	SMC4
MMP23B	1.07E-150	1.40603061	0.891	0.442	2.13E-147	SMC4
HTRA1	1.30E-149	1.57074762	0.981	0.585	2.60E-146	SMC4
SPARC	1.41E-149	1.830546	0.981	0.629	2.83E-146	SMC4
FNDC1	6.95E-149	0.28469439	0.829	0.339	1.39E-145	SMC4
LGALS1	2.89E-147	1.54379721	1	0.834	5.78E-144	SMC4
CSRP1	5.83E-147	1.75206521	0.944	0.532	1.17E-143	SMC4
COL6A2	1.48E-146	1.56237953	0.979	0.571	2.95E-143	SMC4
LUM	2.33E-146	1.32293002	0.952	0.586	4.66E-143	SMC4
PRSS23	3.31E-146	1.73114278	0.949	0.562	6.61E-143	SMC4
CNN3	7.50E-146	1.386798	0.981	0.564	1.50E-142	SMC4

CDH11	7.54E-145	1.06687368	0.931	0.5	1.51E-141	SMC4
FRZB	1.14E-144	1.53260937	0.952	0.479	2.28E-141	SMC4
CCDC80	1.20E-144	1.48473133	0.968	0.558	2.39E-141	SMC4
FBLIM1	4.77E-144	1.47900415	0.915	0.427	9.54E-141	SMC4
TIMP1	2.38E-142	1.72899616	1	0.798	4.76E-139	SMC4
COL4A2	4.62E-141	1.4381925	0.957	0.528	9.25E-138	SMC4
PCDH10	1.98E-140	0.6430836	0.869	0.398	3.97E-137	SMC4
ADIRF	1.17E-139	1.59483435	0.997	0.571	2.34E-136	SMC4
SULF1	7.42E-139	1.59453203	0.907	0.364	1.48E-135	SMC4
SSPN	1.35E-138	1.4502293	0.925	0.48	2.69E-135	SMC4
PCOLCE	1.28E-137	1.07023065	0.96	0.525	2.56E-134	SMC4
C1S	2.70E-137	1.11477186	0.952	0.429	5.41E-134	SMC4
C1QTNF7	1.14E-136	0.50164806	0.819	0.29	2.28E-133	SMC4
FHL2	2.27E-136	0.86294443	0.901	0.503	4.53E-133	SMC4
COPZ2	2.34E-134	0.97166661	0.928	0.439	4.69E-131	SMC4
MAP1B	8.62E-134	1.4733533	0.939	0.471	1.72E-130	SMC4
OMD	4.80E-133	1.75682347	0.877	0.321	9.60E-130	SMC4
SLC7A2	5.84E-132	0.25695434	0.843	0.321	1.17E-128	SMC4
FAP	1.08E-131	0.55163528	0.891	0.477	2.17E-128	SMC4
MFAP2	4.29E-131	0.56723941	0.848	0.316	8.58E-128	SMC4
CFH	5.38E-131	1.46421459	0.971	0.554	1.08E-127	SMC4
TNFRSF12A	6.09E-131	1.73939911	0.92	0.562	1.22E-127	SMC4
ID4	1.10E-129	1.57150376	0.92	0.472	2.21E-126	SMC4
GAP43	1.55E-129	0.27070628	0.856	0.436	3.10E-126	SMC4
SOD3	2.86E-129	1.45405924	0.968	0.491	5.71E-126	SMC4
RARRES2	3.76E-129	1.38377734	0.933	0.519	7.53E-126	SMC4
SERPINE1	7.42E-129	1.48054512	0.877	0.415	1.48E-125	SMC4
EFEMP2	1.36E-128	1.14484084	0.907	0.448	2.72E-125	SMC4
LGALS3BP	1.37E-128	1.13276919	0.941	0.561	2.73E-125	SMC4
CTSK	3.30E-128	0.56857552	0.923	0.49	6.61E-125	SMC4
S100A6	9.68E-128	1.23562037	1	0.936	1.94E-124	SMC4
COL4A1	1.65E-127	1.42588247	0.949	0.545	3.31E-124	SMC4
INHBA	9.12E-127	1.69166305	0.853	0.357	1.82E-123	SMC4
PTN	5.24E-126	0.63500681	0.872	0.382	1.05E-122	SMC4
CYTL1	7.42E-126	0.74292891	0.845	0.413	1.48E-122	SMC4
FXYD1	9.08E-126	1.58431596	0.901	0.471	1.82E-122	SMC4
NOTCH3	6.56E-125	0.79080911	0.869	0.383	1.31E-121	SMC4
COL6A1	3.92E-124	1.46636594	0.912	0.476	7.84E-121	SMC4
COL3A1	9.87E-124	1.29295103	0.928	0.513	1.97E-120	SMC4
GPX8	1.92E-123	0.87486631	0.883	0.437	3.85E-120	SMC4
COX7A1	2.28E-123	1.26359627	0.936	0.499	4.56E-120	SMC4
HCFC1R1	6.82E-123	1.28976512	0.944	0.559	1.36E-119	SMC4
AGT	3.41E-122	0.38114236	0.88	0.403	6.83E-119	SMC4
CAVIN3	6.44E-122	1.3784547	0.957	0.591	1.29E-118	SMC4
ADH1B	1.46E-120	1.86150888	0.859	0.304	2.91E-117	SMC4
SVIL	2.16E-120	1.24235289	0.925	0.489	4.32E-117	SMC4
PDGFA	5.84E-120	1.12117602	0.909	0.534	1.17E-116	SMC4
TGM2	1.19E-119	0.8011837	0.92	0.431	2.37E-116	SMC4
DPT	1.30E-119	0.38057134	0.872	0.493	2.59E-116	SMC4
PALLD	1.62E-119	1.39425534	0.893	0.459	3.24E-116	SMC4
KRT17	3.03E-118	2.28420604	0.832	0.438	6.06E-115	SMC4
SELENOM	3.67E-118	1.41522694	0.968	0.65	7.34E-115	SMC4
FBN1	1.00E-117	0.87966142	0.909	0.454	2.01E-114	SMC4

CPE	1.82E-117	1.45698949	0.933	0.522	3.65E-114	SMC4
SPINT2	3.07E-116	1.49883523	0.941	0.576	6.14E-113	SMC4
F3	3.50E-116	0.38141503	0.843	0.519	7.00E-113	SMC4
RAMP1	1.04E-115	1.38465981	0.856	0.418	2.09E-112	SMC4
PLN	1.25E-115	1.15638656	0.867	0.337	2.49E-112	SMC4
NNMT	1.33E-115	1.24637331	0.989	0.605	2.66E-112	SMC4
PPIC	1.70E-114	1.0258113	0.936	0.548	3.39E-111	SMC4
RCN3	1.92E-113	0.81461702	0.872	0.42	3.83E-110	SMC4
SERPING1	1.38E-112	1.16705971	0.971	0.561	2.76E-109	SMC4
CAVIN1	1.41E-110	1.28061143	0.925	0.545	2.82E-107	SMC4
ISLR	3.17E-110	1.06451601	0.845	0.346	6.34E-107	SMC4
ELN	3.28E-110	1.2320091	0.837	0.31	6.56E-107	SMC4
SLC25A4	1.89E-109	1.39414791	0.867	0.474	3.79E-106	SMC4
TGFB1I1	1.23E-108	1.33030144	0.867	0.426	2.47E-105	SMC4
PAWR	2.29E-108	1.15639038	0.875	0.493	4.59E-105	SMC4
GEM	3.10E-108	1.56652515	0.885	0.588	6.20E-105	SMC4
SFRP1	5.46E-108	0.28627593	0.763	0.238	1.09E-104	SMC4
CTHRC1	1.93E-107	0.85222797	0.877	0.547	3.87E-104	SMC4
MAP3K7CL	7.12E-107	0.66275756	0.824	0.4	1.42E-103	SMC4
LIMCH1	7.03E-106	1.05903128	0.869	0.405	1.41E-102	SMC4
FSTL1	9.22E-106	1.35174567	0.851	0.435	1.84E-102	SMC4
C12orf75	2.02E-105	1.34224689	0.923	0.571	4.04E-102	SMC4
FOXC1	2.19E-105	1.02252514	0.904	0.477	4.38E-102	SMC4
NDUFA4L2	4.43E-105	1.60051291	0.84	0.442	8.86E-102	SMC4
ANTXR1	4.46E-105	1.34237011	0.837	0.405	8.91E-102	SMC4
LHFPL6	7.32E-105	1.13781996	0.907	0.465	1.46E-101	SMC4
C9orf3	8.70E-105	1.21325246	0.888	0.488	1.74E-101	SMC4
IGFBP5	1.77E-104	0.94663321	0.941	0.559	3.53E-101	SMC4
COL21A1	2.39E-104	1.0663609	0.877	0.48	4.78E-101	SMC4
CRIP2	3.36E-104	1.16845721	0.957	0.614	6.72E-101	SMC4
THY1	3.97E-104	0.55063513	0.893	0.582	7.94E-101	SMC4
A2M	4.18E-104	1.32235114	0.976	0.653	8.36E-101	SMC4
ALDH1A1	8.05E-104	0.3562087	0.84	0.393	1.61E-100	SMC4
LMCD1	1.19E-103	1.31319918	0.888	0.553	2.37E-100	SMC4
NUPR1	2.45E-103	1.16701206	0.912	0.483	4.90E-100	SMC4
TMEM47	5.30E-103	1.29876683	0.84	0.378	1.06E-99	SMC4
GPX3	5.58E-103	0.51668753	0.893	0.449	1.12E-99	SMC4
CP	9.80E-103	0.43451311	0.856	0.4	1.96E-99	SMC4
PPP1R12B	8.29E-102	1.15133574	0.84	0.388	1.66E-98	SMC4
S100A13	2.19E-101	0.93523865	0.957	0.541	4.37E-98	SMC4
MT1M	3.20E-101	0.47767674	0.861	0.366	6.40E-98	SMC4
PDLIM7	1.43E-99	1.35117052	0.84	0.463	2.87E-96	SMC4
LTBP4	2.43E-99	1.2051935	0.869	0.452	4.87E-96	SMC4
GSTM3	4.81E-99	0.49387211	0.912	0.617	9.61E-96	SMC4
FBLN1	4.91E-99	0.93396911	0.811	0.351	9.82E-96	SMC4
AKAP12	5.81E-99	1.29833594	0.835	0.423	1.16E-95	SMC4
TINAGL1	9.54E-99	1.26432244	0.88	0.503	1.91E-95	SMC4
S100A4	2.26E-98	1.09160433	1	0.893	4.51E-95	SMC4
NR2F2	5.88E-98	0.96313843	0.955	0.61	1.18E-94	SMC4
PRRX2	6.37E-98	0.72556016	0.827	0.385	1.27E-94	SMC4
GSN	1.81E-97	1.06602512	0.984	0.647	3.62E-94	SMC4
HAPLN1	2.20E-97	0.39707584	0.795	0.447	4.40E-94	SMC4
C11orf96	3.08E-97	0.98749977	0.931	0.501	6.15E-94	SMC4

PCOLCE2	7.11E-97	1.06030535	0.789	0.255	1.42E-93	SMC4
LOXL1	7.26E-97	0.74429429	0.821	0.414	1.45E-93	SMC4
COL5A2	3.02E-96	0.65225765	0.835	0.458	6.03E-93	SMC4
KDELR3	1.33E-95	0.34089981	0.819	0.419	2.67E-92	SMC4
OLFML3	1.90E-95	0.36322509	0.811	0.37	3.80E-92	SMC4
TFPI	2.82E-95	0.89621248	0.928	0.564	5.64E-92	SMC4
GGT5	8.70E-94	0.52372099	0.867	0.528	1.74E-90	SMC4
BACE2	1.94E-93	0.53564983	0.877	0.455	3.89E-90	SMC4
VCAM1	4.24E-93	0.39076532	0.877	0.535	8.47E-90	SMC4
LMO7	1.42E-92	0.88535722	0.819	0.409	2.84E-89	SMC4
TMEM98	2.26E-92	0.76345346	0.824	0.363	4.51E-89	SMC4
PDGFRA	3.01E-92	0.46944924	0.811	0.348	6.02E-89	SMC4
TM4SF1	8.58E-92	0.78945803	0.957	0.618	1.72E-88	SMC4
FGF7	1.26E-91	0.29876033	0.853	0.532	2.52E-88	SMC4
UACA	2.03E-91	0.98638007	0.909	0.581	4.06E-88	SMC4
FAM114A1	2.88E-91	0.79169539	0.888	0.506	5.75E-88	SMC4
CXCL12	4.17E-91	0.4188683	0.931	0.642	8.33E-88	SMC4
CDH13	1.36E-90	1.10503781	0.816	0.349	2.72E-87	SMC4
POSTN	1.41E-89	1.42115766	0.872	0.547	2.83E-86	SMC4
CTSF	1.59E-89	0.95015582	0.848	0.449	3.19E-86	SMC4
CAV1	1.52E-88	1.04393704	0.944	0.618	3.03E-85	SMC4
SPARCL1	1.87E-88	0.92192959	0.971	0.614	3.73E-85	SMC4
VASN	2.73E-88	0.84836177	0.813	0.417	5.46E-85	SMC4
KCNK15	4.79E-88	0.34273925	0.755	0.316	9.59E-85	SMC4
LOX	8.00E-88	0.45781958	0.771	0.297	1.60E-84	SMC4
PTGIS	9.32E-88	1.24826187	0.8	0.329	1.86E-84	SMC4
MSRB3	1.09E-87	1.15724236	0.832	0.408	2.18E-84	SMC4
PRRX1	6.29E-87	0.91405541	0.845	0.389	1.26E-83	SMC4
KRT16	2.93E-86	0.67169488	0.675	0.185	5.86E-83	SMC4
SGCE	8.79E-86	0.72539535	0.816	0.406	1.76E-82	SMC4
CHPF	1.87E-85	0.75097345	0.805	0.459	3.73E-82	SMC4
KRT7	2.88E-85	1.3394226	0.757	0.353	5.75E-82	SMC4
LAMA4	4.80E-85	0.52121711	0.891	0.572	9.60E-82	SMC4
LRP1	5.04E-85	1.03975964	0.859	0.467	1.01E-81	SMC4
PMP22	1.28E-84	0.78183538	0.955	0.563	2.55E-81	SMC4
CD9	3.71E-84	0.96127437	0.984	0.664	7.42E-81	SMC4
RCAN2	4.38E-84	1.1529025	0.837	0.561	8.76E-81	SMC4
MYLK	4.65E-84	1.16381924	0.819	0.431	9.31E-81	SMC4
FILIP1L	5.52E-84	1.19473702	0.893	0.552	1.10E-80	SMC4
SERPINH1	1.43E-83	0.62811597	0.912	0.572	2.85E-80	SMC4
SNCG	4.72E-83	0.82536263	0.893	0.588	9.43E-80	SMC4
CD200	1.23E-82	0.50187528	0.877	0.55	2.45E-79	SMC4
SORBS2	1.47E-82	0.7068688	0.813	0.415	2.94E-79	SMC4
CAV2	1.59E-81	1.07865328	0.843	0.474	3.17E-78	SMC4
CRIM1	2.87E-81	1.34522619	0.805	0.435	5.74E-78	SMC4
CBR3	8.48E-81	0.25067373	0.816	0.482	1.70E-77	SMC4
PCDH7	1.28E-79	1.08542	0.781	0.333	2.55E-76	SMC4
CLU	2.43E-79	1.06307215	0.917	0.519	4.87E-76	SMC4
PDE5A	5.85E-79	0.97060192	0.808	0.409	1.17E-75	SMC4
FEZ1	6.12E-79	0.73040348	0.808	0.418	1.22E-75	SMC4
NBL1	2.06E-78	0.4923544	0.915	0.575	4.13E-75	SMC4
IFITM3	2.65E-78	0.99051926	1	0.734	5.30E-75	SMC4
C1QTNF1	1.54E-76	0.68998189	0.797	0.362	3.08E-73	SMC4

CNN1	3.22E-76	1.20012296	0.744	0.264	6.45E-73	SMC4
COL15A1	5.49E-76	0.2865779	0.877	0.511	1.10E-72	SMC4
SEPT4	9.99E-76	0.37208522	0.84	0.508	2.00E-72	SMC4
BICC1	1.43E-75	0.40749558	0.76	0.334	2.86E-72	SMC4
ANKRD37	1.98E-73	0.54554907	0.861	0.562	3.96E-70	SMC4
KRT18	2.66E-73	2.05520247	0.792	0.563	5.33E-70	SMC4
SDC2	5.61E-73	0.88405027	0.811	0.42	1.12E-69	SMC4
IL13RA2	5.79E-73	0.26217161	0.683	0.22	1.16E-69	SMC4
PROCR	9.53E-71	0.28802151	0.835	0.455	1.91E-67	SMC4
ITGA1	2.94E-70	1.08321944	0.787	0.484	5.87E-67	SMC4
PLOD2	4.01E-70	0.37183855	0.736	0.281	8.02E-67	SMC4
CST3	1.14E-69	0.70833495	0.997	0.735	2.29E-66	SMC4
PRDX4	5.79E-69	0.7082213	0.928	0.65	1.16E-65	SMC4
KRT8	6.74E-69	0.86331721	0.768	0.423	1.35E-65	SMC4
PRKG1	7.25E-68	0.84460749	0.768	0.348	1.45E-64	SMC4
COL18A1	1.12E-66	0.95697405	0.835	0.525	2.24E-63	SMC4
ANGPT1	1.25E-66	0.90808696	0.776	0.43	2.50E-63	SMC4
FBLN2	3.17E-66	0.43889762	0.765	0.372	6.34E-63	SMC4
SPTBN1	5.05E-66	0.64474947	0.904	0.507	1.01E-62	SMC4
MMP2	5.59E-65	0.66031103	0.8	0.483	1.12E-61	SMC4
AKR1C2	2.18E-64	0.5521996	0.731	0.324	4.36E-61	SMC4
FKBP10	2.47E-64	0.61081167	0.771	0.408	4.94E-61	SMC4
IGFBP3	6.14E-64	1.31434965	0.747	0.38	1.23E-60	SMC4
MT1E	7.63E-64	0.44405971	0.88	0.582	1.53E-60	SMC4
EPB41L2	3.15E-63	0.61685059	0.864	0.531	6.31E-60	SMC4
CD59	7.06E-63	0.77113257	0.909	0.58	1.41E-59	SMC4
IL34	2.22E-62	0.68015408	0.787	0.564	4.43E-59	SMC4
MXRA8	8.89E-62	0.91351895	0.771	0.437	1.78E-58	SMC4
CYBRD1	9.49E-62	0.70975281	0.789	0.448	1.90E-58	SMC4
FGL2	1.24E-61	0.50236413	0.907	0.519	2.48E-58	SMC4
GDF7	1.33E-61	0.4394226	0.675	0.252	2.66E-58	SMC4
S100A16	1.41E-61	0.46455214	0.853	0.516	2.81E-58	SMC4
RGS3	2.15E-61	0.3183979	0.837	0.512	4.29E-58	SMC4
AKR1C3	3.52E-61	0.26649221	0.864	0.534	7.04E-58	SMC4
FMOD	4.32E-61	0.56750451	0.701	0.242	8.64E-58	SMC4
AQP1	1.62E-60	0.8775751	0.835	0.537	3.24E-57	SMC4
ID3	1.77E-60	0.68981121	0.936	0.641	3.55E-57	SMC4
PHLDA2	2.60E-60	0.46120227	0.88	0.622	5.21E-57	SMC4
CCL2	2.81E-60	0.60565025	0.893	0.534	5.61E-57	SMC4
TWIST1	3.07E-60	0.60084863	0.755	0.396	6.13E-57	SMC4
PDLIM4	3.16E-60	0.44596346	0.805	0.498	6.32E-57	SMC4
CRISPLD1	1.05E-59	0.45411707	0.771	0.46	2.09E-56	SMC4
NUCB2	2.33E-59	0.72612108	0.963	0.664	4.67E-56	SMC4
PHLDA3	2.57E-58	0.55977752	0.819	0.55	5.13E-55	SMC4
HAND2	2.17E-57	0.28088424	0.68	0.299	4.34E-54	SMC4
SOST	2.29E-57	0.57815364	0.653	0.186	4.58E-54	SMC4
MGLL	3.06E-57	0.405716	0.861	0.507	6.12E-54	SMC4
HES4	7.24E-57	0.63164766	0.821	0.523	1.45E-53	SMC4
TFPI2	1.15E-56	0.68694588	0.688	0.263	2.31E-53	SMC4
PGF	1.82E-56	0.52923784	0.787	0.514	3.64E-53	SMC4
NPR3	9.09E-56	0.73176843	0.659	0.18	1.82E-52	SMC4
ACTB	9.66E-56	0.58760477	1	0.968	1.93E-52	SMC4
KCNMB1	1.08E-54	0.9346422	0.731	0.395	2.15E-51	SMC4

EGR1	2.01E-54	0.52196595	0.92	0.587	4.01E-51	SMC4
TPH1	5.83E-54	0.71429002	0.677	0.342	1.17E-50	SMC4
HSP90B1	1.98E-53	0.68783449	0.989	0.827	3.96E-50	SMC4
ECM1	9.01E-53	0.36604862	0.765	0.504	1.80E-49	SMC4
PLD3	4.49E-51	0.37391907	0.885	0.497	8.98E-48	SMC4
CPXM2	1.04E-50	0.91933392	0.731	0.426	2.07E-47	SMC4
RERG	3.26E-50	0.44153271	0.691	0.269	6.52E-47	SMC4
CST6	6.10E-50	0.65115786	0.688	0.335	1.22E-46	SMC4
EMILIN1	2.82E-49	0.82831633	0.715	0.39	5.65E-46	SMC4
TNFAIP6	7.30E-49	0.29512518	0.704	0.406	1.46E-45	SMC4
FOXS1	8.20E-49	0.48169317	0.696	0.371	1.64E-45	SMC4
PDLIM1	5.59E-48	0.555807	0.869	0.617	1.12E-44	SMC4
PLPP1	6.56E-48	0.9380604	0.76	0.559	1.31E-44	SMC4
ERLEC1	7.56E-48	0.58047318	0.888	0.615	1.51E-44	SMC4
TMEM204	2.89E-47	0.45577779	0.797	0.552	5.78E-44	SMC4
ENAH	3.44E-47	0.76613583	0.739	0.441	6.88E-44	SMC4
FST	5.14E-46	0.63861201	0.688	0.372	1.03E-42	SMC4
GJA4	1.11E-45	0.44794041	0.704	0.39	2.22E-42	SMC4
LNP1	1.11E-45	0.2951464	0.664	0.341	2.22E-42	SMC4
PMEPA1	4.24E-45	0.53253088	0.835	0.593	8.48E-42	SMC4
ANGPT2	4.09E-44	0.55460647	0.768	0.534	8.18E-41	SMC4
DAAM1	5.71E-44	0.64377354	0.771	0.498	1.14E-40	SMC4
CCDC3	2.58E-43	0.71223323	0.717	0.442	5.16E-40	SMC4
P4HA2	8.13E-43	0.46295273	0.731	0.463	1.63E-39	SMC4
RND3	3.66E-42	0.39902261	0.771	0.468	7.31E-39	SMC4
TUBB6	1.00E-41	0.4818024	0.805	0.486	2.00E-38	SMC4
MCAM	1.03E-41	0.69421501	0.728	0.481	2.07E-38	SMC4
FKBP2	1.44E-41	0.52782401	0.979	0.727	2.89E-38	SMC4
PDGFD	5.32E-41	0.4384431	0.715	0.403	1.06E-37	SMC4
PDGFRB	6.20E-41	0.87806807	0.712	0.39	1.24E-37	SMC4
WISP2	7.08E-41	0.86079212	0.691	0.377	1.42E-37	SMC4
SLC14A1	4.04E-40	0.44260907	0.675	0.396	8.09E-37	SMC4
INMT	7.39E-40	0.86859072	0.675	0.295	1.48E-36	SMC4
F10	2.53E-39	0.36171465	0.64	0.222	5.06E-36	SMC4
IFI6	2.73E-39	0.26242843	0.896	0.591	5.45E-36	SMC4
GADD45A	3.82E-39	0.53253993	0.829	0.589	7.64E-36	SMC4
ENPP1	3.11E-38	0.29188226	0.677	0.442	6.21E-35	SMC4
DPYSL3	3.79E-38	0.53466766	0.715	0.4	7.58E-35	SMC4
ENG	2.24E-37	0.65930717	0.803	0.53	4.47E-34	SMC4
MRC2	4.12E-37	0.55050354	0.696	0.429	8.24E-34	SMC4
UGDH	6.14E-37	0.31102614	0.691	0.421	1.23E-33	SMC4
FAT1	2.34E-36	0.42363129	0.651	0.351	4.68E-33	SMC4
CTSZ	7.57E-34	0.26123088	0.933	0.696	1.51E-30	SMC4
F2R	1.11E-33	0.77866046	0.731	0.54	2.22E-30	SMC4
CD55	3.37E-33	0.31962325	0.931	0.636	6.73E-30	SMC4
TGFB2	9.40E-33	0.44320367	0.632	0.311	1.88E-29	SMC4
SYNPO2	2.25E-32	0.74922252	0.664	0.362	4.50E-29	SMC4
UCHL1	2.31E-32	0.29573822	0.624	0.326	4.62E-29	SMC4
GUCY1A1	2.68E-32	0.49769132	0.723	0.47	5.35E-29	SMC4
EFHD1	5.64E-32	0.85035077	0.661	0.331	1.13E-28	SMC4
KCNE4	1.64E-31	0.28512034	0.688	0.454	3.29E-28	SMC4
MIR4435-2H	2.24E-31	0.26826873	0.771	0.553	4.48E-28	SMC4
SLIT3	3.56E-31	0.67563399	0.64	0.306	7.12E-28	SMC4

MRGPRF	3.66E-31	0.72719401	0.616	0.174	7.31E-28	SMC4
ALCAM	2.03E-30	0.63038476	0.693	0.438	4.06E-27	SMC4
GPNMB	4.58E-30	0.30775833	0.733	0.446	9.16E-27	SMC4
ANXA2	2.10E-29	0.36928409	0.981	0.815	4.20E-26	SMC4
GUCY1B1	2.42E-29	0.33048908	0.677	0.412	4.84E-26	SMC4
GMDS	1.20E-28	0.52211102	0.704	0.5	2.40E-25	SMC4
NFIB	1.05E-27	0.54046971	0.72	0.419	2.09E-24	SMC4
AXL	1.71E-27	0.56748762	0.656	0.347	3.43E-24	SMC4
PFN2	5.57E-27	0.49622011	0.651	0.387	1.11E-23	SMC4
CDO1	4.04E-26	0.38787973	0.653	0.354	8.08E-23	SMC4
GJA1	1.93E-25	0.69962907	0.651	0.364	3.86E-22	SMC4
CARMN	2.48E-25	0.67367654	0.635	0.339	4.96E-22	SMC4
SSR4	8.29E-25	0.32014103	0.981	0.871	1.66E-21	SMC4
WFDC1	1.85E-24	0.8825703	0.605	0.24	3.69E-21	SMC4
SYNPO	3.77E-24	0.61680105	0.661	0.441	7.54E-21	SMC4
ABCA8	5.85E-24	0.40575263	0.597	0.265	1.17E-20	SMC4
TSPAN2	6.19E-24	0.61150546	0.629	0.401	1.24E-20	SMC4
CCND1	6.35E-24	0.59715175	0.733	0.61	1.27E-20	SMC4
CITED4	8.23E-24	0.40407494	0.667	0.435	1.65E-20	SMC4
ECM2	9.31E-24	0.64234866	0.621	0.324	1.86E-20	SMC4
PID1	9.89E-24	0.35003554	0.659	0.387	1.98E-20	SMC4
PRSS35	1.62E-23	0.48102124	0.589	0.267	3.24E-20	SMC4
TBX2	1.75E-23	0.44878368	0.621	0.343	3.49E-20	SMC4
ITGA10	2.13E-23	0.56781938	0.651	0.383	4.26E-20	SMC4
CYFIP2	1.69E-22	0.8656849	0.635	0.478	3.38E-19	SMC4
TUBA1A	1.24E-21	0.28880763	0.981	0.837	2.48E-18	SMC4
WWTR1	7.54E-20	0.43896915	0.688	0.453	1.51E-16	SMC4
LRRC17	4.87E-19	0.63768633	0.624	0.386	9.73E-16	SMC4
CERCAM	7.33E-19	0.34393338	0.605	0.352	1.47E-15	SMC4
RGS4	1.61E-18	0.4384588	0.6	0.331	3.22E-15	SMC4
CTNNAL1	1.28E-17	0.42594715	0.651	0.485	2.56E-14	SMC4
CRLF1	4.64E-17	1.09920977	0.565	0.274	9.29E-14	SMC4
FIBIN	1.71E-16	0.52311143	0.563	0.249	3.41E-13	SMC4
MAP9	5.08E-15	0.54495218	0.603	0.375	1.02E-11	SMC4
WTIP	5.68E-15	0.55180335	0.584	0.305	1.14E-11	SMC4
ACTG2	7.24E-15	1.25744447	0.555	0.252	1.45E-11	SMC4
SEMA5A	9.13E-15	0.41111714	0.581	0.31	1.83E-11	SMC4
USP53	3.09E-14	0.48762564	0.605	0.43	6.18E-11	SMC4
DNAJB4	6.35E-14	0.38722182	0.672	0.539	1.27E-10	SMC4
IL33	8.03E-13	0.33595995	0.616	0.419	1.61E-09	SMC4
FBXO32	1.02E-12	0.496148	0.629	0.51	2.05E-09	SMC4
FOXC2	1.94E-11	0.43050001	0.573	0.298	3.88E-08	SMC4
DAPL1	2.33E-09	0.26979758	0.531	0.362	4.67E-06	SMC4
HMCN1	1.87E-08	0.75418616	0.533	0.297	3.75E-05	SMC4
DSP	3.09E-08	0.269618	0.488	0.177	6.18E-05	SMC4
DEPP1	2.04E-07	0.43508388	0.552	0.376	0.00040806	SMC4
ITIH5	3.08E-07	0.37601097	0.368	0.352	0.00061548	SMC4
PTGER3	3.99E-07	0.45754318	0.528	0.334	0.00079738	SMC4
CDH2	4.40E-07	0.6228276	0.531	0.336	0.0008805	SMC4
COL12A1	6.36E-07	0.58431177	0.549	0.426	0.00127292	SMC4
DLX6-AS1	0	2.0608535	0.9	0.21	0	SMC3
CRABP1	0	0.4709123	0.873	0.17	0	SMC3
FRZB	3.73E-307	3.05473145	0.996	0.478	7.46E-304	SMC3

PDE5A	1.43E-303	2.82346316	0.985	0.406	2.86E-300	SMC3
EFEMP1	1.74E-282	2.70061146	0.992	0.453	3.48E-279	SMC3
NDNF	4.26E-280	1.19393294	0.892	0.301	8.53E-277	SMC3
RAMP1	3.57E-268	2.34808779	0.958	0.415	7.15E-265	SMC3
ADIRF	1.96E-265	2.37970109	1	0.57	3.93E-262	SMC3
IGFBP2	2.31E-265	2.38454184	0.994	0.564	4.62E-262	SMC3
OGN	4.28E-257	2.4789545	0.975	0.414	8.56E-254	SMC3
TPM2	4.60E-256	2.43896873	1	0.506	9.20E-253	SMC3
C2orf40	3.29E-253	2.21329052	0.983	0.584	6.58E-250	SMC3
SOD3	6.52E-250	2.14840832	0.998	0.489	1.30E-246	SMC3
MYL9	1.47E-245	2.30003289	0.996	0.476	2.93E-242	SMC3
GUCY1A1	5.05E-240	1.94096783	0.965	0.467	1.01E-236	SMC3
BGN	1.35E-238	2.14155584	0.998	0.543	2.69E-235	SMC3
DSTN	3.36E-236	1.95075261	0.998	0.699	6.71E-233	SMC3
DKK3	5.87E-227	1.67546428	0.963	0.456	1.17E-223	SMC3
RARRES2	1.90E-226	1.62339753	0.975	0.518	3.80E-223	SMC3
CLU	1.03E-225	1.93884956	0.994	0.517	2.06E-222	SMC3
GREM2	5.79E-222	1.66565351	0.882	0.43	1.16E-218	SMC3
IGFBP7	3.66E-220	2.08019564	1	0.765	7.31E-217	SMC3
ITGA8	6.28E-219	1.67527949	0.933	0.4	1.26E-215	SMC3
TAGLN	2.45E-218	2.17727601	1	0.544	4.91E-215	SMC3
IGFBP5	4.54E-216	2.33768819	0.979	0.558	9.07E-213	SMC3
CTGF	3.67E-213	2.14004215	0.99	0.583	7.35E-210	SMC3
MYH10	3.87E-212	1.93944111	0.923	0.41	7.73E-209	SMC3
FHL1	4.68E-210	1.85954977	0.965	0.499	9.36E-207	SMC3
SELENOM	6.87E-210	1.65555952	0.985	0.648	1.37E-206	SMC3
S100A6	6.39E-209	1.4225894	0.996	0.936	1.28E-205	SMC3
CD151	9.94E-204	1.69257539	0.979	0.604	1.99E-200	SMC3
SERPING1	5.52E-203	1.54773213	0.988	0.559	1.10E-199	SMC3
ID4	6.32E-202	1.85564317	0.929	0.47	1.26E-198	SMC3
CRYAB	7.90E-202	1.89535424	0.946	0.502	1.58E-198	SMC3
MAP1B	4.91E-200	1.54866591	0.956	0.469	9.83E-197	SMC3
IGFBP6	1.53E-198	2.24158426	0.938	0.459	3.07E-195	SMC3
PCK1	2.44E-195	0.54028609	0.676	0.125	4.87E-192	SMC3
CALD1	9.10E-193	1.68223487	0.998	0.584	1.82E-189	SMC3
TPM1	9.68E-192	1.80519221	0.979	0.57	1.94E-188	SMC3
ACTA2	2.52E-187	2.26288912	0.944	0.498	5.03E-184	SMC3
FXYD1	1.76E-186	1.64638544	0.923	0.47	3.52E-183	SMC3
PHGDH	4.93E-186	0.85883549	0.846	0.31	9.86E-183	SMC3
C1R	7.27E-185	1.20486434	0.983	0.501	1.45E-181	SMC3
PLAC9	7.34E-183	1.43369141	0.952	0.527	1.47E-179	SMC3
TINAGL1	2.33E-182	1.3286672	0.946	0.501	4.67E-179	SMC3
LMOD1	1.77E-181	1.54380888	0.896	0.316	3.54E-178	SMC3
CDO1	1.84E-180	1.46846983	0.861	0.351	3.67E-177	SMC3
NOV	8.14E-180	1.5959993	0.906	0.491	1.63E-176	SMC3
MFGE8	3.15E-178	1.47405712	0.938	0.49	6.31E-175	SMC3
CP	1.41E-177	0.90964054	0.882	0.399	2.82E-174	SMC3
CAVIN3	2.03E-177	1.3993044	0.975	0.59	4.07E-174	SMC3
CYR61	2.22E-177	1.6334612	0.969	0.565	4.44E-174	SMC3
LGALS3BP	3.55E-176	1.22789048	0.936	0.56	7.10E-173	SMC3
DLX5	8.53E-175	1.59465147	0.821	0.309	1.71E-171	SMC3
LGALS1	3.14E-174	1.34504968	0.996	0.833	6.29E-171	SMC3
C11orf96	3.25E-174	1.61838898	0.96	0.499	6.51E-171	SMC3

GEM	3.32E-174	1.69922314	0.911	0.587	6.64E-171	SMC3
VCAN	1.60E-173	1.29386962	0.985	0.528	3.19E-170	SMC3
MYH11	1.96E-173	1.46776944	0.913	0.378	3.92E-170	SMC3
MGP	4.42E-173	1.7006306	0.998	0.729	8.83E-170	SMC3
NBL1	4.26E-172	1.59716626	0.911	0.574	8.51E-169	SMC3
NUPR1	6.99E-168	1.38492622	0.923	0.482	1.40E-164	SMC3
EDIL3	2.17E-165	1.08386965	0.929	0.522	4.33E-162	SMC3
GSTM3	3.72E-164	0.99517854	0.911	0.616	7.44E-161	SMC3
SSPN	7.98E-161	1.23438895	0.892	0.479	1.60E-157	SMC3
RCAN2	1.12E-160	1.28606629	0.894	0.56	2.24E-157	SMC3
RERG	2.49E-156	0.36542609	0.803	0.266	4.97E-153	SMC3
PPP1R14A	2.74E-155	1.68498598	0.879	0.437	5.49E-152	SMC3
PCOLCE	7.20E-155	1.05557634	0.915	0.525	1.44E-151	SMC3
AEBP1	5.68E-153	0.796935	0.95	0.519	1.14E-149	SMC3
COX7A1	7.16E-153	1.31819454	0.904	0.498	1.43E-149	SMC3
KCNMB1	8.85E-153	0.63976397	0.85	0.393	1.77E-149	SMC3
PALLD	1.74E-152	1.23645083	0.892	0.458	3.47E-149	SMC3
ID3	2.18E-151	1.37645462	0.967	0.639	4.35E-148	SMC3
PRELP	1.00E-150	1.18789377	0.869	0.37	2.00E-147	SMC3
HAPLN1	6.66E-147	0.46535812	0.809	0.446	1.33E-143	SMC3
CSRP1	2.12E-146	1.45723814	0.917	0.531	4.25E-143	SMC3
SNCG	2.46E-146	1.60304549	0.902	0.587	4.93E-143	SMC3
PLS3	4.45E-145	1.1893328	0.913	0.554	8.90E-142	SMC3
VASN	1.95E-144	1.11277863	0.834	0.416	3.91E-141	SMC3
MRGPRF	1.42E-143	0.54367129	0.767	0.171	2.85E-140	SMC3
GUCY1B1	9.06E-142	0.57624261	0.829	0.41	1.81E-138	SMC3
RBP1	1.21E-141	0.53615556	0.838	0.417	2.41E-138	SMC3
LMO7	4.60E-140	1.56499543	0.823	0.407	9.20E-137	SMC3
NOTCH3	1.16E-139	0.37758838	0.817	0.383	2.32E-136	SMC3
KCNK17	1.54E-139	0.9187166	0.776	0.298	3.08E-136	SMC3
S100A4	7.32E-139	1.13280826	1	0.893	1.46E-135	SMC3
TNC	6.08E-138	0.30879292	0.857	0.507	1.22E-134	SMC3
LTBP1	1.58E-137	1.06361605	0.859	0.379	3.16E-134	SMC3
PDLIM3	2.37E-137	1.18998173	0.894	0.468	4.75E-134	SMC3
PAWR	2.57E-137	1.08468643	0.859	0.492	5.14E-134	SMC3
PRRX2	8.60E-137	1.04321506	0.827	0.384	1.72E-133	SMC3
C1S	1.12E-136	0.6900175	0.882	0.428	2.25E-133	SMC3
CPE	2.30E-135	0.99121638	0.904	0.521	4.60E-132	SMC3
CYP1B1	2.05E-134	0.25991821	0.83	0.309	4.11E-131	SMC3
LMO2	4.10E-134	1.45639475	0.877	0.497	8.19E-131	SMC3
COL14A1	1.51E-132	1.0609813	0.877	0.471	3.01E-129	SMC3
SEPT4	2.28E-132	0.88289176	0.836	0.507	4.56E-129	SMC3
TGFB1I1	3.36E-132	1.1226252	0.854	0.425	6.71E-129	SMC3
TIMP3	9.80E-130	1.04686537	0.884	0.513	1.96E-126	SMC3
SPINT2	4.06E-127	1.15781212	0.892	0.576	8.11E-124	SMC3
SGCA	8.18E-127	0.71099665	0.819	0.438	1.64E-123	SMC3
CRIP2	1.37E-126	1.03933539	0.944	0.613	2.75E-123	SMC3
AKR1C3	1.83E-126	0.86184703	0.892	0.533	3.66E-123	SMC3
THBS2	5.76E-126	0.42241924	0.848	0.462	1.15E-122	SMC3
CDH11	8.44E-124	0.27007978	0.85	0.5	1.69E-120	SMC3
NEXN	5.04E-123	1.3427399	0.838	0.418	1.01E-119	SMC3
MT1M	1.83E-122	0.93917629	0.817	0.365	3.66E-119	SMC3
GSN	2.08E-122	1.07970939	0.971	0.646	4.16E-119	SMC3

ENPP2	2.34E-122	1.31103569	0.8	0.43	4.67E-119	SMC3
LMO3	8.03E-122	1.21891222	0.759	0.292	1.61E-118	SMC3
CAV1	1.41E-121	0.95195638	0.965	0.617	2.83E-118	SMC3
LTBP4	1.62E-118	0.93361881	0.85	0.451	3.24E-115	SMC3
NR2F2	3.72E-118	0.81593452	0.931	0.609	7.44E-115	SMC3
NNMT	6.01E-118	0.94568376	0.944	0.604	1.20E-114	SMC3
CCDC3	1.19E-117	0.34586812	0.809	0.44	2.37E-114	SMC3
HCFC1R1	5.55E-117	1.06054532	0.89	0.559	1.11E-113	SMC3
ATP1B1	7.67E-117	1.40628855	0.832	0.509	1.53E-113	SMC3
LHFPL6	8.14E-117	0.85776928	0.869	0.465	1.63E-113	SMC3
LMCD1	1.20E-116	1.31259988	0.84	0.552	2.40E-113	SMC3
EGR1	1.55E-116	1.32729738	0.908	0.586	3.09E-113	SMC3
CNN3	2.84E-116	0.77289423	0.911	0.564	5.69E-113	SMC3
CAVIN1	5.57E-116	0.96819931	0.906	0.544	1.11E-112	SMC3
CRISPLD1	7.18E-116	0.30311753	0.802	0.459	1.44E-112	SMC3
MFAP4	2.18E-115	1.2195319	0.817	0.368	4.36E-112	SMC3
OSR1	5.95E-114	1.13312727	0.738	0.22	1.19E-110	SMC3
TNFRSF11B	1.50E-113	0.60269137	0.809	0.465	3.00E-110	SMC3
CD9	1.71E-113	0.96115118	0.979	0.663	3.42E-110	SMC3
PMEPA1	2.79E-113	0.85327316	0.886	0.592	5.57E-110	SMC3
COL8A1	1.84E-112	0.86643463	0.813	0.384	3.68E-109	SMC3
DPT	4.09E-112	1.307314	0.802	0.492	8.17E-109	SMC3
A2M	5.14E-112	0.91054602	0.958	0.652	1.03E-108	SMC3
CXCL12	2.32E-111	0.70209656	0.884	0.641	4.63E-108	SMC3
SDC2	3.13E-111	0.65718954	0.842	0.419	6.27E-108	SMC3
HSPB8	6.15E-111	0.32072399	0.765	0.339	1.23E-107	SMC3
RASL11A	1.80E-110	0.45506445	0.802	0.485	3.59E-107	SMC3
PFN2	1.05E-107	1.09264063	0.769	0.385	2.10E-104	SMC3
EFEMP2	4.19E-107	0.83617991	0.815	0.448	8.37E-104	SMC3
ISLR	1.75E-106	0.40669062	0.775	0.345	3.50E-103	SMC3
IFITM3	1.87E-104	0.94869512	0.996	0.733	3.74E-101	SMC3
TBX2	1.04E-103	0.40534266	0.742	0.341	2.09E-100	SMC3
SGCE	4.30E-103	0.53145873	0.788	0.405	8.59E-100	SMC3
PTN	1.09E-101	2.15938241	0.753	0.381	2.17E-98	SMC3
TIMP1	1.45E-101	0.55707577	0.992	0.797	2.89E-98	SMC3
SOST	2.82E-100	1.17682864	0.699	0.185	5.64E-97	SMC3
ANGPT1	3.27E-99	0.46284281	0.775	0.429	6.55E-96	SMC3
SUCNR1	3.93E-98	1.54018682	0.713	0.286	7.86E-95	SMC3
COL6A2	1.06E-97	0.67617011	0.892	0.571	2.13E-94	SMC3
NRGN	1.70E-97	1.29129671	0.796	0.462	3.41E-94	SMC3
RRAD	2.82E-97	0.85821281	0.79	0.51	5.63E-94	SMC3
CCDC80	4.78E-97	0.26357747	0.869	0.558	9.56E-94	SMC3
F10	1.38E-96	0.65138728	0.705	0.22	2.77E-93	SMC3
PTHLH	3.01E-96	0.26767033	0.711	0.305	6.03E-93	SMC3
SPON1	5.55E-96	0.39169926	0.723	0.402	1.11E-92	SMC3
HTRA1	1.20E-95	0.79688242	0.879	0.585	2.40E-92	SMC3
S100A13	1.34E-95	0.95824097	0.869	0.541	2.68E-92	SMC3
HES1	2.17E-94	0.67735521	0.852	0.522	4.34E-91	SMC3
GPM6B	1.96E-93	0.51370449	0.767	0.463	3.92E-90	SMC3
CYBRD1	7.66E-92	0.55184786	0.802	0.447	1.53E-88	SMC3
RGS3	1.05E-91	0.90954868	0.798	0.511	2.10E-88	SMC3
CCND1	7.71E-91	0.88487918	0.846	0.609	1.54E-87	SMC3
MT1E	2.55E-90	0.89532062	0.838	0.581	5.10E-87	SMC3

TSC22D1	4.52E-90	0.94138862	0.9	0.633	9.04E-87	SMC3
FILIP1L	1.96E-89	0.79435594	0.898	0.551	3.92E-86	SMC3
ISYNA1	6.21E-89	0.88431778	0.78	0.473	1.24E-85	SMC3
HMCN1	3.02E-88	1.01095767	0.732	0.294	6.04E-85	SMC3
CAV2	3.76E-87	0.7803628	0.825	0.474	7.53E-84	SMC3
MBNL1-AS1	4.40E-87	1.54943995	0.736	0.403	8.81E-84	SMC3
NDUFA4L2	6.96E-85	0.92376064	0.753	0.442	1.39E-81	SMC3
IL13RA2	2.51E-84	0.26319729	0.672	0.219	5.02E-81	SMC3
C1QTNF7	5.98E-84	0.38893035	0.69	0.29	1.20E-80	SMC3
LTBP2	3.32E-83	0.47058992	0.78	0.475	6.64E-80	SMC3
CDKN1C	3.47E-83	0.93745331	0.85	0.601	6.94E-80	SMC3
SFRP4	4.02E-83	0.28358795	0.746	0.406	8.03E-80	SMC3
EMILIN1	2.25E-82	0.48088211	0.744	0.389	4.50E-79	SMC3
LOXL1	3.79E-82	0.45592877	0.751	0.414	7.57E-79	SMC3
FOXC1	1.35E-81	0.96778558	0.792	0.477	2.69E-78	SMC3
PTGIS	2.85E-81	0.93754523	0.742	0.328	5.70E-78	SMC3
CCL2	9.80E-81	0.42423375	0.9	0.533	1.96E-77	SMC3
TCEAL2	1.17E-80	1.13042426	0.717	0.372	2.35E-77	SMC3
THSD4	1.21E-80	0.95914196	0.686	0.259	2.43E-77	SMC3
SLC25A4	2.26E-80	0.99121048	0.759	0.474	4.53E-77	SMC3
GPX3	6.63E-80	0.26805825	0.786	0.449	1.33E-76	SMC3
AKR1C1	1.06E-79	0.31104568	0.738	0.42	2.13E-76	SMC3
IL33	1.27E-79	0.64268831	0.753	0.417	2.53E-76	SMC3
GAS6	2.49E-79	0.80171351	0.857	0.62	4.97E-76	SMC3
MECOM	9.21E-79	1.31870655	0.717	0.355	1.84E-75	SMC3
NUDT4	4.62E-77	0.32070005	0.832	0.491	9.23E-74	SMC3
GPRC5C	1.26E-76	0.55976743	0.74	0.404	2.53E-73	SMC3
PRDM6	1.21E-75	1.08905884	0.694	0.213	2.42E-72	SMC3
JUN	1.29E-75	1.21909288	0.99	0.812	2.58E-72	SMC3
PDGFA	1.32E-75	0.63417713	0.792	0.534	2.64E-72	SMC3
TMEM47	5.35E-75	0.32865204	0.738	0.378	1.07E-71	SMC3
GJA4	2.30E-74	0.27484631	0.724	0.388	4.60E-71	SMC3
C1QTNF1	1.17E-73	0.8408296	0.728	0.362	2.34E-70	SMC3
RSPO2	1.28E-73	0.74142769	0.622	0.118	2.56E-70	SMC3
PDGFRA	1.43E-73	0.69960159	0.715	0.347	2.86E-70	SMC3
PLK2	2.28E-73	1.14794597	0.761	0.518	4.57E-70	SMC3
IL1R1	2.31E-72	0.6871975	0.751	0.437	4.63E-69	SMC3
SMOC2	6.86E-72	0.2624253	0.757	0.431	1.37E-68	SMC3
IER5L	1.28E-71	0.67458995	0.89	0.663	2.55E-68	SMC3
ELN	2.29E-71	0.67184772	0.705	0.31	4.57E-68	SMC3
GDF10	2.38E-71	0.53168591	0.647	0.27	4.76E-68	SMC3
C9orf3	3.94E-71	0.73064722	0.792	0.488	7.88E-68	SMC3
ACTG2	3.79E-70	0.74405233	0.669	0.25	7.58E-67	SMC3
FBLIM1	3.28E-69	0.9677442	0.742	0.427	6.57E-66	SMC3
ANTXR1	1.95E-68	0.54608507	0.723	0.404	3.89E-65	SMC3
RGS5	4.53E-68	0.76898464	0.751	0.451	9.05E-65	SMC3
ID1	1.27E-66	0.38210981	0.809	0.541	2.53E-63	SMC3
FBLN5	4.13E-65	0.91607698	0.699	0.315	8.26E-62	SMC3
IL34	5.13E-65	0.25969908	0.78	0.563	1.03E-61	SMC3
TFPI	2.76E-64	0.36008758	0.832	0.564	5.51E-61	SMC3
CARMN	8.62E-64	0.33978412	0.697	0.337	1.72E-60	SMC3
AQP1	2.09E-63	0.4814026	0.823	0.536	4.18E-60	SMC3
FBXO32	2.38E-63	0.29519815	0.782	0.508	4.76E-60	SMC3

RHOB	7.89E-63	0.73655328	0.869	0.571	1.58E-59	SMC3
FOXC2	3.78E-62	0.80309131	0.682	0.296	7.57E-59	SMC3
UACA	5.62E-62	0.30076722	0.836	0.581	1.12E-58	SMC3
PLN	8.01E-62	0.4259352	0.684	0.337	1.60E-58	SMC3
EPB41L2	2.24E-61	0.77604452	0.786	0.531	4.48E-58	SMC3
EFHD1	2.53E-61	0.97057128	0.682	0.33	5.06E-58	SMC3
GMDS	4.84E-61	1.0263049	0.738	0.499	9.69E-58	SMC3
CTSK	8.49E-61	0.40095498	0.751	0.491	1.70E-57	SMC3
COL18A1	9.94E-61	0.3438892	0.821	0.524	1.99E-57	SMC3
SLC40A1	9.54E-59	0.67962322	0.767	0.51	1.91E-55	SMC3
MXRA8	1.38E-58	0.5875042	0.723	0.436	2.76E-55	SMC3
FEZ1	4.70E-58	0.6206235	0.701	0.418	9.39E-55	SMC3
LGALS3	9.28E-58	0.50171052	0.971	0.747	1.86E-54	SMC3
FSTL1	1.16E-57	0.62815641	0.734	0.435	2.32E-54	SMC3
MSRB3	7.47E-57	0.74548721	0.723	0.408	1.49E-53	SMC3
COPZ2	1.01E-56	0.66697549	0.724	0.44	2.02E-53	SMC3
TMEM98	1.38E-56	0.81124036	0.697	0.363	2.77E-53	SMC3
HES4	1.46E-56	0.52611845	0.769	0.522	2.92E-53	SMC3
PDGFRB	1.96E-56	0.35337764	0.709	0.389	3.92E-53	SMC3
MFAP2	2.12E-56	0.29336122	0.671	0.317	4.25E-53	SMC3
DNAJB4	1.13E-55	0.37032742	0.809	0.537	2.26E-52	SMC3
SVIL	1.36E-53	0.53257146	0.773	0.489	2.72E-50	SMC3
ATF3	2.66E-53	0.70939731	0.919	0.63	5.32E-50	SMC3
CD59	3.29E-53	0.40416839	0.879	0.579	6.58E-50	SMC3
SPARC	1.05E-52	0.32680575	0.861	0.63	2.10E-49	SMC3
PPIC	1.52E-51	0.551174	0.771	0.549	3.04E-48	SMC3
TWIST1	2.52E-51	0.42139793	0.69	0.395	5.05E-48	SMC3
CNN1	5.35E-51	0.83350479	0.651	0.264	1.07E-47	SMC3
CST3	6.25E-51	0.25984632	1	0.734	1.25E-47	SMC3
OMD	1.02E-50	0.96245755	0.663	0.322	2.04E-47	SMC3
PDLIM7	1.57E-50	0.65305928	0.726	0.463	3.14E-47	SMC3
NFIB	3.87E-50	0.58635719	0.744	0.418	7.73E-47	SMC3
P4HA2	9.11E-48	0.36465942	0.701	0.462	1.82E-44	SMC3
COL6A1	2.80E-47	0.31759098	0.753	0.476	5.59E-44	SMC3
SYNPO	2.92E-47	0.69642363	0.692	0.44	5.83E-44	SMC3
PRRX1	5.70E-47	0.46023537	0.707	0.389	1.14E-43	SMC3
CAMK2N1	6.09E-47	0.33600029	0.674	0.447	1.22E-43	SMC3
MAP9	7.63E-47	0.73278046	0.663	0.374	1.53E-43	SMC3
LIMCH1	5.49E-46	0.67283334	0.696	0.405	1.10E-42	SMC3
MYLK	6.35E-46	0.64055994	0.709	0.431	1.27E-42	SMC3
NTS	1.33E-45	1.18120517	0.229	0.091	2.66E-42	SMC3
SMPX	1.39E-45	0.42131873	0.572	0.171	2.79E-42	SMC3
ACTC1	2.32E-45	0.88727839	0.628	0.309	4.65E-42	SMC3
C12orf75	3.37E-45	0.35631801	0.805	0.571	6.73E-42	SMC3
MCAM	3.25E-44	0.38059394	0.717	0.48	6.50E-41	SMC3
TNFRSF12A	8.49E-44	0.64052092	0.778	0.562	1.70E-40	SMC3
SERPINH1	1.08E-42	0.27327115	0.788	0.572	2.16E-39	SMC3
WWTR1	1.01E-41	0.5463647	0.717	0.452	2.02E-38	SMC3
PLPP5	1.33E-40	0.30988743	0.767	0.551	2.67E-37	SMC3
LRRC17	1.81E-40	0.34085705	0.663	0.385	3.63E-37	SMC3
WTIP	9.47E-40	0.43625422	0.622	0.304	1.89E-36	SMC3
CTSF	1.63E-39	0.66730616	0.678	0.449	3.26E-36	SMC3
IGFBP3	2.02E-38	0.81257307	0.645	0.38	4.04E-35	SMC3

PDLIM1	6.10E-38	0.28979701	0.815	0.617	1.22E-34	SMC3
PRDX4	6.75E-38	0.35904667	0.852	0.65	1.35E-34	SMC3
GPX8	9.31E-38	0.28322706	0.674	0.438	1.86E-34	SMC3
NRP1	2.55E-37	0.43486107	0.788	0.553	5.11E-34	SMC3
ITM2C	4.97E-37	0.53625415	0.738	0.566	9.94E-34	SMC3
PCDH7	1.29E-36	0.57254475	0.64	0.333	2.59E-33	SMC3
MT1X	6.39E-36	0.56023599	0.746	0.541	1.28E-32	SMC3
PPP1R12B	7.17E-36	0.3892683	0.649	0.388	1.43E-32	SMC3
NUCB2	7.91E-36	0.31942328	0.9	0.664	1.58E-32	SMC3
ITGA10	2.60E-35	0.59610411	0.661	0.383	5.20E-32	SMC3
SYNPO2	4.60E-35	0.39387411	0.626	0.362	9.20E-32	SMC3
ERLEC1	6.27E-35	0.32931286	0.832	0.615	1.25E-31	SMC3
PLPP1	3.80E-34	0.47338545	0.721	0.559	7.60E-31	SMC3
SPTBN1	5.68E-34	0.25216476	0.763	0.507	1.14E-30	SMC3
GADD45A	9.09E-34	0.26324673	0.807	0.588	1.82E-30	SMC3
LGALSL	2.57E-33	0.25442629	0.657	0.469	5.13E-30	SMC3
TIMP4	1.24E-32	0.39333779	0.607	0.325	2.48E-29	SMC3
KCNE4	1.66E-32	0.72085707	0.651	0.454	3.32E-29	SMC3
NPR3	2.36E-32	0.3418497	0.572	0.18	4.73E-29	SMC3
FIBIN	4.54E-32	0.69356386	0.578	0.248	9.07E-29	SMC3
ITGBL1	1.97E-31	0.67986224	0.63	0.359	3.95E-28	SMC3
LBH	3.29E-31	0.30648498	0.838	0.655	6.58E-28	SMC3
CKB	1.18E-30	0.51257008	0.62	0.402	2.37E-27	SMC3
HSPA1B	1.88E-30	0.3982307	0.855	0.663	3.76E-27	SMC3
COL21A1	2.10E-29	0.47492879	0.663	0.481	4.21E-26	SMC3
SORBS2	5.77E-29	0.37938648	0.647	0.415	1.15E-25	SMC3
VCAM1	1.57E-28	0.299568	0.686	0.536	3.15E-25	SMC3
FBXL22	4.15E-28	0.25742319	0.597	0.409	8.30E-25	SMC3
INMT	1.18E-27	0.70396588	0.609	0.295	2.36E-24	SMC3
FBLN1	2.29E-27	0.2907757	0.599	0.352	4.58E-24	SMC3
ECM2	2.47E-27	0.3433157	0.584	0.324	4.95E-24	SMC3
ENPP1	3.46E-27	0.32099658	0.622	0.442	6.92E-24	SMC3
ZNF503	4.93E-27	0.59314275	0.655	0.483	9.87E-24	SMC3
TSPAN13	6.21E-26	0.9226502	0.62	0.463	1.24E-22	SMC3
DLX6	6.53E-26	0.66640242	0.545	0.086	1.31E-22	SMC3
FKBP2	2.18E-24	0.28889527	0.911	0.727	4.36E-21	SMC3
SEMA5A	1.97E-23	0.52977965	0.584	0.31	3.95E-20	SMC3
LRP1	2.08E-23	0.47788622	0.669	0.467	4.17E-20	SMC3
PMP22	1.05E-22	0.353003	0.74	0.565	2.10E-19	SMC3
TPD52L1	1.26E-22	0.71907209	0.595	0.379	2.51E-19	SMC3
PRSS35	1.88E-22	0.33969279	0.555	0.266	3.75E-19	SMC3
FAM114A1	2.44E-22	0.50353056	0.661	0.507	4.89E-19	SMC3
NT5DC2	3.98E-22	0.50839123	0.615	0.451	7.97E-19	SMC3
CERCAM	3.01E-21	0.31378062	0.584	0.351	6.02E-18	SMC3
PHLDA3	4.65E-21	0.38080765	0.663	0.551	9.31E-18	SMC3
KRT19	8.38E-21	0.39953152	0.551	0.305	1.68E-17	SMC3
BEX2	8.29E-20	0.58246249	0.59	0.403	1.66E-16	SMC3
RGS7BP	4.13E-19	0.80405934	0.563	0.279	8.26E-16	SMC3
FGL2	4.34E-19	0.34701002	0.717	0.52	8.69E-16	SMC3
HSPB6	7.48E-19	0.56066947	0.555	0.281	1.50E-15	SMC3
ERRFI1	9.56E-17	0.35393791	0.601	0.454	1.91E-13	SMC3
MRC2	2.01E-15	0.48260921	0.58	0.429	4.02E-12	SMC3
SCX	4.20E-15	0.34456733	0.539	0.354	8.40E-12	SMC3

ASPN	9.48E-15	0.66620644	0.574	0.383	1.90E-11	SMC3
CST6	1.16E-14	0.35407991	0.547	0.335	2.31E-11	SMC3
HAND2	1.74E-14	0.71960519	0.545	0.299	3.49E-11	SMC3
SAMD11	2.04E-14	0.53685071	0.532	0.275	4.09E-11	SMC3
PDLIM4	2.65E-14	0.50144742	0.611	0.499	5.31E-11	SMC3
CITED4	3.55E-14	0.37200461	0.599	0.435	7.10E-11	SMC3
PGF	6.76E-14	0.5509069	0.597	0.515	1.35E-10	SMC3
LSAMP	2.63E-12	0.33385751	0.511	0.303	5.25E-09	SMC3
DEPP1	3.30E-12	0.66152645	0.557	0.375	6.60E-09	SMC3
SCRG1	4.47E-12	0.91289376	0.534	0.389	8.93E-09	SMC3
MAP1A	1.24E-11	0.42486794	0.565	0.436	2.48E-08	SMC3
DAPL1	7.31E-11	0.36875935	0.53	0.362	1.46E-07	SMC3
CYTL1	1.06E-10	0.41068522	0.557	0.415	2.12E-07	SMC3
NPTX2	2.48E-10	0.51110886	0.53	0.333	4.96E-07	SMC3
HSPB7	7.71E-10	0.54864781	0.516	0.319	1.54E-06	SMC3
ASS1	1.19E-09	0.26960265	0.555	0.42	2.38E-06	SMC3
AKR1C2	1.46E-09	0.55739553	0.518	0.325	2.92E-06	SMC3
ANKH	2.00E-09	0.30747855	0.63	0.52	4.01E-06	SMC3
LNP1	7.66E-09	0.53386163	0.524	0.342	1.53E-05	SMC3
GJA1	1.51E-07	0.48201757	0.539	0.365	0.0003022	SMC3
PDGFD	2.65E-07	0.54079641	0.539	0.404	0.00053028	SMC3
LDOC1	6.91E-07	0.29046135	0.532	0.396	0.00138273	SMC3
AKAP12	2.43E-06	0.25380485	0.547	0.425	0.00486668	SMC3
CDH13	7.42E-06	0.3680735	0.528	0.351	0.01484056	SMC3
ENAH	2.03E-05	0.35134612	0.563	0.442	0.04066831	SMC3
MS4A6A	5.04E-67	1.30713794	0.947	0.55	1.01E-63	SMC5
AIF1	4.03E-66	1.30050727	0.981	0.555	8.06E-63	SMC5
LYZ	1.63E-64	1.27408551	0.957	0.567	3.26E-61	SMC5
CD14	1.15E-63	1.34580708	0.967	0.556	2.30E-60	SMC5
FTL	3.48E-63	1.15690472	1	0.981	6.97E-60	SMC5
IGSF6	2.61E-61	0.98005139	0.928	0.537	5.22E-58	SMC5
HLA-DPA1	3.90E-61	1.41408871	1	0.803	7.79E-58	SMC5
HLA-DRA	4.89E-61	1.34273938	1	0.815	9.79E-58	SMC5
HLA-DQB1	6.07E-59	1.13319647	0.99	0.685	1.21E-55	SMC5
PLAUR	8.62E-59	1.16219112	0.957	0.618	1.72E-55	SMC5
F13A1	4.64E-58	1.0362546	0.909	0.552	9.27E-55	SMC5
HLA-DPB1	1.83E-57	1.30671148	0.995	0.789	3.66E-54	SMC5
HLA-DRB1	2.45E-57	1.33336853	0.99	0.79	4.90E-54	SMC5
CST3	5.15E-57	1.13091671	1	0.736	1.03E-53	SMC5
FCER1G	2.33E-56	1.14648226	0.981	0.566	4.66E-53	SMC5
HLA-DQA1	8.81E-56	1.20738561	0.967	0.661	1.76E-52	SMC5
FTH1	2.33E-55	0.97226708	1	0.976	4.67E-52	SMC5
TYROBP	3.61E-55	1.08238018	1	0.582	7.22E-52	SMC5
HLA-DRB5	4.58E-55	1.24506649	0.981	0.797	9.16E-52	SMC5
CEBPD	6.27E-55	1.19088783	1	0.698	1.25E-51	SMC5
FCGR2A	2.11E-54	0.95238993	0.904	0.492	4.23E-51	SMC5
C1QC	2.58E-54	1.45466301	0.885	0.546	5.16E-51	SMC5
HLA-DMA	4.56E-54	1.04245383	0.967	0.652	9.12E-51	SMC5
CPVL	2.17E-53	1.09007336	0.895	0.505	4.34E-50	SMC5
SERPINA1	2.74E-53	0.85455025	0.866	0.445	5.47E-50	SMC5
CTSB	4.82E-53	0.85028741	0.981	0.591	9.65E-50	SMC5
C1QA	1.33E-52	1.47076579	0.904	0.614	2.67E-49	SMC5
CYBB	9.26E-52	0.89986648	0.909	0.527	1.85E-48	SMC5

SPI1	1.25E-51	0.81661107	0.9	0.493	2.50E-48	SMC5
CTSZ	1.88E-51	0.91013667	0.986	0.697	3.77E-48	SMC5
HLA-DMB	2.29E-51	0.80370289	0.923	0.553	4.59E-48	SMC5
TMEM176B	2.65E-51	0.89221492	0.909	0.497	5.30E-48	SMC5
CTSS	2.79E-51	0.89723349	0.986	0.682	5.59E-48	SMC5
HLA-DQA2	3.71E-51	1.12265692	0.976	0.71	7.42E-48	SMC5
C5AR1	8.79E-51	1.05721072	0.88	0.487	1.76E-47	SMC5
PLEK	1.26E-50	1.0693185	0.928	0.535	2.52E-47	SMC5
LST1	2.01E-50	0.80809584	0.9	0.514	4.02E-47	SMC5
NAMPT	4.68E-50	0.97186425	0.976	0.645	9.35E-47	SMC5
PSAP	7.12E-50	0.8802471	1	0.671	1.42E-46	SMC5
CD68	9.74E-50	0.77918834	0.933	0.504	1.95E-46	SMC5
SAT1	1.35E-49	0.9222973	1	0.835	2.69E-46	SMC5
CD74	1.29E-47	1.01950301	0.995	0.897	2.59E-44	SMC5
TMEM176A	1.41E-47	0.62420279	0.871	0.487	2.82E-44	SMC5
MNDA	2.64E-47	1.10874781	0.866	0.493	5.28E-44	SMC5
FCGRT	2.98E-46	0.89954233	0.967	0.628	5.96E-43	SMC5
GRN	1.54E-45	0.79830795	0.962	0.652	3.08E-42	SMC5
CXCL16	5.26E-45	0.68656664	0.866	0.511	1.05E-41	SMC5
CLEC7A	6.20E-45	0.62547134	0.861	0.519	1.24E-41	SMC5
MAFB	1.08E-44	0.90409642	0.909	0.581	2.16E-41	SMC5
MS4A7	2.10E-44	1.11688901	0.847	0.473	4.19E-41	SMC5
SPINT2	5.05E-44	0.59248812	0.923	0.577	1.01E-40	SMC5
C1QB	6.93E-44	1.52816428	0.847	0.542	1.39E-40	SMC5
FILIP1L	2.35E-43	0.73253352	0.928	0.553	4.70E-40	SMC5
CEBPB	2.96E-42	0.76371523	0.981	0.621	5.92E-39	SMC5
TYMP	4.02E-42	0.67004575	0.967	0.665	8.04E-39	SMC5
TNFSF13B	6.13E-42	0.61797233	0.885	0.588	1.23E-38	SMC5
RAB31	6.18E-42	0.51390727	0.904	0.567	1.24E-38	SMC5
NPC2	7.11E-42	0.83662364	0.995	0.722	1.42E-38	SMC5
SGK1	1.02E-41	0.75891708	0.928	0.613	2.04E-38	SMC5
CTSH	1.06E-41	0.76582282	0.919	0.611	2.12E-38	SMC5
CFD	1.21E-41	0.82383763	0.866	0.473	2.42E-38	SMC5
CALD1	1.30E-41	0.75373539	0.947	0.586	2.60E-38	SMC5
FCGR3A	1.63E-41	0.87441474	0.842	0.453	3.25E-38	SMC5
DAB2	4.45E-41	0.68339752	0.909	0.603	8.89E-38	SMC5
FGL2	6.33E-41	0.9354442	0.895	0.52	1.27E-37	SMC5
MRC1	6.48E-41	0.9091722	0.823	0.572	1.30E-37	SMC5
CSF1R	1.29E-40	0.53859544	0.809	0.433	2.58E-37	SMC5
GLUL	1.48E-40	0.74474544	0.947	0.568	2.96E-37	SMC5
IL18	2.37E-40	0.55210219	0.794	0.457	4.74E-37	SMC5
ADIRF	3.08E-40	0.59259451	0.962	0.573	6.16E-37	SMC5
MGP	6.30E-40	0.52045967	0.976	0.731	1.26E-36	SMC5
MYL9	6.96E-40	0.98564861	0.847	0.48	1.39E-36	SMC5
C11orf96	3.44E-39	0.92119992	0.847	0.502	6.87E-36	SMC5
S100A9	2.97E-38	1.35157894	0.804	0.486	5.95E-35	SMC5
CD163	8.47E-38	0.73360998	0.804	0.496	1.69E-34	SMC5
IFI30	9.40E-38	0.66031287	0.823	0.52	1.88E-34	SMC5
GAS6	1.00E-37	0.64480997	0.914	0.622	2.01E-34	SMC5
KLF4	1.18E-37	0.62602348	0.909	0.598	2.36E-34	SMC5
CD83	2.20E-37	0.59469248	0.904	0.615	4.39E-34	SMC5
MARCKS	3.29E-37	0.6786417	0.99	0.711	6.59E-34	SMC5
SPARCL1	8.93E-37	0.46795553	0.962	0.615	1.79E-33	SMC5

DSTN	4.12E-36	0.68667892	0.99	0.701	8.23E-33	SMC5
A2M	7.85E-36	0.55394938	0.962	0.654	1.57E-32	SMC5
PPT1	8.94E-36	0.50267693	0.89	0.532	1.79E-32	SMC5
IER3	1.43E-35	0.83376179	0.933	0.675	2.85E-32	SMC5
IGFBP7	1.65E-35	0.45186819	1	0.766	3.30E-32	SMC5
TKT	1.66E-35	0.60028035	0.976	0.658	3.32E-32	SMC5
BLVRB	1.73E-35	0.70914497	0.9	0.611	3.47E-32	SMC5
RAB20	1.78E-35	0.58027505	0.789	0.492	3.57E-32	SMC5
GPR34	2.15E-35	0.52548016	0.77	0.478	4.29E-32	SMC5
ADAP2	2.31E-35	0.56362881	0.809	0.516	4.62E-32	SMC5
PYCARD	3.17E-35	0.57266303	0.938	0.682	6.34E-32	SMC5
SERPING1	5.50E-35	0.55949509	0.9	0.562	1.10E-31	SMC5
ATF3	6.78E-35	0.83707809	0.938	0.632	1.36E-31	SMC5
GSN	1.79E-34	0.75304752	0.99	0.648	3.58E-31	SMC5
TPM2	2.76E-34	0.88839171	0.799	0.51	5.53E-31	SMC5
FCGR1A	3.37E-34	0.81462281	0.785	0.485	6.73E-31	SMC5
CTSL	3.77E-34	0.91517392	0.89	0.619	7.53E-31	SMC5
CD86	1.91E-33	0.68543919	0.766	0.484	3.82E-30	SMC5
CLU	2.07E-33	0.92763839	0.833	0.521	4.14E-30	SMC5
VSIG4	2.70E-33	0.63855772	0.785	0.47	5.41E-30	SMC5
HTRA1	2.75E-33	0.39168194	0.904	0.587	5.50E-30	SMC5
IGFBP2	7.77E-33	0.51666135	0.847	0.567	1.55E-29	SMC5
LAP3	7.85E-33	0.57020645	0.943	0.621	1.57E-29	SMC5
PTPRE	8.87E-33	0.58386183	0.871	0.55	1.77E-29	SMC5
THBD	9.18E-33	0.75508252	0.852	0.554	1.84E-29	SMC5
FCGR2B	1.17E-32	0.55910177	0.794	0.573	2.34E-29	SMC5
ASAH1	1.20E-32	0.535295	0.923	0.593	2.41E-29	SMC5
CD151	2.08E-32	0.46824825	0.952	0.606	4.17E-29	SMC5
BGN	3.33E-32	0.41228329	0.842	0.546	6.65E-29	SMC5
TIMP1	1.03E-31	0.70673693	1	0.798	2.05E-28	SMC5
LILRB4	1.07E-31	0.54980617	0.756	0.43	2.14E-28	SMC5
MGST2	1.13E-31	0.48812381	0.856	0.545	2.26E-28	SMC5
SELENOM	1.21E-31	0.50537606	0.957	0.651	2.42E-28	SMC5
DBI	1.65E-31	0.68743985	0.971	0.756	3.30E-28	SMC5
LY86	1.70E-31	0.60028101	0.785	0.549	3.41E-28	SMC5
PALLD	1.86E-31	0.56652566	0.761	0.461	3.73E-28	SMC5
PLTP	1.94E-31	0.79793283	0.818	0.532	3.88E-28	SMC5
HMOX1	2.74E-31	0.85713648	0.813	0.536	5.48E-28	SMC5
CAVIN1	5.22E-31	0.40496518	0.876	0.546	1.04E-27	SMC5
JAML	5.56E-31	0.46426957	0.756	0.455	1.11E-27	SMC5
CAVIN3	1.04E-30	0.30154222	0.919	0.593	2.07E-27	SMC5
MS4A4A	1.25E-30	0.77771037	0.751	0.483	2.49E-27	SMC5
CAV1	1.38E-30	0.39885606	0.928	0.619	2.76E-27	SMC5
CTSD	1.76E-30	0.65609439	0.986	0.678	3.51E-27	SMC5
TPM1	1.85E-30	0.67073266	0.852	0.573	3.71E-27	SMC5
LGMN	2.53E-30	0.57344059	0.809	0.536	5.05E-27	SMC5
COL14A1	2.71E-30	0.65889934	0.761	0.474	5.41E-27	SMC5
IFITM3	2.73E-30	0.64677259	1	0.735	5.47E-27	SMC5
ARHGAP18	3.43E-30	0.45207551	0.919	0.651	6.86E-27	SMC5
SELENOP	3.94E-30	1.15035143	0.89	0.664	7.88E-27	SMC5
LTC4S	5.03E-30	0.40564227	0.828	0.555	1.01E-26	SMC5
CCL3	1.38E-29	1.34800589	0.823	0.621	2.76E-26	SMC5
SOD2	1.49E-29	0.64650862	0.909	0.601	2.98E-26	SMC5

MAP1B	1.61E-29	0.47598705	0.775	0.473	3.21E-26	SMC5
AP1S2	1.64E-29	0.53110832	0.876	0.562	3.28E-26	SMC5
FOLR2	2.35E-29	0.87540044	0.775	0.557	4.69E-26	SMC5
RGS2	2.36E-29	0.74507087	0.861	0.654	4.73E-26	SMC5
NNMT	2.84E-29	0.35203935	0.904	0.606	5.68E-26	SMC5
SOD3	2.87E-29	0.51705716	0.785	0.493	5.74E-26	SMC5
LTBP1	3.15E-29	0.46115266	0.699	0.383	6.31E-26	SMC5
CCDC88A	4.75E-29	0.4606758	0.828	0.537	9.50E-26	SMC5
RHOB	5.70E-29	0.70698545	0.871	0.573	1.14E-25	SMC5
CPM	6.13E-29	0.47724493	0.751	0.521	1.23E-25	SMC5
ATP6V1B2	6.68E-29	0.40642552	0.785	0.478	1.34E-25	SMC5
PLS3	9.08E-29	0.46578619	0.813	0.557	1.82E-25	SMC5
TAGLN	9.34E-29	0.87719212	0.785	0.547	1.87E-25	SMC5
IGFBP5	1.47E-28	0.43765566	0.818	0.561	2.94E-25	SMC5
PRRX1	1.59E-28	0.32083557	0.713	0.391	3.17E-25	SMC5
LMO2	1.60E-28	0.53883875	0.818	0.499	3.20E-25	SMC5
HBEGF	2.05E-28	0.65655307	0.742	0.483	4.11E-25	SMC5
CXCL2	2.27E-28	0.91508592	0.742	0.456	4.55E-25	SMC5
RAB32	2.78E-28	0.54151954	0.804	0.526	5.56E-25	SMC5
TGFBI	5.45E-28	0.38264338	0.775	0.529	1.09E-24	SMC5
CHMP1B	7.85E-28	0.63210701	0.837	0.548	1.57E-24	SMC5
CXorf21	8.05E-28	0.3766328	0.718	0.48	1.61E-24	SMC5
MFSD1	8.90E-28	0.49235294	0.813	0.513	1.78E-24	SMC5
COX7A1	1.02E-27	0.36063834	0.828	0.5	2.04E-24	SMC5
NINJ1	1.53E-27	0.45568551	0.833	0.512	3.06E-24	SMC5
KCTD12	1.72E-27	0.32546599	0.794	0.492	3.45E-24	SMC5
CLEC10A	2.22E-27	0.75643324	0.665	0.385	4.44E-24	SMC5
LMOD1	2.34E-27	0.62656999	0.636	0.32	4.67E-24	SMC5
NRP1	2.56E-27	0.25444201	0.871	0.554	5.12E-24	SMC5
LYN	3.06E-27	0.52674268	0.794	0.512	6.12E-24	SMC5
RAMP1	3.28E-27	0.55724496	0.67	0.42	6.55E-24	SMC5
NCF2	3.48E-27	0.48123558	0.713	0.372	6.96E-24	SMC5
SVIL	4.12E-27	0.41753536	0.785	0.491	8.23E-24	SMC5
EFEMP1	4.98E-27	0.4719208	0.761	0.457	9.96E-24	SMC5
DSC2	5.52E-27	0.34130992	0.679	0.362	1.10E-23	SMC5
EDIL3	5.60E-27	0.26875286	0.785	0.525	1.12E-23	SMC5
GK	5.98E-27	0.48689274	0.751	0.466	1.20E-23	SMC5
LHFPL6	7.08E-27	0.34162973	0.775	0.467	1.42E-23	SMC5
C1R	8.26E-27	0.32452599	0.789	0.505	1.65E-23	SMC5
CXCL8	8.48E-27	1.13274269	0.718	0.39	1.70E-23	SMC5
S100A13	1.02E-26	0.31922359	0.866	0.543	2.03E-23	SMC5
NUPR1	1.56E-26	0.41780222	0.789	0.485	3.12E-23	SMC5
FHL1	2.01E-26	0.67353544	0.77	0.503	4.03E-23	SMC5
VEGFA	2.26E-26	0.45837117	0.737	0.484	4.52E-23	SMC5
LPCAT2	2.98E-26	0.40625944	0.746	0.454	5.96E-23	SMC5
MFGE8	5.21E-26	0.33488546	0.775	0.493	1.04E-22	SMC5
VAMP8	5.29E-26	0.54844391	0.928	0.689	1.06E-22	SMC5
FN1	6.84E-26	0.84931029	0.813	0.576	1.37E-22	SMC5
OTUD1	7.11E-26	0.56481825	0.742	0.47	1.42E-22	SMC5
CTSC	7.54E-26	0.58054615	0.976	0.782	1.51E-22	SMC5
MXD1	1.55E-25	0.47618061	0.713	0.476	3.11E-22	SMC5
STAB1	1.76E-25	0.31258659	0.775	0.549	3.51E-22	SMC5
OGN	2.10E-25	0.73133332	0.679	0.418	4.19E-22	SMC5

GPR183	2.15E-25	0.81588221	0.885	0.701	4.30E-22	SMC5
RASGEF1B	2.32E-25	0.36235095	0.813	0.537	4.64E-22	SMC5
MYH10	2.60E-25	0.56491977	0.675	0.414	5.20E-22	SMC5
PHACTR1	2.93E-25	0.64688978	0.732	0.478	5.85E-22	SMC5
HSPA1A	3.66E-25	0.73509545	0.99	0.757	7.33E-22	SMC5
P2RY13	4.00E-25	0.39218225	0.708	0.454	8.00E-22	SMC5
C3AR1	6.00E-25	0.65167757	0.722	0.437	1.20E-21	SMC5
FCGR1B	6.98E-25	0.40270013	0.66	0.367	1.40E-21	SMC5
COL6A2	7.80E-25	0.3679085	0.818	0.573	1.56E-21	SMC5
NR4A3	8.50E-25	0.49465694	0.746	0.531	1.70E-21	SMC5
LINC01094	8.84E-25	0.37214104	0.675	0.39	1.77E-21	SMC5
CD300A	9.39E-25	0.31712539	0.651	0.322	1.88E-21	SMC5
NCF4	1.06E-24	0.42153655	0.785	0.597	2.12E-21	SMC5
CSTB	1.11E-24	0.59940189	0.957	0.73	2.22E-21	SMC5
MYH11	1.17E-24	0.87254629	0.641	0.383	2.35E-21	SMC5
CCL2	1.35E-24	1.22498436	0.813	0.536	2.70E-21	SMC5
CD9	1.41E-24	0.3803474	0.976	0.665	2.81E-21	SMC5
RASSF4	1.72E-24	0.46190199	0.737	0.455	3.45E-21	SMC5
RNASE6	2.00E-24	0.45801791	0.742	0.499	4.00E-21	SMC5
GCA	2.01E-24	0.44213923	0.775	0.511	4.02E-21	SMC5
SLC1A3	2.40E-24	0.34600858	0.665	0.344	4.81E-21	SMC5
HNMT	2.92E-24	0.47237286	0.785	0.559	5.83E-21	SMC5
C1orf162	3.61E-24	0.54510472	0.785	0.555	7.22E-21	SMC5
CDKN1A	3.67E-24	0.35560442	0.938	0.658	7.33E-21	SMC5
GADD45B	4.66E-24	0.53735791	0.981	0.747	9.31E-21	SMC5
MYLK	4.77E-24	0.4775392	0.689	0.433	9.54E-21	SMC5
ID3	4.86E-24	0.36249045	0.923	0.642	9.72E-21	SMC5
MSRB3	6.87E-24	0.30227708	0.694	0.41	1.37E-20	SMC5
BASP1	6.96E-24	0.64468763	0.761	0.536	1.39E-20	SMC5
RNF144B	7.43E-24	0.3277053	0.703	0.465	1.49E-20	SMC5
HCFC1R1	8.02E-24	0.41914354	0.837	0.561	1.60E-20	SMC5
INSIG1	9.16E-24	0.8234853	0.761	0.568	1.83E-20	SMC5
SHTN1	9.45E-24	0.29348117	0.713	0.448	1.89E-20	SMC5
FABP5	1.03E-23	0.79376585	0.871	0.656	2.05E-20	SMC5
VCAN	1.06E-23	0.50173668	0.809	0.532	2.11E-20	SMC5
ATP1B3	1.13E-23	0.53509574	0.923	0.687	2.25E-20	SMC5
TINAGL1	1.20E-23	0.2605486	0.785	0.504	2.41E-20	SMC5
LAIR1	1.29E-23	0.35337493	0.737	0.445	2.58E-20	SMC5
PLIN2	1.63E-23	0.58263182	0.909	0.678	3.25E-20	SMC5
PLBD1	1.93E-23	0.43750976	0.694	0.42	3.86E-20	SMC5
CSRP1	3.11E-23	0.50912258	0.789	0.534	6.22E-20	SMC5
HSPH1	3.64E-23	0.38773653	0.866	0.628	7.27E-20	SMC5
NR2F2	3.65E-23	0.30984831	0.852	0.611	7.30E-20	SMC5
SLC40A1	4.20E-23	0.68379088	0.761	0.512	8.41E-20	SMC5
IL1B	4.78E-23	1.01827854	0.675	0.403	9.57E-20	SMC5
LGALS3	5.25E-23	0.54530477	0.971	0.749	1.05E-19	SMC5
GPNMB	5.42E-23	0.49250135	0.727	0.447	1.08E-19	SMC5
BMP2K	6.94E-23	0.3444701	0.737	0.513	1.39E-19	SMC5
CD36	7.11E-23	0.47729984	0.722	0.466	1.42E-19	SMC5
JDP2	8.94E-23	0.38005479	0.742	0.516	1.79E-19	SMC5
MGLL	9.68E-23	0.28761677	0.78	0.508	1.94E-19	SMC5
TSC22D1	1.04E-22	0.32225504	0.876	0.635	2.09E-19	SMC5
CD4	1.20E-22	0.25065326	0.732	0.455	2.39E-19	SMC5

RNASET2	1.23E-22	0.40667538	0.938	0.686	2.46E-19	SMC5
NRP2	1.47E-22	0.33819424	0.794	0.532	2.95E-19	SMC5
EFHD2	2.36E-22	0.4271657	0.89	0.624	4.72E-19	SMC5
TGFB1I1	2.87E-22	0.36598752	0.694	0.428	5.74E-19	SMC5
MSR1	5.52E-22	0.61664121	0.656	0.331	1.10E-18	SMC5
CAPG	5.84E-22	0.39188648	0.799	0.527	1.17E-18	SMC5
NEXN	6.47E-22	0.53421002	0.67	0.421	1.29E-18	SMC5
DSE	7.55E-22	0.39965979	0.737	0.476	1.51E-18	SMC5
CPE	7.74E-22	0.30713867	0.751	0.524	1.55E-18	SMC5
RGS10	9.46E-22	0.33232416	0.928	0.634	1.89E-18	SMC5
RIPK2	1.15E-21	0.35001937	0.718	0.5	2.30E-18	SMC5
C1S	1.54E-21	0.27971696	0.703	0.431	3.08E-18	SMC5
TLR2	1.76E-21	0.46771716	0.679	0.417	3.52E-18	SMC5
AEBP1	2.68E-21	0.47622722	0.756	0.523	5.37E-18	SMC5
PPP1R14A	2.86E-21	0.74693688	0.646	0.441	5.73E-18	SMC5
LGALS9	3.18E-21	0.32885584	0.766	0.561	6.36E-18	SMC5
PDLIM3	3.50E-21	0.38341431	0.746	0.471	7.01E-18	SMC5
CH25H	3.66E-21	1.08531905	0.722	0.569	7.31E-18	SMC5
ACTA2	3.94E-21	0.89366737	0.703	0.502	7.89E-18	SMC5
PLD3	4.05E-21	0.44310909	0.794	0.499	8.10E-18	SMC5
OLR1	4.86E-21	0.69437714	0.689	0.499	9.71E-18	SMC5
AXL	5.87E-21	0.32559101	0.636	0.348	1.17E-17	SMC5
AQP1	1.01E-20	0.28426972	0.789	0.538	2.02E-17	SMC5
LRP1	1.05E-20	0.37132087	0.727	0.468	2.11E-17	SMC5
CSRP2	1.64E-20	0.36023856	0.675	0.415	3.29E-17	SMC5
FXYD1	2.20E-20	0.55781957	0.689	0.473	4.41E-17	SMC5
GUCY1A1	2.82E-20	0.42319433	0.694	0.471	5.64E-17	SMC5
PILRA	4.08E-20	0.47050951	0.67	0.411	8.17E-17	SMC5
LGALS1	4.37E-20	0.51600148	0.995	0.834	8.74E-17	SMC5
FRZB	5.16E-20	0.57005337	0.689	0.482	1.03E-16	SMC5
NFIL3	7.18E-20	0.33305943	0.78	0.573	1.44E-16	SMC5
LAT2	8.23E-20	0.26025216	0.665	0.428	1.65E-16	SMC5
SYAP1	8.70E-20	0.34897667	0.833	0.56	1.74E-16	SMC5
PMP22	1.10E-19	0.35199556	0.833	0.565	2.21E-16	SMC5
SSPN	1.27E-19	0.27998405	0.713	0.482	2.54E-16	SMC5
AC020656.1	1.49E-19	0.65707466	0.627	0.321	2.97E-16	SMC5
PDXK	1.65E-19	0.34130664	0.766	0.542	3.29E-16	SMC5
CXCL3	1.66E-19	0.81762971	0.646	0.406	3.31E-16	SMC5
SYNPO2	2.08E-19	0.33386315	0.579	0.364	4.16E-16	SMC5
PLEKHO1	2.10E-19	0.2772084	0.866	0.653	4.20E-16	SMC5
MFAP4	2.13E-19	0.61758444	0.608	0.372	4.26E-16	SMC5
C15orf48	2.17E-19	0.68995285	0.646	0.422	4.33E-16	SMC5
S100A6	2.76E-19	0.4766992	1	0.936	5.52E-16	SMC5
COL1A2	3.16E-19	0.25875976	0.746	0.528	6.32E-16	SMC5
NLRP3	4.48E-19	0.37348133	0.708	0.503	8.95E-16	SMC5
ITGA8	4.71E-19	0.60647849	0.622	0.405	9.42E-16	SMC5
BCL2A1	4.75E-19	0.45037076	0.751	0.514	9.50E-16	SMC5
AOAH	5.34E-19	0.26129986	0.737	0.511	1.07E-15	SMC5
ATP1B1	5.64E-19	0.36171913	0.713	0.512	1.13E-15	SMC5
HSPA1B	6.48E-19	0.3906483	0.904	0.664	1.30E-15	SMC5
CYBA	9.21E-19	0.45142977	1	0.907	1.84E-15	SMC5
TMEM47	9.76E-19	0.3072124	0.608	0.38	1.95E-15	SMC5
ADM	1.01E-18	0.28044755	0.746	0.549	2.01E-15	SMC5

CRYAB	1.09E-18	0.42995379	0.708	0.506	2.17E-15	SMC5
TUBA1A	1.10E-18	0.41347263	0.99	0.838	2.21E-15	SMC5
LILRB2	1.27E-18	0.33663774	0.656	0.403	2.53E-15	SMC5
PDE5A	1.36E-18	0.43979255	0.622	0.411	2.72E-15	SMC5
EMILIN2	1.37E-18	0.34750102	0.694	0.472	2.73E-15	SMC5
PAK1	1.37E-18	0.28756984	0.684	0.486	2.75E-15	SMC5
ITGB2	1.42E-18	0.29055789	0.947	0.721	2.84E-15	SMC5
HES4	1.50E-18	0.28888293	0.722	0.524	3.00E-15	SMC5
FPR3	3.32E-18	0.33678381	0.608	0.347	6.64E-15	SMC5
SOX4	4.46E-18	0.31432891	0.809	0.615	8.92E-15	SMC5
HAVCR2	4.81E-18	0.29640989	0.651	0.433	9.62E-15	SMC5
ZNF331	5.09E-18	0.49007835	0.871	0.67	1.02E-14	SMC5
ANXA2	5.10E-18	0.41732683	0.99	0.816	1.02E-14	SMC5
CTGF	5.19E-18	0.25950908	0.785	0.587	1.04E-14	SMC5
SULF1	1.32E-17	0.35314318	0.589	0.367	2.64E-14	SMC5
PDK4	1.37E-17	0.42322063	0.722	0.502	2.73E-14	SMC5
BHLHE41	1.41E-17	0.292817	0.67	0.44	2.83E-14	SMC5
PHLDA2	1.45E-17	0.28388823	0.785	0.623	2.90E-14	SMC5
COTL1	3.19E-17	0.42717448	0.933	0.763	6.38E-14	SMC5
FBLIM1	3.30E-17	0.34789955	0.656	0.429	6.61E-14	SMC5
SLC16A3	4.79E-17	0.28952346	0.708	0.533	9.57E-14	SMC5
PRELP	5.24E-17	0.27449856	0.622	0.374	1.05E-13	SMC5
MPEG1	5.89E-17	0.28803626	0.636	0.372	1.18E-13	SMC5
IL1RN	6.48E-17	0.90510929	0.589	0.357	1.30E-13	SMC5
EPB41L2	1.15E-16	0.27510838	0.742	0.533	2.30E-13	SMC5
HERPUD1	1.23E-16	0.49231924	0.981	0.839	2.46E-13	SMC5
GRASP	1.58E-16	0.30103647	0.775	0.576	3.17E-13	SMC5
MAFF	1.67E-16	0.26809911	0.761	0.545	3.34E-13	SMC5
PPA1	1.83E-16	0.27385147	0.9	0.663	3.66E-13	SMC5
CCL8	1.86E-16	0.8079838	0.651	0.482	3.73E-13	SMC5
EPB41L3	1.99E-16	0.35867102	0.665	0.407	3.98E-13	SMC5
TUBB6	2.23E-16	0.28495778	0.751	0.488	4.47E-13	SMC5
RARRES2	2.74E-16	0.31234136	0.699	0.522	5.49E-13	SMC5
FCER1A	2.88E-16	0.55615724	0.603	0.419	5.76E-13	SMC5
GEM	3.24E-16	0.52415104	0.718	0.59	6.48E-13	SMC5
FKBP1A	3.87E-16	0.32081499	0.909	0.641	7.74E-13	SMC5
TUBA1B	5.98E-16	0.40988499	0.995	0.887	1.20E-12	SMC5
RGS5	6.39E-16	0.54131763	0.617	0.453	1.28E-12	SMC5
TREM2	8.46E-16	0.4727332	0.646	0.504	1.69E-12	SMC5
PDLIM7	1.35E-15	0.29491803	0.684	0.465	2.70E-12	SMC5
ITGAX	1.43E-15	0.36159566	0.636	0.412	2.85E-12	SMC5
FMO2	1.49E-15	0.25180992	0.632	0.464	2.98E-12	SMC5
FCN1	1.78E-15	0.89484723	0.593	0.361	3.56E-12	SMC5
C1orf54	2.03E-15	0.27062423	0.746	0.528	4.05E-12	SMC5
PLN	2.24E-15	0.62603722	0.545	0.34	4.48E-12	SMC5
MARCH1	2.94E-15	0.31384802	0.67	0.488	5.89E-12	SMC5
EIF4A3	4.27E-15	0.33406104	0.9	0.701	8.54E-12	SMC5
LGALS2	4.59E-15	0.3124945	0.579	0.375	9.18E-12	SMC5
ANGPT1	6.25E-15	0.25881996	0.617	0.432	1.25E-11	SMC5
TBXAS1	6.70E-15	0.31636466	0.665	0.51	1.34E-11	SMC5
FPR1	7.36E-15	0.40555493	0.627	0.387	1.47E-11	SMC5
TGM2	1.93E-14	0.35383597	0.66	0.434	3.87E-11	SMC5
HCK	3.20E-14	0.34849317	0.646	0.472	6.40E-11	SMC5

NPL	4.37E-14	0.3402676	0.632	0.459	8.73E-11	SMC5
PPIF	5.20E-14	0.38318709	0.651	0.47	1.04E-10	SMC5
SLC8A1	5.42E-14	0.35911245	0.665	0.502	1.08E-10	SMC5
FBP1	5.91E-14	0.53323187	0.646	0.496	1.18E-10	SMC5
CCL3L1	6.13E-14	1.06227468	0.656	0.527	1.23E-10	SMC5
OLFML2B	6.81E-14	0.27695246	0.617	0.425	1.36E-10	SMC5
HSP90B1	6.87E-14	0.38287549	0.99	0.828	1.37E-10	SMC5
GLA	7.38E-14	0.31897338	0.651	0.475	1.48E-10	SMC5
MT1X	7.85E-14	0.46706802	0.732	0.542	1.57E-10	SMC5
PLAU	9.15E-14	0.40684096	0.675	0.546	1.83E-10	SMC5
PCDH7	9.33E-14	0.32504408	0.536	0.336	1.87E-10	SMC5
RBM47	1.09E-13	0.2521029	0.627	0.379	2.19E-10	SMC5
SNCG	1.21E-13	0.31253536	0.761	0.59	2.42E-10	SMC5
CREB5	1.58E-13	0.2648955	0.579	0.409	3.17E-10	SMC5
PROCR	2.67E-13	0.26279269	0.641	0.457	5.35E-10	SMC5
MT2A	2.94E-13	0.69418928	0.923	0.791	5.87E-10	SMC5
FUCA1	4.35E-13	0.28349207	0.656	0.495	8.70E-10	SMC5
NOV	4.43E-13	0.41545705	0.641	0.494	8.86E-10	SMC5
SLAMF8	1.01E-12	0.32730024	0.641	0.509	2.02E-09	SMC5
OGFRL1	1.24E-12	0.30523431	0.656	0.508	2.47E-09	SMC5
KCNMB1	1.41E-12	0.26625641	0.565	0.397	2.83E-09	SMC5
GMDS	1.72E-12	0.30360618	0.651	0.501	3.43E-09	SMC5
PID1	1.77E-12	0.32782771	0.593	0.388	3.55E-09	SMC5
KCNMA1	1.88E-12	0.39188581	0.608	0.426	3.76E-09	SMC5
VMO1	2.16E-12	0.5904059	0.612	0.47	4.31E-09	SMC5
C2	2.69E-12	0.28398667	0.622	0.466	5.37E-09	SMC5
BID	4.16E-12	0.30334175	0.722	0.578	8.31E-09	SMC5
LMCD1	4.36E-12	0.34284559	0.689	0.555	8.73E-09	SMC5
RCAN1	4.38E-12	0.28885386	0.646	0.493	8.77E-09	SMC5
GNA15	5.17E-12	0.25541981	0.584	0.406	1.03E-08	SMC5
MTSS1	5.51E-12	0.2779867	0.651	0.488	1.10E-08	SMC5
CSTA	6.02E-12	0.42246791	0.579	0.375	1.20E-08	SMC5
TFEC	9.02E-12	0.26756353	0.612	0.432	1.80E-08	SMC5
RNASE1	2.37E-11	0.50785347	0.761	0.62	4.74E-08	SMC5
B3GNT5	2.86E-11	0.31388629	0.598	0.47	5.73E-08	SMC5
SAP30	3.02E-11	0.28724413	0.761	0.61	6.04E-08	SMC5
AREG	3.63E-11	1.14500489	0.689	0.572	7.26E-08	SMC5
PTAFR	7.90E-11	0.25206725	0.565	0.371	1.58E-07	SMC5
TFRC	1.18E-10	0.53804894	0.646	0.499	2.37E-07	SMC5
TNFAIP2	1.21E-10	0.28206596	0.632	0.472	2.42E-07	SMC5
C2orf40	1.42E-10	0.48666409	0.727	0.588	2.83E-07	SMC5
ADH1B	1.56E-10	0.42681998	0.493	0.307	3.11E-07	SMC5
SLC11A1	4.76E-10	0.26486219	0.574	0.442	9.53E-07	SMC5
LIMCH1	6.03E-10	0.25536557	0.603	0.407	1.21E-06	SMC5
TREM1	7.62E-10	0.42145545	0.584	0.441	1.52E-06	SMC5
OSM	7.99E-10	0.36857793	0.565	0.411	1.60E-06	SMC5
MIR181A1H	1.26E-09	0.28755611	0.603	0.503	2.52E-06	SMC5
CFP	1.80E-09	0.26876814	0.569	0.445	3.59E-06	SMC5
ASPN	1.91E-09	0.30651587	0.536	0.384	3.82E-06	SMC5
APOE	2.25E-09	1.28816314	0.622	0.48	4.49E-06	SMC5
ACTB	2.42E-09	0.27083614	1	0.968	4.84E-06	SMC5
CYCS	3.50E-09	0.37562006	0.904	0.761	6.99E-06	SMC5
ANPEP	3.50E-09	0.29154559	0.579	0.472	6.99E-06	SMC5

SLC16A10	1.01E-08	0.32761458	0.56	0.422	2.03E-05	SMC5
ANTXR1	1.19E-08	0.25047308	0.536	0.407	2.39E-05	SMC5
C12orf75	1.48E-08	0.31657399	0.679	0.573	2.96E-05	SMC5
ACSL1	1.48E-08	0.25381167	0.555	0.433	2.97E-05	SMC5
S100A10	3.54E-08	0.26174211	1	0.913	7.09E-05	SMC5
ID4	4.44E-08	0.3363812	0.603	0.474	8.87E-05	SMC5
CCL4L2	4.45E-08	0.42849821	0.818	0.733	8.90E-05	SMC5
NRGN	4.88E-08	0.27828745	0.603	0.465	9.76E-05	SMC5
SLCO2B1	5.31E-08	0.27341999	0.612	0.521	0.00010625	SMC5
THBS1	5.51E-08	0.54185281	0.632	0.535	0.00011019	SMC5
CLEC4A	6.94E-08	0.31037556	0.569	0.44	0.00013876	SMC5
S100A8	5.73E-07	1.17733304	0.526	0.406	0.00114563	SMC5
ACP5	7.31E-07	0.32076948	0.56	0.507	0.00146136	SMC5
ENPP2	1.21E-06	0.25521618	0.565	0.433	0.00242804	SMC5
LILRB3	1.31E-06	0.25401688	0.536	0.347	0.00261989	SMC5
CCL21	1.78E-06	0.72890465	0.632	0.547	0.00356516	SMC5
MARCO	2.64E-06	0.27064193	0.522	0.428	0.00528184	SMC5
DEPP1	2.81E-06	0.39085024	0.488	0.377	0.00561545	SMC5
APOC1	4.20E-06	0.62343136	0.502	0.392	0.00839354	SMC5
ASGR1	5.32E-06	0.30502563	0.483	0.301	0.01064211	SMC5
G0S2	9.29E-06	0.99755515	0.493	0.41	0.01858984	SMC5
CD1C	1.14E-05	0.27150524	0.45	0.305	0.02275808	SMC5
KYNU	1.84E-05	0.25057254	0.555	0.44	0.03682324	SMC5
RGS5	0	4.425362	0.99	0.446	0	Pericyte
NDUFA4L2	0	3.67820007	0.954	0.437	0	Pericyte
APOE	0	3.34300756	0.927	0.474	0	Pericyte
STEAP4	0	2.78861863	0.935	0.554	0	Pericyte
THY1	0	2.78625015	0.949	0.579	0	Pericyte
LHFPL6	0	2.74405419	0.982	0.461	0	Pericyte
CPE	0	2.58542474	0.982	0.518	0	Pericyte
PLAC9	0	2.53979094	0.983	0.525	0	Pericyte
CCDC102B	0	2.53747902	0.956	0.487	0	Pericyte
IGFBP7	0	2.2271337	1	0.764	0	Pericyte
C11orf96	0	2.18918273	0.959	0.497	0	Pericyte
MYL9	0	2.12529765	0.99	0.474	0	Pericyte
CALD1	0	2.08245967	1	0.582	0	Pericyte
TFPI	0	2.0346178	0.969	0.561	0	Pericyte
ABCC9	0	1.99789519	0.871	0.331	0	Pericyte
PMEPA1	0	1.99535165	0.938	0.59	0	Pericyte
SPARC	0	1.99015117	0.973	0.627	0	Pericyte
NR2F2	0	1.94673775	0.967	0.607	0	Pericyte
PGF	0	1.93526206	0.897	0.51	0	Pericyte
SPET4	0	1.81497122	0.906	0.504	0	Pericyte
MAP1B	0	1.81010243	0.947	0.467	0	Pericyte
GUCY1B1	0	1.62406354	0.881	0.407	0	Pericyte
PDGFRB	0	1.6109583	0.885	0.385	0	Pericyte
HIGD1B	4.42E-307	1.1957626	0.762	0.297	8.84E-304	Pericyte
PCOLCE	5.91E-307	1.69397311	0.938	0.522	1.18E-303	Pericyte
SOD3	2.23E-303	1.85636831	0.96	0.487	4.47E-300	Pericyte
UACA	2.23E-302	1.82304334	0.937	0.578	4.47E-299	Pericyte
FRZB	4.84E-298	1.63968297	0.929	0.476	9.67E-295	Pericyte
PRRX1	1.78E-296	1.5382545	0.895	0.385	3.56E-293	Pericyte
RGS16	3.18E-294	2.1589631	0.927	0.642	6.37E-291	Pericyte

IFITM3	5.99E-293	1.63885231	0.999	0.732	1.20E-289	Pericyte
SPARCL1	8.92E-292	2.10897008	0.991	0.611	1.78E-288	Pericyte
TPM2	2.29E-291	1.55716971	0.978	0.504	4.57E-288	Pericyte
GUCY1A1	2.11E-290	1.63062172	0.908	0.465	4.22E-287	Pericyte
ADIRF	2.70E-290	1.80442352	0.985	0.568	5.39E-287	Pericyte
TINAGL1	3.22E-286	1.64047886	0.917	0.499	6.44E-283	Pericyte
SERPING1	2.60E-283	1.56806539	0.967	0.557	5.20E-280	Pericyte
SMOC2	9.54E-283	1.53001119	0.888	0.427	1.91E-279	Pericyte
NOTCH3	9.12E-280	1.63675326	0.841	0.38	1.82E-276	Pericyte
TAGLN	3.51E-278	1.61868119	0.982	0.541	7.01E-275	Pericyte
ARHGAP29	1.02E-276	1.60365704	0.929	0.57	2.03E-273	Pericyte
COX4I2	2.20E-275	2.02319596	0.776	0.257	4.40E-272	Pericyte
CAVIN3	1.76E-271	1.63362023	0.958	0.588	3.51E-268	Pericyte
CSRP2	2.53E-270	1.49516875	0.873	0.409	5.06E-267	Pericyte
CYGB	1.08E-267	1.1179901	0.776	0.357	2.17E-264	Pericyte
KCNE4	8.65E-266	1.56998282	0.828	0.45	1.73E-262	Pericyte
IGFBP4	4.06E-261	1.70421986	0.938	0.602	8.11E-258	Pericyte
TPM1	3.74E-259	1.6299465	0.963	0.568	7.48E-256	Pericyte
BGN	1.05E-254	1.27924059	0.978	0.541	2.10E-251	Pericyte
COL6A2	7.02E-252	1.54625232	0.922	0.569	1.40E-248	Pericyte
COL18A1	3.20E-251	1.74848541	0.892	0.522	6.41E-248	Pericyte
FILIP1L	3.61E-245	1.81352053	0.917	0.549	7.22E-242	Pericyte
TGFB1I1	4.50E-244	1.34448007	0.865	0.422	8.99E-241	Pericyte
TIMP1	2.31E-236	1.32770256	0.996	0.796	4.62E-233	Pericyte
MT2A	1.58E-235	1.69021368	0.986	0.788	3.16E-232	Pericyte
SERPINF1	1.33E-232	1.46810126	0.853	0.545	2.66E-229	Pericyte
TDO2	7.86E-231	0.8894016	0.763	0.491	1.57E-227	Pericyte
C1R	7.97E-231	1.27121693	0.922	0.5	1.59E-227	Pericyte
SELENOM	2.73E-229	1.33358207	0.965	0.647	5.45E-226	Pericyte
ACTA2	5.44E-228	1.82513935	0.906	0.497	1.09E-224	Pericyte
CYR61	2.30E-227	1.62877601	0.923	0.564	4.59E-224	Pericyte
PTGER3	5.64E-222	0.77143514	0.729	0.329	1.13E-218	Pericyte
FAM162B	4.77E-220	1.17200441	0.738	0.362	9.54E-217	Pericyte
COL1A2	7.01E-218	0.82617703	0.9	0.523	1.40E-214	Pericyte
LPL	8.79E-218	0.52349831	0.696	0.318	1.76E-214	Pericyte
EGR1	6.74E-217	1.68416375	0.896	0.585	1.35E-213	Pericyte
ID4	1.49E-216	1.84113308	0.836	0.469	2.98E-213	Pericyte
COL4A2	6.43E-215	1.38327493	0.871	0.526	1.29E-211	Pericyte
COL3A1	4.47E-213	1.00374556	0.862	0.511	8.94E-210	Pericyte
CAMK2N1	8.53E-213	1.35301249	0.785	0.444	1.71E-209	Pericyte
TBX2	1.06E-211	1.35386154	0.758	0.338	2.13E-208	Pericyte
PLXDC1	3.85E-206	1.14109305	0.767	0.496	7.69E-203	Pericyte
COL14A1	3.07E-202	1.22378963	0.879	0.469	6.15E-199	Pericyte
HES4	1.89E-199	1.39977624	0.821	0.52	3.79E-196	Pericyte
ANGPT2	2.32E-199	1.50504991	0.806	0.532	4.63E-196	Pericyte
LGALS1	1.08E-198	1.22570526	0.994	0.833	2.16E-195	Pericyte
MGLL	1.60E-197	1.35196067	0.832	0.504	3.20E-194	Pericyte
GGT5	3.15E-197	1.27243105	0.803	0.526	6.29E-194	Pericyte
AGT	6.05E-197	1.73916071	0.767	0.401	1.21E-193	Pericyte
RARRES2	6.02E-193	1.27055052	0.841	0.517	1.20E-189	Pericyte
PRRX2	8.57E-193	0.69231469	0.753	0.383	1.71E-189	Pericyte
MGP	1.25E-192	0.3519065	0.996	0.728	2.50E-189	Pericyte
TBX2-AS1	4.33E-191	0.76500256	0.672	0.197	8.66E-188	Pericyte

LGALS3BP	6.49E-191	1.03698683	0.851	0.559	1.30E-187	Pericyte
CAV1	4.58E-187	1.11131591	0.941	0.615	9.15E-184	Pericyte
COL6A3	8.31E-185	1.14451896	0.776	0.491	1.66E-181	Pericyte
TIMP3	9.41E-183	1.34115405	0.829	0.512	1.88E-179	Pericyte
GJA4	8.14E-182	1.30997371	0.751	0.386	1.63E-178	Pericyte
MYLK	4.15E-181	1.24855633	0.796	0.428	8.30E-178	Pericyte
MYO1B	1.45E-180	1.24501683	0.785	0.508	2.91E-177	Pericyte
A2M	4.36E-180	1.03266943	0.951	0.65	8.72E-177	Pericyte
CFH	1.63E-178	1.37401766	0.874	0.552	3.27E-175	Pericyte
CTGF	8.44E-178	1.03975203	0.904	0.582	1.69E-174	Pericyte
COL6A1	1.23E-175	1.04218196	0.821	0.474	2.46E-172	Pericyte
GPM6B	1.42E-175	0.68782434	0.747	0.462	2.85E-172	Pericyte
COL4A1	1.79E-174	1.35144816	0.842	0.543	3.59E-171	Pericyte
SGCE	2.93E-172	0.83790657	0.745	0.404	5.85E-169	Pericyte
JUN	1.04E-171	1.52936924	0.974	0.811	2.08E-168	Pericyte
DSTN	3.34E-170	0.86535567	0.977	0.698	6.67E-167	Pericyte
GEM	4.89E-167	1.4708904	0.819	0.587	9.78E-164	Pericyte
GUCY1A2	1.17E-166	0.66568908	0.659	0.286	2.34E-163	Pericyte
LAMA4	3.56E-166	1.00433359	0.815	0.571	7.13E-163	Pericyte
AVPR1A	5.51E-166	0.3514654	0.585	0.183	1.10E-162	Pericyte
CARMN	1.19E-161	0.94037837	0.723	0.335	2.38E-158	Pericyte
FAM13C	3.82E-160	1.08033263	0.723	0.4	7.64E-157	Pericyte
MFGE8	5.48E-160	0.79498072	0.855	0.489	1.10E-156	Pericyte
COX7A1	9.70E-159	1.09186612	0.819	0.497	1.94E-155	Pericyte
COL1A1	1.59E-157	0.68688365	0.768	0.463	3.18E-154	Pericyte
C1S	2.76E-157	1.03655241	0.787	0.427	5.52E-154	Pericyte
DLX5	7.27E-157	0.66262842	0.671	0.308	1.45E-153	Pericyte
FOXS1	5.35E-153	0.81257964	0.681	0.369	1.07E-149	Pericyte
ISYNA1	2.26E-151	1.25074098	0.74	0.472	4.53E-148	Pericyte
NNMT	3.87E-149	0.96024074	0.891	0.603	7.75E-146	Pericyte
TGFBI	1.71E-148	0.81224377	0.806	0.525	3.42E-145	Pericyte
PHLDA1	1.59E-147	1.24788471	0.841	0.618	3.19E-144	Pericyte
TPPP3	1.63E-147	1.22690279	0.697	0.372	3.26E-144	Pericyte
CD36	1.63E-146	1.2698992	0.759	0.463	3.27E-143	Pericyte
SFTA1P	1.31E-144	0.31121148	0.635	0.274	2.63E-141	Pericyte
RBP5	3.02E-144	0.43337205	0.663	0.367	6.04E-141	Pericyte
HGF	1.27E-141	0.97084584	0.735	0.452	2.55E-138	Pericyte
KLHL23	1.64E-141	0.72177435	0.667	0.405	3.28E-138	Pericyte
KCNK17	6.62E-140	0.52946335	0.649	0.298	1.32E-136	Pericyte
CNN3	1.30E-138	1.0174599	0.818	0.563	2.60E-135	Pericyte
FN1	2.03E-138	0.43850404	0.899	0.572	4.06E-135	Pericyte
ADGRF5	1.22E-137	0.75554653	0.749	0.544	2.43E-134	Pericyte
IFIT3	5.80E-137	0.38310306	0.729	0.457	1.16E-133	Pericyte
SPTBN1	2.92E-136	0.78911769	0.829	0.505	5.83E-133	Pericyte
CD9	5.84E-136	0.92074751	0.953	0.662	1.17E-132	Pericyte
IL34	8.20E-133	0.76633205	0.74	0.563	1.64E-129	Pericyte
DKK3	1.84E-132	0.76193637	0.777	0.457	3.69E-129	Pericyte
PRKG1	6.19E-131	0.83193265	0.695	0.345	1.24E-127	Pericyte
CD248	5.85E-127	0.4796204	0.631	0.307	1.17E-123	Pericyte
PPP1R14A	1.91E-126	0.90347783	0.762	0.437	3.82E-123	Pericyte
SIX1	5.77E-125	0.2935015	0.613	0.304	1.15E-121	Pericyte
MYC	6.83E-125	1.11028678	0.741	0.516	1.37E-121	Pericyte
PDLIM1	1.04E-124	0.81211941	0.851	0.616	2.09E-121	Pericyte

LGALS3	2.32E-124	0.799368	0.971	0.746	4.65E-121	Pericyte
SERPINH1	1.17E-122	0.77244435	0.804	0.571	2.35E-119	Pericyte
TWIST1	2.47E-122	0.63430517	0.691	0.394	4.94E-119	Pericyte
COLEC11	2.34E-121	0.27426917	0.562	0.194	4.68E-118	Pericyte
SCIN	3.20E-120	0.42276914	0.621	0.329	6.40E-117	Pericyte
CD59	7.52E-120	0.78546084	0.872	0.578	1.50E-116	Pericyte
PAWR	4.43E-119	0.93369428	0.732	0.492	8.85E-116	Pericyte
GSN	5.08E-119	0.62655698	0.947	0.645	1.02E-115	Pericyte
CAV2	2.69E-118	0.97116843	0.765	0.473	5.39E-115	Pericyte
KCNMB1	3.87E-118	0.63105005	0.69	0.393	7.74E-115	Pericyte
CCL21	1.02E-117	2.80244347	0.705	0.545	2.03E-114	Pericyte
TNC	1.89E-116	0.79853954	0.722	0.507	3.79E-113	Pericyte
PLS3	2.34E-115	0.81487563	0.799	0.554	4.69E-112	Pericyte
CCL19	2.66E-115	4.01499191	0.747	0.669	5.32E-112	Pericyte
PTP4A3	3.12E-115	1.23448859	0.706	0.486	6.25E-112	Pericyte
NBL1	8.19E-115	0.86454867	0.794	0.574	1.64E-111	Pericyte
PDGFA	8.83E-115	1.06947514	0.75	0.534	1.77E-111	Pericyte
ITGA1	5.08E-114	0.88321823	0.71	0.483	1.02E-110	Pericyte
RBP1	3.37E-113	0.55474042	0.679	0.417	6.74E-110	Pericyte
MRGPRF	2.65E-111	0.52768699	0.585	0.171	5.30E-108	Pericyte
IFIT2	1.18E-110	0.38766942	0.688	0.457	2.36E-107	Pericyte
IGFBP2	3.08E-110	0.41749354	0.821	0.564	6.15E-107	Pericyte
MT1E	1.16E-109	1.23520006	0.764	0.581	2.31E-106	Pericyte
MCAM	4.36E-108	1.22262669	0.718	0.479	8.73E-105	Pericyte
ANGPT1	7.48E-107	0.91739873	0.688	0.429	1.50E-103	Pericyte
IGFBP5	3.64E-106	1.48117465	0.765	0.559	7.28E-103	Pericyte
C2orf40	5.76E-106	1.05167731	0.753	0.586	1.15E-102	Pericyte
VASN	1.91E-104	0.83803296	0.672	0.416	3.82E-101	Pericyte
NUPR1	3.26E-104	0.716496	0.777	0.482	6.52E-101	Pericyte
COL5A2	4.51E-103	0.52899067	0.685	0.457	9.03E-100	Pericyte
EGFLAM	1.50E-102	0.42860211	0.603	0.279	3.00E-99	Pericyte
BATF3	1.16E-101	0.44251901	0.672	0.479	2.31E-98	Pericyte
HCFC1R1	2.36E-99	0.7995492	0.796	0.558	4.72E-96	Pericyte
CD151	2.36E-99	0.70557002	0.855	0.604	4.73E-96	Pericyte
FBLIM1	3.59E-99	0.71757532	0.699	0.426	7.18E-96	Pericyte
MIR4435-2H	1.14E-97	0.56149858	0.751	0.552	2.29E-94	Pericyte
CST3	1.70E-97	0.48675926	0.999	0.733	3.41E-94	Pericyte
NRP1	3.36E-97	0.7180217	0.786	0.552	6.73E-94	Pericyte
SYNPO2	8.41E-96	0.94522573	0.642	0.36	1.68E-92	Pericyte
CDH6	2.93E-95	0.70829164	0.572	0.212	5.86E-92	Pericyte
SEMA5A	1.59E-94	0.75154274	0.603	0.308	3.17E-91	Pericyte
MMP11	1.52E-93	0.41705898	0.635	0.453	3.04E-90	Pericyte
RERG	4.02E-93	0.7063719	0.608	0.267	8.04E-90	Pericyte
RASL11A	1.93E-92	0.82792245	0.663	0.485	3.86E-89	Pericyte
TMEM98	5.18E-92	0.45161628	0.635	0.362	1.04E-88	Pericyte
CRYAB	1.03E-91	0.67006733	0.758	0.503	2.05E-88	Pericyte
RERGL	1.54E-89	0.56001313	0.6	0.353	3.09E-86	Pericyte
IL1R1	1.56E-89	0.43905035	0.686	0.437	3.12E-86	Pericyte
MAP9	2.98E-88	0.58924489	0.631	0.373	5.96E-85	Pericyte
PALLD	7.55E-87	0.84005898	0.705	0.459	1.51E-83	Pericyte
ID3	1.39E-86	0.93870801	0.835	0.64	2.78E-83	Pericyte
HOPX	1.39E-86	0.41888259	0.737	0.599	2.79E-83	Pericyte
LINC01615	2.06E-85	0.40787306	0.651	0.457	4.11E-82	Pericyte

PPIC	6.84E-85	0.43925262	0.759	0.548	1.37E-81	Pericyte
CRIP2	1.62E-84	0.6557537	0.824	0.613	3.24E-81	Pericyte
GPX3	1.23E-83	0.7253165	0.676	0.449	2.45E-80	Pericyte
GPRC5C	1.53E-83	0.82291637	0.649	0.403	3.05E-80	Pericyte
ERRFI1	1.98E-83	0.38277178	0.644	0.452	3.95E-80	Pericyte
CCND1	1.38E-82	0.76692486	0.758	0.609	2.77E-79	Pericyte
MAP3K7CL	1.81E-82	0.80459726	0.612	0.4	3.62E-79	Pericyte
TSC22D1	1.34E-81	0.79418936	0.803	0.633	2.68E-78	Pericyte
S100A4	2.59E-81	0.77690258	0.986	0.893	5.19E-78	Pericyte
PRDX4	2.74E-81	0.47372664	0.853	0.649	5.48E-78	Pericyte
SSPN	6.27E-81	0.60356216	0.692	0.48	1.25E-77	Pericyte
CAVIN1	1.75E-80	0.57865867	0.785	0.544	3.50E-77	Pericyte
EFEMP2	7.35E-80	0.58304869	0.682	0.448	1.47E-76	Pericyte
GSTM3	1.14E-79	0.6609651	0.723	0.617	2.27E-76	Pericyte
S100A6	7.85E-79	0.49498852	0.995	0.936	1.57E-75	Pericyte
PTGER1	9.00E-78	0.28500241	0.495	0.145	1.80E-74	Pericyte
SLC7A2	9.33E-78	0.81569623	0.601	0.32	1.87E-74	Pericyte
ECM1	3.73E-77	0.41994208	0.654	0.504	7.46E-74	Pericyte
SERPINI1	1.04E-75	0.71324436	0.636	0.488	2.09E-72	Pericyte
C1QTNF1	2.92E-75	0.82831841	0.629	0.361	5.83E-72	Pericyte
EDIL3	1.51E-74	0.43261866	0.729	0.523	3.02E-71	Pericyte
PMP22	2.80E-74	0.57748438	0.776	0.563	5.60E-71	Pericyte
RASL12	8.90E-74	0.55325523	0.563	0.266	1.78E-70	Pericyte
NT5DC2	3.74E-73	0.89381176	0.631	0.45	7.49E-70	Pericyte
RND3	5.09E-73	0.48742268	0.676	0.467	1.02E-69	Pericyte
CRISPLD2	4.24E-72	0.66342599	0.551	0.295	8.49E-69	Pericyte
SRPX	7.21E-72	0.45396658	0.605	0.393	1.44E-68	Pericyte
SERPINE1	8.87E-72	0.30589068	0.636	0.415	1.77E-68	Pericyte
FILIP1	2.46E-71	0.77563493	0.647	0.424	4.92E-68	Pericyte
AKR1C3	2.27E-70	0.34165742	0.721	0.534	4.55E-67	Pericyte
EPB41L2	1.34E-69	0.76985113	0.712	0.531	2.68E-66	Pericyte
FEZ1	1.49E-68	0.49082039	0.621	0.418	2.97E-65	Pericyte
SOX4	2.58E-68	0.65281954	0.769	0.614	5.15E-65	Pericyte
LBH	6.69E-68	0.78464326	0.799	0.654	1.34E-64	Pericyte
ADAMTS1	9.03E-68	0.79097124	0.686	0.483	1.81E-64	Pericyte
HNMT	9.76E-68	0.35175345	0.744	0.557	1.95E-64	Pericyte
ATF3	3.55E-67	0.68941951	0.814	0.63	7.11E-64	Pericyte
C9orf3	2.47E-66	0.62530843	0.679	0.488	4.93E-63	Pericyte
PDLIM3	2.13E-65	0.72536531	0.687	0.469	4.26E-62	Pericyte
PDE5A	3.27E-65	0.65855606	0.635	0.408	6.55E-62	Pericyte
AXL	2.69E-64	0.65755519	0.604	0.345	5.38E-61	Pericyte
RCAN2	7.10E-64	0.84938767	0.674	0.561	1.42E-60	Pericyte
FHL1	1.02E-63	0.47658955	0.704	0.501	2.04E-60	Pericyte
ASPN	5.13E-63	0.63073138	0.615	0.381	1.03E-59	Pericyte
SLC40A1	1.45E-62	0.34038542	0.695	0.51	2.89E-59	Pericyte
S100A3	1.90E-62	0.33840922	0.601	0.399	3.80E-59	Pericyte
TMEM204	1.94E-61	0.34498329	0.688	0.552	3.87E-58	Pericyte
MT1M	6.26E-60	0.87715664	0.599	0.366	1.25E-56	Pericyte
PTN	6.73E-60	1.00876328	0.613	0.382	1.35E-56	Pericyte
EDNRB	9.13E-60	0.5810394	0.59	0.398	1.83E-56	Pericyte
CLEC11A	5.18E-59	0.58322033	0.619	0.461	1.04E-55	Pericyte
LMOD1	7.08E-59	0.52786053	0.617	0.317	1.42E-55	Pericyte
LNP1	8.23E-59	0.25125247	0.545	0.341	1.65E-55	Pericyte

HES1	9.88E-59	0.69804157	0.683	0.523	1.98E-55	Pericyte
APOLD1	4.97E-58	0.36764326	0.612	0.438	9.94E-55	Pericyte
VCAM1	9.07E-58	0.63409379	0.663	0.535	1.81E-54	Pericyte
FXYD1	1.38E-57	0.58462259	0.658	0.471	2.75E-54	Pericyte
FXYD6	1.41E-57	0.54743051	0.604	0.429	2.83E-54	Pericyte
FOXC2	2.32E-57	0.54423971	0.55	0.296	4.64E-54	Pericyte
FMO2	3.28E-57	0.46133623	0.631	0.462	6.56E-54	Pericyte
NCKAP5	9.24E-57	0.48512717	0.556	0.376	1.85E-53	Pericyte
CD200	1.94E-55	0.5136308	0.682	0.55	3.87E-52	Pericyte
FKBP10	3.54E-55	0.38496549	0.603	0.408	7.08E-52	Pericyte
FAT1	1.61E-53	0.57689905	0.567	0.35	3.21E-50	Pericyte
OR51E1	1.63E-53	0.33096049	0.487	0.247	3.27E-50	Pericyte
ZNF503	8.54E-53	0.51790599	0.65	0.483	1.71E-49	Pericyte
CCDC80	1.00E-52	0.52792339	0.687	0.559	2.01E-49	Pericyte
EDNRA	2.79E-52	0.7254102	0.576	0.382	5.57E-49	Pericyte
LRRC17	8.46E-52	0.56676802	0.588	0.385	1.69E-48	Pericyte
CYTOR	2.35E-50	0.43566803	0.813	0.712	4.69E-47	Pericyte
CPM	2.70E-50	0.31055893	0.655	0.52	5.40E-47	Pericyte
SDC2	3.47E-48	0.46470098	0.617	0.42	6.94E-45	Pericyte
ESAM	1.59E-47	0.59468995	0.637	0.489	3.19E-44	Pericyte
EGFL6	1.72E-47	0.25644316	0.526	0.337	3.44E-44	Pericyte
CTSF	1.89E-47	0.3451583	0.615	0.449	3.78E-44	Pericyte
HSP90B1	3.89E-47	0.43619678	0.971	0.826	7.79E-44	Pericyte
GAS6	1.09E-46	0.45025299	0.756	0.621	2.18E-43	Pericyte
PLK2	1.77E-46	0.29583586	0.65	0.519	3.55E-43	Pericyte
RBPMS2	3.39E-46	0.25548104	0.44	0.188	6.77E-43	Pericyte
NRGN	5.91E-46	0.61242154	0.636	0.463	1.18E-42	Pericyte
NTRK2	9.47E-46	0.63408105	0.567	0.425	1.89E-42	Pericyte
PRSS23	2.19E-45	0.29098874	0.696	0.563	4.38E-42	Pericyte
C7	2.35E-45	0.90630045	0.626	0.548	4.70E-42	Pericyte
TUBA1A	6.96E-44	0.49848889	0.949	0.837	1.39E-40	Pericyte
C1orf54	3.43E-42	0.53502252	0.662	0.526	6.86E-39	Pericyte
FAM241A	3.53E-41	0.37312089	0.59	0.46	7.06E-38	Pericyte
FKBP2	9.21E-41	0.34556358	0.883	0.726	1.84E-37	Pericyte
OLFML2B	2.81E-40	0.57001109	0.576	0.423	5.61E-37	Pericyte
CEBPD	1.40E-39	0.32391216	0.872	0.696	2.81E-36	Pericyte
CSRP1	1.48E-39	0.55806402	0.679	0.533	2.96E-36	Pericyte
PLN	1.71E-39	0.54152541	0.555	0.337	3.42E-36	Pericyte
CCL2	4.52E-39	0.90705137	0.681	0.534	9.04E-36	Pericyte
DIO2	5.88E-39	0.44514407	0.521	0.363	1.18E-35	Pericyte
DNAJB4	5.14E-38	0.53618437	0.638	0.539	1.03E-34	Pericyte
NET1	1.20E-36	0.48310917	0.588	0.439	2.40E-33	Pericyte
NFIB	1.68E-36	0.48006109	0.606	0.419	3.36E-33	Pericyte
PHLDA2	2.15E-36	0.61038024	0.679	0.623	4.30E-33	Pericyte
KRT18	5.72E-36	0.48932987	0.609	0.564	1.14E-32	Pericyte
RCN3	2.84E-34	0.27420926	0.562	0.421	5.67E-31	Pericyte
SLIT3	1.47E-33	0.61212723	0.512	0.306	2.95E-30	Pericyte
MTUS1	2.82E-33	0.28499185	0.61	0.481	5.64E-30	Pericyte
EFHD1	1.40E-32	0.60696309	0.523	0.33	2.80E-29	Pericyte
SGCA	3.82E-32	0.30788574	0.577	0.439	7.63E-29	Pericyte
ECM2	1.20E-30	0.3707541	0.496	0.324	2.40E-27	Pericyte
MSRB3	1.41E-29	0.47707706	0.569	0.409	2.83E-26	Pericyte
CYBRD1	2.76E-27	0.25543074	0.568	0.449	5.52E-24	Pericyte

LTBP4	9.05E-27	0.30176008	0.577	0.454	1.81E-23	Pericyte
GBP1	1.50E-25	0.29877548	0.642	0.543	3.00E-22	Pericyte
MT1A	6.78E-25	0.63111332	0.487	0.365	1.36E-21	Pericyte
SNCG	1.11E-24	0.52734719	0.638	0.589	2.22E-21	Pericyte
FGF7	2.37E-24	0.83470966	0.562	0.534	4.75E-21	Pericyte
RHOB	5.66E-22	0.31050992	0.679	0.573	1.13E-18	Pericyte
FSTL1	5.86E-22	0.27434975	0.551	0.437	1.17E-18	Pericyte
EMILIN1	1.04E-21	0.36877245	0.504	0.391	2.08E-18	Pericyte
PLPP1	7.16E-21	0.35093342	0.6	0.56	1.43E-17	Pericyte
ENAH	1.56E-20	0.51741937	0.549	0.441	3.12E-17	Pericyte
CSF1	1.66E-20	0.37453977	0.54	0.484	3.31E-17	Pericyte
ERLEC1	2.15E-20	0.25400033	0.692	0.616	4.29E-17	Pericyte
BCAM	1.01E-19	0.44660432	0.551	0.468	2.02E-16	Pericyte
NUDT4	1.08E-19	0.38744585	0.569	0.494	2.15E-16	Pericyte
MDK	2.75E-19	0.49374935	0.582	0.547	5.50E-16	Pericyte
TUBB6	1.54E-18	0.26588853	0.595	0.487	3.09E-15	Pericyte
ADAMTS4	6.53E-18	0.38434234	0.478	0.393	1.31E-14	Pericyte
ACTG2	6.75E-18	0.25729665	0.21	0.255	1.35E-14	Pericyte
TMEM47	7.47E-18	0.48494507	0.505	0.379	1.49E-14	Pericyte
FHL5	1.61E-17	0.30990915	0.418	0.225	3.21E-14	Pericyte
ANGPTL4	2.45E-17	0.31417983	0.492	0.388	4.90E-14	Pericyte
DEPP1	2.71E-16	0.64626043	0.476	0.376	5.43E-13	Pericyte
COL12A1	1.23E-15	0.40727531	0.501	0.426	2.46E-12	Pericyte
NGF	1.46E-14	0.25859035	0.41	0.164	2.92E-11	Pericyte
TCIM	1.59E-14	0.47533275	0.501	0.437	3.17E-11	Pericyte
PROCR	5.11E-13	0.52825427	0.513	0.457	1.02E-09	Pericyte
NEXN	1.41E-12	0.45347767	0.513	0.42	2.81E-09	Pericyte
AKAP12	1.02E-11	0.44445763	0.491	0.425	2.04E-08	Pericyte
ITIH5	8.64E-11	0.29016663	0.447	0.35	1.73E-07	Pericyte
LGALSL	2.00E-10	0.50137732	0.503	0.47	4.00E-07	Pericyte
IL33	2.17E-10	0.29850338	0.481	0.419	4.34E-07	Pericyte
NES	4.11E-09	0.30208174	0.494	0.423	8.23E-06	Pericyte
IER5L	3.55E-08	0.32895598	0.655	0.665	7.10E-05	Pericyte
LINC00924	3.78E-08	0.29099657	0.41	0.323	7.56E-05	Pericyte
RASD1	3.97E-08	0.38583198	0.46	0.432	7.93E-05	Pericyte
TNFRSF12A	1.02E-07	0.41011282	0.568	0.565	0.00020382	Pericyte
MAP1A	2.25E-06	0.31055108	0.46	0.437	0.00450421	Pericyte
CXCL9	6.08E-06	0.28173483	0.547	0.546	0.01216033	Pericyte
ADRA2A	2.01E-05	0.28405581	0.431	0.404	0.04017714	Pericyte

Table S3. Markers for cluster annotation in human ascending aorta (GSE216860)

gene	p_val	avg_log2FC	pct.1	pct.2	p_val_adj	cluster
ACKR1	0	4.13250228	0.885	0.867	0	EC
VWF	0	3.79798692	0.942	0.819	0	EC
CLDN5	0	3.3793367	0.906	0.817	0	EC
AQP1	0	3.17963032	0.905	0.807	0	EC
LIFR	0	3.09755806	0.937	0.831	0	EC
IFI27	0	3.06549685	0.94	0.713	0	EC
PECAM1	0	3.06543132	0.942	0.802	0	EC
RAMP3	0	2.93641849	0.882	0.795	0	EC
ID1	0	2.92150884	0.892	0.735	0	EC
PLVAP	0	2.88448974	0.859	0.746	0	EC
FABP4	0	2.86472544	0.953	0.907	0	EC
POSTN	0	2.79054335	0.758	0.65	0	EC
SLC9A3R2	0	2.68262347	0.887	0.737	0	EC
CCL14	0	2.66336737	0.831	0.796	0	EC
ADGRL4	0	2.65283839	0.85	0.751	0	EC
RBP7	0	2.56901492	0.873	0.841	0	EC
CAVIN2	0	2.53725038	0.866	0.623	0	EC
EGFL7	0	2.50524784	0.915	0.817	0	EC
PALMD	0	2.43163582	0.887	0.703	0	EC
EMCN	0	2.42439763	0.818	0.694	0	EC
FABP5	0	2.42022341	0.867	0.74	0	EC
CD36	0	2.40995485	0.835	0.796	0	EC
HYAL2	0	2.31286165	0.86	0.675	0	EC
GIMAP7	0	2.26553499	0.891	0.74	0	EC
NPDC1	0	2.25388211	0.921	0.84	0	EC
FLT1	0	2.12032068	0.831	0.722	0	EC
PCAT19	0	2.10046345	0.789	0.776	0	EC
CLEC14A	0	2.07948196	0.841	0.708	0	EC
ECSCR	0	2.06876674	0.812	0.735	0	EC
PTPRB	0	2.0494843	0.781	0.658	0	EC
GNG11	0	2.02965879	0.923	0.861	0	EC
ITGA6	0	2.01980227	0.82	0.721	0	EC
OLFM1	0	2.01886392	0.777	0.754	0	EC
MMRN1	0	2.016803	0.649	0.644	0	EC
EPAS1	0	2.01333628	0.922	0.761	0	EC
ENG	0	2.01222862	0.864	0.695	0	EC
TSPAN7	0	1.98284837	0.794	0.678	0	EC
CALCRL	0	1.976106	0.818	0.651	0	EC
CYYR1	0	1.97313911	0.809	0.73	0	EC
RAMP2	0	1.97171524	0.859	0.77	0	EC
GIMAP4	0	1.95086861	0.88	0.682	0	EC
BTNL9	0	1.94350291	0.775	0.817	0	EC
TMEM88	0	1.9429639	0.775	0.743	0	EC
DUSP23	0	1.92041875	0.856	0.68	0	EC
CD93	0	1.91808268	0.841	0.634	0	EC
MMRN2	0	1.88824921	0.761	0.606	0	EC
DIPK2B	0	1.8755235	0.8	0.739	0	EC
FAM110D	0	1.84890336	0.794	0.728	0	EC
DOCK9	0	1.83977889	0.828	0.61	0	EC
TGM2	0	1.83183312	0.769	0.655	0	EC
GIMAP1	0	1.82300293	0.868	0.728	0	EC

F8	0	1.81977957	0.76	0.658	0	EC
NRN1	0	1.81567828	0.785	0.664	0	EC
EDN1	0	1.79940568	0.687	0.623	0	EC
A2M	0	1.7930484	0.954	0.887	0	EC
PLAT	0	1.78977332	0.777	0.755	0	EC
CDH5	0	1.74119939	0.738	0.655	0	EC
RDX	0	1.72993272	0.89	0.742	0	EC
MTUS1	0	1.71214021	0.883	0.787	0	EC
TMTC1	0	1.70960311	0.833	0.693	0	EC
NOSTRIN	0	1.70725297	0.791	0.718	0	EC
PODXL	0	1.70608559	0.721	0.672	0	EC
MRTFB	0	1.70150689	0.789	0.62	0	EC
ADGRF5	0	1.67508325	0.693	0.739	0	EC
TIE1	0	1.66409583	0.81	0.748	0	EC
HLA-DRB1	0	1.65859317	0.929	0.791	0	EC
CAV1	0	1.64492585	0.953	0.841	0	EC
LDB2	0	1.64486618	0.791	0.692	0	EC
KDR	0	1.63701444	0.675	0.727	0	EC
NUAK1	0	1.62693922	0.761	0.55	0	EC
RAPGEF3	0	1.62370363	0.82	0.732	0	EC
SNHG7	0	1.62266544	0.884	0.732	0	EC
LYST	0	1.60485906	0.755	0.553	0	EC
HLA-DRB6	0	1.60316727	0.901	0.802	0	EC
SYNE2	0	1.58831857	0.889	0.675	0	EC
ADAM15	0	1.57379648	0.815	0.656	0	EC
ELK3	0	1.56851744	0.786	0.58	0	EC
ICAM2	0	1.56821494	0.722	0.64	0	EC
TFPI	0	1.55994409	0.761	0.697	0	EC
TM4SF1	0	1.55987431	0.766	0.671	0	EC
CD74	0	1.5583129	0.958	0.836	0	EC
IL1R1	0	1.55670978	0.773	0.563	0	EC
S100A16	0	1.55550313	0.832	0.66	0	EC
EFNA1	0	1.53938739	0.782	0.637	0	EC
RALGAPA2	0	1.53716617	0.753	0.532	0	EC
HLA-B	0	1.52298027	0.979	0.941	0	EC
PRCP	0	1.51876919	0.771	0.72	0	EC
COL15A1	0	1.51436179	0.844	0.805	0	EC
ROBO4	0	1.50976034	0.766	0.679	0	EC
KLF2	0	1.49277713	0.912	0.786	0	EC
LMO2	0	1.47113825	0.762	0.625	0	EC
SOX18	0	1.46910761	0.621	0.523	0	EC
BST2	0	1.46649633	0.852	0.686	0	EC
SEC14L1	0	1.46347641	0.853	0.711	0	EC
TJP1	0	1.45990769	0.848	0.681	0	EC
THSD7A	0	1.45855969	0.682	0.555	0	EC
CD300LG	0	1.44903956	0.762	0.818	0	EC
GRASP	0	1.44669545	0.769	0.634	0	EC
MYCT1	0	1.43968087	0.671	0.609	0	EC
RASIP1	0	1.43784643	0.785	0.689	0	EC
FAM107A	0	1.43643397	0.745	0.605	0	EC
S1PR1	0	1.41974486	0.752	0.655	0	EC
SH3BGRL2	0	1.4164339	0.765	0.662	0	EC
JAM2	0	1.40623757	0.727	0.67	0	EC

MECOM	0	1.40396403	0.69	0.523	0	EC
GPIHBP1	0	1.39355615	0.784	0.795	0	EC
THBD	0	1.39143852	0.629	0.445	0	EC
IL3RA	0	1.38368555	0.746	0.595	0	EC
ZNF385D	0	1.37727057	0.752	0.65	0	EC
HLA-DRA	0	1.36606271	0.937	0.826	0	EC
LMCD1	0	1.36399014	0.752	0.585	0	EC
CRIP2	0	1.36159889	0.896	0.777	0	EC
RPGR	0	1.36030832	0.798	0.62	0	EC
NOTCH4	0	1.35853069	0.708	0.66	0	EC
ID3	0	1.35472928	0.862	0.752	0	EC
SMAD1	0	1.35406196	0.685	0.48	0	EC
STC1	0	1.35360173	0.602	0.577	0	EC
TM4SF18	0	1.34589854	0.724	0.751	0	EC
TNFSF10	0	1.34437219	0.776	0.629	0	EC
ETS2	0	1.33229594	0.775	0.653	0	EC
GIMAP6	0	1.3257345	0.785	0.665	0	EC
SOCS2	0	1.32018553	0.724	0.546	0	EC
IL33	0	1.3114307	0.73	0.669	0	EC
ARL4A	0	1.30386108	0.812	0.643	0	EC
PDLIM1	0	1.3032373	0.845	0.714	0	EC
PPFIBP1	0	1.29423273	0.762	0.655	0	EC
PREX2	0	1.26883455	0.717	0.684	0	EC
SULF2	0	1.26741977	0.718	0.545	0	EC
SELP	0	1.2644584	0.679	0.643	0	EC
FLNB	0	1.25708319	0.738	0.553	0	EC
ITGB4	0	1.24846611	0.703	0.708	0	EC
KANK3	0	1.24650108	0.718	0.628	0	EC
HEG1	0	1.24491085	0.679	0.516	0	EC
EPHA4	0	1.2372682	0.784	0.635	0	EC
PKP4	0	1.2368571	0.698	0.493	0	EC
BCAM	0	1.22572776	0.887	0.72	0	EC
ADCY4	0	1.21744981	0.696	0.685	0	EC
ERG	0	1.21702056	0.629	0.65	0	EC
ABLIM1	0	1.19986668	0.874	0.785	0	EC
GIMAP8	0	1.18069446	0.712	0.652	0	EC
SEMA6A	0	1.17798383	0.636	0.573	0	EC
MCF2L	0	1.17240058	0.737	0.673	0	EC
ADAMTS9	0	1.1545266	0.546	0.516	0	EC
MGLL	0	1.15176679	0.812	0.631	0	EC
RASAL2	0	1.15041254	0.78	0.616	0	EC
CLU	0	1.14747749	0.919	0.92	0	EC
NEDD9	0	1.14595324	0.765	0.61	0	EC
CA4	0	1.1428724	0.727	0.787	0	EC
IGFBP4	0	1.14146376	0.921	0.878	0	EC
TEK	0	1.13975827	0.626	0.637	0	EC
PGM5	0	1.12952054	0.714	0.593	0	EC
PIK3R3	0	1.12813493	0.633	0.616	0	EC
FNBP1L	0	1.1246064	0.756	0.709	0	EC
EHD4	0	1.12066753	0.707	0.51	0	EC
CD34	0	1.11584867	0.79	0.758	0	EC
GATA2	0	1.11423525	0.604	0.497	0	EC
GPR146	0	1.10926134	0.752	0.619	0	EC

PTPRM	0	1.10873082	0.648	0.524	0	EC
EDNRB	0	1.09905918	0.697	0.722	0	EC
JCAD	0	1.09748875	0.669	0.661	0	EC
ST6GAL1	0	1.08705723	0.764	0.654	0	EC
ADM5	0	1.08554753	0.671	0.692	0	EC
CFI	0	1.07226136	0.647	0.563	0	EC
ITGA5	0	1.0655963	0.714	0.522	0	EC
MET	0	1.06414401	0.651	0.462	0	EC
CD320	0	1.06147189	0.736	0.695	0	EC
LTC4S	0	1.05728397	0.766	0.805	0	EC
ADGRG1	0	1.0510675	0.718	0.717	0	EC
TSPAN13	0	1.04318321	0.729	0.691	0	EC
SLCO2A1	0	1.04119706	0.559	0.569	0	EC
CPXM2	0	1.02698635	0.695	0.638	0	EC
HLA-DPA1	0	1.02140365	0.903	0.768	0	EC
ESAM	0	1.02042883	0.698	0.631	0	EC
LRRC32	0	1.02009409	0.613	0.473	0	EC
GFOD1	0	1.01964396	0.729	0.596	0	EC
TPD52L1	0	1.01605584	0.719	0.697	0	EC
IGFBP2	0	1.01423708	0.823	0.685	0	EC
SLCO4A1	0	1.01236315	0.712	0.661	0	EC
FAM167B	0	1.00717691	0.716	0.74	0	EC
LPAR6	0	1.00660283	0.662	0.582	0	EC
PDE2A	0	1.00370063	0.689	0.616	0	EC
WARS	0	0.99550142	0.727	0.62	0	EC
LRRC1	0	0.98253812	0.693	0.55	0	EC
PLCXD3	0	0.98210523	0.642	0.621	0	EC
NRP2	0	0.97879416	0.574	0.523	0	EC
ETS1	0	0.97143996	0.742	0.596	0	EC
SHE	0	0.96680303	0.542	0.514	0	EC
HLA-DQB1	0	0.96333302	0.766	0.574	0	EC
SPRY1	0	0.96066816	0.845	0.725	0	EC
ARHGEF15	0	0.95190862	0.663	0.69	0	EC
SHROOM4	0	0.94790075	0.659	0.538	0	EC
PIM3	0	0.94283818	0.622	0.578	0	EC
ATOH8	0	0.93842725	0.768	0.573	0	EC
SHANK3	0	0.93648599	0.526	0.478	0	EC
PLCB4	0	0.93556725	0.635	0.604	0	EC
CASKIN2	0	0.93513576	0.632	0.525	0	EC
HLA-DRB5	0	0.92751718	0.846	0.678	0	EC
PCDH1	0	0.92561972	0.757	0.679	0	EC
GALNT15	0	0.91391776	0.783	0.708	0	EC
DEPP1	0	0.90497398	0.803	0.784	0	EC
TSPAN18	0	0.90116311	0.641	0.538	0	EC
AKR1C3	0	0.89940289	0.684	0.617	0	EC
PTPN14	0	0.88909628	0.599	0.518	0	EC
SOX7	0	0.88908722	0.558	0.591	0	EC
TMEM273	0	0.88873964	0.587	0.581	0	EC
CADM3-AS1	0	0.88050335	0.665	0.607	0	EC
CCDC68	0	0.87660724	0.702	0.553	0	EC
EPHB4	0	0.87462334	0.57	0.506	0	EC
HLA-DQA2	0	0.87033292	0.722	0.719	0	EC
CMIP	0	0.86961455	0.605	0.451	0	EC

SLFN5	0	0.86387625	0.705	0.554	0	EC
AFAP1L1	0	0.86307207	0.597	0.615	0	EC
CD79B	0	0.85597676	0.718	0.66	0	EC
FAM13C	0	0.85288185	0.788	0.698	0	EC
SOX17	0	0.8458949	0.563	0.482	0	EC
HLA-DQA1	0	0.84216468	0.718	0.572	0	EC
DOC2B	0	0.8372463	0.603	0.616	0	EC
CCL23	0	0.8302395	0.675	0.633	0	EC
HLA-DMA	0	0.82412742	0.78	0.567	0	EC
LEPR	0	0.82088507	0.677	0.602	0	EC
KCTD12	0	0.82065925	0.81	0.744	0	EC
MYO5C	0	0.81871419	0.567	0.633	0	EC
PRXL2A	0	0.81744348	0.786	0.779	0	EC
TLL1	0	0.81317424	0.506	0.506	0	EC
RHOU	0	0.80500638	0.633	0.574	0	EC
C1orf115	0	0.80056895	0.544	0.484	0	EC
ASS1	0	0.79919239	0.634	0.568	0	EC
NES	0	0.79602732	0.681	0.614	0	EC
AC118754.1	0	0.79290896	0.541	0.476	0	EC
HECW2	0	0.79109374	0.65	0.572	0	EC
NOVA2	0	0.78983584	0.55	0.64	0	EC
OCIAD2	0	0.78251883	0.648	0.506	0	EC
CRIM1	0	0.77229054	0.701	0.596	0	EC
ICA1	0	0.76961906	0.588	0.616	0	EC
CRHBP	0	0.76809662	0.705	0.68	0	EC
PPARG	0	0.76123547	0.699	0.544	0	EC
CTSH	0	0.76018565	0.762	0.653	0	EC
AC007681.1	0	0.75346826	0.677	0.581	0	EC
GPM6A	0	0.73214993	0.675	0.665	0	EC
DACH1	0	0.73026828	0.65	0.694	0	EC
CDC42EP3	0	0.72829549	0.632	0.488	0	EC
GBP4	0	0.72768475	0.602	0.538	0	EC
CNKSR3	0	0.72365064	0.568	0.543	0	EC
LCN6	0	0.70924335	0.583	0.563	0	EC
ENPP2	0	0.70766551	0.598	0.549	0	EC
NEURL1B	0	0.69566984	0.646	0.549	0	EC
HLA-DPB1	0	0.6950291	0.865	0.755	0	EC
AIF1L	0	0.691281	0.618	0.593	0	EC
ANXA3	0	0.69111742	0.564	0.569	0	EC
EFNB1	0	0.68908449	0.647	0.535	0	EC
CPLX1	0	0.685835	0.6	0.561	0	EC
CETP	0	0.67709409	0.665	0.75	0	EC
NET1	0	0.67517148	0.691	0.704	0	EC
PLCB1	0	0.67176038	0.605	0.426	0	EC
TSHZ2	0	0.66553292	0.833	0.799	0	EC
ABCG2	0	0.66198231	0.65	0.601	0	EC
VWA1	0	0.66080251	0.694	0.662	0	EC
LHX6	0	0.65272631	0.508	0.402	0	EC
IFI44L	0	0.647278	0.517	0.447	0	EC
ADGRG6	0	0.64714408	0.58	0.552	0	EC
HSPA12B	0	0.64621312	0.542	0.558	0	EC
JAG2	0	0.64516824	0.598	0.582	0	EC
LIMCH1	0	0.63295491	0.667	0.633	0	EC

GJA1	0	0.62896079	0.578	0.531	0	EC
HAPLN3	0	0.62874797	0.594	0.538	0	EC
AHNAK2	0	0.62637939	0.564	0.527	0	EC
DIPK1B	0	0.62242043	0.649	0.59	0	EC
MARCKSL1	0	0.62241056	0.583	0.526	0	EC
LGALS3	0	0.62217305	0.937	0.887	0	EC
SEMA6B	0	0.61285981	0.533	0.489	0	EC
DSG2	0	0.61035547	0.638	0.644	0	EC
AL583785.1	0	0.60549452	0.684	0.664	0	EC
EMP1	0	0.59504066	0.71	0.718	0	EC
MYRIP	0	0.59129969	0.554	0.615	0	EC
SNCG	0	0.59094882	0.765	0.738	0	EC
STMN1	0	0.58738993	0.716	0.649	0	EC
MX1	0	0.58659021	0.541	0.467	0	EC
MAST4	0	0.57995725	0.562	0.493	0	EC
TPO	0	0.57877996	0.53	0.527	0	EC
TCN2	0	0.57304557	0.629	0.551	0	EC
ACE	0	0.56996506	0.568	0.578	0	EC
LINC02185	0	0.56983158	0.649	0.585	0	EC
DNASE1L3	0	0.55838565	0.514	0.423	0	EC
DLL4	0	0.5559846	0.555	0.602	0	EC
MLPH	0	0.55086116	0.526	0.405	0	EC
TIMP3	0	0.54954686	0.936	0.906	0	EC
ITGA10	0	0.54803299	0.627	0.55	0	EC
ACTN1	0	0.53756259	0.719	0.673	0	EC
PERP	0	0.52506598	0.529	0.496	0	EC
SELE	0	0.51626906	0.214	0.303	0	EC
ITPR2	0	0.50090359	0.63	0.538	0	EC
RAB3C	0	0.49698914	0.536	0.496	0	EC
CX3CL1	0	0.4957284	0.508	0.444	0	EC
DUSP6	0	0.49088794	0.735	0.579	0	EC
TIMP4	0	0.48068921	0.664	0.621	0	EC
SYNJ2	0	0.45763553	0.573	0.445	0	EC
GMFG	0	0.45551572	0.751	0.702	0	EC
TNFRSF1B	0	0.45300442	0.674	0.574	0	EC
GABRA2	0	0.45195199	0.598	0.559	0	EC
PDGFB	0	0.38775056	0.561	0.468	0	EC
ADAMTS6	0	0.38768773	0.531	0.388	0	EC
KLHL4	0	0.37902143	0.481	0.401	0	EC
CDH13	0	0.37699458	0.504	0.42	0	EC
TINAGL1	0	0.35821755	0.787	0.685	0	EC
NPC2	0	0.35786458	0.859	0.805	0	EC
IL32	0	0.35644271	0.742	0.691	0	EC
TNFRSF4	0	0.34375862	0.575	0.593	0	EC
CDA	0	0.33524781	0.515	0.5	0	EC
KRT7	0	0.33499674	0.56	0.416	0	EC
SCNN1B	0	0.31184474	0.55	0.599	0	EC
KCNB1	0	0.30449425	0.626	0.562	0	EC
OTC	0	0.28766894	0.642	0.581	0	EC
PRSS23	0	0.28209004	0.62	0.56	0	EC
GJC2	0	0.22139912	0.47	0.376	0	EC
HLA-DMB	0	0.20708534	0.666	0.575	0	EC
MCTP1	2.99E-296	1.02602182	0.58	0.655	5.99E-293	EC

NR2F2	3.22E-291	0.36184119	0.784	0.755	6.45E-288	EC
BMP6	7.09E-282	0.2448369	0.553	0.616	1.42E-278	EC
SEMA3G	1.84E-277	0.68075403	0.666	0.657	3.69E-274	EC
HLA-DOA	4.80E-273	0.33985177	0.571	0.594	9.61E-270	EC
HMCN1	5.63E-263	0.33760979	0.521	0.463	1.13E-259	EC
PLA1A	1.49E-259	0.22377881	0.468	0.448	2.98E-256	EC
SLC2A3	5.51E-259	0.61581822	0.623	0.589	1.10E-255	EC
VCAM1	2.38E-250	0.27336065	0.324	0.524	4.76E-247	EC
DSP	3.93E-250	0.78989521	0.529	0.572	7.85E-247	EC
ABCB1	1.54E-246	0.34991568	0.473	0.412	3.09E-243	EC
MMP28	7.26E-245	0.51160999	0.509	0.514	1.45E-241	EC
GCNT2	1.05E-242	0.21288954	0.411	0.326	2.09E-239	EC
ANGPT2	1.47E-238	0.75575249	0.665	0.663	2.94E-235	EC
PLEKHG1	4.26E-234	0.85505042	0.497	0.502	8.51E-231	EC
ALDH1A3	4.74E-232	0.24640515	0.496	0.512	9.49E-229	EC
MDK	6.44E-230	0.45767082	0.657	0.674	1.29E-226	EC
FCN3	3.80E-224	0.853004	0.627	0.714	7.60E-221	EC
LXN	2.46E-222	0.36927038	0.506	0.484	4.93E-219	EC
RUNDC3B	4.15E-210	0.3186081	0.513	0.536	8.30E-207	EC
FKBP11	3.89E-207	0.20747083	0.637	0.608	7.78E-204	EC
NDRG4	1.00E-203	0.33495414	0.451	0.406	2.00E-200	EC
PTP4A3	1.88E-196	0.20030458	0.532	0.514	3.76E-193	EC
PROX1	1.08E-186	1.22466675	0.514	0.555	2.16E-183	EC
APOLD1	7.39E-186	0.68950851	0.613	0.668	1.48E-182	EC
PLIN5	1.05E-185	0.41522915	0.449	0.419	2.10E-182	EC
HEY1	8.04E-179	0.75473077	0.636	0.681	1.61E-175	EC
INMT	1.21E-178	0.24443953	0.577	0.55	2.41E-175	EC
F2R	3.38E-177	0.22247515	0.582	0.593	6.76E-174	EC
KRT18	2.84E-176	0.28091224	0.429	0.377	5.67E-173	EC
KRT8	2.22E-167	0.24505549	0.447	0.424	4.43E-164	EC
C1QTNF9	1.39E-166	0.37560552	0.459	0.446	2.78E-163	EC
FAM241A	3.75E-166	0.71569555	0.517	0.526	7.51E-163	EC
CLDN11	2.68E-159	0.28057626	0.474	0.455	5.35E-156	EC
APLNR	5.13E-156	0.30325614	0.328	0.489	1.03E-152	EC
PKHD1L1	5.66E-150	0.88407878	0.471	0.47	1.13E-146	EC
SGK1	4.08E-147	0.33571619	0.537	0.506	8.16E-144	EC
RFLNB	1.55E-145	0.260604	0.504	0.495	3.10E-142	EC
STXBP6	1.17E-142	0.29184181	0.577	0.566	2.34E-139	EC
MALL	9.63E-141	0.82662448	0.459	0.46	1.93E-137	EC
CTSC	1.03E-138	0.40551846	0.704	0.694	2.06E-135	EC
C17orf58	8.75E-136	0.22640403	0.449	0.44	1.75E-132	EC
CYSLTR1	1.36E-130	0.45705396	0.503	0.526	2.72E-127	EC
LRG1	1.68E-126	0.51750508	0.453	0.459	3.37E-123	EC
COX7A1	4.60E-113	0.21366139	0.746	0.728	9.20E-110	EC
EFNB2	1.30E-111	1.10229499	0.495	0.567	2.59E-108	EC
TMEM150C	2.34E-111	0.2810466	0.474	0.52	4.67E-108	EC
IER3	1.32E-107	0.29838403	0.658	0.66	2.64E-104	EC
PNP	1.33E-102	0.32479564	0.442	0.43	2.66E-99	EC
TGFBR3	4.02E-94	0.32467568	0.723	0.816	8.04E-91	EC
ARL15	8.12E-94	0.47045359	0.529	0.58	1.62E-90	EC
IFI6	1.02E-92	0.2450755	0.552	0.53	2.04E-89	EC
IFIT3	3.39E-91	0.3454494	0.464	0.469	6.78E-88	EC
PNPLA2	5.08E-91	0.29271064	0.587	0.577	1.02E-87	EC

MEOX1	1.38E-87	0.53802838	0.516	0.629	2.75E-84	EC
SEC11C	3.55E-84	0.29892195	0.601	0.617	7.09E-81	EC
SYT1	2.78E-83	0.21324904	0.474	0.476	5.56E-80	EC
LIPE	7.83E-79	0.2438469	0.428	0.427	1.57E-75	EC
ALDH1A2	3.11E-77	0.34394983	0.41	0.399	6.22E-74	EC
CR381670.1	1.75E-76	0.3867054	0.38	0.571	3.49E-73	EC
TNFRSF10D	4.92E-74	0.58288298	0.401	0.393	9.84E-71	EC
FAM43A	2.71E-70	0.43741933	0.537	0.604	5.42E-67	EC
TACR1	2.62E-69	0.3367423	0.432	0.439	5.23E-66	EC
ADM	9.26E-65	0.3457931	0.501	0.642	1.85E-61	EC
TFF3	1.38E-59	1.97404162	0.541	0.639	2.77E-56	EC
CLIC5	2.78E-56	0.40175813	0.554	0.619	5.56E-53	EC
MYO10	1.08E-54	0.47839657	0.475	0.527	2.16E-51	EC
TSPAN5	1.40E-46	0.71768612	0.556	0.633	2.79E-43	EC
RAPGEF5	3.00E-46	0.67817347	0.521	0.607	6.00E-43	EC
SCG3	7.97E-45	0.61048612	0.427	0.57	1.59E-41	EC
KCNN3	1.16E-42	0.4450682	0.354	0.471	2.32E-39	EC
GJA5	5.12E-42	0.31358341	0.359	0.487	1.02E-38	EC
MPZL2	4.09E-40	0.49925729	0.393	0.367	8.19E-37	EC
AQP7	3.26E-37	0.33723602	0.502	0.559	6.51E-34	EC
PDK4	1.90E-35	0.21464896	0.808	0.848	3.79E-32	EC
PGM5-AS1	5.27E-34	0.38948018	0.413	0.455	1.05E-30	EC
ITLN1	5.16E-33	1.12507681	0.561	0.587	1.03E-29	EC
MEOX2	1.02E-30	0.35925102	0.496	0.565	2.04E-27	EC
NOS3	8.78E-29	0.42227131	0.378	0.516	1.76E-25	EC
RSAD2	1.32E-25	0.23425623	0.375	0.393	2.64E-22	EC
CD9	1.32E-24	0.65680125	0.64	0.718	2.64E-21	EC
C2CD4B	1.13E-22	0.45829933	0.367	0.341	2.27E-19	EC
TBX1	1.60E-20	0.58086631	0.519	0.6	3.20E-17	EC
CRTAC1	1.04E-17	0.40945496	0.417	0.469	2.08E-14	EC
BHLHE40	8.04E-17	0.29231372	0.422	0.466	1.61E-13	EC
IGFBP3	2.90E-16	0.49201513	0.743	0.789	5.80E-13	EC
ICAM1	4.36E-15	0.40409652	0.405	0.527	8.71E-12	EC
ART4	5.09E-11	0.21124516	0.501	0.539	1.02E-07	EC
NR2F1	2.80E-09	0.48805013	0.412	0.522	5.61E-06	EC
RGCC	1.07E-08	0.99221309	0.666	0.671	2.15E-05	EC
LPL	1.21E-08	0.27126417	0.458	0.5	2.43E-05	EC
INHBB	1.57E-08	0.25758777	0.33	0.369	3.14E-05	EC
PTPRE	2.39E-08	0.30421186	0.416	0.459	4.78E-05	EC
BCL6B	5.55E-08	0.54900379	0.399	0.533	0.000111	EC
SNAI1	5.99E-08	0.35102409	0.403	0.511	0.00011981	EC
PCDH19	2.62E-07	0.39326842	0.381	0.502	0.00052482	EC
CCL21	5.54E-07	2.56865955	0.496	0.59	0.00110732	EC
SEMA3A	2.39E-06	0.20667961	0.333	0.399	0.00477599	EC
ST8SIA6	2.86E-06	0.38636731	0.389	0.431	0.00571006	EC
FAM124B	3.23E-06	0.23877896	0.34	0.368	0.00645814	EC
GJA4	0.00018328	0.37195534	0.532	0.631	0.36656013	EC
CYTL1	0.00031488	0.36819356	0.393	0.483	0.62976352	EC
PCDH17	0.00073413	0.58389564	0.467	0.628	1	EC
BCL3	0.00161475	0.30693198	0.354	0.393	1	EC
CCL19	0	3.94202451	0.877	0.576	0	Pericyte
STEAP4	0	3.8078537	0.987	0.651	0	Pericyte
C2orf40	0	2.91981067	0.898	0.587	0	Pericyte

APOE	0	2.65110649	0.949	0.603	0	Pericyte
RRAD	0	2.5425282	0.967	0.603	0	Pericyte
SYNPO2	0	2.41211619	0.939	0.644	0	Pericyte
TGFBI	0	2.29155503	0.968	0.651	0	Pericyte
RGS5	0	2.27068851	0.748	0.545	0	Pericyte
NR2F2	0	1.97217178	0.972	0.747	0	Pericyte
TIMP3	0	1.88705969	0.997	0.906	0	Pericyte
NDUFA4L2	0	1.82667037	0.902	0.659	0	Pericyte
NOTCH3	0	1.82554716	0.965	0.58	0	Pericyte
INPP4B	0	1.79081048	0.893	0.499	0	Pericyte
ID4	0	1.78167679	0.814	0.486	0	Pericyte
SLC7A2	0	1.76423081	0.928	0.618	0	Pericyte
CALD1	0	1.76040729	0.998	0.903	0	Pericyte
TAGLN	0	1.74585181	0.997	0.899	0	Pericyte
LGI4	0	1.700059	0.872	0.604	0	Pericyte
FILIP1L	0	1.63609742	0.982	0.749	0	Pericyte
TINAGL1	0	1.63161314	0.927	0.691	0	Pericyte
TPM1	0	1.63059288	0.993	0.868	0	Pericyte
PMEPA1	0	1.61995757	0.818	0.517	0	Pericyte
RASL11A	0	1.60226178	0.882	0.537	0	Pericyte
GUCY1A2	0	1.56895469	0.809	0.523	0	Pericyte
CCL21	0	1.56380478	0.82	0.555	0	Pericyte
AVPR1A	0	1.56169102	0.884	0.58	0	Pericyte
CCL2	0	1.52453973	0.698	0.435	0	Pericyte
LMOD1	0	1.52410078	0.94	0.69	0	Pericyte
GGT5	0	1.52081488	0.859	0.628	0	Pericyte
TMEM176A	0	1.46769732	0.912	0.653	0	Pericyte
FHL5	0	1.46520723	0.894	0.616	0	Pericyte
MT1A	0	1.45698954	0.921	0.744	0	Pericyte
MT1L	0	1.45098861	0.86	0.592	0	Pericyte
COX4I2	0	1.43661773	0.734	0.578	0	Pericyte
CLSTN2	0	1.41684478	0.863	0.593	0	Pericyte
FAM13C	0	1.40964192	0.894	0.705	0	Pericyte
TM4SF1	0	1.40885783	0.812	0.682	0	Pericyte
FGF7	0	1.39768065	0.758	0.547	0	Pericyte
RGS16	0	1.39395234	0.74	0.567	0	Pericyte
RARRES2	0	1.39355359	0.963	0.769	0	Pericyte
ABCC9	0	1.36148769	0.911	0.71	0	Pericyte
CNR1	0	1.35915582	0.915	0.606	0	Pericyte
FHL2	0	1.35009573	0.839	0.577	0	Pericyte
PRXL2A	0	1.34369702	0.931	0.771	0	Pericyte
ISYNA1	0	1.33001581	0.785	0.507	0	Pericyte
DEPP1	0	1.29339974	0.92	0.779	0	Pericyte
MT2A	0	1.27156343	0.999	0.964	0	Pericyte
GJA4	0	1.26892159	0.725	0.604	0	Pericyte
H19	0	1.25912752	0.845	0.688	0	Pericyte
MAP1B	0	1.25662738	0.962	0.703	0	Pericyte
4-Sep	0	1.24642319	0.769	0.558	0	Pericyte
SEMA5A	0	1.22546515	0.772	0.558	0	Pericyte
MYL9	0	1.20738453	0.998	0.883	0	Pericyte
SSTR2	0	1.20181172	0.695	0.543	0	Pericyte
COL25A1	0	1.17371293	0.781	0.475	0	Pericyte
FAM162B	0	1.16758125	0.714	0.517	0	Pericyte

ADGRF5	0	1.16726171	0.917	0.718	0	Pericyte
ANGPT2	0	1.15903773	0.784	0.656	0	Pericyte
ACTA2	0	1.1551702	0.977	0.758	0	Pericyte
MT1F	0	1.13919175	0.842	0.615	0	Pericyte
MYLK	0	1.13768713	0.885	0.569	0	Pericyte
SYTL2	0	1.13469314	0.675	0.411	0	Pericyte
ITGA7	0	1.12267084	0.805	0.454	0	Pericyte
COL4A1	0	1.11028619	0.899	0.659	0	Pericyte
ADGRL3	0	1.10858526	0.76	0.564	0	Pericyte
ANGPT1	0	1.10483418	0.761	0.458	0	Pericyte
COL4A2	0	1.10061433	0.943	0.723	0	Pericyte
GUCY1A1	0	1.08625078	0.807	0.571	0	Pericyte
FBLIM1	0	1.08035155	0.895	0.531	0	Pericyte
MT1M	0	1.07984494	0.971	0.88	0	Pericyte
MDK	0	1.0797158	0.789	0.663	0	Pericyte
CXCL12	0	1.07691213	0.908	0.789	0	Pericyte
MYC	0	1.06184599	0.842	0.627	0	Pericyte
SLIT3	0	1.04976159	0.856	0.645	0	Pericyte
MT1E	0	1.04821669	0.988	0.839	0	Pericyte
FABP4	0	1.04644981	0.968	0.913	0	Pericyte
CARMN	0	1.03284428	0.835	0.448	0	Pericyte
ADAMTS1	0	1.0323767	0.88	0.713	0	Pericyte
IGFBP5	0	1.02445172	0.982	0.885	0	Pericyte
TPPP3	0	1.00705158	0.678	0.5	0	Pericyte
LINC00924	0	1.00487411	0.788	0.51	0	Pericyte
NEXN	0	0.99930253	0.945	0.683	0	Pericyte
NCKAP5	0	0.9721508	0.698	0.476	0	Pericyte
TMEM176B	0	0.97042993	0.883	0.674	0	Pericyte
VCL	0	0.95673328	0.883	0.638	0	Pericyte
LPP	0	0.91710921	0.949	0.769	0	Pericyte
MRVI1	0	0.89053529	0.811	0.522	0	Pericyte
AP000892.3	0	0.87053869	0.836	0.527	0	Pericyte
C11orf96	0	0.86278134	0.915	0.677	0	Pericyte
CAV1	0	0.85506446	0.983	0.856	0	Pericyte
GEM	0	0.85400105	0.817	0.498	0	Pericyte
TIMP4	0	0.8393615	0.799	0.618	0	Pericyte
PDE5A	0	0.81583193	0.824	0.573	0	Pericyte
HSPB6	0	0.81262701	0.789	0.615	0	Pericyte
CTSC	0	0.81136666	0.878	0.684	0	Pericyte
PROCR	0	0.80718842	0.785	0.696	0	Pericyte
CCL8	0	0.80673396	0.628	0.354	0	Pericyte
LRRC17	0	0.80630673	0.896	0.642	0	Pericyte
CCN1	0	0.79970872	0.838	0.671	0	Pericyte
CCND1	0	0.79587524	0.747	0.502	0	Pericyte
PPP1R14A	0	0.7790615	0.919	0.637	0	Pericyte
CSRP1	0	0.73262304	0.894	0.607	0	Pericyte
PALLD	0	0.72619653	0.94	0.757	0	Pericyte
PDGFA	0	0.72252232	0.711	0.437	0	Pericyte
BMP2	0	0.71152462	0.784	0.339	0	Pericyte
EDIL3	0	0.70829518	0.762	0.498	0	Pericyte
TJP1	0	0.70363226	0.861	0.705	0	Pericyte
TBX3	0	0.68901567	0.655	0.461	0	Pericyte
CD36	0	0.68340315	0.873	0.799	0	Pericyte

PTGER1	0	0.65835401	0.686	0.53	0	Pericyte
TPM2	0	0.64034726	0.977	0.822	0	Pericyte
PDLIM1	0	0.63406028	0.881	0.731	0	Pericyte
A2M	0	0.63308995	0.992	0.894	0	Pericyte
VIPR1	0	0.62477461	0.698	0.525	0	Pericyte
PPARG	0	0.61928317	0.772	0.562	0	Pericyte
TFPI	0	0.60323827	0.882	0.698	0	Pericyte
PRKAR2B	0	0.5998775	0.7	0.488	0	Pericyte
APOLD1	0	0.5974564	0.834	0.646	0	Pericyte
SMOC2	0	0.59541244	0.793	0.473	0	Pericyte
ADAMTS5	0	0.59221843	0.75	0.567	0	Pericyte
ACTG2	0	0.59038764	0.763	0.523	0	Pericyte
CLMN	0	0.59022901	0.833	0.572	0	Pericyte
FLNA	0	0.56773686	0.965	0.794	0	Pericyte
KCNE4	0	0.54387268	0.735	0.518	0	Pericyte
APOC1	0	0.52628497	0.675	0.427	0	Pericyte
TBX2	0	0.51448051	0.747	0.424	0	Pericyte
CCDC71L	0	0.4998716	0.84	0.68	0	Pericyte
BGN	0	0.49124108	0.948	0.762	0	Pericyte
CYGB	0	0.49024007	0.822	0.729	0	Pericyte
ALKAL2	0	0.47964423	0.769	0.478	0	Pericyte
CRIP1	0	0.47774978	0.896	0.789	0	Pericyte
MYO10	0	0.45009742	0.694	0.505	0	Pericyte
CP	0	0.4487214	0.66	0.394	0	Pericyte
GPX3	0	0.43288243	0.967	0.861	0	Pericyte
AEBP1	0	0.42680158	0.928	0.748	0	Pericyte
NGFR	0	0.40766294	0.691	0.517	0	Pericyte
EDNRB	0	0.40160553	0.835	0.709	0	Pericyte
EZR	0	0.38387556	0.836	0.545	0	Pericyte
RCAN2	0	0.37830591	0.804	0.567	0	Pericyte
PTGER3	0	0.34026727	0.682	0.418	0	Pericyte
CCL13	0	0.33617544	0.81	0.475	0	Pericyte
ASPN	0	0.3285542	0.806	0.467	0	Pericyte
GPM6B	0	0.32844508	0.722	0.602	0	Pericyte
PAPPA	0	0.32401836	0.792	0.486	0	Pericyte
RBP5	0	0.30318064	0.709	0.557	0	Pericyte
FST	0	0.30284926	0.766	0.573	0	Pericyte
ADRA2C	0	0.26547429	0.769	0.541	0	Pericyte
GPRIN3	0	0.25880499	0.786	0.517	0	Pericyte
FGF10	0	0.24614561	0.674	0.352	0	Pericyte
COCH	0	0.24591409	0.643	0.462	0	Pericyte
SUCNR1	0	0.2204146	0.738	0.406	0	Pericyte
SNCG	4.51E-308	0.56245908	0.85	0.736	9.03E-305	Pericyte
FHL1	2.80E-294	0.51342045	0.85	0.704	5.61E-291	Pericyte
HES4	5.81E-290	0.61892592	0.754	0.619	1.16E-286	Pericyte
THY1	4.25E-289	1.06377464	0.706	0.658	8.50E-286	Pericyte
EDNRA	4.03E-288	1.00842279	0.661	0.514	8.06E-285	Pericyte
LMCD1	3.97E-286	0.52795226	0.75	0.61	7.93E-283	Pericyte
ITIH5	3.68E-283	0.40077603	0.761	0.621	7.36E-280	Pericyte
IGFBP2	6.39E-281	0.78713963	0.847	0.704	1.28E-277	Pericyte
TMEM47	3.08E-269	0.38456915	0.735	0.573	6.16E-266	Pericyte
PHLDA1	1.11E-265	0.42413744	0.652	0.422	2.23E-262	Pericyte
KCNK3	7.55E-260	0.43886747	0.584	0.337	1.51E-256	Pericyte

RHOBTB3	1.34E-256	0.52068883	0.77	0.621	2.68E-253	Pericyte
CRYAB	5.70E-255	0.33758226	0.859	0.734	1.14E-251	Pericyte
AP002956.1	1.07E-252	0.40839388	0.737	0.487	2.14E-249	Pericyte
FMO2	2.81E-252	0.24162026	0.876	0.773	5.61E-249	Pericyte
CHRDL1	1.73E-250	0.31827758	0.83	0.728	3.46E-247	Pericyte
COL6A3	3.86E-238	0.28830632	0.807	0.7	7.72E-235	Pericyte
KLHL23	9.76E-234	0.6197051	0.664	0.53	1.95E-230	Pericyte
TUBA1C	1.79E-231	0.4805755	0.78	0.572	3.57E-228	Pericyte
DKK3	2.06E-224	0.23268739	0.753	0.589	4.11E-221	Pericyte
ANGPTL4	1.91E-222	0.57889415	0.727	0.638	3.81E-219	Pericyte
IGFBP4	1.83E-220	0.29669633	0.972	0.881	3.66E-217	Pericyte
PMP22	2.64E-216	0.27767353	0.941	0.847	5.28E-213	Pericyte
C12orf75	4.74E-209	0.38357907	0.739	0.552	9.48E-206	Pericyte
CRIM1	1.45E-198	0.54168878	0.748	0.608	2.89E-195	Pericyte
COX7A1	1.48E-197	0.55614628	0.802	0.727	2.97E-194	Pericyte
HIGD1B	6.95E-197	1.13595214	0.612	0.533	1.39E-193	Pericyte
HES1	3.45E-192	0.69869221	0.717	0.598	6.90E-189	Pericyte
SCGN	5.77E-187	0.21299271	0.669	0.53	1.15E-183	Pericyte
TCIM	1.21E-179	0.61424926	0.725	0.682	2.43E-176	Pericyte
CADM1	8.10E-172	0.26934183	0.617	0.504	1.62E-168	Pericyte
PGF	1.92E-162	0.2891174	0.684	0.545	3.84E-159	Pericyte
CBLN1	5.43E-157	0.28072952	0.285	0.317	1.09E-153	Pericyte
CCDC102B	6.13E-157	0.8340451	0.601	0.447	1.23E-153	Pericyte
PCOLCE	2.12E-156	0.26083958	0.82	0.757	4.25E-153	Pericyte
MGLL	2.71E-148	0.37569864	0.746	0.662	5.43E-145	Pericyte
SPARC	1.36E-146	0.3854824	0.913	0.838	2.72E-143	Pericyte
NR2F1	1.59E-138	0.34547287	0.608	0.493	3.18E-135	Pericyte
HOPX	4.62E-132	0.72658274	0.579	0.447	9.23E-129	Pericyte
EBF2	6.89E-128	0.51322165	0.65	0.558	1.38E-124	Pericyte
AGT	1.18E-123	0.83603013	0.582	0.443	2.37E-120	Pericyte
NID1	1.49E-123	0.36756955	0.713	0.602	2.98E-120	Pericyte
FAM241A	3.58E-117	0.24907671	0.645	0.516	7.16E-114	Pericyte
TBX2-AS1	4.29E-115	0.3509967	0.598	0.454	8.59E-112	Pericyte
TYMP	6.95E-115	0.20641834	0.803	0.651	1.39E-111	Pericyte
KRT8	6.70E-113	0.31506529	0.569	0.419	1.34E-109	Pericyte
ADAMTS4	5.79E-111	0.68465464	0.553	0.42	1.16E-107	Pericyte
NT5DC2	1.61E-108	0.67093789	0.609	0.508	3.22E-105	Pericyte
KCNAB1	2.63E-107	0.4479059	0.62	0.525	5.26E-104	Pericyte
PAPPA2	1.79E-100	0.34652149	0.53	0.436	3.58E-97	Pericyte
ANGPT4	3.89E-100	0.35058716	0.554	0.379	7.77E-97	Pericyte
LINC00702	1.04E-91	0.22718472	0.61	0.497	2.07E-88	Pericyte
NEURL1B	1.21E-89	0.69927085	0.6	0.566	2.41E-86	Pericyte
PCDH9	7.06E-84	0.24109632	0.576	0.455	1.41E-80	Pericyte
COLEC11	2.57E-81	0.24469634	0.521	0.392	5.13E-78	Pericyte
COL5A3	1.54E-73	0.74758318	0.501	0.354	3.08E-70	Pericyte
SNAI2	1.15E-68	0.27162581	0.635	0.566	2.30E-65	Pericyte
CCL26	1.93E-66	0.21801236	0.428	0.535	3.87E-63	Pericyte
TNC	8.17E-65	0.2479942	0.51	0.387	1.63E-61	Pericyte
PTP4A3	1.58E-64	0.2718494	0.597	0.512	3.15E-61	Pericyte
CYTOR	3.86E-56	0.48230797	0.549	0.437	7.73E-53	Pericyte
TNMD	3.07E-49	0.22555081	0.373	0.396	6.13E-46	Pericyte
ADRA2A	2.22E-48	0.70308111	0.501	0.386	4.43E-45	Pericyte
RASD1	1.96E-46	0.50442053	0.561	0.492	3.92E-43	Pericyte

PI15	1.74E-44	0.28802323	0.397	0.468	3.48E-41	Pericyte
SLC12A2	9.68E-44	0.2247562	0.601	0.538	1.94E-40	Pericyte
NPY1R	6.46E-43	0.23989551	0.598	0.502	1.29E-39	Pericyte
CAMK2N1	1.07E-41	0.46526339	0.611	0.577	2.13E-38	Pericyte
L1TD1	2.52E-41	0.22549355	0.377	0.401	5.04E-38	Pericyte
ESAM	1.62E-32	0.31859938	0.639	0.645	3.25E-29	Pericyte
SOX4	6.00E-30	0.24510057	0.666	0.655	1.20E-26	Pericyte
ITM2C	9.85E-26	0.46027953	0.561	0.538	1.97E-22	Pericyte
FOXS1	1.93E-23	0.32052704	0.381	0.361	3.86E-20	Pericyte
FRZB	4.21E-17	0.25853414	0.508	0.432	8.42E-14	Pericyte
SUSD2	2.13E-14	0.48029762	0.414	0.424	4.27E-11	Pericyte
EGFL6	1.34E-12	0.32154165	0.388	0.272	2.68E-09	Pericyte
CKB	1.15E-11	0.23131486	0.596	0.572	2.30E-08	Pericyte
MEOX2	5.40E-10	0.30468585	0.544	0.552	1.08E-06	Pericyte
PTN	1.52E-09	0.35375406	0.542	0.517	3.05E-06	Pericyte
CH25H	8.18E-08	0.36616317	0.495	0.446	0.00016363	Pericyte
CCDC3	7.16E-06	0.25184299	0.517	0.469	0.0143249	Pericyte
RCAN1	2.04E-05	0.2042205	0.557	0.528	0.0408166	Pericyte
IFIT3	0.00024136	0.20335608	0.442	0.47	0.48271913	Pericyte
LINC02381	0.00362094	0.32707047	0.478	0.508	1	Pericyte
VGLL3	0.00363193	0.46950527	0.477	0.46	1	Pericyte
PLAU	0.00375764	0.24629088	0.416	0.319	1	Pericyte
KRT18	0.0046934	0.489714	0.454	0.383	1	Pericyte
PLA2G2A	0	3.92015258	0.954	0.886	0	Fibroblast
DCN	0	3.69983069	0.998	0.886	0	Fibroblast
FBLN1	0	3.37621052	0.994	0.868	0	Fibroblast
CXCL14	0	3.21038299	0.814	0.571	0	Fibroblast
CFD	0	3.19445947	0.979	0.913	0	Fibroblast
SERPINF1	0	3.15953856	0.986	0.815	0	Fibroblast
ADH1B	0	3.14761087	0.98	0.799	0	Fibroblast
C3	0	3.08538726	0.971	0.767	0	Fibroblast
IGFBP6	0	3.01442235	0.986	0.892	0	Fibroblast
C7	0	2.87779261	0.895	0.752	0	Fibroblast
CCDC80	0	2.86787005	0.97	0.694	0	Fibroblast
RARRES1	0	2.77478483	0.847	0.675	0	Fibroblast
C1R	0	2.738321	0.991	0.863	0	Fibroblast
MMP2	0	2.73094615	0.956	0.67	0	Fibroblast
SCARA5	0	2.71477766	0.914	0.64	0	Fibroblast
SFRP2	0	2.67081337	0.837	0.498	0	Fibroblast
IGF1	0	2.59891307	0.877	0.592	0	Fibroblast
C1S	0	2.52387638	0.977	0.764	0	Fibroblast
ABCA8	0	2.50813085	0.955	0.706	0	Fibroblast
LUM	0	2.45910714	0.937	0.647	0	Fibroblast
ABI3BP	0	2.40915328	0.934	0.66	0	Fibroblast
PODN	0	2.3584504	0.92	0.668	0	Fibroblast
MFAP5	0	2.27236698	0.804	0.558	0	Fibroblast
GAS1	0	2.25294892	0.876	0.609	0	Fibroblast
COL1A2	0	2.25143409	0.978	0.703	0	Fibroblast
FBN1	0	2.20049205	0.913	0.66	0	Fibroblast
IGFBP5	0	2.19379142	0.987	0.857	0	Fibroblast
MT1X	0	2.12197196	0.939	0.779	0	Fibroblast
COL1A1	0	2.11596255	0.944	0.611	0	Fibroblast
MGP	0	2.11432014	0.999	0.962	0	Fibroblast

GPX3	0	2.09502821	0.968	0.832	0	Fibroblast
HTRA3	0	2.09115237	0.863	0.626	0	Fibroblast
COL6A3	0	2.03650478	0.88	0.646	0	Fibroblast
PDGFRA	0	1.98673018	0.83	0.559	0	Fibroblast
PI16	0	1.97158277	0.822	0.704	0	Fibroblast
MT1M	0	1.96341122	0.944	0.865	0	Fibroblast
SFRP1	0	1.96230314	0.842	0.648	0	Fibroblast
OGN	0	1.95039217	0.946	0.651	0	Fibroblast
EFEMP1	0	1.95000684	0.919	0.634	0	Fibroblast
MEG3	0	1.87396041	0.832	0.516	0	Fibroblast
COL14A1	0	1.84862373	0.954	0.755	0	Fibroblast
COL3A1	0	1.8414808	0.924	0.687	0	Fibroblast
ABCA6	0	1.82958107	0.856	0.652	0	Fibroblast
ITGBL1	0	1.82294934	0.89	0.666	0	Fibroblast
PRELP	0	1.76427345	0.922	0.669	0	Fibroblast
ABCA10	0	1.75919571	0.792	0.591	0	Fibroblast
CRISPLD2	0	1.74582778	0.89	0.634	0	Fibroblast
MYOC	0	1.73287979	0.729	0.574	0	Fibroblast
SRPX	0	1.73106749	0.835	0.634	0	Fibroblast
PCOLCE	0	1.7133319	0.912	0.708	0	Fibroblast
TIMP1	0	1.69645007	0.983	0.878	0	Fibroblast
FSTL1	0	1.68579364	0.9	0.595	0	Fibroblast
RSPO3	0	1.65760101	0.793	0.68	0	Fibroblast
IGSF10	0	1.64368685	0.783	0.651	0	Fibroblast
CTSK	0	1.62932906	0.862	0.643	0	Fibroblast
OLFML3	0	1.62344084	0.793	0.503	0	Fibroblast
ABCA9	0	1.61649125	0.773	0.657	0	Fibroblast
NNMT	0	1.59110901	0.926	0.815	0	Fibroblast
FBLN2	0	1.56404036	0.839	0.619	0	Fibroblast
HSP90B1	0	1.54918208	0.969	0.822	0	Fibroblast
SLIT2	0	1.54855942	0.792	0.579	0	Fibroblast
FMO2	0	1.54297458	0.86	0.751	0	Fibroblast
PDGFRL	0	1.53653577	0.795	0.648	0	Fibroblast
CFH	0	1.52261357	0.917	0.724	0	Fibroblast
BICC1	0	1.5195533	0.803	0.63	0	Fibroblast
SEMA3C	0	1.51154045	0.694	0.476	0	Fibroblast
TSHZ2	0	1.49998415	0.9	0.773	0	Fibroblast
VCAN	0	1.49208148	0.904	0.633	0	Fibroblast
PLPP3	0	1.48792111	0.838	0.696	0	Fibroblast
MT1E	0	1.45773364	0.958	0.81	0	Fibroblast
MT2A	0	1.45518331	0.994	0.957	0	Fibroblast
DCLK1	0	1.45126485	0.775	0.589	0	Fibroblast
SPOCK1	0	1.45025059	0.706	0.497	0	Fibroblast
MGST1	0	1.42385295	0.678	0.421	0	Fibroblast
LOX	0	1.40495125	0.742	0.49	0	Fibroblast
LAMA2	0	1.38713516	0.736	0.488	0	Fibroblast
TGFBR3	0	1.3618218	0.876	0.77	0	Fibroblast
GPNMB	0	1.3490713	0.874	0.657	0	Fibroblast
HELLPAR	0	1.33168579	0.684	0.514	0	Fibroblast
SVEP1	0	1.31548695	0.634	0.603	0	Fibroblast
CHRDL1	0	1.30817512	0.782	0.717	0	Fibroblast
IGFBP3	0	1.30435126	0.745	0.792	0	Fibroblast
USP53	0	1.29269196	0.886	0.719	0	Fibroblast

DPT	0	1.28651682	0.693	0.488	0	Fibroblast
AEBP1	0	1.27410471	0.93	0.699	0	Fibroblast
BOC	0	1.26416017	0.71	0.54	0	Fibroblast
GLUL	0	1.25999455	0.958	0.845	0	Fibroblast
FBLN5	0	1.2417054	0.77	0.485	0	Fibroblast
FLRT2	0	1.23593907	0.741	0.617	0	Fibroblast
GPC3	0	1.22813046	0.675	0.562	0	Fibroblast
CPB1	0	1.22775132	0.765	0.607	0	Fibroblast
AKAP12	0	1.19552403	0.885	0.756	0	Fibroblast
SLPI	0	1.15640109	0.508	0.445	0	Fibroblast
RARRES2	0	1.14892818	0.885	0.745	0	Fibroblast
ABCC9	0	1.14443286	0.81	0.692	0	Fibroblast
UAP1	0	1.12857121	0.748	0.437	0	Fibroblast
GFRA1	0	1.12333202	0.708	0.627	0	Fibroblast
CADM3	0	1.08836628	0.826	0.736	0	Fibroblast
MT1A	0	1.08245926	0.813	0.735	0	Fibroblast
NEGR1	0	1.07529119	0.618	0.443	0	Fibroblast
C16orf89	0	1.07228626	0.751	0.677	0	Fibroblast
ADAMTS5	0	1.06672486	0.696	0.537	0	Fibroblast
APOD	0	1.05574599	0.906	0.86	0	Fibroblast
LTBP1	0	1.05494155	0.81	0.654	0	Fibroblast
TNXB	0	1.04711783	0.773	0.665	0	Fibroblast
FST	0	1.02035015	0.68	0.551	0	Fibroblast
PTGIS	0	1.01320734	0.671	0.517	0	Fibroblast
VEGFD	0	0.9949854	0.528	0.524	0	Fibroblast
HHIP	0	0.99155764	0.703	0.516	0	Fibroblast
LINC01697	0	0.98514791	0.741	0.647	0	Fibroblast
CLU	0	0.95756922	0.968	0.903	0	Fibroblast
SFRP4	0	0.93465856	0.659	0.457	0	Fibroblast
PROCR	0	0.90377905	0.799	0.667	0	Fibroblast
THY1	0	0.89467207	0.743	0.633	0	Fibroblast
AOX1	0	0.87916501	0.679	0.521	0	Fibroblast
NTRK2	0	0.87864485	0.853	0.736	0	Fibroblast
CNTFR	0	0.86867702	0.733	0.609	0	Fibroblast
PMP22	0	0.86237497	0.932	0.825	0	Fibroblast
SERPINE2	0	0.86207534	0.602	0.57	0	Fibroblast
PCOLCE2	0	0.86185452	0.59	0.512	0	Fibroblast
AKR1C1	0	0.85265468	0.717	0.454	0	Fibroblast
PTGFR	0	0.85076612	0.586	0.499	0	Fibroblast
RDH10	0	0.85019811	0.742	0.579	0	Fibroblast
LAMB1	0	0.84329	0.692	0.693	0	Fibroblast
CD248	0	0.83764345	0.56	0.396	0	Fibroblast
TNFSF13B	0	0.83638138	0.788	0.587	0	Fibroblast
MSX1	0	0.83424344	0.642	0.454	0	Fibroblast
SPRY1	0	0.82785746	0.84	0.717	0	Fibroblast
H19	0	0.82769288	0.683	0.703	0	Fibroblast
F3	0	0.82668238	0.661	0.545	0	Fibroblast
MT1G	0	0.81846334	0.714	0.474	0	Fibroblast
MEDAG	0	0.81298765	0.59	0.466	0	Fibroblast
VIT	0	0.8123118	0.555	0.458	0	Fibroblast
BMP4	0	0.80393639	0.77	0.493	0	Fibroblast
CD34	0	0.79812644	0.802	0.751	0	Fibroblast
NID1	0	0.78932599	0.637	0.599	0	Fibroblast

CCN5	0	0.78635487	0.684	0.494	0	Fibroblast
OAF	0	0.78461496	0.594	0.434	0	Fibroblast
TMEM45A	0	0.78373418	0.603	0.458	0	Fibroblast
IGFBP4	0	0.76589609	0.934	0.87	0	Fibroblast
THBS2	0	0.76021203	0.705	0.465	0	Fibroblast
EMP1	0	0.76010488	0.786	0.692	0	Fibroblast
OSR1	0	0.75085672	0.549	0.484	0	Fibroblast
LRRC17	0	0.7432981	0.718	0.636	0	Fibroblast
OMD	0	0.74310715	0.713	0.585	0	Fibroblast
PTGDS	0	0.74220098	0.773	0.693	0	Fibroblast
LINC00632	0	0.73857871	0.6	0.465	0	Fibroblast
FMOD	0	0.73759115	0.665	0.48	0	Fibroblast
SNAI2	0	0.7358357	0.676	0.533	0	Fibroblast
HSPB6	0	0.71600893	0.746	0.583	0	Fibroblast
HP	0	0.7100033	0.612	0.447	0	Fibroblast
SPON2	0	0.69162886	0.622	0.503	0	Fibroblast
UCHL1	0	0.69059053	0.757	0.552	0	Fibroblast
BHLHE41	0	0.67082778	0.754	0.565	0	Fibroblast
ACKR3	0	0.64924809	0.617	0.392	0	Fibroblast
FOXD1	0	0.64923626	0.774	0.672	0	Fibroblast
SNED1	0	0.64443025	0.617	0.57	0	Fibroblast
PDK4	0	0.64262691	0.889	0.823	0	Fibroblast
SPON1	0	0.6381628	0.532	0.471	0	Fibroblast
CES1	0	0.63479393	0.687	0.552	0	Fibroblast
BNC2	0	0.63128758	0.605	0.569	0	Fibroblast
ABLIM1	0	0.6195521	0.827	0.794	0	Fibroblast
GPRC5A	0	0.61869029	0.623	0.493	0	Fibroblast
ADGRD1	0	0.61085472	0.515	0.479	0	Fibroblast
CYGB	0	0.60412553	0.712	0.742	0	Fibroblast
WNT5A	0	0.60372021	0.526	0.492	0	Fibroblast
RND3	0	0.60038497	0.671	0.558	0	Fibroblast
SEMA3D	0	0.58192081	0.601	0.542	0	Fibroblast
TSPAN8	0	0.5758002	0.64	0.48	0	Fibroblast
ADGRG2	0	0.56853779	0.549	0.507	0	Fibroblast
TCIM	0	0.56717264	0.746	0.663	0	Fibroblast
EBF2	0	0.54365926	0.583	0.557	0	Fibroblast
CAMK2N1	0	0.54352859	0.667	0.549	0	Fibroblast
LGALS3	0	0.54326743	0.946	0.879	0	Fibroblast
PLTP	0	0.54257864	0.909	0.796	0	Fibroblast
ACKR4	0	0.5423345	0.664	0.588	0	Fibroblast
CYP27A1	0	0.53773623	0.688	0.528	0	Fibroblast
ANK2	0	0.5366901	0.603	0.481	0	Fibroblast
CCDC102B	0	0.52982913	0.524	0.433	0	Fibroblast
GFPT2	0	0.52861947	0.531	0.484	0	Fibroblast
CHI3L2	0	0.52644756	0.638	0.665	0	Fibroblast
CYP1B1	0	0.52478689	0.711	0.53	0	Fibroblast
CCL26	0	0.5182137	0.661	0.482	0	Fibroblast
ECM1	0	0.51646651	0.672	0.566	0	Fibroblast
SULF1	0	0.50291884	0.728	0.548	0	Fibroblast
FGL2	0	0.50005235	0.794	0.567	0	Fibroblast
ELN	0	0.49545789	0.611	0.462	0	Fibroblast
ADAMTS1	0	0.49520342	0.815	0.691	0	Fibroblast
ATF5	0	0.49377688	0.669	0.481	0	Fibroblast

CGNL1	0	0.48947323	0.666	0.582	0	Fibroblast
ADH1C	0	0.47850512	0.582	0.614	0	Fibroblast
RBP4	0	0.47726798	0.506	0.343	0	Fibroblast
OSR2	0	0.47019802	0.503	0.422	0	Fibroblast
CA12	0	0.46083385	0.667	0.4	0	Fibroblast
CENPW	0	0.45944369	0.674	0.462	0	Fibroblast
CYP4B1	0	0.45365436	0.638	0.539	0	Fibroblast
APCDD1	0	0.45212334	0.544	0.513	0	Fibroblast
SPARC	0	0.44356719	0.91	0.82	0	Fibroblast
PCDH17	0	0.43018937	0.72	0.553	0	Fibroblast
FAM180A	0	0.42155938	0.698	0.554	0	Fibroblast
LMO3	0	0.41567361	0.537	0.437	0	Fibroblast
CAMP	0	0.41446033	0.676	0.634	0	Fibroblast
FAIM2	0	0.41206975	0.657	0.506	0	Fibroblast
SOD2	0	0.4065496	0.811	0.693	0	Fibroblast
CLSTN2	0	0.38593349	0.62	0.606	0	Fibroblast
FMO3	0	0.38435242	0.619	0.54	0	Fibroblast
GALNT15	0	0.3714269	0.692	0.734	0	Fibroblast
FKBP11	0	0.36856497	0.741	0.57	0	Fibroblast
PDGFD	0	0.36630253	0.55	0.486	0	Fibroblast
FIBIN	0	0.34006963	0.649	0.587	0	Fibroblast
STEAP1	0	0.33929246	0.546	0.468	0	Fibroblast
LSAMP	0	0.33545051	0.49	0.396	0	Fibroblast
SLC26A7	0	0.32704862	0.562	0.442	0	Fibroblast
PTHLH	0	0.32553741	0.586	0.535	0	Fibroblast
GREM2	0	0.32469008	0.602	0.54	0	Fibroblast
BCAT1	0	0.32007936	0.715	0.649	0	Fibroblast
KCNE4	0	0.29622325	0.613	0.502	0	Fibroblast
INMT	0	0.28902241	0.586	0.544	0	Fibroblast
TCEAL7	0	0.28587645	0.507	0.465	0	Fibroblast
DIO2	0	0.28506561	0.484	0.395	0	Fibroblast
SEMA3B	0	0.28497907	0.613	0.551	0	Fibroblast
BMP3	0	0.28149292	0.688	0.555	0	Fibroblast
LRRN3	0	0.28071376	0.613	0.537	0	Fibroblast
ANGPTL5	0	0.27576468	0.552	0.577	0	Fibroblast
GGT5	0	0.26513289	0.675	0.63	0	Fibroblast
CCDC71L	0	0.25570584	0.733	0.675	0	Fibroblast
BGN	0	0.23845873	0.882	0.735	0	Fibroblast
ANGPTL4	0	0.2333887	0.636	0.646	0	Fibroblast
PHEX	0	0.22757489	0.5	0.437	0	Fibroblast
IRX3	0	0.22317133	0.646	0.449	0	Fibroblast
AKR1C2	0	0.21749334	0.582	0.527	0	Fibroblast
MT1F	0	0.21730421	0.704	0.603	0	Fibroblast
C6	0	0.20056115	0.559	0.553	0	Fibroblast
PDPN	8.99E-302	0.42425697	0.491	0.445	1.80E-298	Fibroblast
LINC01133	1.29E-299	0.46131056	0.443	0.335	2.59E-296	Fibroblast
CSTB	2.33E-293	0.29262142	0.814	0.716	4.66E-290	Fibroblast
CTSL	4.04E-286	0.36183465	0.695	0.641	8.08E-283	Fibroblast
CXCL12	5.20E-285	0.71089145	0.754	0.812	1.04E-281	Fibroblast
FADS1	1.93E-277	0.24099846	0.635	0.601	3.87E-274	Fibroblast
LSP1	5.16E-272	0.3857223	0.502	0.413	1.03E-268	Fibroblast
PLA2G5	8.36E-261	0.21472102	0.46	0.373	1.67E-257	Fibroblast
XBP1	2.47E-257	0.25380783	0.664	0.578	4.93E-254	Fibroblast

CILP	2.40E-246	0.84430496	0.465	0.395	4.80E-243	Fibroblast
CLMP	5.38E-240	0.74108374	0.476	0.417	1.08E-236	Fibroblast
CIDEC	8.28E-238	0.20390428	0.386	0.334	1.66E-234	Fibroblast
FAP	3.38E-231	0.4118801	0.465	0.416	6.75E-228	Fibroblast
COL12A1	8.43E-228	0.56404942	0.521	0.49	1.69E-224	Fibroblast
SCRG1	1.57E-221	0.25799323	0.479	0.464	3.15E-218	Fibroblast
TMEM176A	2.09E-211	0.2350954	0.664	0.671	4.19E-208	Fibroblast
GCHFR	3.44E-211	0.34143459	0.589	0.598	6.87E-208	Fibroblast
PCDH7	4.31E-206	0.29830717	0.481	0.433	8.61E-203	Fibroblast
CRLF1	2.28E-185	0.57955134	0.48	0.435	4.55E-182	Fibroblast
GAS7	7.22E-183	0.52076384	0.584	0.605	1.44E-179	Fibroblast
CPXM1	5.59E-171	0.55896078	0.476	0.483	1.12E-167	Fibroblast
FGF10	1.47E-170	0.39237733	0.441	0.348	2.94E-167	Fibroblast
TMEM100	2.44E-162	0.32600207	0.514	0.541	4.88E-159	Fibroblast
NEO1	1.73E-160	0.25807761	0.525	0.543	3.45E-157	Fibroblast
CRABP2	7.55E-154	0.45151582	0.504	0.518	1.51E-150	Fibroblast
HSD11B1	2.11E-141	0.34335564	0.451	0.42	4.22E-138	Fibroblast
NTM	3.60E-118	0.29018107	0.359	0.501	7.21E-115	Fibroblast
APOC1	1.23E-114	0.38311778	0.46	0.436	2.47E-111	Fibroblast
BCYRN1	1.43E-95	0.28024238	0.498	0.496	2.86E-92	Fibroblast
PCSK5	1.46E-94	0.34863821	0.493	0.533	2.93E-91	Fibroblast
FGF7	1.29E-66	0.47671898	0.519	0.574	2.57E-63	Fibroblast
LINC02381	1.79E-59	0.25714205	0.502	0.507	3.57E-56	Fibroblast
MATN2	1.28E-58	0.24714898	0.491	0.498	2.56E-55	Fibroblast
MXRA5	4.22E-55	0.23941913	0.441	0.451	8.45E-52	Fibroblast
SLIT3	4.13E-50	0.21768268	0.618	0.672	8.27E-47	Fibroblast
PI15	1.02E-45	0.24053121	0.392	0.489	2.04E-42	Fibroblast
TNNT3	9.75E-45	0.53464126	0.405	0.579	1.95E-41	Fibroblast
CDO1	7.47E-43	0.27518096	0.446	0.484	1.49E-39	Fibroblast
STEAP2	2.23E-41	0.34787636	0.383	0.351	4.46E-38	Fibroblast
SAA1	2.61E-40	0.2253806	0.314	0.275	5.22E-37	Fibroblast
PTX3	3.75E-34	0.24553776	0.367	0.324	7.51E-31	Fibroblast
MEGF6	2.97E-26	0.20597687	0.532	0.576	5.93E-23	Fibroblast
MLXIPL	1.35E-22	0.21178821	0.427	0.456	2.70E-19	Fibroblast
G0S2	3.14E-17	0.20214355	0.379	0.389	6.29E-14	Fibroblast
MEST	2.61E-15	0.33905402	0.406	0.458	5.23E-12	Fibroblast
TNFAIP6	2.29E-13	0.38753885	0.36	0.406	4.59E-10	Fibroblast
FGF18	6.17E-11	0.27024666	0.315	0.324	1.23E-07	Fibroblast
HAS2	1.83E-06	0.53950508	0.349	0.36	0.00366774	Fibroblast
SRPX2	1.53E-05	0.38935305	0.399	0.436	0.03052215	Fibroblast
RHOBTB3	0.00059764	0.42405568	0.533	0.664	1	Fibroblast
MEG8	0.00126855	0.40906697	0.386	0.437	1	Fibroblast
C1QA	0	5.10917079	0.971	0.664	0	Mono/Mac
C1QB	0	5.03328658	0.967	0.674	0	Mono/Mac
S100A9	0	4.3703264	0.926	0.682	0	Mono/Mac
AIF1	0	3.95354466	0.945	0.529	0	Mono/Mac
F13A1	0	3.8755069	0.955	0.679	0	Mono/Mac
C1QC	0	3.83700631	0.947	0.53	0	Mono/Mac
S100A8	0	3.79767765	0.688	0.61	0	Mono/Mac
CD163	0	3.77003391	0.945	0.514	0	Mono/Mac
RNASE1	0	3.67728616	0.944	0.734	0	Mono/Mac
TYROBP	0	3.63951642	0.934	0.501	0	Mono/Mac
MS4A6A	0	3.5786446	0.938	0.539	0	Mono/Mac

MRC1	0	3.42886654	0.928	0.595	0	Mono/Mac
CTSS	0	3.28297628	0.915	0.464	0	Mono/Mac
LYZ	0	3.17437069	0.854	0.314	0	Mono/Mac
MS4A7	0	3.17418204	0.897	0.475	0	Mono/Mac
MS4A4A	0	3.12898429	0.928	0.571	0	Mono/Mac
CD14	0	3.11213887	0.921	0.461	0	Mono/Mac
FOLR2	0	3.11050073	0.912	0.612	0	Mono/Mac
CTSB	0	3.0194647	0.942	0.641	0	Mono/Mac
CSF1R	0	2.91243777	0.928	0.524	0	Mono/Mac
STAB1	0	2.89372942	0.932	0.578	0	Mono/Mac
FCER1G	0	2.83052707	0.882	0.442	0	Mono/Mac
CPM	0	2.7958538	0.925	0.607	0	Mono/Mac
VSIG4	0	2.79379921	0.908	0.501	0	Mono/Mac
LYVE1	0	2.76583256	0.903	0.549	0	Mono/Mac
SELENOP	0	2.62092301	0.943	0.839	0	Mono/Mac
LAPTM5	0	2.6045418	0.917	0.5	0	Mono/Mac
ITGB2	0	2.54005803	0.922	0.492	0	Mono/Mac
FTL	0	2.50135876	1	0.996	0	Mono/Mac
C1orf162	0	2.43781498	0.89	0.457	0	Mono/Mac
CYBB	0	2.43317083	0.841	0.412	0	Mono/Mac
RNASET2	0	2.42162687	0.915	0.603	0	Mono/Mac
HCLS1	0	2.41958671	0.93	0.505	0	Mono/Mac
LST1	0	2.41168878	0.798	0.427	0	Mono/Mac
CCL3	0	2.41167738	0.692	0.376	0	Mono/Mac
HLA-DPA1	0	2.40361527	0.958	0.766	0	Mono/Mac
LGMN	0	2.34251985	0.844	0.558	0	Mono/Mac
MAF	0	2.34186943	0.903	0.406	0	Mono/Mac
FCGR2A	0	2.33012983	0.897	0.502	0	Mono/Mac
HLA-DPB1	0	2.31652898	0.929	0.75	0	Mono/Mac
GPR34	0	2.28522413	0.918	0.462	0	Mono/Mac
HLA-DRA	0	2.27720389	0.967	0.827	0	Mono/Mac
PLTP	0	2.22049076	0.942	0.805	0	Mono/Mac
SMAP2	0	2.21792469	0.924	0.548	0	Mono/Mac
PLEK	0	2.18694488	0.855	0.396	0	Mono/Mac
DAB2	0	2.13805923	0.938	0.638	0	Mono/Mac
CTSZ	0	2.13258444	0.929	0.753	0	Mono/Mac
FCGR3A	0	2.1258779	0.784	0.433	0	Mono/Mac
CD74	0	2.11239471	0.974	0.84	0	Mono/Mac
GPX1	0	2.0527197	0.921	0.696	0	Mono/Mac
SLCO2B1	0	2.04794797	0.902	0.517	0	Mono/Mac
SPI1	0	2.04632142	0.838	0.436	0	Mono/Mac
VAMP8	0	2.03548327	0.882	0.458	0	Mono/Mac
MAFB	0	2.01893634	0.924	0.751	0	Mono/Mac
IGSF6	0	1.99601408	0.805	0.404	0	Mono/Mac
CXCL8	0	1.98012083	0.75	0.342	0	Mono/Mac
NPC2	0	1.96336181	0.966	0.789	0	Mono/Mac
SAT1	0	1.94282735	0.962	0.761	0	Mono/Mac
MSR1	0	1.91553983	0.773	0.312	0	Mono/Mac
CD83	0	1.88632775	0.787	0.37	0	Mono/Mac
C5AR1	0	1.88317969	0.776	0.362	0	Mono/Mac
HLA-DRB1	0	1.86258736	0.962	0.793	0	Mono/Mac
HMOX1	0	1.85878243	0.802	0.462	0	Mono/Mac
RGS2	0	1.8565957	0.826	0.491	0	Mono/Mac

MARCO	0	1.84729069	0.89	0.621	0	Mono/Mac
HLA-DMA	0	1.84636791	0.898	0.558	0	Mono/Mac
SLC1A3	0	1.81729592	0.918	0.365	0	Mono/Mac
RNASE6	0	1.81383419	0.83	0.504	0	Mono/Mac
FYB1	0	1.7972516	0.84	0.484	0	Mono/Mac
SRGN	0	1.77949573	0.917	0.596	0	Mono/Mac
LILRB5	0	1.76157344	0.881	0.505	0	Mono/Mac
ALOX5AP	0	1.71863574	0.838	0.462	0	Mono/Mac
C3AR1	0	1.71541291	0.785	0.379	0	Mono/Mac
CYBA	0	1.71038321	0.956	0.82	0	Mono/Mac
MPEG1	0	1.70311449	0.764	0.37	0	Mono/Mac
CPVL	0	1.69159802	0.868	0.532	0	Mono/Mac
ASAH1	0	1.68656538	0.868	0.593	0	Mono/Mac
HLA-DRB5	0	1.68331146	0.883	0.682	0	Mono/Mac
SIGLEC1	0	1.68301971	0.759	0.451	0	Mono/Mac
SLA	0	1.67978068	0.884	0.446	0	Mono/Mac
CTSD	0	1.64620768	0.937	0.82	0	Mono/Mac
HLA-DQA1	0	1.64436097	0.802	0.566	0	Mono/Mac
DUSP6	0	1.64178344	0.882	0.562	0	Mono/Mac
BLVRB	0	1.64024839	0.926	0.734	0	Mono/Mac
1-Mar	0	1.63810009	0.773	0.475	0	Mono/Mac
ARRB2	0	1.62389589	0.79	0.485	0	Mono/Mac
ADAP2	0	1.62014106	0.799	0.422	0	Mono/Mac
IQGAP2	0	1.5991593	0.81	0.534	0	Mono/Mac
MFSD1	0	1.5953328	0.831	0.522	0	Mono/Mac
FCGR2B	0	1.59155068	0.867	0.585	0	Mono/Mac
TTN	0	1.58917465	0.844	0.515	0	Mono/Mac
EVI2B	0	1.58900704	0.822	0.394	0	Mono/Mac
CD84	0	1.58485755	0.786	0.491	0	Mono/Mac
NCKAP1L	0	1.55485333	0.782	0.397	0	Mono/Mac
MTSS1	0	1.55246214	0.908	0.608	0	Mono/Mac
HLA-DMB	0	1.54553146	0.811	0.554	0	Mono/Mac
LAIR1	0	1.53548037	0.743	0.399	0	Mono/Mac
HLA-DRB6	0	1.52549124	0.949	0.799	0	Mono/Mac
CD4	0	1.52313217	0.779	0.335	0	Mono/Mac
UCP2	0	1.51401273	0.774	0.417	0	Mono/Mac
LCP1	0	1.51386078	0.858	0.39	0	Mono/Mac
PLIN2	0	1.51067745	0.854	0.662	0	Mono/Mac
RBM47	0	1.50704728	0.786	0.366	0	Mono/Mac
LIPA	0	1.50462693	0.888	0.592	0	Mono/Mac
IL18	0	1.49918143	0.788	0.431	0	Mono/Mac
PTPRC	0	1.48250539	0.851	0.423	0	Mono/Mac
CD53	0	1.47499312	0.817	0.495	0	Mono/Mac
FCGBP	0	1.47338088	0.822	0.423	0	Mono/Mac
RGS1	0	1.45698469	0.699	0.452	0	Mono/Mac
HLA-DQB1	0	1.45086531	0.745	0.588	0	Mono/Mac
RGS10	0	1.44083396	0.836	0.552	0	Mono/Mac
COTL1	0	1.43584317	0.737	0.479	0	Mono/Mac
KCTD12	0	1.426949	0.945	0.724	0	Mono/Mac
LGALS9	0	1.41775861	0.789	0.502	0	Mono/Mac
GPR183	0	1.40920196	0.775	0.372	0	Mono/Mac
POU2F2	0	1.39717447	0.727	0.409	0	Mono/Mac
CLEC7A	0	1.39225876	0.66	0.331	0	Mono/Mac

ADA2	0	1.38990666	0.73	0.385	0	Mono/Mac
CR1	0	1.37372682	0.867	0.458	0	Mono/Mac
NCF4	0	1.36849997	0.782	0.376	0	Mono/Mac
MNDA	0	1.35973889	0.645	0.339	0	Mono/Mac
SERPINA1	0	1.35953887	0.622	0.356	0	Mono/Mac
IER3	0	1.35286527	0.851	0.625	0	Mono/Mac
ITGAM	0	1.33957526	0.831	0.504	0	Mono/Mac
GPSM3	0	1.31924971	0.791	0.491	0	Mono/Mac
NCF2	0	1.31599904	0.718	0.425	0	Mono/Mac
CTSC	0	1.29465596	0.863	0.667	0	Mono/Mac
LILRB2	0	1.29194369	0.615	0.356	0	Mono/Mac
CD37	0	1.28544776	0.785	0.521	0	Mono/Mac
CLEC10A	0	1.28256676	0.76	0.468	0	Mono/Mac
LILRB3	0	1.26761108	0.671	0.438	0	Mono/Mac
LYN	0	1.26698437	0.709	0.439	0	Mono/Mac
OGFRL1	0	1.26224285	0.864	0.611	0	Mono/Mac
HSPH1	0	1.26210745	0.871	0.546	0	Mono/Mac
TNFAIP2	0	1.26162833	0.736	0.399	0	Mono/Mac
NR4A2	0	1.2510156	0.814	0.541	0	Mono/Mac
FPR1	0	1.24820221	0.662	0.427	0	Mono/Mac
CLEC4E	0	1.24278293	0.834	0.453	0	Mono/Mac
TLR2	0	1.23316753	0.774	0.41	0	Mono/Mac
MYO1F	0	1.23274164	0.698	0.464	0	Mono/Mac
SLC40A1	0	1.23012468	0.856	0.54	0	Mono/Mac
SLC11A1	0	1.22509926	0.615	0.434	0	Mono/Mac
CCR1	0	1.20526399	0.705	0.359	0	Mono/Mac
GMFG	0	1.20462441	0.883	0.682	0	Mono/Mac
SHTN1	0	1.19719152	0.721	0.377	0	Mono/Mac
IFI30	0	1.19564937	0.594	0.54	0	Mono/Mac
LRRC25	0	1.18371657	0.71	0.362	0	Mono/Mac
LCP2	0	1.18145838	0.707	0.435	0	Mono/Mac
TBXAS1	0	1.1786134	0.788	0.468	0	Mono/Mac
CXCL16	0	1.16664021	0.704	0.508	0	Mono/Mac
SLC7A7	0	1.16382137	0.694	0.406	0	Mono/Mac
SYK	0	1.1627027	0.636	0.325	0	Mono/Mac
STXBP2	0	1.16211955	0.607	0.39	0	Mono/Mac
CD86	0	1.16024575	0.708	0.423	0	Mono/Mac
SGK1	0	1.15992351	0.688	0.481	0	Mono/Mac
P2RY13	0	1.15918668	0.749	0.298	0	Mono/Mac
ACSL1	0	1.15907267	0.788	0.417	0	Mono/Mac
IL1B	0	1.15720025	0.586	0.304	0	Mono/Mac
PTPRE	0	1.13430646	0.648	0.416	0	Mono/Mac
JAML	0	1.13425298	0.689	0.276	0	Mono/Mac
RUNX1	0	1.13041467	0.76	0.365	0	Mono/Mac
ACP5	0	1.11753766	0.687	0.438	0	Mono/Mac
TYMP	0	1.11665438	0.809	0.634	0	Mono/Mac
CXCL3	0	1.10620139	0.586	0.415	0	Mono/Mac
AC020656.1	0	1.10573111	0.637	0.36	0	Mono/Mac
IL10RA	0	1.09572222	0.629	0.343	0	Mono/Mac
ARHGAP18	0	1.09459046	0.786	0.667	0	Mono/Mac
CYTH4	0	1.09390025	0.67	0.461	0	Mono/Mac
TNFRSF1B	0	1.07364467	0.746	0.567	0	Mono/Mac
EFHD2	0	1.05611208	0.743	0.515	0	Mono/Mac

RASGEF1B	0	1.05527358	0.636	0.503	0	Mono/Mac
LY86	0	1.04852438	0.712	0.472	0	Mono/Mac
CD209	0	1.04061784	0.793	0.502	0	Mono/Mac
ATP2B1-AS1	0	1.03793358	0.722	0.527	0	Mono/Mac
EMB	0	1.03701596	0.8	0.419	0	Mono/Mac
HCK	0	1.03179813	0.622	0.386	0	Mono/Mac
SNX10	0	1.03042685	0.623	0.306	0	Mono/Mac
PTPN6	0	1.02985355	0.638	0.437	0	Mono/Mac
FUCA1	0	1.02841678	0.816	0.553	0	Mono/Mac
HCST	0	1.01236372	0.797	0.485	0	Mono/Mac
BCAT1	0	1.00401101	0.82	0.639	0	Mono/Mac
FMN1	0	1.00372866	0.781	0.407	0	Mono/Mac
TGFBI	0	1.00128914	0.889	0.632	0	Mono/Mac
IL1R2	0	0.99203644	0.635	0.458	0	Mono/Mac
C2	0	0.97857116	0.742	0.404	0	Mono/Mac
EVI2A	0	0.97764529	0.687	0.365	0	Mono/Mac
HAVCR2	0	0.97577363	0.594	0.405	0	Mono/Mac
HLA-DQA2	0	0.96686974	0.867	0.694	0	Mono/Mac
TNFAIP3	0	0.95367067	0.716	0.443	0	Mono/Mac
FCGR1A	0	0.94988918	0.765	0.429	0	Mono/Mac
PMAIP1	0	0.94490482	0.777	0.449	0	Mono/Mac
TFEC	0	0.94466233	0.586	0.324	0	Mono/Mac
TRIB1	0	0.93788742	0.753	0.504	0	Mono/Mac
CXCL2	0	0.93717474	0.592	0.395	0	Mono/Mac
FAM49B	0	0.93713405	0.674	0.465	0	Mono/Mac
NPL	0	0.93687423	0.596	0.367	0	Mono/Mac
WAS	0	0.90958377	0.629	0.422	0	Mono/Mac
SCN9A	0	0.90243042	0.842	0.497	0	Mono/Mac
LILRB4	0	0.88909639	0.631	0.287	0	Mono/Mac
SLC15A3	0	0.88807759	0.646	0.486	0	Mono/Mac
SLAMF8	0	0.87099719	0.824	0.522	0	Mono/Mac
ALOX5	0	0.8699415	0.67	0.453	0	Mono/Mac
HSPA6	0	0.86617501	0.744	0.512	0	Mono/Mac
FERMT3	0	0.85705602	0.592	0.383	0	Mono/Mac
OTUD1	0	0.85371476	0.75	0.531	0	Mono/Mac
RNF144B	0	0.84362541	0.751	0.498	0	Mono/Mac
DOK2	0	0.83503332	0.646	0.382	0	Mono/Mac
NR4A3	0	0.83299337	0.714	0.438	0	Mono/Mac
CSF2RA	0	0.82171897	0.577	0.384	0	Mono/Mac
CTSH	0	0.79057896	0.821	0.649	0	Mono/Mac
SOD2	0	0.76393966	0.839	0.703	0	Mono/Mac
FGL2	0	0.7619207	0.799	0.595	0	Mono/Mac
GATM	0	0.75976176	0.875	0.443	0	Mono/Mac
TFRC	0	0.74853675	0.619	0.412	0	Mono/Mac
CXCR4	0	0.74733903	0.754	0.536	0	Mono/Mac
RGS18	0	0.7431929	0.575	0.27	0	Mono/Mac
GNG2	0	0.74014315	0.798	0.541	0	Mono/Mac
TCN2	0	0.73983872	0.659	0.551	0	Mono/Mac
SIGLEC10	0	0.73928039	0.75	0.425	0	Mono/Mac
ITPR2	0	0.7387773	0.732	0.525	0	Mono/Mac
PRKCB	0	0.71476943	0.592	0.324	0	Mono/Mac
ARL4C	0	0.69938227	0.727	0.47	0	Mono/Mac
INSIG1	0	0.67853988	0.655	0.449	0	Mono/Mac

ALCAM	0	0.67604347	0.681	0.506	0	Mono/Mac
SORL1	0	0.66799806	0.639	0.417	0	Mono/Mac
CD55	0	0.64846595	0.827	0.606	0	Mono/Mac
TMEM176B	0	0.63922591	0.822	0.663	0	Mono/Mac
GAS2L3	0	0.6369188	0.689	0.464	0	Mono/Mac
THBS1	0	0.62636502	0.757	0.503	0	Mono/Mac
CCL4	0	0.6162414	0.664	0.412	0	Mono/Mac
G0S2	0	0.59587945	0.594	0.35	0	Mono/Mac
CCL18	0	0.59214663	0.615	0.353	0	Mono/Mac
CORO1A	0	0.59204056	0.648	0.423	0	Mono/Mac
CCDC141	0	0.58873303	0.82	0.47	0	Mono/Mac
NAPSB	0	0.55479419	0.562	0.349	0	Mono/Mac
LHFPL2	0	0.55004521	0.633	0.368	0	Mono/Mac
IGSF21	0	0.53815146	0.709	0.442	0	Mono/Mac
HMGB2	0	0.52427803	0.792	0.587	0	Mono/Mac
AD000864.1	0	0.51707876	0.61	0.465	0	Mono/Mac
OAS1	0	0.5139126	0.612	0.461	0	Mono/Mac
JPT1	0	0.51384368	0.708	0.539	0	Mono/Mac
RNU2-63P	0	0.51271827	0.785	0.451	0	Mono/Mac
HLA-B	0	0.49486796	0.978	0.943	0	Mono/Mac
HPGDS	0	0.48872722	0.76	0.331	0	Mono/Mac
HLA-DOA	0	0.4847032	0.811	0.55	0	Mono/Mac
ADAM28	0	0.48177528	0.634	0.384	0	Mono/Mac
DNAJA4	0	0.47425243	0.615	0.421	0	Mono/Mac
KCNQ1OT1	0	0.46240416	0.781	0.536	0	Mono/Mac
ALOX15B	0	0.45798758	0.759	0.36	0	Mono/Mac
CD93	0	0.45112649	0.846	0.646	0	Mono/Mac
BRCA2	0	0.43561333	0.679	0.519	0	Mono/Mac
VMO1	0	0.42347269	0.67	0.525	0	Mono/Mac
KCNMA1	0	0.42240467	0.847	0.519	0	Mono/Mac
TNF	0	0.41600418	0.633	0.4	0	Mono/Mac
CREB5	0	0.41448796	0.699	0.566	0	Mono/Mac
ANKRD22	0	0.4125485	0.716	0.246	0	Mono/Mac
TNFRSF11A	0	0.40797421	0.621	0.315	0	Mono/Mac
RCAN1	0	0.39558197	0.785	0.485	0	Mono/Mac
ETS2	0	0.39168435	0.84	0.648	0	Mono/Mac
MCTP1	0	0.38507565	0.73	0.624	0	Mono/Mac
RNASE2	0	0.36335759	0.331	0.272	0	Mono/Mac
AHSP	0	0.36184266	0.157	0.361	0	Mono/Mac
TREM1	0	0.35543927	0.291	0.308	0	Mono/Mac
PTGS2	0	0.33939048	0.6	0.43	0	Mono/Mac
KPNA2	0	0.3366471	0.767	0.461	0	Mono/Mac
CLECL1	0	0.33447242	0.611	0.372	0	Mono/Mac
ADGRG6	0	0.32436939	0.823	0.51	0	Mono/Mac
SPINT2	0	0.32205262	0.606	0.436	0	Mono/Mac
P2RY14	0	0.31363049	0.686	0.535	0	Mono/Mac
GLUL	0	0.30941302	0.915	0.867	0	Mono/Mac
CLEC4G	0	0.3084654	0.763	0.534	0	Mono/Mac
GIMAP4	0	0.3027172	0.861	0.697	0	Mono/Mac
KCNJ2	0	0.30104097	0.746	0.436	0	Mono/Mac
RETN	0	0.29889611	0.298	0.18	0	Mono/Mac
GADD45G	0	0.25824574	0.889	0.746	0	Mono/Mac
DUSP5	0	0.24848332	0.633	0.453	0	Mono/Mac

SDS	0	0.24601028	0.291	0.187	0	Mono/Mac
CCL8	0	0.24518544	0.583	0.334	0	Mono/Mac
PLD4	0	0.23348907	0.732	0.454	0	Mono/Mac
CENPE	0	0.23198202	0.588	0.417	0	Mono/Mac
CLEC5A	0	0.22904711	0.55	0.3	0	Mono/Mac
FFAR2	0	0.22102924	0.242	0.39	0	Mono/Mac
LMO2	0	0.21801694	0.725	0.64	0	Mono/Mac
CCL13	0	0.2146964	0.806	0.441	0	Mono/Mac
TMEM176A	0	0.21007275	0.803	0.645	0	Mono/Mac
CKAP2	0	0.20835461	0.642	0.465	0	Mono/Mac
CMTM2	0	0.2014926	0.557	0.235	0	Mono/Mac
ABRACL	1.65E-299	0.60868843	0.577	0.384	3.30E-296	Mono/Mac
CSTB	4.76E-296	0.50778772	0.822	0.727	9.51E-293	Mono/Mac
PHACTR1	6.59E-295	1.26663433	0.591	0.488	1.32E-291	Mono/Mac
FCGR3B	3.16E-294	0.38557114	0.547	0.351	6.32E-291	Mono/Mac
CTSL	7.51E-294	0.52277766	0.766	0.635	1.50E-290	Mono/Mac
SMIM25	4.18E-283	0.84761514	0.548	0.354	8.36E-280	Mono/Mac
ZNF331	3.76E-280	0.62724467	0.669	0.564	7.52E-277	Mono/Mac
LUCAT1	2.08E-270	0.63947411	0.351	0.416	4.17E-267	Mono/Mac
PILRA	1.98E-265	0.69095221	0.57	0.428	3.96E-262	Mono/Mac
GPNMB	6.62E-244	0.37296568	0.781	0.701	1.32E-240	Mono/Mac
FCN1	6.60E-240	1.38786769	0.533	0.315	1.32E-236	Mono/Mac
IFI44L	1.84E-238	0.28857783	0.615	0.434	3.67E-235	Mono/Mac
BASP1	1.46E-224	0.97209752	0.587	0.466	2.91E-221	Mono/Mac
FGR	2.51E-220	0.83449749	0.56	0.41	5.03E-217	Mono/Mac
CKS2	1.14E-214	0.32465284	0.597	0.434	2.28E-211	Mono/Mac
PTGER4	3.00E-214	0.57801956	0.582	0.43	6.01E-211	Mono/Mac
CSF3R	1.65E-213	1.05482808	0.544	0.346	3.29E-210	Mono/Mac
SELL	1.64E-210	0.27878308	0.389	0.457	3.28E-207	Mono/Mac
EPSTI1	3.31E-203	0.46049713	0.575	0.407	6.62E-200	Mono/Mac
STAT1	9.65E-202	0.30943545	0.628	0.445	1.93E-198	Mono/Mac
IKZF1	6.11E-194	0.56857927	0.562	0.385	1.22E-190	Mono/Mac
EZR	4.85E-179	0.26171609	0.679	0.542	9.70E-176	Mono/Mac
IRF8	1.22E-177	0.63840608	0.54	0.408	2.44E-174	Mono/Mac
ATP1B1	9.02E-175	0.46095948	0.557	0.402	1.80E-171	Mono/Mac
SAMSN1	2.10E-173	0.98642324	0.542	0.341	4.19E-170	Mono/Mac
LILRA5	4.36E-172	0.38299074	0.368	0.334	8.72E-169	Mono/Mac
GAS7	5.81E-171	0.48611172	0.642	0.592	1.16E-167	Mono/Mac
GAPT	2.24E-161	0.23588175	0.363	0.236	4.48E-158	Mono/Mac
CYTIP	4.89E-157	0.38538847	0.577	0.52	9.78E-154	Mono/Mac
NFKBIA	4.97E-156	0.38682141	0.899	0.89	9.93E-153	Mono/Mac
GLRX	1.73E-154	0.60517335	0.586	0.463	3.46E-151	Mono/Mac
CD38	5.40E-153	0.22704895	0.531	0.361	1.08E-149	Mono/Mac
MMP9	5.98E-150	0.25785713	0.509	0.333	1.20E-146	Mono/Mac
ARHGAP30	2.07E-145	0.72516432	0.556	0.418	4.14E-142	Mono/Mac
WDFY4	8.16E-144	0.3257481	0.376	0.391	1.63E-140	Mono/Mac
CXCL9	1.50E-143	0.21329559	0.323	0.313	3.00E-140	Mono/Mac
CSTA	3.36E-139	0.82035394	0.514	0.318	6.72E-136	Mono/Mac
EREG	2.41E-137	0.55903095	0.504	0.298	4.82E-134	Mono/Mac
SUCNR1	5.83E-127	0.21293118	0.399	0.431	1.17E-123	Mono/Mac
IFIT2	2.88E-123	0.44954589	0.58	0.418	5.76E-120	Mono/Mac
LPAR6	3.26E-116	0.44765062	0.629	0.593	6.53E-113	Mono/Mac
PDE4B	2.25E-115	0.34037845	0.503	0.336	4.50E-112	Mono/Mac

CD300E	5.25E-111	0.41706463	0.349	0.204	1.05E-107	Mono/Mac
PLA2G7	6.39E-109	0.22206302	0.352	0.23	1.28E-105	Mono/Mac
ICAM1	1.42E-106	0.42436726	0.588	0.488	2.85E-103	Mono/Mac
GK	2.03E-104	0.45130017	0.516	0.39	4.06E-101	Mono/Mac
CCL4L2	7.31E-102	0.76594733	0.524	0.393	1.46E-98	Mono/Mac
BIRC3	1.31E-99	0.37153359	0.428	0.513	2.62E-96	Mono/Mac
EHD4	2.59E-98	0.23080904	0.618	0.537	5.18E-95	Mono/Mac
CXCL11	1.56E-95	0.21839743	0.374	0.385	3.13E-92	Mono/Mac
TNFSF13B	6.66E-92	0.40139695	0.632	0.64	1.33E-88	Mono/Mac
ADGRE2	1.24E-90	0.61722316	0.49	0.27	2.48E-87	Mono/Mac
MX2	3.61E-90	0.61488708	0.524	0.379	7.22E-87	Mono/Mac
ITGA4	1.23E-86	0.50445787	0.54	0.407	2.46E-83	Mono/Mac
RAC2	1.16E-85	0.36241751	0.54	0.375	2.31E-82	Mono/Mac
PDK4	1.78E-83	0.21107073	0.891	0.831	3.55E-80	Mono/Mac
APOC1	3.37E-82	0.2769754	0.416	0.447	6.74E-79	Mono/Mac
TACC3	6.57E-78	0.32580384	0.531	0.495	1.31E-74	Mono/Mac
EGR2	8.06E-70	0.36001522	0.388	0.386	1.61E-66	Mono/Mac
ITGAX	1.01E-67	0.71212004	0.494	0.331	2.01E-64	Mono/Mac
PIK3AP1	2.20E-65	0.81209694	0.526	0.494	4.41E-62	Mono/Mac
S100A12	1.69E-64	1.94875063	0.434	0.524	3.37E-61	Mono/Mac
RSAD2	1.95E-64	0.26163375	0.512	0.368	3.89E-61	Mono/Mac
BCL2A1	3.15E-61	1.22822193	0.499	0.347	6.30E-58	Mono/Mac
IL4I1	1.72E-60	0.34058017	0.459	0.209	3.44E-57	Mono/Mac
P2RX7	8.17E-59	0.55921473	0.54	0.526	1.63E-55	Mono/Mac
EIF4E	3.67E-58	0.45140612	0.578	0.496	7.33E-55	Mono/Mac
APOBEC3A	7.87E-58	0.31522033	0.348	0.201	1.57E-54	Mono/Mac
PRDM1	6.57E-57	0.48808449	0.562	0.536	1.31E-53	Mono/Mac
PLAUR	3.34E-56	0.77325705	0.544	0.494	6.68E-53	Mono/Mac
AQP9	1.12E-50	0.34245933	0.365	0.264	2.25E-47	Mono/Mac
IL10	4.82E-49	0.48305922	0.402	0.367	9.64E-46	Mono/Mac
GPRIN3	1.12E-46	0.23616171	0.554	0.53	2.25E-43	Mono/Mac
ANPEP	4.06E-46	0.43757326	0.411	0.383	8.12E-43	Mono/Mac
SOX4	1.42E-42	0.26741568	0.741	0.641	2.84E-39	Mono/Mac
FBP1	2.48E-42	0.57789543	0.514	0.485	4.97E-39	Mono/Mac
NRP2	7.51E-39	0.28521927	0.568	0.527	1.50E-35	Mono/Mac
CX3CR1	1.69E-38	0.21344387	0.489	0.372	3.37E-35	Mono/Mac
SLC16A3	3.21E-37	0.59034358	0.498	0.41	6.42E-34	Mono/Mac
MDM2	1.48E-34	0.30748865	0.554	0.477	2.96E-31	Mono/Mac
HMGA1	2.22E-34	0.3450538	0.442	0.488	4.44E-31	Mono/Mac
C5AR2	2.06E-32	0.50214834	0.516	0.483	4.12E-29	Mono/Mac
CXCL10	1.03E-27	0.52860608	0.435	0.255	2.05E-24	Mono/Mac
SLC6A6	1.66E-27	0.36715423	0.461	0.528	3.33E-24	Mono/Mac
TREM2	5.18E-21	0.67925877	0.397	0.257	1.04E-17	Mono/Mac
CCL3L1	5.95E-19	1.56615617	0.481	0.383	1.19E-15	Mono/Mac
MIR3945HG	6.58E-18	0.29018961	0.426	0.332	1.32E-14	Mono/Mac
B3GNT5	9.51E-17	0.4039437	0.468	0.386	1.90E-13	Mono/Mac
LILRB1	1.93E-14	0.68410226	0.474	0.316	3.86E-11	Mono/Mac
GPR65	4.07E-12	0.32342344	0.492	0.382	8.14E-09	Mono/Mac
PTAFR	9.47E-12	0.61094179	0.439	0.384	1.89E-08	Mono/Mac
DHRS9	2.10E-11	0.32082191	0.45	0.24	4.21E-08	Mono/Mac
PNP	7.11E-11	0.20077919	0.436	0.432	1.42E-07	Mono/Mac
OLR1	2.57E-10	0.46031277	0.444	0.275	5.14E-07	Mono/Mac
FPR3	6.03E-10	0.59011529	0.45	0.251	1.21E-06	Mono/Mac

C15orf48	3.97E-09	0.47379172	0.406	0.33	7.93E-06	Mono/Mac
AZIN1-AS1	7.11E-08	0.21410129	0.463	0.42	0.0001421	Mono/Mac
ATP13A3	8.80E-08	0.21764588	0.462	0.454	0.00017595	Mono/Mac
TAGAP	3.01E-06	0.40215618	0.482	0.414	0.0060233	Mono/Mac
CAPG	4.20E-06	0.47454737	0.479	0.507	0.00839092	Mono/Mac
CXorf21	4.73E-06	0.49680109	0.457	0.273	0.00945865	Mono/Mac
NLRP3	0.00011367	0.59537974	0.42	0.272	0.22734774	Mono/Mac
KYNU	0.00356488	0.56959653	0.425	0.282	1	Mono/Mac
CD48	0.00694178	0.23120672	0.487	0.418	1	Mono/Mac
MYH11	0	3.75757308	0.962	0.618	0	SMC
ACTA2	0	3.5178999	0.981	0.719	0	SMC
TPM2	0	3.34517081	0.982	0.794	0	SMC
RERGL	0	3.26687425	0.941	0.623	0	SMC
TAGLN	0	3.1771589	0.996	0.882	0	SMC
PLN	0	3.16780164	0.909	0.595	0	SMC
MYL9	0	2.97104827	0.986	0.867	0	SMC
DSTN	0	2.71412808	0.978	0.917	0	SMC
C11orf96	0	2.66639559	0.945	0.629	0	SMC
PPP1R14A	0	2.65541908	0.924	0.588	0	SMC
CNN1	0	2.5081479	0.831	0.518	0	SMC
FLNA	0	2.41797879	0.976	0.762	0	SMC
ACTG2	0	2.26066896	0.782	0.478	0	SMC
NEXN	0	2.16412622	0.929	0.642	0	SMC
CSRP2	0	2.13572698	0.847	0.688	0	SMC
LMOD1	0	2.13465686	0.912	0.654	0	SMC
CRIP1	0	2.12190742	0.953	0.757	0	SMC
AP002956.1	0	2.11924501	0.843	0.418	0	SMC
SORBS2	0	2.04784061	0.915	0.669	0	SMC
TPM1	0	2.04373565	0.976	0.851	0	SMC
CCN2	0	2.03604376	0.844	0.653	0	SMC
MYLK	0	1.96573465	0.871	0.518	0	SMC
ITGA8	0	1.93782409	0.761	0.488	0	SMC
RCAN2	0	1.85212133	0.815	0.524	0	SMC
NOTCH3	0	1.84222975	0.944	0.52	0	SMC
CSRP1	0	1.83929528	0.886	0.559	0	SMC
PPP1R12B	0	1.77245752	0.83	0.49	0	SMC
BGN	0	1.7689202	0.95	0.729	0	SMC
CALD1	0	1.73878842	0.987	0.889	0	SMC
FILIP1L	0	1.73240771	0.938	0.72	0	SMC
LPP	0	1.72365509	0.926	0.745	0	SMC
CARMN	0	1.7150615	0.81	0.389	0	SMC
RGS5	0	1.65268071	0.759	0.508	0	SMC
LBH	0	1.64179984	0.779	0.571	0	SMC
CCN1	0	1.62639217	0.889	0.629	0	SMC
PALLD	0	1.61468007	0.929	0.728	0	SMC
CRYAB	0	1.60867033	0.907	0.701	0	SMC
HES4	0	1.60061457	0.898	0.561	0	SMC
NDUFA4L2	0	1.58282509	0.818	0.638	0	SMC
MRVI1	0	1.54908611	0.774	0.482	0	SMC
SNCG	0	1.53506669	0.872	0.711	0	SMC
MAP1B	0	1.51087295	0.905	0.673	0	SMC
C12orf75	0	1.4871018	0.796	0.506	0	SMC
VCL	0	1.45771493	0.871	0.599	0	SMC

DES	0	1.4185253	0.625	0.532	0	SMC
CCN3	0	1.40671088	0.698	0.479	0	SMC
AP000892.3	0	1.39658443	0.756	0.494	0	SMC
KCNAB1	0	1.39221292	0.792	0.466	0	SMC
FN1	0	1.39144595	0.828	0.609	0	SMC
FHL1	0	1.38551186	0.887	0.67	0	SMC
RAMP1	0	1.37527948	0.722	0.451	0	SMC
BCAM	0	1.31724169	0.947	0.705	0	SMC
ACTN1	0	1.31421711	0.87	0.636	0	SMC
GUCY1A1	0	1.29622163	0.828	0.526	0	SMC
FBXO32	0	1.22132235	0.758	0.508	0	SMC
SYNPO2	0	1.19884407	0.825	0.622	0	SMC
ITGA7	0	1.19874166	0.747	0.408	0	SMC
ITIH5	0	1.1975903	0.818	0.583	0	SMC
PDE5A	0	1.18967843	0.812	0.533	0	SMC
TINAGL1	0	1.17824792	0.851	0.669	0	SMC
PHLDA2	0	1.1737358	0.697	0.396	0	SMC
CKB	0	1.16853731	0.766	0.526	0	SMC
RRAD	0	1.16800738	0.795	0.583	0	SMC
TBX2	0	1.15709432	0.731	0.373	0	SMC
CCDC3	0	1.14447828	0.695	0.417	0	SMC
SERPINE1	0	1.12605764	0.74	0.524	0	SMC
TBX2-AS1	0	1.09501919	0.648	0.417	0	SMC
CLMN	0	1.08665477	0.839	0.527	0	SMC
ATP1A2	0	1.08565593	0.678	0.442	0	SMC
MYOCD	0	1.08476827	0.623	0.368	0	SMC
KCNMB1	0	1.04247955	0.638	0.402	0	SMC
LINC00702	0	1.0303387	0.774	0.437	0	SMC
SLIT3	0	1.02935844	0.882	0.603	0	SMC
PDLIM3	0	1.02783327	0.811	0.554	0	SMC
ASPN	0	1.01877764	0.699	0.436	0	SMC
FRZB	0	1.01513675	0.576	0.402	0	SMC
RBPMS2	0	1.00027045	0.736	0.413	0	SMC
NRGN	0	0.99696449	0.7	0.413	0	SMC
KLHL23	0	0.98706318	0.735	0.49	0	SMC
EDNRA	0	0.98400403	0.75	0.467	0	SMC
SMTN	0	0.98239579	0.686	0.569	0	SMC
SYNM	0	0.98021597	0.643	0.503	0	SMC
SORBS1	0	0.96834869	0.678	0.54	0	SMC
EDIL3	0	0.94888933	0.742	0.457	0	SMC
ISYNA1	0	0.91133327	0.712	0.478	0	SMC
NTRK2	0	0.9112378	0.911	0.73	0	SMC
CASQ2	0	0.90923166	0.558	0.334	0	SMC
TNFRSF12A	0	0.90738553	0.574	0.348	0	SMC
TNFRSF11B	0	0.90401636	0.629	0.424	0	SMC
CCND1	0	0.89931755	0.721	0.466	0	SMC
LTBP1	0	0.88767905	0.831	0.661	0	SMC
KCNMA1	0	0.86896344	0.693	0.537	0	SMC
SLC7A2	0	0.85605211	0.887	0.576	0	SMC
NET1	0	0.84953675	0.791	0.679	0	SMC
CRIP2	0	0.84936288	0.884	0.78	0	SMC
COL4A2	0	0.84785691	0.857	0.706	0	SMC
TUBA1C	0	0.83987443	0.807	0.531	0	SMC

PDGFA	0	0.83482641	0.589	0.421	0	SMC
NT5DC2	0	0.83116265	0.666	0.476	0	SMC
ID4	0	0.82630341	0.767	0.442	0	SMC
PRPH	0	0.80007569	0.684	0.476	0	SMC
DKK3	0	0.79333263	0.796	0.551	0	SMC
FBLIM1	0	0.792143	0.769	0.5	0	SMC
FHL5	0	0.78548601	0.791	0.593	0	SMC
CAV1	0	0.7516339	0.956	0.841	0	SMC
INPP4B	0	0.74894962	0.749	0.468	0	SMC
TMEM47	0	0.72944289	0.72	0.549	0	SMC
SUSD5	0	0.72079828	0.601	0.341	0	SMC
PTN	0	0.70217057	0.68	0.479	0	SMC
MAP3K7CL	0	0.69672756	0.716	0.438	0	SMC
CRIM1	0	0.6937882	0.749	0.584	0	SMC
TCF15	0	0.69229373	0.794	0.658	0	SMC
ENTPD3	0	0.68411756	0.673	0.436	0	SMC
COX4I2	0	0.68302367	0.717	0.555	0	SMC
ANGPT1	0	0.66437557	0.643	0.436	0	SMC
SPARC	0	0.65157168	0.905	0.828	0	SMC
MICAL2	0	0.64898202	0.67	0.456	0	SMC
NPY1R	0	0.63273393	0.743	0.45	0	SMC
AEBP1	0	0.63142445	0.826	0.742	0	SMC
MT1L	0	0.6211352	0.773	0.568	0	SMC
HES1	0	0.61748495	0.779	0.562	0	SMC
FOXC2	0	0.61425608	0.704	0.457	0	SMC
GEM	0	0.6123795	0.771	0.455	0	SMC
IGFBP2	0	0.61012785	0.859	0.677	0	SMC
NR2F2	0	0.60358235	0.882	0.731	0	SMC
LGI4	0	0.59845823	0.815	0.572	0	SMC
PMEPA1	0	0.58888886	0.668	0.503	0	SMC
GJA4	0	0.57763146	0.699	0.59	0	SMC
COX7A1	0	0.54605978	0.753	0.727	0	SMC
COL4A1	0	0.54415599	0.763	0.652	0	SMC
PRUNE2	0	0.54401628	0.647	0.424	0	SMC
MBNL1-AS1	0	0.527786	0.646	0.457	0	SMC
ITIH3	0	0.47661309	0.667	0.379	0	SMC
CD9	0	0.46634095	0.822	0.673	0	SMC
RAPGEF5	0	0.46309399	0.73	0.555	0	SMC
GADD45G	0	0.45818265	0.888	0.738	0	SMC
AVPR1A	0	0.45336076	0.828	0.542	0	SMC
KRT14	0	0.43931808	0.673	0.38	0	SMC
RASL11A	0	0.43134144	0.688	0.526	0	SMC
THBS1	0	0.42112801	0.767	0.485	0	SMC
FIBIN	0	0.37318557	0.731	0.571	0	SMC
KRT17	0	0.34452414	0.629	0.344	0	SMC
A2M	0	0.32517501	0.972	0.882	0	SMC
CH25H	0	0.30999133	0.657	0.397	0	SMC
COL14A1	0	0.30700233	0.902	0.783	0	SMC
ANGPT4	0	0.28876155	0.646	0.327	0	SMC
ADGRL3	0	0.28282997	0.755	0.532	0	SMC
FAM162B	0	0.28154888	0.738	0.478	0	SMC
FHL2	0	0.28131963	0.74	0.557	0	SMC
ALKAL2	0	0.2586476	0.662	0.454	0	SMC

PEG10	0	0.22900575	0.672	0.409	0	SMC
VGLL3	0	0.21764774	0.656	0.413	0	SMC
ABRA	0	0.21307453	0.795	0.497	0	SMC
LINC02381	6.74E-307	0.29559135	0.631	0.475	1.35E-303	SMC
ESAM	1.05E-299	0.40629797	0.694	0.632	2.10E-296	SMC
4-Sep	3.25E-292	0.63112979	0.623	0.559	6.50E-289	SMC
CAMK2N1	1.16E-272	0.24367454	0.675	0.556	2.33E-269	SMC
HSPB6	2.15E-250	0.51231619	0.696	0.608	4.29E-247	SMC
AGT	2.15E-250	0.31828948	0.592	0.417	4.31E-247	SMC
HMCN1	2.99E-247	0.35047085	0.589	0.446	5.98E-244	SMC
TFPI2	1.83E-240	0.3726835	0.518	0.334	3.66E-237	SMC
COL8A1	3.37E-220	0.29135051	0.619	0.451	6.75E-217	SMC
MYH10	6.47E-200	0.79888325	0.62	0.508	1.29E-196	SMC
SMOC2	8.67E-191	0.54750284	0.597	0.466	1.73E-187	SMC
GAP43	4.11E-190	0.22808046	0.494	0.363	8.21E-187	SMC
CCL2	2.50E-183	0.23789426	0.591	0.416	5.00E-180	SMC
TPPP3	3.09E-179	0.29815212	0.615	0.485	6.19E-176	SMC
LTBP2	3.14E-176	1.06370113	0.587	0.47	6.27E-173	SMC
WFDC1	1.90E-168	0.63901035	0.527	0.306	3.81E-165	SMC
BCYRN1	3.47E-168	0.22082892	0.435	0.512	6.94E-165	SMC
C5orf46	3.61E-164	0.21340704	0.477	0.302	7.23E-161	SMC
SULF1	4.34E-154	0.38573751	0.683	0.573	8.68E-151	SMC
MGP	4.72E-154	0.42520213	0.99	0.967	9.44E-151	SMC
ACTC1	2.78E-148	0.49800927	0.487	0.274	5.56E-145	SMC
SHROOM3	1.74E-137	0.28159672	0.528	0.355	3.48E-134	SMC
S1PR3	4.76E-131	0.23746257	0.543	0.384	9.52E-128	SMC
APOE	2.44E-130	0.421444	0.696	0.607	4.89E-127	SMC
HEYL	5.13E-124	0.33105484	0.393	0.382	1.03E-120	SMC
SYTL2	8.53E-124	0.54176849	0.543	0.399	1.71E-120	SMC
ITM2C	2.44E-122	0.38057828	0.601	0.524	4.88E-119	SMC
RGS16	3.82E-113	0.34672813	0.642	0.562	7.63E-110	SMC
TSPAN2	8.72E-108	0.2620644	0.432	0.463	1.74E-104	SMC
GLDN	9.26E-108	0.28307712	0.556	0.463	1.85E-104	SMC
LINC00632	3.24E-107	0.23851482	0.605	0.474	6.48E-104	SMC
SCN3A	1.82E-106	0.26023259	0.476	0.196	3.63E-103	SMC
ELN	4.17E-98	0.84280307	0.587	0.48	8.35E-95	SMC
SLC22A3	1.64E-84	0.27280185	0.503	0.355	3.27E-81	SMC
PGF	1.29E-82	0.64906859	0.582	0.546	2.58E-79	SMC
ACAN	2.60E-80	0.34330278	0.505	0.339	5.19E-77	SMC
CYFIP2	3.01E-62	0.2340152	0.542	0.432	6.02E-59	SMC
FOXS1	1.71E-60	0.36463579	0.503	0.328	3.42E-57	SMC
PCDH10	4.60E-56	0.20625596	0.543	0.462	9.20E-53	SMC
ITGA10	1.49E-55	0.30717417	0.627	0.55	2.98E-52	SMC
OMD	4.80E-51	0.36999176	0.59	0.625	9.61E-48	SMC
SUSD2	1.34E-46	0.48533437	0.515	0.401	2.67E-43	SMC
OGN	3.54E-44	0.46126965	0.753	0.721	7.09E-41	SMC
HIGD1B	7.12E-44	0.23226236	0.484	0.552	1.42E-40	SMC
PLCB4	1.80E-33	0.20182788	0.599	0.613	3.59E-30	SMC
OR51E1	1.26E-22	0.42374013	0.519	0.451	2.51E-19	SMC
TNC	5.75E-22	0.35871016	0.483	0.373	1.15E-18	SMC
COMP	3.26E-21	0.32422404	0.458	0.419	6.52E-18	SMC
CDH13	5.79E-21	0.44728284	0.506	0.42	1.16E-17	SMC
TBX3	6.81E-21	0.24304036	0.524	0.46	1.36E-17	SMC

PRSS23	7.04E-17	0.4362678	0.586	0.569	1.41E-13	SMC
PCDH7	8.87E-17	0.33416173	0.499	0.432	1.77E-13	SMC
PGAM2	1.23E-16	0.35386051	0.41	0.29	2.45E-13	SMC
PPP1R1A	1.36E-15	0.36066365	0.48	0.375	2.72E-12	SMC
PTP4A3	9.24E-14	0.53285029	0.483	0.526	1.85E-10	SMC
NRARP	3.02E-11	0.2434678	0.539	0.476	6.04E-08	SMC
VCAN	1.08E-09	0.42463049	0.771	0.686	2.17E-06	SMC
SCUBE3	3.92E-09	0.33835892	0.437	0.388	7.84E-06	SMC
INHBA	2.82E-06	0.58647916	0.438	0.349	0.0056307	SMC
SBSPON	6.30E-06	0.38852368	0.465	0.425	0.01259092	SMC
TCEAL2	8.81E-05	0.23439707	0.494	0.402	0.17618737	SMC
THBS2	0.00073132	0.23141293	0.573	0.516	1	SMC
KCNE4	0.00205	0.35535944	0.525	0.533	1	SMC
HEMGN	1.60E-255	0.75734989	0.997	0.312	3.20E-252	Erythrocyte
RHAG	3.01E-251	0.52722671	0.997	0.261	6.03E-248	Erythrocyte
SPTA1	1.03E-246	0.46361777	0.997	0.233	2.06E-243	Erythrocyte
AHSP	8.67E-246	1.50262337	1	0.328	1.73E-242	Erythrocyte
ALAS2	6.92E-245	0.80907416	1	0.524	1.38E-241	Erythrocyte
AC106865.1	2.23E-241	0.25727465	0.997	0.336	4.45E-238	Erythrocyte
CA1	2.60E-241	1.34356145	0.997	0.174	5.20E-238	Erythrocyte
SLC4A1	3.92E-236	0.77499987	1	0.476	7.83E-233	Erythrocyte
ARHGAP11A	5.34E-216	0.31653635	0.986	0.389	1.07E-212	Erythrocyte
TNMD	6.55E-208	0.26352141	0.992	0.392	1.31E-204	Erythrocyte
SLC2A1	8.52E-204	0.50811428	1	0.461	1.70E-200	Erythrocyte
B3GNT5	1.44E-180	0.23421258	0.994	0.395	2.88E-177	Erythrocyte
PIK3AP1	1.08E-173	0.21437793	0.997	0.497	2.15E-170	Erythrocyte
GDF15	4.03E-173	0.60493022	0.958	0.395	8.07E-170	Erythrocyte
SLAMF8	2.21E-172	0.20880607	1	0.565	4.42E-169	Erythrocyte
CA2	4.24E-155	1.58063487	0.919	0.284	8.48E-152	Erythrocyte
BRCA2	2.08E-148	0.37616538	0.994	0.541	4.16E-145	Erythrocyte
SLPI	3.79E-131	1.56628781	0.93	0.459	7.59E-128	Erythrocyte
SFTPA1	1.61E-130	2.77898297	0.861	0.232	3.22E-127	Erythrocyte
SFTPC	8.26E-127	2.87499697	0.858	0.326	1.65E-123	Erythrocyte
SPOCK2	5.79E-126	0.30046301	0.123	0.484	1.16E-122	Erythrocyte
FADS1	7.12E-126	0.50564108	0.994	0.608	1.42E-122	Erythrocyte
MDM2	8.40E-124	0.59758882	0.994	0.487	1.68E-120	Erythrocyte
ABL2	2.92E-121	0.4140335	0.997	0.526	5.85E-118	Erythrocyte
SFTPA2	5.79E-118	2.3535619	0.844	0.231	1.16E-114	Erythrocyte
RUNX1	1.49E-115	0.40070858	0.992	0.422	2.99E-112	Erythrocyte
DHCR24	1.02E-111	0.97570648	0.872	0.523	2.04E-108	Erythrocyte
CST6	4.51E-108	0.31552656	0.164	0.325	9.02E-105	Erythrocyte
TNC	2.62E-107	0.23897774	0.958	0.392	5.23E-104	Erythrocyte
PCLAF	2.27E-106	0.23151133	0.167	0.488	4.54E-103	Erythrocyte
TENT5C	1.49E-102	0.52519556	0.997	0.485	2.98E-99	Erythrocyte
CADM1	4.12E-100	1.1101847	0.861	0.509	8.23E-97	Erythrocyte
SFRP5	2.70E-99	0.21509619	0.864	0.445	5.39E-96	Erythrocyte
RTKN2	6.66E-99	0.53648922	0.181	0.271	1.33E-95	Erythrocyte
FAM111B	1.51E-93	0.2753447	0.164	0.423	3.02E-90	Erythrocyte
NSG1	5.10E-92	0.21607458	0.17	0.308	1.02E-88	Erythrocyte
ORM1	6.62E-91	0.20862605	0.209	0.433	1.32E-87	Erythrocyte
MALL	2.03E-84	0.78471775	0.844	0.458	4.06E-81	Erythrocyte
LAMP3	4.46E-84	1.10544937	0.772	0.209	8.93E-81	Erythrocyte
NCMAP	7.84E-83	0.21952317	0.206	0.576	1.57E-79	Erythrocyte

PNP	2.46E-79	0.47185004	0.958	0.43	4.92E-76	Erythrocyte
ADGRF5	1.86E-78	0.46414416	0.903	0.729	3.72E-75	Erythrocyte
NPC2	1.29E-74	1.07448658	0.947	0.815	2.58E-71	Erythrocyte
VAMP8	1.54E-71	0.58667185	0.925	0.519	3.08E-68	Erythrocyte
SPINT2	1.88E-69	0.71833986	0.83	0.46	3.77E-66	Erythrocyte
GPM6A	9.09E-68	0.31774656	0.989	0.665	1.82E-64	Erythrocyte
CTSH	1.26E-65	0.7009166	0.919	0.674	2.52E-62	Erythrocyte
CPM	1.00E-62	0.36199051	0.897	0.653	2.00E-59	Erythrocyte
SEMA3E	3.31E-60	0.27961194	0.201	0.532	6.62E-57	Erythrocyte
C16orf89	5.68E-59	0.7293451	0.813	0.695	1.14E-55	Erythrocyte
SEC11C	1.39E-50	0.76128432	0.83	0.613	2.77E-47	Erythrocyte
SLC40A1	2.00E-50	0.3352049	0.986	0.586	3.99E-47	Erythrocyte
WIF1	2.72E-50	1.07544291	0.724	0.183	5.45E-47	Erythrocyte
LMO3	2.34E-49	0.70714485	0.76	0.462	4.67E-46	Erythrocyte
ETS2	1.12E-48	0.38247064	0.994	0.676	2.24E-45	Erythrocyte
BCAT1	2.22E-48	0.21832863	1	0.664	4.45E-45	Erythrocyte
FTL	2.77E-47	0.31965708	1	0.996	5.54E-44	Erythrocyte
CD55	6.56E-47	0.69832341	0.886	0.638	1.31E-43	Erythrocyte
TYMS	8.17E-47	0.21293824	0.167	0.494	1.63E-43	Erythrocyte
FASN	2.13E-44	0.62060136	0.763	0.446	4.25E-41	Erythrocyte
BLVRB	8.74E-43	0.6708346	0.911	0.762	1.75E-39	Erythrocyte
CTSZ	2.16E-42	0.2465409	1	0.778	4.32E-39	Erythrocyte
IRX3	1.68E-40	0.87404258	0.719	0.499	3.37E-37	Erythrocyte
LDLR	1.33E-39	0.59487062	0.766	0.506	2.67E-36	Erythrocyte
SUSD2	1.78E-39	0.61955812	0.741	0.422	3.56E-36	Erythrocyte
ATP1B1	6.32E-37	0.4976435	0.766	0.423	1.26E-33	Erythrocyte
RBM47	1.63E-34	0.7223424	0.719	0.428	3.25E-31	Erythrocyte
HHIP	1.69E-34	1.03503946	0.724	0.564	3.38E-31	Erythrocyte
TFRC	3.29E-34	0.34550613	0.847	0.441	6.59E-31	Erythrocyte
FBP1	7.26E-33	0.59795887	0.716	0.488	1.45E-29	Erythrocyte
MLPH	1.11E-32	0.59092903	0.708	0.428	2.23E-29	Erythrocyte
KRT18	1.93E-32	0.69207857	0.696	0.386	3.86E-29	Erythrocyte
ALCAM	8.50E-32	0.55974054	0.772	0.531	1.70E-28	Erythrocyte
SLC39A8	1.17E-31	0.85485997	0.699	0.437	2.34E-28	Erythrocyte
GPRC5A	7.28E-31	1.02175531	0.741	0.525	1.46E-27	Erythrocyte
PLLP	6.99E-30	0.31333254	0.331	0.567	1.40E-26	Erythrocyte
ANK3	2.99E-29	0.66960282	0.694	0.507	5.98E-26	Erythrocyte
RDX	1.14E-28	0.20783478	0.992	0.77	2.29E-25	Erythrocyte
FABP5	2.35E-28	0.24270742	0.852	0.765	4.69E-25	Erythrocyte
LMO7	3.79E-28	1.08210503	0.685	0.435	7.58E-25	Erythrocyte
ALOX15B	3.90E-28	0.2316219	0.334	0.42	7.79E-25	Erythrocyte
NRGN	6.31E-28	0.53650607	0.73	0.468	1.26E-24	Erythrocyte
SCD	3.61E-26	0.48907383	0.699	0.427	7.23E-23	Erythrocyte
KRT19	1.56E-24	1.32972904	0.657	0.345	3.12E-21	Erythrocyte
SDF2L1	2.58E-24	0.4382971	0.786	0.612	5.16E-21	Erythrocyte
BMP3	3.63E-24	0.84578687	0.666	0.589	7.25E-21	Erythrocyte
CCND1	1.02E-23	0.46724952	0.777	0.515	2.03E-20	Erythrocyte
XBP1	1.06E-23	0.3188232	0.825	0.599	2.12E-20	Erythrocyte
MICAL2	2.46E-23	0.55911582	0.705	0.498	4.92E-20	Erythrocyte
MFSD2A	5.41E-23	0.70426909	0.649	0.268	1.08E-19	Erythrocyte
ALPL	7.11E-23	0.26836495	0.345	0.4	1.42E-19	Erythrocyte
SMC4	1.57E-21	0.49549147	0.666	0.464	3.14E-18	Erythrocyte
MET	2.22E-21	0.33412519	0.691	0.499	4.44E-18	Erythrocyte

COL4A4	5.23E-21	0.41642587	0.348	0.403	1.05E-17	Erythrocyte
PCLO	8.09E-18	0.27192771	0.373	0.291	1.62E-14	Erythrocyte
KLF5	1.58E-16	0.6423467	0.646	0.565	3.16E-13	Erythrocyte
BCYRN1	4.83E-16	0.27400962	0.382	0.497	9.66E-13	Erythrocyte
AQP3	5.06E-16	0.41043985	0.638	0.457	1.01E-12	Erythrocyte
FMN1	1.87E-15	0.49701445	0.635	0.463	3.75E-12	Erythrocyte
TNFSF15	5.62E-13	0.48458671	0.393	0.348	1.12E-09	Erythrocyte
KRT7	7.00E-12	1.59535142	0.401	0.445	1.40E-08	Erythrocyte
TPPP3	9.31E-11	0.91659689	0.632	0.51	1.86E-07	Erythrocyte
DUSP6	2.61E-10	0.30969851	0.735	0.609	5.22E-07	Erythrocyte
SEL1L3	7.39E-10	0.41399899	0.613	0.449	1.48E-06	Erythrocyte
LGALS3	9.94E-10	0.2244039	0.997	0.896	1.99E-06	Erythrocyte
ATP13A3	6.00E-09	0.31727638	0.529	0.455	1.20E-05	Erythrocyte
TSPAN13	2.47E-08	0.47684413	0.643	0.699	4.93E-05	Erythrocyte
C2	8.14E-08	0.45701688	0.61	0.454	0.00016274	Erythrocyte
HOPX	1.21E-07	0.4017832	0.621	0.454	0.0002415	Erythrocyte
CA12	6.43E-07	0.48613827	0.435	0.47	0.00128505	Erythrocyte
GCHFR	2.30E-06	0.29324013	0.641	0.596	0.004595	Erythrocyte
KRT8	7.03E-06	0.44337557	0.588	0.428	0.01405166	Erythrocyte
PEG10	1.50E-05	1.08515925	0.596	0.46	0.03002705	Erythrocyte
TNFRSF12A	1.93E-05	0.58264928	0.607	0.392	0.03854836	Erythrocyte
SFN	2.03E-05	0.49790558	0.437	0.389	0.04059739	Erythrocyte
EHF	2.38E-05	0.51496442	0.443	0.318	0.04758496	Erythrocyte
SHROOM3	3.74E-05	0.39274438	0.582	0.388	0.07474477	Erythrocyte
NEBL	6.14E-05	0.44580502	0.44	0.407	0.12288105	Erythrocyte
SDC1	0.00011149	0.53661522	0.56	0.41	0.22297001	Erythrocyte
CGNL1	0.00013582	0.20434509	0.621	0.604	0.27163968	Erythrocyte
SERPINA1	0.00015883	1.02748247	0.579	0.395	0.317662	Erythrocyte
NEDD9	0.00044011	0.29951541	0.632	0.641	0.880212	Erythrocyte
GALNT5	0.00052868	0.57642835	0.549	0.192	1	Erythrocyte
WFDC2	0.00074759	0.59440017	0.563	0.521	1	Erythrocyte
SLC22A3	0.00183623	0.36468925	0.446	0.384	1	Erythrocyte
BMP2	0.00328343	0.43249235	0.565	0.365	1	Erythrocyte
CLIC3	0.00347253	0.63302409	0.577	0.578	1	Erythrocyte
C19orf33	0.00424301	0.35614055	0.462	0.452	1	Erythrocyte
HMGA1	0.0058974	0.53850723	0.549	0.48	1	Erythrocyte
OCIAD2	0.00714409	0.37964786	0.552	0.535	1	Erythrocyte
F3	0.00750475	0.33910417	0.602	0.575	1	Erythrocyte
PDZK1IP1	0.00967541	0.80470216	0.538	0.305	1	Erythrocyte
ANGPTL7	0	3.65129291	0.702	0.54	0	Epithelial
CDH19	0	3.40293962	0.805	0.423	0	Epithelial
GPM6B	0	3.29197661	0.812	0.602	0	Epithelial
NRXN1	0	3.08760081	0.701	0.433	0	Epithelial
SCN7A	0	2.74739177	0.749	0.549	0	Epithelial
CLDN1	0	1.74374407	0.701	0.38	0	Epithelial
MATN2	0	1.69996324	0.7	0.489	0	Epithelial
PLP1	2.18E-306	3.63429318	0.696	0.593	4.36E-303	Epithelial
TTYH1	6.47E-294	1.25887833	0.648	0.51	1.29E-290	Epithelial
SBSPON	4.65E-288	1.30617956	0.637	0.426	9.31E-285	Epithelial
MPZ	1.96E-283	4.41293077	0.753	0.729	3.92E-280	Epithelial
CHL1	1.93E-270	2.52178897	0.645	0.462	3.87E-267	Epithelial
AATK	1.19E-252	1.52215466	0.629	0.443	2.38E-249	Epithelial
ATP1A2	5.73E-251	1.22771995	0.683	0.482	1.15E-247	Epithelial

SEMA3B	6.60E-251	1.8359648	0.694	0.563	1.32E-247	Epithelial
MAL	8.69E-248	2.13389164	0.708	0.504	1.74E-244	Epithelial
PMP22	1.68E-240	2.27767303	0.91	0.851	3.35E-237	Epithelial
GFRA3	6.22E-223	1.17449327	0.65	0.569	1.24E-219	Epithelial
GAS2L3	1.32E-220	1.41369065	0.636	0.493	2.64E-217	Epithelial
SHOX2	1.12E-217	0.53357646	0.527	0.276	2.24E-214	Epithelial
XKR4	4.40E-214	1.15893329	0.507	0.323	8.81E-211	Epithelial
ITGB8	5.90E-209	2.39160431	0.637	0.532	1.18E-205	Epithelial
OGFRL1	8.79E-205	1.24901299	0.753	0.645	1.76E-201	Epithelial
PLLP	1.31E-203	1.1847577	0.654	0.563	2.61E-200	Epithelial
LGI4	2.57E-203	1.83751544	0.736	0.616	5.13E-200	Epithelial
ABCA8	6.48E-203	0.89023477	0.826	0.768	1.30E-199	Epithelial
COL28A1	1.70E-197	1.80668646	0.638	0.538	3.40E-194	Epithelial
CADM1	2.54E-190	1.47272037	0.696	0.504	5.07E-187	Epithelial
TENM2	5.80E-189	1.08540124	0.415	0.233	1.16E-185	Epithelial
USP53	1.65E-188	1.36542332	0.818	0.761	3.30E-185	Epithelial
TUBB2B	1.59E-187	0.99115902	0.594	0.438	3.17E-184	Epithelial
GAS7	1.64E-181	1.4555998	0.717	0.596	3.28E-178	Epithelial
SEMA5A	8.48E-181	0.6494165	0.677	0.567	1.70E-177	Epithelial
MYOT	8.12E-178	0.83036105	0.34	0.175	1.62E-174	Epithelial
KANK4	1.32E-177	1.13195401	0.57	0.414	2.63E-174	Epithelial
CRYAB	1.26E-172	1.87872339	0.821	0.739	2.52E-169	Epithelial
MT3	1.13E-169	1.94724145	0.724	0.63	2.25E-166	Epithelial
UCHL1	2.60E-167	0.56907725	0.695	0.602	5.20E-164	Epithelial
PDZRN4	3.87E-160	0.70870081	0.514	0.4	7.73E-157	Epithelial
S100B	8.52E-160	3.98568107	0.674	0.599	1.70E-156	Epithelial
SHISA3	1.06E-157	0.2474726	0.562	0.433	2.12E-154	Epithelial
CLU	3.53E-157	0.66050331	0.954	0.918	7.06E-154	Epithelial
INHBA	1.01E-156	0.66118143	0.531	0.361	2.02E-153	Epithelial
PTGDS	1.73E-152	3.86736049	0.738	0.713	3.45E-149	Epithelial
LAMB1	8.58E-152	1.05195068	0.728	0.691	1.72E-148	Epithelial
FBXO32	2.04E-150	0.28469905	0.708	0.552	4.08E-147	Epithelial
NR2F2	3.87E-150	0.73994461	0.814	0.759	7.75E-147	Epithelial
FGFBP2	1.17E-143	1.76987329	0.643	0.612	2.35E-140	Epithelial
APOD	3.85E-140	4.5143636	0.845	0.873	7.69E-137	Epithelial
NLGN4X	9.21E-138	0.7398733	0.575	0.49	1.84E-134	Epithelial
NTM	1.07E-137	1.12004186	0.582	0.46	2.13E-134	Epithelial
SOX10	3.97E-135	1.2853575	0.626	0.52	7.94E-132	Epithelial
RARRES2	2.04E-130	0.95720933	0.798	0.781	4.09E-127	Epithelial
DKK3	2.45E-127	0.83575984	0.683	0.596	4.89E-124	Epithelial
F3	1.73E-125	0.28273664	0.661	0.572	3.45E-122	Epithelial
ITGB4	1.38E-122	1.37941931	0.689	0.708	2.76E-119	Epithelial
CD9	6.29E-119	1.19984247	0.764	0.701	1.26E-115	Epithelial
ITGA6	4.16E-114	1.28173522	0.732	0.741	8.33E-111	Epithelial
RHOBTB3	6.27E-114	0.94789498	0.706	0.627	1.25E-110	Epithelial
CYP1B1	2.30E-111	1.39771442	0.69	0.573	4.60E-108	Epithelial
ABCA9	2.52E-110	0.62019388	0.728	0.686	5.03E-107	Epithelial
CDH1	1.62E-108	0.22781214	0.387	0.226	3.24E-105	Epithelial
SFRP5	1.34E-107	1.05080858	0.536	0.444	2.67E-104	Epithelial
PI16	1.67E-107	1.08041383	0.745	0.735	3.35E-104	Epithelial
VIT	2.23E-105	1.06564213	0.572	0.481	4.46E-102	Epithelial
STXBP6	1.40E-104	0.61536755	0.637	0.566	2.79E-101	Epithelial
LAMA2	1.82E-103	0.68487419	0.636	0.549	3.63E-100	Epithelial

IGSF1	4.24E-101	0.31451352	0.403	0.262	8.47E-98	Epithelial
TMEM100	7.80E-101	0.46243382	0.584	0.533	1.56E-97	Epithelial
MRAS	8.03E-101	1.00745693	0.542	0.421	1.61E-97	Epithelial
MDK	4.18E-100	1.10525023	0.71	0.67	8.37E-97	Epithelial
AKAP12	3.57E-99	0.969582	0.816	0.789	7.14E-96	Epithelial
FGL2	1.80E-97	1.52256847	0.696	0.624	3.60E-94	Epithelial
MMP28	3.70E-97	0.29015503	0.591	0.51	7.41E-94	Epithelial
GPC3	3.06E-96	0.90609489	0.646	0.59	6.13E-93	Epithelial
PCDH9	2.19E-94	1.38534964	0.553	0.46	4.39E-91	Epithelial
VWA1	1.81E-93	1.67269481	0.685	0.668	3.62E-90	Epithelial
MEG3	1.77E-92	0.75217391	0.688	0.595	3.54E-89	Epithelial
BIRC3	3.22E-89	0.44989857	0.604	0.497	6.44E-86	Epithelial
TMPRSS5	8.03E-89	0.59873834	0.551	0.423	1.61E-85	Epithelial
C2orf40	2.16E-88	1.02319265	0.685	0.604	4.31E-85	Epithelial
STMN1	8.61E-88	0.95073726	0.702	0.661	1.72E-84	Epithelial
LPL	1.43E-85	1.35503161	0.569	0.489	2.86E-82	Epithelial
MAP1B	3.19E-83	0.51223375	0.793	0.717	6.38E-80	Epithelial
PDGFRL	7.08E-83	0.58396219	0.73	0.685	1.42E-79	Epithelial
EZR	4.45E-81	0.68500704	0.646	0.56	8.91E-78	Epithelial
ECM1	3.15E-80	0.63087062	0.655	0.591	6.30E-77	Epithelial
FN1	3.86E-79	0.87128831	0.75	0.649	7.72E-76	Epithelial
ALCAM	1.95E-78	0.69263443	0.633	0.529	3.89E-75	Epithelial
CDH7	5.79E-77	0.38307299	0.442	0.259	1.16E-73	Epithelial
FLRT3	1.42E-75	0.62462879	0.538	0.555	2.84E-72	Epithelial
AC104051.2	1.24E-74	0.68292845	0.513	0.503	2.48E-71	Epithelial
NRXN3	9.03E-69	1.19287047	0.427	0.41	1.81E-65	Epithelial
WFDC2	1.10E-68	0.21515483	0.585	0.519	2.20E-65	Epithelial
AQP3	1.20E-68	0.3997279	0.566	0.454	2.40E-65	Epithelial
NCMAP	1.68E-67	0.57448275	0.625	0.572	3.36E-64	Epithelial
FGF7	7.90E-67	0.59856341	0.601	0.559	1.58E-63	Epithelial
TCIM	1.48E-66	0.82632572	0.697	0.684	2.95E-63	Epithelial
PRELP	9.23E-63	0.56276139	0.77	0.733	1.85E-59	Epithelial
RGCC	6.02E-60	0.97646046	0.715	0.668	1.20E-56	Epithelial
SFRP4	4.48E-59	0.74402544	0.584	0.507	8.96E-56	Epithelial
SOX8	4.45E-57	0.87523912	0.513	0.483	8.89E-54	Epithelial
GCGR	3.12E-54	0.28215785	0.472	0.393	6.23E-51	Epithelial
LUM	4.51E-54	1.38253795	0.7	0.723	9.03E-51	Epithelial
SORBS1	6.67E-50	0.93544467	0.592	0.567	1.33E-46	Epithelial
KLF5	1.85E-49	1.32980403	0.575	0.565	3.70E-46	Epithelial
NGFR	3.02E-48	1.00807489	0.535	0.527	6.04E-45	Epithelial
TNNC1	1.28E-46	1.19174998	0.543	0.474	2.57E-43	Epithelial
CSRP1	2.47E-46	0.52383881	0.669	0.623	4.94E-43	Epithelial
SEMA3C	5.07E-46	0.35501721	0.578	0.531	1.01E-42	Epithelial
EPB41L4B	9.91E-46	0.32075405	0.477	0.407	1.98E-42	Epithelial
NR2F1	2.62E-45	1.13509723	0.529	0.499	5.24E-42	Epithelial
ABCA6	4.08E-45	0.43391701	0.7	0.705	8.15E-42	Epithelial
MAG	5.89E-45	0.48177819	0.573	0.603	1.18E-41	Epithelial
P2RY14	5.85E-43	0.58848223	0.527	0.559	1.17E-39	Epithelial
KCNK12	1.85E-42	0.42275436	0.465	0.369	3.70E-39	Epithelial
LGALS3	4.63E-42	0.42682755	0.889	0.897	9.25E-39	Epithelial
ANK3	7.53E-41	1.67011911	0.491	0.508	1.51E-37	Epithelial
ENTPD2	2.65E-40	0.66108813	0.49	0.471	5.30E-37	Epithelial
RELN	2.87E-39	1.45145702	0.575	0.547	5.73E-36	Epithelial

OSR2	6.90E-39	0.48331039	0.453	0.442	1.38E-35	Epithelial
GJC3	1.38E-36	0.75216861	0.464	0.603	2.76E-33	Epithelial
DRP2	1.85E-36	0.53090021	0.543	0.484	3.69E-33	Epithelial
TIMP1	5.00E-36	0.2209749	0.908	0.905	9.99E-33	Epithelial
PMP2	5.32E-36	2.02915509	0.57	0.574	1.06E-32	Epithelial
FLNB	1.09E-35	0.33246097	0.631	0.589	2.18E-32	Epithelial
NRP2	1.69E-34	0.93691605	0.54	0.533	3.38E-31	Epithelial
GNG2	6.54E-34	0.64514098	0.574	0.58	1.31E-30	Epithelial
FLRT2	6.64E-34	0.54331599	0.657	0.649	1.33E-30	Epithelial
GJB2	7.78E-34	0.22808459	0.423	0.313	1.56E-30	Epithelial
PCSK2	2.24E-33	0.85955205	0.421	0.394	4.49E-30	Epithelial
OMD	3.01E-33	0.34133391	0.662	0.616	6.02E-30	Epithelial
CNKSR3	3.23E-32	0.23411299	0.559	0.548	6.46E-29	Epithelial
EBF2	7.57E-31	0.55966456	0.572	0.563	1.51E-27	Epithelial
IFI6	1.12E-29	0.35548659	0.594	0.532	2.24E-26	Epithelial
TSPAN5	1.68E-29	0.4514089	0.576	0.619	3.36E-26	Epithelial
ABCA10	1.75E-29	0.56410642	0.617	0.644	3.51E-26	Epithelial
PSAT1	1.80E-29	0.25331518	0.34	0.472	3.60E-26	Epithelial
SOX2	2.88E-29	1.30034517	0.439	0.415	5.75E-26	Epithelial
APCDD1	3.56E-29	0.29030468	0.556	0.52	7.13E-26	Epithelial
CLMP	5.92E-29	0.31081516	0.458	0.432	1.18E-25	Epithelial
SPARC	5.14E-25	0.64454584	0.806	0.844	1.03E-21	Epithelial
CDO1	6.22E-25	0.67931831	0.464	0.475	1.24E-21	Epithelial
CFH	2.39E-24	0.72706399	0.746	0.775	4.78E-21	Epithelial
FAM43A	3.61E-24	0.3056911	0.628	0.589	7.21E-21	Epithelial
ALDH1A3	8.99E-24	0.30414894	0.444	0.511	1.80E-20	Epithelial
SLC22A3	2.40E-23	0.96100226	0.372	0.385	4.80E-20	Epithelial
IGFBP6	5.35E-23	0.59415895	0.848	0.919	1.07E-19	Epithelial
PRG4	1.08E-22	0.22312129	0.192	0.368	2.15E-19	Epithelial
THBS4	1.40E-22	0.90757085	0.468	0.527	2.79E-19	Epithelial
S1PR3	7.76E-22	0.53001684	0.459	0.414	1.55E-18	Epithelial
SOX9	3.71E-21	0.6594489	0.31	0.304	7.42E-18	Epithelial
THSD7A	1.63E-20	0.65259494	0.547	0.581	3.27E-17	Epithelial
OAF	1.90E-20	0.65421496	0.497	0.474	3.79E-17	Epithelial
MBP	2.06E-20	2.01757773	0.66	0.665	4.13E-17	Epithelial
GLDN	2.10E-20	0.83953646	0.504	0.481	4.20E-17	Epithelial
RETREG1	2.69E-20	0.72801996	0.481	0.485	5.37E-17	Epithelial
COL4A2	5.35E-20	0.26244363	0.743	0.736	1.07E-16	Epithelial
SOX4	8.07E-20	0.54726101	0.663	0.656	1.61E-16	Epithelial
RNF157	8.43E-20	0.41182069	0.466	0.436	1.69E-16	Epithelial
CAPN6	1.68E-19	0.41993677	0.485	0.538	3.37E-16	Epithelial
FSTL5	3.19E-19	0.30604326	0.303	0.124	6.39E-16	Epithelial
CREB5	1.04E-18	0.26460846	0.615	0.585	2.07E-15	Epithelial
PEG10	4.15E-18	0.42389448	0.499	0.46	8.31E-15	Epithelial
SNED1	7.00E-18	0.3446327	0.582	0.583	1.40E-14	Epithelial
TJP1	1.54E-17	0.70184303	0.664	0.716	3.09E-14	Epithelial
BNC2	4.25E-17	0.93956437	0.577	0.578	8.49E-14	Epithelial
MME	2.66E-16	0.21245337	0.424	0.536	5.32E-13	Epithelial
MFAP5	4.29E-16	0.6322755	0.612	0.622	8.58E-13	Epithelial
COL4A1	5.78E-16	0.28733525	0.669	0.674	1.16E-12	Epithelial
DCX	1.04E-15	0.20281961	0.421	0.338	2.08E-12	Epithelial
BICC1	3.37E-15	0.23386701	0.622	0.676	6.73E-12	Epithelial
IL17B	5.78E-15	0.25461167	0.458	0.405	1.16E-11	Epithelial

FOXS1	6.32E-15	0.61468165	0.345	0.363	1.26E-11	Epithelial
ATOH8	9.45E-15	0.30306058	0.494	0.616	1.89E-11	Epithelial
MT2A	1.47E-14	0.24586757	0.969	0.966	2.95E-11	Epithelial
MEOX2	3.09E-14	0.59197059	0.509	0.553	6.19E-11	Epithelial
IQGAP2	4.44E-14	0.5442786	0.574	0.576	8.88E-11	Epithelial
GABRA2	6.09E-14	0.2789924	0.435	0.571	1.22E-10	Epithelial
L1TD1	1.54E-13	0.41137376	0.294	0.403	3.09E-10	Epithelial
PCDH10	1.96E-13	0.38832122	0.464	0.478	3.92E-10	Epithelial
CDH11	2.80E-13	0.45222878	0.44	0.423	5.60E-10	Epithelial
IFIT3	6.39E-13	0.37347884	0.466	0.468	1.28E-09	Epithelial
SCN9A	1.23E-12	0.81753082	0.528	0.549	2.46E-09	Epithelial
LSAMP	1.51E-12	0.34239978	0.345	0.423	3.02E-09	Epithelial
BAMBI	3.38E-12	0.21006558	0.451	0.439	6.76E-09	Epithelial
VGLL3	9.89E-12	0.46840856	0.366	0.464	1.98E-08	Epithelial
SLC12A2	3.42E-11	0.65529432	0.497	0.544	6.84E-08	Epithelial
CYP4B1	1.09E-10	0.27918292	0.437	0.569	2.18E-07	Epithelial
NRN1	1.23E-10	0.31511635	0.572	0.692	2.46E-07	Epithelial
THBS1	1.59E-10	0.32692171	0.568	0.54	3.19E-07	Epithelial
CCL2	3.97E-10	0.23089479	0.476	0.45	7.94E-07	Epithelial
TSPAN8	5.62E-10	0.79527121	0.5	0.522	1.12E-06	Epithelial
ERBB3	1.33E-09	1.14422174	0.437	0.577	2.66E-06	Epithelial
FIBIN	1.62E-09	0.77658761	0.55	0.605	3.25E-06	Epithelial
SORCS1	5.36E-09	1.58340001	0.492	0.567	1.07E-05	Epithelial
RND3	1.68E-08	0.58962137	0.547	0.589	3.35E-05	Epithelial
LINC00632	3.04E-08	0.49519148	0.492	0.5	6.08E-05	Epithelial
TMEM176A	3.67E-08	0.65252653	0.68	0.669	7.34E-05	Epithelial
PHLDA1	5.89E-08	0.20156111	0.432	0.436	0.00011772	Epithelial
RCAN1	9.52E-08	0.3717601	0.433	0.533	0.0001904	Epithelial
EFNB1	2.36E-07	0.26135355	0.535	0.558	0.00047167	Epithelial
C1QL1	3.21E-07	0.47842013	0.455	0.499	0.00064122	Epithelial
SLC2A1	3.49E-07	1.0942511	0.434	0.465	0.00069868	Epithelial
CYP27A1	3.54E-07	0.25112262	0.52	0.571	0.00070791	Epithelial
HES1	3.81E-07	0.27132988	0.484	0.609	0.00076103	Epithelial
CSTB	4.64E-07	0.26922262	0.713	0.742	0.00092846	Epithelial
TMEM176B	5.94E-07	0.73242772	0.598	0.69	0.00118828	Epithelial
ANXA3	9.96E-07	0.24348577	0.448	0.572	0.00199181	Epithelial
AZGP1	3.01E-06	1.01228886	0.492	0.602	0.00602775	Epithelial
C19orf33	5.51E-06	0.38533515	0.314	0.457	0.01102126	Epithelial
MLIP	5.64E-06	0.8911868	0.321	0.403	0.01128596	Epithelial
ADAMTS8	1.28E-05	0.37621939	0.37	0.42	0.0255006	Epithelial
PRSS23	1.57E-05	0.31618133	0.574	0.572	0.03136364	Epithelial
BMP3	2.04E-05	0.49604323	0.514	0.592	0.04082382	Epithelial
COL8A1	2.82E-05	0.45061609	0.45	0.486	0.0563199	Epithelial
COL5A3	2.84E-05	0.3633525	0.318	0.364	0.05684694	Epithelial
LIMCH1	4.96E-05	0.76159017	0.512	0.644	0.09913659	Epithelial
MOXD1	8.15E-05	0.25911742	0.298	0.25	0.16290667	Epithelial
IFIT2	9.42E-05	0.22262599	0.419	0.443	0.18834968	Epithelial
RDX	0.0003847	0.31431541	0.707	0.774	0.76939887	Epithelial
NEGR1	0.00042465	0.29287295	0.463	0.489	0.84929904	Epithelial
SLC15A3	0.00056036	0.42960737	0.485	0.511	1	Epithelial
UGT8	0.00065437	0.92341251	0.424	0.509	1	Epithelial
TNNT2	0.00091905	0.44459653	0.357	0.381	1	Epithelial
RDH10	0.001211	0.42333216	0.512	0.625	1	Epithelial

KRT19	0.00207829	1.03385488	0.236	0.35	1	Epithelial
PTN	0.00267749	0.22994045	0.435	0.521	1	Epithelial
CHRDL1	0.00304227	0.32059151	0.629	0.738	1	Epithelial
LMO7	0.00519104	0.65009585	0.354	0.439	1	Epithelial
CCL5	0	4.85170211	0.939	0.463	0	T_cell
NKG7	0	4.32224303	0.97	0.63	0	T_cell
GNLY	0	4.12896865	0.95	0.659	0	T_cell
CCL4	0	3.50736571	0.842	0.414	0	T_cell
CD69	0	3.16657981	0.868	0.424	0	T_cell
CD52	0	3.09027848	0.922	0.4	0	T_cell
CXCR4	0	3.01618582	0.922	0.536	0	T_cell
GZMA	0	2.9262726	0.889	0.466	0	T_cell
HCST	0	2.80723315	0.94	0.494	0	T_cell
CD3E	0	2.71605192	0.836	0.409	0	T_cell
CTSW	0	2.70855542	0.922	0.561	0	T_cell
CD3D	0	2.63425643	0.726	0.352	0	T_cell
CD2	0	2.51415522	0.745	0.332	0	T_cell
IL32	0	2.51330031	0.873	0.685	0	T_cell
KLRB1	0	2.45164907	0.648	0.424	0	T_cell
PTPRC	0	2.41030383	0.898	0.45	0	T_cell
TRBC1	0	2.40054213	0.657	0.38	0	T_cell
GZMB	0	2.39336875	0.909	0.499	0	T_cell
CST7	0	2.36804708	0.748	0.449	0	T_cell
IFNG	0	2.29737099	0.903	0.485	0	T_cell
GZMH	0	2.2743736	0.934	0.505	0	T_cell
LCP1	0	2.26592878	0.944	0.416	0	T_cell
KLRD1	0	2.23084406	0.958	0.536	0	T_cell
CORO1A	0	2.22243905	0.842	0.421	0	T_cell
GZMM	0	2.20041608	0.915	0.447	0	T_cell
TRBC2	0	2.18620997	0.701	0.36	0	T_cell
PRF1	0	2.17741195	0.886	0.418	0	T_cell
DUSP2	0	2.17312703	0.787	0.41	0	T_cell
CD7	0	2.16333644	0.809	0.508	0	T_cell
BCL11B	0	2.16001038	0.72	0.432	0	T_cell
RUNX3	0	2.1526213	0.735	0.404	0	T_cell
RAC2	0	2.13065316	0.75	0.367	0	T_cell
CD3G	0	2.06788072	0.659	0.393	0	T_cell
SRGN	0	2.03535197	0.931	0.618	0	T_cell
PCED1B-AS1	0	1.97369513	0.742	0.493	0	T_cell
CD48	0	1.94223769	0.738	0.4	0	T_cell
ARL4C	0	1.90463047	0.712	0.49	0	T_cell
ISG20	0	1.89741871	0.827	0.496	0	T_cell
PYHIN1	0	1.86672804	0.873	0.348	0	T_cell
NR4A2	0	1.85945447	0.816	0.561	0	T_cell
GZMK	0	1.77265329	0.643	0.336	0	T_cell
CLIC3	0	1.77086312	0.919	0.547	0	T_cell
CD247	0	1.71904147	0.647	0.366	0	T_cell
LCK	0	1.65943193	0.677	0.47	0	T_cell
TRAC	0	1.59717755	0.631	0.393	0	T_cell
GPR65	0	1.56963647	0.703	0.37	0	T_cell
IKZF3	0	1.55670137	0.68	0.323	0	T_cell
FYB1	0	1.52604365	0.731	0.52	0	T_cell
ETS1	0	1.52111046	0.795	0.609	0	T_cell

MATK	0	1.50684254	0.91	0.43	0	T_cell
CD8A	0	1.45714578	0.687	0.325	0	T_cell
XCL2	0	1.4558807	0.778	0.391	0	T_cell
SAMD3	0	1.42493123	0.803	0.353	0	T_cell
CD37	0	1.38652494	0.706	0.547	0	T_cell
CD53	0	1.37403078	0.696	0.529	0	T_cell
ITGA4	0	1.36552822	0.725	0.4	0	T_cell
TRGC2	0	1.35839502	0.672	0.307	0	T_cell
DOK2	0	1.33320315	0.668	0.399	0	T_cell
PLAC8	0	1.31683346	0.779	0.327	0	T_cell
CD8B	0	1.26344508	0.677	0.264	0	T_cell
CYTIP	0	1.25590587	0.635	0.518	0	T_cell
PTGER4	0	1.23005733	0.72	0.429	0	T_cell
ITGB2	0	1.22090502	0.831	0.532	0	T_cell
AC243960.1	0	1.18911131	0.767	0.429	0	T_cell
SLA	0	1.16084531	0.693	0.495	0	T_cell
MBP	0	1.14429913	0.856	0.647	0	T_cell
TRDC	0	1.10659019	0.835	0.464	0	T_cell
CYBA	0	1.10237926	0.934	0.832	0	T_cell
ARHGAP30	0	1.09822926	0.75	0.41	0	T_cell
GMFG	0	1.08969042	0.74	0.709	0	T_cell
TPSAB1	0	1.07079152	0.14	0.525	0	T_cell
EFHD2	0	1.06581545	0.883	0.519	0	T_cell
FAM49B	0	1.06521821	0.753	0.473	0	T_cell
SPON2	0	1.06168136	0.754	0.513	0	T_cell
FGFBP2	0	1.05012342	0.926	0.584	0	T_cell
RIPOR2	0	1.03036646	0.68	0.434	0	T_cell
NFKBIA	0	1.0217764	0.892	0.891	0	T_cell
IL2RB	0	1.01128184	0.642	0.272	0	T_cell
WAS	0	0.99857638	0.706	0.43	0	T_cell
LBH	0	0.94345496	0.743	0.6	0	T_cell
CYFIP2	0	0.92370574	0.705	0.43	0	T_cell
LAIR2	0	0.90641424	0.886	0.412	0	T_cell
PRKCB	0	0.8994944	0.762	0.328	0	T_cell
UCP2	0	0.89759402	0.786	0.441	0	T_cell
HLA-B	0	0.82949596	0.983	0.945	0	T_cell
GPR171	0	0.80530206	0.299	0.285	0	T_cell
KLRC1	0	0.75977447	0.728	0.308	0	T_cell
TNFRSF18	0	0.758439	0.872	0.58	0	T_cell
KLRC2	0	0.75714392	0.842	0.472	0	T_cell
LAX1	0	0.75572608	0.775	0.464	0	T_cell
KLRF1	0	0.72716955	0.834	0.399	0	T_cell
EOMES	0	0.72346311	0.63	0.273	0	T_cell
NR4A3	0	0.70096064	0.751	0.454	0	T_cell
TIGIT	0	0.64812181	0.698	0.491	0	T_cell
NCR3	0	0.63598363	0.829	0.423	0	T_cell
LY9	0	0.6149671	0.594	0.213	0	T_cell
CD160	0	0.60197025	0.842	0.513	0	T_cell
TMIGD2	0	0.54689446	0.653	0.311	0	T_cell
SDF2L1	0	0.53802676	0.842	0.591	0	T_cell
TRGC1	0	0.52917201	0.602	0.256	0	T_cell
SLAMF7	0	0.49056643	0.641	0.263	0	T_cell
PLEK	0	0.48348683	0.79	0.435	0	T_cell

HPGD	0	0.45288986	0.312	0.416	0	T_cell
FCGR3A	0	0.45008403	0.765	0.46	0	T_cell
CX3CR1	0	0.44271828	0.897	0.344	0	T_cell
CCL3L1	0	0.43563224	0.717	0.368	0	T_cell
LINC02446	0	0.41316275	0.761	0.303	0	T_cell
PPP1R2C	0	0.40389799	0.897	0.465	0	T_cell
JAML	0	0.38320551	0.315	0.34	0	T_cell
ZBP1	0	0.36189074	0.704	0.274	0	T_cell
ADGRG1	0	0.34114623	0.91	0.7	0	T_cell
SORL1	0	0.32385031	0.309	0.463	0	T_cell
TNFRSF4	0	0.32207176	0.707	0.579	0	T_cell
ATAD2	0	0.31204615	0.688	0.427	0	T_cell
MT1F	0	0.31114813	0.379	0.652	0	T_cell
ZNF683	0	0.30966533	0.739	0.452	0	T_cell
RNU2-63P	0	0.30573801	0.796	0.474	0	T_cell
SELL	0	0.30310018	0.732	0.42	0	T_cell
MT1G	0	0.29926316	0.372	0.551	0	T_cell
CD28	0	0.29502695	0.187	0.309	0	T_cell
CENPK	0	0.28808452	0.666	0.482	0	T_cell
AURKA	0	0.27642301	0.68	0.467	0	T_cell
STXBP2	0	0.27182704	0.684	0.399	0	T_cell
EPSTI1	0	0.26107871	0.672	0.41	0	T_cell
RTKN2	0	0.25986502	0.68	0.233	0	T_cell
SNX10	0	0.23530761	0.793	0.313	0	T_cell
MIR155HG	0	0.23298498	0.308	0.385	0	T_cell
ENC1	0	0.22583906	0.722	0.427	0	T_cell
SMAP2	1.02E-306	0.77830735	0.718	0.594	2.05E-303	T_cell
IL2RG	9.77E-304	1.5897055	0.61	0.319	1.95E-300	T_cell
LTB	1.91E-302	1.4776765	0.619	0.435	3.83E-299	T_cell
LCP2	5.75E-277	0.88562513	0.645	0.46	1.15E-273	T_cell
CCL3	1.17E-276	1.1151962	0.65	0.402	2.34E-273	T_cell
SLFN5	1.55E-268	0.92835422	0.732	0.57	3.09E-265	T_cell
MS4A2	8.30E-266	0.29144967	0.037	0.321	1.66E-262	T_cell
ZNF331	2.89E-261	1.75584609	0.663	0.572	5.77E-258	T_cell
CPA3	2.94E-259	0.62451032	0.38	0.509	5.88E-256	T_cell
SPOCK2	8.38E-253	0.91640444	0.351	0.495	1.68E-249	T_cell
PCNA	1.26E-251	0.32684846	0.712	0.499	2.53E-248	T_cell
TPSB2	4.84E-247	1.07424716	0.587	0.308	9.67E-244	T_cell
PIM1	1.34E-244	0.75649589	0.632	0.42	2.68E-241	T_cell
TAGAP	4.50E-234	1.39424007	0.604	0.407	9.00E-231	T_cell
SYTL3	3.64E-233	1.37057707	0.6	0.466	7.27E-230	T_cell
GPR183	2.66E-229	0.63895518	0.359	0.439	5.31E-226	T_cell
IL7R	4.23E-224	2.30759557	0.588	0.362	8.45E-221	T_cell
RUNX1	1.95E-216	0.24194914	0.644	0.404	3.89E-213	T_cell
PRDM1	4.28E-213	1.2987297	0.624	0.532	8.55E-210	T_cell
CCL4L2	3.82E-207	1.24230053	0.594	0.396	7.65E-204	T_cell
IL10RA	1.10E-202	0.56086378	0.359	0.388	2.20E-199	T_cell
KIF20B	4.19E-201	0.27257927	0.645	0.457	8.38E-198	T_cell
HOPX	2.01E-199	1.42534925	0.604	0.441	4.02E-196	T_cell
HMGA1	4.98E-189	0.38170462	0.373	0.491	9.96E-186	T_cell
OCIAD2	1.24E-188	0.57187642	0.384	0.549	2.48E-185	T_cell
TNFAIP3	5.13E-183	1.50475194	0.614	0.472	1.03E-179	T_cell
PDE4B	2.47E-180	0.78544377	0.333	0.364	4.94E-177	T_cell

GNG2	3.40E-178	0.99644426	0.655	0.573	6.80E-175	T_cell
SKAP1	5.00E-170	1.4494659	0.583	0.477	1.00E-166	T_cell
IKZF1	1.53E-169	1.5176134	0.587	0.395	3.06E-166	T_cell
EZH2	6.78E-166	0.35791334	0.58	0.394	1.36E-162	T_cell
HIST1H1D	8.46E-166	0.60096593	0.391	0.496	1.69E-162	T_cell
GPSM3	1.29E-162	1.10617401	0.624	0.528	2.59E-159	T_cell
CTSC	5.66E-155	0.21840356	0.831	0.684	1.13E-151	T_cell
EMB	7.51E-155	1.06271579	0.609	0.464	1.50E-151	T_cell
OSM	9.56E-151	0.38178288	0.549	0.314	1.91E-147	T_cell
FCMR	1.60E-144	1.00887642	0.574	0.409	3.20E-141	T_cell
JPT1	2.48E-143	0.95241383	0.647	0.556	4.96E-140	T_cell
XCL1	1.30E-142	1.006766	0.551	0.237	2.61E-139	T_cell
FKBP11	2.47E-142	0.34920129	0.47	0.627	4.94E-139	T_cell
HSPA6	4.12E-142	0.66224877	0.46	0.555	8.23E-139	T_cell
AQP3	5.12E-141	0.43058745	0.382	0.465	1.02E-137	T_cell
LAPTM5	4.13E-138	0.470554	0.676	0.552	8.25E-135	T_cell
EZR	3.86E-135	1.15057861	0.626	0.557	7.71E-132	T_cell
PHLDA1	9.74E-133	0.51377458	0.609	0.42	1.95E-129	T_cell
ICOS	8.99E-132	0.65544854	0.35	0.173	1.80E-128	T_cell
TNFRSF9	2.07E-128	0.20633641	0.544	0.354	4.14E-125	T_cell
DNAJA4	9.64E-125	0.36738046	0.385	0.456	1.93E-121	T_cell
SH2D1A	1.72E-124	0.95291521	0.549	0.253	3.44E-121	T_cell
TSPAN5	1.15E-121	0.37633061	0.651	0.614	2.30E-118	T_cell
EGR2	1.03E-116	0.26482461	0.551	0.371	2.06E-113	T_cell
CYTH4	1.79E-116	0.42056662	0.605	0.482	3.58E-113	T_cell
PTPRE	3.20E-107	0.34885336	0.612	0.436	6.39E-104	T_cell
TENT5C	3.91E-107	0.81535936	0.407	0.494	7.82E-104	T_cell
SLC18A2	1.96E-106	0.24738969	0.433	0.298	3.91E-103	T_cell
TACC3	2.04E-106	0.24278836	0.592	0.492	4.07E-103	T_cell
HSPH1	9.43E-106	0.63392965	0.675	0.587	1.89E-102	T_cell
MYO1F	3.04E-104	0.6492168	0.604	0.49	6.07E-101	T_cell
LINC00892	3.32E-104	0.54379045	0.555	0.477	6.64E-101	T_cell
RASGEF1B	1.79E-103	0.27442018	0.439	0.531	3.58E-100	T_cell
CKS2	1.30E-102	1.00667169	0.58	0.448	2.59E-99	T_cell
PIM2	4.62E-95	0.54983833	0.387	0.433	9.24E-92	T_cell
GLRX	3.36E-94	0.21779833	0.417	0.487	6.72E-91	T_cell
EIF4E	1.26E-93	0.22046812	0.664	0.495	2.51E-90	T_cell
DUSP4	1.56E-92	0.60422578	0.536	0.37	3.12E-89	T_cell
SPN	2.96E-78	0.94992998	0.545	0.409	5.91E-75	T_cell
CTSG	1.31E-76	0.20405876	0.658	0.584	2.63E-73	T_cell
CDC42EP3	7.98E-70	0.39288321	0.451	0.523	1.60E-66	T_cell
PTPN6	4.27E-69	0.71163049	0.571	0.457	8.53E-66	T_cell
IQGAP2	2.76E-63	0.84338385	0.596	0.574	5.52E-60	T_cell
RNF157	1.37E-57	0.29226324	0.544	0.427	2.75E-54	T_cell
EVI2A	7.14E-57	0.49452672	0.425	0.413	1.43E-53	T_cell
RGS2	1.47E-56	0.32738009	0.466	0.548	2.94E-53	T_cell
RHOH	1.55E-56	1.35206951	0.537	0.432	3.11E-53	T_cell
CD55	7.90E-51	0.3762368	0.514	0.65	1.58E-47	T_cell
CD84	1.49E-50	0.3091951	0.46	0.542	2.99E-47	T_cell
TNFRSF1B	2.74E-49	0.36990644	0.663	0.588	5.48E-46	T_cell
GCHFR	3.55E-49	0.24134182	0.53	0.602	7.11E-46	T_cell
SAT1	8.26E-47	0.21107031	0.798	0.791	1.65E-43	T_cell
GIMAP7	9.93E-47	0.40350722	0.694	0.777	1.99E-43	T_cell

TFRC	5.89E-46	0.58097346	0.563	0.432	1.18E-42	T_cell
SLAMF1	3.46E-43	0.60644363	0.372	0.224	6.92E-40	T_cell
PNP	8.14E-42	0.53865612	0.409	0.435	1.63E-38	T_cell
NCKAP1L	1.04E-41	0.39643874	0.569	0.445	2.09E-38	T_cell
SLC2A1	6.31E-41	0.23712622	0.537	0.457	1.26E-37	T_cell
EVI2B	3.58E-40	0.6433583	0.449	0.459	7.15E-37	T_cell
ARHGAP18	1.15E-37	0.26500092	0.723	0.681	2.30E-34	T_cell
SLC2A3	1.34E-34	0.56582216	0.628	0.593	2.69E-31	T_cell
ISG15	5.47E-34	0.52150689	0.65	0.593	1.09E-30	T_cell
HIST1H1C	1.35E-33	0.20970239	0.592	0.453	2.70E-30	T_cell
CKAP2	2.70E-33	0.38985106	0.554	0.486	5.40E-30	T_cell
HCLS1	4.76E-33	0.4613084	0.606	0.565	9.51E-30	T_cell
PMAIP1	7.59E-32	0.73578301	0.46	0.502	1.52E-28	T_cell
ALOX5AP	1.38E-31	0.54944539	0.467	0.523	2.76E-28	T_cell
DUSP5	1.68E-31	0.80645598	0.546	0.474	3.35E-28	T_cell
HIST1H4C	2.67E-29	0.45053808	0.726	0.767	5.33E-26	T_cell
SEC11C	3.56E-29	0.25206844	0.563	0.619	7.13E-26	T_cell
FERMT3	4.54E-28	0.58460854	0.53	0.403	9.08E-25	T_cell
MARCKSL1	7.85E-28	0.65712103	0.576	0.534	1.57E-24	T_cell
CYTOR	8.58E-26	0.77568078	0.537	0.435	1.72E-22	T_cell
CCR7	4.17E-25	0.42000916	0.408	0.346	8.33E-22	T_cell
LSP1	2.27E-24	0.89729485	0.527	0.427	4.53E-21	T_cell
KCNQ1OT1	3.23E-22	0.34679073	0.678	0.563	6.46E-19	T_cell
SMC4	8.91E-21	0.33385151	0.558	0.456	1.78E-17	T_cell
C12orf75	4.07E-20	0.25526292	0.618	0.559	8.14E-17	T_cell
COTL1	8.07E-20	0.5719152	0.572	0.513	1.61E-16	T_cell
AREG	4.53E-19	1.60659107	0.505	0.371	9.07E-16	T_cell
TNFSF14	4.91E-19	0.57983159	0.446	0.462	9.82E-16	T_cell
CRTAM	9.54E-19	0.8597821	0.53	0.48	1.91E-15	T_cell
ATP2B1-AS1	1.75E-18	0.51492662	0.609	0.552	3.49E-15	T_cell
ABCB1	2.66E-17	0.24471778	0.455	0.422	5.31E-14	T_cell
LINC01871	1.84E-16	0.81945131	0.492	0.296	3.68E-13	T_cell
IL2RA	5.50E-15	0.37961869	0.497	0.363	1.10E-11	T_cell
TNF	1.07E-14	1.15602178	0.509	0.429	2.14E-11	T_cell
AC016074.2	2.78E-13	0.58548389	0.447	0.428	5.56E-10	T_cell
GPRIN3	1.89E-12	0.59434066	0.487	0.538	3.79E-09	T_cell
SIT1	4.47E-11	0.64398902	0.45	0.464	8.94E-08	T_cell
XBP1	8.54E-11	0.64528042	0.601	0.6	1.71E-07	T_cell
STMN1	1.61E-10	0.5343371	0.632	0.665	3.22E-07	T_cell
PTTG1	8.14E-09	0.5057879	0.479	0.488	1.63E-05	T_cell
OASL	1.58E-08	0.37072965	0.429	0.316	3.15E-05	T_cell
KPNA2	4.37E-08	0.72076028	0.541	0.504	8.74E-05	T_cell
RGCC	7.64E-08	0.369813	0.541	0.682	0.00015281	T_cell
GBP5	1.04E-07	0.67332507	0.477	0.321	0.00020851	T_cell
VAMP8	2.27E-07	0.49069547	0.564	0.517	0.00045402	T_cell
BIRC3	3.10E-07	1.06614358	0.467	0.503	0.00061929	T_cell
RGS1	8.32E-07	1.01665478	0.476	0.49	0.00166342	T_cell
PTPN7	9.01E-07	1.31165195	0.488	0.349	0.00180189	T_cell
HMGB2	0.00018114	0.29372962	0.573	0.622	0.36228188	T_cell
BLK	0	1.79672922	0.95	0.556	0	B_cell
IGKV3-11	0	0.46095655	0.904	0.288	0	B_cell
IGHV3-7	2.61E-302	2.04047174	0.906	0.292	5.22E-299	B_cell
MS4A1	9.02E-299	3.3587925	0.927	0.413	1.80E-295	B_cell

IGLV1-40	1.76E-297	1.36656329	0.913	0.53	3.51E-294	B_cell
IGKV1-27	3.33E-288	0.28084043	0.832	0.331	6.66E-285	B_cell
IGHGP	6.48E-288	0.50505769	0.913	0.595	1.30E-284	B_cell
IGHV3-23	2.24E-284	1.17397314	0.897	0.359	4.49E-281	B_cell
ISG20	3.06E-262	2.47594251	0.963	0.521	6.12E-259	B_cell
IGHD	1.57E-261	2.19127999	0.867	0.373	3.14E-258	B_cell
FCRL5	1.08E-255	1.63317105	0.881	0.294	2.17E-252	B_cell
CD52	1.70E-250	2.76030116	0.979	0.44	3.39E-247	B_cell
AC243960.1	5.19E-245	1.12483319	0.924	0.454	1.04E-241	B_cell
LY9	5.63E-240	1.4240101	0.869	0.241	1.13E-236	B_cell
SIT1	4.57E-236	0.72784665	0.894	0.46	9.14E-233	B_cell
FCRLA	1.25E-230	0.83847611	0.846	0.36	2.51E-227	B_cell
CCNB1	3.89E-230	0.32724348	0.901	0.53	7.79E-227	B_cell
DUSP4	1.63E-223	0.2275256	0.894	0.38	3.27E-220	B_cell
IGHM	8.87E-220	8.4429031	0.89	0.389	1.77E-216	B_cell
TNFRSF13C	1.63E-219	1.53822571	0.853	0.351	3.26E-216	B_cell
CD79A	2.50E-215	3.17136876	0.871	0.448	5.01E-212	B_cell
POU2F2	1.11E-211	2.14736011	0.91	0.454	2.21E-208	B_cell
GINS2	1.24E-206	0.21919125	0.913	0.533	2.49E-203	B_cell
PYHIN1	4.29E-189	0.25724509	0.881	0.389	8.59E-186	B_cell
IGLV1-47	9.03E-176	1.02096452	0.803	0.32	1.81E-172	B_cell
CD37	7.24E-174	2.79734361	0.917	0.558	1.45E-170	B_cell
CLECL1	3.13E-173	0.91348245	0.833	0.405	6.26E-170	B_cell
IKZF3	7.03E-167	1.63080124	0.839	0.349	1.41E-163	B_cell
IGLC3	1.88E-166	7.92696396	0.8	0.249	3.76E-163	B_cell
CENPM	5.84E-160	0.47040091	0.823	0.446	1.17E-156	B_cell
CD86	1.68E-158	0.25386001	0.869	0.463	3.37E-155	B_cell
LAPTM5	8.11E-157	1.61668561	0.954	0.56	1.62E-153	B_cell
IGLC2	2.33E-155	8.36772291	0.807	0.294	4.66E-152	B_cell
SPIB	1.74E-154	1.06181093	0.789	0.324	3.47E-151	B_cell
CXCR4	4.25E-153	1.93581175	0.897	0.566	8.51E-150	B_cell
SMAP2	6.19E-153	1.43191203	0.949	0.602	1.24E-149	B_cell
NCR3	1.56E-152	0.32559601	0.835	0.455	3.11E-149	B_cell
IGLC7	8.98E-152	0.27357761	0.761	0.246	1.80E-148	B_cell
LAX1	1.93E-150	0.77686491	0.819	0.488	3.86E-147	B_cell
CORO1A	2.71E-148	1.53010574	0.881	0.454	5.43E-145	B_cell
PNOC	1.55E-146	0.71712473	0.768	0.357	3.10E-143	B_cell
GPR65	3.19E-144	0.71124698	0.858	0.395	6.37E-141	B_cell
BASP1	6.84E-143	1.24922246	0.895	0.481	1.37E-139	B_cell
LTB	2.86E-140	2.4616849	0.833	0.448	5.72E-137	B_cell
LMNB1	1.79E-136	0.30914837	0.812	0.366	3.58E-133	B_cell
FKBP11	2.57E-135	1.69804684	0.911	0.612	5.13E-132	B_cell
IGKC	6.37E-129	7.64082709	0.77	0.306	1.27E-125	B_cell
CD79B	8.09E-126	1.7183458	0.872	0.67	1.62E-122	B_cell
SEL1L3	1.18E-122	1.405385	0.78	0.447	2.35E-119	B_cell
IRF4	8.35E-121	1.08744169	0.755	0.294	1.67E-117	B_cell
BANK1	1.07E-117	1.74686675	0.771	0.416	2.14E-114	B_cell
IGHG3	3.22E-117	6.03254603	0.727	0.192	6.45E-114	B_cell
MZB1	3.76E-109	3.65724437	0.73	0.278	7.53E-106	B_cell
HLA-DPA1	1.69E-108	1.19794069	0.993	0.793	3.38E-105	B_cell
IRF8	9.98E-107	1.09524105	0.785	0.425	2.00E-103	B_cell
CYTOR	1.50E-102	1.28114437	0.848	0.441	3.00E-99	B_cell
PTPN6	6.78E-102	1.32352588	0.794	0.465	1.36E-98	B_cell

ALOX5	2.08E-101	0.38560037	0.809	0.483	4.17E-98	B_cell
NR4A3	1.50E-100	0.59535672	0.895	0.476	3.00E-97	B_cell
HLA-DQA2	4.32E-100	0.78402579	0.885	0.718	8.63E-97	B_cell
IGHV1-18	8.60E-100	0.68051299	0.555	0.109	1.72E-96	B_cell
CLSPN	4.75E-95	0.27199849	0.785	0.524	9.50E-92	B_cell
CYBA	1.06E-94	1.1505732	0.936	0.84	2.12E-91	B_cell
LGALS9	4.15E-94	0.27285139	0.894	0.542	8.29E-91	B_cell
ANKRD36BF	3.67E-93	0.92734007	0.722	0.384	7.34E-90	B_cell
MYBL2	1.95E-89	0.45964521	0.706	0.328	3.90E-86	B_cell
TNFRSF13B	2.01E-89	1.15465892	0.7	0.177	4.01E-86	B_cell
HLA-DPB1	7.01E-88	1.48951521	0.977	0.776	1.40E-84	B_cell
FCMR	9.86E-88	1.56305763	0.757	0.421	1.97E-84	B_cell
CD24	2.34E-84	0.83460434	0.23	0.367	4.68E-81	B_cell
COL4A4	2.99E-82	0.24434407	0.748	0.4	5.98E-79	B_cell
NAPSB	8.64E-82	0.78495552	0.734	0.378	1.73E-78	B_cell
PCLAF	6.24E-81	0.32947087	0.794	0.484	1.25E-77	B_cell
LINC01480	6.49E-81	0.98914192	0.723	0.482	1.30E-77	B_cell
CADM1	1.24E-79	0.35782456	0.853	0.508	2.48E-76	B_cell
IGHV4-59	3.58E-78	0.69828764	0.661	0.083	7.15E-75	B_cell
CPNE5	8.12E-78	1.05526399	0.723	0.545	1.62E-74	B_cell
PCDH9	8.83E-78	0.36244331	0.828	0.46	1.77E-74	B_cell
CD74	3.16E-74	0.98374997	0.968	0.859	6.33E-71	B_cell
HLA-DMB	5.94E-72	0.73979331	0.84	0.591	1.19E-68	B_cell
PIM2	1.58E-70	0.96864134	0.693	0.428	3.16E-67	B_cell
RGS1	1.01E-69	0.97758029	0.761	0.487	2.02E-66	B_cell
ASF1B	2.71E-69	0.20386678	0.805	0.518	5.41E-66	B_cell
PIK3AP1	2.32E-67	0.21665159	0.246	0.501	4.65E-64	B_cell
HLA-DRB5	9.09E-67	0.93936788	0.902	0.71	1.82E-63	B_cell
BIRC3	3.07E-66	1.59262785	0.745	0.498	6.15E-63	B_cell
IGLV3-1	1.38E-65	0.64787542	0.257	0.095	2.77E-62	B_cell
IGHA1	6.30E-64	6.46581148	0.665	0.303	1.26E-60	B_cell
CD38	1.03E-63	0.81230987	0.684	0.385	2.06E-60	B_cell
CCR7	1.26E-63	0.76214889	0.255	0.352	2.51E-60	B_cell
CYB5D2	1.47E-63	0.20959005	0.826	0.577	2.94E-60	B_cell
WAS	2.17E-63	0.5388387	0.748	0.451	4.34E-60	B_cell
VPREB3	3.82E-62	0.76518719	0.27	0.207	7.64E-59	B_cell
RNASET2	9.59E-62	0.51869714	0.879	0.648	1.92E-58	B_cell
CYTIP	2.17E-59	1.10792149	0.722	0.527	4.34E-56	B_cell
TK1	6.19E-59	0.21478989	0.709	0.385	1.24E-55	B_cell
ALOX5AP	8.09E-59	0.2441781	0.787	0.516	1.62E-55	B_cell
IGLV6-57	3.63E-57	1.08843535	0.651	0.27	7.26E-54	B_cell
TYMS	6.49E-57	0.23210434	0.718	0.491	1.30E-53	B_cell
CCDC141	2.87E-55	0.25058501	0.757	0.521	5.73E-52	B_cell
PDE4B	3.87E-55	1.07122252	0.69	0.359	7.73E-52	B_cell
NUF2	1.14E-54	0.20032502	0.746	0.405	2.29E-51	B_cell
TNFRSF17	1.97E-54	0.90069889	0.635	0.165	3.93E-51	B_cell
MIR155HG	2.80E-54	0.25165428	0.229	0.38	5.59E-51	B_cell
BCL2A1	3.49E-54	1.00010086	0.683	0.368	6.98E-51	B_cell
ASPM	5.11E-54	0.48027482	0.716	0.444	1.02E-50	B_cell
GPR183	5.12E-54	1.32799342	0.713	0.431	1.02E-50	B_cell
SEC11C	3.00E-53	2.06977113	0.752	0.613	5.99E-50	B_cell
JPT1	3.19E-53	0.72666211	0.809	0.562	6.38E-50	B_cell
HLA-DRA	4.89E-53	1.57388217	0.984	0.847	9.79E-50	B_cell

PLEK	1.58E-51	0.49401976	0.759	0.463	3.15E-48	B_cell
AL139020.1	2.10E-51	1.01186052	0.316	0.411	4.20E-48	B_cell
COTL1	6.13E-51	0.67897812	0.807	0.515	1.23E-47	B_cell
RAC2	2.82E-50	0.96021016	0.707	0.397	5.63E-47	B_cell
HLA-DRB1	1.85E-49	0.69948666	0.963	0.817	3.69E-46	B_cell
IGKV4-1	1.18E-48	0.99630443	0.633	0.265	2.35E-45	B_cell
AURKB	5.45E-48	0.21386325	0.317	0.501	1.09E-44	B_cell
POU2AF1	5.36E-47	1.25993995	0.635	0.239	1.07E-43	B_cell
SOX4	8.90E-46	0.82784928	0.897	0.654	1.78E-42	B_cell
OTUD1	1.43E-45	0.5194427	0.775	0.563	2.85E-42	B_cell
HLA-B	7.64E-45	0.56453208	0.984	0.948	1.53E-41	B_cell
SLAMF7	8.94E-45	1.09769837	0.637	0.292	1.79E-41	B_cell
CENPF	2.25E-44	0.34283343	0.849	0.482	4.51E-41	B_cell
PTPRC	1.68E-43	0.89573836	0.752	0.485	3.37E-40	B_cell
CD83	1.16E-42	1.56677924	0.704	0.431	2.32E-39	B_cell
ZBP1	1.86E-42	0.77057547	0.631	0.308	3.72E-39	B_cell
STMN1	5.61E-41	0.66145981	0.872	0.661	1.12E-37	B_cell
LBH	1.58E-40	0.30061323	0.863	0.61	3.16E-37	B_cell
ACP5	9.10E-40	0.39041774	0.697	0.474	1.82E-36	B_cell
ATP2B1-AS1	1.56E-39	0.70865899	0.778	0.555	3.12E-36	B_cell
EVI2B	3.56E-39	1.03999019	0.697	0.456	7.13E-36	B_cell
ARHGAP30	2.83E-38	0.62186043	0.686	0.437	5.66E-35	B_cell
EZR	3.36E-36	1.16815125	0.727	0.561	6.71E-33	B_cell
VAMP8	3.36E-35	0.21163051	0.77	0.52	6.73E-32	B_cell
GMFG	4.89E-34	0.47868731	0.819	0.711	9.78E-31	B_cell
IGHV3-74	5.49E-34	0.61732973	0.596	0.196	1.10E-30	B_cell
ST6GAL1	2.30E-33	0.71085375	0.748	0.676	4.60E-30	B_cell
KCNQ1OT1	2.54E-33	0.66647427	0.826	0.571	5.07E-30	B_cell
IGHV3-33	2.94E-33	0.30618071	0.319	0.008	5.89E-30	B_cell
PIM1	4.80E-33	0.28016129	0.697	0.436	9.60E-30	B_cell
HIST1H1B	3.78E-32	0.26515972	0.599	0.28	7.57E-29	B_cell
IGLV2-14	6.60E-32	0.63707141	0.294	0.115	1.32E-28	B_cell
NRARP	6.82E-32	0.28446001	0.661	0.488	1.36E-28	B_cell
DUSP5	1.05E-31	0.54657731	0.688	0.478	2.10E-28	B_cell
KLF2	7.24E-30	0.56516654	0.949	0.81	1.45E-26	B_cell
HIST1H1D	8.77E-30	0.50442954	0.665	0.486	1.75E-26	B_cell
NR4A2	1.98E-29	0.38132865	0.8	0.581	3.97E-26	B_cell
HLA-DQB1	2.78E-29	1.69505775	0.715	0.611	5.57E-26	B_cell
GAPT	5.79E-29	0.61489615	0.619	0.252	1.16E-25	B_cell
MDM2	9.67E-29	0.38868808	0.704	0.487	1.93E-25	B_cell
PCED1B-AS1	2.78E-28	0.68534052	0.656	0.513	5.57E-25	B_cell
JCHAIN	2.05E-27	4.37513092	0.569	0.24	4.10E-24	B_cell
IGKV3-20	2.10E-27	0.93197327	0.534	0.174	4.20E-24	B_cell
IGHV4-39	1.03E-26	0.74898202	0.589	0.269	2.06E-23	B_cell
PLD4	1.28E-26	0.27636434	0.362	0.497	2.56E-23	B_cell
MX1	1.58E-26	0.24014055	0.75	0.479	3.17E-23	B_cell
PHACTR1	1.33E-25	1.29711527	0.649	0.502	2.65E-22	B_cell
MKI67	1.55E-25	0.39294479	0.59	0.37	3.09E-22	B_cell
CYTH4	7.40E-25	0.24497662	0.663	0.491	1.48E-21	B_cell
ETS1	6.79E-24	0.38247325	0.775	0.624	1.36E-20	B_cell
GPSM3	8.01E-24	0.6494408	0.679	0.535	1.60E-20	B_cell
HELLS	8.68E-24	0.32974929	0.66	0.505	1.74E-20	B_cell
BIRC5	1.18E-23	0.21531136	0.709	0.442	2.37E-20	B_cell

KLHL14	3.36E-23	0.5731521	0.587	0.356	6.71E-20	B_cell
IGHG1	3.81E-23	5.37543347	0.576	0.308	7.62E-20	B_cell
IGHV4-34	4.49E-23	0.34330282	0.371	0.392	8.99E-20	B_cell
JSRP1	2.11E-21	0.35363184	0.36	0.428	4.22E-18	B_cell
LINC02397	2.86E-21	0.87417763	0.371	0.469	5.72E-18	B_cell
CKAP2	2.87E-20	0.33318559	0.371	0.493	5.75E-17	B_cell
ITM2C	2.27E-19	1.09069172	0.638	0.538	4.54E-16	B_cell
CD69	2.61E-19	1.46749007	0.619	0.46	5.23E-16	B_cell
ITGA4	4.79E-19	0.44728451	0.362	0.428	9.59E-16	B_cell
HMGA1	7.78E-19	0.74530396	0.615	0.48	1.56E-15	B_cell
TAGAP	1.65E-18	0.60922395	0.365	0.424	3.29E-15	B_cell
AD000864.1	2.48E-18	0.8344202	0.355	0.488	4.96E-15	B_cell
HIST1H1C	3.14E-18	0.6073366	0.652	0.463	6.27E-15	B_cell
LINC01781	9.64E-17	0.57428536	0.374	0.13	1.93E-13	B_cell
DERL3	1.11E-16	1.55920725	0.598	0.543	2.21E-13	B_cell
SYK	3.00E-16	0.51641063	0.362	0.372	6.01E-13	B_cell
SDC1	4.95E-16	0.52362498	0.574	0.409	9.91E-13	B_cell
LSP1	1.71E-15	0.76695902	0.608	0.434	3.42E-12	B_cell
HLA-DQA1	4.99E-15	1.3555037	0.656	0.601	9.98E-12	B_cell
CXorf21	7.30E-15	0.31567216	0.564	0.298	1.46E-11	B_cell
NUSAP1	8.86E-15	0.23561826	0.535	0.376	1.77E-11	B_cell
ISG15	1.24E-14	0.40405715	0.794	0.597	2.49E-11	B_cell
SPN	5.02E-14	0.21717752	0.576	0.419	1.00E-10	B_cell
NCKAP1L	1.47E-13	0.41130547	0.621	0.454	2.93E-10	B_cell
BCL3	1.62E-13	0.20904013	0.569	0.384	3.23E-10	B_cell
LYN	2.50E-13	0.70402334	0.619	0.479	4.99E-10	B_cell
WDFY4	7.96E-13	0.33598766	0.571	0.388	1.59E-09	B_cell
IGHG2	9.57E-13	4.1756286	0.356	0.306	1.91E-09	B_cell
CYBB	2.47E-12	0.35516126	0.61	0.475	4.94E-09	B_cell
IGHV3-43	3.08E-12	0.47827361	0.472	0.181	6.15E-09	B_cell
GLRX	3.26E-12	0.37724116	0.651	0.48	6.52E-09	B_cell
TTN	1.38E-11	0.26570311	0.424	0.565	2.76E-08	B_cell
SELL	2.81E-11	0.88929999	0.381	0.447	5.62E-08	B_cell
TOP2A	3.86E-11	0.2188588	0.674	0.436	7.71E-08	B_cell
PLAC8	8.43E-11	1.20002986	0.569	0.364	1.69E-07	B_cell
UCP2	1.03E-10	0.56526446	0.608	0.469	2.06E-07	B_cell
PRDM1	1.13E-10	1.03889066	0.58	0.54	2.27E-07	B_cell
IGLV3-19	1.76E-10	0.68499235	0.525	0.196	3.53E-07	B_cell
CKS2	2.65E-10	0.35000863	0.637	0.458	5.31E-07	B_cell
TRIB1	3.97E-10	0.66806621	0.64	0.541	7.94E-07	B_cell
IL2RG	7.56E-10	0.81746455	0.553	0.342	1.51E-06	B_cell
XBP1	1.37E-09	1.47760259	0.578	0.6	2.74E-06	B_cell
TRBC2	1.01E-08	0.43884655	0.402	0.388	2.02E-05	B_cell
HSP90B1	1.08E-08	0.67860953	0.878	0.86	2.16E-05	B_cell
CD27	1.22E-08	1.05238992	0.543	0.399	2.43E-05	B_cell
LILRB1	3.66E-08	0.25155764	0.39	0.339	7.32E-05	B_cell
SLAMF1	8.96E-08	0.2594479	0.491	0.234	0.00017912	B_cell
LCP1	9.93E-08	0.53077246	0.621	0.459	0.00019863	B_cell
IGKV1-16	1.00E-07	0.29868751	0.413	0.154	0.00020039	B_cell
RNU2-63P	1.11E-07	0.50597641	0.603	0.501	0.00022137	B_cell
SPINT2	1.55E-07	0.23722538	0.383	0.462	0.00031005	B_cell
AC104024.1	2.17E-07	0.48150067	0.411	0.344	0.00043314	B_cell
PTPN7	6.50E-07	0.46494316	0.394	0.36	0.00130035	B_cell

OCIAD2	7.70E-07	0.33469999	0.587	0.534	0.00154024	B_cell
CD48	8.18E-07	1.04581999	0.559	0.428	0.00163533	B_cell
NCF4	1.37E-06	0.26973833	0.431	0.437	0.0027361	B_cell
EZH2	3.59E-06	0.21562909	0.551	0.409	0.00718437	B_cell
RRM2	1.78E-05	0.60756431	0.502	0.431	0.0355373	B_cell
PTTG1	3.06E-05	0.2151122	0.434	0.488	0.06124415	B_cell
IL2RA	3.64E-05	0.2928429	0.523	0.373	0.07286498	B_cell
EIF4E	4.27E-05	0.23029655	0.644	0.508	0.08533638	B_cell
RNASE6	6.41E-05	0.32672172	0.574	0.553	0.12825085	B_cell
1-Mar	8.37E-05	0.59873647	0.589	0.519	0.16733861	B_cell
FCRL2	8.58E-05	0.75621585	0.516	0.306	0.17151789	B_cell
LY86	0.00023348	0.63526301	0.445	0.509	0.46695798	B_cell
SPAG4	0.00024311	0.27565494	0.427	0.471	0.48621863	B_cell
IGHV3-21	0.00050737	0.43678531	0.312	0.057	1	B_cell
IGHG4	0.0008086	4.55063956	0.468	0.216	1	B_cell
IFI30	0.00083467	0.21972695	0.578	0.548	1	B_cell
FAM49B	0.00165451	0.25931219	0.589	0.496	1	B_cell
AREG	0.00195644	0.5013626	0.426	0.382	1	B_cell
IKZF1	0.00287524	0.75042444	0.539	0.41	1	B_cell
KIF20B	0.00299899	0.27143591	0.452	0.473	1	B_cell
SAMSN1	0.00329389	0.24127996	0.431	0.371	1	B_cell
FAM30A	0.00470016	1.10536222	0.491	0.193	1	B_cell
IGHV3-15	0.00502692	0.66205604	0.379	0.221	1	B_cell
RGS2	0.00791985	0.3736421	0.589	0.541	1	B_cell
STMN1	2.13E-165	3.41575057	0.983	0.662	4.27E-162	NK/T
TOP2A	3.41E-156	2.7396278	0.93	0.436	6.82E-153	NK/T
HMGB2	1.53E-136	2.59169256	0.953	0.617	3.07E-133	NK/T
PCLAF	8.06E-132	2.35917396	0.9	0.485	1.61E-128	NK/T
TYMS	1.73E-128	2.59701665	0.89	0.491	3.46E-125	NK/T
MATK	6.67E-128	1.24624915	0.93	0.468	1.33E-124	NK/T
GZMA	4.21E-127	2.82021859	0.944	0.5	8.43E-124	NK/T
MKI67	3.56E-123	2.86975768	0.88	0.37	7.11E-120	NK/T
SMC4	3.21E-122	2.08122424	0.914	0.463	6.42E-119	NK/T
CTSW	1.10E-120	2.00618456	0.96	0.589	2.20E-117	NK/T
CD8B	4.43E-118	0.73240329	0.857	0.296	8.85E-115	NK/T
DHFR	4.57E-118	1.68420267	0.887	0.421	9.14E-115	NK/T
CENPK	4.06E-117	1.52756352	0.874	0.496	8.11E-114	NK/T
JPT1	2.92E-116	2.04547865	0.963	0.562	5.84E-113	NK/T
GZMM	8.77E-104	1.15290256	0.9	0.485	1.75E-100	NK/T
CENPM	2.14E-102	1.92251244	0.844	0.447	4.28E-99	NK/T
NKG7	2.58E-100	2.52784522	0.953	0.658	5.17E-97	NK/T
PBK	6.14E-99	1.19087552	0.764	0.194	1.23E-95	NK/T
TPX2	1.62E-98	1.94044325	0.824	0.406	3.25E-95	NK/T
GNLY	1.06E-97	3.13895166	0.887	0.682	2.13E-94	NK/T
CCL5	1.19E-97	2.12638221	0.917	0.501	2.39E-94	NK/T
CD8A	3.96E-97	1.02761765	0.837	0.354	7.93E-94	NK/T
PCNA	7.39E-97	2.06644524	0.884	0.515	1.48E-93	NK/T
IFNG	1.66E-96	1.55316834	0.884	0.519	3.31E-93	NK/T
EFHD2	1.66E-96	1.33905918	0.953	0.548	3.32E-93	NK/T
GZMH	3.99E-96	1.86027829	0.874	0.54	7.98E-93	NK/T
ESCO2	7.15E-94	1.24960761	0.814	0.41	1.43E-90	NK/T
CST7	2.14E-93	1.50015085	0.857	0.472	4.27E-90	NK/T
CLSPN	4.38E-93	1.98984077	0.834	0.524	8.76E-90	NK/T

ICOS	6.06E-92	0.39893284	0.748	0.186	1.21E-88	NK/T
NEK2	9.19E-89	0.70281253	0.801	0.467	1.84E-85	NK/T
UBE2C	1.06E-88	1.96915169	0.791	0.309	2.13E-85	NK/T
RRM2	1.65E-88	2.16579656	0.831	0.43	3.29E-85	NK/T
ARL4C	4.55E-88	1.4133549	0.924	0.507	9.10E-85	NK/T
UBE2T	4.42E-87	1.29758477	0.807	0.471	8.83E-84	NK/T
HPGD	1.90E-86	0.33540017	0.821	0.406	3.79E-83	NK/T
HOPX	7.48E-86	1.45536498	0.85	0.454	1.50E-82	NK/T
GTSE1	1.44E-85	1.47071617	0.787	0.384	2.87E-82	NK/T
AURKB	1.16E-84	1.37640529	0.807	0.499	2.32E-81	NK/T
KIF4A	1.62E-84	0.85060633	0.757	0.354	3.25E-81	NK/T
CKS2	5.68E-84	1.22331237	0.874	0.457	1.14E-80	NK/T
CD3E	6.33E-84	1.33983023	0.86	0.444	1.27E-80	NK/T
PRF1	3.72E-83	1.4839701	0.831	0.456	7.43E-80	NK/T
ISG20	1.81E-81	0.87431259	0.9	0.522	3.62E-78	NK/T
ATAD2	1.01E-80	1.84278117	0.814	0.447	2.01E-77	NK/T
CYTOR	1.23E-80	1.31699051	0.867	0.442	2.47E-77	NK/T
RAC2	2.96E-80	1.56615645	0.874	0.398	5.92E-77	NK/T
BIRC5	3.30E-79	1.81721815	0.797	0.442	6.61E-76	NK/T
EZR	6.54E-79	1.08291179	0.93	0.561	1.31E-75	NK/T
KIFC1	6.02E-77	1.28556396	0.774	0.408	1.20E-73	NK/T
CKAP2	1.31E-76	1.37307773	0.807	0.49	2.62E-73	NK/T
RNF157	1.38E-75	0.60050385	0.787	0.435	2.76E-72	NK/T
MAD2L1	4.62E-75	1.19827835	0.784	0.489	9.25E-72	NK/T
DUSP4	9.73E-75	0.32538008	0.767	0.382	1.95E-71	NK/T
KNL1	3.45E-72	1.66576641	0.754	0.336	6.89E-69	NK/T
HELLS	6.25E-72	1.61025128	0.797	0.505	1.25E-68	NK/T
PIMREG	1.28E-71	0.7571857	0.741	0.384	2.55E-68	NK/T
NCR3	4.69E-71	0.75226576	0.801	0.456	9.37E-68	NK/T
HIST1H4C	9.20E-71	2.72457688	0.95	0.763	1.84E-67	NK/T
RAD51AP1	1.73E-70	1.13171747	0.754	0.338	3.45E-67	NK/T
ASPM	1.73E-70	2.36686958	0.774	0.445	3.47E-67	NK/T
NUSAP1	2.04E-70	1.68713808	0.761	0.375	4.08E-67	NK/T
PKMYT1	3.51E-70	0.907296	0.738	0.358	7.02E-67	NK/T
CCL4	1.11E-69	1.06356201	0.864	0.448	2.22E-66	NK/T
CORO1A	1.89E-69	2.09456965	0.831	0.455	3.78E-66	NK/T
KLRC2	2.66E-69	0.38698372	0.804	0.502	5.32E-66	NK/T
XCL2	1.04E-68	0.85256295	0.784	0.422	2.09E-65	NK/T
ZWINT	6.55E-67	1.17127877	0.757	0.46	1.31E-63	NK/T
HMGA1	3.06E-66	0.92779088	0.85	0.479	6.11E-63	NK/T
HMMR	5.56E-66	1.31448522	0.754	0.468	1.11E-62	NK/T
PTPN7	2.12E-65	1.22347303	0.777	0.359	4.25E-62	NK/T
CENPF	2.49E-65	2.56663592	0.764	0.484	4.98E-62	NK/T
TK1	3.15E-65	1.81136692	0.754	0.386	6.29E-62	NK/T
EMB	4.36E-64	1.0245744	0.827	0.475	8.72E-61	NK/T
CD160	7.94E-64	0.49544839	0.794	0.54	1.59E-60	NK/T
LMNB1	3.84E-63	1.24401125	0.751	0.367	7.69E-60	NK/T
PCED1B-AS1	8.69E-63	0.78555463	0.841	0.513	1.74E-59	NK/T
IQGAP2	1.07E-62	0.72560711	0.907	0.574	2.14E-59	NK/T
CD247	1.79E-62	1.17330324	0.777	0.388	3.57E-59	NK/T
CENPU	3.40E-62	1.01859916	0.748	0.434	6.79E-59	NK/T
CXCR4	4.77E-62	0.67091096	0.91	0.567	9.54E-59	NK/T
PTTG1	2.08E-61	1.85065912	0.771	0.487	4.16E-58	NK/T

BRCA2	2.59E-61	1.20386988	0.787	0.542	5.18E-58	NK/T
TUBA1C	3.35E-61	1.2936742	0.92	0.584	6.69E-58	NK/T
KIF20B	1.12E-60	1.42526431	0.777	0.471	2.24E-57	NK/T
EZH2	5.35E-59	1.5345139	0.754	0.408	1.07E-55	NK/T
GMFG	5.71E-59	0.86008848	0.95	0.711	1.14E-55	NK/T
C12orf75	6.71E-58	0.82875107	0.887	0.562	1.34E-54	NK/T
CRIP1	2.20E-57	0.89837141	0.97	0.795	4.39E-54	NK/T
KIF15	3.94E-57	0.89541072	0.708	0.407	7.89E-54	NK/T
CRTAM	8.36E-57	0.313189	0.771	0.483	1.67E-53	NK/T
IRF4	8.60E-56	0.38536137	0.711	0.295	1.72E-52	NK/T
BUB1	2.81E-55	0.8667101	0.708	0.367	5.62E-52	NK/T
HCST	4.68E-55	1.18459076	0.864	0.53	9.36E-52	NK/T
LCP1	6.45E-55	1.71124086	0.811	0.459	1.29E-51	NK/T
PTPRC	8.23E-55	1.32608334	0.864	0.486	1.65E-51	NK/T
SDF2L1	1.40E-54	0.99027345	0.877	0.611	2.79E-51	NK/T
KIF11	4.36E-53	1.38100856	0.714	0.362	8.73E-50	NK/T
LAIR2	5.92E-53	0.79812252	0.764	0.451	1.18E-49	NK/T
AHSP	1.48E-52	0.69131282	0.744	0.329	2.96E-49	NK/T
SNX10	1.62E-52	0.3815766	0.751	0.352	3.24E-49	NK/T
DUSP2	3.11E-52	0.80462807	0.811	0.44	6.23E-49	NK/T
SAMD3	4.77E-52	0.78633152	0.734	0.389	9.55E-49	NK/T
IL2RB	4.92E-52	0.62293096	0.704	0.302	9.84E-49	NK/T
CDCA3	1.87E-51	1.03846264	0.728	0.494	3.74E-48	NK/T
ADGRG1	2.71E-51	0.26841992	0.857	0.717	5.42E-48	NK/T
SKAP1	3.89E-51	0.82589843	0.774	0.485	7.78E-48	NK/T
PRDM1	6.75E-51	0.5652173	0.814	0.539	1.35E-47	NK/T
CD53	2.51E-50	0.77286386	0.844	0.542	5.02E-47	NK/T
SRGN	1.49E-49	1.09149275	0.953	0.643	2.98E-46	NK/T
SH2D1A	1.57E-49	0.80578474	0.708	0.276	3.15E-46	NK/T
IL2RG	7.54E-49	1.09125721	0.764	0.342	1.51E-45	NK/T
FAM111B	2.65E-48	1.49870564	0.708	0.421	5.30E-45	NK/T
PTPN6	2.85E-48	0.74142662	0.784	0.466	5.70E-45	NK/T
IL10RA	2.93E-48	0.26784321	0.771	0.384	5.87E-45	NK/T
RUNX3	4.84E-48	1.19345143	0.761	0.431	9.69E-45	NK/T
PLEK	2.33E-46	0.32843112	0.824	0.464	4.67E-43	NK/T
MYO1F	2.41E-46	0.55307645	0.784	0.498	4.81E-43	NK/T
CDC45	4.70E-46	0.64578592	0.674	0.348	9.40E-43	NK/T
NCAPG	1.10E-45	1.49824707	0.701	0.406	2.20E-42	NK/T
HIST1H1B	1.50E-45	1.55926962	0.658	0.281	3.00E-42	NK/T
MXD3	1.90E-45	0.92972904	0.691	0.403	3.80E-42	NK/T
NDC80	4.72E-45	1.17438554	0.704	0.403	9.44E-42	NK/T
IKZF3	2.61E-44	1.00099927	0.728	0.351	5.22E-41	NK/T
NUF2	2.55E-43	1.16058861	0.691	0.406	5.09E-40	NK/T
SYTL3	3.19E-43	0.32059944	0.757	0.476	6.39E-40	NK/T
LCK	4.43E-43	0.84199808	0.748	0.486	8.87E-40	NK/T
GINS2	4.85E-43	0.9918951	0.711	0.535	9.70E-40	NK/T
KIF2C	1.06E-42	0.70227875	0.684	0.417	2.13E-39	NK/T
LAX1	1.40E-42	0.45225806	0.751	0.489	2.79E-39	NK/T
ITGA4	1.41E-42	1.06370556	0.754	0.426	2.81E-39	NK/T
CDT1	2.99E-42	1.17986072	0.704	0.566	5.99E-39	NK/T
ALOX5AP	5.39E-42	0.31976145	0.827	0.517	1.08E-38	NK/T
KPNA2	1.63E-41	0.96561663	0.781	0.506	3.25E-38	NK/T
ABRACL	1.74E-41	0.93863535	0.754	0.412	3.47E-38	NK/T

KLRB1	2.47E-41	0.81344473	0.694	0.442	4.94E-38	NK/T
GZMB	2.75E-41	1.96581826	0.741	0.533	5.50E-38	NK/T
TACC3	2.80E-41	1.43395142	0.711	0.499	5.60E-38	NK/T
NCAPH	6.35E-41	0.75157585	0.691	0.458	1.27E-37	NK/T
CKAP2L	1.30E-40	1.1354695	0.664	0.301	2.60E-37	NK/T
TSPAN5	1.99E-40	0.35190324	0.791	0.617	3.99E-37	NK/T
PYHIN1	2.77E-40	0.56205151	0.721	0.391	5.53E-37	NK/T
PRC1	1.07E-39	0.91026925	0.674	0.462	2.14E-36	NK/T
CYBA	4.02E-39	0.88893254	0.98	0.84	8.03E-36	NK/T
TTK	1.09E-38	0.93444028	0.681	0.423	2.19E-35	NK/T
APOLD1	1.35E-38	0.7192417	0.834	0.657	2.71E-35	NK/T
HIST1H3G	2.12E-38	0.6798342	0.704	0.527	4.25E-35	NK/T
VAMP8	5.22E-38	0.41701265	0.837	0.52	1.04E-34	NK/T
CD3D	2.36E-37	1.33613322	0.704	0.382	4.71E-34	NK/T
IL32	7.70E-37	1.31090614	0.824	0.701	1.54E-33	NK/T
AC243960.1	2.34E-36	0.4650153	0.721	0.457	4.68E-33	NK/T
KLRD1	2.52E-36	1.2812529	0.724	0.571	5.04E-33	NK/T
CYTIP	6.26E-36	0.76125438	0.728	0.527	1.25E-32	NK/T
CCNB2	7.56E-36	1.04361208	0.661	0.417	1.51E-32	NK/T
TRBC1	1.21E-35	1.31182637	0.691	0.402	2.41E-32	NK/T
TRAC	5.29E-35	1.19427906	0.718	0.411	1.06E-31	NK/T
CD37	6.71E-35	0.43342264	0.794	0.559	1.34E-31	NK/T
CENPW	1.40E-34	0.99273995	0.704	0.516	2.81E-31	NK/T
ASF1B	2.42E-34	1.07886125	0.691	0.519	4.84E-31	NK/T
SGO1	3.39E-34	0.85363462	0.648	0.353	6.78E-31	NK/T
E2F1	5.57E-34	1.00810795	0.658	0.443	1.11E-30	NK/T
LBH	8.77E-34	0.3648542	0.874	0.611	1.75E-30	NK/T
SGO2	9.32E-34	0.97653898	0.701	0.495	1.86E-30	NK/T
FABP5	1.10E-33	0.31602195	0.937	0.765	2.20E-30	NK/T
CA1	1.87E-33	0.79028413	0.538	0.176	3.75E-30	NK/T
MYBL2	2.06E-33	0.94311215	0.661	0.329	4.12E-30	NK/T
CTSC	5.04E-33	0.42142221	0.93	0.695	1.01E-29	NK/T
SPON2	7.58E-33	0.60792808	0.781	0.533	1.52E-29	NK/T
GZMK	8.22E-33	0.88967716	0.678	0.361	1.64E-29	NK/T
UCP2	1.46E-32	0.83284377	0.744	0.469	2.91E-29	NK/T
ITGB2	1.51E-32	0.50787434	0.827	0.556	3.02E-29	NK/T
CDK1	2.90E-32	1.36575177	0.664	0.481	5.81E-29	NK/T
TIGIT	5.35E-32	0.39580701	0.701	0.508	1.07E-28	NK/T
CDKN3	1.39E-31	1.42772236	0.671	0.528	2.78E-28	NK/T
HIST1H3B	4.90E-31	0.97112935	0.674	0.502	9.79E-28	NK/T
EVI2B	5.31E-31	0.21494021	0.751	0.457	1.06E-27	NK/T
TNF	1.46E-30	0.63575761	0.668	0.435	2.91E-27	NK/T
AD000864.1	1.57E-30	0.36396529	0.714	0.486	3.13E-27	NK/T
HJURP	5.12E-30	0.98880326	0.648	0.399	1.02E-26	NK/T
EIF4E	1.12E-29	0.42191041	0.767	0.508	2.24E-26	NK/T
HIST1H2AL	1.40E-29	0.67077907	0.704	0.549	2.81E-26	NK/T
SKA3	1.99E-29	0.55231176	0.674	0.457	3.98E-26	NK/T
SLC39A8	3.40E-29	0.21181603	0.674	0.438	6.81E-26	NK/T
PHLDA1	3.49E-29	0.38453477	0.721	0.435	6.99E-26	NK/T
SIT1	4.64E-29	0.44335831	0.688	0.462	9.29E-26	NK/T
COTL1	4.88E-29	0.8407069	0.791	0.516	9.75E-26	NK/T
AURKA	7.44E-29	0.44894334	0.671	0.484	1.49E-25	NK/T
DEPDC1	8.08E-29	0.62261815	0.581	0.153	1.62E-25	NK/T

CDCA8	1.29E-28	0.88678511	0.621	0.355	2.59E-25	NK/T
LDLR	1.89E-28	0.59548005	0.714	0.506	3.78E-25	NK/T
GNG2	1.89E-28	0.71548588	0.774	0.579	3.78E-25	NK/T
ARHGAP30	2.58E-28	0.94936037	0.704	0.438	5.16E-25	NK/T
FANCI	4.39E-28	0.95897445	0.661	0.485	8.79E-25	NK/T
GPSM3	7.22E-28	0.59684925	0.764	0.535	1.44E-24	NK/T
HIST1H1C	1.83E-27	1.26046188	0.708	0.463	3.66E-24	NK/T
DHCR24	3.72E-27	0.22549941	0.701	0.524	7.44E-24	NK/T
FCGR3A	6.81E-27	0.29780593	0.741	0.485	1.36E-23	NK/T
FGFBP2	6.94E-27	0.50507474	0.728	0.612	1.39E-23	NK/T
PRKCB	9.11E-27	0.57250002	0.648	0.363	1.82E-23	NK/T
CD52	1.71E-26	1.63413592	0.688	0.443	3.42E-23	NK/T
ETS1	1.83E-25	0.87673651	0.764	0.625	3.66E-22	NK/T
OCIAD2	2.00E-25	0.31782339	0.711	0.534	4.00E-22	NK/T
AREG	2.89E-25	0.28771428	0.668	0.382	5.78E-22	NK/T
CD3G	4.60E-25	1.03822518	0.678	0.414	9.20E-22	NK/T
HIST1H1D	9.88E-25	1.28856231	0.674	0.487	1.98E-21	NK/T
SHCBP1	5.71E-24	0.86462474	0.628	0.38	1.14E-20	NK/T
SPC25	7.49E-24	1.03330076	0.618	0.36	1.50E-20	NK/T
SYNE2	1.05E-23	0.39992189	0.914	0.717	2.10E-20	NK/T
KIF14	1.44E-23	0.69454034	0.631	0.513	2.87E-20	NK/T
CCNA2	4.07E-23	1.09380805	0.621	0.434	8.15E-20	NK/T
GUCY1A2	5.90E-23	0.26055053	0.754	0.54	1.18E-19	NK/T
CX3CR1	8.39E-23	0.34656187	0.618	0.389	1.68E-19	NK/T
TRGC2	9.07E-23	0.57949906	0.621	0.336	1.81E-19	NK/T
PLK1	1.51E-22	0.68900039	0.615	0.365	3.02E-19	NK/T
SMAP2	3.22E-22	0.29539897	0.804	0.603	6.44E-19	NK/T
DOK2	3.29E-22	0.40970544	0.684	0.42	6.57E-19	NK/T
WAS	4.94E-22	0.58678727	0.688	0.452	9.88E-19	NK/T
SAMSN1	1.60E-21	0.29632913	0.648	0.37	3.20E-18	NK/T
SEC11C	1.86E-21	0.42682616	0.767	0.614	3.71E-18	NK/T
HLA-B	1.89E-21	0.4819853	0.997	0.948	3.77E-18	NK/T
DIAPH3	3.02E-21	0.83887876	0.598	0.378	6.04E-18	NK/T
NT5DC2	4.81E-21	0.31819442	0.701	0.513	9.61E-18	NK/T
DLGAP5	7.10E-21	1.35255454	0.605	0.273	1.42E-17	NK/T
KCNQ1OT1	9.35E-20	0.31540785	0.85	0.572	1.87E-16	NK/T
CDCA5	3.28E-19	0.87336898	0.621	0.494	6.57E-16	NK/T
KLRF1	1.14E-18	0.56599576	0.631	0.435	2.27E-15	NK/T
CENPA	2.36E-18	0.47086157	0.591	0.367	4.72E-15	NK/T
FAM49B	3.29E-18	0.4997218	0.698	0.496	6.58E-15	NK/T
KIF23	6.10E-18	1.00709408	0.571	0.26	1.22E-14	NK/T
TFRC	6.41E-18	0.27041614	0.668	0.442	1.28E-14	NK/T
GPR65	6.64E-18	0.46397403	0.645	0.397	1.33E-14	NK/T
CENPE	7.16E-18	1.23477753	0.608	0.443	1.43E-14	NK/T
LINC01871	1.00E-17	0.63027128	0.558	0.311	2.01E-14	NK/T
IQGAP3	1.14E-17	0.30618672	0.568	0.376	2.29E-14	NK/T
CD320	1.20E-17	0.26330291	0.85	0.702	2.39E-14	NK/T
TNFAIP3	2.02E-17	0.57025767	0.721	0.483	4.05E-14	NK/T
TROAP	3.49E-17	0.6729679	0.591	0.329	6.97E-14	NK/T
DUSP5	4.28E-17	0.2293781	0.694	0.479	8.55E-14	NK/T
ARHGAP11A	7.26E-17	0.80740595	0.578	0.39	1.45E-13	NK/T
MBP	9.15E-17	0.35489259	0.831	0.664	1.83E-13	NK/T
ANLN	1.73E-16	0.7638317	0.588	0.411	3.47E-13	NK/T

LSP1	3.06E-16	0.70573688	0.654	0.435	6.12E-13	NK/T
LCP2	5.14E-16	0.3614374	0.674	0.475	1.03E-12	NK/T
RHOH	6.82E-16	0.55464203	0.615	0.44	1.36E-12	NK/T
ISG15	2.00E-15	0.43542314	0.771	0.598	4.00E-12	NK/T
CYTH4	2.67E-15	0.26977101	0.641	0.492	5.33E-12	NK/T
RGS1	2.86E-15	0.24898343	0.688	0.488	5.72E-12	NK/T
CD69	7.05E-15	1.25111037	0.664	0.46	1.41E-11	NK/T
CEP55	7.83E-15	1.00393179	0.585	0.402	1.57E-11	NK/T
KIF20A	2.08E-14	0.64987716	0.548	0.345	4.16E-11	NK/T
SEL1L3	3.11E-14	0.29736238	0.611	0.449	6.21E-11	NK/T
ARRB2	3.97E-14	0.25777898	0.678	0.53	7.94E-11	NK/T
CLIC3	4.29E-14	0.47872625	0.708	0.577	8.59E-11	NK/T
LTB	5.40E-14	0.8587451	0.342	0.451	1.08E-10	NK/T
TRBC2	5.44E-14	0.92949881	0.635	0.388	1.09E-10	NK/T
POLQ	6.49E-14	0.40230407	0.326	0.472	1.30E-10	NK/T
IL7R	1.98E-13	0.9275321	0.276	0.382	3.96E-10	NK/T
GPR171	3.21E-13	0.25400576	0.575	0.286	6.42E-10	NK/T
SPN	4.80E-13	0.8230053	0.598	0.42	9.61E-10	NK/T
MELK	1.14E-12	0.75594883	0.575	0.443	2.28E-09	NK/T
CCND1	1.38E-12	0.48828653	0.741	0.516	2.76E-09	NK/T
E2F8	5.10E-12	0.61537476	0.598	0.473	1.02E-08	NK/T
PIM1	8.03E-12	0.40977678	0.588	0.437	1.61E-08	NK/T
RFLNB	1.31E-11	0.46278326	0.641	0.496	2.62E-08	NK/T
RGS5	1.76E-11	0.66458887	0.777	0.557	3.53E-08	NK/T
KLRC1	4.50E-11	0.58613179	0.551	0.342	9.00E-08	NK/T
IFI6	4.91E-11	0.21399186	0.731	0.534	9.82E-08	NK/T
RNU2-63P	5.11E-11	0.36688341	0.615	0.501	1.02E-07	NK/T
MCM10	5.17E-11	0.56798442	0.588	0.537	1.03E-07	NK/T
SAPCD2	6.23E-11	0.6532754	0.538	0.305	1.25E-07	NK/T
CD7	2.69E-10	1.16660601	0.631	0.533	5.39E-07	NK/T
HIST1H3F	2.92E-10	0.58810763	0.571	0.425	5.85E-07	NK/T
CD38	4.90E-10	0.55761126	0.558	0.386	9.81E-07	NK/T
XCL1	1.06E-09	0.34484082	0.532	0.262	2.12E-06	NK/T
FERMT3	3.63E-09	0.2233628	0.598	0.413	7.26E-06	NK/T
TAGAP	5.32E-09	0.31158352	0.598	0.423	1.06E-05	NK/T
PTGER4	1.44E-08	0.22055493	0.615	0.452	2.89E-05	NK/T
NRGN	2.63E-08	0.41799853	0.585	0.469	5.26E-05	NK/T
CCNB1	2.99E-08	1.11967627	0.578	0.532	5.97E-05	NK/T
FOXM1	4.13E-08	0.87487113	0.561	0.504	8.26E-05	NK/T
CDC20	5.42E-08	0.92111501	0.548	0.437	0.00010845	NK/T
RGS16	8.07E-08	0.22145434	0.701	0.577	0.00016136	NK/T
TNFRSF1B	1.02E-07	0.21127012	0.694	0.594	0.00020471	NK/T
EPSTI1	1.53E-07	0.27734086	0.558	0.431	0.00030683	NK/T
MDK	6.09E-07	0.46579114	0.751	0.671	0.00121848	NK/T
HMGB3	6.24E-07	0.57307645	0.535	0.387	0.00124822	NK/T
NCKAP1L	8.70E-07	0.22714643	0.601	0.455	0.00173965	NK/T
SPOCK2	1.15E-06	0.38502098	0.385	0.483	0.00230857	NK/T
FCMR	2.11E-06	0.35720344	0.568	0.422	0.0042296	NK/T
PNP	2.43E-06	0.36743933	0.551	0.432	0.0048608	NK/T
IKZF1	4.32E-06	0.93626117	0.548	0.411	0.00864857	NK/T
ANKRD1	1.15E-05	0.29619939	0.292	0.342	0.02294884	NK/T
SLA	1.52E-05	0.21854355	0.611	0.511	0.03035854	NK/T
CAPG	2.52E-05	0.22558707	0.608	0.503	0.05048958	NK/T

HEMGN	4.55E-05	0.23310612	0.359	0.315	0.09104524	NK/T
PRR11	6.18E-05	0.59484869	0.548	0.514	0.12366024	NK/T
ANGPT2	0.00010125	0.20777229	0.694	0.663	0.20249304	NK/T
SLAMF1	0.00013424	0.31368608	0.329	0.236	0.26848149	NK/T
TRDC	0.00039114	1.09228911	0.555	0.495	0.78228486	NK/T
STAT1	0.00069881	0.29913923	0.558	0.472	1	NK/T
GBP5	0.00076422	0.25711215	0.349	0.334	1	NK/T
CD48	0.00117953	0.53647413	0.535	0.428	1	NK/T
CDCA2	0.00172162	0.53547147	0.522	0.426	1	NK/T
INPP4B	0.00214334	0.46797499	0.551	0.523	1	NK/T
COL4A1	0.00440327	0.20910063	0.784	0.673	1	NK/T
C1QL1	0.00484204	0.30563611	0.512	0.497	1	NK/T
PTN	0.00747933	0.8666297	0.525	0.519	1	NK/T
LINC00892	0.00874685	0.27079898	0.432	0.484	1	NK/T
FBLN1	1.13E-38	1.36349051	1	0.9	2.26E-35	NK
DCN	8.91E-37	1.34510154	1	0.915	1.78E-33	NK
ABCA8	3.58E-35	1.03710576	0.992	0.77	7.17E-32	NK
C3	5.10E-35	1.4214942	0.984	0.819	1.02E-31	NK
CCDC80	5.89E-35	1.33903437	0.984	0.765	1.18E-31	NK
CCL5	8.69E-35	1.57419922	0.859	0.502	1.74E-31	NK
IGFBP6	1.49E-34	1.25973201	1	0.916	2.98E-31	NK
C1S	3.74E-34	1.14170511	1	0.819	7.49E-31	NK
MMP2	4.43E-34	1.10214918	0.984	0.744	8.87E-31	NK
SCARA5	1.18E-33	1.09801818	0.953	0.711	2.36E-30	NK
ABI3BP	3.09E-33	1.03954298	0.969	0.731	6.18E-30	NK
CD69	7.46E-33	1.46691338	0.812	0.461	1.49E-29	NK
MFAP5	2.83E-31	1.15649557	0.922	0.621	5.65E-28	NK
HCST	5.76E-31	1.03606836	0.891	0.531	1.15E-27	NK
SERPINF1	1.44E-30	1.0454695	1	0.859	2.89E-27	NK
PODN	1.47E-30	0.92723104	0.977	0.733	2.95E-27	NK
C1R	4.79E-30	1.07075403	0.992	0.896	9.58E-27	NK
FBN1	1.18E-29	1.12498168	0.961	0.725	2.36E-26	NK
CFD	7.12E-29	1.32714281	0.984	0.93	1.42E-25	NK
SFRP2	7.42E-29	1.29928162	0.883	0.585	1.48E-25	NK
COL14A1	1.56E-28	1.02664499	0.992	0.807	3.12E-25	NK
NKG7	4.14E-28	1.86897167	0.812	0.658	8.29E-25	NK
OGN	6.13E-28	0.86086611	0.977	0.727	1.23E-24	NK
ADH1B	6.38E-28	0.91089916	0.977	0.845	1.28E-24	NK
RAC2	9.47E-28	0.99683381	0.766	0.399	1.89E-24	NK
GZMA	1.47E-27	1.36280889	0.789	0.501	2.93E-24	NK
COL1A2	3.45E-27	0.9925148	0.984	0.774	6.90E-24	NK
GAS1	3.78E-27	0.90802411	0.891	0.678	7.55E-24	NK
PLA2G2A	3.87E-27	1.77964278	0.969	0.903	7.73E-24	NK
IGSF10	4.16E-27	0.84706602	0.875	0.685	8.33E-24	NK
PTPRC	5.17E-27	0.55061325	0.914	0.487	1.03E-23	NK
MGP	6.82E-27	0.82158292	1	0.971	1.36E-23	NK
IL32	2.39E-26	1.07071746	0.891	0.701	4.78E-23	NK
EFEMP1	4.17E-26	0.94593756	0.945	0.707	8.33E-23	NK
ITGBL1	1.16E-25	0.89977902	0.891	0.723	2.32E-22	NK
COL1A1	1.43E-25	1.05296721	0.969	0.697	2.86E-22	NK
LOX	4.97E-25	0.7871208	0.828	0.555	9.95E-22	NK
LUM	1.11E-24	0.8663758	0.93	0.722	2.21E-21	NK
PRELP	1.27E-24	0.73594811	0.961	0.734	2.53E-21	NK

MBP	2.53E-24	0.63085712	0.898	0.664	5.05E-21	NK
VCAN	1.38E-23	0.76627296	0.969	0.703	2.77E-20	NK
HTRA3	1.59E-23	1.05909426	0.852	0.687	3.18E-20	NK
UAP1	1.81E-23	0.7081375	0.828	0.517	3.61E-20	NK
IGFBP5	2.19E-23	1.14845717	1	0.891	4.38E-20	NK
ABCA10	3.45E-23	0.67216482	0.852	0.643	6.90E-20	NK
IGF1	4.48E-23	0.99454671	0.906	0.666	8.95E-20	NK
TSHZ2	6.87E-23	0.69942437	0.977	0.805	1.37E-19	NK
CLIC3	1.57E-22	1.14082694	0.781	0.578	3.13E-19	NK
HSP90B1	4.18E-22	0.92415485	0.984	0.86	8.36E-19	NK
CTSW	4.43E-22	1.35354489	0.766	0.591	8.85E-19	NK
LINC01697	4.55E-22	0.56580567	0.805	0.671	9.10E-19	NK
RSPO3	4.58E-22	0.53872558	0.852	0.709	9.17E-19	NK
SLIT2	7.58E-22	0.71915506	0.82	0.634	1.52E-18	NK
SEMA3C	9.15E-22	0.7933486	0.781	0.532	1.83E-18	NK
TIMP1	2.13E-21	0.7147257	0.984	0.905	4.27E-18	NK
CTSK	2.55E-21	0.76201681	0.852	0.7	5.11E-18	NK
GZMB	3.97E-21	1.63782996	0.727	0.533	7.94E-18	NK
C16orf89	4.74E-21	0.52948199	0.812	0.696	9.48E-18	NK
FSTL1	5.13E-21	0.71320674	0.977	0.673	1.03E-17	NK
OLFML3	1.12E-20	0.69751532	0.836	0.578	2.24E-17	NK
GPNMB	1.26E-20	0.74865856	0.93	0.712	2.52E-17	NK
GZMM	1.83E-20	1.04946953	0.734	0.486	3.66E-17	NK
GPX3	2.11E-20	0.65935683	0.992	0.867	4.23E-17	NK
PI16	2.32E-20	0.87867167	0.828	0.735	4.65E-17	NK
CRISPLD2	2.86E-20	0.74355151	0.898	0.7	5.72E-17	NK
BICC1	3.24E-20	0.71883996	0.844	0.674	6.49E-17	NK
SPON2	4.12E-20	1.02634276	0.781	0.533	8.23E-17	NK
FBLN2	1.51E-19	0.7191674	0.883	0.676	3.01E-16	NK
CORO1A	1.97E-19	0.97149398	0.75	0.456	3.95E-16	NK
CST7	2.88E-19	1.43027253	0.711	0.473	5.76E-16	NK
PDGFRA	3.07E-19	0.6326851	0.859	0.629	6.14E-16	NK
ABCA6	3.91E-19	0.6111936	0.859	0.705	7.82E-16	NK
SFRP1	8.04E-19	0.87550258	0.844	0.698	1.61E-15	NK
CPB1	1.20E-18	0.50624549	0.781	0.648	2.41E-15	NK
COL6A3	2.29E-18	0.747334	0.875	0.706	4.58E-15	NK
CFH	2.31E-18	0.51721637	0.945	0.774	4.62E-15	NK
MT1X	3.80E-18	0.9130995	0.961	0.82	7.61E-15	NK
PCOLCE	4.39E-18	0.57475693	0.93	0.761	8.78E-15	NK
GPC3	1.00E-17	0.74076208	0.797	0.591	2.01E-14	NK
IFNG	1.22E-17	1.43693754	0.711	0.52	2.45E-14	NK
LCP1	1.25E-17	0.482891	0.805	0.46	2.50E-14	NK
BOC	1.47E-17	0.55675142	0.797	0.584	2.93E-14	NK
LAMA2	2.12E-17	0.5384782	0.797	0.551	4.24E-14	NK
ARL4C	3.02E-17	0.82021211	0.742	0.508	6.04E-14	NK
HHIP	3.06E-17	0.52905298	0.742	0.564	6.13E-14	NK
RARRES1	3.86E-17	1.15639867	0.852	0.719	7.71E-14	NK
CD52	4.48E-17	0.79865387	0.742	0.443	8.97E-14	NK
SRGN	7.33E-17	0.47593162	0.914	0.644	1.47E-13	NK
CADM3	1.79E-16	0.50335578	0.844	0.759	3.58E-13	NK
COL3A1	3.53E-16	0.89102203	0.922	0.748	7.05E-13	NK
BHLHE41	4.03E-16	0.26438913	0.797	0.613	8.06E-13	NK
CENPW	4.46E-16	0.33550575	0.742	0.517	8.92E-13	NK

MEG3	4.55E-16	0.99790977	0.812	0.598	9.09E-13	NK
NTRK2	6.45E-16	0.59054984	0.914	0.766	1.29E-12	NK
FLRT2	9.71E-16	0.39985224	0.82	0.649	1.94E-12	NK
KLRD1	1.82E-15	1.38503097	0.703	0.572	3.63E-12	NK
PDGFRL	2.29E-15	0.61019624	0.797	0.686	4.58E-12	NK
PLPP3	3.03E-15	0.69971265	0.875	0.732	6.05E-12	NK
ABCA9	4.06E-15	0.47863384	0.812	0.687	8.12E-12	NK
NNMT	5.93E-15	0.66171022	0.93	0.844	1.19E-11	NK
FMOD	7.53E-15	0.46462965	0.758	0.528	1.51E-11	NK
DPT	9.63E-15	0.59936772	0.727	0.541	1.93E-11	NK
CXCL14	1.01E-14	0.9056213	0.805	0.634	2.02E-11	NK
CYBA	2.03E-14	0.48034792	0.969	0.84	4.05E-11	NK
ABCC9	2.17E-14	0.58447676	0.836	0.722	4.34E-11	NK
OMD	2.25E-14	0.4404095	0.773	0.618	4.51E-11	NK
DUSP2	2.63E-14	0.94518495	0.672	0.441	5.27E-11	NK
CXCR4	3.35E-14	0.57293306	0.766	0.568	6.70E-11	NK
LTBP1	6.78E-14	0.42759432	0.883	0.694	1.36E-10	NK
AEBP1	1.10E-13	0.43668609	0.945	0.759	2.20E-10	NK
CCL4	1.17E-13	1.76959739	0.695	0.45	2.34E-10	NK
CD248	2.23E-13	0.55890058	0.688	0.438	4.46E-10	NK
ETS1	2.28E-13	0.42133794	0.805	0.625	4.55E-10	NK
FKBP11	2.63E-13	0.36813396	0.789	0.614	5.26E-10	NK
SRPX	4.55E-13	0.60593575	0.812	0.686	9.09E-10	NK
C7	6.43E-13	0.67234404	0.891	0.789	1.29E-09	NK
AKAP12	6.90E-13	0.41809292	0.938	0.789	1.38E-09	NK
CD34	8.66E-13	0.41222128	0.859	0.764	1.73E-09	NK
FOXD1	2.48E-12	0.3007944	0.812	0.698	4.95E-09	NK
PTGIS	2.76E-12	0.60919309	0.734	0.557	5.52E-09	NK
CD48	2.78E-12	0.66064274	0.656	0.428	5.55E-09	NK
GLUL	2.94E-12	0.37911849	0.984	0.874	5.89E-09	NK
PMP22	4.66E-12	0.3393424	0.953	0.852	9.32E-09	NK
MT1E	4.71E-12	0.5448072	0.977	0.848	9.42E-09	NK
CD53	5.12E-12	0.53450293	0.727	0.543	1.02E-08	NK
CLU	5.16E-12	0.44598595	0.992	0.919	1.03E-08	NK
PYHIN1	5.20E-12	0.75673599	0.609	0.392	1.04E-08	NK
DCLK1	1.83E-11	0.44848671	0.789	0.637	3.67E-08	NK
ISG20	2.18E-11	0.63682225	0.695	0.524	4.37E-08	NK
ANK2	2.50E-11	0.222108	0.703	0.512	4.99E-08	NK
TGFBR3	2.76E-11	0.41173394	0.891	0.797	5.52E-08	NK
ADGRG2	6.52E-11	0.29803248	0.648	0.518	1.30E-07	NK
PROCR	8.22E-11	0.56934267	0.781	0.701	1.64E-07	NK
GFRA1	8.37E-11	0.48984821	0.781	0.648	1.67E-07	NK
PCED1B-AS1	1.30E-10	0.62847706	0.656	0.514	2.60E-07	NK
MT1M	1.70E-10	0.74251769	0.969	0.885	3.40E-07	NK
USP53	1.70E-10	0.46243341	0.852	0.762	3.41E-07	NK
MYOC	2.38E-10	0.56718554	0.703	0.614	4.76E-07	NK
FBLN5	2.73E-10	0.40028141	0.734	0.558	5.46E-07	NK
MT2A	3.07E-10	0.56285443	0.992	0.966	6.13E-07	NK
TNXB	3.14E-10	0.53975465	0.773	0.693	6.29E-07	NK
CD3E	3.48E-10	0.71064985	0.633	0.445	6.95E-07	NK
ACKR4	4.48E-10	0.43948979	0.695	0.607	8.95E-07	NK
HELLPAR	4.52E-10	0.49178022	0.688	0.558	9.05E-07	NK
LSP1	4.93E-10	0.54292453	0.695	0.435	9.86E-07	NK

SVEP1	5.71E-10	0.73190353	0.703	0.611	1.14E-06	NK
GZMH	6.71E-10	1.36254544	0.656	0.541	1.34E-06	NK
FGL2	6.79E-10	0.37964188	0.812	0.626	1.36E-06	NK
UCHL1	1.01E-09	0.30364127	0.711	0.605	2.01E-06	NK
FAM49B	1.02E-09	0.27098154	0.695	0.496	2.04E-06	NK
CCN5	1.18E-09	0.57299495	0.68	0.543	2.37E-06	NK
NCR3	1.61E-09	0.57268723	0.617	0.457	3.21E-06	NK
KLRB1	1.91E-09	1.38252868	0.586	0.443	3.81E-06	NK
RUNX3	2.10E-09	0.64877418	0.609	0.432	4.21E-06	NK
PRF1	2.58E-09	1.21435466	0.594	0.457	5.15E-06	NK
MS4A1	2.86E-09	0.20437747	0.172	0.417	5.72E-06	NK
GPRC5A	2.88E-09	0.50223142	0.688	0.526	5.77E-06	NK
FCGR3A	4.09E-09	0.51501213	0.672	0.486	8.19E-06	NK
ITGA4	4.22E-09	0.47228344	0.617	0.427	8.44E-06	NK
IL2RG	4.57E-09	0.58235314	0.57	0.343	9.13E-06	NK
GNLY	5.78E-09	1.9542409	0.688	0.683	1.16E-05	NK
FMO2	9.91E-09	0.50510437	0.836	0.779	1.98E-05	NK
XBP1	1.15E-08	0.29546362	0.805	0.6	2.29E-05	NK
SYTL3	1.17E-08	0.69015402	0.609	0.477	2.34E-05	NK
CD247	1.20E-08	1.13509145	0.602	0.389	2.40E-05	NK
SULF1	1.37E-08	0.31195801	0.734	0.595	2.73E-05	NK
PLAC8	1.37E-08	0.88123992	0.578	0.365	2.74E-05	NK
HOPX	1.39E-08	1.02010077	0.578	0.455	2.79E-05	NK
FST	1.49E-08	0.25213211	0.672	0.584	2.98E-05	NK
CYP27A1	2.63E-08	0.27324925	0.672	0.569	5.26E-05	NK
SPOCK1	2.67E-08	0.52453689	0.68	0.551	5.35E-05	NK
ABLIM1	3.13E-08	0.40843841	0.836	0.803	6.25E-05	NK
LAIR2	3.64E-08	0.54926735	0.562	0.452	7.28E-05	NK
NFKBIA	3.78E-08	0.28385264	0.969	0.891	7.56E-05	NK
CYTIP	4.18E-08	0.73610644	0.625	0.528	8.36E-05	NK
CHRDL1	4.40E-08	0.39888057	0.742	0.734	8.80E-05	NK
IKZF1	4.46E-08	0.60155745	0.57	0.411	8.91E-05	NK
GPR65	5.01E-08	0.77780916	0.562	0.398	0.00010015	NK
LRRC17	5.83E-08	0.25701415	0.742	0.657	0.00011661	NK
KLRF1	8.94E-08	0.74556791	0.547	0.436	0.00017878	NK
VEGFD	9.54E-08	0.20794339	0.625	0.525	0.00019079	NK
SFRP4	1.26E-07	0.79337417	0.633	0.509	0.00025111	NK
LCK	1.93E-07	0.60161617	0.57	0.487	0.00038591	NK
SPN	2.10E-07	0.43962462	0.531	0.42	0.0004209	NK
SLFN5	2.78E-07	0.26314597	0.758	0.584	0.00055625	NK
SLC2A3	2.89E-07	0.32296743	0.773	0.595	0.00057789	NK
MT1A	3.50E-07	0.45880209	0.828	0.755	0.00069969	NK
FGF10	4.19E-07	0.20634627	0.555	0.372	0.0008371	NK
THBS2	6.71E-07	0.27474769	0.633	0.527	0.00134136	NK
ADAMTS5	8.14E-07	0.51416672	0.664	0.578	0.00162827	NK
SDF2L1	8.32E-07	0.21057153	0.758	0.612	0.00166471	NK
OSR1	9.14E-07	0.37197881	0.609	0.501	0.00182881	NK
CD7	1.28E-06	1.15285677	0.594	0.533	0.00256525	NK
SAMD3	1.35E-06	0.63098642	0.531	0.39	0.00270479	NK
IKZF3	1.50E-06	0.68979898	0.562	0.353	0.00299214	NK
TRBC1	2.06E-06	0.88051386	0.531	0.403	0.0041192	NK
KCNE4	2.12E-06	0.28191623	0.672	0.531	0.00423023	NK
CX3CR1	2.51E-06	0.42723388	0.484	0.39	0.00501066	NK

CD2	2.69E-06	0.54164259	0.562	0.367	0.00537885	NK
HP	3.18E-06	0.30734841	0.602	0.49	0.00636668	NK
CES1	3.79E-06	0.33565101	0.664	0.587	0.00758304	NK
UCP2	4.40E-06	0.21687184	0.625	0.47	0.00879763	NK
TAGAP	5.21E-06	0.50077917	0.547	0.424	0.01041576	NK
GAS7	5.76E-06	0.47415864	0.672	0.599	0.01152965	NK
CGNL1	6.23E-06	0.2499508	0.648	0.604	0.01246641	NK
CD37	6.69E-06	0.39540749	0.656	0.56	0.01337597	NK
ACKR3	6.81E-06	0.54223647	0.562	0.45	0.01362771	NK
FAM180A	7.01E-06	0.24649221	0.633	0.591	0.01402776	NK
ALOX5AP	9.16E-06	0.3256572	0.602	0.518	0.01832622	NK
IL2RB	1.10E-05	0.69472421	0.438	0.303	0.02207073	NK
OAF	1.14E-05	0.26063402	0.625	0.475	0.0227762	NK
TRBC2	1.23E-05	0.82628009	0.531	0.388	0.02463795	NK
APCDD1	1.48E-05	0.29361658	0.602	0.521	0.02953759	NK
THY1	1.63E-05	0.31933212	0.703	0.661	0.03257402	NK
LINC00892	1.69E-05	0.2005666	0.312	0.484	0.03371974	NK
PCOLCE2	1.78E-05	0.58937883	0.602	0.532	0.03565567	NK
BCL11B	2.34E-05	0.3446481	0.562	0.456	0.04683695	NK
ECM1	3.65E-05	0.41469661	0.617	0.593	0.07307684	NK
FGFBP2	3.96E-05	0.63690454	0.609	0.613	0.0791906	NK
SNAI2	4.08E-05	0.30624248	0.641	0.57	0.08164029	NK
CYFIP2	4.10E-05	0.34426874	0.523	0.453	0.0820015	NK
TNFAIP3	4.64E-05	0.65187392	0.602	0.484	0.09274657	NK
PTPN7	4.78E-05	0.5378894	0.5	0.36	0.09550807	NK
EFHD2	5.62E-05	0.46997857	0.648	0.549	0.1123328	NK
CLMP	7.37E-05	0.39163432	0.547	0.432	0.14740013	NK
BMP4	0.00010312	0.23577831	0.641	0.565	0.20624529	NK
CRABP2	0.0003007	0.27503396	0.562	0.514	0.60139661	NK
FYB1	0.00034416	0.26881472	0.617	0.537	0.68832929	NK
SEMA3D	0.00042929	0.27591987	0.578	0.557	0.85858583	NK
FGF7	0.00060814	0.25155914	0.602	0.56	1	NK
CAMK2N1	0.0006195	0.21686169	0.641	0.579	1	NK
WNT5A	0.00091082	0.25673039	0.555	0.5	1	NK
FIBIN	0.00114766	0.24231682	0.609	0.603	1	NK
CD160	0.00124426	0.58758287	0.562	0.541	1	NK
RHOH	0.00144123	0.42910451	0.523	0.441	1	NK
NEGR1	0.00146092	0.44370379	0.562	0.488	1	NK
GFPT2	0.0015359	0.45719966	0.531	0.496	1	NK
GALNT15	0.00177594	0.40767744	0.672	0.723	1	NK
CILP	0.00188643	0.43429464	0.516	0.413	1	NK
MATK	0.00222114	0.55834997	0.453	0.47	1	NK
SIT1	0.00244464	0.20277747	0.312	0.463	1	NK
KLRC2	0.0029479	0.49100563	0.5	0.503	1	NK
MSX1	0.00314235	0.28629992	0.539	0.502	1	NK
ADGRD1	0.00336498	0.36285605	0.539	0.488	1	NK
CA12	0.00338389	0.20965457	0.523	0.469	1	NK
GBP5	0.00358251	0.31996766	0.398	0.334	1	NK
SLPI	0.0037032	0.63958588	0.539	0.461	1	NK
CHI3L2	0.003714	0.3765228	0.641	0.658	1	NK
ZNF331	0.00377707	0.38173529	0.609	0.58	1	NK
FAP	0.00451806	0.23392755	0.508	0.428	1	NK
PTGER4	0.00498379	0.23052919	0.516	0.453	1	NK

HIST1H1D	0.00593034	0.22409352	0.531	0.487	1	NK
AOX1	0.00687405	0.41431237	0.57	0.562	1	NK
PTGFR	0.00759521	0.42863609	0.531	0.521	1	NK
TRGC1	0.0079931	0.34675661	0.359	0.284	1	NK
TMEM45A	0.00833565	0.28944929	0.57	0.495	1	NK
CD3D	0.00886811	0.65231745	0.484	0.383	1	NK

Table S4. Differentially expressed genes in each smooth muscle cell cluster (GSE216860)

gene	p_val	avg_log2FC	pct.1	pct.2	p_val_adj	cluster
RERGL	0	3.05637901	0.988	0.893	0	1
LBH	0	1.97383159	0.952	0.602	0	1
SNCG	0	1.94182209	0.935	0.808	0	1
CRIP1	0	1.91567019	0.994	0.911	0	1
CSRP2	0	1.77173249	0.983	0.708	0	1
BCAM	0	1.60474763	0.982	0.91	0	1
NDUFA4L2	0	1.49148996	0.828	0.807	0	1
NTRK2	0	1.44554181	0.947	0.874	0	1
PLN	0	1.37159074	0.996	0.819	0	1
PHLDA2	0	1.3291412	0.806	0.584	0	1
TBX2-AS1	0	1.30857203	0.748	0.545	0	1
SORBS2	0	1.28551724	0.958	0.871	0	1
HES4	0	1.27807571	0.92	0.875	0	1
ITIH5	0	1.26655203	0.888	0.748	0	1
DSTN	0	1.24786056	1	0.955	0	1
KCNAB1	0	1.20939697	0.827	0.758	0	1
RCAN2	0	1.20237574	0.955	0.671	0	1
RRAD	0	1.19097044	0.901	0.686	0	1
TCF15	0	1.16682234	0.807	0.781	0	1
NET1	0	1.14882389	0.887	0.693	0	1
TAGLN	0	1.13363658	1	0.992	0	1
GJA4	0	1.11759292	0.733	0.665	0	1
RBPMS2	0	1.09226958	0.752	0.72	0	1
MYL9	0	1.08609632	1	0.972	0	1
TUBA1C	0	1.05462022	0.861	0.752	0	1
CLMN	0	1.04856109	0.901	0.775	0	1
CAV1	0	1.04161416	0.996	0.914	0	1
CCDC3	0	1.03871911	0.805	0.584	0	1
TBX2	0	1.01182944	0.82	0.64	0	1
COX4I2	0	0.99036273	0.695	0.739	0	1
CASQ2	0	0.96493442	0.705	0.408	0	1
TINAGL1	0	0.93526459	0.934	0.767	0	1
TPM2	0	0.92874925	1	0.964	0	1
NR2F2	0	0.91620695	0.946	0.816	0	1
EDNRA	0	0.91000847	0.767	0.732	0	1
MT1L	0	0.89357506	0.852	0.692	0	1
ISYNA1	0	0.89215503	0.78	0.641	0	1
CRIP2	0	0.88916865	0.953	0.814	0	1
NOTCH3	0	0.85997715	0.976	0.911	0	1
GADD45G	0	0.84652174	0.948	0.827	0	1
C11orf96	0	0.83615558	0.988	0.901	0	1
ACTA2	0	0.83375383	1	0.961	0	1
FRZB	0	0.82910643	0.677	0.473	0	1
CCND1	0	0.8069187	0.812	0.628	0	1
NT5DC2	0	0.80583092	0.74	0.591	0	1
TGFBI	0	0.80447666	0.946	0.832	0	1
LMOD1	0	0.79909663	0.979	0.843	0	1
INPP4B	0	0.76938037	0.866	0.629	0	1
KLF2	0	0.74664515	0.921	0.773	0	1
MGLL	0	0.73507719	0.778	0.55	0	1
NEXN	0	0.72703049	0.987	0.869	0	1

SYNE2	0	0.72162989	0.854	0.663	0	1
SLC7A2	0	0.7213508	0.935	0.838	0	1
AP002956.1	0	0.70670957	0.975	0.709	0	1
RAPGEF5	0	0.70550293	0.927	0.529	0	1
FABP4	0	0.69186705	0.976	0.955	0	1
TPM1	0	0.68847739	0.999	0.954	0	1
RGS16	0	0.60278783	0.828	0.451	0	1
CSRP1	0	0.59827758	0.951	0.82	0	1
MYC	0	0.57523193	0.836	0.637	0	1
RGCC	0	0.57289476	0.935	0.768	0	1
MYH11	0	0.57256633	0.999	0.923	0	1
TIMP3	0	0.5489518	0.978	0.884	0	1
FAM162B	0	0.52601747	0.813	0.66	0	1
AVPR1A	0	0.51566736	0.86	0.795	0	1
CAMK2N1	0	0.49657712	0.795	0.553	0	1
ETS2	0	0.47663299	0.852	0.557	0	1
CH25H	0	0.45461769	0.745	0.568	0	1
A2M	0	0.43994932	0.997	0.947	0	1
ACTG2	0	0.42096366	0.914	0.647	0	1
FHL5	0	0.41433551	0.873	0.708	0	1
PEG10	0	0.384376	0.787	0.555	0	1
EPHA4	0	0.37118301	0.916	0.585	0	1
SMAP2	0	0.36347738	0.637	0.385	0	1
APOLD1	0	0.35766389	0.846	0.761	0	1
PHLDA1	0	0.29804665	0.694	0.297	0	1
SCN3A	0	0.29203893	0.671	0.277	0	1
LDB2	0	-0.2535319	0.628	0.831	0	1
LSP1	0	-0.2689503	0.243	0.514	0	1
PAPPA	0	-0.2719751	0.563	0.684	0	1
GUCY1A2	0	-0.2744275	0.496	0.782	0	1
TNFSF10	0	-0.2779392	0.421	0.744	0	1
CCDC144NL	0	-0.2963075	0.542	0.676	0	1
ABCA10	0	-0.3200768	0.707	0.854	0	1
TCIM	0	-0.3535994	0.744	0.847	0	1
ART4	0	-0.3721306	0.322	0.656	0	1
SULF2	0	-0.3727273	0.495	0.683	0	1
BOC	0	-0.3810856	0.479	0.689	0	1
CHRDL1	0	-0.3935547	0.784	0.84	0	1
RSPO3	0	-0.3953422	0.626	0.837	0	1
STEAP1	0	-0.398838	0.541	0.74	0	1
CTSC	0	-0.4087568	0.51	0.814	0	1
IGSF10	0	-0.4366663	0.603	0.77	0	1
CD34	0	-0.4504524	0.603	0.828	0	1
CGNL1	0	-0.4674963	0.708	0.718	0	1
SNED1	0	-0.4850098	0.363	0.664	0	1
CLSTN2	0	-0.4887653	0.693	0.812	0	1
FLRT2	0	-0.4927651	0.598	0.823	0	1
IL32	0	-0.5050353	0.743	0.803	0	1
ABCA9	0	-0.5246902	0.676	0.799	0	1
BICC1	0	-0.5360071	0.65	0.728	0	1
LRRC17	0	-0.5438458	0.65	0.892	0	1
MEGF6	0	-0.5725265	0.491	0.686	0	1
PI16	0	-0.5802237	0.649	0.783	0	1

TGFBR3	0	-0.5909553	0.683	0.87	0	1
LTC4S	0	-0.6143883	0.825	0.78	0	1
PLAT	0	-0.6334664	0.737	0.81	0	1
MATN2	0	-0.6370958	0.402	0.699	0	1
ABCA6	0	-0.6382998	0.573	0.792	0	1
SERPINE2	0	-0.6792658	0.535	0.849	0	1
OSR1	0	-0.682715	0.541	0.65	0	1
TFPI	0	-0.7376804	0.474	0.796	0	1
KRT14	0	-0.7477512	0.552	0.796	0	1
FTL	0	-0.7622557	1	0.99	0	1
SPRY1	0	-0.7640367	0.454	0.796	0	1
ABLIM1	0	-0.7661906	0.816	0.888	0	1
KCTD12	0	-0.7772852	0.559	0.832	0	1
PLTP	0	-0.7853732	0.816	0.884	0	1
TNXB	0	-0.793281	0.668	0.786	0	1
SCARA5	0	-0.811449	0.542	0.726	0	1
F2R	0	-0.8123642	0.617	0.715	0	1
LAMB1	0	-0.8672997	0.768	0.772	0	1
HTRA3	0	-0.876841	0.682	0.901	0	1
PODN	0	-0.9217458	0.578	0.819	0	1
TSHZ2	0	-0.950883	0.76	0.823	0	1
FBLN2	0	-0.9516163	0.479	0.832	0	1
IL33	0	-0.9553136	0.67	0.742	0	1
HMCN1	0	-1.0177658	0.439	0.742	0	1
PCOLCE2	0	-1.0287361	0.545	0.826	0	1
USP53	0	-1.0810966	0.794	0.815	0	1
ABI3BP	0	-1.0913437	0.504	0.829	0	1
PRSS23	0	-1.2038921	0.49	0.683	0	1
COL14A1	0	-1.2066157	0.907	0.897	0	1
PTGDS	0	-1.2163911	0.767	0.864	0	1
ABCA8	0	-1.2274556	0.688	0.857	0	1
HSP90B1	0	-1.2448887	0.816	0.833	0	1
PRELP	0	-1.2646384	0.652	0.84	0	1
C3	0	-1.2851356	0.683	0.871	0	1
FGL2	0	-1.3909168	0.349	0.725	0	1
NNMT	0	-1.399334	0.865	0.8	0	1
LTBP1	0	-1.4721011	0.825	0.836	0	1
SULF1	0	-1.5078381	0.612	0.755	0	1
C1S	0	-1.5289395	0.792	0.767	0	1
AEBP1	0	-1.5524648	0.783	0.87	0	1
ITGBL1	0	-1.5801853	0.627	0.808	0	1
MMP2	0	-1.5884549	0.761	0.777	0	1
FBLN5	0	-1.6496224	0.494	0.749	0	1
COL1A1	0	-1.6691002	0.67	0.885	0	1
ELN	0	-1.7184025	0.437	0.74	0	1
MYH10	0	-1.7295734	0.457	0.786	0	1
FSTL1	0	-1.7428442	0.522	0.78	0	1
SERPINE1	0	-1.7496402	0.734	0.746	0	1
C7	0	-1.7551283	0.722	0.838	0	1
COL3A1	0	-1.8199986	0.739	0.923	0	1
IGFBP2	0	-1.8366615	0.86	0.857	0	1
CCN3	0	-1.9046812	0.645	0.753	0	1
LTBP2	0	-1.9531379	0.464	0.713	0	1

MEG3	0	-2.0234904	0.372	0.778	0	1
CCN2	0	-2.0593679	0.819	0.871	0	1
COL1A2	0	-2.1168105	0.794	0.882	0	1
C1R	0	-2.1949986	0.887	0.919	0	1
CFH	0	-2.2652348	0.711	0.774	0	1
EFEMP1	0	-2.3741513	0.695	0.795	0	1
FN1	0	-2.5102903	0.764	0.892	0	1
DCN	0	-2.5174083	0.911	0.936	0	1
FBLN1	0	-2.5443298	0.918	0.955	0	1
TIMP1	0	-2.5588478	0.919	0.915	0	1
ADH1B	0	-2.6785676	0.8	0.872	0	1
OGN	0	-3.0291544	0.702	0.806	0	1
VCAN	0	-3.1679733	0.716	0.827	0	1
MGP	0	-3.2533485	0.996	0.984	0	1
NRGN	8.16E-308	0.97509427	0.725	0.673	1.63E-304	1
SLIT3	9.51E-303	0.52959162	0.868	0.895	1.90E-299	1
GALNT15	1.78E-302	-0.5360433	0.747	0.71	3.57E-299	1
OLFML3	4.78E-299	-0.6620603	0.312	0.608	9.57E-296	1
GUCY1A1	4.95E-299	0.60495944	0.858	0.797	9.91E-296	1
GAP43	5.11E-299	-0.5306588	0.37	0.622	1.02E-295	1
ASPN	7.00E-298	-1.3505863	0.681	0.718	1.40E-294	1
FBXO32	6.35E-297	0.50181753	0.899	0.614	1.27E-293	1
ATP1A2	4.44E-296	0.63115169	0.769	0.586	8.87E-293	1
SRPX	6.88E-296	-0.5642933	0.628	0.764	1.38E-292	1
LEPR	2.79E-292	-0.2542269	0.586	0.76	5.58E-289	1
KCNE4	3.77E-291	0.52406551	0.657	0.39	7.55E-288	1
FAM241A	1.01E-290	0.34886424	0.663	0.42	2.01E-287	1
CCL21	8.16E-283	-0.6614633	0.632	0.763	1.63E-279	1
APOD	1.38E-281	-1.6109537	0.896	0.856	2.76E-278	1
ANGPT4	8.67E-281	0.45283587	0.682	0.61	1.73E-277	1
JCAD	2.85E-280	-0.3460204	0.68	0.68	5.69E-277	1
HELLPAR	9.08E-278	-0.2789428	0.441	0.637	1.82E-274	1
INHBA	4.25E-272	-0.4014245	0.302	0.577	8.50E-269	1
CLU	4.40E-272	-1.1609056	0.956	0.892	8.79E-269	1
G0S2	2.76E-268	-0.255026	0.33	0.594	5.53E-265	1
LINC00702	2.83E-268	0.34357184	0.9	0.644	5.67E-265	1
SFRP1	6.01E-268	-1.1447937	0.644	0.67	1.20E-264	1
COL15A1	1.78E-265	-0.5667884	0.825	0.805	3.56E-262	1
COL4A1	3.37E-259	-0.9812275	0.696	0.831	6.74E-256	1
FBN1	5.09E-257	-1.1749551	0.697	0.769	1.02E-253	1
KRT17	1.25E-256	-0.5931293	0.603	0.657	2.50E-253	1
SUSD5	1.78E-254	0.32218254	0.745	0.453	3.56E-251	1
CCL19	8.16E-254	-0.5129752	0.499	0.724	1.63E-250	1
HMCN2	1.07E-250	-0.283241	0.609	0.676	2.15E-247	1
FLNB	3.77E-249	-0.5263849	0.539	0.665	7.54E-246	1
CRISPLD2	4.96E-246	-0.5029895	0.429	0.73	9.92E-243	1
CFD	3.84E-240	-2.0598249	0.928	0.957	7.69E-237	1
CCDC80	5.45E-236	-1.1217766	0.689	0.862	1.09E-232	1
LAMA2	8.64E-236	-0.8350021	0.432	0.62	1.73E-232	1
KLHL23	5.74E-235	1.04558606	0.706	0.765	1.15E-231	1
OMD	3.49E-233	-1.5998525	0.546	0.634	6.97E-230	1
SPOCK1	3.68E-233	-0.4931649	0.482	0.662	7.36E-230	1
PDGFRL	3.61E-232	-0.4716789	0.651	0.712	7.22E-229	1

PPP1R14A	1.43E-230	0.39792705	0.98	0.866	2.85E-227	1
LOX	3.83E-228	-0.5404512	0.437	0.607	7.67E-225	1
LGI4	1.55E-227	0.72475735	0.797	0.833	3.10E-224	1
IGF1	6.29E-227	-1.0302194	0.53	0.683	1.26E-223	1
PROCR	2.21E-226	-0.3405652	0.668	0.793	4.42E-223	1
TM4SF1	4.71E-226	-0.848962	0.694	0.807	9.43E-223	1
4-Sep	5.79E-225	0.73738484	0.677	0.568	1.16E-221	1
ANK3	1.30E-221	-0.3122451	0.411	0.626	2.59E-218	1
ANGPT1	1.85E-221	0.39740416	0.749	0.535	3.70E-218	1
PMP22	2.04E-221	-0.851942	0.747	0.859	4.08E-218	1
TMEM45A	3.35E-219	-0.3442615	0.319	0.543	6.70E-216	1
C1QB	5.81E-218	-0.2637396	0.658	0.861	1.16E-214	1
ERG	5.58E-216	-0.3876595	0.616	0.664	1.12E-212	1
EPAS1	3.80E-212	0.34554877	0.909	0.812	7.60E-209	1
FBLIM1	2.79E-208	-0.7730559	0.762	0.775	5.58E-205	1
CYBA	3.30E-208	-0.4800266	0.694	0.907	6.60E-205	1
PDGFRA	1.18E-206	-0.8247896	0.376	0.591	2.35E-203	1
HIST1H4C	3.53E-204	0.5662893	0.829	0.73	7.06E-201	1
MEG8	1.47E-202	-0.2738927	0.276	0.566	2.94E-199	1
ABCC9	6.22E-201	-0.388747	0.625	0.81	1.24E-197	1
PCDH7	1.29E-198	-0.9745885	0.402	0.599	2.58E-195	1
TFPI2	1.08E-197	-0.7483515	0.457	0.581	2.16E-194	1
THSD4	2.14E-197	-0.2538896	0.631	0.651	4.28E-194	1
C12orf75	1.83E-196	0.59405386	0.932	0.658	3.66E-193	1
PDLIM1	1.38E-195	0.50761302	0.81	0.734	2.76E-192	1
COL8A1	5.63E-195	-0.923296	0.595	0.645	1.13E-191	1
SPON2	4.91E-192	-0.3028786	0.462	0.628	9.83E-189	1
IGFBP6	1.70E-191	-1.1590411	0.949	0.907	3.39E-188	1
MT1E	6.32E-191	0.44379761	0.972	0.845	1.26E-187	1
CKB	4.02E-188	0.58459985	0.805	0.727	8.04E-185	1
AKAP12	1.12E-183	-0.7815987	0.663	0.847	2.23E-180	1
SMOC2	2.89E-183	-0.7618561	0.524	0.671	5.78E-180	1
LUM	3.59E-182	-1.6212534	0.724	0.732	7.18E-179	1
PRPH	1.10E-181	1.06225407	0.664	0.704	2.19E-178	1
ACKR4	1.17E-180	-0.2591724	0.485	0.607	2.34E-177	1
HES1	1.25E-180	0.54736974	0.803	0.755	2.50E-177	1
SPARC	1.44E-174	-0.9126298	0.926	0.882	2.88E-171	1
TNC	1.39E-170	-0.2958759	0.592	0.372	2.78E-167	1
CTSK	3.03E-169	-0.7272951	0.716	0.744	6.06E-166	1
COL4A2	7.87E-168	-0.7289037	0.847	0.866	1.57E-164	1
COL6A3	1.05E-165	-1.1492011	0.691	0.708	2.09E-162	1
ITGA7	2.73E-158	0.29626628	0.765	0.729	5.46E-155	1
AQP1	1.32E-157	-0.8929927	0.858	0.802	2.64E-154	1
PTGIS	4.26E-157	-0.8795464	0.446	0.604	8.52E-154	1
GJA1	8.33E-157	-0.7788651	0.332	0.571	1.67E-153	1
TNFRSF11B	4.98E-156	-1.4137651	0.625	0.634	9.96E-153	1
SPON1	1.19E-154	-0.631303	0.473	0.584	2.37E-151	1
LIMCH1	2.47E-154	-0.7415107	0.689	0.664	4.94E-151	1
FILIP1L	6.50E-153	0.41410702	0.986	0.889	1.30E-149	1
TNFAIP6	5.58E-149	-0.2808206	0.605	0.649	1.12E-145	1
PALMD	5.59E-149	-0.2504959	0.798	0.775	1.12E-145	1
TMEM176B	1.74E-147	-0.4537708	0.765	0.726	3.48E-144	1
FMO2	1.24E-146	-0.9585378	0.791	0.727	2.49E-143	1

PLA2G2A	8.54E-145	-2.207589	0.91	0.941	1.71E-141	1
CXCL12	1.12E-144	-0.7099329	0.762	0.829	2.23E-141	1
POSTN	1.12E-144	-0.613481	0.766	0.462	2.25E-141	1
CRLF1	5.42E-143	-0.7311625	0.492	0.613	1.08E-139	1
FIBIN	7.96E-142	-0.6569217	0.695	0.767	1.59E-138	1
CNN1	1.17E-136	0.34488053	0.899	0.761	2.35E-133	1
H19	1.33E-136	-0.4849374	0.728	0.817	2.67E-133	1
SLC9A3R2	1.16E-133	-0.3209619	0.634	0.766	2.33E-130	1
COX7A1	2.88E-133	0.36498478	0.821	0.683	5.75E-130	1
SEMA3D	2.97E-132	-0.4684842	0.505	0.578	5.94E-129	1
RHOU	2.47E-131	-0.2938406	0.543	0.668	4.94E-128	1
THBS2	5.61E-131	-1.124233	0.551	0.595	1.12E-127	1
PGF	9.88E-131	0.87320751	0.614	0.549	1.98E-127	1
HLA-DRA	2.89E-128	-0.5110327	0.757	0.907	5.78E-125	1
CCL2	4.59E-128	-0.2612391	0.724	0.455	9.17E-125	1
CCN1	2.71E-126	-0.8161661	0.913	0.865	5.41E-123	1
GAS7	1.06E-125	-0.3274581	0.44	0.591	2.12E-122	1
CPXM2	1.77E-124	-0.6670763	0.635	0.612	3.54E-121	1
TCEAL2	7.72E-124	-0.5074564	0.426	0.563	1.54E-120	1
MGST1	1.03E-121	-0.5267212	0.527	0.341	2.05E-118	1
TPD52L1	9.81E-119	-0.349319	0.673	0.66	1.96E-115	1
MAP3K7CL	2.31E-116	0.61869286	0.718	0.713	4.62E-113	1
DAB2	1.86E-115	-0.4783143	0.538	0.623	3.71E-112	1
APOE	1.96E-114	-1.4515882	0.623	0.77	3.91E-111	1
RASL11A	3.83E-113	0.5125831	0.711	0.665	7.66E-110	1
FST	1.14E-112	-0.3828557	0.63	0.655	2.28E-109	1
EGFL7	6.36E-111	-0.3282724	0.844	0.896	1.27E-107	1
CREB5	1.14E-110	-0.4850206	0.803	0.737	2.28E-107	1
HIGD1B	2.55E-110	0.41395584	0.373	0.597	5.09E-107	1
IGFBP3	3.35E-110	-0.9849881	0.817	0.75	6.71E-107	1
PLCB4	2.90E-107	0.34379117	0.647	0.549	5.81E-104	1
FKBP11	1.60E-106	-0.2764522	0.56	0.66	3.21E-103	1
SMOC1	2.29E-105	-0.3520698	0.435	0.574	4.59E-102	1
LIFR	3.89E-105	-0.3114009	0.834	0.905	7.79E-102	1
IFI27	3.96E-105	-0.3600815	0.59	0.836	7.93E-102	1
SORL1	9.15E-105	0.2724169	0.596	0.476	1.83E-101	1
GPC3	6.82E-104	-0.4916423	0.491	0.578	1.36E-100	1
AP000892.3	1.53E-101	0.28302919	0.825	0.685	3.06E-98	1
PTP4A3	3.94E-100	0.82918856	0.559	0.406	7.88E-97	1
RNASE1	2.05E-98	-0.285424	0.636	0.814	4.11E-95	1
WFDC1	4.98E-97	0.62064333	0.584	0.468	9.95E-94	1
MAFB	4.23E-96	0.37115651	0.774	0.735	8.46E-93	1
TNFRSF12A	1.29E-94	-0.6421157	0.515	0.633	2.57E-91	1
GPRIN3	7.57E-93	0.39497367	0.645	0.692	1.51E-89	1
GEM	1.60E-90	-0.7117011	0.769	0.774	3.21E-87	1
ENTPD3	6.04E-89	0.9429053	0.587	0.761	1.21E-85	1
SLC40A1	7.39E-87	-0.3342245	0.425	0.621	1.48E-83	1
ESAM	1.44E-86	0.52094307	0.685	0.704	2.88E-83	1
TGM2	3.24E-86	-0.6396653	0.841	0.739	6.48E-83	1
FMOD	3.10E-85	-0.5879264	0.548	0.601	6.20E-82	1
ITGA8	1.30E-78	-0.6631622	0.732	0.791	2.59E-75	1
RARRES1	3.59E-78	-0.9928606	0.814	0.752	7.18E-75	1
BASP1	9.72E-78	-0.48093	0.443	0.549	1.94E-74	1

CTSZ	1.21E-75	-0.3898543	0.735	0.875	2.42E-72	1
PDLIM3	3.49E-74	-0.5742529	0.84	0.781	6.98E-71	1
HIST1H1C	1.67E-73	0.31671691	0.557	0.412	3.34E-70	1
PDGFD	1.77E-73	-0.6163278	0.458	0.555	3.54E-70	1
FHL1	3.75E-73	-0.546033	0.931	0.842	7.50E-70	1
SPINT2	8.61E-72	-0.6634069	0.42	0.541	1.72E-68	1
SYNM	1.34E-70	0.31422985	0.679	0.607	2.67E-67	1
CD36	5.88E-69	-0.4300984	0.863	0.916	1.18E-65	1
SRGN	3.32E-67	-0.382252	0.417	0.564	6.64E-64	1
PCDH1	2.38E-66	0.32813167	0.707	0.719	4.77E-63	1
CAPG	8.24E-64	0.2930683	0.628	0.55	1.65E-60	1
CYFIP2	9.16E-64	-0.3399742	0.64	0.443	1.83E-60	1
KCNMB1	5.07E-63	0.33675398	0.672	0.603	1.01E-59	1
PGAM2	2.47E-60	0.421177	0.492	0.326	4.95E-57	1
NPDC1	3.94E-59	-0.450421	0.814	0.798	7.87E-56	1
DUSP6	1.73E-58	-0.539097	0.513	0.556	3.46E-55	1
ITPR2	9.88E-58	-0.3659829	0.566	0.583	1.98E-54	1
PRUNE2	5.99E-56	-0.6228258	0.663	0.63	1.20E-52	1
DPT	4.31E-55	-0.6031622	0.35	0.51	8.63E-52	1
BGN	9.70E-55	-0.6184192	0.985	0.915	1.94E-51	1
NEDD9	2.15E-50	-0.3381643	0.852	0.591	4.31E-47	1
HLA-DRB1	1.56E-49	-0.341005	0.747	0.829	3.13E-46	1
CADM1	8.77E-49	0.26110973	0.507	0.383	1.75E-45	1
ATP1B1	7.43E-48	-0.3394867	0.52	0.389	1.49E-44	1
ITGA10	1.15E-47	-0.8730438	0.666	0.587	2.29E-44	1
LMCD1	3.00E-46	-0.7398727	0.721	0.648	6.00E-43	1
ECM1	3.99E-46	-0.3562131	0.606	0.612	7.97E-43	1
CTSD	6.49E-46	-0.4300136	0.789	0.821	1.30E-42	1
NPY1R	1.11E-44	0.44856628	0.74	0.747	2.22E-41	1
NPC2	8.41E-43	-0.4241921	0.717	0.772	1.68E-39	1
PLIN2	1.45E-42	-0.3396438	0.669	0.516	2.90E-39	1
CYP1B1	6.12E-42	-0.4596722	0.449	0.496	1.22E-38	1
SLPI	5.49E-41	-0.3452012	0.529	0.417	1.10E-37	1
DKK3	2.03E-40	-0.4388467	0.81	0.782	4.07E-37	1
DES	2.43E-39	-1.0336716	0.742	0.506	4.86E-36	1
RBP4	8.40E-38	-0.3074781	0.317	0.329	1.68E-34	1
UCHL1	2.84E-37	-0.2917358	0.528	0.543	5.68E-34	1
DIO2	4.31E-37	-0.355406	0.665	0.482	8.62E-34	1
MBP	4.10E-36	-0.2934966	0.667	0.616	8.21E-33	1
IL13RA2	1.11E-35	-0.254749	0.287	0.336	2.21E-32	1
SLIT2	2.12E-35	-0.5974653	0.586	0.547	4.24E-32	1
FNBP1L	5.27E-35	0.31517056	0.817	0.852	1.05E-31	1
CCN5	2.77E-33	-0.8693701	0.493	0.529	5.54E-30	1
AGT	9.58E-33	-0.430384	0.558	0.626	1.92E-29	1
APOC1	3.82E-32	-0.2662413	0.556	0.608	7.63E-29	1
FAM180A	1.48E-31	-0.2695913	0.785	0.647	2.96E-28	1
GGT5	4.51E-31	-0.7556575	0.653	0.633	9.01E-28	1
PPFIBP1	1.58E-30	-0.3002227	0.629	0.646	3.15E-27	1
MT1X	1.32E-25	-0.2867165	0.933	0.771	2.64E-22	1
TMEM176A	3.22E-25	-0.3894172	0.641	0.612	6.43E-22	1
HLA-B	1.18E-23	-0.3856141	0.885	0.94	2.36E-20	1
LINC02381	2.85E-22	0.4014538	0.501	0.764	5.70E-19	1
CNKSR3	3.68E-22	-0.276085	0.559	0.558	7.36E-19	1

C5orf46	6.26E-22	-0.3984836	0.45	0.504	1.25E-18	1
OSR2	3.71E-21	-0.3973226	0.504	0.516	7.41E-18	1
NID1	5.93E-21	-0.3433732	0.671	0.685	1.19E-17	1
SYTL2	9.45E-21	0.35092576	0.552	0.534	1.89E-17	1
NEGR1	6.50E-20	-0.3456994	0.483	0.421	1.30E-16	1
PECAM1	1.87E-19	-0.27705	0.857	0.885	3.73E-16	1
NRARP	2.14E-19	0.52524699	0.52	0.559	4.28E-16	1
TNMD	4.55E-19	0.26844379	0.412	0.504	9.11E-16	1
CDC42EP3	7.68E-19	-0.4770341	0.416	0.488	1.54E-15	1
ADGRF5	7.92E-19	-0.3392573	0.766	0.652	1.58E-15	1
SUSD2	2.49E-18	0.44469055	0.517	0.513	4.98E-15	1
ITGA5	2.63E-18	-0.5161011	0.72	0.51	5.27E-15	1
IGFBP4	3.56E-18	-0.3171834	0.894	0.774	7.12E-15	1
ALCAM	1.45E-17	-0.4368176	0.407	0.483	2.90E-14	1
ISG15	3.35E-17	0.28660614	0.63	0.634	6.70E-14	1
GPM6B	7.38E-17	-0.6438399	0.729	0.622	1.48E-13	1
CPM	1.68E-13	0.38740763	0.62	0.725	3.35E-10	1
UAP1	3.49E-13	-0.2804323	0.467	0.413	6.97E-10	1
PPP1R1A	1.06E-12	0.40665392	0.506	0.453	2.12E-09	1
MEDAG	2.15E-12	-0.3289534	0.58	0.468	4.30E-09	1
PCOLCE	3.19E-12	-0.6555624	0.806	0.787	6.39E-09	1
VGLL3	5.50E-12	-0.3857034	0.679	0.631	1.10E-08	1
EMP1	6.42E-12	-0.7010863	0.705	0.607	1.28E-08	1
SCRG1	1.66E-11	-0.2679518	0.355	0.413	3.32E-08	1
CES1	8.30E-11	-0.4173047	0.71	0.489	1.66E-07	1
PCDH9	1.29E-10	-0.3318636	0.475	0.495	2.58E-07	1
CDH13	1.66E-10	-0.3917068	0.47	0.542	3.33E-07	1
CD74	2.48E-10	-0.4998559	0.77	0.792	4.97E-07	1
TBX3	3.40E-10	0.33292277	0.521	0.528	6.79E-07	1
CD55	4.24E-10	-0.3928131	0.615	0.574	8.48E-07	1
ADAP2	1.24E-09	0.53746423	0.493	0.511	2.47E-06	1
CP	1.73E-09	-0.4824031	0.387	0.478	3.46E-06	1
INMT	4.64E-09	-0.3424016	0.533	0.464	9.29E-06	1
STEAP4	5.66E-09	-0.7103066	0.803	0.757	1.13E-05	1
CLMP	6.69E-09	-0.2587066	0.35	0.415	1.34E-05	1
NRP2	1.24E-08	-0.3125648	0.732	0.543	2.48E-05	1
MAP1B	1.30E-08	-0.2551344	0.942	0.867	2.60E-05	1
MYOC	1.99E-08	-0.4870628	0.753	0.58	3.98E-05	1
AKR1C1	4.92E-08	-0.3849839	0.537	0.44	9.84E-05	1
KCNQ1OT1	1.38E-07	-0.3719526	0.444	0.5	0.00027561	1
HLA-DPA1	4.98E-07	-0.322705	0.778	0.771	0.00099554	1
GAS1	6.60E-07	-0.4235317	0.69	0.598	0.00131955	1
SVEP1	9.16E-06	-0.3247596	0.736	0.607	0.01832436	1
THY1	1.48E-05	-0.4344125	0.703	0.536	0.02952782	1
ENG	2.15E-05	-0.4987961	0.679	0.616	0.04293046	1
IER3	2.61E-05	-0.322608	0.568	0.578	0.05215245	1
SAT1	2.87E-05	-0.3710501	0.716	0.728	0.05745587	1
VWF	0.00022971	-0.4079742	0.862	0.842	0.4594197	1
FHL2	0.00024965	-0.2802173	0.777	0.702	0.49930725	1
NEURL1B	0.00043131	0.4002841	0.519	0.673	0.86262338	1
GNG11	0.00046666	-0.287633	0.86	0.871	0.93331415	1
GRIA2	0.00048192	-0.3541583	0.273	0.432	0.96384091	1
ENPP2	0.00094863	-0.3093154	0.485	0.449	1	1

CDO1	0.00122059	-0.3089104	0.638	0.54	1	1
AKR1C3	0.00281318	-0.3014332	0.601	0.522	1	1
BMP4	0.00340031	-0.2592776	0.518	0.505	1	1
CXCL14	0.00340449	-1.1716074	0.601	0.54	1	1
CYTL1	0.00563312	-0.357633	0.465	0.439	1	1
PDGFB	0.00603063	0.31566415	0.388	0.275	1	1
COL4A4	0.00639349	-0.2889029	0.381	0.433	1	1
EBF2	0.00842617	0.32759381	0.582	0.685	1	1
MGP	0	2.98469084	1	0.986	0	2
OGN	0	2.82118899	0.949	0.672	0	2
VCAN	0	2.68588793	0.947	0.698	0	2
FN1	0	2.4245681	0.985	0.762	0	2
CCN2	0	2.40292265	0.987	0.785	0	2
TIMP1	0	2.39352664	0.989	0.887	0	2
LTBP2	0	2.0365057	0.879	0.466	0	2
CCN3	0	2.01257009	0.916	0.608	0	2
SERPINE1	0	1.9797073	0.931	0.661	0	2
EFEMP1	0	1.97547017	0.931	0.667	0	2
OMD	0	1.78714411	0.792	0.506	0	2
COL1A2	0	1.76890948	0.992	0.773	0	2
IGFBP2	0	1.72590088	0.965	0.814	0	2
LTBP1	0	1.70760318	0.976	0.77	0	2
ASPN	0	1.6917928	0.885	0.622	0	2
ELN	0	1.68462544	0.92	0.448	0	2
MYH10	0	1.67705438	0.922	0.494	0	2
CFH	0	1.66386804	0.88	0.685	0	2
AEBP1	0	1.58424211	0.983	0.761	0	2
FBLN5	0	1.57394119	0.897	0.505	0	2
SULF1	0	1.51287548	0.918	0.585	0	2
COL3A1	0	1.47004595	0.983	0.767	0	2
TNFRSF11B	0	1.46133727	0.778	0.568	0	2
FSTL1	0	1.41770662	0.931	0.532	0	2
PRSS23	0	1.35096449	0.85	0.476	0	2
ADH1B	0	1.32713175	0.95	0.787	0	2
FGL2	0	1.26832258	0.835	0.41	0	2
ITGBL1	0	1.25620535	0.876	0.65	0	2
MEG3	0	1.23027473	0.836	0.464	0	2
BGN	0	1.22312629	0.994	0.932	0	2
SPARC	0	1.21698441	0.985	0.871	0	2
CLU	0	1.16843597	0.986	0.899	0	2
CCN1	0	1.14999185	0.976	0.853	0	2
HSP90B1	0	1.12674333	0.97	0.764	0	2
FBLN1	0	1.11682486	0.987	0.915	0	2
COL14A1	0	1.10159129	0.982	0.869	0	2
COL1A1	0	1.09942669	0.979	0.692	0	2
PRELP	0	1.09874495	0.957	0.657	0	2
HMCN1	0	1.09515422	0.92	0.451	0	2
KRT14	0	1.05849588	0.958	0.554	0	2
IL33	0	1.00366647	0.909	0.621	0	2
PCOLCE2	0	0.98520587	0.899	0.595	0	2
TFPI2	0	0.97358831	0.696	0.445	0	2
AQP1	0	0.96914	0.937	0.787	0	2
F2R	0	0.93122316	0.835	0.595	0	2

C1R	0	0.92957171	0.953	0.882	0	2
FBLIM1	0	0.92381186	0.897	0.715	0	2
TNFRSF12A	0	0.92049773	0.811	0.475	0	2
COL8A1	0	0.91363023	0.774	0.555	0	2
FHL1	0	0.90051322	0.959	0.857	0	2
SMOC2	0	0.89862907	0.849	0.492	0	2
NNMT	0	0.86302576	0.909	0.801	0	2
FIBIN	0	0.80765028	0.836	0.687	0	2
KRT17	0	0.78590701	0.758	0.576	0	2
PCDH7	0	0.7835434	0.74	0.399	0	2
ITGA10	0	0.7816993	0.721	0.588	0	2
COL4A1	0	0.77785484	0.97	0.677	0	2
COL4A2	0	0.76242948	0.971	0.809	0	2
ITGA8	0	0.76131156	0.933	0.69	0	2
GEM	0	0.74946934	0.836	0.745	0	2
SFRP1	0	0.74438588	0.749	0.618	0	2
GAP43	0	0.73826304	0.739	0.393	0	2
LIMCH1	0	0.73469864	0.811	0.621	0	2
OSR1	0	0.73359333	0.848	0.49	0	2
SPINT2	0	0.72412829	0.691	0.393	0	2
PTGIS	0	0.7122404	0.734	0.437	0	2
SERPINE2	0	0.7104804	0.911	0.599	0	2
DKK3	0	0.70399155	0.923	0.743	0	2
PDLIM3	0	0.6844386	0.913	0.768	0	2
PRUNE2	0	0.66296762	0.748	0.605	0	2
TM4SF1	0	0.65402163	0.891	0.691	0	2
TGM2	0	0.6524063	0.842	0.769	0	2
CPXM2	0	0.64993746	0.715	0.586	0	2
MMP2	0	0.63294395	0.855	0.733	0	2
MATN2	0	0.61919279	0.879	0.412	0	2
FBN1	0	0.61208388	0.861	0.68	0	2
LRRC17	0	0.61171705	0.934	0.701	0	2
CTSZ	0	0.60356611	0.962	0.739	0	2
PTGDS	0	0.59474791	0.91	0.775	0	2
LAMB1	0	0.59056123	0.83	0.745	0	2
CRLF1	0	0.57075827	0.749	0.47	0	2
INHBA	0	0.5451184	0.787	0.293	0	2
DUSP6	0	0.49024264	0.721	0.456	0	2
FBLN2	0	0.48804666	0.882	0.559	0	2
CGNL1	0	0.47666412	0.833	0.663	0	2
MEGF6	0	0.47233461	0.846	0.481	0	2
STEAP1	0	0.47163593	0.878	0.54	0	2
C7	0	0.45261327	0.915	0.723	0	2
CREB5	0	0.43843084	0.905	0.714	0	2
KCTD12	0	0.43579668	0.859	0.626	0	2
ERG	0	0.42900073	0.725	0.604	0	2
TFPI	0	0.42289103	0.87	0.535	0	2
CCDC144NL	0	0.41519358	0.876	0.497	0	2
C1S	0	0.41245001	0.873	0.741	0	2
SMOC1	0	0.39318523	0.799	0.381	0	2
ABLIM1	0	0.38434891	0.94	0.815	0	2
SEMA3D	0	0.37779525	0.721	0.466	0	2
GALNT15	0	0.37095388	0.821	0.69	0	2

LOX	0	0.34859402	0.77	0.418	0	2
HTRA3	0	0.34384712	0.943	0.727	0	2
JCAD	0	0.33963511	0.831	0.617	0	2
TMEM45A	0	0.3046938	0.696	0.32	0	2
CXCL12	0	0.3045551	0.924	0.741	0	2
MYOZ2	0	0.3022853	0.815	0.353	0	2
ITPR2	0	0.29596309	0.742	0.505	0	2
ABCA8	0	0.27958295	0.948	0.698	0	2
FAP	0	0.26916989	0.747	0.295	0	2
THSD4	0	0.25854805	0.771	0.587	0	2
PPARG	0	-0.2975196	0.595	0.715	0	2
SCN3A	0	-0.3178459	0.24	0.573	0	2
EPHA4	0	-0.342746	0.588	0.821	0	2
COL25A1	0	-0.344495	0.708	0.778	0	2
C3	0	-0.3538701	0.97	0.696	0	2
RGCC	0	-0.4949839	0.732	0.902	0	2
FAM162B	0	-0.575609	0.662	0.769	0	2
APOLD1	0	-0.6965263	0.743	0.829	0	2
RAPGEF5	0	-0.7203874	0.515	0.82	0	2
AVPR1A	0	-0.7785835	0.838	0.824	0	2
GADD45G	0	-0.8217068	0.828	0.913	0	2
INPP4B	0	-0.9824047	0.59	0.815	0	2
NR2F2	0	-1.0851894	0.865	0.889	0	2
TCF15	0	-1.0955509	0.866	0.765	0	2
TGFBI	0	-1.1192002	0.871	0.897	0	2
TIMP3	0	-1.158651	0.893	0.947	0	2
NOTCH3	0	-1.1990994	0.936	0.947	0	2
SORBS2	0	-1.2185689	0.911	0.916	0	2
RRAD	0	-1.2467097	0.637	0.86	0	2
SYNE2	0	-1.2718958	0.7	0.784	0	2
ITIH5	0	-1.2834519	0.759	0.843	0	2
CLMN	0	-1.3124342	0.801	0.855	0	2
CSRP2	0	-1.4795531	0.766	0.881	0	2
CRIP1	0	-1.4900624	0.943	0.957	0	2
BCAM	0	-1.6258433	0.962	0.94	0	2
NTRK2	0	-1.6578962	0.904	0.914	0	2
SNCG	0	-1.7289639	0.828	0.891	0	2
FABP4	0	-1.9368202	0.977	0.961	0	2
LBH	0	-2.0120571	0.67	0.824	0	2
RERGL	0	-3.1181164	0.935	0.943	0	2
PLAT	3.29E-307	0.36623993	0.846	0.743	6.57E-304	2
ART4	9.11E-306	0.34099982	0.715	0.393	1.82E-302	2
ADAMTS4	2.05E-301	-0.306704	0.294	0.605	4.09E-298	2
CCDC3	4.05E-297	-0.9515603	0.557	0.753	8.10E-294	2
SLC7A2	2.19E-296	-0.9194007	0.885	0.888	4.37E-293	2
LAMA2	1.36E-285	0.52298486	0.712	0.448	2.71E-282	2
SULF2	1.98E-281	0.2683973	0.727	0.53	3.96E-278	2
HES4	7.98E-279	-1.0950775	0.931	0.884	1.60E-275	2
PHLDA1	8.41E-278	-0.3659849	0.329	0.567	1.68E-274	2
TINAGL1	9.53E-277	-0.921144	0.859	0.848	1.91E-273	2
SPRY1	1.24E-274	0.41678934	0.838	0.535	2.47E-271	2
THBS2	8.89E-272	0.96324668	0.696	0.522	1.78E-268	2
BASP1	8.79E-271	0.47680081	0.683	0.417	1.76E-267	2

SPON1	1.73E-268	0.60521608	0.683	0.464	3.46E-265	2
USP53	3.32E-266	0.45670846	0.836	0.791	6.64E-263	2
GJA4	1.64E-264	-1.1759829	0.638	0.724	3.27E-261	2
IL32	1.02E-263	0.39600176	0.85	0.74	2.05E-260	2
TIMP4	3.75E-246	-0.3562191	0.416	0.704	7.50E-243	2
PMP22	1.05E-244	0.50189042	0.904	0.76	2.11E-241	2
APOE	2.41E-237	1.57500187	0.901	0.611	4.81E-234	2
CAV1	2.08E-231	-0.6428966	0.983	0.944	4.17E-228	2
TBX2	2.61E-230	-1.1021704	0.692	0.747	5.22E-227	2
CBLN1	3.21E-230	-0.2610938	0.525	0.279	6.43E-227	2
MYC	8.61E-227	-0.665757	0.644	0.777	1.72E-223	2
PDGFD	8.65E-227	0.61317119	0.666	0.44	1.73E-223	2
TCEAL2	1.97E-226	0.55757733	0.666	0.422	3.94E-223	2
CYBA	2.03E-226	0.26176556	0.95	0.737	4.06E-223	2
CTSK	6.81E-218	0.51303091	0.795	0.703	1.36E-214	2
TNXB	7.54E-215	0.36877187	0.791	0.7	1.51E-211	2
CASQ2	4.33E-213	-0.9401166	0.404	0.622	8.66E-210	2
DSTN	2.21E-211	-0.5902022	0.994	0.971	4.41E-208	2
MAP1B	8.23E-210	0.50873583	0.972	0.877	1.65E-206	2
CH25H	8.42E-209	-0.5052506	0.563	0.696	1.68E-205	2
A2M	4.03E-207	-0.6554687	0.971	0.973	8.05E-204	2
KCNAB1	5.59E-205	-1.1678813	0.857	0.766	1.12E-201	2
TSPAN2	3.20E-204	0.25969956	0.622	0.353	6.39E-201	2
RGS16	1.85E-203	-0.7834742	0.496	0.702	3.69E-200	2
TNFAIP6	3.69E-203	0.30118416	0.706	0.594	7.39E-200	2
LMCD1	1.76E-202	0.51970593	0.739	0.662	3.53E-199	2
C5orf46	2.85E-202	0.47793842	0.654	0.403	5.69E-199	2
AP002956.1	2.99E-201	-0.5712565	0.777	0.871	5.98E-198	2
NET1	7.67E-198	-1.0187442	0.841	0.771	1.53E-194	2
AKAP12	1.35E-197	0.51059619	0.901	0.693	2.69E-194	2
PTN	1.13E-196	0.47306075	0.893	0.592	2.26E-193	2
CCDC80	1.09E-194	0.35694519	0.932	0.709	2.18E-191	2
AGT	1.04E-193	0.5248018	0.76	0.522	2.07E-190	2
FMO2	3.30E-192	0.46522729	0.799	0.743	6.60E-189	2
RAMP1	1.74E-191	0.63200756	0.83	0.677	3.48E-188	2
TMEM47	1.52E-189	0.48338641	0.795	0.688	3.05E-186	2
CDH13	2.25E-188	0.57526364	0.692	0.428	4.49E-185	2
RCAN2	2.50E-188	-0.9050117	0.778	0.83	5.00E-185	2
MGLL	2.71E-185	-0.8151722	0.583	0.699	5.41E-182	2
APCDD1	2.92E-177	0.36428118	0.782	0.601	5.84E-174	2
PHLDA2	2.00E-172	-1.108616	0.692	0.698	4.01E-169	2
MT2A	3.90E-172	-0.6637766	0.995	0.978	7.79E-169	2
EDNRA	9.88E-170	-0.9225519	0.775	0.739	1.98E-166	2
GPRIN3	9.18E-169	-0.4747622	0.694	0.658	1.84E-165	2
ADRA2A	2.93E-168	-0.2911686	0.33	0.249	5.85E-165	2
CD9	2.38E-164	0.44482103	0.935	0.775	4.75E-161	2
COX4I2	3.25E-164	-1.1405665	0.788	0.687	6.50E-161	2
FTL	1.16E-162	0.26950869	1	0.993	2.33E-159	2
LTC4S	3.96E-159	0.56281008	0.753	0.823	7.92E-156	2
PDGFRA	2.46E-156	0.37529977	0.656	0.41	4.92E-153	2
PPFIBP1	3.19E-156	0.27404291	0.745	0.592	6.38E-153	2
OLFML3	9.68E-153	0.32849273	0.662	0.374	1.94E-149	2
KCNE4	2.22E-145	-0.5167315	0.411	0.572	4.43E-142	2

CSTB	4.68E-141	0.26360213	0.87	0.671	9.37E-138	2
HSPB7	6.92E-141	0.42560036	0.637	0.387	1.38E-137	2
PLN	4.12E-139	-0.7434828	0.922	0.903	8.23E-136	2
TPD52L1	1.84E-134	0.28529872	0.684	0.659	3.67E-131	2
MT1G	1.67E-133	-0.3368503	0.422	0.603	3.34E-130	2
TAGLN	8.09E-131	-0.4749362	0.999	0.995	1.62E-127	2
MICAL2	4.63E-128	0.36523398	0.729	0.645	9.25E-125	2
ISYNA1	6.11E-128	-0.8953888	0.719	0.708	1.22E-124	2
MYL9	2.12E-127	-0.4359839	0.996	0.982	4.23E-124	2
S100A16	3.21E-123	0.48263703	0.721	0.672	6.42E-120	2
SHROOM3	9.17E-121	0.45574139	0.607	0.495	1.83E-117	2
ETS1	6.79E-119	-0.2717657	0.422	0.603	1.36E-115	2
TUBA1C	3.88E-118	-0.8720943	0.803	0.808	7.75E-115	2
LUM	4.78E-118	0.76901501	0.77	0.71	9.55E-115	2
COL15A1	5.10E-118	0.43794349	0.8	0.821	1.02E-114	2
ANGPTL4	5.89E-118	-0.3885252	0.793	0.711	1.18E-114	2
VGLL3	3.24E-117	0.39842678	0.682	0.645	6.48E-114	2
COL6A3	4.02E-115	0.39309694	0.735	0.684	8.03E-112	2
KLHL23	4.25E-115	-1.0757794	0.817	0.701	8.50E-112	2
MT1L	9.72E-115	-0.8386318	0.706	0.801	1.94E-111	2
MTUS1	8.57E-113	-0.3950407	0.829	0.878	1.71E-109	2
ALCAM	4.41E-110	0.39221908	0.607	0.377	8.82E-107	2
GGT5	9.68E-109	0.3705664	0.722	0.61	1.94E-105	2
MT1M	2.35E-106	-0.865976	0.889	0.897	4.70E-103	2
RBPMS2	2.56E-106	-1.0394417	0.856	0.687	5.11E-103	2
CARMN	5.91E-106	-0.7199618	0.789	0.819	1.18E-102	2
PLCB4	2.07E-104	-0.4950802	0.51	0.635	4.15E-101	2
TBX2-AS1	2.00E-103	-1.1169651	0.679	0.635	4.01E-100	2
CD55	7.91E-101	0.31236745	0.687	0.556	1.58E-97	2
APOC1	5.01E-100	0.26417874	0.664	0.548	1.00E-96	2
FHL2	4.72E-97	0.31760095	0.785	0.721	9.43E-94	2
ATP1A2	1.55E-95	-0.6286813	0.599	0.711	3.09E-92	2
4-Sep	1.48E-94	-0.8057701	0.608	0.63	2.97E-91	2
C11orf96	6.76E-94	-0.4188836	0.948	0.944	1.35E-90	2
PCDH1	3.10E-93	-0.3925931	0.703	0.717	6.21E-90	2
COMP	5.06E-93	0.82063389	0.596	0.401	1.01E-89	2
ITGA7	5.45E-93	-0.7646901	0.749	0.747	1.09E-89	2
SORL1	1.05E-92	-0.3917148	0.447	0.574	2.10E-89	2
PRPH	6.82E-92	-0.9984178	0.758	0.653	1.36E-88	2
MT1A	4.09E-91	-0.5433613	0.695	0.779	8.17E-88	2
GNG11	1.84E-88	0.27457413	0.926	0.84	3.68E-85	2
EMP1	2.23E-88	0.25427495	0.71	0.634	4.47E-85	2
SORBS1	5.23E-88	0.25462468	0.8	0.627	1.05E-84	2
LGI4	6.07E-88	-0.9004617	0.91	0.775	1.21E-84	2
TPM1	3.92E-87	-0.2753334	0.991	0.97	7.84E-84	2
GJA1	1.00E-86	0.67720549	0.6	0.388	2.01E-83	2
IER3	1.34E-86	0.30501412	0.686	0.526	2.67E-83	2
FRZB	3.74E-83	-0.7653605	0.579	0.575	7.48E-80	2
FHL5	1.72E-82	-0.2867615	0.682	0.837	3.44E-79	2
MT1E	3.92E-82	-0.6778597	0.949	0.893	7.84E-79	2
ANGPT2	7.26E-79	-0.3659662	0.773	0.548	1.45E-75	2
ANGPT4	3.01E-78	-0.4153158	0.702	0.623	6.02E-75	2
KLF2	9.48E-78	-0.7952566	0.853	0.846	1.90E-74	2

SLIT3	1.70E-74	-0.63941	0.937	0.859	3.40E-71	2
FAM241A	9.26E-74	-0.4179725	0.475	0.57	1.85E-70	2
PCOLCE	1.01E-73	0.52505687	0.854	0.773	2.02E-70	2
NT5DC2	1.06E-73	-0.6672439	0.685	0.658	2.11E-70	2
CPM	1.97E-72	-0.6134892	0.705	0.658	3.93E-69	2
FAM180A	4.18E-72	0.28009926	0.662	0.74	8.37E-69	2
H19	9.13E-71	-0.3415979	0.89	0.723	1.83E-67	2
FMOD	3.61E-69	0.2986188	0.631	0.55	7.21E-66	2
LMO7	8.98E-68	0.40324367	0.574	0.414	1.80E-64	2
CDH11	4.03E-67	0.42983687	0.578	0.468	8.07E-64	2
CRIP2	3.97E-64	-0.5390782	0.918	0.87	7.94E-61	2
PPP1R14A	4.71E-64	0.35087075	0.974	0.903	9.42E-61	2
CCND1	1.06E-63	-0.6743871	0.743	0.712	2.12E-60	2
LMOD1	6.83E-63	-0.4124256	0.938	0.901	1.37E-59	2
NDUFA4L2	1.61E-61	-1.2051968	0.897	0.785	3.22E-58	2
DEPP1	8.50E-61	-0.350161	0.624	0.721	1.70E-57	2
GPM6B	5.34E-58	0.68088179	0.639	0.691	1.07E-54	2
SCUBE3	1.16E-55	0.39388609	0.574	0.38	2.32E-52	2
SRGN	4.60E-54	0.25243474	0.598	0.445	9.20E-51	2
PGAM2	8.44E-51	-0.3397005	0.355	0.432	1.69E-47	2
CDC42EP3	7.91E-48	0.41033965	0.566	0.404	1.58E-44	2
HIGD1B	1.63E-47	-0.5115536	0.641	0.418	3.26E-44	2
RASL11A	6.80E-47	-0.522925	0.654	0.703	1.36E-43	2
HLA-DRA	2.11E-46	-0.2528206	0.937	0.787	4.22E-43	2
THBS1	2.20E-46	-0.4773243	0.716	0.788	4.41E-43	2
RHOBTB3	1.24E-43	-0.2852181	0.642	0.695	2.48E-40	2
LINC02381	1.32E-43	-0.4463265	0.845	0.542	2.65E-40	2
CRYAB	1.01E-40	0.34873603	0.97	0.881	2.03E-37	2
ADAMTS5	1.05E-39	-0.3235447	0.766	0.46	2.09E-36	2
C1QB	2.21E-39	-0.3204973	0.908	0.696	4.42E-36	2
SCG2	5.01E-39	0.27243461	0.398	0.351	1.00E-35	2
POSTN	5.74E-35	0.50597004	0.484	0.67	1.15E-31	2
MRTFB	1.34E-34	-0.3582721	0.803	0.649	2.69E-31	2
SMAP2	8.81E-33	-0.3352045	0.474	0.528	1.76E-29	2
NRGN	3.68E-32	-0.8168519	0.82	0.649	7.35E-29	2
CCN5	5.07E-32	0.50185155	0.566	0.488	1.01E-28	2
IGFBP3	1.54E-30	0.58687277	0.757	0.795	3.09E-27	2
ETS2	5.61E-29	-0.4026409	0.653	0.728	1.12E-25	2
MAFB	5.29E-28	-0.6904261	0.788	0.741	1.06E-24	2
EBF2	6.82E-28	-0.7067931	0.716	0.599	1.36E-24	2
SELENOP	3.24E-27	-0.5180806	0.928	0.855	6.47E-24	2
HES1	1.17E-26	-0.6745738	0.818	0.763	2.34E-23	2
EPAS1	2.12E-26	-0.420131	0.909	0.841	4.24E-23	2
ENPP2	2.93E-26	0.32752606	0.539	0.438	5.87E-23	2
NEDD9	3.31E-26	0.33172801	0.646	0.755	6.63E-23	2
GLUL	3.06E-23	-0.4842647	0.908	0.925	6.12E-20	2
SCARA5	6.64E-23	-0.3872371	0.697	0.607	1.33E-19	2
TNC	7.88E-22	0.3086126	0.435	0.503	1.58E-18	2
RND3	1.06E-19	-0.3530787	0.59	0.619	2.13E-16	2
CFD	1.12E-19	-0.9521312	0.98	0.927	2.24E-16	2
PDK4	1.25E-19	-0.3960057	0.893	0.849	2.50E-16	2
WFDC1	2.80E-19	-0.5757813	0.573	0.508	5.61E-16	2
IFI44L	4.00E-19	-0.2954816	0.483	0.395	8.00E-16	2

ADAP2	4.70E-19	-0.5484346	0.474	0.514	9.39E-16	2
IGFBP4	9.04E-19	-0.4582661	0.825	0.839	1.81E-15	2
IFI27	2.81E-18	-0.4233152	0.862	0.649	5.62E-15	2
PEG10	5.81E-18	-0.2634148	0.715	0.655	1.16E-14	2
PGF	6.80E-18	-0.8190253	0.637	0.559	1.36E-14	2
NPY1R	1.18E-17	-0.3318394	0.826	0.709	2.36E-14	2
RASAL2	4.89E-17	-0.3721874	0.629	0.652	9.78E-14	2
FABP5	3.53E-16	-0.8212689	0.932	0.722	7.05E-13	2
CYFIP2	5.09E-16	0.53300005	0.551	0.539	1.02E-12	2
CKB	5.68E-16	-0.3959061	0.805	0.75	1.14E-12	2
CAMK2N1	2.00E-15	-0.3677506	0.694	0.668	4.01E-12	2
CD36	2.25E-15	-0.5230785	0.961	0.859	4.50E-12	2
OR51E1	2.64E-15	-0.6106088	0.71	0.44	5.27E-12	2
COL4A4	8.00E-15	0.32099089	0.502	0.368	1.60E-11	2
MT1X	1.27E-14	-0.2717558	0.838	0.86	2.54E-11	2
STEAP4	1.36E-14	-0.3295092	0.749	0.793	2.71E-11	2
PRXL2A	2.27E-14	-0.4014888	0.77	0.763	4.55E-11	2
SUSD5	6.60E-14	-0.2661506	0.574	0.612	1.32E-10	2
PTP4A3	3.59E-13	-0.8296243	0.494	0.479	7.18E-10	2
TPH1	6.68E-13	0.26790089	0.491	0.313	1.34E-09	2
PDLIM1	7.31E-13	-0.3777423	0.808	0.757	1.46E-09	2
IGLC2	1.25E-12	-0.2541289	0.402	0.308	2.50E-09	2
PDGFB	3.24E-12	-0.3151069	0.297	0.347	6.49E-09	2
ANGPT1	8.87E-12	-0.383011	0.672	0.631	1.77E-08	2
HIST1H4C	1.66E-11	-0.3386214	0.812	0.766	3.33E-08	2
SUSD2	2.00E-11	-0.5557965	0.539	0.505	4.00E-08	2
FNBP1L	9.23E-11	-0.3409562	0.901	0.806	1.85E-07	2
RASD1	4.34E-10	-0.3552394	0.468	0.501	8.68E-07	2
DIO2	5.42E-10	0.32616992	0.59	0.568	1.08E-06	2
CCL19	1.10E-09	-0.4761317	0.718	0.565	2.21E-06	2
ACKR3	1.19E-09	-0.2952143	0.317	0.329	2.38E-06	2
MRVI1	2.90E-09	-0.2898281	0.799	0.764	5.81E-06	2
CYTL1	1.15E-08	0.3685374	0.495	0.434	2.30E-05	2
SNHG7	2.28E-08	-0.2870255	0.734	0.756	4.56E-05	2
DPT	4.45E-08	0.40712986	0.519	0.392	8.91E-05	2
CTSB	7.03E-08	-0.3481784	0.652	0.609	0.00014053	2
OSR2	1.05E-07	0.30676502	0.528	0.502	0.00020993	2
CP	2.03E-07	0.37992021	0.462	0.419	0.00040562	2
C1QA	6.71E-07	-0.3656425	0.879	0.667	0.00134119	2
NRARP	7.63E-07	-0.5259634	0.603	0.513	0.00152541	2
SFRP4	9.16E-07	0.41593455	0.457	0.452	0.00183256	2
ITGA5	5.81E-06	0.274304	0.577	0.633	0.01162021	2
NES	9.00E-06	-0.2753158	0.709	0.597	0.01800472	2
SLC2A3	1.06E-05	-0.2918442	0.606	0.511	0.02121355	2
ID1	2.16E-05	-0.4090333	0.755	0.761	0.04327354	2
SUGCT	2.24E-05	0.26403913	0.443	0.32	0.04472234	2
GPX3	2.34E-05	-0.5990422	0.861	0.868	0.04679287	2
DES	3.60E-05	1.183363	0.563	0.651	0.07193754	2
IL13RA2	4.00E-05	0.35977784	0.462	0.248	0.07993906	2
SERPINF1	6.96E-05	-0.3145539	0.932	0.828	0.13910843	2
TBX3	8.92E-05	-0.4621582	0.611	0.489	0.17839365	2
IGHM	0.00018279	-0.2869695	0.314	0.319	0.36557673	2
ESAM	0.0005417	-0.4631088	0.761	0.667	1	2

ANK2	0.00103276	-0.3303268	0.565	0.54	1	2
ATP1B1	0.00124583	0.33201623	0.489	0.441	1	2
IGKC	0.00129335	-0.3620001	0.253	0.312	1	2
GUCY1A1	0.00531061	-0.3042675	0.943	0.781	1	2
CCL19	0	0.89626003	0.815	0.576	0	3
BTNL9	0	0.60301162	0.847	0.894	0	3
RAMP3	0	0.42214698	0.836	0.817	0	3
CCL14	0	0.40163684	0.841	0.872	0	3
ECSCR	0	0.36890932	0.824	0.752	0	3
HLA-DQA2	0	0.25939191	0.806	0.744	0	3
TMEM47	0	-0.2586692	0.353	0.78	0	3
PEG10	0	-0.3432342	0.281	0.737	0	3
GADD45G	0	-0.4467259	0.801	0.902	0	3
FBXO32	0	-0.508386	0.344	0.827	0	3
CAMK2N1	0	-0.5313272	0.217	0.751	0	3
OMD	0	-0.537616	0.223	0.65	0	3
COX7A1	0	-0.5492124	0.262	0.834	0	3
GUCY1A1	0	-0.5505256	0.499	0.883	0	3
BCAM	0	-0.6536874	0.81	0.969	0	3
KCNMB1	0	-0.6577786	0.278	0.698	0	3
CD9	0	-0.660493	0.367	0.898	0	3
HIST1H4C	0	-0.6776162	0.494	0.827	0	3
NET1	0	-0.7244887	0.343	0.866	0	3
HES4	0	-0.7281518	0.789	0.916	0	3
CALD1	0	-0.7369335	0.913	0.999	0	3
CRIP2	0	-0.7923022	0.569	0.936	0	3
RCAN2	0	-0.8698901	0.401	0.883	0	3
TBX2-AS1	0	-0.8974846	0.259	0.712	0	3
CAV1	0	-0.9374216	0.742	0.991	0	3
PHLDA2	0	-0.9672177	0.34	0.756	0	3
NEXN	0	-0.9721629	0.66	0.973	0	3
C12orf75	0	-0.9909564	0.388	0.864	0	3
ACTG2	0	-1.0038056	0.387	0.848	0	3
LBH	0	-1.0390269	0.368	0.847	0	3
BGN	0	-1.1527375	0.722	0.988	0	3
NDUFA4L2	0	-1.1798472	0.624	0.85	0	3
CRYAB	0	-1.2260285	0.531	0.969	0	3
CSRP2	0	-1.2770306	0.529	0.9	0	3
SNCG	0	-1.3380169	0.755	0.892	0	3
PPP1R14A	0	-1.5556869	0.611	0.976	0	3
PLN	0	-1.5896805	0.584	0.962	0	3
TAGLN	0	-1.6438376	0.974	1	0	3
MYL9	0	-1.6658902	0.911	0.999	0	3
ACTA2	0	-1.6783648	0.899	0.994	0	3
CRIP1	0	-1.7184203	0.831	0.973	0	3
DSTN	0	-1.7351142	0.86	0.998	0	3
TPM2	0	-1.7994481	0.885	0.998	0	3
RERGL	0	-2.1111419	0.827	0.959	0	3
TUBA1C	1.10E-307	-0.7788298	0.634	0.835	2.19E-304	3
CCND1	9.05E-306	-0.4891862	0.347	0.783	1.81E-302	3
TPM1	1.30E-299	-0.7780131	0.859	0.996	2.59E-296	3
NTRK2	1.13E-298	-0.7527737	0.785	0.932	2.26E-295	3
EMP1	3.61E-297	0.34252138	0.289	0.718	7.21E-294	3

ITGA6	1.95E-296	0.32993126	0.815	0.739	3.90E-293	3
HSPB6	6.36E-296	-0.3352361	0.251	0.77	1.27E-292	3
SERPINE1	1.46E-294	-0.3452332	0.373	0.801	2.93E-291	3
RAMP1	3.92E-294	-0.533536	0.34	0.786	7.83E-291	3
HSPH1	3.37E-283	0.30274579	0.238	0.708	6.74E-280	3
LMOD1	3.70E-277	-0.7498232	0.609	0.962	7.40E-274	3
LGALS3	3.50E-276	-0.3533092	0.832	0.96	7.00E-273	3
OSR1	4.66E-271	-0.2586893	0.256	0.651	9.33E-268	3
NUAK1	5.11E-268	0.64349044	0.742	0.433	1.02E-264	3
TTN	6.09E-267	0.4383315	0.677	0.447	1.22E-263	3
RAMP2	7.47E-261	0.25417436	0.492	0.825	1.49E-257	3
ID3	1.47E-260	-0.6828126	0.496	0.855	2.94E-257	3
PCOLCE	8.83E-258	-0.2711608	0.578	0.833	1.77E-254	3
F13A1	2.02E-252	0.31070627	0.807	0.816	4.04E-249	3
NRGN	1.09E-251	-0.6024403	0.388	0.751	2.18E-248	3
TMEM88	5.83E-250	0.36538782	0.776	0.83	1.17E-246	3
CD36	2.33E-244	1.24498148	0.859	0.894	4.67E-241	3
HLA-DMA	2.00E-243	0.56819428	0.664	0.413	4.01E-240	3
GRASP	2.69E-242	0.30362505	0.789	0.705	5.38E-239	3
ASPN	2.82E-242	-0.5600597	0.365	0.755	5.64E-239	3
MT1E	4.72E-241	-0.4229167	0.565	0.966	9.45E-238	3
C11orf96	1.40E-231	-0.7417216	0.779	0.973	2.80E-228	3
IGLC3	5.31E-231	0.47108377	0.604	0.272	1.06E-227	3
FILIP1L	8.00E-231	-0.6416479	0.756	0.969	1.60E-227	3
PDLIM1	1.11E-225	-0.3223382	0.551	0.809	2.22E-222	3
OGN	1.28E-224	-1.5047092	0.438	0.805	2.55E-221	3
LINC00632	4.28E-223	-0.2954659	0.279	0.659	8.56E-220	3
HSP90B1	3.50E-221	-0.6194851	0.484	0.881	6.99E-218	3
C1QC	3.32E-219	0.40530993	0.759	0.552	6.63E-216	3
CSRP1	2.28E-218	-0.5494629	0.577	0.938	4.55E-215	3
IGLC2	2.59E-217	0.55909015	0.589	0.294	5.18E-214	3
RBPMS2	3.50E-216	-0.5124366	0.496	0.776	7.00E-213	3
KCNAB1	3.52E-216	-0.5127157	0.596	0.825	7.03E-213	3
COMP	1.66E-214	0.33178199	0.179	0.505	3.32E-211	3
FHL1	6.23E-213	-0.4603171	0.561	0.941	1.25E-209	3
IGHM	9.53E-210	0.52011842	0.556	0.278	1.91E-206	3
CNN1	2.61E-204	-0.7272456	0.594	0.87	5.22E-201	3
ISYNA1	3.18E-204	-0.3216969	0.451	0.755	6.36E-201	3
PALLD	1.99E-203	-0.3277011	0.661	0.973	3.97E-200	3
C1QB	4.04E-201	0.93318898	0.823	0.748	8.09E-198	3
FRZB	1.09E-200	-0.3848281	0.218	0.635	2.18E-197	3
RBP7	4.35E-200	0.4770373	0.84	0.896	8.69E-197	3
TINAGL1	3.53E-198	-0.2833968	0.531	0.904	7.06E-195	3
SORBS2	7.24E-197	-0.5388939	0.797	0.935	1.45E-193	3
MYH11	9.94E-196	-0.5789946	0.844	0.981	1.99E-192	3
S100A8	1.73E-194	0.45722541	0.778	0.704	3.45E-191	3
CYTOR	7.74E-191	0.29888121	0.285	0.627	1.55E-187	3
LTBP1	2.19E-190	-0.5774375	0.527	0.881	4.38E-187	3
ABCA6	1.42E-189	0.27284129	0.787	0.664	2.85E-186	3
NT5DC2	1.93E-188	-0.4447442	0.371	0.715	3.86E-185	3
ITIH5	5.87E-187	-0.4825645	0.735	0.832	1.17E-183	3
MT1L	4.22E-185	-0.4632787	0.676	0.789	8.45E-182	3
ID4	5.63E-184	0.26545806	0.408	0.826	1.13E-180	3

EMCN	2.39E-183	0.2532076	0.81	0.775	4.78E-180	3
PALMD	1.34E-181	0.46922995	0.783	0.787	2.67E-178	3
EGFL7	2.33E-181	0.77565653	0.844	0.874	4.67E-178	3
CTSZ	1.35E-180	-0.2833688	0.702	0.821	2.70E-177	3
DOCK9	2.75E-180	0.27772854	0.699	0.526	5.50E-177	3
CDH19	2.30E-179	0.2832898	0.187	0.499	4.60E-176	3
CD14	3.25E-179	0.36906372	0.697	0.533	6.49E-176	3
MAST4	1.32E-175	0.2624237	0.357	0.713	2.65E-172	3
ELK3	5.71E-175	0.33255181	0.747	0.587	1.14E-171	3
CKB	2.69E-174	-0.3434819	0.539	0.804	5.38E-171	3
MAP3K7CL	3.83E-174	-0.4799021	0.574	0.739	7.66E-171	3
MS4A6A	1.23E-173	0.33519631	0.706	0.551	2.46E-170	3
SPARC	4.13E-172	-0.5004235	0.626	0.951	8.27E-169	3
ACTN1	8.81E-171	-0.2696152	0.576	0.919	1.76E-167	3
RGS5	5.96E-170	-0.9716403	0.502	0.802	1.19E-166	3
MPZ	4.44E-169	0.28810083	0.745	0.784	8.87E-166	3
CLU	1.32E-165	-0.4457821	0.657	0.969	2.64E-162	3
NCKAP5	4.05E-163	0.2955757	0.668	0.496	8.10E-160	3
CXCL12	2.31E-160	0.32595419	0.6	0.827	4.62E-157	3
LPL	3.01E-153	0.37123988	0.689	0.59	6.03E-150	3
POSTN	1.84E-152	0.36640593	0.33	0.663	3.67E-149	3
GMFG	2.98E-152	0.34994982	0.777	0.735	5.95E-149	3
GPAM	2.27E-151	0.25595081	0.664	0.466	4.55E-148	3
PCSK5	5.43E-151	0.25248787	0.685	0.52	1.09E-147	3
FHL5	9.88E-151	-0.2791591	0.723	0.803	1.98E-147	3
TSHZ2	3.49E-149	0.52141212	0.805	0.789	6.98E-146	3
CD93	2.37E-144	0.39294086	0.697	0.498	4.75E-141	3
EDNRA	1.71E-143	-0.3561623	0.66	0.765	3.43E-140	3
GGT5	3.46E-141	0.5460225	0.367	0.689	6.91E-138	3
RRAD	3.78E-141	-0.4538409	0.741	0.803	7.55E-138	3
SERPINF1	6.38E-139	-0.3012858	0.786	0.87	1.28E-135	3
MYLK	1.77E-137	-0.3044827	0.561	0.922	3.54E-134	3
CNR1	7.59E-137	0.45707669	0.838	0.811	1.52E-133	3
CCN1	3.03E-134	-0.2878827	0.609	0.936	6.06E-131	3
ITGA10	7.28E-134	0.43388872	0.338	0.674	1.46E-130	3
RPGR	2.94E-133	0.28328307	0.778	0.684	5.87E-130	3
CCL2	8.12E-133	0.34625243	0.309	0.638	1.62E-129	3
ADGRF5	1.28E-132	0.78447739	0.791	0.696	2.55E-129	3
KCNE4	3.79E-132	-0.314719	0.248	0.571	7.58E-129	3
EFEMP1	5.33E-132	-0.3813747	0.445	0.795	1.07E-128	3
NPY1R	2.33E-131	-0.2769625	0.676	0.755	4.65E-128	3
ADAMTS1	5.98E-131	0.46812438	0.575	0.869	1.20E-127	3
IGFBP4	1.29E-129	0.32534862	0.582	0.877	2.58E-126	3
CALCRL	1.40E-129	0.26812486	0.815	0.751	2.80E-126	3
HLA-DRB5	1.54E-127	0.36482338	0.74	0.715	3.09E-124	3
FLNB	1.65E-127	0.73693919	0.718	0.582	3.31E-124	3
FBLN5	4.39E-126	-0.2879514	0.402	0.656	8.78E-123	3
CRLF1	6.76E-126	0.28516155	0.327	0.589	1.35E-122	3
THBS2	2.89E-122	0.3311883	0.313	0.616	5.77E-119	3
DEPP1	8.57E-121	0.33982045	0.432	0.736	1.71E-117	3
C1S	7.05E-119	0.32397673	0.453	0.834	1.41E-115	3
PTN	7.63E-119	-0.6024777	0.634	0.688	1.53E-115	3
ADAMTS6	5.40E-117	0.25490887	0.169	0.388	1.08E-113	3

SSTR2	2.39E-116	0.29213579	0.777	0.667	4.79E-113	3
PGF	8.10E-116	-0.4561288	0.364	0.618	1.62E-112	3
RARRES1	1.15E-114	-0.2670495	0.6	0.814	2.31E-111	3
CCDC3	1.39E-114	-0.483006	0.625	0.707	2.78E-111	3
HLA-DPA1	3.50E-114	0.88518227	0.803	0.77	6.99E-111	3
AIF1	1.62E-113	0.30264664	0.372	0.617	3.23E-110	3
CRIM1	7.94E-113	0.25770585	0.519	0.787	1.59E-109	3
SNED1	2.20E-112	0.38787008	0.675	0.485	4.39E-109	3
ENTPD3	2.32E-112	-0.4997809	0.67	0.674	4.64E-109	3
DNAJA4	5.70E-103	0.29995581	0.637	0.51	1.14E-99	3
GPX3	7.09E-102	0.39360569	0.659	0.9	1.42E-98	3
SRPX	1.35E-101	0.33661033	0.788	0.68	2.70E-98	3
ISG15	1.01E-99	-0.2611463	0.458	0.661	2.02E-96	3
RAPGEF3	3.67E-98	0.35627765	0.808	0.826	7.33E-95	3
PODN	2.86E-95	0.29169475	0.798	0.68	5.73E-92	3
CASQ2	5.96E-95	-0.4120658	0.356	0.591	1.19E-91	3
TGM2	1.05E-93	0.28315882	0.523	0.835	2.09E-90	3
THBS1	5.07E-93	0.47870798	0.585	0.797	1.01E-89	3
CCN2	8.38E-93	-0.720938	0.625	0.881	1.68E-89	3
MEGF6	2.04E-92	0.30624998	0.384	0.621	4.07E-89	3
KRT14	2.03E-90	-0.3800214	0.502	0.701	4.06E-87	3
ITGA5	4.43E-90	0.56571172	0.326	0.664	8.87E-87	3
PTP4A3	5.99E-90	-0.3100479	0.205	0.529	1.20E-86	3
NPC2	4.35E-89	0.2736428	0.614	0.765	8.70E-86	3
DAB2	1.15E-88	0.53928112	0.705	0.56	2.31E-85	3
PCAT19	4.10E-88	0.34241596	0.796	0.855	8.20E-85	3
PIM3	8.69E-88	0.33329142	0.812	0.662	1.74E-84	3
SORBS1	1.29E-85	0.28327155	0.42	0.721	2.57E-82	3
ABI3BP	5.52E-85	0.41741081	0.796	0.643	1.10E-81	3
NRP2	7.33E-85	0.27935137	0.417	0.675	1.47E-81	3
MRC1	5.20E-82	0.31146128	0.718	0.738	1.04E-78	3
VCAN	1.28E-80	-0.6195686	0.523	0.812	2.56E-77	3
TNXB	6.57E-79	0.35135106	0.769	0.719	1.31E-75	3
S1PR3	4.29E-78	0.37538393	0.292	0.585	8.57E-75	3
IFI27	3.34E-77	1.10288073	0.859	0.687	6.69E-74	3
CAVIN2	8.79E-76	0.33586782	0.707	0.571	1.76E-72	3
IGHG4	3.43E-74	0.31244904	0.118	0.327	6.86E-71	3
LMCD1	1.12E-73	0.58140499	0.43	0.727	2.24E-70	3
COX4I2	1.28E-73	-0.2747993	0.676	0.723	2.56E-70	3
MGST1	1.78E-70	0.42634223	0.223	0.47	3.55E-67	3
KCNQ1OT1	1.95E-70	0.56523312	0.238	0.51	3.89E-67	3
MRVI1	4.19E-70	0.39307076	0.538	0.813	8.37E-67	3
ENG	4.45E-69	0.50453694	0.47	0.677	8.90E-66	3
PLVAP	1.58E-68	0.38569227	0.622	0.77	3.16E-65	3
TCF15	4.47E-67	-0.5860839	0.736	0.804	8.94E-64	3
TIMP1	2.16E-66	-0.8803165	0.727	0.948	4.33E-63	3
EHD4	1.21E-65	0.3341617	0.659	0.498	2.42E-62	3
APOE	1.40E-65	-0.4213311	0.493	0.729	2.79E-62	3
PHACTR1	1.72E-64	0.2570388	0.317	0.547	3.44E-61	3
CTSS	2.75E-64	0.37054741	0.514	0.389	5.50E-61	3
IGKC	4.07E-64	0.71148303	0.419	0.274	8.14E-61	3
FCER1G	5.29E-64	0.3922291	0.332	0.556	1.06E-60	3
ID1	1.21E-63	0.30924007	0.666	0.775	2.41E-60	3

WFDC1	5.86E-63	-0.2605891	0.257	0.571	1.17E-59	3
COL12A1	6.72E-61	0.47686895	0.746	0.559	1.34E-57	3
ACKR1	2.79E-60	1.02906423	0.879	0.928	5.58E-57	3
COL1A2	1.03E-59	-0.3702203	0.607	0.875	2.05E-56	3
TFPI2	1.39E-55	-0.2538147	0.323	0.551	2.77E-52	3
S100A9	1.52E-55	0.71514501	0.763	0.777	3.04E-52	3
HLA-B	3.57E-54	0.47315225	0.873	0.919	7.15E-51	3
SAT1	4.85E-53	0.57571905	0.473	0.764	9.71E-50	3
SYNPO2	8.80E-52	0.36706345	0.508	0.877	1.76E-48	3
ANK3	3.71E-51	0.27568224	0.629	0.499	7.42E-48	3
SLC9A3R2	7.07E-51	0.56432529	0.807	0.681	1.41E-47	3
SMTN	1.75E-50	0.45125083	0.497	0.717	3.51E-47	3
CCDC102B	2.25E-50	0.28911656	0.313	0.479	4.49E-47	3
SOD2	7.23E-49	0.53562463	0.604	0.713	1.45E-45	3
SLPI	9.39E-49	0.28155059	0.552	0.461	1.88E-45	3
MGP	9.60E-48	-1.4581991	0.945	0.997	1.92E-44	3
GJA4	9.20E-47	-0.4343727	0.74	0.692	1.84E-43	3
ADAM15	7.24E-46	0.28830956	0.786	0.726	1.45E-42	3
TM4SF1	1.43E-45	0.42270403	0.662	0.764	2.87E-42	3
CLDN5	3.70E-44	0.48007989	0.848	0.899	7.41E-41	3
PCDH7	5.45E-44	0.49385153	0.357	0.523	1.09E-40	3
CTSC	1.19E-43	0.4776863	0.782	0.64	2.38E-40	3
PCDH9	1.48E-43	0.56497167	0.587	0.468	2.96E-40	3
ITGA8	1.67E-43	0.28348893	0.565	0.794	3.34E-40	3
COL6A3	1.85E-43	0.48547459	0.566	0.721	3.70E-40	3
PPP1R12B	8.96E-43	0.35891092	0.571	0.873	1.79E-39	3
SOX4	1.51E-42	0.33194618	0.573	0.676	3.03E-39	3
SYNE2	4.88E-42	0.40500046	0.514	0.8	9.76E-39	3
FABP4	2.02E-40	0.80305748	0.906	0.975	4.05E-37	3
IGHG3	1.04E-39	0.27525719	0.399	0.26	2.08E-36	3
LYVE1	3.00E-37	0.31502318	0.714	0.708	6.00E-34	3
CCL21	3.69E-37	0.76210172	0.723	0.692	7.38E-34	3
RGS2	5.48E-37	0.25221159	0.366	0.584	1.10E-33	3
HLA-DRA	1.51E-36	1.19214362	0.857	0.827	3.02E-33	3
GRIA2	2.07E-36	0.52010584	0.263	0.367	4.14E-33	3
KLHL23	2.57E-34	-0.3754071	0.733	0.735	5.14E-31	3
SLC41A1	5.67E-34	0.26081489	0.298	0.502	1.13E-30	3
TMEM176A	9.43E-33	0.3501781	0.655	0.622	1.89E-29	3
ZNF385D	1.24E-32	0.33518967	0.409	0.614	2.48E-29	3
SLC2A3	1.58E-31	0.47713373	0.434	0.556	3.15E-28	3
ABCC9	2.37E-31	0.4932434	0.798	0.703	4.73E-28	3
HMCN2	8.62E-29	0.33444745	0.611	0.647	1.72E-25	3
FBLN1	8.00E-28	-0.3038606	0.871	0.947	1.60E-24	3
CPM	3.34E-27	0.25412854	0.751	0.659	6.67E-24	3
HLA-DRB1	8.07E-27	0.68955965	0.648	0.811	1.61E-23	3
CCN5	1.38E-24	0.64038557	0.374	0.533	2.75E-21	3
RNASE1	2.22E-23	0.83894908	0.767	0.717	4.43E-20	3
CTSB	2.93E-23	0.38184022	0.553	0.632	5.86E-20	3
PLIN2	1.46E-22	0.29941325	0.537	0.603	2.93E-19	3
COL25A1	3.36E-22	0.37894598	0.787	0.753	6.72E-19	3
GUCY1A2	1.37E-21	0.36882906	0.701	0.627	2.74E-18	3
OR51E1	1.52E-21	-0.3480396	0.342	0.549	3.04E-18	3
FABP5	5.83E-21	0.73702388	0.788	0.783	1.17E-17	3

FMOD	8.72E-21	0.40652515	0.472	0.591	1.74E-17	3
COL1A1	1.10E-20	0.31932636	0.649	0.798	2.20E-17	3
CP	1.56E-20	0.2856965	0.496	0.421	3.12E-17	3
C1QA	1.59E-20	0.82747665	0.757	0.724	3.18E-17	3
C1R	8.35E-20	0.25040494	0.817	0.917	1.67E-16	3
SCD	3.51E-18	0.36333629	0.593	0.495	7.03E-15	3
CRISPLD2	4.54E-18	0.4877555	0.687	0.56	9.08E-15	3
TYROBP	2.12E-17	0.32226569	0.381	0.536	4.24E-14	3
COL4A1	2.34E-17	0.55242516	0.553	0.798	4.69E-14	3
GPRC5A	2.21E-15	0.41259588	0.543	0.648	4.42E-12	3
IGFBP3	2.73E-15	0.27604153	0.75	0.789	5.45E-12	3
TYMP	3.48E-15	0.3223744	0.71	0.655	6.96E-12	3
KCTD12	4.44E-15	0.38999529	0.765	0.683	8.88E-12	3
CCDC71L	6.22E-15	0.2688224	0.81	0.795	1.24E-11	3
TGFBR3	1.39E-14	0.30324534	0.802	0.771	2.78E-11	3
CDC42EP3	2.80E-14	0.29172041	0.349	0.469	5.59E-11	3
ETS1	3.84E-14	0.32175042	0.654	0.532	7.69E-11	3
LYST	5.62E-14	0.31001296	0.582	0.683	1.12E-10	3
ITGA7	6.78E-14	0.65350778	0.7	0.755	1.36E-10	3
PRPH	3.21E-13	-0.625534	0.686	0.683	6.43E-10	3
NR4A2	7.83E-13	0.38144188	0.615	0.618	1.57E-09	3
PPP1R1A	8.09E-13	-0.2596204	0.402	0.492	1.62E-09	3
S100B	1.29E-12	0.30795877	0.516	0.709	2.57E-09	3
RHOBTB3	1.81E-12	0.25630773	0.643	0.685	3.61E-09	3
RASAL2	3.47E-12	0.2802402	0.626	0.648	6.94E-09	3
STEAP4	3.63E-12	1.09063857	0.743	0.786	7.26E-09	3
PTGDS	7.68E-12	0.584435	0.777	0.821	1.54E-08	3
CNKSR3	6.42E-11	0.34638453	0.544	0.561	1.28E-07	3
PECAM1	2.64E-10	0.64130928	0.815	0.88	5.28E-07	3
TMTC1	5.38E-09	0.3219073	0.792	0.746	1.08E-05	3
A2M	6.90E-09	0.36919936	0.886	0.986	1.38E-05	3
AKR1C3	3.45E-08	0.26650895	0.462	0.578	6.89E-05	3
SELENOP	3.60E-08	0.33532418	0.813	0.887	7.19E-05	3
CFD	4.45E-08	0.67664474	0.895	0.951	8.89E-05	3
LIFR	7.80E-08	0.63955145	0.846	0.873	0.00015593	3
TFPI	4.17E-07	0.51588799	0.694	0.624	0.00083375	3
STAB1	7.55E-07	0.43837509	0.58	0.545	0.00151032	3
FTL	3.53E-06	0.3501854	0.967	1	0.0070669	3
CD74	9.62E-06	1.01480028	0.725	0.79	0.01924226	3
LDLR	8.00E-05	0.46215682	0.617	0.68	0.16000356	3
USP53	0.00011979	0.64056561	0.711	0.82	0.23958859	3
ARL4A	0.00015181	0.25528406	0.6	0.597	0.30361984	3
THY1	0.00019457	0.30159938	0.636	0.618	0.38914933	3
SRGN	0.00028391	0.36440454	0.442	0.497	0.56781215	3
ADAMTS4	0.0008598	0.57886956	0.495	0.517	1	3
APOD	0.00144872	0.87224573	0.684	0.908	1	3
H19	0.00145619	0.56517033	0.687	0.786	1	3
ADAMTS9	0.00246247	0.42579517	0.502	0.556	1	3
LAMB1	0.00260162	0.30592435	0.679	0.785	1	3
UCP1	0.00290397	0.25107793	0.566	0.482	1	3
CLSTN2	0.00387442	0.29706784	0.661	0.767	1	3
THBD	0.00547197	0.3535472	0.224	0.299	1	3
CCL3	0.00601816	0.34110862	0.31	0.312	1	3

LAPTM5	0.00677018	0.26978087	0.49	0.511	1	3
CARMN	0.00848638	1.08321085	0.656	0.835	1	3
PLA2G2A	0	4.18601131	0.974	0.922	0	4
DCN	0	3.63958908	1	0.918	0	4
CFD	0	3.50442698	0.992	0.94	0	4
C3	0	2.88456864	0.993	0.763	0	4
CXCL14	0	2.82921035	0.897	0.55	0	4
IGF1	0	2.68053767	0.923	0.586	0	4
IGFBP6	0	2.56459617	0.995	0.924	0	4
FBLN1	0	2.46976887	1	0.932	0	4
SFRP2	0	2.44554987	0.901	0.553	0	4
SCARA5	0	2.42943351	0.946	0.614	0	4
SERPINF1	0	2.33436092	0.996	0.849	0	4
MMP2	0	2.23054145	0.978	0.756	0	4
MFAP5	0	2.21330979	0.885	0.538	0	4
ABCA8	0	2.13615031	0.97	0.759	0	4
ADH1B	0	2.09305167	0.993	0.825	0	4
C1R	0	2.08795337	0.999	0.897	0	4
C1S	0	2.08384162	0.995	0.766	0	4
PODN	0	2.0028704	0.954	0.681	0	4
TSHZ2	0	1.98301453	0.922	0.783	0	4
CCDC80	0	1.95069376	0.988	0.761	0	4
LUM	0	1.89635191	0.979	0.712	0	4
ABI3BP	0	1.84502246	0.957	0.646	0	4
GPX3	0	1.80097869	0.996	0.858	0	4
PLTP	0	1.63468349	0.972	0.842	0	4
CPB1	0	1.08311667	0.853	0.636	0	4
TNFSF13B	6.04E-299	0.69182965	0.855	0.646	1.21E-295	4
FTL	1.18E-297	1.06179307	1	0.995	2.37E-294	4
HTRA3	1.64E-295	1.63097762	0.927	0.782	3.28E-292	4
RARRES1	2.70E-292	2.41830511	0.911	0.775	5.40E-289	4
PDGFRL	5.03E-291	1.43138962	0.89	0.668	1.01E-287	4
C16orf89	1.56E-286	0.79857448	0.858	0.693	3.13E-283	4
CADM3	8.31E-282	1.02667782	0.874	0.712	1.66E-278	4
IGFBP5	2.38E-279	1.77440423	0.999	0.959	4.77E-276	4
PMP22	6.97E-276	1.29690781	0.987	0.791	1.39E-272	4
SLIT2	2.87E-275	1.27414522	0.861	0.548	5.73E-272	4
FBN1	2.75E-271	1.63864702	0.948	0.719	5.50E-268	4
ABCA6	1.82E-270	1.35978402	0.879	0.669	3.65E-267	4
TGFBR3	4.64E-270	1.47163946	0.933	0.766	9.28E-267	4
C7	1.87E-269	2.38543135	0.939	0.769	3.74E-266	4
BICC1	1.89E-266	1.08214963	0.875	0.677	3.78E-263	4
CA12	1.01E-265	0.40729084	0.752	0.352	2.02E-262	4
LINC01697	7.45E-262	0.74357917	0.833	0.668	1.49E-258	4
COL6A3	1.75E-261	1.41119359	0.915	0.686	3.50E-258	4
IGFBP4	3.24E-260	1.43434633	0.984	0.825	6.47E-257	4
GAS1	2.74E-257	1.6473863	0.865	0.63	5.47E-254	4
CYP1B1	2.36E-256	0.85477558	0.814	0.451	4.72E-253	4
FBLN2	1.04E-249	1.35507192	0.879	0.64	2.08E-246	4
ABLIM1	8.73E-249	1.16545448	0.917	0.848	1.75E-245	4
OLFML3	5.84E-247	1.124336	0.875	0.432	1.17E-243	4
PI16	8.55E-246	1.75429358	0.862	0.706	1.71E-242	4
CRISPLD2	4.02E-242	1.19156251	0.919	0.557	8.04E-239	4

NNMT	3.54E-239	1.22882327	0.982	0.823	7.08E-236	4
UCHL1	9.57E-239	0.46071007	0.837	0.516	1.91E-235	4
MEG3	1.72E-236	1.05080994	0.912	0.552	3.43E-233	4
TNFSF10	1.95E-234	0.86620537	0.831	0.565	3.91E-231	4
GLUL	8.44E-234	1.18408618	0.99	0.916	1.69E-230	4
BMP4	1.83E-233	0.64229333	0.82	0.492	3.66E-230	4
COL1A2	3.35E-233	0.96999623	0.998	0.827	6.71E-230	4
MGST1	2.26E-232	1.18176245	0.794	0.412	4.53E-229	4
CTSK	2.58E-231	1.01622996	0.904	0.719	5.16E-228	4
USP53	7.86E-228	0.91456599	0.958	0.794	1.57E-224	4
FMO2	7.98E-224	1.28814454	0.939	0.748	1.60E-220	4
CYP4B1	1.26E-223	0.39686865	0.773	0.583	2.51E-220	4
GAS7	2.44E-223	0.86114916	0.794	0.497	4.88E-220	4
COL1A1	2.56E-222	1.04121598	0.988	0.763	5.11E-219	4
ITGBL1	5.98E-221	0.85104395	0.947	0.702	1.20E-217	4
CFH	4.46E-220	0.85374116	0.968	0.728	8.93E-217	4
PDGFRA	8.27E-219	1.23408996	0.828	0.461	1.65E-215	4
HSP90B1	9.53E-215	1.19398876	0.993	0.814	1.91E-211	4
RDH10	2.20E-211	0.66535613	0.831	0.661	4.39E-208	4
RAMP2	2.08E-210	1.12829923	0.855	0.773	4.17E-207	4
MT1X	1.67E-208	1.43972063	0.99	0.844	3.34E-205	4
MYOC	1.12E-206	1.33044972	0.812	0.658	2.23E-203	4
MSX1	2.13E-206	0.72349492	0.748	0.348	4.27E-203	4
PCDH17	5.97E-206	0.54789195	0.796	0.62	1.19E-202	4
EMP1	1.36E-205	0.962538	0.86	0.644	2.71E-202	4
MT1A	2.81E-204	0.83394058	0.93	0.743	5.62E-201	4
PCOLCE	1.39E-203	1.00066667	0.956	0.786	2.79E-200	4
TIMP1	1.75E-203	0.5416746	1	0.912	3.51E-200	4
NEGR1	2.38E-203	0.89221474	0.771	0.433	4.76E-200	4
UAP1	7.58E-203	0.82534402	0.805	0.417	1.52E-199	4
SEMA3C	7.96E-202	1.24525757	0.786	0.514	1.59E-198	4
PROCR	1.51E-201	0.98781654	0.882	0.721	3.02E-198	4
AKR1C1	1.13E-196	0.56611271	0.785	0.47	2.26E-193	4
CD34	5.35E-196	1.22084136	0.851	0.706	1.07E-192	4
PDK4	6.01E-195	0.86637738	0.956	0.856	1.20E-191	4
IGSF10	1.61E-194	1.29522297	0.807	0.678	3.22E-191	4
MGP	7.33E-194	0.45820107	1	0.989	1.47E-190	4
TMEM176A	4.92E-192	0.81929175	0.803	0.616	9.84E-189	4
SRPX	5.46E-192	1.36822529	0.828	0.687	1.09E-188	4
THY1	4.46E-187	0.90613991	0.794	0.609	8.92E-184	4
EFEMP1	1.09E-184	0.85632982	0.964	0.731	2.19E-181	4
ABCC9	1.60E-184	1.01767828	0.882	0.706	3.20E-181	4
HP	3.88E-184	0.35467947	0.727	0.495	7.75E-181	4
MT1G	2.49E-182	0.49305019	0.828	0.533	4.98E-179	4
RSPO3	3.25E-182	1.38174154	0.801	0.726	6.50E-179	4
GPNMB	1.05E-178	1.05544618	0.916	0.669	2.10E-175	4
FSTL1	3.23E-178	0.87084803	0.954	0.63	6.45E-175	4
LOX	7.61E-178	0.98010743	0.789	0.504	1.52E-174	4
HELLPAR	7.23E-177	1.04088092	0.754	0.525	1.45E-173	4
AKAP12	9.37E-174	0.95208201	0.96	0.741	1.87E-170	4
FOXD1	1.01E-170	0.47711314	0.854	0.843	2.02E-167	4
OGN	4.64E-167	0.61119225	0.98	0.739	9.27E-164	4
MT1M	4.19E-166	1.26154272	0.988	0.889	8.38E-163	4

MYH11	7.11E-164	-1.4429345	0.94	0.963	1.42E-160	4
ACTA2	3.47E-163	-1.3038614	0.994	0.98	6.94E-160	4
TMEM176B	2.56E-161	0.7556206	0.814	0.741	5.12E-158	4
CNTFR	8.91E-159	0.62914262	0.77	0.727	1.78E-155	4
SPRY1	2.13E-158	0.95191852	0.88	0.607	4.27E-155	4
FLNA	2.20E-157	-1.2524976	0.983	0.976	4.39E-154	4
LAMA2	3.03E-157	0.83411097	0.787	0.509	6.05E-154	4
DPT	8.15E-156	0.7565724	0.743	0.41	1.63E-152	4
APOD	1.33E-155	2.34331276	0.94	0.872	2.67E-152	4
GALNT15	3.26E-153	0.46392354	0.823	0.723	6.52E-150	4
CAMP	3.83E-153	0.39922227	0.786	0.806	7.66E-150	4
SFRP1	4.79E-153	1.16120256	0.827	0.646	9.57E-150	4
CYGB	1.83E-152	0.54831698	0.822	0.79	3.67E-149	4
GPC3	1.26E-151	1.15112829	0.748	0.52	2.52E-148	4
PLPP3	1.40E-151	0.94315613	0.898	0.847	2.80E-148	4
BNC2	9.72E-151	0.57271326	0.715	0.526	1.94E-147	4
MT2A	1.26E-150	1.00245512	1	0.982	2.53E-147	4
CYP27A1	2.33E-149	0.37272633	0.78	0.565	4.66E-146	4
FGL2	7.64E-149	0.37934843	0.881	0.513	1.53E-145	4
ABCA9	5.88E-144	1.27226322	0.771	0.735	1.18E-140	4
LTC4S	1.87E-143	0.35746318	0.864	0.799	3.74E-140	4
DCLK1	2.03E-143	1.00943331	0.79	0.622	4.07E-140	4
SFRP4	2.79E-143	0.57230833	0.731	0.436	5.59E-140	4
HHIP	9.64E-143	0.73354072	0.684	0.412	1.93E-139	4
SELENOP	1.89E-142	0.80654818	0.949	0.872	3.78E-139	4
TPM2	3.47E-142	-1.2217395	0.995	0.981	6.95E-139	4
F3	2.59E-141	0.56114439	0.771	0.711	5.18E-138	4
TAGLN	4.51E-141	-1.1135303	1	0.996	9.02E-138	4
CHRDL1	4.10E-140	1.12059941	0.797	0.813	8.20E-137	4
ECM1	2.13E-138	0.30249621	0.769	0.599	4.26E-135	4
TCIM	3.99E-138	0.62047622	0.819	0.793	7.99E-135	4
PDPN	6.80E-137	0.34528329	0.666	0.368	1.36E-133	4
BCAT1	6.71E-135	0.43355841	0.772	0.631	1.34E-131	4
OAF	6.82E-135	0.88367297	0.694	0.39	1.36E-131	4
PPP1R14A	9.75E-135	-1.1885269	0.944	0.923	1.95E-131	4
DSTN	3.19E-134	-1.1057203	0.993	0.977	6.37E-131	4
KCTD12	4.03E-134	0.6257584	0.861	0.684	8.06E-131	4
FLRT2	3.60E-133	1.0897712	0.775	0.705	7.20E-130	4
VCAN	6.15E-133	0.32094122	0.961	0.759	1.23E-129	4
PRELP	2.82E-132	0.79312564	0.947	0.732	5.63E-129	4
CCDC71L	8.42E-130	0.33991117	0.879	0.792	1.68E-126	4
CYBA	1.33E-128	0.68602096	0.94	0.791	2.66E-125	4
COL3A1	1.54E-128	0.64498773	0.979	0.821	3.08E-125	4
CES1	6.34E-125	0.27468612	0.797	0.589	1.27E-121	4
TNXB	6.83E-125	0.90756184	0.805	0.722	1.37E-121	4
MT1E	1.76E-123	0.85833389	1	0.903	3.53E-120	4
SOD2	1.98E-123	0.6738045	0.912	0.684	3.96E-120	4
ABCA10	6.00E-123	1.19075157	0.751	0.781	1.20E-119	4
SLC2A3	5.11E-120	0.47340143	0.812	0.522	1.02E-116	4
COL15A1	8.67E-119	0.48681	0.891	0.81	1.73E-115	4
RARRES2	1.62E-118	0.71892344	0.971	0.866	3.24E-115	4
H19	8.12E-118	1.30123886	0.763	0.772	1.62E-114	4
TSPAN8	6.21E-117	0.40156621	0.798	0.604	1.24E-113	4

SERPINE2	5.22E-116	0.40879256	0.798	0.684	1.04E-112	4
PLN	5.40E-116	-1.3742614	0.872	0.911	1.08E-112	4
TPM1	9.21E-114	-0.818407	0.997	0.975	1.84E-110	4
PTGDS	9.46E-114	0.97609539	0.846	0.813	1.89E-110	4
TMEM45A	1.63E-112	0.48084844	0.686	0.414	3.27E-109	4
COL14A1	1.69E-111	0.65405047	0.987	0.897	3.39E-108	4
NPDC1	1.67E-110	0.47401589	0.928	0.799	3.33E-107	4
AOX1	2.85E-108	0.52716221	0.712	0.617	5.69E-105	4
CXCL12	3.30E-103	0.89209788	0.907	0.788	6.61E-100	4
FBLN5	2.10E-101	0.50677137	0.843	0.606	4.19E-98	4
FMOD	3.85E-101	0.41190763	0.76	0.562	7.70E-98	4
MYL9	1.20E-100	-0.9645181	0.999	0.985	2.39E-97	4
FGF18	5.46E-100	0.39386041	0.581	0.277	1.09E-96	4
IL32	3.35E-99	0.28286939	0.791	0.771	6.71E-96	4
MAFB	1.38E-98	0.56665374	0.907	0.745	2.76E-95	4
C11orf96	4.59E-98	-1.1739116	0.965	0.944	9.19E-95	4
CAV1	1.02E-97	-0.8963294	0.988	0.953	2.04E-94	4
ADAMTS1	6.46E-97	0.60421498	0.947	0.819	1.29E-93	4
ADAMTS5	4.74E-95	0.85226077	0.702	0.54	9.47E-92	4
SPOCK1	3.29E-94	0.97802673	0.697	0.563	6.58E-91	4
CLU	4.29E-93	0.74159322	0.992	0.92	8.57E-90	4
IL33	9.38E-93	0.32820852	0.833	0.698	1.88E-89	4
AP002956.1	1.30E-91	-1.0285311	0.865	0.842	2.60E-88	4
SNED1	3.38E-91	0.41259646	0.713	0.499	6.76E-88	4
NPC2	1.40E-89	0.44469448	0.948	0.731	2.80E-86	4
DAB2	1.48E-88	0.56332585	0.734	0.571	2.97E-85	4
MT1F	1.26E-87	0.31483625	0.845	0.731	2.53E-84	4
FKBP11	5.73E-87	0.35297266	0.731	0.602	1.15E-83	4
BHLHE41	9.56E-87	0.35215042	0.72	0.57	1.91E-83	4
PLAT	2.26E-85	0.69819881	0.801	0.771	4.52E-82	4
GFPT2	6.18E-83	0.36634292	0.692	0.558	1.24E-79	4
GGT5	1.85E-82	0.44580049	0.829	0.631	3.69E-79	4
TCF15	2.34E-82	-0.536062	0.472	0.815	4.68E-79	4
FAP	5.81E-82	0.25645074	0.647	0.414	1.16E-78	4
PRCP	1.08E-80	0.40424543	0.85	0.71	2.16E-77	4
NID1	3.97E-80	0.61819096	0.751	0.673	7.93E-77	4
CTSD	1.15E-77	0.48973359	0.953	0.795	2.31E-74	4
NFKBIA	2.08E-77	0.41430897	0.975	0.883	4.15E-74	4
MEDAG	1.56E-76	0.59993808	0.654	0.517	3.11E-73	4
PTGIS	1.82E-76	0.59048993	0.714	0.512	3.64E-73	4
LGALS3	7.90E-76	0.50366978	0.989	0.939	1.58E-72	4
ATF5	2.83E-75	0.3465655	0.709	0.55	5.67E-72	4
CLSTN2	9.78E-75	0.74253	0.749	0.752	1.96E-71	4
GCHFR	7.05E-74	0.37442498	0.698	0.623	1.41E-70	4
DEPP1	1.13E-73	0.33932289	0.879	0.681	2.27E-70	4
GPX1	1.97E-72	0.38226642	0.892	0.584	3.93E-69	4
CD320	8.78E-71	0.25458635	0.824	0.642	1.76E-67	4
A2M	2.41E-70	-0.8234474	0.977	0.972	4.83E-67	4
ITGA8	1.14E-69	-1.1757027	0.633	0.769	2.28E-66	4
MDK	1.28E-69	0.47264206	0.782	0.645	2.57E-66	4
ACTN1	2.11E-69	-0.8322175	0.858	0.871	4.21E-66	4
CD248	6.00E-69	0.53035279	0.623	0.36	1.20E-65	4
PLIN2	1.43E-68	0.39607088	0.735	0.584	2.86E-65	4

CTSB	2.70E-68	0.35759982	0.825	0.608	5.41E-65	4
MBP	3.53E-68	0.37698254	0.713	0.637	7.05E-65	4
CENPW	4.54E-68	0.26776085	0.623	0.454	9.08E-65	4
GFRA1	1.33E-67	0.78098413	0.735	0.695	2.67E-64	4
CD55	4.50E-67	0.42564399	0.73	0.586	8.99E-64	4
CSRP1	5.45E-66	-0.9349376	0.905	0.885	1.09E-62	4
LMOD1	1.26E-65	-0.8978674	0.938	0.91	2.52E-62	4
EPHA4	1.97E-65	-0.3483558	0.635	0.76	3.94E-62	4
PPP1R12B	8.90E-65	-0.9936176	0.788	0.833	1.78E-61	4
STEAP4	5.03E-64	0.91929475	0.827	0.777	1.01E-60	4
IL1R1	8.24E-64	0.29609065	0.682	0.529	1.65E-60	4
TIMP3	1.24E-62	0.46741052	1	0.927	2.49E-59	4
VIT	3.02E-60	0.6183055	0.606	0.461	6.04E-57	4
VEGFD	3.51E-60	0.83772389	0.582	0.39	7.02E-57	4
BCAM	3.74E-60	-1.0407549	0.891	0.95	7.49E-57	4
SEMA3D	4.02E-60	0.46358375	0.674	0.533	8.05E-57	4
LGMN	3.47E-59	0.25681662	0.749	0.522	6.93E-56	4
LINC00702	8.14E-58	-0.660888	0.574	0.786	1.63E-54	4
HLA-B	1.62E-57	0.35857436	0.987	0.908	3.25E-54	4
XBP1	1.78E-57	0.36085404	0.798	0.585	3.56E-54	4
CCL26	3.82E-57	0.36825408	0.612	0.521	7.64E-54	4
TNNT3	1.09E-56	0.47668834	0.625	0.578	2.17E-53	4
CNN1	1.55E-56	-1.156223	0.785	0.833	3.11E-53	4
MATN2	1.79E-56	0.28563205	0.683	0.54	3.57E-53	4
CSTB	1.23E-55	0.34878494	0.904	0.718	2.46E-52	4
RGCC	9.80E-55	-0.4880538	0.791	0.856	1.96E-51	4
BOC	3.75E-54	0.79581472	0.65	0.579	7.50E-51	4
ACKR3	4.08E-54	0.39049047	0.557	0.311	8.15E-51	4
FST	9.55E-51	0.83722406	0.649	0.642	1.91E-47	4
LRRC17	3.42E-49	0.40573561	0.826	0.766	6.85E-46	4
RND3	2.51E-48	0.30549462	0.789	0.599	5.01E-45	4
KCNQ1OT1	1.52E-47	0.58667142	0.633	0.461	3.04E-44	4
HES4	4.10E-47	-0.8389183	0.8	0.904	8.20E-44	4
CTSL	1.78E-46	0.33178474	0.809	0.666	3.56E-43	4
RAMP1	2.19E-45	-0.8493263	0.53	0.734	4.38E-42	4
SLC40A1	2.26E-45	0.28665784	0.691	0.511	4.52E-42	4
NEXN	5.66E-45	-0.8847535	0.978	0.926	1.13E-41	4
RERGL	9.08E-45	-1.4364084	0.838	0.947	1.82E-41	4
CRIP1	5.09E-44	-1.0568945	0.942	0.953	1.02E-40	4
FAM13C	6.08E-44	0.31699384	0.794	0.636	1.22E-40	4
MYLK	1.17E-43	-0.7436819	0.916	0.868	2.35E-40	4
CRIP2	9.17E-42	-0.7396524	0.885	0.884	1.83E-38	4
ADM	6.98E-41	0.41835083	0.725	0.67	1.40E-37	4
IGFBP3	7.57E-41	0.94897283	0.713	0.788	1.51E-37	4
MTUS1	5.14E-40	0.26452823	0.878	0.862	1.03E-36	4
IFIT1	5.96E-40	0.4219966	0.55	0.416	1.19E-36	4
LPP	6.94E-40	-0.6931648	0.971	0.923	1.39E-36	4
CALD1	1.11E-39	-0.3937591	1	0.986	2.23E-36	4
LEPR	1.42E-39	0.39879904	0.706	0.67	2.85E-36	4
FOXC2	2.63E-38	-0.4566793	0.5	0.717	5.26E-35	4
CCL21	1.61E-37	0.75410023	0.771	0.692	3.22E-34	4
ACTG2	1.30E-36	-0.9346989	0.783	0.782	2.60E-33	4
SORBS2	2.75E-36	-0.8913243	0.852	0.919	5.50E-33	4

BLVRB	1.53E-33	0.25597682	0.861	0.751	3.06E-30	4
CARMN	4.92E-33	-0.9360039	0.749	0.814	9.85E-30	4
RCAN2	1.27E-32	-0.8422575	0.789	0.816	2.55E-29	4
MICAL2	6.35E-32	-0.5323289	0.488	0.681	1.27E-28	4
CSRP2	1.02E-31	-1.0609333	0.856	0.847	2.03E-28	4
FGF10	5.38E-31	0.32818525	0.53	0.359	1.08E-27	4
KCNAB1	2.12E-30	-0.6931594	0.653	0.801	4.24E-27	4
IFI6	2.25E-30	0.25647853	0.638	0.485	4.50E-27	4
C12orf75	3.39E-30	-0.833348	0.678	0.804	6.78E-27	4
MEST	8.75E-30	0.35735957	0.566	0.545	1.75E-26	4
FGF7	1.13E-29	0.590329	0.679	0.673	2.26E-26	4
CLMP	1.38E-29	0.51425921	0.516	0.374	2.76E-26	4
TINAGL1	7.91E-29	-0.6328955	0.878	0.849	1.58E-25	4
LSP1	5.68E-28	0.49015618	0.523	0.368	1.14E-24	4
VCL	6.60E-28	-0.6547481	0.862	0.872	1.32E-24	4
GUCY1A1	6.79E-28	-0.655288	0.792	0.83	1.36E-24	4
FBXO32	7.17E-28	-0.6951811	0.665	0.764	1.43E-24	4
PTGFR	1.00E-27	0.58455299	0.568	0.527	2.01E-24	4
SPON2	1.15E-27	0.63547868	0.586	0.542	2.30E-24	4
PRPH	1.46E-27	-0.2528883	0.481	0.697	2.91E-24	4
BGN	1.52E-27	-0.6317828	0.989	0.948	3.04E-24	4
SVEP1	1.96E-27	0.89041513	0.609	0.676	3.91E-24	4
MAP1B	2.67E-27	-0.4950864	0.95	0.902	5.35E-24	4
TBX2	2.95E-27	-0.7044853	0.613	0.738	5.90E-24	4
ITIH3	1.82E-26	-0.2717928	0.534	0.676	3.63E-23	4
LAMB1	1.26E-25	0.58613797	0.707	0.774	2.52E-22	4
ID3	1.57E-25	0.26591101	0.927	0.796	3.13E-22	4
EPAS1	7.02E-25	-0.6406451	0.881	0.86	1.40E-21	4
FILIP1L	8.20E-25	-0.770256	0.964	0.937	1.64E-21	4
ENTPD3	9.86E-25	-0.3412825	0.464	0.687	1.97E-21	4
NOTCH3	2.07E-24	-0.6509026	0.942	0.944	4.15E-21	4
OSR1	2.45E-24	0.48703251	0.618	0.594	4.89E-21	4
NPY1R	4.85E-24	-0.2756266	0.528	0.757	9.69E-21	4
ITIH5	6.81E-24	-0.6901279	0.721	0.824	1.36E-20	4
FAM43A	7.11E-24	0.39087648	0.641	0.686	1.42E-20	4
ADGRD1	1.14E-23	0.52528362	0.61	0.608	2.27E-20	4
KCNMA1	1.64E-23	-0.5761023	0.556	0.701	3.28E-20	4
TFPI	2.53E-23	0.28039383	0.679	0.631	5.07E-20	4
SLC22A3	2.77E-23	-0.2592896	0.288	0.517	5.55E-20	4
SORL1	4.05E-23	-0.2514531	0.359	0.548	8.11E-20	4
SNAI2	4.31E-23	0.37771242	0.645	0.629	8.62E-20	4
EDIL3	1.07E-22	-0.4574897	0.573	0.753	2.14E-19	4
PCSK5	1.69E-22	0.33597761	0.584	0.541	3.38E-19	4
HMCN1	2.64E-22	-0.3703585	0.72	0.581	5.28E-19	4
LBH	1.31E-21	-0.9483573	0.827	0.776	2.62E-18	4
AP000892.3	1.52E-21	-0.6566067	0.671	0.761	3.05E-18	4
LIFR	2.22E-21	0.4095255	0.838	0.871	4.43E-18	4
SNCG	2.82E-21	-1.010567	0.84	0.874	5.64E-18	4
CLMN	3.44E-21	-0.5390925	0.66	0.85	6.87E-18	4
NTM	3.93E-21	0.30199291	0.351	0.526	7.86E-18	4
IGFBP2	1.16E-20	-0.7300286	0.832	0.86	2.31E-17	4
WNT5A	3.09E-20	0.50926781	0.552	0.559	6.17E-17	4
DES	4.52E-20	-0.7221058	0.494	0.634	9.05E-17	4

RBPMS2	1.31E-19	-0.5001338	0.583	0.746	2.63E-16	4
CRLF1	4.56E-18	0.25010896	0.623	0.547	9.11E-15	4
EBF2	4.95E-18	0.31097957	0.64	0.633	9.89E-15	4
IFIT3	6.25E-18	0.29402189	0.479	0.374	1.25E-14	4
PLCB4	1.37E-17	-0.3330603	0.457	0.608	2.73E-14	4
MBNL1-AS1	1.42E-17	-0.3289975	0.533	0.654	2.85E-14	4
ACTC1	1.53E-17	-0.3489956	0.336	0.497	3.06E-14	4
CCDC3	3.15E-17	-0.592607	0.615	0.7	6.31E-14	4
PALLD	5.25E-17	-0.4689139	0.978	0.926	1.05E-13	4
RAPGEF5	7.32E-17	-0.4424338	0.727	0.73	1.46E-13	4
MRVI1	1.01E-16	-0.6455048	0.748	0.776	2.02E-13	4
SUSD5	1.49E-16	-0.4108016	0.381	0.614	2.97E-13	4
CRYAB	5.64E-16	-0.541388	0.977	0.903	1.13E-12	4
IGF2.1	8.96E-16	0.41647661	0.523	0.477	1.79E-12	4
PCOLCE2	1.58E-15	0.27826178	0.686	0.684	3.15E-12	4
ADGRG2	1.74E-15	0.38515411	0.532	0.425	3.49E-12	4
TNFRSF12A	3.27E-15	-0.702175	0.438	0.582	6.55E-12	4
C17orf58	5.16E-15	0.35758466	0.485	0.422	1.03E-11	4
CCN1	8.25E-15	-0.8417554	0.928	0.887	1.65E-11	4
PRUNE2	1.17E-14	-0.4156093	0.604	0.65	2.34E-11	4
LTBP2	1.50E-14	-0.8233213	0.717	0.579	3.01E-11	4
ATP1A2	4.17E-14	-0.5333483	0.576	0.685	8.34E-11	4
SMTN	4.54E-14	-0.6194577	0.648	0.688	9.08E-11	4
COL4A2	5.06E-14	-0.499691	0.888	0.855	1.01E-10	4
ADGRF5	1.76E-13	0.26517327	0.733	0.708	3.52E-10	4
KLHL23	3.75E-13	-0.4543974	0.584	0.745	7.50E-10	4
CTSH	1.07E-12	0.32751324	0.623	0.658	2.14E-09	4
TGM2	1.25E-12	-0.5436548	0.747	0.794	2.49E-09	4
ITGA10	2.26E-12	-0.498639	0.52	0.633	4.53E-09	4
TBX2-AS1	3.86E-12	-0.5590266	0.571	0.653	7.73E-09	4
NEDD9	4.31E-12	-0.3442227	0.764	0.72	8.63E-09	4
GUCY1A2	1.28E-11	0.35658573	0.618	0.639	2.56E-08	4
CCN2	1.42E-11	-1.3068015	0.882	0.842	2.85E-08	4
RHOBTB3	1.87E-11	0.38527984	0.626	0.683	3.74E-08	4
PDLIM3	2.59E-11	-0.5230086	0.803	0.811	5.17E-08	4
NRGN	6.80E-11	-0.4731616	0.628	0.704	1.36E-07	4
CPXM1	1.18E-10	0.38944717	0.41	0.607	2.35E-07	4
HIST1H1C	1.50E-10	-0.2638529	0.427	0.489	2.99E-07	4
ITGA7	4.51E-10	-0.5760429	0.698	0.75	9.02E-07	4
SYNM	9.83E-10	-0.5190198	0.623	0.645	1.97E-06	4
SORBS1	1.35E-09	-0.5646036	0.644	0.68	2.70E-06	4
ACKR4	1.37E-09	0.31292481	0.551	0.545	2.74E-06	4
SLPI	1.63E-09	1.08480663	0.503	0.472	3.26E-06	4
SLC7A2	2.10E-09	-0.3199572	0.756	0.895	4.19E-06	4
PHLDA2	3.17E-09	-0.5632068	0.637	0.7	6.35E-06	4
DKK3	9.78E-09	-0.4759366	0.79	0.796	1.96E-05	4
MYH10	1.60E-08	-0.5370373	0.76	0.611	3.20E-05	4
CD9	8.81E-08	-0.383997	0.901	0.817	0.00017619	4
FHL5	6.76E-07	-0.2734527	0.801	0.791	0.00135101	4
RRAD	8.21E-07	-0.4749461	0.792	0.795	0.00164201	4
ANGPT1	1.19E-06	-0.2559653	0.543	0.649	0.00238811	4
NT5DC2	1.41E-06	-0.4773294	0.648	0.667	0.00282079	4
HLA-DRA	1.92E-06	-0.2740445	0.875	0.828	0.00384425	4

ELN	2.33E-06	-0.539407	0.739	0.577	0.00465648	4
CKB	2.61E-06	-0.6069287	0.788	0.765	0.00521461	4
HAS2	4.07E-06	0.36767307	0.447	0.312	0.00813904	4
PTN	4.48E-06	-0.327163	0.574	0.687	0.00895835	4
CRIM1	4.74E-06	-0.4618553	0.722	0.751	0.00948081	4
ESAM	6.06E-06	-0.3648471	0.637	0.698	0.01211466	4
CTSC	6.36E-06	0.47672345	0.585	0.665	0.01271525	4
FBLIM1	6.45E-06	-0.4019983	0.774	0.768	0.012893	4
NET1	1.96E-05	-0.4134506	0.806	0.79	0.03915109	4
FN1	2.03E-05	-0.8120681	0.905	0.823	0.04061322	4
MYOCD	2.26E-05	-0.4662696	0.546	0.628	0.04517045	4
HES1	4.07E-05	-0.4452588	0.713	0.783	0.08143306	4
GJA4	4.45E-05	-0.3685713	0.617	0.704	0.0890543	4
PRSS23	5.41E-05	-0.4238704	0.514	0.59	0.1082329	4
ID4	5.86E-05	-0.2971799	0.734	0.769	0.11713833	4
ADH1C	6.86E-05	0.32928702	0.553	0.767	0.13717157	4
PDE5A	8.50E-05	-0.3704379	0.75	0.816	0.17006061	4
TNC	0.00013087	-0.2978625	0.389	0.489	0.26174131	4
MT1L	0.00056371	-0.2750949	0.66	0.78	1	4
CCDC102B	0.00067313	0.27370052	0.464	0.455	1	4
COL8A1	0.00113989	-0.3070952	0.648	0.618	1	4
ISYNA1	0.00114885	-0.3896192	0.711	0.712	1	4
CCN3	0.00119996	-0.8434529	0.768	0.694	1	4
CPM	0.00184077	-0.2687092	0.76	0.666	1	4
KCNMB1	0.00254861	-0.3642868	0.636	0.638	1	4
CCND1	0.00426095	-0.3573063	0.731	0.72	1	4
INHBA	0.00525217	-0.3473371	0.421	0.439	1	4
FHL1	0.00613123	-0.4396985	0.944	0.884	1	4

Table S5. Markers for cluster annotation in human ascending aorta (GSE213740)

gene	p_val	avg_log2FC	pct.1	pct.2	p_val_adj	cluster
DCN	0	4.20160902	0.984	0.509	0	Fibroblast
APOD	0	3.95119586	0.818	0.464	0	Fibroblast
PLA2G2A	0	3.89232615	0.801	0.351	0	Fibroblast
C3	0	3.84279218	0.956	0.591	0	Fibroblast
CFD	0	3.72433623	0.964	0.731	0	Fibroblast
FBLN1	0	3.70954977	0.952	0.51	0	Fibroblast
IGFBP6	0	3.40919611	0.956	0.521	0	Fibroblast
COL1A1	0	3.33942029	0.99	0.765	0	Fibroblast
FBN1	0	3.26487987	0.924	0.569	0	Fibroblast
C7	0	3.26422974	0.794	0.36	0	Fibroblast
C1R	0	3.25568555	0.971	0.506	0	Fibroblast
COL1A2	0	3.22652346	0.988	0.755	0	Fibroblast
SFRP2	0	3.19111769	0.826	0.41	0	Fibroblast
CXCL14	0	3.16587619	0.656	0.225	0	Fibroblast
COL3A1	0	3.10355432	0.978	0.761	0	Fibroblast
C1S	0	3.06267784	0.964	0.463	0	Fibroblast
ABCA8	0	3.05586918	0.944	0.504	0	Fibroblast
MGP	0	3.03565831	0.98	0.781	0	Fibroblast
GSN	0	3.03031381	0.99	0.825	0	Fibroblast
SCARA5	0	2.95431969	0.845	0.47	0	Fibroblast
MMP2	0	2.92706843	0.883	0.302	0	Fibroblast
PODN	0	2.85432169	0.897	0.465	0	Fibroblast
IGF1	0	2.83540776	0.84	0.427	0	Fibroblast
ADH1B	0	2.74450215	0.779	0.293	0	Fibroblast
MFAP5	0	2.73974206	0.75	0.273	0	Fibroblast
SERPINF1	0	2.65438291	0.944	0.532	0	Fibroblast
COL6A3	0	2.59869553	0.836	0.46	0	Fibroblast
H19	0	2.58580714	0.842	0.564	0	Fibroblast
LUM	0	2.51974711	0.762	0.246	0	Fibroblast
FSTL1	0	2.51173137	0.902	0.464	0	Fibroblast
RARRES1	0	2.44420479	0.662	0.389	0	Fibroblast
CCDC80	0	2.40036049	0.927	0.446	0	Fibroblast
OGN	0	2.39073893	0.821	0.349	0	Fibroblast
PI16	0	2.33833004	0.675	0.347	0	Fibroblast
ABI3BP	0	2.30061479	0.819	0.412	0	Fibroblast
ABCA9	0	2.28643186	0.795	0.376	0	Fibroblast
ABCA10	0	2.24266974	0.788	0.467	0	Fibroblast
GPX3	0	2.20429672	0.877	0.481	0	Fibroblast
PDGFRA	0	2.19243009	0.776	0.316	0	Fibroblast
SRPX	0	2.17749265	0.802	0.35	0	Fibroblast
COL14A1	0	2.16899059	0.885	0.545	0	Fibroblast
PLPP3	0	2.15920019	0.832	0.507	0	Fibroblast
COL6A1	0	2.15366429	0.956	0.67	0	Fibroblast
IGFBP5	0	2.15188888	0.978	0.831	0	Fibroblast
ITM2A	0	2.14611293	0.873	0.426	0	Fibroblast
MEG3	0	2.13743048	0.744	0.286	0	Fibroblast
TSHZ2	0	2.11312901	0.881	0.566	0	Fibroblast
NID1	0	2.10570717	0.819	0.495	0	Fibroblast
SFRP1	0	2.08361437	0.693	0.315	0	Fibroblast
VCAN	0	2.04653769	0.824	0.415	0	Fibroblast
AC080038.1	0	2.02355048	0.762	0.267	0	Fibroblast

GAS1	0	2.02193386	0.749	0.391	0	Fibroblast
LTBP1	0	2.01970966	0.785	0.443	0	Fibroblast
IGFBP3	0	1.97431501	0.637	0.3	0	Fibroblast
FBLN2	0	1.96728444	0.788	0.36	0	Fibroblast
EFEMP1	0	1.94179992	0.729	0.225	0	Fibroblast
SPOCK1	0	1.92648507	0.7	0.359	0	Fibroblast
HTRA3	0	1.91714606	0.666	0.278	0	Fibroblast
GPC3	0	1.91505662	0.768	0.381	0	Fibroblast
LTBP4	0	1.91301109	0.841	0.442	0	Fibroblast
CD248	0	1.89576178	0.788	0.471	0	Fibroblast
CFH	0	1.85421446	0.785	0.304	0	Fibroblast
FBLN5	0	1.84689102	0.753	0.256	0	Fibroblast
SEMA3C	0	1.82504137	0.667	0.38	0	Fibroblast
PRELP	0	1.79353065	0.828	0.416	0	Fibroblast
PDGFRL	0	1.78818672	0.703	0.332	0	Fibroblast
DCLK1	0	1.72142924	0.761	0.43	0	Fibroblast
AKAP12	0	1.7192523	0.844	0.592	0	Fibroblast
ABCA6	0	1.707181	0.754	0.453	0	Fibroblast
SVEP1	0	1.70176162	0.633	0.408	0	Fibroblast
OLFML3	0	1.67962444	0.714	0.266	0	Fibroblast
LOX	0	1.67678604	0.658	0.342	0	Fibroblast
IGSF10	0	1.66632648	0.7	0.451	0	Fibroblast
PCOLCE	0	1.65864377	0.756	0.36	0	Fibroblast
BOC	0	1.60452842	0.698	0.434	0	Fibroblast
FLRT2	0	1.5967096	0.743	0.516	0	Fibroblast
CILP	0	1.55883201	0.605	0.263	0	Fibroblast
AEBP1	0	1.55038229	0.872	0.532	0	Fibroblast
CHRDL1	0	1.54704249	0.724	0.461	0	Fibroblast
TGFBR3	0	1.53647638	0.806	0.512	0	Fibroblast
LAMA2	0	1.52810867	0.603	0.241	0	Fibroblast
COL5A1	0	1.52419332	0.759	0.489	0	Fibroblast
FN1	0	1.514107	0.836	0.488	0	Fibroblast
LAMA4	0	1.50568548	0.752	0.55	0	Fibroblast
CXCL12	0	1.49836147	0.73	0.449	0	Fibroblast
RSPO3	0	1.48277796	0.679	0.427	0	Fibroblast
BICC1	0	1.46483282	0.656	0.352	0	Fibroblast
PMP22	0	1.46023312	0.85	0.469	0	Fibroblast
ITGBL1	0	1.45844606	0.573	0.247	0	Fibroblast
HELLPAR	0	1.44797598	0.656	0.444	0	Fibroblast
TNXB	0	1.43952373	0.655	0.315	0	Fibroblast
FMO2	0	1.41975906	0.556	0.352	0	Fibroblast
PTGIS	0	1.40698817	0.616	0.294	0	Fibroblast
SLPI	0	1.40416606	0.431	0.25	0	Fibroblast
SLIT2	0	1.38697217	0.619	0.384	0	Fibroblast
GFRA1	0	1.3861461	0.711	0.477	0	Fibroblast
CRISPLD2	0	1.38589811	0.651	0.394	0	Fibroblast
MYOC	0	1.38283043	0.447	0.232	0	Fibroblast
FST	0	1.35673494	0.582	0.399	0	Fibroblast
ELN	0	1.35565992	0.74	0.47	0	Fibroblast
MGST1	0	1.33698412	0.511	0.259	0	Fibroblast
NEGR1	0	1.29718052	0.512	0.305	0	Fibroblast
DPT	0	1.29587128	0.524	0.239	0	Fibroblast
SPRY1	0	1.29177585	0.626	0.374	0	Fibroblast

LAMB1	0	1.28275821	0.609	0.318	0	Fibroblast
RARRES2	0	1.271356	0.78	0.585	0	Fibroblast
DDR2	0	1.2673795	0.744	0.552	0	Fibroblast
PPL	0	1.25742102	0.64	0.391	0	Fibroblast
SPARC	0	1.25066071	0.912	0.703	0	Fibroblast
TIMP1	0	1.24226689	0.929	0.776	0	Fibroblast
F10	0	1.2352423	0.69	0.445	0	Fibroblast
KCNQ1OT1	0	1.22921665	0.722	0.417	0	Fibroblast
COL5A2	0	1.22427897	0.701	0.442	0	Fibroblast
PCOLCE2	0	1.19623964	0.444	0.197	0	Fibroblast
VIT	0	1.1882481	0.531	0.194	0	Fibroblast
EPHX1	0	1.18708069	0.686	0.431	0	Fibroblast
ABCC9	0	1.15353248	0.645	0.463	0	Fibroblast
CD34	0	1.13848079	0.744	0.389	0	Fibroblast
CRLF1	0	1.11929767	0.504	0.195	0	Fibroblast
ALDH1A1	0	1.11487582	0.707	0.454	0	Fibroblast
LRRC17	0	1.10236235	0.628	0.399	0	Fibroblast
OAF	0	1.10058265	0.658	0.452	0	Fibroblast
SPON2	0	1.09762514	0.627	0.347	0	Fibroblast
RHOBTB3	0	1.08614008	0.594	0.381	0	Fibroblast
CDKN1C	0	1.08495537	0.742	0.42	0	Fibroblast
VEGFD	0	1.07587474	0.557	0.345	0	Fibroblast
CCDC102B	0	1.03196274	0.591	0.308	0	Fibroblast
CPXM1	0	1.03082276	0.585	0.408	0	Fibroblast
NOVA1	0	1.0284614	0.53	0.242	0	Fibroblast
MFAP4	0	1.02562824	0.625	0.316	0	Fibroblast
CLMP	0	1.01237418	0.558	0.32	0	Fibroblast
CTSK	0	1.00077676	0.519	0.287	0	Fibroblast
FGF7	0	0.9985078	0.556	0.412	0	Fibroblast
EMP1	0	0.99495353	0.643	0.435	0	Fibroblast
COL12A1	0	0.99338906	0.595	0.394	0	Fibroblast
CPE	0	0.9867262	0.842	0.664	0	Fibroblast
IL11RA	0	0.98207763	0.549	0.238	0	Fibroblast
COL4A1	0	0.95719962	0.679	0.571	0	Fibroblast
ADAMTS5	0	0.94612949	0.575	0.403	0	Fibroblast
IGFBP4	0	0.92833938	0.828	0.519	0	Fibroblast
LINC00632	0	0.92010129	0.653	0.467	0	Fibroblast
THY1	0	0.91753098	0.538	0.344	0	Fibroblast
MEDAG	0	0.91702216	0.471	0.278	0	Fibroblast
PTGFR	0	0.9123812	0.518	0.313	0	Fibroblast
CYGB	0	0.9121352	0.534	0.308	0	Fibroblast
S100A10	0	0.90688755	0.873	0.632	0	Fibroblast
BNC2	0	0.88009234	0.568	0.348	0	Fibroblast
TNNT3	0	0.86276231	0.554	0.376	0	Fibroblast
FSTL3	0	0.84470072	0.504	0.218	0	Fibroblast
PLTP	0	0.84320839	0.769	0.419	0	Fibroblast
NNMT	0	0.84147129	0.599	0.235	0	Fibroblast
EBF2	0	0.83483608	0.657	0.496	0	Fibroblast
WNT5A	0	0.83198296	0.55	0.289	0	Fibroblast
CLEC11A	0	0.8233091	0.583	0.395	0	Fibroblast
CADM3	0	0.82088445	0.506	0.346	0	Fibroblast
CLU	0	0.81157396	0.793	0.586	0	Fibroblast
MSX1	0	0.80993702	0.433	0.231	0	Fibroblast

SPTBN1	0	0.80875334	0.815	0.626	0	Fibroblast
PRDX4	0	0.80798657	0.652	0.492	0	Fibroblast
PLAT	0	0.80528094	0.597	0.327	0	Fibroblast
MEST	0	0.79868282	0.541	0.343	0	Fibroblast
HSPB6	0	0.79604778	0.643	0.454	0	Fibroblast
CPB1	0	0.78442197	0.542	0.294	0	Fibroblast
ABLIM1	0	0.78000769	0.582	0.362	0	Fibroblast
CLSTN2	0	0.7621492	0.601	0.471	0	Fibroblast
TMEM45A	0	0.76041296	0.451	0.28	0	Fibroblast
HHIP	0	0.750316	0.483	0.282	0	Fibroblast
ADGRD1	0	0.74803751	0.514	0.283	0	Fibroblast
FMOD	0	0.742557	0.477	0.228	0	Fibroblast
MATN2	0	0.7404444	0.497	0.394	0	Fibroblast
IGF2.1	0	0.73735033	0.575	0.37	0	Fibroblast
HAS2	0	0.72972412	0.422	0.251	0	Fibroblast
DIO3OS	0	0.72961634	0.459	0.244	0	Fibroblast
NTM	0	0.72847439	0.561	0.206	0	Fibroblast
PROCR	0	0.7273644	0.593	0.426	0	Fibroblast
GGT5	0	0.72150932	0.497	0.331	0	Fibroblast
APOC1	0	0.71605321	0.467	0.303	0	Fibroblast
CNTFR	0	0.70386843	0.452	0.282	0	Fibroblast
G0S2	0	0.70302954	0.532	0.356	0	Fibroblast
LINC01697	0	0.6891056	0.514	0.304	0	Fibroblast
SERPINE2	0	0.68798872	0.446	0.326	0	Fibroblast
AOX1	0	0.68521394	0.379	0.189	0	Fibroblast
IL6ST	0	0.68495522	0.81	0.53	0	Fibroblast
SNAI2	0	0.68432871	0.501	0.404	0	Fibroblast
BMPER	0	0.6840929	0.494	0.24	0	Fibroblast
DLC1	0	0.66286213	0.647	0.495	0	Fibroblast
THBS2	0	0.65519899	0.429	0.171	0	Fibroblast
OMD	0	0.65413907	0.377	0.181	0	Fibroblast
FGF18	0	0.61237011	0.516	0.289	0	Fibroblast
CRABP2	0	0.60961337	0.553	0.382	0	Fibroblast
SLIT3	0	0.60190496	0.758	0.637	0	Fibroblast
ADH1C	0	0.59089117	0.467	0.204	0	Fibroblast
AUXG01000	0	0.59034876	0.542	0.383	0	Fibroblast
CREB3L1	0	0.58613826	0.503	0.274	0	Fibroblast
TIMP3	0	0.58175594	0.883	0.729	0	Fibroblast
AKR1C1	0	0.57953477	0.374	0.16	0	Fibroblast
NTRK2	0	0.56470321	0.732	0.659	0	Fibroblast
SULF1	0	0.56032966	0.446	0.28	0	Fibroblast
ITGA11	0	0.55958283	0.414	0.237	0	Fibroblast
PI15	0	0.55625468	0.498	0.278	0	Fibroblast
SGCD	0	0.549884	0.425	0.265	0	Fibroblast
GFPT2	0	0.54685604	0.359	0.193	0	Fibroblast
CDH11	0	0.54640587	0.41	0.268	0	Fibroblast
ANTXR1	0	0.5405012	0.485	0.296	0	Fibroblast
FGF10	0	0.53390554	0.435	0.295	0	Fibroblast
LIFR	0	0.53043706	0.612	0.476	0	Fibroblast
PCDH7	0	0.52498906	0.446	0.284	0	Fibroblast
C16orf89	0	0.51561147	0.385	0.186	0	Fibroblast
NDRG1	0	0.51002873	0.604	0.397	0	Fibroblast
SVIL	0	0.49993176	0.62	0.453	0	Fibroblast

VEGFA	0	0.49520358	0.529	0.391	0	Fibroblast
CAMK2N1	0	0.4897843	0.493	0.335	0	Fibroblast
PEG10	0	0.48129141	0.543	0.416	0	Fibroblast
USP53	0	0.47895085	0.437	0.255	0	Fibroblast
LINC01133	0	0.47686489	0.426	0.186	0	Fibroblast
BHLHE41	0	0.46103844	0.481	0.242	0	Fibroblast
PLXDC1	0	0.42479741	0.4	0.238	0	Fibroblast
MYH10	0	0.41218736	0.513	0.365	0	Fibroblast
NR2F1	0	0.40913165	0.5	0.418	0	Fibroblast
FHL1	0	0.40369556	0.593	0.365	0	Fibroblast
HSPG2	0	0.39185425	0.787	0.655	0	Fibroblast
FOXD1	0	0.38871911	0.473	0.269	0	Fibroblast
ACKR4	0	0.38863951	0.425	0.224	0	Fibroblast
SLC26A7	0	0.38043163	0.475	0.207	0	Fibroblast
TMEM176A	0	0.37889291	0.456	0.29	0	Fibroblast
SEMA5A	0	0.37812475	0.54	0.382	0	Fibroblast
C1QTNF3	0	0.36857606	0.429	0.186	0	Fibroblast
CGNL1	0	0.35838977	0.444	0.249	0	Fibroblast
TMEM176B	0	0.35817177	0.479	0.299	0	Fibroblast
TFPI	0	0.34501968	0.555	0.408	0	Fibroblast
MT1E	0	0.34432681	0.722	0.551	0	Fibroblast
FAIM2	0	0.34097459	0.339	0.136	0	Fibroblast
FAM43A	0	0.3372768	0.491	0.239	0	Fibroblast
CHODL	0	0.32373367	0.465	0.249	0	Fibroblast
RDH10	0	0.31598243	0.368	0.214	0	Fibroblast
DLK1	0	0.31428306	0.383	0.142	0	Fibroblast
FMO1	0	0.31318519	0.444	0.207	0	Fibroblast
FSCN1	0	0.31279526	0.528	0.325	0	Fibroblast
MME	0	0.27827041	0.409	0.235	0	Fibroblast
LDB2	0	0.27530443	0.582	0.438	0	Fibroblast
AHNAK2	0	0.26544691	0.456	0.329	0	Fibroblast
GREM2	0	0.25118756	0.398	0.13	0	Fibroblast
BMP3	0	0.24795689	0.413	0.083	0	Fibroblast
ENTPD2	0	0.21331379	0.459	0.267	0	Fibroblast
PTGDS	2.27E-299	1.73896124	0.477	0.339	4.55E-296	Fibroblast
UAP1	6.64E-297	0.60783856	0.397	0.221	1.33E-293	Fibroblast
PCDH17	3.21E-294	0.2025091	0.449	0.328	6.41E-291	Fibroblast
ANGPTL1	2.10E-287	0.58249941	0.577	0.501	4.20E-284	Fibroblast
MT1M	1.34E-286	0.50106506	0.552	0.396	2.68E-283	Fibroblast
CHL1	4.24E-286	0.22658315	0.427	0.308	8.49E-283	Fibroblast
CCN5	3.39E-282	0.68414312	0.348	0.181	6.78E-279	Fibroblast
CYP1B1	3.63E-281	0.7346882	0.425	0.263	7.26E-278	Fibroblast
SPON1	3.84E-279	0.74191861	0.393	0.248	7.68E-276	Fibroblast
MXRA5	2.80E-278	0.45966491	0.389	0.197	5.61E-275	Fibroblast
LTBP2	1.09E-275	0.44043814	0.368	0.237	2.17E-272	Fibroblast
APOE	7.16E-274	1.54153954	0.61	0.508	1.43E-270	Fibroblast
PTHLH	2.27E-264	0.28897439	0.406	0.318	4.54E-261	Fibroblast
SNED1	1.60E-262	0.86494471	0.4	0.253	3.20E-259	Fibroblast
GUCY1A2	5.99E-258	0.21187615	0.498	0.422	1.20E-254	Fibroblast
PCSK5	3.08E-247	0.43164239	0.422	0.322	6.16E-244	Fibroblast
SMOC2	3.73E-238	0.48286504	0.396	0.278	7.47E-235	Fibroblast
MEG8	1.03E-224	0.60799788	0.37	0.207	2.05E-221	Fibroblast
PAMR1	1.07E-218	0.32649696	0.324	0.162	2.15E-215	Fibroblast

MEGF6	9.85E-217	0.51039311	0.335	0.203	1.97E-213	Fibroblast
PDLIM4	1.35E-211	0.30243314	0.37	0.264	2.70E-208	Fibroblast
COL4A2	3.54E-210	0.57692907	0.688	0.669	7.08E-207	Fibroblast
IGFBP2	5.47E-208	0.68253087	0.399	0.274	1.09E-204	Fibroblast
RBP4	1.49E-201	0.43454994	0.357	0.266	2.99E-198	Fibroblast
TCIM	5.82E-199	0.22759813	0.44	0.378	1.16E-195	Fibroblast
BASP1	4.70E-196	0.51561042	0.495	0.412	9.40E-193	Fibroblast
SEMA3B	9.60E-195	0.42737835	0.426	0.327	1.92E-191	Fibroblast
ZNF385D	1.93E-194	0.23491112	0.482	0.407	3.87E-191	Fibroblast
PDPN	4.32E-188	0.32999743	0.337	0.212	8.63E-185	Fibroblast
F3	1.94E-185	0.40321371	0.322	0.212	3.87E-182	Fibroblast
CA3	3.19E-183	0.21429957	0.275	0.155	6.37E-180	Fibroblast
C17orf58	2.62E-173	0.58782722	0.457	0.375	5.24E-170	Fibroblast
PRSS23	1.22E-169	0.43048659	0.386	0.3	2.43E-166	Fibroblast
LSAMP	8.77E-169	0.28393634	0.338	0.189	1.75E-165	Fibroblast
SFRP4	5.22E-161	0.83260056	0.32	0.204	1.04E-157	Fibroblast
GAS7	8.39E-158	0.54771202	0.569	0.525	1.68E-154	Fibroblast
TSPAN8	5.21E-157	0.58879113	0.345	0.264	1.04E-153	Fibroblast
IL33	1.41E-155	0.26690544	0.491	0.416	2.83E-152	Fibroblast
CREB5	6.20E-155	0.37256475	0.498	0.399	1.24E-151	Fibroblast
CHI3L2	1.32E-153	0.23900471	0.277	0.401	2.64E-150	Fibroblast
APCDD1	4.00E-144	0.52298791	0.342	0.225	8.01E-141	Fibroblast
ADAMTS1	4.55E-138	0.39782246	0.516	0.444	9.09E-135	Fibroblast
ANK2	3.67E-125	0.48630079	0.497	0.476	7.34E-122	Fibroblast
COL5A3	6.65E-125	0.38112845	0.421	0.37	1.33E-121	Fibroblast
TMEM100	1.21E-122	0.36196846	0.338	0.25	2.42E-119	Fibroblast
RND3	1.76E-122	0.45775657	0.445	0.375	3.52E-119	Fibroblast
BCYRN1	1.69E-117	0.38667733	0.435	0.384	3.37E-114	Fibroblast
ACVRL1	2.43E-117	0.3415863	0.454	0.392	4.86E-114	Fibroblast
AKR1C2	4.50E-112	0.21799065	0.281	0.179	8.99E-109	Fibroblast
GJA1	1.02E-108	0.21032953	0.355	0.29	2.03E-105	Fibroblast
LEPR	1.15E-107	0.40229397	0.38	0.304	2.30E-104	Fibroblast
PDGFD	5.86E-107	0.3832753	0.337	0.246	1.17E-103	Fibroblast
SRPX2	1.79E-89	0.63184144	0.356	0.278	3.57E-86	Fibroblast
MLXIPL	1.71E-87	0.34362513	0.32	0.221	3.42E-84	Fibroblast
COL15A1	7.99E-75	0.33160397	0.403	0.349	1.60E-71	Fibroblast
INMT	1.82E-68	0.40280679	0.338	0.289	3.65E-65	Fibroblast
CLDN11	5.24E-66	0.20696096	0.231	0.32	1.05E-62	Fibroblast
SAA1	5.24E-59	0.4903342	0.181	0.254	1.05E-55	Fibroblast
OSR2	1.22E-55	0.49855997	0.356	0.311	2.44E-52	Fibroblast
PLIN1	2.60E-52	0.2452864	0.324	0.234	5.19E-49	Fibroblast
THBS1	1.38E-44	0.27724208	0.33	0.299	2.75E-41	Fibroblast
TMEM59L	1.17E-37	0.40129839	0.331	0.304	2.34E-34	Fibroblast
HSD11B1	4.74E-35	0.29948707	0.296	0.187	9.48E-32	Fibroblast
SCG2	5.89E-35	0.31175601	0.25	0.166	1.18E-31	Fibroblast
ANGPTL2	4.14E-34	0.26890214	0.321	0.283	8.29E-31	Fibroblast
CDO1	1.15E-26	0.30156345	0.254	0.195	2.30E-23	Fibroblast
PQLC2L	2.09E-21	0.23194476	0.252	0.139	4.19E-18	Fibroblast
TIMP4	7.50E-21	0.23352715	0.256	0.225	1.50E-17	Fibroblast
VCAM1	2.81E-19	0.23803801	0.32	0.308	5.61E-16	Fibroblast
COL8A1	5.44E-18	0.26400353	0.258	0.235	1.09E-14	Fibroblast
ECM1	3.48E-15	0.28596725	0.311	0.3	6.96E-12	Fibroblast
SOX9	1.48E-14	0.41656041	0.252	0.201	2.96E-11	Fibroblast

CCL26	4.38E-13	0.27572824	0.326	0.307	8.76E-10	Fibroblast
CYP27A1	9.37E-10	0.22871975	0.303	0.269	1.87E-06	Fibroblast
TSKU	1.52E-09	0.24972624	0.251	0.276	3.04E-06	Fibroblast
TNFAIP6	3.03E-07	0.4378642	0.302	0.351	0.000606	Fibroblast
PLA2G5	9.69E-07	0.26435498	0.265	0.255	0.00193823	Fibroblast
BMP4	2.80E-06	0.22491757	0.265	0.238	0.00559268	Fibroblast
MEOX2	1.61E-05	0.2027653	0.424	0.468	0.03213128	Fibroblast
VGLL3	1.88E-05	0.27576746	0.288	0.333	0.03752874	Fibroblast
LTB	0	3.78853495	0.959	0.295	0	NK/T
IL32	0	3.4302676	0.902	0.227	0	NK/T
CCL5	0	3.35752207	0.754	0.186	0	NK/T
IL7R	0	2.89019511	0.739	0.137	0	NK/T
CD3D	0	2.63046596	0.773	0.172	0	NK/T
CD52	0	2.58391798	0.926	0.442	0	NK/T
KLRB1	0	2.49023742	0.838	0.169	0	NK/T
CD3G	0	2.47198619	0.726	0.169	0	NK/T
TRBC1	0	2.31133293	0.66	0.199	0	NK/T
NKG7	0	2.22064	0.668	0.166	0	NK/T
CD69	0	2.21288809	0.815	0.252	0	NK/T
CD7	0	2.19850463	0.923	0.172	0	NK/T
TRAC	0	2.19163709	0.76	0.19	0	NK/T
PTPRC	0	2.16859697	0.928	0.515	0	NK/T
BCL11B	0	2.12643848	0.664	0.204	0	NK/T
TRBC2	0	2.10744706	0.695	0.161	0	NK/T
CORO1A	0	1.95868162	0.831	0.448	0	NK/T
RAC2	0	1.95164088	0.749	0.342	0	NK/T
CTSW	0	1.87549878	0.744	0.193	0	NK/T
CD2	0	1.85396055	0.73	0.187	0	NK/T
IKZF1	0	1.77362146	0.788	0.36	0	NK/T
GZMM	0	1.72473373	0.799	0.176	0	NK/T
CD3E	0	1.70533893	0.782	0.172	0	NK/T
ISG20	0	1.6415813	0.721	0.296	0	NK/T
CD8A	0	1.59902698	0.643	0.117	0	NK/T
HCST	0	1.59553001	0.829	0.481	0	NK/T
LCP1	0	1.41499221	0.932	0.566	0	NK/T
ITGA4	0	1.36099566	0.88	0.409	0	NK/T
TRGC2	0	1.35238707	0.646	0.146	0	NK/T
ARHGAP30	0	1.34552464	0.95	0.402	0	NK/T
GIMAP7	0	1.34259038	0.851	0.444	0	NK/T
DUSP2	0	1.14903359	0.686	0.19	0	NK/T
PRF1	0	1.14686039	0.657	0.15	0	NK/T
CXCR4	0	1.10512889	0.785	0.304	0	NK/T
CYFIP2	0	1.09544756	0.93	0.232	0	NK/T
GBP5	0	1.0796493	0.923	0.328	0	NK/T
TRDC	0	1.01352164	0.896	0.151	0	NK/T
LCP2	0	0.9737963	0.839	0.355	0	NK/T
TNFRSF18	0	0.91504394	0.895	0.175	0	NK/T
MATK	0	0.88024991	0.748	0.236	0	NK/T
PLAC8	0	0.86927661	0.919	0.32	0	NK/T
SLFN5	0	0.86296599	0.889	0.565	0	NK/T
PIM1	0	0.81773437	0.83	0.242	0	NK/T
IL2RB	0	0.81318515	0.723	0.157	0	NK/T
TNFSF14	0	0.80922388	0.684	0.198	0	NK/T

ICOS	0	0.73074762	0.278	0.138	0	NK/T
CRTAM	0	0.70267623	0.297	0.207	0	NK/T
CCR7	0	0.69123011	0.657	0.166	0	NK/T
SLAMF7	0	0.68123831	0.793	0.331	0	NK/T
LAX1	0	0.67862814	0.693	0.185	0	NK/T
TNFRSF4	0	0.65057703	0.84	0.196	0	NK/T
MYO1F	0	0.63235066	0.93	0.476	0	NK/T
MT1F	0	0.62542181	0.273	0.245	0	NK/T
HLA-B	0	0.60198372	0.965	0.847	0	NK/T
CLIC3	0	0.5557365	0.811	0.223	0	NK/T
KLRC1	0	0.54791841	0.739	0.166	0	NK/T
LY9	0	0.51306755	0.28	0.179	0	NK/T
UCP2	0	0.49234892	0.938	0.47	0	NK/T
TRGC1	0	0.49015334	0.868	0.174	0	NK/T
ZNF683	0	0.47387033	0.886	0.104	0	NK/T
OASL	0	0.47226213	0.76	0.384	0	NK/T
TMIGD2	0	0.45581707	0.878	0.12	0	NK/T
HPGD	0	0.44495056	0.626	0.149	0	NK/T
NCKAP1L	0	0.41786221	0.911	0.448	0	NK/T
GNLY	0	0.41413351	0.641	0.187	0	NK/T
HMGA1	0	0.40706256	0.338	0.393	0	NK/T
STAT1	0	0.40505846	0.956	0.621	0	NK/T
PNP	0	0.39042541	0.278	0.227	0	NK/T
HIST1H1C	0	0.38413583	0.899	0.478	0	NK/T
LINC02446	0	0.38338807	0.897	0.068	0	NK/T
ADA	0	0.35785198	0.829	0.328	0	NK/T
CENPK	0	0.32794375	0.76	0.234	0	NK/T
LDLR	0	0.29953494	0.902	0.276	0	NK/T
MCM7	0	0.29367585	0.863	0.34	0	NK/T
RNU2-63P	0	0.2906772	0.929	0.378	0	NK/T
MAL	0	0.2870205	0.83	0.253	0	NK/T
TSPAN5	0	0.28064147	0.209	0.267	0	NK/T
EFHD2	0	0.2607444	0.947	0.509	0	NK/T
TNFRSF9	0	0.25650926	0.676	0.157	0	NK/T
IL2RA	0	0.24054088	0.264	0.196	0	NK/T
HELLS	0	0.22783996	0.897	0.271	0	NK/T
FOXP3	0	0.22139271	0.685	0.076	0	NK/T
LMNB1	0	0.21013503	0.728	0.257	0	NK/T
GBP4	0	0.20728431	0.931	0.458	0	NK/T
IRF4	0	0.20430192	0.261	0.181	0	NK/T
DUSP4	1.29E-304	0.56570437	0.277	0.156	2.58E-301	NK/T
HSPA6	2.49E-299	0.8340502	0.259	0.179	4.97E-296	NK/T
EZH2	3.06E-296	0.34081099	0.685	0.303	6.12E-293	NK/T
FYB1	2.38E-284	1.19723954	0.74	0.483	4.75E-281	NK/T
ARL4A	4.36E-284	0.2184369	0.332	0.388	8.73E-281	NK/T
ARL4C	1.31E-259	1.61028341	0.64	0.249	2.61E-256	NK/T
IRF1	1.34E-249	0.89338162	0.764	0.531	2.68E-246	NK/T
PTPN7	1.38E-245	1.73813054	0.638	0.279	2.77E-242	NK/T
RUNX3	3.09E-244	1.38447383	0.628	0.194	6.19E-241	NK/T
KLRD1	3.65E-242	0.87721395	0.599	0.193	7.30E-239	NK/T
SH3BGRL3	6.24E-235	0.28143859	0.943	0.798	1.25E-231	NK/T
TNFRSF1B	1.47E-234	0.2987645	0.844	0.484	2.94E-231	NK/T
XCL1	3.38E-223	0.70179418	0.318	0.131	6.77E-220	NK/T

HCLS1	6.15E-208	0.87016647	0.713	0.347	1.23E-204	NK/T
DDIT4	5.67E-207	0.83219485	0.701	0.363	1.13E-203	NK/T
OCIAD2	2.66E-198	0.61676706	0.329	0.191	5.32E-195	NK/T
TFRC	5.49E-193	0.3617684	0.341	0.307	1.10E-189	NK/T
GIMAP1	5.73E-191	0.28627713	0.379	0.348	1.15E-187	NK/T
KIF20B	3.58E-187	0.20224246	0.717	0.363	7.16E-184	NK/T
CD37	3.11E-186	0.97603515	0.673	0.4	6.22E-183	NK/T
KLRC2	2.34E-184	0.2112852	0.423	0.061	4.67E-181	NK/T
IKZF3	9.46E-182	1.51144431	0.607	0.197	1.89E-178	NK/T
MARCKSL1	6.28E-180	0.2270751	0.366	0.312	1.26E-176	NK/T
FERMT3	2.49E-179	0.47810575	0.697	0.325	4.99E-176	NK/T
TENT5C	4.35E-163	0.8169451	0.349	0.207	8.69E-160	NK/T
PCED1B-AS1	9.27E-162	1.43249288	0.6	0.228	1.85E-158	NK/T
CD28	1.12E-160	0.5228628	0.593	0.185	2.23E-157	NK/T
GPR183	3.94E-158	0.70177185	0.367	0.323	7.88E-155	NK/T
LSP1	1.53E-157	0.60946599	0.795	0.561	3.07E-154	NK/T
NFKBIA	1.65E-157	0.61284924	0.83	0.598	3.29E-154	NK/T
GPR65	2.95E-156	0.94516557	0.679	0.407	5.90E-153	NK/T
SLC2A1	3.91E-156	0.23128857	0.304	0.155	7.81E-153	NK/T
RGS10	1.37E-154	0.63891839	0.737	0.432	2.74E-151	NK/T
AQP3	6.16E-151	0.63391743	0.354	0.194	1.23E-147	NK/T
STMN1	3.15E-148	0.23524696	0.387	0.356	6.29E-145	NK/T
TIGIT	4.78E-147	0.52652039	0.347	0.165	9.55E-144	NK/T
PTPN6	1.09E-146	0.5480624	0.699	0.422	2.17E-143	NK/T
CD8B	7.37E-145	0.85787571	0.338	0.125	1.47E-141	NK/T
IL10RA	2.07E-135	0.48964169	0.413	0.407	4.15E-132	NK/T
CD48	3.51E-135	1.42628892	0.612	0.335	7.03E-132	NK/T
CYBA	1.12E-134	0.37864542	0.855	0.749	2.23E-131	NK/T
AD000864.1	3.43E-126	0.54678605	0.366	0.21	6.87E-123	NK/T
SDF2L1	1.77E-113	0.21039029	0.8	0.475	3.54E-110	NK/T
DUSP5	1.79E-112	0.57507196	0.367	0.197	3.57E-109	NK/T
CYTOR	4.87E-112	0.33757344	0.422	0.385	9.74E-109	NK/T
SLC2A3	3.27E-111	1.05406332	0.647	0.43	6.54E-108	NK/T
GMFG	1.12E-110	0.77184167	0.678	0.427	2.24E-107	NK/T
CCL4L2	2.00E-108	0.89567869	0.299	0.139	3.99E-105	NK/T
PTTG1	2.20E-107	0.53104084	0.371	0.217	4.39E-104	NK/T
EVI2B	1.42E-105	0.56433962	0.405	0.359	2.83E-102	NK/T
WAS	3.17E-105	0.57564892	0.416	0.387	6.34E-102	NK/T
UPP1	3.94E-103	0.37378804	0.376	0.271	7.88E-100	NK/T
CD247	1.42E-97	1.2888842	0.561	0.139	2.85E-94	NK/T
RETREG1	9.99E-97	0.36648799	0.338	0.198	2.00E-93	NK/T
CKS2	9.26E-94	0.5322472	0.379	0.201	1.85E-90	NK/T
AL031777.3	2.14E-91	0.57128993	0.486	0.391	4.29E-88	NK/T
SAMSN1	2.37E-91	0.30050862	0.434	0.418	4.74E-88	NK/T
RGS2	1.29E-88	0.39687789	0.376	0.272	2.59E-85	NK/T
ATAD2	9.55E-82	0.26733527	0.598	0.277	1.91E-78	NK/T
IFNG	6.94E-76	0.91661573	0.381	0.13	1.39E-72	NK/T
SERPINB9	7.87E-74	0.38431433	0.409	0.27	1.57E-70	NK/T
ZBP1	8.81E-74	0.25375284	0.412	0.227	1.76E-70	NK/T
AC243960.1	1.85E-68	0.96453178	0.565	0.255	3.69E-65	NK/T
GZMH	6.70E-68	0.78893538	0.356	0.15	1.34E-64	NK/T
EZR	8.11E-68	1.05199586	0.593	0.318	1.62E-64	NK/T
NR4A2	2.43E-67	0.57139465	0.631	0.341	4.86E-64	NK/T

PRDM1	1.42E-65	0.68165973	0.411	0.275	2.84E-62	NK/T
CKAP2	3.01E-63	0.38485333	0.612	0.331	6.03E-60	NK/T
MBP	8.84E-62	0.71296338	0.651	0.436	1.77E-58	NK/T
ALOX5AP	1.52E-61	0.64670306	0.401	0.199	3.04E-58	NK/T
CLSPN	2.83E-61	0.20793124	0.548	0.255	5.66E-58	NK/T
IQGAP2	7.00E-57	0.42299867	0.453	0.377	1.40E-53	NK/T
NEDD9	2.05E-55	0.30611039	0.56	0.244	4.10E-52	NK/T
GADD45G	2.03E-48	0.27830013	0.675	0.372	4.07E-45	NK/T
F2R	1.45E-45	0.21970095	0.378	0.197	2.90E-42	NK/T
XBP1	2.80E-42	0.65739478	0.68	0.481	5.60E-39	NK/T
NEFM	1.83E-41	0.26997022	0.432	0.149	3.67E-38	NK/T
HSPH1	4.05E-41	0.37539916	0.623	0.343	8.10E-38	NK/T
PMAIP1	1.11E-40	0.91833373	0.412	0.245	2.21E-37	NK/T
GZMA	1.58E-39	2.00357277	0.533	0.176	3.16E-36	NK/T
SSR4	2.14E-39	0.422086	0.77	0.723	4.29E-36	NK/T
SYTL3	1.07E-37	0.88694347	0.524	0.17	2.15E-34	NK/T
GPSM3	5.15E-36	0.80565645	0.598	0.388	1.03E-32	NK/T
ITGB2	3.03E-31	0.31946997	0.666	0.519	6.05E-28	NK/T
PIM2	1.33E-29	0.70036843	0.385	0.177	2.66E-26	NK/T
SYNE2	1.89E-29	0.21339401	0.891	0.71	3.77E-26	NK/T
AREG	4.54E-29	1.05510322	0.42	0.184	9.09E-26	NK/T
TNFAIP3	7.93E-29	1.03928295	0.556	0.292	1.59E-25	NK/T
HIST1H4C	4.73E-27	0.46653523	0.691	0.601	9.46E-24	NK/T
LAPTM5	9.05E-25	0.26161307	0.665	0.486	1.81E-21	NK/T
SLA	1.60E-24	0.87791711	0.451	0.353	3.19E-21	NK/T
GIMAP4	2.02E-24	0.46427068	0.646	0.456	4.03E-21	NK/T
CCL4	8.75E-22	1.22549746	0.525	0.221	1.75E-18	NK/T
DOK2	1.54E-21	0.68252866	0.471	0.379	3.08E-18	NK/T
CD27	1.44E-20	1.03756364	0.432	0.183	2.88E-17	NK/T
CEMIP2	7.16E-20	0.81448699	0.423	0.237	1.43E-16	NK/T
NABP1	2.99E-16	0.49150254	0.669	0.539	5.98E-13	NK/T
GLRX	1.22E-15	0.27414701	0.65	0.423	2.44E-12	NK/T
MT1X	3.60E-15	0.4497865	0.615	0.407	7.20E-12	NK/T
VAMP8	1.43E-14	0.27182274	0.652	0.476	2.85E-11	NK/T
RGCC	5.57E-13	0.86319818	0.52	0.167	1.11E-09	NK/T
SLAMF1	1.25E-11	0.81542856	0.436	0.136	2.51E-08	NK/T
FAM49B	3.54E-09	0.67471603	0.564	0.37	7.07E-06	NK/T
FKBP11	6.18E-09	0.69254882	0.508	0.228	1.24E-05	NK/T
JAKMIP2	7.94E-07	0.21181278	0.438	0.177	0.00158739	NK/T
PHLDA1	2.97E-06	0.37310498	0.433	0.251	0.00593799	NK/T
CST7	4.30E-06	1.26687238	0.493	0.171	0.00860378	NK/T
PCNA	6.24E-06	0.26926294	0.537	0.286	0.01247832	NK/T
MIR155HG	1.94E-05	0.25666954	0.413	0.16	0.03889644	NK/T
GZMK	0.00812976	1.58624457	0.48	0.145	1	NK/T
TYROBP	0	3.59676165	0.945	0.597	0	Mono/Mac
S100A9	0	3.51853101	0.818	0.442	0	Mono/Mac
LYZ	0	3.47260938	0.887	0.54	0	Mono/Mac
AIF1	0	3.38320285	0.889	0.519	0	Mono/Mac
CTSS	0	3.35252634	0.951	0.588	0	Mono/Mac
C1QA	0	3.34936706	0.815	0.494	0	Mono/Mac
FCER1G	0	3.33486624	0.916	0.504	0	Mono/Mac
C1QB	0	3.2326028	0.778	0.456	0	Mono/Mac
SPP1	0	3.19101376	0.666	0.516	0	Mono/Mac

CTSB	0	3.08513473	0.954	0.666	0	Mono/Mac
CYBB	0	3.02651598	0.899	0.511	0	Mono/Mac
S100A8	0	2.94901022	0.61	0.434	0	Mono/Mac
MS4A6A	0	2.92752546	0.879	0.415	0	Mono/Mac
FTL	0	2.89259406	0.999	0.996	0	Mono/Mac
HLA-DRA	0	2.88055521	0.954	0.614	0	Mono/Mac
CD14	0	2.8428636	0.858	0.475	0	Mono/Mac
CD74	0	2.81266244	0.971	0.622	0	Mono/Mac
LGMN	0	2.76359527	0.841	0.607	0	Mono/Mac
CTSD	0	2.71430209	0.944	0.713	0	Mono/Mac
SAT1	0	2.70480507	0.98	0.691	0	Mono/Mac
IGSF6	0	2.68318743	0.848	0.498	0	Mono/Mac
RNASE1	0	2.67163416	0.778	0.49	0	Mono/Mac
C1QC	0	2.66204188	0.714	0.345	0	Mono/Mac
TYMP	0	2.57723717	0.944	0.646	0	Mono/Mac
CCL3	0	2.56511949	0.711	0.316	0	Mono/Mac
HLA-DPA1	0	2.51933266	0.867	0.362	0	Mono/Mac
GPX1	0	2.51741994	0.902	0.594	0	Mono/Mac
MNDA	0	2.46917707	0.835	0.489	0	Mono/Mac
CD163	0	2.44427508	0.844	0.323	0	Mono/Mac
FCN1	0	2.43004916	0.785	0.52	0	Mono/Mac
LAPTM5	0	2.38516158	0.89	0.406	0	Mono/Mac
CSF1R	0	2.38305151	0.824	0.427	0	Mono/Mac
ITGB2	0	2.34077197	0.89	0.443	0	Mono/Mac
HLA-DRB1	0	2.33609934	0.89	0.428	0	Mono/Mac
LST1	0	2.30385657	0.845	0.503	0	Mono/Mac
HMOX1	0	2.26031672	0.782	0.374	0	Mono/Mac
CTSZ	0	2.25464993	0.92	0.625	0	Mono/Mac
HLA-DPB1	0	2.25265121	0.852	0.4	0	Mono/Mac
SPI1	0	2.24106401	0.806	0.439	0	Mono/Mac
MS4A7	0	2.23574332	0.796	0.325	0	Mono/Mac
ADAP2	0	2.19753614	0.856	0.533	0	Mono/Mac
STAB1	0	2.18900846	0.728	0.304	0	Mono/Mac
FOLR2	0	2.17754745	0.667	0.365	0	Mono/Mac
PLEK	0	2.15643874	0.789	0.357	0	Mono/Mac
MSR1	0	2.15277309	0.768	0.371	0	Mono/Mac
MRC1	0	2.13177315	0.651	0.262	0	Mono/Mac
SRGN	0	2.12606933	0.878	0.409	0	Mono/Mac
VMP1	0	2.07933254	0.914	0.5	0	Mono/Mac
ADA2	0	2.07478703	0.819	0.427	0	Mono/Mac
MAFB	0	2.07465097	0.906	0.593	0	Mono/Mac
SIGLEC1	0	2.05932964	0.801	0.461	0	Mono/Mac
HLA-DRB5	0	2.05409119	0.821	0.349	0	Mono/Mac
SH3BGRL3	0	2.04972004	0.934	0.783	0	Mono/Mac
VSIG4	0	2.0406008	0.724	0.272	0	Mono/Mac
RNASET2	0	2.03203121	0.853	0.442	0	Mono/Mac
MARCKS	0	2.01787506	0.957	0.781	0	Mono/Mac
COTL1	0	2.00944937	0.814	0.381	0	Mono/Mac
ASAH1	0	2.00277308	0.885	0.595	0	Mono/Mac
VAMP8	0	1.97631477	0.786	0.42	0	Mono/Mac
1-Mar	0	1.96701081	0.792	0.419	0	Mono/Mac
CCL3L1	0	1.96389565	0.587	0.352	0	Mono/Mac
LILRB2	0	1.94724548	0.803	0.457	0	Mono/Mac

LGALS9	0	1.94436386	0.853	0.513	0	Mono/Mac
MAF	0	1.94396054	0.831	0.542	0	Mono/Mac
HLA-DQB1	0	1.93425514	0.787	0.283	0	Mono/Mac
MPEG1	0	1.93051308	0.754	0.417	0	Mono/Mac
FCGR3A	0	1.92906411	0.683	0.386	0	Mono/Mac
LCP1	0	1.91267809	0.915	0.527	0	Mono/Mac
CYBA	0	1.88726162	0.931	0.717	0	Mono/Mac
F13A1	0	1.88338527	0.461	0.254	0	Mono/Mac
HLA-DMA	0	1.86961782	0.806	0.309	0	Mono/Mac
C1orf162	0	1.86371326	0.747	0.406	0	Mono/Mac
AC020656.1	0	1.85180677	0.753	0.479	0	Mono/Mac
FYB1	0	1.85138013	0.873	0.418	0	Mono/Mac
NCKAP1L	0	1.80775339	0.813	0.419	0	Mono/Mac
HLA-DMB	0	1.80657	0.793	0.378	0	Mono/Mac
HLA-DQA1	0	1.77754364	0.689	0.277	0	Mono/Mac
UCP2	0	1.74627041	0.819	0.445	0	Mono/Mac
GLUL	0	1.73603628	0.885	0.549	0	Mono/Mac
LYN	0	1.72279761	0.775	0.438	0	Mono/Mac
POU2F2	0	1.71784497	0.736	0.402	0	Mono/Mac
CTSC	0	1.7139265	0.867	0.5	0	Mono/Mac
EFHD2	0	1.70834824	0.839	0.486	0	Mono/Mac
C5AR1	0	1.70720226	0.674	0.283	0	Mono/Mac
CD84	0	1.70391339	0.733	0.376	0	Mono/Mac
ARRB2	0	1.69629832	0.781	0.375	0	Mono/Mac
C3AR1	0	1.68998493	0.676	0.314	0	Mono/Mac
IFI30	0	1.68989634	0.74	0.313	0	Mono/Mac
CPM	0	1.68874809	0.657	0.197	0	Mono/Mac
DAB2	0	1.68539255	0.757	0.487	0	Mono/Mac
SERPINA1	0	1.65239802	0.693	0.364	0	Mono/Mac
NCF2	0	1.64667917	0.753	0.407	0	Mono/Mac
CSTB	0	1.64563649	0.817	0.636	0	Mono/Mac
CPVL	0	1.62635111	0.702	0.341	0	Mono/Mac
PHACTR1	0	1.62601305	0.601	0.29	0	Mono/Mac
CLEC7A	0	1.62423545	0.702	0.36	0	Mono/Mac
PLD3	0	1.61608737	0.857	0.58	0	Mono/Mac
HLA-DRB6	0	1.59859196	0.796	0.336	0	Mono/Mac
LAIR1	0	1.58881091	0.721	0.375	0	Mono/Mac
CCR1	0	1.56406106	0.74	0.428	0	Mono/Mac
FCGR2A	0	1.5551514	0.65	0.248	0	Mono/Mac
IL1RN	0	1.55500928	0.622	0.44	0	Mono/Mac
GPNMB	0	1.53904222	0.802	0.638	0	Mono/Mac
MS4A4A	0	1.53879068	0.644	0.273	0	Mono/Mac
HCST	0	1.510162	0.835	0.438	0	Mono/Mac
MFSD1	0	1.4995631	0.732	0.335	0	Mono/Mac
SGK1	0	1.499445	0.639	0.297	0	Mono/Mac
RNASE6	0	1.48483584	0.599	0.347	0	Mono/Mac
IQGAP2	0	1.48480556	0.683	0.308	0	Mono/Mac
BLVRB	0	1.4821068	0.829	0.689	0	Mono/Mac
CD53	0	1.47774594	0.737	0.362	0	Mono/Mac
CD4	0	1.45645241	0.669	0.353	0	Mono/Mac
MYO1F	0	1.44248181	0.782	0.461	0	Mono/Mac
SYK	0	1.44066136	0.686	0.386	0	Mono/Mac
RBM47	0	1.43421044	0.633	0.306	0	Mono/Mac

DUSP6	0	1.43285432	0.708	0.359	0	Mono/Mac
FGL2	0	1.43083163	0.753	0.469	0	Mono/Mac
FABP5	0	1.42988084	0.76	0.6	0	Mono/Mac
CD83	0	1.42939949	0.595	0.251	0	Mono/Mac
NPL	0	1.42249443	0.706	0.369	0	Mono/Mac
PTPRE	0	1.41319127	0.716	0.355	0	Mono/Mac
SAMSN1	0	1.40738764	0.706	0.345	0	Mono/Mac
LILRB4	0	1.40490699	0.691	0.359	0	Mono/Mac
IL4I1	0	1.40476879	0.696	0.474	0	Mono/Mac
TNFSF13B	0	1.396596	0.756	0.436	0	Mono/Mac
LIPA	0	1.39345956	0.642	0.383	0	Mono/Mac
HAVCR2	0	1.38939112	0.669	0.389	0	Mono/Mac
TMIGD3	0	1.38312837	0.58	0.37	0	Mono/Mac
TNFRSF1B	0	1.37952107	0.784	0.457	0	Mono/Mac
CYTH4	0	1.37662398	0.716	0.356	0	Mono/Mac
CD36	0	1.36830731	0.685	0.399	0	Mono/Mac
APOBEC3A	0	1.35408999	0.585	0.353	0	Mono/Mac
HLA-B	0	1.35347975	0.959	0.834	0	Mono/Mac
TNFAIP2	0	1.34836477	0.793	0.487	0	Mono/Mac
SHTN1	0	1.32092938	0.684	0.393	0	Mono/Mac
MTSS1	0	1.31919441	0.618	0.435	0	Mono/Mac
CTSH	0	1.31157911	0.768	0.421	0	Mono/Mac
SLC15A3	0	1.31045281	0.733	0.435	0	Mono/Mac
NABP1	0	1.30722782	0.797	0.49	0	Mono/Mac
CTSL	0	1.30057988	0.796	0.6	0	Mono/Mac
BCL2A1	0	1.29413687	0.637	0.359	0	Mono/Mac
ARHGAP18	0	1.28997364	0.665	0.304	0	Mono/Mac
GMFG	0	1.28909398	0.746	0.38	0	Mono/Mac
HCLS1	0	1.27810672	0.736	0.297	0	Mono/Mac
IL10RA	0	1.25919875	0.67	0.339	0	Mono/Mac
HCK	0	1.25913627	0.611	0.279	0	Mono/Mac
LILRB1	0	1.25104676	0.673	0.415	0	Mono/Mac
RGS2	0	1.24562875	0.567	0.209	0	Mono/Mac
ACP5	0	1.24434351	0.587	0.412	0	Mono/Mac
SNX10	0	1.24191089	0.658	0.364	0	Mono/Mac
CLEC12A	0	1.24042885	0.669	0.368	0	Mono/Mac
LSP1	0	1.23886873	0.839	0.522	0	Mono/Mac
TFEC	0	1.23352986	0.657	0.349	0	Mono/Mac
LRRC25	0	1.23031323	0.629	0.324	0	Mono/Mac
CXCL16	0	1.22847209	0.587	0.295	0	Mono/Mac
FPR3	0	1.21803489	0.58	0.319	0	Mono/Mac
PTPN6	0	1.21103961	0.704	0.388	0	Mono/Mac
SLC7A7	0	1.21079113	0.604	0.329	0	Mono/Mac
EVI2B	0	1.21023111	0.619	0.297	0	Mono/Mac
NCF4	0	1.206284	0.584	0.304	0	Mono/Mac
CD86	0	1.20262708	0.559	0.353	0	Mono/Mac
TCN2	0	1.20134342	0.659	0.236	0	Mono/Mac
FERMT3	0	1.18701368	0.672	0.288	0	Mono/Mac
TREM2	0	1.18384964	0.526	0.287	0	Mono/Mac
OGFRL1	0	1.17629412	0.609	0.337	0	Mono/Mac
SLC11A1	0	1.17627072	0.483	0.315	0	Mono/Mac
GPSM3	0	1.17333418	0.687	0.34	0	Mono/Mac
SOD2	0	1.17189612	0.7	0.457	0	Mono/Mac

KCTD12	0	1.16774449	0.832	0.601	0	Mono/Mac
PTPRC	0	1.15702594	0.885	0.477	0	Mono/Mac
GM2A	0	1.15253015	0.692	0.441	0	Mono/Mac
IFIT2	0	1.14448071	0.695	0.557	0	Mono/Mac
MARCO	0	1.14339225	0.545	0.277	0	Mono/Mac
MPP1	0	1.1420712	0.639	0.407	0	Mono/Mac
RASGEF1B	0	1.13330679	0.521	0.256	0	Mono/Mac
GBP1	0	1.13233617	0.713	0.523	0	Mono/Mac
PLAUR	0	1.12833595	0.564	0.291	0	Mono/Mac
PLIN2	0	1.11052355	0.602	0.34	0	Mono/Mac
PIK3AP1	0	1.10692358	0.597	0.412	0	Mono/Mac
PTAFR	0	1.09693524	0.623	0.385	0	Mono/Mac
EPSTI1	0	1.09362497	0.755	0.575	0	Mono/Mac
OAS1	0	1.08151172	0.74	0.558	0	Mono/Mac
DOK2	0	1.06383763	0.608	0.332	0	Mono/Mac
SLA	0	1.06078475	0.609	0.301	0	Mono/Mac
WAS	0	1.05339702	0.618	0.331	0	Mono/Mac
FAM49B	0	1.05118652	0.645	0.326	0	Mono/Mac
MX2	0	1.04831281	0.715	0.524	0	Mono/Mac
CD37	0	1.04325066	0.674	0.368	0	Mono/Mac
DHRS9	0	1.03954878	0.635	0.329	0	Mono/Mac
C15orf48	0	1.030623	0.44	0.233	0	Mono/Mac
IL18	0	1.024682	0.496	0.265	0	Mono/Mac
THEMIS2	0	1.01816475	0.498	0.27	0	Mono/Mac
RGS10	0	1.01723831	0.65	0.419	0	Mono/Mac
NR4A2	0	1.01347323	0.592	0.317	0	Mono/Mac
LY86	0	1.01307728	0.583	0.277	0	Mono/Mac
CXCL11	0	1.01030875	0.513	0.369	0	Mono/Mac
ITGAX	0	1.00994738	0.607	0.388	0	Mono/Mac
GPR65	0	1.0095596	0.702	0.369	0	Mono/Mac
TLR2	0	1.00858954	0.483	0.313	0	Mono/Mac
ATF5	0	0.99734806	0.666	0.385	0	Mono/Mac
CAPG	0	0.99242819	0.618	0.47	0	Mono/Mac
ACSL1	0	0.97036393	0.49	0.26	0	Mono/Mac
GLRX	0	0.95283749	0.668	0.392	0	Mono/Mac
C2	0	0.95104064	0.399	0.214	0	Mono/Mac
CSTA	0	0.94183777	0.592	0.319	0	Mono/Mac
RNASE2	0	0.93743462	0.604	0.278	0	Mono/Mac
CIITA	0	0.92327141	0.601	0.424	0	Mono/Mac
LCP2	0	0.92308437	0.583	0.364	0	Mono/Mac
CXCR4	0	0.92032688	0.519	0.316	0	Mono/Mac
P2RY13	0	0.91671967	0.467	0.297	0	Mono/Mac
CLEC10A	0	0.91463684	0.433	0.268	0	Mono/Mac
CSF2RA	0	0.90079013	0.45	0.243	0	Mono/Mac
PILRA	0	0.89188758	0.605	0.314	0	Mono/Mac
CLEC2B	0	0.88990665	0.725	0.412	0	Mono/Mac
IER3	0	0.88990443	0.65	0.405	0	Mono/Mac
SLC16A3	0	0.88897929	0.597	0.43	0	Mono/Mac
GPR183	0	0.88086212	0.472	0.291	0	Mono/Mac
NRP2	0	0.86945565	0.636	0.357	0	Mono/Mac
BIRC3	0	0.8680799	0.597	0.308	0	Mono/Mac
FUCA1	0	0.86497869	0.493	0.332	0	Mono/Mac
STAT1	0	0.86427403	0.796	0.623	0	Mono/Mac

GPR34	0	0.84773575	0.429	0.252	0	Mono/Mac
FBP1	0	0.84225313	0.52	0.342	0	Mono/Mac
ARHGAP30	0	0.8403471	0.68	0.407	0	Mono/Mac
PLA2G7	0	0.83295683	0.536	0.33	0	Mono/Mac
NFKBIA	0	0.83146357	0.761	0.588	0	Mono/Mac
P2RX7	0	0.8296475	0.575	0.395	0	Mono/Mac
KYNU	0	0.82124392	0.546	0.252	0	Mono/Mac
IFI6	0	0.82114636	0.908	0.809	0	Mono/Mac
SLCO2B1	0	0.81852495	0.467	0.267	0	Mono/Mac
CD180	0	0.81666986	0.611	0.367	0	Mono/Mac
CORO1A	0	0.80579146	0.764	0.42	0	Mono/Mac
NLRP3	0	0.79889863	0.456	0.284	0	Mono/Mac
TRIB1	0	0.79789487	0.522	0.368	0	Mono/Mac
IFI44L	0	0.79294065	0.807	0.669	0	Mono/Mac
GIMAP4	0	0.78843855	0.724	0.414	0	Mono/Mac
OAS2	0	0.77708588	0.696	0.485	0	Mono/Mac
WARS	0	0.76570647	0.638	0.482	0	Mono/Mac
IFIT3	0	0.76456284	0.723	0.632	0	Mono/Mac
ATP1B1	0	0.74685141	0.544	0.256	0	Mono/Mac
ITPR2	0	0.74237221	0.629	0.354	0	Mono/Mac
MX1	0	0.71864859	0.774	0.638	0	Mono/Mac
CXorf21	0	0.71648929	0.526	0.3	0	Mono/Mac
CDKN1A	0	0.70668406	0.513	0.286	0	Mono/Mac
SMIM25	0	0.69089426	0.475	0.248	0	Mono/Mac
PRDM1	0	0.67806871	0.441	0.252	0	Mono/Mac
ANPEP	0	0.67590947	0.466	0.275	0	Mono/Mac
CCRL2	0	0.66747458	0.515	0.335	0	Mono/Mac
CD300E	0	0.66169815	0.527	0.291	0	Mono/Mac
EHD4	0	0.6595712	0.613	0.349	0	Mono/Mac
TGFBI	0	0.63644601	0.72	0.597	0	Mono/Mac
OAS3	0	0.62729637	0.681	0.574	0	Mono/Mac
GAS7	0	0.59672917	0.643	0.509	0	Mono/Mac
HLA-DQA2	0	0.59382384	0.528	0.263	0	Mono/Mac
FFAR2	0	0.58830618	0.507	0.304	0	Mono/Mac
HHEX	0	0.58629948	0.494	0.261	0	Mono/Mac
S100A12	0	0.5696883	0.396	0.182	0	Mono/Mac
CXCL9	0	0.56529036	0.469	0.332	0	Mono/Mac
HMGA1	0	0.56291673	0.532	0.349	0	Mono/Mac
NAPSB	0	0.55907521	0.495	0.33	0	Mono/Mac
LILRA5	0	0.54701683	0.459	0.283	0	Mono/Mac
LGALS2	0	0.54319642	0.521	0.26	0	Mono/Mac
SUCNR1	0	0.53367261	0.517	0.274	0	Mono/Mac
LMO2	0	0.52918146	0.659	0.402	0	Mono/Mac
IKZF1	0	0.51790961	0.627	0.351	0	Mono/Mac
OASL	0	0.50822015	0.584	0.385	0	Mono/Mac
IFIH1	0	0.5067322	0.613	0.466	0	Mono/Mac
CCL8	0	0.49846099	0.405	0.225	0	Mono/Mac
ITGA4	0	0.49643433	0.605	0.424	0	Mono/Mac
CD48	0	0.49158472	0.635	0.296	0	Mono/Mac
SLC6A6	0	0.47737092	0.511	0.382	0	Mono/Mac
MCTP1	0	0.46937108	0.565	0.357	0	Mono/Mac
MBP	0	0.42969528	0.658	0.409	0	Mono/Mac
HERC5	0	0.42968952	0.584	0.368	0	Mono/Mac

CD72	0	0.4247746	0.449	0.314	0	Mono/Mac
BST2	0	0.40577237	0.851	0.694	0	Mono/Mac
CXCR2P1	0	0.3870729	0.51	0.235	0	Mono/Mac
CD38	0	0.37431664	0.529	0.312	0	Mono/Mac
RAD51AP1	0	0.37220454	0.525	0.301	0	Mono/Mac
GBP5	0	0.36708506	0.526	0.361	0	Mono/Mac
CLECL1	0	0.36424292	0.509	0.281	0	Mono/Mac
GIMAP1	0	0.32609409	0.551	0.299	0	Mono/Mac
CD52	0	0.32300575	0.676	0.45	0	Mono/Mac
AQP9	0	0.31927244	0.426	0.169	0	Mono/Mac
GADD45G	0	0.31570686	0.579	0.361	0	Mono/Mac
KIF20B	0	0.30257947	0.502	0.377	0	Mono/Mac
CYSLTR1	0	0.2598813	0.471	0.335	0	Mono/Mac
METTL7B	0	0.24214144	0.476	0.219	0	Mono/Mac
PKIB	0	0.23645135	0.38	0.133	0	Mono/Mac
GPR84	0	0.22996143	0.341	0.151	0	Mono/Mac
HAMP	0	0.20338817	0.43	0.163	0	Mono/Mac
TNFRSF11A	3.81E-306	0.45495135	0.391	0.23	7.63E-303	Mono/Mac
SLC1A3	2.65E-296	0.83239763	0.422	0.237	5.30E-293	Mono/Mac
FPR1	6.65E-296	1.11417816	0.468	0.304	1.33E-292	Mono/Mac
CXCL10	1.22E-291	1.97578461	0.567	0.516	2.45E-288	Mono/Mac
CCL2	5.42E-288	0.42885234	0.494	0.315	1.08E-284	Mono/Mac
IFIT1	1.98E-279	0.77871166	0.69	0.626	3.97E-276	Mono/Mac
ADGRE2	2.78E-270	0.65480308	0.413	0.27	5.56E-267	Mono/Mac
RAB42	8.13E-269	0.36777583	0.479	0.362	1.63E-265	Mono/Mac
BRCA2	2.89E-266	0.44859351	0.476	0.393	5.79E-263	Mono/Mac
PRR11	6.18E-262	0.21410115	0.425	0.33	1.24E-258	Mono/Mac
SDF2L1	7.26E-260	0.54504262	0.631	0.481	1.45E-256	Mono/Mac
BCAT1	3.15E-257	0.90416387	0.489	0.373	6.30E-254	Mono/Mac
SLAMF7	4.40E-255	0.40327078	0.483	0.358	8.79E-252	Mono/Mac
CXCL3	1.31E-247	0.96619222	0.327	0.145	2.63E-244	Mono/Mac
ISG15	8.04E-242	0.78966908	0.897	0.832	1.61E-238	Mono/Mac
RSAD2	1.71E-232	0.67727972	0.61	0.539	3.43E-229	Mono/Mac
RGS1	1.13E-229	0.80908376	0.429	0.275	2.27E-226	Mono/Mac
BASP1	2.01E-229	0.45960341	0.529	0.41	4.02E-226	Mono/Mac
CXCL2	5.43E-220	0.80783219	0.313	0.147	1.09E-216	Mono/Mac
HSPH1	1.12E-209	0.40443856	0.512	0.338	2.24E-206	Mono/Mac
PDK4	1.28E-209	0.58133084	0.469	0.305	2.55E-206	Mono/Mac
LYVE1	8.18E-202	1.34162181	0.344	0.229	1.64E-198	Mono/Mac
CCL4	2.13E-198	1.2629467	0.39	0.22	4.26E-195	Mono/Mac
SLAMF8	4.71E-196	0.59972125	0.369	0.228	9.41E-193	Mono/Mac
GBP4	1.35E-193	0.41270616	0.592	0.49	2.69E-190	Mono/Mac
ARL4C	5.24E-193	0.38908125	0.428	0.258	1.05E-189	Mono/Mac
GIMAP6	6.02E-189	0.2933149	0.506	0.395	1.20E-185	Mono/Mac
PNP	1.17E-187	0.30754065	0.345	0.204	2.34E-184	Mono/Mac
ALCAM	7.80E-184	0.66061208	0.499	0.392	1.56E-180	Mono/Mac
UPP1	2.58E-181	0.48712264	0.408	0.25	5.16E-178	Mono/Mac
FCGR1A	7.69E-180	0.72668145	0.397	0.254	1.54E-176	Mono/Mac
EIF4E	4.04E-177	0.4361396	0.449	0.319	8.08E-174	Mono/Mac
LPL	4.39E-171	0.34654772	0.32	0.199	8.78E-168	Mono/Mac
CYP27A1	7.11E-171	0.34520095	0.379	0.252	1.42E-167	Mono/Mac
TNFSF10	1.26E-166	0.36365329	0.709	0.604	2.51E-163	Mono/Mac
ADM	8.95E-166	0.34138844	0.404	0.27	1.79E-162	Mono/Mac

OTOA	3.38E-164	0.21366338	0.337	0.166	6.76E-161	Mono/Mac
IRF8	4.53E-162	0.68971303	0.398	0.277	9.06E-159	Mono/Mac
OTUD1	8.64E-158	0.48363724	0.437	0.328	1.73E-154	Mono/Mac
PLTP	2.89E-156	1.04255915	0.573	0.499	5.77E-153	Mono/Mac
ICAM1	6.65E-156	0.46944405	0.399	0.249	1.33E-152	Mono/Mac
SPINT2	3.80E-154	0.28147153	0.321	0.201	7.59E-151	Mono/Mac
CX3CR1	7.42E-154	0.26638551	0.411	0.31	1.48E-150	Mono/Mac
LGALS3	1.09E-150	1.00364027	0.858	0.863	2.19E-147	Mono/Mac
CREB5	5.84E-150	0.39340317	0.499	0.407	1.17E-146	Mono/Mac
MIR3945HG	1.66E-148	0.35811593	0.341	0.19	3.32E-145	Mono/Mac
TFRC	8.15E-148	0.66822289	0.407	0.286	1.63E-144	Mono/Mac
TIMP1	1.50E-142	0.29525017	0.867	0.805	3.01E-139	Mono/Mac
EGR2	5.78E-141	0.50472516	0.298	0.168	1.16E-137	Mono/Mac
AD000864.1	7.26E-141	0.42045331	0.337	0.199	1.45E-137	Mono/Mac
LILRB5	1.03E-136	1.31113779	0.403	0.319	2.06E-133	Mono/Mac
TNFAIP3	2.46E-134	0.65193941	0.417	0.297	4.91E-131	Mono/Mac
ANKRD22	1.18E-133	0.37538245	0.358	0.249	2.37E-130	Mono/Mac
HSPA6	2.11E-132	0.39596896	0.302	0.159	4.23E-129	Mono/Mac
ALOX5AP	1.27E-127	0.42162602	0.308	0.199	2.54E-124	Mono/Mac
NR4A3	1.32E-126	0.38687835	0.267	0.131	2.64E-123	Mono/Mac
IRF1	1.40E-119	0.28000963	0.669	0.528	2.79E-116	Mono/Mac
FGR	1.84E-116	0.87186307	0.416	0.321	3.68E-113	Mono/Mac
FNIP2	5.21E-107	0.64211801	0.478	0.398	1.04E-103	Mono/Mac
CEMIP2	1.38E-106	0.20217264	0.334	0.238	2.77E-103	Mono/Mac
TOP2A	2.52E-106	0.24014239	0.416	0.374	5.04E-103	Mono/Mac
HMGB2	5.03E-105	0.31296582	0.487	0.367	1.01E-101	Mono/Mac
GAS2L3	2.60E-100	0.52726858	0.41	0.36	5.19E-97	Mono/Mac
MT1H	2.71E-93	0.28808029	0.283	0.203	5.41E-90	Mono/Mac
INSIG1	8.19E-88	0.47036053	0.381	0.292	1.64E-84	Mono/Mac
TACC3	2.84E-86	0.37284525	0.347	0.276	5.69E-83	Mono/Mac
ARL4A	2.29E-85	0.28914449	0.463	0.36	4.57E-82	Mono/Mac
ZNF331	2.40E-80	0.41495655	0.365	0.272	4.80E-77	Mono/Mac
SDS	1.74E-79	0.59151653	0.356	0.288	3.48E-76	Mono/Mac
CSF2RB	3.82E-79	0.37187847	0.379	0.303	7.65E-76	Mono/Mac
CXCL8	3.93E-76	1.08928024	0.293	0.183	7.86E-73	Mono/Mac
PMAIP1	1.15E-74	0.50829971	0.344	0.242	2.31E-71	Mono/Mac
LUCAT1	5.45E-71	0.6959401	0.335	0.269	1.09E-67	Mono/Mac
RASGRP3	6.56E-66	0.24837837	0.407	0.371	1.31E-62	Mono/Mac
HPGDS	3.17E-65	0.32416527	0.34	0.284	6.34E-62	Mono/Mac
GK	2.75E-52	0.35969587	0.246	0.324	5.49E-49	Mono/Mac
GIMAP8	2.07E-42	0.31307067	0.436	0.405	4.13E-39	Mono/Mac
SCN9A	1.68E-40	0.72071915	0.26	0.338	3.36E-37	Mono/Mac
RNF144B	3.37E-37	0.58714537	0.342	0.289	6.74E-34	Mono/Mac
SELENOP	2.77E-35	1.4870445	0.604	0.66	5.55E-32	Mono/Mac
MDM2	4.60E-35	0.36842172	0.386	0.338	9.20E-32	Mono/Mac
LHFPL2	1.69E-33	0.54713446	0.385	0.339	3.39E-30	Mono/Mac
IL10	7.34E-32	0.65770861	0.28	0.199	1.47E-28	Mono/Mac
B3GNT5	3.48E-31	0.38692956	0.319	0.265	6.97E-28	Mono/Mac
IDO1	1.46E-29	0.25503339	0.306	0.285	2.92E-26	Mono/Mac
VMO1	2.88E-29	0.32301918	0.25	0.314	5.75E-26	Mono/Mac
RBP7	3.60E-29	0.2438149	0.297	0.222	7.20E-26	Mono/Mac
ABL2	1.36E-27	0.21940878	0.342	0.303	2.72E-24	Mono/Mac
IL1R2	5.17E-27	0.50918842	0.301	0.232	1.03E-23	Mono/Mac

ST6GAL1	5.62E-27	0.24385907	0.357	0.33	1.12E-23	Mono/Mac
MMP19	3.93E-19	0.22633744	0.278	0.253	7.85E-16	Mono/Mac
CCDC141	1.43E-13	0.59445863	0.33	0.326	2.86E-10	Mono/Mac
CMIP	2.55E-12	0.29004818	0.456	0.459	5.10E-09	Mono/Mac
C5AR2	4.05E-12	0.57656851	0.288	0.225	8.10E-09	Mono/Mac
MT1X	2.26E-11	0.37932944	0.452	0.425	4.52E-08	Mono/Mac
CD209	1.83E-09	0.62425867	0.27	0.246	3.66E-06	Mono/Mac
APOC1	3.60E-09	0.4555084	0.358	0.345	7.19E-06	Mono/Mac
FCGR2B	1.91E-08	0.53437909	0.299	0.301	3.82E-05	Mono/Mac
SLC40A1	5.77E-07	0.94297072	0.485	0.552	0.00115394	Mono/Mac
SERPINB9	4.61E-06	0.2623412	0.301	0.281	0.00921881	Mono/Mac
CLEC4E	5.89E-06	0.47349629	0.271	0.227	0.0117838	Mono/Mac
TTN	1.31E-05	1.85978001	0.425	0.468	0.02617348	Mono/Mac
ACKR1	0	4.56839791	0.699	0.358	0	EC
VWF	0	4.15349464	0.886	0.308	0	EC
ID1	0	4.0616179	0.893	0.3	0	EC
CLDN5	0	3.77956366	0.857	0.326	0	EC
AQP1	0	3.63379839	0.805	0.385	0	EC
PECAM1	0	3.23183782	0.923	0.407	0	EC
FABP4	0	3.0375655	0.808	0.542	0	EC
EGFL7	0	3.03195854	0.89	0.309	0	EC
IFI27	0	2.97253325	0.904	0.659	0	EC
PLVAP	0	2.83030164	0.638	0.235	0	EC
EMCN	0	2.72073781	0.802	0.269	0	EC
RAMP3	0	2.6815096	0.768	0.303	0	EC
SOX18	0	2.62815397	0.822	0.288	0	EC
ADGRL4	0	2.54727598	0.731	0.263	0	EC
NPDC1	0	2.43685299	0.817	0.442	0	EC
RAMP2	0	2.42290793	0.826	0.343	0	EC
GNG11	0	2.35067334	0.886	0.575	0	EC
MMRN1	0	2.33475783	0.551	0.255	0	EC
ENG	0	2.29128593	0.809	0.395	0	EC
SLC9A3R2	0	2.2432412	0.731	0.384	0	EC
TM4SF1	0	2.20593124	0.833	0.382	0	EC
FLT1	0	2.18559945	0.704	0.257	0	EC
ECSCR	0	2.17441731	0.772	0.237	0	EC
CLEC14A	0	2.16496331	0.693	0.263	0	EC
PALMD	0	2.15932409	0.733	0.332	0	EC
CDH5	0	2.15724224	0.782	0.265	0	EC
MMRN2	0	2.14585586	0.74	0.232	0	EC
CLU	0	2.13348012	0.887	0.613	0	EC
CD93	0	2.10418894	0.731	0.344	0	EC
A2M	0	2.0887433	0.887	0.692	0	EC
ITGA6	0	2.05826151	0.77	0.261	0	EC
PCAT19	0	2.04393886	0.739	0.243	0	EC
ELK3	0	2.02058388	0.837	0.461	0	EC
TGM2	0	2.00959437	0.747	0.326	0	EC
CYYR1	0	2.00654863	0.746	0.252	0	EC
PTPRB	0	1.99819221	0.708	0.31	0	EC
PODXL	0	1.9927221	0.564	0.286	0	EC
PRCP	0	1.97446536	0.834	0.417	0	EC
TNFSF10	0	1.96029364	0.885	0.595	0	EC
FAM110D	0	1.95147433	0.663	0.293	0	EC

NRN1	0	1.8854106	0.751	0.324	0	EC
CALCRL	0	1.87445078	0.7	0.392	0	EC
CAVIN2	0	1.82877017	0.622	0.305	0	EC
TSPAN7	0	1.82451901	0.652	0.22	0	EC
TFPI	0	1.8048221	0.692	0.42	0	EC
ID3	0	1.80241203	0.833	0.54	0	EC
LIFR	0	1.78983737	0.785	0.481	0	EC
LMO2	0	1.77244896	0.766	0.419	0	EC
TMEM88	0	1.76494776	0.621	0.192	0	EC
MECOM	0	1.76393656	0.669	0.214	0	EC
OLFM1	0	1.75961683	0.562	0.308	0	EC
ADAM15	0	1.74102279	0.761	0.408	0	EC
FABP5	0	1.72869142	0.855	0.606	0	EC
IGFBP4	0	1.71586645	0.882	0.57	0	EC
IL33	0	1.70540815	0.74	0.401	0	EC
DIPK2B	0	1.70532805	0.733	0.221	0	EC
JAM2	0	1.70322692	0.673	0.312	0	EC
POSTN	0	1.70137343	0.513	0.131	0	EC
S100A16	0	1.69585805	0.771	0.321	0	EC
EFNB2	0	1.68841915	0.573	0.184	0	EC
CCL14	0	1.64936831	0.546	0.253	0	EC
HEG1	0	1.64687063	0.72	0.341	0	EC
THSD7A	0	1.64223203	0.668	0.289	0	EC
NUAK1	0	1.63445625	0.646	0.317	0	EC
HYAL2	0	1.6139591	0.634	0.333	0	EC
GIMAP4	0	1.60143976	0.819	0.437	0	EC
IL6ST	0	1.59986368	0.862	0.576	0	EC
ICAM2	0	1.59921148	0.681	0.426	0	EC
ROBO4	0	1.59745492	0.722	0.225	0	EC
PIM3	0	1.59350326	0.71	0.242	0	EC
GIMAP1	0	1.58343125	0.772	0.302	0	EC
ADGRF5	0	1.56798938	0.686	0.404	0	EC
CD34	0	1.558969	0.804	0.448	0	EC
PKP4	0	1.55469842	0.658	0.341	0	EC
TM4SF18	0	1.54623496	0.656	0.249	0	EC
GIMAP7	0	1.54535258	0.791	0.454	0	EC
PROX1	0	1.54534075	0.491	0.227	0	EC
LDB2	0	1.54210284	0.732	0.447	0	EC
MRTFB	0	1.54150357	0.726	0.487	0	EC
KDR	0	1.53572585	0.626	0.292	0	EC
ETS2	0	1.5320885	0.731	0.375	0	EC
RALGAPA2	0	1.52763692	0.723	0.397	0	EC
MYCT1	0	1.52484727	0.711	0.299	0	EC
RASIP1	0	1.52297938	0.658	0.215	0	EC
TIE1	0	1.50196063	0.661	0.318	0	EC
HSPG2	0	1.50027699	0.869	0.67	0	EC
SELP	0	1.49484726	0.616	0.253	0	EC
SPTBN1	0	1.47966379	0.893	0.652	0	EC
EDN1	0	1.47768636	0.544	0.248	0	EC
DUSP23	0	1.47090679	0.674	0.398	0	EC
IL1R1	0	1.46803811	0.612	0.35	0	EC
THBD	0	1.46493241	0.611	0.237	0	EC
ITGB4	0	1.44304708	0.679	0.279	0	EC

SMAD1	0	1.44034722	0.719	0.359	0 EC
NOSTRIN	0	1.42364577	0.53	0.265	0 EC
EPAS1	0	1.42094902	0.82	0.556	0 EC
COL15A1	0	1.41369834	0.613	0.334	0 EC
ERG	0	1.41135645	0.63	0.267	0 EC
TJP1	0	1.40778261	0.801	0.497	0 EC
F8	0	1.38982639	0.521	0.18	0 EC
GRASP	0	1.38939899	0.624	0.243	0 EC
RDX	0	1.38841416	0.81	0.554	0 EC
PREX2	0	1.37309742	0.672	0.318	0 EC
KCTD12	0	1.35632786	0.819	0.628	0 EC
SLFN5	0	1.35508275	0.818	0.576	0 EC
SHE	0	1.35035618	0.697	0.187	0 EC
ADCY4	0	1.3409683	0.604	0.218	0 EC
SYNE2	0	1.33812325	0.887	0.712	0 EC
BST2	0	1.32736215	0.874	0.709	0 EC
SULF2	0	1.31369498	0.653	0.363	0 EC
TEK	0	1.30996578	0.669	0.256	0 EC
FLNB	0	1.30734048	0.69	0.451	0 EC
PPFIBP1	0	1.30626056	0.628	0.371	0 EC
DOCK9	0	1.30385137	0.682	0.342	0 EC
MCTP1	0	1.29135771	0.67	0.368	0 EC
PTPRM	0	1.28187449	0.659	0.172	0 EC
RBP7	0	1.26995776	0.497	0.207	0 EC
ZNF385D	0	1.26723305	0.617	0.405	0 EC
KANK3	0	1.2482406	0.647	0.349	0 EC
CAV1	0	1.24384849	0.916	0.738	0 EC
MALL	0	1.24101163	0.607	0.261	0 EC
EFNA1	0	1.23526437	0.564	0.236	0 EC
LMCD1	0	1.22256541	0.649	0.258	0 EC
SEMA6A	0	1.22010894	0.618	0.373	0 EC
PIK3R3	0	1.21877652	0.531	0.258	0 EC
GATA2	0	1.21865473	0.564	0.143	0 EC
LTC4S	0	1.2157016	0.588	0.265	0 EC
GIMAP6	0	1.20827318	0.705	0.384	0 EC
ECE1	0	1.19561504	0.75	0.454	0 EC
RAPGEF3	0	1.19269143	0.533	0.267	0 EC
MET	0	1.18718255	0.664	0.253	0 EC
HLA-B	0	1.18203554	0.979	0.846	0 EC
ABLIM1	0	1.17931469	0.751	0.383	0 EC
IFI44L	0	1.17281513	0.824	0.683	0 EC
C1orf115	0	1.16012316	0.668	0.317	0 EC
PGM5	0	1.15899351	0.669	0.392	0 EC
CD320	0	1.158887	0.649	0.342	0 EC
ADM5	0	1.14409203	0.509	0.194	0 EC
DSP	0	1.14302959	0.645	0.233	0 EC
IL3RA	0	1.13501455	0.577	0.219	0 EC
MTUS1	0	1.13424504	0.634	0.361	0 EC
DOC2B	0	1.12747713	0.573	0.212	0 EC
GIMAP8	0	1.11899512	0.662	0.381	0 EC
CMIP	0	1.11638359	0.667	0.434	0 EC
JCAD	0	1.11440663	0.672	0.322	0 EC
MARCKSL1	0	1.1099285	0.616	0.283	0 EC

SOX7	0	1.10405363	0.521	0.223	0	EC
NNMT	0	1.10241563	0.624	0.3	0	EC
S1PR1	0	1.0919261	0.54	0.229	0	EC
PLK2	0	1.05827295	0.62	0.334	0	EC
ARL4A	0	1.03008233	0.622	0.353	0	EC
JAG2	0	1.02952366	0.526	0.29	0	EC
CFI	0	1.02551512	0.561	0.245	0	EC
CRIP2	0	1.02308671	0.809	0.524	0	EC
BMX	0	1.01105841	0.563	0.254	0	EC
TMEM273	0	1.0087652	0.584	0.371	0	EC
ITGA5	0	1.00088628	0.59	0.315	0	EC
EPHB4	0	0.98800454	0.591	0.345	0	EC
PLEKHG1	0	0.98635519	0.571	0.271	0	EC
GPR146	0	0.98615989	0.596	0.314	0	EC
ARHGEF15	0	0.98418419	0.596	0.218	0	EC
FGD5	0	0.9730756	0.577	0.25	0	EC
TSPAN18	0	0.96808812	0.634	0.421	0	EC
CTNNAL1	0	0.96248409	0.644	0.264	0	EC
CPLX1	0	0.94074572	0.54	0.238	0	EC
ACVRL1	0	0.93924381	0.667	0.379	0	EC
IGFBP2	0	0.92509549	0.594	0.274	0	EC
NDRG1	0	0.91975406	0.694	0.425	0	EC
NOVA2	0	0.91720184	0.538	0.162	0	EC
LEPR	0	0.90910933	0.604	0.292	0	EC
SEMA6B	0	0.90900572	0.488	0.187	0	EC
AFAP1L1	0	0.90469024	0.565	0.156	0	EC
HLA-DRB6	0	0.89558764	0.643	0.406	0	EC
CRIM1	0	0.87936541	0.74	0.468	0	EC
MEOX1	0	0.87855884	0.53	0.237	0	EC
HLA-DRB1	0	0.8629047	0.733	0.499	0	EC
ENPP2	0	0.85732263	0.568	0.294	0	EC
EPHA4	0	0.85120437	0.501	0.237	0	EC
HHEX	0	0.8470644	0.607	0.274	0	EC
CD74	0	0.83118831	0.89	0.671	0	EC
TACR1	0	0.82574897	0.584	0.197	0	EC
OCIAD2	0	0.81501112	0.581	0.162	0	EC
FAM241A	0	0.80782803	0.566	0.268	0	EC
DIPK1B	0	0.80642626	0.573	0.279	0	EC
WARS	0	0.79656559	0.698	0.493	0	EC
APLNR	0	0.78748704	0.559	0.192	0	EC
LHX6	0	0.7829689	0.498	0.225	0	EC
ST8SIA6	0	0.78126131	0.542	0.238	0	EC
TLL1	0	0.75287177	0.504	0.177	0	EC
S100A10	0	0.74648031	0.876	0.676	0	EC
ST6GAL1	0	0.71145334	0.557	0.31	0	EC
RNASE1	0	0.70838438	0.842	0.515	0	EC
SPHK1	0	0.70247529	0.509	0.267	0	EC
ADGRG6	0	0.69236146	0.476	0.218	0	EC
MDK	0	0.67691797	0.689	0.512	0	EC
HMCN1	0	0.67651875	0.531	0.312	0	EC
TSHZ2	0	0.66481667	0.829	0.63	0	EC
MEOX2	0	0.66313033	0.656	0.433	0	EC
ARL15	0	0.64394047	0.514	0.266	0	EC

RSAD2	0	0.62959288	0.673	0.54	0	EC
CSF2RB	0	0.62957899	0.554	0.291	0	EC
BCAM	0	0.61723035	0.836	0.624	0	EC
SCN3B	0	0.5207986	0.501	0.138	0	EC
AL583785.1	0	0.51528528	0.518	0.248	0	EC
DACH1	0	0.50392204	0.533	0.164	0	EC
PDGFB	0	0.47062501	0.592	0.355	0	EC
PCDH19	0	0.45603403	0.474	0.21	0	EC
KLHL4	0	0.40986122	0.544	0.164	0	EC
RUNDC3B	0	0.36147258	0.49	0.246	0	EC
CLDN11	0	0.36049535	0.493	0.273	0	EC
CACNG8	0	0.26861971	0.468	0.135	0	EC
RAPGEF5	6.60E-308	0.98920243	0.561	0.332	1.32E-304	EC
CASKIN2	9.17E-302	0.97372694	0.53	0.291	1.83E-298	EC
HLA-DRA	5.63E-298	0.57252784	0.837	0.666	1.13E-294	EC
NDRG4	3.38E-290	0.5030813	0.474	0.236	6.76E-287	EC
SH3BGRL2	1.48E-287	0.77979269	0.486	0.263	2.96E-284	EC
PTPN14	6.36E-287	0.96367816	0.523	0.265	1.27E-283	EC
ACE	9.45E-287	0.86291291	0.54	0.341	1.89E-283	EC
FAM43A	1.82E-286	0.88639791	0.548	0.279	3.64E-283	EC
HSPA12B	2.66E-285	0.70216675	0.512	0.285	5.32E-282	EC
CARD10	8.27E-282	0.78717692	0.522	0.33	1.65E-278	EC
TSPAN13	2.64E-280	0.589157	0.45	0.215	5.28E-277	EC
PCDH17	4.73E-279	0.87881094	0.54	0.34	9.46E-276	EC
GBP4	3.72E-278	0.76298921	0.665	0.493	7.45E-275	EC
EHD4	1.08E-276	0.87315842	0.608	0.38	2.16E-273	EC
LXN	3.09E-272	0.37355672	0.462	0.233	6.19E-269	EC
FBLN2	1.17E-265	0.28226847	0.713	0.448	2.34E-262	EC
TGFBR3	2.89E-263	0.76819312	0.744	0.574	5.79E-260	EC
STMN1	6.17E-262	0.69213209	0.6	0.331	1.23E-258	EC
HAPLN3	1.24E-261	0.77119585	0.561	0.374	2.48E-258	EC
ADGRG1	7.01E-260	0.81283013	0.441	0.18	1.40E-256	EC
PLAT	3.86E-259	1.22721626	0.591	0.378	7.72E-256	EC
HLA-DRB5	7.32E-258	0.64631852	0.669	0.42	1.46E-254	EC
TSPAN5	6.88E-257	0.82715402	0.483	0.235	1.38E-253	EC
MX1	2.47E-255	0.84004571	0.781	0.653	4.95E-252	EC
LINC02185	5.94E-254	0.47079473	0.431	0.178	1.19E-250	EC
TMEM178A	9.15E-254	0.21769638	0.385	0.18	1.83E-250	EC
BCL3	3.09E-251	0.85821543	0.491	0.246	6.18E-248	EC
IFIH1	3.09E-249	0.4923602	0.646	0.479	6.18E-246	EC
ICA1	2.13E-245	0.85428115	0.484	0.253	4.25E-242	EC
CPXM2	8.73E-245	0.83992406	0.45	0.19	1.75E-241	EC
CAVIN1	2.08E-244	0.47275772	0.931	0.8	4.16E-241	EC
ICAM1	1.18E-242	0.94363485	0.502	0.254	2.35E-239	EC
ESAM	1.59E-242	1.04887197	0.635	0.485	3.18E-239	EC
NRP2	3.20E-241	1.28968354	0.599	0.393	6.40E-238	EC
ACTN1	6.06E-239	0.67046862	0.782	0.624	1.21E-235	EC
LIMS2	2.78E-238	0.87383069	0.661	0.487	5.56E-235	EC
SEC11C	3.12E-231	0.40334411	0.568	0.318	6.23E-228	EC
KRT7	2.83E-230	0.21410341	0.396	0.2	5.65E-227	EC
FNIP2	1.15E-228	0.64944157	0.593	0.394	2.29E-225	EC
SNCG	6.17E-226	0.49749772	0.776	0.637	1.23E-222	EC
ITM2A	1.70E-224	0.46281575	0.749	0.523	3.39E-221	EC

GABRA2	2.11E-222	0.33947003	0.433	0.256	4.22E-219	EC
RHOU	9.40E-221	0.87279087	0.522	0.322	1.88E-217	EC
SOCS3	1.91E-215	0.81612831	0.573	0.332	3.82E-212	EC
LRRC32	2.37E-208	0.99395027	0.625	0.481	4.75E-205	EC
DUSP6	2.70E-207	0.46721268	0.629	0.408	5.39E-204	EC
ADAMTS9	2.72E-205	1.26173162	0.493	0.281	5.44E-202	EC
STXBP6	3.67E-203	0.3111707	0.395	0.214	7.35E-200	EC
PRXL2A	3.70E-201	0.93635566	0.545	0.33	7.41E-198	EC
EMP1	5.69E-199	0.68992429	0.655	0.472	1.14E-195	EC
SHANK3	6.57E-199	0.83269948	0.436	0.203	1.31E-195	EC
MYO10	9.76E-198	0.65852981	0.462	0.279	1.95E-194	EC
TPD52L1	1.16E-195	0.62869775	0.442	0.224	2.32E-192	EC
CCDC71L	5.34E-195	0.30277583	0.47	0.258	1.07E-191	EC
BCL6B	1.01E-194	0.70750297	0.442	0.235	2.01E-191	EC
MYRIP	1.05E-194	0.57149005	0.451	0.253	2.11E-191	EC
FAM13C	3.84E-191	0.76378214	0.568	0.425	7.67E-188	EC
SLCO2A1	8.65E-185	1.02810576	0.441	0.227	1.73E-181	EC
HLA-DMA	4.72E-184	0.52813823	0.607	0.388	9.43E-181	EC
MYO5C	4.07E-179	0.71539564	0.444	0.226	8.14E-176	EC
CYSLTR1	1.17E-177	0.56129913	0.512	0.346	2.34E-174	EC
FAM107A	1.64E-172	0.74177794	0.37	0.132	3.27E-169	EC
SHROOM4	4.84E-171	0.84238569	0.456	0.227	9.69E-168	EC
IFIT3	6.19E-171	0.62486043	0.736	0.64	1.24E-167	EC
HLA-DPA1	2.21E-170	0.24482645	0.674	0.442	4.42E-167	EC
NTS	8.81E-170	1.45130502	0.432	0.159	1.76E-166	EC
HLA-DPB1	1.90E-169	0.33640158	0.678	0.472	3.80E-166	EC
LGALS3	1.11E-168	0.39964257	0.892	0.859	2.22E-165	EC
OAS3	5.31E-168	0.44893257	0.687	0.586	1.06E-164	EC
ASS1	2.75E-165	0.89342868	0.474	0.295	5.50E-162	EC
SLC6A6	4.52E-164	0.29597674	0.551	0.392	9.04E-161	EC
ADAMTS1	2.69E-163	0.97396861	0.608	0.447	5.38E-160	EC
GMFG	6.15E-162	0.24935995	0.657	0.432	1.23E-158	EC
TNFRSF4	1.04E-159	0.21491135	0.458	0.247	2.07E-156	EC
NOTCH4	3.06E-156	1.10201658	0.472	0.301	6.11E-153	EC
C1QTNF9	7.89E-156	0.30120496	0.371	0.107	1.58E-152	EC
TNFRSF1B	7.56E-155	0.35028747	0.679	0.507	1.51E-151	EC
HLA-DQA1	1.63E-153	0.48368559	0.538	0.341	3.25E-150	EC
CLEC2B	7.43E-153	0.40682934	0.63	0.458	1.49E-149	EC
PLIN5	9.08E-151	0.45077164	0.466	0.313	1.82E-147	EC
DLL4	1.49E-149	0.52910389	0.403	0.152	2.97E-146	EC
AC118754.1	2.27E-148	1.03119309	0.49	0.33	4.54E-145	EC
HERC5	6.90E-148	0.21995995	0.555	0.396	1.38E-144	EC
CRHBP	8.35E-144	0.5232017	0.4	0.203	1.67E-140	EC
F2R	1.98E-139	0.28551283	0.397	0.197	3.96E-136	EC
IFIT1	1.66E-138	0.35871234	0.708	0.631	3.33E-135	EC
SNAI1	1.97E-137	0.52683343	0.377	0.181	3.94E-134	EC
CHRM3	1.38E-134	0.25958972	0.4	0.186	2.75E-131	EC
SYNPO	5.08E-134	0.71875557	0.482	0.303	1.02E-130	EC
CTSH	8.23E-125	0.50703968	0.623	0.477	1.65E-121	EC
SOCS2	2.40E-124	0.86010416	0.433	0.242	4.80E-121	EC
AKR1C3	6.57E-121	0.72336947	0.408	0.216	1.31E-117	EC
CD300LG	2.72E-117	0.31094499	0.375	0.168	5.44E-114	EC
BMP4	3.94E-117	0.41563291	0.402	0.227	7.89E-114	EC

TMEM150C	2.92E-116	0.33837929	0.407	0.251	5.85E-113	EC
MAST4	2.52E-114	0.80740371	0.529	0.395	5.04E-111	EC
IFI6	4.36E-113	0.48986658	0.884	0.823	8.72E-110	EC
CADM3-AS1	6.78E-111	0.67134238	0.37	0.14	1.36E-107	EC
NFKBIA	7.93E-109	0.47463976	0.746	0.609	1.59E-105	EC
CXCL12	1.61E-108	0.97289673	0.643	0.511	3.23E-105	EC
CTSC	6.07E-105	0.26472982	0.719	0.559	1.21E-101	EC
ITPR2	6.02E-104	0.62343755	0.55	0.394	1.20E-100	EC
TMTC1	3.29E-103	0.96222277	0.472	0.327	6.58E-100	EC
MCF2L	9.04E-103	0.59232731	0.442	0.31	1.81E-99	EC
CFH	2.25E-102	0.23822009	0.607	0.414	4.51E-99	EC
GJC2	3.52E-102	0.33444256	0.423	0.293	7.04E-99	EC
RFLNB	4.80E-98	0.41148575	0.45	0.318	9.60E-95	EC
NOS3	2.16E-95	0.57047119	0.355	0.107	4.32E-92	EC
SLC2A3	7.72E-95	0.55876696	0.579	0.44	1.54E-91	EC
VWA1	1.99E-93	0.87459712	0.507	0.378	3.98E-90	EC
PDE2A	2.26E-93	0.85805005	0.467	0.339	4.52E-90	EC
PNP	5.69E-92	0.43217023	0.395	0.214	1.14E-88	EC
NES	9.07E-91	0.96580278	0.519	0.418	1.81E-87	EC
SOX17	2.66E-90	1.21559755	0.376	0.165	5.32E-87	EC
GPM6A	8.39E-90	0.61917603	0.426	0.294	1.68E-86	EC
CD36	9.92E-90	0.70968722	0.554	0.446	1.98E-86	EC
IER3	1.21E-89	0.65480126	0.594	0.44	2.42E-86	EC
HLA-DQA2	3.79E-87	0.6541851	0.445	0.303	7.58E-84	EC
AC004540.2	1.10E-86	0.21970876	0.382	0.19	2.20E-83	EC
ITGA1	3.37E-83	0.26800707	0.594	0.464	6.73E-80	EC
NR2F2	4.99E-83	0.29601096	0.791	0.726	9.98E-80	EC
RAB3C	8.30E-83	0.50843559	0.396	0.265	1.66E-79	EC
PTPRE	2.55E-81	0.33224172	0.563	0.414	5.11E-78	EC
CETP	2.57E-80	0.27341581	0.351	0.149	5.15E-77	EC
COL21A1	1.37E-78	0.26926459	0.462	0.352	2.74E-75	EC
CD9	1.95E-78	0.69046088	0.586	0.45	3.91E-75	EC
FAM167B	5.66E-78	0.65909035	0.403	0.274	1.13E-74	EC
OAS2	1.67E-75	0.3456004	0.615	0.518	3.35E-72	EC
VCAM1	2.78E-75	0.77638424	0.436	0.297	5.56E-72	EC
PCDH1	3.88E-75	0.68876108	0.494	0.397	7.75E-72	EC
UGCG	4.13E-75	0.52173289	0.477	0.341	8.25E-72	EC
FAM189A2	3.10E-73	0.4451533	0.274	0.287	6.19E-70	EC
AC007681.1	3.43E-69	0.23705328	0.376	0.199	6.85E-66	EC
LIMCH1	4.06E-69	0.6020102	0.406	0.261	8.11E-66	EC
ANGPT2	5.49E-69	0.60234623	0.384	0.513	1.10E-65	EC
NEDD9	7.26E-68	0.5461125	0.417	0.263	1.45E-64	EC
PLS3	9.47E-68	0.41889181	0.677	0.592	1.89E-64	EC
ENAH	2.93E-67	0.46600437	0.579	0.479	5.86E-64	EC
EDNRB	1.51E-65	0.71576833	0.379	0.237	3.03E-62	EC
CLIC5	6.03E-65	0.5335202	0.376	0.242	1.21E-61	EC
TPO	1.10E-64	0.4695346	0.376	0.178	2.19E-61	EC
FNBP1L	1.88E-64	0.68733811	0.465	0.354	3.77E-61	EC
KBTBD11	3.53E-64	0.2958136	0.46	0.358	7.07E-61	EC
SRGN	1.46E-63	0.22816058	0.664	0.487	2.91E-60	EC
GALNT15	5.93E-61	0.42684191	0.383	0.254	1.19E-57	EC
SLC12A2	1.61E-56	0.4994995	0.389	0.266	3.21E-53	EC
HES1	5.87E-56	0.73891285	0.54	0.437	1.17E-52	EC

EXOC3L2	8.33E-55	0.36036628	0.34	0.108	1.67E-51	EC
RPGR	4.85E-54	0.62669352	0.371	0.213	9.69E-51	EC
TNXB	1.43E-53	0.22966975	0.523	0.394	2.86E-50	EC
MLPH	7.55E-50	0.63709876	0.349	0.177	1.51E-46	EC
DEPP1	3.24E-49	0.79296872	0.418	0.293	6.48E-46	EC
NEURL1B	7.72E-47	0.41236984	0.57	0.515	1.54E-43	EC
CYB5D2	8.24E-47	0.20276393	0.362	0.243	1.65E-43	EC
GJA4	1.98E-45	0.81473217	0.589	0.534	3.96E-42	EC
GJA1	4.95E-45	0.7656483	0.397	0.297	9.91E-42	EC
C2CD4B	2.28E-44	0.88995128	0.354	0.222	4.56E-41	EC
SOX4	2.79E-43	0.37881915	0.724	0.656	5.58E-40	EC
TCN2	1.24E-42	0.3360003	0.437	0.31	2.47E-39	EC
MYH10	3.28E-42	0.29328897	0.497	0.394	6.55E-39	EC
CD79B	8.12E-41	0.36236593	0.458	0.384	1.62E-37	EC
CCL21	1.25E-40	3.27947987	0.243	0.298	2.50E-37	EC
CYTL1	4.10E-38	0.46619264	0.309	0.188	8.20E-35	EC
PCSK5	4.05E-37	0.40580536	0.417	0.341	8.09E-34	EC
TMEM100	1.55E-36	0.21200974	0.379	0.262	3.09E-33	EC
CNKSR3	1.78E-36	0.63774907	0.367	0.247	3.55E-33	EC
FOXC2	1.53E-35	0.27132081	0.272	0.346	3.07E-32	EC
CCL23	1.25E-34	0.36918418	0.291	0.159	2.50E-31	EC
ADM	4.17E-33	0.48833679	0.261	0.302	8.34E-30	EC
UPP1	3.22E-31	0.25627817	0.38	0.271	6.43E-28	EC
LYVE1	3.79E-30	0.26870937	0.335	0.243	7.57E-27	EC
NEBL	2.96E-26	0.28770535	0.337	0.17	5.93E-23	EC
CDH13	3.89E-26	0.41474818	0.351	0.262	7.79E-23	EC
BTNL9	3.98E-26	0.80370255	0.347	0.241	7.95E-23	EC
SYNJ2	2.10E-25	0.48876418	0.372	0.278	4.20E-22	EC
CYP1B1	5.76E-25	0.84775264	0.388	0.298	1.15E-21	EC
ITLN1	2.20E-24	0.42045165	0.3	0.207	4.40E-21	EC
RASGRP3	6.33E-24	0.52433389	0.425	0.373	1.27E-20	EC
TNFRSF10D	1.98E-23	0.38254908	0.284	0.131	3.96E-20	EC
SULF1	6.89E-23	0.87180078	0.399	0.317	1.38E-19	EC
FXYD6	1.79E-22	0.38653796	0.533	0.494	3.59E-19	EC
PLCXD3	1.11E-21	0.82131227	0.334	0.246	2.22E-18	EC
FSCN1	2.52E-20	0.44878422	0.448	0.372	5.04E-17	EC
TMEM47	9.09E-20	0.26649322	0.39	0.333	1.82E-16	EC
VEGFC	1.02E-19	0.53916366	0.337	0.19	2.05E-16	EC
BHLHE40	5.67E-18	0.4018059	0.395	0.302	1.13E-14	EC
AIF1L	6.41E-18	0.67889336	0.462	0.424	1.28E-14	EC
CDA	6.89E-18	0.28006285	0.266	0.224	1.38E-14	EC
LRG1	5.54E-17	0.69086234	0.347	0.228	1.11E-13	EC
UNC5B	2.05E-16	0.36124116	0.267	0.208	4.10E-13	EC
TBX1	6.71E-16	0.57356015	0.359	0.372	1.34E-12	EC
SEMA3G	1.04E-15	1.12873942	0.422	0.349	2.09E-12	EC
CGNL1	5.88E-15	0.30405915	0.384	0.292	1.18E-11	EC
KIF19	6.46E-15	0.2561144	0.284	0.147	1.29E-11	EC
SELE	2.96E-14	1.05434691	0.36	0.287	5.92E-11	EC
HEY1	5.92E-14	0.63470853	0.328	0.242	1.18E-10	EC
DDIT4	7.53E-13	0.26524793	0.461	0.394	1.51E-09	EC
COL8A1	3.93E-11	0.46297108	0.312	0.233	7.85E-08	EC
NR2F1	5.91E-11	0.9686558	0.424	0.442	1.18E-07	EC
PDGFD	1.82E-10	0.26785162	0.328	0.264	3.65E-07	EC

TCIM	3.11E-10	0.64520121	0.416	0.392	6.22E-07	EC
ADAMTS6	3.18E-09	0.38984976	0.397	0.35	6.37E-06	EC
LDLR	6.76E-09	0.2254807	0.333	0.348	1.35E-05	EC
HS3ST1	2.35E-08	0.38931663	0.375	0.279	4.70E-05	EC
PLA1A	2.99E-08	0.21298722	0.303	0.17	5.98E-05	EC
DNAJC18	3.28E-08	0.2631407	0.329	0.34	6.56E-05	EC
MMP28	4.39E-08	0.49019025	0.299	0.205	8.79E-05	EC
ABL2	7.49E-08	0.21577388	0.366	0.305	0.00014985	EC
IGFBP3	3.02E-07	0.32377364	0.424	0.387	0.00060462	EC
SLCO4A1	4.06E-07	0.47733241	0.292	0.206	0.00081155	EC
ANXA3	6.39E-07	0.52742873	0.303	0.194	0.00127718	EC
RCAN1	8.59E-07	0.32535771	0.321	0.25	0.00171862	EC
EFNB1	1.35E-06	0.38681466	0.335	0.352	0.00269717	EC
GPIHBP1	2.16E-06	0.63915274	0.328	0.231	0.00432169	EC
MRAS	5.81E-06	0.22769046	0.351	0.312	0.01161528	EC
PRSS23	7.75E-06	0.2414511	0.322	0.323	0.01549691	EC
ADRA2C	9.55E-05	0.26146229	0.315	0.268	0.19099055	EC
MPZL2	0.00018105	0.69491872	0.335	0.264	0.36209394	EC
CA4	0.00022934	0.3068745	0.291	0.201	0.45867596	EC
FN1	0.0002844	0.39443398	0.64	0.576	0.56880471	EC
INHBB	0.00034576	0.43008732	0.311	0.24	0.69151008	EC
FCN3	0.00035585	0.3167195	0.298	0.136	0.71170535	EC
DKK3	0.00036184	0.32635449	0.334	0.339	0.72367023	EC
KCNN3	0.00039986	0.66645017	0.345	0.25	0.79972456	EC
ITGA10	0.00045388	0.34263964	0.281	0.214	0.90776482	EC
PGM5-AS1	0.00058595	0.51394167	0.309	0.232	1	EC
APOLD1	0.00178096	0.31946609	0.329	0.275	1	EC
LCN6	0.00483558	0.5244253	0.285	0.211	1	EC
MYH11	0	3.91830341	0.973	0.627	0	SMC
ACTA2	0	3.69497324	0.993	0.762	0	SMC
TAGLN	0	3.55194477	0.997	0.804	0	SMC
TPM2	0	3.53306202	0.994	0.813	0	SMC
MYL9	0	3.39695261	0.986	0.762	0	SMC
RERGL	0	3.24126433	0.876	0.543	0	SMC
TPM1	0	3.15338239	0.982	0.685	0	SMC
C11orf96	0	3.07284894	0.954	0.437	0	SMC
AP002956.1	0	3.0558087	0.977	0.529	0	SMC
CARMN	0	3.04602538	0.942	0.504	0	SMC
ADIRF	0	2.98130843	0.97	0.832	0	SMC
DSTN	0	2.9476153	0.982	0.827	0	SMC
SORBS2	0	2.93093096	0.922	0.557	0	SMC
PPP1R14A	0	2.88554204	0.933	0.421	0	SMC
NOTCH3	0	2.87289637	0.974	0.591	0	SMC
MYLK	0	2.70736878	0.955	0.534	0	SMC
ACTG2	0	2.61804988	0.889	0.468	0	SMC
CALD1	0	2.55956145	0.992	0.824	0	SMC
CNN1	0	2.54429134	0.81	0.403	0	SMC
FLNA	0	2.53485458	0.987	0.793	0	SMC
NDUFA4L2	0	2.50001226	0.72	0.391	0	SMC
LMOD1	0	2.47767533	0.879	0.475	0	SMC
CSRP1	0	2.3638166	0.888	0.509	0	SMC
CRIP1	0	2.36029811	0.925	0.443	0	SMC
PLN	0	2.35216664	0.803	0.352	0	SMC

BCAM	0	2.31469433	0.93	0.545	0	SMC
NET1	0	2.2788608	0.846	0.452	0	SMC
HES4	0	2.24902252	0.901	0.441	0	SMC
TINAGL1	0	2.24387375	0.92	0.478	0	SMC
TBX2	0	2.23321932	0.821	0.458	0	SMC
SYNPO2	0	2.21089955	0.852	0.41	0	SMC
SNCG	0	2.12897361	0.843	0.583	0	SMC
CCND1	0	2.10804608	0.852	0.403	0	SMC
RRAD	0	2.10128744	0.788	0.311	0	SMC
PHLDA2	0	2.07059408	0.788	0.317	0	SMC
ITIH5	0	2.06543839	0.855	0.515	0	SMC
LPP	0	2.05782562	0.958	0.728	0	SMC
CKB	0	1.99250384	0.827	0.436	0	SMC
CAVIN3	0	1.97837394	0.949	0.648	0	SMC
MAP1B	0	1.96892797	0.875	0.454	0	SMC
PPP1R12B	0	1.94974218	0.848	0.524	0	SMC
C2orf40	0	1.93936047	0.654	0.451	0	SMC
CASQ2	0	1.9109143	0.771	0.486	0	SMC
MFGE8	0	1.87343386	0.915	0.614	0	SMC
KCNAB1	0	1.85598276	0.774	0.49	0	SMC
ISYNA1	0	1.84960362	0.843	0.445	0	SMC
COX4I2	0	1.84228194	0.758	0.498	0	SMC
GUCY1A1	0	1.8413134	0.865	0.537	0	SMC
COL18A1	0	1.83265742	0.844	0.521	0	SMC
CCDC3	0	1.81286111	0.808	0.4	0	SMC
RCAN2	0	1.79878499	0.703	0.36	0	SMC
ITGA8	0	1.79582852	0.62	0.298	0	SMC
CAVIN1	0	1.7500325	0.982	0.753	0	SMC
MRVI1	0	1.74098823	0.73	0.37	0	SMC
RGS5	0	1.70992427	0.44	0.259	0	SMC
CSRP2	0	1.7004888	0.751	0.475	0	SMC
4-Sep	0	1.69937663	0.746	0.446	0	SMC
CAV1	0	1.66020288	0.98	0.677	0	SMC
BGN	0	1.63666473	0.867	0.438	0	SMC
PDLIM7	0	1.63100145	0.838	0.583	0	SMC
CRIP2	0	1.58994298	0.843	0.451	0	SMC
CRYAB	0	1.58411745	0.791	0.352	0	SMC
VCL	0	1.56893227	0.849	0.596	0	SMC
NT5DC2	0	1.53584149	0.77	0.442	0	SMC
KLHL23	0	1.52884827	0.686	0.399	0	SMC
ACTN1	0	1.51576136	0.828	0.573	0	SMC
FILIP1L	0	1.46795535	0.724	0.408	0	SMC
SMTN	0	1.46740231	0.618	0.445	0	SMC
SYNM	0	1.43628714	0.616	0.283	0	SMC
C1QTNF1	0	1.43057526	0.708	0.369	0	SMC
GJA4	0	1.41702176	0.745	0.466	0	SMC
PALLD	0	1.41691738	0.738	0.48	0	SMC
NRGN	0	1.40536251	0.692	0.481	0	SMC
NR2F2	0	1.39839686	0.938	0.659	0	SMC
NEXN	0	1.38968189	0.641	0.327	0	SMC
AP000892.3	0	1.37160451	0.596	0.296	0	SMC
NRARP	0	1.36364181	0.688	0.301	0	SMC
WFDC1	0	1.35292995	0.591	0.342	0	SMC

EFHD1	0	1.32628916	0.616	0.337	0	SMC
FRZB	0	1.31672816	0.521	0.309	0	SMC
PTP4A3	0	1.30046316	0.631	0.426	0	SMC
ATP1A2	0	1.27908324	0.639	0.458	0	SMC
SLIT3	0	1.2419914	0.854	0.604	0	SMC
TIMP3	0	1.23062689	0.962	0.702	0	SMC
C12orf75	0	1.22730577	0.556	0.379	0	SMC
NTRK2	0	1.22017706	0.863	0.613	0	SMC
LGI4	0	1.21844979	0.745	0.464	0	SMC
PDGFA	0	1.21321223	0.556	0.328	0	SMC
ENTPD3	0	1.20588269	0.603	0.463	0	SMC
SYTL2	0	1.17654098	0.648	0.375	0	SMC
PLS3	0	1.17606191	0.766	0.542	0	SMC
RAMP1	0	1.17071604	0.456	0.289	0	SMC
EPAS1	0	1.14427278	0.823	0.499	0	SMC
MYOCD	0	1.13967329	0.5	0.375	0	SMC
COL4A2	0	1.13822542	0.851	0.611	0	SMC
PAWR	0	1.13301881	0.557	0.382	0	SMC
PTN	0	1.12824985	0.613	0.259	0	SMC
PGF	0	1.10757473	0.584	0.28	0	SMC
CLMN	0	1.10280143	0.625	0.495	0	SMC
ID4	0	1.09648805	0.454	0.278	0	SMC
NEURL1B	0	1.08806488	0.68	0.464	0	SMC
MGLL	0	1.08616887	0.846	0.627	0	SMC
SORBS1	0	1.0674978	0.502	0.316	0	SMC
OR51E1	0	1.06169214	0.586	0.302	0	SMC
FOXS1	0	1.0513089	0.611	0.42	0	SMC
INPP4B	0	1.03389792	0.642	0.349	0	SMC
MT1L	0	1.03119659	0.506	0.335	0	SMC
CRIM1	0	1.02932372	0.673	0.434	0	SMC
LIMS2	0	1.00588033	0.631	0.461	0	SMC
HIGD1B	0	0.99200257	0.521	0.298	0	SMC
CPE	0	0.99034091	0.915	0.64	0	SMC
FBXO32	0	0.97814451	0.417	0.214	0	SMC
SUSD2	0	0.96911583	0.537	0.376	0	SMC
ESAM	0	0.94957405	0.664	0.443	0	SMC
TUBA1C	0	0.93721443	0.738	0.427	0	SMC
PRPH	0	0.92773779	0.498	0.437	0	SMC
FHL1	0	0.91473272	0.594	0.367	0	SMC
A2M	0	0.89730633	0.937	0.632	0	SMC
ANGPT1	0	0.87803207	0.527	0.324	0	SMC
EDIL3	0	0.84871708	0.43	0.322	0	SMC
TGFBI	0	0.82621984	0.707	0.593	0	SMC
ITGA1	0	0.82284874	0.56	0.448	0	SMC
CCN1	0	0.81322322	0.521	0.362	0	SMC
CCN2	0	0.81051266	0.448	0.282	0	SMC
LINC02381	0	0.792833	0.616	0.398	0	SMC
MT1E	0	0.79183645	0.803	0.524	0	SMC
SBSPON	0	0.78139772	0.519	0.434	0	SMC
CCL19	0	0.77940787	0.37	0.309	0	SMC
COL4A1	0	0.76735821	0.803	0.527	0	SMC
PGAM2	0	0.76601046	0.441	0.34	0	SMC
MT1M	0	0.76340769	0.608	0.378	0	SMC

CD9	0	0.76249976	0.647	0.399	0	SMC
PDLIM3	0	0.75483986	0.418	0.294	0	SMC
HSPB6	0	0.73863503	0.599	0.472	0	SMC
TCF15	0	0.73850672	0.449	0.347	0	SMC
ITM2C	0	0.73224276	0.569	0.343	0	SMC
HES1	0	0.72881202	0.643	0.378	0	SMC
MAP3K7CL	0	0.68355663	0.447	0.345	0	SMC
NPY1R	0	0.67788613	0.53	0.395	0	SMC
LRRC10B	0	0.67417302	0.482	0.348	0	SMC
ANGPTL1	0	0.65172197	0.563	0.506	0	SMC
TPPP3	0	0.61891585	0.415	0.265	0	SMC
ALKAL2	0	0.61592739	0.444	0.369	0	SMC
NMNAT2	0	0.61052121	0.492	0.299	0	SMC
RGS7BP	0	0.60229281	0.435	0.252	0	SMC
LRRC32	0	0.57711857	0.583	0.465	0	SMC
TNMD	0	0.55537667	0.441	0.273	0	SMC
KCNE4	0	0.5401748	0.44	0.35	0	SMC
ACKR3	0	0.53789359	0.541	0.39	0	SMC
SYNE2	0	0.52201238	0.848	0.688	0	SMC
ADRA2A	0	0.51082671	0.426	0.336	0	SMC
TBX3	0	0.50933108	0.481	0.399	0	SMC
ITIH3	0	0.48824542	0.391	0.284	0	SMC
AIF1L	0	0.47458016	0.489	0.406	0	SMC
NES	0	0.44589552	0.52	0.397	0	SMC
ID3	0	0.43347187	0.711	0.521	0	SMC
MT2A	0	0.42398697	0.946	0.855	0	SMC
SPARC	0	0.42156803	0.877	0.718	0	SMC
AEBP1	0	0.40255686	0.771	0.572	0	SMC
CBLN1	0	0.39492305	0.39	0.211	0	SMC
GLDN	0	0.39011873	0.332	0.22	0	SMC
HIST1H4C	0	0.37368792	0.746	0.563	0	SMC
CAMK2N1	0	0.37311476	0.495	0.336	0	SMC
RARRES2	0	0.36528538	0.724	0.607	0	SMC
ACAN	0	0.33315883	0.347	0.201	0	SMC
KCNB1	0	0.3097106	0.391	0.23	0	SMC
IGFBP5	0	0.27769983	0.986	0.83	0	SMC
PDGFB	0	0.24194636	0.456	0.353	0	SMC
SLC38A11	0	0.2060815	0.33	0.111	0	SMC
DLC1	5.00E-304	0.53301842	0.617	0.508	1.00E-300	SMC
EBF2	5.99E-298	0.37309125	0.596	0.52	1.20E-294	SMC
LINC00924	2.96E-296	0.35584093	0.372	0.257	5.92E-293	SMC
DKK3	1.46E-291	0.59307438	0.435	0.304	2.92E-288	SMC
PCDH1	1.21E-290	0.30181718	0.445	0.394	2.42E-287	SMC
SOX4	1.82E-290	0.4496483	0.737	0.637	3.63E-287	SMC
FABP3	3.78E-285	0.42651878	0.416	0.362	7.56E-282	SMC
SVIL	1.16E-264	0.68725021	0.554	0.478	2.32E-261	SMC
COL5A3	1.95E-254	0.24930482	0.436	0.365	3.90E-251	SMC
DES	4.20E-246	1.83797272	0.425	0.383	8.40E-243	SMC
AVPR1A	1.77E-230	0.53060715	0.419	0.408	3.55E-227	SMC
RGS16	2.24E-229	0.45736673	0.309	0.222	4.48E-226	SMC
ANTXR1	8.85E-229	0.40624943	0.419	0.321	1.77E-225	SMC
RND3	4.73E-227	0.27553626	0.453	0.373	9.46E-224	SMC
TNFRSF12A	5.89E-212	0.53611376	0.268	0.172	1.18E-208	SMC

INHBA	4.51E-210	0.70070608	0.37	0.291	9.02E-207	SMC
FNBP1L	3.73E-209	0.20850745	0.408	0.351	7.47E-206	SMC
ADGRL3	3.18E-199	0.54074363	0.454	0.425	6.36E-196	SMC
SLC7A2	1.04E-192	0.68626498	0.436	0.415	2.09E-189	SMC
SLC22A3	5.80E-192	0.28252551	0.186	0.312	1.16E-188	SMC
FXYD6	2.82E-177	0.2853997	0.537	0.485	5.65E-174	SMC
TJP1	3.23E-167	0.33819082	0.561	0.518	6.45E-164	SMC
FHL5	9.16E-155	0.7045821	0.383	0.355	1.83E-151	SMC
ANK2	1.83E-139	0.25647541	0.486	0.48	3.67E-136	SMC
FBLIM1	1.16E-133	0.55133126	0.336	0.297	2.32E-130	SMC
HEYL	4.45E-129	0.54079684	0.308	0.259	8.90E-126	SMC
PARM1	1.12E-125	0.79541939	0.38	0.338	2.25E-122	SMC
KCNK3	9.26E-100	0.22011201	0.271	0.183	1.85E-96	SMC
HSPB7	1.21E-97	0.74538024	0.316	0.291	2.42E-94	SMC
MAST4	1.53E-89	0.22876945	0.411	0.409	3.06E-86	SMC
SEMA5A	2.26E-83	0.36059316	0.427	0.424	4.52E-80	SMC
MFAP4	6.80E-71	0.20712516	0.421	0.392	1.36E-67	SMC
FOXC2	4.21E-68	0.55689331	0.339	0.338	8.41E-65	SMC
CX3CL1	1.37E-66	0.46637871	0.348	0.369	2.74E-63	SMC
ENAH	2.86E-62	0.57079617	0.48	0.493	5.73E-59	SMC
ANGPT4	2.75E-57	0.31086039	0.35	0.351	5.51E-54	SMC
TMEM47	7.47E-45	0.4813884	0.34	0.339	1.49E-41	SMC
ANGPT2	4.42E-43	0.51136302	0.467	0.511	8.84E-40	SMC
ADAMTS1	1.87E-40	0.23406143	0.458	0.466	3.75E-37	SMC
ISG15	1.33E-38	0.22448105	0.857	0.842	2.66E-35	SMC
HAPLN3	1.96E-38	0.2633764	0.377	0.4	3.93E-35	SMC
PGM5	3.75E-38	0.34317777	0.4	0.429	7.49E-35	SMC
MYH10	9.88E-38	0.2138744	0.288	0.447	1.98E-34	SMC
SUSD5	1.27E-32	0.65037901	0.316	0.346	2.55E-29	SMC
ACTC1	7.40E-29	0.55782037	0.291	0.34	1.48E-25	SMC
SCN3A	1.42E-18	0.20761007	0.228	0.254	2.83E-15	SMC
SYNPO	1.49E-18	0.33482665	0.308	0.327	2.99E-15	SMC
SMOC2	9.64E-17	0.31115649	0.287	0.319	1.93E-13	SMC
SSTR2	1.58E-13	0.32553632	0.253	0.302	3.16E-10	SMC
CDH13	4.00E-13	0.44761732	0.249	0.279	8.00E-10	SMC
GUCY1A2	1.80E-10	0.41845238	0.366	0.47	3.60E-07	SMC
TM4SF1	4.27E-10	0.50619891	0.396	0.442	8.54E-07	SMC
BCYRN1	1.05E-07	0.37601583	0.352	0.414	0.00021055	SMC
DEPP1	2.57E-07	0.32466603	0.276	0.317	0.00051481	SMC
GGT5	2.02E-05	0.34201899	0.334	0.391	0.04031738	SMC
ADRA2C	2.90E-05	0.30094043	0.202	0.298	0.05791368	SMC
STEAP4	0.00015697	0.81368448	0.249	0.326	0.31393888	SMC
CR2	0	0.76690409	0.639	0.003	0	B_cell
AC104024.1	5.59E-287	0.20116113	0.567	0.01	1.12E-283	B_cell
TNFRSF17	5.36E-220	0.33355667	0.611	0.017	1.07E-216	B_cell
BLK	5.82E-203	1.90927605	0.711	0.086	1.16E-199	B_cell
PNOC	1.06E-146	0.83496596	0.628	0.023	2.13E-143	B_cell
TNFRSF13B	5.69E-140	1.85023902	0.733	0.117	1.14E-136	B_cell
MS4A1	6.82E-136	4.74357976	0.978	0.129	1.36E-132	B_cell
VPREB3	7.62E-128	2.66755367	0.794	0.115	1.52E-124	B_cell
BANK1	2.81E-123	3.39897494	0.878	0.162	5.62E-120	B_cell
POU2AF1	3.56E-108	1.59714909	0.678	0.117	7.12E-105	B_cell
UGT8	8.45E-106	0.26583561	0.561	0.061	1.69E-102	B_cell

CD79A	2.00E-101	3.68660141	0.856	0.206	4.00E-98	B_cell
CD37	1.79E-97	3.54812412	0.917	0.429	3.58E-94	B_cell
CD1C	5.77E-97	0.59738037	0.628	0.08	1.15E-93	B_cell
CD79B	5.85E-95	3.4906562	0.889	0.389	1.17E-91	B_cell
FCRL5	2.62E-93	1.61660759	0.711	0.094	5.24E-90	B_cell
LTB	6.67E-93	2.73118696	0.922	0.368	1.33E-89	B_cell
HLA-DQA1	2.02E-91	3.09256353	0.928	0.359	4.05E-88	B_cell
CD74	1.02E-85	2.35841357	0.972	0.693	2.04E-82	B_cell
IGHD	4.86E-84	3.3962237	0.733	0.157	9.73E-81	B_cell
LINC02397	4.25E-82	1.84423127	0.733	0.177	8.50E-79	B_cell
CD52	3.89E-79	2.87904705	0.9	0.495	7.78E-76	B_cell
HLA-DQB1	3.85E-76	2.43059488	0.906	0.385	7.70E-73	B_cell
HLA-DRA	4.34E-75	2.39909879	0.95	0.683	8.68E-72	B_cell
FAM30A	1.63E-71	1.67673453	0.644	0.107	3.26E-68	B_cell
IGHM	4.15E-71	3.47341923	0.867	0.575	8.29E-68	B_cell
HLA-DPB1	5.20E-68	2.48871396	0.883	0.492	1.04E-64	B_cell
IGLL5	1.90E-65	0.30287358	0.578	0.089	3.79E-62	B_cell
POU2F2	6.55E-65	2.3355724	0.872	0.47	1.31E-61	B_cell
HLA-DPA1	3.05E-64	2.09885085	0.894	0.464	6.10E-61	B_cell
CPNE5	6.14E-64	1.7954583	0.728	0.326	1.23E-60	B_cell
HLA-DQA2	7.53E-64	2.02103303	0.767	0.316	1.51E-60	B_cell
NAPSB	5.64E-61	1.92474873	0.767	0.363	1.13E-57	B_cell
MACC1	2.42E-58	0.24934133	0.578	0.138	4.84E-55	B_cell
SIGLEC10	5.88E-58	1.03134452	0.656	0.205	1.18E-54	B_cell
AC012236.1	4.40E-56	0.24440242	0.494	0.034	8.81E-53	B_cell
HLA-DOA	5.67E-56	0.98964819	0.689	0.246	1.13E-52	B_cell
HLA-DMA	1.51E-54	1.88133684	0.844	0.41	3.02E-51	B_cell
ZBP1	3.13E-52	0.66351807	0.639	0.246	6.26E-49	B_cell
PHACTR1	9.46E-52	1.98390317	0.772	0.352	1.89E-48	B_cell
MZB1	1.15E-51	0.87495929	0.672	0.297	2.31E-48	B_cell
IRF4	3.39E-51	0.6646233	0.6	0.188	6.78E-48	B_cell
CLNK	4.76E-51	0.45344053	0.567	0.143	9.52E-48	B_cell
IGKC	2.67E-50	1.87323321	0.8	0.587	5.35E-47	B_cell
CD72	3.39E-49	1.65349657	0.689	0.341	6.79E-46	B_cell
EZR	3.41E-49	1.73652185	0.767	0.347	6.82E-46	B_cell
PLAC8	3.80E-49	1.84866613	0.733	0.386	7.59E-46	B_cell
HLA-DRB1	4.30E-49	1.76991534	0.872	0.522	8.60E-46	B_cell
RAC2	1.24E-48	1.81505497	0.817	0.386	2.47E-45	B_cell
CORO1A	3.62E-48	1.47703939	0.856	0.49	7.24E-45	B_cell
AC020951.1	4.29E-48	0.30851861	0.578	0.219	8.59E-45	B_cell
DNASE1L3	1.55E-47	0.34635294	0.617	0.233	3.11E-44	B_cell
RASGRP3	8.47E-46	1.33306657	0.706	0.377	1.69E-42	B_cell
PTPRC	1.70E-45	1.53493987	0.889	0.56	3.41E-42	B_cell
AC243960.1	9.59E-44	0.83763755	0.656	0.288	1.92E-40	B_cell
EGR2	1.20E-42	0.45726899	0.617	0.193	2.41E-39	B_cell
PCED1B-AS1	7.62E-42	0.95450519	0.683	0.268	1.52E-38	B_cell
PIF1	1.17E-40	0.29692444	0.583	0.272	2.34E-37	B_cell
KYNU	1.24E-40	0.98914337	0.656	0.311	2.49E-37	B_cell
PTPN6	3.54E-40	1.71582991	0.761	0.452	7.08E-37	B_cell
HLA-DMB	5.97E-40	1.53741362	0.794	0.462	1.19E-36	B_cell
HHEX	1.01E-38	1.08138407	0.689	0.308	2.02E-35	B_cell
BCL2A1	2.06E-38	1.15632672	0.728	0.415	4.13E-35	B_cell
ITGA4	2.97E-38	1.34906622	0.772	0.46	5.95E-35	B_cell

HCLS1	4.67E-38	1.26309456	0.778	0.386	9.35E-35	B_cell
EVI2B	1.00E-37	1.31320762	0.733	0.362	2.01E-34	B_cell
SYK	1.51E-37	1.373587	0.739	0.447	3.03E-34	B_cell
CCDC141	3.61E-36	0.58613546	0.639	0.325	7.23E-33	B_cell
COCH	4.18E-36	0.46424438	0.617	0.309	8.36E-33	B_cell
CHI3L2	5.47E-36	0.91845701	0.633	0.366	1.09E-32	B_cell
TTN	7.45E-36	0.80926286	0.717	0.458	1.49E-32	B_cell
CYFIP2	9.21E-36	0.91094807	0.722	0.309	1.84E-32	B_cell
PTPN7	3.44E-35	0.88604251	0.678	0.317	6.88E-32	B_cell
HLA-DRB5	4.14E-35	1.27286309	0.806	0.445	8.28E-32	B_cell
HELLS	4.46E-35	0.54169638	0.628	0.34	8.92E-32	B_cell
CIITA	4.07E-34	1.44653675	0.7	0.46	8.15E-31	B_cell
CD69	1.44E-33	1.63143541	0.7	0.314	2.87E-30	B_cell
PLEKHG1	3.92E-33	0.43370212	0.617	0.301	7.85E-30	B_cell
LAPTM5	7.10E-33	1.25754836	0.822	0.505	1.42E-29	B_cell
SPON1	9.81E-33	0.37967566	0.633	0.286	1.96E-29	B_cell
LYN	1.32E-32	1.32452782	0.761	0.506	2.64E-29	B_cell
CXorf21	1.54E-31	0.49059795	0.633	0.345	3.07E-28	B_cell
ACP5	2.43E-31	1.15612197	0.689	0.447	4.86E-28	B_cell
HLA-B	1.16E-30	0.85553126	0.961	0.86	2.33E-27	B_cell
APOLD1	2.04E-30	0.44191382	0.628	0.279	4.09E-27	B_cell
IKZF1	9.90E-30	1.0478366	0.744	0.407	1.98E-26	B_cell
RNF144B	8.70E-28	0.37896691	0.078	0.301	1.74E-24	B_cell
L1TD1	1.07E-27	0.2166105	0.6	0.35	2.14E-24	B_cell
CLECL1	1.28E-27	0.95669497	0.661	0.326	2.57E-24	B_cell
SYNJ2	1.08E-26	0.43064671	0.622	0.287	2.16E-23	B_cell
LILRB1	2.04E-26	0.87288873	0.678	0.467	4.07E-23	B_cell
FAM49B	2.94E-26	0.7662929	0.711	0.391	5.87E-23	B_cell
HLA-DRB6	1.22E-25	0.76356354	0.756	0.429	2.43E-22	B_cell
AC002558.3	3.07E-25	0.42059702	0.594	0.303	6.15E-22	B_cell
CD180	2.46E-24	0.65774348	0.644	0.416	4.93E-21	B_cell
TFRC	3.06E-24	0.62829104	0.656	0.309	6.13E-21	B_cell
BIRC3	4.65E-24	1.90771955	0.644	0.366	9.30E-21	B_cell
PCDH9	2.86E-23	0.30607291	0.072	0.288	5.71E-20	B_cell
UCP2	1.05E-22	1.09202702	0.761	0.521	2.10E-19	B_cell
IKZF3	1.05E-22	1.56827104	0.589	0.241	2.11E-19	B_cell
LCP1	3.53E-22	0.76658061	0.844	0.606	7.06E-19	B_cell
UGCG	1.06E-21	0.83598926	0.683	0.354	2.12E-18	B_cell
ADAMTS6	1.10E-21	0.43791649	0.133	0.356	2.19E-18	B_cell
SMC4	3.58E-21	0.34066608	0.656	0.34	7.15E-18	B_cell
SNX10	2.65E-20	0.21875751	0.189	0.426	5.29E-17	B_cell
ARL4C	5.42E-20	0.27203175	0.639	0.292	1.08E-16	B_cell
CD38	5.73E-20	0.42537272	0.611	0.356	1.15E-16	B_cell
LSP1	7.68E-20	0.93365186	0.806	0.587	1.54E-16	B_cell
CYTH4	1.58E-19	0.33252234	0.65	0.43	3.15E-16	B_cell
PIK3AP1	2.17E-19	0.69833031	0.217	0.451	4.34E-16	B_cell
PLEK	1.28E-18	0.46383801	0.7	0.445	2.57E-15	B_cell
GM2A	3.00E-18	0.52358716	0.683	0.492	6.00E-15	B_cell
IL10RA	1.37E-17	0.20403886	0.189	0.408	2.73E-14	B_cell
IGLC2	4.23E-16	1.22388005	0.611	0.414	8.46E-13	B_cell
SLC2A3	3.55E-15	0.97653648	0.65	0.453	7.09E-12	B_cell
CKAP2	1.13E-14	0.69911191	0.611	0.361	2.25E-11	B_cell
SPIB	6.32E-14	2.05422205	0.506	0.184	1.26E-10	B_cell

CYBA	2.47E-13	0.57364209	0.828	0.761	4.95E-10	B_cell
AD000864.1	2.53E-11	1.69729542	0.461	0.227	5.06E-08	B_cell
HIST1H1D	4.24E-11	0.36133138	0.606	0.364	8.47E-08	B_cell
CXCR4	9.12E-11	0.71825169	0.589	0.357	1.82E-07	B_cell
GPR65	1.05E-10	0.21164173	0.256	0.439	2.10E-07	B_cell
ARHGAP30	1.08E-10	0.53952811	0.661	0.463	2.17E-07	B_cell
NCKAP1L	1.18E-10	0.47154912	0.689	0.5	2.36E-07	B_cell
SAMSN1	1.43E-10	0.22960411	0.244	0.421	2.86E-07	B_cell
CYSLTR1	1.78E-10	0.70091747	0.239	0.364	3.56E-07	B_cell
RGS1	1.19E-09	0.30679819	0.183	0.307	2.38E-06	B_cell
CD53	2.62E-09	1.15879221	0.606	0.439	5.23E-06	B_cell
CD83	1.18E-08	0.78287619	0.539	0.321	2.36E-05	B_cell
DUSP2	1.50E-08	0.98630096	0.483	0.245	3.01E-05	B_cell
IFI30	2.50E-08	0.34897309	0.261	0.402	4.99E-05	B_cell
MARCKSL1	2.69E-08	0.63204729	0.539	0.317	5.38E-05	B_cell
SERPINB9	2.98E-08	0.38553673	0.211	0.286	5.97E-05	B_cell
CYBB	2.99E-08	0.25106766	0.717	0.591	5.98E-05	B_cell
RGS10	3.07E-08	0.20773486	0.661	0.466	6.14E-05	B_cell
HMGA1	4.41E-08	0.63283299	0.644	0.386	8.82E-05	B_cell
EPSTI1	5.52E-08	0.54285583	0.678	0.612	0.00011045	B_cell
FLNB	7.34E-07	0.28664031	0.639	0.475	0.00146791	B_cell
NFKBIA	7.44E-07	0.49443351	0.722	0.623	0.00148769	B_cell
ICAM2	9.67E-07	0.23026928	0.622	0.453	0.00193399	B_cell
MCM7	1.58E-06	0.2950076	0.311	0.399	0.00315973	B_cell
MYC	2.62E-06	0.20691697	0.572	0.366	0.00523787	B_cell
NR4A2	3.25E-06	0.65618514	0.544	0.373	0.00650654	B_cell
OTUD1	7.73E-06	0.54131117	0.25	0.351	0.01546571	B_cell
GPSM3	1.14E-05	0.89754963	0.533	0.411	0.02278806	B_cell
EIF4E	1.40E-05	0.31150815	0.206	0.346	0.02795271	B_cell
CD48	1.44E-05	1.32328027	0.517	0.366	0.02881265	B_cell
IRF8	2.13E-05	1.30334381	0.467	0.301	0.04263998	B_cell
NCF4	3.09E-05	0.45117078	0.283	0.362	0.06180916	B_cell
MTSS1	3.76E-05	0.57739688	0.311	0.473	0.07523617	B_cell
BHLHE41	6.32E-05	0.71610224	0.25	0.307	0.12631804	B_cell
HIST1H4C	0.00017594	0.43817162	0.65	0.611	0.35188828	B_cell
ST6GAL1	0.00019425	0.87581048	0.289	0.336	0.38850271	B_cell
MX1	0.000362	0.30380498	0.717	0.666	0.72399163	B_cell
HSPH1	0.00044243	0.42375444	0.5	0.374	0.88484906	B_cell
CTSH	0.00047264	0.37845214	0.378	0.493	0.94528691	B_cell
ISG20	0.00060559	0.97704645	0.478	0.343	1	B_cell
FCGR2B	0.00164664	0.76702938	0.261	0.301	1	B_cell
RASGEF1B	0.00201611	0.47324159	0.278	0.311	1	B_cell
WAS	0.00386071	0.62480948	0.317	0.39	1	B_cell
RNASET2	0.00590278	0.32753643	0.606	0.527	1	B_cell
CD24	0.00635483	1.83608791	0.422	0.246	1	B_cell
TRBC2	0.00757692	0.28032864	0.261	0.221	1	B_cell
ZNF331	0.00808187	0.51075075	0.411	0.29	1	B_cell
NKG7	0	4.32464628	1	0.213	0	NK
GZMB	0	4.09836978	0.959	0.18	0	NK
KLRD1	0	3.44204948	0.975	0.23	0	NK
CTSW	0	3.42061407	0.981	0.246	0	NK
PRF1	0	3.20499723	0.985	0.198	0	NK
TRDC	0	2.91837957	0.956	0.226	0	NK

IL2RB	0	2.52095413	0.965	0.212	0	NK
FGFBP2	0	2.46526363	0.927	0.151	0	NK
KLRF1	0	2.38777957	0.954	0.136	0	NK
CX3CR1	0	1.78680533	0.94	0.323	0	NK
LAIR2	0	1.2954811	0.913	0.136	0	NK
SH2D1B	0	0.87315416	0.853	0.091	0	NK
KIR2DS4	0	0.8432152	0.884	0.087	0	NK
GZMM	1.65E-301	2.45175075	0.977	0.237	3.29E-298	NK
PLAC8	6.93E-298	2.45459127	0.963	0.381	1.39E-294	NK
CD247	3.45E-288	2.83046961	0.954	0.177	6.89E-285	NK
ADGRG1	3.31E-267	1.621687	0.915	0.199	6.62E-264	NK
CLIC3	3.50E-262	2.28091373	0.938	0.282	7.01E-259	NK
GNLY	3.61E-254	5.34098187	0.9	0.23	7.22E-251	NK
GZMA	1.52E-252	3.25908231	0.938	0.208	3.04E-249	NK
IKZF3	1.07E-245	1.53542053	0.923	0.235	2.13E-242	NK
GINS2	7.97E-237	0.22345568	0.871	0.331	1.59E-233	NK
TNFRSF18	3.46E-223	1.19889489	0.88	0.249	6.92E-220	NK
CENPK	5.44E-222	0.36449066	0.871	0.286	1.09E-218	NK
CD38	2.73E-219	0.89294315	0.905	0.35	5.45E-216	NK
CORO1A	7.97E-217	2.43098146	0.977	0.486	1.59E-213	NK
HCST	2.48E-211	2.17586238	0.979	0.514	4.95E-208	NK
CD7	2.63E-206	2.19731824	0.894	0.249	5.26E-203	NK
CCL5	3.53E-203	2.2505046	0.892	0.242	7.06E-200	NK
MATK	6.00E-203	1.9225871	0.88	0.286	1.20E-199	NK
CD79B	5.40E-195	0.42656447	0.884	0.386	1.08E-191	NK
PTPRC	4.28E-191	1.97018139	0.981	0.557	8.57E-188	NK
ITGAX	3.57E-189	0.94937454	0.907	0.428	7.13E-186	NK
FCGR3A	1.57E-173	1.64132196	0.944	0.441	3.15E-170	NK
EFHD2	3.47E-173	1.84761231	0.977	0.554	6.94E-170	NK
SLAMF7	4.42E-172	0.88868652	0.878	0.377	8.83E-169	NK
ARL4C	1.37E-170	2.0295945	0.896	0.286	2.73E-167	NK
SPON2	6.07E-167	1.8858467	0.944	0.417	1.21E-163	NK
MYO1F	3.67E-162	1.45845794	0.936	0.522	7.35E-159	NK
BRCA2	4.96E-160	0.20844554	0.867	0.404	9.92E-157	NK
GBP5	1.25E-157	0.80966509	0.88	0.389	2.51E-154	NK
PLEK	1.43E-157	1.34415698	0.959	0.44	2.85E-154	NK
AKR1C3	9.31E-148	0.47563331	0.849	0.229	1.86E-144	NK
LCP1	2.65E-140	1.46305526	0.983	0.602	5.30E-137	NK
SLC12A2	4.36E-140	0.27198334	0.853	0.272	8.72E-137	NK
LCP2	3.55E-135	0.92576858	0.907	0.403	7.09E-132	NK
IRF1	3.69E-134	1.70512948	0.896	0.553	7.39E-131	NK
GIMAP7	1.31E-131	1.2273371	0.959	0.484	2.62E-128	NK
HAVCR2	4.66E-129	0.33603992	0.871	0.441	9.32E-126	NK
CST7	4.43E-128	2.37519912	0.788	0.2	8.86E-125	NK
MCM7	2.58E-124	0.35380483	0.871	0.393	5.15E-121	NK
IL32	1.33E-122	1.72239619	0.832	0.297	2.66E-119	NK
SDF2L1	2.26E-122	1.1022237	0.942	0.506	4.51E-119	NK
FERMT3	1.70E-121	0.7873006	0.888	0.361	3.40E-118	NK
PIM1	2.74E-121	1.07507232	0.84	0.302	5.47E-118	NK
SRGN	2.52E-120	1.10403549	0.965	0.5	5.04E-117	NK
ICAM2	9.00E-116	0.33300087	0.894	0.448	1.80E-112	NK
TRBC1	9.64E-116	1.94082893	0.784	0.244	1.93E-112	NK
GIMAP6	1.11E-111	0.39363872	0.882	0.412	2.22E-108	NK

CD69	2.21E-110	1.55821237	0.795	0.31	4.42E-107	NK
FAM49B	9.38E-110	1.33625143	0.869	0.386	1.88E-106	NK
FCER1G	1.25E-109	0.94266527	0.971	0.584	2.50E-106	NK
RAC2	1.47E-109	1.86941997	0.817	0.383	2.94E-106	NK
HMGA1	3.71E-109	0.6065138	0.844	0.381	7.43E-106	NK
CYBA	5.30E-109	1.36594911	0.992	0.758	1.06E-105	NK
MDM2	3.90E-107	0.36519443	0.878	0.342	7.80E-104	NK
HLA-B	1.53E-106	0.9995111	0.994	0.858	3.06E-103	NK
LDLR	2.47E-104	0.20565661	0.876	0.34	4.93E-101	NK
AL031777.3	2.26E-101	0.52499759	0.803	0.397	4.51E-98	NK
RUNX3	3.77E-95	1.68307268	0.747	0.237	7.54E-92	NK
LYN	4.55E-94	0.34584256	0.894	0.503	9.10E-91	NK
PTGDS	1.79E-92	0.83697255	0.853	0.371	3.59E-89	NK
ADA	9.11E-92	0.23729618	0.855	0.379	1.82E-88	NK
UCP2	3.12E-89	0.7564527	0.927	0.518	6.25E-86	NK
ARHGAP30	5.08E-85	0.73117551	0.873	0.458	1.02E-81	NK
CD320	2.55E-84	0.26891147	0.859	0.368	5.10E-81	NK
C12orf75	6.12E-84	0.6213917	0.89	0.42	1.22E-80	NK
XCL2	3.80E-81	2.64042459	0.687	0.147	7.60E-78	NK
VAMP8	1.56E-80	0.74703358	0.902	0.491	3.12E-77	NK
IKZF1	3.29E-80	1.27866586	0.801	0.403	6.58E-77	NK
DUSP2	1.20E-76	1.15109007	0.718	0.24	2.39E-73	NK
HCLS1	1.81E-75	0.95836044	0.834	0.383	3.61E-72	NK
ARRB2	1.00E-74	0.3647381	0.869	0.454	2.00E-71	NK
NEDD9	1.37E-72	0.83816519	0.747	0.273	2.74E-69	NK
ZNF683	1.00E-69	0.63245615	0.672	0.186	2.01E-66	NK
NCKAP1L	1.26E-65	0.22603769	0.9	0.496	2.51E-62	NK
ICAM1	1.28E-64	0.35274282	0.751	0.274	2.56E-61	NK
UPP1	1.75E-64	0.86507559	0.737	0.277	3.51E-61	NK
PCNA	8.60E-64	0.3981395	0.751	0.309	1.72E-60	NK
FGR	2.39E-62	0.64693876	0.732	0.336	4.78E-59	NK
SLFN5	3.55E-60	0.85516312	0.917	0.597	7.10E-57	NK
ITGB2	2.93E-59	1.26907071	0.815	0.532	5.86E-56	NK
SNAI1	6.35E-59	0.33478788	0.645	0.196	1.27E-55	NK
CYFIP2	7.16E-58	0.88521253	0.737	0.305	1.43E-54	NK
EIF4E	1.88E-56	0.43542342	0.78	0.34	3.75E-53	NK
TYROBP	2.24E-56	0.47929948	0.902	0.666	4.48E-53	NK
SYTL3	3.92E-53	0.97294928	0.658	0.204	7.85E-50	NK
CLSPN	7.87E-50	0.22147949	0.672	0.283	1.57E-46	NK
TNFRSF1B	2.11E-49	0.53557939	0.842	0.521	4.22E-46	NK
LSP1	2.99E-49	1.01289425	0.817	0.585	5.98E-46	NK
DOK2	1.07E-47	1.30391627	0.718	0.385	2.13E-44	NK
TNFRSF4	2.49E-46	0.34497186	0.674	0.264	4.97E-43	NK
KLRC1	8.37E-45	1.71195966	0.633	0.226	1.67E-41	NK
CLNK	9.47E-45	0.63804149	0.286	0.144	1.89E-41	NK
CRTAM	5.67E-44	0.81497625	0.253	0.217	1.13E-40	NK
CD52	4.51E-42	1.07909449	0.747	0.493	9.02E-39	NK
CCL4	2.80E-41	1.41671849	0.683	0.25	5.60E-38	NK
NR4A2	1.39E-40	0.74961028	0.745	0.369	2.78E-37	NK
XCL1	1.32E-38	2.21462942	0.6	0.146	2.64E-35	NK
CD53	1.60E-36	1.16655608	0.695	0.436	3.19E-33	NK
AC243960.1	9.59E-35	0.70754728	0.295	0.29	1.92E-31	NK
FYB1	1.36E-34	0.78501223	0.759	0.509	2.73E-31	NK

CYTH4	2.44E-34	0.24032592	0.745	0.427	4.89E-31	NK
SEC11C	4.10E-34	0.30303308	0.743	0.339	8.20E-31	NK
MBP	9.52E-34	0.96372705	0.718	0.457	1.90E-30	NK
KLRC2	9.90E-34	1.49559497	0.558	0.096	1.98E-30	NK
DDIT4	4.07E-32	0.81364045	0.712	0.397	8.14E-29	NK
HMGB2	4.02E-31	0.94435209	0.662	0.389	8.05E-28	NK
FKBP11	1.04E-30	0.323832	0.301	0.259	2.09E-27	NK
PHLDA1	4.98E-30	0.39804309	0.28	0.271	9.96E-27	NK
OASL	4.90E-29	0.51963249	0.714	0.423	9.80E-26	NK
WAS	8.16E-29	0.64362286	0.678	0.386	1.63E-25	NK
SSR4	4.34E-28	0.59373754	0.886	0.726	8.67E-25	NK
TNFRSF9	8.33E-28	0.20851243	0.568	0.212	1.67E-24	NK
KRT81	5.11E-27	0.6578342	0.317	0.144	1.02E-23	NK
ITGA4	7.85E-27	0.96506618	0.72	0.459	1.57E-23	NK
KPNA2	2.55E-26	0.289449	0.286	0.2	5.09E-23	NK
AD000864.1	6.47E-26	1.11241894	0.564	0.224	1.29E-22	NK
ALOX5AP	1.21E-25	1.04755896	0.604	0.217	2.41E-22	NK
MIR155HG	1.49E-24	0.24688459	0.544	0.184	2.98E-21	NK
CTSC	1.39E-23	0.41839012	0.857	0.572	2.79E-20	NK
CLEC2B	1.56E-23	1.07220046	0.685	0.474	3.12E-20	NK
JAKMIP2	3.50E-23	0.48023645	0.305	0.205	6.99E-20	NK
TNF	3.54E-23	0.57795798	0.301	0.285	7.09E-20	NK
SERPINB9	4.92E-23	0.43818445	0.29	0.285	9.84E-20	NK
GZMH	8.50E-23	1.74194735	0.587	0.168	1.70E-19	NK
GIMAP4	8.66E-22	0.54307069	0.737	0.475	1.73E-18	NK
PIM2	1.71E-21	0.39189272	0.309	0.2	3.42E-18	NK
BCL11B	2.03E-19	0.80533428	0.589	0.252	4.07E-16	NK
KLRB1	2.10E-19	1.7170197	0.61	0.24	4.20E-16	NK
BHLHE40	3.07E-19	0.43207171	0.322	0.312	6.13E-16	NK
SYNE2	1.94E-18	0.40435309	0.938	0.728	3.88E-15	NK
PTPN7	4.46E-18	0.96826012	0.6	0.316	8.92E-15	NK
NFKBIA	2.32E-16	0.66065361	0.809	0.622	4.64E-13	NK
ZBP1	2.56E-16	0.24150002	0.55	0.244	5.12E-13	NK
IQGAP2	2.76E-16	0.8446305	0.629	0.383	5.52E-13	NK
BCL3	4.48E-16	0.22127561	0.303	0.271	8.96E-13	NK
CYTOR	4.27E-14	0.34517685	0.363	0.39	8.53E-11	NK
CD37	5.64E-14	0.54166756	0.658	0.428	1.13E-10	NK
XBP1	3.74E-13	0.73129973	0.668	0.502	7.48E-10	NK
GMFG	6.15E-12	0.71163462	0.649	0.453	1.23E-08	NK
HSPH1	1.25E-11	0.53672058	0.614	0.371	2.49E-08	NK
MARCKSL1	2.06E-11	0.30839966	0.338	0.317	4.12E-08	NK
BST2	2.38E-11	0.2688988	0.905	0.725	4.77E-08	NK
EVI2B	1.35E-10	0.2910105	0.384	0.363	2.71E-07	NK
GPSM3	9.22E-10	0.72980438	0.616	0.409	1.84E-06	NK
AREG	6.87E-09	0.91493531	0.512	0.207	1.37E-05	NK
HIST1H1D	2.75E-08	0.57599967	0.396	0.365	5.50E-05	NK
CD48	3.23E-08	0.9042595	0.571	0.364	6.47E-05	NK
CD3E	7.21E-08	0.93260915	0.527	0.237	0.00014413	NK
PRDM1	1.67E-06	0.40013011	0.392	0.29	0.00333964	NK
PNP	3.17E-06	0.36478861	0.297	0.232	0.00633212	NK
CD2	2.06E-05	1.00848279	0.512	0.245	0.04124385	NK
UAP1	2.98E-05	0.20134921	0.332	0.268	0.05957124	NK
HSPA6	3.13E-05	0.62356914	0.386	0.186	0.06267095	NK

CCL4L2	3.57E-05	0.98280856	0.369	0.155	0.07132538	NK
SLC2A3	8.93E-05	0.29782091	0.643	0.452	0.1785237	NK
CD3G	0.00013267	0.31304316	0.405	0.229	0.26534445	NK
EZH2	0.00039707	0.27932257	0.45	0.345	0.79413639	NK
CKS2	0.00052995	0.23982313	0.4	0.219	1	NK
IL10RA	0.00133268	0.44525889	0.566	0.406	1	NK
ATAD2	0.00135737	0.23291645	0.423	0.312	1	NK
PCED1B-AS1	0.00154601	1.10236968	0.517	0.267	1	NK
CCL3	0.00170477	1.36941874	0.473	0.396	1	NK
TRBC2	0.00249418	0.71976635	0.523	0.217	1	NK
BIRC3	0.00302243	0.54716067	0.438	0.367	1	NK
PMAIP1	0.00434641	0.62240912	0.417	0.262	1	NK
SMC4	0.00602688	0.22529327	0.544	0.339	1	NK
LINC02446	0.00729798	0.60760327	0.496	0.158	1	NK
GZMK	0.00757615	0.95946054	0.405	0.18	1	NK
ISG20	0.00877068	0.53994982	0.535	0.341	1	NK
SPINK2	3.81E-80	0.36680081	1	0.105	7.61E-77	Mast_cell
FAM30A	9.15E-75	0.44184847	1	0.108	1.83E-71	Mast_cell
CPA3	2.65E-65	4.88490705	1	0.092	5.30E-62	Mast_cell
TPSB2	4.40E-58	6.37439099	1	0.126	8.79E-55	Mast_cell
PNMT	1.03E-56	0.60829856	1	0.158	2.06E-53	Mast_cell
TPSAB1	5.69E-56	5.78609111	0.986	0.114	1.14E-52	Mast_cell
KLRC1	9.49E-56	0.25703671	1	0.229	1.90E-52	Mast_cell
RORB	9.06E-55	0.34640676	1	0.136	1.81E-51	Mast_cell
TMIGD2	1.38E-54	0.30739671	1	0.204	2.76E-51	Mast_cell
TRGC1	1.40E-54	0.22661953	1	0.251	2.79E-51	Mast_cell
KIT	2.04E-54	3.52710514	0.959	0.261	4.09E-51	Mast_cell
ST8SIA6	2.36E-52	0.59682978	0.986	0.269	4.73E-49	Mast_cell
TNFRSF18	7.86E-51	0.72169041	1	0.255	1.57E-47	Mast_cell
TRDC	6.05E-48	0.21535849	1	0.234	1.21E-44	Mast_cell
CLIC3	6.71E-48	0.66286781	1	0.288	1.34E-44	Mast_cell
CCDC141	1.69E-47	0.32900999	1	0.325	3.39E-44	Mast_cell
COL4A4	6.65E-47	0.26554237	1	0.183	1.33E-43	Mast_cell
TNFSF14	1.05E-46	0.41920285	1	0.251	2.09E-43	Mast_cell
KLF5	1.98E-46	0.25385802	1	0.263	3.95E-43	Mast_cell
LINC00520	2.97E-46	0.60448027	0.77	0.1	5.94E-43	Mast_cell
HPGDS	5.62E-46	3.85058656	0.959	0.294	1.12E-42	Mast_cell
BRCA2	5.19E-44	0.29316945	1	0.409	1.04E-40	Mast_cell
UNC5B	1.06E-42	0.21178426	0.986	0.213	2.11E-39	Mast_cell
CD7	2.19E-42	0.58823712	1	0.255	4.38E-39	Mast_cell
EZH2	3.09E-41	0.25783405	1	0.345	6.17E-38	Mast_cell
AC002558.3	4.32E-41	0.35016002	1	0.303	8.64E-38	Mast_cell
MS4A2	6.75E-40	3.5056316	0.851	0.055	1.35E-36	Mast_cell
HPGD	7.33E-39	3.47490974	0.905	0.202	1.47E-35	Mast_cell
RSAD2	1.40E-37	1.01247216	1	0.553	2.81E-34	Mast_cell
FCER1G	9.06E-37	2.34175503	1	0.588	1.81E-33	Mast_cell
CAVIN2	1.11E-36	1.89850528	1	0.337	2.23E-33	Mast_cell
CD79B	1.44E-36	0.32383831	1	0.391	2.88E-33	Mast_cell
RHEX	4.20E-34	3.2555682	0.824	0.046	8.40E-31	Mast_cell
ALOX5AP	5.32E-34	3.45491485	0.878	0.22	1.06E-30	Mast_cell
EDNRB	1.38E-33	0.41536599	0.959	0.251	2.76E-30	Mast_cell
NTM	3.61E-33	0.78911457	0.986	0.301	7.22E-30	Mast_cell
GPR183	4.52E-32	0.63317727	1	0.327	9.04E-29	Mast_cell

CTSW	4.63E-31	0.97727893	0.932	0.253	9.27E-28	Mast_cell
TRGC2	5.82E-31	0.32741898	0.932	0.201	1.16E-27	Mast_cell
SNX10	5.57E-29	0.21660043	1	0.424	1.11E-25	Mast_cell
OSM	8.18E-29	0.26037391	0.905	0.175	1.64E-25	Mast_cell
KRT19	3.66E-28	0.92490543	0.838	0.18	7.33E-25	Mast_cell
FBP1	7.71E-28	0.40789878	0.986	0.378	1.54E-24	Mast_cell
TNFRSF9	8.31E-28	0.2179625	0.919	0.215	1.66E-24	Mast_cell
CLEC12A	8.78E-28	0.40707619	1	0.429	1.76E-24	Mast_cell
CAPG	3.36E-27	2.18483714	0.986	0.499	6.72E-24	Mast_cell
RAC2	6.58E-27	2.45189612	0.892	0.387	1.32E-23	Mast_cell
ITGA4	9.68E-27	0.94613326	0.986	0.461	1.94E-23	Mast_cell
C1orf162	1.24E-26	1.15452326	1	0.476	2.48E-23	Mast_cell
MYO1F	8.96E-26	0.529165	1	0.526	1.79E-22	Mast_cell
GATA2	3.54E-25	2.9862042	0.824	0.186	7.09E-22	Mast_cell
B3GNT5	3.20E-24	0.30396628	0.081	0.277	6.39E-21	Mast_cell
CTSG	3.51E-24	4.20314178	0.811	0.105	7.02E-21	Mast_cell
G0S2	2.57E-22	0.58701323	1	0.403	5.15E-19	Mast_cell
IQGAP2	6.85E-22	0.6723087	0.973	0.385	1.37E-18	Mast_cell
CLECL1	1.62E-21	0.80958148	0.932	0.327	3.25E-18	Mast_cell
SGK1	7.68E-21	2.27461839	0.905	0.367	1.54E-17	Mast_cell
CD69	1.03E-20	1.16301331	0.865	0.315	2.06E-17	Mast_cell
SAMSN1	1.37E-20	2.04876982	0.851	0.419	2.74E-17	Mast_cell
CD52	1.60E-20	0.80747865	1	0.495	3.21E-17	Mast_cell
CSF2RB	3.15E-20	0.66899419	0.203	0.319	6.30E-17	Mast_cell
SH3BGRL3	8.20E-20	1.23190863	1	0.814	1.64E-16	Mast_cell
ARHGAP30	3.00E-19	0.56093377	1	0.462	6.00E-16	Mast_cell
PTPN7	3.38E-19	1.6073952	0.838	0.318	6.75E-16	Mast_cell
CKAP2	3.43E-19	0.27080198	0.203	0.363	6.86E-16	Mast_cell
RGS10	1.25E-18	2.06412853	0.811	0.466	2.50E-15	Mast_cell
NFKBIA	1.32E-18	1.38481108	0.905	0.623	2.65E-15	Mast_cell
PTPN6	2.44E-18	1.14253414	0.919	0.452	4.88E-15	Mast_cell
UCP2	3.48E-18	0.47594821	1	0.522	6.97E-15	Mast_cell
RGS1	9.76E-18	2.61461101	0.757	0.306	1.95E-14	Mast_cell
LTC4S	1.24E-17	2.53615739	0.784	0.298	2.49E-14	Mast_cell
PHACTR1	1.30E-17	0.83084554	0.959	0.353	2.60E-14	Mast_cell
TYROBP	2.14E-17	1.00516651	0.986	0.669	4.28E-14	Mast_cell
CYSLTR1	2.59E-17	0.28521569	0.959	0.362	5.19E-14	Mast_cell
HCST	7.24E-17	0.57905586	1	0.519	1.45E-13	Mast_cell
FTL	1.23E-16	0.39017278	1	0.996	2.46E-13	Mast_cell
BCL2A1	1.58E-16	0.33827655	0.257	0.417	3.17E-13	Mast_cell
VAMP8	3.42E-16	0.93793071	0.973	0.495	6.83E-13	Mast_cell
FSCN1	3.92E-16	0.28042356	0.986	0.379	7.84E-13	Mast_cell
PROCR	6.90E-16	0.72742241	0.986	0.471	1.38E-12	Mast_cell
TTN	2.71E-15	0.30420854	0.892	0.459	5.42E-12	Mast_cell
MS4A4A	3.03E-15	0.26327967	0.257	0.35	6.07E-12	Mast_cell
CYTOR	3.22E-15	0.21647342	1	0.388	6.43E-12	Mast_cell
SLC2A3	4.57E-15	1.56784452	0.824	0.454	9.14E-12	Mast_cell
TMEM233	8.57E-15	0.54752112	0.716	0.23	1.71E-11	Mast_cell
ARHGAP18	1.00E-14	2.29456162	0.757	0.378	2.00E-11	Mast_cell
SRGN	2.64E-14	0.64359509	0.973	0.505	5.28E-11	Mast_cell
HEY1	2.75E-14	0.20396432	0.878	0.25	5.50E-11	Mast_cell
MEG3	2.97E-14	0.87904551	0.986	0.409	5.93E-11	Mast_cell
PTTG1	9.19E-14	0.30725942	0.824	0.234	1.84E-10	Mast_cell

CD37	1.24E-13	1.79731288	0.77	0.43	2.47E-10	Mast_cell
PKIB	2.07E-13	1.29422582	0.27	0.184	4.15E-10	Mast_cell
CXCL16	2.87E-13	1.5536534	0.784	0.354	5.74E-10	Mast_cell
SOCS2	3.46E-13	0.23771977	0.189	0.263	6.92E-10	Mast_cell
AREG	4.15E-13	2.04385523	0.743	0.21	8.30E-10	Mast_cell
IKZF3	7.35E-13	0.37243262	0.784	0.242	1.47E-09	Mast_cell
SOX4	8.12E-13	1.63803284	0.986	0.663	1.62E-09	Mast_cell
ASAH1	1.19E-12	1.35798017	0.919	0.655	2.37E-09	Mast_cell
CLU	3.51E-12	0.99331563	0.865	0.642	7.02E-09	Mast_cell
DHRS9	8.57E-12	0.56106712	0.946	0.391	1.71E-08	Mast_cell
ADGRE2	8.69E-12	0.97920948	0.27	0.3	1.74E-08	Mast_cell
LGALS3	1.30E-11	1.0259855	0.932	0.862	2.60E-08	Mast_cell
GLRX	1.70E-11	0.42730034	1	0.448	3.41E-08	Mast_cell
MLPH	3.63E-11	1.02395352	0.284	0.195	7.26E-08	Mast_cell
GPX1	1.73E-10	0.60908918	0.905	0.657	3.45E-07	Mast_cell
BHLHE40	2.23E-10	0.39091162	0.297	0.312	4.45E-07	Mast_cell
DUSP6	2.99E-10	0.61566204	0.919	0.43	5.97E-07	Mast_cell
MATK	3.93E-10	0.25138501	0.784	0.293	7.86E-07	Mast_cell
ADCYAP1	6.91E-10	3.14194859	0.649	0.061	1.38E-06	Mast_cell
RUNX3	7.20E-10	0.73009403	0.27	0.243	1.44E-06	Mast_cell
LYN	9.25E-10	0.20575184	0.351	0.508	1.85E-06	Mast_cell
CD9	1.41E-09	1.36910552	0.73	0.464	2.83E-06	Mast_cell
HHEX	4.53E-09	0.2864927	0.216	0.31	9.06E-06	Mast_cell
CCL4	4.99E-09	0.28063911	0.203	0.255	9.97E-06	Mast_cell
ITM2C	9.83E-09	0.96323975	0.811	0.402	1.97E-05	Mast_cell
TSPAN7	1.38E-08	0.24727712	0.757	0.265	2.77E-05	Mast_cell
SLC18A2	1.87E-08	2.51982551	0.622	0.046	3.75E-05	Mast_cell
SSR4	2.14E-08	1.10219952	0.811	0.728	4.28E-05	Mast_cell
OLR1	3.70E-08	0.22088904	0.716	0.224	7.40E-05	Mast_cell
FCER1A	5.32E-08	1.40340037	0.365	0.058	0.00010638	Mast_cell
DLC1	6.47E-08	0.35058494	1	0.535	0.00012948	Mast_cell
SDF2L1	7.62E-08	0.62444091	0.973	0.511	0.00015234	Mast_cell
IER3	1.31E-07	0.39548965	0.959	0.455	0.00026176	Mast_cell
IL1RL1	3.05E-07	2.10547346	0.649	0.205	0.00061013	Mast_cell
CTSD	3.52E-07	0.37609255	0.905	0.761	0.00070321	Mast_cell
APOC1	8.89E-07	0.2697014	0.311	0.348	0.00177749	Mast_cell
TNFSF10	1.74E-06	0.34271492	1	0.625	0.00348897	Mast_cell
RGS2	1.80E-06	1.77902057	0.649	0.283	0.00360153	Mast_cell
TMEM273	6.09E-06	0.23186341	0.757	0.393	0.01218972	Mast_cell
GLUL	1.03E-05	0.68570806	0.797	0.619	0.02052286	Mast_cell
SPINT2	1.15E-05	0.51415834	0.351	0.225	0.02300479	Mast_cell
PLIN2	1.22E-05	0.97200245	0.716	0.394	0.02449953	Mast_cell
TNFRSF4	1.47E-05	0.42500631	0.689	0.268	0.02942239	Mast_cell
TIMP1	1.52E-05	0.5263687	0.905	0.818	0.03035986	Mast_cell
TMEM176B	4.11E-05	1.86164522	0.635	0.347	0.08220189	Mast_cell
VEGFA	5.96E-05	0.60955305	0.378	0.429	0.11914978	Mast_cell
LXN	7.47E-05	1.2969741	0.338	0.257	0.14948756	Mast_cell
S100A10	9.59E-05	0.41173251	0.973	0.697	0.19176432	Mast_cell
CD84	0.00012247	1.15061815	0.689	0.45	0.24493878	Mast_cell
CD53	0.00015608	0.60018441	0.378	0.439	0.31215781	Mast_cell
CKS2	0.00015967	0.51945846	0.351	0.221	0.31933436	Mast_cell
ALDH1A1	0.0001756	0.45909299	0.419	0.523	0.35120824	Mast_cell
SOCS3	0.00018783	0.21133244	0.284	0.357	0.37565387	Mast_cell

CD83	0.00027038	1.31951475	0.635	0.322	0.54075026	Mast_cell
GRASP	0.0003549	0.44343957	0.662	0.282	0.70980001	Mast_cell
LAX1	0.00037529	0.67720779	0.595	0.241	0.75058371	Mast_cell
C17orf58	0.00040244	0.27782806	0.486	0.397	0.80487272	Mast_cell
NR4A2	0.00150481	0.53274318	0.716	0.373	1	Mast_cell
TWISTNB	0.00248951	0.74896983	0.405	0.353	1	Mast_cell
LCP2	0.00282613	0.88103208	0.419	0.409	1	Mast_cell
NCF4	0.00450911	0.64864048	0.662	0.361	1	Mast_cell
USP53	0.00517772	0.5005192	0.324	0.304	1	Mast_cell
PTGS2	0.00581529	1.80855218	0.351	0.165	1	Mast_cell
RHOBTB3	0.00627496	0.82651601	0.662	0.438	1	Mast_cell
AD000864.1	0.00723798	0.23473128	0.446	0.227	1	Mast_cell
TNFRSF17	0	1.29759009	0.6	0.014	0	pDC
IGLV1-47	0	0.70084915	0.357	0.002	0	pDC
AC104024.1	0	0.44895283	0.493	0.008	0	pDC
IGHV3-33	0	0.35877864	0.301	0.004	0	pDC
IGLV3-21	0	0.2964191	0.288	0.004	0	pDC
IGHV1-46	0	0.26235181	0.379	0.001	0	pDC
KLHL14	7.05E-170	0.39891842	0.464	0.074	1.41E-166	pDC
TNFRSF13B	5.94E-159	0.8060885	0.579	0.115	1.19E-155	pDC
POU2AF1	1.73E-154	0.87937345	0.592	0.115	3.46E-151	pDC
MYBL2	1.68E-153	1.10496224	0.64	0.175	3.36E-150	pDC
HIST1H3B	4.97E-147	0.79404858	0.528	0.075	9.94E-144	pDC
HIST1H3G	7.64E-147	0.58683377	0.512	0.101	1.53E-143	pDC
DLGAP5	7.95E-143	1.17358304	0.528	0.114	1.59E-139	pDC
HJURP	1.14E-126	0.98834807	0.56	0.106	2.29E-123	pDC
MKI67	1.80E-126	2.76261368	0.733	0.321	3.59E-123	pDC
DERL3	1.50E-125	1.86636437	0.659	0.169	3.00E-122	pDC
ASPM	1.78E-122	2.27718095	0.675	0.248	3.56E-119	pDC
SMC4	2.66E-122	1.68370349	0.843	0.336	5.33E-119	pDC
IGHV1-18	4.77E-120	0.59213661	0.368	0.017	9.53E-117	pDC
KNL1	7.12E-117	1.39761791	0.643	0.242	1.42E-113	pDC
ESCO2	1.81E-109	0.95883299	0.563	0.147	3.61E-106	pDC
CENPM	3.10E-107	1.18432973	0.629	0.207	6.20E-104	pDC
TOP2A	6.14E-106	2.68958983	0.728	0.379	1.23E-102	pDC
DIAPH3	1.21E-105	0.86609647	0.584	0.163	2.41E-102	pDC
BUB1	4.04E-105	0.84044643	0.603	0.222	8.07E-102	pDC
ANKRD36BF	5.84E-104	0.63538204	0.525	0.146	1.17E-100	pDC
IRF4	3.43E-99	0.92140081	0.581	0.186	6.86E-96	pDC
NCAPG	1.68E-98	1.11572384	0.613	0.227	3.37E-95	pDC
IGKV3-11	2.09E-94	0.48194525	0.373	0.022	4.17E-91	pDC
FOXM1	6.40E-93	0.62624496	0.52	0.135	1.28E-89	pDC
HMMR	6.60E-93	1.10975678	0.581	0.18	1.32E-89	pDC
SMC2	3.04E-91	1.42376112	0.763	0.381	6.07E-88	pDC
GTSE1	7.32E-91	1.18724957	0.624	0.259	1.46E-87	pDC
AURKB	2.72E-90	1.09345692	0.573	0.206	5.43E-87	pDC
LINC02362	3.41E-90	0.27526049	0.469	0.126	6.83E-87	pDC
AC012236.1	1.68E-88	0.3280072	0.405	0.032	3.37E-85	pDC
CD38	5.73E-88	1.29405485	0.696	0.353	1.15E-84	pDC
HELLS	7.18E-88	1.47081788	0.709	0.338	1.44E-84	pDC
EZH2	8.05E-88	1.27666265	0.688	0.343	1.61E-84	pDC
NUSAP1	2.53E-87	1.25338107	0.605	0.252	5.07E-84	pDC
JCHAIN	5.28E-87	5.46985546	0.653	0.311	1.06E-83	pDC

NUF2	1.46E-85	0.87151266	0.552	0.201	2.91E-82	pDC
BUB1B	6.58E-84	0.74353931	0.536	0.168	1.32E-80	pDC
SDF2L1	3.64E-83	1.37980806	0.845	0.508	7.29E-80	pDC
KIF11	3.82E-83	1.13536898	0.64	0.285	7.65E-80	pDC
BIRC5	3.95E-83	1.34197393	0.592	0.229	7.90E-80	pDC
AURKA	6.04E-82	0.52306614	0.496	0.156	1.21E-78	pDC
BRCA2	1.26E-81	1.11153039	0.701	0.407	2.52E-78	pDC
SPC24	2.68E-81	0.46929958	0.512	0.176	5.35E-78	pDC
STMN1	7.36E-81	2.67753636	0.765	0.355	1.47E-77	pDC
IGHV6-1	5.12E-79	0.30763527	0.283	0.016	1.02E-75	pDC
CEP55	6.10E-79	0.72476882	0.565	0.227	1.22E-75	pDC
ATAD2	2.17E-78	1.56178641	0.709	0.31	4.34E-75	pDC
SEC11C	1.67E-77	1.97780144	0.699	0.341	3.34E-74	pDC
UBE2T	9.12E-77	0.79445989	0.56	0.198	1.82E-73	pDC
PNOC	4.27E-76	0.27554326	0.389	0.022	8.54E-73	pDC
MAD2L1	5.14E-76	0.83689459	0.568	0.223	1.03E-72	pDC
RRM2	1.94E-74	1.8812352	0.637	0.319	3.88E-71	pDC
LMNB1	4.82E-73	1.11264099	0.648	0.307	9.64E-70	pDC
HMGB2	6.07E-73	2.30261655	0.755	0.388	1.21E-69	pDC
VPREB3	2.30E-72	0.38165726	0.443	0.115	4.60E-69	pDC
TACC3	5.07E-72	1.20879361	0.621	0.288	1.01E-68	pDC
SLAMF7	2.26E-71	1.02239065	0.669	0.381	4.52E-68	pDC
CDK1	6.74E-71	1.2190194	0.584	0.255	1.35E-67	pDC
CYTOR	1.17E-70	1.36162813	0.747	0.386	2.33E-67	pDC
IGHV4-39	5.84E-70	0.64839334	0.325	0.012	1.17E-66	pDC
TPX2	9.84E-69	1.53057165	0.669	0.35	1.97E-65	pDC
FKBP11	4.31E-65	1.90755185	0.605	0.256	8.63E-62	pDC
IGHV3-30	7.45E-65	0.68889798	0.368	0.049	1.49E-61	pDC
IGKV1-16	1.72E-64	0.47436491	0.349	0.05	3.45E-61	pDC
CENPE	2.32E-64	1.24640826	0.573	0.265	4.65E-61	pDC
KIF23	3.95E-64	0.81116075	0.568	0.243	7.89E-61	pDC
TYMS	5.84E-64	2.0829816	0.64	0.299	1.17E-60	pDC
DHFR	1.18E-62	0.94772918	0.648	0.321	2.35E-59	pDC
PIF1	4.89E-61	0.58759148	0.509	0.271	9.78E-58	pDC
FAM111B	7.54E-61	1.05840316	0.581	0.266	1.51E-57	pDC
ANLN	1.75E-59	0.6737535	0.507	0.205	3.50E-56	pDC
SHCBP1	4.46E-59	0.71169451	0.547	0.226	8.92E-56	pDC
IKZF3	2.57E-58	0.82279622	0.584	0.24	5.14E-55	pDC
IGHM	3.06E-56	7.40858474	0.709	0.575	6.13E-53	pDC
MXD3	3.65E-55	0.88490764	0.584	0.304	7.30E-52	pDC
ITM2C	6.46E-55	1.33712408	0.696	0.4	1.29E-51	pDC
HIST1H1D	1.47E-54	1.94108492	0.643	0.362	2.94E-51	pDC
IGLV1-44	2.43E-54	1.89476087	0.421	0.149	4.85E-51	pDC
CENPA	4.04E-54	0.37521903	0.427	0.126	8.09E-51	pDC
KIF14	1.17E-53	0.73234169	0.536	0.269	2.33E-50	pDC
XBP1	1.75E-52	2.26442637	0.744	0.501	3.50E-49	pDC
FAM83D	6.17E-51	0.23976131	0.411	0.145	1.23E-47	pDC
HIST1H1B	6.31E-51	1.91970214	0.571	0.299	1.26E-47	pDC
PBK	8.38E-51	0.69052952	0.368	0.094	1.68E-47	pDC
SGO1	1.02E-50	0.68146853	0.467	0.203	2.04E-47	pDC
IKZF1	1.88E-50	0.6647742	0.688	0.406	3.75E-47	pDC
IGKV3-20	1.19E-49	1.32651146	0.331	0.093	2.38E-46	pDC
CD79B	1.95E-49	0.75668785	0.629	0.389	3.90E-46	pDC

MZB1	2.18E-49	4.14175341	0.56	0.296	4.36E-46	pDC
FANCI	3.95E-49	0.94032068	0.6	0.37	7.91E-46	pDC
CCNB2	1.37E-48	0.67136051	0.472	0.184	2.74E-45	pDC
SSR4	2.57E-48	1.64130604	0.883	0.727	5.13E-45	pDC
ZWINT	3.32E-48	0.80244248	0.587	0.314	6.64E-45	pDC
CCNA2	4.50E-48	0.97412981	0.549	0.295	9.01E-45	pDC
TK1	1.04E-47	1.26029946	0.589	0.3	2.09E-44	pDC
CDKN3	6.79E-47	0.94934489	0.509	0.242	1.36E-43	pDC
HIST1H1C	2.18E-46	1.8331178	0.749	0.524	4.36E-43	pDC
KIF20B	3.90E-46	0.95297253	0.669	0.4	7.79E-43	pDC
KIF4A	3.92E-46	0.46281874	0.443	0.141	7.85E-43	pDC
TENT5C	5.94E-46	0.96175938	0.52	0.22	1.19E-42	pDC
DEPDC1	1.04E-45	0.40249974	0.408	0.112	2.08E-42	pDC
KIFC1	6.89E-45	1.05814798	0.507	0.248	1.38E-41	pDC
CYFIP2	1.38E-44	0.35318333	0.595	0.308	2.75E-41	pDC
TROAP	1.49E-44	0.39282654	0.392	0.087	2.99E-41	pDC
IGKC	7.37E-44	8.39026793	0.733	0.586	1.47E-40	pDC
ISG20	9.95E-43	1.20171914	0.611	0.341	1.99E-39	pDC
KIF15	1.10E-42	0.57734996	0.477	0.212	2.20E-39	pDC
NDC80	3.89E-42	0.88122184	0.573	0.345	7.78E-39	pDC
PRC1	5.13E-42	0.40817262	0.461	0.221	1.03E-38	pDC
CD320	9.64E-41	0.26655713	0.667	0.372	1.93E-37	pDC
CPNE5	1.11E-40	0.76107598	0.56	0.325	2.21E-37	pDC
ASF1B	2.13E-40	0.76601778	0.515	0.245	4.25E-37	pDC
IGHV3-15	3.73E-40	0.82673032	0.309	0.073	7.45E-37	pDC
MCM4	3.20E-39	0.90024112	0.637	0.4	6.39E-36	pDC
AC243960.1	1.97E-38	0.62709498	0.509	0.288	3.94E-35	pDC
FCRL5	3.95E-38	0.83051212	0.419	0.093	7.90E-35	pDC
CLSPN	4.91E-38	1.49326174	0.544	0.285	9.82E-35	pDC
PRDM1	6.35E-38	0.78975837	0.549	0.288	1.27E-34	pDC
RASGRP3	1.22E-37	0.2818703	0.547	0.377	2.45E-34	pDC
TTK	2.10E-37	0.62661777	0.44	0.181	4.21E-34	pDC
CYBA	3.16E-37	0.55373942	0.936	0.76	6.31E-34	pDC
ITGA4	3.41E-37	0.71816289	0.691	0.46	6.82E-34	pDC
APOLD1	4.96E-37	0.45400816	0.541	0.278	9.93E-34	pDC
PRR11	8.25E-36	0.55381355	0.539	0.348	1.65E-32	pDC
KIF20A	4.58E-35	0.4371769	0.461	0.238	9.16E-32	pDC
CCNB1	6.85E-35	0.69134212	0.461	0.266	1.37E-31	pDC
CD79A	7.61E-35	1.56942319	0.488	0.206	1.52E-31	pDC
TFRC	1.04E-34	0.3350877	0.579	0.308	2.08E-31	pDC
CD27	5.27E-34	1.11273926	0.493	0.209	1.05E-30	pDC
PRF1	9.26E-34	0.4613122	0.429	0.205	1.85E-30	pDC
CCL5	1.48E-33	0.31640067	0.493	0.248	2.95E-30	pDC
AQP3	1.59E-33	0.42475082	0.419	0.21	3.19E-30	pDC
HMGA1	2.52E-32	0.5722587	0.619	0.385	5.05E-29	pDC
IGLV2-14	3.30E-31	0.69291681	0.307	0.08	6.59E-28	pDC
EZR	4.87E-31	0.65959879	0.651	0.346	9.73E-28	pDC
MCM7	6.21E-31	1.15702137	0.632	0.397	1.24E-27	pDC
IGHV3-23	1.10E-30	1.12571298	0.336	0.115	2.20E-27	pDC
HCLS1	2.47E-30	0.22329487	0.669	0.386	4.95E-27	pDC
RAD51AP1	1.52E-29	0.73876823	0.539	0.345	3.04E-26	pDC
HIST1H4C	3.41E-29	2.66395218	0.779	0.61	6.83E-26	pDC
SPC25	4.35E-29	1.03672231	0.483	0.229	8.69E-26	pDC

CD48	1.73E-28	0.21204757	0.581	0.364	3.45E-25	pDC
IGHV3-43	2.11E-28	0.71577086	0.253	0.025	4.22E-25	pDC
ZBP1	8.78E-28	0.60266119	0.451	0.246	1.76E-24	pDC
CENPF	4.36E-27	2.39284099	0.485	0.28	8.73E-24	pDC
PTTG1	4.51E-27	1.25090782	0.451	0.233	9.02E-24	pDC
NT5DC2	7.00E-27	0.31763334	0.725	0.527	1.40E-23	pDC
PIM2	1.36E-26	0.72151511	0.432	0.199	2.71E-23	pDC
E2F8	1.41E-26	0.4103761	0.352	0.116	2.81E-23	pDC
RNU2-63P	2.22E-25	0.53567744	0.552	0.439	4.44E-22	pDC
LAX1	5.60E-25	0.63365897	0.453	0.24	1.12E-21	pDC
MS4A1	4.60E-24	0.23586876	0.299	0.132	9.20E-21	pDC
ADA	4.60E-24	0.22438087	0.533	0.383	9.21E-21	pDC
UBE2C	1.74E-22	1.3803398	0.397	0.158	3.47E-19	pDC
PCNA	2.86E-22	1.48026862	0.523	0.312	5.71E-19	pDC
RAC2	3.78E-22	0.9780271	0.573	0.386	7.55E-19	pDC
PKMYT1	4.20E-22	0.64608078	0.389	0.128	8.40E-19	pDC
PTPN6	5.09E-21	0.30016985	0.605	0.452	1.02E-17	pDC
CDC20	5.11E-21	0.88506138	0.459	0.293	1.02E-17	pDC
PIM1	1.00E-20	0.25581429	0.496	0.306	2.01E-17	pDC
LSP1	2.34E-20	0.35724731	0.752	0.586	4.68E-17	pDC
ARHGAP30	2.78E-20	0.41921576	0.624	0.462	5.56E-17	pDC
TUBA1C	4.76E-20	0.66758348	0.723	0.507	9.52E-17	pDC
GMFG	1.44E-19	0.32453643	0.667	0.454	2.88E-16	pDC
CKS2	2.47E-19	0.6754511	0.432	0.219	4.94E-16	pDC
IGHV3-74	1.86E-18	0.51705647	0.251	0.023	3.73E-15	pDC
PCLAF	5.07E-18	1.79266417	0.491	0.289	1.01E-14	pDC
KIF2C	5.77E-18	0.60589069	0.397	0.223	1.15E-14	pDC
ARL4C	6.21E-17	0.39256696	0.493	0.292	1.24E-13	pDC
IGHA1	1.06E-16	6.19036638	0.467	0.347	2.13E-13	pDC
FAM30A	3.04E-16	0.75024462	0.328	0.107	6.08E-13	pDC
SKA3	5.16E-15	0.32860563	0.384	0.174	1.03E-11	pDC
KPNA2	1.30E-14	0.72025212	0.368	0.199	2.60E-11	pDC
PTPRC	2.88E-14	0.49879731	0.704	0.56	5.75E-11	pDC
IGHG3	7.21E-14	5.07838384	0.405	0.307	1.44E-10	pDC
NDUFA4L2	1.50E-13	0.39961289	0.624	0.477	3.00E-10	pDC
AL031777.3	1.73E-13	0.24127365	0.504	0.401	3.45E-10	pDC
PRDX4	6.34E-13	0.62626504	0.624	0.535	1.27E-09	pDC
IGLL5	8.23E-13	2.78046573	0.267	0.089	1.65E-09	pDC
CENPU	6.27E-12	0.87340286	0.392	0.227	1.25E-08	pDC
IGLV6-57	1.21E-11	0.98698962	0.251	0.039	2.42E-08	pDC
IRF1	2.73E-11	0.47773408	0.688	0.556	5.46E-08	pDC
IGLC2	2.88E-11	7.8652749	0.496	0.414	5.76E-08	pDC
IGLV2-11	1.38E-10	0.40560445	0.256	0.102	2.75E-07	pDC
HMGB3	7.87E-10	0.68529304	0.435	0.36	1.57E-06	pDC
CENPW	1.16E-09	0.60179686	0.501	0.396	2.33E-06	pDC
ST6GAL1	1.36E-09	0.25777434	0.461	0.334	2.71E-06	pDC
RUNX3	2.44E-09	0.55215722	0.395	0.241	4.87E-06	pDC
CKAP2L	4.89E-09	0.9375777	0.365	0.193	9.77E-06	pDC
CENPK	2.02E-08	0.94054659	0.397	0.292	4.05E-05	pDC
TRIB1	3.66E-08	0.30405693	0.472	0.399	7.32E-05	pDC
CORO1A	4.50E-08	1.11024983	0.571	0.491	9.00E-05	pDC
DDIT4	5.90E-08	0.24133155	0.517	0.4	0.00011805	pDC
IGHG4	7.14E-08	4.63958734	0.352	0.277	0.00014284	pDC

BHLHE41	8.08E-08	0.20872425	0.416	0.306	0.00016161	pDC
CKAP2	1.26E-07	0.89615153	0.435	0.362	0.00025115	pDC
PTPN7	6.18E-07	0.6784492	0.429	0.318	0.00123558	pDC
IGHG1	1.85E-06	4.69604725	0.307	0.202	0.00369707	pDC
CXCR4	2.83E-06	0.25420012	0.469	0.357	0.00566048	pDC
CDCA8	4.12E-06	0.68371673	0.357	0.253	0.0082468	pDC
MELK	1.03E-05	0.5888379	0.301	0.139	0.02058924	pDC
PCED1B-AS1	1.19E-05	0.23154016	0.387	0.268	0.0237814	pDC
E2F1	1.32E-05	0.76073941	0.381	0.3	0.02637613	pDC
MATK	2.09E-05	0.30861122	0.219	0.294	0.0418239	pDC
BIRC3	3.19E-05	0.23528669	0.459	0.367	0.0637857	pDC
IGLC3	0.00011068	6.49219493	0.288	0.206	0.22136564	pDC
CD52	0.00012894	0.4357416	0.557	0.496	0.25787499	pDC
NEK2	0.00013859	0.31034386	0.261	0.188	0.27718472	pDC
CTSW	0.00026685	0.53111067	0.197	0.255	0.53369272	pDC
MCM10	0.000332	0.2962686	0.347	0.194	0.66400863	pDC
GINS2	0.0010358	0.69862489	0.387	0.337	1	pDC
LCP1	0.00200881	0.4889566	0.64	0.607	1	pDC
PTN	0.00269112	0.38149695	0.235	0.354	1	pDC
TRBC2	0.00427291	0.67782357	0.296	0.22	1	pDC
DUSP5	0.00777596	0.26454685	0.28	0.216	1	pDC
GPM6B	4.82E-291	4.37304704	0.922	0.224	9.64E-288	Neuron
S100B	2.36E-262	5.36877801	0.875	0.276	4.72E-259	Neuron
PLP1	1.45E-212	3.95870417	0.842	0.401	2.90E-209	Neuron
NCMAP	9.99E-205	1.24599226	0.326	0.025	2.00E-201	Neuron
COL28A1	1.65E-197	2.83534551	0.717	0.234	3.30E-194	Neuron
CDH19	1.22E-196	3.58743757	0.745	0.166	2.44E-193	Neuron
NRXN1	1.31E-196	4.18752893	0.77	0.3	2.61E-193	Neuron
PCSK2	7.60E-190	1.78196725	0.474	0.095	1.52E-186	Neuron
RELN	6.27E-176	2.08877045	0.526	0.09	1.25E-172	Neuron
KIF1A	1.30E-172	1.16004721	0.363	0.026	2.59E-169	Neuron
CRYAB	1.10E-163	2.62734188	0.836	0.463	2.19E-160	Neuron
LGI4	5.33E-157	2.96503292	0.819	0.535	1.07E-153	Neuron
BCAS1	2.45E-156	1.96996516	0.396	0.058	4.90E-153	Neuron
POU3F2	2.14E-154	0.3786513	0.257	0.015	4.29E-151	Neuron
MPZ	3.09E-154	5.80675723	0.752	0.403	6.18E-151	Neuron
PMP22	2.95E-151	3.08031118	0.846	0.569	5.90E-148	Neuron
ERBB3	1.35E-150	2.70584417	0.708	0.336	2.70E-147	Neuron
PMP2	1.15E-146	2.64081136	0.507	0.137	2.31E-143	Neuron
SOX10	1.30E-140	2.4686015	0.591	0.194	2.60E-137	Neuron
GJC3	4.57E-134	1.68177926	0.388	0.051	9.14E-131	Neuron
FA2H	4.38E-131	0.60707673	0.267	0.002	8.76E-128	Neuron
ANK3	8.67E-123	2.4295157	0.626	0.236	1.73E-119	Neuron
SORCS1	1.18E-120	2.17947741	0.497	0.08	2.35E-117	Neuron
XKR4	3.44E-109	2.234359	0.509	0.178	6.88E-106	Neuron
MAL	6.31E-109	2.98469627	0.663	0.313	1.26E-105	Neuron
CTNNAL1	2.99E-108	2.47183752	0.641	0.3	5.98E-105	Neuron
CD9	2.37E-102	1.99434708	0.737	0.461	4.74E-99	Neuron
SEMA3B	3.08E-95	2.47465061	0.643	0.35	6.15E-92	Neuron
GPR37L1	1.13E-94	0.79707323	0.314	0.064	2.25E-91	Neuron
UGT8	4.55E-92	1.37602228	0.353	0.06	9.09E-89	Neuron
SCN7A	7.75E-87	2.96717311	0.544	0.228	1.55E-83	Neuron
VWA1	5.60E-83	2.32820551	0.634	0.388	1.12E-79	Neuron

LAMB1	2.71E-82	1.9416825	0.665	0.394	5.42E-79	Neuron
AATK	7.57E-82	2.56600448	0.585	0.293	1.51E-78	Neuron
SHC4	9.03E-82	1.56680913	0.444	0.18	1.81E-78	Neuron
FGL2	3.42E-79	2.27188216	0.735	0.525	6.85E-76	Neuron
SOX2	9.41E-79	1.92959568	0.452	0.154	1.88E-75	Neuron
CDH7	1.79E-72	1.0989601	0.341	0.098	3.59E-69	Neuron
S100A10	7.62E-72	1.3694765	0.84	0.696	1.52E-68	Neuron
STMN1	1.43E-71	1.87448213	0.612	0.356	2.86E-68	Neuron
MAG	8.45E-71	1.44972728	0.388	0.116	1.69E-67	Neuron
GAS7	3.56E-66	1.87146258	0.692	0.535	7.12E-63	Neuron
SPARC	7.51E-66	1.30307233	0.823	0.759	1.50E-62	Neuron
SLITRK6	5.20E-65	1.20210832	0.31	0.091	1.04E-61	Neuron
CHL1	3.17E-64	2.74689929	0.554	0.338	6.34E-61	Neuron
PTPRZ1	3.41E-64	1.53567407	0.413	0.186	6.82E-61	Neuron
PRX	1.48E-63	2.67258216	0.419	0.2	2.97E-60	Neuron
CADM3	3.31E-63	1.85328251	0.587	0.387	6.63E-60	Neuron
CLDN19	3.84E-62	2.00384366	0.435	0.189	7.69E-59	Neuron
GAS2L3	3.69E-60	2.25209568	0.556	0.368	7.38E-57	Neuron
ITGA6	1.08E-58	1.64442515	0.55	0.311	2.15E-55	Neuron
KCNK12	3.04E-52	1.34026869	0.409	0.209	6.08E-49	Neuron
TMEM176B	2.20E-50	2.14778388	0.522	0.345	4.39E-47	Neuron
SCD	3.98E-45	1.76760248	0.544	0.389	7.95E-42	Neuron
IQGAP2	8.87E-45	1.36910516	0.554	0.384	1.77E-41	Neuron
MATN2	1.73E-44	1.70851938	0.561	0.42	3.46E-41	Neuron
MLIP	2.27E-44	1.3799966	0.372	0.17	4.53E-41	Neuron
AZGP1	6.66E-43	1.91048879	0.402	0.176	1.33E-39	Neuron
ATP1A2	4.39E-42	1.56258776	0.598	0.504	8.79E-39	Neuron
KIF19	6.29E-41	0.95270508	0.335	0.159	1.26E-37	Neuron
CLU	6.36E-41	0.77449619	0.776	0.64	1.27E-37	Neuron
FIBIN	2.37E-40	0.71020644	0.331	0.214	4.74E-37	Neuron
LIMCH1	4.20E-40	1.93955048	0.439	0.274	8.39E-37	Neuron
RCAN1	4.65E-40	1.10586764	0.407	0.255	9.29E-37	Neuron
SOX8	4.97E-40	1.03106398	0.316	0.152	9.94E-37	Neuron
MT2A	2.35E-39	0.78333564	0.883	0.879	4.70E-36	Neuron
SORBS1	6.95E-38	1.34778867	0.507	0.364	1.39E-34	Neuron
PLLP	1.53E-37	2.0029109	0.446	0.265	3.05E-34	Neuron
ITGB8	3.15E-37	2.63935947	0.503	0.365	6.30E-34	Neuron
GLDN	8.92E-36	1.45166387	0.388	0.248	1.78E-32	Neuron
ANGPTL7	1.53E-34	0.6870044	0.3	0.203	3.05E-31	Neuron
KRT14	2.37E-34	0.4040201	0.31	0.16	4.73E-31	Neuron
MARCKS	4.53E-34	0.78375521	0.858	0.817	9.06E-31	Neuron
FGFBP2	9.20E-34	0.29862783	0.267	0.159	1.84E-30	Neuron
CNKSR3	1.09E-33	0.78916653	0.357	0.258	2.18E-30	Neuron
TNFAIP6	6.57E-33	1.08603796	0.446	0.336	1.31E-29	Neuron
RDX	3.30E-32	1.2128554	0.649	0.58	6.60E-29	Neuron
EGR2	3.38E-32	0.47978965	0.298	0.193	6.76E-29	Neuron
ITGB4	3.44E-31	1.53845691	0.441	0.32	6.87E-28	Neuron
SCN9A	1.66E-30	1.43029243	0.429	0.32	3.33E-27	Neuron
S100A1	4.84E-30	0.76435079	0.271	0.159	9.68E-27	Neuron
MBP	5.39E-30	3.03091948	0.548	0.459	1.08E-26	Neuron
NR4A2	1.49E-29	1.11939317	0.505	0.372	2.98E-26	Neuron
VGLL3	4.57E-28	1.17870918	0.413	0.32	9.14E-25	Neuron
NLGN4X	7.81E-28	1.11097951	0.32	0.215	1.56E-24	Neuron

ABCA8	2.03E-26	1.05966822	0.68	0.623	4.07E-23	Neuron
RETREG1	5.38E-26	1.66602453	0.353	0.212	1.08E-22	Neuron
PDLIM4	2.21E-25	1.91316124	0.409	0.292	4.41E-22	Neuron
OAS3	2.38E-25	0.2009161	0.372	0.599	4.76E-22	Neuron
SPTBN1	7.49E-25	1.02459816	0.69	0.677	1.50E-21	Neuron
RGCC	8.66E-25	0.89082685	0.331	0.206	1.73E-21	Neuron
CLMN	3.74E-23	0.20622627	0.234	0.533	7.48E-20	Neuron
THSD7A	4.23E-23	1.10166918	0.423	0.328	8.46E-20	Neuron
EFNB2	6.73E-23	0.30051638	0.3	0.224	1.35E-19	Neuron
LSAMP	8.94E-23	0.95800988	0.322	0.228	1.79E-19	Neuron
CASKIN2	5.18E-22	0.92757333	0.402	0.316	1.04E-18	Neuron
SPHK1	1.53E-21	0.75094481	0.343	0.292	3.06E-18	Neuron
SEMA5A	1.16E-20	1.05308551	0.489	0.424	2.33E-17	Neuron
ADAMTS9	1.54E-20	0.74277729	0.363	0.303	3.09E-17	Neuron
SLC12A2	5.95E-20	0.34704728	0.335	0.278	1.19E-16	Neuron
LMO7	6.57E-20	0.51889045	0.285	0.236	1.31E-16	Neuron
PCDH1	9.20E-20	0.27536362	0.179	0.41	1.84E-16	Neuron
C1QL1	1.16E-19	0.69626996	0.296	0.168	2.33E-16	Neuron
RARRES2	2.20E-19	1.18388291	0.614	0.638	4.41E-16	Neuron
TMEM176A	6.06E-19	1.60998793	0.396	0.334	1.21E-15	Neuron
PTN	6.47E-19	0.39869141	0.421	0.352	1.29E-15	Neuron
EDNRB	6.75E-19	0.35106361	0.283	0.252	1.35E-15	Neuron
COL4A2	8.66E-19	0.57344554	0.727	0.674	1.73E-15	Neuron
COL5A3	6.55E-18	0.87714239	0.427	0.383	1.31E-14	Neuron
PCDH9	1.65E-17	1.55586226	0.355	0.287	3.30E-14	Neuron
LINC00632	3.93E-17	1.02166635	0.55	0.517	7.85E-14	Neuron
NTM	4.79E-17	1.46317805	0.417	0.301	9.58E-14	Neuron
ADGRG6	1.17E-16	0.60141861	0.279	0.245	2.34E-13	Neuron
GABRA2	5.60E-16	0.66181091	0.339	0.274	1.12E-12	Neuron
KANK4	6.91E-16	1.34303624	0.402	0.343	1.38E-12	Neuron
ADGRL3	7.76E-16	0.52260815	0.224	0.435	1.55E-12	Neuron
TUBB2B	2.81E-15	1.56524769	0.329	0.239	5.62E-12	Neuron
MIA	5.42E-15	1.17769959	0.251	0.11	1.08E-11	Neuron
SFRP5	1.08E-14	1.42935342	0.292	0.136	2.16E-11	Neuron
MDK	1.18E-14	1.66192416	0.511	0.531	2.37E-11	Neuron
GRASP	1.24E-14	0.58206819	0.322	0.282	2.49E-11	Neuron
MT1E	2.50E-14	0.75011696	0.628	0.597	5.00E-11	Neuron
NDRG1	2.59E-14	1.0457817	0.474	0.453	5.18E-11	Neuron
HSPG2	2.68E-14	0.82819684	0.659	0.691	5.36E-11	Neuron
COL4A1	3.65E-14	0.52213362	0.661	0.599	7.30E-11	Neuron
MT3	7.21E-14	1.73646257	0.337	0.261	1.44E-10	Neuron
NR2F2	1.64E-13	0.59629837	0.721	0.733	3.28E-10	Neuron
USP53	2.94E-13	0.72455339	0.355	0.304	5.89E-10	Neuron
IFI6	2.95E-13	0.88509209	0.739	0.83	5.90E-10	Neuron
NEDD9	3.28E-13	0.40088566	0.318	0.279	6.57E-10	Neuron
COL5A2	3.47E-13	0.30916263	0.269	0.516	6.94E-10	Neuron
FASN	4.66E-13	0.31815257	0.144	0.35	9.32E-10	Neuron
PPFIBP1	1.22E-12	0.76072426	0.427	0.398	2.43E-09	Neuron
PLAT	3.83E-12	0.78651324	0.446	0.4	7.66E-09	Neuron
LPL	1.51E-11	1.28979774	0.271	0.223	3.03E-08	Neuron
TJP1	1.71E-11	0.86999465	0.522	0.529	3.42E-08	Neuron
PAPPA	5.03E-11	0.21144159	0.078	0.262	1.01E-07	Neuron
CRTAC1	6.25E-11	0.21113416	0.086	0.259	1.25E-07	Neuron

GPM6A	8.61E-11	0.2899921	0.3	0.308	1.72E-07	Neuron
DAB1	1.06E-10	0.4278499	0.279	0.25	2.12E-07	Neuron
IFIH1	1.76E-10	0.22479835	0.32	0.498	3.53E-07	Neuron
FGF7	2.56E-10	0.72891505	0.439	0.451	5.12E-07	Neuron
CARD10	8.35E-10	0.27134938	0.158	0.353	1.67E-06	Neuron
L1CAM	1.56E-09	1.74712463	0.329	0.258	3.13E-06	Neuron
ECE1	1.62E-09	0.28514314	0.265	0.488	3.24E-06	Neuron
RDH10	3.00E-09	1.13956005	0.285	0.255	6.01E-06	Neuron
TTYH1	9.11E-09	1.77371237	0.363	0.327	1.82E-05	Neuron
SERPINE2	1.10E-08	0.32677842	0.175	0.361	2.19E-05	Neuron
LAMA4	1.32E-08	0.87793051	0.581	0.605	2.64E-05	Neuron
NES	2.72E-08	0.64468654	0.439	0.429	5.44E-05	Neuron
BHLHE40	4.74E-08	0.4081514	0.304	0.312	9.47E-05	Neuron
ENTPD2	6.59E-08	0.5936437	0.193	0.32	0.00013172	Neuron
COL15A1	1.12E-07	0.28783917	0.353	0.364	0.00022399	Neuron
IL11RA	1.19E-07	0.21124557	0.318	0.323	0.000237	Neuron
SLIT2	6.73E-07	0.3949732	0.259	0.45	0.00134578	Neuron
LTBP4	7.58E-07	0.82966351	0.526	0.55	0.00151677	Neuron
NRP2	7.59E-07	1.01923422	0.402	0.415	0.00151777	Neuron
LAMA2	1.18E-06	0.21011993	0.337	0.34	0.00236657	Neuron
L1TD1	1.28E-06	0.20070078	0.195	0.353	0.00256571	Neuron
LGALS3	1.46E-06	0.43518987	0.758	0.863	0.00291151	Neuron
TIMP3	2.07E-06	0.24577152	0.752	0.771	0.00414312	Neuron
MARCKSL1	2.37E-06	0.28979906	0.324	0.318	0.00473991	Neuron
SGCD	3.13E-06	0.41524818	0.294	0.309	0.00626747	Neuron
SMTN	3.64E-06	0.66808173	0.458	0.491	0.00727307	Neuron
IFIT1	6.27E-06	0.59858155	0.46	0.642	0.0125481	Neuron
PEG10	1.01E-05	0.79468295	0.275	0.452	0.02018444	Neuron
TCIM	1.18E-05	1.08184767	0.228	0.397	0.02357681	Neuron
MAF	2.05E-05	0.46865935	0.587	0.602	0.04107727	Neuron
CCL2	3.04E-05	0.20166763	0.175	0.354	0.06074563	Neuron
MX2	3.56E-05	0.41759788	0.402	0.565	0.07124203	Neuron
SLC15A3	6.35E-05	0.84959458	0.296	0.499	0.12706439	Neuron
NRN1	0.00010749	1.4467467	0.363	0.369	0.21497778	Neuron
FSTL3	0.00014131	1.03424734	0.277	0.296	0.28261188	Neuron
ID4	0.00014792	0.50111852	0.312	0.324	0.29584105	Neuron
COL21A1	0.00021247	0.76246756	0.205	0.365	0.42493879	Neuron
DUSP6	0.00025671	0.29935619	0.407	0.431	0.51341583	Neuron
ITM2C	0.00035672	0.46818135	0.38	0.403	0.71343296	Neuron
OAF	0.00050339	0.25712994	0.349	0.51	1	Neuron
CDKN1C	0.0006962	0.44436749	0.466	0.508	1	Neuron
RAPGEF5	0.00079572	0.73913418	0.3	0.357	1	Neuron
PTPRE	0.00090069	0.21014838	0.4	0.43	1	Neuron
MAP1B	0.00095876	0.27103161	0.534	0.565	1	Neuron
SOX4	0.00099837	0.78906155	0.591	0.664	1	Neuron
RSAD2	0.00099857	0.52703129	0.374	0.556	1	Neuron
AIF1L	0.00103942	0.40451371	0.368	0.429	1	Neuron
LIMS2	0.00135797	0.53654874	0.454	0.506	1	Neuron
MEG3	0.00212144	0.46825542	0.357	0.411	1	Neuron
HES1	0.00223358	0.50880154	0.402	0.448	1	Neuron
ALCAM	0.00268995	1.39485477	0.37	0.415	1	Neuron
TSPAN8	0.0029257	0.70147729	0.232	0.287	1	Neuron
FXYD6	0.00321574	0.60774091	0.433	0.499	1	Neuron

PRSS23	0.00346016	0.82263853	0.283	0.324	1	Neuron
DKK3	0.00451917	0.62196361	0.304	0.339	1	Neuron
ISG15	0.00641625	0.74991664	0.719	0.847	1	Neuron
KCTD12	0.00711577	0.36704693	0.587	0.649	1	Neuron

Table S6. Differentially expressed genes in each smooth muscle cell cluster (GSE213740)

gene	p_val	avg_log2FC	pct.1	pct.2	p_val_adj	cluster
RERGL	0	2.01972764	0.957	0.677	0	1
NDUFA4L2	0	1.80894832	0.791	0.546	0	1
ADIRF	0	1.72590908	0.999	0.899	0	1
NET1	0	1.4830467	0.92	0.664	0	1
PHLDA2	0	1.40548215	0.861	0.608	0	1
CRIP1	0	1.23607322	0.967	0.822	0	1
SNCG	0	1.21234096	0.877	0.76	0	1
TPM2	0	1.21081637	1	0.98	0	1
CASQ2	0	1.15364507	0.819	0.654	0	1
MYL9	0	1.12742643	0.999	0.951	0	1
ACTA2	0	1.10261534	1	0.975	0	1
DSTN	0	1.08432607	0.999	0.94	0	1
PLN	0	1.06818913	0.886	0.6	0	1
CCND1	0	1.04884934	0.884	0.774	0	1
C11orf96	0	1.02736125	0.989	0.869	0	1
KCNAB1	0	1.01202296	0.817	0.666	0	1
TBX2	0	0.99950046	0.887	0.66	0	1
CAVIN3	0	0.94319813	0.967	0.905	0	1
CSRP1	0	0.93256249	0.95	0.734	0	1
TAGLN	0	0.92436538	1	0.99	0	1
BCAM	0	0.90543973	0.972	0.827	0	1
ITIH5	0	0.87844581	0.911	0.72	0	1
CRIP2	0	0.87333125	0.893	0.721	0	1
HES4	0	0.75470345	0.931	0.828	0	1
AP002956.1	0	0.69942521	0.993	0.936	0	1
SORBS2	0	0.63724843	0.946	0.865	0	1
KIT	0	-0.2617388	0.08	0.463	0	1
GRASP	0	-0.2784079	0.082	0.447	0	1
PRSS35	0	-0.2791121	0.157	0.522	0	1
CNTFR	0	-0.3734615	0.114	0.519	0	1
UPP1	0	-0.4018289	0.098	0.46	0	1
CADM3	0	-0.4430107	0.167	0.564	0	1
PI15	0	-0.4612319	0.277	0.567	0	1
PPL	0	-0.4758765	0.145	0.571	0	1
TCIM	0	-0.4870726	0.143	0.516	0	1
PDGFRL	0	-0.5122865	0.163	0.554	0	1
LOX	0	-0.5185955	0.13	0.528	0	1
IGSF10	0	-0.5380201	0.198	0.575	0	1
VGLL3	0	-0.5455668	0.167	0.571	0	1
SEMA3C	0	-0.5833879	0.119	0.508	0	1
SPOCK1	0	-0.6273012	0.101	0.516	0	1
ABCA10	0	-0.6583128	0.27	0.595	0	1
FLNB	0	-0.6783537	0.139	0.506	0	1
FLRT2	0	-0.6792914	0.259	0.646	0	1
ABCA6	0	-0.6912773	0.192	0.562	0	1
ABCA9	0	-0.710111	0.184	0.56	0	1
SPON2	0	-0.7163383	0.243	0.612	0	1
SEMA5A	0	-0.729152	0.321	0.689	0	1
OAF	0	-0.7579667	0.321	0.691	0	1
SSTR2	0	-0.765243	0.135	0.541	0	1
MATN2	0	-0.7836453	0.211	0.592	0	1

PROCR	0	-0.8140024	0.22	0.627	0	1
RARRES1	0	-0.8379143	0.149	0.558	0	1
FGF7	0	-0.866919	0.32	0.658	0	1
EMP1	0	-0.9166717	0.128	0.585	0	1
CHRDL1	0	-0.9222883	0.209	0.688	0	1
ABCC9	0	-0.9725013	0.374	0.748	0	1
GPC3	0	-1.1049402	0.206	0.709	0	1
KRT18	0	-1.1323853	0.126	0.506	0	1
MFAP5	0	-1.212916	0.132	0.539	0	1
TSHZ2	0	-1.2548687	0.303	0.701	0	1
CLSTN2	0	-1.4070941	0.262	0.727	0	1
SFRP2	0	-1.4356823	0.213	0.602	0	1
PODN	0	-1.490976	0.308	0.752	0	1
H19	0	-1.5133492	0.338	0.734	0	1
ABCA8	0	-1.5594286	0.265	0.745	0	1
GGT5	0	-1.7197575	0.206	0.648	0	1
COL6A3	0	-1.8012169	0.236	0.818	0	1
COL1A1	0	-2.0151496	0.775	0.965	0	1
C1S	0	-2.1985092	0.294	0.771	0	1
C1R	0	-2.2768905	0.355	0.732	0	1
CFD	0	-3.0854073	0.5	0.844	0	1
HELLPAR	5.85E-307	-0.3723109	0.265	0.557	1.17E-303	1
HAS2	3.88E-305	-0.2913947	0.189	0.498	7.76E-302	1
GJA4	2.05E-303	0.97154978	0.786	0.643	4.11E-300	1
BNC2	3.25E-303	-0.2641944	0.221	0.514	6.51E-300	1
BICC1	3.40E-303	-0.4541173	0.154	0.509	6.80E-300	1
FRZB	7.56E-303	0.7385206	0.627	0.26	1.51E-299	1
NRARP	2.05E-302	1.18408548	0.732	0.581	4.09E-299	1
PPP1R14A	4.88E-301	0.7803222	0.971	0.841	9.76E-298	1
CCL26	6.83E-295	-0.5444953	0.226	0.549	1.37E-291	1
TGFBR3	1.49E-293	-0.8530683	0.317	0.67	2.98E-290	1
LHFPL2	2.21E-292	-0.2598292	0.073	0.419	4.41E-289	1
COL3A1	1.30E-290	-1.6777121	0.797	0.957	2.61E-287	1
FST	4.97E-285	-0.7306915	0.227	0.59	9.94E-282	1
RCAN2	6.51E-285	0.86061457	0.769	0.542	1.30E-281	1
WFDC1	6.95E-284	1.05319766	0.655	0.432	1.39E-280	1
CSRP2	2.40E-283	1.04629317	0.801	0.63	4.80E-280	1
TNFAIP2	8.64E-280	-0.5201737	0.208	0.556	1.73E-276	1
COX4I2	3.27E-278	1.18244395	0.796	0.667	6.53E-275	1
TUBA1C	3.06E-277	1.01640339	0.781	0.63	6.13E-274	1
NID1	1.86E-270	-0.9475141	0.295	0.679	3.73E-267	1
TMEM176B	5.81E-270	-0.8890615	0.226	0.575	1.16E-266	1
HMCN2	1.32E-268	-0.6828058	0.206	0.56	2.64E-265	1
OSR2	6.12E-266	-0.7299021	0.177	0.544	1.22E-262	1
SLIT2	1.54E-264	-0.568251	0.127	0.484	3.08E-261	1
MFGE8	3.37E-264	0.65290653	0.975	0.766	6.74E-261	1
BGN	6.17E-264	0.65686025	0.932	0.707	1.23E-260	1
CD34	7.56E-261	-0.4914236	0.158	0.515	1.51E-257	1
GAS1	1.32E-258	-0.6782146	0.273	0.585	2.64E-255	1
TPM1	2.77E-258	0.58857429	0.997	0.944	5.53E-255	1
NT5DC2	5.99E-257	0.90875414	0.805	0.682	1.20E-253	1
SLPI	6.94E-257	-0.6837666	0.088	0.429	1.39E-253	1
PCSK1N	1.20E-256	-0.350052	0.201	0.529	2.39E-253	1

MX2	2.28E-256	-0.3453305	0.292	0.567	4.56E-253	1
CLMP	8.10E-256	-0.3462989	0.138	0.474	1.62E-252	1
GAS7	2.09E-254	-0.3641457	0.23	0.523	4.18E-251	1
FBLN1	9.59E-253	-2.0853428	0.322	0.691	1.92E-249	1
SCARA5	3.96E-251	-1.1814004	0.278	0.595	7.92E-248	1
FAM13C	4.78E-250	-0.4802519	0.254	0.612	9.57E-247	1
ACVRL1	1.71E-244	-0.292637	0.189	0.512	3.43E-241	1
COL1A2	3.69E-243	-1.4147678	0.842	0.957	7.39E-240	1
VEGFD	4.68E-238	-0.3093922	0.182	0.501	9.36E-235	1
CCDC3	3.30E-235	0.71128444	0.838	0.735	6.59E-232	1
MYH11	2.30E-232	0.5414849	0.999	0.911	4.61E-229	1
C3	1.34E-227	-2.0569879	0.438	0.708	2.68E-224	1
DCLK1	1.30E-222	-0.5223909	0.388	0.667	2.61E-219	1
PTP4A3	1.06E-219	0.88497592	0.682	0.504	2.13E-216	1
LAMA4	8.10E-216	-0.7158558	0.388	0.727	1.62E-212	1
NTRK2	3.48E-210	0.62588447	0.884	0.813	6.96E-207	1
SRPX	8.00E-206	-0.8196011	0.297	0.624	1.60E-202	1
MARCKS	9.63E-206	-1.1042758	0.516	0.831	1.93E-202	1
ADGRL3	4.48E-204	-0.8914108	0.359	0.689	8.96E-201	1
FLNA	5.48E-199	0.37132003	0.997	0.963	1.10E-195	1
PLTP	3.61E-198	-0.8163625	0.353	0.683	7.23E-195	1
SVEP1	3.98E-198	-0.5327134	0.244	0.52	7.96E-195	1
RSPO3	4.58E-195	-0.4392728	0.243	0.515	9.16E-192	1
IL33	1.58E-194	-0.634901	0.104	0.46	3.16E-191	1
CRYAB	1.77E-193	0.65538654	0.865	0.61	3.54E-190	1
PMP22	3.76E-192	-0.9304506	0.433	0.787	7.52E-189	1
S100A10	3.94E-189	-1.6246068	0.244	0.614	7.87E-186	1
TNFRSF1B	4.11E-188	-0.2842177	0.176	0.464	8.21E-185	1
IGFBP6	2.14E-186	-1.7713524	0.554	0.751	4.27E-183	1
ADGRF5	1.86E-184	-0.9803088	0.246	0.55	3.71E-181	1
4-Sep	1.85E-183	1.00578607	0.772	0.683	3.70E-180	1
CREB5	7.88E-180	-0.4194291	0.133	0.451	1.58E-176	1
ABI3BP	2.42E-179	-0.7692467	0.344	0.637	4.84E-176	1
CCDC80	2.51E-179	-1.232124	0.621	0.861	5.01E-176	1
PAPPA	7.80E-179	-0.2790282	0.136	0.425	1.56E-175	1
AUXG01000	5.92E-178	-0.4418094	0.269	0.522	1.18E-174	1
AEBP1	9.45E-178	-1.0356947	0.732	0.865	1.89E-174	1
SYNM	9.72E-174	0.59103541	0.664	0.498	1.94E-170	1
SFRP4	4.98E-171	-0.3620364	0.098	0.392	9.96E-168	1
CD9	2.07E-166	0.35738823	0.725	0.456	4.14E-163	1
ACTN1	6.81E-161	0.43105072	0.877	0.708	1.36E-157	1
VIT	1.70E-160	-0.322505	0.076	0.37	3.40E-157	1
UAP1	3.39E-160	-0.3247122	0.122	0.427	6.78E-157	1
ENTPD3	1.28E-159	0.8745925	0.625	0.551	2.56E-156	1
ID1	1.42E-159	0.31955825	0.385	0.209	2.84E-156	1
CNN1	3.27E-158	0.57298183	0.868	0.668	6.54E-155	1
ACTG2	1.21E-156	0.38994532	0.912	0.832	2.41E-153	1
LSP1	2.50E-155	-0.3353322	0.244	0.501	5.00E-152	1
COL14A1	5.39E-154	-1.1079102	0.66	0.865	1.08E-150	1
PIM3	8.80E-154	-0.2561227	0.136	0.427	1.76E-150	1
CILP	4.78E-148	-0.4145098	0.172	0.449	9.57E-145	1
NR2F1	2.11E-145	-0.4278937	0.227	0.516	4.21E-142	1
CD248	6.86E-145	-0.4428386	0.401	0.638	1.37E-141	1

HTRA3	1.43E-144	-0.5679316	0.234	0.496	2.86E-141	1
BOC	5.70E-144	-0.4005762	0.264	0.525	1.14E-140	1
RRAD	2.01E-143	0.63256247	0.802	0.755	4.02E-140	1
NABP1	3.42E-143	-0.4175117	0.317	0.558	6.85E-140	1
COL6A1	7.97E-143	-0.8948548	0.827	0.904	1.59E-139	1
GUCY1A1	1.13E-142	0.42273905	0.876	0.837	2.26E-139	1
NRGN	1.54E-142	0.86084022	0.713	0.64	3.09E-139	1
MFSD1	4.14E-140	-0.2557689	0.178	0.45	8.29E-137	1
CTSC	9.20E-139	-0.6974312	0.208	0.512	1.84E-135	1
C7	6.05E-138	-1.7278954	0.184	0.491	1.21E-134	1
AIF1	7.79E-136	-0.3667177	0.331	0.556	1.56E-132	1
TCF15	4.99E-134	0.72399029	0.48	0.372	9.98E-131	1
FTL	6.60E-134	-0.5201586	0.998	0.983	1.32E-130	1
SLC40A1	2.50E-131	-0.5550533	0.407	0.706	5.00E-128	1
JCAD	4.31E-131	-0.2755643	0.151	0.413	8.62E-128	1
COL5A1	1.94E-130	-0.659088	0.397	0.676	3.88E-127	1
OR51E1	1.19E-126	0.9565677	0.598	0.559	2.37E-123	1
KLHL23	3.23E-125	0.83859096	0.705	0.639	6.47E-122	1
VWA1	6.11E-125	-0.2520604	0.216	0.495	1.22E-121	1
LUM	9.20E-123	-1.1393191	0.112	0.422	1.84E-119	1
SLC2A3	3.13E-122	-0.5841781	0.337	0.6	6.26E-119	1
TNXB	4.62E-121	-0.9237103	0.139	0.471	9.24E-118	1
CALCRL	8.78E-120	-0.2866062	0.328	0.571	1.76E-116	1
FN1	7.12E-119	-1.1530271	0.504	0.702	1.42E-115	1
RGS5	1.51E-116	1.00396323	0.487	0.325	3.02E-113	1
CAVIN1	2.41E-116	0.30864503	0.991	0.961	4.82E-113	1
KCNK3	6.19E-116	-0.2751186	0.199	0.446	1.24E-112	1
IL6ST	1.86E-112	-0.773219	0.472	0.761	3.72E-109	1
PGF	2.88E-110	0.74880074	0.601	0.542	5.75E-107	1
MGP	1.74E-106	-0.8686666	0.924	0.93	3.48E-103	1
TM4SF1	6.34E-106	-2.2708047	0.319	0.584	1.27E-102	1
ITM2A	1.58E-105	-0.9984455	0.194	0.479	3.15E-102	1
MEDAG	6.07E-104	-0.4575596	0.086	0.362	1.21E-100	1
FCER1G	1.22E-102	-0.3551117	0.327	0.519	2.43E-99	1
ANK3	3.78E-101	-0.5062901	0.187	0.454	7.55E-98	1
NRP2	1.81E-99	-0.3613363	0.106	0.351	3.61E-96	1
HIST1H4C	5.28E-97	0.50402296	0.756	0.723	1.06E-93	1
ALDH1A1	3.10E-96	-0.9068602	0.237	0.549	6.21E-93	1
PAPPA2	1.15E-94	-0.44127	0.182	0.179	2.30E-91	1
KCNQ1OT1	2.77E-94	-0.842719	0.378	0.608	5.53E-91	1
MMP2	4.96E-94	-1.3386903	0.179	0.476	9.92E-91	1
C1QTNF1	1.11E-92	0.32334351	0.719	0.68	2.22E-89	1
MEG3	4.37E-89	-1.4167912	0.122	0.44	8.75E-86	1
TJP1	6.11E-89	-0.6363911	0.485	0.747	1.22E-85	1
LDB2	6.51E-88	-0.3083098	0.29	0.515	1.30E-84	1
ADAMTS1	8.91E-88	-0.8506311	0.383	0.643	1.78E-84	1
NTM	2.02E-87	-0.2667574	0.22	0.465	4.05E-84	1
MDK	1.66E-84	-0.2654502	0.392	0.635	3.33E-81	1
GFRA1	2.42E-84	-0.4511817	0.396	0.666	4.84E-81	1
KRT8	4.76E-84	-0.3785915	0.078	0.326	9.52E-81	1
VWF	2.96E-83	-0.4098595	0.118	0.098	5.93E-80	1
EFHD1	3.75E-83	0.3680404	0.647	0.542	7.50E-80	1
ELN	4.84E-82	-1.1163858	0.516	0.723	9.68E-79	1

VCAN	9.73E-81	-1.614575	0.173	0.435	1.95E-77	1
PLPP3	1.18E-80	-0.5772272	0.396	0.618	2.37E-77	1
TIMP1	1.98E-80	-0.7221825	0.731	0.783	3.97E-77	1
MAP3K7CL	7.34E-80	0.6591891	0.464	0.406	1.47E-76	1
RARRES2	1.16E-79	-0.7576524	0.661	0.881	2.32E-76	1
MGLL	1.92E-79	0.28739694	0.851	0.835	3.84E-76	1
C2orf40	4.51E-79	-2.2629023	0.591	0.807	9.02E-76	1
MBP	9.03E-79	-0.380772	0.139	0.389	1.81E-75	1
FOXS1	1.35E-75	0.55953027	0.613	0.607	2.71E-72	1
RGS7BP	1.01E-74	0.46718682	0.448	0.402	2.02E-71	1
PTN	4.72E-74	0.95606861	0.623	0.589	9.43E-71	1
SPP1	2.72E-72	-0.4416456	0.349	0.526	5.44E-69	1
CLMN	5.02E-70	0.44069682	0.634	0.602	1.00E-66	1
F10	1.95E-69	-0.3316915	0.467	0.713	3.91E-66	1
AKAP12	3.84E-69	-0.7060864	0.601	0.82	7.67E-66	1
STEAP4	4.00E-68	-1.4708068	0.173	0.437	8.00E-65	1
IFIT3	4.37E-67	-0.2899314	0.423	0.659	8.74E-64	1
LTBP4	1.96E-65	-1.2349359	0.228	0.506	3.92E-62	1
APOD	2.78E-64	-1.6668325	0.325	0.512	5.56E-61	1
OMD	4.42E-63	-0.3603222	0.103	0.114	8.84E-60	1
DCN	5.06E-63	-2.5499788	0.445	0.58	1.01E-59	1
LINC00924	2.71E-62	-0.5123503	0.304	0.539	5.41E-59	1
FAM241A	3.29E-62	0.25579916	0.362	0.316	6.58E-59	1
TYMP	3.90E-61	-0.4103461	0.479	0.661	7.81E-58	1
NEXN	9.87E-61	0.28120065	0.667	0.577	1.97E-57	1
LIMCH1	1.30E-59	-0.2767617	0.137	0.144	2.60E-56	1
RGS10	4.22E-59	-0.3684441	0.193	0.419	8.45E-56	1
CFH	2.61E-58	-1.4066912	0.147	0.452	5.22E-55	1
ISG15	4.86E-58	0.58351249	0.85	0.872	9.71E-55	1
COL12A1	7.25E-57	-0.3452808	0.391	0.617	1.45E-53	1
CPXM2	3.75E-56	-0.3233805	0.096	0.119	7.49E-53	1
GSN	1.44E-54	-0.9014017	0.947	0.861	2.88E-51	1
MAST4	1.57E-54	-0.3060155	0.347	0.569	3.14E-51	1
TMEM176A	3.93E-54	-0.7955066	0.174	0.422	7.85E-51	1
ATP1A2	9.95E-54	0.4028359	0.636	0.645	1.99E-50	1
APOE	1.08E-53	-0.7114177	0.595	0.787	2.17E-50	1
SERPINE1	7.97E-51	-0.325763	0.089	0.111	1.59E-47	1
PRELP	6.01E-50	-0.8559893	0.522	0.641	1.20E-46	1
MT1G	9.80E-50	-0.3951508	0.144	0.34	1.96E-46	1
ITGA1	2.92E-49	-0.6630273	0.504	0.698	5.84E-46	1
ASAH1	6.90E-49	-0.3486887	0.484	0.732	1.38E-45	1
COMP	1.57E-48	-0.7383609	0.08	0.121	3.13E-45	1
IGFBP5	2.87E-48	-0.7353972	0.986	0.983	5.74E-45	1
NEGR1	4.35E-48	-0.2898902	0.098	0.15	8.71E-45	1
SYNJ2	4.48E-48	-0.260517	0.113	0.297	8.96E-45	1
CKB	4.62E-47	0.27798435	0.814	0.859	9.25E-44	1
THBD	9.43E-47	-0.3443131	0.171	0.382	1.89E-43	1
GALNT15	1.26E-46	-0.3047975	0.097	0.135	2.52E-43	1
SYNPO2	1.62E-45	-0.812066	0.825	0.919	3.24E-42	1
POSTN	2.31E-45	-0.2944859	0.089	0.119	4.63E-42	1
ESAM	8.21E-45	0.3450184	0.655	0.684	1.64E-41	1
DES	1.02E-44	-1.3965436	0.368	0.566	2.04E-41	1
RGS16	1.60E-44	-0.3774619	0.32	0.283	3.20E-41	1

AGT	1.42E-43	-0.3031796	0.154	0.178	2.84E-40	1
SPON1	3.46E-43	-0.3038023	0.077	0.114	6.91E-40	1
GUCY1A2	4.99E-43	-0.870012	0.303	0.522	9.98E-40	1
COL4A2	8.51E-43	-0.9024234	0.829	0.904	1.70E-39	1
GPNMB	1.18E-42	-0.3620212	0.547	0.748	2.35E-39	1
AIF1L	2.80E-42	0.59231666	0.481	0.509	5.60E-39	1
ANGPTL1	1.63E-40	-0.4076537	0.5	0.72	3.25E-37	1
FSTL1	3.33E-40	-1.2816788	0.371	0.544	6.66E-37	1
CYBA	4.22E-39	-0.4175134	0.525	0.71	8.45E-36	1
GJA1	4.63E-38	-0.3004281	0.089	0.133	9.26E-35	1
TNC	2.40E-36	-0.3726379	0.095	0.114	4.80E-33	1
MEGF6	4.71E-36	-0.5137654	0.084	0.271	9.41E-33	1
CXCL12	1.71E-34	-1.3723216	0.32	0.517	3.43E-31	1
DUSP6	2.29E-34	-0.3384793	0.129	0.187	4.58E-31	1
ADRA2C	1.32E-33	-0.3388343	0.146	0.34	2.64E-30	1
KCTD12	1.91E-32	-0.5741302	0.294	0.492	3.82E-29	1
LINC02381	2.48E-32	0.41864197	0.598	0.66	4.96E-29	1
VMP1	4.07E-32	-0.5898832	0.357	0.539	8.15E-29	1
ENG	4.60E-32	-0.3425625	0.173	0.217	9.20E-29	1
MEST	5.08E-32	-0.2788005	0.275	0.462	1.02E-28	1
FXYD6	2.97E-31	0.46677406	0.53	0.554	5.94E-28	1
CDKN1A	5.41E-31	-0.4071411	0.265	0.237	1.08E-27	1
SPINT2	2.07E-30	-0.3045968	0.137	0.178	4.15E-27	1
ADH1B	2.09E-30	-1.4745046	0.169	0.387	4.18E-27	1
NPDC1	2.16E-30	-0.3632091	0.269	0.474	4.32E-27	1
TSPAN18	2.70E-30	-0.2838713	0.248	0.437	5.39E-27	1
MYOC	3.96E-29	-0.4530147	0.069	0.129	7.91E-26	1
FBN1	1.52E-28	-1.5181477	0.383	0.542	3.03E-25	1
IGHG4	1.76E-28	-0.2504145	0.15	0.315	3.52E-25	1
CDH11	1.97E-28	-0.4772516	0.133	0.332	3.95E-25	1
SPRY1	2.55E-28	-0.3054104	0.375	0.366	5.11E-25	1
IGKC	2.71E-28	-0.410604	0.475	0.614	5.43E-25	1
MFAP4	3.93E-28	-0.5853628	0.362	0.566	7.85E-25	1
CCL19	5.32E-28	-1.4671003	0.321	0.491	1.06E-24	1
CRISPLD2	5.47E-28	-0.4829738	0.418	0.601	1.09E-24	1
CCN3	1.90E-27	-0.3940399	0.186	0.198	3.79E-24	1
INPP4B	5.14E-27	-0.3112667	0.599	0.749	1.03E-23	1
CTSL	1.24E-26	-0.3311603	0.484	0.67	2.48E-23	1
AC080038.1	1.97E-26	-0.9277743	0.098	0.33	3.94E-23	1
IGFBP4	2.39E-26	-0.8319758	0.55	0.637	4.78E-23	1
LMO7	4.82E-26	-0.366634	0.124	0.161	9.63E-23	1
GPX3	8.52E-26	-1.0722576	0.498	0.565	1.70E-22	1
GLUL	1.76E-25	-0.2930035	0.47	0.434	3.53E-22	1
IFI27	1.84E-25	0.27751074	0.568	0.56	3.68E-22	1
MYH10	8.47E-25	-1.2093703	0.241	0.403	1.69E-21	1
CGNL1	9.83E-25	-0.2697615	0.078	0.265	1.97E-21	1
ID4	1.50E-24	-1.3383425	0.424	0.527	3.01E-21	1
LRRC10B	1.52E-24	0.54658664	0.471	0.508	3.03E-21	1
PDGFD	1.73E-24	-0.4332238	0.097	0.145	3.46E-21	1
COL4A1	6.32E-24	-0.8208505	0.779	0.86	1.26E-20	1
ITGA10	1.57E-23	-0.5842039	0.113	0.166	3.14E-20	1
TBX3	1.71E-23	0.32960965	0.473	0.5	3.42E-20	1
ASPN	2.95E-23	-0.2616284	0.159	0.199	5.91E-20	1

HLA-DRA	4.82E-23	-0.695396	0.485	0.618	9.64E-20	1
FHL2	6.21E-23	-0.4821668	0.181	0.24	1.24E-19	1
CTSD	7.20E-23	-0.7815659	0.632	0.764	1.44E-19	1
SLC9A3R2	3.91E-22	-0.6009632	0.337	0.508	7.81E-19	1
IGFBP3	9.09E-22	-0.7342354	0.153	0.235	1.82E-18	1
PLIN2	9.61E-22	-0.4423741	0.191	0.264	1.92E-18	1
IRF1	3.16E-21	-0.5258323	0.452	0.599	6.32E-18	1
PRUNE2	4.32E-21	-0.475225	0.197	0.225	8.65E-18	1
TGFBI	5.94E-21	-0.8483412	0.661	0.819	1.19E-17	1
SOCS3	9.96E-21	-0.5501187	0.312	0.318	1.99E-17	1
FMO2	5.28E-20	-1.0033911	0.196	0.382	1.06E-16	1
PLAT	1.48E-19	-0.4374747	0.112	0.324	2.96E-16	1
COL5A3	1.62E-19	-0.2658713	0.391	0.547	3.23E-16	1
PCOLCE	2.60E-19	-0.5437161	0.415	0.576	5.20E-16	1
FBLN2	3.00E-19	-0.7945442	0.144	0.354	5.99E-16	1
TNFRSF11B	1.45E-18	-0.7929657	0.096	0.144	2.90E-15	1
EPHX1	4.71E-18	-0.2925274	0.426	0.602	9.42E-15	1
GPRC5A	6.73E-18	-0.3061765	0.092	0.156	1.35E-14	1
FMOD	2.05E-17	-0.5432092	0.153	0.222	4.10E-14	1
ITGA11	1.78E-16	-0.6831132	0.125	0.201	3.56E-13	1
DAB2	3.59E-16	-0.52405	0.172	0.359	7.19E-13	1
RGS2	1.22E-15	-0.2694711	0.111	0.264	2.44E-12	1
LRRC32	6.01E-15	0.25297682	0.575	0.603	1.20E-11	1
LTBP1	1.77E-14	-0.4376527	0.493	0.433	3.54E-11	1
ITGA5	2.29E-14	-0.620835	0.119	0.155	4.58E-11	1
CAVIN2	2.29E-14	-0.4934392	0.269	0.322	4.58E-11	1
COL8A1	3.10E-14	-0.7673008	0.116	0.165	6.20E-11	1
SFRP1	6.02E-14	-0.7479885	0.135	0.334	1.20E-10	1
HLA-DPB1	8.31E-14	-0.2893729	0.271	0.422	1.66E-10	1
PDLIM3	1.28E-13	-0.4026363	0.426	0.396	2.56E-10	1
PRSS23	2.70E-13	-0.4492088	0.22	0.278	5.41E-10	1
FHL1	4.47E-13	-0.6200952	0.578	0.631	8.93E-10	1
ADAP2	6.25E-13	0.5513614	0.526	0.558	1.25E-09	1
ACKR3	8.24E-13	0.27749933	0.516	0.603	1.65E-09	1
PDGFB	1.11E-12	0.26985286	0.427	0.527	2.22E-09	1
HMCN1	2.02E-12	-0.4401682	0.133	0.204	4.04E-09	1
AKR1C1	2.08E-12	-0.3106094	0.081	0.247	4.16E-09	1
ENAH	4.50E-12	-0.3118034	0.491	0.452	9.00E-09	1
HLA-DPA1	6.14E-12	-0.3890045	0.282	0.318	1.23E-08	1
BASP1	7.90E-12	-0.4318758	0.128	0.304	1.58E-08	1
NMNAT2	1.54E-11	0.49808907	0.477	0.528	3.07E-08	1
ATP1B1	3.29E-11	-0.3772211	0.108	0.198	6.58E-08	1
ITGBL1	4.27E-11	-0.7630558	0.096	0.201	8.54E-08	1
PI16	6.36E-11	-0.6329128	0.161	0.251	1.27E-07	1
LMCD1	1.72E-10	-1.1770931	0.176	0.364	3.44E-07	1
CAPG	2.64E-10	0.31200814	0.429	0.503	5.28E-07	1
C1QA	4.95E-10	-0.3737021	0.426	0.545	9.89E-07	1
NDRG1	6.97E-10	-0.2644612	0.327	0.476	1.39E-06	1
NUAK1	2.02E-09	-0.4188386	0.217	0.356	4.03E-06	1
SELENOP	2.29E-09	-0.3505294	0.56	0.71	4.58E-06	1
HSPB6	3.97E-09	-0.3081007	0.616	0.558	7.93E-06	1
NR2F2	7.46E-09	-0.6400811	0.932	0.952	1.49E-05	1
ADAMTS4	1.03E-08	-0.2684001	0.23	0.363	2.06E-05	1

MT1X	2.49E-08	-0.3020737	0.323	0.353	4.98E-05	1
AQP1	3.58E-08	-0.3168397	0.352	0.371	7.16E-05	1
INMT	3.74E-08	-0.6760908	0.207	0.287	7.49E-05	1
PRPH	4.87E-08	0.70486706	0.49	0.517	9.73E-05	1
PCSK5	8.69E-08	-0.2840542	0.079	0.24	0.00017371	1
SNED1	1.81E-07	-0.4463952	0.059	0.226	0.00036212	1
SYTL2	2.45E-07	-0.3167127	0.6	0.764	0.00049041	1
ZNF385D	2.65E-07	-0.2627463	0.175	0.233	0.00052919	1
PCOLCE2	3.02E-07	-0.4483196	0.113	0.26	0.00060401	1
CYFIP2	5.32E-07	-0.2881084	0.166	0.244	0.001064	1
ALCAM	6.99E-07	-0.299298	0.201	0.332	0.00139706	1
C12orf75	1.09E-06	0.36435978	0.528	0.624	0.00218174	1
CCL21	1.32E-06	-1.4010639	0.081	0.22	0.00264379	1
OLFML3	1.37E-06	-0.4927543	0.25	0.411	0.00273089	1
PGAM2	3.79E-06	0.54706481	0.416	0.503	0.00757283	1
CCDC102B	5.84E-06	-0.782317	0.367	0.475	0.01168145	1
IGFBP2	6.92E-06	-0.2577478	0.245	0.301	0.01383141	1
FSTL3	7.27E-06	-0.4755787	0.221	0.296	0.01454142	1
PTGDS	8.72E-06	-1.1040546	0.196	0.307	0.01744472	1
MYO10	1.82E-05	-0.3510153	0.075	0.254	0.03634677	1
LIFR	2.17E-05	-0.2982551	0.283	0.358	0.0434658	1
CLU	3.19E-05	-0.422551	0.641	0.586	0.06375106	1
NEDD9	3.44E-05	-0.5597132	0.139	0.259	0.06875585	1
PLA2G2A	4.74E-05	-2.1546548	0.299	0.363	0.09489171	1
ALKAL2	5.95E-05	0.33057818	0.42	0.504	0.11892716	1
SULF1	9.68E-05	-0.8854975	0.156	0.318	0.19361547	1
HLA-B	0.00010177	-0.4857953	0.772	0.856	0.20353304	1
SVIL	0.00012047	-0.3765934	0.56	0.54	0.2409487	1
LAMA2	0.00013984	-0.7366742	0.074	0.199	0.27967325	1
EFEMP1	0.0002248	-1.0387175	0.167	0.335	0.44960249	1
RAMP2	0.00030724	-0.4282861	0.152	0.254	0.6144735	1
CCL2	0.00036872	-0.8006983	0.211	0.296	0.73744553	1
CYGB	0.00042661	-0.2693548	0.178	0.287	0.85321532	1
CBLN1	0.00057785	0.32124187	0.347	0.494	1	1
SAT1	0.00074788	-0.4505079	0.617	0.58	1	1
CYP1B1	0.00111692	-0.2894898	0.094	0.2	1	1
ABLIM1	0.00114003	-0.3574695	0.106	0.232	1	1
SULF2	0.00133109	-0.3156877	0.144	0.25	1	1
PCDH9	0.00166061	-0.3853453	0.154	0.257	1	1
RNASE1	0.00216636	-0.367798	0.4	0.447	1	1
SOD2	0.00248914	-0.3809985	0.402	0.517	1	1
HIGD1B	0.00386533	0.80273237	0.509	0.55	1	1
MGST1	0.00433231	-0.5499471	0.083	0.197	1	1
IGF1	0.00585261	-1.1725929	0.234	0.383	1	1
CXCL14	0.00599914	-1.2457266	0.163	0.295	1	1
VCL	0.00654746	-0.2626568	0.836	0.882	1	1
CCN1	0.00701642	-0.2710572	0.521	0.523	1	1
FLNB	0	0.90372101	0.77	0.163	0	2
EMP1	0	0.83048816	0.798	0.175	0	2
SLPI	0	0.66954137	0.73	0.101	0	2
SFRP2	0	0.57256691	0.842	0.244	0	2
PTPRE	0	0.46213138	0.786	0.143	0	2
MFAP5	0	0.45911377	0.819	0.16	0	2

COTL1	0	0.42551447	0.823	0.225	0	2
TSHZ2	0	0.40717666	0.851	0.35	0	2
LHFPL2	0	0.39290481	0.71	0.089	0	2
MATN2	0	0.39215516	0.802	0.245	0	2
C1orf162	0	0.38876969	0.792	0.26	0	2
BIRC3	0	0.37851877	0.761	0.229	0	2
PLA2G5	0	0.3734302	0.734	0.081	0	2
EGFL7	0	0.37214935	0.116	0.139	0	2
GRASP	0	0.35607057	0.777	0.095	0	2
CORO1A	0	0.34153317	0.803	0.242	0	2
GAS7	0	0.32131576	0.837	0.232	0	2
UPP1	0	0.31673515	0.734	0.119	0	2
TCEAL2	0	0.31406422	0.073	0.104	0	2
RARRES1	0	0.30936339	0.818	0.18	0	2
PPL	0	0.30873443	0.842	0.178	0	2
ABCA6	0	0.30712943	0.792	0.222	0	2
ABCA10	0	0.30098139	0.839	0.289	0	2
ITPR2	0	0.30050458	0.103	0.094	0	2
CCL3L1	0	0.29696251	0.82	0.262	0	2
GPR183	0	0.29609006	0.819	0.172	0	2
ARRB2	0	0.29073845	0.812	0.231	0	2
BICC1	0	0.27983888	0.801	0.17	0	2
BNC2	0	0.27263908	0.843	0.221	0	2
AC135050.7	0	0.26809883	0.593	0.139	0	2
CD52	0	0.26101069	0.794	0.246	0	2
SPOCK1	0	0.25541318	0.754	0.137	0	2
UNC5B	0	0.25158602	0.127	0.119	0	2
CAVIN3	0	-1.1444722	0.885	0.959	0	2
DSTN	0	-1.1667859	0.894	0.996	0	2
ACTA2	0	-1.4550671	0.96	0.998	0	2
MYL9	0	-1.6638038	0.904	0.998	0	2
TPM2	0	-1.7706968	0.962	0.999	0	2
TAGLN	0	-1.8828036	0.98	1	0	2
ADIRF	0	-2.1714094	0.849	0.989	0	2
CRIP1	8.47E-308	-1.0489031	0.871	0.933	1.69E-304	2
AIF1	2.61E-307	0.67629857	0.839	0.326	5.21E-304	2
CCL4	1.68E-300	0.42937967	0.124	0.083	3.35E-297	2
PCLO	1.98E-300	0.26736264	0.668	0.081	3.95E-297	2
CDH19	9.86E-298	0.57825516	0.117	0.075	1.97E-294	2
VGLL3	2.95E-294	0.35837995	0.809	0.201	5.89E-291	2
CRYAB	1.18E-293	-1.1301745	0.348	0.861	2.35E-290	2
NABP1	4.77E-293	0.35697858	0.842	0.314	9.54E-290	2
VWF	4.02E-292	0.72133148	0.107	0.113	8.04E-289	2
OAS2	1.17E-291	-0.2941576	0.255	0.342	2.34E-288	2
HTRA3	5.73E-287	0.25701381	0.769	0.238	1.15E-283	2
SEMA3C	1.59E-286	0.28308184	0.731	0.153	3.18E-283	2
FCER1G	2.89E-282	0.68058035	0.807	0.315	5.77E-279	2
ABCA8	2.49E-280	0.56851013	0.839	0.335	4.99E-277	2
CAV1	4.00E-277	-1.037213	0.898	0.993	8.01E-274	2
SPP1	3.72E-274	0.82221723	0.847	0.33	7.43E-271	2
DUSP6	2.09E-272	0.31777734	0.155	0.144	4.19E-269	2
HSPH1	6.46E-272	0.41544332	0.13	0.206	1.29E-268	2
RERGL	1.76E-265	-1.8340766	0.895	0.873	3.53E-262	2

SPON1	3.53E-265	0.41871744	0.101	0.085	7.06E-262	2
CLMP	2.85E-262	0.35328191	0.71	0.16	5.70E-259	2
IGLC2	5.99E-259	0.34408685	0.813	0.34	1.20E-255	2
MYOC	7.86E-259	0.30115236	0.107	0.083	1.57E-255	2
GEM	2.61E-257	0.25746353	0.126	0.146	5.21E-254	2
VWA1	1.17E-251	0.33158659	0.803	0.217	2.33E-248	2
COL4A4	2.50E-249	0.37428393	0.117	0.067	5.00E-246	2
ECM1	2.51E-249	0.30621538	0.674	0.1	5.01E-246	2
VAMP8	1.73E-244	0.36507073	0.756	0.238	3.46E-241	2
S100A9	1.97E-243	0.38720847	0.8	0.317	3.94E-240	2
ART4	1.87E-242	0.32330106	0.147	0.087	3.74E-239	2
CYTL1	7.33E-240	0.44477228	0.112	0.037	1.47E-236	2
LSP1	1.92E-238	0.2510402	0.752	0.25	3.84E-235	2
SLIT2	5.74E-237	0.48827742	0.703	0.155	1.15E-233	2
TPM1	5.28E-234	-0.8878069	0.896	0.996	1.06E-230	2
ATAD2	1.53E-231	0.34848406	0.728	0.168	3.05E-228	2
GRIA2	5.53E-231	0.7725145	0.149	0.073	1.11E-227	2
CPXM2	3.22E-230	0.54676079	0.153	0.095	6.44E-227	2
PIM3	3.57E-225	0.29214776	0.711	0.143	7.15E-222	2
LAPTM5	4.82E-223	0.36434719	0.748	0.276	9.64E-220	2
OMD	5.25E-223	0.63467612	0.136	0.102	1.05E-219	2
C11orf96	9.22E-221	-0.977972	0.782	0.981	1.84E-217	2
SGK1	8.49E-220	0.40691893	0.706	0.182	1.70E-216	2
S100B	9.53E-220	0.30714472	0.165	0.101	1.91E-216	2
TNC	1.61E-216	0.52393091	0.103	0.1	3.22E-213	2
FRZB	2.00E-216	-0.3533936	0.231	0.567	4.00E-213	2
INSIG1	6.65E-216	0.35118283	0.725	0.19	1.33E-212	2
ENG	1.62E-215	0.44051599	0.181	0.186	3.23E-212	2
CALD1	1.11E-214	-0.6735414	0.94	1	2.21E-211	2
DUSP5	1.48E-214	0.276945	0.632	0.069	2.96E-211	2
CCND1	4.01E-205	-0.9081496	0.872	0.849	8.03E-202	2
WARS	4.41E-202	0.26058203	0.77	0.235	8.82E-199	2
COMP	2.77E-200	1.244515	0.179	0.078	5.54E-197	2
C1QC	5.91E-200	0.43968981	0.762	0.284	1.18E-196	2
CP	6.62E-200	0.61310824	0.111	0.068	1.32E-196	2
PDGFRA	1.47E-199	0.39376799	0.177	0.154	2.94E-196	2
NTRK2	2.19E-198	-0.7381292	0.893	0.858	4.38E-195	2
CXCL14	2.92E-198	0.28367101	0.221	0.198	5.85E-195	2
SERPINE1	1.03E-197	0.65913935	0.167	0.084	2.06E-194	2
INMT	9.19E-197	0.25318828	0.178	0.238	1.84E-193	2
HSPB6	2.49E-195	-0.3720351	0.304	0.646	4.98E-192	2
HCLS1	5.23E-195	0.34006813	0.654	0.12	1.05E-191	2
MFSD1	2.58E-193	0.43154696	0.711	0.185	5.15E-190	2
CAVIN1	1.68E-187	-0.5379528	0.928	0.991	3.37E-184	2
PRSS23	1.11E-183	0.27270564	0.2	0.243	2.22E-180	2
UAP1	1.15E-183	0.37172234	0.68	0.136	2.31E-180	2
FMOD	4.59E-180	0.68483005	0.18	0.171	9.17E-177	2
CD163	8.46E-179	0.28899215	0.724	0.246	1.69E-175	2
GJA1	9.74E-178	0.48496922	0.177	0.09	1.95E-174	2
NRP2	1.37E-177	0.68535897	0.645	0.103	2.75E-174	2
ITGBL1	7.85E-176	0.90596345	0.172	0.119	1.57E-172	2
IGLC3	6.77E-175	0.25264124	0.655	0.167	1.35E-171	2
BCAM	1.51E-174	-0.337352	0.918	0.932	3.01E-171	2

EFHD1	6.32E-174	-0.7623063	0.47	0.639	1.26E-170	2
GALNT15	5.28E-172	0.49376836	0.188	0.096	1.06E-168	2
GPRC5A	2.02E-170	0.49474016	0.174	0.101	4.05E-167	2
CSRP1	2.04E-169	-0.7997409	0.62	0.93	4.07E-166	2
SSTR2	2.12E-169	-0.3126271	0.701	0.182	4.24E-166	2
NDUFA4L2	3.04E-169	-2.0558292	0.634	0.734	6.08E-166	2
C3	1.66E-168	0.36884103	0.866	0.461	3.31E-165	2
FIBIN	2.04E-167	0.41312138	0.179	0.1	4.08E-164	2
SNCG	2.27E-165	-1.0797497	0.87	0.839	4.54E-162	2
CDH13	3.23E-163	0.51272402	0.203	0.256	6.45E-160	2
GUCY1A1	3.83E-163	-0.5218967	0.888	0.861	7.65E-160	2
NET1	1.03E-161	-1.0944218	0.884	0.84	2.06E-158	2
TFRC	2.85E-161	0.35948803	0.687	0.187	5.69E-158	2
LMO7	5.83E-160	0.58465466	0.182	0.127	1.17E-156	2
SFRP4	3.60E-159	0.45719983	0.626	0.113	7.19E-156	2
LMOD1	5.57E-159	-0.704607	0.581	0.926	1.11E-155	2
MGLL	1.15E-158	-0.5333934	0.875	0.842	2.29E-155	2
POSTN	4.12E-158	0.45592571	0.184	0.084	8.25E-155	2
LIMCH1	2.02E-157	0.54205828	0.209	0.128	4.05E-154	2
ADAMTS6	2.51E-157	0.35331033	0.265	0.234	5.01E-154	2
ITGA11	1.81E-155	0.93707399	0.199	0.139	3.62E-152	2
HMCN1	1.18E-154	0.71051659	0.216	0.144	2.36E-151	2
NT5DC2	1.48E-154	-0.9327133	0.807	0.764	2.97E-151	2
PNP	8.17E-154	0.3252868	0.59	0.074	1.63E-150	2
ACTG2	2.41E-153	-0.90543	0.874	0.891	4.83E-150	2
IGHA1	1.88E-152	0.26627151	0.683	0.276	3.77E-149	2
SMC4	8.29E-152	0.39212751	0.761	0.253	1.66E-148	2
HES4	4.29E-151	-0.4940097	0.899	0.902	8.57E-148	2
CNN1	7.66E-150	-0.7611661	0.571	0.848	1.53E-146	2
CLDN5	3.24E-145	0.26545797	0.218	0.145	6.47E-142	2
SLC2A3	1.01E-144	0.62471204	0.828	0.347	2.01E-141	2
TUBA1C	9.89E-143	-1.0311421	0.775	0.732	1.98E-139	2
CRLF1	1.90E-142	0.80442144	0.204	0.18	3.79E-139	2
COL6A3	1.92E-141	0.31977083	0.825	0.338	3.83E-138	2
ISYNA1	2.00E-141	-0.6211837	0.879	0.837	4.00E-138	2
COL15A1	2.24E-141	0.37190275	0.71	0.223	4.47E-138	2
PGM5	1.50E-140	-0.3118648	0.35	0.408	3.00E-137	2
PLN	4.94E-140	-0.6455616	0.661	0.825	9.89E-137	2
ISG15	2.18E-133	-1.1694979	0.914	0.847	4.36E-130	2
LAMA2	7.13E-133	0.82953868	0.186	0.098	1.43E-129	2
PODN	1.88E-132	0.32926714	0.807	0.378	3.77E-129	2
LGALS3	2.40E-132	-0.4175873	0.883	0.87	4.80E-129	2
COX4I2	6.16E-132	-1.4276249	0.855	0.743	1.23E-128	2
PPP1R14A	1.05E-131	-0.6773691	0.737	0.964	2.11E-128	2
CBLN1	2.78E-131	-0.2670472	0.843	0.318	5.55E-128	2
ASPN	1.33E-129	0.35715036	0.217	0.163	2.66E-126	2
LMCD1	1.94E-128	0.33063118	0.244	0.228	3.88E-125	2
GPM6B	6.90E-127	0.36604719	0.188	0.143	1.38E-123	2
MT1F	3.94E-126	-0.5606047	0.318	0.272	7.88E-123	2
RRAD	5.32E-125	-0.7546557	0.831	0.781	1.06E-121	2
CCL2	1.02E-124	0.31159517	0.239	0.234	2.04E-121	2
ITGA10	2.15E-124	1.05887595	0.23	0.113	4.30E-121	2
DIO2	5.53E-123	0.34712791	0.186	0.091	1.11E-119	2

ID3	6.14E-123	-0.5316269	0.434	0.755	1.23E-119	2
MGST1	4.48E-122	0.44533118	0.19	0.105	8.95E-119	2
FILIP1L	2.76E-121	-0.5462923	0.46	0.766	5.52E-118	2
CSRP2	4.96E-121	-0.8539887	0.719	0.756	9.92E-118	2
CCDC3	1.95E-119	-0.6070936	0.876	0.797	3.89E-116	2
MT1E	1.11E-117	-0.4328508	0.576	0.839	2.22E-114	2
FSTL3	2.32E-117	0.51250091	0.233	0.244	4.64E-114	2
SOCS3	4.46E-117	0.78593263	0.262	0.322	8.92E-114	2
TYROBP	2.95E-114	0.44107654	0.853	0.424	5.91E-111	2
GJA4	1.62E-113	-0.8500575	0.861	0.726	3.24E-110	2
FBLN1	1.87E-113	0.80287893	0.827	0.366	3.75E-110	2
HIST1H1C	1.87E-112	-0.3861795	0.361	0.388	3.74E-109	2
LAMB1	6.05E-112	0.44866466	0.255	0.213	1.21E-108	2
COL12A1	2.29E-111	0.41956357	0.86	0.393	4.58E-108	2
IGKC	4.29E-110	0.86728649	0.862	0.461	8.58E-107	2
SYNJ2	1.51E-108	0.53207185	0.569	0.102	3.02E-105	2
ENAH	2.69E-108	0.32109453	0.296	0.509	5.37E-105	2
NOVA1	3.92E-108	0.32981986	0.216	0.152	7.84E-105	2
KCNAB1	1.63E-107	-0.7320011	0.88	0.757	3.26E-104	2
HIST1H4C	1.65E-107	-0.6946861	0.778	0.741	3.30E-104	2
PALLD	3.67E-106	-0.2624074	0.482	0.779	7.34E-103	2
PDGFD	4.77E-106	0.73714478	0.21	0.096	9.55E-103	2
RGS5	1.07E-105	-0.9879904	0.375	0.45	2.15E-102	2
CREB5	3.15E-105	0.35869597	0.628	0.162	6.29E-102	2
CASQ2	6.58E-104	-0.9039834	0.887	0.753	1.32E-100	2
PLTP	5.55E-103	0.37819837	0.844	0.386	1.11E-99	2
PTGDS	9.65E-103	0.25659751	0.303	0.216	1.93E-99	2
KCNQ1OT1	1.16E-102	0.89751283	0.797	0.389	2.32E-99	2
PHLDA2	1.60E-102	-1.0377008	0.867	0.775	3.21E-99	2
DPT	2.83E-101	0.30596281	0.202	0.084	5.65E-98	2
PLPP3	2.23E-100	0.25699154	0.84	0.401	4.45E-97	2
CRIP2	2.33E-100	-0.4588603	0.805	0.849	4.65E-97	2
SPINT2	5.52E-100	0.49580445	0.251	0.133	1.10E-96	2
NEXN	2.54E-96	-0.4525299	0.516	0.66	5.09E-93	2
ABI3BP	1.51E-92	0.29638976	0.771	0.375	3.01E-89	2
ITM2C	1.52E-92	-0.6611568	0.602	0.563	3.05E-89	2
4-Sep	3.24E-92	-1.2195469	0.863	0.727	6.48E-89	2
CCL19	2.13E-90	-0.6338428	0.761	0.308	4.26E-87	2
PRPH	9.43E-90	-0.5012553	0.851	0.442	1.89E-86	2
FBLN5	1.65E-89	0.64900089	0.27	0.199	3.29E-86	2
WFDC1	2.14E-89	-0.8273133	0.621	0.586	4.28E-86	2
SMOC2	2.40E-89	0.63903329	0.272	0.29	4.80E-86	2
DKK3	2.89E-89	0.3982402	0.322	0.453	5.77E-86	2
CDKN1A	4.21E-89	0.84857144	0.239	0.26	8.41E-86	2
CKB	2.45E-88	-0.4454545	0.89	0.817	4.89E-85	2
HLA-DRA	5.79E-88	1.24981902	0.842	0.474	1.16E-84	2
TIMP3	4.79E-87	-0.2958172	0.867	0.976	9.57E-84	2
ANK3	9.88E-85	0.30862243	0.655	0.203	1.98E-81	2
TYMP	1.76E-84	0.37244834	0.864	0.479	3.52E-81	2
ADGRL3	4.56E-84	-0.2663583	0.853	0.391	9.12E-81	2
PTP4A3	4.92E-81	-0.7054103	0.657	0.627	9.85E-78	2
RCAN2	1.92E-80	-0.4351095	0.688	0.706	3.85E-77	2
SORBS1	5.16E-77	0.42967288	0.359	0.525	1.03E-73	2

CDH2	6.69E-77	0.30330693	0.234	0.083	1.34E-73	2
MAST4	5.65E-76	0.43316006	0.784	0.352	1.13E-72	2
LGI4	1.10E-75	-0.5129027	0.873	0.725	2.21E-72	2
FSTL1	3.52E-75	0.52074926	0.331	0.436	7.05E-72	2
PECAM1	3.75E-75	0.30353825	0.258	0.145	7.51E-72	2
FABP3	6.18E-75	-0.3281369	0.814	0.353	1.24E-71	2
GPX3	5.23E-72	0.44622176	0.356	0.543	1.05E-68	2
TBX2	6.59E-72	-0.4443476	0.904	0.808	1.32E-68	2
TNFRSF11B	1.86E-71	1.39276919	0.246	0.089	3.72E-68	2
ITGA5	5.49E-71	1.10674158	0.216	0.115	1.10E-67	2
NRARP	2.79E-69	-1.085739	0.855	0.662	5.58E-66	2
SLC40A1	3.77E-69	0.42940879	0.851	0.437	7.53E-66	2
RCAN1	5.83E-69	0.25066283	0.347	0.263	1.17E-65	2
NNMT	1.13E-68	0.65703881	0.305	0.208	2.26E-65	2
RAMP2	5.82E-68	0.42121888	0.287	0.165	1.16E-64	2
MYLK	9.56E-68	-0.2961913	0.921	0.96	1.91E-64	2
TGM2	3.25E-67	0.67014819	0.311	0.281	6.51E-64	2
MT1G	5.03E-66	0.46460419	0.563	0.143	1.01E-62	2
ELN	1.52E-63	1.47490343	0.847	0.533	3.04E-60	2
FN1	1.94E-62	1.45227494	0.712	0.538	3.88E-59	2
PTGIS	3.31E-62	0.80528687	0.298	0.217	6.61E-59	2
ITIH5	8.87E-62	-0.2940106	0.92	0.845	1.77E-58	2
ALKAL2	1.64E-61	-0.2797663	0.845	0.381	3.29E-58	2
PGAM2	1.81E-61	-0.4116638	0.844	0.377	3.62E-58	2
EFEMP1	2.39E-61	0.83926609	0.302	0.202	4.78E-58	2
THBD	2.47E-61	0.47735746	0.606	0.173	4.93E-58	2
CD14	1.57E-60	0.30805596	0.639	0.295	3.14E-57	2
CFH	4.73E-59	1.06967853	0.308	0.224	9.47E-56	2
TWISTNB	7.00E-58	-0.3100709	0.397	0.34	1.40E-54	2
FBN1	2.12E-57	0.25583766	0.412	0.432	4.23E-54	2
HIGD1B	2.11E-56	-0.8046241	0.844	0.47	4.22E-53	2
PLK2	2.84E-56	0.40840098	0.703	0.286	5.67E-53	2
NR4A2	3.58E-56	0.48023208	0.326	0.235	7.16E-53	2
SYNM	8.37E-56	-0.296446	0.635	0.613	1.67E-52	2
IFIT1	2.53E-55	-0.325373	0.853	0.425	5.06E-52	2
C1QA	2.63E-55	0.7702707	0.77	0.412	5.26E-52	2
MDK	2.58E-54	-0.4008915	0.819	0.406	5.17E-51	2
OGN	1.43E-52	0.41768382	0.351	0.289	2.87E-49	2
ADRA2A	1.63E-52	-0.3098331	0.775	0.371	3.25E-49	2
LUM	6.13E-52	1.07429013	0.56	0.145	1.23E-48	2
NMNAT2	1.42E-51	-0.440112	0.849	0.435	2.85E-48	2
SHTN1	1.69E-50	0.25562009	0.548	0.168	3.37E-47	2
LTBP4	1.13E-49	0.73698656	0.338	0.304	2.27E-46	2
C7	2.10E-47	0.76085104	0.602	0.221	4.20E-44	2
F2R	2.82E-47	0.48065509	0.277	0.079	5.64E-44	2
PCDH9	4.89E-47	0.41126012	0.324	0.162	9.78E-44	2
ADAP2	7.51E-45	-0.3915049	0.858	0.484	1.50E-41	2
CARMN	3.56E-44	1.54656797	0.98	0.936	7.12E-41	2
ADAMTS1	5.23E-44	1.06663801	0.768	0.409	1.05E-40	2
COL8A1	1.08E-43	1.31321241	0.277	0.107	2.17E-40	2
LRRC10B	1.68E-43	-0.3999412	0.855	0.423	3.35E-40	2
LINC00924	4.99E-43	-0.381325	0.736	0.314	9.98E-40	2
IFI6	3.04E-42	-0.4925796	0.896	0.779	6.08E-39	2

RARRES2	7.03E-42	-0.5208564	0.858	0.703	1.41E-38	2
TNMD	8.48E-42	-0.5357898	0.844	0.377	1.70E-38	2
CCL3	1.41E-41	0.26404246	0.39	0.241	2.81E-38	2
LTBP2	1.41E-40	1.11682162	0.314	0.154	2.82E-37	2
GUCY1A2	1.61E-40	-0.5689603	0.47	0.35	3.22E-37	2
MT1X	2.37E-40	0.48775549	0.366	0.326	4.75E-37	2
NEURL1B	3.73E-40	-0.517963	0.879	0.649	7.46E-37	2
COL5A1	1.22E-39	0.35319344	0.787	0.429	2.45E-36	2
CCN3	6.09E-39	0.86631023	0.322	0.168	1.22E-35	2
TFPI2	2.00E-38	0.30393772	0.249	0.075	4.01E-35	2
IL33	2.66E-37	0.37856337	0.549	0.153	5.31E-34	2
PRUNE2	1.12E-36	0.86974851	0.332	0.185	2.24E-33	2
HSPA6	4.29E-36	0.31122378	0.488	0.093	8.58E-33	2
MT1L	9.94E-36	-0.6314312	0.839	0.453	1.99E-32	2
CYBA	1.82E-35	0.59038981	0.86	0.534	3.63E-32	2
KCNT2	2.64E-35	0.3697978	0.292	0.091	5.28E-32	2
IFIT3	3.11E-35	-0.3795672	0.849	0.435	6.23E-32	2
TGFBI	6.50E-35	-0.5222376	0.879	0.679	1.30E-31	2
CCN5	1.51E-34	0.89765862	0.324	0.149	3.02E-31	2
CTSL	3.95E-33	0.47633741	0.855	0.488	7.90E-30	2
COL4A2	6.69E-32	1.3054348	0.922	0.839	1.34E-28	2
ADAMTS4	1.77E-31	0.34727528	0.587	0.218	3.55E-28	2
ZNF385D	9.64E-31	0.51008079	0.33	0.17	1.93E-27	2
SAA1	1.10E-30	0.25098173	0.461	0.074	2.21E-27	2
ECE1	2.43E-30	0.31735056	0.438	0.333	4.85E-27	2
TCF15	3.35E-30	-0.6765636	0.562	0.431	6.69E-27	2
PDK4	1.20E-29	0.38151836	0.648	0.289	2.40E-26	2
FBLN2	1.25E-29	0.41704905	0.352	0.181	2.51E-26	2
ADM	3.06E-29	0.25259015	0.331	0.138	6.12E-26	2
PDLIM3	7.16E-29	0.70285642	0.396	0.421	1.43E-25	2
STAT1	1.12E-28	-0.251749	0.842	0.466	2.23E-25	2
PTPN14	1.68E-28	0.31215356	0.586	0.221	3.36E-25	2
RGS4	1.24E-27	0.30773576	0.505	0.117	2.48E-24	2
C2orf40	2.48E-27	-1.407052	0.819	0.628	4.96E-24	2
COL5A2	6.05E-27	0.28852998	0.791	0.448	1.21E-23	2
FMO2	1.47E-26	0.78105439	0.344	0.235	2.94E-23	2
MEGF6	1.66E-26	0.86009524	0.47	0.086	3.32E-23	2
FBLIM1	4.80E-26	0.69535137	0.382	0.329	9.60E-23	2
FOXS1	6.91E-26	-0.7013329	0.813	0.58	1.38E-22	2
PLD3	1.72E-25	0.28915824	0.463	0.37	3.43E-22	2
SNED1	6.97E-25	0.47774543	0.289	0.079	1.39E-21	2
PCDH7	1.89E-24	1.14013189	0.341	0.142	3.79E-21	2
IRF1	2.30E-24	0.67877151	0.765	0.452	4.59E-21	2
COL1A1	2.54E-24	1.14709468	0.933	0.813	5.07E-21	2
PTN	2.61E-24	-0.9710215	0.771	0.588	5.21E-21	2
LINC02381	4.95E-23	-0.7075334	0.852	0.579	9.91E-20	2
MEDAG	6.04E-22	0.3283232	0.475	0.117	1.21E-18	2
ALCAM	1.56E-21	0.64445778	0.543	0.191	3.12E-18	2
LTBP1	4.12E-21	0.53514169	0.445	0.481	8.24E-18	2
CYTOR	6.70E-21	0.32251751	0.692	0.358	1.34E-17	2
SULF1	3.67E-20	1.19800184	0.363	0.178	7.35E-17	2
APOD	8.62E-20	0.88469959	0.583	0.347	1.72E-16	2
ADH1B	1.00E-19	1.10554974	0.351	0.213	2.01E-16	2

MAFB	1.28E-19	0.45069589	0.87	0.532	2.57E-16	2
USP53	1.33E-19	0.99061417	0.348	0.21	2.65E-16	2
SVIL	1.84E-19	0.59007347	0.49	0.564	3.68E-16	2
KLHL23	2.48E-19	-0.6856812	0.876	0.656	4.96E-16	2
COL14A1	3.30E-19	1.11129216	0.826	0.702	6.60E-16	2
HLA-B	3.32E-19	0.49136602	0.856	0.787	6.65E-16	2
ESAM	4.42E-19	-0.2700759	0.857	0.633	8.83E-16	2
CCDC80	5.74E-19	1.09956487	0.885	0.66	1.15E-15	2
NRGN	1.12E-18	-0.5958844	0.863	0.665	2.23E-15	2
VMP1	1.40E-18	0.89861887	0.669	0.369	2.81E-15	2
AC080038.1	2.31E-18	0.84155672	0.337	0.138	4.63E-15	2
VCL	6.01E-18	0.34364068	0.91	0.84	1.20E-14	2
AEBP1	1.18E-17	0.89196423	0.779	0.769	2.36E-14	2
DAB2	1.66E-17	0.40249864	0.383	0.201	3.31E-14	2
ACKR1	2.37E-17	0.71679522	0.351	0.132	4.74E-14	2
ANGPT2	3.94E-17	-0.2881099	0.602	0.445	7.88E-14	2
CNKSR3	6.92E-17	0.3822694	0.325	0.091	1.38E-13	2
SOD2	7.43E-17	0.25445712	0.531	0.421	1.49E-13	2
COL21A1	1.23E-16	0.93031512	0.37	0.18	2.45E-13	2
IGHG4	2.10E-16	0.48524644	0.497	0.151	4.20E-13	2
NFKBIA	2.96E-16	0.7493024	0.503	0.452	5.93E-13	2
GPNMB	3.06E-16	0.25927004	0.872	0.563	6.13E-13	2
SFRP1	4.13E-16	0.54845528	0.372	0.164	8.25E-13	2
AIF1L	7.36E-16	-0.431653	0.83	0.435	1.47E-12	2
HLA-DPB1	8.15E-16	0.45077648	0.602	0.27	1.63E-12	2
LPP	2.03E-15	0.28289062	0.897	0.968	4.06E-12	2
TM4SF1	9.03E-15	-0.594799	0.509	0.378	1.81E-11	2
TPD52L1	5.45E-14	0.26946336	0.306	0.078	1.09E-10	2
PEG10	7.22E-14	-0.2935848	0.724	0.37	1.44E-10	2
MAP3K7CL	1.16E-13	-0.6132841	0.611	0.421	2.32E-10	2
THBS2	3.10E-13	1.56715762	0.324	0.118	6.20E-10	2
STEAP4	3.34E-13	-0.2581802	0.407	0.224	6.68E-10	2
VCAN	2.45E-12	1.70177907	0.487	0.211	4.91E-09	2
FABP4	3.99E-12	-0.5083421	0.841	0.636	7.97E-09	2
CTSB	4.67E-12	0.68783775	0.845	0.562	9.33E-09	2
FOXC2	4.84E-12	0.47994822	0.597	0.298	9.69E-09	2
HLA-DPA1	5.28E-12	0.70768619	0.447	0.268	1.06E-08	2
MEG3	9.64E-12	1.296946	0.403	0.185	1.93E-08	2
CCN1	1.21E-11	0.46264229	0.508	0.523	2.41E-08	2
ENPP2	2.60E-11	0.29392302	0.445	0.071	5.20E-08	2
C1S	4.56E-11	0.8168123	0.631	0.401	9.12E-08	2
COL6A1	1.46E-09	0.76421664	0.854	0.848	2.93E-06	2
NPDC1	2.92E-09	0.35894533	0.593	0.287	5.84E-06	2
SYNPO2	3.93E-09	0.25252018	0.918	0.842	7.87E-06	2
EPHX1	4.11E-09	-0.3082001	0.633	0.453	8.21E-06	2
S100A10	5.72E-09	0.42357398	0.472	0.332	1.14E-05	2
NUAK1	1.65E-08	0.45158055	0.42	0.232	3.30E-05	2
RGS7BP	5.45E-08	-0.4058838	0.611	0.407	0.00010899	2
C12orf75	6.43E-08	-0.5275036	0.742	0.526	0.00012865	2
CKS2	9.50E-08	0.25848968	0.461	0.145	0.00018994	2
SH3BGRL3	9.74E-08	0.82471869	0.78	0.679	0.00019484	2
PMP22	9.96E-08	0.34742835	0.8	0.494	0.0001993	2
SERPINF1	1.65E-07	0.33636046	0.872	0.681	0.00033002	2

IGFBP2	1.78E-07	0.4192412	0.431	0.235	0.0003554	2
FGL2	1.83E-07	0.86270015	0.411	0.203	0.00036673	2
SAT1	2.07E-07	0.84570886	0.533	0.618	0.00041318	2
NEDD9	2.33E-07	1.02021274	0.446	0.131	0.0004666	2
PRELP	4.33E-07	0.97160292	0.6	0.55	0.00086581	2
FKBP11	5.19E-07	0.2828862	0.446	0.118	0.00103868	2
CAMK2N1	1.68E-06	-0.3943266	0.814	0.444	0.00335067	2
TBX3	1.72E-06	-0.2950948	0.772	0.435	0.00344818	2
RGS2	2.30E-06	0.54423276	0.439	0.11	0.00459631	2
FHL5	8.41E-06	-0.4461146	0.579	0.352	0.01682591	2
RND3	1.18E-05	0.40707994	0.678	0.417	0.02360096	2
ENTPD3	1.47E-05	-0.6295756	0.87	0.561	0.02937208	2
C1QB	3.13E-05	0.33823317	0.548	0.376	0.06252842	2
CRISPLD2	4.15E-05	0.28737537	0.721	0.431	0.08301355	2
AKR1C1	5.40E-05	0.47896175	0.415	0.084	0.10792192	2
ITGA1	0.00011412	0.67184761	0.735	0.533	0.22824848	2
RNASE1	0.00021489	0.66060561	0.565	0.39	0.42977652	2
ADRA2C	0.00028339	0.39536629	0.463	0.161	0.56678397	2
CRIM1	0.00033742	0.37613161	0.828	0.648	0.67483426	2
OR51E1	0.00082199	-0.8450592	0.853	0.544	1	2
COL1A2	0.00171435	0.84737289	0.934	0.866	1	2
PGF	0.00228027	-0.5327655	0.839	0.544	1	2
EFNB2	0.00278254	0.29219682	0.403	0.088	1	2
FXYD6	0.00517701	-0.5803699	0.844	0.488	1	2
ITGA8	0.00781067	0.75327851	0.642	0.616	1	2
C2orf40	0	3.63495293	0.96	0.619	0	3
TM4SF1	0	3.39653484	0.835	0.345	0	3
GGT5	0	2.46391154	0.8	0.28	0	3
CLSTN2	0	2.21150822	0.762	0.354	0	3
ID4	0	2.08624768	0.868	0.406	0	3
COL6A3	0	1.85512596	0.828	0.356	0	3
GUCY1A2	0	1.8096407	0.74	0.323	0	3
RARRES2	0	1.56302397	0.947	0.699	0	3
NR2F2	0	1.29565977	0.988	0.932	0	3
ITIH5	0	-1.9876961	0.381	0.91	0	3
TBX2	0	-2.0923663	0.261	0.886	0	3
PHLDA2	0	-2.3282758	0.189	0.857	0	3
NET1	0	-2.7762717	0.275	0.912	0	3
RERGL	0	-3.5833721	0.264	0.946	0	3
BCAM	1.41E-306	-1.7479054	0.637	0.964	2.83E-303	3
C1R	2.12E-304	1.35948054	0.895	0.414	4.24E-301	3
MFGE8	2.18E-301	-1.5409282	0.709	0.939	4.37E-298	3
ADIRF	5.64E-297	-1.525723	0.915	0.977	1.13E-293	3
C1S	4.85E-294	1.47780205	0.849	0.384	9.71E-291	3
COL1A1	6.40E-289	1.19991199	0.991	0.811	1.28E-285	3
SYNPO2	9.72E-287	1.39824842	0.974	0.838	1.94E-283	3
AP002956.1	4.52E-278	-1.4137848	0.873	0.989	9.05E-275	3
LMCD1	5.84E-277	1.74652474	0.644	0.182	1.17E-273	3
CASQ2	3.62E-275	-1.6511712	0.324	0.823	7.25E-272	3
ENTPD3	1.63E-254	-1.3483555	0.157	0.655	3.25E-251	3
NRARP	7.10E-254	-1.468948	0.216	0.743	1.42E-250	3
STEAP4	2.03E-250	2.43099301	0.631	0.205	4.05E-247	3
ADGRF5	2.15E-249	1.76112319	0.665	0.296	4.30E-246	3

IGFBP5	3.19E-245	1.20001483	1	0.984	6.38E-242	3
TGFBI	1.09E-241	1.8417195	0.869	0.688	2.18E-238	3
PLN	2.81E-239	-1.658367	0.406	0.849	5.62E-236	3
KCNAB1	6.02E-236	-1.4183452	0.356	0.822	1.20E-232	3
CXCL12	2.68E-234	1.87279401	0.727	0.336	5.36E-231	3
FHL1	1.52E-233	1.29028223	0.9	0.558	3.04E-230	3
ALDH1A1	3.53E-224	1.37468615	0.686	0.286	7.07E-221	3
COL3A1	1.06E-220	1.00036756	0.972	0.828	2.11E-217	3
CCL21	9.37E-220	2.32246718	0.442	0.084	1.87E-216	3
SSTR2	1.80E-216	1.5795181	0.559	0.217	3.60E-213	3
MYH11	7.73E-214	-1.1304452	0.804	0.993	1.55E-210	3
BGN	3.27E-213	-1.286589	0.599	0.897	6.54E-210	3
RCAN2	7.50E-209	-1.5485025	0.262	0.754	1.50E-205	3
S100A10	4.59E-206	1.44847151	0.699	0.311	9.18E-203	3
CRIP1	2.52E-204	-1.4289058	0.694	0.951	5.04E-201	3
ADGRL3	1.11E-202	1.72052381	0.705	0.425	2.21E-199	3
COL18A1	2.17E-200	-1.4905341	0.517	0.881	4.34E-197	3
SORBS2	2.26E-199	-1.4437659	0.762	0.941	4.51E-196	3
WFDC1	1.10E-194	-1.4615259	0.147	0.642	2.20E-191	3
ABCC9	4.69E-194	1.55596646	0.715	0.455	9.38E-191	3
PROCR	1.31E-190	1.40239049	0.619	0.305	2.63E-187	3
CRIP2	1.87E-190	-1.3665061	0.545	0.877	3.74E-187	3
OR51E1	1.01E-187	-1.1525322	0.224	0.628	2.03E-184	3
FTL	8.00E-182	0.68579139	0.998	0.993	1.60E-178	3
TJP1	4.42E-176	1.31351811	0.778	0.536	8.84E-173	3
THY1	3.54E-173	1.11710024	0.486	0.182	7.08E-170	3
NRGN	2.28E-165	-1.2293953	0.334	0.733	4.57E-162	3
PGF	5.68E-165	-1.0332566	0.191	0.629	1.14E-161	3
GJA4	5.18E-163	-1.0837471	0.34	0.791	1.04E-159	3
TMEM176A	5.51E-163	1.40118744	0.517	0.215	1.10E-159	3
SLC9A3R2	6.28E-163	1.12794385	0.665	0.354	1.26E-159	3
LRRC10B	6.78E-160	-0.7408711	0.15	0.52	1.36E-156	3
FHL2	2.49E-159	1.08117201	0.508	0.162	4.97E-156	3
IGFBP4	6.63E-159	0.9977239	0.818	0.547	1.33E-155	3
CCDC102B	1.29E-157	1.40739657	0.628	0.372	2.58E-154	3
NDUFA4L2	2.95E-156	-2.0824794	0.335	0.765	5.91E-153	3
CSRP2	2.41E-154	-1.3949465	0.443	0.787	4.82E-151	3
DSTN	3.22E-154	-0.8440539	0.974	0.983	6.43E-151	3
PRPH	6.44E-154	-0.9898781	0.179	0.535	1.29E-150	3
MARCKS	5.07E-151	1.22731091	0.792	0.586	1.01E-147	3
SNCG	2.52E-150	-1.4552996	0.571	0.874	5.03E-147	3
TMEM176B	4.79E-150	1.41046818	0.57	0.299	9.58E-147	3
AEBP1	2.61E-148	0.82166764	0.945	0.75	5.23E-145	3
SEMA5A	2.69E-147	1.16507496	0.653	0.401	5.38E-144	3
CTSC	7.40E-142	1.31637534	0.526	0.269	1.48E-138	3
TIMP3	2.47E-141	0.98190461	0.966	0.961	4.94E-138	3
IGFBP6	1.27E-136	1.14646753	0.843	0.584	2.53E-133	3
LGI4	8.08E-136	1.05768847	0.844	0.734	1.62E-132	3
COX4I2	4.23E-135	-0.9779305	0.401	0.8	8.47E-132	3
AIF1L	1.32E-134	-0.7593327	0.188	0.524	2.65E-131	3
KRT18	2.31E-134	2.02058395	0.485	0.207	4.63E-131	3
KRT8	4.06E-133	0.70945293	0.357	0.126	8.12E-130	3
CLMN	9.33E-130	-0.8482624	0.264	0.666	1.87E-126	3

COL1A2	1.18E-127	0.56806544	0.969	0.865	2.36E-124	3
CFH	8.50E-127	0.74736617	0.515	0.203	1.70E-123	3
FLNA	7.34E-125	-0.7467532	0.965	0.989	1.47E-121	3
CAVIN2	7.74E-125	0.93550752	0.502	0.259	1.55E-121	3
KLHL23	8.26E-125	-1.0369071	0.353	0.725	1.65E-121	3
TUBA1C	8.21E-123	-1.0513432	0.403	0.776	1.64E-119	3
GPC3	6.01E-120	1.39270949	0.574	0.326	1.20E-116	3
LTBP4	6.05E-120	0.84963119	0.584	0.277	1.21E-116	3
PTP4A3	9.19E-120	-1.033428	0.29	0.67	1.84E-116	3
CAV1	1.08E-118	0.6196347	0.994	0.979	2.16E-115	3
CAPG	1.25E-118	-0.5398345	0.169	0.483	2.50E-115	3
TNXB	1.35E-118	0.91793906	0.472	0.208	2.70E-115	3
GPM6B	6.60E-118	0.61474174	0.372	0.123	1.32E-114	3
FAM43A	7.75E-118	0.36537159	0.279	0.094	1.55E-114	3
ATP1B1	9.11E-117	0.55076227	0.343	0.11	1.82E-113	3
HES4	2.12E-116	-0.9518033	0.703	0.924	4.24E-113	3
ADAP2	2.14E-114	-0.7466544	0.258	0.567	4.29E-111	3
CSRP1	1.02E-112	-0.953481	0.782	0.9	2.04E-109	3
PMP22	1.05E-112	0.85765625	0.74	0.512	2.10E-109	3
CHRDL1	1.35E-112	1.15656173	0.525	0.328	2.69E-109	3
CGNL1	2.82E-112	0.37386192	0.299	0.113	5.63E-109	3
SYNM	4.74E-112	-0.8634579	0.272	0.656	9.49E-109	3
ITGA8	7.35E-112	-1.0269045	0.269	0.66	1.47E-108	3
CCND1	1.17E-110	-1.127581	0.63	0.878	2.33E-107	3
PGAM2	4.85E-110	-0.7654645	0.163	0.473	9.71E-107	3
C11orf96	2.12E-107	-0.8489052	0.926	0.958	4.24E-104	3
IL33	5.85E-107	0.70619192	0.417	0.182	1.17E-103	3
KIT	1.30E-106	0.59122411	0.36	0.171	2.60E-103	3
INMT	1.77E-106	1.0218677	0.475	0.201	3.53E-103	3
NMNAT2	5.34E-103	-0.5470478	0.227	0.522	1.07E-99	3
TFPI	6.12E-103	1.01103933	0.551	0.325	1.22E-99	3
TCF15	1.28E-102	-0.8147444	0.153	0.483	2.56E-99	3
AKAP12	1.15E-101	0.83296368	0.817	0.647	2.31E-98	3
LMOD1	4.46E-101	0.65956751	0.972	0.869	8.92E-98	3
FGF7	1.94E-100	1.33955165	0.579	0.399	3.88E-97	3
PAWR	9.76E-100	0.76214026	0.763	0.534	1.95E-96	3
HSPG2	1.56E-99	-0.960264	0.419	0.743	3.12E-96	3
OAF	9.90E-99	1.17189401	0.579	0.41	1.98E-95	3
CRABP2	9.88E-97	-0.2968964	0.134	0.408	1.98E-93	3
HMCN2	1.62E-95	1.23256068	0.496	0.287	3.24E-92	3
SYTL2	4.99E-95	0.90349919	0.759	0.635	9.98E-92	3
MATN2	1.63E-93	0.99459152	0.506	0.3	3.25E-90	3
HSPB6	5.80E-93	0.86766223	0.791	0.577	1.16E-89	3
PODN	1.27E-92	0.7596636	0.614	0.416	2.55E-89	3
FRZB	2.50E-91	-1.2607871	0.166	0.562	4.99E-88	3
INPP4B	3.49E-91	0.9336715	0.749	0.63	6.98E-88	3
CALD1	6.87E-89	0.40619803	1	0.991	1.37E-85	3
ITGA1	1.05E-88	0.67724083	0.745	0.539	2.11E-85	3
ACTN1	1.79E-88	-0.8628458	0.711	0.842	3.57E-85	3
APOE	8.42E-88	1.42351303	0.782	0.635	1.68E-84	3
FAM13C	1.25E-87	1.02237849	0.53	0.338	2.50E-84	3
SPON2	3.51E-87	1.05867382	0.495	0.333	7.02E-84	3
CCL2	5.26E-87	1.26318592	0.446	0.211	1.05E-83	3

KCNB1	7.50E-87	-0.3031315	0.152	0.419	1.50E-83	3
HS3ST1	2.09E-84	-0.3305895	0.199	0.432	4.17E-81	3
NT5DC2	3.03E-84	-0.8173746	0.494	0.801	6.07E-81	3
TPM2	7.94E-84	-0.6097405	0.994	0.994	1.59E-80	3
RGS10	2.61E-83	0.61261582	0.41	0.241	5.23E-80	3
IL6ST	3.26E-83	0.78536081	0.72	0.537	6.53E-80	3
ACTA2	1.47E-82	-0.5877545	0.983	0.994	2.94E-79	3
MYL9	3.23E-82	-0.5289945	0.991	0.985	6.46E-79	3
PPP1R14A	1.41E-81	-0.6602536	0.911	0.936	2.83E-78	3
PRSS35	1.47E-81	0.63609154	0.405	0.246	2.95E-78	3
FXYD6	1.49E-81	-0.3565573	0.236	0.572	2.98E-78	3
FSTL1	1.89E-81	0.73832674	0.64	0.396	3.77E-78	3
GLDN	2.20E-81	-0.4713777	0.06	0.363	4.41E-78	3
SERPINF1	2.26E-80	-0.826389	0.405	0.742	4.52E-77	3
H19	7.52E-77	1.5039744	0.57	0.439	1.50E-73	3
4-Sep	1.14E-76	-0.7543819	0.452	0.78	2.28E-73	3
ITIH3	2.37E-76	-0.3695594	0.162	0.417	4.74E-73	3
CX3CL1	6.05E-76	-0.6507111	0.068	0.38	1.21E-72	3
CFD	6.96E-76	1.11375007	0.736	0.584	1.39E-72	3
KCTD12	7.98E-76	0.64325657	0.504	0.334	1.60E-72	3
ALKAL2	4.81E-75	-0.2630504	0.199	0.473	9.61E-72	3
CCDC3	8.29E-75	-0.6359347	0.551	0.838	1.66E-71	3
MAP3K7CL	3.52E-73	-0.7423259	0.178	0.478	7.04E-70	3
RGS7BP	1.16E-72	-0.4885128	0.188	0.463	2.31E-69	3
CBLN1	1.18E-72	-0.3932667	0.174	0.415	2.36E-69	3
COL6A1	1.92E-72	0.51309785	0.931	0.839	3.84E-69	3
CD9	2.56E-72	-0.8771762	0.394	0.676	5.12E-69	3
BASP1	1.53E-71	0.41629887	0.324	0.163	3.06E-68	3
MBP	2.25E-70	0.35424455	0.347	0.195	4.50E-67	3
NR2F1	4.84E-70	0.82902411	0.426	0.297	9.68E-67	3
CRLF1	6.95E-70	0.45477468	0.349	0.164	1.39E-66	3
ASAH1	1.25E-69	0.69875012	0.688	0.541	2.51E-66	3
VGLL3	1.39E-69	0.69881457	0.454	0.264	2.78E-66	3
LAMA4	3.86E-69	0.92361086	0.611	0.472	7.73E-66	3
CAVIN3	8.87E-69	-0.6562346	0.894	0.956	1.77E-65	3
PPP1R12B	2.73E-68	-0.7544333	0.674	0.868	5.46E-65	3
COL15A1	5.01E-68	-0.2964536	0.066	0.315	1.00E-64	3
NTRK2	5.67E-67	-0.6774596	0.65	0.888	1.13E-63	3
PDGFB	1.35E-66	-0.3879415	0.243	0.481	2.71E-63	3
FILIP1L	5.39E-65	0.70247163	0.848	0.71	1.08E-61	3
CYFIP2	3.73E-64	0.740765	0.358	0.169	7.45E-61	3
LINC00924	8.52E-64	1.2716797	0.494	0.358	1.70E-60	3
PRSS23	1.07E-62	0.56125303	0.395	0.218	2.14E-59	3
MYO10	3.96E-62	0.72192582	0.29	0.108	7.93E-59	3
ATP1A2	4.34E-62	-0.5741347	0.398	0.666	8.67E-59	3
PLPP3	8.35E-62	-0.5309418	0.205	0.49	1.67E-58	3
FBLIM1	1.58E-61	0.60213779	0.52	0.315	3.15E-58	3
TBX3	1.59E-61	-0.3129101	0.237	0.509	3.17E-58	3
GPX3	3.56E-61	0.57620179	0.669	0.5	7.11E-58	3
PTN	4.63E-61	-1.0689476	0.358	0.643	9.27E-58	3
HIGD1B	1.95E-60	-0.7343007	0.284	0.548	3.89E-57	3
MGP	2.03E-60	0.56446892	0.947	0.924	4.06E-57	3
PLIN2	4.13E-60	0.69796694	0.38	0.193	8.26E-57	3

CYGB	1.14E-59	0.45147629	0.31	0.198	2.29E-56	3
ACTC1	2.16E-59	-0.4344802	0.057	0.318	4.33E-56	3
MT1L	9.42E-59	-0.6172416	0.244	0.536	1.88E-55	3
COL14A1	4.55E-58	0.3487709	0.88	0.701	9.10E-55	3
DUSP6	1.71E-56	0.31273311	0.258	0.133	3.41E-53	3
MFAP4	1.57E-54	0.80816792	0.578	0.403	3.14E-51	3
TPPP3	1.46E-53	0.69007959	0.532	0.402	2.92E-50	3
CADM3	1.85E-53	0.62366829	0.371	0.272	3.71E-50	3
LTBP1	3.06E-53	-0.9874605	0.237	0.503	6.11E-50	3
CLU	6.68E-53	-0.8427396	0.427	0.648	1.34E-49	3
SMOC2	1.03E-52	0.50973406	0.458	0.268	2.07E-49	3
ACKR3	4.62E-52	-0.4157307	0.339	0.565	9.25E-49	3
LRRC32	5.34E-52	-0.3669712	0.336	0.611	1.07E-48	3
ACAN	7.75E-52	-0.3326651	0.165	0.368	1.55E-48	3
PARM1	2.75E-51	0.54035163	0.528	0.363	5.51E-48	3
NUAK1	2.76E-51	0.40246332	0.376	0.244	5.51E-48	3
JCAD	5.25E-51	0.30743145	0.314	0.217	1.05E-47	3
ABCA8	5.77E-51	0.49430125	0.528	0.389	1.15E-47	3
FMO2	1.30E-49	0.6185515	0.395	0.233	2.60E-46	3
HLA-B	1.47E-49	0.46312375	0.854	0.79	2.93E-46	3
LAMB1	4.39E-48	0.43703625	0.348	0.204	8.78E-45	3
IFI6	4.57E-48	0.52837258	0.858	0.787	9.13E-45	3
GLUL	3.17E-47	0.47873226	0.608	0.443	6.34E-44	3
COL4A1	3.33E-46	0.45176973	0.874	0.794	6.66E-43	3
NES	7.79E-46	-0.3692339	0.291	0.547	1.56E-42	3
ID3	2.45E-45	0.71213173	0.817	0.699	4.90E-42	3
SLC40A1	3.69E-45	0.59835849	0.612	0.48	7.37E-42	3
CCDC80	6.82E-45	0.33866588	0.782	0.68	1.36E-41	3
F5	1.54E-44	0.25797518	0.239	0.171	3.08E-41	3
UPP1	2.31E-44	0.5175896	0.306	0.191	4.62E-41	3
C1QTNF1	1.41E-43	-0.5514974	0.497	0.732	2.82E-40	3
SULF2	4.39E-43	0.30784055	0.267	0.164	8.78E-40	3
ADRA2C	9.98E-43	0.34695972	0.314	0.189	2.00E-39	3
MT1F	2.35E-42	0.83446572	0.386	0.266	4.71E-39	3
RGS16	5.74E-42	0.67707466	0.437	0.294	1.15E-38	3
ANGPT2	9.07E-42	0.8815322	0.549	0.457	1.81E-38	3
PDLIM4	2.78E-41	0.35497017	0.217	0.108	5.55E-38	3
RAMP1	3.70E-41	-0.6912644	0.248	0.48	7.41E-38	3
CTSK	1.37E-39	0.47890721	0.321	0.201	2.74E-36	3
FOXS1	1.63E-39	-0.3208707	0.394	0.636	3.27E-36	3
SMTN	1.81E-39	-0.3266088	0.408	0.642	3.62E-36	3
ANK3	3.39E-39	0.71740828	0.376	0.251	6.78E-36	3
PAPPA	1.03E-38	0.35994389	0.286	0.212	2.07E-35	3
HAPLN3	2.95E-38	-0.5199567	0.182	0.399	5.89E-35	3
PCOLCE	1.66E-37	0.54415036	0.557	0.451	3.31E-34	3
MAFB	2.22E-37	-0.3386189	0.368	0.602	4.44E-34	3
ENAH	3.56E-37	0.35400228	0.615	0.465	7.12E-34	3
SUSD5	9.32E-37	-0.7997054	0.081	0.344	1.86E-33	3
SPTBN1	1.29E-36	0.38790359	0.65	0.509	2.58E-33	3
CCDC71L	1.50E-36	0.34380763	0.222	0.122	3.00E-33	3
SCARA5	1.96E-36	-0.4943054	0.199	0.389	3.92E-33	3
GFRA1	2.09E-36	0.63124409	0.541	0.466	4.17E-33	3
CPE	2.20E-36	0.31049467	0.941	0.913	4.40E-33	3

FHL5	2.47E-36	0.68514129	0.491	0.37	4.95E-33	3
PIM1	4.09E-36	0.25470511	0.171	0.087	8.18E-33	3
TIMP1	1.91E-35	0.32640589	0.838	0.736	3.82E-32	3
SELENOP	3.34E-35	0.53562406	0.683	0.595	6.68E-32	3
CXCL14	8.24E-35	-0.4217217	0.256	0.195	1.65E-31	3
PAPPA2	1.13E-34	0.99952604	0.284	0.17	2.27E-31	3
IFI27	7.25E-33	-0.7247804	0.373	0.588	1.45E-29	3
PCSK1N	1.38E-32	0.78707848	0.358	0.289	2.75E-29	3
MDK	2.21E-32	0.82382723	0.518	0.456	4.42E-29	3
PRDX4	7.81E-31	0.29099836	0.546	0.428	1.56E-27	3
ANGPTL1	1.37E-30	0.82021918	0.604	0.559	2.74E-27	3
PTGDS	2.40E-30	1.41399002	0.324	0.217	4.80E-27	3
TMEM47	4.87E-30	0.26371344	0.433	0.329	9.73E-27	3
ADAMTS1	6.24E-30	0.38891963	0.551	0.447	1.25E-26	3
C3	8.64E-30	-0.9697446	0.365	0.534	1.73E-26	3
SOD2	2.67E-29	0.49322735	0.529	0.425	5.35E-26	3
DEPP1	2.28E-28	0.42981764	0.369	0.266	4.57E-25	3
DAB2	4.45E-28	0.34789333	0.296	0.218	8.90E-25	3
VCL	6.66E-28	0.33480141	0.871	0.847	1.33E-24	3
TNMD	1.51E-27	-0.2825248	0.286	0.459	3.02E-24	3
IFIT3	1.08E-26	0.75919043	0.525	0.487	2.15E-23	3
AGT	1.47E-25	0.48418523	0.233	0.152	2.94E-22	3
CCL26	2.44E-25	0.93913485	0.377	0.313	4.89E-22	3
FLRT2	2.46E-25	0.52595594	0.408	0.367	4.92E-22	3
EDIL3	3.13E-25	0.38706615	0.523	0.419	6.25E-22	3
AUXG01000	6.85E-25	0.62300929	0.369	0.339	1.37E-21	3
MT1E	7.10E-25	0.34903365	0.875	0.795	1.42E-21	3
FAM241A	7.37E-25	-0.2892764	0.183	0.368	1.47E-21	3
RGS5	2.35E-24	-0.9161856	0.234	0.464	4.70E-21	3
SOCS3	2.91E-24	0.27045513	0.406	0.303	5.81E-21	3
ISYNA1	2.37E-23	0.41421779	0.815	0.846	4.74E-20	3
EMP1	1.11E-22	0.37155127	0.336	0.251	2.21E-19	3
LTBP2	6.61E-22	0.33225625	0.248	0.168	1.32E-18	3
CD36	8.07E-22	0.54895309	0.262	0.197	1.61E-18	3
EPHX1	1.51E-21	0.58523398	0.516	0.473	3.01E-18	3
SFRP2	2.04E-20	-0.5639078	0.184	0.342	4.07E-17	3
MFAP5	2.20E-20	-0.4445065	0.096	0.268	4.41E-17	3
TNFRSF1B	2.87E-20	0.40671773	0.301	0.255	5.74E-17	3
TCIM	4.64E-20	0.76722543	0.311	0.244	9.29E-17	3
F10	5.74E-20	0.59925456	0.574	0.534	1.15E-16	3
SLIT3	4.05E-19	0.30200736	0.847	0.854	8.10E-16	3
EZR	4.59E-19	0.30222902	0.354	0.278	9.17E-16	3
SOX4	4.61E-19	-0.3875004	0.588	0.754	9.21E-16	3
ACVRL1	4.55E-18	0.37567845	0.314	0.279	9.10E-15	3
PDGFRA	6.36E-18	-0.3019964	0.162	0.157	1.27E-14	3
TSPAN18	1.33E-17	0.38734737	0.342	0.298	2.66E-14	3
FOXC2	9.95E-17	-0.4142121	0.183	0.357	1.99E-13	3
RAPGEF5	1.04E-16	-0.3422131	0.124	0.312	2.07E-13	3
COL5A1	2.89E-16	0.40784373	0.514	0.474	5.78E-13	3
PI15	6.48E-16	0.71905898	0.392	0.357	1.30E-12	3
FST	7.05E-16	0.97650345	0.39	0.325	1.41E-12	3
PCDH9	1.19E-15	0.3318021	0.234	0.178	2.38E-12	3
MX2	2.32E-15	0.51455312	0.383	0.37	4.64E-12	3

LDB2	2.44E-15	0.37331107	0.381	0.352	4.88E-12	3
ESAM	1.66E-14	-0.2613175	0.521	0.68	3.33E-11	3
ADA	2.71E-14	0.31929895	0.275	0.262	5.41E-11	3
NID1	2.72E-14	0.53404539	0.444	0.402	5.44E-11	3
CTSB	5.32E-14	-0.3320884	0.473	0.615	1.06E-10	3
KCNK3	2.27E-13	0.54632302	0.308	0.266	4.54E-10	3
SLC2A3	4.42E-13	0.46811711	0.441	0.41	8.83E-10	3
LRRC17	5.29E-13	0.27992801	0.324	0.284	1.06E-09	3
SRPX	9.15E-13	0.52961472	0.418	0.389	1.83E-09	3
NOTCH3	2.37E-12	-0.2827783	0.932	0.979	4.73E-09	3
MAP1B	3.47E-12	-0.3066645	0.876	0.875	6.94E-09	3
CD93	1.54E-11	0.28313288	0.358	0.331	3.07E-08	3
SLPI	4.55E-11	-0.2696165	0.064	0.201	9.10E-08	3
ID1	2.09E-10	-0.6555902	0.169	0.354	4.18E-07	3
CCL13	2.77E-10	0.33817027	0.143	0.133	5.53E-07	3
MEST	3.12E-10	0.37018227	0.322	0.33	6.23E-07	3
ITM2A	8.25E-10	-0.3514923	0.155	0.291	1.65E-06	3
SORBS1	1.21E-09	-0.3525193	0.391	0.515	2.42E-06	3
NEURL1B	1.58E-09	0.43086543	0.643	0.685	3.17E-06	3
FBXO32	6.54E-09	-0.393702	0.294	0.431	1.31E-05	3
SLC22A3	1.07E-08	-0.332193	0.152	0.19	2.14E-05	3
OGN	1.21E-08	-0.4126085	0.171	0.312	2.41E-05	3
ALCAM	1.51E-08	-0.2876134	0.125	0.253	3.03E-05	3
SLFN5	2.05E-08	0.36246318	0.458	0.47	4.11E-05	3
TGM2	4.02E-08	-0.6227907	0.164	0.299	8.05E-05	3
OSR2	9.70E-08	1.20393536	0.32	0.279	0.00019402	3
STAT1	1.09E-07	0.32400688	0.512	0.518	0.00021835	3
CH25H	1.38E-07	0.38356275	0.162	0.149	0.00027576	3
AC118754.1	1.77E-07	0.38785537	0.24	0.244	0.00035362	3
AQP1	5.14E-07	-0.3896618	0.244	0.371	0.00102815	3
CNTFR	5.32E-07	0.31226465	0.226	0.232	0.00106333	3
TSHZ2	5.47E-07	0.48659572	0.433	0.416	0.00109469	3
CARMN	5.71E-07	-0.4699981	0.92	0.944	0.00114268	3
MEOX2	8.47E-07	0.35759981	0.38	0.382	0.00169447	3
ACTG2	8.89E-07	0.370699	0.75	0.905	0.00177832	3
ITM2C	1.05E-06	0.43071008	0.557	0.57	0.00209451	3
COL5A3	2.05E-06	0.59520007	0.427	0.437	0.00409786	3
MYOCD	2.10E-06	-0.2698796	0.403	0.511	0.00420785	3
CALCRL	2.11E-06	0.54591328	0.399	0.398	0.00422706	3
PLTP	3.24E-06	0.47622081	0.444	0.449	0.0064818	3
NABP1	1.03E-05	0.31069728	0.292	0.397	0.02069684	3
MRTFB	1.35E-05	0.36295413	0.481	0.525	0.02691158	3
FBLN2	0.00022934	-0.2998897	0.167	0.209	0.45868054	3
IFIT1	0.00026457	0.35376596	0.46	0.486	0.52914705	3
TNFRSF12A	0.00029726	-0.4749786	0.145	0.282	0.59451004	3
EPHA4	0.00035592	-0.3239891	0.108	0.235	0.71183506	3
IRF1	0.00040273	0.33091654	0.47	0.498	0.80546865	3
CCN3	0.00044183	-0.3583566	0.085	0.201	0.88365887	3
APOD	0.00067254	-0.7364101	0.299	0.389	1	3
DKK3	0.00072615	-0.2883122	0.335	0.447	1	3
LINC00632	0.00088573	0.29045438	0.488	0.515	1	3
ZNF385D	0.00115716	-0.2860604	0.074	0.205	1	3
PLAU	0.00129186	0.35416035	0.316	0.325	1	3

CRISPLD2	0.00131962	0.25941097	0.443	0.474	1	3
TNFRSF11B	0.00203926	-0.3606346	0.069	0.115	1	3
BCYRN1	0.00233629	0.37181152	0.338	0.354	1	3
IGF1	0.00396413	-0.3947867	0.187	0.288	1	3
CDH13	0.00398607	-0.3643556	0.147	0.261	1	3
SFRP1	0.00425745	-0.2859058	0.16	0.196	1	3
COCH	0.00497852	0.48557365	0.278	0.302	1	3
PRKAR2B	0.0058008	0.37143469	0.241	0.261	1	3
FGL2	0.00829864	-0.2745693	0.146	0.241	1	3
DCN	0	4.08349664	0.998	0.457	0	4
C3	0	3.69940965	0.992	0.492	0	4
FBLN1	0	3.36780432	0.983	0.4	0	4
ABCA8	3.19E-295	2.40060556	0.939	0.376	6.38E-292	4
GSN	2.18E-286	2.11122745	0.998	0.918	4.36E-283	4
MMP2	3.64E-281	2.54260065	0.898	0.232	7.28E-278	4
ITM2A	9.59E-279	2.51869272	0.862	0.246	1.92E-275	4
C1R	9.87E-279	2.53650677	0.988	0.437	1.97E-275	4
CFD	1.68E-277	3.78547883	0.985	0.579	3.36E-274	4
C1S	4.00E-275	2.17417418	0.992	0.403	8.00E-272	4
FBN1	3.36E-262	2.90910495	0.948	0.402	6.73E-259	4
IGF1	7.69E-246	2.72772911	0.823	0.249	1.54E-242	4
PODN	3.59E-236	2.30307396	0.891	0.413	7.19E-233	4
SCARA5	5.12E-236	2.77907333	0.827	0.346	1.02E-232	4
PDGFRA	5.55E-230	1.63624606	0.745	0.127	1.11E-226	4
SFRP2	2.04E-225	2.7641148	0.814	0.3	4.08E-222	4
TSHZ2	3.92E-224	2.07854457	0.846	0.396	7.84E-221	4
FBLN2	6.13E-217	1.80502568	0.749	0.176	1.23E-213	4
IGFBP6	5.66E-216	2.59768062	0.969	0.592	1.13E-212	4
AC080038.1	6.35E-216	1.42640997	0.735	0.135	1.27E-212	4
COL1A2	1.98E-209	1.79454198	0.996	0.869	3.96E-206	4
COL1A1	1.53E-202	1.79652857	0.998	0.821	3.06E-199	4
VCAN	1.45E-200	1.61136602	0.802	0.22	2.90E-197	4
SERPINF1	4.63E-199	1.65205042	0.973	0.693	9.26E-196	4
MGP	7.93E-197	1.77803485	0.998	0.922	1.59E-193	4
FSTL1	1.62E-196	1.77376323	0.931	0.395	3.23E-193	4
PLPP3	6.51E-190	1.7044271	0.869	0.439	1.30E-186	4
ADH1B	4.29E-189	2.07463224	0.758	0.205	8.58E-186	4
MEG3	7.46E-188	1.43115998	0.727	0.188	1.49E-184	4
PLA2G2A	1.91E-184	3.9424858	0.789	0.293	3.81E-181	4
FBLN5	1.85E-182	1.40050384	0.722	0.182	3.69E-179	4
LUM	1.38E-179	1.66916575	0.695	0.176	2.76E-176	4
C7	1.11E-178	2.91857269	0.741	0.249	2.22E-175	4
COL3A1	9.40E-175	1.626269	0.996	0.835	1.88E-171	4
MFAP5	2.23E-173	2.51041903	0.697	0.226	4.47E-170	4
ABCA9	2.26E-168	1.83284005	0.697	0.272	4.53E-165	4
S100A10	1.72E-166	1.66998737	0.829	0.326	3.44E-163	4
NID1	5.18E-163	1.71514226	0.798	0.386	1.04E-159	4
GPX3	2.55E-162	1.61123638	0.925	0.496	5.10E-159	4
EFEMP1	9.29E-161	1.61783088	0.678	0.192	1.86E-157	4
OGN	1.12E-159	1.61520797	0.764	0.273	2.25E-156	4
LTBP4	5.49E-154	1.22831573	0.808	0.283	1.10E-150	4
TGFBR3	1.04E-149	1.55538533	0.777	0.401	2.08E-146	4
CCDC80	1.90E-147	1.22633844	0.958	0.677	3.79E-144	4

APOD	3.61E-146	2.85094354	0.764	0.359	7.21E-143	4
SPOCK1	7.38E-146	1.4636735	0.622	0.2	1.48E-142	4
CFH	6.58E-145	1.21399947	0.718	0.21	1.32E-141	4
ABCA6	2.77E-140	1.54556752	0.66	0.281	5.53E-137	4
ABI3BP	2.91E-138	1.69159023	0.758	0.412	5.81E-135	4
SRPX	5.74E-138	1.59259668	0.737	0.374	1.15E-134	4
CD34	5.38E-136	1.39536177	0.637	0.242	1.08E-132	4
H19	2.11E-134	1.9667916	0.758	0.436	4.22E-131	4
SFRP1	6.40E-133	1.54512951	0.595	0.172	1.28E-129	4
CXCL14	2.59E-132	2.68189373	0.582	0.182	5.19E-129	4
IGFBP3	4.88E-132	1.69054763	0.566	0.156	9.77E-129	4
LAMA2	1.27E-127	0.99193141	0.484	0.091	2.53E-124	4
COL6A3	4.48E-127	1.48512255	0.779	0.385	8.96E-124	4
PDGFRL	1.43E-125	1.44555167	0.614	0.259	2.87E-122	4
GAS1	1.48E-122	1.689435	0.67	0.347	2.96E-119	4
PI16	1.88E-114	1.93311417	0.516	0.17	3.76E-111	4
NEGR1	2.55E-114	0.9905661	0.434	0.096	5.10E-111	4
CD248	2.47E-109	1.35960741	0.739	0.456	4.95E-106	4
PLTP	1.09E-108	1.23024825	0.741	0.434	2.18E-105	4
ABLIM1	2.44E-106	0.98770835	0.455	0.126	4.87E-103	4
MGST1	5.66E-105	1.13027791	0.417	0.101	1.13E-101	4
PMP22	2.51E-104	1.03780169	0.848	0.519	5.03E-101	4
OLFML3	2.25E-101	0.98483474	0.639	0.279	4.49E-98	4
ABCA10	2.91E-101	1.53861302	0.62	0.351	5.82E-98	4
DPT	1.23E-95	0.81128513	0.367	0.086	2.47E-92	4
ITGBL1	2.04E-94	0.86978888	0.42	0.111	4.07E-91	4
SEMA3C	5.52E-94	1.4786967	0.514	0.217	1.10E-90	4
LTBP1	6.36E-93	1.3073914	0.806	0.458	1.27E-89	4
LOX	9.73E-92	1.25327701	0.526	0.231	1.95E-88	4
PTGIS	1.68E-91	0.86563905	0.56	0.21	3.35E-88	4
GPC3	4.87E-91	1.08085078	0.631	0.337	9.74E-88	4
TNXB	1.20E-89	1.03079146	0.532	0.22	2.40E-86	4
IGSF10	3.56E-89	1.34882549	0.566	0.294	7.12E-86	4
TIMP1	1.14E-87	0.93842082	0.942	0.736	2.28E-84	4
MARCKS	2.53E-87	1.25793251	0.816	0.597	5.05E-84	4
RARRES1	8.84E-86	1.92975074	0.532	0.254	1.77E-82	4
CHRDL1	2.60E-85	1.11559146	0.603	0.335	5.19E-82	4
SLIT2	1.49E-83	0.99642503	0.493	0.216	2.97E-80	4
MYOC	2.16E-83	1.11275653	0.298	0.075	4.33E-80	4
DCLK1	3.23E-83	1.11780469	0.693	0.457	6.46E-80	4
PPL	3.68E-82	1.00166565	0.516	0.255	7.35E-79	4
LAMB1	8.12E-82	0.95402484	0.499	0.204	1.62E-78	4
PLAT	2.70E-81	0.8210968	0.426	0.16	5.39E-78	4
FLRT2	3.03E-81	1.30984233	0.595	0.359	6.05E-78	4
EMP1	4.45E-77	0.84207709	0.522	0.247	8.91E-74	4
PRELP	4.73E-77	0.88467107	0.848	0.541	9.46E-74	4
COL14A1	1.38E-76	0.8320948	0.94	0.708	2.76E-73	4
COL5A1	4.06E-76	0.93651522	0.71	0.466	8.12E-73	4
COL6A1	4.31E-75	0.69875892	0.983	0.842	8.61E-72	4
BICC1	5.15E-71	1.02800945	0.48	0.245	1.03E-67	4
HTRA3	9.83E-71	1.45565526	0.526	0.299	1.97E-67	4
PCOLCE	1.27E-70	0.80643903	0.741	0.447	2.54E-67	4
CALD1	3.21E-69	-0.6206496	1	0.991	6.43E-66	4

AKAP12	2.15E-68	0.90533097	0.877	0.653	4.30E-65	4
SPTBN1	5.35E-68	0.73707279	0.831	0.508	1.07E-64	4
MSX1	2.18E-66	0.62905246	0.328	0.132	4.35E-63	4
SNED1	9.21E-66	0.50678437	0.276	0.098	1.84E-62	4
ALDH1A1	1.39E-62	0.57730374	0.62	0.312	2.79E-59	4
CILP	4.70E-62	1.21225061	0.447	0.242	9.40E-59	4
FAM43A	5.29E-62	0.4564026	0.273	0.105	1.06E-58	4
MEDAG	5.93E-62	0.65347831	0.372	0.155	1.19E-58	4
VIT	1.60E-61	0.76833412	0.338	0.152	3.20E-58	4
CXCL12	2.89E-61	0.91187363	0.641	0.363	5.77E-58	4
IL6ST	5.03E-60	0.67512022	0.816	0.542	1.01E-56	4
KCNQ1OT1	2.29E-59	1.04099531	0.649	0.434	4.59E-56	4
SPON1	2.26E-58	0.35908473	0.23	0.08	4.52E-55	4
TMEM45A	1.27E-57	0.48514756	0.307	0.136	2.54E-54	4
PCOLCE2	1.33E-57	0.86795645	0.338	0.146	2.67E-54	4
FN1	3.00E-57	0.63128031	0.845	0.547	6.00E-54	4
TPM1	5.29E-57	-0.6604009	1	0.981	1.06E-53	4
NNMT	1.43E-56	0.63196157	0.449	0.209	2.87E-53	4
LRRC17	1.50E-56	0.65136098	0.518	0.277	3.01E-53	4
CLU	1.59E-56	0.86462385	0.864	0.613	3.18E-53	4
IGFBP4	2.67E-56	0.6805111	0.841	0.561	5.34E-53	4
SLPI	1.45E-55	1.20581938	0.365	0.177	2.91E-52	4
RSPO3	7.15E-55	1.35490113	0.491	0.313	1.43E-51	4
FLNA	8.49E-55	-0.7054643	1	0.986	1.70E-51	4
CLMP	2.67E-54	0.75245395	0.415	0.226	5.34E-51	4
CRLF1	3.12E-54	0.59576883	0.388	0.173	6.24E-51	4
PLD3	1.82E-53	0.57612302	0.641	0.369	3.65E-50	4
BOC	1.84E-53	1.04728853	0.509	0.33	3.68E-50	4
NOVA1	2.40E-53	0.4591118	0.336	0.152	4.81E-50	4
SVEP1	1.13E-52	1.39907517	0.491	0.315	2.26E-49	4
FMO2	2.91E-52	0.84234412	0.457	0.239	5.82E-49	4
RAMP2	9.91E-52	0.63447164	0.349	0.173	1.98E-48	4
IL11RA	1.45E-51	0.53604841	0.386	0.193	2.89E-48	4
DAB2	2.39E-51	0.49778038	0.424	0.215	4.78E-48	4
TAGLN	3.39E-51	-0.6112764	1	0.997	6.79E-48	4
CYP1B1	1.22E-50	0.59161368	0.257	0.117	2.44E-47	4
BASP1	7.53E-50	0.6025774	0.338	0.171	1.51E-46	4
AOX1	8.93E-50	0.42740514	0.221	0.095	1.79E-46	4
SGCD	4.64E-49	0.30343242	0.196	0.089	9.29E-46	4
RHOBTB3	3.64E-48	0.70438273	0.497	0.291	7.29E-45	4
IGFBP5	7.23E-48	0.6495405	1	0.985	1.45E-44	4
CDKN1C	1.08E-47	0.58503494	0.793	0.577	2.15E-44	4
HEG1	1.16E-47	0.34272342	0.292	0.149	2.33E-44	4
FMOD	4.28E-47	0.32824527	0.345	0.164	8.56E-44	4
SELENOP	1.87E-46	0.64831451	0.772	0.595	3.74E-43	4
EPHX1	1.41E-44	0.57494646	0.699	0.466	2.81E-41	4
ACTA2	4.38E-44	-0.5830001	1	0.992	8.77E-41	4
SPRY1	1.53E-43	0.76369968	0.56	0.362	3.06E-40	4
FTL	9.98E-43	0.40549622	1	0.993	2.00E-39	4
A2M	1.78E-41	-0.6934914	0.95	0.937	3.56E-38	4
DDR2	1.08E-40	0.61655593	0.808	0.603	2.17E-37	4
DSTN	7.84E-40	-0.523527	0.998	0.982	1.57E-36	4
FGL2	1.39E-39	0.48261228	0.388	0.223	2.77E-36	4

LIFR	3.70E-39	0.7006844	0.457	0.296	7.40E-36	4
AP002956.1	4.87E-39	-0.6662585	0.977	0.977	9.74E-36	4
MYH11	5.78E-39	-0.6938879	1	0.972	1.16E-35	4
MYLK	9.03E-39	-0.7935769	0.962	0.955	1.81E-35	4
GFRA1	9.38E-39	0.83352625	0.593	0.468	1.88E-35	4
MYL9	2.41E-38	-0.4988685	1	0.985	4.82E-35	4
CAV1	5.46E-38	-0.6049409	0.988	0.98	1.09E-34	4
CTSK	1.19E-37	0.45266244	0.349	0.207	2.39E-34	4
TINAGL1	3.94E-37	-0.7712978	0.902	0.921	7.88E-34	4
CYBA	6.77E-36	0.44167025	0.735	0.57	1.35E-32	4
LAMA4	1.14E-35	0.80197974	0.616	0.48	2.29E-32	4
PRDX4	1.60E-35	0.43476525	0.643	0.429	3.20E-32	4
PCSK5	6.72E-35	0.36945384	0.202	0.122	1.34E-31	4
AKR1C1	7.12E-35	0.27379514	0.213	0.125	1.42E-31	4
ELN	3.43E-34	0.42954836	0.775	0.566	6.86E-31	4
FSTL3	3.83E-34	0.28031284	0.397	0.234	7.66E-31	4
CXCL10	5.33E-34	0.27251278	0.102	0.39	1.07E-30	4
OAF	7.65E-34	0.7037004	0.555	0.421	1.53E-30	4
PPP1R14A	2.63E-33	-0.5778282	0.981	0.931	5.26E-30	4
UAP1	4.37E-33	0.49310353	0.324	0.204	8.73E-30	4
TPM2	5.91E-33	-0.5207149	1	0.994	1.18E-29	4
CRISPLD2	8.64E-33	0.73327311	0.603	0.464	1.73E-29	4
SYNPO2	1.23E-32	-1.0057956	0.808	0.855	2.45E-29	4
HELLPAR	1.92E-31	0.97885779	0.449	0.344	3.85E-28	4
GFPT2	2.51E-31	0.3094062	0.203	0.128	5.02E-28	4
CAVIN1	3.10E-31	-0.4859722	0.992	0.982	6.20E-28	4
CYGB	6.27E-31	0.39975269	0.305	0.204	1.25E-27	4
LSP1	8.53E-31	0.67947485	0.426	0.313	1.71E-27	4
C11orf96	4.17E-30	-0.6528106	0.99	0.952	8.34E-27	4
TNFAIP2	2.32E-29	0.80395514	0.417	0.303	4.65E-26	4
IL33	6.87E-29	0.25584145	0.301	0.202	1.37E-25	4
MARCKSL1	7.05E-29	0.35123292	0.259	0.176	1.41E-25	4
NOTCH3	1.10E-28	-0.6532755	0.969	0.975	2.20E-25	4
GAS7	1.78E-27	0.79216852	0.415	0.309	3.56E-24	4
MEG8	3.07E-27	0.34243684	0.182	0.134	6.14E-24	4
CARMN	3.85E-27	-1.0293287	0.929	0.943	7.69E-24	4
SPARC	8.68E-27	0.36832191	0.987	0.871	1.74E-23	4
LINC00924	2.79E-26	-0.2732754	0.094	0.386	5.58E-23	4
FST	4.54E-26	0.93275669	0.422	0.327	9.09E-23	4
LPP	3.00E-25	-0.5925161	0.971	0.957	6.01E-22	4
CREB5	4.01E-25	0.46398019	0.309	0.221	8.03E-22	4
LMOD1	4.23E-25	-0.604016	0.914	0.878	8.46E-22	4
NDRG1	5.71E-25	0.41571701	0.497	0.364	1.14E-21	4
GPNMB	1.17E-24	0.48482569	0.714	0.6	2.34E-21	4
CNTFR	6.72E-24	0.53452535	0.299	0.228	1.34E-20	4
LEPR	8.72E-24	0.29949848	0.2	0.154	1.74E-20	4
THY1	1.06E-23	0.30749143	0.271	0.21	2.12E-20	4
KCTD12	1.24E-23	0.55825055	0.447	0.346	2.48E-20	4
TFPI	2.17E-22	0.25634913	0.476	0.342	4.35E-19	4
ADGRL3	4.41E-22	-0.3732151	0.203	0.467	8.81E-19	4
C2orf40	4.50E-22	-1.6672062	0.455	0.664	9.01E-19	4
COL5A2	4.88E-22	0.40309921	0.608	0.488	9.75E-19	4
MAP1B	6.16E-22	-0.5605216	0.908	0.873	1.23E-18	4

GNG11	1.47E-21	0.33996081	0.656	0.515	2.95E-18	4
MBP	2.82E-21	0.29895443	0.259	0.209	5.63E-18	4
AEBP1	2.93E-21	0.26478507	0.933	0.762	5.85E-18	4
ADIRF	1.52E-20	-0.4318373	1	0.969	3.04E-17	4
FGF18	5.95E-20	0.50845247	0.234	0.198	1.19E-16	4
FSCN1	6.17E-20	0.38035495	0.265	0.219	1.23E-16	4
ACKR4	7.35E-20	0.38186558	0.211	0.178	1.47E-16	4
CTSH	1.62E-19	0.35274778	0.248	0.202	3.23E-16	4
INPP4B	1.62E-19	-0.5488208	0.417	0.654	3.24E-16	4
ADGRF5	2.33E-19	-0.3768219	0.071	0.348	4.67E-16	4
XBP1	2.48E-19	0.26723661	0.514	0.396	4.95E-16	4
F10	5.04E-19	0.4587689	0.618	0.534	1.01E-15	4
ALKAL2	1.10E-18	-0.2573201	0.202	0.457	2.19E-15	4
HMCN2	2.43E-18	-0.2784945	0.06	0.322	4.86E-15	4
SERPINE2	3.94E-18	0.39683161	0.217	0.192	7.88E-15	4
PTGDS	4.42E-18	0.59107733	0.282	0.225	8.84E-15	4
CCL19	7.61E-18	-0.7148329	0.127	0.383	1.52E-14	4
ACTN1	4.44E-17	-0.5418214	0.854	0.827	8.88E-14	4
TNFSF10	4.63E-17	0.71657195	0.392	0.34	9.26E-14	4
PTGFR	5.54E-17	0.67822342	0.307	0.25	1.11E-13	4
IGF2.1	3.58E-16	0.54564029	0.269	0.224	7.15E-13	4
HIGD1B	9.18E-16	-0.2682029	0.294	0.533	1.84E-12	4
LINC00632	1.00E-15	0.39665802	0.585	0.509	2.00E-12	4
DES	1.64E-15	-1.3701956	0.194	0.437	3.29E-12	4
ADAMTS5	1.69E-15	0.60641464	0.547	0.503	3.37E-12	4
PLP1	3.81E-15	0.2802244	0.165	0.173	7.62E-12	4
FGF10	7.86E-15	0.33648965	0.167	0.17	1.57E-11	4
VEGFD	8.06E-15	0.90438951	0.309	0.272	1.61E-11	4
CADM3	2.76E-14	0.59639245	0.317	0.28	5.53E-11	4
CNN1	5.05E-14	-0.666674	0.848	0.808	1.01E-10	4
TYROBP	5.14E-14	-0.2705824	0.267	0.494	1.03E-10	4
TGFBI	7.06E-14	-0.6901894	0.551	0.715	1.41E-10	4
NR2F2	1.08E-13	-0.6553958	0.956	0.937	2.17E-10	4
VCL	1.35E-13	-0.556877	0.827	0.851	2.70E-10	4
SRPX2	1.85E-13	0.39432431	0.234	0.219	3.70E-10	4
SPON2	3.43E-13	0.64435979	0.386	0.348	6.87E-10	4
ADM	4.18E-13	0.2551501	0.161	0.164	8.36E-10	4
ABCC9	7.91E-13	0.45790163	0.524	0.48	1.58E-09	4
CREB3L1	8.15E-13	0.47581264	0.265	0.245	1.63E-09	4
SYTL2	9.69E-13	-0.5057101	0.463	0.657	1.94E-09	4
ACTG2	1.17E-12	-0.7786883	0.887	0.889	2.34E-09	4
CCL21	2.21E-12	-0.6135668	0.048	0.125	4.43E-09	4
PRCP	5.97E-12	0.28748834	0.372	0.329	1.19E-08	4
RRAD	9.96E-12	-0.5805641	0.729	0.791	1.99E-08	4
SORBS2	1.12E-11	-0.5595583	0.896	0.924	2.24E-08	4
CSRP1	2.25E-11	-0.4629337	0.948	0.885	4.49E-08	4
BCAM	2.25E-11	-0.5537531	0.973	0.928	4.49E-08	4
MRVI1	8.53E-11	-0.5656074	0.702	0.731	1.71E-07	4
PDLIM7	1.11E-10	-0.4719652	0.839	0.837	2.22E-07	4
CRIP2	1.15E-10	-0.4810651	0.856	0.842	2.31E-07	4
SBSPON	1.69E-10	-0.3339507	0.338	0.528	3.38E-07	4
EBF2	1.89E-10	0.34060367	0.612	0.595	3.78E-07	4
EPAS1	2.03E-10	-0.4940041	0.837	0.822	4.07E-07	4

PPP1R12B	5.83E-10	-0.5076783	0.848	0.848	1.17E-06	4
TPPP3	7.28E-10	-0.3087691	0.202	0.426	1.46E-06	4
ITGA10	1.07E-09	-0.2824746	0.063	0.132	2.14E-06	4
DLK1	1.24E-09	0.25445764	0.109	0.056	2.48E-06	4
BGN	1.57E-09	-0.3514186	0.931	0.863	3.14E-06	4
CCDC3	1.99E-09	-0.4758692	0.727	0.812	3.98E-06	4
HES4	5.22E-09	-0.4272891	0.896	0.901	1.04E-05	4
PLN	5.57E-09	-0.4940195	0.837	0.801	1.11E-05	4
CRIP1	7.82E-09	-0.5064922	0.956	0.923	1.56E-05	4
C17orf58	1.53E-08	0.57503237	0.276	0.284	3.05E-05	4
CRIM1	1.73E-08	-0.501755	0.562	0.678	3.47E-05	4
C16orf89	1.96E-08	0.37172365	0.194	0.212	3.92E-05	4
SEMA3B	2.00E-08	0.26764893	0.292	0.288	4.01E-05	4
NEURL1B	2.66E-08	-0.3969069	0.52	0.689	5.33E-05	4
TNFRSF11B	2.73E-08	-0.3939557	0.021	0.115	5.47E-05	4
CAVIN3	3.92E-08	-0.3279765	0.983	0.948	7.83E-05	4
BMPER	5.32E-08	0.52836797	0.25	0.261	0.00010643	4
LGI4	6.16E-08	-0.5005509	0.639	0.75	0.00012325	4
SUSD2	7.03E-08	-0.2929914	0.365	0.546	0.0001405	4
FHL5	1.05E-07	-0.371961	0.186	0.393	0.00021039	4
ANGPT2	2.85E-07	-0.3293863	0.288	0.476	0.00056988	4
PALLD	3.14E-07	-0.4267963	0.787	0.736	0.00062755	4
IGKC	4.43E-07	-0.3986752	0.347	0.524	0.00088558	4
LINC01697	4.76E-07	0.40224132	0.198	0.232	0.00095223	4
NEXN	5.79E-07	-0.4769323	0.608	0.642	0.00115867	4
NTM	6.04E-07	0.6478936	0.294	0.291	0.00120714	4
FILIP1L	7.58E-07	-0.546944	0.76	0.722	0.0015162	4
CRYAB	1.15E-06	-0.4296887	0.893	0.786	0.00230674	4
C1QTNF1	1.54E-06	-0.433941	0.643	0.711	0.00308905	4
GGT5	1.55E-06	-0.3828113	0.349	0.333	0.0030946	4
ISYNA1	1.56E-06	-0.4236133	0.793	0.846	0.00311223	4
GUCY1A1	1.70E-06	-0.3796662	0.837	0.867	0.00339553	4
HLA-DRA	2.18E-06	-0.4959942	0.38	0.531	0.00435033	4
CPXM1	4.52E-06	0.67759252	0.326	0.331	0.00903006	4
TJP1	4.69E-06	-0.4161204	0.432	0.568	0.0093744	4
APOE	6.96E-06	0.45167603	0.651	0.651	0.01392202	4
C1QA	7.31E-06	-0.2936853	0.301	0.469	0.01462174	4
ESAM	8.11E-06	-0.3548825	0.549	0.67	0.01621484	4
PAWR	1.68E-05	-0.5299911	0.468	0.562	0.03362962	4
ID4	1.87E-05	-0.9631701	0.342	0.46	0.03740741	4
CLEC11A	2.06E-05	0.34104908	0.415	0.422	0.04119208	4
BHLHE41	2.21E-05	0.28832109	0.248	0.281	0.04423653	4
DIO3OS	2.43E-05	0.50202325	0.276	0.303	0.04854227	4
ACVRL1	2.57E-05	0.29224537	0.257	0.284	0.0514672	4
LIMS2	2.77E-05	-0.4310973	0.587	0.634	0.05534995	4
TNNT3	2.79E-05	0.48239072	0.257	0.288	0.05573694	4
ADGRD1	3.51E-05	0.55710009	0.29	0.315	0.07014643	4
ITGA8	3.78E-05	-0.5718615	0.61	0.62	0.07561281	4
COL15A1	3.96E-05	0.42504509	0.263	0.291	0.07911552	4
COL4A2	4.33E-05	-0.4790903	0.881	0.849	0.08659941	4
SEMA6A	4.59E-05	0.26017111	0.175	0.336	0.09177201	4
GADD45G	5.11E-05	-0.25857	0.232	0.399	0.10222565	4
SLFN5	6.11E-05	0.26750868	0.461	0.47	0.12221195	4

PLS3	8.04E-05	-0.3579688	0.758	0.766	0.16070045	4
KRT18	9.33E-05	-0.5058645	0.029	0.247	0.18665639	4
TNFAIP6	0.00010351	0.33260888	0.144	0.207	0.20702654	4
HLA-DRB1	0.00010599	-0.2658329	0.155	0.335	0.21197661	4
WNT5A	0.0001107	0.57918269	0.28	0.29	0.22140277	4
GJA4	0.00014278	-0.3312751	0.676	0.748	0.28555288	4
CDH13	0.00014361	-0.2900191	0.194	0.252	0.28721987	4
IFI27	0.00015743	0.37731583	0.549	0.567	0.31486872	4
MGLL	0.00016978	-0.3515	0.808	0.848	0.33956816	4
CDKN1A	0.0001975	-0.3176844	0.215	0.259	0.39499092	4
APOC1	0.00021157	0.42324658	0.251	0.297	0.42314972	4
ATP1A2	0.00023815	-0.3181042	0.534	0.644	0.47629886	4
C12orf75	0.00025893	-0.4558282	0.464	0.561	0.51786749	4
CCND1	0.00029117	-0.4527643	0.808	0.855	0.58234531	4
CPB1	0.00043139	0.70004148	0.255	0.246	0.86277039	4
IFIT1	0.00048393	0.32826527	0.451	0.485	0.9678631	4
ANGPT1	0.00077086	-0.2958075	0.409	0.533	1	4
MYOCD	0.00090241	-0.4308333	0.393	0.505	1	4
TNFRSF12A	0.00091071	-0.3391853	0.215	0.27	1	4
PTP4A3	0.00095124	-0.3603791	0.532	0.636	1	4
ITGA1	0.00096589	-0.5135011	0.495	0.564	1	4
FHL2	0.00129409	-0.3492042	0.092	0.203	1	4
ITIH5	0.00266434	-0.3612656	0.877	0.854	1	4
KCNAB1	0.00270612	-0.3304261	0.726	0.776	1	4
TM4SF1	0.00278985	-1.3968144	0.265	0.402	1	4
COL18A1	0.0028687	-0.4645025	0.891	0.841	1	4
SSTR2	0.00334541	-0.3335707	0.063	0.263	1	4
CKB	0.00427061	-0.4677556	0.841	0.826	1	4
AP000892.3	0.00450287	-0.4147701	0.589	0.597	1	4
DEPP1	0.00690981	-0.4486661	0.209	0.28	1	4
CCN2	0.00690994	-0.5224339	0.363	0.453	1	4
ACTC1	0.00770514	-0.2870816	0.127	0.299	1	4
CD9	0.00932364	-0.3649699	0.676	0.646	1	4
SOCS3	0.00946218	-0.4256812	0.288	0.315	1	4

Table S7. Top 100 cluster-spectific genes used for scoring

gene	p_val	avg_log2FC	pct.1	pct.2	p_val_adj	subtype
RERGL	0	1.57570075	0.824	0.507	0	SMC1
TAGLN	0	1.45247881	0.998	0.873	0	SMC1
CSRP2	0	1.37928089	0.833	0.503	0	SMC1
MYL9	0	1.37069697	0.994	0.769	0	SMC1
SOD3	0	1.35601736	0.979	0.581	0	SMC1
ADIRF	0	1.30483054	0.988	0.702	0	SMC1
TPM2	0	1.30134799	0.995	0.763	0	SMC1
ACTA2	0	1.28935686	0.991	0.842	0	SMC1
DSTN	0	1.26370649	0.994	0.777	0	SMC1
SPARCL1	0	1.26275395	0.987	0.734	0	SMC1
C11orf96	0	1.24340288	0.962	0.683	0	SMC1
C12orf75	0	1.17414945	0.918	0.628	0	SMC1
IGFBP7	0	1.13503956	0.999	0.936	0	SMC1
PLN	0	1.13472413	0.897	0.428	0	SMC1
ACTB	0	1.02507578	1	0.828	0	SMC1
SELENOM	0	0.98870859	0.977	0.579	0	SMC1
CPE	0	0.89193445	0.962	0.781	0	SMC1
RCAN2	0	0.86907253	0.932	0.498	0	SMC1
CCDC3	0	0.77201863	0.866	0.56	0	SMC1
GSTM3	0	0.48235285	0.896	0.594	0	SMC1
LBH	1.17E-305	0.88752203	0.907	0.672	2.35E-302	SMC1
MT1A	7.29E-305	0.43716631	0.751	0.359	1.46E-301	SMC1
NRGN	3.69E-291	0.6780364	0.847	0.516	7.38E-288	SMC1
RGS5	2.51E-289	1.05512897	0.949	0.627	5.01E-286	SMC1
LHFPL6	3.32E-289	0.87880306	0.914	0.573	6.64E-286	SMC1
TINAGL1	8.57E-286	0.79336106	0.943	0.673	1.71E-282	SMC1
CAV1	1.56E-280	0.87449953	0.953	0.691	3.11E-277	SMC1
CAVIN3	4.06E-274	0.91309986	0.946	0.71	8.13E-271	SMC1
FRZB	9.57E-273	0.73962158	0.958	0.608	1.91E-269	SMC1
S100A6	1.99E-268	0.82764803	0.999	0.85	3.97E-265	SMC1
4-Sep	6.59E-263	0.54920244	0.912	0.818	1.32E-259	SMC1
TBX2-AS1	8.65E-262	0.50827219	0.743	0.408	1.73E-258	SMC1
IFITM3	2.08E-261	0.89513634	0.994	0.837	4.16E-258	SMC1
PHLDA2	4.40E-247	0.49391034	0.86	0.607	8.81E-244	SMC1
NET1	8.19E-247	0.75215475	0.84	0.566	1.64E-243	SMC1
PPP1R14A	5.24E-239	0.74245617	0.932	0.572	1.05E-235	SMC1
SMOC2	1.46E-236	0.77370318	0.877	0.789	2.92E-233	SMC1
RRAD	4.57E-235	0.64820201	0.809	0.553	9.14E-232	SMC1
GPRC5C	4.77E-232	0.42897353	0.814	0.61	9.55E-229	SMC1
MFAP4	5.03E-227	0.95564188	0.842	0.457	1.01E-223	SMC1
CSRP1	1.28E-225	0.78521623	0.91	0.653	2.56E-222	SMC1
CAV2	6.88E-225	0.79421101	0.82	0.479	1.38E-221	SMC1
ID4	1.36E-223	0.73844012	0.95	0.694	2.72E-220	SMC1
PTP4A3	6.67E-221	0.66024042	0.737	0.48	1.33E-217	SMC1
HCFC1R1	2.83E-219	0.63000684	0.925	0.723	5.65E-216	SMC1
CD151	1.29E-216	0.74703016	0.944	0.637	2.58E-213	SMC1
TGFB1I1	3.76E-208	0.70935247	0.847	0.571	7.53E-205	SMC1
A2M	8.96E-208	0.69086991	0.973	0.818	1.79E-204	SMC1
S100A4	8.73E-205	0.89068978	0.993	0.821	1.75E-201	SMC1
TM4SF1	2.00E-203	0.47772391	0.934	0.689	3.99E-200	SMC1
PMEPA1	8.28E-203	0.5792321	0.904	0.756	1.66E-199	SMC1

NR2F2	1.14E-202	0.53241662	0.966	0.84	2.28E-199	SMC1
SERPINI1	2.31E-202	0.42661669	0.734	0.509	4.62E-199	SMC1
NUPR1	9.31E-194	0.55987001	0.914	0.592	1.86E-190	SMC1
ANGPT2	1.51E-192	0.43715812	0.83	0.613	3.03E-189	SMC1
SNCG	1.15E-191	0.44585233	0.865	0.575	2.31E-188	SMC1
PHLDA3	2.27E-187	0.43954663	0.806	0.613	4.53E-184	SMC1
MT1M	5.62E-185	0.53829289	0.79	0.444	1.12E-181	SMC1
LGALS1	7.36E-183	0.63375428	0.99	0.683	1.47E-179	SMC1
CRIP2	1.63E-180	0.52651589	0.939	0.689	3.25E-177	SMC1
HES4	1.59E-179	0.44122916	0.832	0.562	3.18E-176	SMC1
CD9	6.20E-179	0.61435502	0.963	0.82	1.24E-175	SMC1
PLS3	4.07E-178	0.60014681	0.896	0.652	8.15E-175	SMC1
MT1E	6.62E-178	0.52783274	0.869	0.563	1.32E-174	SMC1
RASL11A	1.16E-177	0.52997359	0.771	0.537	2.31E-174	SMC1
LMOD1	1.86E-176	0.56774887	0.925	0.612	3.73E-173	SMC1
FXYD1	9.73E-172	0.57513932	0.867	0.486	1.95E-168	SMC1
FILIP1L	5.86E-171	0.73722196	0.905	0.672	1.17E-167	SMC1
ACTC1	2.57E-169	0.67758456	0.671	0.333	5.15E-166	SMC1
TSC22D1	3.11E-169	0.52016553	0.886	0.769	6.21E-166	SMC1
RARRES2	8.00E-169	0.49282405	0.906	0.637	1.60E-165	SMC1
CRYAB	1.48E-167	0.50847745	0.932	0.667	2.97E-164	SMC1
MYH11	5.05E-165	0.57783713	0.957	0.671	1.01E-161	SMC1
ISYNA1	9.99E-163	0.46411595	0.776	0.529	2.00E-159	SMC1
ID3	1.69E-160	0.67915789	0.941	0.634	3.39E-157	SMC1
MFGE8	2.32E-160	0.55928927	0.957	0.708	4.64E-157	SMC1
SERPING1	9.27E-157	0.49315859	0.965	0.723	1.85E-153	SMC1
COL14A1	1.14E-156	0.5218221	0.933	0.585	2.28E-153	SMC1
GUCY1A1	5.31E-155	0.51477266	0.861	0.603	1.06E-151	SMC1
NDUFA4L2	1.06E-152	0.46788418	0.805	0.496	2.12E-149	SMC1
IGFBP5	3.16E-152	0.72390767	0.943	0.693	6.32E-149	SMC1
C2orf40	4.63E-149	0.43090618	0.932	0.833	9.26E-146	SMC1
NNMT	1.36E-148	0.5043414	0.964	0.843	2.73E-145	SMC1
EDIL3	2.51E-148	0.47777786	0.877	0.664	5.02E-145	SMC1
SLC25A4	8.89E-143	0.71901635	0.789	0.68	1.78E-139	SMC1
PDLIM1	2.67E-141	0.49277753	0.854	0.725	5.34E-138	SMC1
IGFBP2	1.61E-137	0.56682522	0.942	0.695	3.23E-134	SMC1
PDLIM3	1.28E-134	0.42629361	0.874	0.559	2.55E-131	SMC1
NEXN	9.82E-134	0.45930355	0.836	0.573	1.96E-130	SMC1
COX7A1	1.35E-132	0.42738754	0.834	0.582	2.70E-129	SMC1
MT2A	1.63E-126	0.48551412	0.966	0.811	3.26E-123	SMC1
KCNMB1	4.10E-123	0.43916334	0.738	0.495	8.20E-120	SMC1
GUCY1B1	5.08E-123	0.42560874	0.742	0.618	1.02E-119	SMC1
TUBA1B	4.42E-121	0.43407683	0.973	0.757	8.84E-118	SMC1
CLU	3.53E-106	0.5196931	0.908	0.707	7.07E-103	SMC1
CYR61	7.91E-97	0.55794761	0.975	0.738	1.58E-93	SMC1
EFHD1	5.26E-86	0.53724792	0.67	0.496	1.05E-82	SMC1
SPINT2	3.94E-85	0.53773374	0.799	0.672	7.88E-82	SMC1
FHL5	3.28E-81	0.45406719	0.594	0.387	6.55E-78	SMC1
ACTG2	2.12E-30	0.42252326	0.557	0.389	4.24E-27	SMC1
IGKC	0	2.00625573	0.983	0.991	0	SMC2
IGLC2	0	1.53952771	0.946	0.958	0	SMC2
IGHA1	0	1.25388822	0.883	0.911	0	SMC2
IGHG1	1.53E-276	0.47388688	0.827	0.814	3.06E-273	SMC2

IGHG3	4.48E-241	0.37338791	0.828	0.84	8.95E-238	SMC2
GPX3	3.21E-221	0.30545076	0.404	0.812	6.42E-218	SMC2
LAMA2	7.86E-188	0.46478504	0.694	0.434	1.57E-184	SMC2
PDGFRA	4.06E-179	0.37265165	0.361	0.749	8.12E-176	SMC2
IGLC3	8.81E-171	0.82137584	0.838	0.874	1.76E-167	SMC2
NR4A2	7.34E-154	0.2913664	0.226	0.544	1.47E-150	SMC2
ABCA6	3.13E-144	0.56906586	0.721	0.711	6.26E-141	SMC2
SLPI	1.16E-142	0.45921137	0.648	0.811	2.31E-139	SMC2
AC007952.4	6.67E-133	0.2888334	0.717	0.629	1.33E-129	SMC2
ANKRD28	2.39E-129	0.72095121	0.778	0.647	4.77E-126	SMC2
THY1	1.99E-127	0.31138988	0.588	0.854	3.98E-124	SMC2
CRISPLD2	7.46E-121	0.29719772	0.344	0.669	1.49E-117	SMC2
DUSP6	1.12E-119	0.54054257	0.813	0.761	2.24E-116	SMC2
CCDC88A	3.36E-116	0.31328897	0.256	0.516	6.73E-113	SMC2
FBN1	3.68E-98	0.58254523	0.371	0.659	7.35E-95	SMC2
ITGA10	1.10E-94	0.46191974	0.359	0.639	2.21E-91	SMC2
CFH	2.08E-92	0.66547772	0.795	0.915	4.16E-89	SMC2
SERPINF1	1.18E-86	0.29122721	0.769	0.829	2.35E-83	SMC2
KLF4	5.16E-86	0.34257943	0.34	0.604	1.03E-82	SMC2
LAMB1	4.02E-84	0.4407245	0.375	0.663	8.03E-81	SMC2
SFRP2	2.71E-78	0.60699808	0.695	0.839	5.41E-75	SMC2
MEG3	2.20E-69	0.9730215	0.573	0.364	4.39E-66	SMC2
APOE	5.06E-69	0.97638269	0.534	0.75	1.01E-65	SMC2
COL12A1	1.85E-65	0.34824223	0.434	0.672	3.70E-62	SMC2
PDGFRB	4.48E-65	0.7071737	0.477	0.758	8.96E-62	SMC2
PRKG1	7.83E-65	0.41741885	0.424	0.731	1.57E-61	SMC2
BIRC3	7.87E-65	0.32712212	0.699	0.6	1.57E-61	SMC2
ELN	4.34E-62	0.45573629	0.475	0.719	8.69E-59	SMC2
USP53	1.18E-60	0.6191104	0.278	0.513	2.36E-57	SMC2
C7	5.61E-57	0.58151071	0.753	0.798	1.12E-53	SMC2
POSTN	4.09E-56	0.38054546	0.794	0.684	8.18E-53	SMC2
EPB41L2	3.13E-55	0.35967801	0.536	0.749	6.26E-52	SMC2
RND3	3.15E-53	0.48760976	0.491	0.737	6.31E-50	SMC2
DCN	3.24E-53	0.85084135	0.704	0.832	6.48E-50	SMC2
MMP2	4.71E-53	0.33037283	0.591	0.692	9.42E-50	SMC2
CCDC102B	5.98E-53	0.79894691	0.87	0.854	1.20E-49	SMC2
COL6A1	2.75E-48	0.46978415	0.426	0.728	5.50E-45	SMC2
CCDC144A	4.61E-47	0.4124325	0.359	0.536	9.22E-44	SMC2
MAP1B	1.53E-46	0.36881822	0.586	0.931	3.07E-43	SMC2
LEPR	5.29E-46	0.32625803	0.485	0.307	1.06E-42	SMC2
MARCKS	3.66E-45	0.31195078	0.86	0.902	7.32E-42	SMC2
AC023157.3	5.39E-44	0.33573727	0.741	0.706	1.08E-40	SMC2
KCNQ1OT1	4.78E-41	0.99128994	0.619	0.628	9.56E-38	SMC2
NUDT4	1.27E-38	0.33269685	0.642	0.78	2.54E-35	SMC2
TNC	1.01E-37	0.68156465	0.854	0.803	2.03E-34	SMC2
COL5A2	1.34E-36	0.39590307	0.353	0.496	2.67E-33	SMC2
EGR1	5.07E-36	0.3189428	0.718	0.928	1.01E-32	SMC2
ART4	5.68E-36	0.39073926	0.483	0.66	1.14E-32	SMC2
CYTOR	1.60E-35	0.3715606	0.775	0.881	3.20E-32	SMC2
PTGIS	1.94E-35	0.29279	0.43	0.594	3.89E-32	SMC2
CFD	5.77E-35	0.60676525	0.282	0.414	1.15E-31	SMC2
PCDH7	6.37E-33	0.46584801	0.448	0.692	1.27E-29	SMC2
COL6A2	1.29E-31	0.42939379	0.553	0.883	2.59E-28	SMC2

HSPA1B	6.61E-31	0.7361804	0.758	0.879	1.32E-27	SMC2
HSPH1	3.18E-29	0.66077891	0.7	0.646	6.36E-26	SMC2
CCDC80	3.36E-29	0.89435032	0.755	0.887	6.72E-26	SMC2
LRP1	8.76E-29	0.46332237	0.422	0.613	1.75E-25	SMC2
MAP1A	4.00E-28	0.27865667	0.724	0.588	8.00E-25	SMC2
SULF1	2.50E-26	0.53802792	0.54	0.695	5.00E-23	SMC2
COL1A2	1.33E-24	0.68481569	0.58	0.873	2.66E-21	SMC2
HSPA1A	7.95E-24	0.80258713	0.873	0.941	1.59E-20	SMC2
ITGA1	2.56E-23	0.7782628	0.51	0.684	5.13E-20	SMC2
SPTBN1	1.20E-22	0.62890879	0.538	0.713	2.39E-19	SMC2
IER5	6.39E-20	0.4326709	0.772	0.765	1.28E-16	SMC2
BTG2	2.56E-19	0.37564506	0.742	0.81	5.13E-16	SMC2
PLA2G2A	1.75E-18	0.52910505	0.37	0.532	3.51E-15	SMC2
DNAJB1	2.03E-18	0.56319059	0.737	0.853	4.06E-15	SMC2
SERPINE1	5.30E-18	0.56178511	0.586	0.773	1.06E-14	SMC2
DPYSL3	7.77E-18	0.29748871	0.435	0.524	1.55E-14	SMC2
AEBP1	9.55E-18	0.39891628	0.741	0.924	1.91E-14	SMC2
HEG1	1.05E-17	0.43395519	0.433	0.576	2.09E-14	SMC2
CCL21	2.20E-17	0.94894364	0.811	0.812	4.41E-14	SMC2
FSTL1	1.08E-16	0.31298636	0.556	0.635	2.16E-13	SMC2
SOX4	3.60E-14	0.85680775	0.832	0.839	7.19E-11	SMC2
HSPG2	6.24E-14	0.59890008	0.314	0.368	1.25E-10	SMC2
ABI3BP	3.95E-13	0.39314952	0.438	0.355	7.90E-10	SMC2
CCL19	8.77E-13	2.2362213	0.862	0.847	1.75E-09	SMC2
COL3A1	1.41E-12	1.10604609	0.565	0.724	2.82E-09	SMC2
IER3	2.74E-12	0.68843716	0.842	0.838	5.48E-09	SMC2
SAT1	3.27E-12	0.54331068	0.663	0.779	6.55E-09	SMC2
SOD2	5.29E-12	0.70709007	0.769	0.666	1.06E-08	SMC2
ABHD5	8.28E-12	0.51994232	0.709	0.75	1.66E-08	SMC2
ABCA9	3.34E-10	0.28548646	0.316	0.34	6.67E-07	SMC2
F2R	4.27E-10	0.27917393	0.707	0.751	8.53E-07	SMC2
PDK4	6.13E-10	0.27792435	0.329	0.448	1.23E-06	SMC2
GPRC5A	9.52E-10	0.38951936	0.502	0.434	1.90E-06	SMC2
CPXM2	2.27E-09	0.36197105	0.659	0.598	4.53E-06	SMC2
SVEP1	6.33E-09	0.30468504	0.511	0.67	1.27E-05	SMC2
LAMA4	6.97E-08	0.46478501	0.808	0.814	0.00013947	SMC2
THBS2	3.83E-07	0.38396575	0.792	0.864	0.00076588	SMC2
TNXB	1.37E-06	0.89128699	0.373	0.421	0.00273616	SMC2
COL6A3	1.53E-06	1.04897281	0.618	0.588	0.00306823	SMC2
STEAP4	2.76E-06	0.65957567	0.873	0.862	0.00552098	SMC2
ABCA8	2.99E-06	0.55787739	0.491	0.645	0.00597227	SMC2
PI16	6.94E-06	0.44446175	0.328	0.406	0.01388038	SMC2
UACA	2.07E-05	1.17932185	0.672	0.857	0.04141962	SMC2
CRABP1	2.13E-186	0.45061661	0.873	0.575	4.26E-183	SMC3
DLX6-AS1	5.49E-184	1.86563296	0.9	0.609	1.10E-180	SMC3
NDNF	4.60E-177	1.13017329	0.892	0.638	9.20E-174	SMC3
PDE5A	8.00E-161	1.63836835	0.985	0.686	1.60E-157	SMC3
GREM2	1.73E-155	1.57415156	0.882	0.751	3.47E-152	SMC3
LMO2	2.10E-152	1.66015823	0.877	0.573	4.20E-149	SMC3
EFEMP1	1.33E-150	1.50190431	0.992	0.819	2.66E-147	SMC3
FRZB	1.88E-150	1.48292971	0.996	0.767	3.76E-147	SMC3
DLX5	3.34E-110	1.37232946	0.821	0.663	6.67E-107	SMC3
ATP1B1	5.89E-106	1.39692326	0.832	0.692	1.18E-102	SMC3

RAMP1	1.10E-103	1.13789809	0.958	0.612	2.21E-100	SMC3
ENPP2	7.16E-102	1.26757524	0.8	0.588	1.43E-98	SMC3
CDO1	4.67E-100	1.12450645	0.861	0.627	9.35E-97	SMC3
C2orf40	1.40E-99	1.00580942	0.983	0.875	2.81E-96	SMC3
PHGDH	3.37E-97	0.53331211	0.846	0.399	6.74E-94	SMC3
NBL1	4.74E-93	1.02597601	0.911	0.792	9.48E-90	SMC3
OGN	5.85E-89	1.00356284	0.975	0.746	1.17E-85	SMC3
IGFBP6	2.69E-88	1.40627916	0.938	0.613	5.38E-85	SMC3
LMO7	3.80E-85	1.04738055	0.823	0.485	7.61E-82	SMC3
IGFBP2	2.84E-83	0.76257991	0.994	0.805	5.69E-80	SMC3
OSR1	1.01E-76	0.84359151	0.738	0.312	2.01E-73	SMC3
S100A6	7.74E-74	0.65831283	0.996	0.92	1.55E-70	SMC3
SUCNR1	3.44E-69	1.43499064	0.713	0.542	6.88E-66	SMC3
LGALS3BP	7.73E-69	0.69222735	0.936	0.833	1.55E-65	SMC3
SNCG	1.06E-65	0.97459304	0.902	0.707	2.13E-62	SMC3
KCNK17	4.13E-64	0.78630138	0.776	0.614	8.25E-61	SMC3
GUCY1A1	4.96E-64	0.7502642	0.965	0.714	9.91E-61	SMC3
DKK3	7.01E-63	0.63280701	0.963	0.756	1.40E-59	SMC3
TIMP3	4.10E-62	0.63351194	0.884	0.626	8.21E-59	SMC3
BGN	5.21E-62	0.63393091	0.998	0.837	1.04E-58	SMC3
LGALS1	1.02E-60	0.6463337	0.996	0.826	2.05E-57	SMC3
ID3	2.32E-60	0.67787136	0.967	0.774	4.65E-57	SMC3
PFN2	8.05E-59	0.64870784	0.769	0.448	1.61E-55	SMC3
RGS3	1.36E-58	0.65021725	0.798	0.549	2.71E-55	SMC3
RARRES2	2.25E-58	0.59857016	0.975	0.756	4.49E-55	SMC3
AKR1C3	4.51E-58	0.58351473	0.892	0.652	9.01E-55	SMC3
CLU	2.60E-57	0.65472499	0.994	0.793	5.20E-54	SMC3
ADIRF	2.32E-56	0.59077	1	0.834	4.63E-53	SMC3
LGALS3	2.43E-55	0.60110096	0.971	0.831	4.87E-52	SMC3
LMO3	3.89E-54	0.75304368	0.759	0.562	7.78E-51	SMC3
ID1	4.89E-54	0.75359841	0.809	0.708	9.78E-51	SMC3
THSD4	1.02E-52	0.79434469	0.686	0.479	2.04E-49	SMC3
S100A10	2.46E-52	0.47473194	0.975	0.868	4.93E-49	SMC3
GSTM3	1.09E-51	0.51497248	0.911	0.734	2.17E-48	SMC3
MECOM	5.20E-51	1.1404385	0.717	0.604	1.04E-47	SMC3
S100A13	2.18E-50	0.63990473	0.869	0.73	4.36E-47	SMC3
S100A4	4.56E-50	0.53397754	1	0.9	9.13E-47	SMC3
PCK1	1.03E-48	0.51333853	0.676	0.585	2.07E-45	SMC3
SOST	5.59E-48	0.86594491	0.699	0.505	1.12E-44	SMC3
PTN	1.13E-47	1.76355471	0.753	0.779	2.25E-44	SMC3
TCEAL2	1.46E-47	0.67707179	0.717	0.387	2.93E-44	SMC3
CCND1	5.07E-47	0.63655476	0.846	0.766	1.01E-43	SMC3
GPM6B	1.19E-45	0.40116052	0.767	0.654	2.38E-42	SMC3
PLK2	3.15E-45	0.86165337	0.761	0.62	6.30E-42	SMC3
CXCL12	1.59E-44	0.45623506	0.884	0.815	3.19E-41	SMC3
MBNL1-AS1	3.22E-44	1.03842084	0.736	0.613	6.43E-41	SMC3
CDKN1C	6.40E-43	0.66880322	0.85	0.749	1.28E-39	SMC3
DPT	3.30E-42	0.99144232	0.802	0.76	6.61E-39	SMC3
LMCD1	3.37E-42	0.54770154	0.84	0.689	6.74E-39	SMC3
IGFBP5	8.23E-41	0.83838007	0.979	0.806	1.65E-37	SMC3
F10	8.77E-41	0.42768287	0.705	0.519	1.75E-37	SMC3
ITM2C	1.57E-40	0.69736411	0.738	0.538	3.14E-37	SMC3
IER5L	2.89E-40	0.45231382	0.89	0.732	5.77E-37	SMC3

SERPING1	3.86E-40	0.43327183	0.988	0.834	7.73E-37	SMC3
PRRX2	2.21E-39	0.59792323	0.827	0.676	4.43E-36	SMC3
HMCN1	1.97E-38	0.49685894	0.732	0.53	3.94E-35	SMC3
GEM	2.44E-38	0.58674456	0.911	0.841	4.87E-35	SMC3
NPC2	4.06E-38	0.42456847	0.95	0.868	8.11E-35	SMC3
GSN	2.56E-37	0.50906763	0.971	0.84	5.12E-34	SMC3
PCOLCE	4.82E-37	0.45313381	0.915	0.817	9.64E-34	SMC3
VASN	1.28E-36	0.48429199	0.834	0.642	2.56E-33	SMC3
PRDM6	1.33E-36	0.50379367	0.694	0.437	2.65E-33	SMC3
COX7A1	1.80E-36	0.49240095	0.904	0.693	3.59E-33	SMC3
IGFBP3	7.30E-35	0.57305135	0.645	0.38	1.46E-31	SMC3
ANXA2	3.26E-34	0.42247282	0.99	0.813	6.53E-31	SMC3
CD9	8.32E-34	0.48077541	0.979	0.885	1.66E-30	SMC3
SELENOM	3.91E-33	0.40448036	0.985	0.764	7.82E-30	SMC3
SOD3	1.85E-32	0.39736631	0.998	0.765	3.71E-29	SMC3
BEX2	2.08E-32	0.65941061	0.59	0.364	4.17E-29	SMC3
IL1R1	2.27E-31	0.49344829	0.751	0.652	4.53E-28	SMC3
NRP1	3.07E-31	0.40325981	0.788	0.625	6.13E-28	SMC3
FXYD1	4.45E-31	0.43424498	0.923	0.659	8.90E-28	SMC3
NRGN	6.99E-31	0.64071859	0.796	0.674	1.40E-27	SMC3
CTGF	1.15E-30	0.57210894	0.99	0.945	2.30E-27	SMC3
RHOB	5.59E-30	0.41075079	0.869	0.585	1.12E-26	SMC3
CRYAB	8.23E-30	0.46355455	0.946	0.789	1.65E-26	SMC3
RSPO2	6.07E-29	0.70581836	0.622	0.584	1.21E-25	SMC3
CD151	1.19E-28	0.41204274	0.979	0.777	2.38E-25	SMC3
TSPAN13	5.72E-24	0.98342986	0.62	0.597	1.14E-20	SMC3
GMDS	1.56E-20	0.48038377	0.738	0.616	3.12E-17	SMC3
STMN1	2.22E-20	0.43192908	0.613	0.416	4.44E-17	SMC3
RGS7BP	2.22E-18	0.57000546	0.563	0.277	4.45E-15	SMC3
GDF10	8.43E-18	0.44781001	0.647	0.686	1.69E-14	SMC3
TMEM98	3.75E-17	0.40925791	0.697	0.589	7.50E-14	SMC3
TPD52L1	5.05E-13	0.51746226	0.595	0.542	1.01E-09	SMC3
DLX6	1.79E-10	0.60585541	0.545	0.432	3.58E-07	SMC3
SCRG1	3.14E-10	0.52616931	0.534	0.393	6.27E-07	SMC3
AKR1C2	4.11E-09	0.41812278	0.518	0.407	8.21E-06	SMC3
CAVIN2	2.89E-06	0.6620294	0.522	0.504	0.00577333	SMC3
LNP1	1.79E-05	0.42653659	0.524	0.506	0.03583505	SMC3
TNFRSF11B	5.34E-146	2.48095924	0.963	0.719	1.07E-142	SMC4
MGP	2.60E-140	1.91255711	1	0.978	5.19E-137	SMC4
NOV	6.03E-139	1.98968112	0.992	0.79	1.21E-135	SMC4
FN1	1.91E-136	2.01786309	1	0.862	3.81E-133	SMC4
ASPN	2.43E-135	1.83340236	0.96	0.674	4.85E-132	SMC4
MMP23B	1.70E-134	1.33464593	0.891	0.529	3.39E-131	SMC4
PRSS23	7.66E-134	1.69462992	0.949	0.709	1.53E-130	SMC4
SFRP4	4.72E-130	1.20676262	0.939	0.579	9.44E-127	SMC4
EFEMP1	2.25E-122	1.57943675	0.984	0.823	4.49E-119	SMC4
ITGBL1	4.00E-114	1.37616138	0.971	0.473	7.99E-111	SMC4
OGN	1.02E-113	1.49893337	0.987	0.75	2.03E-110	SMC4
COL8A1	7.45E-109	1.50081097	0.965	0.672	1.49E-105	SMC4
COL1A2	3.05E-108	1.60924331	0.995	0.765	6.11E-105	SMC4
LTBP2	5.48E-108	1.29927786	0.976	0.732	1.10E-104	SMC4
CYTL1	4.79E-104	0.73194631	0.845	0.691	9.57E-101	SMC4
SUGCT	1.46E-103	1.32199426	0.925	0.698	2.93E-100	SMC4

THY1	1.98E-103	0.73730578	0.893	0.76	3.97E-100	SMC4
FHL1	1.30E-101	1.23914261	0.992	0.813	2.60E-98	SMC4
SERPINE1	2.01E-101	1.22015539	0.877	0.702	4.02E-98	SMC4
BGN	4.49E-101	1.14964928	1	0.84	8.98E-98	SMC4
LUM	4.81E-101	1.71755074	0.952	0.869	9.63E-98	SMC4
VCAN	7.16E-101	1.50504424	0.992	0.916	1.43E-97	SMC4
SCG2	2.68E-98	0.84815833	0.827	0.554	5.35E-95	SMC4
C5orf46	1.18E-95	0.76043679	0.867	0.786	2.36E-92	SMC4
FHL2	2.27E-95	0.69139062	0.901	0.562	4.55E-92	SMC4
CTHRC1	9.73E-95	0.81971067	0.877	0.704	1.95E-91	SMC4
TIMP1	5.15E-93	1.29745329	1	0.961	1.03E-89	SMC4
SPARC	1.23E-91	1.23703192	0.981	0.824	2.46E-88	SMC4
PPP1R14A	4.74E-91	1.23463475	0.984	0.741	9.48E-88	SMC4
IGFBP7	1.09E-90	0.97651945	1	0.966	2.18E-87	SMC4
POSTN	2.27E-89	1.79510852	0.872	0.71	4.55E-86	SMC4
CFH	9.44E-89	1.30074351	0.971	0.87	1.89E-85	SMC4
IGFBP2	1.86E-87	1.32631969	0.997	0.809	3.73E-84	SMC4
CTGF	1.84E-85	1.45749015	1	0.945	3.67E-82	SMC4
AEBP1	5.14E-85	1.15405596	0.995	0.857	1.03E-81	SMC4
OMD	7.70E-85	1.25136433	0.877	0.577	1.54E-81	SMC4
CST3	4.92E-84	0.82556462	0.997	0.898	9.84E-81	SMC4
TNFRSF12A	2.90E-83	1.25826155	0.92	0.662	5.81E-80	SMC4
THBS2	7.59E-83	1.0770598	0.973	0.833	1.52E-79	SMC4
PCOLCE2	8.22E-83	0.98976062	0.789	0.424	1.64E-79	SMC4
HTRA1	2.38E-82	1.00441666	0.981	0.718	4.76E-79	SMC4
PRELP	2.00E-80	1.03688343	0.947	0.601	4.00E-77	SMC4
INHBA	8.16E-80	1.2091526	0.853	0.589	1.63E-76	SMC4
KRT17	3.43E-79	2.03577063	0.832	0.773	6.86E-76	SMC4
COL1A1	3.83E-78	0.93463664	0.949	0.613	7.66E-75	SMC4
COL4A1	1.07E-77	1.16542747	0.949	0.706	2.15E-74	SMC4
LGALS1	1.74E-76	0.84879471	1	0.829	3.48E-73	SMC4
FBLN5	4.17E-76	0.87374868	0.912	0.486	8.35E-73	SMC4
CCDC80	2.68E-75	0.90078349	0.968	0.836	5.35E-72	SMC4
CD55	1.61E-72	0.70772335	0.931	0.734	3.21E-69	SMC4
TPM1	4.18E-72	0.82381456	0.997	0.913	8.36E-69	SMC4
COL4A2	1.60E-71	0.93031838	0.957	0.688	3.19E-68	SMC4
KRT18	9.28E-70	2.08365805	0.792	0.647	1.86E-66	SMC4
SULF1	2.56E-67	0.90469843	0.907	0.629	5.11E-64	SMC4
NEXN	7.23E-67	0.93729159	0.957	0.691	1.45E-63	SMC4
COL3A1	2.20E-65	0.99144897	0.928	0.657	4.39E-62	SMC4
FSTL1	1.77E-64	0.84531889	0.851	0.594	3.55E-61	SMC4
KRT7	2.51E-64	1.23084619	0.757	0.549	5.02E-61	SMC4
IGFBP6	6.29E-63	0.95557918	0.955	0.619	1.26E-59	SMC4
PLAC9	1.30E-62	0.8915531	0.973	0.779	2.61E-59	SMC4
GAS6	1.36E-62	0.94541973	0.965	0.774	2.72E-59	SMC4
PPIC	1.23E-61	0.68826448	0.936	0.693	2.45E-58	SMC4
PRDX4	1.17E-60	0.67504686	0.928	0.761	2.35E-57	SMC4
LTBP1	2.63E-60	0.87929441	0.963	0.718	5.26E-57	SMC4
C2orf40	3.88E-59	0.77743363	0.965	0.878	7.76E-56	SMC4
SSR4	7.59E-59	0.79360021	0.981	0.866	1.52E-55	SMC4
CNN3	2.34E-58	0.74429842	0.981	0.858	4.68E-55	SMC4
COL6A1	3.74E-58	0.85581121	0.912	0.613	7.48E-55	SMC4
COL14A1	3.34E-56	0.89604511	0.987	0.748	6.68E-53	SMC4

IGFBP3	1.86E-55	1.10795401	0.747	0.38	3.73E-52	SMC4
PDLIM3	4.60E-55	0.79848048	0.976	0.704	9.21E-52	SMC4
CTSZ	7.18E-55	0.78258625	0.933	0.757	1.44E-51	SMC4
ACTB	1.26E-54	0.70566016	1	0.91	2.52E-51	SMC4
NUCB2	2.68E-54	0.71336462	0.963	0.749	5.36E-51	SMC4
COL6A2	5.01E-54	0.74905024	0.979	0.763	1.00E-50	SMC4
ACTA2	9.44E-53	0.79327641	1	0.913	1.89E-49	SMC4
MYH10	1.75E-52	0.8004669	0.976	0.71	3.50E-49	SMC4
DKK3	1.66E-50	0.69880573	0.949	0.762	3.31E-47	SMC4
TAGLN	1.50E-49	0.73631575	1	0.933	3.01E-46	SMC4
FMO2	2.79E-49	0.81223355	0.933	0.789	5.57E-46	SMC4
TM4SF1	6.01E-48	0.74619064	0.957	0.805	1.20E-44	SMC4
MFAP4	7.81E-48	0.85143195	0.933	0.636	1.56E-44	SMC4
TPM2	9.19E-47	0.72060304	1	0.873	1.84E-43	SMC4
AKAP12	2.87E-45	0.73394769	0.835	0.651	5.75E-42	SMC4
TMEM47	5.07E-45	0.73132082	0.84	0.53	1.01E-41	SMC4
C1R	1.75E-44	0.72845772	0.987	0.862	3.51E-41	SMC4
EDIL3	2.55E-44	0.76137315	0.955	0.761	5.10E-41	SMC4
ADH1B	1.56E-43	0.71471698	0.859	0.551	3.13E-40	SMC4
NDUFA4L2	3.33E-43	0.84854686	0.84	0.642	6.67E-40	SMC4
ISLR	6.07E-43	0.71490059	0.845	0.706	1.21E-39	SMC4
SPINT2	8.56E-43	0.74682854	0.941	0.724	1.71E-39	SMC4
CRYAB	1.06E-41	0.85710886	0.957	0.792	2.12E-38	SMC4
CYR61	1.23E-40	0.71887196	0.997	0.85	2.47E-37	SMC4
S100A10	1.53E-39	0.77704771	0.973	0.871	3.06E-36	SMC4
CRIM1	1.34E-38	0.67163197	0.805	0.552	2.69E-35	SMC4
ENG	2.43E-32	0.68268668	0.803	0.669	4.87E-29	SMC4
F2R	4.99E-30	0.82212909	0.731	0.737	9.97E-27	SMC4
PLPP1	5.64E-30	0.70542993	0.76	0.622	1.13E-26	SMC4
CYFIP2	3.00E-21	0.81033429	0.635	0.52	6.01E-18	SMC4
CRLF1	3.08E-14	1.06758213	0.565	0.389	6.17E-11	SMC4
TYROBP	1.13E-129	3.41349507	1	0.594	2.25E-126	SMC5
HLA-DRA	4.22E-125	4.76300477	1	0.874	8.43E-122	SMC5
AIF1	2.53E-123	3.47033542	0.981	0.483	5.07E-120	SMC5
CD74	3.41E-123	4.20216641	0.995	0.834	6.82E-120	SMC5
HLA-DPA1	4.19E-123	4.47478537	1	0.761	8.38E-120	SMC5
HLA-DRB1	1.57E-121	4.31519467	0.99	0.665	3.15E-118	SMC5
HLA-DQB1	1.44E-120	3.06195184	0.99	0.695	2.88E-117	SMC5
FCER1G	2.14E-120	3.25849177	0.981	0.504	4.27E-117	SMC5
HLA-DPB1	9.94E-120	3.99287247	0.995	0.729	1.99E-116	SMC5
SRGN	4.71E-118	3.01217796	1	0.706	9.42E-115	SMC5
CYBA	1.31E-117	2.53252069	1	0.847	2.63E-114	SMC5
CTSS	3.50E-117	2.56086363	0.986	0.66	7.00E-114	SMC5
HLA-DRB5	1.20E-116	4.00118484	0.981	0.808	2.40E-113	SMC5
CD14	5.42E-114	3.34706094	0.967	0.503	1.08E-110	SMC5
HLA-DQA1	2.49E-113	3.47539047	0.967	0.497	4.99E-110	SMC5
HLA-DQA2	4.99E-113	2.61770233	0.976	0.704	9.97E-110	SMC5
LYZ	1.67E-111	3.75257408	0.957	0.444	3.34E-108	SMC5
FTL	7.06E-111	2.31555701	1	0.969	1.41E-107	SMC5
HLA-DMA	1.47E-110	2.48224923	0.967	0.689	2.93E-107	SMC5
MS4A6A	7.90E-110	2.65975853	0.947	0.512	1.58E-106	SMC5
SAT1	1.72E-109	2.67694596	1	0.733	3.44E-106	SMC5
LAPTM5	1.95E-108	1.81952953	0.971	0.699	3.91E-105	SMC5

FTH1	9.63E-105	1.86385226	1	0.962	1.93E-101	SMC5
PLAUR	2.18E-104	2.45729074	0.957	0.603	4.35E-101	SMC5
HLA-DMB	2.43E-102	1.65742702	0.923	0.447	4.85E-99	SMC5
IGSF6	4.74E-102	2.02963402	0.928	0.516	9.48E-99	SMC5
ITGB2	5.33E-99	1.8722644	0.947	0.752	1.07E-95	SMC5
CTSB	2.49E-98	2.00161761	0.981	0.551	4.98E-95	SMC5
CD68	7.37E-98	1.80566944	0.933	0.517	1.47E-94	SMC5
PLEK	1.26E-97	2.21465981	0.928	0.494	2.53E-94	SMC5
CYBB	8.34E-97	1.8762846	0.909	0.44	1.67E-93	SMC5
NAMPT	2.11E-96	1.9965283	0.976	0.571	4.23E-93	SMC5
COTL1	3.09E-96	2.07750818	0.933	0.585	6.17E-93	SMC5
SPI1	1.04E-95	1.54315421	0.9	0.392	2.07E-92	SMC5
VAMP8	8.21E-95	1.89728897	0.928	0.71	1.64E-91	SMC5
F13A1	1.34E-94	1.8970049	0.909	0.509	2.69E-91	SMC5
TYMP	2.93E-94	1.61375141	0.967	0.824	5.86E-91	SMC5
TMEM176B	3.93E-92	1.91039614	0.909	0.402	7.85E-89	SMC5
SGK1	4.17E-92	1.96505601	0.928	0.668	8.33E-89	SMC5
CTSH	1.49E-91	1.61624271	0.919	0.408	2.97E-88	SMC5
FCGR2A	1.91E-91	1.77755217	0.904	0.309	3.82E-88	SMC5
CPVL	7.45E-90	2.15812805	0.895	0.448	1.49E-86	SMC5
PYCARD	1.21E-88	1.38803049	0.938	0.719	2.42E-85	SMC5
CD83	2.99E-88	1.87672146	0.904	0.604	5.97E-85	SMC5
C1QA	6.20E-88	3.63341217	0.904	0.545	1.24E-84	SMC5
LST1	2.12E-87	1.57832402	0.9	0.594	4.25E-84	SMC5
FCGRT	2.71E-86	1.62651599	0.967	0.733	5.43E-83	SMC5
HCST	3.14E-86	1.50917685	0.928	0.655	6.27E-83	SMC5
FYB1	9.24E-86	1.49561986	0.914	0.646	1.85E-82	SMC5
C5AR1	6.35E-84	1.97179629	0.88	0.462	1.27E-80	SMC5
CTSC	9.94E-84	1.60467922	0.976	0.86	1.99E-80	SMC5
C1QC	1.62E-83	3.24833103	0.885	0.536	3.23E-80	SMC5
GRN	2.86E-82	1.43883547	0.962	0.719	5.72E-79	SMC5
SERPINA1	1.65E-81	1.51016128	0.866	0.545	3.30E-78	SMC5
GPR183	1.43E-80	2.69772265	0.885	0.514	2.85E-77	SMC5
PTPRE	1.80E-80	1.46367974	0.871	0.45	3.60E-77	SMC5
MNDA	3.90E-80	2.09643079	0.866	0.391	7.80E-77	SMC5
EFHD2	1.04E-78	1.4034403	0.89	0.529	2.08E-75	SMC5
CTSZ	8.91E-76	1.44755161	0.986	0.76	1.78E-72	SMC5
PSAP	1.58E-75	1.40974472	1	0.702	3.16E-72	SMC5
CFD	3.75E-75	1.5500554	0.866	0.355	7.51E-72	SMC5
PLIN2	1.10E-74	1.80771931	0.909	0.592	2.20E-71	SMC5
FCGR3A	3.01E-74	1.87164325	0.842	0.421	6.02E-71	SMC5
GLUL	3.12E-74	1.45348585	0.947	0.503	6.24E-71	SMC5
MAFB	5.13E-73	1.69137516	0.909	0.525	1.03E-69	SMC5
MS4A7	5.73E-73	2.03849252	0.847	0.399	1.15E-69	SMC5
HERPUD1	1.36E-68	1.73731176	0.981	0.754	2.72E-65	SMC5
FABP5	2.75E-67	2.26206742	0.871	0.621	5.51E-64	SMC5
RGS2	3.11E-67	1.78317383	0.861	0.578	6.23E-64	SMC5
ZNF331	2.89E-65	1.77658483	0.871	0.501	5.77E-62	SMC5
FCGR1A	1.00E-64	1.43313869	0.785	0.325	2.00E-61	SMC5
C1QB	7.63E-63	3.44016534	0.847	0.555	1.53E-59	SMC5
CEBPD	3.70E-60	1.39099793	1	0.86	7.40E-57	SMC5
IER3	4.64E-60	1.49414966	0.933	0.836	9.29E-57	SMC5
RGS1	5.11E-59	1.46763904	0.861	0.737	1.02E-55	SMC5

HMOX1	1.08E-56	1.69408584	0.813	0.545	2.17E-53	SMC5
MRC1	1.54E-55	1.52261882	0.823	0.712	3.08E-52	SMC5
S100A9	5.77E-55	3.10458848	0.804	0.628	1.15E-51	SMC5
CCL3	8.60E-52	3.5731105	0.823	0.699	1.72E-48	SMC5
NFKBIA	1.33E-51	1.40939905	0.986	0.88	2.67E-48	SMC5
RGCC	2.82E-50	1.56772762	0.852	0.721	5.63E-47	SMC5
CXCL3	8.43E-48	1.8394275	0.646	0.219	1.69E-44	SMC5
CTSL	1.83E-47	1.48290037	0.89	0.598	3.67E-44	SMC5
CXCL2	5.44E-45	1.84842488	0.742	0.507	1.09E-41	SMC5
PLTP	2.42E-44	1.41649447	0.818	0.584	4.83E-41	SMC5
INSIG1	3.28E-44	1.59377423	0.761	0.61	6.55E-41	SMC5
CH25H	7.63E-42	1.56103242	0.722	0.519	1.53E-38	SMC5
FOLR2	9.21E-38	1.68099258	0.775	0.716	1.84E-34	SMC5
IL1B	2.13E-37	2.02366365	0.675	0.37	4.25E-34	SMC5
CXCL8	2.96E-37	2.29414454	0.718	0.467	5.91E-34	SMC5
AREG	1.24E-35	3.0987883	0.689	0.499	2.47E-32	SMC5
RNASE1	2.75E-31	2.30739174	0.761	0.703	5.49E-28	SMC5
CCL3L1	6.56E-25	2.16949728	0.656	0.574	1.31E-21	SMC5
CCL4L2	6.66E-24	2.99397491	0.818	0.798	1.33E-20	SMC5
CCL4	3.94E-21	2.59283438	0.871	0.871	7.88E-18	SMC5
SELENOP	1.09E-19	1.80480621	0.89	0.833	2.18E-16	SMC5
FCN1	9.68E-18	1.57966466	0.593	0.488	1.94E-14	SMC5
IFI27	1.05E-13	1.41506455	0.813	0.742	2.10E-10	SMC5
S100A8	1.82E-11	2.39266794	0.526	0.445	3.64E-08	SMC5
SPP1	2.44E-06	2.53643769	0.622	0.736	0.00488536	SMC5

Table S8. Tackgenes identified in pseudotime trajectory analysis

gene_short_name	p_value	morans_test_statistic	morans_I	status	q_value
FAM41C	5.73E-05	3.857214416	0.00983377	OK	0.00011702
AL645608.1	2.25E-07	5.04616416	0.01280534	OK	5.09E-07
SAMD11	1.11E-233	32.61869188	0.08373217	OK	7.35E-233
NOC2L	4.23E-09	5.759046757	0.01473487	OK	1.01E-08
HES4	0	79.96941947	0.20557898	OK	0
ISG15	0	45.73470065	0.11754543	OK	0
AGRN	6.45E-06	4.361767468	0.01112354	OK	1.37E-05
TNFRSF18	1.29E-07	5.151556885	0.01310639	OK	2.94E-07
TNFRSF4	3.70E-11	6.512467624	0.01663194	OK	9.28E-11
SDF4	3.74E-179	28.51572734	0.07326529	OK	2.21E-178
B3GALT6	2.70E-18	8.644670713	0.0221521	OK	7.71E-18
UBE2J2	9.75E-48	14.46730769	0.03713106	OK	3.63E-47
PUSL1	6.17E-07	4.850107473	0.01239319	OK	1.37E-06
CPTP	0.00044547	3.322875319	0.00846701	OK	0.00086705
DVL1	6.26E-08	5.285774446	0.0135096	OK	1.44E-07
MXRA8	0	111.8425539	0.28752781	OK	0
AURKAIP1	0	70.4053189	0.18100436	OK	0
CCNL2	3.41E-06	4.499483635	0.01149583	OK	7.34E-06
MRPL20	0	56.49457935	0.14522663	OK	0
ANKRD65	3.68E-17	8.341174979	0.02116527	OK	1.03E-16
VWA1	0	48.50078115	0.12440364	OK	0
ATAD3B	9.58E-05	3.729906928	0.00950093	OK	0.00019348
ATAD3A	2.33E-14	7.541176313	0.01930581	OK	6.24E-14
SSU72	0	45.89388211	0.11796152	OK	0
FNDC10	2.85E-57	15.90656467	0.04078847	OK	1.12E-56
MMP23B	0	60.60189446	0.15564815	OK	0
CDK11B	3.74E-11	6.510651225	0.01666789	OK	9.39E-11
NADK	2.17E-11	6.59229835	0.01687465	OK	5.46E-11
GNB1	0	38.31560812	0.09847	OK	0
GABRD	1.17E-30	11.45077444	0.02897899	OK	3.82E-30
FAAP20	4.90E-212	31.05485772	0.07979553	OK	3.13E-211
SKI	1.02E-19	9.010787147	0.02309317	OK	2.98E-19
RER1	0	71.87786073	0.1847917	OK	0
PEX10	5.00E-10	6.109533069	0.01563076	OK	1.22E-09
HES5	6.53E-13	7.093644608	0.01808834	OK	1.70E-12
AL139246.5	1.20E-57	15.96048366	0.0409504	OK	4.74E-57
TNFRSF14-AS1	1.25E-05	4.215142662	0.01073198	OK	2.63E-05
TNFRSF14	0	38.6875534	0.09941743	OK	0
FAM213B	4.14E-06	4.457842946	0.01137578	OK	8.89E-06
LINC00982	0	52.42566622	0.13469012	OK	0
PRDM16	3.75E-104	21.64029522	0.05552103	OK	1.81E-103
MEGF6	3.50E-280	35.74844934	0.09180198	OK	2.46E-279
TPRG1L	1.49E-16	8.173829069	0.02094382	OK	4.15E-16
WRAP73	2.75E-05	4.033498849	0.01029618	OK	5.69E-05
TP73	6.82E-21	9.303042985	0.02209403	OK	2.03E-20
SMIM1	1.11E-19	9.001478147	0.02305503	OK	3.24E-19
LRRC47	2.71E-08	5.437262239	0.01390432	OK	6.30E-08
C1orf174	3.14E-34	12.14249202	0.03114982	OK	1.07E-33
AJAP1	5.31E-08	5.315883142	0.01333474	OK	1.22E-07
KCNAB2	0	64.78804782	0.16652116	OK	0
RPL22	0	173.7763292	0.44680057	OK	0

ICMT	3.84E-19	8.864665317	0.02271577	OK	1.11E-18
ACOT7	8.47E-133	24.49441933	0.0628792	OK	4.47E-132
TNFRSF25	1.63E-05	4.153817335	0.01057221	OK	3.43E-05
PLEKHG5	6.06E-35	12.27646135	0.0313522	OK	2.06E-34
ZBTB48	0.00043776	3.327747071	0.00847882	OK	0.00085286
KLHL21	2.04E-05	4.10269245	0.01046893	OK	4.26E-05
PHF13	8.12E-05	3.771251914	0.00962058	OK	0.00016464
THAP3	9.03E-16	7.9539558	0.02037192	OK	2.48E-15
CAMTA1	0	66.27327421	0.17037636	OK	0
VAMP3	0	38.95637877	0.10011848	OK	0
PER3	5.83E-76	18.40641626	0.04725685	OK	2.51E-75
TNFRSF9	2.24E-19	8.924411979	0.02280827	OK	6.50E-19
PARK7	0	126.2503078	0.32462818	OK	0
ERRFI1	1.64E-164	27.30948674	0.07014102	OK	9.37E-164
SLC45A1	6.36E-07	4.84393141	0.01230441	OK	1.41E-06
RERE	7.12E-06	4.340010942	0.01108676	OK	1.52E-05
ENO1	0	170.8196071	0.43925846	OK	0
SLC2A5	0	100.353959	0.25793625	OK	0
GPR157	6.97E-245	33.39951927	0.08568476	OK	4.67E-244
SPSB1	2.21E-79	18.82837027	0.04831667	OK	9.68E-79
SLC25A33	2.04E-208	30.78558266	0.07908471	OK	1.29E-207
PIK3CD	1.28E-34	12.21568951	0.03131245	OK	4.35E-34
PIK3CD-AS2	1.07E-07	5.186148192	0.01320535	OK	2.45E-07
CLSTN1	3.06E-33	11.95484308	0.03066562	OK	1.03E-32
CTNNBIP1	2.41E-220	31.66428055	0.08135243	OK	1.56E-219
LZIC	5.20E-72	17.90704006	0.04597773	OK	2.19E-71
RBP7	9.86E-267	34.87205847	0.08957177	OK	6.83E-266
KIF1B	3.01E-112	22.48347795	0.05774508	OK	1.50E-111
PGD	0	47.43119365	0.12189091	OK	0
CENPS	1.11E-46	14.29892543	0.03663386	OK	4.11E-46
CORT	9.13E-06	4.285112677	0.01066899	OK	1.94E-05
DFFA	1.37E-13	7.306735769	0.01871356	OK	3.62E-13
C1orf127	1.29E-13	7.315016301	0.01845119	OK	3.40E-13
SRM	2.93E-230	32.3766027	0.08319369	OK	1.93E-229
AL109811.2	0.00047281	3.306226899	0.00832925	OK	0.00091842
FBXO2	6.48E-47	14.33640002	0.03672016	OK	2.40E-46
FBXO44	3.00E-11	6.543614797	0.0167338	OK	7.55E-11
FBXO6	4.56E-63	16.72159594	0.04291391	OK	1.85E-62
MAD2L2	6.89E-127	23.93332725	0.06146921	OK	3.57E-126
AGTRAP	0	49.89900931	0.12825385	OK	0
C1orf167	9.67E-23	9.745237186	0.02490062	OK	2.95E-22
MTHFR	3.71E-30	11.34990175	0.02910683	OK	1.21E-29
KIAA2013	3.09E-18	8.629057974	0.02211576	OK	8.83E-18
PLOD1	4.00E-11	6.500417059	0.01663672	OK	1.00E-10
MIIP	8.28E-56	15.69426029	0.04028203	OK	3.22E-55
TNFRSF8	4.65E-10	6.12092673	0.01559914	OK	1.14E-09
TNFRSF1B	0	149.5241477	0.38447901	OK	0
DHRS3	1.03E-212	31.10490943	0.07991464	OK	6.60E-212
PDPN	9.09E-133	24.49154991	0.06283225	OK	4.80E-132
PRDM2	0.00031607	3.417439555	0.00871419	OK	0.00062061
KAZN	4.02E-89	19.98112477	0.05124883	OK	1.83E-88
TMEM51	0	41.35206731	0.10625618	OK	0
FHAD1	1.06E-110	22.32497509	0.05728573	OK	5.22E-110

EFHD2	0	255.0789262	0.65597429	OK	0
CASP9	1.75E-52	15.20079604	0.03898728	OK	6.66E-52
DDI2	6.25E-11	6.433012533	0.01645942	OK	1.56E-10
PLEKHM2	5.31E-282	35.86530747	0.09216228	OK	3.74E-281
SLC25A34-AS1	1.24E-163	27.23527052	0.06988615	OK	7.10E-163
FBLIM1	0	182.657954	0.46968701	OK	0
HSPB7	0	100.7245412	0.25891385	OK	0
EPHA2	0.00019316	3.549254915	0.00901201	OK	0.00038414
SZRD1	9.21E-71	17.74627787	0.04556531	OK	3.86E-70
NECAP2	0	43.92636479	0.11289567	OK	0
CROCC	9.36E-08	5.211526474	0.01330887	OK	2.14E-07
BX284668.5	7.89E-47	14.3227209	0.03674916	OK	2.92E-46
MFAP2	0	48.01763293	0.12332766	OK	0
AL049569.1	0	39.44352145	0.07031487	OK	0
ATP13A2	8.96E-15	7.664669052	0.01961968	OK	2.42E-14
SDHB	0	38.91245437	0.10000479	OK	0
PADI2	1.58E-41	13.44796272	0.03426546	OK	5.66E-41
PADI4	4.19E-26	10.50280283	0.02616725	OK	1.32E-25
RCC2	0	39.75730745	0.10215324	OK	0
ARHGEF10L	5.82E-07	4.86164026	0.01242518	OK	1.29E-06
IGSF21	0	149.1894072	0.38355241	OK	0
UBR4	1.11E-16	8.210028288	0.02103646	OK	3.08E-16
MRTO4	4.56E-226	32.07737025	0.08241396	OK	2.98E-225
AKR7A2	0	38.42069261	0.09873897	OK	0
PQLC2	4.04E-10	6.143207107	0.01571138	OK	9.90E-10
CAPZB	0	100.7023127	0.25892309	OK	0
MINOS1	0	71.3237782	0.18336657	OK	0
NBL1	0	128.9254462	0.33148339	OK	0
TMCO4	0.00045995	3.313943525	0.00844368	OK	0.00089428
PLA2G2A	0	44.65607488	0.11414886	OK	0
PLA2G5	7.15E-35	12.26309779	0.03138175	OK	2.43E-34
PLA2G2D	7.81E-23	9.766936017	0.02488599	OK	2.38E-22
UBXN10	5.01E-07	4.891400355	0.01219354	OK	1.11E-06
CAMK2N1	0	53.06024249	0.1363127	OK	0
CDA	2.92E-198	30.01723491	0.0770032	OK	1.81E-197
PINK1	1.61E-70	17.71470516	0.04548067	OK	6.76E-70
DDOST	0	40.28530515	0.10353498	OK	0
HP1BP3	1.28E-60	16.38243135	0.04206041	OK	5.11E-60
EIF4G3	2.32E-25	10.33990597	0.02651564	OK	7.28E-25
ECE1	6.32E-149	25.96400228	0.06665933	OK	3.48E-148
ALPL	3.62E-09	5.785303225	0.01374121	OK	8.64E-09
USP48	1.02E-06	4.750293519	0.01214191	OK	2.23E-06
HSPG2	0	53.0274029	0.13626841	OK	0
CDC42	0	143.9425668	0.37012622	OK	0
ZBTB40	0.00027452	3.455616986	0.00880562	OK	0.0005409
C1QA	0	322.7535956	0.83001879	OK	0
C1QC	0	332.4707561	0.85500929	OK	0
C1QB	0	318.0540216	0.81792828	OK	0
EPHB2	2.18E-85	19.54707947	0.05015297	OK	9.81E-85
KDM1A	9.24E-10	6.010633339	0.01538007	OK	2.24E-09
LUZP1	2.69E-74	18.19780473	0.04672536	OK	1.14E-73
LINC01355	7.39E-05	3.794621524	0.00960235	OK	0.00015015
AL109936.6	1.08E-30	11.45719828	0.02793762	OK	3.55E-30

HNRNPR	7.94E-227	32.13176878	0.08256658	OK	5.19E-226
TCEA3	0	79.978203	0.2055702	OK	0
ASAP3	6.90E-06	4.34717716	0.01101584	OK	1.47E-05
E2F2	0	45.84066456	0.11405165	OK	0
ID3	0	120.4150991	0.30961902	OK	0
RPL11	0	197.6827798	0.50821057	OK	0
ELOA	4.12E-55	15.59201084	0.04002262	OK	1.60E-54
PITHD1	1.44E-129	24.18933951	0.06213631	OK	7.54E-129
LYPLA2	7.44E-64	16.82932481	0.04320213	OK	3.02E-63
GALE	7.01E-161	27.0019529	0.06934417	OK	3.98E-160
HMGCL	2.78E-27	10.75574099	0.02758405	OK	8.88E-27
FUCA1	0	88.56142475	0.22768422	OK	0
PNRC2	1.25E-220	31.68509614	0.08141761	OK	8.05E-220
SRSF10	9.02E-20	9.024589979	0.02313637	OK	2.63E-19
NIPAL3	1.37E-08	5.557354459	0.01419619	OK	3.21E-08
RCAN3	1.58E-67	17.32289793	0.04445556	OK	6.53E-67
SRRM1	0	47.20020812	0.12132097	OK	0
CLIC4	0	85.17617802	0.21898144	OK	0
RUNX3	0	44.67355508	0.1147664	OK	0
SYF2	0	50.24274988	0.12914698	OK	0
RSRP1	6.30E-28	10.89182886	0.02793892	OK	2.02E-27
TMEM50A	0	96.13494262	0.24717817	OK	0
MACO1	2.20E-05	4.085258117	0.01043056	OK	4.59E-05
LDLRAP1	2.37E-07	5.036510072	0.01286647	OK	5.35E-07
AL606491.1	4.24E-05	3.930284934	0.0099881	OK	8.71E-05
MAN1C1	1.71E-11	6.627649791	0.01694938	OK	4.31E-11
SELENON	8.50E-14	7.370441005	0.01887116	OK	2.26E-13
MTFR1L	5.69E-129	24.13256063	0.06198131	OK	2.97E-128
AUNIP	7.59E-36	12.44342353	0.03130955	OK	2.60E-35
STMN1	0	93.20806445	0.23964292	OK	0
SLC30A2	8.28E-05	3.766463909	0.00852214	OK	0.00016778
FAM110D	0	99.1530368	0.25179599	OK	0
ZNF593	8.60E-184	28.88738039	0.07421573	OK	5.15E-183
CEP85	0.00050378	3.288409095	0.00835483	OK	0.00097699
SH3BGRL3	0	238.0192703	0.61208206	OK	0
UBXN11	1.03E-55	15.6804863	0.04023491	OK	4.00E-55
CD52	0	225.726582	0.58044943	OK	0
DHDDS	2.26E-10	6.234742587	0.01595421	OK	5.57E-10
HMGN2	0	74.90989619	0.19258825	OK	0
RPS6KA1	0	39.29322086	0.10095161	OK	0
ARID1A	6.99E-10	6.055741437	0.01549795	OK	1.70E-09
PIGV	7.83E-08	5.244592898	0.01339926	OK	1.79E-07
ZDHHC18	8.77E-05	3.752160538	0.00956746	OK	0.00017742
GPN2	2.64E-06	4.553069104	0.01162831	OK	5.73E-06
NUDC	0	52.79879014	0.13572075	OK	0
TRNP1	0	39.77694051	0.10217508	OK	0
TENT5B	1.27E-154	26.46355823	0.06791067	OK	7.14E-154
TMEM222	1.13E-24	10.18696291	0.02611909	OK	3.53E-24
SYTL1	1.68E-60	16.36555107	0.04189306	OK	6.74E-60
WASF2	0	69.25447896	0.17804348	OK	0
AHDC1	5.52E-05	3.866647133	0.00986076	OK	0.00011269
FGR	0	118.8557619	0.3055682	OK	0
IFI6	0	74.04877992	0.19037114	OK	0

STX12	8.20E-217	31.40667214	0.08069941	OK	5.26E-216
THEMIS2	0	116.8269255	0.30037791	OK	0
RPA2	2.23E-53	15.33495937	0.03936217	OK	8.56E-53
SMPDL3B	1.89E-74	18.21710299	0.04564428	OK	8.05E-74
EYA3	2.86E-13	7.2068033	0.01845348	OK	7.52E-13
PTAFR	0	114.3338767	0.29396039	OK	0
DNAJC8	0	61.84758653	0.1589942	OK	0
ATP5IF1	0	74.55591265	0.19167891	OK	0
SESN2	1.53E-44	13.95203876	0.03579209	OK	5.58E-44
PHACTR4	5.59E-05	3.86342083	0.00986043	OK	0.00011416
RCC1	4.23E-28	10.92814752	0.02799839	OK	1.36E-27
TRNAU1AP	5.03E-85	19.50446632	0.05008331	OK	2.26E-84
SNHG12	8.40E-84	19.35994202	0.04970846	OK	3.75E-83
TAF12	4.97E-129	24.13813935	0.0620028	OK	2.60E-128
RAB42	0	64.01708932	0.16450459	OK	0
AL360012.1	2.58E-07	5.020404941	0.01279678	OK	5.81E-07
GMEB1	1.49E-09	5.932563387	0.0151782	OK	3.60E-09
YTHDF2	4.50E-127	23.95111958	0.06152282	OK	2.33E-126
EPB41	1.55E-18	8.707801728	0.02231242	OK	4.44E-18
TMEM200B	4.36E-09	5.754105313	0.01468229	OK	1.04E-08
SRSF4	3.88E-17	8.334753253	0.02136164	OK	1.09E-16
MATN1-AS1	1.80E-06	4.632862193	0.01175898	OK	3.93E-06
LAPTM5	0	312.5110308	0.80369153	OK	0
SDC3	0	67.05557923	0.17235237	OK	0
SNRNP40	1.15E-55	15.67360781	0.04023273	OK	4.45E-55
ZCCHC17	5.92E-88	19.84636575	0.05096606	OK	2.69E-87
FABP3	6.21E-259	34.3536711	0.08825031	OK	4.24E-258
SERINC2	1.39E-11	6.658240861	0.01701989	OK	3.52E-11
TINAGL1	0	207.3833529	0.53327333	OK	0
PEF1	6.40E-167	27.51150045	0.07067627	OK	3.69E-166
COL16A1	5.72E-250	33.74800746	0.08666317	OK	3.86E-249
ADGRB2	1.84E-07	5.084691731	0.01258576	OK	4.17E-07
SPOCD1	3.92E-277	35.55162146	0.09118932	OK	2.74E-276
PTP4A2	0	56.1077663	0.1442314	OK	0
KHDRBS1	0	45.06794227	0.11583788	OK	0
TMEM39B	1.90E-10	6.262187364	0.01602404	OK	4.68E-10
CCDC28B	1.55E-07	5.117853387	0.01307697	OK	3.51E-07
EIF3I	0	54.94924086	0.14125205	OK	0
FAM167B	0	51.55843158	0.1312696	OK	0
LCK	4.96E-186	29.06507892	0.0745733	OK	2.98E-185
HDAC1	3.58E-65	17.00796283	0.04366342	OK	1.47E-64
MARCKSL1	0	96.47035657	0.24797765	OK	0
ZBTB8A	1.47E-10	6.301483071	0.01609659	OK	3.65E-10
ZBTB8OS	5.79E-167	27.51510624	0.07068963	OK	3.34E-166
RBBP4	5.90E-23	9.795300692	0.02511704	OK	1.80E-22
SYNC	9.02E-56	15.68880689	0.04025944	OK	3.51E-55
YARS	6.81E-25	10.23646545	0.02624586	OK	2.12E-24
FNDC5	1.85E-39	13.09115071	0.03353756	OK	6.51E-39
TMEM54	1.49E-172	27.9784138	0.07182536	OK	8.69E-172
RNF19B	5.54E-37	12.6507199	0.03244826	OK	1.91E-36
AK2	9.64E-68	17.35130953	0.04454874	OK	3.99E-67
ZNF362	3.94E-16	8.056025093	0.02063526	OK	1.09E-15
PHC2	7.61E-53	15.25511949	0.03915771	OK	2.91E-52

CSMD2	0.00028164	3.448704765	0.00841117	OK	0.00055449
SMIM12	1.14E-210	30.95349965	0.07953181	OK	7.24E-210
GJA4	0	91.35087014	0.23475847	OK	0
DLGAP3	3.27E-21	9.381047192	0.02400707	OK	9.74E-21
SFPQ	6.00E-166	27.43011673	0.07047426	OK	3.45E-165
ZMYM4	0.00029383	3.437245668	0.0087645	OK	0.00057772
KIAA0319L	0.00012698	3.658241774	0.00932917	OK	0.00025496
AC004865.2	6.77E-11	6.421066784	0.01641385	OK	1.69E-10
PSMB2	0	53.86660912	0.1384673	OK	0
C1orf216	1.90E-05	4.118957757	0.01051105	OK	3.98E-05
CLSPN	0	107.696947	0.27646024	OK	0
AGO4	6.71E-21	9.304824227	0.02384544	OK	1.99E-20
ADPRHL2	1.13E-29	11.25216804	0.02886047	OK	3.68E-29
COL8A2	1.23E-34	12.21915511	0.03132595	OK	4.17E-34
TRAPPC3	9.16E-184	28.88521032	0.07421473	OK	5.48E-183
MAP7D1	4.76E-166	27.43851497	0.07049195	OK	2.74E-165
THRAP3	0	41.7026995	0.10718275	OK	0
SH3D21	7.96E-09	5.651293543	0.01443258	OK	1.88E-08
EVA1B	3.52E-230	32.3709148	0.08317851	OK	2.32E-229
STK40	1.60E-18	8.703835897	0.02230287	OK	4.60E-18
LSM10	0	41.92319804	0.10774673	OK	0
MRPS15	3.67E-282	35.87561639	0.0921929	OK	2.59E-281
CSF3R	0	50.85182387	0.13067653	OK	0
LINC01137	2.02E-05	4.104942199	0.01046563	OK	4.22E-05
ZC3H12A	3.84E-189	29.31019573	0.07528825	OK	2.33E-188
MEAF6	3.48E-258	34.30347223	0.088151	OK	2.38E-257
SNIP1	0.00040643	3.348381717	0.00853374	OK	0.00079336
DNALI1	1.62E-21	9.454864802	0.02419472	OK	4.85E-21
GNL2	4.75E-62	16.58136982	0.04256548	OK	1.92E-61
C1orf109	9.55E-05	3.730722146	0.00951273	OK	0.00019287
CDCA8	0	73.83775405	0.18874836	OK	0
YRDC	6.41E-64	16.83809727	0.04322282	OK	2.61E-63
C1orf122	0	41.98248711	0.1079006	OK	0
MTF1	3.87E-07	4.941574042	0.01262636	OK	8.67E-07
SF3A3	7.69E-74	18.14009295	0.04657718	OK	3.27E-73
FHL3	9.05E-84	19.35611201	0.04970235	OK	4.04E-83
UTP11	4.61E-104	21.63074282	0.05555343	OK	2.23E-103
RRAGC	9.23E-58	15.97708488	0.04101298	OK	3.63E-57
MYCBP	8.52E-14	7.370235778	0.01887288	OK	2.26E-13
AKIRIN1	5.20E-226	32.07327688	0.08241223	OK	3.39E-225
NDUFS5	0	113.7194438	0.29240087	OK	0
MACF1	1.11E-57	15.9655598	0.04098712	OK	4.37E-57
PABPC4	0	74.0018707	0.19025024	OK	0
HEYL	8.56E-21	9.278863488	0.02372419	OK	2.54E-20
PPIE	1.58E-83	19.32754567	0.04963007	OK	7.02E-83
OXCT2	9.68E-06	4.272130134	0.01086001	OK	2.05E-05
MYCL	4.02E-62	16.5914042	0.04250012	OK	1.62E-61
MFSD2A	2.99E-39	13.05458634	0.0334549	OK	1.05E-38
CAP1	0	87.43786083	0.2248096	OK	0
PPT1	0	125.4318131	0.32252753	OK	0
RLF	9.46E-05	3.732939802	0.00952475	OK	0.00019126
AL050341.2	9.03E-08	5.218261112	0.01332787	OK	2.06E-07
ZMPSTE24	8.07E-31	11.48255309	0.02945264	OK	2.65E-30

COL9A2	4.16E-19	8.855526008	0.02267306	OK	1.20E-18
SMAP2	0	192.8611594	0.49594613	OK	0
RIMS3	3.92E-11	6.503609213	0.0166011	OK	9.84E-11
NFYC	4.33E-47	14.36433583	0.03686408	OK	1.61E-46
KCNQ4	1.34E-10	6.316649474	0.01602079	OK	3.32E-10
CITED4	1.04E-196	29.89820516	0.0768001	OK	6.40E-196
CTPS1	1.46E-35	12.39133538	0.03177541	OK	4.98E-35
SCMH1	2.33E-19	8.919888754	0.02284094	OK	6.77E-19
FOXO6	0.0001854	3.560044406	0.0090122	OK	0.00036903
HIVEP3	6.63E-47	14.33477921	0.0367687	OK	2.46E-46
PPCS	0	38.9340241	0.10006077	OK	0
PPIH	7.47E-143	25.42057037	0.06529771	OK	4.06E-142
YBX1	0	213.563987	0.54914604	OK	0
SVBP	1.46E-160	26.97476849	0.06929974	OK	8.28E-160
ERMAP	1.79E-07	5.089641528	0.01300027	OK	4.06E-07
SLC2A1	4.34E-38	12.84932282	0.03292019	OK	1.51E-37
EBNA1BP2	7.47E-132	24.40550206	0.06268992	OK	3.94E-131
TIE1	1.12E-206	30.65537313	0.07761568	OK	7.06E-206
CDC20	0	175.6367857	0.44953813	OK	0
ELOVL1	3.05E-98	21.00350387	0.05394088	OK	1.44E-97
MED8	5.09E-122	23.46108806	0.06026118	OK	2.60E-121
HYI	0	69.88637766	0.17965361	OK	0
PTPRF	3.21E-34	12.14082391	0.03109849	OK	1.09E-33
ARTN	1.17E-121	23.42566592	0.05981257	OK	5.97E-121
ATP6V0B	0	212.8298557	0.5473092	OK	0
B4GALT2	2.74E-48	14.55439129	0.03733087	OK	1.02E-47
SLC6A9	3.50E-23	9.847972147	0.0250961	OK	1.07E-22
AL139220.2	2.95E-07	4.994412023	0.01269985	OK	6.63E-07
DMAP1	6.75E-07	4.832177789	0.0123509	OK	1.50E-06
ERI3	3.94E-54	15.44716254	0.039649	OK	1.52E-53
RNF220	3.54E-14	7.486363679	0.01917353	OK	9.46E-14
TMEM53	2.92E-54	15.46654314	0.03968285	OK	1.12E-53
ARMH1	3.38E-34	12.13647746	0.03107965	OK	1.15E-33
KIF2C	0	74.17227472	0.18792944	OK	0
RPS8	0	181.7184607	0.46716461	OK	0
BEST4	9.70E-06	4.271652058	0.01061423	OK	2.05E-05
PLK3	1.52E-142	25.39264133	0.06522602	OK	8.26E-142
EIF2B3	2.29E-13	7.237250566	0.01853528	OK	6.03E-13
UROD	5.45E-81	19.02351036	0.04884995	OK	2.40E-80
HPDL	3.64E-08	5.384013888	0.01333119	OK	8.44E-08
PRDX1	0	160.9268761	0.41380446	OK	0
AKR1A1	0	111.6260833	0.28702225	OK	0
NASP	0	45.23711009	0.11626955	OK	0
GPBP1L1	1.03E-10	6.356332335	0.01627141	OK	2.57E-10
TMEM69	2.57E-09	5.842865187	0.01494632	OK	6.14E-09
IPP	4.25E-14	7.462221071	0.01909241	OK	1.14E-13
PIK3R3	2.83E-07	5.002218626	0.01276345	OK	6.37E-07
LURAP1	3.11E-22	9.625796314	0.02464802	OK	9.42E-22
RAD54L	2.21E-100	21.23630261	0.05251525	OK	1.05E-99
LRRC41	1.71E-05	4.143824451	0.01058005	OK	3.58E-05
UQCRH	0	116.0593724	0.29841843	OK	0
MKNK1	0	42.58488927	0.10943973	OK	0
MOB3C	7.20E-07	4.819402375	0.01231161	OK	1.59E-06

ATPAF1	2.81E-46	14.234152	0.03652658	OK	1.04E-45
EFCAB14	2.30E-32	11.78608291	0.03023663	OK	7.69E-32
CYP4B1	4.33E-156	26.5908676	0.06709204	OK	2.43E-155
CYP4X1	2.14E-299	36.96435067	0.09492335	OK	1.55E-298
CYP4Z1	1.24E-22	9.719771123	0.02043638	OK	3.78E-22
TAL1	3.59E-141	25.26801242	0.06294671	OK	1.94E-140
STIL	8.22E-46	14.15899356	0.03588384	OK	3.02E-45
CMPK1	3.25E-179	28.5206557	0.07327731	OK	1.92E-178
FOXD2	1.42E-259	34.39645302	0.088329	OK	9.75E-259
TRABD2B	5.20E-75	18.28756504	0.04688748	OK	2.22E-74
SPATA6	2.86E-13	7.206934122	0.01845557	OK	7.52E-13
BEND5	1.17E-166	27.48949463	0.07056722	OK	6.76E-166
ELAVL4	6.87E-53	15.26178083	0.03909091	OK	2.63E-52
FAF1	7.49E-09	5.661853858	0.01448222	OK	1.77E-08
CDKN2C	0	55.47086336	0.14255462	OK	0
RNF11	2.56E-163	27.20879703	0.06989772	OK	1.46E-162
EPS15	2.98E-103	21.54451638	0.05533134	OK	1.43E-102
OSBPL9	2.91E-51	15.01541491	0.03854036	OK	1.10E-50
NRDC	5.03E-66	17.1226243	0.04396277	OK	2.07E-65
TXNDC12	0	45.81088888	0.11774533	OK	0
KTI12	1.56E-09	5.925067179	0.01515706	OK	3.76E-09
BTF3L4	0	51.17798264	0.13155118	OK	0
ZFYVE9	2.63E-36	12.52771327	0.03210431	OK	9.05E-36
ORC1	2.23E-20	9.176325364	0.02320674	OK	6.59E-20
PRPF38A	3.58E-24	10.07441588	0.02583303	OK	1.11E-23
TUT4	2.32E-13	7.23544232	0.01853356	OK	6.11E-13
GPX7	1.28E-37	12.76527898	0.03273399	OK	4.45E-37
SHISAL2A	1.04E-09	5.991093372	0.01526994	OK	2.52E-09
COA7	7.94E-06	4.316173875	0.01101432	OK	1.68E-05
ECHDC2	9.50E-186	29.04274618	0.07459859	OK	5.71E-185
SCP2	0	45.06739983	0.11583634	OK	0
PODN	5.70E-278	35.60576048	0.09134321	OK	4.00E-277
CPT2	4.26E-14	7.461935264	0.01910406	OK	1.14E-13
C1orf123	0	39.62683522	0.10184019	OK	0
MAGOH	0	43.14652344	0.11089513	OK	0
AC119428.2	3.35E-05	3.986708427	0.01009537	OK	6.91E-05
NDC1	8.42E-07	4.78811449	0.01221392	OK	1.86E-06
YIPF1	2.78E-20	9.152658576	0.02345646	OK	8.19E-20
HSPB11	6.19E-164	27.26076294	0.07003639	OK	3.54E-163
LRRC42	7.87E-14	7.380821551	0.01890022	OK	2.09E-13
TMEM59	0	111.8185538	0.28751166	OK	0
MRPL37	5.85E-93	20.41743303	0.05243188	OK	2.72E-92
SSBP3	8.15E-56	15.69521257	0.04028404	OK	3.17E-55
ACOT11	2.01E-09	5.883552906	0.0150359	OK	4.82E-09
DHCR24	8.42E-06	4.303230982	0.01092799	OK	1.79E-05
PLPP3	4.00E-199	30.08336865	0.07727485	OK	2.48E-198
PRKAA2	1.05E-49	14.77579899	0.03785183	OK	3.96E-49
OMA1	3.51E-05	3.975936547	0.01014624	OK	7.23E-05
TACSTD2	0	101.1198732	0.25857766	OK	0
JUN	0	46.85880603	0.12044109	OK	0
FGGY	2.36E-06	4.577281781	0.01168835	OK	5.11E-06
NFIA	0	155.1787321	0.39901748	OK	0
TM2D1	2.20E-118	23.1021262	0.0593395	OK	1.11E-117

AC099791.2	0.00041915	3.339831599	0.00848611	OK	0.00081776
PATJ	9.72E-67	17.21806884	0.04416801	OK	4.01E-66
USP1	1.85E-90	20.1342973	0.05170439	OK	8.47E-90
ATG4C	3.25E-87	19.76056643	0.05073521	OK	1.47E-86
FOXD3	4.86E-23	9.814849623	0.02200486	OK	1.49E-22
ITGB3BP	1.60E-71	17.84445626	0.04579718	OK	6.71E-71
EFCAB7	0.00024258	3.488823787	0.00887801	OK	0.00047955
PGM1	7.78E-116	22.8472525	0.05867175	OK	3.90E-115
ROR1	1.61E-63	16.78345694	0.04302655	OK	6.55E-63
CACHD1	1.50E-05	4.172896415	0.01058672	OK	3.16E-05
RAVER2	3.91E-25	10.28990107	0.0263291	OK	1.22E-24
JAK1	0	54.66297147	0.14051582	OK	0
AK4	1.88E-75	18.34301979	0.04707101	OK	8.05E-75
LEPROT	0	73.12017334	0.18798623	OK	0
LEPR	7.01E-206	30.59557376	0.07846114	OK	4.40E-205
PDE4B	0	80.17070126	0.20609396	OK	0
SGIP1	2.03E-169	27.71945307	0.07116953	OK	1.18E-168
TCTEX1D1	1.05E-08	5.603360749	0.01428903	OK	2.48E-08
MIER1	5.74E-187	29.13913524	0.07486849	OK	3.46E-186
IL12RB2	1.87E-12	6.946831401	0.01763449	OK	4.83E-12
SERBP1	0	82.28192393	0.21154824	OK	0
GADD45A	0	84.04328189	0.2160644	OK	0
GNG12	0	90.31048174	0.23216788	OK	0
DIRAS3	3.39E-98	20.99835481	0.05383274	OK	1.61E-97
WLS	1.95E-158	26.79308407	0.06882126	OK	1.10E-157
DEPDC1	0	135.1843309	0.34439118	OK	0
LRRC40	0.00024744	3.483518272	0.00887952	OK	0.00048903
SRSF11	2.61E-07	5.017866542	0.01283166	OK	5.88E-07
HHLA3	9.37E-07	4.766573881	0.01217392	OK	2.06E-06
PTGER3	0	43.84824057	0.11263054	OK	0
ZRANB2	1.21E-92	20.38198186	0.05234604	OK	5.61E-92
NEGR1	1.89E-133	24.5553882	0.06298905	OK	1.00E-132
CRYZ	1.50E-13	7.29444768	0.01867646	OK	3.96E-13
TYW3	2.09E-111	22.39734824	0.05752533	OK	1.03E-110
ACADM	8.07E-72	17.88249785	0.04591495	OK	3.40E-71
RABGGTB	3.26E-62	16.60391098	0.04262498	OK	1.32E-61
PIGK	2.77E-91	20.22801121	0.05193853	OK	1.28E-90
AK5	1.81E-30	11.41270316	0.02917449	OK	5.92E-30
USP33	9.96E-09	5.612740227	0.01435932	OK	2.35E-08
MIGA1	8.73E-11	6.382252701	0.01633573	OK	2.17E-10
NEXN	0	225.8320179	0.58073165	OK	0
FUBP1	1.69E-10	6.28033223	0.01607671	OK	4.18E-10
DNAJB4	0	40.84723834	0.10496896	OK	0
GIPC2	6.38E-32	11.6998708	0.0298837	OK	2.12E-31
AC103591.3	9.36E-54	15.39129525	0.03948604	OK	3.60E-53
PTGFR	1.02E-15	7.938325051	0.0195817	OK	2.81E-15
IFI44L	1.35E-217	31.46409383	0.08082404	OK	8.64E-217
IFI44	0	48.15674859	0.1237569	OK	0
ADGRL4	0	134.6451211	0.34396075	OK	0
ADGRL2	9.08E-74	18.13096485	0.04650077	OK	3.86E-73
TTLL7	0	119.7286167	0.30782554	OK	0
AL359504.2	1.80E-34	12.18795156	0.03120685	OK	6.12E-34
PRKACB	2.56E-142	25.37212211	0.06517117	OK	1.39E-141

DNASE2B	1.57E-26	10.59506964	0.02695074	OK	4.97E-26
RPF1	3.26E-18	8.623013333	0.02209802	OK	9.31E-18
GNG5	0	171.4559742	0.44089078	OK	0
CTBS	4.73E-175	28.18295678	0.07240725	OK	2.78E-174
SSX2IP	7.36E-09	5.664763982	0.01447764	OK	1.74E-08
MCOLN2	1.22E-211	31.02551546	0.07957295	OK	7.77E-211
MCOLN3	6.93E-14	7.39759032	0.01888151	OK	1.84E-13
SYDE2	1.00E-13	7.348357155	0.01878638	OK	2.66E-13
C1orf52	3.27E-51	15.00776585	0.03852115	OK	1.24E-50
BCL10	3.16E-177	28.35990004	0.07286003	OK	1.87E-176
DDAH1	1.52E-52	15.20978674	0.03894691	OK	5.81E-52
AC092807.3	4.22E-72	17.91866041	0.04594066	OK	1.78E-71
CYR61	0	175.6378058	0.45163529	OK	0
ZNHIT6	1.45E-19	8.972452194	0.02299495	OK	4.22E-19
ODF2L	9.18E-25	10.20747815	0.02617078	OK	2.86E-24
SH3GLB1	0	71.14970336	0.18291855	OK	0
SELENOF	0	45.50521517	0.11696217	OK	0
HS2ST1	5.51E-14	7.428171528	0.01901738	OK	1.47E-13
LMO4	0	41.60803969	0.10693796	OK	0
PKN2	0.00033668	3.400203771	0.00867078	OK	0.0006602
GTF2B	0	83.64673791	0.21505531	OK	0
KYAT3	2.30E-08	5.466147605	0.013979	OK	5.36E-08
RBMXL1	6.94E-08	5.266807962	0.01346455	OK	1.59E-07
GBP3	2.34E-15	7.835248373	0.02006655	OK	6.37E-15
GBP1	4.06E-49	14.68429223	0.03768714	OK	1.53E-48
GBP2	3.51E-172	27.94777862	0.07180259	OK	2.05E-171
GBP7	5.81E-06	4.384431567	0.01114851	OK	1.24E-05
GBP4	2.74E-173	28.03867695	0.07200785	OK	1.61E-172
GBP5	1.89E-204	30.48773384	0.0782877	OK	1.19E-203
LRRC8B	1.50E-05	4.174185922	0.0106396	OK	3.14E-05
LRRC8C-DT	1.23E-05	4.218551442	0.01075465	OK	2.59E-05
LRRC8C	4.86E-51	14.98137819	0.03843293	OK	1.84E-50
LRRC8D	5.77E-18	8.557523761	0.02190933	OK	1.64E-17
ZNF326	1.12E-14	7.636456345	0.01956404	OK	3.01E-14
ZNF644	6.17E-15	7.712456821	0.01976033	OK	1.67E-14
TGFBR3	1.13E-65	17.07551834	0.0437822	OK	4.63E-65
RPAP2	8.20E-05	3.768748667	0.00961574	OK	0.00016627
EVI5	3.17E-34	12.14184812	0.03115102	OK	1.07E-33
RPL5	0	184.2693248	0.47378262	OK	0
FAM69A	1.06E-08	5.602113975	0.01430228	OK	2.49E-08
MTF2	5.31E-16	8.019439132	0.02054661	OK	1.46E-15
TMED5	0	77.13758748	0.19831146	OK	0
CCDC18	4.79E-82	19.15057863	0.04913519	OK	2.12E-81
DR1	1.03E-77	18.62381545	0.0478209	OK	4.47E-77
FNBP1L	6.20E-41	13.34655346	0.03422648	OK	2.21E-40
BCAR3	8.61E-07	4.783627623	0.01222015	OK	1.90E-06
DNTTIP2	0	39.61880893	0.10182125	OK	0
GCLM	4.51E-16	8.039643743	0.02059661	OK	1.24E-15
ARHGAP29	0	87.03089238	0.22371371	OK	0
F3	6.27E-136	24.78650398	0.06358097	OK	3.34E-135
CNN3	0	155.2212761	0.39910666	OK	0
AC105942.1	1.22E-86	19.6935939	0.05050115	OK	5.52E-86
ALG14	8.75E-10	6.0194271	0.01539972	OK	2.13E-09

TMEM56	1.42E-136	24.84631894	0.06375944	OK	7.57E-136
PTBP2	2.35E-21	9.415731505	0.02412842	OK	7.02E-21
DPYD	2.82E-133	24.53927331	0.06302838	OK	1.49E-132
SNX7	0	38.41340399	0.09869531	OK	0
PALMD	0	56.79206096	0.1455369	OK	0
FRRS1	4.65E-06	4.432676373	0.01129821	OK	9.97E-06
AGL	3.96E-13	7.162655463	0.01834151	OK	1.04E-12
SASS6	8.67E-07	4.78218781	0.01221603	OK	1.91E-06
RTCA	9.73E-73	18.0000844	0.04621395	OK	4.11E-72
GPR88	5.67E-15	7.723351844	0.01969289	OK	1.53E-14
VCAM1	0	39.49758934	0.10145103	OK	0
EXTL2	2.39E-82	19.18669249	0.04924593	OK	1.06E-81
SLC30A7	6.76E-08	5.27168903	0.01347837	OK	1.55E-07
DPH5	5.25E-07	4.882032596	0.01247237	OK	1.17E-06
AC093157.1	0.00026152	3.468670622	0.00882677	OK	0.00051613
AL109741.1	3.10E-17	8.361375531	0.02109388	OK	8.70E-17
S1PR1	3.62E-36	12.50243296	0.03201938	OK	1.24E-35
COL11A1	3.61E-09	5.785734147	0.01422766	OK	8.62E-09
RNPC3	2.26E-07	5.045486284	0.01289949	OK	5.10E-07
NTNG1	8.01E-24	9.995085571	0.02486453	OK	2.47E-23
VAV3	0	42.50232526	0.10920165	OK	0
AL390036.1	1.64E-132	24.46756508	0.06278857	OK	8.63E-132
SLC25A24	0	50.44560548	0.12966207	OK	0
FAM102B	7.88E-56	15.69736036	0.04028434	OK	3.07E-55
HENMT1	1.28E-12	6.9998831	0.01791064	OK	3.32E-12
PRPF38B	7.45E-201	30.21532926	0.0776364	OK	4.63E-200
STXBP3	8.20E-26	10.43934465	0.02677313	OK	2.58E-25
AKNAD1	1.59E-05	4.160000897	0.01039711	OK	3.34E-05
GPSM2	7.27E-13	7.078890275	0.01810466	OK	1.89E-12
WDR47	5.58E-06	4.393481029	0.01121869	OK	1.19E-05
TAF13	1.84E-13	7.266933042	0.01861066	OK	4.85E-13
TMEM167B	8.72E-20	9.028321283	0.02314251	OK	2.54E-19
SARS	8.64E-104	21.60178757	0.05548177	OK	4.17E-103
PSRC1	3.30E-11	6.529425642	0.01666787	OK	8.30E-11
SORT1	2.11E-112	22.4992612	0.05778435	OK	1.05E-111
PSMA5	0	58.34762507	0.14999125	OK	0
AMIGO1	0.00035267	3.387495277	0.00856139	OK	0.00069112
GNAI3	0	54.88997677	0.14109752	OK	0
AMPD2	4.04E-14	7.469151945	0.01912764	OK	1.08E-13
GSTM4	6.74E-66	17.10557162	0.04390366	OK	2.77E-65
GSTM2	1.63E-05	4.154033014	0.01058838	OK	3.42E-05
GSTM5	3.66E-47	14.37598111	0.03682116	OK	1.36E-46
GSTM3	0	64.050868	0.16462254	OK	0
CSF1	4.26E-74	18.17256015	0.04662456	OK	1.81E-73
AHCYL1	6.67E-18	8.540762105	0.02189028	OK	1.89E-17
SLC16A4	2.20E-151	26.18076957	0.06720992	OK	1.22E-150
LAMTOR5	0	71.81833248	0.18463871	OK	0
KCNA3	0.00011207	3.690127853	0.00934959	OK	0.00022567
CD53	0	230.1596704	0.59188189	OK	0
LRIF1	1.22E-54	15.5223373	0.03983694	OK	4.73E-54
DRAM2	0	107.9867031	0.27765486	OK	0
CEPT1	5.73E-09	5.707437034	0.01459956	OK	1.36E-08
DENND2D	8.26E-82	19.12212131	0.0490786	OK	3.65E-81

CHI3L2	2.49E-05	4.056433827	0.01031779	OK	5.17E-05
WDR77	8.96E-13	7.049833087	0.01805025	OK	2.33E-12
ATP5PB	0	68.3988332	0.17584407	OK	0
C1orf162	0	202.18306	0.51991975	OK	0
TMIGD3	2.10E-128	24.07841122	0.0617913	OK	1.10E-127
ADORA3	8.25E-99	21.06546647	0.05402918	OK	3.91E-98
RAP1A	0	67.22766664	0.17283144	OK	0
CTTNBP2NL	2.83E-135	24.72568726	0.06351233	OK	1.51E-134
WNT2B	1.17E-11	6.682946968	0.0170412	OK	2.98E-11
ST7L	3.83E-06	4.474223215	0.01142662	OK	8.25E-06
CAPZA1	0	87.12188151	0.22399699	OK	0
MOV10	0.00046789	3.309157522	0.00842175	OK	0.00090898
AL603832.1	2.36E-05	4.069081493	0.00824312	OK	4.91E-05
RHOC	0	76.05577404	0.1955369	OK	0
PPM1J	1.07E-89	20.04717738	0.05138348	OK	4.88E-89
FAM19A3	1.72E-16	8.157146728	0.02030354	OK	4.77E-16
LINC01356	0.00038493	3.363407571	0.00836197	OK	0.00075254
AL390729.1	8.91E-10	6.016582421	0.01531316	OK	2.16E-09
SLC16A1	6.93E-05	3.810766342	0.00972028	OK	0.00014089
MAGI3	5.42E-12	6.794817577	0.017367	OK	1.39E-11
PHTF1	9.46E-20	9.0193191	0.02310655	OK	2.76E-19
RSBN1	1.10E-20	9.251843696	0.02371731	OK	3.27E-20
PTPN22	6.36E-281	35.79606809	0.09193699	OK	4.47E-280
DCLRE1B	1.30E-17	8.463089043	0.02166643	OK	3.68E-17
HIPK1	7.64E-09	5.658324307	0.01447607	OK	1.81E-08
OLFML3	4.02E-92	20.32303547	0.0521632	OK	1.86E-91
SYT6	2.91E-05	4.019873449	0.01009583	OK	6.02E-05
TRIM33	9.94E-07	4.754720744	0.01215234	OK	2.19E-06
BCAS2	0	40.1385884	0.10315682	OK	0
DENND2C	2.16E-07	5.054222093	0.01287876	OK	4.88E-07
NRAS	3.45E-137	24.90296942	0.0639578	OK	1.85E-136
CSDE1	0	47.42232183	0.12189298	OK	0
SIKE1	1.38E-16	8.183741293	0.02096969	OK	3.83E-16
TSPAN2	0	92.75647756	0.23843015	OK	0
NGF	2.95E-48	14.54925456	0.03723377	OK	1.10E-47
VANGL1	5.50E-05	3.867322675	0.00985198	OK	0.00011241
CASQ2	0	119.0274022	0.30583958	OK	0
SLC22A15	0.00028096	3.449363826	0.00876613	OK	0.00055324
ATP1A1	0	38.69380144	0.09944081	OK	0
CD58	4.92E-270	35.08923894	0.09016787	OK	3.42E-269
CD2	0	56.96971374	0.14634715	OK	0
PTGFRN	1.01E-08	5.609477194	0.01432599	OK	2.39E-08
CD101	1.04E-32	11.85305927	0.03026604	OK	3.48E-32
TTF2	2.46E-39	13.06947425	0.03352044	OK	8.65E-39
MAN1A2	8.01E-75	18.2639858	0.04689435	OK	3.42E-74
TENT5C	6.02E-81	19.01830716	0.04879944	OK	2.65E-80
GDAP2	0.00017198	3.579715504	0.00912855	OK	0.00034289
WDR3	7.06E-06	4.341850343	0.01108674	OK	1.50E-05
SPAG17	2.50E-11	6.571005769	0.01562821	OK	6.29E-11
TBX15	2.94E-07	4.99506122	0.01269153	OK	6.61E-07
WARS2	1.18E-12	7.010946163	0.01794469	OK	3.07E-12
PHGDH	0	76.4308407	0.19644003	OK	0
NOTCH2	5.81E-14	7.420943434	0.01900763	OK	1.55E-13

SEC22B	1.15E-36	12.59345826	0.03231288	OK	3.95E-36
LINC00623	7.89E-25	10.22217822	0.02620953	OK	2.46E-24
FCGR1B	0	158.7976948	0.40828715	OK	0
FAM72B	1.03E-11	6.702120366	0.0168868	OK	2.61E-11
SRGAP2C	5.40E-16	8.01736211	0.02053927	OK	1.49E-15
AC245595.1	1.19E-25	10.40404104	0.02666399	OK	3.73E-25
NBPF15	0.00043457	3.329780069	0.00844788	OK	0.00084688
SRGAP2B	8.27E-55	15.54749292	0.03989765	OK	3.20E-54
AC245014.3	4.45E-136	24.80026524	0.06366445	OK	2.38E-135
RNF115	2.22E-34	12.17081774	0.03122631	OK	7.54E-34
NUDT17	6.18E-07	4.849711776	0.01236149	OK	1.37E-06
PIAS3	8.07E-05	3.772845675	0.00961994	OK	0.00016362
ANKRD35	0	44.15150241	0.11342796	OK	0
ITGA10	0	51.96762736	0.1335347	OK	0
RBM8A	0	89.70582106	0.23064416	OK	0
LIX1L-AS1	8.49E-09	5.640268432	0.01439141	OK	2.00E-08
LIX1L	3.14E-81	19.05231863	0.04891748	OK	1.39E-80
POLR3GL	4.32E-154	26.41744827	0.06786722	OK	2.41E-153
TXNIP	0	84.18980013	0.2164554	OK	0
PRKAB2	6.28E-13	7.099154209	0.01817094	OK	1.64E-12
CHD1L	1.69E-07	5.10041237	0.01303746	OK	3.84E-07
BCL9	5.49E-06	4.396770671	0.01120206	OK	1.17E-05
ACP6	1.66E-11	6.631641725	0.01694911	OK	4.20E-11
GJA5	2.47E-61	16.4819099	0.04223211	OK	9.95E-61
PDE4DIP	1.10E-21	9.494871608	0.02434337	OK	3.31E-21
NBPF9	6.10E-14	7.414599995	0.0189429	OK	1.62E-13
AC245297.3	1.38E-07	5.139159339	0.01314082	OK	3.14E-07
FCGR1A	0	226.8041469	0.58323091	OK	0
HIST2H2BF	1.24E-05	4.216296905	0.01074114	OK	2.62E-05
AC243772.2	3.23E-05	3.995155102	0.01011865	OK	6.68E-05
HIST2H4B	1.60E-08	5.530481364	0.01357238	OK	3.74E-08
HIST2H2AC	1.16E-13	7.329408142	0.01877076	OK	3.06E-13
BOLA1	5.02E-36	12.47634953	0.03199555	OK	1.72E-35
SV2A	1.25E-30	11.4446378	0.02929819	OK	4.10E-30
SF3B4	7.83E-67	17.2305462	0.04423908	OK	3.23E-66
MTMR11	3.34E-38	12.86942943	0.03299544	OK	1.17E-37
OTUD7B	1.61E-40	13.27528552	0.03401878	OK	5.71E-40
PLEKHO1	0	102.4188428	0.26334002	OK	0
ANP32E	7.02E-231	32.42066174	0.0833074	OK	4.62E-230
APH1A	0	46.66708052	0.11994747	OK	0
C1orf54	0	58.42233557	0.15017931	OK	0
CIART	8.10E-12	6.736690471	0.01721657	OK	2.07E-11
MRPS21	0	50.09127758	0.12875757	OK	0
RPRD2	1.38E-17	8.456536668	0.02166734	OK	3.89E-17
ECM1	0	39.36837456	0.10110369	OK	0
ADAMTSL4	4.64E-38	12.84417203	0.0329358	OK	1.61E-37
MCL1	0	133.9513897	0.34444036	OK	0
ENSA	0	48.91267735	0.12572627	OK	0
GOLPH3L	6.47E-12	6.769242891	0.01732119	OK	1.66E-11
CTSS	0	317.2751159	0.81594332	OK	0
CTSK	0	54.1005007	0.13901019	OK	0
CERS2	2.36E-28	10.98110394	0.02816539	OK	7.59E-28
C1orf56	7.49E-167	27.50574887	0.0706631	OK	4.32E-166

CDC42SE1	0	53.22311676	0.13680796	OK	0
MLLT11	2.16E-07	5.054141376	0.01291185	OK	4.88E-07
TNFAIP8L2	0	62.72989673	0.1612319	OK	0
SCNM1	3.79E-238	32.93231288	0.08461886	OK	2.52E-237
LYSMD1	2.17E-05	4.088611503	0.01040583	OK	4.52E-05
VPS72	1.69E-27	10.80190154	0.02770152	OK	5.39E-27
PSMD4	0	42.4547258	0.10911656	OK	0
PI4KB	3.68E-09	5.782335927	0.01479132	OK	8.79E-09
RFX5	5.23E-17	8.299427071	0.02125814	OK	1.46E-16
AL391069.3	4.10E-98	20.98940294	0.05383118	OK	1.94E-97
SELENBP1	0	86.45587622	0.2222289	OK	0
PSMB4	0	46.94858381	0.1206743	OK	0
POGZ	3.76E-05	3.959492709	0.01010691	OK	7.73E-05
SNX27	1.09E-38	12.9556545	0.03324051	OK	3.82E-38
MRPL9	9.72E-85	19.47072574	0.04999796	OK	4.36E-84
OAZ3	4.21E-07	4.925400069	0.01253065	OK	9.40E-07
TDRKH	1.17E-07	5.170435901	0.01318554	OK	2.66E-07
RORC	0.00020219	3.537212156	0.00832416	OK	0.00040151
THEM4	6.00E-11	6.439252935	0.01647515	OK	1.50E-10
S100A10	0	215.1628529	0.55329793	OK	0
S100A11	0	226.7886941	0.58316273	OK	0
TCHH	1.58E-31	11.62272766	0.02975013	OK	5.23E-31
LOR	6.04E-154	26.40475885	0.06483085	OK	3.37E-153
S100A9	0	151.8645516	0.39049222	OK	0
S100A12	0	58.01913638	0.1487455	OK	0
S100A8	0	98.88513486	0.25417565	OK	0
S100A6	0	213.7793297	0.54969739	OK	0
S100A4	0	137.916206	0.35461099	OK	0
S100A3	1.01E-90	20.16405624	0.0516662	OK	4.65E-90
S100A2	4.23E-10	6.136201436	0.01567303	OK	1.03E-09
S100A16	0	73.27859096	0.18833922	OK	0
S100A13	0	87.7689565	0.22565023	OK	0
CHTOP	1.70E-13	7.277842423	0.01864127	OK	4.47E-13
SNAPIN	3.19E-117	22.98635811	0.05904198	OK	1.61E-116
ILF2	0	46.87610771	0.12048719	OK	0
NPR1	0	39.78059587	0.10215889	OK	0
INTS3	5.78E-10	6.086139465	0.01556753	OK	1.41E-09
SLC27A3	1.27E-14	7.620313026	0.01952009	OK	3.40E-14
DENND4B	4.75E-23	9.817150093	0.02515837	OK	1.45E-22
SLC39A1	1.66E-283	35.96168112	0.09241534	OK	1.18E-282
JTB	0	62.23663453	0.15999498	OK	0
RAB13	0	80.06155845	0.20583938	OK	0
RPS27	0	158.8844843	0.40849136	OK	0
TPM3	0	247.3886736	0.63619711	OK	0
C1orf43	0	56.67967557	0.14570277	OK	0
UBAP2L	5.05E-09	5.72900347	0.0146563	OK	1.20E-08
HAX1	1.80E-252	33.91806458	0.08715973	OK	1.22E-251
AQP10	1.42E-08	5.551291713	0.01293862	OK	3.33E-08
ATP8B2	3.32E-26	10.52479125	0.02695734	OK	1.05E-25
IL6R	0	49.60146818	0.12746754	OK	0
SHE	9.45E-81	18.99461315	0.04855122	OK	4.16E-80
UBE2Q1	5.68E-28	10.9012835	0.02795879	OK	1.82E-27
ADAR	1.17E-17	8.475793651	0.0217231	OK	3.30E-17

KCNN3	6.80E-152	26.22552267	0.06620863	OK	3.78E-151
PMVK	3.93E-219	31.57610979	0.08113466	OK	2.53E-218
PBXIP1	0	45.00477429	0.11565813	OK	0
AL451085.2	3.85E-06	4.47355483	0.01140751	OK	8.27E-06
SHC1	1.04E-07	5.19170912	0.01327171	OK	2.38E-07
CKS1B	0	43.03288768	0.1105826	OK	0
FLAD1	1.78E-10	6.272560987	0.01605142	OK	4.39E-10
ZBTB7B	5.17E-15	7.734958046	0.01980658	OK	1.40E-14
ADAM15	3.32E-83	19.2890991	0.04951953	OK	1.48E-82
EFNA1	5.07E-98	20.97928513	0.05373746	OK	2.39E-97
SLC50A1	1.97E-101	21.34945501	0.05482623	OK	9.43E-101
DPM3	3.15E-103	21.54195412	0.05532813	OK	1.52E-102
KRTCAP2	0	43.34237319	0.11139969	OK	0
THBS3	3.18E-10	6.181229277	0.01580255	OK	7.80E-10
MTX1	4.24E-147	25.80175712	0.06628036	OK	2.32E-146
GBA	3.47E-58	16.03800657	0.04116095	OK	1.37E-57
SCAMP3	5.64E-16	8.012161882	0.0205275	OK	1.55E-15
FDPS	4.99E-139	25.07228875	0.06440755	OK	2.69E-138
ASH1L	6.05E-21	9.315835788	0.02388425	OK	1.80E-20
DAP3	1.08E-130	24.29604273	0.06240779	OK	5.66E-130
SYT11	7.84E-22	9.530315294	0.02443133	OK	2.36E-21
RIT1	0	65.1465261	0.16747213	OK	0
ARHGEF2	5.44E-21	9.32701419	0.02391042	OK	1.62E-20
SSR2	0	91.28959344	0.23471653	OK	0
LAMTOR2	0	106.4416588	0.27368745	OK	0
LMNA	0	47.3315095	0.12165829	OK	0
SEMA4A	1.87E-83	19.31871573	0.04958714	OK	8.33E-83
SLC25A44	7.11E-08	5.26232032	0.01344308	OK	1.63E-07
PMF1	6.46E-59	16.14207734	0.04143856	OK	2.56E-58
GLMP	1.16E-194	29.74022438	0.07640683	OK	7.12E-194
CCT3	0	45.76968547	0.11764228	OK	0
IQGAP3	3.64E-133	24.52883247	0.06252284	OK	1.92E-132
NAXE	1.29E-135	24.75753283	0.06359758	OK	6.85E-135
GPATCH4	1.48E-28	11.02282317	0.02826916	OK	4.79E-28
AL365181.4	7.24E-05	3.799830969	0.00891734	OK	0.00014713
HAPLN2	4.28E-58	16.02492277	0.03951858	OK	1.69E-57
NES	4.56E-140	25.16735249	0.06458315	OK	2.46E-139
CRABP2	1.03E-26	10.6345936	0.02712071	OK	3.26E-26
ISG20L2	5.39E-37	12.65292577	0.03246248	OK	1.86E-36
RRNAD1	0.00042062	3.338855532	0.00850683	OK	0.00082056
MRPL24	5.00E-79	18.7851324	0.04823479	OK	2.19E-78
HDGF	2.37E-131	24.35830015	0.06257164	OK	1.24E-130
PRCC	6.35E-10	6.071084452	0.01553628	OK	1.55E-09
SH2D2A	2.86E-17	8.370768226	0.02136855	OK	8.04E-17
PEAR1	1.75E-25	10.36691719	0.02653025	OK	5.50E-25
ARHGEF11	0.00013633	3.639973979	0.00927974	OK	0.0002733
ETV3	2.94E-106	21.86270666	0.05614077	OK	1.43E-105
FCRL1	3.18E-07	4.979920596	0.01149009	OK	7.14E-07
CD5L	0.00029984	3.431757673	0.00837586	OK	0.00058938
KIRREL1	8.01E-45	13.99805844	0.03587393	OK	2.93E-44
LINC01704	7.11E-31	11.49351898	0.02390879	OK	2.34E-30
CD1D	0	71.27745115	0.18309663	OK	0
AL138899.1	0	51.57756574	0.13198261	OK	0

CD1A	5.97E-82	19.1390896	0.04857727	OK	2.64E-81
CD1C	0	239.1388285	0.61425691	OK	0
CD1B	0	38.11882414	0.09713795	OK	0
CD1E	0	154.2376323	0.39579366	OK	0
MNDA	0	253.4829587	0.65186341	OK	0
PYHIN1	3.82E-23	9.839061177	0.02512462	OK	1.17E-22
IFI16	0	94.13472726	0.24203454	OK	0
AIM2	1.22E-117	23.02793564	0.05903121	OK	6.17E-117
ACKR1	0	50.00728818	0.12792608	OK	0
FCER1A	0	253.4598577	0.65142233	OK	0
DUSP23	0	54.90330513	0.14112967	OK	0
FCRL6	1.57E-41	13.44861383	0.03397415	OK	5.62E-41
SLAMF8	0	119.6178741	0.30754502	OK	0
TAGLN2	0	114.1895283	0.29360809	OK	0
SLAMF9	0	198.1441691	0.50940881	OK	0
LINC01133	3.58E-98	20.99587957	0.05342536	OK	1.69E-97
IGSF8	7.90E-102	21.39216243	0.05493016	OK	3.78E-101
ATP1A2	0	49.09428203	0.12612079	OK	0
PEA15	0	51.1442923	0.13146465	OK	0
PEX19	1.14E-59	16.2485142	0.04170318	OK	4.56E-59
COPA	3.44E-17	8.349086546	0.02139764	OK	9.66E-17
NCSTN	2.79E-26	10.54133086	0.02703037	OK	8.79E-26
SLAMF6	1.18E-06	4.719484185	0.01201792	OK	2.59E-06
CD84	0	164.3669838	0.42265008	OK	0
SLAMF1	1.03E-13	7.344250283	0.01873773	OK	2.74E-13
AL121985.1	6.27E-09	5.692221686	0.01393015	OK	1.48E-08
CD48	0	121.259023	0.3117774	OK	0
SLAMF7	0	40.27177034	0.10338777	OK	0
LY9	1.37E-17	8.457326165	0.02163566	OK	3.87E-17
CD244	2.33E-16	8.120086971	0.02069075	OK	6.46E-16
ITLN1	6.35E-09	5.690131031	0.01453847	OK	1.50E-08
ITLN2	7.54E-07	4.810182427	0.01215675	OK	1.67E-06
F11R	2.55E-27	10.76373944	0.02758374	OK	8.14E-27
TSTD1	0	49.27512514	0.12664905	OK	0
ARHGAP30	0	72.18786503	0.1855656	OK	0
NECTIN4	1.21E-08	5.578761543	0.01416868	OK	2.85E-08
KLHDC9	7.91E-40	13.15548136	0.03368292	OK	2.79E-39
PFDN2	0	75.57848563	0.19430963	OK	0
NIT1	1.98E-11	6.605448829	0.01691146	OK	5.00E-11
DEDD	1.47E-06	4.675028065	0.01194495	OK	3.21E-06
UFC1	0	65.80819298	0.16918084	OK	0
PPOX	1.91E-05	4.117654203	0.01050641	OK	4.00E-05
B4GALT3	1.97E-09	5.886623836	0.01506022	OK	4.73E-09
ADAMTS4	0	41.75000816	0.10685431	OK	0
NDUFS2	1.05E-224	31.97939229	0.08217167	OK	6.87E-224
FCER1G	0	348.5318332	0.8963384	OK	0
PCP4L1	7.80E-223	31.84467219	0.08121453	OK	5.06E-222
SDHC	2.06E-254	34.04952152	0.08749821	OK	1.40E-253
FCGR2A	0	256.1801207	0.65879948	OK	0
HSPA6	0	38.16493647	0.09803638	OK	0
FCGR3A	0	281.7791388	0.72464065	OK	0
FCGR2B	0	152.5654116	0.39228434	OK	0
FCRLB	1.63E-08	5.526691264	0.01411573	OK	3.82E-08

DUSP12	2.24E-17	8.399502546	0.02152329	OK	6.31E-17
ATF6	3.33E-234	32.65567279	0.08390544	OK	2.20E-233
OLFML2B	8.47E-201	30.21108241	0.07762067	OK	5.26E-200
NOS1AP	2.67E-10	6.208617761	0.01585074	OK	6.57E-10
C1orf226	6.11E-09	5.696521822	0.01432651	OK	1.45E-08
UHMK1	3.17E-27	10.74378907	0.02755348	OK	1.01E-26
UAP1	2.06E-22	9.668250634	0.02478153	OK	6.24E-22
DDR2	0	51.33005854	0.13189457	OK	0
HSD17B7	0.00031418	3.419071048	0.00871299	OK	0.00061695
RGS4	9.35E-220	31.62148221	0.08118868	OK	6.04E-219
RGS5	0	264.1619446	0.6793199	OK	0
NUF2	0	131.9323049	0.33774191	OK	0
PBX1	0	45.36062182	0.11654698	OK	0
MGST3	0	175.888766	0.45228641	OK	0
ALDH9A1	5.78E-56	15.71700321	0.04034516	OK	2.25E-55
TMCO1	0	39.77893208	0.10223452	OK	0
UCK2	2.76E-22	9.638121175	0.02468865	OK	8.36E-22
DUSP27	4.77E-05	3.901907841	0.00985693	OK	9.77E-05
CD247	1.03E-125	23.82045058	0.06106263	OK	5.29E-125
CREG1	0	118.4318428	0.30452478	OK	0
RCSD1	0	57.06883789	0.14668187	OK	0
MPZL1	8.21E-23	9.761769571	0.02502685	OK	2.51E-22
MPC2	0	49.15405567	0.12634646	OK	0
GPR161	1.15E-09	5.974518342	0.01526428	OK	2.79E-09
TIPRL	3.27E-121	23.38187558	0.0600599	OK	1.66E-120
SFT2D2	2.50E-28	10.97568675	0.02815067	OK	8.06E-28
XCL2	7.38E-21	9.294704474	0.02369898	OK	2.19E-20
XCL1	4.06E-15	7.76561905	0.01981465	OK	1.10E-14
DPT	0	80.35421877	0.20648645	OK	0
ATP1B1	0	70.39661959	0.18097564	OK	0
NME7	6.77E-24	10.01170386	0.0256632	OK	2.09E-23
BLZF1	1.42E-16	8.179771499	0.02095774	OK	3.96E-16
CCDC181	3.96E-06	4.467563305	0.01134329	OK	8.50E-06
SLC19A2	3.31E-16	8.077478596	0.02067887	OK	9.15E-16
F5	4.92E-132	24.42262495	0.06250227	OK	2.59E-131
SELP	0	39.81533223	0.10121885	OK	0
SELL	0	48.06083864	0.12344202	OK	0
SELE	2.89E-78	18.69178279	0.04696583	OK	1.26E-77
METTL18	3.15E-13	7.193706845	0.01840559	OK	8.28E-13
KIFAP3	7.52E-52	15.10486023	0.03876847	OK	2.86E-51
GORAB	8.50E-11	6.386332573	0.01633999	OK	2.11E-10
PRRX1	0	126.3937447	0.32496516	OK	0
Z97200.1	3.69E-71	17.79752706	0.0455119	OK	1.55E-70
FMO3	6.32E-41	13.34517574	0.03405927	OK	2.25E-40
FMO2	0	90.12589154	0.23167843	OK	0
FMO1	0.000118	3.676988778	0.00896302	OK	0.00023733
PRRC2C	1.22E-48	14.6096095	0.03750074	OK	4.57E-48
MYOC	2.26E-33	11.98003855	0.03058057	OK	7.62E-33
VAMP4	1.08E-16	8.212518605	0.02104223	OK	3.02E-16
METTL13	4.31E-05	3.926615528	0.01001538	OK	8.84E-05
DNM3OS	7.92E-59	16.1294753	0.04135116	OK	3.14E-58
PIGC	2.38E-18	8.659162618	0.02219298	OK	6.80E-18
SUCO	6.82E-15	7.699681058	0.01972466	OK	1.85E-14

TNFSF18	1.93E-32	11.80077432	0.03011709	OK	6.46E-32
TNFSF4	5.49E-06	4.396770263	0.01115083	OK	1.17E-05
PRDX6	0	85.65301793	0.22021991	OK	0
KLHL20	8.87E-05	3.749130128	0.00956283	OK	0.00017953
CENPL	1.38E-07	5.13895411	0.01309982	OK	3.14E-07
DARS2	1.67E-06	4.648556252	0.01185932	OK	3.65E-06
RABGAP1L	1.86E-19	8.944918048	0.02292605	OK	5.41E-19
CACYBP	0	52.71833834	0.13551401	OK	0
MRPS14	2.09E-72	17.95773235	0.04610597	OK	8.82E-72
TNN	5.53E-10	6.09316883	0.01429863	OK	1.35E-09
KIAA0040	2.05E-62	16.63189362	0.04269422	OK	8.27E-62
COP1	1.45E-26	10.6024124	0.02718474	OK	4.60E-26
RASAL2	3.87E-42	13.55169088	0.03476889	OK	1.39E-41
RALGPS2	5.11E-24	10.03943113	0.02572278	OK	1.58E-23
ANGPTL1	0	75.08458481	0.19293786	OK	0
FAM20B	1.41E-11	6.655484213	0.01703085	OK	3.58E-11
TOR3A	6.06E-198	29.99299482	0.07705565	OK	3.74E-197
ABL2	3.05E-217	31.43808853	0.08076449	OK	1.96E-216
SOAT1	0	41.39710489	0.10638084	OK	0
TOR1AIP2	7.78E-83	19.24492334	0.04941962	OK	3.46E-82
TOR1AIP1	1.22E-18	8.734626546	0.02238722	OK	3.51E-18
CEP350	1.03E-14	7.646583317	0.01959076	OK	2.78E-14
QSOX1	1.38E-45	14.12252749	0.03624147	OK	5.06E-45
ACBD6	2.78E-21	9.398187961	0.02409562	OK	8.29E-21
STX6	0	40.57657574	0.10427374	OK	0
MR1	7.37E-43	13.67290764	0.03508388	OK	2.66E-42
IER5	0	84.12021506	0.21626902	OK	0
GLUL	0	207.2129128	0.53286474	OK	0
RNASEL	0.00040991	3.346015628	0.00852576	OK	0.00080002
RGS16	0	72.60937328	0.18660534	OK	0
NPL	0	183.9299539	0.47296015	OK	0
DHX9	9.61E-41	13.31386493	0.03416508	OK	3.42E-40
LAMC1	1.95E-182	28.77917805	0.07391435	OK	1.16E-181
NMNAT2	0	54.61355002	0.13967982	OK	0
SMG7	4.79E-07	4.900234202	0.01252364	OK	1.07E-06
NCF2	0	140.977555	0.36249484	OK	0
ARPC5	0	153.9194459	0.39578439	OK	0
RGL1	0	55.30896262	0.14214681	OK	0
COLGALT2	7.83E-19	8.784855869	0.02216289	OK	2.25E-18
TSEN15	5.23E-28	10.90883353	0.02797509	OK	1.68E-27
C1orf21	5.21E-184	28.90471458	0.07423344	OK	3.12E-183
EDEM3	6.68E-08	5.273841519	0.01348512	OK	1.53E-07
FAM129A	0	55.09915728	0.14163558	OK	0
RNF2	1.21E-29	11.24582579	0.02883934	OK	3.95E-29
TRMT1L	6.42E-07	4.842236212	0.01237185	OK	1.42E-06
IVNS1ABP	0	52.06368755	0.13381628	OK	0
HMCN1	0	80.33758885	0.20647935	OK	0
PRG4	1.35E-16	8.185882864	0.02024058	OK	3.76E-16
TPR	4.45E-67	17.26316519	0.04432555	OK	1.84E-66
PTGS2	3.56E-306	37.3839849	0.09603621	OK	2.59E-305
PLA2G4A	7.70E-14	7.383694295	0.01889581	OK	2.04E-13
BRINP3	4.14E-11	6.495477717	0.01526747	OK	1.04E-10
RGS18	0	68.69861523	0.17654967	OK	0

AL390957.1	9.38E-07	4.766311208	0.01188239	OK	2.07E-06
AL136987.1	5.77E-38	12.82729315	0.03285495	OK	2.01E-37
RGS1	0	181.2005647	0.46594911	OK	0
RGS13	1.46E-07	5.128191069	0.01304879	OK	3.32E-07
RGS2	0	193.1812293	0.49677043	OK	0
UCHL5	8.48E-50	14.79010256	0.03795996	OK	3.20E-49
TROVE2	4.45E-72	17.91561879	0.04600196	OK	1.88E-71
GLRX2	3.24E-201	30.24287177	0.07770132	OK	2.01E-200
CDC73	1.53E-62	16.64924129	0.04274439	OK	6.20E-62
KCNT2	9.48E-175	28.15830787	0.07214951	OK	5.57E-174
CFH	0	126.0677735	0.32407606	OK	0
ASPM	0	210.7506083	0.53962694	OK	0
DENND1B	1.07E-83	19.34728457	0.04966816	OK	4.79E-83
C1orf53	7.35E-12	6.75073467	0.01725387	OK	1.88E-11
NEK7	3.47E-28	10.94619198	0.02807278	OK	1.11E-27
PTPRC	0	277.064203	0.71252167	OK	0
MIR181A1HG	0	80.38258777	0.20662156	OK	0
NR5A2	4.46E-91	20.20456899	0.05002804	OK	2.05E-90
ZNF281	5.49E-29	11.11197338	0.02849582	OK	1.78E-28
KIF14	0	65.61683281	0.16654139	OK	0
DDX59	6.70E-05	3.818884066	0.0097446	OK	0.00013638
CAMSAP2	6.02E-22	9.5577513	0.02449955	OK	1.82E-21
TMEM9	3.67E-191	29.46831873	0.07570698	OK	2.23E-190
TNNT2	8.33E-18	8.515009537	0.01871064	OK	2.36E-17
LAD1	0	39.17916381	0.09878986	OK	0
PHLDA3	0	77.62227596	0.19952526	OK	0
CSRP1	0	217.6396375	0.55967678	OK	0
NAV1	3.53E-28	10.94457069	0.02806284	OK	1.13E-27
SHISA4	0	51.81549971	0.13316124	OK	0
LMOD1	0	259.638668	0.66768633	OK	0
TIMM17A	0	39.12190933	0.10053799	OK	0
RNPEP	0	38.44424462	0.09879086	OK	0
GPR37L1	6.86E-74	18.14636724	0.03743957	OK	2.92E-73
ARL8A	5.13E-300	37.00294371	0.09509099	OK	3.71E-299
PTPN7	3.50E-210	30.91725873	0.07940712	OK	2.22E-209
LGR6	4.59E-139	25.07556692	0.06433754	OK	2.47E-138
UBE2T	0	72.66613858	0.18650698	OK	0
PPP1R12B	0	144.5916288	0.37178109	OK	0
KDM5B	7.38E-09	5.66431536	0.01448875	OK	1.75E-08
RABIF	2.95E-111	22.38200032	0.05747783	OK	1.46E-110
ADIPOR1	0	49.07110155	0.12612824	OK	0
CYB5R1	1.72E-125	23.79873003	0.06112965	OK	8.87E-125
TMEM183A	1.47E-66	17.19409972	0.04414406	OK	6.06E-66
CHI3L1	0	80.94624781	0.20763151	OK	0
CHIT1	1.33E-08	5.562334008	0.01411757	OK	3.13E-08
LINC01353	1.05E-06	4.743432574	0.01207667	OK	2.31E-06
LINC01136	1.10E-08	5.594764048	0.01422395	OK	2.60E-08
BTG2	0	84.72401273	0.21782933	OK	0
FMOD	0	59.03146847	0.15168421	OK	0
PRELP	0	181.9553678	0.46786936	OK	0
ATP2B4	1.61E-121	23.41220025	0.06012964	OK	8.18E-121
SNRPE	0	56.961682	0.1464267	OK	0
SOX13	2.18E-09	5.869615459	0.01497424	OK	5.24E-09

ETNK2	2.80E-16	8.097699214	0.02069245	OK	7.76E-16
PLEKHA6	7.58E-07	4.809156307	0.01199103	OK	1.68E-06
PPP1R15B	0	39.02039793	0.1002745	OK	0
NFASC	3.38E-62	16.6018275	0.04253195	OK	1.36E-61
DSTYK	0.00016174	3.595736215	0.00916802	OK	0.00032302
NUAK2	1.25E-15	7.913464267	0.02023889	OK	3.42E-15
CDK18	1.48E-09	5.933665119	0.01516612	OK	3.57E-09
NUCKS1	0	109.1145241	0.28055639	OK	0
RAB29	6.67E-31	11.49898155	0.02949394	OK	2.20E-30
SLC41A1	1.18E-09	5.971274315	0.01526726	OK	2.85E-09
RAB7B	0	51.86904951	0.13327814	OK	0
RHEX	1.31E-125	23.81022619	0.05949317	OK	6.75E-125
FAM72A	1.22E-26	10.61834231	0.02720217	OK	3.88E-26
SRGAP2	6.12E-185	28.97858817	0.07444232	OK	3.67E-184
IKBKE	1.27E-18	8.730340871	0.02234689	OK	3.64E-18
RASSF5	0	60.86134107	0.15642115	OK	0
EIF2D	3.54E-39	13.04166152	0.03345918	OK	1.24E-38
MAPKAPK2	1.13E-41	13.47263025	0.03457122	OK	4.06E-41
IL10	0	49.80079319	0.12795172	OK	0
FCMR	2.87E-34	12.14994661	0.03112028	OK	9.73E-34
PIGR	2.57E-07	5.021082015	0.01221423	OK	5.79E-07
PFKFB2	1.26E-09	5.960677623	0.01523067	OK	3.03E-09
C4BPB	2.36E-24	10.11561963	0.02539967	OK	7.31E-24
CD55	0	75.86882263	0.19504743	OK	0
CR1	0	63.81201526	0.16396944	OK	0
CD46	3.51E-49	14.69429306	0.03771848	OK	1.32E-48
MIR29B2CHG	9.69E-07	4.759692637	0.01216028	OK	2.13E-06
CD34	0	43.2528445	0.11103898	OK	0
PLXNA2	1.13E-06	4.728302293	0.01207525	OK	2.49E-06
LAMB3	0.00037227	3.372626787	0.00854783	OK	0.00072844
AL031316.1	2.04E-19	8.934738927	0.02234262	OK	5.93E-19
G0S2	0	70.24271121	0.18051928	OK	0
HSD11B1	8.96E-36	12.43018326	0.03160404	OK	3.07E-35
TRAF3IP3	2.53E-231	32.4520924	0.08335959	OK	1.67E-230
IRF6	0.00014101	3.631286105	0.00919917	OK	0.00028241
SERTAD4-AS1	0	66.0145779	0.16964842	OK	0
SERTAD4	3.32E-59	16.18307116	0.04147848	OK	1.32E-58
RCOR3	2.62E-10	6.211764036	0.01589818	OK	6.44E-10
TRAF5	1.48E-22	9.701707945	0.02486893	OK	4.51E-22
LINC00467	4.58E-12	6.819072389	0.01744957	OK	1.18E-11
SLC30A1	5.86E-83	19.25960313	0.04944381	OK	2.61E-82
NEK2	0	71.01692972	0.1790171	OK	0
LPGAT1	6.00E-33	11.89878779	0.03052503	OK	2.01E-32
DTL	0	51.42292701	0.13105936	OK	0
PPP2R5A	2.75E-59	16.19476379	0.04156908	OK	1.09E-58
TMEM206	7.70E-98	20.95937554	0.05381085	OK	3.64E-97
NENF	0	84.59408022	0.217496	OK	0
LINC01740	2.38E-05	4.067153338	0.01015487	OK	4.94E-05
AC092803.2	3.76E-90	20.09903921	0.05159053	OK	1.72E-89
ATF3	0	88.55540026	0.2276843	OK	0
BATF3	0	44.6778731	0.11478611	OK	0
NSL1	3.10E-136	24.81478219	0.06374437	OK	1.66E-135
TATDN3	2.02E-75	18.33891171	0.04707888	OK	8.68E-75

FLVCR1-DT	0.00026036	3.469861449	0.00883675	OK	0.00051398
RPS6KC1	0.00049796	3.291674936	0.00838704	OK	0.00096588
PROX1	7.41E-46	14.16629485	0.03620999	OK	2.72E-45
SMYD2	6.18E-26	10.4660731	0.02683076	OK	1.95E-25
PTPN14	1.31E-58	16.09847202	0.04128032	OK	5.18E-58
CENPF	0	153.3182175	0.39356521	OK	0
RRP15	2.77E-24	10.09980261	0.02589858	OK	8.58E-24
TGFB2	3.09E-48	14.54613473	0.03730015	OK	1.15E-47
LYPLAL1	6.35E-69	17.50687594	0.0449476	OK	2.64E-68
EPRS	1.11E-75	18.37158651	0.04717399	OK	4.76E-75
IARS2	3.31E-30	11.35981595	0.02913916	OK	1.08E-29
MARK1	4.79E-41	13.36580683	0.03421667	OK	1.71E-40
C1orf115	2.83E-21	9.396180812	0.02401347	OK	8.45E-21
MARCH2	2.34E-76	18.45591307	0.04734192	OK	1.01E-75
MARCH1	4.23E-12	6.830423826	0.01744703	OK	1.09E-11
HLX	1.04E-206	30.65765649	0.07874751	OK	6.58E-206
DUSP10	4.67E-263	34.62875282	0.0889621	OK	3.21E-262
LINC02257	9.44E-15	7.658073197	0.01815458	OK	2.55E-14
LINC01705	9.82E-201	30.20618442	0.07737915	OK	6.10E-200
MIA3	1.36E-24	10.1692182	0.02607927	OK	4.23E-24
AIDA	1.49E-47	14.43827684	0.03705647	OK	5.53E-47
BROX	6.62E-112	22.44846565	0.05765881	OK	3.28E-111
FAM177B	5.32E-57	15.86745637	0.0405176	OK	2.09E-56
AL392172.1	4.29E-141	25.26098332	0.06487048	OK	2.32E-140
TLR5	0	40.21703796	0.10332728	OK	0
CAPN2	3.81E-186	29.07416042	0.07469928	OK	2.29E-185
TP53BP2	4.25E-30	11.33800609	0.02908223	OK	1.39E-29
DEGS1	1.72E-279	35.70385323	0.09175296	OK	1.21E-278
CNIH4	0	49.75954974	0.12790073	OK	0
WDR26	2.09E-51	15.03721948	0.03859087	OK	7.96E-51
LBR	0	42.74881155	0.10986405	OK	0
ENAH	0	102.8294612	0.26436009	OK	0
SRP9	0	58.59792698	0.15063609	OK	0
EPHX1	0	49.62396677	0.12754141	OK	0
PYCR2	3.99E-24	10.06379598	0.02580482	OK	1.24E-23
LEFTY2	2.78E-58	16.05177516	0.04111888	OK	1.10E-57
SDE2	0	41.33694717	0.1062225	OK	0
H3F3A	0	223.4121414	0.57446249	OK	0
ACBD3	7.69E-200	30.13804765	0.07743678	OK	4.77E-199
PARP1	0	49.26323071	0.12662316	OK	0
STUM	1.19E-06	4.71815632	0.01186454	OK	2.61E-06
PSEN2	3.60E-08	5.386141253	0.01376784	OK	8.34E-08
CDC42BPA	1.14E-170	27.82301436	0.07145877	OK	6.66E-170
SNAP47	1.78E-05	4.134970563	0.01055526	OK	3.71E-05
ARF1	0	89.45766938	0.23000303	OK	0
C1orf35	1.79E-59	16.22120633	0.04164062	OK	7.11E-59
MRPL55	1.01E-120	23.3337113	0.05993532	OK	5.13E-120
GUK1	0	130.1594299	0.33468042	OK	0
TRIM11	4.05E-10	6.14298127	0.01571569	OK	9.91E-10
HIST3H2A	1.58E-11	6.638721788	0.01698283	OK	4.01E-11
RNF187	3.36E-95	20.66797968	0.05307867	OK	1.57E-94
RHOU	4.92E-52	15.13274574	0.03883675	OK	1.87E-51
RAB4A	3.75E-161	27.02509593	0.06942953	OK	2.13E-160

CCSAP	1.00E-183	28.88205432	0.07418647	OK	6.00E-183
GALNT2	1.14E-28	11.04635474	0.02832977	OK	3.69E-28
PGBD5	1.45E-07	5.130458533	0.01307274	OK	3.28E-07
AGT	0	88.65357839	0.22781232	OK	0
C1orf198	0	49.47269351	0.12712243	OK	0
ARV1	1.04E-10	6.355191135	0.01626631	OK	2.59E-10
FAM89A	1.55E-160	26.97254071	0.06927615	OK	8.79E-160
C1orf131	9.00E-25	10.20939625	0.02617828	OK	2.80E-24
EGLN1	2.83E-13	7.208301901	0.01846067	OK	7.44E-13
AL445524.1	6.22E-22	9.5542781	0.02447824	OK	1.88E-21
TSNAX	1.79E-61	16.50133082	0.04236234	OK	7.22E-61
DISC1	2.12E-33	11.98531835	0.03070146	OK	7.15E-33
SIPA1L2	7.29E-55	15.55554973	0.03991568	OK	2.82E-54
NTPCR	2.62E-39	13.06459702	0.03352277	OK	9.22E-39
PCNX2	8.35E-05	3.764321332	0.00958408	OK	0.00016916
COA6	0	53.6848314	0.13799207	OK	0
TARBP1	0.0001195	3.673776264	0.00935746	OK	0.00024025
IRF2BP2	1.90E-121	23.40504679	0.06012083	OK	9.67E-121
AL391832.2	7.58E-08	5.250697364	0.01340019	OK	1.74E-07
AL391832.3	8.55E-05	3.758369721	0.00951869	OK	0.00017316
AL732292.2	2.20E-05	4.085714266	0.01016716	OK	4.58E-05
TOMM20	0	85.5285612	0.21989923	OK	0
ARID4B	2.84E-18	8.638911339	0.02214436	OK	8.11E-18
GGPS1	4.93E-10	6.11150526	0.01564044	OK	1.21E-09
LYST	3.15E-47	14.38629765	0.03691977	OK	1.17E-46
NID1	1.89E-18	8.685192747	0.02221252	OK	5.42E-18
GPR137B	0	76.4007875	0.19640578	OK	0
ERO1B	2.64E-22	9.642854224	0.02471766	OK	7.99E-22
LGALS8	3.28E-75	18.31266348	0.04701654	OK	1.40E-74
ACTN2	1.54E-08	5.536494943	0.01412516	OK	3.62E-08
RYR2	1.01E-58	16.11445041	0.04128499	OK	4.00E-58
CHRM3	1.35E-13	7.309026012	0.0182166	OK	3.56E-13
GREM2	1.37E-162	27.14712054	0.06957759	OK	7.80E-162
RGS7	4.07E-06	4.461561927	0.01055534	OK	8.74E-06
FH	2.36E-60	16.34499635	0.04195765	OK	9.44E-60
KMO	4.63E-92	20.31611129	0.05212821	OK	2.14E-91
OPN3	0	53.80026753	0.13826609	OK	0
CHML	6.72E-163	27.17327172	0.06980006	OK	3.83E-162
EXO1	0	45.93478261	0.11596031	OK	0
PLD5	2.51E-140	25.19111393	0.06458298	OK	1.35E-139
CEP170	0	61.16698308	0.15723702	OK	0
SDCCAG8	7.53E-143	25.42028215	0.06530188	OK	4.09E-142
AKT3	2.38E-227	32.16923387	0.08263846	OK	1.56E-226
ZBTB18	0.00024215	3.489289575	0.00888495	OK	0.00047876
ADSS	8.86E-90	20.05650526	0.05150462	OK	4.05E-89
DESI2	3.35E-115	22.78344309	0.05851947	OK	1.67E-114
COX20	0	54.52548063	0.14016082	OK	0
HNRNPU	6.60E-252	33.87982823	0.08706125	OK	4.47E-251
AL356512.1	2.80E-05	4.029460732	0.01025384	OK	5.79E-05
SMYD3	0.00017654	3.572873893	0.00910833	OK	0.00035189
TFB2M	2.87E-10	6.197462134	0.01585195	OK	7.05E-10
SCCPDH	4.65E-86	19.62583146	0.05039624	OK	2.10E-85
AHCTF1	6.31E-05	3.833592127	0.00978348	OK	0.0001287

ZNF124	7.54E-36	12.44401336	0.03190931	OK	2.58E-35
NLRP3	0	89.49453974	0.23005103	OK	0
GCSAML	1.59E-124	23.70524323	0.05824664	OK	8.18E-124
ZNF672	7.28E-39	12.98669367	0.03331336	OK	2.55E-38
SH3YL1	0.00022105	3.513592503	0.0089588	OK	0.00043801
ACP1	1.21E-260	34.4679042	0.08857334	OK	8.33E-260
ALKAL2	3.05E-16	8.087470522	0.02045527	OK	8.43E-16
TMEM18	4.63E-187	29.14649424	0.07488498	OK	2.79E-186
SNTG2	1.60E-57	15.94263958	0.03475783	OK	6.30E-57
TPO	1.21E-36	12.58896061	0.02994489	OK	4.18E-36
PXDN	2.76E-79	18.8166542	0.0482677	OK	1.21E-78
AC093390.1	2.02E-113	22.6031587	0.05797087	OK	1.00E-112
EIPR1	7.59E-20	9.043492312	0.02316852	OK	2.22E-19
TRAPPC12	4.85E-20	9.0921789	0.02330488	OK	1.42E-19
ADI1	0	101.6409866	0.26133854	OK	0
RNASEH1	7.58E-27	10.66302101	0.02734434	OK	2.41E-26
RNASEH1-AS1	1.84E-12	6.949198054	0.01778561	OK	4.75E-12
RPS7	0	200.9926369	0.51677748	OK	0
COLEC11	3.13E-43	13.73516313	0.03476908	OK	1.13E-42
DCDC2C	3.10E-13	7.195962022	0.01820031	OK	8.14E-13
CMPK2	1.47E-10	6.302270067	0.0161138	OK	3.63E-10
RSAD2	3.06E-09	5.813195091	0.01485206	OK	7.32E-09
LINC01871	8.01E-07	4.798103874	0.0121643	OK	1.77E-06
ID2	2.85E-263	34.64296092	0.08902372	OK	1.96E-262
KIDINS220	1.77E-09	5.904014441	0.01510937	OK	4.27E-09
MBOAT2	6.76E-11	6.421142013	0.01641559	OK	1.69E-10
ASAP2	1.03E-176	28.31821934	0.07273043	OK	6.08E-176
ITGB1BP1	0	38.29991258	0.09842832	OK	0
CPSF3	9.58E-12	6.712317424	0.01718125	OK	2.44E-11
IAH1	2.17E-195	29.79653539	0.07655998	OK	1.33E-194
ADAM17	0	39.01083374	0.10024867	OK	0
YWHAQ	0	65.90630341	0.16943286	OK	0
AC082651.3	2.06E-05	4.100137259	0.00894795	OK	4.31E-05
TAF1B	8.39E-11	6.388282754	0.01635035	OK	2.09E-10
AC104794.2	8.62E-07	4.783404379	0.01221661	OK	1.90E-06
KLF11	1.09E-52	15.23174988	0.03908648	OK	4.15E-52
CYS1	0	45.54366281	0.11697722	OK	0
RRM2	0	178.6233174	0.45749839	OK	0
AC007240.1	0	72.85480389	0.1779761	OK	0
C2orf48	0.00037559	3.370181141	0.00800479	OK	0.00073474
HPCAL1	0	62.13942628	0.15973119	OK	0
ODC1	3.31E-137	24.90474104	0.06397429	OK	1.77E-136
PDIA6	0	95.04969645	0.24438814	OK	0
LINC01954	1.16E-07	5.172078757	0.01313388	OK	2.64E-07
KCNF1	3.26E-25	10.30732722	0.02086614	OK	1.02E-24
PQLC3	1.95E-134	24.64771879	0.06331554	OK	1.03E-133
ROCK2	0	60.32059395	0.15505927	OK	0
E2F6	0.00043116	3.331973663	0.0084872	OK	0.0008406
LPIN1	1.23E-05	4.218648394	0.010771	OK	2.59E-05
TRIB2	1.22E-21	9.483942549	0.02428261	OK	3.68E-21
FAM84A	8.03E-05	3.774036993	0.00955344	OK	0.00016287
DDX1	4.74E-69	17.52356519	0.04499109	OK	1.97E-68
MYCN	4.26E-10	6.134781543	0.01460737	OK	1.04E-09

FAM49A	0	69.24590463	0.17799943	OK	0
SMC6	1.86E-06	4.625990291	0.01181873	OK	4.06E-06
GEN1	2.37E-06	4.576321502	0.01166052	OK	5.13E-06
KCNS3	1.86E-49	14.73728592	0.03773968	OK	6.99E-49
RDH14	2.85E-68	17.42116845	0.0447236	OK	1.18E-67
OSR1	0	68.34499391	0.17561766	OK	0
TTC32	4.54E-14	7.453666241	0.01909044	OK	1.21E-13
MATN3	6.53E-07	4.838779036	0.01226364	OK	1.45E-06
LAPTM4A	0	135.9320782	0.34952466	OK	0
RHOB	0	69.82164348	0.17950206	OK	0
HS1BP3	9.12E-07	4.772106532	0.01219453	OK	2.01E-06
GDF7	8.43E-133	24.49458968	0.0628362	OK	4.46E-132
APOB	4.14E-34	12.11994779	0.03093603	OK	1.40E-33
AC018742.1	6.02E-56	15.71441205	0.04010602	OK	2.35E-55
UBXN2A	9.51E-12	6.713415465	0.01718821	OK	2.42E-11
FKBP1B	1.47E-53	15.36202212	0.03941014	OK	5.65E-53
SF3B6	0	64.57960471	0.16602082	OK	0
FAM228B	4.28E-33	11.92696979	0.03059278	OK	1.44E-32
TP53I3	6.98E-120	23.25080637	0.05971235	OK	3.54E-119
ITSN2	6.18E-124	23.64805214	0.06074426	OK	3.17E-123
NCOA1	3.93E-05	3.948620493	0.01007944	OK	8.08E-05
PTRHD1	0	48.72100843	0.12522567	OK	0
CENPO	1.44E-24	10.16365942	0.02585238	OK	4.48E-24
ADCY3	4.06E-36	12.49339842	0.03202646	OK	1.39E-35
POMC	1.18E-136	24.85367225	0.0638258	OK	6.31E-136
DNMT3A	8.91E-08	5.220677719	0.01334639	OK	2.04E-07
DTNB	2.41E-21	9.412928037	0.02410372	OK	7.21E-21
ASXL2	2.46E-11	6.573620887	0.01682512	OK	6.19E-11
RAB10	0	78.41425702	0.2016014	OK	0
HADHA	0	64.31835208	0.16534918	OK	0
HADHB	0	47.57867556	0.12229468	OK	0
KCNK3	1.68E-79	18.84297375	0.04828781	OK	7.35E-79
SLC35F6	1.80E-143	25.47641915	0.06543753	OK	9.79E-143
CENPA	0	132.8297722	0.33938093	OK	0
TMEM214	1.04E-06	4.744853829	0.01212179	OK	2.29E-06
OST4	0	124.2339938	0.31944006	OK	0
EMILIN1	0	66.02012332	0.16968388	OK	0
KHK	1.11E-13	7.334906838	0.01877625	OK	2.94E-13
CGREF1	2.36E-13	7.233123405	0.01832766	OK	6.21E-13
PREB	5.68E-13	7.112981402	0.01821276	OK	1.48E-12
TCF23	1.79E-05	4.132732308	0.00984029	OK	3.75E-05
ATRAID	0	61.85282183	0.15900766	OK	0
SLC30A3	3.08E-19	8.889185289	0.02264851	OK	8.92E-19
MPV17	9.76E-90	20.05168996	0.05149312	OK	4.46E-89
AC074117.1	1.03E-05	4.257552747	0.0108535	OK	2.18E-05
SNX17	1.91E-302	37.15376839	0.0954818	OK	1.38E-301
PPM1G	0	41.45497941	0.1065446	OK	0
NRBP1	9.29E-100	21.16867599	0.05436741	OK	4.42E-99
FNDC4	1.31E-89	20.03703914	0.0513944	OK	5.98E-89
ZNF512	1.68E-12	6.96195358	0.01781337	OK	4.34E-12
CCDC121	2.03E-18	8.677153989	0.0222109	OK	5.81E-18
GPN1	3.74E-18	8.607263164	0.02205503	OK	1.07E-17
SLC4A1AP	1.05E-26	10.63309193	0.02726984	OK	3.31E-26

MRPL33	0	67.26155515	0.17291697	OK	0
RBKS	4.82E-20	9.093045167	0.0232877	OK	1.41E-19
BABAM2	2.66E-28	10.97015893	0.02813517	OK	8.56E-28
FOSL2	0	62.37570193	0.16034596	OK	0
PLB1	0	44.8678522	0.1152641	OK	0
AC092164.1	0	41.97868216	0.10783009	OK	0
PPP1CB	0	83.97522631	0.21590328	OK	0
WDR43	9.44E-105	21.70383707	0.0557415	OK	4.57E-104
CLIP4	8.23E-17	8.245472946	0.02111966	OK	2.30E-16
YPEL5	0	45.36728018	0.1166071	OK	0
LBH	0	145.7740481	0.37481305	OK	0
LINC01936	1.76E-10	6.274323527	0.0159941	OK	4.34E-10
LCLAT1	2.11E-07	5.058603168	0.01292182	OK	4.77E-07
GALNT14	3.78E-06	4.477032894	0.01116693	OK	8.14E-06
EHD3	1.75E-14	7.578535545	0.01936282	OK	4.69E-14
DPY30	4.32E-183	28.83148492	0.07407677	OK	2.58E-182
SPAST	1.88E-26	10.57834216	0.02712237	OK	5.93E-26
NLRC4	8.03E-16	7.968573192	0.02038545	OK	2.20E-15
BIRC6	0.00021114	3.525753382	0.00899286	OK	0.00041888
LTBP1	0	206.8504575	0.53190744	OK	0
RASGRP3	2.74E-225	32.02142334	0.08223611	OK	1.79E-224
FAM98A	6.71E-47	14.33398436	0.03678499	OK	2.49E-46
AC009414.2	2.37E-211	31.00410832	0.0795997	OK	1.51E-210
CRIM1	0	103.9962648	0.26736495	OK	0
FEZ2	0	48.62168496	0.12497563	OK	0
VIT	0	45.54308472	0.11550933	OK	0
GPATCH11	1.20E-121	23.42447264	0.06016467	OK	6.14E-121
EIF2AK2	7.45E-73	18.01484286	0.046257	OK	3.15E-72
CEBPZOS	3.80E-20	9.118688524	0.02337588	OK	1.12E-19
CEBPZ	5.81E-35	12.27984711	0.0315072	OK	1.98E-34
PRKD3	8.36E-06	4.304600156	0.01099376	OK	1.77E-05
QPCT	0	41.71540103	0.10715878	OK	0
CDC42EP3	1.48E-170	27.81384627	0.0714568	OK	8.59E-170
RMDN2	0.0003824	3.365231151	0.00857117	OK	0.00074772
CYP1B1	9.98E-227	32.1246433	0.08249353	OK	6.52E-226
ATL2	7.14E-07	4.821013361	0.01231745	OK	1.58E-06
GALM	1.43E-153	26.37218305	0.06773788	OK	7.97E-153
SRSF7	2.53E-215	31.29736046	0.08042062	OK	1.62E-214
GEMIN6	3.87E-15	7.771832214	0.01990769	OK	1.05E-14
MORN2	9.06E-51	14.9398956	0.03833806	OK	3.43E-50
ARHGEF33	5.32E-05	3.875413798	0.00970516	OK	0.0001088
SOS1	3.22E-31	11.56177343	0.02965625	OK	1.06E-30
MAP4K3	2.03E-12	6.935279049	0.01775602	OK	5.23E-12
AC007388.1	2.37E-33	11.97619955	0.03071437	OK	7.97E-33
THUMPD2	1.20E-70	17.73130045	0.04551563	OK	5.04E-70
SLC8A1	0	94.5021414	0.24296264	OK	0
LINC01914	4.99E-10	6.10961374	0.01480865	OK	1.22E-09
PKDCC	1.44E-125	23.80626436	0.06110037	OK	7.42E-125
EML4	0	59.23833082	0.15227856	OK	0
COX7A2L	0	73.59985102	0.18922036	OK	0
MTA3	4.14E-13	7.156412085	0.01831552	OK	1.08E-12
HAAO	2.91E-97	20.89601698	0.05364919	OK	1.37E-96
ZFP36L2	0	57.56399775	0.14797605	OK	0

PLEKHH2	4.57E-204	30.45886297	0.07821506	OK	2.86E-203
C1GALT1C1L	0.00017883	3.569502211	0.00902075	OK	0.00035625
DYNC2LI1	2.46E-09	5.849771056	0.01496204	OK	5.90E-09
PPM1B	4.17E-05	3.934442697	0.01004273	OK	8.57E-05
PREPL	7.53E-87	19.71815825	0.05062631	OK	3.41E-86
CAMKMT	7.54E-07	4.81018689	0.01228345	OK	1.67E-06
SIX2	1.37E-10	6.312656134	0.0160719	OK	3.40E-10
SRBD1	7.52E-05	3.790424055	0.00967032	OK	0.00015269
EPAS1	0	96.09010228	0.24704446	OK	0
RHOQ	0	41.15210909	0.10576591	OK	0
PIGF	1.12E-71	17.86447724	0.04586218	OK	4.69E-71
CRIPT	2.40E-45	14.08354635	0.03614408	OK	8.78E-45
SOCS5	2.78E-06	4.542430243	0.01160006	OK	6.01E-06
MCFD2	1.56E-136	24.84239036	0.0638165	OK	8.35E-136
TTC7A	6.01E-36	12.46214112	0.03195525	OK	2.06E-35
CALM2	0	160.6378273	0.41304388	OK	0
EPCAM	9.09E-68	17.35467718	0.04427512	OK	3.76E-67
MSH2	1.16E-13	7.328681331	0.01875635	OK	3.08E-13
KCNK12	2.81E-05	4.027906868	0.00963123	OK	5.83E-05
MSH6	1.25E-23	9.950486	0.02551371	OK	3.86E-23
FBXO11	4.75E-14	7.447575855	0.0190773	OK	1.27E-13
FOXN2	2.49E-234	32.66449266	0.0839268	OK	1.65E-233
PPP1R21	1.61E-06	4.656671609	0.01189817	OK	3.51E-06
STON1	1.93E-11	6.609668531	0.01689267	OK	4.87E-11
NRXN1	7.53E-09	5.660849758	0.01355973	OK	1.78E-08
CHAC2	7.10E-12	6.755944147	0.01725441	OK	1.81E-11
ERLEC1	0	38.38475019	0.09864686	OK	0
PSME4	4.53E-16	8.038983589	0.02059216	OK	1.25E-15
ACYP2	7.92E-48	14.4816024	0.03716534	OK	2.95E-47
SPTBN1	0	95.71751423	0.24607645	OK	0
RTN4	0	69.97386941	0.17989187	OK	0
RPS27A	0	198.0377163	0.50914349	OK	0
MTIF2	7.49E-28	10.87610881	0.02789053	OK	2.40E-27
CCDC88A	0	87.11942419	0.22398909	OK	0
CFAP36	0	52.68686853	0.1354208	OK	0
PPP4R3B	1.36E-05	4.195689601	0.01071574	OK	2.86E-05
PNPT1	1.27E-07	5.153996694	0.01317721	OK	2.90E-07
EFEMP1	0	210.1673635	0.54042342	OK	0
VRK2	5.71E-20	9.074550141	0.02325745	OK	1.67E-19
FANCL	6.22E-05	3.837355295	0.00978723	OK	0.00012677
MIR4432HG	2.43E-17	8.389955696	0.01815772	OK	6.84E-17
BCL11A	0	40.89820875	0.10501244	OK	0
PAPOLG	1.46E-07	5.128297322	0.01310818	OK	3.32E-07
REL	0	185.4016823	0.47676579	OK	0
PEX13	1.16E-06	4.723659583	0.01207011	OK	2.54E-06
C2orf74	1.53E-52	15.209442	0.03903846	OK	5.84E-52
XPO1	2.00E-58	16.07211902	0.04125868	OK	7.91E-58
FAM161A	7.32E-12	6.751369864	0.01727028	OK	1.87E-11
CCT4	0	41.92036639	0.10774213	OK	0
COMMD1	1.59E-195	29.80680645	0.07658522	OK	9.81E-195
B3GNT2	0	45.8167748	0.11775513	OK	0
TMEM17	0.00045601	3.316351725	0.00836972	OK	0.00088692
EHBP1	3.55E-16	8.068850951	0.02066409	OK	9.81E-16

AC009501.1	0.00025026	3.480473395	0.00873159	OK	0.00049449
MDH1	0	61.29050528	0.15756166	OK	0
UGP2	1.80E-102	21.46110122	0.05512141	OK	8.62E-102
PELI1	0	47.41681775	0.12185538	OK	0
AC012368.1	1.04E-85	19.58489899	0.0502274	OK	4.68E-85
LGALSL	0	52.37623866	0.13455721	OK	0
AC008074.3	2.14E-11	6.593825887	0.0168375	OK	5.41E-11
AFTPH	4.90E-20	9.091109643	0.02330272	OK	1.44E-19
SERTAD2	3.85E-22	9.603838175	0.02462066	OK	1.17E-21
SLC1A4	5.77E-10	6.086424973	0.0155688	OK	1.41E-09
CEP68	4.45E-13	7.14642572	0.01829952	OK	1.17E-12
RAB1A	0	37.92383792	0.09746277	OK	0
ACTR2	0	109.2206952	0.28083455	OK	0
MEIS1	2.20E-76	18.45916178	0.04734339	OK	9.49E-76
ETAA1	9.62E-10	6.004030829	0.01535349	OK	2.33E-09
C1D	5.80E-209	30.82639268	0.07920782	OK	3.68E-208
PNO1	4.12E-41	13.37704886	0.03432396	OK	1.47E-40
PPP3R1	1.65E-110	22.30518027	0.05728117	OK	8.13E-110
CNRIP1	8.34E-167	27.50187041	0.07064462	OK	4.81E-166
PLEK	0	276.02858	0.70985392	OK	0
ARHGAP25	6.36E-174	28.09072966	0.07213862	OK	3.73E-173
ANTXR1	0	96.61859142	0.24838049	OK	0
GFPT1	1.95E-24	10.134226	0.02598338	OK	6.05E-24
NFU1	4.30E-106	21.84534815	0.05610445	OK	2.09E-105
AAK1	2.09E-105	21.77306896	0.05592201	OK	1.01E-104
ANXA4	0	52.06393792	0.13383	OK	0
GMCL1	8.08E-08	5.238924672	0.0133957	OK	1.85E-07
SNRNP27	2.70E-45	14.07506273	0.03612297	OK	9.90E-45
MXD1	0	77.19182706	0.19843718	OK	0
PCBP1	0	110.726416	0.28470408	OK	0
TIA1	0.00027512	3.455029905	0.00880939	OK	0.00054204
PCYOX1	0	45.36494388	0.11658186	OK	0
SNRPG	0	76.35365692	0.19630338	OK	0
FAM136A	6.88E-109	22.13750939	0.05685655	OK	3.38E-108
TGFA	1.29E-17	8.464502702	0.02161196	OK	3.64E-17
FIGLA	2.28E-10	6.23380527	0.01529133	OK	5.61E-10
CLEC4F	5.22E-144	25.52488431	0.06476902	OK	2.84E-143
CD207	1.13E-128	24.10415122	0.06069547	OK	5.90E-128
AC007040.2	1.45E-06	4.677317985	0.01022371	OK	3.18E-06
TEX261	2.05E-12	6.93403375	0.01774978	OK	5.28E-12
NAGK	0	128.5209641	0.33047334	OK	0
MCEE	5.03E-09	5.729721367	0.01465541	OK	1.20E-08
MPHOSPH10	4.85E-32	11.72307464	0.03007373	OK	1.62E-31
PAIP2B	3.43E-11	6.523529441	0.01667113	OK	8.62E-11
ZNF638	8.98E-11	6.377889434	0.01632792	OK	2.23E-10
DYSF	8.05E-16	7.968273014	0.02036949	OK	2.21E-15
CYP26B1	5.49E-07	4.873064298	0.01238694	OK	1.22E-06
SPR	1.10E-31	11.65379341	0.0298854	OK	3.64E-31
RAB11FIP5	2.50E-05	4.056088201	0.01033503	OK	5.18E-05
PRADC1	1.15E-33	12.03601978	0.03087395	OK	3.88E-33
CCT7	0	48.02725933	0.1234478	OK	0
EGR4	6.68E-05	3.819667639	0.00956381	OK	0.00013596
TPRKB	5.63E-211	30.97626039	0.07959051	OK	3.58E-210

DUSP11	2.12E-14	7.553644625	0.01934922	OK	5.67E-14
STAMBP	1.66E-35	12.3805735	0.03176405	OK	5.69E-35
ACTG2	0	149.30028	0.38384143	OK	0
DGUOK	4.43E-288	36.25319243	0.09316522	OK	3.14E-287
AC073046.1	4.01E-05	3.943952395	0.00988498	OK	8.24E-05
TET3	6.44E-57	15.85553543	0.04067088	OK	2.52E-56
BOLA3	5.40E-214	31.19958413	0.0801633	OK	3.45E-213
BOLA3-AS1	1.62E-08	5.527847821	0.01411414	OK	3.80E-08
MOB1A	0	85.22677809	0.2191239	OK	0
MTHFD2	2.16E-272	35.24343769	0.09056667	OK	1.51E-271
DCTN1	2.98E-07	4.99283174	0.01276387	OK	6.68E-07
C2orf81	0.00028915	3.441594965	0.00873061	OK	0.00056892
WDR54	4.30E-74	18.1720041	0.04664327	OK	1.83E-73
CCDC142	0.00044159	3.325314481	0.00845289	OK	0.00085995
LBX2-AS1	2.92E-25	10.31794873	0.02641269	OK	9.15E-25
TLX2	2.38E-28	10.98018748	0.01903399	OK	7.67E-28
AUP1	0	56.86642112	0.14618054	OK	0
HTRA2	7.05E-41	13.33699346	0.03422108	OK	2.51E-40
LOXL3	1.62E-08	5.527739789	0.01412743	OK	3.80E-08
DOK1	7.15E-120	23.24980102	0.05970823	OK	3.62E-119
M1AP	3.37E-07	4.968474836	0.01260853	OK	7.56E-07
HK2	5.27E-125	23.75173513	0.06093659	OK	2.71E-124
AC104135.1	4.30E-11	6.489730868	0.01340715	OK	1.08E-10
POLE4	0	37.75852873	0.09703715	OK	0
TACR1	0.00039891	3.353550519	0.00803577	OK	0.0007791
MRPL19	6.27E-39	12.99815688	0.03335258	OK	2.19E-38
SUCLG1	2.06E-194	29.72089162	0.0763638	OK	1.27E-193
TRABD2A	2.44E-36	12.53379675	0.03206776	OK	8.39E-36
TMSB10	0	229.2572462	0.58950921	OK	0
KCMF1	4.51E-59	16.16414096	0.04149516	OK	1.79E-58
TCF7L1	5.49E-115	22.76172503	0.05841731	OK	2.75E-114
TGOLN2	0	64.38084834	0.1655095	OK	0
CAPG	0	206.5957657	0.53127431	OK	0
MAT2A	0	39.3146162	0.10103458	OK	0
GGCX	9.26E-31	11.47071647	0.02942058	OK	3.04E-30
VAMP8	0	309.5319854	0.79603083	OK	0
VAMP5	0	43.20876679	0.11105435	OK	0
RNF181	0	90.17209758	0.23184339	OK	0
TMEM150A	6.69E-62	16.56076596	0.04250778	OK	2.70E-61
USP39	1.93E-07	5.075626734	0.012975	OK	4.37E-07
C2orf68	1.84E-10	6.26735869	0.01603853	OK	4.53E-10
SFTPB	1.44E-05	4.182824859	0.00856501	OK	3.02E-05
GNLY	4.57E-09	5.745868615	0.01467002	OK	1.09E-08
ATOH8	1.59E-132	24.46866966	0.06281004	OK	8.41E-132
ST3GAL5	1.02E-142	25.4084109	0.06524883	OK	5.53E-142
PTCD3	0.00016738	3.586800917	0.00914677	OK	0.00033399
IMMT	8.82E-127	23.9230122	0.06145061	OK	4.57E-126
MRPL35	4.06E-55	15.59309985	0.04002526	OK	1.57E-54
REEP1	2.22E-68	17.43533567	0.04462045	OK	9.23E-68
KDM3A	3.56E-05	3.972207757	0.01013648	OK	7.34E-05
CHMP3	5.59E-197	29.91886777	0.07687261	OK	3.45E-196
RMND5A	7.31E-08	5.257364977	0.01344175	OK	1.68E-07
CD8A	4.27E-19	8.85276299	0.02262289	OK	1.23E-18

CD8B	1.06E-38	12.95815429	0.03310503	OK	3.69E-38
LINC01943	2.77E-206	30.62584499	0.0786067	OK	1.74E-205
CYTOR	8.15E-176	28.24518659	0.07256328	OK	4.80E-175
AC133644.2	1.16E-06	4.722965799	0.01178191	OK	2.55E-06
KRCC1	2.77E-38	12.88411449	0.03305917	OK	9.64E-38
EIF2AK3	1.85E-08	5.504925335	0.01407122	OK	4.32E-08
RPIA	1.10E-70	17.73635382	0.04552857	OK	4.60E-70
IGKC	4.13E-87	19.74850321	0.05071468	OK	1.87E-86
MAL	1.84E-23	9.912413143	0.02534671	OK	5.65E-23
MRPS5	1.27E-30	11.44312606	0.02935508	OK	4.17E-30
KCNIP3	1.24E-06	4.709560497	0.01195443	OK	2.72E-06
FAHD2A	2.26E-05	4.07895552	0.01041207	OK	4.71E-05
ANKRD36C	2.78E-87	19.76859062	0.05074885	OK	1.26E-86
GPAT2	1.20E-09	5.968575852	0.01515101	OK	2.89E-09
ADRA2B	1.90E-23	9.90902362	0.02487533	OK	5.85E-23
DUSP2	0	116.155386	0.29864983	OK	0
STARD7	1.14E-68	17.47363612	0.04485855	OK	4.73E-68
TMEM127	2.49E-75	18.3277095	0.0470529	OK	1.07E-74
CIAO1	7.09E-35	12.26369546	0.03146306	OK	2.41E-34
ITPRIPL1	7.03E-05	3.806983213	0.00969002	OK	0.000143
NCAPH	0	63.0868963	0.16137649	OK	0
NEURL3	0.0001613	3.596435793	0.0090391	OK	0.00032218
ARID5A	0	40.367296	0.10374334	OK	0
LMAN2L	6.50E-05	3.826454043	0.00974929	OK	0.00013238
ANKRD39	1.02E-24	10.1967663	0.02614252	OK	3.19E-24
SEMA4C	4.39E-13	7.148259587	0.01828771	OK	1.15E-12
FAM178B	1.23E-07	5.160939703	0.01307231	OK	2.80E-07
FAHD2B	6.08E-41	13.34806997	0.03421609	OK	2.16E-40
ANKRD36	8.79E-61	16.40510464	0.04209567	OK	3.52E-60
AC092683.1	5.59E-27	10.691349	0.02739289	OK	1.78E-26
ANKRD36B	1.56E-29	11.22350327	0.02876238	OK	5.08E-29
COX5B	0	141.1576372	0.3629629	OK	0
ACTR1B	1.55E-23	9.929121914	0.02545667	OK	4.78E-23
ZAP70	8.71E-37	12.61517348	0.03222882	OK	3.00E-36
INPP4A	7.03E-12	6.757334322	0.01729404	OK	1.80E-11
COA5	2.43E-74	18.20335301	0.04673971	OK	1.03E-73
UNC50	4.29E-77	18.54727486	0.04762466	OK	1.86E-76
MGAT4A	0	123.7534918	0.31819564	OK	0
AC109826.1	3.74E-154	26.42289809	0.06782412	OK	2.09E-153
KIAA1211L	1.09E-70	17.73704317	0.04548318	OK	4.55E-70
MITD1	4.40E-34	12.11489179	0.03108095	OK	1.49E-33
MRPL30	4.14E-06	4.45765339	0.01138556	OK	8.89E-06
TXNDC9	7.32E-63	16.69342772	0.04285514	OK	2.96E-62
EIF5B	5.80E-191	29.45278355	0.07567654	OK	3.53E-190
AFF3	3.43E-227	32.15786362	0.0824993	OK	2.24E-226
LONRF2	2.04E-11	6.601481752	0.01682454	OK	5.14E-11
PDCL3	0	44.4400407	0.11421804	OK	0
RPL31	0	116.2793662	0.29897646	OK	0
TBC1D8	7.20E-14	7.39254386	0.01891345	OK	1.91E-13
CNOT11	0.00014098	3.631340552	0.00926126	OK	0.00028238
RNF149	0	123.5696361	0.31773373	OK	0
MAP4K4	2.50E-06	4.564487544	0.01166337	OK	5.43E-06
IL1R2	0	106.903551	0.27463528	OK	0

IL1R1	2.30E-164	27.29699387	0.07011376	OK	1.32E-163
IL1RL1	1.78E-17	8.426448289	0.0211978	OK	5.02E-17
IL18R1	5.26E-83	19.2652626	0.04933286	OK	2.34E-82
MRPS9	2.70E-30	11.37761151	0.02918281	OK	8.84E-30
C2orf49	6.29E-13	7.098903546	0.01818044	OK	1.64E-12
FHL2	0	52.01281067	0.13362964	OK	0
NCK2	1.41E-30	11.43412151	0.02932588	OK	4.63E-30
C2orf40	0	131.0344046	0.33688416	OK	0
UXS1	9.68E-15	7.654783491	0.01960737	OK	2.61E-14
ST6GAL2	7.91E-285	36.04618203	0.09255615	OK	5.60E-284
SULT1C2	2.18E-30	11.39652442	0.02892331	OK	7.12E-30
SULT1C4	1.51E-07	5.121874275	0.01302064	OK	3.43E-07
GCC2	2.14E-21	9.425573667	0.02416739	OK	6.40E-21
LIMS1	0	98.07103482	0.25215903	OK	0
RANBP2	2.70E-98	21.00928573	0.05395654	OK	1.28E-97
SH3RF3	3.13E-06	4.517419325	0.01152457	OK	6.76E-06
SEPT10	0	59.31217073	0.15246016	OK	0
RGPD5	0.00011755	3.677961143	0.00937497	OK	0.00023645
AC013271.1	6.04E-07	4.854328011	0.01236489	OK	1.34E-06
MALL	1.42E-164	27.31477287	0.06982381	OK	8.11E-164
SMIM37	1.70E-192	29.57231565	0.07598109	OK	1.04E-191
BUB1	0	121.2217259	0.3102719	OK	0
MIR4435-2HG	8.74E-66	17.09044965	0.04386852	OK	3.59E-65
BCL2L11	2.02E-137	24.92444137	0.06400272	OK	1.08E-136
MERTK	0	59.4208988	0.15272342	OK	0
TMEM87B	4.76E-05	3.902511143	0.00995891	OK	9.75E-05
FBLN7	1.50E-78	18.72668033	0.04802354	OK	6.54E-78
ZC3H8	3.46E-09	5.792743278	0.01481878	OK	8.26E-09
ZC3H6	4.01E-31	11.54277732	0.02960724	OK	1.32E-30
TTL	8.30E-23	9.760685528	0.0250165	OK	2.53E-22
CHCHD5	1.24E-190	29.42703285	0.07560581	OK	7.54E-190
SLC20A1	8.88E-106	21.81220736	0.0560117	OK	4.31E-105
CKAP2L	0	146.3892123	0.37283652	OK	0
IL1A	7.91E-07	4.800465102	0.01215523	OK	1.75E-06
IL1B	0	151.7094439	0.39006747	OK	0
IL36B	7.41E-61	16.4154701	0.04130641	OK	2.97E-60
IL36RN	5.10E-39	13.01388267	0.03255621	OK	1.79E-38
IL1RN	0	191.3907331	0.49212619	OK	0
PSD4	3.09E-37	12.69664842	0.03254672	OK	1.07E-36
PAX8	3.29E-67	17.28064143	0.04432242	OK	1.36E-66
CBWD2	1.65E-06	4.651487389	0.01188357	OK	3.60E-06
SLC35F5	0.0003137	3.419486965	0.00871683	OK	0.00061606
AC110769.2	2.68E-05	4.039345933	0.01029125	OK	5.56E-05
ACTR3	0	106.6762546	0.2742911	OK	0
DDX18	7.11E-158	26.74472377	0.06871139	OK	4.01E-157
CCDC93	3.72E-08	5.37998525	0.01375878	OK	8.63E-08
INSIG2	1.85E-67	17.313835	0.04444696	OK	7.64E-67
MARCO	0	236.0503248	0.60695315	OK	0
STEAP3	3.77E-31	11.54811465	0.02959867	OK	1.24E-30
C2orf76	1.74E-09	5.906952751	0.01511143	OK	4.19E-09
DBI	0	178.4672433	0.45892819	OK	0
TMEM37	0	112.2522601	0.28857997	OK	0
TMEM185B	4.45E-05	3.918529777	0.00999393	OK	9.13E-05

RALB	1.61E-110	22.30619421	0.05728849	OK	7.95E-110
GLI2	3.83E-05	3.954536797	0.00996825	OK	7.89E-05
TFCP2L1	1.42E-16	8.180086923	0.02088324	OK	3.95E-16
NIFK-AS1	6.90E-23	9.779490175	0.02505957	OK	2.11E-22
NIFK	9.59E-242	33.18260039	0.08526583	OK	6.41E-241
TSN	1.82E-32	11.80580412	0.03028461	OK	6.09E-32
GYPC	0	88.75661145	0.22820234	OK	0
BIN1	0	49.92798284	0.12831455	OK	0
MAP3K2	3.65E-117	22.98053768	0.05902725	OK	1.84E-116
PROC	6.94E-05	3.810422043	0.00954617	OK	0.00014107
LIMS2	0	73.71648022	0.18947414	OK	0
WDR33	2.64E-20	9.157999091	0.02347801	OK	7.80E-20
POLR2D	7.50E-06	4.328661527	0.01105424	OK	1.59E-05
UGGT1	7.33E-07	4.815701598	0.01230572	OK	1.62E-06
HS6ST1	0.00016996	3.582807057	0.00912473	OK	0.00033901
MZT2B	0	96.17160878	0.24727115	OK	0
CCDC115	3.91E-180	28.59465903	0.07346611	OK	2.32E-179
IMP4	6.11E-85	19.49450526	0.05006058	OK	2.74E-84
PTPN18	0	41.33705777	0.10623316	OK	0
ARHGEF4	0.00044356	3.324074451	0.00838923	OK	0.00086356
FAM168B	5.72E-05	3.857915147	0.00984491	OK	0.00011671
PLEKHB2	0	88.87962841	0.22851145	OK	0
MZT2A	0	52.0868296	0.13388773	OK	0
CCDC74A	3.04E-11	6.54190096	0.01667797	OK	7.64E-11
LYPD1	2.34E-05	4.071059137	0.01022478	OK	4.86E-05
NCKAP5	1.09E-117	23.03312986	0.05907945	OK	5.48E-117
MGAT5	1.61E-24	10.15301238	0.02602755	OK	4.99E-24
TMEM163	2.02E-178	28.45666555	0.07303106	OK	1.19E-177
RAB3GAP1	1.06E-25	10.41495168	0.02670742	OK	3.33E-25
R3HDM1	5.41E-07	4.876234404	0.01246315	OK	1.20E-06
UBXN4	0	38.5156156	0.09898558	OK	0
MCM6	2.06E-25	10.35164961	0.02651749	OK	6.44E-25
DARS	1.33E-73	18.10995768	0.04650097	OK	5.64E-73
DARS-AS1	1.36E-06	4.690208209	0.01192829	OK	2.99E-06
CXCR4	0	176.6722406	0.45430698	OK	0
HNMT	0	111.9850106	0.28794249	OK	0
SPOPL	7.41E-09	5.663657803	0.01448855	OK	1.75E-08
KYNU	0	70.7006401	0.18173649	OK	0
ARHGAP15	0	40.35393118	0.10367894	OK	0
GTDC1	6.39E-06	4.363734101	0.01114227	OK	1.36E-05
ZEB2	0	88.48854311	0.22751226	OK	0
ZEB2-AS1	1.02E-150	26.12217823	0.06707646	OK	5.65E-150
TEX41	1.07E-125	23.81857998	0.06111997	OK	5.54E-125
AC092484.1	1.20E-86	19.69441854	0.0501027	OK	5.44E-86
EPC2	1.25E-12	7.003428163	0.01793328	OK	3.24E-12
MMADHC	1.15E-179	28.5569813	0.07337043	OK	6.81E-179
RND3	1.14E-146	25.76327575	0.06617427	OK	6.26E-146
RBM43	5.62E-101	21.3004835	0.05469215	OK	2.68E-100
NMI	0	39.84815983	0.10240184	OK	0
TNFAIP6	1.87E-23	9.910608514	0.02534106	OK	5.76E-23
ARL5A	6.60E-08	5.276075127	0.01349097	OK	1.52E-07
CACNB4	2.01E-35	12.36525899	0.03163425	OK	6.88E-35
STAM2	4.70E-19	8.842072683	0.02266053	OK	1.36E-18

FMNL2	0	43.30339226	0.11128338	OK	0
PRPF40A	0	43.70125876	0.11232212	OK	0
ARL6IP6	3.29E-35	12.3257321	0.03162082	OK	1.12E-34
GALNT13	1.87E-22	9.678109704	0.02401462	OK	5.67E-22
NR4A2	0	176.0578801	0.45273189	OK	0
GPD2	3.92E-08	5.370855399	0.01372468	OK	9.07E-08
GALNT5	1.72E-28	11.00945204	0.0280109	OK	5.55E-28
CYTIP	0	161.6578668	0.41568398	OK	0
DAPL1	1.07E-25	10.41373822	0.02668917	OK	3.37E-25
TANC1	4.38E-75	18.2968858	0.04693781	OK	1.87E-74
BAZ2B	1.63E-29	11.21983918	0.02878019	OK	5.30E-29
AC009506.1	0.00014782	3.619082861	0.0092233	OK	0.00029585
MARCH7	3.08E-11	6.539940745	0.01674496	OK	7.74E-11
CD302	0	78.2826451	0.20125916	OK	0
LY75	0.0001551	3.606623304	0.0091863	OK	0.00031017
PLA2R1	5.69E-225	31.99864809	0.08216534	OK	3.71E-224
RBMS1	1.29E-71	17.85628805	0.0458499	OK	5.43E-71
TANK	0	41.96876465	0.10786232	OK	0
PSMD14	2.05E-260	34.45271834	0.08853078	OK	1.41E-259
DPP4	1.39E-67	17.33008234	0.04436123	OK	5.77E-67
FAP	0	44.02339169	0.1129848	OK	0
IFIH1	1.67E-31	11.61776653	0.02979491	OK	5.54E-31
GCA	0	111.7477167	0.28732574	OK	0
FIGN	1.81E-104	21.67374703	0.05561034	OK	8.77E-104
COBLL1	0	52.7511314	0.13555335	OK	0
AC019197.1	5.04E-15	7.738312723	0.01874734	OK	1.37E-14
SLC38A11	1.74E-64	16.91521668	0.04253414	OK	7.08E-64
SCN3A	2.01E-44	13.9325012	0.03559051	OK	7.33E-44
CSRNP3	1.63E-26	10.59141787	0.02705574	OK	5.17E-26
GALNT3	3.13E-133	24.53501334	0.06288393	OK	1.65E-132
TTC21B	4.21E-06	4.45403391	0.01137046	OK	9.04E-06
SCN9A	0	39.25721999	0.10076416	OK	0
STK39	4.58E-10	6.123388192	0.01565788	OK	1.12E-09
CERS6	3.51E-52	15.1549217	0.03889091	OK	1.34E-51
NOSTRIN	0	72.94857864	0.18517057	OK	0
SPC25	0	183.9118015	0.46883858	OK	0
DHRS9	0	102.7646694	0.26415342	OK	0
PPIG	3.89E-260	34.4341684	0.08848827	OK	2.66E-259
PHOSPHO2	0.00022196	3.512494793	0.00893785	OK	0.00043975
KLHL23	0	89.82477233	0.23071303	OK	0
SSB	0	63.77920838	0.16396196	OK	0
METTL5	0	41.50614956	0.10667461	OK	0
SP5	3.08E-06	4.520575207	0.01137357	OK	6.66E-06
AC007405.3	7.66E-15	7.684895681	0.01961421	OK	2.07E-14
ERICH2	0.00049141	3.295399027	0.00831679	OK	0.00095367
GORASP2	1.04E-40	13.30777582	0.03414775	OK	3.71E-40
TLK1	1.08E-31	11.65514454	0.02989945	OK	3.58E-31
CYBRD1	0	42.62599422	0.10953906	OK	0
DYNC1I2	9.51E-235	32.6939746	0.0840122	OK	6.30E-234
SLC25A12	4.22E-24	10.0584045	0.02577939	OK	1.31E-23
HAT1	4.81E-95	20.65072571	0.05303401	OK	2.25E-94
METAP1D	1.15E-07	5.172574185	0.01320544	OK	2.63E-07
DLX1	8.50E-32	11.67549518	0.02986335	OK	2.82E-31

DLX2	4.68E-57	15.87549407	0.04066975	OK	1.84E-56
ITGA6	1.06E-82	19.22875511	0.04934137	OK	4.72E-82
AC078883.3	9.12E-20	9.023332336	0.02305103	OK	2.66E-19
AC078883.1	4.63E-20	9.097394449	0.02326729	OK	1.36E-19
PDK1	6.05E-20	9.068184174	0.02322921	OK	1.77E-19
RAPGEF4	4.38E-11	6.486924251	0.01581066	OK	1.10E-10
MAP3K20	0	82.04607118	0.21092447	OK	0
CDCA7	3.42E-154	26.42628549	0.06738013	OK	1.91E-153
SP3	1.39E-10	6.310092884	0.01615313	OK	3.46E-10
OLA1	7.05E-289	36.30378465	0.0932933	OK	5.01E-288
CIR1	4.47E-124	23.6617594	0.06078115	OK	2.29E-123
SCRN3	1.60E-05	4.15891985	0.01061391	OK	3.35E-05
GPR155	0	49.31256119	0.12671757	OK	0
WIPF1	0	54.32053444	0.13962831	OK	0
CHN1	7.21E-66	17.10169037	0.04384958	OK	2.96E-65
ATF2	7.43E-11	6.406734895	0.0163992	OK	1.85E-10
ATP5MC3	0	135.8142205	0.34922725	OK	0
LNPK	8.45E-25	10.21553235	0.02619349	OK	2.63E-24
HOXD9	3.49E-42	13.55930401	0.03198943	OK	1.26E-41
HOXD8	0	53.74519863	0.13496558	OK	0
HAGLROS	1.09E-24	10.19099587	0.02525053	OK	3.38E-24
HOXD1	2.25E-131	24.36031349	0.05916671	OK	1.19E-130
MTX2	4.09E-44	13.88162134	0.03562167	OK	1.49E-43
LINC01116	5.67E-10	6.089171309	0.01539881	OK	1.38E-09
LINC01117	5.60E-05	3.862918226	0.00912513	OK	0.00011438
HNRNPA3	0	42.21129416	0.10849034	OK	0
NFE2L2	0	81.11482983	0.2085479	OK	0
AC079305.1	7.32E-15	7.69062718	0.01966739	OK	1.98E-14
AGPS	9.65E-45	13.98479685	0.03588483	OK	3.52E-44
OSBPL6	0.00016837	3.585255029	0.00911178	OK	0.00033588
PRKRA	1.43E-111	22.41408373	0.0575676	OK	7.10E-111
FKBP7	0	37.53646105	0.09642957	OK	0
PLEKHA3	1.80E-73	18.09328728	0.04645793	OK	7.64E-73
TTN	3.45E-196	29.85801636	0.07651237	OK	2.13E-195
CCDC141	1.32E-43	13.79721964	0.03528848	OK	4.80E-43
SESTD1	2.28E-92	20.35089845	0.05225641	OK	1.06E-91
ZNF385B	2.15E-05	4.090298485	0.01033958	OK	4.49E-05
CWC22	1.71E-15	7.874555692	0.02017562	OK	4.67E-15
UBE2E3	3.00E-254	34.03851088	0.08746892	OK	2.04E-253
LINC01934	3.95E-08	5.369517159	0.01358405	OK	9.13E-08
ITGA4	0	132.2570262	0.34005207	OK	0
SSFA2	7.35E-14	7.389901833	0.01892961	OK	1.95E-13
PDE1A	0	59.35434045	0.15253041	OK	0
DNAJC10	5.07E-20	9.087354738	0.02329485	OK	1.49E-19
FRZB	0	271.2615188	0.69756205	OK	0
NCKAP1	0	58.9153545	0.1514073	OK	0
DUSP19	7.13E-39	12.98828359	0.03327875	OK	2.50E-38
AC096667.1	0	37.82966063	0.0971829	OK	0
ZNF804A	2.04E-07	5.064817488	0.01292421	OK	4.62E-07
ZC3H15	0	42.35528923	0.10886103	OK	0
ITGAV	9.08E-43	13.65767308	0.03504774	OK	3.28E-42
FAM171B	1.78E-31	11.61253208	0.02969642	OK	5.88E-31
CALCRL	0	48.24487577	0.12395002	OK	0

TFPI	0	68.33751974	0.17566494	OK	0
GULP1	0	84.29793226	0.2166849	OK	0
COL3A1	0	100.6169075	0.25865303	OK	0
COL5A2	0	38.21876036	0.09816272	OK	0
SLC40A1	0	97.30990376	0.25018967	OK	0
ASNSD1	1.08E-13	7.338318533	0.01879638	OK	2.86E-13
ANKAR	1.24E-20	9.239224969	0.0236719	OK	3.68E-20
OSGEPL1	0.00027079	3.459299463	0.00881075	OK	0.00053375
ORMDL1	3.57E-59	16.17857708	0.04153548	OK	1.42E-58
HIBCH	2.46E-53	15.32863601	0.03934113	OK	9.43E-53
INPP1	7.43E-11	6.406709561	0.01639768	OK	1.85E-10
MFSD6	1.64E-08	5.525805059	0.01411707	OK	3.84E-08
NAB1	0.00017903	3.569214755	0.00910255	OK	0.00035661
GLS	1.44E-49	14.75451791	0.03787302	OK	5.42E-49
STAT1	2.17E-222	31.81256429	0.08174082	OK	1.40E-221
STAT4	3.68E-25	10.29581493	0.02635222	OK	1.15E-24
MYO1B	0	45.88402521	0.11787338	OK	0
NABP1	0	109.6323631	0.28187894	OK	0
CAVIN2	6.63E-115	22.7534534	0.05839718	OK	3.31E-114
TMEFF2	4.45E-09	5.750462522	0.01460437	OK	1.06E-08
LINC01827	3.69E-05	3.963522437	0.00978976	OK	7.60E-05
SLC39A10	4.74E-153	26.32673324	0.0676246	OK	2.64E-152
STK17B	0	140.8110018	0.36207682	OK	0
AC114760.2	1.49E-36	12.57260177	0.03222088	OK	5.14E-36
AC020571.1	3.99E-13	7.161666693	0.01764914	OK	1.04E-12
ANKRD44	3.92E-59	16.17277594	0.04150914	OK	1.56E-58
AC010746.1	2.30E-05	4.075077057	0.00971987	OK	4.78E-05
SF3B1	8.67E-37	12.61551674	0.03237224	OK	2.99E-36
COQ10B	6.41E-47	14.33711804	0.03679757	OK	2.38E-46
HSPD1	0	78.10020538	0.20079468	OK	0
HSPE1	0	81.08531968	0.20847231	OK	0
MOB4	1.71E-79	18.84212344	0.04838273	OK	7.47E-79
RFTN2	2.50E-62	16.61993842	0.04260377	OK	1.01E-61
PLCL1	6.68E-169	27.67658037	0.07107662	OK	3.87E-168
FTCDNL1	5.01E-17	8.304572255	0.02124175	OK	1.40E-16
MAIP1	1.28E-09	5.957030329	0.01523786	OK	3.10E-09
SPATS2L	0	45.16851719	0.11609286	OK	0
SGO2	2.10E-212	31.08210504	0.07977263	OK	1.34E-211
AOX1	3.43E-97	20.88823394	0.05331906	OK	1.61E-96
BZW1	0	111.3126519	0.28621476	OK	0
CLK1	1.44E-30	11.4326296	0.02932827	OK	4.71E-30
PPIL3	4.78E-19	8.840090611	0.02265364	OK	1.38E-18
NIF3L1	8.78E-10	6.018986759	0.01539931	OK	2.13E-09
NDUFB3	0	59.34887358	0.15256718	OK	0
CFLAR	0	38.45676714	0.098832	OK	0
CASP10	2.66E-84	19.41909353	0.04982953	OK	1.19E-83
CASP8	9.13E-188	29.20206432	0.07500806	OK	5.51E-187
TRAK2	6.17E-12	6.776140213	0.01734611	OK	1.58E-11
TMEM237	1.68E-233	32.60609287	0.08374019	OK	1.11E-232
FZD7	4.16E-10	6.138552911	0.01568852	OK	1.02E-09
SUMO1	0	57.22347785	0.14710141	OK	0
NOP58	1.80E-38	12.91714053	0.03314555	OK	6.29E-38
BMPR2	7.81E-134	24.59135879	0.06316011	OK	4.14E-133

ICA1L	2.59E-11	6.565717109	0.0167718	OK	6.52E-11
WDR12	4.64E-16	8.036155304	0.02058239	OK	1.28E-15
NBEAL1	1.65E-49	14.74521771	0.03784725	OK	6.22E-49
CYP20A1	8.52E-27	10.65213629	0.02732055	OK	2.71E-26
ABI2	1.48E-16	8.17494168	0.02094695	OK	4.12E-16
RAPH1	1.42E-18	8.71743697	0.02233952	OK	4.08E-18
CD28	0	38.59376883	0.09910983	OK	0
CTLA4	2.51E-40	13.24212013	0.03384184	OK	8.88E-40
ICOS	5.03E-48	14.51278147	0.03711949	OK	1.88E-47
PARD3B	8.18E-47	14.32020723	0.03670672	OK	3.03E-46
NRP2	0	100.8001775	0.25916863	OK	0
NDUFS1	5.42E-31	11.51693126	0.02954268	OK	1.78E-30
EEF1B2	0	164.6443259	0.42335549	OK	0
ZDBF2	0.00027105	3.459044871	0.00879906	OK	0.00053421
ADAM23	1.48E-05	4.176666642	0.01058282	OK	3.11E-05
KLF7	7.60E-20	9.04333776	0.02317661	OK	2.22E-19
CREB1	2.13E-19	8.929790396	0.02289004	OK	6.20E-19
METTL21A	2.21E-73	18.08192799	0.04641689	OK	9.38E-73
LINC01857	2.50E-180	28.61038016	0.07344144	OK	1.48E-179
CCNYL1	2.26E-40	13.24997657	0.0339901	OK	8.00E-40
FZD5	2.00E-06	4.611597257	0.01176853	OK	4.35E-06
IDH1	0	52.47829758	0.13487818	OK	0
PIKFYVE	0.00027886	3.451380039	0.0087975	OK	0.00054922
MAP2	4.91E-48	14.51442797	0.03720488	OK	1.83E-47
RPE	2.30E-06	4.582572339	0.01170832	OK	4.99E-06
KANSL1L	7.57E-16	7.975866216	0.02042593	OK	2.08E-15
ACADL	4.97E-76	18.41514823	0.04722468	OK	2.14E-75
LANCL1	1.66E-12	6.963653976	0.01781914	OK	4.29E-12
ERBB4	2.69E-97	20.89981267	0.05361696	OK	1.27E-96
SPAG16	7.66E-274	35.33801294	0.09078263	OK	5.34E-273
BARD1	4.30E-32	11.73329815	0.03009382	OK	1.43E-31
ATIC	5.80E-31	11.51111214	0.02952445	OK	1.91E-30
FN1	0	153.7955269	0.39547636	OK	0
MREG	0	38.6638167	0.09914748	OK	0
XRCC5	0	58.22665333	0.14968157	OK	0
SMARCAL1	4.16E-05	3.93505374	0.01003594	OK	8.54E-05
RPL37A	0	151.1125982	0.38853029	OK	0
IGFBP2	0	252.2618563	0.64870152	OK	0
IGFBP5	0	247.7147018	0.63701661	OK	0
AC007563.2	6.29E-16	7.99859425	0.02033921	OK	1.73E-15
TNS1	0	99.96164116	0.25700258	OK	0
ARPC2	0	186.7748031	0.48027705	OK	0
GPBAR1	6.69E-32	11.69588354	0.02996285	OK	2.22E-31
AAMP	3.37E-54	15.45719196	0.03967783	OK	1.30E-53
PNKD	0	48.76567487	0.12534576	OK	0
TMBIM1	0	39.22300609	0.10080192	OK	0
SLC11A1	0	191.465694	0.49231417	OK	0
CTDSP1	8.70E-42	13.49210162	0.0346244	OK	3.12E-41
VIL1	4.80E-05	3.900590792	0.00981675	OK	9.82E-05
USP37	9.63E-18	8.498175411	0.02177375	OK	2.73E-17
CNOT9	1.87E-45	14.10104253	0.0361864	OK	6.86E-45
BCS1L	1.72E-10	6.277549559	0.01606145	OK	4.25E-10
RNF25	2.69E-05	4.038055484	0.01030309	OK	5.59E-05

TTLL4	7.80E-09	5.654760483	0.0144352	OK	1.84E-08
CYP27A1	0	118.3375239	0.30424615	OK	0
IHH	1.03E-06	4.747323908	0.01172926	OK	2.27E-06
SLC23A3	2.35E-10	6.228554125	0.01566739	OK	5.80E-10
CNPPD1	5.72E-14	7.423196849	0.01901462	OK	1.52E-13
RETREG2	1.60E-20	9.212329052	0.02361575	OK	4.72E-20
ZFAND2B	1.30E-11	6.667847956	0.01707294	OK	3.29E-11
GLB1L	1.67E-25	10.37137594	0.02656826	OK	5.25E-25
STK16	9.38E-15	7.658838196	0.01961886	OK	2.53E-14
TUBA4A	1.44E-203	30.42126033	0.07815718	OK	8.99E-203
DNAJB2	4.83E-08	5.333159466	0.01363771	OK	1.11E-07
DNPEP	3.40E-14	7.491554733	0.01919159	OK	9.10E-14
DES	0	52.46533732	0.1346591	OK	0
SPEG	0	68.32104407	0.17559433	OK	0
GMPPA	4.32E-19	8.851504328	0.02268373	OK	1.25E-18
CHPF	1.33E-294	36.6648304	0.09417153	OK	9.53E-294
OBSL1	1.11E-112	22.52793342	0.05781241	OK	5.49E-112
EPHA4	1.69E-25	10.37025182	0.02652837	OK	5.31E-25
FARSB	0.0001705	3.581984793	0.00913328	OK	0.00034005
ACSL3	1.86E-127	23.98793833	0.06161927	OK	9.65E-127
KCNE4	0	65.69954257	0.16883781	OK	0
SCG2	1.26E-302	37.16483732	0.09511571	OK	9.16E-302
AP1S3	8.37E-122	23.43994693	0.05998132	OK	4.27E-121
WDFY1	1.60E-08	5.529729028	0.01414292	OK	3.76E-08
MRPL44	9.33E-33	11.8619409	0.03042513	OK	3.13E-32
SERPINE2	4.16E-186	29.07116534	0.07448759	OK	2.50E-185
CUL3	8.81E-15	7.666973927	0.01964256	OK	2.38E-14
DOCK10	1.09E-120	23.33056698	0.0599237	OK	5.52E-120
AC016717.2	0.00022109	3.513545064	0.00752614	OK	0.00043805
IRS1	2.16E-17	8.403639699	0.02148398	OK	6.10E-17
COL4A4	1.16E-39	13.12640059	0.03359851	OK	4.10E-39
COL4A3	2.68E-11	6.560300737	0.0167141	OK	6.76E-11
MFF	1.56E-77	18.60156218	0.04776578	OK	6.76E-77
AGFG1	0	53.02496423	0.1362917	OK	0
CCL20	0	47.83025719	0.12269452	OK	0
DAW1	1.95E-37	12.73271059	0.03216709	OK	6.74E-37
PID1	1.22E-289	36.35204253	0.09341142	OK	8.69E-289
TRIP12	1.48E-17	8.448234901	0.02165257	OK	4.18E-17
FBXO36	1.78E-05	4.134068681	0.0105106	OK	3.73E-05
SP110	0	56.82521957	0.1460715	OK	0
SP140	2.92E-116	22.89002633	0.05874282	OK	1.47E-115
SP140L	9.18E-48	14.47146011	0.03713767	OK	3.42E-47
SP100	1.07E-212	31.10380382	0.07992236	OK	6.83E-212
CAB39	5.95E-103	21.51245516	0.05524843	OK	2.86E-102
ITM2C	0	80.86143611	0.20787315	OK	0
PSMD1	4.18E-79	18.79458677	0.04826216	OK	1.83E-78
HTR2B	7.14E-22	9.540009171	0.02437145	OK	2.15E-21
ARMC9	3.31E-08	5.401143079	0.01379134	OK	7.68E-08
B3GNT7	2.75E-66	17.15772208	0.04400195	OK	1.13E-65
NCL	0	94.65657494	0.24337003	OK	0
PTMA	0	187.4818139	0.48195564	OK	0
PDE6D	0.00028951	3.441259416	0.00877423	OK	0.00056957
EIF4E2	0	70.22957691	0.18054623	OK	0

EFHD1	0	164.6592898	0.42337132	OK	0
GIGYF2	3.63E-07	4.954076691	0.01266528	OK	8.14E-07
INPP5D	0	39.88967259	0.10247782	OK	0
DGKD	4.22E-06	4.453961049	0.01137143	OK	9.04E-06
HJURP	0	92.59724267	0.23341843	OK	0
ARL4C	0	169.0777553	0.43477194	OK	0
SH3BP4	2.03E-74	18.21313391	0.04668854	OK	8.66E-74
AGAP1	1.40E-10	6.309787908	0.01611206	OK	3.46E-10
ACKR3	0	66.81346328	0.17167075	OK	0
COPS8	8.66E-124	23.63379964	0.06070485	OK	4.44E-123
COL6A3	0	47.37731193	0.12167455	OK	0
MLPH	1.68E-17	8.43333881	0.02143023	OK	4.74E-17
LRRFIP1	0	74.6617491	0.19194892	OK	0
RAMP1	0	274.7806677	0.70661781	OK	0
UBE2F	0	39.27228539	0.10092475	OK	0
KLHL30	2.52E-17	8.385937748	0.02139939	OK	7.08E-17
ILKAP	4.49E-05	3.916668803	0.00999706	OK	9.20E-05
HES6	5.99E-11	6.439502992	0.01646434	OK	1.50E-10
PER2	5.73E-11	6.446437154	0.01650125	OK	1.43E-10
TRAF3IP1	1.41E-20	9.225239834	0.02363996	OK	4.19E-20
TWIST2	3.27E-32	11.75638867	0.0300999	OK	1.09E-31
AC093802.1	1.07E-05	4.249535393	0.01056236	OK	2.26E-05
NDUFA10	1.24E-162	27.15076175	0.06975402	OK	7.07E-162
COPS9	0	68.94381249	0.17724543	OK	0
GPC1	8.11E-180	28.56920754	0.07334317	OK	4.81E-179
DUSP28	4.83E-09	5.7366549	0.01467245	OK	1.15E-08
RNPEPL1	0	39.25004941	0.10086683	OK	0
GPR35	9.27E-104	21.59852277	0.05542113	OK	4.47E-103
SNED1	6.98E-30	11.29461082	0.02889918	OK	2.27E-29
MTERF4	3.60E-10	6.161466754	0.0157687	OK	8.84E-10
PPP1R7	4.22E-289	36.31790148	0.09333184	OK	3.00E-288
HDLBP	2.40E-102	21.44767567	0.05508694	OK	1.15E-101
SEPT2	9.27E-228	32.19848109	0.08273811	OK	6.07E-227
FARP2	1.09E-05	4.245666736	0.01083403	OK	2.30E-05
STK25	3.28E-26	10.52598435	0.02699472	OK	1.03E-25
THAP4	8.05E-07	4.796996013	0.01225708	OK	1.78E-06
ATG4B	0.00040024	3.352631805	0.00854519	OK	0.00078162
DTYMK	0	43.55772323	0.11193301	OK	0
NEU4	3.56E-22	9.611929781	0.02453492	OK	1.08E-21
PDCD1	3.61E-21	9.370374224	0.02391323	OK	1.08E-20
LINC01266	0.00012512	3.662010746	0.00926873	OK	0.00025133
CNTN4	4.36E-304	37.25524678	0.09565629	OK	3.17E-303
TRNT1	3.34E-06	4.503715057	0.01150452	OK	7.20E-06
CRBN	4.92E-173	28.01788611	0.07198126	OK	2.88E-172
SUMF1	2.39E-17	8.39219138	0.02150237	OK	6.71E-17
LRRN1	0.00042363	3.336874384	0.00838588	OK	0.00082629
SETMAR	4.16E-27	10.71876571	0.02745893	OK	1.32E-26
ITPR1	9.95E-09	5.612817714	0.01435513	OK	2.35E-08
BHLHE40	0	42.50046945	0.10922544	OK	0
ARL8B	0	59.53999138	0.1530561	OK	0
EDEM1	4.16E-228	32.2233452	0.0827826	OK	2.73E-227
LMCD1	0	161.604943	0.41552087	OK	0
CAV3	1.25E-05	4.21554635	0.01047587	OK	2.62E-05

OXTR	4.16E-23	9.830569878	0.02497826	OK	1.27E-22
RAD18	4.90E-12	6.809271683	0.01741409	OK	1.26E-11
SRGAP3	5.34E-278	35.60762326	0.09145253	OK	3.74E-277
THUMPD3-AS1	3.43E-10	6.169447235	0.01579144	OK	8.40E-10
THUMPD3	6.02E-35	12.27701161	0.03149633	OK	2.05E-34
MTMR14	0	54.1709324	0.13923381	OK	0
CAMK1	1.78E-276	35.50900647	0.09124579	OK	1.25E-275
TADA3	1.19E-118	23.12889949	0.05940934	OK	6.00E-118
ARPC4	0	77.7885298	0.19999376	OK	0
TTLL3	5.45E-05	3.86953242	0.009865	OK	0.00011143
AC022382.1	4.30E-07	4.921068379	0.01189926	OK	9.60E-07
RPUSD3	1.31E-10	6.319984156	0.0161759	OK	3.25E-10
JAGN1	1.59E-64	16.92038392	0.04343948	OK	6.49E-64
IL17RE	6.48E-05	3.827109792	0.00975048	OK	0.00013204
CRELD1	4.24E-07	4.923820491	0.01258469	OK	9.47E-07
AC018809.2	6.92E-26	10.45537771	0.02597189	OK	2.18E-25
EMC3	0	38.66606257	0.09937034	OK	0
FANCD2	1.34E-77	18.60984234	0.04762051	OK	5.80E-77
BRK1	0	95.74184805	0.24616675	OK	0
VHL	2.03E-27	10.78484601	0.02765536	OK	6.48E-27
IRAK2	8.77E-15	7.667511265	0.01962167	OK	2.37E-14
TATDN2	7.08E-07	4.822735867	0.01231095	OK	1.57E-06
GHRL	4.84E-112	22.46241738	0.05755859	OK	2.40E-111
SEC13	2.34E-115	22.79910214	0.05855992	OK	1.17E-114
ATP2B2	2.58E-05	4.048709002	0.00915616	OK	5.35E-05
SLC6A1	5.28E-08	5.316775483	0.01344287	OK	1.22E-07
HRH1	2.25E-124	23.6905833	0.06082769	OK	1.16E-123
ATG7	7.26E-109	22.13507423	0.05684016	OK	3.57E-108
VGLL4	0	70.33982996	0.18082056	OK	0
TIMP4	1.08E-164	27.32457452	0.07015028	OK	6.21E-164
PPARG	0	110.3975789	0.28381238	OK	0
MKRN2	2.07E-08	5.484463471	0.01402545	OK	4.84E-08
CAND2	9.25E-10	6.010411771	0.0153094	OK	2.24E-09
RPL32	0	178.664442	0.45932499	OK	0
IQSEC1	4.57E-11	6.480428203	0.01658802	OK	1.15E-10
NUP210	1.25E-71	17.85789592	0.04577224	OK	5.28E-71
HDAC11	8.15E-16	7.966710293	0.02039721	OK	2.24E-15
FBLN2	0	78.240282	0.2007961	OK	0
CHCHD4	1.35E-05	4.197305997	0.01071414	OK	2.84E-05
TMEM43	4.64E-247	33.54907692	0.08619993	OK	3.12E-246
XPC	4.45E-16	8.041166942	0.02060329	OK	1.23E-15
LSM3	0	59.9236906	0.15404592	OK	0
SLC6A6	0	64.17213864	0.16493201	OK	0
GRIP2	5.64E-18	8.560165937	0.02184738	OK	1.60E-17
CCDC174	7.16E-21	9.297952807	0.02383627	OK	2.13E-20
FGD5	1.07E-11	6.695604208	0.01702871	OK	2.73E-11
FGD5-AS1	8.29E-96	20.73548318	0.05325302	OK	3.89E-95
MRPS25	5.63E-105	21.72753878	0.05579927	OK	2.73E-104
SH3BP5	1.54E-155	26.54328455	0.06818532	OK	8.63E-155
EAF1	1.94E-294	36.65450783	0.09417644	OK	1.39E-293
EAF1-AS1	0.00024595	3.485130468	0.0088291	OK	0.00048614
HACL1	6.77E-08	5.271331794	0.0134778	OK	1.55E-07
BTD	1.48E-11	6.648766495	0.01701805	OK	3.75E-11

ANKRD28	0	58.13142569	0.1494219	OK	0
GALNT15	9.29E-106	21.81018415	0.05576915	OK	4.51E-105
DPH3	9.25E-189	29.28026874	0.07522787	OK	5.60E-188
OXNAD1	2.77E-17	8.374660126	0.02143944	OK	7.78E-17
RFTN1	3.58E-135	24.71619402	0.06347783	OK	1.90E-134
PLCL2	1.33E-10	6.317753982	0.01616286	OK	3.29E-10
TBC1D5	2.26E-15	7.839720985	0.02008561	OK	6.15E-15
SATB1	2.69E-99	21.11848176	0.05422347	OK	1.28E-98
RAB5A	1.89E-78	18.71445637	0.04805532	OK	8.23E-78
KAT2B	0.00018045	3.567143909	0.00909268	OK	0.00035934
SGO1	0	105.6889803	0.27042161	OK	0
ZNF385D	0	58.36735833	0.1499784	OK	0
UBE2E2	6.68E-51	14.96025724	0.03839957	OK	2.53E-50
UBE2E1	9.00E-109	22.12540148	0.05682647	OK	4.42E-108
NKIRAS1	1.18E-05	4.227418006	0.0107943	OK	2.49E-05
RPL15	0	191.132597	0.49142668	OK	0
NR1D2	4.58E-68	17.39400805	0.04465515	OK	1.90E-67
THRB	1.27E-198	30.04489755	0.07715278	OK	7.88E-198
RARB	3.52E-06	4.492455764	0.01138861	OK	7.59E-06
TOP2B	4.47E-05	3.917772288	0.01000032	OK	9.16E-05
NGLY1	2.46E-57	15.91588921	0.04085396	OK	9.65E-57
SLC4A7	8.49E-274	35.33509813	0.09080063	OK	5.92E-273
CMC1	3.06E-162	27.11757199	0.06966687	OK	1.74E-161
AZI2	5.31E-19	8.828287654	0.02262953	OK	1.53E-18
ZCWPW2	0.00015959	3.599210441	0.00914875	OK	0.00031885
RBMS3	0	75.4988054	0.19405762	OK	0
RBMS3-AS3	3.88E-16	8.057950331	0.02058421	OK	1.07E-15
LINC01985	1.19E-09	5.969033676	0.01487115	OK	2.89E-09
TGFBR2	4.28E-151	26.15539958	0.06719099	OK	2.37E-150
GADL1	0.00020342	3.535606475	0.00892073	OK	0.00040389
STT3B	1.21E-13	7.323240776	0.01875897	OK	3.20E-13
OSBPL10	2.43E-09	5.8517793	0.01494312	OK	5.83E-09
GPD1L	1.66E-21	9.452023953	0.02420114	OK	4.98E-21
CMTM8	6.89E-84	19.37018464	0.04964368	OK	3.08E-83
CMTM7	0	69.56266762	0.17881677	OK	0
CMTM6	0	127.8838927	0.32883312	OK	0
DYNC1LI1	1.75E-234	32.67532266	0.08396248	OK	1.16E-233
CCR4	3.63E-05	3.967629118	0.00989272	OK	7.48E-05
GLB1	0	40.19036503	0.10327857	OK	0
CRTAP	0	67.66699151	0.17395932	OK	0
SUSD5	0	80.69258128	0.20739319	OK	0
FBXL2	8.04E-16	7.96838718	0.02037904	OK	2.21E-15
PDCD6IP	8.03E-35	12.25364965	0.03144006	OK	2.73E-34
STAC	4.40E-10	6.129806926	0.01562664	OK	1.08E-09
TRANK1	0.00017169	3.580168984	0.00912167	OK	0.00034233
EPM2AIP1	1.00E-05	4.264315752	0.01089064	OK	2.12E-05
MLH1	3.64E-05	3.966667272	0.01012296	OK	7.50E-05
LRRFIP2	3.76E-138	24.99170544	0.06419953	OK	2.02E-137
GOLGA4	1.41E-47	14.44186237	0.03706896	OK	5.25E-47
ITGA9	1.61E-08	5.52871049	0.0141269	OK	3.78E-08
CTDSPL	0	64.81799983	0.16659396	OK	0
PLCD1	4.10E-43	13.71556148	0.03514785	OK	1.48E-42
ACAA1	0	46.29465072	0.11898783	OK	0

MYD88	0	58.03810577	0.14917182	OK	0
OXSR1	1.24E-153	26.37757973	0.06775503	OK	6.91E-153
WDR48	0.0001907	3.552627401	0.00905994	OK	0.00037932
CSRNP1	0	48.96007201	0.12583847	OK	0
CX3CR1	0	116.9326531	0.30055253	OK	0
SLC25A38	1.39E-29	11.23365951	0.02880488	OK	4.53E-29
RPSA	0	148.0577111	0.38069685	OK	0
MOBP	4.66E-07	4.905585798	0.01017003	OK	1.04E-06
MYRIP	7.24E-19	8.793583416	0.02144106	OK	2.08E-18
EIF1B	0	60.37915962	0.15521752	OK	0
ENTPD3	0	61.23962074	0.15633213	OK	0
RPL14	0	197.9648984	0.50898735	OK	0
ZNF620	7.98E-11	6.395883045	0.01634392	OK	1.99E-10
CTNNB1	0	37.93852152	0.0975005	OK	0
TRAK1	6.88E-13	7.086521336	0.01814146	OK	1.79E-12
SEC22C	8.57E-16	7.960528054	0.02039411	OK	2.35E-15
SS18L2	7.38E-282	35.85613299	0.09213854	OK	5.20E-281
NKTR	1.42E-46	14.28188569	0.03665521	OK	5.25E-46
ZBTB47	1.22E-07	5.161488865	0.01317934	OK	2.79E-07
HIGD1A	0	39.49793584	0.10150714	OK	0
KRBOX1	7.67E-16	7.974123018	0.02036878	OK	2.11E-15
FAM198A	0.00010998	3.694927902	0.00937564	OK	0.00022153
SNRK	8.40E-29	11.0738617	0.02839525	OK	2.72E-28
SNRK-AS1	9.06E-05	3.743841218	0.00916087	OK	0.00018327
ABHD5	2.13E-117	23.00390859	0.05908262	OK	1.07E-116
TCAIM	1.46E-13	7.297896595	0.01868903	OK	3.86E-13
ZKSCAN7	0.00015514	3.606556393	0.00908762	OK	0.00031022
ZNF660	2.19E-05	4.086833496	0.01041073	OK	4.56E-05
ZNF502	3.39E-06	4.500175	0.01146062	OK	7.32E-06
KIAA1143	3.17E-28	10.95416461	0.02809488	OK	1.02E-27
KIF15	0	70.83341467	0.18008578	OK	0
ZDHHC3	1.26E-55	15.66766585	0.04021678	OK	4.89E-55
EXOSC7	7.06E-65	16.96823477	0.04356125	OK	2.88E-64
CLEC3B	4.09E-34	12.12100118	0.03095261	OK	1.38E-33
CDCP1	0	39.16849797	0.10061681	OK	0
TMEM158	0	96.67654533	0.24841181	OK	0
SACM1L	1.00E-05	4.264014223	0.01088936	OK	2.12E-05
FYCO1	1.06E-75	18.37424186	0.04715436	OK	4.54E-75
CXCR6	2.02E-18	8.677854565	0.0221137	OK	5.77E-18
XCR1	0	92.37497773	0.23593428	OK	0
CCR1	0	85.89940227	0.22082467	OK	0
CCR2	8.44E-100	21.17316494	0.05423382	OK	4.02E-99
AC098613.1	5.34E-20	9.081867898	0.02323512	OK	1.56E-19
CCR5	1.02E-121	23.43147798	0.06013787	OK	5.21E-121
CCRL2	0	42.93133263	0.11030565	OK	0
PTH1R	3.53E-20	9.126802529	0.02329261	OK	1.04E-19
CCDC12	9.92E-84	19.35141371	0.04969358	OK	4.42E-83
NBEAL2	3.43E-05	3.980883016	0.0101397	OK	7.08E-05
KIF9	5.99E-06	4.377980728	0.01117821	OK	1.28E-05
KLHL18	4.37E-19	8.850107671	0.02267621	OK	1.26E-18
ELP6	1.39E-10	6.310502132	0.01615171	OK	3.45E-10
SMARCC1	2.42E-32	11.78185691	0.03022337	OK	8.08E-32
DHX30	2.38E-08	5.460201568	0.01396534	OK	5.54E-08

MAP4	0	70.73253389	0.18184272	OK	0
CDC25A	1.07E-121	23.42963387	0.05851871	OK	5.44E-121
CAMP	7.74E-20	9.041292608	0.02301046	OK	2.26E-19
NME6	2.47E-07	5.028793028	0.01285516	OK	5.56E-07
PLXNB1	5.42E-29	11.11303262	0.02842684	OK	1.76E-28
CCDC51	7.48E-07	4.811872781	0.01229117	OK	1.65E-06
TMA7	0	123.1503443	0.3166526	OK	0
SHISA5	2.72E-89	20.00059785	0.05136312	OK	1.24E-88
PFKFB4	0.00039665	3.355118936	0.00852758	OK	0.00077484
UQCRC1	0	38.63611883	0.09929344	OK	0
IP6K2	1.54E-13	7.290949921	0.01867387	OK	4.06E-13
PRKAR2A	1.14E-21	9.491706589	0.02433547	OK	3.41E-21
ARIH2	5.38E-10	6.097770409	0.01560682	OK	1.31E-09
P4HTM	5.82E-47	14.34390672	0.03680756	OK	2.16E-46
DALRD3	1.72E-16	8.156506614	0.02089781	OK	4.79E-16
NDUFAF3	0	53.63189733	0.13786378	OK	0
IMPDH2	5.07E-152	26.23666754	0.06740026	OK	2.82E-151
QARS	3.79E-120	23.27706404	0.05978359	OK	1.92E-119
LAMB2	0	41.83241591	0.10748537	OK	0
KLHDC8B	1.09E-160	26.98553451	0.06931536	OK	6.19E-160
USP4	2.85E-16	8.095593442	0.02074236	OK	7.89E-16
RHOA	0	158.8330243	0.40841568	OK	0
TCTA	1.18E-22	9.724555795	0.0249286	OK	3.60E-22
DAG1	9.65E-88	19.8218317	0.05086785	OK	4.38E-87
APEH	9.86E-24	9.974492209	0.02557211	OK	3.04E-23
GMPPB	3.36E-39	13.04569231	0.03346439	OK	1.18E-38
IP6K1	0.00028621	3.444353706	0.00878062	OK	0.00056329
INKA1	2.46E-09	5.849759428	0.01494396	OK	5.90E-09
TRAIP	1.49E-09	5.932501184	0.01503834	OK	3.60E-09
RBM6	1.61E-06	4.656235537	0.01190037	OK	3.52E-06
RBM5	2.92E-08	5.423671743	0.01387236	OK	6.79E-08
SEMA3F	1.65E-06	4.651729884	0.0118069	OK	3.59E-06
GNAI2	0	149.3476181	0.38403365	OK	0
SEMA3B	2.74E-73	18.07002481	0.04633086	OK	1.16E-72
LSMEM2	1.33E-55	15.66436595	0.03985809	OK	5.15E-55
IFRD2	1.09E-19	9.003858542	0.02307865	OK	3.17E-19
NAA80	4.26E-06	4.451656734	0.01135877	OK	9.14E-06
HYAL2	1.11E-194	29.74166816	0.07638505	OK	6.82E-194
TUSC2	1.37E-96	20.82208105	0.05347454	OK	6.42E-96
RASSF1	1.15E-100	21.26680816	0.05461617	OK	5.50E-100
RASSF1-AS1	0.00043561	3.329118618	0.0084601	OK	0.00084874
CYB561D2	0	54.99369505	0.14134866	OK	0
TMEM115	1.42E-14	7.605807031	0.0194822	OK	3.80E-14
CYB561D2.1	1.68E-16	8.1595445	0.02081865	OK	4.67E-16
C3orf18	2.78E-06	4.542717504	0.01157316	OK	6.01E-06
CISH	2.74E-12	6.89279696	0.01763408	OK	7.04E-12
MAPKAPK3	0	59.42375555	0.15273874	OK	0
MANF	1.43E-200	30.19379091	0.07757853	OK	8.87E-200
RBM15B	0.00040027	3.352606459	0.00854501	OK	0.00078163
TEX264	0	39.60323313	0.10177924	OK	0
RRP9	1.64E-06	4.652046384	0.01188279	OK	3.59E-06
PCBP4	5.89E-148	25.87797478	0.06643163	OK	3.24E-147
ABHD14B	4.19E-68	17.39912528	0.04467067	OK	1.74E-67

ABHD14A	3.52E-49	14.69403064	0.03771007	OK	1.32E-48
RPL29	0	193.6524648	0.49790707	OK	0
POC1A	0	42.47149528	0.10880132	OK	0
ALAS1	3.90E-20	9.115826039	0.02336186	OK	1.15E-19
TWF2	0	114.9028631	0.2954482	OK	0
PPM1M	3.76E-70	17.66706475	0.0453527	OK	1.57E-69
WDR82	5.88E-05	3.850875765	0.0098284	OK	0.00012006
GLYCTK	1.37E-12	6.990276513	0.01788056	OK	3.56E-12
SEMA3G	8.86E-21	9.275210497	0.02066698	OK	2.63E-20
TNNC1	2.50E-20	9.163879336	0.02339198	OK	7.39E-20
NISCH	3.37E-64	16.8761229	0.04331624	OK	1.37E-63
STAB1	0	146.900605	0.37768939	OK	0
NT5DC2	1.65E-294	36.65894549	0.09418328	OK	1.18E-293
SMIM4	6.03E-183	28.81995805	0.07404396	OK	3.60E-182
PBRM1	3.26E-66	17.1477984	0.04402619	OK	1.34E-65
GNL3	5.07E-76	18.41397474	0.04727867	OK	2.18E-75
GLT8D1	1.59E-13	7.28673959	0.01866218	OK	4.19E-13
SPCS1	0	95.04478171	0.24437338	OK	0
ITIH3	1.10E-150	26.11929742	0.06690598	OK	6.09E-150
AC006254.1	5.79E-41	13.35174	0.03399663	OK	2.06E-40
MUSTN1	0.00036167	3.380574912	0.00835936	OK	0.00070813
SFMBT1	3.84E-08	5.37443632	0.01372875	OK	8.89E-08
RFT1	2.81E-07	5.003644717	0.01278527	OK	6.32E-07
PRKCD	2.15E-92	20.35376025	0.05225737	OK	9.97E-92
TKT	0	104.1340319	0.26775279	OK	0
SELENOK	0	101.5138842	0.26101133	OK	0
CACNA2D3	0	50.69650599	0.13022576	OK	0
CCDC66	8.43E-10	6.025534051	0.01542111	OK	2.05E-09
ARHGEF3	2.40E-14	7.537211806	0.01928568	OK	6.43E-14
IL17RD	1.46E-05	4.180272323	0.01052394	OK	3.06E-05
HESX1	9.36E-05	3.735730108	0.00949746	OK	0.00018917
APPL1	0	44.04475555	0.11320539	OK	0
ARF4	0	47.0199898	0.12085825	OK	0
SLMAP	0	71.17716866	0.18296506	OK	0
FLNB	9.04E-74	18.13118808	0.04652439	OK	3.84E-73
DNASE1L3	0	96.44780326	0.24744239	OK	0
ABHD6	4.96E-20	9.089750184	0.02328279	OK	1.46E-19
PXK	7.73E-23	9.767965349	0.02504208	OK	2.36E-22
PDHB	1.55E-117	23.01779054	0.05912314	OK	7.80E-117
AC135507.1	7.45E-06	4.330097607	0.01076321	OK	1.58E-05
ACOX2	1.48E-08	5.544360436	0.01416142	OK	3.46E-08
FAM107A	1.10E-38	12.95507566	0.033081	OK	3.84E-38
FAM3D	2.26E-06	4.585852742	0.01131404	OK	4.91E-06
FHIT	1.13E-58	16.10726842	0.04131978	OK	4.49E-58
PTPRG	6.73E-183	28.8161311	0.07399689	OK	4.02E-182
C3orf14	9.73E-23	9.74452558	0.02497941	OK	2.97E-22
THOC7	0	41.63353197	0.10700384	OK	0
PSMD6	1.38E-127	24.00036427	0.06165053	OK	7.16E-127
AC092040.1	0.00022975	3.50331594	0.00888135	OK	0.00045486
PRICKLE2	4.18E-20	9.108395521	0.02328142	OK	1.23E-19
ADAMTS9	5.86E-30	11.30998651	0.02889889	OK	1.91E-29
ADAMTS9-AS2	3.06E-58	16.04575657	0.04113165	OK	1.21E-57
MAGI1	3.72E-24	10.07080525	0.02577895	OK	1.15E-23

SLC25A26	3.25E-11	6.531597454	0.01672015	OK	8.18E-11
LRIG1	2.38E-71	17.82209851	0.0457204	OK	1.00E-70
KBTBD8	1.03E-293	36.60885204	0.09404713	OK	7.41E-293
SUCLG2	5.41E-96	20.7559886	0.05330418	OK	2.54E-95
EOGT	1.29E-16	8.19148388	0.02097991	OK	3.59E-16
TMF1	2.73E-67	17.29145648	0.04439679	OK	1.13E-66
UBA3	5.75E-20	9.073749942	0.02325892	OK	1.68E-19
ARL6IP5	0	133.8464944	0.34416163	OK	0
FRMD4B	0	132.5362289	0.34078837	OK	0
MITF	0	63.50074903	0.16322468	OK	0
SAMMSON	1.31E-15	7.90767532	0.02018246	OK	3.59E-15
FOXP1	0	39.61572253	0.10181258	OK	0
EIF4E3	8.77E-08	5.223631441	0.01334734	OK	2.01E-07
PROK2	1.14E-08	5.589591675	0.01412867	OK	2.68E-08
LINC00877	5.38E-44	13.86208314	0.03550063	OK	1.95E-43
RYBP	2.92E-27	10.75123764	0.02757184	OK	9.32E-27
GXYLT2	1.55E-203	30.41883241	0.07807898	OK	9.67E-203
PPP4R2	6.24E-91	20.1880192	0.05184558	OK	2.87E-90
PDZRN3	8.05E-72	17.88265113	0.04584041	OK	3.39E-71
CNTN3	7.69E-13	7.071015935	0.01802917	OK	2.00E-12
ROBO2	3.84E-10	6.151389129	0.01563802	OK	9.41E-10
ROBO1	3.16E-16	8.083146045	0.02067776	OK	8.73E-16
GBE1	5.16E-170	27.76882623	0.07132923	OK	3.00E-169
VGLL3	3.84E-81	19.04180083	0.04883629	OK	1.69E-80
CHMP2B	3.86E-262	34.56771013	0.0888309	OK	2.65E-261
CGGBP1	1.25E-40	13.29443085	0.03411683	OK	4.43E-40
C3orf38	9.25E-16	7.950971194	0.02037138	OK	2.54E-15
EPHA3	2.42E-53	15.32985253	0.03926265	OK	9.26E-53
PROS1	5.11E-272	35.21905142	0.09047977	OK	3.56E-271
ARL13B	5.28E-07	4.88077544	0.01247475	OK	1.18E-06
MTRNR2L12	0	62.59182643	0.16090143	OK	0
ARL6	3.75E-07	4.948042852	0.01261996	OK	8.39E-07
RIOX2	0.00043291	3.330848432	0.00848592	OK	0.00084393
CLDND1	6.45E-114	22.65347079	0.05818656	OK	3.21E-113
ST3GAL6-AS1	6.77E-20	9.056013994	0.02307757	OK	1.98E-19
ST3GAL6	0	39.0615512	0.10036376	OK	0
DCBLD2	5.99E-58	16.00403816	0.04106388	OK	2.36E-57
COL8A1	0	149.6057715	0.3846386	OK	0
CMSS1	7.77E-54	15.40332659	0.03953101	OK	2.99E-53
FILIP1L	0	135.374127	0.34809929	OK	0
TBC1D23	7.16E-05	3.80263517	0.0097034	OK	0.00014552
NIT2	4.14E-76	18.42502832	0.04730837	OK	1.78E-75
TOMM70	2.50E-16	8.111488367	0.02078384	OK	6.93E-16
LNP1	1.05E-184	28.95993581	0.07432902	OK	6.31E-184
TMEM45A	9.91E-212	31.03222366	0.07969218	OK	6.31E-211
TFG	1.14E-73	18.11842901	0.04652355	OK	4.84E-73
ABI3BP	0	57.4606992	0.14764035	OK	0
SENP7	6.18E-09	5.694569721	0.0145683	OK	1.46E-08
TRMT10C	7.41E-93	20.40589275	0.05240578	OK	3.44E-92
PCNP	0	51.76154664	0.13305095	OK	0
ZBTB11	6.38E-23	9.787292292	0.02509136	OK	1.95E-22
RPL24	0	176.5789206	0.45401304	OK	0
NFKBIZ	0	41.08425604	0.1055823	OK	0

ALCAM	1.79E-207	30.715043	0.0789198	OK	1.13E-206
CBLB	1.16E-22	9.726633725	0.02492605	OK	3.53E-22
DUBR	6.63E-42	13.51210797	0.03462901	OK	2.38E-41
AC063944.3	2.20E-05	4.085097699	0.00938405	OK	4.59E-05
BBX	0	41.18411545	0.10584566	OK	0
CD47	0	41.64940832	0.10704505	OK	0
IFT57	9.51E-142	25.32047191	0.06503877	OK	5.15E-141
MYH15	1.00E-44	13.98207862	0.03315823	OK	3.66E-44
CIP2A	1.92E-39	13.08820866	0.03351062	OK	6.77E-39
TRAT1	1.74E-91	20.25094342	0.05186864	OK	8.04E-91
NECTIN3	3.67E-58	16.0343925	0.04112827	OK	1.45E-57
CD96	4.03E-103	21.53054063	0.05515552	OK	1.94E-102
PHLDB2	0	55.57553165	0.14281782	OK	0
ABHD10	2.69E-06	4.549548536	0.0116183	OK	5.82E-06
GCSAM	0	58.12463463	0.14848589	OK	0
CD200	3.74E-268	34.96572094	0.08979599	OK	2.59E-267
BTLA	5.33E-202	30.30237807	0.07736387	OK	3.32E-201
ATG3	0	102.8919335	0.26455641	OK	0
CCDC80	0	125.3186169	0.32216948	OK	0
CD200R1	2.51E-84	19.42209299	0.04979935	OK	1.12E-83
GTPBP8	3.36E-10	6.172642527	0.01579593	OK	8.24E-10
BOC	1.52E-125	23.80402432	0.0608776	OK	7.83E-125
CFAP44	9.50E-07	4.7637747	0.01209869	OK	2.09E-06
SIDT1	1.61E-59	16.22752918	0.04158691	OK	6.42E-59
NAA50	2.88E-263	34.64270365	0.0890169	OK	1.98E-262
ATP6V1A	0	39.21708881	0.10077562	OK	0
QTRT2	4.96E-21	9.336854835	0.02392906	OK	1.48E-20
AC093010.2	6.42E-07	4.842336566	0.01198898	OK	1.42E-06
TIGIT	1.85E-20	9.196249302	0.02345153	OK	5.48E-20
ZBTB20	0	63.40266708	0.16298185	OK	0
ZBTB20-AS2	0.0001786	3.56983627	0.00905558	OK	0.00035583
GAP43	7.51E-84	19.36575406	0.04959433	OK	3.35E-83
LSAMP	7.03E-29	11.08982957	0.02834254	OK	2.28E-28
B4GALT4	7.53E-06	4.327811308	0.01105208	OK	1.60E-05
ARHGAP31	1.60E-09	5.92048969	0.01513933	OK	3.86E-09
TMEM39A	7.01E-07	4.82458752	0.01232673	OK	1.55E-06
POGLUT1	4.88E-05	3.896698124	0.00994371	OK	9.98E-05
TIMMDC1	9.58E-78	18.62769679	0.04783217	OK	4.16E-77
CD80	5.91E-41	13.35011536	0.03421157	OK	2.11E-40
AC073352.2	1.63E-39	13.10055686	0.03353244	OK	5.76E-39
ADPRH	2.41E-40	13.24514212	0.03396566	OK	8.53E-40
PLA1A	5.74E-11	6.446145168	0.01641384	OK	1.43E-10
POPDC2	2.44E-11	6.574469594	0.01678373	OK	6.16E-11
COX17	0	60.34628596	0.15513288	OK	0
LRRC58	5.50E-13	7.117403212	0.01822737	OK	1.44E-12
FSTL1	0	106.3473275	0.27338891	OK	0
NDUFB4	0	88.94681084	0.22869079	OK	0
POLQ	1.78E-165	27.39052315	0.06934101	OK	1.02E-164
HCLS1	0	194.3537741	0.49978511	OK	0
GOLGB1	9.11E-26	10.42926617	0.02674902	OK	2.87E-25
IQCB1	2.70E-05	4.037763294	0.0103069	OK	5.59E-05
EAF2	0	72.17429535	0.18552245	OK	0
ILDR1	0.00010213	3.713696336	0.00923775	OK	0.00020604

CD86	0	187.5279381	0.48222409	OK	0
CSTA	0	118.7221336	0.30524061	OK	0
CCDC58	1.57E-13	7.288501364	0.01866236	OK	4.14E-13
FAM162A	0	65.47505378	0.16832178	OK	0
KPNA1	5.74E-06	4.387233611	0.0112065	OK	1.22E-05
PARP9	1.73E-55	15.64752712	0.04016292	OK	6.70E-55
DTX3L	1.40E-42	13.62637121	0.03496124	OK	5.03E-42
PARP15	3.19E-06	4.51326936	0.01149565	OK	6.89E-06
PARP14	2.06E-203	30.4094942	0.07813268	OK	1.28E-202
HSPBAP1	1.26E-81	19.09992087	0.04901544	OK	5.58E-81
DIRC2	5.37E-287	36.18430724	0.0929604	OK	3.81E-286
LINC02035	2.93E-05	4.018296807	0.01022256	OK	6.06E-05
SEMA5B	6.11E-17	8.280921799	0.02091738	OK	1.71E-16
PDIA5	7.46E-14	7.387848046	0.01891261	OK	1.98E-13
SEC22A	0.00015181	3.612189154	0.0092071	OK	0.00030375
ADCY5	8.01E-127	23.92703507	0.06138245	OK	4.15E-126
HACD2	4.05E-14	7.468612396	0.01912807	OK	1.08E-13
MYLK	0	198.3031208	0.50992382	OK	0
CCDC14	1.12E-08	5.592284511	0.01430432	OK	2.64E-08
KALRN	2.44E-224	31.95322831	0.08205815	OK	1.59E-223
UMPS	5.32E-05	3.875490641	0.009888	OK	0.00010878
ITGB5	0	52.77682183	0.13565211	OK	0
HEG1	7.42E-100	21.17922623	0.05434844	OK	3.53E-99
SLC12A8	1.39E-07	5.137960151	0.01299325	OK	3.16E-07
ZNF148	8.00E-27	10.65801087	0.02733505	OK	2.54E-26
SNX4	3.14E-65	17.01573053	0.0436833	OK	1.28E-64
OSBPL11	5.95E-266	34.82052568	0.08946438	OK	4.11E-265
SLC41A3	4.23E-105	21.74064601	0.05582313	OK	2.05E-104
ALDH1L1	6.13E-16	8.001718516	0.02042839	OK	1.69E-15
KLF15	1.17E-148	25.94040691	0.06656435	OK	6.42E-148
CHST13	8.33E-128	24.02129051	0.06164959	OK	4.33E-127
CHCHD6	2.61E-107	21.97298686	0.05641636	OK	1.28E-106
PLXNA1	3.10E-10	6.185180305	0.01581651	OK	7.61E-10
TPRA1	1.70E-31	11.61643446	0.0297951	OK	5.62E-31
MCM2	2.13E-63	16.76700529	0.04282339	OK	8.63E-63
PODXL2	1.63E-85	19.56197965	0.05013083	OK	7.33E-85
ABTB1	3.43E-40	13.2186295	0.03391944	OK	1.21E-39
MGLL	0	92.78851128	0.23856434	OK	0
KBTBD12	0.00020228	3.537086749	0.00894922	OK	0.00040167
SEC61A1	3.70E-37	12.68239634	0.03253742	OK	1.28E-36
RUVBL1	2.76E-58	16.05211415	0.0412016	OK	1.09E-57
EEFSEC	8.23E-08	5.235452231	0.01338484	OK	1.88E-07
GATA2	5.97E-32	11.70556149	0.02992773	OK	1.98E-31
GATA2-AS1	4.62E-13	7.14147878	0.01822334	OK	1.21E-12
RPN1	0	47.57852885	0.12229052	OK	0
RAB7A	0	80.83138307	0.20781976	OK	0
ACAD9	2.94E-05	4.017674916	0.01025387	OK	6.08E-05
ISY1	2.22E-12	6.922202771	0.01772407	OK	5.74E-12
CNBP	0	105.8140349	0.27206918	OK	0
COPG1	1.41E-06	4.68345994	0.01196649	OK	3.09E-06
HMCES	1.46E-25	10.38420492	0.02662391	OK	4.59E-25
H1FX	0	42.7948296	0.10999142	OK	0
MBD4	6.99E-109	22.13678364	0.05685722	OK	3.44E-108

PLXND1	2.10E-256	34.18388924	0.08782862	OK	1.43E-255
TMCC1	0.00012854	3.655108391	0.00932155	OK	0.00025802
ATP2C1	1.47E-39	13.10861684	0.03363157	OK	5.18E-39
NEK11	0.00013776	3.637293013	0.00925575	OK	0.00027611
NUDT16	6.94E-194	29.68010103	0.07625578	OK	4.25E-193
MRPL3	1.75E-191	29.49337985	0.07577762	OK	1.07E-190
AC107027.1	6.12E-06	4.373403739	0.01080844	OK	1.30E-05
CPNE4	7.51E-44	13.83808825	0.03530831	OK	2.73E-43
ACPP	3.14E-81	19.05244436	0.04882164	OK	1.38E-80
DNAJC13	5.90E-11	6.442003544	0.01648918	OK	1.47E-10
ACKR4	0	46.26124002	0.1187738	OK	0
UBA5	7.74E-07	4.804933512	0.0122803	OK	1.71E-06
NPHP3	1.19E-14	7.6277761	0.01952865	OK	3.21E-14
CDV3	0	136.5896324	0.35122635	OK	0
TOPBP1	2.60E-21	9.404901281	0.02410364	OK	7.78E-21
SRPRB	2.10E-35	12.36206135	0.0317138	OK	7.16E-35
RAB6B	3.13E-83	19.29214375	0.04941839	OK	1.39E-82
SLCO2A1	0	78.84770323	0.19882813	OK	0
RYK	2.08E-27	10.78266962	0.02765165	OK	6.63E-27
AMOTL2	4.64E-77	18.54308606	0.04755709	OK	2.01E-76
ANAPC13	5.05E-50	14.82493183	0.03805184	OK	1.91E-49
CEP63	6.36E-13	7.097369088	0.01817741	OK	1.66E-12
PPP2R3A	1.31E-07	5.14835913	0.01315039	OK	2.99E-07
PCCB	2.90E-08	5.425028683	0.01387186	OK	6.74E-08
STAG1	0.00036078	3.381253574	0.00862019	OK	0.00070645
SLC35G2	7.38E-05	3.795094941	0.00965034	OK	0.00014991
AC096992.2	0.00048294	3.300283565	0.0083774	OK	0.00093756
NCK1-DT	0	38.33068596	0.09846666	OK	0
NCK1	6.19E-43	13.68560729	0.03511922	OK	2.24E-42
MRAS	1.60E-258	34.32621099	0.08819351	OK	1.09E-257
CEP70	6.93E-46	14.17100108	0.03632944	OK	2.55E-45
FAIM	6.73E-166	27.42595415	0.07044222	OK	3.87E-165
PIK3CB	1.45E-58	16.09218764	0.04129745	OK	5.73E-58
MRPS22	1.91E-43	13.77073989	0.03533954	OK	6.92E-43
COPB2	6.23E-116	22.85694556	0.0587105	OK	3.13E-115
RBP1	0	52.10689652	0.13389772	OK	0
CLSTN2	3.03E-07	4.989424718	0.01253674	OK	6.80E-07
SLC25A36	4.58E-08	5.342663374	0.013666	OK	1.06E-07
ZBTB38	1.03E-290	36.419798	0.09359135	OK	7.39E-290
RASA2	3.53E-05	3.97420212	0.01014439	OK	7.28E-05
RNF7	0	87.15037796	0.22407144	OK	0
ATP1B3	0	114.0468458	0.29324408	OK	0
TFDP2	1.30E-218	31.53825531	0.08102605	OK	8.36E-218
GK5	2.88E-12	6.885551786	0.01761621	OK	7.41E-12
XRN1	2.33E-06	4.579734839	0.01170324	OK	5.05E-06
TRPC1	1.03E-48	14.62116989	0.03747218	OK	3.85E-48
PCOLCE2	0	45.8176119	0.11771133	OK	0
U2SURP	1.04E-17	8.489628433	0.02176054	OK	2.94E-17
CHST2	4.10E-98	20.98933855	0.05383826	OK	1.94E-97
SLC9A9	0	47.1438796	0.1211412	OK	0
C3orf58	7.20E-58	15.99255381	0.04105271	OK	2.84E-57
PLOD2	4.15E-45	14.04466837	0.03602296	OK	1.52E-44
PLSCR4	0	60.13029042	0.1545197	OK	0

PLSCR1	0	133.8906174	0.34428382	OK	0
AGTR1	5.17E-46	14.19149009	0.03624917	OK	1.90E-45
CPA3	2.64E-56	15.76659006	0.03955797	OK	1.03E-55
GYG1	5.80E-218	31.49084116	0.0809122	OK	3.72E-217
HLTF	3.46E-103	21.53761043	0.05530987	OK	1.66E-102
HPS3	1.29E-116	22.92569529	0.0588745	OK	6.48E-116
CP	0	56.67388582	0.14560956	OK	0
TM4SF18	0	111.0811524	0.2817143	OK	0
TM4SF1	0	139.0718066	0.3575668	OK	0
WWTR1	0	77.15431564	0.19831824	OK	0
COMMD2	9.93E-61	16.39771882	0.04209674	OK	3.98E-60
RNF13	0	103.9343534	0.26723868	OK	0
PFN2	0	169.0917633	0.43476381	OK	0
TSC22D2	1.38E-106	21.89720671	0.05623633	OK	6.72E-106
SERP1	0	136.0173241	0.34975342	OK	0
EIF2A	8.15E-181	28.64943366	0.07360886	OK	4.84E-180
SELENOT	0	71.14436132	0.18290486	OK	0
SIAH2	2.90E-190	29.3981945	0.07552586	OK	1.76E-189
GPR171	4.10E-29	11.13801254	0.02845127	OK	1.33E-28
P2RY14	0	51.18835894	0.13145063	OK	0
P2RY13	0	165.6692884	0.42595256	OK	0
P2RY12	1.73E-43	13.77780397	0.03527064	OK	6.28E-43
IGSF10	4.51E-07	4.911888619	0.0125255	OK	1.01E-06
SUCNR1	6.38E-291	36.43307565	0.09357563	OK	4.55E-290
MBNL1	2.35E-107	21.97768458	0.05645069	OK	1.15E-106
MBNL1-AS1	0	91.69102977	0.23570134	OK	0
P2RY1	6.47E-11	6.427793282	0.01643173	OK	1.62E-10
RAP2B	0	109.9352465	0.28266472	OK	0
ARHGEF26	2.42E-91	20.23467622	0.05191737	OK	1.12E-90
DHX36	4.78E-15	7.744914314	0.0198451	OK	1.30E-14
MME	2.27E-142	25.37681832	0.06505855	OK	1.23E-141
GMPS	6.21E-29	11.10085678	0.02847233	OK	2.01E-28
KCNAB1	0	94.79364262	0.24363528	OK	0
SSR3	0	89.54582182	0.23023257	OK	0
TIPARP	0	96.77587343	0.24882073	OK	0
CCNL1	0	41.69220748	0.10715423	OK	0
PTX3	7.94E-67	17.22978317	0.0441808	OK	3.27E-66
SHOX2	3.26E-34	12.13958684	0.03109481	OK	1.10E-33
RSRC1	3.79E-38	12.85971593	0.03299873	OK	1.32E-37
MLF1	3.92E-18	8.60203372	0.02203907	OK	1.12E-17
GFM1	4.42E-07	4.915807495	0.01256449	OK	9.86E-07
LXN	2.48E-282	35.88652764	0.09220299	OK	1.75E-281
RARRES1	0	48.86776385	0.12554779	OK	0
MFSD1	0	179.9153461	0.46265384	OK	0
AC080013.5	0.00014855	3.617810705	0.00920069	OK	0.00029728
C3orf80	1.63E-11	6.634636671	0.01622838	OK	4.12E-11
SMC4	2.91E-205	30.54908731	0.07847795	OK	1.82E-204
TRIM59	0.00025991	3.470329177	0.00881844	OK	0.00051313
KPNA4	8.43E-100	21.17320204	0.05438022	OK	4.02E-99
PPM1L	3.68E-14	7.481224084	0.01915325	OK	9.84E-14
B3GALNT1	3.14E-07	4.982645196	0.01272901	OK	7.04E-07
NMD3	4.72E-12	6.814721043	0.01744894	OK	1.21E-11
SPTSSB	1.22E-05	4.220842471	0.01047781	OK	2.57E-05

PDCD10	5.56E-283	35.92810362	0.09232972	OK	3.93E-282
SERPINI1	0	79.40245632	0.20399622	OK	0
GOLIM4	0	42.54333775	0.10934224	OK	0
MECOM	0	44.45439054	0.1141626	OK	0
LRRC34	1.77E-05	4.135030983	0.01051466	OK	3.71E-05
SEC62	0	96.56577934	0.24828133	OK	0
GPR160	2.34E-128	24.07404044	0.06179434	OK	1.22E-127
PRKCI	1.33E-06	4.695406233	0.01199601	OK	2.91E-06
SKIL	0	44.41959634	0.11416742	OK	0
CLDN11	3.18E-12	6.871537105	0.01746133	OK	8.16E-12
RPL22L1	0	57.16418462	0.14694657	OK	0
EIF5A2	2.88E-15	7.808971362	0.01999654	OK	7.84E-15
FNDC3B	7.43E-159	26.82892515	0.06892033	OK	4.20E-158
TNFSF10	0	122.9937079	0.31621993	OK	0
NCEH1	0	51.8235076	0.13317948	OK	0
ECT2	3.57E-68	17.40826338	0.04463562	OK	1.48E-67
NLGN1	4.37E-37	12.66933707	0.03242806	OK	1.51E-36
NAALADL2	1.01E-65	17.08180098	0.04380238	OK	4.16E-65
TBL1XR1	1.42E-39	13.11105134	0.03364488	OK	5.02E-39
ZMAT3	1.72E-33	12.0028392	0.03078637	OK	5.79E-33
PIK3CA	3.81E-13	7.167676865	0.01835516	OK	1.00E-12
ZNF639	3.35E-06	4.502880158	0.01150302	OK	7.23E-06
MFN1	4.21E-08	5.357998822	0.01370123	OK	9.72E-08
GNB4	1.21E-240	33.10618731	0.08506855	OK	8.07E-240
ACTL6A	1.88E-43	13.77208893	0.03533883	OK	6.80E-43
MRPL47	2.29E-133	24.54772188	0.06305698	OK	1.21E-132
NDUFB5	0	62.62039088	0.16098154	OK	0
TTC14	5.90E-05	3.850270281	0.00982723	OK	0.00012034
AC108734.4	5.46E-103	21.5164516	0.05418915	OK	2.62E-102
FXR1	4.01E-105	21.74311236	0.0558469	OK	1.94E-104
DNAJC19	0	38.63877907	0.09929922	OK	0
DCUN1D1	2.45E-15	7.82956517	0.02005941	OK	6.66E-15
MCCC1	1.03E-06	4.748353543	0.01212977	OK	2.25E-06
LAMP3	0	38.27073447	0.09760658	OK	0
MCF2L2	5.47E-10	6.09519648	0.01554171	OK	1.33E-09
B3GNT5	0	73.03618893	0.18773869	OK	0
KLHL6	1.75E-165	27.39116842	0.07034157	OK	1.00E-164
KLHL24	2.51E-19	8.911608624	0.02284232	OK	7.29E-19
PARL	1.87E-222	31.81715626	0.08175375	OK	1.21E-221
ABCC5	6.08E-117	22.95832703	0.05894887	OK	3.06E-116
EIF2B5	5.84E-06	4.383634455	0.01119659	OK	1.24E-05
AP2M1	0	63.475	0.1631801	OK	0
ABCF3	6.68E-08	5.273726829	0.01348191	OK	1.54E-07
ALG3	2.10E-104	21.66703843	0.055644	OK	1.01E-103
EEF1AKMT4	1.01E-33	12.04645234	0.03087449	OK	3.42E-33
PSMD2	1.75E-155	26.53830953	0.06817824	OK	9.84E-155
EIF4G1	9.85E-48	14.46658613	0.03712907	OK	3.67E-47
FAM131A	0.00020973	3.527530944	0.00899299	OK	0.00041615
POLR2H	1.26E-57	15.95777365	0.04096283	OK	4.95E-57
CHRD	5.61E-29	11.10996378	0.02837587	OK	1.82E-28
MAGEF1	2.83E-180	28.6059995	0.07348111	OK	1.68E-179
VPS8	2.32E-05	4.073089726	0.01039627	OK	4.82E-05
C3orf70	7.90E-148	25.86668762	0.06636297	OK	4.33E-147

MAP3K13	1.90E-70	17.70549416	0.04545974	OK	7.96E-70
TMEM41A	0.00017645	3.573013776	0.00911077	OK	0.00035173
LIPH	1.12E-19	9.001046317	0.02255382	OK	3.26E-19
IGF2BP2	2.68E-64	16.88961612	0.04331149	OK	1.09E-63
TRA2B	4.98E-77	18.53924618	0.04760688	OK	2.15E-76
ETV5	0	72.9786699	0.18759359	OK	0
DGKG	8.05E-49	14.63787941	0.03748743	OK	3.02E-48
DNAJB11	1.24E-287	36.22480972	0.09308433	OK	8.79E-287
EIF4A2	0	79.03654231	0.20320252	OK	0
RFC4	1.15E-59	16.24830948	0.04168494	OK	4.58E-59
ST6GAL1	1.43E-222	31.82560027	0.08174653	OK	9.28E-222
RPL39L	5.87E-148	25.87815214	0.06646457	OK	3.22E-147
MASP1	1.20E-19	8.993507087	0.02288133	OK	3.48E-19
BCL6	3.95E-39	13.03342684	0.03344051	OK	1.39E-38
LPP	0	136.0853899	0.34992404	OK	0
TPRG1	2.99E-10	6.191046884	0.0158107	OK	7.34E-10
P3H2	7.51E-33	11.8801117	0.03044499	OK	2.52E-32
CLDN1	0	53.7465178	0.13806165	OK	0
CLDN16	3.81E-06	4.475766767	0.0110294	OK	8.19E-06
IL1RAP	1.92E-143	25.47383808	0.06539926	OK	1.05E-142
CCDC50	2.29E-195	29.79469382	0.07655099	OK	1.41E-194
MB21D2	1.83E-51	15.04627824	0.03858572	OK	6.94E-51
OPA1	4.22E-07	4.924966797	0.01258828	OK	9.42E-07
HES1	1.21E-269	35.06354894	0.0900954	OK	8.41E-269
ATP13A3	0	48.81870726	0.12547513	OK	0
LINC00884	1.10E-23	9.963946198	0.02551938	OK	3.38E-23
LSG1	4.16E-05	3.935244134	0.01004348	OK	8.54E-05
FAM43A	4.53E-08	5.344531998	0.01364465	OK	1.05E-07
XXYLT1	5.60E-09	5.711542591	0.01459751	OK	1.33E-08
ACAP2	6.41E-221	31.70605649	0.08146949	OK	4.15E-220
PPP1R2	0	42.24773856	0.10858331	OK	0
APOD	0	83.34615198	0.21355303	OK	0
MUC20-OT1	1.13E-24	10.18739488	0.0261221	OK	3.51E-24
TFRC	0	113.0322319	0.29061103	OK	0
ZDHHC19	2.98E-15	7.804602562	0.01990275	OK	8.12E-15
PCYT1A	8.71E-22	9.51939219	0.02440266	OK	2.62E-21
TCTEX1D2	8.83E-10	6.017954196	0.01538964	OK	2.14E-09
TM4SF19-TCTEX	1.68E-11	6.629912767	0.01654674	OK	4.25E-11
TM4SF19	0	84.05242925	0.21586324	OK	0
RNF168	4.39E-69	17.52788937	0.04500182	OK	1.83E-68
FBXO45	1.44E-08	5.548389747	0.01418631	OK	3.38E-08
NRROS	3.20E-252	33.90118472	0.08708272	OK	2.17E-251
PIGX	1.16E-13	7.32884583	0.0187689	OK	3.07E-13
PAK2	0	94.1458633	0.24206377	OK	0
NCBP2	3.30E-53	15.30951261	0.03929778	OK	1.26E-52
NCBP2-AS2	1.07E-96	20.83386305	0.0535038	OK	5.02E-96
MELTF	9.37E-18	8.501291615	0.02173997	OK	2.66E-17
MELTF-AS1	3.11E-11	6.538139114	0.01668606	OK	7.83E-11
BDH1	1.05E-05	4.253977445	0.01079424	OK	2.22E-05
FYTTD1	1.68E-77	18.59761798	0.0477552	OK	7.28E-77
IQCG	6.18E-13	7.101356991	0.01817723	OK	1.61E-12
RPL35A	0	186.8970933	0.48053301	OK	0
LMLN	3.71E-05	3.96263623	0.01009028	OK	7.63E-05

ZNF595	0.00011546	3.682544578	0.00937797	OK	0.00023232
ZNF141	1.20E-07	5.164954682	0.01320348	OK	2.74E-07
PDE6B	1.98E-13	7.256756936	0.01855838	OK	5.22E-13
ATP5ME	0	79.54962974	0.20452202	OK	0
MYL5	1.55E-09	5.925747531	0.015154	OK	3.75E-09
SLC49A3	4.40E-140	25.16877914	0.06463239	OK	2.38E-139
CPLX1	2.22E-73	18.0816762	0.04552078	OK	9.42E-73
GAK	4.59E-14	7.452058921	0.01908419	OK	1.22E-13
TMEM175	1.44E-10	6.305219039	0.01613565	OK	3.56E-10
IDUA	3.87E-06	4.472477473	0.01141853	OK	8.32E-06
FGFRL1	2.60E-08	5.444009847	0.01390279	OK	6.07E-08
SPON2	3.03E-10	6.188808184	0.01576091	OK	7.44E-10
CTBP1	6.37E-12	6.771621443	0.01733848	OK	1.63E-11
MAEA	4.55E-20	9.099291277	0.02332268	OK	1.34E-19
UVSSA	9.72E-05	3.726227795	0.00950436	OK	0.00019625
AC147067.2	2.95E-28	10.96063841	0.01985622	OK	9.51E-28
AC147067.1	1.04E-12	7.029021117	0.01798756	OK	2.70E-12
SLBP	2.36E-260	34.44865775	0.08852188	OK	1.62E-259
TMEM129	4.06E-10	6.142698837	0.01571778	OK	9.93E-10
TACC3	0	39.33604347	0.10103404	OK	0
FGFR3	4.57E-06	4.436640182	0.010978	OK	9.79E-06
LETM1	7.86E-08	5.243943989	0.01340704	OK	1.80E-07
NSD2	1.03E-09	5.992686817	0.01532706	OK	2.50E-09
C4orf48	0	153.7432219	0.39533408	OK	0
MXD4	2.76E-199	30.09564438	0.07732751	OK	1.71E-198
ZFYVE28	3.83E-13	7.167068833	0.01833962	OK	1.00E-12
RNF4	1.63E-11	6.634188283	0.01698244	OK	4.13E-11
TNIP2	9.72E-79	18.74977881	0.04814389	OK	4.24E-78
SH3BP2	0	56.03407527	0.14401612	OK	0
ADD1	1.31E-103	21.58239785	0.05543033	OK	6.33E-103
MFSD10	0	40.99586242	0.1053606	OK	0
NOP14	3.48E-16	8.071314826	0.02067739	OK	9.62E-16
HTT	1.26E-29	11.24255389	0.02883216	OK	4.10E-29
RGS12	1.13E-10	6.343124832	0.01622773	OK	2.80E-10
DOK7	3.35E-06	4.503127217	0.01131022	OK	7.22E-06
LRPAP1	0	72.75173454	0.1870389	OK	0
AC141928.1	5.01E-11	6.466514973	0.01642229	OK	1.26E-10
ADRA2C	0	53.42588494	0.13722361	OK	0
TMEM128	1.22E-08	5.577479116	0.01426487	OK	2.87E-08
LYAR	6.89E-163	27.1723752	0.06980434	OK	3.93E-162
STX18	2.09E-15	7.849325067	0.02010761	OK	5.70E-15
MSX1	1.01E-54	15.53447364	0.03982809	OK	3.92E-54
CYTL1	3.17E-106	21.85923639	0.0560857	OK	1.54E-105
EVC2	2.10E-06	4.600977032	0.01171996	OK	4.58E-06
EVC	9.82E-13	7.037024191	0.01798558	OK	2.56E-12
WFS1	7.55E-107	21.92470919	0.05626968	OK	3.68E-106
MAN2B2	2.78E-06	4.542174519	0.01160257	OK	6.02E-06
MRFAP1	0	96.80838461	0.24891046	OK	0
LINC02482	2.87E-06	4.535450765	0.01157853	OK	6.21E-06
AC093323.1	7.88E-08	5.243534044	0.0133989	OK	1.80E-07
S100P	6.04E-09	5.698664656	0.01435318	OK	1.43E-08
MRFAP1L1	7.84E-35	12.25562667	0.03143464	OK	2.67E-34
BLOC1S4	1.12E-38	12.95353158	0.03323685	OK	3.92E-38

KIAA0232	1.44E-30	11.43252976	0.02932542	OK	4.71E-30
TBC1D14	6.03E-115	22.75759491	0.05843706	OK	3.02E-114
GRPEL1	0	51.05340117	0.13122676	OK	0
AFAP1	4.36E-25	10.27947592	0.02633193	OK	1.36E-24
SH3TC1	9.30E-246	33.459685	0.08594231	OK	6.24E-245
HTRA3	2.70E-65	17.02453922	0.04329778	OK	1.10E-64
SLC2A9	1.05E-32	11.85233477	0.03037333	OK	3.51E-32
WDR1	0	37.6490068	0.09675534	OK	0
CLNK	0	148.6243672	0.37947951	OK	0
HS3ST1	0	60.9945476	0.15675777	OK	0
RAB28	8.34E-05	3.764500545	0.00960326	OK	0.00016906
BOD1L1	1.28E-20	9.235736889	0.0236796	OK	3.80E-20
CPEB2-DT	0.00015933	3.59964064	0.00866297	OK	0.00031838
C1QTNF7	0	46.49749078	0.11943276	OK	0
AC099550.1	0.00014343	3.626888569	0.00891	OK	0.00028718
CC2D2A	2.23E-80	18.94955001	0.04862132	OK	9.79E-80
FBXL5	9.15E-09	5.627267302	0.0143972	OK	2.16E-08
FAM200B	3.54E-101	21.32205391	0.05476123	OK	1.69E-100
BST1	4.24E-224	31.93589642	0.08203947	OK	2.76E-223
CD38	6.44E-85	19.4918467	0.04998529	OK	2.89E-84
TAPT1	1.73E-05	4.141362873	0.01057272	OK	3.61E-05
LDB2	1.93E-231	32.46038437	0.08317972	OK	1.27E-230
QDPR	6.49E-67	17.24140634	0.04426416	OK	2.68E-66
LAP3	0	163.4388842	0.42027947	OK	0
MED28	3.36E-90	20.10466293	0.05163051	OK	1.54E-89
DCAF16	2.91E-10	6.194970273	0.01584851	OK	7.16E-10
NCAPG	0	154.2503799	0.39444418	OK	0
LCORL	2.21E-89	20.01105981	0.05137683	OK	1.01E-88
SLIT2	0	48.72908388	0.12508857	OK	0
ADGRA3	1.43E-12	6.984422804	0.01783929	OK	3.71E-12
DHX15	5.10E-18	8.571728971	0.02196782	OK	1.45E-17
SOD3	0	319.282161	0.82109123	OK	0
CCDC149	1.08E-05	4.247152665	0.01084309	OK	2.29E-05
LGI2	4.00E-135	24.71175833	0.06343148	OK	2.12E-134
PI4K2B	7.19E-05	3.80161586	0.00968549	OK	0.00014608
SEL1L3	1.99E-56	15.78432002	0.04045161	OK	7.79E-56
SMIM20	5.17E-120	23.26365878	0.05975427	OK	2.62E-119
RBPJ	0	73.92491721	0.19005508	OK	0
STIM2	8.23E-56	15.69465978	0.04028008	OK	3.20E-55
PCDH7	0	146.9242337	0.37776895	OK	0
ARAP2	7.37E-100	21.17955606	0.05436462	OK	3.51E-99
DTHD1	8.96E-09	5.630909924	0.01424644	OK	2.11E-08
RELL1	5.97E-55	15.56841521	0.03994747	OK	2.31E-54
PGM2	1.93E-98	21.02510186	0.05399053	OK	9.16E-98
TBC1D1	1.00E-86	19.70356559	0.05059866	OK	4.54E-86
LINC02513	7.82E-30	11.28459582	0.0275697	OK	2.55E-29
KLF3	0	56.57408611	0.14542907	OK	0
TLR10	1.99E-180	28.61822345	0.07330142	OK	1.18E-179
TLR1	0	75.84336002	0.19495292	OK	0
TLR6	5.45E-25	10.25794101	0.02627974	OK	1.70E-24
FAM114A1	0	43.14938427	0.11089268	OK	0
TMEM156	0	37.86625106	0.09712276	OK	0
KLHL5	2.73E-124	23.68252837	0.06082371	OK	1.40E-123

WDR19	3.50E-07	4.96121127	0.01266398	OK	7.85E-07
RFC1	2.84E-119	23.19042085	0.05956765	OK	1.44E-118
RPL9	0	174.881621	0.44964152	OK	0
LIAS	0.00011916	3.674488193	0.00937121	OK	0.00023962
UGDH	3.56E-33	11.9422798	0.03062296	OK	1.20E-32
SMIM14	0	39.54429636	0.10162647	OK	0
UBE2K	1.75E-91	20.25073122	0.05200735	OK	8.08E-91
PDS5A	2.96E-14	7.509715868	0.01923544	OK	7.93E-14
N4BP2	4.86E-27	10.7042376	0.027444	OK	1.55E-26
RHOH	0	43.61721159	0.1120481	OK	0
RBM47	0	102.8452676	0.26441417	OK	0
NSUN7	0.00015947	3.599403823	0.00915848	OK	0.00031864
APBB2	1.85E-95	20.69678486	0.05312457	OK	8.67E-95
UCHL1	1.05E-124	23.72288413	0.06087099	OK	5.38E-124
LIMCH1	0	137.7549921	0.35418189	OK	0
TMEM33	7.66E-173	28.00208756	0.07193673	OK	4.48E-172
SHISA3	1.19E-61	16.52633015	0.04197865	OK	4.77E-61
GNPDA2	8.31E-66	17.09343026	0.04386376	OK	3.41E-65
COMMD8	0	46.48389222	0.11947236	OK	0
ATP10D	4.62E-12	6.817969149	0.01745666	OK	1.18E-11
NIPAL1	2.52E-11	6.569515853	0.01646576	OK	6.36E-11
CNGA1	1.34E-55	15.66374941	0.03979133	OK	5.20E-55
TXK	1.92E-06	4.62011993	0.01172209	OK	4.18E-06
TEC	2.77E-42	13.57613459	0.03481424	OK	9.98E-42
SLAIN2	1.03E-52	15.23514633	0.03910662	OK	3.95E-52
SLC10A4	1.21E-09	5.966252257	0.01379951	OK	2.93E-09
OCIAD1	0	48.34340896	0.12426218	OK	0
OCIAD2	0	45.44056304	0.11675869	OK	0
DCUN1D4	5.44E-21	9.327097768	0.02390248	OK	1.62E-20
SGCB	0	103.511412	0.26610468	OK	0
SPATA18	3.92E-13	7.163987881	0.01826809	OK	1.03E-12
USP46	3.43E-11	6.523547935	0.0166974	OK	8.62E-11
RASL11B	4.45E-12	6.82317924	0.01742426	OK	1.14E-11
FIP1L1	1.86E-15	7.864125347	0.02014912	OK	5.07E-15
CHIC2	1.38E-109	22.20974623	0.05704129	OK	6.82E-109
PDGFRA	0	56.32958177	0.14473592	OK	0
KIT	9.83E-32	11.6631676	0.02947397	OK	3.26E-31
KDR	1.10E-62	16.66916287	0.04234583	OK	4.44E-62
SRD5A3	0	45.05441461	0.11579093	OK	0
TMEM165	1.22E-95	20.71683553	0.05320794	OK	5.72E-95
EXOC1	2.71E-09	5.833651309	0.01492732	OK	6.48E-09
CEP135	3.71E-05	3.962487124	0.01011233	OK	7.63E-05
KIAA1211	1.18E-12	7.010793776	0.01749989	OK	3.07E-12
PPAT	0.0001962	3.545144728	0.00903412	OK	0.00039008
PAICS	1.26E-190	29.42649708	0.07559123	OK	7.65E-190
SRP72	0	45.13914922	0.1160204	OK	0
HOPX	1.29E-56	15.8117806	0.04052422	OK	5.04E-56
SPINK2	3.96E-09	5.769962461	0.01468286	OK	9.45E-09
REST	1.77E-48	14.58417554	0.03743378	OK	6.62E-48
NOA1	3.39E-26	10.52285002	0.02698067	OK	1.07E-25
POLR2B	5.58E-08	5.306789726	0.01357269	OK	1.28E-07
IGFBP7	0	318.4504523	0.81896219	OK	0
ADGRL3	5.79E-96	20.75270123	0.05322907	OK	2.72E-95

CENPC	4.64E-29	11.12686048	0.02854063	OK	1.51E-28
UBA6	7.26E-20	9.048233631	0.02319164	OK	2.12E-19
YTHDC1	6.92E-33	11.88697671	0.03049744	OK	2.32E-32
SULT1B1	7.12E-15	7.694115638	0.01947407	OK	1.93E-14
UTP3	1.09E-16	8.212255837	0.02104324	OK	3.03E-16
RUFY3	3.79E-20	9.118917635	0.02337084	OK	1.12E-19
GRSF1	9.72E-57	15.82963797	0.04063517	OK	3.80E-56
MOB1B	3.91E-49	14.68691427	0.03768663	OK	1.47E-48
DCK	0	45.49496501	0.11691592	OK	0
SLC4A4	0.00015898	3.600209253	0.00909597	OK	0.00031775
ADAMTS3	0.00023673	3.495344603	0.00882632	OK	0.00046833
ANKRD17	1.91E-06	4.620708756	0.01180744	OK	4.17E-06
CXCL8	0	143.3301148	0.36854152	OK	0
CXCL1	1.94E-169	27.72114099	0.07115202	OK	1.13E-168
PPBP	1.85E-20	9.196319438	0.02305966	OK	5.48E-20
CXCL5	0	50.02315946	0.12834525	OK	0
CXCL3	0	111.242776	0.28600258	OK	0
CXCL2	0	107.1490838	0.27548447	OK	0
MTHFD2L	4.08E-30	11.34159157	0.02908698	OK	1.33E-29
EREG	0	166.0548107	0.42686142	OK	0
AREG	0	121.7355004	0.31295859	OK	0
BTC	0	45.29560221	0.11631806	OK	0
PARM1	0	38.08428315	0.09780521	OK	0
RCHY1	4.21E-16	8.047965569	0.02061872	OK	1.16E-15
CDKL2	3.54E-31	11.55366749	0.02953445	OK	1.17E-30
G3BP2	0	39.01789685	0.10027586	OK	0
USO1	1.11E-38	12.95456222	0.0332413	OK	3.87E-38
NAAA	0	55.84143566	0.14353502	OK	0
SDAD1	3.46E-43	13.72788906	0.03522983	OK	1.25E-42
CXCL9	2.15E-10	6.242864885	0.015945	OK	5.29E-10
ART3	3.02E-06	4.525290783	0.01097532	OK	6.52E-06
CXCL10	5.44E-39	13.00901762	0.03331844	OK	1.90E-38
NUP54	2.90E-10	6.195863058	0.01585792	OK	7.12E-10
SCARB2	0	44.04647652	0.11320819	OK	0
FAM47E	0.00034618	3.392585617	0.00857564	OK	0.00067871
STBD1	6.18E-09	5.69472856	0.01447668	OK	1.46E-08
CCDC158	1.12E-08	5.59224082	0.01417168	OK	2.64E-08
SHROOM3	3.84E-218	31.50387139	0.08089282	OK	2.47E-217
SEPT11	2.26E-233	32.59706887	0.08375965	OK	1.49E-232
CCNI	0	133.9997013	0.34455338	OK	0
CCNG2	1.67E-26	10.58948027	0.02715269	OK	5.27E-26
CNOT6L	3.20E-50	14.85556759	0.03812718	OK	1.21E-49
MRPL1	3.02E-23	9.862691198	0.02528747	OK	9.26E-23
ANXA3	3.73E-19	8.867674079	0.02259717	OK	1.08E-18
LINC01094	0	97.48869241	0.25061576	OK	0
AC098818.2	2.44E-07	5.031261816	0.01283906	OK	5.49E-07
BMP2K	0	108.6273603	0.27929058	OK	0
PAQR3	0.00039496	3.356301369	0.00854278	OK	0.0007716
LINC01088	8.29E-234	32.62775095	0.08376242	OK	5.47E-233
LINC00989	7.10E-22	9.540617203	0.02435728	OK	2.14E-21
ANTXR2	1.01E-11	6.704770162	0.01716621	OK	2.57E-11
PRDM8	3.76E-10	6.154735463	0.01573515	OK	9.21E-10
RASGEF1B	0	173.6576312	0.44655489	OK	0

HNRNPD	0	50.1280794	0.12885234	OK	0
HNRNPDL	0	52.29493413	0.13442143	OK	0
ENOPH1	8.39E-68	17.35931711	0.044565	OK	3.47E-67
TMEM150C	6.76E-08	5.271570021	0.01315544	OK	1.55E-07
SEC31A	8.65E-74	18.13360765	0.04656266	OK	3.68E-73
THAP9-AS1	3.88E-17	8.334936943	0.02135669	OK	1.09E-16
COPS4	2.70E-53	15.32273082	0.03933061	OK	1.03E-52
PLAC8	0	47.99284266	0.12317123	OK	0
COQ2	1.23E-277	35.58411994	0.09142464	OK	8.64E-277
HPSE	2.40E-307	37.45596143	0.09621586	OK	1.75E-306
MRPS18C	0	38.23008425	0.09825008	OK	0
ABRAXAS1	8.20E-22	9.525657034	0.0244174	OK	2.47E-21
GPAT3	0	96.53875857	0.24810645	OK	0
CDS1	3.67E-07	4.952182807	0.01253018	OK	8.21E-07
WDFY3	7.80E-08	5.245295211	0.01341159	OK	1.79E-07
ARHGAP24	3.36E-81	19.04878867	0.04890561	OK	1.48E-80
MAPK10	1.18E-90	20.1565612	0.05171487	OK	5.41E-90
PTPN13	2.64E-18	8.647172161	0.02212996	OK	7.55E-18
SLC10A6	8.98E-05	3.74609135	0.00805831	OK	0.00018167
AFF1	3.02E-27	10.74819309	0.02756391	OK	9.63E-27
KLHL8	3.04E-15	7.802241453	0.0199816	OK	8.27E-15
HSD17B11	0	74.27100819	0.19094329	OK	0
NUDT9	9.45E-35	12.24040228	0.03140161	OK	3.21E-34
SPARCL1	0	261.4310706	0.67230469	OK	0
AC093895.1	6.13E-189	29.29428719	0.07518644	OK	3.72E-188
SPP1	0	277.4907496	0.71357905	OK	0
PKD2	0	44.38077495	0.11403882	OK	0
ABCG2	1.22E-51	15.07314978	0.0386119	OK	4.62E-51
PPM1K	5.37E-99	21.08575662	0.05414653	OK	2.55E-98
HERC5	2.87E-25	10.31981685	0.02646167	OK	8.97E-25
PYURF	0	48.47121945	0.12459056	OK	0
NAP1L5	1.30E-33	12.02589428	0.03082001	OK	4.38E-33
FAM13A	1.94E-05	4.114731865	0.01050549	OK	4.05E-05
GPRIN3	0	68.27754859	0.17550955	OK	0
SNCA	0	62.97791057	0.16186643	OK	0
MMRN1	0	46.74007877	0.1183785	OK	0
CCSER1	5.69E-283	35.92749602	0.09208995	OK	4.01E-282
GRID2	3.01E-24	10.09144075	0.02575371	OK	9.34E-24
HPGDS	0	52.25509582	0.13424017	OK	0
PDLIM5	0	75.27829666	0.19352943	OK	0
BMPR1B	2.03E-109	22.19251507	0.05692193	OK	1.00E-108
UNC5C	4.30E-22	9.592534779	0.02454259	OK	1.30E-21
AC106881.1	7.07E-18	8.533956779	0.02181034	OK	2.01E-17
STPG2	3.03E-14	7.506846252	0.0159588	OK	8.10E-14
RAP1GDS1	2.09E-56	15.78132376	0.0405065	OK	8.17E-56
TSPAN5	1.25E-16	8.195317098	0.02097746	OK	3.48E-16
EIF4E	0	42.52504931	0.10929285	OK	0
METAP1	5.09E-18	8.571834265	0.02196505	OK	1.45E-17
ADH5	0	120.775206	0.31054972	OK	0
AP002026.1	8.90E-10	6.016653817	0.01534005	OK	2.16E-09
ADH4	0.00014266	3.628281534	0.00880138	OK	0.00028566
ADH1A	9.42E-08	5.210481937	0.01296502	OK	2.15E-07
ADH1B	0	128.7268424	0.33096324	OK	0

DAPP1	0	76.81109739	0.19742659	OK	0
LAMTOR3	1.23E-58	16.10248876	0.04133547	OK	4.85E-58
DNAJB14	2.45E-55	15.62516272	0.04011051	OK	9.51E-55
H2AFZ	0	122.0043853	0.31370884	OK	0
DDIT4L	9.98E-31	11.46415652	0.0293379	OK	3.28E-30
EMCN	0	110.9605849	0.28337994	OK	0
PPP3CA	1.27E-54	15.52022044	0.03983966	OK	4.89E-54
AP001816.1	0.00012168	3.669142006	0.00935647	OK	0.00024449
BANK1	3.82E-120	23.27664108	0.05959401	OK	1.94E-119
SLC39A8	0	108.8562068	0.2798344	OK	0
NFKB1	0	37.97049651	0.09757086	OK	0
MANBA	1.60E-237	32.88857938	0.0844964	OK	1.06E-236
UBE2D3	0	85.93665014	0.22094882	OK	0
CISD2	0	74.2568014	0.19090465	OK	0
SLC9B2	9.90E-05	3.721511679	0.00947272	OK	0.00019986
BDH2	0	46.44766151	0.11936649	OK	0
CENPE	0	45.68886227	0.11721927	OK	0
AC004069.1	1.16E-26	10.62325149	0.02714338	OK	3.68E-26
TET2	0	40.29999666	0.10356332	OK	0
PPA2	2.61E-138	25.0063334	0.06423454	OK	1.40E-137
INTS12	8.68E-11	6.383024453	0.01633625	OK	2.16E-10
NPNT	0	56.79076701	0.14592487	OK	0
AIMP1	3.39E-170	27.78392799	0.07138287	OK	1.97E-169
DKK2	2.22E-33	11.98151376	0.03047874	OK	7.48E-33
PAPSS1	4.75E-226	32.07612867	0.08241776	OK	3.10E-225
SGMS2	1.26E-59	16.24278346	0.04167866	OK	5.01E-59
CYP2U1	1.50E-22	9.70017595	0.02485253	OK	4.57E-22
HADH	1.59E-167	27.56191918	0.07080408	OK	9.20E-167
LEF1	3.83E-34	12.12642223	0.03092702	OK	1.30E-33
RPL34	0	178.2829079	0.45835202	OK	0
OSTC	0	74.42891574	0.19135277	OK	0
MCUB	0	141.5542091	0.36397269	OK	0
CASP6	1.23E-10	6.329459108	0.01619631	OK	3.05E-10
PLA2G12A	2.60E-16	8.106568388	0.02075922	OK	7.21E-16
CFI	6.59E-55	15.56204682	0.03987822	OK	2.55E-54
GAR1	5.19E-19	8.830906933	0.02263382	OK	1.50E-18
ENPEP	0	38.28203927	0.09770128	OK	0
FAM241A	5.41E-46	14.18833122	0.03640381	OK	1.99E-45
AP1AR	1.10E-36	12.59689718	0.03231433	OK	3.78E-36
TIFA	1.90E-31	11.60684011	0.02976777	OK	6.29E-31
ALPK1	2.27E-37	12.72057878	0.03262826	OK	7.87E-37
ZGRF1	0.00013902	3.63494213	0.00923369	OK	0.00027859
LARP7	1.80E-108	22.09418649	0.05674952	OK	8.82E-108
ANK2	2.10E-139	25.10672846	0.06431926	OK	1.13E-138
CAMK2D	3.86E-14	7.475164997	0.01914688	OK	1.03E-13
ARSJ	3.21E-18	8.624923601	0.02205749	OK	9.16E-18
SNHG8	0	48.82380323	0.12549674	OK	0
SEC24D	8.64E-05	3.755649652	0.00958262	OK	0.00017501
SYNPO2	0	156.6251008	0.40272542	OK	0
MYOZ2	1.59E-259	34.39326056	0.0882777	OK	1.09E-258
USP53	2.29E-74	18.20642477	0.04674442	OK	9.78E-74
C4orf3	0	117.9119996	0.30318513	OK	0
PDE5A	0	194.6189767	0.5004429	OK	0

MAD2L1	0	96.18394322	0.24693955	OK	0
PRDM5	2.05E-06	4.606562061	0.01173557	OK	4.46E-06
NDNF	1.90E-131	24.36732939	0.06233677	OK	9.99E-131
ANXA5	0	167.0812002	0.42962939	OK	0
EXOSC9	1.84E-30	11.41095599	0.02926753	OK	6.03E-30
CCNA2	0	142.4774121	0.36434883	OK	0
BBS7	3.21E-09	5.805580001	0.01485003	OK	7.66E-09
TRPC3	2.78E-06	4.542484551	0.00962881	OK	6.01E-06
KIAA1109	2.16E-16	8.129283768	0.02083109	OK	5.99E-16
IL2	0.00015277	3.61055662	0.00881229	OK	0.00030555
FGF2	0	40.80655709	0.10481513	OK	0
SPATA5	9.74E-05	3.72575176	0.00949467	OK	0.0001966
SPRY1	0	40.7274587	0.1046212	OK	0
LINC01091	6.90E-06	4.347000621	0.01078943	OK	1.47E-05
FAT4	6.90E-62	16.55892093	0.04246001	OK	2.78E-61
INTU	2.16E-68	17.43706041	0.04473863	OK	8.96E-68
HSPA4L	4.01E-73	18.04904593	0.04629692	OK	1.70E-72
PLK4	3.50E-49	14.69435051	0.03729465	OK	1.32E-48
PGRMC2	7.13E-120	23.24989094	0.05971605	OK	3.61E-119
AC078850.1	8.26E-280	35.7244143	0.09154072	OK	5.81E-279
JADE1	5.10E-06	4.412981638	0.01127015	OK	1.09E-05
SCLT1	7.95E-28	10.87071459	0.02787275	OK	2.55E-27
C4orf33	7.84E-39	12.98097131	0.03329501	OK	2.74E-38
PCDH10	4.52E-239	32.99675903	0.08473336	OK	3.01E-238
PCDH18	3.53E-109	22.16761212	0.05687618	OK	1.74E-108
AC116563.1	2.57E-20	9.161027483	0.02333876	OK	7.59E-20
SLC7A11	1.44E-22	9.70435208	0.02477881	OK	4.39E-22
NOCT	0	39.96890905	0.10262282	OK	0
ELF2	1.08E-17	8.484629583	0.02174696	OK	3.06E-17
MGARP	1.04E-181	28.72107213	0.07361568	OK	6.20E-181
NDUFC1	0	83.72463241	0.21526059	OK	0
NAA15	1.50E-12	6.977501067	0.01786787	OK	3.89E-12
AC097376.2	0.00043511	3.329437139	0.00848264	OK	0.00084785
RAB33B	2.24E-18	8.66580439	0.02220296	OK	6.42E-18
SETD7	3.34E-06	4.50357619	0.01150568	OK	7.21E-06
MGST2	0	72.88679093	0.18738265	OK	0
MAML3	3.61E-14	7.483832226	0.01916355	OK	9.64E-14
SCOC	4.26E-295	36.69577868	0.09429958	OK	3.06E-294
ELMOD2	1.70E-07	5.100187979	0.01303883	OK	3.84E-07
TBC1D9	7.30E-176	28.24908242	0.07256008	OK	4.30E-175
RNF150	4.68E-41	13.36751368	0.03427504	OK	1.67E-40
ZNF330	1.97E-118	23.107036	0.05935008	OK	9.94E-118
LINC02432	9.25E-07	4.769185652	0.01205705	OK	2.04E-06
AC097504.2	3.66E-10	6.158799608	0.01406185	OK	8.98E-10
IL15	1.10E-31	11.653808	0.02988606	OK	3.64E-31
INPP4B	0	41.13293347	0.10566414	OK	0
USP38	1.09E-10	6.348470208	0.01624325	OK	2.70E-10
GAB1	8.57E-21	9.278784325	0.02377254	OK	2.55E-20
SMARCA5	2.00E-49	14.73231014	0.03781594	OK	7.52E-49
ANAPC10	1.53E-19	8.966259324	0.02298113	OK	4.46E-19
ABCE1	1.46E-63	16.78946239	0.04310326	OK	5.92E-63
SMAD1	4.22E-07	4.925121517	0.01257671	OK	9.41E-07
ZNF827	5.36E-41	13.35748549	0.03420969	OK	1.91E-40

LSM6	0	40.80975888	0.10488306	OK	0
EDNRA	0	72.12686695	0.18530678	OK	0
PRMT9	1.15E-76	18.49403874	0.04747737	OK	4.98E-76
ARHGAP10	2.71E-273	35.3022862	0.09071653	OK	1.89E-272
NR3C2	9.17E-05	3.740895984	0.00951776	OK	0.00018541
DCLK2	3.38E-36	12.5078093	0.0320475	OK	1.16E-35
LRBA	0.00014673	3.621000238	0.00923052	OK	0.00029375
MAB21L2	1.56E-284	36.02739229	0.09246313	OK	1.10E-283
RPS3A	0	176.9449594	0.45492663	OK	0
SH3D19	2.27E-274	35.37236575	0.09083957	OK	1.58E-273
FAM160A1	2.74E-05	4.033877176	0.01003551	OK	5.68E-05
GATB	9.51E-09	5.620623593	0.01436675	OK	2.24E-08
AC097375.1	2.66E-28	10.97014092	0.02269814	OK	8.56E-28
FBXW7	1.49E-12	6.978555908	0.01786961	OK	3.86E-12
TMEM154	1.25E-135	24.75853987	0.06351102	OK	6.68E-135
ARFIP1	2.99E-21	9.390515416	0.02407425	OK	8.91E-21
TRIM2	3.56E-47	14.37798971	0.03685499	OK	1.32E-46
MND1	0	95.29330948	0.24405249	OK	0
TMEM131L	8.23E-07	4.79266916	0.01224352	OK	1.82E-06
TLR2	0	118.3477254	0.30428556	OK	0
SFRP2	0	80.61076863	0.20704059	OK	0
PLRG1	2.50E-38	12.89189052	0.03307848	OK	8.73E-38
AC104407.1	7.32E-84	19.36706351	0.04952187	OK	3.27E-83
NPY2R	5.04E-05	3.888648375	0.00981263	OK	0.00010312
MAP9	0	78.32971544	0.20133579	OK	0
GUCY1A1	0	192.2887135	0.49445325	OK	0
GUCY1B1	0	96.40157491	0.24779906	OK	0
TDO2	2.57E-33	11.96945101	0.03044299	OK	8.65E-33
CTSO	1.49E-37	12.75358069	0.03271612	OK	5.16E-37
PDGFC	5.70E-187	29.13934803	0.07485028	OK	3.44E-186
GLRB	4.70E-55	15.58371837	0.03992264	OK	1.82E-54
GRIA2	0	47.00680867	0.12075876	OK	0
FAM198B	1.56E-76	18.47764368	0.04744103	OK	6.74E-76
TMEM144	1.02E-05	4.260738311	0.01087781	OK	2.15E-05
RXFP1	2.45E-12	6.908657268	0.01710608	OK	6.31E-12
C4orf46	1.23E-09	5.964383541	0.01524476	OK	2.97E-09
PPID	1.26E-39	13.12046817	0.03366668	OK	4.43E-39
FNIP2	0	43.47756482	0.11173644	OK	0
NAF1	5.78E-62	16.56964674	0.04252865	OK	2.33E-61
NPY1R	0	39.41929226	0.10119296	OK	0
NPY5R	2.49E-13	7.225995031	0.01833081	OK	6.54E-13
TMA16	2.51E-39	13.06806207	0.03353152	OK	8.81E-39
MARCH11	0	135.2354309	0.3477151	OK	0
TMEM192	6.28E-13	7.099113748	0.01817835	OK	1.64E-12
KLHL2	6.02E-09	5.699224358	0.01457427	OK	1.43E-08
GK3P	5.07E-06	4.414115861	0.01113393	OK	1.09E-05
MSMO1	3.29E-05	3.990970292	0.01018493	OK	6.79E-05
CPE	0	247.9094512	0.63751296	OK	0
TLL1	7.67E-05	3.785423408	0.0090295	OK	0.00015576
DDX60L	3.74E-280	35.74656224	0.09183142	OK	2.63E-279
PALLD	0	190.2744421	0.48928933	OK	0
NEK1	1.55E-11	6.641450275	0.0170002	OK	3.93E-11
CLCN3	2.82E-07	5.00313068	0.01278983	OK	6.34E-07

HPF1	1.79E-142	25.38623566	0.06521232	OK	9.71E-142
MFAP3L	5.67E-06	4.389931667	0.01116391	OK	1.21E-05
AADAT	3.97E-07	4.937055231	0.01253888	OK	8.86E-07
GALNT7	3.88E-10	6.149560212	0.01573605	OK	9.52E-10
HMGB2	0	59.50753293	0.15296993	OK	0
AC097534.2	1.45E-15	7.895154353	0.02021872	OK	3.96E-15
SAP30	0	74.9665949	0.19272682	OK	0
SCRG1	0	126.8027455	0.32598937	OK	0
HAND2	0	47.68882485	0.12249298	OK	0
HAND2-AS1	1.16E-37	12.7727716	0.03269516	OK	4.04E-37
LINC02269	3.66E-42	13.55589284	0.03466451	OK	1.31E-41
FBXO8	1.00E-12	7.034105591	0.01800992	OK	2.61E-12
HPGD	4.55E-98	20.98441105	0.05380297	OK	2.15E-97
GPM6A	0.00012159	3.669347124	0.00911313	OK	0.00024431
SPATA4	8.21E-10	6.029810993	0.01422194	OK	1.99E-09
SPCS3	0	79.1322557	0.20344875	OK	0
VEGFC	7.88E-16	7.97082606	0.01992284	OK	2.16E-15
NEIL3	2.55E-69	17.55867722	0.04434345	OK	1.06E-68
AGA	5.03E-34	12.10399819	0.03104834	OK	1.70E-33
DCTD	2.01E-42	13.59981777	0.03489796	OK	7.23E-42
WWC2	1.05E-07	5.190696215	0.0132692	OK	2.39E-07
CDKN2AIP	2.24E-16	8.125038041	0.0208197	OK	6.20E-16
ING2	0	49.09516043	0.12618806	OK	0
RWDD4	1.59E-56	15.79860566	0.04055487	OK	6.22E-56
IRF2	3.63E-138	24.99315948	0.06420437	OK	1.95E-137
CASP3	1.73E-31	11.61471479	0.02979033	OK	5.74E-31
CENPU	0	77.22785704	0.19837681	OK	0
ACSL1	0	65.0848486	0.16730091	OK	0
AC084871.2	4.65E-07	4.905927589	0.01249155	OK	1.04E-06
MIR3945HG	1.76E-278	35.63875507	0.09153537	OK	1.23E-277
SLC25A4	0	211.0445945	0.54268789	OK	0
CFAP97	0	55.0264329	0.14144423	OK	0
SNX25	2.21E-08	5.473269119	0.01397942	OK	5.15E-08
ANKRD37	6.23E-66	17.11022408	0.04392494	OK	2.56E-65
UFSP2	1.08E-80	18.98747359	0.04875245	OK	4.76E-80
CCDC110	2.67E-38	12.88686059	0.03298964	OK	9.31E-38
AC106897.1	1.24E-31	11.64366943	0.02979679	OK	4.09E-31
PDLIM3	0	230.8513186	0.59364661	OK	0
SORBS2	0	177.9947207	0.45767093	OK	0
TLR3	4.29E-05	3.927391063	0.01001133	OK	8.81E-05
FAM149A	6.06E-19	8.813542256	0.02254471	OK	1.75E-18
FAT1	2.79E-224	31.9489486	0.08204189	OK	1.82E-223
AC108865.2	1.99E-07	5.070218651	0.01093967	OK	4.49E-07
LINC02515	2.51E-13	7.224878937	0.01815914	OK	6.60E-13
FRG1	1.54E-191	29.49774322	0.07579089	OK	9.38E-191
CCDC127	2.80E-07	5.004661058	0.01279362	OK	6.29E-07
SDHA	7.45E-36	12.44488902	0.03192907	OK	2.56E-35
PDCD6	0	51.47924194	0.13232743	OK	0
BRD9	0.00023239	3.500270827	0.00892595	OK	0.00046
TRIP13	1.30E-162	27.14890645	0.069343	OK	7.43E-162
SLC12A7	1.27E-31	11.64162284	0.0298376	OK	4.19E-31
CLPTM1L	3.11E-39	13.05161348	0.03348942	OK	1.09E-38
LPCAT1	7.16E-34	12.07501163	0.03096598	OK	2.42E-33

MRPL36	6.60E-216	31.34028964	0.08052738	OK	4.22E-215
NDUFS6	0	59.78018039	0.153677	OK	0
IRX2	2.43E-21	9.412273024	0.02404033	OK	7.26E-21
C5orf38	1.13E-114	22.73005438	0.05831777	OK	5.64E-114
IRX1	4.79E-16	8.032154552	0.02050568	OK	1.32E-15
ICE1	1.62E-05	4.155666846	0.01061255	OK	3.40E-05
MED10	7.43E-96	20.74075483	0.05326794	OK	3.48E-95
LINC01018	1.71E-08	5.518078415	0.01362012	OK	4.01E-08
SRD5A1	0.00045235	3.31859781	0.00845101	OK	0.00088005
ADCY2	1.02E-06	4.750288282	0.01204579	OK	2.23E-06
FASTKD3	0.00024395	3.487313045	0.00888279	OK	0.00048223
MIR4458HG	6.83E-23	9.780388532	0.02506364	OK	2.09E-22
SEMA5A	1.22E-294	36.66707846	0.09415434	OK	8.78E-294
SNHG18	0	47.63697015	0.12238773	OK	0
CCT5	0	70.85171047	0.18215188	OK	0
AC012640.4	1.64E-05	4.153262333	0.00998967	OK	3.43E-05
CMBL	0	49.55542634	0.1273351	OK	0
MARCH6	6.05E-11	6.438131867	0.01648262	OK	1.51E-10
ANKRD33B	0.00018682	3.558038339	0.00903539	OK	0.00037176
DAP	2.00E-98	21.02336609	0.05399311	OK	9.50E-98
DNAH5	3.33E-15	7.790878352	0.01928381	OK	9.04E-15
TRIO	4.81E-09	5.737145653	0.01467381	OK	1.14E-08
OTULINL	0	100.7692569	0.25906997	OK	0
OTULIN	4.42E-40	13.19934991	0.03386644	OK	1.56E-39
ANKH	6.89E-47	14.33210407	0.03678162	OK	2.55E-46
FBXL7	1.25E-14	7.621487436	0.01947171	OK	3.37E-14
ZNF622	3.57E-176	28.27434765	0.07263797	OK	2.11E-175
RETREG1	1.41E-11	6.655291689	0.01698538	OK	3.59E-11
MYO10	2.67E-06	4.55068306	0.01159845	OK	5.79E-06
BASP1	0	102.2940057	0.26301517	OK	0
CDH6	0	101.4447006	0.26046237	OK	0
PDZD2	1.09E-07	5.183427242	0.01317314	OK	2.48E-07
GOLPH3	8.59E-17	8.240307898	0.02111608	OK	2.40E-16
ZFR	7.79E-63	16.68969875	0.04284909	OK	3.15E-62
SUB1	0	101.9808483	0.26221181	OK	0
NPR3	0	43.33320667	0.11123795	OK	0
TARS	1.16E-35	12.40931638	0.03183723	OK	3.98E-35
ADAMTS12	3.24E-09	5.803920394	0.01462463	OK	7.74E-09
RAI14	2.06E-128	24.07938747	0.06182948	OK	1.07E-127
AC026801.2	0.00011273	3.688641766	0.00934545	OK	0.00022697
TTC23L	5.30E-23	9.806132778	0.02331503	OK	1.62E-22
RAD1	1.66E-05	4.150215935	0.01059633	OK	3.48E-05
BRIX1	8.22E-55	15.5479293	0.03990737	OK	3.18E-54
DNAJC21	6.77E-132	24.4095257	0.06270439	OK	3.57E-131
PRLR	1.44E-11	6.652596004	0.01695858	OK	3.65E-11
IL7R	0	44.92916797	0.11541738	OK	0
SKP2	0.00010994	3.6950106	0.0094105	OK	0.00022148
NADK2	7.38E-06	4.332196026	0.01106071	OK	1.57E-05
RANBP3L	1.94E-79	18.83522309	0.04830234	OK	8.51E-79
SLC1A3	0	114.2497827	0.29372163	OK	0
NIPBL	0.00044834	3.321088388	0.00846731	OK	0.00087246
CPLANE1	2.42E-32	11.78192506	0.03020722	OK	8.08E-32
WDR70	9.75E-10	6.001969716	0.01535836	OK	2.36E-09

EGFLAM	2.36E-92	20.34914951	0.05217351	OK	1.09E-91
AC010457.1	3.20E-05	3.997889624	0.00964541	OK	6.60E-05
LIFR	4.41E-37	12.66860786	0.03245659	OK	1.52E-36
LIFR-AS1	6.01E-15	7.715843966	0.01970359	OK	1.63E-14
OSMR-AS1	0.00022602	3.507676091	0.00888799	OK	0.00044759
OSMR	5.25E-80	18.90440406	0.04849041	OK	2.30E-79
FYB1	0	229.3091113	0.58968965	OK	0
DAB2	0	187.7052129	0.48268449	OK	0
TTC33	1.52E-10	6.296328187	0.01611154	OK	3.77E-10
PTGER4	0	130.3541711	0.33517251	OK	0
RPL37	0	169.6207229	0.43610075	OK	0
CARD6	3.27E-46	14.22363933	0.03648538	OK	1.20E-45
C7	3.58E-252	33.89789304	0.08684057	OK	2.42E-251
C6	5.56E-107	21.93862434	0.03981808	OK	2.72E-106
PLCXD3	9.27E-11	6.373013037	0.01623111	OK	2.30E-10
OXCT1	8.39E-143	25.41598771	0.06525482	OK	4.56E-142
C5orf51	8.86E-06	4.291932701	0.01096058	OK	1.88E-05
FBXO4	9.31E-05	3.736978038	0.00953157	OK	0.00018825
GHR	5.75E-55	15.57080434	0.03990683	OK	2.23E-54
CCDC152	1.07E-44	13.97745866	0.03585092	OK	3.90E-44
SELENOP	0	230.149141	0.59183664	OK	0
AC008875.1	2.49E-11	6.571616868	0.01631543	OK	6.27E-11
ANXA2R	1.23E-11	6.675315233	0.01706895	OK	3.13E-11
AC025171.4	0.0001069	3.702125239	0.00935319	OK	0.00021544
ZNF131	7.55E-11	6.404322993	0.01639388	OK	1.88E-10
HMGCS1	0.00020434	3.534408647	0.00901111	OK	0.00040565
CCL28	0.00030067	3.431013247	0.00873014	OK	0.00059095
PAIP1	4.36E-146	25.71141936	0.06605086	OK	2.38E-145
NNT-AS1	1.00E-75	18.37721365	0.04717332	OK	4.30E-75
NNT	0.00011441	3.684860399	0.00939752	OK	0.0002303
FGF10	7.86E-91	20.17658151	0.0498777	OK	3.61E-90
MRPS30-DT	1.09E-10	6.348112417	0.01621346	OK	2.71E-10
MRPS30	1.21E-10	6.332178916	0.0162083	OK	3.00E-10
EMB	0	113.152109	0.29091081	OK	0
PARP8	9.28E-15	7.660276786	0.01961942	OK	2.50E-14
ITGA1	0	67.94958626	0.17464183	OK	0
PELO	9.63E-56	15.68465959	0.04025726	OK	3.74E-55
ITGA2	7.17E-10	6.051675543	0.01541974	OK	1.74E-09
MOCS2	0	45.01101075	0.11568053	OK	0
AC008966.1	0.00020423	3.534559043	0.00894896	OK	0.00040546
FST	1.41E-302	37.16182233	0.09531846	OK	1.02E-301
NDUFS4	0	43.6937348	0.11230137	OK	0
SNX18	4.36E-158	26.76300889	0.06875006	OK	2.46E-157
ESM1	7.59E-09	5.659506997	0.01393157	OK	1.79E-08
GZMK	1.21E-210	30.95156379	0.07944809	OK	7.69E-210
GZMA	0	40.71485739	0.10456037	OK	0
GPX8	0	59.32472904	0.15243491	OK	0
CCNO	5.52E-19	8.824042066	0.02251391	OK	1.59E-18
DHX29	2.13E-13	7.247334882	0.0185646	OK	5.60E-13
MTREX	0.00021672	3.518838865	0.00897417	OK	0.00042971
PLPP1	0	104.5274434	0.26872098	OK	0
SLC38A9	4.18E-05	3.933701288	0.0100321	OK	8.59E-05
IL6ST	5.58E-296	36.75113373	0.09444196	OK	4.01E-295

MAP3K1	8.75E-65	16.95558247	0.04351757	OK	3.57E-64
SETD9	1.72E-05	4.142742035	0.01057699	OK	3.59E-05
GPBP1	2.12E-94	20.57893923	0.0528528	OK	9.89E-94
PLK2	0	46.99722785	0.12077783	OK	0
GAPT	0	71.27484241	0.18315514	OK	0
RAB3C	0	37.71227283	0.0938018	OK	0
PDE4D	7.32E-74	18.14278186	0.04656026	OK	3.11E-73
PART1	0	48.65449939	0.12495654	OK	0
DEPDC1B	0	82.79599012	0.21106844	OK	0
NDUFAF2	1.25E-224	31.97415787	0.08215556	OK	8.12E-224
SMIM15	1.92E-154	26.44813897	0.06794478	OK	1.07E-153
ZSWIM6	6.52E-149	25.96278767	0.06668306	OK	3.59E-148
KIF2A	1.81E-117	23.01107401	0.05910394	OK	9.10E-117
DIMT1	1.24E-21	9.482837884	0.02430906	OK	3.72E-21
IPO11	7.46E-09	5.662556024	0.01445928	OK	1.76E-08
RNF180	0	40.31275812	0.10355604	OK	0
RGS7BP	0	78.00151316	0.20046109	OK	0
SREK1IP1	1.49E-212	31.09305462	0.07989309	OK	9.54E-212
CWC27	3.19E-22	9.623105065	0.02467186	OK	9.67E-22
CENPK	0	85.98731396	0.22074625	OK	0
TRIM23	3.53E-23	9.846918324	0.02523748	OK	1.08E-22
SGTB	4.35E-54	15.44076823	0.03963236	OK	1.68E-53
NLN	2.56E-107	21.97389893	0.05641467	OK	1.25E-106
AC010359.1	3.05E-06	4.523196625	0.0111895	OK	6.58E-06
ERBIN	5.84E-23	9.796345472	0.0251181	OK	1.78E-22
SREK1	4.69E-06	4.430886363	0.01132096	OK	1.00E-05
MAST4	3.25E-74	18.18741818	0.04667148	OK	1.38E-73
MAST4-AS1	2.82E-05	4.027241279	0.01020381	OK	5.84E-05
CD180	2.21E-176	28.29133423	0.07263995	OK	1.30E-175
PIK3R1	0	38.07289972	0.09784512	OK	0
SLC30A5	0.00035393	3.386519589	0.00863217	OK	0.00069346
CCNB1	0	84.82682585	0.21762011	OK	0
CENPH	5.73E-305	37.30962767	0.09579171	OK	4.17E-304
MRPS36	0	38.35661151	0.09857537	OK	0
CCDC125	3.38E-18	8.61886197	0.02207864	OK	9.65E-18
AK6	3.23E-82	19.17110936	0.04922896	OK	1.43E-81
TAF9	0	45.02527504	0.11572213	OK	0
RAD17	9.47E-14	7.356034002	0.0188365	OK	2.51E-13
SMN1	0.00039074	3.359276077	0.00855062	OK	0.00076375
NAIP	0	76.84891559	0.19754917	OK	0
MAP1B	0	258.6960327	0.66526816	OK	0
MRPS27	7.07E-24	10.00747227	0.02565355	OK	2.18E-23
ZNF366	1.58E-44	13.94950506	0.03523794	OK	5.78E-44
TNPO1	1.54E-07	5.118633452	0.01308921	OK	3.49E-07
FCHO2	0	65.70930465	0.16891804	OK	0
FOXD1	8.20E-44	13.83176366	0.03545441	OK	2.98E-43
BTF3	0	182.1515986	0.46837321	OK	0
ANKRA2	4.80E-08	5.333987688	0.01363717	OK	1.11E-07
ARHGEF28	2.92E-23	9.866000683	0.02524852	OK	8.96E-23
ENC1	5.77E-25	10.25237737	0.02626559	OK	1.80E-24
HEXB	0	98.47263323	0.25318929	OK	0
NSA2	0	63.52094348	0.16329785	OK	0
GCNT4	2.12E-09	5.874867334	0.01496182	OK	5.08E-09

HMGCR	2.57E-31	11.5810211	0.02969505	OK	8.49E-31
COL4A3BP	5.60E-63	16.70935001	0.04289813	OK	2.27E-62
POLK	2.18E-06	4.593472061	0.01173836	OK	4.74E-06
AC113404.1	1.56E-05	4.16416803	0.00990035	OK	3.28E-05
IQGAP2	0	180.5437106	0.46425951	OK	0
F2R	0	59.56494178	0.15299263	OK	0
S100Z	3.89E-16	8.057645732	0.02059297	OK	1.07E-15
CRHBP	4.05E-107	21.95301645	0.05631251	OK	1.98E-106
ZBED3	2.71E-19	8.903281211	0.02281537	OK	7.86E-19
PDE8B	2.02E-24	10.13087684	0.02590489	OK	6.26E-24
WDR41	1.19E-14	7.627780343	0.01953938	OK	3.21E-14
TBCA	0	92.78018697	0.23854934	OK	0
AP3B1	8.17E-59	16.12753782	0.04140122	OK	3.24E-58
SCAMP1-AS1	1.88E-07	5.080898954	0.01298047	OK	4.25E-07
SCAMP1	1.86E-26	10.57914159	0.02713095	OK	5.88E-26
LHFPL2	0	89.99158574	0.23134876	OK	0
ARSB	3.56E-25	10.29904365	0.02639617	OK	1.11E-24
BHMT2	4.15E-68	17.39964602	0.04461569	OK	1.72E-67
BHMT	2.26E-92	20.35124823	0.0431179	OK	1.05E-91
JMY	2.62E-26	10.54723298	0.02704446	OK	8.26E-26
TENT2	5.64E-44	13.85869523	0.03556123	OK	2.05E-43
CMYA5	1.11E-05	4.242218653	0.01077635	OK	2.34E-05
MTX3	0.00026895	3.461135303	0.00881269	OK	0.00053022
SERINC5	1.62E-105	21.78463633	0.05593686	OK	7.87E-105
ZFYVE16	0	56.13523507	0.14429632	OK	0
DHFR	6.18E-263	34.62064435	0.08888146	OK	4.25E-262
RASGRF2	0.00027378	3.45634167	0.00875481	OK	0.00053955
CKMT2-AS1	4.25E-05	3.929916442	0.01001222	OK	8.72E-05
CKMT2	0	51.50204809	0.13227409	OK	0
ZCCHC9	3.14E-20	9.139489473	0.0234276	OK	9.25E-20
SSBP2	3.66E-261	34.5026221	0.08865301	OK	2.51E-260
RPS23	0	191.816125	0.49314741	OK	0
ATP6AP1L	1.30E-90	20.15161919	0.05169447	OK	5.98E-90
TMEM167A	0	71.56526484	0.18398545	OK	0
XRCC4	2.56E-68	17.42720101	0.04473334	OK	1.06E-67
VCAN	0	183.8384877	0.47274146	OK	0
HAPLN1	1.54E-167	27.56308433	0.07069157	OK	8.91E-167
EDIL3	0	192.9840139	0.49623674	OK	0
AC113383.1	7.45E-19	8.790437202	0.02248064	OK	2.14E-18
COX7C	0	156.9989068	0.40369368	OK	0
RASA1	1.71E-13	7.27704943	0.01863501	OK	4.50E-13
CCNH	0	48.7236151	0.12523534	OK	0
LINC00461	4.69E-14	7.449254166	0.01895339	OK	1.25E-13
MEF2C	0	58.26913328	0.14979022	OK	0
MEF2C-AS1	3.08E-10	6.186337984	0.01580386	OK	7.56E-10
CETN3	2.36E-09	5.856965491	0.01498172	OK	5.65E-09
MBLAC2	0.00024748	3.483466658	0.00886306	OK	0.00048908
POLR3G	1.67E-05	4.148832309	0.01054852	OK	3.50E-05
LYSMD3	4.89E-06	4.421865003	0.0112951	OK	1.05E-05
ADGRV1	1.57E-20	9.214096238	0.02168171	OK	4.65E-20
LUCAT1	3.57E-216	31.35990131	0.0805232	OK	2.28E-215
ARRDC3	0	43.95467048	0.1129632	OK	0
NR2F1-AS1	5.04E-08	5.325284779	0.01348153	OK	1.16E-07

NR2F1	4.56E-14	7.453157061	0.01888537	OK	1.21E-13
FAM172A	5.29E-13	7.122850073	0.0182406	OK	1.38E-12
KIAA0825	0.00038009	3.366904094	0.00855582	OK	0.00074326
SLF1	5.41E-07	4.876002839	0.01245784	OK	1.20E-06
MCTP1	0	55.56409968	0.14279463	OK	0
TTC37	1.67E-20	9.207430643	0.0236041	OK	4.94E-20
ARSK	4.16E-28	10.92969939	0.02801192	OK	1.33E-27
RHOBTB3	4.58E-42	13.53935501	0.03474077	OK	1.64E-41
GLRX	0	126.5876586	0.32549885	OK	0
ELL2	0	54.54744608	0.14020703	OK	0
CAST	0	60.28239723	0.15496483	OK	0
ERAP1	0.00016044	3.597836165	0.0091734	OK	0.00032051
ERAP2	1.94E-20	9.191182501	0.02355306	OK	5.74E-20
LNPEP	0.00017802	3.570692281	0.00910815	OK	0.00035473
RIOK2	2.90E-08	5.424654091	0.01387405	OK	6.75E-08
CHD1	0	65.38500482	0.16808783	OK	0
FAM174A	1.42E-34	12.20730836	0.03131566	OK	4.82E-34
ST8SIA4	0	81.98953901	0.2107746	OK	0
PAM	0	67.96786697	0.17469875	OK	0
EFNA5	4.66E-21	9.34359506	0.0238913	OK	1.39E-20
AC008467.1	0.00014757	3.619534149	0.00908229	OK	0.00029536
PJA2	9.45E-220	31.6211679	0.08125338	OK	6.10E-219
MAN2A1	1.09E-15	7.931036014	0.02031732	OK	2.98E-15
TSLP	5.74E-10	6.087348994	0.01554426	OK	1.40E-09
CAMK4	1.63E-24	10.1515092	0.025895	OK	5.07E-24
STARD4	1.84E-12	6.949037574	0.01777692	OK	4.75E-12
NREP	3.11E-10	6.184567301	0.01582392	OK	7.64E-10
EPB41L4A	2.02E-09	5.882752845	0.0149969	OK	4.84E-09
EPB41L4A-AS1	0	49.05824847	0.12608517	OK	0
SRP19	2.52E-152	26.26333868	0.06747092	OK	1.40E-151
REEP5	0	91.72845748	0.23584521	OK	0
DCP2	1.90E-188	29.25565397	0.07516265	OK	1.15E-187
PGGT1B	3.05E-20	9.142628214	0.02343494	OK	8.99E-20
CCDC112	1.13E-171	27.90600475	0.07169226	OK	6.58E-171
FEM1C	9.63E-05	3.728583519	0.00950999	OK	0.00019444
AC010226.1	0.00020004	3.540038112	0.00901659	OK	0.00039745
TMED7	7.38E-53	15.2571122	0.03916204	OK	2.82E-52
CDO1	0	91.82859382	0.23601519	OK	0
ATG12	7.67E-28	10.87399512	0.02789156	OK	2.46E-27
AP3S1	0	39.4655288	0.10142688	OK	0
LVRN	7.76E-06	4.321211044	0.01072923	OK	1.65E-05
COMMD10	4.12E-104	21.63598213	0.05556581	OK	1.99E-103
SEMA6A	1.02E-34	12.23407022	0.03095063	OK	3.47E-34
TNFAIP8	0	48.52345467	0.1247181	OK	0
HSD17B4	0	44.27300801	0.11378613	OK	0
PRR16	2.15E-55	15.63352776	0.04006836	OK	8.34E-55
AC114284.1	0.00013033	3.65155956	0.00921202	OK	0.00026147
SRFBP1	2.31E-48	14.56601864	0.03738553	OK	8.64E-48
LOX	9.12E-169	27.66536569	0.0709359	OK	5.28E-168
SNCAIP	1.16E-15	7.923401614	0.02025495	OK	3.16E-15
LINC02201	5.11E-10	6.105858395	0.01509868	OK	1.25E-09
SNX2	0	100.7754229	0.25911474	OK	0
SNX24	1.26E-194	29.73732301	0.07638942	OK	7.76E-194

PPIC	0	104.21723	0.26793405	OK	0
PRDM6	0	87.35498049	0.22454307	OK	0
CEP120	1.02E-05	4.261295943	0.01087588	OK	2.15E-05
ZNF608	2.07E-07	5.06242908	0.01290477	OK	4.67E-07
GRAMD2B	4.81E-135	24.70424736	0.06342797	OK	2.56E-134
ALDH7A1	5.64E-44	13.85858792	0.03555716	OK	2.05E-43
PHAX	3.84E-103	21.53278344	0.05530202	OK	1.85E-102
LMNB1	0	71.11417323	0.18269611	OK	0
MARCH3	9.86E-19	8.758848217	0.02241011	OK	2.83E-18
PRRC1	1.41E-10	6.308769242	0.01614684	OK	3.49E-10
LINC01184	0.00035226	3.387813829	0.00863506	OK	0.00069038
ISOC1	4.13E-19	8.856326077	0.02269302	OK	1.20E-18
CHSY3	1.89E-20	9.194086626	0.02351254	OK	5.59E-20
HINT1	0	140.498033	0.36126819	OK	0
LYRM7	9.56E-07	4.762525381	0.01216837	OK	2.10E-06
CDC42SE2	0	39.83756086	0.10237555	OK	0
FNIP1	8.05E-46	14.16043832	0.03634269	OK	2.96E-45
P4HA2	2.01E-130	24.27054872	0.06233153	OK	1.05E-129
PDLIM4	0	60.62278518	0.15579588	OK	0
SLC22A4	3.16E-14	7.501192722	0.01914042	OK	8.46E-14
IRF1	0	55.08055356	0.14158559	OK	0
KIF3A	2.89E-26	10.53776333	0.0270156	OK	9.13E-26
SPET8	6.15E-55	15.56643967	0.0399399	OK	2.38E-54
UQCRQ	0	81.58393923	0.209754	OK	0
AFF4	4.30E-20	9.105263348	0.02334314	OK	1.26E-19
ZCCHC10	7.70E-31	11.48661847	0.02946461	OK	2.53E-30
HSPA4	4.38E-107	21.94945646	0.05637536	OK	2.14E-106
C5orf15	1.73E-33	12.002408	0.03079111	OK	5.82E-33
VDAC1	0	94.7199583	0.24354048	OK	0
TCF7	5.54E-10	6.093116543	0.01556952	OK	1.35E-09
SKP1	0	148.3291428	0.3814027	OK	0
PPP2CA	1.05E-227	32.19453369	0.08272475	OK	6.89E-227
UBE2B	0	60.07236691	0.15442802	OK	0
CDKN2AIPNL	1.55E-14	7.59428111	0.01945041	OK	4.16E-14
SAR1B	4.58E-38	12.84504746	0.03296035	OK	1.60E-37
SEC24A	3.24E-05	3.994694598	0.01019063	OK	6.69E-05
CAMLG	0	46.38498978	0.11922266	OK	0
DDX46	1.51E-91	20.25792394	0.05202787	OK	6.98E-91
C5orf24	3.18E-265	34.77236747	0.08935194	OK	2.20E-264
TXNDC15	1.71E-56	15.79409539	0.04054409	OK	6.67E-56
PCBD2	5.12E-22	9.574444493	0.02453349	OK	1.55E-21
H2AFY	0	194.9850838	0.50141598	OK	0
TIFAB	1.93E-29	11.20489929	0.02867974	OK	6.27E-29
AC022092.1	9.07E-280	35.72178647	0.09160043	OK	6.38E-279
CXCL14	0	49.97092662	0.12752698	OK	0
TGFBI	0	97.16316542	0.24981286	OK	0
SMAD5	1.08E-35	12.41517618	0.03185152	OK	3.70E-35
SPOCK1	7.25E-17	8.260511153	0.02108943	OK	2.03E-16
KLHL3	0.00015302	3.610127096	0.00917041	OK	0.00030603
HNRNPA0	0	40.46978367	0.10401158	OK	0
MYOT	3.16E-20	9.138813031	0.02175373	OK	9.31E-20
FAM13B	1.40E-08	5.553179244	0.01420484	OK	3.29E-08
BRD8	5.18E-10	6.103808901	0.01562001	OK	1.26E-09

KIF20A	0	69.67647323	0.17566451	OK	0
CDC25C	0	53.03522114	0.13282197	OK	0
FAM53C	9.37E-297	36.79958364	0.09455532	OK	6.75E-296
KDM3B	3.16E-06	4.515717014	0.01153478	OK	6.81E-06
REEP2	1.30E-41	13.46275436	0.03447246	OK	4.64E-41
EGR1	0	82.44305229	0.21195513	OK	0
ETF1	0	61.46162004	0.15799714	OK	0
HSPA9	4.74E-155	26.50086716	0.0680833	OK	2.66E-154
CTNNA1	1.66E-93	20.47900134	0.05259529	OK	7.71E-93
SIL1	2.71E-49	14.7117004	0.03776028	OK	1.02E-48
PAIP2	0	53.6595756	0.13793519	OK	0
DNAJC18	1.73E-42	13.61069763	0.03489834	OK	6.23E-42
ECSCR	0	133.2230279	0.34086385	OK	0
TMEM173	0	42.95413391	0.11039436	OK	0
UBE2D2	0	69.26716585	0.17807712	OK	0
AC113361.1	2.46E-08	5.453953823	0.01023876	OK	5.74E-08
CXXC5	0	68.50808189	0.17611115	OK	0
PURA	1.30E-162	27.14901011	0.06974888	OK	7.41E-162
CYSTM1	0	97.14197886	0.2497683	OK	0
PFDN1	5.71E-164	27.26377526	0.07004533	OK	3.26E-163
HBEGF	0	138.1911493	0.35532205	OK	0
ANKHD1	1.21E-05	4.221319502	0.01077516	OK	2.56E-05
SRA1	0	69.82488724	0.17950762	OK	0
APBB3	5.04E-13	7.129377887	0.01825016	OK	1.32E-12
SLC35A4	3.06E-27	10.74698965	0.02755573	OK	9.76E-27
CD14	0	322.9285078	0.83048188	OK	0
NDUFA2	0	70.43269427	0.18107482	OK	0
IK	0	42.47013821	0.10915629	OK	0
WDR55	4.68E-12	6.816146946	0.01745133	OK	1.20E-11
HARS	3.65E-48	14.53472005	0.03730154	OK	1.36E-47
ZMAT2	2.41E-166	27.46328561	0.07055737	OK	1.39E-165
PCDHB4	2.31E-44	13.92256312	0.03566472	OK	8.42E-44
PCDHB7	1.22E-09	5.96603044	0.01518591	OK	2.94E-09
PCDHB16	8.20E-13	7.062115715	0.01801724	OK	2.14E-12
PCDHB10	9.39E-06	4.278932138	0.01084609	OK	1.99E-05
PCDHB11	3.55E-24	10.07546406	0.02573255	OK	1.10E-23
PCDHB14	6.58E-05	3.823381931	0.00971545	OK	0.00013398
PCDHB15	1.76E-09	5.904889954	0.01501128	OK	4.24E-09
TAF7	3.45E-131	24.3428682	0.06253306	OK	1.81E-130
PCDHGA10	7.45E-27	10.66462594	0.02729881	OK	2.37E-26
PCDHGB7	0.00015724	3.603074034	0.00912699	OK	0.00031429
DIAPH1	9.85E-48	14.46658379	0.0371245	OK	3.67E-47
HDAC3	1.37E-32	11.82962098	0.03034729	OK	4.59E-32
ARAP3	4.23E-07	4.924309491	0.0125161	OK	9.45E-07
PCDH1	5.82E-94	20.52991061	0.05245015	OK	2.71E-93
DELE1	2.44E-07	5.030932113	0.01286067	OK	5.50E-07
PCDH12	3.71E-87	19.75392011	0.0505984	OK	1.68E-86
RNF14	5.95E-09	5.701270176	0.01458421	OK	1.41E-08
GNPDA1	0	70.90565452	0.18228247	OK	0
NDFIP1	0	46.98237895	0.12076168	OK	0
SPRY4	8.62E-31	11.47687831	0.02937644	OK	2.83E-30
FGF1	2.37E-87	19.7765842	0.05070003	OK	1.07E-86
ARHGAP26	7.01E-264	34.68340457	0.08911328	OK	4.83E-263

NR3C1	1.87E-74	18.21769766	0.04678008	OK	7.97E-74
YIPF5	1.15E-58	16.10657334	0.04134705	OK	4.54E-58
PRELID2	8.03E-05	3.774177229	0.00962167	OK	0.00016279
LARS	4.51E-47	14.36149905	0.03686113	OK	1.67E-46
TCERG1	9.52E-06	4.275869042	0.01092156	OK	2.02E-05
PPP2R2B	2.74E-301	37.08195107	0.09523976	OK	1.99E-300
STK32A	5.54E-09	5.71327542	0.01448565	OK	1.32E-08
DPYSL3	0	50.86514347	0.13071111	OK	0
JAKMIP2	0	101.8978439	0.26187788	OK	0
SPINK1	4.72E-53	15.28624443	0.03898385	OK	1.81E-52
C5orf46	2.17E-260	34.45112651	0.08830061	OK	1.48E-259
AC011352.1	5.95E-22	9.558920055	0.02106156	OK	1.80E-21
SPINK9	9.38E-07	4.766438363	0.01147926	OK	2.06E-06
FBXO38	7.01E-08	5.26502694	0.01345823	OK	1.61E-07
ADRB2	4.16E-241	33.13838907	0.08512178	OK	2.77E-240
AFAP1L1	1.24E-18	8.732580804	0.02222906	OK	3.57E-18
IL17B	2.03E-25	10.35285616	0.0264741	OK	6.36E-25
AC131025.1	1.15E-28	11.04574301	0.02313297	OK	3.71E-28
CARMN	0	157.6266598	0.4052883	OK	0
CSNK1A1	0	39.16349985	0.10065188	OK	0
PPARGC1B	4.04E-44	13.88255054	0.03557241	OK	1.47E-43
SLC26A2	6.75E-07	4.832225387	0.01234768	OK	1.50E-06
HMGXB3	1.26E-46	14.29016329	0.03667387	OK	4.66E-46
CSF1R	0	194.7125459	0.50069596	OK	0
PDGFRB	0	87.57606438	0.2251211	OK	0
CAMK2A	3.16E-12	6.872392329	0.0158006	OK	8.12E-12
TCOF1	3.99E-06	4.465679276	0.01140547	OK	8.58E-06
CD74	0	312.8344432	0.8045181	OK	0
RPS14	0	193.2773415	0.49691089	OK	0
SYNPO	0	78.94931617	0.20292933	OK	0
RBM22	8.90E-79	18.7544906	0.04815897	OK	3.88E-78
DCTN4	1.74E-10	6.275896693	0.01606306	OK	4.30E-10
SMIM3	0	48.45458403	0.12453675	OK	0
GPX3	0	44.37326553	0.11403258	OK	0
TNIP1	1.11E-12	7.019393723	0.01797521	OK	2.89E-12
ANXA6	0	41.20891313	0.10590294	OK	0
CCDC69	6.09E-40	13.17523995	0.03379531	OK	2.15E-39
GM2A	0	59.35979493	0.15257316	OK	0
SLC36A1	7.37E-49	14.6438951	0.03754346	OK	2.76E-48
SPARC	0	172.2075696	0.44281126	OK	0
ATOX1	0	86.98629023	0.22364973	OK	0
G3BP1	2.52E-64	16.89326178	0.04337234	OK	1.03E-63
LINC01933	1.13E-17	8.479119901	0.01810011	OK	3.21E-17
NMUR2	1.12E-05	4.23900013	0.01068654	OK	2.37E-05
GRIA1	9.59E-14	7.354392217	0.018764	OK	2.54E-13
FAM114A2	5.30E-09	5.720768625	0.01463454	OK	1.26E-08
SAP30L	2.83E-14	7.515826668	0.01925068	OK	7.57E-14
LARP1	8.21E-35	12.25181221	0.03143317	OK	2.79E-34
FAXDC2	5.08E-84	19.38587142	0.04975168	OK	2.27E-83
CNOT8	6.37E-14	7.408896245	0.01897627	OK	1.69E-13
MRPL22	1.08E-173	28.07194883	0.0721214	OK	6.32E-173
SGCD	1.53E-125	23.80361846	0.06108308	OK	7.90E-125
TIMD4	1.96E-19	8.939405337	0.02265198	OK	5.68E-19

HAVCR2	0	130.1854856	0.33473732	OK	0
MED7	1.31E-13	7.312702317	0.01872671	OK	3.46E-13
ITK	1.87E-41	13.43571388	0.03434116	OK	6.68E-41
CYFIP2	0	44.27087663	0.11372905	OK	0
ADAM19	0	43.36776349	0.1112952	OK	0
AC106801.1	5.02E-12	6.805967551	0.01441234	OK	1.29E-11
THG1L	5.49E-08	5.309587603	0.0135761	OK	1.26E-07
CLINT1	1.42E-149	26.02136823	0.06684953	OK	7.82E-149
EBF1	0	62.14347577	0.15970712	OK	0
LINC02202	1.01E-41	13.48091215	0.03444536	OK	3.63E-41
RNF145	5.78E-277	35.54073337	0.09132604	OK	4.04E-276
UBLCP1	4.43E-11	6.485318694	0.01660101	OK	1.11E-10
TTC1	1.06E-205	30.58211731	0.07857983	OK	6.64E-205
PWWP2A	0.00036485	3.378168001	0.00861207	OK	0.00071411
C1QTNF2	0	48.95098706	0.12575942	OK	0
ZBED8	1.75E-12	6.955733383	0.01774494	OK	4.53E-12
SLU7	2.02E-62	16.63258025	0.04270134	OK	8.18E-62
PTTG1	0	87.7167119	0.22541269	OK	0
MIR3142HG	0.0001235	3.665348661	0.00927952	OK	0.00024812
GABRB2	1.93E-09	5.88975088	0.01495338	OK	4.65E-09
CCNG1	1.54E-148	25.92972562	0.06660373	OK	8.46E-148
NUDCD2	1.61E-122	23.50994315	0.06038818	OK	8.25E-122
HMMR	0	172.247604	0.44106499	OK	0
MAT2B	8.32E-11	6.389577824	0.01635544	OK	2.07E-10
RARS	4.81E-15	7.744269826	0.01983941	OK	1.30E-14
SLIT3	0	60.68926019	0.15594936	OK	0
SPDL1	1.82E-30	11.41218943	0.02923931	OK	5.95E-30
DOCK2	0	80.61100417	0.20722914	OK	0
LCP2	0	94.88626768	0.2439409	OK	0
KCNMB1	0	96.82334409	0.24892018	OK	0
NPM1	0	146.4413207	0.37654653	OK	0
FGF18	0.00022312	3.511113394	0.00886298	OK	0.00044192
FBXW11	3.38E-15	7.788820838	0.01995601	OK	9.19E-15
STK10	0	37.96913838	0.09755369	OK	0
EFCAB9	3.94E-11	6.503012925	0.01579858	OK	9.87E-11
UBTD2	4.80E-06	4.426159781	0.01130319	OK	1.03E-05
SH3PXD2B	9.83E-08	5.202535701	0.01329606	OK	2.24E-07
NEURL1B	7.98E-245	33.39544307	0.08549465	OK	5.35E-244
DUSP1	0	65.97800382	0.1696086	OK	0
ERGIC1	5.34E-25	10.26000553	0.02631026	OK	1.67E-24
RPL26L1	5.52E-236	32.78087479	0.08423181	OK	3.66E-235
ATP6V0E1	0	148.1812639	0.3810296	OK	0
CREBRF	2.79E-51	15.01818865	0.03854963	OK	1.06E-50
BNIP1	3.30E-08	5.401922688	0.01380891	OK	7.65E-08
STC2	1.25E-07	5.157150507	0.01291943	OK	2.85E-07
BOD1	8.56E-137	24.86657066	0.0638698	OK	4.58E-136
CPEB4	0	43.85854261	0.11271905	OK	0
MSX2	3.42E-154	26.42621508	0.06779117	OK	1.91E-153
DRD1	5.56E-21	9.324830417	0.02373081	OK	1.65E-20
SFXN1	5.39E-06	4.400850932	0.01123969	OK	1.15E-05
HRH2	2.27E-248	33.63890468	0.08642954	OK	1.53E-247
THOC3	6.78E-11	6.420726231	0.01643074	OK	1.69E-10
KIAA1191	1.17E-11	6.682721219	0.01710879	OK	2.98E-11

NOP16	3.78E-162	27.10977016	0.06962759	OK	2.15E-161
HIGD2A	0	124.0496253	0.31897493	OK	0
CLTB	0	55.46300502	0.14257315	OK	0
FAF2	1.55E-11	6.642026979	0.01700537	OK	3.92E-11
RNF44	4.80E-17	8.309557045	0.02128048	OK	1.34E-16
TSPAN17	2.01E-11	6.603032649	0.01689332	OK	5.08E-11
HK3	8.58E-201	30.21066002	0.07754101	OK	5.33E-200
MXD3	1.64E-188	29.26074838	0.07480947	OK	9.92E-188
PRELID1	0	112.1154267	0.28828013	OK	0
LMAN2	0	62.70152439	0.16119037	OK	0
RGS14	3.57E-82	19.16580631	0.04918895	OK	1.58E-81
GRK6	2.28E-94	20.57540247	0.05282981	OK	1.06E-93
PRR7	2.44E-06	4.569574813	0.01166329	OK	5.30E-06
DBN1	2.89E-254	34.03960086	0.08741477	OK	1.97E-253
PDLIM7	0	115.5122527	0.29699974	OK	0
DOK3	0	37.55972342	0.09649046	OK	0
DDX41	1.44E-15	7.89568441	0.02022705	OK	3.95E-15
TMED9	0	60.2309214	0.15483615	OK	0
B4GALT7	9.86E-18	8.495483696	0.02177126	OK	2.79E-17
RMND5B	8.47E-14	7.370951154	0.01887564	OK	2.25E-13
NHP2	0	54.12997578	0.1391439	OK	0
HNRNPAB	0	75.27780478	0.19353532	OK	0
PHYKPL	3.09E-25	10.31270292	0.02644475	OK	9.66E-25
COL23A1	5.82E-33	11.90135361	0.03008778	OK	1.95E-32
ZNF354C	6.67E-05	3.820142298	0.00971245	OK	0.00013571
ADAMTS2	1.50E-20	9.218651302	0.02355205	OK	4.45E-20
RUFY1	7.71E-96	20.73896785	0.05325798	OK	3.61E-95
HNRNPH1	2.01E-11	6.603565622	0.01690995	OK	5.07E-11
CANX	0	69.63262496	0.1790166	OK	0
LTC4S	0	118.4951956	0.30465828	OK	0
MGAT4B	3.72E-189	29.31135626	0.07530412	OK	2.25E-188
SQSTM1	0	71.54725255	0.18393581	OK	0
MRNIP	7.77E-82	19.12532376	0.04909462	OK	3.44E-81
AC008393.1	3.54E-05	3.973394771	0.01009282	OK	7.30E-05
TBC1D9B	1.81E-69	17.57829161	0.04512453	OK	7.53E-69
RNF130	0	176.9526728	0.45503921	OK	0
MAPK9	1.08E-10	6.349197782	0.01624934	OK	2.69E-10
GFPT2	3.15E-40	13.2248348	0.03369598	OK	1.12E-39
MGAT1	0	113.4648282	0.29174781	OK	0
LINC00847	7.19E-66	17.10184862	0.0438949	OK	2.95E-65
TRIM7	3.65E-05	3.965988966	0.01010316	OK	7.53E-05
RACK1	0	185.9218424	0.47803114	OK	0
DUSP22	4.74E-33	11.91858336	0.030575	OK	1.59E-32
IRF4	2.45E-258	34.31366408	0.08802941	OK	1.68E-257
FOXF2	2.66E-15	7.818993801	0.01867532	OK	7.25E-15
FOXC1	0	148.1298703	0.38087731	OK	0
GMDS	0	144.0478306	0.37037176	OK	0
WRNIP1	3.29E-05	3.990702143	0.01018509	OK	6.80E-05
SERPINB1	0	140.5129884	0.36131707	OK	0
SERPINB9P1	2.08E-06	4.603703161	0.01172953	OK	4.52E-06
SERPINB9	0	117.9490525	0.3032469	OK	0
SERPINB6	0	40.48146479	0.1040418	OK	0
NQO2	0	42.43764887	0.1090691	OK	0

AL133351.1	1.45E-14	7.603068002	0.01928126	OK	3.89E-14
RIPK1	9.01E-09	5.63002836	0.01440091	OK	2.13E-08
TUBB2A	8.64E-103	21.49514889	0.05520534	OK	4.15E-102
TUBB2B	1.43E-11	6.653267224	0.0168139	OK	3.63E-11
PSMG4	2.07E-61	16.49260795	0.04233787	OK	8.34E-61
SLC22A23	3.08E-18	8.629479508	0.0220238	OK	8.80E-18
PXDC1	0	47.37121513	0.12175191	OK	0
AL391422.3	2.21E-05	4.084867652	0.01041111	OK	4.59E-05
FAM50B	6.59E-90	20.07122068	0.05152188	OK	3.01E-89
AL590004.4	3.88E-06	4.471740301	0.01114991	OK	8.34E-06
PRPF4B	3.93E-07	4.938705887	0.01262798	OK	8.79E-07
ECI2	2.19E-62	16.62792012	0.04268729	OK	8.84E-62
CDYL	2.02E-08	5.488745988	0.01403917	OK	4.73E-08
RPP40	3.18E-10	6.181234271	0.01580196	OK	7.80E-10
LYRM4	4.15E-47	14.36726606	0.03686728	OK	1.54E-46
FARS2	1.02E-21	9.502587082	0.02435216	OK	3.08E-21
NRN1	7.37E-110	22.23796811	0.05704808	OK	3.64E-109
F13A1	0	241.974921	0.62222431	OK	0
LY86	0	202.9763553	0.52195426	OK	0
RREB1	8.64E-40	13.14885354	0.03373083	OK	3.05E-39
SSR1	0	37.93438777	0.0974887	OK	0
RIOK1	6.61E-13	7.092023548	0.01816217	OK	1.72E-12
DSP	5.82E-32	11.70765263	0.02976963	OK	1.94E-31
SNRNP48	0.00010172	3.714717951	0.00947689	OK	0.00020523
BMP6	4.76E-10	6.117261437	0.01544777	OK	1.16E-09
BLOC1S5	2.74E-13	7.212932801	0.01846965	OK	7.20E-13
EEF1E1	1.39E-123	23.61384276	0.06065225	OK	7.12E-123
TFAP2A	4.58E-10	6.123418455	0.01562631	OK	1.12E-09
GCNT2	8.59E-09	5.638210094	0.01441684	OK	2.03E-08
PAK1IP1	6.05E-49	14.65733554	0.03761736	OK	2.27E-48
TMEM14C	0	107.6840746	0.2768822	OK	0
TMEM14B	0	59.99472858	0.154229	OK	0
SMIM13	8.64E-26	10.43432739	0.02674912	OK	2.72E-25
NEDD9	4.91E-128	24.04325637	0.06175353	OK	2.56E-127
TMEM170B	5.81E-49	14.6600261	0.03760326	OK	2.18E-48
ADTRP	1.85E-10	6.26604398	0.01594503	OK	4.57E-10
HIVEP1	3.04E-05	4.009794429	0.0102361	OK	6.28E-05
EDN1	4.08E-27	10.7204404	0.02743338	OK	1.30E-26
PHACTR1	0	117.4325983	0.30194675	OK	0
TBC1D7	2.87E-141	25.27691716	0.06491858	OK	1.55E-140
GFOD1	2.73E-22	9.63904219	0.02469244	OK	8.29E-22
NOL7	0	60.62461468	0.15584869	OK	0
RANBP9	4.53E-05	3.914702946	0.0099914	OK	9.28E-05
MCUR1	2.03E-181	28.69791002	0.07373097	OK	1.21E-180
CD83	0	279.3372384	0.71836375	OK	0
JARID2	2.02E-247	33.5738644	0.08625303	OK	1.36E-246
DTNBP1	4.09E-108	22.0570404	0.05664957	OK	2.00E-107
MYLIP	8.48E-28	10.86484382	0.02786593	OK	2.72E-27
GMPR	6.25E-57	15.8573887	0.04068617	OK	2.45E-56
ATXN1	1.72E-08	5.517759928	0.01411502	OK	4.02E-08
RBM24	0	57.57026834	0.14763109	OK	0
CAP2	0	74.28791579	0.19092457	OK	0
FAM8A1	2.86E-16	8.095303924	0.02073058	OK	7.91E-16

AL138724.1	0.00024169	3.489803895	0.00887297	OK	0.00047797
KIF13A	5.29E-80	18.90400872	0.04853683	OK	2.32E-79
TPMT	3.13E-296	36.76678767	0.09447515	OK	2.25E-295
KDM1B	1.27E-14	7.619700545	0.01951263	OK	3.42E-14
DEK	0	83.60669407	0.21495523	OK	0
RNF144B	0	85.86020429	0.22071161	OK	0
ID4	0	192.7410122	0.49561609	OK	0
E2F3	0.00012858	3.655013386	0.00932202	OK	0.00025805
SOX4	1.24E-41	13.46627804	0.0345566	OK	4.43E-41
CASC15	1.50E-08	5.541330166	0.01410898	OK	3.52E-08
GPLD1	7.01E-12	6.757632229	0.01723234	OK	1.79E-11
TDP2	0	59.16279859	0.15206763	OK	0
ACOT13	0	43.83097199	0.11264794	OK	0
C6orf62	2.25E-255	34.11443137	0.0876612	OK	1.53E-254
AL133264.2	4.27E-05	3.928569853	0.00997502	OK	8.77E-05
GMNN	1.44E-206	30.64727203	0.07872199	OK	9.04E-206
RIPOR2	1.24E-173	28.06688476	0.07202538	OK	7.28E-173
CARMIL1	3.90E-30	11.34564883	0.02909396	OK	1.27E-29
TRIM38	1.71E-58	16.08191425	0.04128523	OK	6.76E-58
HIST1H1A	2.11E-45	14.09272262	0.03611552	OK	7.72E-45
HIST1H3B	0	134.8018059	0.34248483	OK	0
HIST1H2BB	6.40E-05	3.830432659	0.00954889	OK	0.00013032
HIST1H3C	3.40E-181	28.67983459	0.06716565	OK	2.03E-180
HIST1H1C	2.90E-98	21.0058457	0.05394114	OK	1.37E-97
HFE	1.24E-08	5.575348649	0.01424386	OK	2.90E-08
HIST1H4C	0	64.97962103	0.16704612	OK	0
HIST1H2BC	1.41E-08	5.552547283	0.01416983	OK	3.30E-08
HIST1H2AC	6.34E-10	6.071494979	0.01553195	OK	1.54E-09
HIST1H1E	2.71E-94	20.56706374	0.05281188	OK	1.26E-93
AL353759.1	2.32E-06	4.580797408	0.01087395	OK	5.03E-06
HIST1H2BF	8.30E-60	16.26819971	0.04131161	OK	3.31E-59
HIST1H4E	0.00047287	3.306189431	0.00840999	OK	0.00091842
HIST1H1D	3.93E-250	33.75909381	0.08669618	OK	2.66E-249
HIST1H3F	1.14E-35	12.4108857	0.031065	OK	3.90E-35
HIST1H2BH	5.97E-128	24.03511814	0.06143753	OK	3.11E-127
HIST1H3G	0	100.3521961	0.25464599	OK	0
BTN3A2	7.81E-38	12.80373164	0.03284773	OK	2.71E-37
BTN2A2	4.61E-98	20.98382483	0.05387654	OK	2.18E-97
BTN3A1	0.00026741	3.462683004	0.00882425	OK	0.00052732
BTN3A3	2.71E-06	4.548225489	0.01160955	OK	5.86E-06
BTN2A1	2.66E-20	9.15726714	0.0234696	OK	7.85E-20
HCG11	9.66E-09	5.618033762	0.01436314	OK	2.28E-08
ABT1	3.25E-22	9.621340918	0.02466514	OK	9.84E-22
ZNF322	4.54E-70	17.65642956	0.04532664	OK	1.90E-69
HIST1H2AG	3.69E-108	22.06168947	0.05645001	OK	1.81E-107
HIST1H2AH	0	49.77797465	0.12644904	OK	0
HIST1H2BL	1.55E-25	10.37897944	0.02572127	OK	4.85E-25
HIST1H2AJ	1.97E-234	32.67173678	0.08090242	OK	1.30E-233
HIST1H2AK	4.06E-08	5.364478833	0.01363897	OK	9.39E-08
HIST1H2AL	0	38.13204642	0.0975586	OK	0
HIST1H1B	0	158.3420085	0.40526554	OK	0
HIST1H3J	2.17E-06	4.594316716	0.01023842	OK	4.72E-06
HIST1H2AM	1.62E-17	8.437341824	0.02114863	OK	4.58E-17

AL121944.1	4.40E-05	3.921374103	0.0099985	OK	9.03E-05
ZSCAN16-AS1	4.20E-57	15.88234166	0.04076932	OK	1.65E-56
ZSCAN26	2.09E-17	8.407921611	0.02153381	OK	5.88E-17
ZSCAN31	6.35E-09	5.690073192	0.01451646	OK	1.50E-08
ZBED9	6.78E-18	8.538742724	0.02030054	OK	1.93E-17
TRIM27	3.88E-23	9.837646363	0.02522252	OK	1.19E-22
GABBR1	6.57E-13	7.09292168	0.01815107	OK	1.71E-12
HLA-F	0	41.53524036	0.10674938	OK	0
HLA-A	0	180.6343668	0.46446652	OK	0
ZNRD1	1.34E-59	16.23861913	0.04168601	OK	5.36E-59
PPP1R11	1.84E-111	22.40304771	0.05754171	OK	9.10E-111
TRIM26	2.76E-12	6.891531662	0.01764099	OK	7.11E-12
HLA-E	0	162.2593782	0.41723691	OK	0
GNL1	4.61E-20	9.097760779	0.02331959	OK	1.35E-19
ABCF1	3.96E-82	19.16047277	0.04920328	OK	1.75E-81
PPP1R10	4.51E-196	29.84909417	0.07669461	OK	2.78E-195
MRPS18B	8.82E-94	20.50969814	0.05267147	OK	4.10E-93
C6orf136	1.07E-05	4.249440915	0.01084586	OK	2.26E-05
PPP1R18	0	81.89085718	0.21053827	OK	0
NRM	1.53E-23	9.930447305	0.02545489	OK	4.72E-23
TUBB	0	85.00967442	0.21856432	OK	0
FLOT1	0	53.03526821	0.13632799	OK	0
IER3	0	202.5134076	0.52077656	OK	0
LINC00243	0.00011474	3.684139508	0.00935757	OK	0.00023091
HCG22	9.52E-12	6.713213742	0.01346202	OK	2.42E-11
PSORS1C1	6.78E-07	4.831386174	0.0122335	OK	1.50E-06
TCF19	1.39E-110	22.31269613	0.05716478	OK	6.87E-110
HLA-C	0	164.7447501	0.4236077	OK	0
HLA-B	0	220.3237881	0.56655755	OK	0
MICA	3.80E-06	4.476377229	0.01143218	OK	8.17E-06
MICB	4.48E-09	5.749466782	0.01468794	OK	1.07E-08
NFKBIL1	8.08E-35	12.2531167	0.03143254	OK	2.75E-34
TNF	0	51.30985383	0.13184649	OK	0
LTB	0	52.35079826	0.13449393	OK	0
LST1	0	237.1476499	0.6098513	OK	0
NCR3	9.04E-06	4.287399705	0.01086897	OK	1.92E-05
AIF1	0	347.0324338	0.89248112	OK	0
PRRC2A	0.0004334	3.3305311	0.00848906	OK	0.00084475
BAG6	6.10E-17	8.281227337	0.02122062	OK	1.70E-16
GPANK1	1.05E-16	8.216055349	0.02105217	OK	2.93E-16
CSNK2B	0	74.78901185	0.19227881	OK	0
DDAH2	0	56.44169991	0.14509045	OK	0
CLIC1	0	206.3952619	0.53073802	OK	0
VARS	8.73E-27	10.64987465	0.02729664	OK	2.77E-26
LSM2	2.07E-298	36.90294298	0.09483617	OK	1.49E-297
HSPA1A	0	86.00949127	0.22113457	OK	0
HSPA1B	0	70.11358487	0.18025179	OK	0
C6orf48	3.73E-238	32.93278096	0.08462245	OK	2.48E-237
NEU1	0	50.85855454	0.13072213	OK	0
EHMT2	0.00034886	3.390470565	0.00864223	OK	0.00068379
C2	0	118.7259538	0.3052074	OK	0
NELFE	1.73E-93	20.47702299	0.05258837	OK	8.03E-93
STK19	1.19E-05	4.225752475	0.01079097	OK	2.51E-05

TNXB	0	100.9193055	0.25915339	OK	0
FKBPL	7.88E-08	5.24348844	0.01340171	OK	1.80E-07
PRRT1	6.94E-31	11.49557284	0.02940906	OK	2.28E-30
AL662884.4	7.30E-05	3.797792925	0.0096337	OK	0.00014833
AGPAT1	2.00E-09	5.88420218	0.01505435	OK	4.80E-09
RNF5	2.64E-134	24.6353024	0.06328312	OK	1.40E-133
GPSM3	0	193.5142888	0.4976295	OK	0
NOTCH4	1.46E-11	6.650402342	0.01700029	OK	3.71E-11
HLA-DRA	0	319.9201463	0.82274325	OK	0
HLA-DRB5	0	325.3262389	0.83664858	OK	0
HLA-DRB1	0	326.3807503	0.83935993	OK	0
HLA-DQA1	0	314.131343	0.80784795	OK	0
HLA-DQB1	0	318.5449947	0.81920258	OK	0
HLA-DQA2	0	82.98045523	0.21330117	OK	0
HLA-DQB2	0	54.40685676	0.1397683	OK	0
HLA-DOB	0	101.9458209	0.2615678	OK	0
TAP2	2.97E-81	19.05531826	0.04890886	OK	1.31E-80
PSMB8	0	83.33045192	0.21424629	OK	0
PSMB8-AS1	1.33E-222	31.82788488	0.08176398	OK	8.63E-222
PSMB9	0	90.36192965	0.23232662	OK	0
TAP1	0	40.42031195	0.10386494	OK	0
HLA-DMB	0	287.4583403	0.7392421	OK	0
HLA-DMA	0	312.3552679	0.80328564	OK	0
BRD2	2.49E-95	20.68239806	0.05311816	OK	1.17E-94
HLA-DOA	0	110.8287021	0.28491627	OK	0
HLA-DPA1	0	321.3882016	0.8265183	OK	0
HLA-DPB1	0	314.2027761	0.80803804	OK	0
SLC39A7	3.43E-38	12.86756165	0.03301608	OK	1.19E-37
HSD17B8	1.54E-10	6.294676071	0.01610595	OK	3.81E-10
RING1	1.61E-06	4.656857254	0.01190023	OK	3.51E-06
AL645940.1	1.67E-151	26.19134497	0.06059743	OK	9.25E-151
RPS18	0	185.5058732	0.47694122	OK	0
B3GALT4	4.84E-15	7.743329866	0.0198355	OK	1.31E-14
WDR46	1.38E-14	7.609452635	0.01949298	OK	3.70E-14
PFDN6	1.74E-46	14.26750426	0.03661721	OK	6.45E-46
RGL2	2.45E-09	5.850277232	0.01496776	OK	5.88E-09
TAPBP	5.16E-293	36.56495809	0.09396629	OK	3.69E-292
DAXX	2.67E-13	7.216197043	0.01848025	OK	7.03E-13
KIFC1	0	66.96203322	0.17117621	OK	0
PHF1	1.99E-48	14.57613207	0.03740715	OK	7.45E-48
CUTA	0	60.83281019	0.15638416	OK	0
BAK1	6.53E-71	17.7655582	0.04560122	OK	2.74E-70
ITPR3	3.69E-60	16.31767667	0.04154824	OK	1.48E-59
UQCC2	0	37.80487112	0.0971529	OK	0
IP6K3	2.13E-14	7.552520892	0.01864882	OK	5.72E-14
HMGA1	0	144.8281675	0.37239668	OK	0
SMIM29	3.82E-265	34.76715093	0.08933669	OK	2.64E-264
NUDT3	2.40E-12	6.911182086	0.01769777	OK	6.20E-12
RPS10	0	116.1191381	0.29857247	OK	0
C6orf106	3.35E-11	6.527195282	0.01670959	OK	8.42E-11
AL451165.2	1.18E-06	4.719014067	0.0120525	OK	2.60E-06
SNRPC	0	52.19093074	0.13415714	OK	0
UHRF1BP1	1.38E-17	8.456256466	0.02165119	OK	3.90E-17

TAF11	1.49E-25	10.38265587	0.02662647	OK	4.67E-25
ANKS1A	8.19E-82	19.12260439	0.04909206	OK	3.62E-81
SCUBE3	0	39.77654544	0.10216152	OK	0
DEF6	0	44.60075013	0.11460459	OK	0
PPARD	2.86E-07	5.000602887	0.01277591	OK	6.42E-07
RPL10A	0	175.5727623	0.45143787	OK	0
TEAD3	3.82E-122	23.47329481	0.06023383	OK	1.95E-121
FKBP5	1.24E-302	37.16539445	0.09550787	OK	8.98E-302
SRPK1	9.38E-55	15.53941854	0.03988546	OK	3.63E-54
MAPK13	0	85.98725218	0.22099376	OK	0
ETV7	2.77E-10	6.202869983	0.01585329	OK	6.82E-10
KCTD20	1.48E-46	14.27912788	0.03664616	OK	5.46E-46
STK38	4.54E-11	6.48148013	0.01658832	OK	1.14E-10
SRSF3	0	39.02758799	0.10030225	OK	0
CDKN1A	0	84.65650889	0.21765363	OK	0
PPIL1	2.88E-10	6.196626989	0.0158547	OK	7.09E-10
C6orf89	8.84E-20	9.026721036	0.02313951	OK	2.58E-19
PI16	0	93.41990408	0.23918804	OK	0
MTCH1	0	43.25925962	0.11118578	OK	0
FGD2	0	68.00984268	0.17479176	OK	0
PIM1	1.95E-203	30.41118535	0.07812581	OK	1.22E-202
TMEM217	6.18E-06	4.371029262	0.01110951	OK	1.32E-05
RNF8	2.87E-09	5.824056188	0.01490131	OK	6.87E-09
CCDC167	0	40.82527264	0.1049099	OK	0
ZFAND3	8.13E-14	7.376368894	0.0188945	OK	2.16E-13
GLO1	1.37E-269	35.06010626	0.09009398	OK	9.49E-269
SAYSD1	1.55E-12	6.973323699	0.01785267	OK	4.01E-12
KCNK17	4.19E-302	37.13259964	0.09532898	OK	3.03E-301
DAAM2	1.01E-77	18.6248816	0.04777085	OK	4.38E-77
MOCS1	1.15E-27	10.83724453	0.02772844	OK	3.67E-27
UNC5CL	0.00026556	3.464545872	0.00859157	OK	0.00052387
OARD1	1.20E-09	5.967814975	0.01527214	OK	2.91E-09
TREML1	3.17E-80	18.93098584	0.04823777	OK	1.39E-79
TREM2	0	137.1076249	0.35251931	OK	0
TREM1	0	173.9779787	0.44734104	OK	0
MDFI	1.67E-11	6.630369886	0.01635524	OK	4.24E-11
TFEB	1.50E-14	7.598237726	0.01946025	OK	4.03E-14
BYSL	3.93E-06	4.469094295	0.01140344	OK	8.44E-06
CCND3	1.38E-192	29.57934723	0.07599184	OK	8.41E-192
MRPS10	5.75E-53	15.27345804	0.03920479	OK	2.20E-52
TRERF1	4.42E-07	4.916052505	0.01254847	OK	9.85E-07
TBCC	1.01E-15	7.93987083	0.02034205	OK	2.77E-15
RPL7L1	5.25E-45	14.02798607	0.03600349	OK	1.92E-44
C6orf226	4.50E-17	8.3174002	0.02131269	OK	1.26E-16
PTCRA	2.33E-41	13.41934113	0.03435451	OK	8.32E-41
CNPY3	0	119.843804	0.3081541	OK	0
MEA1	5.32E-206	30.60458969	0.07863693	OK	3.34E-205
KLHDC3	2.76E-31	11.57501859	0.0296909	OK	9.10E-31
RRP36	9.00E-54	15.39384626	0.03951376	OK	3.46E-53
MRPL2	4.07E-91	20.20916781	0.05189461	OK	1.87E-90
DNPH1	0	80.76774948	0.20765236	OK	0
CRIP3	0	46.94333235	0.11944898	OK	0
POLR1C	1.34E-07	5.144026068	0.01314744	OK	3.06E-07

YIPF3	3.93E-170	27.77866705	0.07136913	OK	2.28E-169
GTPBP2	5.54E-05	3.865534689	0.00985338	OK	0.00011319
MAD2L1BP	4.46E-32	11.73029094	0.03008435	OK	1.48E-31
RSPH9	2.74E-08	5.434803677	0.01383697	OK	6.38E-08
MRPS18A	2.60E-46	14.23950203	0.0365426	OK	9.61E-46
VEGFA	0	80.51051313	0.20697529	OK	0
AL109615.3	1.47E-05	4.177468333	0.01044934	OK	3.10E-05
C6orf223	3.08E-20	9.141603125	0.02324324	OK	9.07E-20
MRPL14	0	67.55603916	0.17367288	OK	0
SLC29A1	2.21E-110	22.2919997	0.05724781	OK	1.09E-109
HSP90AB1	0	159.5579428	0.4102477	OK	0
SLC35B2	1.57E-13	7.288418615	0.01866425	OK	4.14E-13
NFKBIE	0	47.8152852	0.12287824	OK	0
CDC5L	5.69E-88	19.84836571	0.05097322	OK	2.59E-87
RUNX2	1.66E-13	7.280996499	0.01862708	OK	4.37E-13
CLIC5	3.95E-07	4.937700284	0.01252841	OK	8.84E-07
AL035701.1	2.91E-08	5.424006584	0.01327941	OK	6.77E-08
ENPP4	0.00035491	3.385756678	0.00862994	OK	0.00069521
ENPP5	2.16E-09	5.871634751	0.01494599	OK	5.18E-09
RCAN2	0	183.7527754	0.47248798	OK	0
CYP39A1	2.03E-07	5.066349364	0.01287047	OK	4.58E-07
SLC25A27	8.40E-05	3.762724822	0.00954765	OK	0.00017021
TDRD6	0.00016411	3.591941616	0.00894835	OK	0.00032767
PLA2G7	0	111.6823334	0.28711361	OK	0
ADGRF5	1.92E-53	15.34460604	0.03915764	OK	7.38E-53
TNFRSF21	1.26E-211	31.02439577	0.07968207	OK	8.05E-211
CD2AP	4.92E-45	14.03269275	0.03601098	OK	1.80E-44
PTCHD4	0.00046785	3.309177968	0.00837451	OK	0.00090898
MUT	3.57E-16	8.068249835	0.02066536	OK	9.86E-16
CENPQ	9.39E-23	9.748191972	0.02498063	OK	2.86E-22
MCM3	7.46E-55	15.55414377	0.03991208	OK	2.89E-54
PAQR8	1.22E-13	7.321725027	0.01873041	OK	3.24E-13
EFHC1	2.44E-06	4.570203542	0.01166961	OK	5.28E-06
TRAM2	2.81E-10	6.200574614	0.01585633	OK	6.91E-10
TMEM14A	0	62.83351113	0.16149785	OK	0
GSTA4	0	38.08331804	0.09781844	OK	0
ICK	5.13E-72	17.90779097	0.04596986	OK	2.16E-71
FBXO9	1.73E-57	15.93798228	0.04091434	OK	6.78E-57
ELOVL5	4.00E-75	18.30189974	0.04698453	OK	1.71E-74
GCLC	1.28E-21	9.479593078	0.02429054	OK	3.83E-21
LRRC1	5.95E-05	3.848050683	0.0097972	OK	0.00012143
HMGCLL1	3.65E-06	4.484465354	0.01116356	OK	7.87E-06
BMP5	6.08E-98	20.97064975	0.0530817	OK	2.87E-97
COL21A1	0	85.67566533	0.22022623	OK	0
DST	0	101.1777268	0.26013767	OK	0
BEND6	4.03E-21	9.358747983	0.02392389	OK	1.20E-20
KIAA1586	1.23E-06	4.711576069	0.01204009	OK	2.69E-06
ZNF451-AS1	1.59E-08	5.530870308	0.01356093	OK	3.73E-08
BAG2	0	50.35409342	0.12939515	OK	0
RAB23	1.86E-156	26.62261119	0.06834557	OK	1.05E-155
PRIM2	2.52E-14	7.530854311	0.01927842	OK	6.75E-14
PTP4A1	1.32E-136	24.84925155	0.06382891	OK	7.04E-136
PHF3	5.92E-39	13.0024894	0.03336632	OK	2.07E-38

AL391807.1	3.71E-13	7.171484991	0.01832831	OK	9.73E-13
ADGRB3	5.12E-07	4.88681256	0.01245173	OK	1.14E-06
LMBRD1	2.78E-65	17.02276846	0.04370217	OK	1.14E-64
SMAP1	1.32E-227	32.18758619	0.08270795	OK	8.62E-227
OGFRL1	0	110.3951762	0.28383932	OK	0
LINC00472	2.73E-06	4.5462772	0.01156619	OK	5.91E-06
KHDC1	7.59E-12	6.746086885	0.01720333	OK	1.94E-11
CGAS	1.13E-184	28.95739401	0.07438288	OK	6.79E-184
EEF1A1	0	164.556228	0.4229606	OK	0
SLC17A5	3.31E-57	15.89733767	0.04079899	OK	1.30E-56
CD109	0	104.2962479	0.26812124	OK	0
COL12A1	5.90E-209	30.8258814	0.07913792	OK	3.73E-208
COX7A2	0	139.5891132	0.35893279	OK	0
TMEM30A	4.68E-204	30.45810446	0.0782588	OK	2.93E-203
FILIP1	0	121.3059967	0.31187248	OK	0
SENP6	5.83E-23	9.796426804	0.02512041	OK	1.78E-22
MYO6	9.13E-46	14.15159351	0.03629028	OK	3.35E-45
IRAK1BP1	2.92E-09	5.821208467	0.01486298	OK	6.98E-09
PHIP	5.59E-06	4.392866706	0.01122341	OK	1.19E-05
HMGN3	0	58.43217557	0.15020942	OK	0
LCA5	4.39E-86	19.62872622	0.05036892	OK	1.98E-85
SH3BGRL2	2.56E-38	12.89014166	0.03295012	OK	8.92E-38
ELOVL4	5.99E-05	3.846366976	0.00977354	OK	0.00012225
TTK	0	67.2462188	0.17133858	OK	0
BCKDHB	7.78E-08	5.245703628	0.01340565	OK	1.78E-07
TENT5A	6.59E-24	10.0143557	0.02568028	OK	2.04E-23
LINC02542	1.53E-12	6.974555557	0.01771189	OK	3.97E-12
TPBG	2.45E-85	19.5412597	0.05013427	OK	1.10E-84
PGM3	3.21E-06	4.512027884	0.01152543	OK	6.93E-06
RWDD2A	1.54E-08	5.536462679	0.01413414	OK	3.62E-08
ME1	0	56.49836281	0.14520069	OK	0
PRSS35	6.98E-219	31.55792771	0.08101084	OK	4.49E-218
RIPPLY2	9.52E-05	3.731436518	0.00923785	OK	0.00019237
CYB5R4	0	59.35488156	0.15256875	OK	0
MRAP2	0	81.02783983	0.20823082	OK	0
TBX18	1.82E-25	10.36325473	0.02653871	OK	5.71E-25
NT5E	3.84E-211	30.98861219	0.07958389	OK	2.44E-210
SNX14	9.74E-23	9.744425077	0.02498106	OK	2.97E-22
SYNCRIP	6.27E-245	33.40264547	0.08583398	OK	4.21E-244
ZNF292	1.21E-16	8.198750627	0.02100922	OK	3.38E-16
SLC35A1	8.88E-10	6.017032093	0.01539124	OK	2.16E-09
ORC3	1.97E-07	5.071727255	0.01296536	OK	4.46E-07
AKIRIN2	0	78.39490886	0.2015467	OK	0
PNRC1	0	140.0051269	0.35998752	OK	0
PM20D2	9.55E-27	10.64154489	0.02727124	OK	3.03E-26
UBE2J1	0	52.71610004	0.1355017	OK	0
RRAGD	0	40.45828763	0.1039662	OK	0
ANKRD6	5.69E-33	11.90326664	0.0305024	OK	1.91E-32
LYRM2	5.40E-118	23.06334733	0.05924004	OK	2.73E-117
CASP8AP2	5.48E-08	5.310020698	0.01357901	OK	1.26E-07
MANEA	3.64E-07	4.953514853	0.01265845	OK	8.16E-07
UFL1	1.80E-75	18.34528631	0.04710607	OK	7.72E-75
FHL5	0	106.8916749	0.27475299	OK	0

NDUFAF4	3.08E-30	11.36619236	0.02915328	OK	1.01E-29
COQ3	1.19E-14	7.628488082	0.01952387	OK	3.20E-14
PNISR	1.07E-154	26.47027932	0.06800499	OK	5.98E-154
TSTD3	1.43E-22	9.705596738	0.02484846	OK	4.34E-22
CCNC	8.87E-15	7.665992195	0.01963942	OK	2.40E-14
BVES	1.16E-08	5.586569262	0.01423184	OK	2.72E-08
PREP	0.00022781	3.505578557	0.00893345	OK	0.00045105
ATG5	1.51E-28	11.02093507	0.02826788	OK	4.89E-28
PRDM1	0	77.07323535	0.19812768	OK	0
CRYBG1	0	56.41666148	0.14499203	OK	0
C6orf203	2.28E-53	15.33353874	0.03934821	OK	8.75E-53
PDSS2	0.00033149	3.404448615	0.00867575	OK	0.00065031
SOBP	2.12E-39	13.08089802	0.03352698	OK	7.45E-39
SCML4	1.18E-12	7.011349777	0.01786994	OK	3.06E-12
SEC63	7.93E-37	12.62250332	0.03238835	OK	2.74E-36
OSTM1	2.18E-60	16.34991753	0.04197311	OK	8.71E-60
SNX3	0	124.2591075	0.31950689	OK	0
FOXO3	9.29E-46	14.1503674	0.03631638	OK	3.41E-45
SESN1	3.90E-84	19.3994246	0.04980805	OK	1.75E-83
CEP57L1	2.50E-10	6.21893879	0.01591234	OK	6.16E-10
CD164	0	61.10222969	0.15707719	OK	0
SMPD2	1.63E-08	5.526675127	0.01412312	OK	3.82E-08
MICAL1	1.32E-28	11.03325326	0.02829029	OK	4.27E-28
ZBTB24	6.05E-12	6.778924463	0.01735448	OK	1.55E-11
FIG4	1.70E-11	6.627941293	0.01696381	OK	4.31E-11
CDC40	1.38E-137	24.93959063	0.06406689	OK	7.43E-137
METTL24	1.53E-08	5.537816602	0.01406841	OK	3.59E-08
SLC22A16	5.39E-10	6.097528509	0.0155598	OK	1.31E-09
CDK19	0.0002151	3.520825488	0.00897608	OK	0.00042655
AMD1	0	51.47448012	0.13231388	OK	0
GTF3C6	0	66.03580721	0.16976573	OK	0
RPF2	2.50E-94	20.57091651	0.05282973	OK	1.17E-93
SLC16A10	0	80.53180068	0.20702	OK	0
REV3L	1.47E-19	8.971001618	0.02299564	OK	4.27E-19
FYN	3.80E-114	22.67671333	0.0582182	OK	1.89E-113
TUBE1	0.00019657	3.544652175	0.00903473	OK	0.00039077
FAM229B	0	40.47381703	0.10397084	OK	0
LAMA4	0	46.77720799	0.12014997	OK	0
MARCKS	0	198.9121439	0.5115173	OK	0
LINC01268	1.95E-20	9.19092067	0.02347912	OK	5.76E-20
HDAC2	1.06E-55	15.67826707	0.0402466	OK	4.14E-55
FRK	8.36E-105	21.70941616	0.05567637	OK	4.04E-104
NT5DC1	1.65E-257	34.2581548	0.08802233	OK	1.12E-256
TSPYL4	5.62E-07	4.868674697	0.01243831	OK	1.25E-06
DSE	0	104.4817443	0.2686355	OK	0
TSPYL1	1.28E-197	29.96815915	0.07699838	OK	7.88E-197
CALHM6	0	61.97625543	0.15926462	OK	0
CALHM5	2.69E-57	15.91021704	0.04078343	OK	1.06E-56
RWDD1	0	77.52937709	0.19932649	OK	0
RSPH4A	5.44E-09	5.71636038	0.01406749	OK	1.29E-08
ZUP1	3.93E-23	9.83630942	0.02521054	OK	1.20E-22
FAM162B	0	67.44206625	0.17291123	OK	0
GOPC	1.13E-52	15.22944192	0.03909196	OK	4.30E-52

NUS1	5.73E-69	17.51271722	0.04495453	OK	2.38E-68
SLC35F1	4.32E-06	4.448806599	0.01130109	OK	9.25E-06
CEP85L	1.76E-30	11.41488624	0.02927561	OK	5.77E-30
PLN	0	247.7762478	0.63715993	OK	0
ASF1A	1.36E-49	14.75841837	0.03787673	OK	5.11E-49
MAN1A1	0	40.60526307	0.10434691	OK	0
TBC1D32	3.73E-07	4.948774263	0.01260902	OK	8.36E-07
GJA1	0	96.31587229	0.24757256	OK	0
HSF2	0.00013188	3.648518983	0.00930327	OK	0.00026451
SERINC1	5.41E-289	36.31109711	0.09331465	OK	3.85E-288
PKIB	0	123.8929597	0.31843784	OK	0
SMPDL3A	0	68.33433811	0.17564083	OK	0
RNF217	1.25E-08	5.573239926	0.01425433	OK	2.94E-08
TPD52L1	0	83.52301555	0.21466226	OK	0
HDDC2	0	45.47368846	0.11687234	OK	0
AL450332.1	2.37E-15	7.833546591	0.01999229	OK	6.46E-15
AL365259.1	1.84E-10	6.267328518	0.01592281	OK	4.53E-10
HEY2	0	82.43997802	0.21189675	OK	0
NCOA7	1.33E-65	17.06588533	0.04381248	OK	5.45E-65
HINT3	7.21E-10	6.050801142	0.0154831	OK	1.75E-09
TRMT11	1.79E-53	15.34921747	0.03938992	OK	6.88E-53
CENPW	0	80.48303491	0.2068588	OK	0
RSPO3	2.50E-07	5.026255451	0.01272089	OK	5.64E-07
RNF146	5.59E-18	8.561115496	0.02194028	OK	1.59E-17
ECHDC1	1.11E-165	27.40769831	0.0704123	OK	6.38E-165
KIAA0408	2.88E-05	4.022259964	0.01017124	OK	5.96E-05
THEMIS	4.21E-32	11.73507138	0.02994504	OK	1.40E-31
PTPRK	5.94E-58	16.00450316	0.04102937	OK	2.34E-57
LAMA2	3.07E-174	28.11659975	0.07213263	OK	1.80E-173
ARHGAP18	0	217.6609059	0.55973538	OK	0
SAMD3	1.02E-23	9.971264936	0.02549366	OK	3.14E-23
TMEM200A	1.73E-21	9.447780027	0.0241238	OK	5.18E-21
EPB41L2	7.23E-230	32.34870881	0.08312119	OK	4.75E-229
AKAP7	2.17E-38	12.9026732	0.03309652	OK	7.59E-38
ENPP3	1.76E-07	5.093136719	0.01185762	OK	3.99E-07
ENPP1	0	58.97920458	0.15155738	OK	0
CTGF	0	194.6540544	0.50054327	OK	0
AL133346.1	4.39E-07	4.917227141	0.01236404	OK	9.79E-07
MOXD1	3.72E-06	4.480976547	0.01118831	OK	8.00E-06
STX7	0	43.63695292	0.11215509	OK	0
VNN1	1.20E-48	14.61059372	0.03744531	OK	4.50E-48
VNN2	1.66E-102	21.46479103	0.05505343	OK	7.96E-102
SLC18B1	2.84E-169	27.70745624	0.07112446	OK	1.65E-168
RPS12	0	163.2415123	0.41966885	OK	0
EYA4	8.92E-12	6.72275499	0.01675578	OK	2.27E-11
TBPL1	9.09E-17	8.233550768	0.02109615	OK	2.54E-16
SLC2A12	3.23E-17	8.356524369	0.02134632	OK	9.07E-17
SGK1	0	266.2012462	0.68457947	OK	0
LINC01010	1.08E-24	10.19158718	0.02602047	OK	3.36E-24
AL596188.1	1.00E-06	4.752989659	0.01080822	OK	2.20E-06
HBS1L	6.52E-24	10.01535221	0.02567894	OK	2.02E-23
AHI1	1.07E-51	15.08154157	0.03870995	OK	4.07E-51
PDE7B	1.56E-05	4.16413935	0.01053082	OK	3.28E-05

MTFR2	7.16E-204	30.4441363	0.07755406	OK	4.48E-203
BCLAF1	1.77E-10	6.273151678	0.01605995	OK	4.37E-10
MAP7	6.68E-12	6.764624137	0.01729949	OK	1.71E-11
MAP3K5	1.35E-06	4.691946421	0.01198953	OK	2.96E-06
IL22RA2	4.17E-19	8.8552594	0.01875076	OK	1.21E-18
IFNGR1	0	163.7989862	0.42120585	OK	0
AL357060.1	4.15E-07	4.927985123	0.0125558	OK	9.28E-07
TNFAIP3	0	61.3178608	0.15760906	OK	0
PERP	0	50.19542193	0.12896307	OK	0
HEBP2	0	54.81301306	0.14090135	OK	0
AL590617.2	2.72E-06	4.547193405	0.01159875	OK	5.89E-06
CCDC28A	4.41E-210	30.9097781	0.07941313	OK	2.80E-209
ABRACL	0	148.949556	0.38301025	OK	0
HECA	3.26E-55	15.60699976	0.04005726	OK	1.26E-54
TXLNB	1.37E-28	11.03001015	0.02825315	OK	4.42E-28
CITED2	0	46.78903465	0.12026166	OK	0
AL138737.1	2.75E-05	4.033226886	0.01013177	OK	5.70E-05
AL035446.1	2.43E-14	7.535695315	0.01925664	OK	6.51E-14
VTA1	2.60E-39	13.06517711	0.03352115	OK	9.15E-39
ADGRG6	3.25E-77	18.56226768	0.04756685	OK	1.40E-76
AIG1	0	57.54709643	0.14792726	OK	0
PEX3	1.50E-07	5.122998847	0.01309629	OK	3.41E-07
FUCA2	0	55.36915591	0.14232466	OK	0
PHACTR2	7.56E-107	21.92462274	0.05631146	OK	3.69E-106
LTV1	1.24E-18	8.733375149	0.02238197	OK	3.55E-18
PLAGL1	5.02E-15	7.738723783	0.01982347	OK	1.36E-14
SF3B5	0	67.36188825	0.17317692	OK	0
STX11	0	116.090504	0.29848111	OK	0
UTRN	7.93E-79	18.76057589	0.04817359	OK	3.46E-78
SHPRH	3.00E-10	6.190393083	0.01584397	OK	7.37E-10
RAB32	0	121.0937777	0.31137018	OK	0
STXBP5-AS1	1.44E-06	4.678549176	0.0118807	OK	3.16E-06
SASH1	5.91E-45	14.01968288	0.03597508	OK	2.16E-44
TAB2	2.66E-16	8.104018765	0.02076247	OK	7.37E-16
SUMO4	0.00040071	3.352303565	0.00853075	OK	0.00078241
ZC3H12D	9.25E-27	10.64452085	0.02726009	OK	2.93E-26
PPIL4	3.18E-128	24.0612914	0.06180813	OK	1.66E-127
GINM1	0	41.49167704	0.10663834	OK	0
KATNA1	2.83E-11	6.55258026	0.01677519	OK	7.12E-11
PCMT1	0	47.0502513	0.12093562	OK	0
LRP11	2.81E-10	6.201013344	0.01584297	OK	6.90E-10
PLEKHG1	0.00011606	3.681223873	0.00934517	OK	0.00023351
MTHFD1L	2.08E-15	7.849724962	0.02010783	OK	5.68E-15
AKAP12	0	76.02118181	0.19539779	OK	0
ZBTB2	1.75E-05	4.138147843	0.01055964	OK	3.66E-05
ARMT1	1.70E-67	17.31880177	0.04446026	OK	7.01E-67
CCDC170	0	40.52534326	0.10410293	OK	0
SYNE1	0	50.40730921	0.12955997	OK	0
AL049548.1	1.61E-07	5.109897875	0.01244478	OK	3.66E-07
MYCT1	0	53.37725506	0.13549051	OK	0
FBXO5	2.44E-32	11.78110141	0.03018031	OK	8.15E-32
MTRF1L	5.39E-39	13.00972853	0.03337799	OK	1.89E-38
RGS17	0	42.68206553	0.10962371	OK	0

IPCEF1	6.22E-22	9.554396217	0.02445268	OK	1.87E-21
CNKSR3	6.28E-44	13.85093497	0.03549331	OK	2.28E-43
AL596202.1	0.00023991	3.491774908	0.00877732	OK	0.0004745
TFB1M	8.93E-11	6.378755	0.01632314	OK	2.22E-10
ARID1B	1.19E-07	5.167600192	0.01321528	OK	2.70E-07
TMEM242	1.50E-43	13.78829408	0.03538463	OK	5.43E-43
ZDHHC14	1.88E-06	4.624160143	0.01180972	OK	4.10E-06
SNX9	7.58E-180	28.57155795	0.07340791	OK	4.50E-179
SYNJ2	1.12E-22	9.729824487	0.02492835	OK	3.42E-22
SERAC1	0.00029662	3.434689938	0.00875171	OK	0.00058314
GTF2H5	2.64E-207	30.70239503	0.07888853	OK	1.67E-206
TULP4	4.23E-05	3.931188412	0.01003399	OK	8.68E-05
DYNLT1	0	55.32845167	0.14222739	OK	0
SYTL3	2.13E-102	21.4533229	0.05505841	OK	1.02E-101
EZR	0	111.6716982	0.28713177	OK	0
RSPH3	2.19E-12	6.924388568	0.01772334	OK	5.65E-12
TAGAP	0	122.3709408	0.31462749	OK	0
FNDC1	1.41E-21	9.469053886	0.0240269	OK	4.23E-21
SOD2	0	99.90262879	0.25686699	OK	0
WTAP	3.49E-300	37.01339005	0.09512084	OK	2.52E-299
ACAT2	1.76E-52	15.20037594	0.03901233	OK	6.70E-52
TCP1	1.31E-157	26.72188112	0.06865149	OK	7.39E-157
MRPL18	0	39.48907217	0.10148844	OK	0
IGF2R	1.35E-217	31.46399673	0.08083668	OK	8.66E-217
SLC22A3	1.81E-245	33.43988554	0.08586317	OK	1.21E-244
AGPAT4	2.78E-22	9.637267924	0.02469187	OK	8.43E-22
QKI	0	94.65939203	0.24338406	OK	0
AL445307.1	1.15E-12	7.014808302	0.01592305	OK	2.99E-12
PDE10A	0.00019923	3.541104563	0.00880172	OK	0.00039592
LINC00473	2.65E-17	8.379945581	0.02068888	OK	7.45E-17
SFT2D1	0	64.46973821	0.16573474	OK	0
MPC1	0	43.01241966	0.1105496	OK	0
RPS6KA2	4.35E-35	12.30325828	0.03152964	OK	1.48E-34
AL159163.1	5.71E-07	4.865593701	0.01239241	OK	1.27E-06
RNASET2	0	251.5661726	0.64694135	OK	0
CCR6	0	38.1436395	0.09765334	OK	0
GPR31	6.98E-58	15.99446017	0.03923692	OK	2.75E-57
AFDN	3.04E-15	7.802430154	0.01998657	OK	8.26E-15
FRMD1	1.67E-07	5.103472928	0.01205326	OK	3.78E-07
SMOC2	0	170.1354242	0.43746391	OK	0
LINC01615	5.29E-168	27.6018438	0.07081771	OK	3.06E-167
LINC02544	1.91E-208	30.78778087	0.07894885	OK	1.21E-207
THBS2	0	96.09941511	0.24702089	OK	0
AL009176.1	3.06E-06	4.522493525	0.01045423	OK	6.60E-06
C6orf120	1.74E-05	4.140130233	0.01057029	OK	3.63E-05
PHF10	1.41E-26	10.60547932	0.02719715	OK	4.45E-26
PSMB1	0	115.2949416	0.29645283	OK	0
PDCD2	2.47E-109	22.18366364	0.05697774	OK	1.22E-108
AC093627.5	0.00030079	3.430904841	0.00865788	OK	0.00059113
AC093627.4	1.02E-12	7.031511375	0.01796643	OK	2.66E-12
FAM20C	0	74.71276498	0.19205215	OK	0
PDGFA	0	78.08486171	0.20073511	OK	0
PRKAR1B	1.35E-82	19.2165191	0.04930848	OK	5.97E-82

AC147651.4	0	42.24842308	0.10854182	OK	0
DNAAF5	0.00032359	3.411027796	0.00868759	OK	0.00063521
SUN1	1.22E-94	20.60560409	0.05290619	OK	5.71E-94
ADAP1	2.98E-113	22.58586119	0.05797781	OK	1.48E-112
COX19	1.53E-54	15.50816504	0.03980563	OK	5.89E-54
C7orf50	0	40.48839492	0.10405765	OK	0
GPR146	1.24E-104	21.69114185	0.05562664	OK	6.01E-104
GPER1	0.00011046	3.693806598	0.00939169	OK	0.00022249
ZFAND2A	4.30E-25	10.28077058	0.02636156	OK	1.34E-24
MICALL2	2.70E-07	5.011295141	0.01278749	OK	6.08E-07
AC102953.2	0.00022715	3.506351044	0.00873151	OK	0.00044978
PSMG3	1.95E-50	14.88873028	0.03821269	OK	7.37E-50
ELFN1-AS1	7.87E-05	3.778940082	0.00943721	OK	0.00015978
MAD1L1	2.24E-194	29.71808661	0.07633861	OK	1.38E-193
MRM2	2.54E-53	15.32665413	0.03933723	OK	9.72E-53
NUDT1	0	74.87892226	0.19250242	OK	0
SNX8	0	58.15710617	0.14948318	OK	0
EIF3B	1.29E-16	8.191226139	0.02098991	OK	3.60E-16
CHST12	3.41E-12	6.861179345	0.01756776	OK	8.78E-12
LFNG	0	47.54234507	0.12217191	OK	0
TTYH3	0	71.04499007	0.18261516	OK	0
GNA12	7.94E-105	21.7117988	0.0557516	OK	3.84E-104
CARD11	7.93E-09	5.651931147	0.01440259	OK	1.87E-08
SDK1	1.29E-40	13.29210528	0.03404924	OK	4.57E-40
AP5Z1	2.40E-06	4.573599098	0.01168175	OK	5.20E-06
RBAK-RBAKDN	0.00046411	3.311425954	0.00843858	OK	0.00090204
WIPI2	9.00E-125	23.72926655	0.06095173	OK	4.63E-124
ACTB	0	205.7162376	0.52883675	OK	0
FSCN1	4.40E-231	32.43504276	0.08330618	OK	2.90E-230
AIMP2	3.58E-62	16.59838289	0.04260743	OK	1.44E-61
EIF2AK1	1.41E-54	15.51315759	0.03981949	OK	5.45E-54
CYTH3	3.30E-28	10.95050606	0.02806609	OK	1.06E-27
RAC1	0	169.2653168	0.43523478	OK	0
DAGLB	5.58E-24	10.03076822	0.02570033	OK	1.73E-23
KDELR2	0	72.65199464	0.18678242	OK	0
ZDHHC4	1.32E-21	9.476076611	0.02429413	OK	3.96E-21
C7orf26	0.00049583	3.292880335	0.00838776	OK	0.00096184
ZNF853	1.45E-40	13.28314074	0.0340163	OK	5.14E-40
ZNF12	2.30E-06	4.582511091	0.01170329	OK	4.99E-06
C1GALT1	9.78E-48	14.4670987	0.03712759	OK	3.64E-47
MIOS	5.50E-22	9.567075907	0.02452009	OK	1.66E-21
RPA3	0	43.81554724	0.11260936	OK	0
GLCCI1	3.75E-06	4.478710814	0.01143385	OK	8.08E-06
ICA1	3.01E-234	32.65877926	0.08387507	OK	1.99E-233
NDUFA4	0	162.7327096	0.41844895	OK	0
PHF14	1.05E-29	11.25863104	0.0288797	OK	3.42E-29
THSD7A	7.90E-133	24.49725981	0.06146335	OK	4.18E-132
TMEM106B	1.27E-160	26.97989171	0.06930709	OK	7.21E-160
SCIN	4.80E-16	8.03177915	0.02055807	OK	1.32E-15
ARL4A	0	118.4287289	0.30450834	OK	0
ETV1	4.12E-53	15.29507698	0.03917497	OK	1.58E-52
DGKB	4.40E-37	12.66881237	0.03207435	OK	1.52E-36
AGMO	1.41E-10	6.3081823	0.01459193	OK	3.50E-10

MEOX2	2.47E-124	23.68672768	0.06075675	OK	1.27E-123
ISPD	0.00025557	3.474855156	0.00879984	OK	0.00050469
ANKMY2	1.17E-21	9.488475023	0.02431672	OK	3.52E-21
BZW2	2.30E-189	29.32770182	0.07534232	OK	1.40E-188
TSPAN13	0	46.55482854	0.11953774	OK	0
AHR	0	41.49025544	0.10662215	OK	0
AC073332.1	3.57E-05	3.971402534	0.01013061	OK	7.36E-05
SNX13	1.61E-15	7.882143159	0.0201937	OK	4.40E-15
HDAC9	1.06E-81	19.10911281	0.04905722	OK	4.68E-81
TWIST1	0	67.3335067	0.17305047	OK	0
TWISTNB	2.28E-57	15.92050795	0.04086785	OK	8.97E-57
MACC1	2.70E-70	17.68573273	0.04534297	OK	1.13E-69
AC004130.1	5.94E-10	6.081754724	0.01554382	OK	1.45E-09
ITGB8	0	56.51290503	0.1451469	OK	0
CDCA7L	1.39E-132	24.47423117	0.06282018	OK	7.34E-132
RAPGEF5	1.32E-251	33.8593643	0.08695705	OK	8.94E-251
STEAP1B	8.79E-22	9.518432704	0.02220239	OK	2.65E-21
AC073072.1	3.74E-06	4.479829101	0.00996557	OK	8.04E-06
IL6	8.82E-297	36.80124516	0.09435236	OK	6.35E-296
TOMM7	0	148.7635539	0.38252328	OK	0
KLHL7	1.27E-08	5.569879305	0.01424487	OK	2.99E-08
NUPL2	3.93E-16	8.056295205	0.02064197	OK	1.09E-15
AC005082.1	4.55E-27	10.71045697	0.02740476	OK	1.45E-26
GPNMB	0	165.2258502	0.42487326	OK	0
MALSU1	5.47E-38	12.83143186	0.03292386	OK	1.90E-37
CCDC126	3.79E-34	12.12728866	0.03109947	OK	1.28E-33
MPP6	4.21E-94	20.54566722	0.05271619	OK	1.96E-93
GSDME	4.72E-218	31.4973761	0.08089455	OK	3.03E-217
OSBPL3	2.12E-35	12.36103659	0.03168174	OK	7.25E-35
CYCS	0	94.06455956	0.24185269	OK	0
NFE2L3	2.54E-206	30.62867953	0.07865688	OK	1.60E-205
HNRNPA2B1	0	50.53146821	0.12988093	OK	0
CBX3	0	63.85429597	0.16415508	OK	0
SNX10	0	101.5899854	0.26118542	OK	0
AC004540.2	1.71E-62	16.6426749	0.04191238	OK	6.91E-62
SKAP2	0	137.6978108	0.35406252	OK	0
HOTAIRM1	3.86E-63	16.73152737	0.04295063	OK	1.56E-62
HOXA2	3.79E-16	8.060806722	0.02059138	OK	1.05E-15
HOXA3	8.02E-181	28.64996455	0.07354709	OK	4.77E-180
HOXA-AS2	1.20E-09	5.967708331	0.01522737	OK	2.91E-09
HOXA4	2.40E-09	5.853908267	0.01492022	OK	5.75E-09
HOXA5	1.97E-10	6.25640036	0.01589609	OK	4.86E-10
HOXA7	8.55E-12	6.72893714	0.01632047	OK	2.18E-11
HOXA9	2.15E-40	13.25346809	0.03353787	OK	7.64E-40
EVX1	2.16E-06	4.595772365	0.01092004	OK	4.69E-06
HIBADH	6.71E-52	15.11235084	0.03878408	OK	2.55E-51
TAX1BP1	0	46.02476991	0.11829848	OK	0
JAZF1	1.56E-17	8.442187913	0.02163113	OK	4.40E-17
CREB5	1.58E-242	33.23688495	0.08539254	OK	1.06E-241
AC005162.3	1.28E-14	7.619115719	0.01939117	OK	3.44E-14
CPVL	0	258.7793357	0.66547654	OK	0
CHN2	2.64E-83	19.3009366	0.04950498	OK	1.17E-82
WIPF3	2.37E-08	5.461160244	0.01390215	OK	5.51E-08

SCRN1	2.42E-20	9.167608347	0.0234959	OK	7.14E-20
FKBP14	2.96E-29	11.16704012	0.02862834	OK	9.59E-29
MTURN	9.30E-100	21.16858451	0.05435929	OK	4.43E-99
AC007036.1	2.04E-12	6.934205792	0.0161626	OK	5.27E-12
ZNRF2	9.35E-224	31.91115052	0.08198493	OK	6.07E-223
GGCT	6.37E-140	25.15412598	0.06461407	OK	3.44E-139
AC005154.6	2.05E-07	5.064179397	0.01223522	OK	4.63E-07
GARS	9.66E-38	12.78723425	0.03280703	OK	3.36E-37
INMT	0	95.83489777	0.24634464	OK	0
MINDY4	1.22E-08	5.57701457	0.01423351	OK	2.88E-08
AQP1	0	132.298262	0.34014836	OK	0
LSM5	0	50.33088205	0.12937269	OK	0
AVL9	4.26E-06	4.451852403	0.01136502	OK	9.13E-06
KBTBD2	7.55E-22	9.534280118	0.02444022	OK	2.27E-21
FKBP9	1.38E-223	31.89893542	0.08193459	OK	8.97E-223
NT5C3A	6.13E-20	9.066761253	0.02323975	OK	1.79E-19
RP9	2.47E-21	9.410639723	0.02412372	OK	7.37E-21
BBS9	2.04E-05	4.102911834	0.01045184	OK	4.26E-05
BMPER	2.54E-07	5.023266007	0.01273426	OK	5.72E-07
HERPUD2	3.79E-10	6.153276186	0.01574876	OK	9.30E-10
AC018647.1	1.20E-50	14.92096003	0.03806212	OK	4.56E-50
SPET7	0	129.4417856	0.33283112	OK	0
EEPD1	2.00E-99	21.13250832	0.05424629	OK	9.50E-99
KIAA0895	3.59E-05	3.97016293	0.01006465	OK	7.40E-05
ANLN	0	138.4201479	0.35350752	OK	0
AOAH	0	149.3611137	0.38403237	OK	0
ELMO1	0	51.00246095	0.13106442	OK	0
GPR141	3.86E-45	14.04977039	0.03599166	OK	1.41E-44
EPDR1	0	40.06287766	0.10290984	OK	0
NME8	1.77E-20	9.201260385	0.02348558	OK	5.23E-20
SFRP4	0	69.06019445	0.1774356	OK	0
STARD3NL	4.22E-188	29.22844911	0.075092	OK	2.55E-187
TRGC2	5.04E-09	5.729327321	0.01456035	OK	1.20E-08
TRG-AS1	3.57E-46	14.21738623	0.03644066	OK	1.32E-45
AMPH	8.45E-10	6.025054389	0.01532822	OK	2.05E-09
VPS41	1.03E-13	7.34455973	0.01881256	OK	2.73E-13
POU6F2	0.00032458	3.410197873	0.00847739	OK	0.00063698
YAE1D1	4.30E-17	8.322537498	0.02131372	OK	1.21E-16
RALA	0	141.0662314	0.36273433	OK	0
MPLKIP	3.27E-69	17.54467686	0.0450469	OK	1.36E-68
SUGCT	0	94.64121221	0.24324542	OK	0
AC004988.1	1.71E-31	11.61572578	0.02968675	OK	5.67E-31
INHBA	0	54.76189954	0.14073622	OK	0
INHBA-AS1	0.00047957	3.302247167	0.008383	OK	0.00093118
GLI3	3.30E-08	5.401866586	0.01373095	OK	7.65E-08
PSMA2	0	41.24978965	0.10601598	OK	0
MRPL32	3.60E-244	33.35033089	0.08569562	OK	2.41E-243
STK17A	1.83E-151	26.18780995	0.06727397	OK	1.01E-150
COA1	2.23E-15	7.841313336	0.02008924	OK	6.07E-15
BLVRA	0	61.44036009	0.15794375	OK	0
URGCP	2.09E-07	5.060353018	0.01292477	OK	4.73E-07
UBE2D4	8.58E-23	9.757372842	0.0250152	OK	2.62E-22
AC004951.1	3.57E-22	9.611607943	0.02459713	OK	1.08E-21

DBNL	8.90E-287	36.17036429	0.0929514	OK	6.32E-286
PGAM2	9.46E-86	19.58970731	0.05020156	OK	4.26E-85
AEBP1	0	214.7017018	0.55210595	OK	0
POLD2	4.15E-116	22.87473901	0.05874838	OK	2.08E-115
GCK	8.57E-08	5.227849845	0.01330778	OK	1.96E-07
YKT6	9.94E-23	9.742357794	0.02497267	OK	3.03E-22
NUDCD3	2.90E-08	5.424825676	0.01387377	OK	6.74E-08
DDX56	6.17E-06	4.371414452	0.01116557	OK	1.32E-05
TMED4	1.39E-243	33.30979831	0.08559569	OK	9.32E-243
OGDH	1.12E-05	4.238543651	0.01082365	OK	2.37E-05
ZMIZ2	5.15E-05	3.88322554	0.00990481	OK	0.00010543
PPIA	0	161.7384082	0.41587665	OK	0
H2AFV	0	91.45745874	0.23514907	OK	0
PURB	1.37E-17	8.456952956	0.02167316	OK	3.88E-17
AC004854.2	3.22E-06	4.511614795	0.01150086	OK	6.94E-06
MYO1G	0	42.3524777	0.10882525	OK	0
SNHG15	0	54.68655626	0.14056758	OK	0
CCM2	3.49E-87	19.75708165	0.05073467	OK	1.58E-86
TBRG4	1.61E-19	8.961207945	0.02296493	OK	4.67E-19
RAMP3	0	73.42015803	0.18804062	OK	0
ADCY1	8.98E-12	6.721715217	0.01711382	OK	2.29E-11
IGFBP3	0	53.37398833	0.1371093	OK	0
TNS3	4.30E-102	21.42054842	0.05500678	OK	2.06E-101
HUS1	3.25E-45	14.06208653	0.03608312	OK	1.19E-44
SUN3	1.24E-07	5.159479817	0.01254485	OK	2.82E-07
UPP1	0	130.66953	0.33598623	OK	0
VWC2	1.40E-13	7.303559475	0.01695184	OK	3.70E-13
IKZF1	0	50.88398137	0.1307684	OK	0
SEC61G	0	110.0554014	0.28298075	OK	0
EGFR	8.55E-121	23.34077122	0.05990133	OK	4.35E-120
VOPP1	0	39.93129337	0.10261791	OK	0
NIPSNAP2	2.91E-268	34.9728947	0.08987185	OK	2.01E-267
MRPS17	3.55E-06	4.490671621	0.01146548	OK	7.65E-06
CCT6A	0	54.12936021	0.13914341	OK	0
SUMF2	1.75E-81	19.0828964	0.0490035	OK	7.73E-81
PHKG1	1.71E-19	8.954178594	0.02294676	OK	4.97E-19
CHCHD2	0	167.2480804	0.43005528	OK	0
NUPR2	2.90E-38	12.88054419	0.03294283	OK	1.01E-37
ZNF680	3.05E-08	5.41599444	0.01384407	OK	7.08E-08
ZNF117	2.70E-08	5.437617165	0.01390002	OK	6.29E-08
ERV3-1	3.02E-10	6.189383986	0.01583668	OK	7.41E-10
VKORC1L1	1.94E-21	9.435823779	0.02418538	OK	5.81E-21
GUSB	0	67.87035398	0.1744778	OK	0
ASL	1.52E-18	8.710313652	0.02232116	OK	4.34E-18
CRCP	1.77E-14	7.576723237	0.01940952	OK	4.76E-14
TPST1	6.57E-42	13.51285746	0.03464789	OK	2.36E-41
RABGEF1	6.05E-28	10.89554931	0.0279318	OK	1.94E-27
AC027644.3	9.61E-46	14.14799103	0.03630576	OK	3.53E-45
TMEM248	3.46E-63	16.73802454	0.04297219	OK	1.40E-62
SBDS	0	110.7067105	0.28465618	OK	0
AUTS2	6.29E-30	11.30375775	0.02897118	OK	2.05E-29
POM121	2.00E-05	4.107766023	0.01048652	OK	4.17E-05
NSUN5	1.39E-10	6.311022804	0.0161507	OK	3.44E-10

BAZ1B	5.84E-19	8.817731686	0.02260184	OK	1.68E-18
BCL7B	2.52E-87	19.77344962	0.05077895	OK	1.14E-86
TBL2	3.98E-28	10.93362624	0.02803688	OK	1.28E-27
MLXIPL	5.70E-41	13.35286576	0.03381973	OK	2.03E-40
VPS37D	1.09E-05	4.245745961	0.01076195	OK	2.30E-05
DNAJC30	5.25E-11	6.459478346	0.0165313	OK	1.31E-10
BUD23	4.11E-119	23.1745692	0.05952589	OK	2.08E-118
ABHD11	7.79E-35	12.25611895	0.03143469	OK	2.65E-34
METTL27	8.88E-26	10.43176627	0.02669563	OK	2.79E-25
ELN	0	73.99659807	0.19018266	OK	0
LIMK1	8.27E-61	16.40882202	0.04209948	OK	3.31E-60
EIF4H	4.07E-285	36.06459745	0.09268058	OK	2.89E-284
LAT2	0	108.3939376	0.27867791	OK	0
RFC2	2.65E-66	17.15986137	0.04404911	OK	1.09E-65
GTF2I	1.23E-26	10.61775996	0.0272328	OK	3.90E-26
NCF1	0	112.1904381	0.28844083	OK	0
RCC1L	1.15E-31	11.64991643	0.02987833	OK	3.81E-31
TRIM73	5.48E-05	3.868081498	0.00986089	OK	0.00011207
HIP1	2.80E-35	12.3387879	0.03165115	OK	9.55E-35
CCL26	4.21E-17	8.325082876	0.02100362	OK	1.18E-16
CCL24	5.79E-11	6.444710671	0.01628247	OK	1.45E-10
RHBDD2	2.61E-12	6.899725478	0.01766728	OK	6.71E-12
POR	7.97E-55	15.54988986	0.03991239	OK	3.08E-54
TMEM120A	3.49E-111	22.3745075	0.05746443	OK	1.72E-110
STYXL1	1.43E-31	11.63150244	0.02982652	OK	4.72E-31
MDH2	0	69.92716002	0.17977494	OK	0
SRRM3	3.14E-09	5.809241421	0.0146613	OK	7.50E-09
HSPB1	0	153.7795487	0.39542823	OK	0
YWHAG	0	37.7569476	0.09703085	OK	0
DTX2	1.22E-17	8.470359487	0.02169224	OK	3.46E-17
CCDC146	5.32E-12	6.797594	0.01738577	OK	1.36E-11
FGL2	0	162.02457	0.41664035	OK	0
GSAP	3.27E-126	23.86830089	0.06128354	OK	1.69E-125
AC004921.1	2.77E-52	15.17052515	0.03888247	OK	1.06E-51
PTPN12	4.97E-248	33.61553563	0.08637804	OK	3.35E-247
RSBN1L	3.94E-25	10.2892312	0.02638859	OK	1.23E-24
TMEM60	1.29E-45	14.12725647	0.03625286	OK	4.74E-45
PHTF2	4.26E-08	5.355774895	0.0136964	OK	9.84E-08
MAGI2	7.78E-129	24.11961068	0.06192403	OK	4.07E-128
GNAI1	4.38E-150	26.06646923	0.06692508	OK	2.42E-149
CD36	0	241.0468292	0.6198407	OK	0
SEMA3C	0	41.22421994	0.10587302	OK	0
HGF	9.40E-178	28.40256243	0.07296029	OK	5.55E-177
CACNA2D1	7.70E-155	26.48253973	0.06798138	OK	4.32E-154
PCLO	2.28E-05	4.076960523	0.01028447	OK	4.75E-05
SEMA3E	5.08E-10	6.107023471	0.01555689	OK	1.24E-09
SEMA3D	4.65E-44	13.87254618	0.03554862	OK	1.69E-43
TMEM243	9.19E-150	26.0380226	0.06688955	OK	5.07E-149
TP53TG1	3.18E-119	23.18565653	0.05954979	OK	1.61E-118
CROT	1.72E-13	7.27602303	0.01862843	OK	4.53E-13
ABCB1	1.33E-13	7.310729336	0.0185772	OK	3.51E-13
SLC25A40	4.45E-13	7.14648069	0.01828936	OK	1.17E-12
DBF4	5.26E-181	28.66470609	0.07362306	OK	3.13E-180

SRI	0	57.60103086	0.14807239	OK	0
STEAP4	0	53.16276205	0.1364341	OK	0
STEAP1	8.10E-86	19.59760247	0.05024898	OK	3.65E-85
STEAP2	3.58E-106	21.85370549	0.05606696	OK	1.74E-105
CFAP69	3.60E-06	4.487848453	0.01138766	OK	7.75E-06
CLDN12	5.32E-07	4.8795435	0.0124512	OK	1.18E-06
CDK14	0.00044356	3.324076152	0.00846932	OK	0.00086356
AC002456.1	1.81E-05	4.130935894	0.01047902	OK	3.78E-05
FZD1	7.79E-72	17.88447424	0.04591131	OK	3.28E-71
AKAP9	1.79E-44	13.94065768	0.03577986	OK	6.54E-44
KRIT1	8.05E-24	9.994533256	0.02562692	OK	2.49E-23
GATAD1	0.00042576	3.335484105	0.00850369	OK	0.00083036
FAM133B	7.16E-143	25.42221932	0.06530976	OK	3.89E-142
CDK6	1.29E-177	28.39154629	0.07293077	OK	7.59E-177
SAMD9	4.38E-103	21.52670609	0.05528184	OK	2.10E-102
SAMD9L	1.49E-68	17.45827744	0.0448167	OK	6.18E-68
CALCR	0.00019034	3.553122597	0.00896311	OK	0.00037864
TFPI2	8.73E-291	36.4244474	0.093541	OK	6.24E-290
GNG11	0	111.4340203	0.28648934	OK	0
BET1	5.93E-07	4.857886464	0.01241575	OK	1.32E-06
AC002074.1	0.00046391	3.311544974	0.0083133	OK	0.00090174
COL1A2	0	174.8157592	0.44950512	OK	0
SGCE	0	74.04386774	0.19030724	OK	0
PEG10	6.46E-78	18.64877429	0.04780456	OK	2.81E-77
PPP1R9A	1.26E-11	6.672462073	0.01702471	OK	3.19E-11
PON2	3.91E-88	19.86722891	0.05101305	OK	1.78E-87
PDK4	0	45.00381022	0.11565536	OK	0
DYNC1I1	5.11E-109	22.15094465	0.05683646	OK	2.51E-108
SLC25A13	5.03E-38	12.83787822	0.03293312	OK	1.75E-37
SEM1	0	98.33627707	0.25283844	OK	0
DLX6-AS1	0	64.02873903	0.16449994	OK	0
DLX6	0	46.23307949	0.11870971	OK	0
DLX5	0	68.98978773	0.1772612	OK	0
SDHAF3	2.40E-38	12.89501954	0.03308187	OK	8.38E-38
TAC1	1.38E-17	8.4560032	0.02054555	OK	3.91E-17
BRI3	0	190.5923179	0.49010451	OK	0
NPTX2	0	45.60237573	0.11711803	OK	0
TMEM130	5.33E-236	32.78193557	0.08413569	OK	3.53E-235
ARPC1A	0	68.32593162	0.17565145	OK	0
ARPC1B	0	176.5815376	0.45408464	OK	0
PDAP1	0	48.47987629	0.12461258	OK	0
BUD31	1.64E-218	31.53094134	0.08102012	OK	1.05E-217
ATP5MF	0	104.3448378	0.26829483	OK	0
ZNF394	4.13E-42	13.54688065	0.034761	OK	1.49E-41
ZNF655	1.91E-17	8.418530852	0.02157398	OK	5.37E-17
CYP3A5	3.02E-08	5.417682569	0.01372942	OK	7.01E-08
TRIM4	2.80E-12	6.8896285	0.01763975	OK	7.20E-12
ZKSCAN1	1.71E-05	4.143552578	0.01058031	OK	3.58E-05
COPS6	0	40.42048023	0.10388385	OK	0
MCM7	3.78E-288	36.25751229	0.09311163	OK	2.69E-287
AP4M1	1.55E-09	5.925985311	0.0151559	OK	3.74E-09
TAF6	2.18E-14	7.549658643	0.0193335	OK	5.85E-14
CNPY4	2.59E-65	17.02702042	0.04369119	OK	1.06E-64

MBLAC1	2.01E-05	4.106241815	0.01045932	OK	4.20E-05
LAMTOR4	0	106.7628808	0.27451135	OK	0
C7orf43	2.03E-09	5.881802067	0.01503582	OK	4.87E-09
GAL3ST4	0	46.13532289	0.11853476	OK	0
PILRB	1.44E-10	6.305641302	0.01612988	OK	3.56E-10
PILRA	0	185.4418229	0.47685929	OK	0
PPP1R35	5.02E-19	8.834553364	0.02264392	OK	1.45E-18
TSC22D4	3.05E-127	23.96726132	0.06156064	OK	1.58E-126
SAP25	1.59E-07	5.112942132	0.01304444	OK	3.60E-07
LRCH4	4.44E-88	19.86080929	0.05098366	OK	2.02E-87
PCOLCE	0	109.2581988	0.28088528	OK	0
MOSPD3	4.36E-26	10.49909532	0.02692261	OK	1.37E-25
GNB2	0	59.51499291	0.15299499	OK	0
POP7	7.82E-130	24.21451783	0.06219742	OK	4.10E-129
EPHB4	1.69E-163	27.22390536	0.06940637	OK	9.67E-163
SLC12A9	0	39.27121056	0.10090482	OK	0
TRIP6	1.70E-260	34.4580728	0.08854283	OK	1.17E-259
SRRT	4.23E-21	9.353618353	0.0239777	OK	1.26E-20
ACHE	9.01E-10	6.014754801	0.01529793	OK	2.19E-09
MUC12	1.31E-30	11.44080328	0.029296	OK	4.29E-30
TRIM56	5.14E-20	9.086034878	0.02328816	OK	1.50E-19
SERPINE1	0	41.78302046	0.10732679	OK	0
AP1S1	0	37.75966047	0.09703081	OK	0
PLOD3	1.79E-38	12.91748529	0.03313861	OK	6.26E-38
ZNHIT1	0	61.03401321	0.1569018	OK	0
FIS1	0	96.20420496	0.24735663	OK	0
IFT22	2.41E-176	28.28823452	0.07265437	OK	1.42E-175
CUX1	5.38E-40	13.18466021	0.03383375	OK	1.90E-39
SH2B2	6.02E-56	15.71441287	0.04028433	OK	2.35E-55
PRKRIP1	4.80E-05	3.900691038	0.00995608	OK	9.82E-05
ORAI2	6.80E-54	15.41194506	0.03955111	OK	2.62E-53
ALKBH4	8.15E-23	9.762587342	0.02501643	OK	2.49E-22
POLR2J	0	52.52643203	0.13502035	OK	0
POLR2J3.1	1.70E-66	17.18570414	0.04412195	OK	6.99E-66
RASA4	4.00E-18	8.599566073	0.02203053	OK	1.14E-17
FBXL13	0	43.33532833	0.11104005	OK	0
LRRC17	0	61.68915042	0.15853195	OK	0
ARMC10	7.48E-48	14.48555926	0.03717766	OK	2.79E-47
NAPEPLD	5.30E-11	6.458219719	0.01651638	OK	1.33E-10
PMPCB	1.46E-116	22.92017448	0.05887066	OK	7.35E-116
DNAJC2	5.83E-104	21.61994465	0.05552551	OK	2.81E-103
PSMC2	1.55E-148	25.92952841	0.06661264	OK	8.51E-148
AC007384.1	2.08E-16	8.133854878	0.02080754	OK	5.77E-16
KMT2E	1.01E-28	11.05775619	0.02836574	OK	3.25E-28
SRPK2	2.91E-59	16.19126666	0.04156542	OK	1.16E-58
SYPL1	0	45.39590754	0.11667944	OK	0
NAMPT	0	215.2287148	0.55348153	OK	0
AC007032.1	4.10E-42	13.54752188	0.03473662	OK	1.47E-41
CCDC71L	8.50E-36	12.43436626	0.03190164	OK	2.91E-35
PIK3CG	1.63E-30	11.42172103	0.02926881	OK	5.34E-30
PRKAR2B	3.22E-82	19.17122362	0.04920377	OK	1.43E-81
HBP1	3.40E-20	9.130935735	0.02340834	OK	1.00E-19
BCAP29	0	55.62694014	0.14299254	OK	0

SLC26A4	4.82E-09	5.737104425	0.01452591	OK	1.14E-08
AC002467.1	0.00026174	3.468441245	0.00883181	OK	0.00051652
CBLL1	4.02E-06	4.464043946	0.01140231	OK	8.64E-06
DLD	4.17E-22	9.595695707	0.0246018	OK	1.26E-21
LAMB1	5.45E-160	26.92599611	0.06908161	OK	3.09E-159
NRCAM	1.29E-17	8.464467079	0.0211259	OK	3.64E-17
PNPLA8	7.67E-259	34.34751794	0.08826228	OK	5.24E-258
THAP5	1.23E-74	18.24041056	0.04683061	OK	5.26E-74
DNAJB9	2.95E-248	33.631028	0.08641493	OK	1.99E-247
IMMP2L	9.97E-106	21.80694947	0.05599235	OK	4.84E-105
LRRN3	5.72E-09	5.707827601	0.01451461	OK	1.36E-08
DOCK4	0	41.25476538	0.10600243	OK	0
ZNF277	8.22E-27	10.65546678	0.02732526	OK	2.61E-26
IFRD1	8.85E-40	13.14702053	0.03373765	OK	3.12E-39
GPR85	1.70E-13	7.277595106	0.01855728	OK	4.48E-13
SMIM30	3.08E-94	20.56088479	0.05280248	OK	1.43E-93
FOXP2	5.09E-15	7.736895792	0.01975139	OK	1.38E-14
MDFIC	2.54E-236	32.8044886	0.08428916	OK	1.69E-235
TFEC	0	85.49024631	0.21977447	OK	0
TES	3.09E-288	36.26306521	0.0931811	OK	2.20E-287
CAV2	0	212.291918	0.54589526	OK	0
CAV1	0	285.8285925	0.73504783	OK	0
MET	1.76E-16	8.154292596	0.02080585	OK	4.88E-16
CAPZA2	0	75.1680169	0.19325369	OK	0
CTTNBP2	9.00E-69	17.48699337	0.04487643	OK	3.74E-68
LSM8	2.61E-132	24.44852637	0.06280499	OK	1.38E-131
TSPAN12	3.27E-51	15.0077737	0.03844783	OK	1.24E-50
CPED1	0	48.66179407	0.12506205	OK	0
FAM3C	2.68E-69	17.55601596	0.04507309	OK	1.11E-68
AASS	3.20E-18	8.625186598	0.02207106	OK	9.14E-18
CADPS2	4.65E-61	16.44380095	0.04215843	OK	1.86E-60
RNF148	2.87E-49	14.70782641	0.03208411	OK	1.08E-48
NDUFA5	0	40.64034021	0.10444917	OK	0
WASL	0	60.07474763	0.1544252	OK	0
AC006333.2	8.58E-32	11.6746919	0.02993888	OK	2.85E-31
POT1-AS1	6.79E-07	4.831062133	0.01228476	OK	1.50E-06
AC003975.1	1.37E-05	4.193505854	0.01044298	OK	2.89E-05
GRM8	4.61E-05	3.910250156	0.00922322	OK	9.45E-05
ZNF800	1.11E-09	5.981090142	0.01530598	OK	2.68E-09
ARF5	0	56.44299598	0.14509322	OK	0
SND1	6.25E-60	16.2855543	0.04180574	OK	2.49E-59
SND1-IT1	0.00011389	3.686039269	0.00938106	OK	0.00022926
LEP	2.41E-10	6.224828497	0.01520311	OK	5.93E-10
RBM28	0.00013933	3.634379899	0.00927103	OK	0.00027917
IMPDH1	1.92E-298	36.90505142	0.09482537	OK	1.38E-297
HILPDA	0.00031918	3.414769373	0.00870067	OK	0.00062661
CALU	1.73E-247	33.578443	0.0862841	OK	1.16E-246
FLNC	5.29E-24	10.0360988	0.02567582	OK	1.64E-23
ATP6V1F	0	192.2403517	0.49435457	OK	0
IRF5	1.46E-65	17.0605731	0.04377118	OK	5.97E-65
TSPAN33	0	122.05656	0.31379114	OK	0
SMO	4.93E-06	4.420415115	0.01123709	OK	1.05E-05
STRIP2	0.00013589	3.640812766	0.00914361	OK	0.00027243

UBE2H	8.48E-131	24.30597356	0.06243632	OK	4.45E-130
CEP41	7.29E-06	4.334789489	0.01105938	OK	1.55E-05
MEST	0.00016796	3.58589955	0.00912297	OK	0.00033508
COPG2	4.45E-06	4.442206035	0.01133608	OK	9.54E-06
AC016831.7	1.96E-59	16.2155032	0.04161799	OK	7.80E-59
LINC00513	1.91E-47	14.42078811	0.036974	OK	7.12E-47
AC016831.1	3.36E-134	24.62555614	0.06322951	OK	1.78E-133
AC016831.5	3.33E-126	23.86749114	0.06129535	OK	1.72E-125
AC058791.1	0	44.47594558	0.11429695	OK	0
PODXL	0	38.23696272	0.09744958	OK	0
PLXNA4	8.67E-14	7.36792591	0.01868158	OK	2.30E-13
CHCHD3	3.58E-114	22.67935806	0.05825127	OK	1.78E-113
EXOC4	0.00010393	3.7092604	0.00946317	OK	0.00020963
AC009275.1	2.97E-10	6.191856765	0.01578905	OK	7.30E-10
AKR1B1	0	119.5165636	0.30730445	OK	0
BPGM	2.38E-25	10.33768906	0.02650164	OK	7.45E-25
CALD1	0	341.712694	0.87879692	OK	0
AGBL3	7.18E-05	3.801835645	0.00966588	OK	0.00014597
CYREN	3.35E-07	4.969839698	0.01270272	OK	7.51E-07
TMEM140	2.43E-24	10.11251131	0.02592352	OK	7.54E-24
WDR91	8.28E-14	7.37393087	0.0188752	OK	2.20E-13
CNOT4	2.86E-14	7.514526393	0.01925146	OK	7.64E-14
NUP205	1.06E-05	4.252116341	0.01084754	OK	2.24E-05
STMP1	0	60.95100886	0.15668751	OK	0
FAM180A	9.08E-117	22.94088787	0.0588467	OK	4.57E-116
MTPN	0	84.65467774	0.21765316	OK	0
PTN	0	54.05845918	0.13886784	OK	0
DGKI	4.71E-07	4.90332299	0.01247559	OK	1.05E-06
CREB3L2	7.65E-06	4.324393575	0.0110461	OK	1.62E-05
TRIM24	5.91E-17	8.284866573	0.02122449	OK	1.65E-16
SVOPL	1.80E-85	19.55693323	0.04020722	OK	8.09E-85
ZC3HAV1	5.05E-151	26.14908967	0.06717082	OK	2.80E-150
FMC1	2.70E-75	18.32316808	0.04704176	OK	1.16E-74
LUC7L2	3.89E-16	8.057501967	0.02064553	OK	1.07E-15
HIPK2	1.56E-24	10.15557099	0.02603696	OK	4.86E-24
TBXAS1	0	157.4806643	0.40493543	OK	0
PARP12	4.25E-68	17.39823635	0.04465036	OK	1.76E-67
KDM7A	3.42E-56	15.75021987	0.0404199	OK	1.33E-55
SLC37A3	4.99E-20	9.08916181	0.02325077	OK	1.46E-19
MKRN1	1.45E-30	11.43190186	0.02932487	OK	4.75E-30
DENND2A	8.48E-05	3.760481148	0.00935418	OK	0.00017173
ADCK2	1.40E-115	22.8215138	0.05860585	OK	7.03E-115
NDUFB2	0	123.3241089	0.31710437	OK	0
MRPS33	9.28E-115	22.73869127	0.05840401	OK	4.63E-114
KIAA1147	3.71E-09	5.78126317	0.01478406	OK	8.84E-09
SSBP1	0	73.5329992	0.18904834	OK	0
TAS2R4	8.88E-07	4.777467911	0.01178113	OK	1.96E-06
CLEC5A	0	128.8959785	0.33133867	OK	0
TRBV28	2.89E-14	7.512920717	0.01837898	OK	7.74E-14
TRBC1	2.46E-175	28.20610655	0.07236905	OK	1.45E-174
TRBC2	0	46.72602159	0.12000592	OK	0
EPHB6	1.25E-15	7.913617199	0.02026996	OK	3.42E-15
TRPV6	4.24E-11	6.491918145	0.01393189	OK	1.06E-10

GSTK1	0	88.95464423	0.22871237	OK	0
TMEM139	1.93E-14	7.565688926	0.01866262	OK	5.17E-14
CASP2	0.00019305	3.549413546	0.00904666	OK	0.00038395
AC093673.2	1.06E-05	4.252364328	0.01059995	OK	2.24E-05
AC093673.1	9.84E-36	12.42269551	0.0318676	OK	3.37E-35
ZYX	0	47.65824527	0.12249964	OK	0
TCAF1	1.11E-21	9.494006504	0.02433321	OK	3.34E-21
ARHGEF35	1.17E-05	4.230493905	0.01073711	OK	2.46E-05
OR2A1-AS1	0.00046476	3.311034656	0.00838775	OK	0.00090323
TPK1	1.81E-26	10.5820168	0.0271271	OK	5.71E-26
AC005229.4	2.04E-07	5.06495185	0.01292863	OK	4.62E-07
CUL1	2.27E-18	8.664290706	0.02220488	OK	6.50E-18
EZH2	0	42.86796786	0.11006711	OK	0
PDIA4	0	44.30224589	0.11386039	OK	0
ZNF467	2.38E-193	29.63854179	0.0761142	OK	1.46E-192
ATP6V0E2	7.51E-57	15.84583613	0.04066515	OK	2.94E-56
RARRES2	0	164.5814832	0.42318375	OK	0
REPIN1	4.61E-24	10.04967529	0.02576612	OK	1.43E-23
AC073111.5	4.06E-06	4.461981504	0.01137216	OK	8.72E-06
LINC00996	3.43E-08	5.394829859	0.01367403	OK	7.95E-08
GIMAP8	0	43.05728186	0.11059101	OK	0
GIMAP7	0	72.52404072	0.18641165	OK	0
GIMAP4	0	142.7279488	0.36698234	OK	0
GIMAP6	8.30E-154	26.3927615	0.06774064	OK	4.63E-153
GIMAP2	9.30E-269	35.00543456	0.08990978	OK	6.45E-268
GIMAP1	0	71.93710324	0.1848988	OK	0
GIMAP5	1.12E-21	9.492924857	0.02426648	OK	3.38E-21
TMEM176B	0	273.7577145	0.70400128	OK	0
TMEM176A	0	228.160678	0.58671707	OK	0
AOC1	7.35E-58	15.99131093	0.04014976	OK	2.89E-57
NOS3	1.09E-172	27.9895346	0.07062754	OK	6.37E-172
ATG9B	0.00023451	3.497858139	0.00846378	OK	0.00046398
ABCB8	3.07E-08	5.414558255	0.01384168	OK	7.13E-08
CDK5	1.34E-80	18.97615371	0.0487146	OK	5.90E-80
SLC4A2	4.07E-13	7.158619621	0.01832775	OK	1.07E-12
FASTK	8.21E-07	4.79302451	0.01225023	OK	1.81E-06
TMUB1	1.32E-53	15.3691067	0.03944876	OK	5.07E-53
AGAP3	5.88E-85	19.49647519	0.05005297	OK	2.64E-84
ABCF2.1	2.60E-05	4.046887485	0.01032617	OK	5.39E-05
CHPF2	1.68E-05	4.147588927	0.01058597	OK	3.52E-05
SMARCD3	0	43.35167728	0.11139069	OK	0
NUB1	2.24E-135	24.73518849	0.06354045	OK	1.19E-134
RHEB	0	81.83823823	0.21040942	OK	0
PRKAG2	2.34E-47	14.40696006	0.03697539	OK	8.70E-47
PRKAG2-AS1	0	44.94590075	0.11549482	OK	0
GALNT11	5.66E-13	7.113494639	0.01821643	OK	1.48E-12
KMT2C	6.40E-15	7.707806741	0.01974818	OK	1.73E-14
LINC01003	4.38E-27	10.71391702	0.02746978	OK	1.39E-26
XRCC2	2.50E-07	5.026296965	0.01260507	OK	5.63E-07
ACTR3B	0.00029957	3.431999661	0.00871824	OK	0.00058891
PAXIP1-AS1	1.51E-07	5.122458619	0.01308991	OK	3.42E-07
AC144652.1	2.16E-87	19.7812996	0.05075844	OK	9.78E-87
INSIG1	0	177.6641738	0.45685052	OK	0

AC009403.1	7.01E-06	4.343476547	0.01108169	OK	1.49E-05
LINC01006	3.72E-05	3.96156029	0.01008603	OK	7.66E-05
DNAJB6	0	75.91306321	0.19516954	OK	0
PTPRN2	4.64E-39	13.02108081	0.03338172	OK	1.63E-38
AC011899.2	1.26E-22	9.7184832	0.02488365	OK	3.82E-22
NCAPG2	1.24E-154	26.46442174	0.06785516	OK	6.98E-154
ESYT2	1.80E-30	11.41313165	0.02927439	OK	5.89E-30
WDR60	3.13E-11	6.53761129	0.01673674	OK	7.86E-11
VIPR2	1.26E-39	13.12031024	0.03360313	OK	4.44E-39
GTPBP6	6.34E-40	13.17216837	0.03380008	OK	2.24E-39
PPP2R3B	8.21E-06	4.308754257	0.01099115	OK	1.74E-05
CRLF2	3.78E-05	3.957891308	0.0096369	OK	7.78E-05
CSF2RA	0	115.0183627	0.29571285	OK	0
IL3RA	2.07E-81	19.07422689	0.04891201	OK	9.12E-81
SLC25A6	0	133.333333	0.34284114	OK	0
ASMTL	4.55E-16	8.03850347	0.02059543	OK	1.25E-15
AKAP17A	4.39E-18	8.589036258	0.02201196	OK	1.25E-17
DHRSX	2.69E-150	26.08507942	0.06700216	OK	1.49E-149
ZBED1	2.18E-15	7.844194531	0.02009407	OK	5.94E-15
CD99	0	131.5340004	0.33821531	OK	0
XG	1.24E-82	19.22076712	0.04923977	OK	5.50E-82
ARSD	0.00019598	3.545445972	0.00903849	OK	0.00038967
MXRA5	3.91E-73	18.05052929	0.04594281	OK	1.65E-72
PRKX	7.25E-61	16.41677361	0.04212521	OK	2.91E-60
AC110995.1	1.66E-41	13.44423933	0.03439368	OK	5.96E-41
PNPLA4	1.42E-12	6.985414509	0.01788301	OK	3.68E-12
ANOS1	2.34E-18	8.660855831	0.02214883	OK	6.70E-18
TBL1X	2.66E-46	14.23802267	0.03653214	OK	9.81E-46
SHROOM2	2.77E-05	4.031318779	0.00923463	OK	5.74E-05
WWC3	1.36E-09	5.947133443	0.01521394	OK	3.29E-09
CLCN4	1.45E-10	6.304113819	0.01600836	OK	3.59E-10
MID1	4.05E-100	21.2077973	0.05439406	OK	1.93E-99
HCCS	1.53E-159	26.88762593	0.06906623	OK	8.67E-159
ARHGAP6	1.14E-29	11.25117881	0.0288561	OK	3.72E-29
AC004554.2	0.00015925	3.599766066	0.00882893	OK	0.00031826
MSL3	7.21E-215	31.26399149	0.08032527	OK	4.61E-214
PRPS2	1.12E-91	20.272524	0.05205199	OK	5.19E-91
TLR7	0	61.41844902	0.15781109	OK	0
TLR8	2.48E-291	36.45900171	0.09363811	OK	1.77E-290
TMSB4X	0	192.7484066	0.4955614	OK	0
LINC02154	2.89E-08	5.425774139	0.01331688	OK	6.71E-08
EGFL6	3.16E-63	16.74350826	0.04225913	OK	1.28E-62
RAB9A	1.61E-204	30.49306986	0.0783466	OK	1.01E-203
TRAPPC2	1.21E-06	4.714092369	0.0120429	OK	2.66E-06
OFD1	1.91E-69	17.5751404	0.04512044	OK	7.96E-69
GPM6B	7.58E-155	26.48313209	0.06797175	OK	4.25E-154
FANCB	3.35E-11	6.527400773	0.01666857	OK	8.41E-11
MOSPD2	3.77E-23	9.840469122	0.0252269	OK	1.15E-22
ASB9	4.26E-10	6.134953843	0.01565601	OK	1.04E-09
PIGA	6.50E-07	4.839889261	0.01235648	OK	1.44E-06
PIR	1.44E-65	17.06128487	0.04378225	OK	5.90E-65
BMX	1.35E-106	21.89821042	0.05553739	OK	6.58E-106
ZRSR2	1.02E-09	5.994252859	0.01533785	OK	2.48E-09

AP1S2	0	102.5623788	0.26370803	OK	0
SYAP1	0	54.69300504	0.14058884	OK	0
RBBP7	0	38.00225526	0.09766121	OK	0
REPS2	2.19E-05	4.086385064	0.01042086	OK	4.56E-05
NHS	1.61E-28	11.01537093	0.02822481	OK	5.20E-28
SCML1	7.00E-34	12.07680307	0.03097962	OK	2.37E-33
RAI2	3.16E-74	18.18895033	0.04663698	OK	1.34E-73
CDKL5	5.95E-08	5.294952617	0.01352879	OK	1.37E-07
ADGRG2	0.00012913	3.653930421	0.00914879	OK	0.00025912
PDHA1	1.29E-90	20.15213953	0.05175196	OK	5.91E-90
SH3KBP1	0	72.5917203	0.18661766	OK	0
BCLAF3	0.00021323	3.523143576	0.00898191	OK	0.00042291
EIF1AX	0	44.82866345	0.11522246	OK	0
RPS6KA3	2.22E-154	26.44251955	0.06792251	OK	1.24E-153
CNKSR2	3.68E-14	7.481303681	0.01900135	OK	9.83E-14
SMPX	3.89E-78	18.67591979	0.04779003	OK	1.69E-77
SMS	0	74.67089809	0.19197341	OK	0
PRDX4	0	58.3970564	0.15011857	OK	0
ACOT9	1.66E-93	20.47886397	0.0525921	OK	7.73E-93
SAT1	0	257.527982	0.66226564	OK	0
APOO	8.99E-17	8.234795026	0.02109221	OK	2.51E-16
EIF2S3	1.42E-198	30.04120526	0.0771882	OK	8.80E-198
ZFX	2.79E-07	5.00542116	0.01279724	OK	6.27E-07
CXorf21	0	91.7765362	0.23592843	OK	0
GK	0	156.6012431	0.40267973	OK	0
GK-AS1	1.68E-07	5.10205724	0.01300462	OK	3.81E-07
DMD	0	90.74365672	0.23327708	OK	0
TMEM47	0	135.7219775	0.34894741	OK	0
PRRG1	2.03E-06	4.607779349	0.01175563	OK	4.43E-06
CYBB	0	246.3654282	0.63355274	OK	0
DYNLT3	0	49.64247831	0.12760055	OK	0
SRPX	3.96E-114	22.67497281	0.0581714	OK	1.97E-113
RPGR	5.08E-12	6.804276818	0.01741445	OK	1.30E-11
TSPAN7	8.14E-136	24.77598023	0.06353622	OK	4.34E-135
MID1IP1	1.03E-128	24.10802993	0.06192432	OK	5.38E-128
AC091808.1	5.86E-20	9.071754035	0.02310531	OK	1.71E-19
ATP6AP2	0	101.9541465	0.26214458	OK	0
CXorf38	8.20E-208	30.74045051	0.07897512	OK	5.18E-207
MED14OS	0.00013073	3.650763816	0.00930342	OK	0.00026226
USP9X	1.30E-17	8.462819978	0.02168858	OK	3.69E-17
DDX3X	0	47.63960055	0.12245157	OK	0
CASK	7.63E-13	7.072069509	0.01810609	OK	1.99E-12
GPR34	0	230.4645393	0.59261156	OK	0
GPR82	2.93E-29	11.16775452	0.02859575	OK	9.52E-29
MAOA	2.69E-289	36.3303168	0.0933284	OK	1.91E-288
MAOB	1.45E-210	30.94578155	0.07943706	OK	9.19E-210
FUNDC1	6.06E-16	8.003224813	0.02050334	OK	1.67E-15
CXorf36	0	78.09253083	0.19749376	OK	0
MIR222HG	7.43E-10	6.045936488	0.01545673	OK	1.81E-09
AC234772.3	0.0004338	3.330278016	0.0083928	OK	0.00084544
KRBOX4	0.00016772	3.586264762	0.00914132	OK	0.00033464
CHST7	1.96E-22	9.673155921	0.02478669	OK	5.95E-22
SLC9A7	6.81E-26	10.45696041	0.02678443	OK	2.14E-25

RP2	3.79E-126	23.86208995	0.06127938	OK	1.96E-125
RGN	0	43.92115981	0.11283813	OK	0
NDUFB11	0	103.2652201	0.26551598	OK	0
UBA1	1.20E-11	6.678918605	0.0170994	OK	3.06E-11
CDK16	7.46E-07	4.812221834	0.01229433	OK	1.65E-06
USP11	2.19E-54	15.48513788	0.03974049	OK	8.43E-54
SYN1	3.48E-06	4.495067031	0.01142202	OK	7.50E-06
TIMP1	0	178.175965	0.45816781	OK	0
CFP	0	106.3744067	0.27334335	OK	0
ELK1	2.14E-20	9.180656459	0.02352437	OK	6.33E-20
UXT	0	66.06037138	0.16982952	OK	0
SLC38A5	2.23E-05	4.082562533	0.01036063	OK	4.64E-05
FTSJ1	8.31E-06	4.305982581	0.01099479	OK	1.76E-05
EBP	2.01E-136	24.83218413	0.06377991	OK	1.08E-135
RBM3	0	63.25796644	0.16262144	OK	0
WDR13	3.51E-98	20.99674758	0.05392176	OK	1.66E-97
WAS	0	110.9824264	0.28534738	OK	0
GATA1	2.21E-17	8.400934414	0.01847279	OK	6.23E-17
TIMM17B	1.79E-192	29.57046215	0.07597598	OK	1.09E-191
PQBP1	1.87E-209	30.86303154	0.07929936	OK	1.19E-208
SLC35A2	6.57E-12	6.767177233	0.01732367	OK	1.68E-11
PIM2	6.52E-62	16.56231187	0.04249335	OK	2.63E-61
OTUD5	1.77E-05	4.1361345	0.01055997	OK	3.69E-05
GRIPAP1	2.20E-18	8.667955191	0.02221481	OK	6.30E-18
PRAF2	0	39.70803674	0.10202129	OK	0
WDR45	7.86E-55	15.55078466	0.03991804	OK	3.04E-54
GPKOW	1.41E-06	4.683438372	0.01196534	OK	3.09E-06
PLP2	0	102.9471395	0.26469909	OK	0
SYP	2.18E-12	6.924867854	0.01771393	OK	5.63E-12
CCDC22	3.94E-21	9.361297728	0.02399472	OK	1.17E-20
FOXP3	2.84E-50	14.86352378	0.03420954	OK	1.07E-49
PPP1R3F	3.11E-12	6.874706525	0.01755841	OK	7.99E-12
CLCN5	3.81E-09	5.776657857	0.01475553	OK	9.09E-09
SHROOM4	3.83E-34	12.12642606	0.03075065	OK	1.30E-33
NUDT10	5.02E-14	7.44045082	0.01895345	OK	1.34E-13
AL158055.1	0.00012856	3.655064333	0.00865158	OK	0.00025804
GSPT2	2.51E-23	9.881402336	0.02532209	OK	7.70E-23
MAGED1	8.85E-97	20.84284588	0.05350367	OK	4.16E-96
TSPYL2	2.16E-61	16.4900052	0.04232957	OK	8.71E-61
KANTR	5.09E-06	4.413289525	0.01125148	OK	1.09E-05
IQSEC2	1.42E-09	5.940808268	0.01519522	OK	3.42E-09
SMC1A	1.57E-75	18.35277123	0.04712452	OK	6.73E-75
HSD17B10	2.44E-204	30.47942904	0.07831372	OK	1.53E-203
HUWE1	6.61E-12	6.766204045	0.01732549	OK	1.69E-11
WNK3	0.00024857	3.482297379	0.00885105	OK	0.00049118
TSR2	0	40.80073294	0.10485535	OK	0
MAGED2	0	119.6844274	0.3077405	OK	0
APEX2	5.12E-13	7.127196844	0.01824552	OK	1.34E-12
FAM104B	1.33E-15	7.906263232	0.02025465	OK	3.63E-15
MAGEH1	7.70E-297	36.80492925	0.09456326	OK	5.54E-296
USP51	4.72E-15	7.746614566	0.01983276	OK	1.28E-14
RRAGB	0.00017974	3.568168748	0.00909818	OK	0.00035797
KLF8	1.74E-08	5.515529522	0.01406853	OK	4.07E-08

NBDY	0	41.37196389	0.1063308	OK	0
SPIN2B	1.94E-28	10.99842848	0.02819147	OK	6.27E-28
ARHGEF9	2.42E-117	22.99830696	0.05906078	OK	1.22E-116
LAS1L	3.28E-05	3.991713629	0.01018826	OK	6.77E-05
MSN	0	68.99657778	0.17738127	OK	0
AL034397.3	1.42E-58	16.09342779	0.04124194	OK	5.61E-58
VSIG4	0	203.9369759	0.5244241	OK	0
HEPH	1.79E-55	15.64538367	0.04010454	OK	6.93E-55
EDA2R	2.88E-08	5.426302171	0.01384233	OK	6.69E-08
AR	0	50.86107167	0.13068924	OK	0
YIPF6	2.29E-45	14.08674392	0.03615234	OK	8.39E-45
STARD8	1.01E-27	10.84850011	0.02778155	OK	3.25E-27
PJA1	2.67E-21	9.402406759	0.0240883	OK	7.96E-21
EDA	3.70E-16	8.063644049	0.02063464	OK	1.02E-15
IGBP1	3.89E-277	35.55185772	0.09136012	OK	2.72E-276
PDZD11	1.68E-91	20.25286477	0.0520091	OK	7.74E-91
KIF4A	0	95.96178268	0.24451087	OK	0
DLG3	1.57E-123	23.60868408	0.06058201	OK	8.04E-123
IL2RG	0	63.61941431	0.16351339	OK	0
AL590764.1	3.53E-05	3.974107042	0.01010876	OK	7.28E-05
NONO	1.12E-68	17.47437894	0.04486629	OK	4.67E-68
ITGB1BP2	4.11E-07	4.930043534	0.0125357	OK	9.18E-07
OGT	6.57E-13	7.092851334	0.01816376	OK	1.71E-12
CXCR3	0	40.76330439	0.10451684	OK	0
LINC00891	1.19E-19	8.994400465	0.02301295	OK	3.46E-19
NHSL2	7.97E-08	5.241313971	0.01339081	OK	1.83E-07
PIN4	3.75E-170	27.78031432	0.07137231	OK	2.18E-169
ERCC6L	3.85E-46	14.21209444	0.03518856	OK	1.42E-45
RPS4X	0	185.2610535	0.47632571	OK	0
PHKA1	0.00047535	3.304724105	0.00836696	OK	0.00092316
NAP1L2	2.00E-31	11.60236282	0.02969043	OK	6.62E-31
CHIC1	1.56E-09	5.924705798	0.01513589	OK	3.77E-09
JPX	1.47E-07	5.126899862	0.01310954	OK	3.34E-07
FTX	1.22E-104	21.69192361	0.05570515	OK	5.91E-104
SLC16A2	7.77E-12	6.742673673	0.01720548	OK	1.99E-11
ABCB7	3.28E-15	7.79251526	0.01995994	OK	8.93E-15
UPRT	1.69E-05	4.146156852	0.01058006	OK	3.54E-05
PBDC1	1.07E-289	36.35560279	0.09342508	OK	7.64E-289
ATRX	1.03E-102	21.48714268	0.05518884	OK	4.93E-102
MAGT1	1.44E-50	14.90913156	0.03826701	OK	5.44E-50
COX7B	0	98.87918507	0.25423619	OK	0
PGK1	0	185.295938	0.47649139	OK	0
TAF9B	2.38E-72	17.95053389	0.04605841	OK	1.00E-71
CYSLTR1	0	53.77197627	0.13817598	OK	0
P2RY10	0	50.821616	0.13028124	OK	0
GPR174	5.92E-16	8.006104255	0.02041675	OK	1.63E-15
ITM2A	0	41.13201486	0.10564388	OK	0
HMGN5	4.69E-31	11.5293678	0.02956648	OK	1.55E-30
SH3BGRL	0	144.8509753	0.37246251	OK	0
RPS6KA6	2.60E-37	12.70995017	0.03255741	OK	9.02E-37
HDX	1.08E-22	9.733791567	0.0249424	OK	3.29E-22
APOOL	6.77E-88	19.83961642	0.05093974	OK	3.08E-87
ZNF711	1.54E-31	11.6250826	0.02977076	OK	5.09E-31

CHM	2.03E-10	6.251772848	0.01600294	OK	5.00E-10
DACH2	2.63E-05	4.043793417	0.00907797	OK	5.46E-05
PABPC5	5.29E-39	13.01105491	0.03330904	OK	1.86E-38
PCDH11X	6.24E-49	14.65521603	0.03754153	OK	2.34E-48
NAP1L3	1.03E-153	26.3844316	0.06771377	OK	5.77E-153
FAM133A	2.55E-14	7.529363423	0.01921098	OK	6.83E-14
DIAPH2	3.43E-08	5.394600007	0.01379912	OK	7.96E-08
TSPAN6	2.68E-51	15.0208564	0.0384942	OK	1.02E-50
SRPX2	2.88E-16	8.094305069	0.02058445	OK	7.97E-16
SYTL4	3.10E-08	5.412815667	0.01377081	OK	7.20E-08
CSTF2	2.37E-05	4.06783071	0.01037696	OK	4.93E-05
TMEM35A	8.07E-77	18.51326051	0.04745124	OK	3.49E-76
CENPI	1.27E-11	6.670489508	0.01691546	OK	3.24E-11
TIMM8A	9.27E-24	9.980564987	0.02557394	OK	2.86E-23
BTK	0	55.71793177	0.14319721	OK	0
RPL36A	0	51.74811793	0.13301876	OK	0
GLA	0	81.97139397	0.21073518	OK	0
HNRNPH2	2.67E-65	17.02518573	0.04371036	OK	1.09E-64
ARMCX4	9.27E-07	4.768820385	0.01216842	OK	2.04E-06
ARMCX1	8.54E-137	24.86664046	0.06387189	OK	4.57E-136
ARMCX6	3.54E-61	16.46028045	0.04225511	OK	1.42E-60
ARMCX3	3.90E-92	20.32449605	0.05219704	OK	1.81E-91
ARMCX2	2.24E-46	14.25012363	0.03655064	OK	8.26E-46
ZMAT1	3.79E-16	8.060725362	0.02064218	OK	1.05E-15
TCEAL2	0	125.7467315	0.32326785	OK	0
TCEAL6	4.85E-120	23.26645776	0.05961501	OK	2.46E-119
BEX5	3.68E-137	24.90047787	0.06391043	OK	1.97E-136
GPRASP1	1.37E-22	9.709543775	0.02484985	OK	4.17E-22
GPRASP2	1.50E-11	6.646494168	0.01693449	OK	3.80E-11
BEX1	4.13E-09	5.763219346	0.01469395	OK	9.83E-09
BEX4	0	47.47962534	0.12203928	OK	0
TCEAL8	6.65E-265	34.75119683	0.08930102	OK	4.59E-264
TCEAL5	1.13E-23	9.960780878	0.02547341	OK	3.48E-23
BEX2	1.14E-161	27.06900448	0.06947972	OK	6.48E-161
TCEAL7	2.55E-161	27.0393513	0.06939297	OK	1.45E-160
TCEAL9	0	44.89451097	0.1153868	OK	0
BEX3	0	163.725152	0.42100665	OK	0
TCEAL4	0	106.7029284	0.27435731	OK	0
TCEAL3	0	76.91593306	0.19774418	OK	0
TCEAL1	0	50.19813416	0.12901783	OK	0
MORF4L2	0	46.52460257	0.11958158	OK	0
RAB9B	1.68E-45	14.10873017	0.03615	OK	6.15E-45
TMSB15B-AS1	0.00035518	3.385549356	0.00844604	OK	0.00069561
TMSB15B.1	2.67E-15	7.818852862	0.01993895	OK	7.25E-15
FAM199X	8.95E-07	4.775692942	0.0122052	OK	1.97E-06
NRK	1.56E-05	4.164819348	0.01056737	OK	3.27E-05
MORC4	1.05E-56	15.82456569	0.04059578	OK	4.12E-56
PRPS1	3.26E-38	12.87147641	0.03302388	OK	1.14E-37
TSC22D3	0	44.36905468	0.11403975	OK	0
PSMD10	3.56E-30	11.35347615	0.0291189	OK	1.16E-29
ATG4A	2.15E-07	5.055349482	0.01291797	OK	4.85E-07
COL4A6	4.87E-18	8.577045861	0.02191453	OK	1.39E-17
COL4A5	8.34E-89	19.94466645	0.05116338	OK	3.80E-88

NXT2	1.10E-19	9.002954155	0.02306266	OK	3.20E-19
KCNE5	3.30E-09	5.800696181	0.01472254	OK	7.88E-09
ACSL4	1.26E-296	36.79156785	0.09454207	OK	9.06E-296
TMEM164	1.58E-09	5.922916504	0.01513739	OK	3.81E-09
CHRDL1	0	53.35980346	0.13643649	OK	0
PAK3	2.89E-13	7.205589992	0.01840098	OK	7.59E-13
ALG13	4.38E-82	19.1552479	0.0491876	OK	1.94E-81
AMOT	9.68E-20	9.016788368	0.0230412	OK	2.82E-19
IL13RA2	9.04E-160	26.90720042	0.06901016	OK	5.12E-159
LRCH2	2.48E-19	8.91300241	0.02279838	OK	7.20E-19
PLS3	0	243.4698008	0.62609895	OK	0
KLHL13	2.33E-14	7.541100018	0.01925519	OK	6.24E-14
DOCK11	3.77E-28	10.93859213	0.02805401	OK	1.21E-27
IL13RA1	0	58.39368876	0.15010567	OK	0
LONRF3	1.18E-45	14.13352298	0.03624254	OK	4.33E-45
PGRMC1	0	97.69096289	0.25117492	OK	0
AC004973.1	3.62E-16	8.066498978	0.01773915	OK	1.00E-15
SLC25A43	1.60E-17	8.43867087	0.02161242	OK	4.53E-17
SLC25A5	0	146.2325834	0.37602687	OK	0
UBE2A	0	63.79670225	0.16400514	OK	0
NKRF	1.69E-11	6.628672013	0.01696037	OK	4.28E-11
SEPT6	0	87.37399196	0.22462635	OK	0
SOWAHD	2.36E-76	18.4554511	0.04733779	OK	1.02E-75
RPL39	0	184.9121282	0.47543416	OK	0
UPF3B	2.69E-13	7.215107399	0.01847917	OK	7.08E-13
RNF113A	1.95E-26	10.57483288	0.0271169	OK	6.16E-26
NDUFA1	0	91.29079826	0.23471923	OK	0
NKAP	8.57E-27	10.65157057	0.02731931	OK	2.72E-26
RHOXF1-AS1	2.99E-05	4.013869531	0.00876878	OK	6.18E-05
TMEM255A	1.33E-08	5.562297686	0.01408101	OK	3.13E-08
LAMP2	0	89.1965632	0.22933149	OK	0
MCTS1	6.96E-273	35.27555761	0.0906478	OK	4.85E-272
C1GALT1C1	1.69E-41	13.44287928	0.03449498	OK	6.06E-41
XIAP	1.69E-30	11.41862782	0.02929038	OK	5.53E-30
STAG2	6.93E-72	17.89099541	0.04593894	OK	2.92E-71
SH2D1A	3.10E-19	8.888218857	0.02269483	OK	8.99E-19
SMARCA1	0	44.324847	0.11388278	OK	0
APLN	3.73E-10	6.156192405	0.01551618	OK	9.13E-10
SASH3	1.19E-169	27.73867638	0.0712326	OK	6.93E-169
UTP14A	3.33E-07	4.971063165	0.01270625	OK	7.47E-07
ELF4	4.69E-11	6.47666278	0.0165556	OK	1.17E-10
AIFM1	1.18E-50	14.92230345	0.03829171	OK	4.47E-50
RAB33A	8.53E-73	18.00732468	0.04615992	OK	3.61E-72
RBMX2	6.43E-40	13.17114001	0.03379671	OK	2.27E-39
ENOX2	4.10E-10	6.140951843	0.01571334	OK	1.00E-09
STK26	1.03E-14	7.646548712	0.01957617	OK	2.78E-14
RAP2C	1.74E-22	9.685199507	0.02482682	OK	5.29E-22
MBNL3	5.46E-19	8.825244685	0.02258806	OK	1.57E-18
GPC4	1.46E-90	20.14591252	0.05168655	OK	6.70E-90
GPC3	1.62E-141	25.29942711	0.06484749	OK	8.78E-141
PHF6	5.78E-23	9.797301419	0.02511952	OK	1.77E-22
HPRT1	0	46.34073071	0.11910414	OK	0
MIR503HG	1.45E-13	7.298614834	0.01860182	OK	3.84E-13

MOSPD1	1.26E-09	5.959869368	0.01524389	OK	3.05E-09
SMIM10	0	105.1425866	0.27030102	OK	0
RTL8B	6.63E-276	35.47204971	0.09111574	OK	4.63E-275
RTL8C	0	59.69834127	0.15345975	OK	0
RTL8A	0	54.77769648	0.14079791	OK	0
ZNF449	0.00029135	3.439541865	0.00875772	OK	0.00057305
MMGT1	3.27E-25	10.30708242	0.02642951	OK	1.02E-24
FHL1	0	258.1480554	0.66385249	OK	0
MAP7D3	0	40.72980323	0.10466699	OK	0
ADGRG4	1.73E-05	4.14044346	0.00943901	OK	3.63E-05
HTATSF1	6.61E-76	18.39969633	0.04724424	OK	2.84E-75
CD40LG	2.93E-76	18.44377577	0.04716469	OK	1.26E-75
ARHGEF6	9.16E-99	21.06050606	0.05407644	OK	4.34E-98
RBMX	9.00E-77	18.50741671	0.04752481	OK	3.89E-76
FGF13	4.40E-83	19.27443544	0.04944305	OK	1.96E-82
FGF13-AS1	3.75E-05	3.959663059	0.01005789	OK	7.72E-05
ATP11C	0.00035512	3.385592446	0.00862785	OK	0.00069556
LINC00632	3.66E-13	7.173265376	0.01825866	OK	9.60E-13
CDR1	1.18E-06	4.719732551	0.01193013	OK	2.59E-06
AL078639.1	1.49E-228	32.25509699	0.08282087	OK	9.79E-228
LDOC1	0	158.8421108	0.40840127	OK	0
FMR1	1.44E-10	6.304909022	0.0161388	OK	3.57E-10
AFF2	2.21E-05	4.084549184	0.00979417	OK	4.60E-05
IDS	5.30E-20	9.082543814	0.02328533	OK	1.55E-19
CXorf40A	4.60E-15	7.749808016	0.01984336	OK	1.25E-14
CXorf40B	3.96E-21	9.360759778	0.02398737	OK	1.18E-20
CD99L2	1.80E-16	8.15146391	0.02087543	OK	4.99E-16
HMGB3	0	73.93681943	0.19001604	OK	0
VMA21	0	63.98879862	0.16449531	OK	0
PRRG3	2.19E-10	6.239716775	0.0151817	OK	5.40E-10
GABRE	3.94E-09	5.771025707	0.01469234	OK	9.39E-09
CETN2	2.12E-154	26.44436707	0.06792762	OK	1.19E-153
NSDHL	1.45E-18	8.715368317	0.02232458	OK	4.16E-18
ZNF185	1.25E-06	4.708274162	0.01201466	OK	2.74E-06
TREX2	1.16E-12	7.013913933	0.01723055	OK	3.01E-12
BGN	0	291.3916766	0.74936247	OK	0
CCNQ	4.97E-46	14.19423459	0.03642734	OK	1.83E-45
PNCK	1.87E-10	6.264576767	0.01592719	OK	4.61E-10
SLC6A8	3.76E-27	10.72793042	0.02748704	OK	1.20E-26
BCAP31	0	101.4971585	0.26097072	OK	0
ABCD1	1.88E-32	11.80299098	0.03026271	OK	6.30E-32
SRPK3	1.21E-24	10.18074669	0.02599899	OK	3.76E-24
IDH3G	2.03E-271	35.17986095	0.09040477	OK	1.41E-270
SSR4	0	114.9241565	0.29549712	OK	0
AVPR2	1.00E-06	4.752608306	0.01163398	OK	2.21E-06
ARHGAP4	0	67.05130802	0.17234461	OK	0
NAA10	1.28E-255	34.13108874	0.08770704	OK	8.68E-255
RENBP	0	56.75305271	0.14585659	OK	0
HCFC1	0.00041103	3.345257457	0.00852473	OK	0.00080207
TMEM187	9.02E-07	4.774169986	0.0121976	OK	1.99E-06
IRAK1	0	83.15125307	0.21376002	OK	0
MECP2	1.41E-35	12.39364921	0.03179824	OK	4.84E-35
TKTL1	1.36E-131	24.38087798	0.0622913	OK	7.18E-131

FLNA	0	168.0886936	0.43223627	OK	0
EMD	1.39E-124	23.71092998	0.06090686	OK	7.15E-124
RPL10	0	183.1572508	0.47081526	OK	0
DNASE1L1	3.28E-61	16.46483999	0.04226106	OK	1.32E-60
TAZ	2.49E-08	5.452069878	0.01394225	OK	5.80E-08
AC244090.1	1.41E-210	30.9465238	0.07950269	OK	8.99E-210
ATP6AP1	0	71.48261691	0.1837727	OK	0
GDI1	2.74E-05	4.033980302	0.01029945	OK	5.68E-05
FAM50A	7.07E-253	33.9456478	0.08723089	OK	4.80E-252
LAGE3	0	50.62518624	0.13012263	OK	0
UBL4A	2.58E-27	10.76279601	0.02759961	OK	8.23E-27
SLC10A3	6.24E-12	6.774557068	0.017343	OK	1.60E-11
FAM3A	7.04E-33	11.8854516	0.03048813	OK	2.36E-32
G6PD	1.22E-26	10.61865153	0.02723158	OK	3.87E-26
IKBKG	3.51E-12	6.857383182	0.01755677	OK	9.01E-12
GAB3	1.87E-18	8.68666716	0.02224805	OK	5.35E-18
DKC1	1.55E-57	15.94458458	0.04092762	OK	6.11E-57
MPP1	0	137.118751	0.35257622	OK	0
F8A1	3.08E-51	15.0117177	0.03852841	OK	1.17E-50
FUNDC2	0	39.74047812	0.10213431	OK	0
CMC4	2.54E-05	4.052177336	0.01033411	OK	5.27E-05
BRCC3	3.09E-17	8.361846962	0.02142614	OK	8.67E-17
VBP1	4.52E-118	23.07105822	0.05925939	OK	2.28E-117
RAB39B	4.41E-22	9.589900068	0.02447406	OK	1.33E-21
CLIC2	0	55.32799979	0.14217811	OK	0
TMLHE	0.00040227	3.351225804	0.00854016	OK	0.00078533
VAMP7	2.65E-12	6.897401012	0.01766038	OK	6.82E-12
ERICH1	7.48E-41	13.33261652	0.03421451	OK	2.66E-40
DLGAP2	5.94E-32	11.70598024	0.02874006	OK	1.97E-31
CLN8	0	68.89998609	0.17711282	OK	0
AC100810.1	0	56.70361145	0.14575483	OK	0
ARHGEF10	7.98E-54	15.40166745	0.03947265	OK	3.07E-53
KBTBD11	1.60E-13	7.285584011	0.01860769	OK	4.22E-13
ANGPT2	0	74.69825357	0.19199121	OK	0
AGPAT5	1.36E-09	5.947299523	0.015216	OK	3.29E-09
DEFB1	6.86E-06	4.34815067	0.01100311	OK	1.46E-05
PRAG1	8.86E-22	9.517573668	0.0243566	OK	2.67E-21
CLDN23	2.02E-30	11.40283567	0.0292231	OK	6.62E-30
MFHAS1	2.66E-19	8.905211007	0.02281463	OK	7.73E-19
ERI1	1.95E-11	6.607947876	0.01691008	OK	4.92E-11
PPP1R3B	9.41E-27	10.64291539	0.0272877	OK	2.98E-26
MSRA	5.27E-242	33.20064596	0.08530733	OK	3.52E-241
SOX7	5.81E-89	19.9627251	0.05027648	OK	2.65E-88
PINX1.1	8.99E-05	3.745805401	0.0095543	OK	0.00018186
NEIL2	3.96E-07	4.937297964	0.01261289	OK	8.85E-07
FDFT1	4.79E-78	18.66472285	0.04792344	OK	2.09E-77
CTSB	0	291.9960986	0.75092188	OK	0
AC145124.1	2.22E-40	13.25114206	0.03397109	OK	7.88E-40
TRMT9B	1.23E-20	9.239792458	0.02363201	OK	3.66E-20
DLC1	0	49.00872047	0.12593177	OK	0
C8orf48	2.40E-06	4.573149413	0.01162792	OK	5.21E-06
TUSC3	6.96E-109	22.13701038	0.05652704	OK	3.42E-108
MSR1	0	247.4741114	0.63640843	OK	0

FGF20	2.59E-05	4.047572053	0.01010731	OK	5.37E-05
MICU3	5.65E-245	33.40576117	0.08580089	OK	3.79E-244
ZDHHC2	4.45E-79	18.79123725	0.04824658	OK	1.95E-78
CNOT7	2.61E-37	12.70961963	0.03261173	OK	9.05E-37
VPS37A	1.75E-05	4.138239401	0.01056724	OK	3.66E-05
MTMR7	7.44E-11	6.406586351	0.01576589	OK	1.86E-10
SLC7A2	0	55.3419239	0.14216808	OK	0
PDGFRL	0	42.12733602	0.1082068	OK	0
MTUS1	0	43.40924403	0.11154068	OK	0
AC087273.2	2.68E-07	5.013306064	0.01268576	OK	6.02E-07
PCM1	3.53E-07	4.959599049	0.01268165	OK	7.91E-07
ASAH1	0	152.472658	0.39207614	OK	0
PSD3	4.31E-18	8.591071196	0.02200802	OK	1.23E-17
AC100849.1	4.54E-08	5.344281465	0.01353262	OK	1.05E-07
SH2D4A	1.04E-13	7.343810857	0.01875779	OK	2.75E-13
CSGALNACT1	2.87E-54	15.46757193	0.03968252	OK	1.11E-53
INTS10	1.01E-19	9.012210212	0.0231016	OK	2.94E-19
LPL	0	125.7629544	0.32309105	OK	0
ATP6V1B2	0	103.2516544	0.26547518	OK	0
LZTS1	1.10E-10	6.346629843	0.01612247	OK	2.73E-10
GFRA2	0	39.54558402	0.10154707	OK	0
DOK2	0	149.1613447	0.38354688	OK	0
DMTN	5.39E-54	15.42694635	0.0395323	OK	2.08E-53
REEP4	1.41E-177	28.38841407	0.07291013	OK	8.30E-177
BMP1	4.45E-20	9.101663851	0.02328348	OK	1.31E-19
SLC39A14	4.69E-08	5.3382525	0.01363142	OK	1.08E-07
SORBS3	0	38.8312492	0.09978298	OK	0
PDLIM2	0	79.60226986	0.20465044	OK	0
EGR3	5.53E-40	13.18257535	0.03381029	OK	1.95E-39
PEBP4	2.59E-21	9.40557558	0.02406633	OK	7.73E-21
TNFRSF10B	5.24E-123	23.55766673	0.06049724	OK	2.68E-122
AC107959.4	1.12E-05	4.240512519	0.01075214	OK	2.35E-05
TNFRSF10A	3.92E-16	8.0566884	0.02062016	OK	1.08E-15
R3HCC1	1.91E-39	13.08858659	0.03358317	OK	6.73E-39
SLC25A37	2.57E-173	28.04102925	0.07203875	OK	1.50E-172
STC1	2.71E-111	22.38568432	0.05656243	OK	1.34E-110
ADAM28	0	106.6642963	0.27420921	OK	0
ADAMDEC1	0	70.63044123	0.18151024	OK	0
NEFL	2.77E-81	19.05889717	0.04620798	OK	1.22E-80
DOCK5	2.14E-17	8.405012865	0.02153836	OK	6.03E-17
KCTD9	4.25E-84	19.39506091	0.04978919	OK	1.90E-83
CDCA2	0	94.6241	0.24057646	OK	0
EBF2	4.44E-134	24.61431982	0.0627912	OK	2.35E-133
PPP2R2A	3.54E-44	13.89199269	0.03565271	OK	1.29E-43
BNIP3L	0	111.5672506	0.28686942	OK	0
PNMA2	2.77E-11	6.55556612	0.01672054	OK	6.98E-11
DPYSL2	0	65.95187947	0.16954645	OK	0
PTK2B	2.68E-79	18.81825898	0.0482951	OK	1.17E-78
EPHX2	3.99E-60	16.31292646	0.04183201	OK	1.60E-59
CLU	0	285.5533436	0.73434095	OK	0
SCARA3	0	65.54911102	0.16844636	OK	0
CCDC25	2.46E-144	25.55436	0.06564588	OK	1.34E-143
ESCO2	0	140.7906208	0.36045209	OK	0

PBK	0	97.26240042	0.24516231	OK	0
SCARA5	0	70.89223399	0.18137626	OK	0
PNOC	5.37E-11	6.456105581	0.01613998	OK	1.34E-10
ZNF395	1.28E-05	4.21017142	0.01074254	OK	2.69E-05
FZD3	1.72E-16	8.156645739	0.02083084	OK	4.79E-16
KIF13B	5.75E-20	9.07371255	0.0232572	OK	1.68E-19
DUSP4	0	56.42335567	0.14495735	OK	0
LINC02099	1.92E-08	5.497634167	0.01377206	OK	4.50E-08
SARAF	0	113.6753913	0.29228868	OK	0
AC044849.1	1.04E-05	4.255853524	0.01085598	OK	2.20E-05
LEPROTL1	0	48.87383721	0.12562219	OK	0
MBOAT4	0.00023922	3.492544239	0.00829071	OK	0.00047318
DCTN6	3.83E-128	24.05353785	0.06178699	OK	2.00E-127
RBPMS-AS1	2.79E-05	4.030231481	0.01024553	OK	5.77E-05
RBPMS	0	175.870105	0.45222099	OK	0
GTF2E2	9.28E-36	12.4273645	0.03188002	OK	3.18E-35
GSR	3.27E-101	21.32577321	0.05475603	OK	1.56E-100
UBXN8	5.41E-17	8.295435108	0.02124447	OK	1.51E-16
PPP2CB	4.57E-157	26.6752005	0.06852864	OK	2.57E-156
NRG1	3.41E-121	23.38001661	0.05978727	OK	1.74E-120
AC090204.1	6.22E-174	28.0915007	0.07213316	OK	3.65E-173
FUT10	0.00015571	3.605615487	0.00918175	OK	0.00031132
MAK16	7.28E-06	4.335391615	0.01106768	OK	1.55E-05
RNF122	1.39E-11	6.658283141	0.01702488	OK	3.51E-11
DUSP26	0	59.14102623	0.15195557	OK	0
ZNF703	1.64E-24	10.15121879	0.02602444	OK	5.08E-24
ERLIN2	4.13E-08	5.361487655	0.01370985	OK	9.54E-08
PLPBP	2.25E-45	14.08817722	0.03615576	OK	8.23E-45
ADGRA2	3.74E-108	22.06110309	0.05654661	OK	1.83E-107
BRF2	2.24E-12	6.921225316	0.01771216	OK	5.77E-12
RAB11FIP1	0	120.1771057	0.30900654	OK	0
ADRB3	1.66E-10	6.283141162	0.01572321	OK	4.10E-10
EIF4EBP1	0	114.9198581	0.29548623	OK	0
LSM1	6.53E-130	24.22197878	0.06221995	OK	3.42E-129
BAG4	2.16E-11	6.592562838	0.01687798	OK	5.45E-11
DDHD2	0.00038372	3.364277441	0.00857208	OK	0.00075024
PLPP5	1.35E-32	11.83119584	0.03034974	OK	4.51E-32
NSD3	7.17E-14	7.393169551	0.01894042	OK	1.90E-13
FGFR1	0	44.42526351	0.11416448	OK	0
TACC1	7.18E-91	20.18106818	0.05182993	OK	3.30E-90
PLEKHA2	8.04E-45	13.99777438	0.0359164	OK	2.94E-44
HTRA4	2.33E-12	6.915419472	0.01764467	OK	6.01E-12
TM2D2	0	37.67710938	0.09682227	OK	0
ADAM9	0	57.24279178	0.14714121	OK	0
IDO1	0	207.6848941	0.53191191	OK	0
IDO2	0	39.99980953	0.1004275	OK	0
TCIM	1.94E-104	21.67063561	0.05553093	OK	9.38E-104
SFRP1	0	38.88447984	0.09978133	OK	0
GOLGA7	8.84E-148	25.86231575	0.06643971	OK	4.85E-147
GINS4	7.27E-75	18.2692656	0.04682488	OK	3.10E-74
GPAT4	1.76E-07	5.092828052	0.01301627	OK	4.00E-07
ANK1	3.29E-05	3.991169615	0.01013646	OK	6.79E-05
KAT6A	3.74E-08	5.379023957	0.0137573	OK	8.67E-08

AP3M2	1.26E-14	7.620574104	0.0195113	OK	3.40E-14
PLAT	7.94E-221	31.69930711	0.08125157	OK	5.13E-220
IKBKB	1.75E-08	5.514815546	0.01410107	OK	4.08E-08
POLB	3.93E-87	19.75101953	0.05070269	OK	1.78E-86
VDAC3	1.73E-237	32.88616452	0.08450545	OK	1.15E-236
SLC20A2	3.32E-23	9.853232684	0.02525486	OK	1.02E-22
SMIM19	3.79E-170	27.77990744	0.07137166	OK	2.21E-169
THAP1	1.35E-12	6.992447936	0.01790475	OK	3.50E-12
HOOK3	3.22E-70	17.67581935	0.04538562	OK	1.35E-69
FNTA	7.60E-65	16.96388768	0.04355236	OK	3.10E-64
HGSNAT	5.38E-06	4.401314036	0.01124256	OK	1.15E-05
SPIDR	0.00043911	3.326884505	0.00847911	OK	0.00085535
CEBPD	0	162.1508001	0.41695739	OK	0
PRKDC	5.86E-89	19.96231814	0.05126577	OK	2.67E-88
MCM4	8.44E-151	26.12944717	0.06704901	OK	4.67E-150
UBE2V2	2.15E-210	30.93302287	0.07948171	OK	1.36E-209
SNAI2	2.22E-61	16.48851751	0.04226203	OK	8.92E-61
PCMTD1	1.16E-165	27.40602594	0.07041074	OK	6.68E-165
ST18	3.90E-37	12.67840462	0.03242552	OK	1.35E-36
ATP6V1H	2.69E-76	18.44835897	0.04736779	OK	1.16E-75
RGS20	3.63E-05	3.967916292	0.00955599	OK	7.47E-05
TCEA1	0	48.26661934	0.12406466	OK	0
LYPLA1	0	48.27038776	0.12406764	OK	0
MRPL15	0	38.14059064	0.09801277	OK	0
SOX17	0	95.84798888	0.2430935	OK	0
RP1	3.05E-05	4.009095522	0.00832852	OK	6.30E-05
TGS1	2.30E-31	11.59055657	0.02973231	OK	7.60E-31
LYN	0	171.0550339	0.43985832	OK	0
RPS20	0	118.1909408	0.30388636	OK	0
CHCHD7	0	45.25767622	0.11631797	OK	0
IMPAD1	2.26E-32	11.78766266	0.03023816	OK	7.55E-32
FAM110B	2.04E-20	9.186061868	0.02352793	OK	6.02E-20
UBXN2B	6.78E-35	12.26735621	0.03146479	OK	2.31E-34
SDCBP	0	199.2933856	0.51249554	OK	0
NSMAF	2.09E-46	14.25480288	0.03657342	OK	7.72E-46
TOX	1.33E-18	8.724709148	0.02227743	OK	3.83E-18
AC090152.1	2.66E-21	9.402755727	0.02401634	OK	7.94E-21
CA8	1.99E-05	4.108623269	0.01034203	OK	4.16E-05
RAB2A	0	68.27642651	0.17552853	OK	0
CHD7	6.61E-52	15.11335369	0.03876333	OK	2.52E-51
AC022182.2	6.18E-07	4.849788402	0.01230142	OK	1.37E-06
ASPH	3.21E-301	37.07771565	0.09528635	OK	2.33E-300
NKAIN3	2.47E-20	9.16532581	0.02328217	OK	7.29E-20
GGH	0	50.24859471	0.12905726	OK	0
YTHDF3-AS1	3.71E-08	5.380573166	0.01374046	OK	8.60E-08
YTHDF3	1.06E-32	11.85144588	0.03040067	OK	3.54E-32
BHLHE22	2.19E-08	5.474577669	0.01373697	OK	5.11E-08
CYP7B1	5.11E-134	24.60859907	0.06305577	OK	2.71E-133
ARMC1	3.16E-21	9.384606209	0.02405778	OK	9.42E-21
MTFR1	0.00045963	3.314139271	0.00844425	OK	0.00089373
PDE7A	2.79E-23	9.870698667	0.0252972	OK	8.56E-23
DNAJC5B	8.16E-20	9.035562393	0.02309563	OK	2.38E-19
RRS1	2.80E-07	5.004618385	0.01278869	OK	6.29E-07

ADHFE1	8.84E-05	3.750163699	0.0095495	OK	0.00017881
VXN	2.16E-05	4.089898435	0.01035024	OK	4.50E-05
MYBL1	3.87E-20	9.116887584	0.0233379	OK	1.14E-19
VCPIP1	3.98E-12	6.839201135	0.01751014	OK	1.02E-11
SGK3	2.75E-282	35.88361913	0.09220614	OK	1.94E-281
COPS5	1.04E-149	26.03314167	0.06687929	OK	5.76E-149
ARFGEF1	4.89E-07	4.896145387	0.0125164	OK	1.09E-06
PREX2	1.55E-182	28.78728289	0.07388364	OK	9.22E-182
C8orf34	1.06E-69	17.60848493	0.04513708	OK	4.42E-69
SULF1	0	101.7312358	0.26152405	OK	0
SLCO5A1	8.95E-52	15.09336611	0.03865779	OK	3.41E-51
AC079089.1	1.21E-10	6.331890055	0.01611537	OK	3.01E-10
TRAM1	0	52.26001329	0.13433534	OK	0
LACTB2	2.23E-46	14.25032116	0.03656865	OK	8.24E-46
MSC-AS1	1.59E-209	30.86826652	0.07926494	OK	1.01E-208
MSC	4.95E-218	31.49587049	0.0808803	OK	3.18E-217
TERF1	2.52E-39	13.06770108	0.03353071	OK	8.85E-39
SBSPON	0	103.2508628	0.26537944	OK	0
RPL7	0	153.25306	0.39404914	OK	0
RDH10	8.83E-07	4.77860186	0.01219627	OK	1.95E-06
UBE2W	1.76E-83	19.32176151	0.04961507	OK	7.85E-83
ELOC	0	95.1320363	0.24459999	OK	0
TMEM70	0	115.4315769	0.29680192	OK	0
LY96	0	177.28652	0.45589012	OK	0
PI15	1.64E-16	8.162919258	0.0205207	OK	4.55E-16
CRISPLD1	0	88.20242153	0.22671024	OK	0
ZFHX4	1.52E-26	10.59808548	0.0270613	OK	4.81E-26
PEX2	5.43E-33	11.90721237	0.03054686	OK	1.82E-32
ZC2HC1A	1.49E-174	28.1421842	0.07229163	OK	8.77E-174
IL7	5.98E-14	7.417312473	0.01897418	OK	1.59E-13
LINC01607	1.20E-09	5.968061099	0.01406034	OK	2.90E-09
MRPS28	7.67E-59	16.13141506	0.04140755	OK	3.04E-58
TPD52	1.72E-76	18.47260468	0.04740968	OK	7.40E-76
ZBTB10	8.51E-22	9.52175823	0.02440927	OK	2.56E-21
ZNF704	0	50.08512148	0.12870425	OK	0
PAG1	0	53.18553532	0.13668962	OK	0
AC079209.1	5.63E-11	6.448917537	0.01646901	OK	1.41E-10
FABP5	0	279.8316561	0.71961606	OK	0
FABP4	0	150.2418704	0.38625	OK	0
IMPA1	2.10E-05	4.095970502	0.0104578	OK	4.38E-05
ZFAND1	0	46.90122688	0.12054186	OK	0
CHMP4C	1.10E-192	29.58694607	0.0759281	OK	6.73E-192
SNX16	0.00014729	3.620025134	0.00923145	OK	0.00029483
RALYL	1.29E-07	5.151707015	0.01133037	OK	2.94E-07
LRRCC1	1.25E-67	17.33643912	0.0444959	OK	5.16E-67
C8orf59	0	54.0028404	0.13881799	OK	0
CA2	6.08E-266	34.81990922	0.08943744	OK	4.20E-265
ATP6V0D2	2.01E-97	20.91357665	0.05340504	OK	9.50E-97
WWP1	0	44.39930662	0.11409391	OK	0
RMDN1	9.09E-76	18.38240094	0.04719766	OK	3.91E-75
CPNE3	0	54.64977744	0.14047772	OK	0
MMP16	5.63E-21	9.323399961	0.02384487	OK	1.68E-20
RIPK2	0	71.96370657	0.18499565	OK	0

OSGIN2	3.78E-12	6.846547736	0.01752489	OK	9.72E-12
NBN	2.19E-35	12.35846706	0.03170791	OK	7.48E-35
DECR1	0	46.19080855	0.11872178	OK	0
NECAB1	1.03E-14	7.646904856	0.01950329	OK	2.78E-14
C8orf88	1.15E-179	28.55701265	0.07332038	OK	6.81E-179
PIP4P2	7.20E-104	21.61023645	0.05550193	OK	3.47E-103
OTUD6B-AS1	7.99E-132	24.4027918	0.06268498	OK	4.20E-131
OTUD6B	0.00034878	3.390534517	0.00864102	OK	0.00068369
RUNX1T1	1.79E-125	23.79700309	0.06108154	OK	9.24E-125
TRIQK	4.35E-07	4.918793005	0.01257294	OK	9.71E-07
FAM92A	7.54E-187	29.12977696	0.0748068	OK	4.54E-186
TMEM67	8.85E-09	5.633134533	0.01438356	OK	2.09E-08
PDP1	1.66E-15	7.878340859	0.02018278	OK	4.53E-15
GEM	0	124.544794	0.32022646	OK	0
VIRMA	9.77E-07	4.758124945	0.01216093	OK	2.15E-06
DPY19L4	7.00E-08	5.26536086	0.01345654	OK	1.61E-07
CCNE2	6.16E-141	25.24665419	0.06467007	OK	3.33E-140
TP53INP1	2.12E-05	4.093663498	0.01044488	OK	4.43E-05
PLEKHF2	1.06E-18	8.750924907	0.02242625	OK	3.04E-18
UQCRB	0	140.6673367	0.36169585	OK	0
MTERF3	0.00028493	3.445567414	0.00878139	OK	0.00056087
PTDSS1	3.15E-31	11.56349139	0.02965429	OK	1.04E-30
AP003548.1	1.38E-60	16.37776994	0.04192674	OK	5.52E-60
SDC2	0	104.373888	0.26836016	OK	0
CPQ	5.56E-246	33.47503063	0.08602001	OK	3.74E-245
MTDH	0	70.60846889	0.18152413	OK	0
LAPTM4B	0	43.69134313	0.1122593	OK	0
MATN2	2.13E-179	28.53535163	0.07319236	OK	1.26E-178
RPL30	0	193.386821	0.49719193	OK	0
ERICH5	1.11E-22	9.731249104	0.02388402	OK	3.38E-22
RIDA	3.93E-07	4.938739529	0.01262061	OK	8.79E-07
POP1	0.00010688	3.702185992	0.00943447	OK	0.0002154
STK3	0.00021432	3.521793122	0.00898101	OK	0.00042503
OSR2	8.96E-55	15.5423565	0.03974261	OK	3.47E-54
AC104986.2	1.36E-14	7.610919296	0.01946385	OK	3.66E-14
COX6C	0	133.2318478	0.34258221	OK	0
RGS22	3.26E-18	8.623004611	0.02180748	OK	9.31E-18
POLR2K	0	51.73815116	0.13299223	OK	0
SPAG1	2.03E-05	4.103717488	0.0104645	OK	4.24E-05
RNF19A	8.97E-13	7.049690262	0.01805595	OK	2.33E-12
ANKRD46	2.82E-09	5.827065376	0.01490004	OK	6.74E-09
PABPC1	0	125.5224689	0.32275709	OK	0
YWHAZ	0	82.91323607	0.21317176	OK	0
ZNF706	0	111.153286	0.28580599	OK	0
GRHL2	4.75E-09	5.739464033	0.0142764	OK	1.13E-08
NCALD	0	40.57465908	0.10422095	OK	0
RRM2B	1.46E-24	10.16218006	0.02605574	OK	4.55E-24
UBR5-AS1	3.41E-08	5.395935911	0.01378137	OK	7.90E-08
UBR5	0.00018782	3.55662922	0.00907146	OK	0.00037373
KLF10	1.56E-127	23.99516794	0.06163563	OK	8.11E-127
AZIN1	3.54E-131	24.34180294	0.06252393	OK	1.86E-130
ATP6V1C1	1.56E-159	26.88696144	0.06906885	OK	8.82E-159
BAALC	5.51E-05	3.867013608	0.00975731	OK	0.00011254

BAALC-AS1	0.00010522	3.706139583	0.00924111	OK	0.00021211
FZD6	1.32E-54	15.51752711	0.03977792	OK	5.10E-54
CTHRC1	0	38.95281053	0.10003729	OK	0
SLC25A32	7.34E-07	4.815446083	0.01230592	OK	1.62E-06
DCAF13	1.07E-137	24.94988891	0.06408726	OK	5.74E-137
DPYS	0.00026468	3.465438667	0.00860648	OK	0.00052223
DCSTAMP	2.05E-49	14.73045179	0.03767743	OK	7.73E-49
LRP12	3.39E-141	25.27026984	0.0648838	OK	1.84E-140
ZFPM2	8.61E-77	18.50979032	0.04747359	OK	3.72E-76
AC027031.2	2.59E-71	17.81733182	0.04571646	OK	1.09E-70
OXR1	3.35E-92	20.33203782	0.05221334	OK	1.55E-91
ABRA	4.95E-23	9.812938925	0.02486028	OK	1.51E-22
ANGPT1	0	104.1461042	0.26773683	OK	0
RSPO2	3.33E-105	21.75163163	0.05567284	OK	1.62E-104
EIF3E	0	95.9043228	0.24658459	OK	0
EMC2	3.33E-94	20.5570033	0.0527928	OK	1.55E-93
TMEM74	3.85E-07	4.942747768	0.01254177	OK	8.62E-07
NUDCD1	1.34E-10	6.31644838	0.01616149	OK	3.32E-10
ENY2	0	83.67221052	0.21512576	OK	0
EBAG9	4.07E-48	14.52730435	0.03728531	OK	1.52E-47
SYBU	5.01E-08	5.32631665	0.01354408	OK	1.16E-07
TRPS1	2.71E-67	17.29191763	0.04439383	OK	1.12E-66
EIF3H	0	98.61873376	0.25356567	OK	0
UTP23	1.97E-35	12.36712579	0.03172971	OK	6.72E-35
RAD21	1.02E-85	19.58602683	0.05029818	OK	4.58E-85
MED30	4.85E-54	15.43380633	0.03961698	OK	1.87E-53
EXT1	2.76E-163	27.20594082	0.06986314	OK	1.58E-162
TNFRSF11B	0	112.7348864	0.28977045	OK	0
NOV	0	199.9752316	0.51420351	OK	0
ENPP2	0	53.23381489	0.13681586	OK	0
DSCC1	7.69E-27	10.66166384	0.02723308	OK	2.44E-26
DEPTOR	1.30E-118	23.12499618	0.05934153	OK	6.56E-118
COL14A1	0	239.7359024	0.61649505	OK	0
MRPL13	0	38.15005998	0.09804249	OK	0
SNTB1	1.70E-59	16.2243825	0.04161299	OK	6.75E-59
HAS2	5.40E-09	5.717626636	0.0145153	OK	1.28E-08
ZHX2	1.00E-05	4.264241624	0.01087064	OK	2.12E-05
DERL1	5.88E-205	30.52606139	0.0784318	OK	3.69E-204
TBC1D31	3.10E-05	4.004900755	0.01019322	OK	6.41E-05
C8orf76	3.01E-128	24.06359007	0.06180073	OK	1.57E-127
ZHX1	4.51E-14	7.454437486	0.0190942	OK	1.20E-13
ATAD2	1.91E-150	26.09817454	0.06701285	OK	1.06E-149
WDYHV1	4.01E-09	5.767877833	0.01475032	OK	9.56E-09
FBXO32	0	106.9060024	0.27484026	OK	0
ANXA13	7.58E-51	14.95175873	0.03084763	OK	2.87E-50
FAM91A1	3.72E-73	18.05320829	0.04634619	OK	1.58E-72
AC090192.2	7.62E-10	6.041868206	0.01513766	OK	1.85E-09
TMEM65	2.40E-25	10.33677344	0.02650094	OK	7.52E-25
RNF139	7.58E-39	12.9836379	0.03331045	OK	2.65E-38
TATDN1	8.60E-27	10.65130681	0.02731737	OK	2.73E-26
NDUFB9	0	74.31889585	0.19106985	OK	0
MTSS1	0	117.3124603	0.30162233	OK	0
SQLE	5.03E-34	12.10402584	0.03104255	OK	1.70E-33

WASHC5	2.98E-08	5.4202429	0.01385927	OK	6.92E-08
NSMCE2	6.37E-36	12.4574992	0.03196019	OK	2.18E-35
TRIB1	0	62.26836284	0.16004222	OK	0
LINC00861	0.00037312	3.371999451	0.00850001	OK	0.00073004
PCAT1	2.54E-07	5.023548044	0.01252455	OK	5.71E-07
MYC	0	51.79550167	0.13307742	OK	0
PVT1	4.27E-10	6.134507179	0.01567276	OK	1.04E-09
CCDC26	8.81E-15	7.666906564	0.01916284	OK	2.38E-14
FAM49B	0	199.7575392	0.51368857	OK	0
ASAP1	0	46.2766248	0.11893692	OK	0
EFR3A	6.23E-60	16.28570826	0.04180868	OK	2.49E-59
KCNQ3	7.09E-28	10.88109728	0.02778837	OK	2.27E-27
LRRC6	7.65E-06	4.32422339	0.01101103	OK	1.63E-05
TMEM71	2.70E-69	17.55539384	0.04502966	OK	1.13E-68
PHF20L1	1.91E-15	7.860854691	0.02014238	OK	5.20E-15
SLA	0	93.78034281	0.24110322	OK	0
NDRG1	4.11E-61	16.45126527	0.04222792	OK	1.65E-60
ST3GAL1	3.46E-61	16.46163378	0.0422559	OK	1.39E-60
AC083843.2	0.00020082	3.539011019	0.00883304	OK	0.00039893
KHDRBS3	3.43E-40	13.21842061	0.03389597	OK	1.22E-39
AC079015.1	4.22E-15	7.760826171	0.019793	OK	1.15E-14
CHRAC1	5.73E-70	17.64330452	0.04529936	OK	2.39E-69
AGO2	2.22E-09	5.866854706	0.01501152	OK	5.33E-09
PTK2	0	69.41480676	0.17842899	OK	0
DENND3	1.07E-56	15.82379048	0.04060448	OK	4.17E-56
AC040970.1	3.36E-12	6.863283916	0.01754988	OK	8.65E-12
SLC45A4	4.52E-31	11.53263318	0.02954959	OK	1.49E-30
GPR20	0	42.95435967	0.11007626	OK	0
PTP4A3	0	140.5506627	0.36127597	OK	0
AC100803.3	3.72E-159	26.85463862	0.06869023	OK	2.10E-158
LY6K	1.20E-76	18.4918851	0.04742025	OK	5.18E-76
THEM6	4.95E-08	5.328669668	0.01361533	OK	1.14E-07
LYNX1	1.27E-42	13.63331066	0.03494734	OK	4.57E-42
LY6E	0	56.7993475	0.14600746	OK	0
GLI4	4.16E-08	5.359951135	0.01370395	OK	9.62E-08
MINCR	0.00019442	3.547538983	0.00903564	OK	0.00038662
TOP1MT	2.27E-19	8.92301725	0.02284703	OK	6.58E-19
RHPN1	3.08E-05	4.006257889	0.01016775	OK	6.37E-05
GSDMD	2.60E-125	23.7814423	0.06108514	OK	1.34E-124
NAPRT	6.79E-147	25.78349458	0.06623118	OK	3.72E-146
EEF1D	0	134.3700236	0.34549594	OK	0
PYCR3	3.38E-09	5.796757331	0.01480091	OK	8.07E-09
TSTA3	4.66E-56	15.73066175	0.04037792	OK	1.82E-55
FAM83H	7.45E-07	4.812582663	0.01218722	OK	1.65E-06
PUF60	1.41E-193	29.6562889	0.07619834	OK	8.62E-193
NRBP2	4.31E-19	8.851739075	0.02266666	OK	1.25E-18
PLEC	2.81E-60	16.33443312	0.04193216	OK	1.12E-59
PARP10	0.00025431	3.476171184	0.00886068	OK	0.00050226
GRINA	0	56.45449603	0.14511731	OK	0
EXOSC4	3.93E-76	18.42780414	0.04731374	OK	1.69E-75
GPAA1	7.64E-79	18.76256628	0.04817946	OK	3.34E-78
CYC1	0	54.68329505	0.1405679	OK	0
SHARPIN	9.60E-70	17.61413301	0.04522593	OK	4.00E-69

MAF1	0	39.59191143	0.10175281	OK	0
WDR97	0.00021222	3.524398149	0.00875306	OK	0.00042099
MROH1	3.52E-15	7.783807898	0.01992556	OK	9.56E-15
BOP1	1.13E-14	7.635048377	0.01955181	OK	3.04E-14
SCX	0	46.2960059	0.11891387	OK	0
HSF1	1.99E-31	11.60297479	0.02976375	OK	6.58E-31
DGAT1	1.86E-26	10.57937002	0.0271247	OK	5.87E-26
SLC52A2	5.36E-112	22.45788031	0.0576783	OK	2.66E-111
FBXL6	2.18E-17	8.402708782	0.02152427	OK	6.14E-17
CPSF1	0.00016601	3.588937867	0.00914383	OK	0.00033129
SLC39A4	0	38.28451626	0.09837106	OK	0
VPS28	0	76.09106422	0.19562562	OK	0
CYHR1	1.22E-130	24.29116497	0.06239592	OK	6.38E-130
PPP1R16A	5.82E-64	16.84388216	0.04322273	OK	2.37E-63
MFSD3	9.49E-12	6.713649643	0.01716974	OK	2.42E-11
RECQL4	7.34E-64	16.83009499	0.04264411	OK	2.99E-63
C8orf82	1.14E-25	10.40775956	0.02668911	OK	3.59E-25
ZNF251	6.19E-06	4.370902082	0.01114263	OK	1.32E-05
ZNF34	2.61E-05	4.0455801	0.01032135	OK	5.41E-05
RPL8	0	195.1373369	0.50170233	OK	0
COMMD5	1.10E-106	21.9075171	0.05626444	OK	5.37E-106
ZNF250	3.21E-07	4.977850306	0.01270955	OK	7.21E-07
C8orf33	3.87E-27	10.72542494	0.02750723	OK	1.23E-26
PGM5P3-AS1	2.84E-09	5.825673082	0.01294439	OK	6.80E-09
CBWD1	5.08E-19	8.833420994	0.02264026	OK	1.46E-18
C9orf66	2.66E-07	5.014354998	0.01280187	OK	5.99E-07
DOCK8	0	99.44348524	0.25567299	OK	0
KANK1	0	50.68655283	0.1302472	OK	0
SMARCA2	6.86E-23	9.77999928	0.02507708	OK	2.10E-22
VLDLR	2.60E-07	5.018720953	0.01279875	OK	5.86E-07
PUM3	2.15E-15	7.846109873	0.02010371	OK	5.85E-15
GLIS3	1.24E-11	6.67493385	0.01708367	OK	3.14E-11
CDC37L1	1.48E-31	11.62837955	0.02982532	OK	4.89E-31
AK3	4.40E-84	19.39319964	0.04979442	OK	1.97E-83
RCL1	1.09E-06	4.735187722	0.01209744	OK	2.40E-06
JAK2	8.64E-17	8.239630456	0.02111181	OK	2.41E-16
PLGRKT	6.22E-275	35.40893657	0.09098534	OK	4.34E-274
PDCD1LG2	3.15E-60	16.32739953	0.04185967	OK	1.26E-59
ERMP1	7.87E-08	5.243685716	0.0133683	OK	1.80E-07
IL33	0	70.59516171	0.18143679	OK	0
AL354707.1	0.00045181	3.318931069	0.00839524	OK	0.00087908
KDM4C	0.00018314	3.56326515	0.00908444	OK	0.0003646
DMAC1	1.79E-217	31.45511878	0.08082244	OK	1.15E-216
PTPRD	6.95E-186	29.05347272	0.07459455	OK	4.18E-185
PTPRD-AS1	8.49E-228	32.20118517	0.08265205	OK	5.57E-227
LURAP1L	1.75E-107	21.99106655	0.0564231	OK	8.58E-107
MPDZ	0	41.00451877	0.10534172	OK	0
LINC01235	0	48.47655916	0.11951319	OK	0
AL583785.1	0	39.37892756	0.09833758	OK	0
NFIB	0	120.7931905	0.31056685	OK	0
ZDHHC21	8.82E-05	3.750614831	0.00956746	OK	0.0001785
FREM1	4.44E-13	7.146728983	0.01821761	OK	1.16E-12
TTC39B	8.84E-30	11.2738145	0.02890118	OK	2.88E-29

SNAPC3	5.00E-35	12.29195883	0.03152421	OK	1.70E-34
PSIP1	0	99.30137396	0.25531908	OK	0
CCDC171	1.70E-06	4.645555348	0.0118614	OK	3.70E-06
BNC2	0.00022312	3.511111921	0.00892978	OK	0.00044192
CNTLN	1.46E-08	5.546119333	0.01418722	OK	3.43E-08
ADAMTSL1	3.02E-09	5.815602919	0.01482023	OK	7.22E-09
RRAGA	0	47.02993601	0.12088079	OK	0
HAUS6	1.86E-10	6.264944355	0.01602939	OK	4.60E-10
PLIN2	0	234.931947	0.60414739	OK	0
DENND4C	3.73E-20	9.120617019	0.02337556	OK	1.10E-19
RPS6	0	170.2208107	0.43766011	OK	0
MLLT3	1.83E-10	6.268202699	0.01603134	OK	4.51E-10
HACD4	0	50.45074658	0.12966823	OK	0
MTAP	2.32E-11	6.581866272	0.01685049	OK	5.86E-11
CDKN2A	2.48E-47	14.40292927	0.0369275	OK	9.22E-47
TUSC1	0	50.29345185	0.12925392	OK	0
CAAP1	7.56E-07	4.809588693	0.0122919	OK	1.67E-06
PLAA	7.75E-07	4.804553522	0.01227546	OK	1.71E-06
IFT74	9.38E-17	8.229700973	0.02108615	OK	2.62E-16
TEK	1.52E-190	29.42001228	0.07477246	OK	9.26E-190
MOB3B	5.97E-08	5.294462323	0.01353096	OK	1.37E-07
C9orf72	0	38.84528247	0.09981711	OK	0
AL360014.1	1.74E-15	7.872493854	0.01891045	OK	4.74E-15
ACO1	3.38E-13	7.184252757	0.01839362	OK	8.87E-13
DDX58	5.59E-06	4.392854413	0.01121787	OK	1.19E-05
TOPORS	5.16E-09	5.725310594	0.01464936	OK	1.23E-08
SMIM27	6.40E-08	5.28159176	0.01350407	OK	1.47E-07
NDUFB6	0	46.04393509	0.11834708	OK	0
APTX	2.07E-06	4.603934228	0.01176241	OK	4.51E-06
DNAJA1	0	108.1004313	0.27795269	OK	0
SMU1	3.43E-86	19.64125857	0.05043898	OK	1.55E-85
B4GALT1	0	69.15793734	0.17779005	OK	0
B4GALT1-AS1	2.11E-06	4.600003379	0.01173801	OK	4.60E-06
BAG1	0	50.61778799	0.13011137	OK	0
CHMP5	0	51.49683703	0.13237264	OK	0
AQP7	4.25E-32	11.73430802	0.02976806	OK	1.42E-31
AQP3	9.10E-12	6.719839572	0.01717589	OK	2.32E-11
AL356489.3	1.19E-37	12.77095435	0.03220261	OK	4.13E-37
UBE2R2	0	60.55000682	0.15565518	OK	0
UBAP2	4.53E-05	3.914296196	0.0099893	OK	9.29E-05
DCAF12	3.20E-36	12.51232654	0.03209165	OK	1.10E-35
UBAP1	6.52E-39	12.99514746	0.03333883	OK	2.28E-38
KIF24	1.83E-10	6.268246942	0.01567943	OK	4.51E-10
NUDT2	4.31E-41	13.37363052	0.03431552	OK	1.54E-40
ENHO	0	59.55270933	0.15258186	OK	0
CNTFR	7.43E-08	5.254373677	0.0128561	OK	1.70E-07
RPP25L	5.37E-13	7.120615339	0.01823436	OK	1.40E-12
DCTN3	0	57.8673396	0.14875659	OK	0
SIGMAR1	1.26E-44	13.9655971	0.03583132	OK	4.61E-44
GALT	6.22E-08	5.286792772	0.01351829	OK	1.43E-07
IL11RA	0	66.73750748	0.17151421	OK	0
AL162231.1	1.34E-20	9.231369272	0.0236358	OK	3.96E-20
CCL19	6.36E-205	30.52348328	0.07822821	OK	3.99E-204

CCL21	4.80E-125	23.75565556	0.06075949	OK	2.47E-124
DNAJB5-DT	2.32E-08	5.464751978	0.01276252	OK	5.40E-08
DNAJB5	1.03E-11	6.701397141	0.01714243	OK	2.63E-11
VCP	3.57E-88	19.87179961	0.05103374	OK	1.62E-87
FANCG	9.62E-10	6.004173527	0.01533116	OK	2.33E-09
PIGO	0.00010505	3.706561377	0.0094499	OK	0.00021179
STOML2	1.13E-236	32.82926682	0.08435787	OK	7.47E-236
FAM214B	3.78E-35	12.31450207	0.03158543	OK	1.29E-34
UNC13B	1.14E-06	4.726007892	0.01205654	OK	2.51E-06
RUSC2	2.48E-09	5.848206233	0.01494671	OK	5.95E-09
TESK1	1.01E-10	6.35974947	0.01627878	OK	2.51E-10
CD72	0	90.67842984	0.23310527	OK	0
SIT1	1.46E-43	13.79004895	0.03529082	OK	5.30E-43
CCDC107	0	57.54464197	0.14792653	OK	0
ARHGEF39	1.99E-47	14.4182197	0.03665033	OK	7.39E-47
TPM2	0	325.229473	0.83639805	OK	0
TLN1	0	101.5494504	0.26109961	OK	0
CREB3	8.00E-49	14.63832087	0.0375711	OK	3.00E-48
NPR2	1.63E-05	4.15517009	0.01056922	OK	3.41E-05
HINT2	2.32E-215	31.30017708	0.08042454	OK	1.48E-214
TMEM8B	5.18E-28	10.90973139	0.0279474	OK	1.66E-27
FAM221B	1.76E-05	4.13692331	0.00952718	OK	3.68E-05
HRCT1	0	75.29026253	0.19348486	OK	0
RECK	6.33E-13	7.097871759	0.01816939	OK	1.65E-12
GLIPR2	0	139.4407988	0.3585502	OK	0
CLTA	0	117.6922947	0.30261986	OK	0
MELK	0	109.0110492	0.27793713	OK	0
EBLN3P	1.84E-09	5.898292693	0.01509335	OK	4.42E-09
ZCCHC7	1.31E-11	6.665936666	0.01706685	OK	3.34E-11
GRHPR	2.45E-294	36.64814624	0.0941801	OK	1.76E-293
POLR1E	3.66E-61	16.45828538	0.04223483	OK	1.47E-60
TOMM5	1.56E-188	29.26247501	0.07517706	OK	9.43E-188
EXOSC3	2.32E-44	13.92216791	0.03572462	OK	8.47E-44
SLC25A51	1.46E-09	5.935477361	0.01517575	OK	3.53E-09
SHB	2.57E-10	6.214591231	0.01589604	OK	6.33E-10
ALDH1B1	0	62.28127324	0.1600508	OK	0
IGFBPL1	2.71E-05	4.036701246	0.01023378	OK	5.62E-05
FAM95B1	5.94E-07	4.857644794	0.01176823	OK	1.32E-06
BX255923.1	1.10E-172	27.9892536	0.05749939	OK	6.42E-172
CNTNAP3B	6.99E-41	13.33767166	0.03411848	OK	2.49E-40
FAM27C	5.35E-05	3.87426983	0.00987163	OK	0.0001093
LINC01410	3.89E-08	5.372205745	0.01370805	OK	9.00E-08
ANKRD20A3	1.01E-08	5.609852021	0.01333233	OK	2.39E-08
ZNF658	0.00013936	3.634322373	0.00924637	OK	0.00027921
PGM5	3.91E-292	36.50956586	0.09377617	OK	2.80E-291
PGM5-AS1	9.53E-12	6.713118363	0.01710291	OK	2.43E-11
PIP5K1B	5.38E-96	20.7562846	0.05325123	OK	2.52E-95
FAM122A	3.48E-68	17.40968973	0.04469008	OK	1.44E-67
FXN	1.01E-12	7.033181151	0.01800688	OK	2.63E-12
FAM189A2	1.17E-15	7.922127321	0.02012327	OK	3.20E-15
MAMDC2	0	40.28256963	0.10342419	OK	0
SMC5	1.81E-06	4.631839092	0.01183735	OK	3.95E-06
KLF9	1.48E-136	24.84452127	0.0638198	OK	7.92E-136

CEMIP2	2.74E-254	34.04122786	0.08745077	OK	1.86E-253
ABHD17B	6.40E-05	3.830242804	0.0097664	OK	0.00013039
C9orf85	3.00E-09	5.816567456	0.01488103	OK	7.18E-09
LINC01504	4.37E-28	10.92515496	0.02798345	OK	1.40E-27
ZFAND5	0	58.38773439	0.15009466	OK	0
ALDH1A1	0	40.11856081	0.10307919	OK	0
ANXA1	0	143.5004274	0.36897852	OK	0
RORB	1.24E-40	13.29468024	0.03391861	OK	4.41E-40
C9orf40	3.70E-18	8.60862973	0.02204737	OK	1.05E-17
NMRK1	3.54E-51	15.00244187	0.0385056	OK	1.34E-50
OSTF1	0	151.4970774	0.38956735	OK	0
RFK	2.19E-38	12.90226797	0.03310459	OK	7.63E-38
GCNT1	1.45E-105	21.7897421	0.05591495	OK	7.04E-105
PRUNE2	0	67.280686	0.17293012	OK	0
VPS13A	5.51E-59	16.1518582	0.04145221	OK	2.19E-58
GNAQ	4.44E-57	15.87888497	0.04076121	OK	1.74E-56
CEP78	6.77E-13	7.088683158	0.01813942	OK	1.77E-12
TLE4	2.08E-46	14.25535445	0.03658592	OK	7.67E-46
TLE1	3.81E-64	16.86885675	0.04328503	OK	1.55E-63
FRMD3	3.59E-26	10.51752407	0.0269323	OK	1.13E-25
IDNK	3.08E-17	8.362005347	0.02142403	OK	8.66E-17
UBQLN1	1.34E-13	7.309274113	0.01872171	OK	3.55E-13
AL354920.1	0.00022206	3.512378158	0.00888599	OK	0.0004399
GKAP1	5.29E-28	10.90773374	0.02796684	OK	1.70E-27
HNRNPK	0	68.72642222	0.17668518	OK	0
RMI1	1.92E-16	8.143694195	0.02084426	OK	5.32E-16
SLC28A3	2.02E-64	16.90632411	0.0430005	OK	8.23E-64
NTRK2	0	113.5369912	0.29176371	OK	0
AGTPBP1	7.77E-83	19.24504854	0.04940847	OK	3.45E-82
GOLM1	7.52E-24	10.00125301	0.02560588	OK	2.32E-23
C9orf153	1.11E-07	5.179412372	0.01167399	OK	2.54E-07
ISCA1	2.40E-77	18.57835995	0.04770605	OK	1.04E-76
TUT7	2.28E-286	36.14441813	0.09287698	OK	1.61E-285
GAS1	3.95E-215	31.28318115	0.08023454	OK	2.53E-214
DAPK1	0	43.22292666	0.11106081	OK	0
CTSL	0	218.3989136	0.56162866	OK	0
SPIN1	4.69E-43	13.70578131	0.03517051	OK	1.69E-42
NXNL2	5.36E-09	5.718792634	0.01453099	OK	1.27E-08
C9orf47	0.00043314	3.330702834	0.00846164	OK	0.0008443
S1PR3	7.48E-94	20.51771325	0.05264832	OK	3.48E-93
SHC3	8.96E-05	3.746762677	0.00937532	OK	0.0001812
CKS2	0	104.9436597	0.26980572	OK	0
SECISBP2	1.46E-06	4.676027278	0.01195086	OK	3.20E-06
SEMA4D	3.15E-33	11.95249705	0.03064212	OK	1.06E-32
GADD45G	3.01E-39	13.05420103	0.03349216	OK	1.06E-38
AL606807.1	4.25E-05	3.930056314	0.00952303	OK	8.72E-05
SYK	0	93.48507399	0.24034166	OK	0
LINC00484	0.00019834	3.542281635	0.0089525	OK	0.00039423
AUH	2.05E-05	4.101460287	0.01046754	OK	4.28E-05
NFIL3	0	79.70246411	0.20490582	OK	0
SPTLC1	7.04E-18	8.534474751	0.02187314	OK	2.00E-17
CENPP	5.58E-16	8.013277988	0.0205082	OK	1.54E-15
OGN	0	266.9510555	0.68648398	OK	0

OMD	0	152.4200529	0.39185585	OK	0
ASPN	0	145.8798592	0.37505054	OK	0
ECM2	0	74.22884879	0.19078129	OK	0
BICD2	1.14E-08	5.589964275	0.01429963	OK	2.67E-08
FGD3	7.82E-29	11.08024985	0.02839212	OK	2.53E-28
SUSD3	3.40E-109	22.16931803	0.05691447	OK	1.67E-108
CARD19	0	50.58476754	0.13002112	OK	0
NINJ1	0	138.5590365	0.35629103	OK	0
FAM120AOS	7.66E-73	18.01333372	0.04625166	OK	3.24E-72
FAM120A	2.77E-40	13.2346458	0.03396049	OK	9.80E-40
PHF2	0.00018106	3.566254062	0.00909275	OK	0.00036053
BARX1	3.86E-31	11.54626103	0.02953635	OK	1.27E-30
MFSD14B	2.78E-10	6.202500636	0.01587276	OK	6.83E-10
FBP1	0	236.00875	0.606875	OK	0
C9orf3	0	165.3056925	0.42505663	OK	0
PTCH1	5.39E-05	3.872108932	0.00984074	OK	0.00011027
LINC00092	6.38E-05	3.831032426	0.00971681	OK	0.00013001
SLC35D2	7.23E-82	19.12908673	0.04911575	OK	3.20E-81
ZNF367	5.99E-09	5.70015289	0.01449981	OK	1.42E-08
HABP4	0	61.51702622	0.15810437	OK	0
CDC14B	2.09E-93	20.4676208	0.05252547	OK	9.73E-93
AAED1	2.97E-275	35.4297744	0.09104618	OK	2.08E-274
CTSV	2.04E-27	10.78435978	0.02755632	OK	6.51E-27
TMOD1	2.59E-191	29.48006174	0.07570858	OK	1.58E-190
XPA	8.05E-78	18.63699414	0.04785597	OK	3.50E-77
TRMO	8.65E-16	7.959281979	0.02038222	OK	2.37E-15
ANP32B	0	76.09716033	0.19564294	OK	0
NANS	0	105.998116	0.27254441	OK	0
TRIM14	0	41.41538725	0.10640634	OK	0
TBC1D2	2.38E-108	22.08142576	0.05667426	OK	1.17E-107
GALNT12	2.38E-06	4.575027558	0.01164899	OK	5.16E-06
COL15A1	1.35E-293	36.60164115	0.09392515	OK	9.64E-293
TGFBR1	3.12E-67	17.28364798	0.04437068	OK	1.29E-66
ALG2	3.76E-188	29.23236203	0.07509832	OK	2.27E-187
SEC61B	0	129.0789958	0.33190262	OK	0
NR4A3	0	115.2216658	0.29624177	OK	0
STX17-AS1	0.0004503	3.319868768	0.0084267	OK	0.00087621
STX17	3.45E-23	9.849352491	0.02525102	OK	1.06E-22
AL358937.1	0.0001862	3.558912877	0.008492	OK	0.00037059
ERP44	0	61.95410969	0.15926634	OK	0
MSANTD3	7.74E-63	16.69007739	0.04283698	OK	3.13E-62
MRPL50	1.97E-66	17.17705188	0.04409828	OK	8.12E-66
TMEM246	6.38E-53	15.26667072	0.03913007	OK	2.44E-52
RNF20	1.54E-19	8.965517245	0.02297927	OK	4.49E-19
SMC2	1.29E-278	35.6474745	0.09158933	OK	9.05E-278
NIPSNAP3A	5.82E-33	11.90143957	0.03053056	OK	1.95E-32
NIPSNAP3B	5.01E-06	4.416798821	0.01125911	OK	1.07E-05
ABCA1	0	121.3599142	0.31204222	OK	0
SLC44A1	1.45E-07	5.129940861	0.01311689	OK	3.29E-07
FSD1L	0.00017739	3.571624844	0.0090937	OK	0.00035351
TMEM38B	9.91E-209	30.8090472	0.07914609	OK	6.27E-208
ZNF462	1.25E-13	7.31907456	0.01871704	OK	3.30E-13
RAD23B	3.47E-77	18.55865027	0.04765448	OK	1.50E-76

KLF4	0	156.2839039	0.4018729	OK	0
CTNNAL1	0	40.18909043	0.10325449	OK	0
TMEM245	1.35E-12	6.992592117	0.01790343	OK	3.50E-12
PTPN3	1.03E-11	6.70128327	0.01711966	OK	2.63E-11
PALM2-AKAP2	5.05E-07	4.889740716	0.01238817	OK	1.12E-06
AKAP2	0.00027429	3.455839149	0.00877773	OK	0.00054051
TXN	0	124.1868696	0.31932301	OK	0
SVEP1	2.00E-100	21.24088021	0.05430427	OK	9.55E-100
MUSK	1.98E-05	4.109319282	0.010339	OK	4.14E-05
LPAR1	1.65E-40	13.27338291	0.03402868	OK	5.86E-40
AL162414.1	2.86E-16	8.095220695	0.02053999	OK	7.91E-16
ECPAS	0.00036296	3.37959899	0.008615	OK	0.00071053
ZNF483	4.01E-18	8.59939711	0.02200571	OK	1.14E-17
PTGR1	7.04E-78	18.64417234	0.04787067	OK	3.06E-77
DNAJC25	1.61E-06	4.656247951	0.01189381	OK	3.52E-06
DNAJC25-GNG10	1.31E-05	4.203449869	0.01071099	OK	2.77E-05
GNG10	8.64E-16	7.959406515	0.02038251	OK	2.37E-15
UGCG	0	75.49718174	0.19409079	OK	0
SUSD1	1.27E-90	20.15275496	0.05172432	OK	5.84E-90
PTBP3	0	42.21760592	0.10849823	OK	0
HSDL2	5.35E-149	25.97043088	0.06671321	OK	2.94E-148
KIAA1958	1.88E-07	5.080400351	0.01295156	OK	4.26E-07
INIP	4.65E-36	12.48249791	0.03202382	OK	1.60E-35
SNX30	6.50E-45	14.01289587	0.03594312	OK	2.37E-44
SLC46A2	1.77E-16	8.153001921	0.02080694	OK	4.93E-16
SLC31A2	0	122.7745255	0.3156799	OK	0
FKBP15	0	47.68778164	0.12255814	OK	0
SLC31A1	0	44.04680978	0.11319481	OK	0
CDC26	1.11E-181	28.71892364	0.07378605	OK	6.59E-181
PRPF4	1.96E-27	10.78806087	0.02766398	OK	6.26E-27
HDHD3	4.67E-09	5.742119017	0.0146823	OK	1.11E-08
ALAD	2.68E-05	4.039781513	0.01031044	OK	5.55E-05
POLE3	4.20E-116	22.87418549	0.05875262	OK	2.11E-115
RGS3	0	68.9271114	0.17715766	OK	0
ZNF618	1.07E-14	7.642429357	0.01957099	OK	2.87E-14
COL27A1	1.11E-43	13.80964662	0.03539773	OK	4.04E-43
AKNA	3.78E-31	11.54785262	0.02961396	OK	1.25E-30
WHRN	4.10E-06	4.459815263	0.01135845	OK	8.81E-06
ATP6V1G1	0	141.54123	0.36395365	OK	0
TMEM268	1.06E-10	6.352201777	0.01623915	OK	2.64E-10
TNFSF15	2.51E-50	14.8719456	0.0381348	OK	9.47E-50
TNFSF8	4.08E-112	22.4700121	0.05767849	OK	2.02E-111
TNC	7.02E-126	23.83633521	0.06115136	OK	3.63E-125
PAPPA	1.15E-09	5.975369073	0.0152202	OK	2.78E-09
ASTN2	8.27E-11	6.390534907	0.01630726	OK	2.06E-10
TRIM32	1.55E-06	4.664370905	0.01188856	OK	3.38E-06
TLR4	0	46.03407997	0.11830226	OK	0
BRINP1	4.89E-11	6.470281573	0.01641482	OK	1.22E-10
CDK5RAP2	2.48E-62	16.62034511	0.04266376	OK	1.00E-61
PSMD5	8.89E-06	4.291129532	0.0109552	OK	1.88E-05
PHF19	4.59E-128	24.04608507	0.0617539	OK	2.39E-127
TRAF1	3.35E-33	11.94743485	0.03062603	OK	1.13E-32
CNTRL	6.16E-74	18.15226421	0.04660401	OK	2.62E-73

RAB14	0	37.5536572	0.09651121	OK	0
GSN	0	130.5772311	0.33575493	OK	0
STOM	0	46.92426533	0.12060863	OK	0
DAB2IP	9.72E-32	11.66415584	0.02987386	OK	3.22E-31
NDUFA8	2.52E-183	28.85016049	0.07412397	OK	1.51E-182
LHX6	1.77E-30	11.41468179	0.02641038	OK	5.78E-30
RBM18	8.86E-25	10.21095134	0.02618181	OK	2.76E-24
MRRF	4.59E-10	6.122971596	0.01566389	OK	1.12E-09
PTGS1	0	49.03607709	0.12600941	OK	0
PDCL	2.11E-23	9.898840401	0.02538063	OK	6.47E-23
RABGAP1	1.77E-255	34.12148731	0.08767108	OK	1.20E-254
DENND1A	1.62E-12	6.966667025	0.01782941	OK	4.20E-12
NEK6	0	41.65680191	0.10703981	OK	0
PSMB7	0	43.16105358	0.11093254	OK	0
NR6A1	3.84E-09	5.775380667	0.01472978	OK	9.15E-09
OLFML2A	9.71E-205	30.50961367	0.07803945	OK	6.09E-204
RPL35	0	152.3677017	0.39175868	OK	0
ARPC5L	0	43.17213195	0.11095757	OK	0
PPP6C	1.24E-30	11.44510199	0.02935831	OK	4.08E-30
RABEPK	1.94E-11	6.608580766	0.01691596	OK	4.90E-11
HSPA5	0	81.51029376	0.20956421	OK	0
GAPVD1	8.29E-18	8.515546734	0.02182259	OK	2.35E-17
MAPKAP1	2.51E-47	14.40198787	0.03696262	OK	9.34E-47
PBX3	1.90E-15	7.861276932	0.02013705	OK	5.18E-15
MVB12B	2.06E-109	22.19179946	0.05694865	OK	1.02E-108
ZBTB43	1.08E-60	16.39259047	0.04207306	OK	4.33E-60
ANGPTL2	1.91E-72	17.96270796	0.04603309	OK	8.06E-72
SLC2A8	2.16E-54	15.48584365	0.03973952	OK	8.34E-54
RPL12	0	181.0761464	0.46553916	OK	0
FAM129B	3.90E-129	24.14823904	0.06200714	OK	2.04E-128
STXBP1	1.10E-10	6.346555664	0.01621929	OK	2.73E-10
PTRH1	2.44E-30	11.3865456	0.02920336	OK	7.98E-30
TOR2A	5.61E-293	36.56268535	0.09394288	OK	4.01E-292
SH2D3C	2.36E-36	12.5364372	0.03207624	OK	8.11E-36
CDK9	3.80E-05	3.956448721	0.01009935	OK	7.83E-05
FPGS	5.08E-08	5.323967488	0.01361353	OK	1.17E-07
ENG	4.77E-285	36.06021301	0.09266587	OK	3.38E-284
AK1	3.70E-76	18.4310589	0.04727847	OK	1.59E-75
ST6GALNAC4	6.11E-23	9.791797178	0.02510072	OK	1.87E-22
DPM2	1.36E-73	18.1087373	0.04649594	OK	5.77E-73
FAM102A	6.13E-17	8.280536088	0.02118942	OK	1.71E-16
SLC25A25	2.32E-17	8.395411992	0.02150869	OK	6.53E-17
PTGES2	4.45E-70	17.65759494	0.04533547	OK	1.86E-69
C9orf16	0	67.59171331	0.1737677	OK	0
CIZ1	7.76E-05	3.782655093	0.00964936	OK	0.00015743
DNM1	3.57E-125	23.76816153	0.06081307	OK	1.84E-124
GOLGA2	1.19E-20	9.243379593	0.02369746	OK	3.54E-20
SWI5	1.38E-53	15.36629355	0.0394399	OK	5.29E-53
TRUB2	9.66E-18	8.497811732	0.02177408	OK	2.74E-17
COQ4	1.85E-16	8.147975255	0.02087805	OK	5.14E-16
SLC27A4	3.43E-05	3.980749334	0.01014646	OK	7.08E-05
URM1	6.45E-59	16.14215697	0.04143827	OK	2.56E-58
CERCAM	2.05E-215	31.30415899	0.08039631	OK	1.31E-214

SPTAN1	7.59E-43	13.67072455	0.03508194	OK	2.74E-42
WDR34	3.18E-102	21.43465479	0.05500905	OK	1.52E-101
SET	0	91.82100557	0.23608151	OK	0
PKN3	6.41E-08	5.28130864	0.01330984	OK	1.47E-07
ZDHHC12	0	56.08214621	0.14415274	OK	0
AL441992.1	8.36E-24	9.990793836	0.02559058	OK	2.58E-23
ENDOG	3.27E-21	9.380798292	0.02404639	OK	9.77E-21
KYAT1	7.44E-05	3.792929364	0.00964334	OK	0.00015117
LRRC8A	6.28E-13	7.099104492	0.01817097	OK	1.64E-12
PHYHD1	1.44E-174	28.14350607	0.07225101	OK	8.45E-174
NUP188	0.00026703	3.463061977	0.00882108	OK	0.00052668
SH3GLB2	4.28E-112	22.46789881	0.05769998	OK	2.12E-111
CRAT	6.31E-17	8.277140706	0.02120242	OK	1.76E-16
PTPA	2.73E-13	7.213366654	0.01847189	OK	7.17E-13
IER5L	1.84E-122	23.50447479	0.0603741	OK	9.38E-122
AL161785.1	8.42E-16	7.962664411	0.02034794	OK	2.31E-15
LINC01503	8.77E-54	15.3955005	0.03950168	OK	3.37E-53
NTMT1	4.76E-86	19.62468497	0.05039334	OK	2.14E-85
PRRX2	0	80.97610973	0.20813005	OK	0
TOR1A	3.99E-63	16.72956229	0.04294726	OK	1.62E-62
C9orf78	1.78E-272	35.24889983	0.09058313	OK	1.24E-271
FNBP1	4.92E-238	32.92438691	0.08460189	OK	3.27E-237
NCS1	0	76.45385442	0.19651717	OK	0
ASS1	9.86E-198	29.9767611	0.07695641	OK	6.09E-197
FUBP3	3.12E-05	4.003542286	0.01021951	OK	6.45E-05
AIF1L	3.48E-163	27.19751246	0.06978218	OK	1.98E-162
NUP214	0	59.26266327	0.15233526	OK	0
FAM78A	3.44E-87	19.75777146	0.05070431	OK	1.56E-86
UCK1	1.23E-12	7.005498441	0.01793409	OK	3.19E-12
RAPGEF1	7.69E-285	36.0469874	0.09262082	OK	5.44E-284
MED27	5.69E-17	8.28939303	0.02124048	OK	1.59E-16
SETX	5.79E-18	8.557071197	0.02193159	OK	1.65E-17
TTF1	1.88E-08	5.501965572	0.01407366	OK	4.39E-08
SPACA9	1.38E-12	6.989006833	0.01787769	OK	3.59E-12
RALGDS	2.72E-150	26.08461731	0.06699375	OK	1.51E-149
GBGT1	5.37E-183	28.82397517	0.07402853	OK	3.21E-182
ABO	6.14E-11	6.435738745	0.01613996	OK	1.53E-10
SURF6	6.65E-10	6.063641951	0.01551643	OK	1.62E-09
RPL7A	0	200.7996645	0.51626847	OK	0
SURF1	4.09E-169	27.69432499	0.0711522	OK	2.37E-168
SURF2	2.85E-33	11.96081035	0.0306836	OK	9.59E-33
SURF4	9.11E-81	18.99652611	0.04877923	OK	4.01E-80
REXO4	1.92E-08	5.497972732	0.01406096	OK	4.49E-08
SLC2A6	1.52E-42	13.61993632	0.03490814	OK	5.49E-42
BRD3OS	3.96E-08	5.369124772	0.01371084	OK	9.15E-08
BRD3	1.13E-11	6.688657031	0.01712409	OK	2.86E-11
WDR5	6.71E-05	3.818629102	0.00973884	OK	0.00013651
RXRA	9.03E-46	14.15236298	0.03631822	OK	3.32E-45
COL5A1	4.73E-108	22.05040158	0.05656836	OK	2.32E-107
FCN1	0	173.7250469	0.44664553	OK	0
OLFM1	1.23E-46	14.29203975	0.03661688	OK	4.54E-46
MRPS2	5.94E-70	17.64122545	0.04528952	OK	2.48E-69
CAMSAP1	4.58E-33	11.92142709	0.03057211	OK	1.54E-32

UBAC1	0	48.98045353	0.12589601	OK	0
NACC2	0.00044011	3.326254261	0.00847343	OK	0.00085713
TMEM250	2.63E-09	5.838446924	0.01493568	OK	6.30E-09
CARD9	1.44E-208	30.79698035	0.0790918	OK	9.09E-208
ENTR1	4.18E-12	6.832069089	0.01749269	OK	1.07E-11
PMPCA	2.99E-06	4.526945903	0.01156559	OK	6.47E-06
NOTCH1	5.59E-06	4.392934326	0.01121172	OK	1.19E-05
NALT1	1.26E-06	4.706275462	0.01194444	OK	2.76E-06
EGFL7	0	58.60017608	0.15057009	OK	0
AGPAT2	0	60.47455367	0.15545603	OK	0
FAM69B	0	55.35162742	0.13899881	OK	0
SNHG7	5.72E-180	28.58142436	0.07343321	OK	3.39E-179
LCN6	1.55E-11	6.641976861	0.01570238	OK	3.92E-11
TMEM141	3.84E-297	36.82378953	0.09463055	OK	2.77E-296
RABL6	3.77E-41	13.38350761	0.03434326	OK	1.35E-40
PHPT1	0	75.82156116	0.19493411	OK	0
EDF1	0	92.27101299	0.23723503	OK	0
FBXW5	1.73E-106	21.88699915	0.0562157	OK	8.41E-106
PTGDS	4.37E-84	19.39362158	0.04961857	OK	1.95E-83
PAXX	8.66E-179	28.48630518	0.07318618	OK	5.12E-178
CLIC3	8.14E-106	21.81623678	0.05596855	OK	3.95E-105
FUT7	0	42.4986369	0.108953	OK	0
NPDC1	0	51.72890525	0.13294095	OK	0
SAPCD2	0	40.47202278	0.10291261	OK	0
UAP1L1	3.17E-50	14.85613071	0.03809577	OK	1.20E-49
MAN1B1	4.17E-08	5.359738267	0.01370679	OK	9.63E-08
DPP7	0	101.3142458	0.26050021	OK	0
SSNA1	0	58.22636612	0.14967966	OK	0
TPRN	2.75E-06	4.54510921	0.01160354	OK	5.94E-06
TMEM203	1.96E-33	11.99195693	0.03076193	OK	6.60E-33
TUBB4B	0	57.06435895	0.1466919	OK	0
TOR4A	2.02E-46	14.25715111	0.03656866	OK	7.47E-46
NRARP	0	86.9638974	0.22350317	OK	0
NSMF	1.69E-15	7.876331766	0.02017019	OK	4.60E-15
MRPL41	0	52.82174513	0.13578005	OK	0
ZMYND19	5.81E-05	3.853885496	0.00982616	OK	0.0001186
ARRDC1	2.35E-202	30.32935882	0.07791535	OK	1.47E-201
EHMT1	9.80E-05	3.724187408	0.00950245	OK	0.00019777
BET1L	4.18E-06	4.455722585	0.01138114	OK	8.97E-06
RIC8A	2.24E-30	11.39384309	0.02922576	OK	7.34E-30
PSMD13	3.54E-206	30.61787889	0.0786709	OK	2.22E-205
AC136475.3	2.42E-12	6.910111623	0.01686346	OK	6.24E-12
IFITM2	0	127.7504659	0.32848939	OK	0
IFITM1	1.01E-219	31.6191772	0.08120625	OK	6.49E-219
AC136475.1	4.44E-07	4.914828717	0.01251471	OK	9.91E-07
IFITM3	0	183.4407377	0.47171038	OK	0
SIGIRR	8.20E-252	33.8734454	0.08704197	OK	5.55E-251
PTDSS2	1.43E-05	4.184384016	0.01067906	OK	3.00E-05
RNH1	0	103.7836903	0.26684963	OK	0
HRAS	6.30E-161	27.00588398	0.06937633	OK	3.58E-160
RASSF7	5.44E-53	15.27702147	0.03918264	OK	2.08E-52
MIR210HG	8.53E-81	18.99997547	0.04871827	OK	3.76E-80
IRF7	0	61.5122169	0.15811445	OK	0

SCT	8.76E-05	3.752327701	0.00897588	OK	0.00017732
DEAF1	8.96E-21	9.27408556	0.02376848	OK	2.66E-20
EPS8L2	1.61E-12	6.967439987	0.01781509	OK	4.18E-12
TMEM80	9.10E-49	14.62952352	0.03754297	OK	3.41E-48
TALDO1	0	123.5107438	0.31758796	OK	0
RPLP2	0	177.4546718	0.456239	OK	0
PNPLA2	4.39E-130	24.23830452	0.06225871	OK	2.30E-129
CD151	0	205.8329805	0.5293137	OK	0
POLR2L	0	75.71397747	0.19465736	OK	0
TSPAN4	0	88.59759966	0.22778806	OK	0
CHID1	3.36E-174	28.11341392	0.07222696	OK	1.97E-173
AP2A2	0	49.4884136	0.12718803	OK	0
AC139749.1	5.66E-07	4.867078188	0.01148285	OK	1.26E-06
TOLLIP	2.32E-169	27.71470162	0.0711974	OK	1.35E-168
BRSK2	5.53E-26	10.47669381	0.02672207	OK	1.74E-25
MOB2	2.40E-270	35.10964007	0.09021596	OK	1.67E-269
DUSP8	2.34E-90	20.12260759	0.05164763	OK	1.07E-89
AC068580.4	5.54E-05	3.865778049	0.00982801	OK	0.00011308
CTSD	0	219.8144453	0.56526636	OK	0
TNNI2	0	55.76825567	0.14330987	OK	0
LSP1	0	231.436001	0.59515568	OK	0
TNNT3	1.41E-06	4.68415326	0.01127379	OK	3.08E-06
MRPL23	0	43.8064205	0.11259062	OK	0
IGF2.1	1.44E-13	7.300133863	0.01761058	OK	3.80E-13
ASCL2	0	38.76009559	0.09956508	OK	0
C11orf21	3.47E-07	4.963045029	0.01257562	OK	7.78E-07
TSPAN32	4.25E-27	10.71666293	0.02740109	OK	1.35E-26
CD81	0	177.647154	0.45679662	OK	0
TSSC4	7.80E-69	17.49513193	0.04491879	OK	3.24E-68
KCNQ1	0	52.46962746	0.13482957	OK	0
KCNQ1OT1	3.26E-45	14.06178017	0.0360772	OK	1.19E-44
CDKN1C	0	47.12927525	0.12113157	OK	0
SLC22A18AS	2.85E-116	22.89112315	0.05875785	OK	1.43E-115
SLC22A18	2.47E-135	24.73125633	0.06350918	OK	1.31E-134
PHLDA2	0	64.51674579	0.16584016	OK	0
NAP1L4	2.10E-74	18.21121873	0.04676165	OK	8.96E-74
CARS	7.26E-08	5.25861517	0.01344564	OK	1.66E-07
OSBPL5	4.69E-22	9.58352041	0.02454293	OK	1.42E-21
NUP98	5.93E-12	6.781985092	0.01736329	OK	1.52E-11
PGAP2	8.26E-06	4.307264361	0.0109937	OK	1.75E-05
RHOG	0	151.9600679	0.39075834	OK	0
RRM1	8.72E-84	19.35803569	0.0496907	OK	3.89E-83
OR52K1	1.44E-05	4.182035761	0.00922441	OK	3.03E-05
OR51E1	0	94.91582602	0.24289492	OK	0
TRIM22	3.12E-75	18.31537306	0.04702591	OK	1.34E-74
CAVIN3	0	254.5982114	0.65472857	OK	0
APBB1	2.54E-38	12.89052989	0.03304421	OK	8.88E-38
ARFIP2	0.00016425	3.591730622	0.00916017	OK	0.00032791
TIMM10B	5.04E-29	11.11951157	0.02851743	OK	1.63E-28
RRP8	9.09E-13	7.047737874	0.01804547	OK	2.37E-12
ILK	0	85.40597005	0.21958305	OK	0
TAF10	0	93.81976009	0.24122466	OK	0
TPP1	0	88.15595382	0.22664807	OK	0

AC091564.6	5.06E-10	6.107409263	0.01542738	OK	1.24E-09
MRPL17	0	37.702754	0.0968886	OK	0
OLFML1	1.75E-168	27.64188467	0.07094752	OK	1.01E-167
CYB5R2	1.08E-14	7.641228225	0.01951582	OK	2.90E-14
EIF3F	0	98.55285433	0.25339695	OK	0
RIC3	4.57E-41	13.36930846	0.03427251	OK	1.63E-40
RPL27A	0	119.2104431	0.30651194	OK	0
ST5	3.30E-104	21.64623583	0.05556785	OK	1.59E-103
AKIP1	5.13E-65	16.98690263	0.04360864	OK	2.10E-64
TMEM9B	0	68.14290555	0.17517834	OK	0
NRIP3	0	89.12046634	0.22905218	OK	0
DENND5A	1.04E-12	7.029225914	0.01799748	OK	2.70E-12
TMEM41B	1.90E-07	5.078770376	0.01298326	OK	4.30E-07
IPO7	3.99E-19	8.860251406	0.02271147	OK	1.15E-18
ZNF143	1.86E-05	4.124695651	0.01053071	OK	3.88E-05
WEE1	7.47E-51	14.95274208	0.03835922	OK	2.83E-50
SWAP70	4.64E-164	27.27136244	0.07006163	OK	2.65E-163
SBF2-AS1	1.13E-54	15.52722304	0.03982455	OK	4.38E-54
ADM	0	44.60238364	0.11461879	OK	0
AMPD3	1.56E-47	14.43496273	0.03702329	OK	5.80E-47
MTRNR2L8	0	47.05774562	0.12094761	OK	0
RNF141	5.30E-50	14.82168468	0.03804043	OK	2.00E-49
LYVE1	0	48.21345481	0.12377057	OK	0
MRVI1	0	80.87704265	0.20789853	OK	0
CTR9	1.87E-08	5.50224468	0.01407465	OK	4.38E-08
EIF4G2	0	46.11259042	0.11852449	OK	0
ZBED5	1.32E-13	7.311579273	0.01872587	OK	3.49E-13
ZBED5-AS1	7.51E-15	7.687324156	0.01968785	OK	2.03E-14
GALNT18	3.20E-97	20.89154237	0.05358374	OK	1.51E-96
USP47	2.35E-05	4.069690812	0.0103912	OK	4.89E-05
DKK3	0	200.4406525	0.51541386	OK	0
MICAL2	0	48.31623131	0.1241664	OK	0
PARVA	0	55.35683049	0.14224562	OK	0
TEAD1	8.58E-153	26.30422947	0.06751346	OK	4.77E-152
ARNTL	2.58E-12	6.901150813	0.01765447	OK	6.64E-12
BTBD10	9.20E-11	6.374129311	0.01631535	OK	2.29E-10
FAR1	5.15E-21	9.332988219	0.0239247	OK	1.53E-20
SPON1	1.90E-57	15.93188504	0.04081541	OK	7.48E-57
RRAS2	4.12E-53	15.2950582	0.03920282	OK	1.58E-52
COPB1	3.64E-139	25.08487429	0.06444064	OK	1.96E-138
PSMA1	0	60.82291852	0.15635895	OK	0
PDE3B	1.86E-55	15.64283195	0.04012551	OK	7.21E-55
INSC	1.19E-15	7.919851044	0.0200823	OK	3.25E-15
SOX6	4.08E-20	9.111115586	0.02326757	OK	1.20E-19
C11orf58	0	75.89121741	0.19511158	OK	0
PLEKHA7	8.05E-08	5.239427852	0.01335227	OK	1.84E-07
RPS13	0	198.7951859	0.51112653	OK	0
PIK3C2A	4.04E-08	5.365222049	0.01372221	OK	9.35E-08
NUCB2	0	46.32510645	0.11906798	OK	0
SERGEF	1.74E-72	17.96771985	0.04612598	OK	7.37E-72
TPH1	0	45.33601445	0.11639409	OK	0
SAAL1	5.86E-07	4.860328426	0.01241752	OK	1.30E-06
SAA2	3.56E-12	6.855034597	0.01654109	OK	9.16E-12

SAA1	6.50E-08	5.278969021	0.01291806	OK	1.49E-07
HPS5	1.05E-137	24.95076115	0.06408356	OK	5.62E-137
LDHA	0	131.4901524	0.33810246	OK	0
TSG101	2.45E-128	24.07218667	0.06183611	OK	1.27E-127
UEVLD	1.41E-05	4.187592942	0.01068637	OK	2.96E-05
SPTY2D1OS	1.02E-05	4.260087272	0.01084658	OK	2.16E-05
SPTY2D1	2.59E-57	15.91253627	0.04084595	OK	1.02E-56
TMEM86A	7.92E-223	31.84420204	0.08177339	OK	5.13E-222
E2F8	0	131.7128128	0.33418807	OK	0
NAV2	7.70E-64	16.82730333	0.04317348	OK	3.13E-63
AC009549.1	1.85E-05	4.125980856	0.01046287	OK	3.86E-05
HTATIP2	4.53E-308	37.50046594	0.09636979	OK	3.30E-307
FANCF	8.33E-07	4.790209341	0.01222852	OK	1.84E-06
SVIP	4.90E-217	31.4230611	0.08073409	OK	3.14E-216
FIBIN	0	110.9100746	0.28511246	OK	0
CCDC34	3.85E-100	21.2101649	0.05443586	OK	1.83E-99
LGR4	3.69E-10	6.157901357	0.01572012	OK	9.03E-10
LIN7C	3.73E-35	12.31567162	0.03159323	OK	1.27E-34
BDNF-AS	4.96E-06	4.4191214	0.01123241	OK	1.06E-05
BDNF	5.17E-101	21.30438563	0.05456512	OK	2.47E-100
KIF18A	1.53E-33	12.01245956	0.03064868	OK	5.15E-33
METTL15	0.00010219	3.713550669	0.00947278	OK	0.00020614
ARL14EP	1.26E-25	10.39856511	0.02666747	OK	3.95E-25
MPPED2	5.46E-70	17.64600684	0.04526636	OK	2.28E-69
AL136088.1	0.00033988	3.397616166	0.00859005	OK	0.00066641
IMMP1L	4.14E-22	9.596481412	0.02459643	OK	1.25E-21
RCN1	0	52.47054534	0.13485862	OK	0
EIF3M	0	57.30317475	0.14730607	OK	0
PRRG4	2.49E-35	12.34815106	0.03165266	OK	8.51E-35
QSER1	7.92E-06	4.316736667	0.01102442	OK	1.68E-05
CSTF3-DT	1.79E-06	4.634707113	0.01003849	OK	3.90E-06
HIPK3	6.67E-08	5.274017148	0.01348925	OK	1.53E-07
CD59	0	108.8657124	0.27992038	OK	0
FBXO3	2.14E-19	8.929540719	0.02288265	OK	6.21E-19
LMO2	0	68.54382978	0.17620614	OK	0
CAPRIN1	7.41E-56	15.70125502	0.0403053	OK	2.89E-55
CAT	0	52.40532196	0.13470676	OK	0
EHF	5.19E-41	13.35976414	0.03293562	OK	1.85E-40
APIP	1.17E-127	24.00729758	0.06166697	OK	6.07E-127
CD44	0	128.8633865	0.3313542	OK	0
PAMR1	1.91E-39	13.08864963	0.0334195	OK	6.73E-39
FJX1	3.75E-07	4.94810891	0.01258397	OK	8.39E-07
TRIM44	8.95E-13	7.050012656	0.01805689	OK	2.33E-12
COMMD9	0	50.5215551	0.12985379	OK	0
PRR5L	5.07E-06	4.414042378	0.01124818	OK	1.09E-05
C11orf74	5.62E-17	8.290932571	0.0212429	OK	1.57E-16
LRRC4C	1.61E-14	7.58912074	0.01936238	OK	4.32E-14
API5	1.38E-10	6.312200067	0.01615697	OK	3.41E-10
HSD17B12	4.37E-248	33.61938748	0.08638909	OK	2.94E-247
ALKBH3	9.37E-07	4.766652764	0.01218138	OK	2.06E-06
C11orf96	0	292.0436363	0.7510335	OK	0
CD82	0	54.9296823	0.14118484	OK	0
TSPAN18	6.76E-115	22.75259914	0.05794989	OK	3.38E-114

TP53I11	1.01E-63	16.81113612	0.04313226	OK	4.11E-63
AC103736.1	1.12E-07	5.177561224	0.0131365	OK	2.56E-07
CHST1	1.00E-42	13.65047361	0.03492776	OK	3.62E-42
AC044839.1	5.94E-20	9.070181604	0.02317735	OK	1.74E-19
SLC35C1	1.54E-17	8.443594367	0.02162426	OK	4.34E-17
MAPK8IP1	1.09E-07	5.182562605	0.01320034	OK	2.50E-07
PEX16	4.11E-23	9.831842275	0.02520808	OK	1.26E-22
CREB3L1	1.41E-08	5.552609031	0.01404794	OK	3.30E-08
DGKZ	1.56E-126	23.89920619	0.06137867	OK	8.08E-126
MDK	2.09E-108	22.08741582	0.05668263	OK	1.02E-107
ARHGAP1	1.16E-65	17.07376747	0.04382464	OK	4.77E-65
CKAP5	9.17E-24	9.981633187	0.02558664	OK	2.83E-23
C11orf49	8.49E-14	7.370654165	0.01887173	OK	2.25E-13
ARFGAP2	0.00043987	3.326408422	0.00847981	OK	0.00085674
PACSIN3	8.65E-213	31.11063311	0.07988245	OK	5.52E-212
DDB2	8.47E-24	9.989472492	0.02561138	OK	2.61E-23
ACP2	0	59.12639905	0.15196732	OK	0
NR1H3	0	51.28931479	0.13178053	OK	0
SPI1	0	278.5442145	0.71632778	OK	0
SLC39A13	7.77E-56	15.69830817	0.04028634	OK	3.02E-55
PSMC3	0	44.87666858	0.11534506	OK	0
CELF1	6.42E-20	9.061651196	0.02322788	OK	1.88E-19
NDUFS3	1.36E-283	35.96715868	0.09242923	OK	9.65E-283
PTPMT1	4.07E-54	15.44515502	0.03964412	OK	1.57E-53
KBTBD4	1.32E-07	5.146825026	0.01314896	OK	3.01E-07
FAM180B	9.15E-29	11.06619084	0.02769115	OK	2.96E-28
MTCH2	0	49.61656395	0.12752936	OK	0
PTPRJ	0	55.71940592	0.14320635	OK	0
APLNR	0	39.54381708	0.0993231	OK	0
TNKS1BP1	3.81E-12	6.845629241	0.01750697	OK	9.78E-12
SSRP1	9.84E-45	13.98338981	0.0358829	OK	3.59E-44
SLC43A3	0	54.73312655	0.14067166	OK	0
TIMM10	1.66E-264	34.72488361	0.08923288	OK	1.14E-263
UBE2L6	0	40.50100339	0.1040876	OK	0
SERPING1	0	185.8570927	0.47793742	OK	0
CLP1	2.04E-10	6.250868471	0.01599251	OK	5.03E-10
ZDHHC5	1.08E-12	7.023582029	0.01798502	OK	2.81E-12
MED19	6.64E-14	7.403311369	0.01896372	OK	1.77E-13
TMX2	3.23E-47	14.38467573	0.03691742	OK	1.20E-46
SELENOH	0	74.45405775	0.19141768	OK	0
CTNND1	2.68E-15	7.81801616	0.0200284	OK	7.30E-15
LPXN	0	60.13603031	0.15456417	OK	0
ZFP91	0.00013398	3.644448201	0.00929598	OK	0.00026864
FAM111B	0	138.6746645	0.35514213	OK	0
FAM111A	3.36E-92	20.33182389	0.05220748	OK	1.56E-91
MPEG1	0	114.2967569	0.29385504	OK	0
OSBP	6.55E-08	5.27745777	0.01349663	OK	1.51E-07
PATL1	4.05E-26	10.50598777	0.02693304	OK	1.28E-25
STX3	2.96E-38	12.87892515	0.03303754	OK	1.03E-37
MRPL16	2.85E-96	20.78678434	0.05338525	OK	1.34E-95
MS4A2	8.21E-56	15.69478593	0.03916494	OK	3.20E-55
MS4A6A	0	313.3619703	0.80586728	OK	0
MS4A4E	2.32E-18	8.662084483	0.0221009	OK	6.63E-18

MS4A4A	0	247.5572365	0.63661098	OK	0
MS4A6E	3.41E-12	6.861428664	0.01730462	OK	8.76E-12
MS4A7	0	296.4534643	0.76238201	OK	0
MS4A14	1.07E-32	11.85057043	0.03036042	OK	3.58E-32
MS4A1	2.25E-05	4.080177439	0.01034906	OK	4.68E-05
CCDC86	1.51E-12	6.976920772	0.01785725	OK	3.91E-12
PTGDR2	6.13E-14	7.413856456	0.01690986	OK	1.63E-13
ZP1	1.46E-21	9.46585367	0.02400759	OK	4.36E-21
PRPF19	1.51E-16	8.172333651	0.02093836	OK	4.21E-16
AP003721.4	5.28E-06	4.405497221	0.01073752	OK	1.13E-05
TMEM109	0	53.89724577	0.13854314	OK	0
SLC15A3	0	75.50549036	0.19409553	OK	0
CD6	1.96E-78	18.71243732	0.04792857	OK	8.55E-78
CD5	2.31E-58	16.06325727	0.04103612	OK	9.12E-58
VPS37C	3.80E-09	5.77698492	0.01477366	OK	9.07E-09
PGA4	6.09E-11	6.437118487	0.01519043	OK	1.52E-10
VWCE	6.09E-05	3.842315935	0.00976507	OK	0.00012428
DDB1	2.75E-06	4.544454936	0.01161135	OK	5.96E-06
TKFC	0.00030628	3.425993274	0.00872758	OK	0.00060182
CYB561A3	3.20E-44	13.89921456	0.03566351	OK	1.17E-43
TMEM138	2.00E-18	8.678875278	0.02224024	OK	5.72E-18
TMEM216	1.59E-14	7.590880375	0.01944115	OK	4.27E-14
SDHAF2	3.38E-35	12.32366485	0.03161709	OK	1.15E-34
LRRC10B	0	87.27587965	0.2236077	OK	0
DAGLA	0.00037789	3.368499615	0.00853065	OK	0.0007391
TMEM258	0	68.65414186	0.17650049	OK	0
FEN1	0	42.97814201	0.11035866	OK	0
FADS2	1.60E-17	8.438920216	0.02160325	OK	4.52E-17
FADS1	9.91E-25	10.19999985	0.02615178	OK	3.09E-24
FADS3	0	41.14457531	0.10573462	OK	0
RAB3IL1	7.24E-134	24.59443785	0.06314447	OK	3.84E-133
BEST1	7.76E-109	22.13211814	0.05682027	OK	3.81E-108
FTH1	0	260.928367	0.6708667	OK	0
INCENP	1.76E-37	12.74037806	0.03261983	OK	6.11E-37
ASRGL1	5.28E-163	27.1821423	0.06978715	OK	3.01E-162
AHNAK	0	38.76202702	0.09961861	OK	0
MTA2	4.53E-06	4.438333762	0.01133175	OK	9.71E-06
ROM1	4.27E-74	18.17237569	0.04661384	OK	1.82E-73
B3GAT3	6.26E-56	15.71203089	0.04033331	OK	2.44E-55
GANAB	4.44E-15	7.754484339	0.01986589	OK	1.20E-14
UQCC3	7.30E-79	18.76497638	0.04818279	OK	3.19E-78
UBXN1	0	77.22906797	0.19855407	OK	0
LRRN4CL	1.70E-63	16.78031499	0.04249286	OK	6.90E-63
TTC9C	8.66E-07	4.78235548	0.01222168	OK	1.91E-06
POLR2G	1.05E-145	25.6772955	0.0659635	OK	5.72E-145
TMEM223	1.61E-49	14.74694731	0.03784666	OK	6.06E-49
TMEM179B	0	57.04122129	0.1466317	OK	0
STX5	1.60E-05	4.158339645	0.01061892	OK	3.36E-05
WDR74	9.23E-06	4.282633627	0.01093716	OK	1.96E-05
SLC3A2	0	101.8621343	0.26190938	OK	0
LGALS12	5.44E-09	5.716384007	0.01436524	OK	1.29E-08
RARRES3	0	39.04089825	0.10032692	OK	0
PLA2G16	0	60.83419847	0.15637632	OK	0

ATL3	1.10E-164	27.32410012	0.07019597	OK	6.29E-164
RTN3	0	41.25590572	0.10603284	OK	0
MARK2	1.09E-18	8.747134969	0.02241566	OK	3.14E-18
NAA40	0.00050394	3.288320651	0.00836688	OK	0.00097721
COX8A	0	137.2276515	0.35286034	OK	0
OTUB1	2.78E-71	17.81346448	0.04573812	OK	1.17E-70
MACROD1	2.67E-170	27.79258482	0.07137791	OK	1.55E-169
STIP1	3.39E-102	21.4315656	0.05504198	OK	1.62E-101
FERMT3	0	153.4600876	0.39460603	OK	0
TRPT1	2.19E-29	11.19349432	0.02870666	OK	7.13E-29
NUDT22	7.21E-137	24.87347916	0.06389231	OK	3.86E-136
DNAJC4	2.23E-189	29.32873832	0.07535163	OK	1.35E-188
VEGFB	0	78.15203785	0.20092814	OK	0
FKBP2	0	71.24403107	0.18316045	OK	0
PPP1R14B	0	117.5728099	0.30231611	OK	0
AP001453.2	1.89E-150	26.09858748	0.06690312	OK	1.05E-149
BAD	0	41.05429857	0.10550944	OK	0
GPR137	9.47E-10	6.006594423	0.01536635	OK	2.30E-09
ESRRA	2.59E-80	18.94168358	0.04863205	OK	1.14E-79
TRMT112	0	98.58660513	0.25348224	OK	0
PRDX5	0	77.2038154	0.19848911	OK	0
AP003774.4	0.00039269	3.357897918	0.0084813	OK	0.00076729
CCDC88B	1.04E-18	8.752768445	0.02242037	OK	2.99E-18
RPS6KA4	2.07E-39	13.08262393	0.03355997	OK	7.28E-39
RASGRP2	2.54E-127	23.97491125	0.06157325	OK	1.32E-126
PYGM	1.67E-15	7.877718606	0.02013855	OK	4.55E-15
SF1	1.51E-13	7.293385849	0.01868404	OK	3.99E-13
MEN1	0.00016484	3.590789577	0.00913719	OK	0.00032904
EHD1	7.72E-15	7.683909049	0.01968076	OK	2.09E-14
ATG2A	5.46E-20	9.079467128	0.02326275	OK	1.60E-19
ARL2	0	71.0858175	0.18275279	OK	0
SNX15	6.21E-05	3.837755531	0.00944429	OK	0.00012657
SAC3D1	5.14E-97	20.86880962	0.05357444	OK	2.42E-96
NAALADL1	1.06E-44	13.97823733	0.03585626	OK	3.86E-44
CDCA5	0	116.6238862	0.29814319	OK	0
ZFPL1	3.88E-27	10.72505248	0.02750374	OK	1.24E-26
VPS51	4.27E-146	25.71219823	0.06604912	OK	2.33E-145
FAU	0	215.6845486	0.55453767	OK	0
MRPL49	2.11E-140	25.19785538	0.06471558	OK	1.14E-139
CAPN1	2.20E-42	13.59308655	0.03488234	OK	7.92E-42
POLA2	8.26E-10	6.028716451	0.01540208	OK	2.01E-09
CDC42EP2	1.11E-132	24.48341026	0.06284306	OK	5.86E-132
DPF2	2.93E-19	8.894810295	0.02279505	OK	8.48E-19
TIGD3	4.40E-13	7.148225974	0.01822087	OK	1.15E-12
SLC25A45	7.06E-08	5.263648214	0.01345334	OK	1.62E-07
FRMD8	1.31E-14	7.61534124	0.01949621	OK	3.54E-14
NEAT1	0	119.9192408	0.30832989	OK	0
MALAT1	0	71.05143984	0.18253348	OK	0
SCYL1	6.65E-12	6.765268618	0.01732122	OK	1.70E-11
LTBP3	0	56.96786066	0.14642515	OK	0
SSSCA1	3.89E-73	18.05073513	0.04634778	OK	1.65E-72
FAM89B	0	47.85118074	0.1229944	OK	0
EHBP1L1	1.44E-05	4.182832949	0.01068281	OK	3.02E-05

MAP3K11	4.15E-141	25.26226221	0.06488245	OK	2.25E-140
PCNX3	1.00E-05	4.264772309	0.01087919	OK	2.12E-05
SIPA1	0	41.62352593	0.10695734	OK	0
RELA	5.57E-11	6.450608908	0.01651318	OK	1.39E-10
KAT5	9.27E-09	5.625179862	0.01438849	OK	2.19E-08
RNASEH2C	0	57.80270289	0.14859018	OK	0
AP5B1	5.30E-46	14.18974441	0.03639127	OK	1.95E-45
CFL1	0	186.1105409	0.47855264	OK	0
EFEMP2	0	104.8923623	0.26966242	OK	0
CTSW	6.48E-89	19.95724736	0.05118915	OK	2.96E-88
FIBP	0	48.62482604	0.12498342	OK	0
CCDC85B	0	62.11262627	0.15967553	OK	0
FOSL1	9.95E-71	17.74194141	0.04552136	OK	4.17E-70
C11orf68	1.26E-55	15.66782153	0.04020684	OK	4.88E-55
DRAP1	0	82.62689122	0.2124378	OK	0
SART1	3.04E-07	4.988376244	0.01275071	OK	6.84E-07
EIF1AD	4.24E-29	11.1349276	0.02855432	OK	1.38E-28
BANF1	0	70.03846322	0.18006057	OK	0
CST6	0	44.86906602	0.11528741	OK	0
CATSPER1	0	52.88471659	0.1358823	OK	0
SF3B2	4.22E-256	34.16343674	0.08779188	OK	2.88E-255
PACS1	3.81E-06	4.47540494	0.01143185	OK	8.20E-06
KLC2	3.44E-22	9.615460561	0.02462412	OK	1.04E-21
RAB1B	4.23E-37	12.67190504	0.03251383	OK	1.46E-36
YIF1A	9.33E-258	34.27475959	0.08807663	OK	6.37E-257
CD248	4.93E-256	34.158886	0.08752155	OK	3.36E-255
RIN1	8.95E-18	8.506609165	0.02176324	OK	2.54E-17
BRMS1	1.88E-153	26.36171497	0.06772035	OK	1.05E-152
B4GAT1	9.39E-83	19.23522239	0.04936285	OK	4.17E-82
MRPL11	3.79E-300	37.01112896	0.09511368	OK	2.74E-299
PELI3	4.51E-16	8.039450937	0.02058187	OK	1.24E-15
DPP3	2.92E-76	18.44384285	0.04733691	OK	1.26E-75
BBS1	5.17E-05	3.882438815	0.00982868	OK	0.00010576
ZDHHC24	1.03E-229	32.33779317	0.08308447	OK	6.76E-229
CTSF	0	82.99305121	0.21335719	OK	0
CCS	5.66E-35	12.28191006	0.03151209	OK	1.93E-34
RBM14	2.01E-05	4.10635877	0.0104806	OK	4.20E-05
RCE1	1.47E-35	12.39054777	0.03178204	OK	5.03E-35
PC	1.11E-21	9.493914136	0.02426796	OK	3.34E-21
LRFN4	1.81E-07	5.087502543	0.01299527	OK	4.11E-07
RHOD	0	63.6591145	0.16358574	OK	0
KDM2A	1.27E-43	13.80026101	0.03541398	OK	4.60E-43
GRK2	0	44.31430472	0.1138828	OK	0
ANKRD13D	2.01E-05	4.105832163	0.01048438	OK	4.20E-05
SSH3	1.88E-10	6.263371142	0.01601665	OK	4.65E-10
CLCF1	2.82E-13	7.208921862	0.01842442	OK	7.41E-13
PPP1CA	0	113.340907	0.29143081	OK	0
TBC1D10C	1.26E-138	25.03543405	0.06426914	OK	6.77E-138
CARNS1	0.00011652	3.680206974	0.0093102	OK	0.00023442
RPS6KB2	2.90E-19	8.895811514	0.02280107	OK	8.41E-19
CORO1B	0	61.62286569	0.15841363	OK	0
CABP4	1.03E-08	5.606239148	0.01427488	OK	2.44E-08
TMEM134	0	37.92641636	0.09746545	OK	0

AIP	2.26E-220	31.66627039	0.08136735	OK	1.46E-219
PITPNM1	0.00049217	3.294967948	0.00839188	OK	0.00095505
CDK2AP2	1.65E-293	36.59605857	0.09404616	OK	1.18E-292
GSTP1	0	172.7736587	0.44425908	OK	0
NDUFV1	7.37E-206	30.59395309	0.07860903	OK	4.62E-205
NUDT8	3.01E-39	13.05411436	0.03347046	OK	1.06E-38
ACY3	2.00E-25	10.35409666	0.02583914	OK	6.28E-25
UNC93B1	0	103.3067097	0.26561045	OK	0
ALDH3B1	6.01E-235	32.70798783	0.08402154	OK	3.98E-234
NDUFS8	0	54.33101057	0.13966168	OK	0
TCIRG1	0	80.49357301	0.20693849	OK	0
CHKA	1.27E-247	33.5877464	0.08628295	OK	8.52E-247
LRP5	9.08E-32	11.66993877	0.02989319	OK	3.01E-31
GAL	0	45.88807168	0.11652594	OK	0
CPT1A	1.41E-09	5.941696936	0.01520262	OK	3.40E-09
MRPL21	4.10E-262	34.56601028	0.08882541	OK	2.81E-261
MRGPRF	0	88.84081376	0.22835689	OK	0
MRGPRF-AS1	4.76E-13	7.137158554	0.01815069	OK	1.25E-12
MYEOV	1.36E-35	12.39684418	0.03150601	OK	4.65E-35
CCND1	0	54.60538019	0.14034848	OK	0
AP003555.3	1.00E-11	6.705333917	0.01404613	OK	2.56E-11
ANO1	5.36E-257	34.22373538	0.0878848	OK	3.66E-256
FADD	5.81E-19	8.818229892	0.0225948	OK	1.67E-18
PPFIA1	1.95E-27	10.78843554	0.02767029	OK	6.24E-27
CTTN	0	88.10965442	0.22650654	OK	0
DHCR7	1.12E-05	4.239157402	0.01080474	OK	2.37E-05
AP002387.2	6.63E-21	9.306169351	0.02383843	OK	1.97E-20
RNF121	6.47E-09	5.686852607	0.01454335	OK	1.53E-08
IL18BP	5.61E-270	35.08550368	0.09012644	OK	3.90E-269
LAMTOR1	0	97.49903127	0.25068779	OK	0
ANAPC15	1.22E-200	30.1991309	0.07758987	OK	7.55E-200
FOLR3	1.76E-11	6.622659759	0.01678051	OK	4.46E-11
FOLR1	1.67E-09	5.913882754	0.01496808	OK	4.02E-09
FOLR2	0	281.7808349	0.72460128	OK	0
INPPL1	2.17E-06	4.594684899	0.01173639	OK	4.71E-06
CLPB	2.69E-33	11.9654943	0.03068474	OK	9.07E-33
PDE2A	8.99E-21	9.273685634	0.02371011	OK	2.67E-20
ARAP1	6.46E-144	25.51652328	0.065532	OK	3.52E-143
STARD10	5.91E-176	28.25657188	0.07257944	OK	3.48E-175
ATG16L2	5.31E-22	9.570620824	0.0245321	OK	1.60E-21
FCHSD2	5.08E-57	15.87044351	0.04073873	OK	1.99E-56
P2RY6	0	38.2890343	0.09835519	OK	0
AP002761.3	5.21E-06	4.408227579	0.01114988	OK	1.11E-05
ARHGEF17	0	42.09792052	0.10814218	OK	0
RELT	0	43.19827555	0.11099901	OK	0
RAB6A	2.71E-07	5.010812947	0.01281203	OK	6.10E-07
MRPL48	1.25E-27	10.82908936	0.02776953	OK	4.01E-27
COA4	1.62E-252	33.921326	0.08716717	OK	1.10E-251
PAAF1	2.50E-06	4.564394977	0.01165989	OK	5.43E-06
UCP2	0	189.7236314	0.48787529	OK	0
UCP3	7.69E-06	4.323073486	0.01065448	OK	1.63E-05
PGM2L1	1.10E-08	5.59539288	0.01431086	OK	2.59E-08
KCNE3	1.85E-109	22.19662743	0.05696895	OK	9.13E-109

POLD3	1.88E-45	14.10084545	0.03618319	OK	6.88E-45
RNF169	0.00039155	3.35869919	0.00856257	OK	0.00076514
XRRA1	4.23E-05	3.931023517	0.01002736	OK	8.68E-05
SPCS2	0	102.951585	0.26470838	OK	0
NEU3	0.00048572	3.298670254	0.00839676	OK	0.0009428
SLCO2B1	0	167.3684097	0.43033724	OK	0
ARRB1	1.11E-208	30.80541604	0.07912845	OK	7.01E-208
RPS3	0	191.0861363	0.49130916	OK	0
GDPD5	1.09E-12	7.022553119	0.01790388	OK	2.83E-12
SERPINH1	0	49.39744676	0.1269456	OK	0
MAP6	2.40E-05	4.065189659	0.01033784	OK	4.99E-05
DGAT2	2.45E-302	37.14702068	0.09524122	OK	1.78E-301
UVRAG	7.12E-164	27.25563939	0.07001887	OK	4.07E-163
THAP12	7.23E-50	14.80084892	0.03798753	OK	2.73E-49
LRRC32	2.60E-242	33.22184375	0.08528728	OK	1.74E-241
TSKU	1.86E-12	6.947697347	0.01770663	OK	4.80E-12
ACER3	0	37.77603889	0.0970628	OK	0
MYO7A	6.69E-85	19.48990919	0.04998386	OK	3.00E-84
PAK1	0	83.88290096	0.21564854	OK	0
AP003680.1	7.31E-07	4.816392961	0.01201502	OK	1.62E-06
CLNS1A	0	48.05973077	0.12352958	OK	0
RSF1	4.97E-127	23.94696494	0.06151481	OK	2.58E-126
AAMDC	0	49.86004783	0.12815489	OK	0
KCTD14	7.86E-06	4.318247566	0.01092609	OK	1.67E-05
NDUFC2	0	100.1546818	0.25751625	OK	0
GAB2	2.37E-07	5.036706874	0.01287278	OK	5.34E-07
AP003086.1	0.0004022	3.351279221	0.00850744	OK	0.00078524
FAM181B	1.15E-92	20.3844658	0.05225687	OK	5.33E-92
PRCP	0	72.8077714	0.18716969	OK	0
DDIAS	2.07E-23	9.900527788	0.02519786	OK	6.36E-23
RAB30	1.33E-62	16.65785684	0.04272167	OK	5.37E-62
RAB30-AS1	4.31E-06	4.449159282	0.01136654	OK	9.24E-06
PCF11	1.84E-63	16.77572224	0.04306858	OK	7.45E-63
CCDC90B	7.68E-124	23.63888191	0.06072134	OK	3.94E-123
DLG2	3.26E-09	5.802676965	0.01481995	OK	7.79E-09
TMEM126B	1.21E-69	17.60096208	0.04519134	OK	5.05E-69
TMEM126A	8.61E-162	27.07941078	0.06956848	OK	4.89E-161
CREBZF	5.57E-10	6.092219919	0.0155914	OK	1.36E-09
SYTL2	4.89E-169	27.68786504	0.0710721	OK	2.83E-168
PICALM	5.62E-106	21.83317184	0.05607658	OK	2.73E-105
EED	3.05E-05	4.009288372	0.01023258	OK	6.29E-05
HIKESHI	9.01E-109	22.12537577	0.05682837	OK	4.42E-108
ME3	1.15E-10	6.339986941	0.01620632	OK	2.85E-10
AP003059.2	5.95E-41	13.34966051	0.02780959	OK	2.12E-40
PRSS23	0	114.6378436	0.29469608	OK	0
AP001528.2	0	39.67736722	0.10191122	OK	0
FZD4	2.12E-110	22.29378415	0.05719871	OK	1.05E-109
TMEM135	6.06E-08	5.291812489	0.01352315	OK	1.39E-07
RAB38	0.00024192	3.489543186	0.00888795	OK	0.00047835
CTSC	0	239.3017272	0.61538885	OK	0
NOX4	1.46E-78	18.72810994	0.04797361	OK	6.37E-78
NAALAD2	1.42E-10	6.307103041	0.01585125	OK	3.52E-10
CHORDC1	5.56E-60	16.29274725	0.041827	OK	2.22E-59

FAT3	7.06E-47	14.33049244	0.03673083	OK	2.61E-46
SLC36A4	5.63E-41	13.35381623	0.03426031	OK	2.00E-40
SMCO4	0	92.84896827	0.23871847	OK	0
TAF1D	1.20E-27	10.83313971	0.02778781	OK	3.84E-27
C11orf54	8.79E-08	5.223363439	0.01335587	OK	2.01E-07
MRE11	3.57E-10	6.163113644	0.01577093	OK	8.75E-10
FUT4	3.80E-09	5.77721948	0.0147675	OK	9.06E-09
AMOTL1	9.30E-87	19.70747126	0.05057025	OK	4.20E-86
CWC15	3.98E-204	30.4634037	0.07827436	OK	2.49E-203
SRSF8	8.86E-135	24.67956343	0.06338994	OK	4.71E-134
ENDOD1	0	41.13798187	0.10570594	OK	0
AP000787.1	0.00010868	3.697937987	0.00935675	OK	0.00021896
SESN3	1.03E-114	22.73395022	0.05838745	OK	5.16E-114
FAM76B	4.24E-07	4.923859572	0.01258547	OK	9.47E-07
CEP57	8.23E-46	14.15887959	0.03633781	OK	3.02E-45
MTMR2	1.04E-09	5.99184634	0.01532761	OK	2.51E-09
MAML2	6.68E-28	10.8865352	0.02792223	OK	2.14E-27
CCDC82	1.69E-39	13.09783746	0.03361106	OK	5.97E-39
JRKL	9.20E-05	3.740118342	0.00953971	OK	0.00018597
CNTN5	0.00029272	3.438274707	0.00868703	OK	0.00057558
ARHGAP42	1.51E-187	29.18480332	0.07491421	OK	9.12E-187
PGR	2.37E-145	25.64547226	0.06583153	OK	1.30E-144
TRPC6	7.65E-19	8.787409145	0.02244465	OK	2.20E-18
ANGPTL5	4.81E-26	10.48984281	0.02600096	OK	1.52E-25
CEP126	1.40E-199	30.11808405	0.07734393	OK	8.71E-199
CFAP300	1.92E-09	5.891042083	0.01501617	OK	4.61E-09
YAP1	0	52.10181108	0.133877	OK	0
BIRC3	0	54.51217321	0.14010086	OK	0
BIRC2	6.92E-59	16.13779956	0.04142576	OK	2.75E-58
TMEM123	0	90.86072404	0.23361272	OK	0
MMP7	0	58.43045984	0.14961789	OK	0
MMP8	2.44E-11	6.574402646	0.01647857	OK	6.16E-11
MMP12	3.24E-48	14.54298977	0.03718736	OK	1.21E-47
DCUN1D5	3.53E-66	17.14318878	0.04401138	OK	1.45E-65
DYNC2H1	2.73E-32	11.77171028	0.03016952	OK	9.11E-32
PDGFD	0	63.31416159	0.16269027	OK	0
CASP4	0	63.23552777	0.162558	OK	0
CASP5	6.33E-183	28.81826559	0.07393774	OK	3.78E-182
CASP1	0	128.4897756	0.33037667	OK	0
CARD16	0	138.7843976	0.35685757	OK	0
KBTBD3	1.29E-06	4.702063629	0.01201095	OK	2.82E-06
AASDHPPT	1.01E-29	11.26164972	0.02888649	OK	3.30E-29
GUCY1A2	1.71E-27	10.80066145	0.02747152	OK	5.46E-27
CWF19L2	7.86E-26	10.44328868	0.02678247	OK	2.47E-25
SLC35F2	2.72E-08	5.436401776	0.01387585	OK	6.33E-08
RAB39A	5.83E-78	18.65422498	0.04785597	OK	2.54E-77
CUL5	1.97E-08	5.493580294	0.01405357	OK	4.60E-08
ACAT1	4.96E-186	29.06510609	0.07467308	OK	2.98E-185
NPAT	2.10E-06	4.600779434	0.01175264	OK	4.58E-06
ATM	9.80E-25	10.20111843	0.02615866	OK	3.05E-24
KDELC2	5.97E-149	25.96616402	0.06667508	OK	3.29E-148
EXPH5	2.57E-06	4.558745944	0.01157501	OK	5.58E-06
RDX	2.38E-206	30.63081232	0.07870423	OK	1.50E-205

ZC3H12C	4.07E-05	3.940402712	0.01004695	OK	8.36E-05
FDX1	0	63.19329028	0.16244771	OK	0
ARHGAP20	9.58E-05	3.729721331	0.00949603	OK	0.00019359
COLCA1	2.31E-16	8.121296736	0.020726	OK	6.40E-16
COLCA2	1.35E-07	5.142953883	0.01305164	OK	3.07E-07
LAYN	0	54.72496588	0.14061327	OK	0
SIK2	4.32E-05	3.925845299	0.01001435	OK	8.87E-05
FDXACB1	9.24E-12	6.717479533	0.01714385	OK	2.36E-11
C11orf1	4.36E-40	13.20052641	0.0338662	OK	1.54E-39
CRYAB	0	271.0999974	0.6971615	OK	0
HSPB2	0	71.55586842	0.18390047	OK	0
DIXDC1	0	74.46152256	0.1913962	OK	0
DLAT	3.56E-06	4.490020292	0.0114644	OK	7.67E-06
NKAPD1	2.25E-23	9.892393583	0.0253633	OK	6.90E-23
TIMM8B	0	56.30657804	0.14474017	OK	0
SDHD	0	44.43154189	0.11420041	OK	0
IL18	0	167.6413276	0.43106833	OK	0
BCO2	5.03E-10	6.108556528	0.01561168	OK	1.23E-09
PTS	2.31E-56	15.77511634	0.04049364	OK	9.01E-56
AP002884.1	0	71.47053015	0.18368353	OK	0
AP000802.1	0.00011926	3.674284843	0.00909618	OK	0.00023979
NCAM1	2.23E-06	4.58849745	0.01168686	OK	4.85E-06
ZBTB16	0	52.57376222	0.13511251	OK	0
NNMT	0	171.6375324	0.44134361	OK	0
C11orf71	3.42E-05	3.981526468	0.0101605	OK	7.06E-05
RBM7	2.14E-21	9.425476529	0.02416339	OK	6.40E-21
REXO2	0	58.85311661	0.15128782	OK	0
CADM1	6.09E-290	36.37113277	0.09339863	OK	4.34E-289
AP000462.3	4.19E-07	4.926149164	0.01132725	OK	9.37E-07
LINC00900	8.95E-18	8.506658495	0.02175252	OK	2.54E-17
ZPR1	1.34E-70	17.72531137	0.04550653	OK	5.60E-70
SIK3	1.46E-06	4.676484111	0.01194879	OK	3.19E-06
PAFAH1B2	1.07E-75	18.37335094	0.04717697	OK	4.61E-75
SIDT2	6.71E-14	7.401850512	0.01895184	OK	1.78E-13
TAGLN	0	331.2845957	0.85197321	OK	0
PCSK7	2.12E-28	10.99072754	0.02818819	OK	6.83E-28
RNF214	3.32E-29	11.15659792	0.02861435	OK	1.08E-28
BACE1	4.64E-23	9.819501934	0.02516201	OK	1.42E-22
CEP164	3.92E-08	5.370720289	0.0137346	OK	9.07E-08
FXYD2	2.46E-05	4.059301532	0.01027171	OK	5.11E-05
FXYD6	9.72E-301	37.04786842	0.09514011	OK	7.03E-300
IL10RA	0	105.998665	0.27252776	OK	0
SCN4B	0.00025926	3.471007206	0.00868677	OK	0.00051189
JAML	0	176.5290947	0.45387727	OK	0
MPZL3	9.85E-09	5.614644964	0.01427909	OK	2.32E-08
MPZL2	1.08E-52	15.2322928	0.03892565	OK	4.12E-52
CD3E	0	40.6491525	0.10436544	OK	0
CD3D	0	49.08077431	0.12605081	OK	0
CD3G	0	51.61741115	0.13254141	OK	0
UBE4A	0.00049374	3.294069623	0.00839627	OK	0.00095786
ATP5MG	0	145.1019706	0.37310555	OK	0
KMT2A	7.99E-155	26.48113945	0.06803133	OK	4.48E-154
TMEM25	7.24E-279	35.66363614	0.09158679	OK	5.08E-278

ARCN1	1.11E-68	17.47490938	0.04486768	OK	4.62E-68
PHLDB1	1.27E-88	19.92361885	0.05112907	OK	5.79E-88
AP002954.1	2.10E-21	9.427319698	0.02411007	OK	6.29E-21
DDX6	6.15E-43	13.68604842	0.03512446	OK	2.22E-42
BCL9L	3.70E-17	8.340597118	0.0213614	OK	1.04E-16
RPS25	0	193.7680768	0.49819597	OK	0
TRAPPC4	1.39E-92	20.37528328	0.05232757	OK	6.43E-92
SLC37A4	1.23E-21	9.483394598	0.02429717	OK	3.70E-21
HYOU1	1.17E-16	8.203716072	0.02101653	OK	3.25E-16
VPS11	0.00046684	3.3097821	0.00843229	OK	0.00090712
HMBS	1.19E-27	10.8339551	0.02777022	OK	3.80E-27
H2AFX	1.08E-165	27.40859786	0.07040144	OK	6.23E-165
DPAGT1	2.87E-17	8.370315535	0.02144316	OK	8.07E-17
C2CD2L	8.22E-15	7.675769747	0.01964783	OK	2.22E-14
CBL	1.02E-89	20.04964214	0.05148344	OK	4.65E-89
MCAM	0	182.2539814	0.46863517	OK	0
RNF26	8.48E-11	6.386629856	0.01633847	OK	2.11E-10
USP2	9.22E-138	24.95584843	0.06399812	OK	4.95E-137
USP2-AS1	7.72E-14	7.383338549	0.0184417	OK	2.05E-13
THY1	0	40.42475485	0.10367254	OK	0
OAF	1.92E-41	13.43359104	0.03443752	OK	6.87E-41
TMEM136	2.37E-106	21.87252618	0.05612393	OK	1.15E-105
ARHGEF12	4.24E-61	16.44936013	0.04221753	OK	1.70E-60
SC5D	5.12E-11	6.463404018	0.01654134	OK	1.28E-10
SORL1	2.48E-68	17.42905945	0.04474104	OK	1.03E-67
UBASH3B	1.37E-130	24.28610482	0.06232881	OK	7.21E-130
CRTAM	7.14E-61	16.41772502	0.04210878	OK	2.86E-60
HSPA8	0	122.1967377	0.31419982	OK	0
CLMP	9.92E-175	28.15671217	0.0722469	OK	5.83E-174
GRAMD1B	8.10E-27	10.65688759	0.02729682	OK	2.57E-26
VWA5A	0	38.2702952	0.09832078	OK	0
OR8G5	1.79E-07	5.089634596	0.01243347	OK	4.06E-07
TBRG1	1.23E-51	15.07244738	0.03868838	OK	4.67E-51
SIAE	0.00011727	3.678571111	0.00936489	OK	0.00023591
SPA17	1.80E-10	6.270151446	0.0160367	OK	4.45E-10
NRGN	0	106.0360065	0.27260594	OK	0
ESAM	0	100.309203	0.25784111	OK	0
AP000866.2	3.29E-20	9.134259097	0.0233491	OK	9.70E-20
MSANTD2	0.00020854	3.529029826	0.00899154	OK	0.00041384
ROBO3	3.81E-33	11.93657634	0.03057249	OK	1.28E-32
ROBO4	1.17E-25	10.40517935	0.02653931	OK	3.69E-25
SLC37A2	8.08E-234	32.62854654	0.08379137	OK	5.33E-233
TMEM218	8.01E-43	13.66681098	0.0350686	OK	2.89E-42
PKNOX2	6.00E-06	4.377417708	0.01107486	OK	1.28E-05
FEZ1	0	88.81846255	0.22831641	OK	0
EI24	2.66E-192	29.55706999	0.07593699	OK	1.63E-191
STT3A	1.57E-12	6.970986291	0.0178483	OK	4.07E-12
CHEK1	0	40.74628227	0.1045057	OK	0
PUS3	0.00015251	3.611006487	0.00920712	OK	0.00030505
CDON	1.48E-07	5.126330655	0.01293127	OK	3.35E-07
FAM118B	0.00011141	3.69162679	0.00941311	OK	0.00022438
SRPRA	1.33E-81	19.09744086	0.04904191	OK	5.85E-81
DCPS	2.58E-48	14.55857187	0.03736197	OK	9.63E-48

ST3GAL4	2.03E-90	20.12974685	0.05167951	OK	9.28E-90
AP003481.1	5.33E-111	22.35558246	0.05715937	OK	2.63E-110
ETS1	1.06E-267	34.93579752	0.08974618	OK	7.37E-267
FLI1	2.38E-244	33.36271102	0.08570098	OK	1.60E-243
SENCR	1.12E-48	14.61547411	0.03747894	OK	4.19E-48
KCNJ5	0	45.84350879	0.11770109	OK	0
C11orf45	2.63E-93	20.45655493	0.05242078	OK	1.22E-92
APLP2	0	105.65332	0.27165791	OK	0
ST14	0	71.71917075	0.18433474	OK	0
ADAMTS8	6.62E-18	8.541496628	0.02181841	OK	1.88E-17
NTM	3.51E-06	4.492923969	0.01107532	OK	7.57E-06
AP003025.2	1.21E-47	14.45270095	0.02939201	OK	4.49E-47
JAM3	0	54.24146779	0.13937455	OK	0
NCAPD3	2.74E-59	16.19489532	0.04155411	OK	1.09E-58
VPS26B	1.48E-20	9.220526673	0.0236323	OK	4.38E-20
THYN1	2.09E-163	27.21614589	0.06992048	OK	1.19E-162
ZMYND11	1.31E-82	19.21777928	0.04934353	OK	5.83E-82
DIP2C	6.34E-12	6.772237426	0.01731757	OK	1.62E-11
PRR26	0.0001041	3.708864809	0.00934963	OK	0.00020994
LARP4B	1.16E-11	6.683946925	0.01710992	OK	2.96E-11
GTPBP4	5.77E-59	16.14898228	0.04145444	OK	2.29E-58
IDI2-AS1	5.92E-08	5.296001226	0.01312255	OK	1.36E-07
IDI1	0	44.23240533	0.113681	OK	0
PFKP	4.29E-91	20.20653843	0.05188209	OK	1.97E-90
KLF6	0	106.0729	0.27273386	OK	0
LINC00702	1.30E-251	33.85994686	0.08695381	OK	8.77E-251
AKR1C1	3.38E-153	26.33952895	0.06759729	OK	1.88E-152
AKR1C2	0	55.93898242	0.14370846	OK	0
AKR1C3	0	74.66366607	0.19190761	OK	0
NET1	0	115.0202742	0.29572091	OK	0
ASB13	2.21E-05	4.084868636	0.0104181	OK	4.59E-05
FAM208B	9.58E-05	3.729713091	0.00951574	OK	0.00019359
GDI2	0	61.69841287	0.15861037	OK	0
FBH1	0.00031904	3.41489203	0.00870437	OK	0.00062638
AL137186.2	1.10E-12	7.021779562	0.01795529	OK	2.85E-12
IL15RA	7.20E-15	7.692749926	0.01969442	OK	1.95E-14
IL2RA	0	76.89170536	0.19754034	OK	0
RBM17	7.02E-218	31.48476868	0.08090135	OK	4.51E-217
PFKFB3	0	48.34526951	0.12425192	OK	0
PRKCQ	0.0001283	3.655585888	0.00920595	OK	0.00025757
SFMBT2	5.17E-49	14.66800921	0.0376131	OK	1.94E-48
ITIH5	0	56.81194783	0.14594464	OK	0
KIN	8.54E-08	5.228692275	0.01337251	OK	1.95E-07
ATP5F1C	0	93.05955351	0.23926949	OK	0
TAF3	0.00017155	3.580377747	0.00913239	OK	0.00034209
GATA3	2.06E-126	23.88759014	0.06111431	OK	1.07E-125
SFTA1P	2.11E-23	9.898530462	0.02516775	OK	6.49E-23
CELF2	2.79E-303	37.20543439	0.09561211	OK	2.02E-302
USP6NL	8.75E-07	4.780384161	0.01221122	OK	1.93E-06
ECHDC3	1.93E-10	6.259225205	0.01600359	OK	4.77E-10
UPF2	2.75E-23	9.872107638	0.02531524	OK	8.44E-23
NUDT5	5.47E-165	27.34953411	0.07025965	OK	3.14E-164
CDC123	1.20E-224	31.97540746	0.08216137	OK	7.80E-224

CAMK1D	3.33E-254	34.03543343	0.08744014	OK	2.26E-253
CCDC3	0	115.5885753	0.29715976	OK	0
OPTN	0	76.04747666	0.19549532	OK	0
MCM10	0	90.42305501	0.23064338	OK	0
PHYH	4.67E-68	17.39288594	0.04465526	OK	1.93E-67
SEPHS1	1.79E-11	6.620289949	0.01694605	OK	4.53E-11
BEND7	5.42E-24	10.03366876	0.02567857	OK	1.68E-23
FRMD4A	6.21E-193	29.60622263	0.07605147	OK	3.80E-192
FAM107B	0	124.2287227	0.31943106	OK	0
HSPA14.1	1.29E-53	15.37075265	0.03944518	OK	4.94E-53
RPP38	1.12E-17	8.480777418	0.02173163	OK	3.17E-17
NMT2	1.33E-07	5.146071214	0.01314794	OK	3.02E-07
FAM171A1	7.32E-20	9.047345697	0.02308843	OK	2.14E-19
ITGA8	0	242.5990404	0.62385777	OK	0
MINDY3	1.15E-08	5.587925275	0.01428797	OK	2.70E-08
PTER	3.31E-07	4.972400602	0.01270132	OK	7.42E-07
RSU1	0	42.10179624	0.10820576	OK	0
CUBN	0.00050859	3.2857312	0.00830575	OK	0.00098597
VIM-AS1	1.31E-26	10.61213946	0.02719694	OK	4.14E-26
VIM	0	200.396345	0.51519875	OK	0
ST8SIA6	6.59E-38	12.81697839	0.03220274	OK	2.29E-37
HACD1	0	75.25215231	0.19341595	OK	0
STAM	5.81E-13	7.109812912	0.01820765	OK	1.52E-12
TMEM236	7.60E-32	11.68501515	0.02990372	OK	2.53E-31
MRC1	0	200.58175	0.51577718	OK	0
CACNB2	5.38E-19	8.826945837	0.02257241	OK	1.55E-18
AL450384.2	4.16E-05	3.935232112	0.00999994	OK	8.54E-05
ARL5B	4.68E-83	19.27132231	0.04947481	OK	2.08E-82
PLXDC2	0	40.3762405	0.10376856	OK	0
DNAJC1	4.47E-116	22.87141874	0.0587473	OK	2.25E-115
COMMD3	1.42E-139	25.12214435	0.06453582	OK	7.68E-139
BMI1	4.92E-18	8.575791784	0.02197013	OK	1.40E-17
PIP4K2A	1.43E-109	22.20822482	0.05704063	OK	7.05E-109
MSRB2	0	42.33762173	0.10881432	OK	0
OTUD1	0	136.4037592	0.35073018	OK	0
KIAA1217	8.07E-93	20.40170492	0.05225177	OK	3.75E-92
ARHGAP21	1.07E-17	8.486474174	0.02175003	OK	3.02E-17
PRTFDC1	2.59E-91	20.23147393	0.05190026	OK	1.19E-90
APBB1IP	0	176.1954394	0.45308044	OK	0
PDSS1	1.00E-25	10.42012914	0.02669211	OK	3.15E-25
ABI1	8.55E-250	33.73609644	0.08668634	OK	5.77E-249
YME1L1	1.97E-08	5.493516334	0.01405426	OK	4.60E-08
MASTL	3.48E-50	14.84997745	0.03806742	OK	1.31E-49
ACBD5	5.78E-91	20.19182193	0.05184755	OK	2.66E-90
RAB18	5.97E-79	18.77569501	0.04821338	OK	2.61E-78
MKX	6.37E-14	7.408905927	0.0189031	OK	1.69E-13
MPP7	9.22E-186	29.04378286	0.07456631	OK	5.54E-185
WAC-AS1	8.79E-15	7.667202108	0.01964259	OK	2.37E-14
WAC	2.64E-25	10.32762599	0.02648676	OK	8.27E-25
BAMBI	1.20E-09	5.967472981	0.01525593	OK	2.91E-09
SVIL	0	168.5290128	0.4333542	OK	0
JCAD	1.25E-35	12.40352122	0.03176183	OK	4.28E-35
MAP3K8	0	116.8096645	0.30035015	OK	0

ZNF438	6.58E-08	5.276605057	0.01348937	OK	1.51E-07
ZEB1-AS1	3.74E-14	7.479067108	0.01911891	OK	1.00E-13
ZEB1	0	74.25463221	0.19086999	OK	0
AL161935.3	1.21E-265	34.80010553	0.08938337	OK	8.37E-265
ARHGAP12	7.08E-18	8.533744285	0.02186682	OK	2.01E-17
KIF5B	0	49.76797273	0.12792596	OK	0
EPC1	3.73E-08	5.379952417	0.01376225	OK	8.63E-08
CCDC7	0.00012486	3.662549712	0.00932267	OK	0.00025082
ITGB1	0	126.0758414	0.32417952	OK	0
NRP1	0	62.28926445	0.16012393	OK	0
PARD3	3.69E-35	12.31645267	0.03155609	OK	1.26E-34
CUL2	5.54E-27	10.69206313	0.02741936	OK	1.76E-26
CREM	0	107.1557206	0.27551833	OK	0
CCNY	1.29E-64	16.93257316	0.04347081	OK	5.28E-64
FZD8	3.28E-49	14.69873529	0.03766311	OK	1.23E-48
ZNF248	5.47E-11	6.453470647	0.0165098	OK	1.37E-10
ZNF25	0.00017283	3.578437361	0.00912069	OK	0.00034454
ZNF33A	9.31E-10	6.009380656	0.01537843	OK	2.26E-09
ZNF37A	0.00042858	3.333643936	0.00849638	OK	0.0008358
ZNF33B	9.96E-16	7.941874767	0.02033788	OK	2.73E-15
AL022344.1	6.15E-32	11.70303533	0.02621687	OK	2.04E-31
BMS1	1.84E-15	7.865501382	0.02015176	OK	5.02E-15
CSGALNACT2	0	37.71531442	0.09691919	OK	0
HNRNPF	0	86.83526138	0.22326145	OK	0
ZNF487	4.22E-08	5.357520665	0.01368797	OK	9.74E-08
ZNF32	4.64E-130	24.23605915	0.06225209	OK	2.43E-129
CXCL12	0	84.81898288	0.21805485	OK	0
RASSF4	0	178.0701	0.4578967	OK	0
DEPP1	0	67.86300348	0.17441944	OK	0
ZNF22	5.43E-44	13.86130926	0.03557374	OK	1.98E-43
ALOX5	0	94.22524663	0.24224492	OK	0
MARCH8	2.90E-05	4.020543373	0.01025889	OK	6.01E-05
WASHC2C	9.14E-12	6.719189345	0.0171995	OK	2.33E-11
TIMM23	2.36E-13	7.233246434	0.01852348	OK	6.21E-13
NCOA4	0	72.80149805	0.18716229	OK	0
GDF10	8.49E-56	15.69268018	0.04015502	OK	3.30E-55
ARHGAP22	1.29E-145	25.66912897	0.06591982	OK	7.06E-145
WDFY4	0	44.93484116	0.11539612	OK	0
VSTM4	6.57E-151	26.13898505	0.06709866	OK	3.64E-150
TMEM273	5.55E-141	25.25078663	0.06483242	OK	3.00E-140
ERCC6	4.88E-12	6.809974172	0.01743154	OK	1.25E-11
SLC18A3	5.38E-06	4.401242428	0.01104652	OK	1.15E-05
OGDHL	7.61E-31	11.48769002	0.02917789	OK	2.50E-30
WASHC2A	4.12E-07	4.929409211	0.01259953	OK	9.21E-07
SGMS1	1.47E-141	25.30331817	0.06498975	OK	7.96E-141
PRKG1	0	112.6086366	0.28951115	OK	0
CSTF2T	6.03E-06	4.376363332	0.01117296	OK	1.29E-05
AC074327.1	2.47E-74	18.2023643	0.04666546	OK	1.05E-73
ZWINT	0	118.341002	0.30381802	OK	0
IPMK	3.64E-275	35.42408758	0.09101027	OK	2.54E-274
CISD1	1.61E-87	19.79613218	0.05083407	OK	7.29E-87
UBE2D1	0	72.30881119	0.18589586	OK	0
TFAM	1.08E-111	22.4268269	0.05760231	OK	5.34E-111

BICC1	0	46.02997986	0.11823494	OK	0
LINC00844	8.91E-263	34.61006386	0.0888456	OK	6.12E-262
PHYHIPL	2.11E-71	17.82874756	0.04566151	OK	8.89E-71
FAM13C	0	52.38961424	0.13460722	OK	0
SLC16A9	1.67E-38	12.92289728	0.03309576	OK	5.84E-38
CCDC6	5.50E-98	20.97537755	0.0538679	OK	2.60E-97
ANK3	3.05E-121	23.38483093	0.06001293	OK	1.55E-120
CDK1	0	172.1583977	0.44132494	OK	0
RHOBTB1	1.03E-20	9.259620781	0.02371492	OK	3.04E-20
TMEM26	6.52E-07	4.839029011	0.01230452	OK	1.45E-06
CABCOCO1	1.09E-23	9.964801097	0.02541989	OK	3.35E-23
AL451049.1	1.48E-159	26.88892091	0.05593314	OK	8.38E-159
ARID5B	0	93.19430419	0.23961402	OK	0
RTKN2	1.20E-09	5.968734856	0.01516767	OK	2.89E-09
ZNF365	3.93E-05	3.948515769	0.01003291	OK	8.09E-05
ADO	1.14E-17	8.478150864	0.0217208	OK	3.24E-17
EGR2	0	78.39424445	0.20150715	OK	0
NRBF2	0	72.73336397	0.18698681	OK	0
JMJD1C	0	49.80293258	0.12801511	OK	0
REEP3	4.61E-218	31.49812214	0.08093485	OK	2.96E-217
CTNNA3	3.05E-58	16.04598942	0.04093771	OK	1.20E-57
DNAJC12	1.31E-06	4.698743612	0.01198258	OK	2.87E-06
SIRT1	3.82E-09	5.776001959	0.01477631	OK	9.12E-09
HNRNPH3	4.66E-122	23.46486556	0.06027507	OK	2.38E-121
RUFY2	1.08E-05	4.247151416	0.01084526	OK	2.29E-05
DNA2	4.46E-37	12.66778773	0.03193741	OK	1.54E-36
TET1	9.47E-17	8.228634475	0.02104268	OK	2.64E-16
CCAR1	9.20E-07	4.770294405	0.01219304	OK	2.03E-06
DDX50	1.03E-37	12.78209819	0.03279604	OK	3.58E-37
DDX21	0	107.4066668	0.27616923	OK	0
KIF1BP	1.15E-19	8.997603911	0.02306186	OK	3.36E-19
SRGN	0	307.4593454	0.79069423	OK	0
VPS26A	3.98E-252	33.89478265	0.08709896	OK	2.69E-251
HK1	3.49E-36	12.50545093	0.03208472	OK	1.20E-35
TSPAN15	0	51.22322672	0.13160577	OK	0
AL450311.2	8.66E-07	4.782431879	0.01214072	OK	1.91E-06
FAM241B	1.67E-53	15.35364175	0.03935897	OK	6.43E-53
COL13A1	3.47E-46	14.21951205	0.03642933	OK	1.28E-45
H2AFY2	8.84E-46	14.15382096	0.03629638	OK	3.25E-45
AIFM2	2.35E-08	5.462578564	0.01395974	OK	5.47E-08
SAR1A	2.37E-131	24.35818281	0.06257241	OK	1.25E-130
PPA1	0	91.03576515	0.23405813	OK	0
EIF4EBP2	2.19E-20	9.178062318	0.02352726	OK	6.48E-20
PALD1	1.54E-81	19.08964713	0.04897432	OK	6.80E-81
PRF1	1.27E-37	12.76602864	0.03262247	OK	4.40E-37
SGPL1	2.08E-148	25.9181917	0.06656249	OK	1.14E-147
PCBD1	0	42.35827444	0.1088678	OK	0
UNC5B	6.15E-71	17.76893428	0.04557194	OK	2.58E-70
UNC5B-AS1	5.31E-12	6.797968221	0.01734705	OK	1.36E-11
SLC29A3	5.08E-136	24.79500026	0.06365755	OK	2.71E-135
CDH23	5.68E-22	9.563801998	0.02446217	OK	1.71E-21
VSIR	0	103.979597	0.2673471	OK	0
PSAP	0	208.8183083	0.53697415	OK	0

CHST3	7.41E-38	12.80782797	0.03279916	OK	2.58E-37
SPOCK2	0	39.87786468	0.10233023	OK	0
ASCC1	1.16E-06	4.722931714	0.0120657	OK	2.55E-06
ANAPC16	0	84.58607877	0.21747581	OK	0
DDIT4	0	67.91910855	0.17460433	OK	0
DNAJB12	2.80E-131	24.35138652	0.0625521	OK	1.47E-130
MICU1	5.61E-31	11.51399037	0.02953483	OK	1.85E-30
P4HA1	0	99.87870463	0.25679326	OK	0
ECD	2.60E-05	4.046676446	0.01032985	OK	5.39E-05
DNAJC9	1.65E-84	19.44358082	0.04992096	OK	7.40E-84
MRPS16	9.05E-251	33.80252837	0.08685998	OK	6.12E-250
ANXA7	0	49.36952759	0.12690048	OK	0
PPP3CB	2.07E-21	9.428773267	0.02417037	OK	6.21E-21
USP54	3.29E-07	4.973577077	0.01268419	OK	7.37E-07
MYOZ1	0	42.64051233	0.10951143	OK	0
SEC24C	1.30E-06	4.700892865	0.012005	OK	2.84E-06
CHCHD1	9.45E-277	35.52688817	0.09129511	OK	6.61E-276
NDST2	3.55E-06	4.490729648	0.01145164	OK	7.65E-06
CAMK2G	0	40.71707846	0.10462489	OK	0
PLAU	0	79.88422454	0.20535347	OK	0
VCL	0	112.2810675	0.28868431	OK	0
AP3M1	4.33E-05	3.925445042	0.01001731	OK	8.88E-05
ADK	4.09E-70	17.66237032	0.04534497	OK	1.71E-69
KAT6B	2.97E-44	13.90457866	0.03567964	OK	1.08E-43
SAMD8	1.09E-08	5.597286656	0.01431654	OK	2.56E-08
VDAC2	0	87.05541305	0.22382669	OK	0
COMTD1	3.86E-45	14.04982967	0.03605494	OK	1.41E-44
ZNF503	3.23E-136	24.81316744	0.06373939	OK	1.72E-135
LRMDA	0	46.03705749	0.11832298	OK	0
KCNMA1	0	60.46597835	0.15543235	OK	0
DLG5	3.22E-08	5.405902846	0.01380047	OK	7.48E-08
RPS24	0	193.226953	0.49676298	OK	0
ZMIZ1-AS1	2.21E-71	17.82616214	0.04561407	OK	9.31E-71
ZMIZ1	1.06E-77	18.62216345	0.04780446	OK	4.61E-77
PPIF	0	136.6241404	0.35128803	OK	0
ZCCHC24	7.46E-40	13.15996025	0.03374658	OK	2.63E-39
SFTPD	2.21E-05	4.084589082	0.01036401	OK	4.60E-05
PLAC9	0	172.406669	0.44330904	OK	0
ANXA11	0	48.35249554	0.12428558	OK	0
MAT1A	3.44E-13	7.181821659	0.01697496	OK	9.02E-13
FAM213A	1.51E-169	27.73012001	0.07123352	OK	8.79E-169
TSPAN14	2.89E-197	29.94090752	0.07691692	OK	1.78E-196
NRG3	9.95E-19	8.75787104	0.02090483	OK	2.86E-18
GHITM	0	61.75852358	0.15876535	OK	0
CCSER2	0	41.08404823	0.10558456	OK	0
GRID1	6.44E-06	4.362162725	0.01091145	OK	1.37E-05
WAPL	2.85E-45	14.0714015	0.03611266	OK	1.04E-44
LDB3	0	45.33220992	0.11643897	OK	0
AC067750.1	2.30E-09	5.861070618	0.01492353	OK	5.51E-09
BMPR1A	2.16E-81	19.07202056	0.04893959	OK	9.51E-81
MMRN2	1.99E-180	28.61820322	0.07314392	OK	1.18E-179
SNCG	0	117.5049527	0.30209007	OK	0
ADIRF	0	313.8691752	0.80717925	OK	0

GLUD1	1.01E-196	29.89902447	0.07682054	OK	6.25E-196
SHLD2	8.24E-22	9.525115352	0.02441552	OK	2.48E-21
NUTM2A-AS1	1.11E-17	8.482037659	0.02173457	OK	3.13E-17
MINPP1	2.10E-09	5.876000421	0.01502595	OK	5.04E-09
PAPSS2	0	40.38491608	0.10376599	OK	0
ATAD1	4.05E-72	17.92085789	0.04600683	OK	1.71E-71
PTEN	6.76E-161	27.00328404	0.06937472	OK	3.84E-160
ANKRD22	9.89E-298	36.8605894	0.09463105	OK	7.13E-297
STAMBPL1	7.41E-17	8.257911373	0.02112084	OK	2.07E-16
ACTA2-AS1	1.12E-13	7.333187721	0.01863429	OK	2.98E-13
ACTA2	0	320.6657221	0.82466003	OK	0
FAS	1.59E-56	15.79854081	0.04054257	OK	6.22E-56
CH25H	0	110.1557627	0.28316831	OK	0
LIPA	0	147.7305357	0.3798651	OK	0
IFIT2	7.44E-94	20.51795336	0.05267471	OK	3.47E-93
IFIT3	1.35E-48	14.60291887	0.03746962	OK	5.04E-48
IFIT1	8.29E-38	12.79916795	0.03283155	OK	2.88E-37
IFIT5	1.71E-07	5.09825222	0.01302962	OK	3.88E-07
PANK1	0.00013256	3.647190504	0.00925864	OK	0.00026584
KIF20B	3.29E-175	28.19584439	0.07241391	OK	1.93E-174
LINC01374	8.92E-06	4.290430659	0.01089454	OK	1.89E-05
HTR7	1.59E-05	4.160214313	0.01058258	OK	3.34E-05
RPP30	6.34E-57	15.85644144	0.04070258	OK	2.48E-56
ANKRD1	1.26E-08	5.571323046	0.01405257	OK	2.97E-08
PCGF5	4.77E-135	24.70460892	0.06346011	OK	2.54E-134
HECTD2	3.28E-13	7.188481864	0.01840416	OK	8.60E-13
PPP1R3C	0	61.92901034	0.15913631	OK	0
TNKS2	1.64E-15	7.879906494	0.02018972	OK	4.47E-15
FGFBP3	0.00010152	3.71521321	0.0094604	OK	0.00020485
MARCH5	3.25E-22	9.621189307	0.02466545	OK	9.85E-22
KIF11	0	106.0038652	0.27124716	OK	0
HHEX	1.90E-233	32.6023461	0.08374691	OK	1.25E-232
EXOC6	2.42E-10	6.224220921	0.0159288	OK	5.96E-10
MYOF	2.57E-41	13.41196591	0.03441502	OK	9.19E-41
CEP55	0	179.1470907	0.45923428	OK	0
FFAR4	1.55E-23	9.929379959	0.02538932	OK	4.77E-23
RBP4	1.14E-16	8.206184931	0.02090879	OK	3.18E-16
FRA10AC1	7.42E-131	24.31144896	0.06244554	OK	3.90E-130
PLCE1	1.84E-32	11.8048207	0.03024365	OK	6.16E-32
NOC3L	3.10E-08	5.412844005	0.01384389	OK	7.20E-08
TBC1D12	1.52E-62	16.64964967	0.04273507	OK	6.15E-62
HELLS	0	50.54697503	0.12976961	OK	0
PDLIM1	0	127.2365105	0.32714882	OK	0
SORBS1	0	97.20907133	0.24989307	OK	0
TCTN3	8.41E-88	19.82872966	0.05090287	OK	3.82E-87
ENTPD1	2.70E-295	36.70820962	0.09431545	OK	1.94E-294
BLNK	0	51.5602029	0.13248016	OK	0
TM9SF3	1.26E-94	20.60406893	0.05291729	OK	5.89E-94
PIK3AP1	0	66.91158205	0.17198793	OK	0
LCOR	5.62E-06	4.391894591	0.01121929	OK	1.20E-05
ARHGAP19	3.96E-08	5.368835187	0.01368971	OK	9.16E-08
FRAT1	1.48E-58	16.09060275	0.04128723	OK	5.87E-58
FRAT2	7.08E-270	35.07884699	0.09012431	OK	4.92E-269

RRP12	1.31E-23	9.946126381	0.02548441	OK	4.04E-23
PGAM1	0	120.2195836	0.30912334	OK	0
EXOSC1	4.79E-44	13.87037843	0.03559429	OK	1.74E-43
ZDHHC16	5.83E-14	7.420629369	0.01899933	OK	1.55E-13
UBTD1	6.78E-38	12.81475699	0.03286795	OK	2.36E-37
ANKRD2	2.09E-34	12.17578664	0.03013272	OK	7.10E-34
MORN4	0.00035436	3.386184682	0.00860662	OK	0.00069425
PI4K2A	3.89E-219	31.57643815	0.08111205	OK	2.51E-218
AVPI1	0	46.72972625	0.1200963	OK	0
MARVELD1	2.52E-123	23.58864813	0.06058145	OK	1.29E-122
SFRP5	2.17E-06	4.594853485	0.01141157	OK	4.71E-06
GOLGA7B	0	57.83262085	0.14842936	OK	0
CRTAC1	2.13E-188	29.25172537	0.07500214	OK	1.29E-187
LOXL4	2.83E-29	11.17085003	0.02837394	OK	9.19E-29
HPS1	4.55E-147	25.79901833	0.06627212	OK	2.49E-146
HPSE2	1.51E-12	6.977098164	0.01778355	OK	3.90E-12
GOT1	2.99E-07	4.991827134	0.01275756	OK	6.72E-07
SLC25A28	5.61E-16	8.012630466	0.02052907	OK	1.54E-15
CUTC	1.57E-17	8.441096515	0.02163075	OK	4.44E-17
COX15	0.00011976	3.673210265	0.00936776	OK	0.00024076
ERLIN1	5.41E-06	4.399920577	0.01123591	OK	1.16E-05
CHUK	7.37E-05	3.795349824	0.00967905	OK	0.00014977
BLOC1S2	0	71.68667983	0.18429156	OK	0
PKD2L1	7.77E-78	18.63891234	0.04776522	OK	3.37E-77
SCD	0	72.53684856	0.18642504	OK	0
NDUFB8	0	94.32340654	0.24251917	OK	0
MRPL43	0	45.7095423	0.11748699	OK	0
LZTS2	1.02E-61	16.53561428	0.04244254	OK	4.09E-61
SFXN3	1.60E-09	5.920992005	0.01515011	OK	3.85E-09
KAZALD1	7.71E-148	25.8676208	0.06638657	OK	4.23E-147
DPCD	6.07E-16	8.003045515	0.02049922	OK	1.67E-15
FBXW4	2.48E-09	5.848258512	0.0149546	OK	5.95E-09
NPM3	5.69E-91	20.19254333	0.05183556	OK	2.62E-90
OGA	0.00031167	3.421249543	0.00872413	OK	0.00061225
ARMH3	3.66E-08	5.382953026	0.01376227	OK	8.49E-08
PPRC1	3.00E-05	4.012736664	0.01023262	OK	6.21E-05
NOLC1	2.37E-28	10.98050491	0.02816212	OK	7.64E-28
NFKB2	3.65E-24	10.07254108	0.025822	OK	1.13E-23
FBXL15	7.28E-86	19.60307615	0.05034037	OK	3.28E-85
CUEDC2	6.38E-184	28.89769816	0.07424668	OK	3.82E-183
MFSD13A	4.12E-25	10.28503687	0.0263553	OK	1.29E-24
ACTR1A	7.47E-82	19.12737158	0.04911607	OK	3.30E-81
SUFU	2.73E-05	4.034868167	0.01028735	OK	5.66E-05
TRIM8	1.05E-81	19.10941637	0.0490699	OK	4.66E-81
ARL3	0	43.2558921	0.11117349	OK	0
CYP17A1-AS1	1.17E-06	4.720785403	0.01198342	OK	2.58E-06
BORCS7	1.06E-63	16.80844233	0.04315306	OK	4.30E-63
CNNM2	1.16E-08	5.586448518	0.01426789	OK	2.73E-08
INA	3.54E-17	8.345804885	0.02068745	OK	9.93E-17
PCGF6	8.52E-05	3.759394789	0.00958556	OK	0.00017246
ATP5MD	0	91.4309064	0.23507916	OK	0
CALHM2	2.00E-58	16.07218464	0.04125525	OK	7.90E-58
NEURL1	7.13E-15	7.693975044	0.01964357	OK	1.93E-14

SH3PXD2A	6.72E-08	5.272674784	0.01347746	OK	1.54E-07
STN1	1.73E-10	6.276248215	0.01606174	OK	4.29E-10
SLK	9.45E-20	9.019454415	0.02312151	OK	2.76E-19
COL17A1	2.56E-21	9.406509857	0.02190325	OK	7.66E-21
SFR1	7.23E-08	5.259334186	0.01344694	OK	1.66E-07
GSTO1	0	160.8829893	0.41370565	OK	0
GSTO2	4.27E-12	6.829053881	0.0174355	OK	1.10E-11
ITPRIP	1.35E-32	11.83125339	0.03034371	OK	4.50E-32
XPNPEP1	3.06E-24	10.08987518	0.02586382	OK	9.49E-24
ADD3	2.19E-165	27.3829779	0.07033059	OK	1.26E-164
MXI1	0	61.57561657	0.15828765	OK	0
SMNDC1	1.40E-48	14.60011247	0.0374741	OK	5.25E-48
DUSP5	0	76.65222134	0.19702637	OK	0
SMC3	5.27E-132	24.41976557	0.06272948	OK	2.78E-131
RBM20	0.00019864	3.541880941	0.00897733	OK	0.00039479
PDCD4-AS1	1.44E-10	6.305554253	0.01611093	OK	3.56E-10
PDCD4	3.08E-138	24.99974598	0.06421926	OK	1.65E-137
BBIP1	9.19E-26	10.42851911	0.02674253	OK	2.89E-25
SHOC2	1.19E-29	11.24739255	0.02885161	OK	3.88E-29
ADRA2A	3.36E-184	28.91985394	0.07404449	OK	2.01E-183
ACSL5	1.22E-42	13.63609971	0.03496112	OK	4.40E-42
ZDHHC6	7.88E-19	8.784142682	0.02251305	OK	2.27E-18
VTI1A	1.11E-16	8.209491161	0.02103179	OK	3.10E-16
TCF7L2	5.40E-65	16.98390258	0.04359586	OK	2.21E-64
AL158212.2	8.66E-05	3.755316904	0.00954865	OK	0.00017523
CASP7	1.24E-06	4.710044392	0.01203462	OK	2.71E-06
NHLRC2	4.49E-09	5.748896821	0.01470452	OK	1.07E-08
ADRB1	5.66E-16	8.011535298	0.02044856	OK	1.56E-15
CCDC186	1.28E-28	11.03597553	0.02830737	OK	4.14E-28
AFAP1L2	4.85E-37	12.66123397	0.03232756	OK	1.67E-36
ABLIM1	1.35E-192	29.57992698	0.07588778	OK	8.27E-192
FAM160B1	7.35E-09	5.664925038	0.01448444	OK	1.74E-08
GFRA1	1.36E-14	7.610620598	0.01920792	OK	3.67E-14
PNLIPRP3	7.03E-54	15.40984769	0.03217718	OK	2.70E-53
HSPA12A	7.15E-13	7.081017934	0.0180749	OK	1.87E-12
SHTN1	0	98.11124377	0.25224974	OK	0
AL731557.1	7.38E-88	19.835308	0.03777076	OK	3.35E-87
SLC18A2	6.29E-59	16.14366927	0.0399826	OK	2.50E-58
PDZD8	0.00023364	3.498844491	0.00892199	OK	0.00046243
EMX2OS	1.27E-16	8.193509129	0.02091956	OK	3.53E-16
EMX2	7.45E-53	15.25649448	0.03908665	OK	2.85E-52
RAB11FIP2	4.74E-05	3.903573511	0.0099623	OK	9.70E-05
FAM204A	6.39E-188	29.21426454	0.07506246	OK	3.86E-187
CACUL1	0	42.78458548	0.10995455	OK	0
EIF3A	1.94E-268	34.98448579	0.0899034	OK	1.34E-267
FAM45A	7.06E-94	20.52053091	0.05269579	OK	3.29E-93
SFXN4	9.81E-14	7.35139667	0.01882422	OK	2.60E-13
PRDX3	0	68.7160687	0.17665985	OK	0
GRK5	1.27E-73	18.11242751	0.04647877	OK	5.40E-73
RGS10	0	218.6317872	0.5622342	OK	0
TIAL1	1.27E-26	10.61465716	0.02722487	OK	4.03E-26
BAG3	5.27E-37	12.65470771	0.03246556	OK	1.82E-36
INPP5F	1.16E-07	5.171785908	0.01321877	OK	2.64E-07

MCMBP	8.58E-24	9.988272925	0.02560747	OK	2.65E-23
SEC23IP	8.90E-06	4.29090812	0.01095628	OK	1.89E-05
PLPP4	4.62E-199	30.07860585	0.07700739	OK	2.86E-198
WDR11	2.11E-34	12.17488468	0.03123061	OK	7.18E-34
FGFR2	3.04E-08	5.416320319	0.01350865	OK	7.07E-08
ATE1	4.49E-05	3.9167202	0.00999632	OK	9.20E-05
NSMCE4A	2.10E-47	14.41449799	0.03699356	OK	7.80E-47
TACC2	5.49E-87	19.73413877	0.0506292	OK	2.48E-86
PLEKHA1	5.45E-71	17.77575905	0.04563358	OK	2.29E-70
HTRA1	0	131.6024883	0.33839284	OK	0
ACADSB	1.32E-08	5.564248285	0.0142169	OK	3.09E-08
BUB3	3.37E-122	23.47860565	0.06030863	OK	1.72E-121
CPXM2	0	62.84461998	0.16148177	OK	0
CHST15	0	38.28883406	0.09835189	OK	0
OAT	6.95E-268	34.94795491	0.08980252	OK	4.82E-267
LHPP	2.78E-07	5.005825821	0.01279443	OK	6.26E-07
FAM53B	4.49E-27	10.71161323	0.02744744	OK	1.43E-26
EEF1AKMT2	8.61E-11	6.384224241	0.01634109	OK	2.14E-10
ABRAXAS2	3.94E-16	8.056122584	0.02064071	OK	1.09E-15
CTBP2	6.15E-20	9.066358893	0.02324123	OK	1.80E-19
AL731571.1	1.63E-28	11.01431414	0.02809637	OK	5.26E-28
UROS	1.02E-56	15.8264578	0.04062532	OK	4.00E-56
BCCIP	3.89E-18	8.602705874	0.02204798	OK	1.11E-17
ADAM12	6.87E-12	6.76062917	0.01715858	OK	1.76E-11
DOCK1	7.40E-21	9.294490281	0.02381726	OK	2.20E-20
PTPRE	0	222.4251527	0.57198521	OK	0
MKI67	0	264.5086239	0.67820774	OK	0
MGMT	2.95E-194	29.70886154	0.07633319	OK	1.81E-193
EBF3	2.91E-10	6.195244067	0.01568266	OK	7.15E-10
GLRX3	1.19E-227	32.19073568	0.08271285	OK	7.79E-227
BNIP3	0	54.8022597	0.14086089	OK	0
DPYSL4	2.57E-13	7.22152166	0.01809094	OK	6.76E-13
STK32C	1.15E-73	18.11798043	0.04650433	OK	4.88E-73
LRRC27	0.00023379	3.498678251	0.00890826	OK	0.00046268
PWWP2B	2.81E-63	16.75044318	0.04297634	OK	1.14E-62
INPP5A	5.94E-195	29.7627152	0.07641613	OK	3.65E-194
UTF1	1.11E-207	30.73059163	0.07885035	OK	7.01E-207
VENTX	3.82E-37	12.67987773	0.03249594	OK	1.32E-36
ADAM8	0	69.12618996	0.17766053	OK	0
TUBGCP2	0	43.68076121	0.11226581	OK	0
ZNF511	1.32E-107	22.00394947	0.05651511	OK	6.46E-107
FUOM	0	114.0344988	0.29320574	OK	0
ECHS1	0	46.43949563	0.11936464	OK	0
PAOX	2.59E-13	7.220235516	0.018477	OK	6.82E-13
CYP2E1	8.03E-95	20.62591132	0.05186472	OK	3.76E-94
AC026369.3	5.92E-22	9.55936097	0.0244123	OK	1.79E-21
AC007406.3	4.11E-173	28.02432904	0.0704944	OK	2.40E-172
SLC6A12	1.49E-06	4.672578902	0.01183176	OK	3.25E-06
CCDC77	3.44E-06	4.497475723	0.01148239	OK	7.41E-06
NINJ2	1.44E-125	23.80624111	0.06111565	OK	7.42E-125
WNK1	1.35E-49	14.75876806	0.03788213	OK	5.09E-49
WNT5B	0.00033298	3.403222946	0.0086081	OK	0.00065312
ADIPOR2	7.88E-19	8.78406103	0.022512	OK	2.27E-18

CACNA2D4	0	42.14134868	0.10826932	OK	0
CACNA1C	7.94E-141	25.23661578	0.06476912	OK	4.29E-140
FKBP4	1.01E-43	13.81701161	0.03545887	OK	3.65E-43
NRIP2	0	56.29875998	0.14443821	OK	0
FOXM1	0	76.71829383	0.19394605	OK	0
RHNO1	1.02E-18	8.75507917	0.02243208	OK	2.93E-18
TULP3	1.47E-39	13.10876606	0.03361752	OK	5.17E-39
TEAD4	1.56E-10	6.293158963	0.01605905	OK	3.85E-10
TSPAN9	2.87E-106	21.86377568	0.05610892	OK	1.40E-105
PARP11	5.30E-14	7.433225808	0.01902866	OK	1.41E-13
CCND2	5.31E-239	32.99190174	0.08477115	OK	3.53E-238
TIGAR	0	53.28678574	0.13695672	OK	0
C12orf4	7.34E-15	7.690283486	0.01969401	OK	1.98E-14
RAD51AP1	0	53.06283041	0.13630706	OK	0
DYRK4	6.96E-49	14.64776837	0.03759411	OK	2.61E-48
NDUFA9	8.58E-131	24.30544526	0.06243377	OK	4.51E-130
KCNA5	0	60.08357221	0.15432418	OK	0
NTF3	0	45.78945936	0.1175957	OK	0
VWF	0	62.74362482	0.16080382	OK	0
CD9	0	137.6465851	0.35394191	OK	0
TNFRSF1A	1.20E-220	31.6863827	0.08141959	OK	7.73E-220
LTBR	5.68E-33	11.90346483	0.0305372	OK	1.91E-32
CD27	1.79E-40	13.26752638	0.03392273	OK	6.33E-40
TAPBPL	1.32E-21	9.476384517	0.02429109	OK	3.95E-21
MRPL51	0	68.60786521	0.17638114	OK	0
NCAPD2	7.88E-61	16.41177118	0.04207742	OK	3.16E-60
GAPDH	0	225.9275887	0.58090525	OK	0
CHD4	8.19E-29	11.07614729	0.02841158	OK	2.65E-28
LPAR5	1.32E-126	23.90605196	0.06133687	OK	6.86E-126
ACRBP	7.61E-92	20.29174	0.0520645	OK	3.51E-91
ING4	4.13E-34	12.12013293	0.03108968	OK	1.40E-33
PIANP	3.63E-61	16.45869784	0.04215751	OK	1.46E-60
COPS7A	1.18E-41	13.46966557	0.03456483	OK	4.23E-41
MLF2	8.93E-286	36.10661743	0.09278904	OK	6.33E-285
PTMS	0	144.3829704	0.37126918	OK	0
LAG3	3.28E-23	9.854505372	0.02521271	OK	1.00E-22
CD4	0	140.8985839	0.3622827	OK	0
GPR162	1.98E-10	6.255265818	0.0159837	OK	4.89E-10
P3H3	3.70E-115	22.77907276	0.05845416	OK	1.85E-114
CDCA3	0	130.7735069	0.33420985	OK	0
USP5	2.60E-09	5.840341119	0.0149362	OK	6.23E-09
TPI1	0	175.6577928	0.45169695	OK	0
LRRC23	1.12E-14	7.635677733	0.01955632	OK	3.03E-14
ENO2	5.11E-58	16.01392447	0.04109224	OK	2.02E-57
ATN1	9.87E-18	8.495356727	0.02176285	OK	2.80E-17
C12orf57	0	198.9924896	0.51172158	OK	0
U47924.2	9.25E-09	5.625476005	0.01429813	OK	2.18E-08
PTPN6	0	130.4159452	0.33533186	OK	0
PHB2	0	57.51337742	0.14784672	OK	0
EMG1	3.43E-33	11.94542799	0.03064304	OK	1.15E-32
LPCAT3	2.50E-29	11.18173668	0.02867207	OK	8.14E-29
C1S	0	123.9852525	0.31876374	OK	0
C1R	0	185.8329724	0.47784877	OK	0

C1RL	4.84E-19	8.838750375	0.02264473	OK	1.40E-18
C1RL-AS1	1.48E-29	11.22826507	0.02867794	OK	4.82E-29
RBP5	7.57E-18	8.526076639	0.02141749	OK	2.15E-17
CLSTN3	2.00E-06	4.61140566	0.01172454	OK	4.36E-06
CD163L1	0	62.34292289	0.16016092	OK	0
CD163	0	226.2662089	0.58185525	OK	0
CLEC4C	1.35E-50	14.91337531	0.03688597	OK	5.10E-50
SLC2A14	2.34E-06	4.578666634	0.0116316	OK	5.08E-06
SLC2A3	0	100.0870779	0.25733507	OK	0
C3AR1	0	235.4827083	0.60555808	OK	0
NECAP1	1.67E-10	6.282160088	0.01607938	OK	4.13E-10
CLEC4A	0	108.8610385	0.27986189	OK	0
FAM66C	0.00050573	3.287319232	0.0083345	OK	0.00098052
LINC00937	4.68E-32	11.72609926	0.03004541	OK	1.56E-31
CLEC4D	5.58E-79	18.77930594	0.04811856	OK	2.44E-78
CLEC4E	0	64.95259871	0.16692506	OK	0
MFAP5	0	56.55037316	0.14498821	OK	0
RIMKLB	1.28E-85	19.57433571	0.05024858	OK	5.76E-85
M6PR	0	117.495135	0.30211595	OK	0
KLRG1	5.19E-21	9.33207609	0.02386312	OK	1.55E-20
A2M-AS1	9.40E-06	4.278576404	0.01090661	OK	1.99E-05
A2M	0	166.8904008	0.42915533	OK	0
KLRB1	1.44E-145	25.66494172	0.0658055	OK	7.86E-145
CLEC2D	7.63E-105	21.71359571	0.05572245	OK	3.69E-104
CLECL1	4.38E-288	36.25345818	0.09309857	OK	3.11E-287
CD69	0	75.38434397	0.19377194	OK	0
KLRF1	5.71E-09	5.708125947	0.01454086	OK	1.35E-08
CLEC2B	0	203.437677	0.52314839	OK	0
AC091814.1	0.00046517	3.310788218	0.00834151	OK	0.00090394
LINC02470	5.48E-07	4.873598253	0.0123088	OK	1.22E-06
CLEC12A	0	62.47698784	0.16054506	OK	0
CLEC9A	0	176.6817493	0.45288292	OK	0
CLEC1A	6.42E-07	4.842283772	0.01232685	OK	1.42E-06
CLEC7A	0	213.4738466	0.54895466	OK	0
OLR1	0	148.3764153	0.38151018	OK	0
TMEM52B	2.08E-43	13.76459929	0.03526881	OK	7.54E-43
GABARAPL1	0	41.81546021	0.1074679	OK	0
KLRD1	6.43E-07	4.841933484	0.0123276	OK	1.43E-06
AC022075.1	3.90E-06	4.470766827	0.01113307	OK	8.38E-06
MAGOHB	4.62E-43	13.70690266	0.03517245	OK	1.67E-42
YBX3	0	68.42220098	0.17590032	OK	0
SMIM10L1	4.57E-78	18.66732765	0.04793435	OK	1.99E-77
LINC01252	3.60E-16	8.066981236	0.0205291	OK	9.96E-16
ETV6	1.73E-187	29.18012142	0.07496789	OK	1.05E-186
BCL2L14	0	73.92449902	0.18814025	OK	0
LRP6	1.50E-100	21.25434371	0.05453043	OK	7.17E-100
MANSC1	1.00E-147	25.85751602	0.06636738	OK	5.49E-147
LOH12CR2	1.75E-05	4.138181349	0.01054412	OK	3.66E-05
BORCS5	1.37E-11	6.659478763	0.01704656	OK	3.49E-11
DUSP16	3.86E-05	3.952903403	0.01007996	OK	7.94E-05
CREBL2	0	52.53556103	0.13502922	OK	0
GPR19	0.00013813	3.636593094	0.00786017	OK	0.00027683
CDKN1B	9.51E-222	31.76613224	0.08162307	OK	6.15E-221

APOLD1	0	64.08591153	0.16456176	OK	0
GPRC5A	1.55E-66	17.19105709	0.04409448	OK	6.38E-66
GPRC5D-AS1	6.28E-06	4.367557498	0.01114364	OK	1.34E-05
HEBP1	0	41.24671243	0.10600848	OK	0
EMP1	0	98.22356779	0.25250786	OK	0
ATF7IP	0.00020529	3.533193719	0.00901168	OK	0.00040748
PLBD1	0	126.5944338	0.32550246	OK	0
H2AFJ	0	56.10344307	0.14422024	OK	0
WBP11	8.34E-70	17.62209787	0.04524654	OK	3.48E-69
SMCO3	3.81E-07	4.944763204	0.01239184	OK	8.53E-07
ART4	0	39.78885125	0.10208787	OK	0
MGP	0	277.6383417	0.71398618	OK	0
ARHGDIB	0	186.1567533	0.47871126	OK	0
RERG	0	82.29532352	0.21152082	OK	0
PTPRO	4.77E-32	11.72458761	0.03004992	OK	1.59E-31
EPS8	0	53.55687904	0.1376648	OK	0
STRAP	2.10E-227	32.17314102	0.08267124	OK	1.37E-226
DERA	0	41.08204885	0.10557599	OK	0
MGST1	0	94.90989398	0.24395834	OK	0
LMO3	0	69.19018355	0.17781704	OK	0
AC007529.2	6.07E-107	21.93465407	0.04348748	OK	2.96E-106
RERGL	0	191.8769902	0.49325254	OK	0
PLEKHA5	1.85E-108	22.09281565	0.0567327	OK	9.09E-108
AEBP2	1.31E-70	17.72667521	0.04550758	OK	5.47E-70
AC024901.1	1.84E-06	4.628852498	0.01174614	OK	4.01E-06
PDE3A	0	51.27297857	0.1317485	OK	0
SLCO1C1	5.61E-68	17.38233772	0.04432035	OK	2.32E-67
RECQL	1.01E-58	16.11428095	0.04136732	OK	4.01E-58
GOLT1B	2.85E-22	9.634714085	0.02470118	OK	8.64E-22
LDHB	0	122.950729	0.31614776	OK	0
KCNJ8	1.70E-86	19.67679548	0.05044635	OK	7.69E-86
ABCC9	2.43E-299	36.96090417	0.09489927	OK	1.76E-298
CMAS	6.84E-43	13.67834157	0.03510096	OK	2.47E-42
ST8SIA1	5.43E-15	7.728870472	0.01970732	OK	1.47E-14
ETNK1	3.57E-119	23.18067884	0.05953979	OK	1.80E-118
SOX5	4.35E-102	21.42002117	0.05496314	OK	2.08E-101
BCAT1	0	113.9972175	0.29308724	OK	0
LRMP	0	44.23665584	0.11364844	OK	0
ETFRF1	7.53E-91	20.1787223	0.05181928	OK	3.46E-90
KRAS	0	41.3064688	0.10616048	OK	0
RASSF8-AS1	3.04E-13	7.198777895	0.01841866	OK	7.98E-13
RASSF8	3.99E-197	29.93013513	0.07688339	OK	2.46E-196
BHLHE41	0	69.62965067	0.17898765	OK	0
SSPN	0	179.5883562	0.4617878	OK	0
AC022509.4	1.96E-06	4.615301394	0.01144593	OK	4.28E-06
ITPR2	0	74.53410148	0.19160438	OK	0
FGFR1OP2	4.48E-75	18.295721	0.04698024	OK	1.91E-74
TM7SF3	1.59E-27	10.80702502	0.02771457	OK	5.10E-27
MED21	2.03E-105	21.77438657	0.05591948	OK	9.84E-105
STK38L	2.40E-46	14.24521238	0.03656095	OK	8.86E-46
ARNTL2	5.64E-06	4.390977375	0.01118163	OK	1.20E-05
PPFIBP1	0	65.53657209	0.1684596	OK	0
MRPS35	1.09E-131	24.39015879	0.06265212	OK	5.72E-131

KLHL42	7.84E-138	24.96234954	0.06410194	OK	4.21E-137
PTHLH	1.45E-21	9.466486643	0.02414936	OK	4.34E-21
CCDC91	1.44E-57	15.94939877	0.04094335	OK	5.65E-57
FAR2	6.43E-76	18.40116637	0.04721397	OK	2.76E-75
ERGIC2	1.39E-156	26.63354009	0.06842272	OK	7.81E-156
TMTC1	1.87E-107	21.98803193	0.05642706	OK	9.17E-107
TSPAN11	9.00E-23	9.752539875	0.02372473	OK	2.74E-22
SINHCAF	0	39.08564639	0.10043658	OK	0
AMN1	1.93E-19	8.941141267	0.02290803	OK	5.59E-19
KIAA1551	2.41E-79	18.82373595	0.04833567	OK	1.06E-78
FGD4	3.00E-95	20.67345073	0.05308779	OK	1.41E-94
DNM1L	1.01E-05	4.261797742	0.01088578	OK	2.14E-05
PKP2	1.55E-66	17.19095505	0.04401866	OK	6.39E-66
CPNE8	2.49E-30	11.38467102	0.02920227	OK	8.15E-30
KIF21A	4.52E-14	7.454286877	0.01907181	OK	1.20E-13
CNTN1	2.25E-12	6.920557377	0.01764325	OK	5.80E-12
PDZRN4	3.65E-161	27.0260312	0.06935376	OK	2.08E-160
GXYLT1	4.22E-05	3.931487196	0.01003094	OK	8.67E-05
YAF2	2.77E-13	7.211335251	0.01846959	OK	7.28E-13
PPHLN1	2.26E-64	16.8998162	0.04338717	OK	9.19E-64
ZCRB1	0	38.79104292	0.09969201	OK	0
PRICKLE1	5.00E-07	4.891714351	0.01242546	OK	1.11E-06
PUS7L	0.00041084	3.345389508	0.0085281	OK	0.00080176
IRAK4	3.87E-08	5.373061022	0.01374147	OK	8.96E-08
TWF1	7.44E-39	12.98499719	0.033318	OK	2.60E-38
NELL2	4.81E-32	11.72376891	0.02953338	OK	1.60E-31
ANO6	3.58E-41	13.38749117	0.03435386	OK	1.28E-40
AC008124.1	1.08E-09	5.985719255	0.01530979	OK	2.61E-09
SCAF11	2.07E-128	24.07910298	0.06185567	OK	1.08E-127
SLC38A1	0	54.78562931	0.14073501	OK	0
SLC38A2	4.44E-25	10.2778149	0.0263594	OK	1.39E-24
AC008035.1	0.00049288	3.294562382	0.008348	OK	0.00095635
AMIGO2	6.09E-143	25.42859604	0.06528369	OK	3.31E-142
PCED1B	3.30E-06	4.506047878	0.01150177	OK	7.13E-06
RPAP3	7.03E-22	9.541686656	0.02446148	OK	2.12E-21
RAPGEF3	7.31E-05	3.797281169	0.00963042	OK	0.00014862
SLC48A1	3.02E-42	13.56997514	0.03481694	OK	1.09E-41
HDAC7	2.21E-06	4.590858627	0.0117259	OK	4.80E-06
VDR	2.88E-158	26.7784282	0.0687037	OK	1.63E-157
TMEM106C	0	97.71303247	0.2512121	OK	0
PFKM	4.34E-142	25.35136775	0.0650885	OK	2.35E-141
ASB8	3.54E-20	9.12631421	0.02339462	OK	1.04E-19
CCDC184	8.11E-05	3.77168801	0.00958409	OK	0.00016436
AC024257.3	0.00043825	3.327432447	0.00840587	OK	0.00085375
ADCY6	1.57E-99	21.1439631	0.05426476	OK	7.46E-99
RND1	3.23E-05	3.995324746	0.01011781	OK	6.67E-05
AC073610.3	3.45E-05	3.979564453	0.00999428	OK	7.12E-05
FKBP11	2.54E-18	8.651459022	0.022161	OK	7.27E-18
ARF3	2.00E-188	29.25402207	0.07516103	OK	1.21E-187
PRKAG1	3.19E-11	6.534490106	0.01672902	OK	8.02E-11
RHEBL1	2.17E-08	5.476068111	0.01396408	OK	5.07E-08
LMBR1L	1.34E-47	14.44554071	0.037066	OK	4.98E-47
TUBA1B	0	152.9768487	0.39335539	OK	0

TUBA1A	0	82.91467066	0.21317432	OK	0
TUBA1C	0	138.1123697	0.3551422	OK	0
AC010173.1	0.00012072	3.671166279	0.00930974	OK	0.00024263
PRPH	0	54.52305269	0.13975599	OK	0
TROAP	0	101.592284	0.25963561	OK	0
SPATS2	2.29E-06	4.583021784	0.01170677	OK	4.98E-06
MCRS1	1.74E-17	8.429384259	0.02160146	OK	4.90E-17
PRPF40B	1.12E-09	5.97903021	0.01527238	OK	2.72E-09
FMNL3	4.75E-48	14.51667093	0.03724304	OK	1.77E-47
TMBIM6	0	166.8468242	0.42902932	OK	0
NCKAP5L	7.06E-30	11.29360104	0.02896061	OK	2.30E-29
BCDIN3D	4.70E-05	3.905815752	0.00995681	OK	9.62E-05
RACGAP1	4.56E-76	18.41970947	0.04719319	OK	1.96E-75
SMARCD1	5.11E-16	8.024161271	0.02055895	OK	1.41E-15
COX14	0	58.15736468	0.14950283	OK	0
CERS5	0.00029123	3.439657203	0.00876947	OK	0.00057285
LIMA1	0	48.47354902	0.12458432	OK	0
LARP4	3.69E-20	9.121969395	0.02338354	OK	1.09E-19
DIP2B	7.69E-26	10.44536426	0.0267697	OK	2.42E-25
ATF1	1.38E-22	9.709022052	0.02489303	OK	4.20E-22
METTL7A	4.74E-148	25.88640167	0.06649744	OK	2.60E-147
SLC11A2	7.84E-130	24.21441118	0.06218533	OK	4.11E-129
LETMD1	6.95E-80	18.88954165	0.04849967	OK	3.05E-79
DAZAP2	0	40.82915922	0.10493547	OK	0
SMAGP	2.18E-18	8.668918368	0.02217228	OK	6.24E-18
BIN2	0	64.7657515	0.16647646	OK	0
GALNT6	8.84E-146	25.68391862	0.0659384	OK	4.83E-145
SLC4A8	0.00022036	3.514416812	0.00894037	OK	0.00043677
AC068987.5	4.46E-05	3.918312071	0.00984907	OK	9.14E-05
ACVRL1	2.21E-99	21.12780796	0.05420255	OK	1.05E-98
GRASP	0	139.0601263	0.35753111	OK	0
NR4A1	0	62.05738631	0.15953017	OK	0
ATG101	1.12E-150	26.1187961	0.06709637	OK	6.17E-150
KRT7	0	48.83732023	0.12533633	OK	0
KRT1	1.55E-10	6.293853831	0.01420419	OK	3.83E-10
KRT8	0	52.67263335	0.13532824	OK	0
KRT18	2.32E-201	30.25381388	0.07760125	OK	1.45E-200
EIF4B	0	63.77225576	0.16394379	OK	0
AC068888.1	0	55.3082555	0.1421273	OK	0
TNS2	1.81E-283	35.95934075	0.09237388	OK	1.28E-282
SPRYD3	2.58E-103	21.55126832	0.05533859	OK	1.24E-102
IGFBP6	0	177.7894029	0.45715417	OK	0
CSAD	1.11E-39	13.13002753	0.03368241	OK	3.91E-39
ITGB7	0	41.29065877	0.10603258	OK	0
RARG	6.40E-12	6.77090557	0.01731707	OK	1.64E-11
MFSD5	3.98E-19	8.860609028	0.02269909	OK	1.15E-18
ESPL1	8.49E-40	13.15019579	0.03298649	OK	3.00E-39
PFDN5	0	167.3773142	0.43036689	OK	0
AC073611.1	0	54.73166779	0.14058338	OK	0
C12orf10	2.01E-179	28.53738457	0.07331819	OK	1.19E-178
AAAS	0.00017067	3.58171518	0.00913281	OK	0.00034037
PRR13	0	109.0288392	0.28034208	OK	0
PCBP2	0	44.11157007	0.11337802	OK	0

MAP3K12	9.04E-08	5.218074985	0.0133429	OK	2.07E-07
TARBP2	1.77E-16	8.153149933	0.02087645	OK	4.92E-16
ATF7	3.63E-17	8.342872929	0.02137819	OK	1.02E-16
ATP5MC2	0	171.1737622	0.44015162	OK	0
CALCOCO1	3.79E-32	11.74393133	0.0301236	OK	1.26E-31
HOXC5	1.86E-07	5.082254631	0.01216054	OK	4.22E-07
AC023794.4	2.72E-10	6.206047389	0.01534421	OK	6.68E-10
SMUG1	8.39E-24	9.990496538	0.02561022	OK	2.59E-23
LINC02381	8.38E-94	20.5121929	0.05258327	OK	3.90E-93
CBX5	5.00E-166	27.43674852	0.07048448	OK	2.88E-165
HNRNPA1	0	104.342125	0.26828238	OK	0
NFE2	2.01E-19	8.936199095	0.02215014	OK	5.85E-19
COPZ1	0	39.46190255	0.10141722	OK	0
GPR84	0	38.07577439	0.09780969	OK	0
ZNF385A	0	109.17764	0.28069404	OK	0
ITGA5	1.24E-144	25.58118984	0.06569482	OK	6.73E-144
GTSF1	7.96E-16	7.969518182	0.02037552	OK	2.19E-15
NCKAP1L	0	108.6280327	0.27929154	OK	0
PPP1R1A	0	42.55765602	0.10924343	OK	0
TESPA1	2.47E-10	6.220736951	0.01583509	OK	6.09E-10
METTL7B	7.46E-58	15.99033566	0.04099101	OK	2.94E-57
ITGA7	0	57.7791894	0.14847678	OK	0
BLOC1S1	0	110.6075046	0.28440127	OK	0
RDH5	1.76E-12	6.955082777	0.01778537	OK	4.55E-12
CD63	0	220.9051518	0.56800458	OK	0
AC009779.2	6.84E-06	4.348879583	0.01109946	OK	1.46E-05
ORMDL2	0	59.50581778	0.15296597	OK	0
MMP19	0	153.949973	0.39581827	OK	0
PYM1	2.92E-147	25.81611269	0.0663147	OK	1.60E-146
DGKA	6.70E-10	6.062435884	0.01550869	OK	1.63E-09
RAB5B	6.59E-16	7.992810086	0.02047629	OK	1.81E-15
RPS26	0	173.1061781	0.44508873	OK	0
PA2G4	0	56.5790231	0.14544371	OK	0
RPL41	0	187.2835285	0.48143426	OK	0
ESYT1	4.89E-13	7.133687758	0.01826745	OK	1.28E-12
MYL6B	1.92E-186	29.09765215	0.07475489	OK	1.16E-185
MYL6	0	185.311995	0.47644236	OK	0
SMARCC2	2.22E-18	8.667117761	0.02221464	OK	6.34E-18
RNF41	1.48E-07	5.126192061	0.01310672	OK	3.36E-07
NABP2	1.76E-29	11.21316826	0.02875806	OK	5.71E-29
CS	3.09E-07	4.985344996	0.01274082	OK	6.94E-07
CNPY2	1.13E-139	25.13146623	0.06456066	OK	6.07E-139
IL23A	1.19E-07	5.167527136	0.01313244	OK	2.70E-07
STAT2	7.10E-12	6.755772812	0.01729822	OK	1.82E-11
TIMELESS	1.30E-82	19.21827669	0.04919004	OK	5.77E-82
SPRYD4	1.01E-07	5.197753386	0.01328541	OK	2.30E-07
RBMS2	9.49E-06	4.276561925	0.01091704	OK	2.01E-05
ATP5F1B	0	73.81909053	0.18978458	OK	0
PTGES3	0	92.42089289	0.23762432	OK	0
NACA	0	190.2063876	0.48905168	OK	0
PRIM1	4.38E-107	21.94945174	0.05633001	OK	2.14E-106
NAB2	4.30E-07	4.921216539	0.01257514	OK	9.60E-07
STAT6	3.02E-08	5.417654288	0.01385682	OK	7.01E-08

LRP1	0	52.8133623	0.13575553	OK	0
SHMT2	1.53E-56	15.80102291	0.04055347	OK	5.98E-56
NDUFA4L2	0	133.2610108	0.34255379	OK	0
STAC3	0	49.20359478	0.12643401	OK	0
R3HDM2	4.61E-22	9.585190348	0.0245772	OK	1.39E-21
ARHGAP9	0	45.49126451	0.11688916	OK	0
DDIT3	1.22E-126	23.90965847	0.06141571	OK	6.29E-126
DCTN2	0	46.56530613	0.11968657	OK	0
KIF5A	2.84E-05	4.025893169	0.01016626	OK	5.88E-05
PIP4K2C	2.19E-08	5.474652731	0.01398894	OK	5.11E-08
DTX3	3.01E-118	23.08871118	0.05925447	OK	1.52E-117
ARHGEF25	2.67E-298	36.89611727	0.0947597	OK	1.92E-297
OS9	0	39.60582907	0.10178866	OK	0
AGAP2-AS1	2.51E-19	8.911836282	0.02280807	OK	7.28E-19
TSPAN31	7.68E-37	12.62510145	0.03239217	OK	2.65E-36
CDK4	9.01E-80	18.87587259	0.0484698	OK	3.95E-79
MARCH9	2.56E-22	9.645931481	0.0247276	OK	7.75E-22
CYP27B1	4.55E-62	16.58403782	0.04227026	OK	1.83E-61
METTL1	4.22E-230	32.36531934	0.08312287	OK	2.77E-229
TSFM	8.11E-33	11.87364875	0.03045843	OK	2.72E-32
CTDSP2	1.01E-14	7.648767382	0.01959555	OK	2.74E-14
AC083805.1	1.30E-06	4.700190635	0.01129711	OK	2.85E-06
AC084033.3	5.87E-32	11.70702254	0.03002058	OK	1.95E-31
ATP23	1.94E-07	5.074596782	0.01296751	OK	4.39E-07
LRIG3	3.61E-71	17.79880736	0.04564376	OK	1.52E-70
SLC16A7	2.25E-10	6.235817269	0.01595488	OK	5.54E-10
USP15	0	38.84596187	0.09983363	OK	0
PPM1H	0.00033337	3.402902261	0.00862933	OK	0.00065383
AVPR1A	4.18E-198	30.00536133	0.07667674	OK	2.58E-197
SRGAP1	0	51.78168912	0.13307866	OK	0
TBK1	2.17E-06	4.594126858	0.01173911	OK	4.72E-06
RASSF3	2.89E-97	20.89642192	0.05366444	OK	1.36E-96
GNS	0	57.58000741	0.14800645	OK	0
TBC1D30	8.38E-12	6.7317508	0.01720839	OK	2.14E-11
MSRB3	0	177.2720503	0.45582652	OK	0
LLPH	1.24E-117	23.02749141	0.05914662	OK	6.23E-117
TMBIM4	0	97.79127019	0.25143791	OK	0
IRAK3	5.22E-193	29.61207624	0.07607218	OK	3.20E-192
HELB	4.50E-07	4.912324379	0.01254731	OK	1.00E-06
GRIP1	1.29E-17	8.464044772	0.02150276	OK	3.65E-17
DYRK2	2.21E-05	4.08436843	0.01042562	OK	4.60E-05
IFNG	7.17E-14	7.393175547	0.01885438	OK	1.90E-13
RAP1B	0	60.15112081	0.15463101	OK	0
SLC35E3	4.73E-08	5.336654343	0.01364835	OK	1.09E-07
MDM2	4.71E-11	6.476114775	0.01658007	OK	1.18E-10
CPM	0	129.0464077	0.33179409	OK	0
CPSF6	4.78E-18	8.57911804	0.02198616	OK	1.36E-17
LYZ	0	298.5662817	0.76781719	OK	0
AC020656.1	0	133.1242653	0.34227663	OK	0
YEATS4	8.61E-83	19.23971957	0.04939723	OK	3.82E-82
FRS2	2.65E-08	5.441071279	0.01391029	OK	6.17E-08
CCT2	0	47.99786333	0.1233726	OK	0
MYRFL	3.10E-16	8.085181679	0.02063169	OK	8.59E-16

CNOT2	3.39E-22	9.617027655	0.02465853	OK	1.03E-21
KCNMB4	3.73E-28	10.93962503	0.02803575	OK	1.20E-27
PTPRB	6.62E-307	37.42889586	0.09510816	OK	4.82E-306
PTPRR	5.72E-05	3.857680431	0.00898691	OK	0.00011681
TSPAN8	5.07E-24	10.04020552	0.02565447	OK	1.57E-23
THAP2	4.18E-06	4.455535968	0.01137979	OK	8.98E-06
TMEM19	2.83E-108	22.07362135	0.05668041	OK	1.39E-107
RAB21	7.11E-74	18.14441532	0.0465906	OK	3.02E-73
TBC1D15	3.80E-18	8.605466558	0.02205557	OK	1.08E-17
ATXN7L3B	4.80E-49	14.67301037	0.03766023	OK	1.80E-48
CAPS2	7.61E-06	4.325488806	0.01101875	OK	1.62E-05
GLIPR1	0	88.7577254	0.22820564	OK	0
KRR1	1.20E-106	21.9037085	0.05625712	OK	5.83E-106
PHLDA1	0	163.8020316	0.42119673	OK	0
NAP1L1	0	65.83491893	0.16924844	OK	0
OSBPL8	0	41.63991318	0.10702006	OK	0
CSRP2	0	211.1410007	0.54292321	OK	0
E2F7	0	39.31492596	0.099319	OK	0
NAV3	1.14E-05	4.234703943	0.01070343	OK	2.41E-05
SYT1	4.17E-06	4.456247822	0.01132426	OK	8.95E-06
PAWR	0	126.2597996	0.32462351	OK	0
PPP1R12A	0	72.26636642	0.18578874	OK	0
PTPRQ	0.00016056	3.597635909	0.00882134	OK	0.00032073
LIN7A	1.65E-08	5.524473558	0.01410698	OK	3.87E-08
ACSS3	2.88E-10	6.196749725	0.01581839	OK	7.08E-10
PPFIA2	2.09E-31	11.59873725	0.02970501	OK	6.91E-31
CCDC59	1.34E-59	16.23895548	0.04168932	OK	5.33E-59
METTL25	0.00030097	3.430735649	0.00874345	OK	0.00059145
RASSF9	2.00E-98	21.02339079	0.05390958	OK	9.49E-98
MGAT4C	1.16E-08	5.58662042	0.01400211	OK	2.72E-08
C12orf29	4.66E-95	20.65226403	0.05302532	OK	2.18E-94
CEP290	0.00012521	3.661835092	0.00934062	OK	0.00025148
TMTC3	3.49E-09	5.791179281	0.01480793	OK	8.34E-09
KITLG	1.46E-42	13.62327105	0.03491174	OK	5.25E-42
DUSP6	0	67.28323696	0.17295959	OK	0
AC010201.2	0.00022016	3.514653248	0.00850659	OK	0.00043642
POC1B	0.00032721	3.407994651	0.00868381	OK	0.00064197
ATP2B1	0	143.3979021	0.36873266	OK	0
ATP2B1-AS1	0	66.07760883	0.16985415	OK	0
LUM	0	110.1786858	0.2831512	OK	0
DCN	0	115.7540094	0.29747436	OK	0
BTG1	0	146.1656206	0.37584609	OK	0
AC025164.1	1.90E-143	25.4742129	0.06542217	OK	1.04E-142
CLLU1OS	2.12E-101	21.34606723	0.05465154	OK	1.01E-100
LINC02397	2.31E-05	4.074544029	0.01032767	OK	4.79E-05
EEA1	2.02E-151	26.18410032	0.06726871	OK	1.12E-150
NUDT4	2.79E-169	27.7081319	0.07118039	OK	1.62E-168
UBE2N	0	46.23538136	0.11883954	OK	0
MRPL42	3.72E-77	18.55486362	0.04764326	OK	1.61E-76
SOCS2	1.01E-108	22.12014341	0.05678654	OK	4.96E-108
CRADD	1.12E-111	22.42527169	0.05756298	OK	5.53E-111
AC012085.2	9.19E-34	12.05442713	0.03075655	OK	3.10E-33
PLXNC1	0	41.40657127	0.10638388	OK	0

TMCC3	7.14E-39	12.98822045	0.0332985	OK	2.50E-38
NDUFA12	0	67.40235851	0.17328082	OK	0
FGD6	7.85E-24	9.997104615	0.02561619	OK	2.42E-23
VEZT	4.57E-06	4.436512853	0.01133415	OK	9.79E-06
METAP2	8.47E-147	25.77491832	0.06621634	OK	4.64E-146
USP44	0.00050479	3.287841113	0.00833391	OK	0.00097879
NTN4	0	50.36266769	0.12938143	OK	0
SNRPF	0	51.20491297	0.13162101	OK	0
AMDHD1	1.23E-09	5.963515601	0.01518642	OK	2.98E-09
LTA4H	0	39.75238365	0.10215957	OK	0
ELK3	5.56E-92	20.30709736	0.05214222	OK	2.57E-91
TMPO-AS1	1.17E-41	13.47040882	0.03445497	OK	4.19E-41
TMPO	1.07E-266	34.86976963	0.08959903	OK	7.39E-266
SLC25A3	0	130.052319	0.33440241	OK	0
IKBIP	0	50.6307359	0.13013834	OK	0
APAF1	1.22E-188	29.27069513	0.07518439	OK	7.42E-188
ANKS1B	4.52E-10	6.125468768	0.01561223	OK	1.11E-09
UHRF1BP1L	1.68E-05	4.148206734	0.01059208	OK	3.51E-05
ACTR6	2.76E-54	15.47008055	0.03970877	OK	1.07E-53
SCYL2	2.20E-05	4.085599669	0.0104297	OK	4.58E-05
GAS2L3	0	51.88545254	0.13333483	OK	0
ANO4	1.82E-08	5.507503736	0.01359575	OK	4.25E-08
ARL1	0	40.57510857	0.10427869	OK	0
SPIC	3.34E-39	13.04624151	0.03322079	OK	1.17E-38
CHPT1	2.23E-91	20.23872486	0.05197516	OK	1.03E-90
GNPTAB	7.28E-91	20.18040981	0.05181994	OK	3.34E-90
DRAM1	0	61.67027105	0.1585202	OK	0
WASHC3	8.14E-159	26.82551039	0.06891685	OK	4.60E-158
NUP37	3.18E-21	9.38373852	0.02405072	OK	9.50E-21
PARPBP	2.15E-164	27.29942679	0.06996814	OK	1.23E-163
IGF1	0	134.9939729	0.3470262	OK	0
NT5DC3	3.19E-11	6.534641239	0.01670493	OK	8.02E-11
HSP90B1	0	105.4049956	0.27101074	OK	0
C12orf73	0.00012858	3.655014055	0.00931997	OK	0.00025805
TDG	2.13E-128	24.0779814	0.06184699	OK	1.11E-127
GLT8D2	0	73.15026545	0.18800532	OK	0
HCFC2	6.96E-15	7.697067381	0.01971195	OK	1.88E-14
NFYB	2.64E-99	21.11939725	0.05423291	OK	1.25E-98
TXNRD1	3.78E-78	18.67742243	0.04796081	OK	1.65E-77
CHST11	0	39.8142862	0.10230784	OK	0
SLC41A2	1.51E-61	16.51179298	0.04237112	OK	6.07E-61
C12orf45	7.06E-206	30.5953276	0.0786066	OK	4.44E-205
ALDH1L2	2.13E-13	7.246972906	0.01851799	OK	5.61E-13
WASHC4	5.14E-149	25.97190521	0.0667171	OK	2.83E-148
APPL2	1.23E-35	12.40463234	0.03182019	OK	4.22E-35
C12orf75	0	252.096531	0.64828293	OK	0
NUAK1	6.21E-42	13.51694692	0.03465025	OK	2.23E-41
CKAP4	6.66E-134	24.59786081	0.06317174	OK	3.53E-133
RIC8B	7.87E-06	4.318198075	0.01101302	OK	1.67E-05
AC007541.1	9.76E-05	3.725149614	0.00947868	OK	0.00019703
TMEM263	2.35E-112	22.49454653	0.05776946	OK	1.17E-111
MTERF2	4.20E-05	3.932756044	0.01002926	OK	8.62E-05
PWP1	1.51E-70	17.71838968	0.04549333	OK	6.33E-70

CMKLR1	2.42E-226	32.09705573	0.08243137	OK	1.58E-225
ISCU	0	116.1159918	0.29856854	OK	0
TMEM119	7.60E-06	4.325865796	0.01099121	OK	1.61E-05
SELPLG	0	47.43759886	0.1218926	OK	0
CORO1C	0	37.53240443	0.09644883	OK	0
SSH1	0.00010078	3.717059051	0.00948089	OK	0.00020339
USP30	1.24E-05	4.216542174	0.01074497	OK	2.61E-05
USP30-AS1	1.06E-11	6.697225007	0.01708778	OK	2.70E-11
ALKBH2	1.65E-12	6.964224935	0.01782943	OK	4.27E-12
UNG	5.21E-08	5.319381722	0.01359376	OK	1.20E-07
ACACB	1.61E-21	9.455270576	0.02419266	OK	4.83E-21
KCTD10	3.56E-64	16.87295716	0.04330578	OK	1.45E-63
UBE3B	0.00044332	3.324227203	0.00846881	OK	0.00086324
MMAB	9.29E-24	9.980309079	0.02558565	OK	2.87E-23
TRPV4	6.30E-29	11.09966824	0.02839802	OK	2.04E-28
GLTP	6.16E-97	20.86022108	0.05357348	OK	2.90E-96
TCHP	4.91E-07	4.895089623	0.01251047	OK	1.09E-06
GIT2	1.51E-69	17.58843057	0.04515122	OK	6.30E-69
ANKRD13A	2.02E-06	4.609539126	0.01177745	OK	4.39E-06
C12orf76	8.25E-25	10.21781237	0.02619976	OK	2.57E-24
IFT81	5.46E-80	18.90232847	0.04851455	OK	2.40E-79
ATP2A2	2.09E-24	10.12729672	0.02597017	OK	6.49E-24
ARPC3	0	236.1978886	0.60739343	OK	0
GPN3	4.14E-172	27.94184741	0.07178709	OK	2.42E-171
VPS29	0	87.79089644	0.22571924	OK	0
PPTC7	2.38E-21	9.414461714	0.02412954	OK	7.11E-21
TCTN1	1.90E-11	6.611378833	0.01691781	OK	4.81E-11
HVCN1	0	39.30072674	0.10098202	OK	0
PPP1CC	7.22E-97	20.85258403	0.05355544	OK	3.40E-96
PHETA1	2.06E-07	5.063188861	0.01293086	OK	4.66E-07
SH2B3	0	46.62445528	0.11981364	OK	0
BRAP	1.56E-05	4.164143177	0.01062944	OK	3.28E-05
ALDH2	0	100.374023	0.25808168	OK	0
MAPKAPK5-AS1	7.87E-22	9.529919929	0.02443177	OK	2.37E-21
ERP29	0	81.12375384	0.20857171	OK	0
TRAFD1	1.54E-06	4.664978911	0.01191579	OK	3.37E-06
HECTD4	4.27E-06	4.45108394	0.01136641	OK	9.16E-06
RPL6	0	177.018476	0.45511012	OK	0
PTPN11	2.57E-84	19.42083093	0.04987108	OK	1.15E-83
OAS1	0	92.48596599	0.23777424	OK	0
OAS3	4.25E-30	11.33799214	0.02906061	OK	1.39E-29
OAS2	1.44E-77	18.60568719	0.04775168	OK	6.26E-77
DTX1	0.00022599	3.507713858	0.00882979	OK	0.00044757
RASAL1	4.88E-14	7.444013782	0.0170624	OK	1.30E-13
DDX54	2.85E-10	6.1984692	0.01586433	OK	7.01E-10
RITA1	7.77E-06	4.320833799	0.01103067	OK	1.65E-05
TPCN1	4.11E-07	4.930000038	0.01260066	OK	9.19E-07
SLC8B1	3.07E-86	19.64698022	0.05044189	OK	1.38E-85
PLBD2	3.18E-36	12.51274796	0.03209772	OK	1.09E-35
SDS	0	93.43421821	0.24019261	OK	0
SDSL	0	62.68543792	0.16112362	OK	0
AC026765.2	1.04E-22	9.737671921	0.01967829	OK	3.17E-22
MAP1LC3B2	9.26E-06	4.28192872	0.01092079	OK	1.96E-05

C12orf49	5.66E-126	23.84536599	0.06123893	OK	2.92E-125
HRK	3.98E-09	5.76938349	0.01449988	OK	9.48E-09
TESC	1.50E-290	36.4095598	0.09351701	OK	1.07E-289
FBXO21	5.52E-50	14.81906658	0.03803108	OK	2.08E-49
RFC5	5.71E-18	8.55866147	0.02191175	OK	1.62E-17
WSB2	5.72E-83	19.26087811	0.04945606	OK	2.55E-82
VSIG10	3.60E-05	3.969618369	0.01012611	OK	7.42E-05
PEBP1	0	169.4582886	0.43575814	OK	0
TAOK3	0	49.00318221	0.12595589	OK	0
SUDS3	1.88E-41	13.43509351	0.03447733	OK	6.74E-41
SRRM4	1.66E-08	5.523420491	0.01403331	OK	3.89E-08
HSPB8	0	70.81582075	0.18198474	OK	0
TMEM233	7.91E-43	13.66775146	0.03493888	OK	2.86E-42
CIT	6.08E-87	19.72897742	0.05044277	OK	2.75E-86
BICDL1	2.16E-13	7.245256849	0.01845211	OK	5.68E-13
RAB35	6.68E-36	12.45367156	0.03194648	OK	2.29E-35
RPLP0	0	177.6473272	0.45677932	OK	0
PXN	6.50E-22	9.549830536	0.02446837	OK	1.96E-21
COX6A1	0	157.0531286	0.40384203	OK	0
TRIAP1	1.13E-42	13.64146303	0.03500533	OK	4.09E-42
SRSF9	0	107.5221037	0.27646651	OK	0
DYNLL1	0	137.4885154	0.35352096	OK	0
COQ5	1.16E-19	8.996561615	0.02305345	OK	3.39E-19
RNF10	0.00020019	3.5398348	0.00902884	OK	0.00039772
POP5	3.57E-59	16.17861164	0.04153153	OK	1.42E-58
CABP1	5.31E-72	17.90578568	0.04575553	OK	2.24E-71
MLEC	0	49.77228483	0.12793681	OK	0
ACADS	6.85E-44	13.84466754	0.0355205	OK	2.49E-43
SPPL3	2.80E-05	4.028816344	0.01028544	OK	5.80E-05
C12orf43	5.92E-10	6.082431234	0.01556434	OK	1.44E-09
OASL	0	55.6055259	0.14288428	OK	0
P2RX7	0	42.87842923	0.11017556	OK	0
P2RX4	0	82.43440392	0.21191837	OK	0
CAMKK2	2.45E-07	5.029943415	0.01285848	OK	5.53E-07
ANAPC5	2.50E-18	8.653403703	0.02218039	OK	7.15E-18
RNF34	4.93E-11	6.468988681	0.01655666	OK	1.23E-10
KDM2B	2.91E-41	13.40277291	0.03437387	OK	1.04E-40
ORAI1	0	48.52740126	0.12471742	OK	0
TMEM120B	6.70E-39	12.99303118	0.03330263	OK	2.35E-38
RHOF	0	39.41807463	0.10124558	OK	0
HPD	1.81E-11	6.619235274	0.01684596	OK	4.56E-11
PSMD9	2.27E-13	7.238302472	0.01853828	OK	5.98E-13
AC069503.1	1.24E-05	4.216672215	0.009881	OK	2.61E-05
BCL7A	1.48E-15	7.892384627	0.02019769	OK	4.05E-15
MLXIP	3.64E-17	8.342259507	0.02137579	OK	1.02E-16
CLIP1	1.98E-07	5.070927557	0.01296727	OK	4.47E-07
ZCCHC8	4.05E-05	3.941254456	0.01005888	OK	8.33E-05
RSRC2	5.02E-81	19.02782038	0.04886419	OK	2.21E-80
KNTC1	2.33E-07	5.039448775	0.01284772	OK	5.27E-07
AC026333.4	9.93E-08	5.200720407	0.01120536	OK	2.27E-07
HCAR2	0	43.39433757	0.11145834	OK	0
HCAR3	7.41E-190	29.36626872	0.07538836	OK	4.50E-189
DENR	3.43E-65	17.01056857	0.04367351	OK	1.40E-64

HIP1R	2.30E-06	4.581939841	0.01165923	OK	5.00E-06
VPS37B	5.43E-70	17.64631173	0.04529832	OK	2.27E-69
ARL6IP4	0	84.51846256	0.21729965	OK	0
MPHOSPH9	3.35E-08	5.39904032	0.01379486	OK	7.77E-08
C12orf65	1.27E-118	23.12579524	0.05938944	OK	6.44E-118
CDK2AP1	6.20E-09	5.694142906	0.01455497	OK	1.47E-08
SBNO1	6.74E-11	6.42158643	0.01643972	OK	1.68E-10
KMT5A	4.76E-31	11.5280643	0.02957248	OK	1.57E-30
RILPL2	0	76.0191815	0.19544187	OK	0
SNRNP35	2.86E-57	15.9063634	0.04083108	OK	1.12E-56
RILPL1	1.43E-211	31.0205221	0.07966716	OK	9.07E-211
TMED2	2.33E-259	34.38217049	0.08835394	OK	1.59E-258
DDX55	5.65E-10	6.089848469	0.01558017	OK	1.38E-09
EIF2B1	3.34E-15	7.790268362	0.01995833	OK	9.09E-15
GTF2H3	1.17E-20	9.245114099	0.02369745	OK	3.48E-20
ATP6V0A2	6.13E-06	4.372790308	0.01116329	OK	1.31E-05
CCDC92	1.20E-36	12.58958173	0.03229696	OK	4.15E-36
NCOR2	4.02E-17	8.330722761	0.02134789	OK	1.13E-16
SCARB1	1.46E-168	27.64827665	0.07096672	OK	8.47E-168
UBC	0	139.2102969	0.35790246	OK	0
BRI3BP	0	39.22414802	0.10078052	OK	0
SLC15A4	2.43E-07	5.032028378	0.01286419	OK	5.47E-07
GLT1D1	1.40E-33	12.0195501	0.03065706	OK	4.73E-33
STX2	4.36E-17	8.32100393	0.02132302	OK	1.22E-16
RAN	0	115.2117122	0.29624082	OK	0
ADGRD1	4.68E-39	13.02043098	0.03313657	OK	1.64E-38
PUS1	2.12E-08	5.480534287	0.0140066	OK	4.95E-08
NOC4L	2.82E-06	4.53927615	0.01159446	OK	6.10E-06
PXMP2	0	39.86213537	0.10241177	OK	0
PGAM5	7.90E-11	6.397380231	0.01636261	OK	1.97E-10
ANKLE2	2.85E-25	10.32024866	0.02646519	OK	8.93E-25
GOLGA3	2.56E-09	5.843180451	0.01495103	OK	6.13E-09
CHFR	1.78E-16	8.152963487	0.02087982	OK	4.93E-16
ZNF891	8.65E-06	4.297173523	0.01096183	OK	1.83E-05
MPHOSPH8	1.41E-168	27.64964481	0.07103865	OK	8.16E-168
PSPC1	1.28E-11	6.669619509	0.01707414	OK	3.26E-11
ZMYM5	9.96E-09	5.61276171	0.0143583	OK	2.35E-08
ZMYM2	1.06E-10	6.35190449	0.01626052	OK	2.64E-10
GJB2	0.00023415	3.498267388	0.00877139	OK	0.00046331
CRYL1	0	46.31737651	0.11904542	OK	0
IFT88	0.00037986	3.367070818	0.0085822	OK	0.00074288
AL161772.1	2.00E-13	7.255842019	0.0185161	OK	5.26E-13
IL17D	3.22E-08	5.406394435	0.01375128	OK	7.46E-08
EEF1AKMT1	3.64E-19	8.870550296	0.02272888	OK	1.05E-18
LATS2	3.03E-06	4.524414627	0.01155791	OK	6.55E-06
SAP18	0	111.4545526	0.28657486	OK	0
SKA3	0	98.03677822	0.25022757	OK	0
MRPL57	0	52.83849323	0.13582313	OK	0
LINC00539	2.38E-05	4.067542106	0.01030221	OK	4.94E-05
ZDHHC20	1.86E-43	13.77255724	0.03533727	OK	6.75E-43
MICU2	3.31E-29	11.15692188	0.02861593	OK	1.07E-28
FGF9	1.68E-05	4.147196952	0.01032904	OK	3.52E-05
AL136962.1	6.59E-11	6.425031091	0.01634749	OK	1.65E-10

SGCG	4.55E-47	14.36090902	0.03669854	OK	1.69E-46
SACS	2.32E-10	6.23078616	0.01594189	OK	5.72E-10
SPATA13	2.69E-09	5.834681811	0.01491799	OK	6.45E-09
PARP4	3.12E-59	16.18691548	0.04154687	OK	1.24E-58
CENPJ	3.25E-08	5.404711585	0.01381405	OK	7.53E-08
MTMR6	1.39E-71	17.85206101	0.04583309	OK	5.86E-71
NUP58	5.20E-47	14.35169347	0.03682787	OK	1.93E-46
RNF6	1.50E-10	6.298535943	0.01611952	OK	3.72E-10
WASF3	8.17E-43	13.66542538	0.03501265	OK	2.95E-42
USP12	4.39E-49	14.67908015	0.03767496	OK	1.65E-48
RPL21	0	164.4911874	0.42291493	OK	0
RASL11A	0	74.11354915	0.19049177	OK	0
GTF3A	0	80.74993471	0.2076099	OK	0
MTIF3	1.82E-162	27.13670986	0.06971828	OK	1.03E-161
POLR1D	0	44.19020789	0.11358011	OK	0
FLT3	0	118.5266788	0.30396439	OK	0
PAN3	1.89E-08	5.501089556	0.01407002	OK	4.41E-08
FLT1	0	66.70485904	0.1713958	OK	0
POMP	0	138.0912324	0.3550846	OK	0
SLC46A3	8.52E-208	30.73920128	0.07895492	OK	5.38E-207
MTUS2	5.93E-62	16.56801452	0.04243041	OK	2.39E-61
SLC7A1	3.78E-13	7.16901306	0.01833668	OK	9.90E-13
UBL3	1.41E-120	23.31950477	0.05989715	OK	7.14E-120
LINC00544	5.29E-08	5.3165722	0.0129268	OK	1.22E-07
HMGB1	0	131.0472572	0.33693095	OK	0
USPL1	1.23E-08	5.57670393	0.01425907	OK	2.88E-08
ALOX5AP	0	128.4826595	0.33035806	OK	0
MEDAG	4.70E-102	21.41638233	0.05494072	OK	2.25E-101
HSPH1	0	69.56758739	0.17883886	OK	0
FRY	5.85E-159	26.83782759	0.06891411	OK	3.31E-158
BRCA2	2.33E-292	36.52375715	0.09381151	OK	1.66E-291
N4BP2L1	5.96E-54	15.42054126	0.03957782	OK	2.29E-53
N4BP2L2	4.29E-43	13.71222112	0.03519267	OK	1.55E-42
PDS5B	3.18E-05	3.999039532	0.01020922	OK	6.57E-05
STARD13	4.34E-09	5.754826539	0.01471485	OK	1.03E-08
RFC3	1.55E-68	17.45597863	0.04474663	OK	6.44E-68
NBEA	2.63E-05	4.043699553	0.01030046	OK	5.46E-05
DCLK1	1.91E-129	24.17774135	0.06186459	OK	9.98E-129
SPART	2.07E-92	20.35566441	0.0522759	OK	9.59E-92
SMAD9	0	64.07119814	0.16464908	OK	0
ALG5	4.05E-146	25.7142721	0.06605641	OK	2.21E-145
EXOSC8	1.03E-83	19.34961374	0.04968817	OK	4.58E-83
POSTN	0	57.09879567	0.14666793	OK	0
TRPC4	3.73E-08	5.379580759	0.01369049	OK	8.64E-08
UFM1	1.29E-192	29.5815586	0.07600593	OK	7.89E-192
NHLRC3	2.29E-16	8.122413327	0.02081095	OK	6.34E-16
LHFPL6	0	211.6557454	0.54426826	OK	0
COG6	0.00021287	3.523591132	0.00897985	OK	0.00042223
FOXO1	5.02E-06	4.416332415	0.0112796	OK	1.07E-05
MRPS31	4.38E-13	7.148719755	0.01830894	OK	1.15E-12
SLC25A15	0.00045763	3.315357577	0.00836929	OK	0.00088992
ELF1	0	128.9518335	0.3315802	OK	0
WBP4	1.41E-45	14.12077879	0.03624124	OK	5.19E-45

KBTBD6	1.66E-07	5.104055126	0.01301937	OK	3.77E-07
AL354696.2	5.38E-06	4.401285912	0.01102772	OK	1.15E-05
KBTBD7	1.91E-07	5.077589032	0.01296698	OK	4.32E-07
RGCC	0	216.5669063	0.55691396	OK	0
DGKH	0.00012004	3.672627559	0.00936517	OK	0.00024129
EPSTI1	0	100.9674253	0.25958876	OK	0
DNAJC15	0	75.60861778	0.19438649	OK	0
ENOX1	2.26E-32	11.78757328	0.02904009	OK	7.55E-32
CCDC122	1.77E-15	7.870567891	0.02013489	OK	4.82E-15
LACC1	1.95E-284	36.02120833	0.09254017	OK	1.38E-283
SERP2	1.67E-114	22.71283212	0.05826441	OK	8.34E-114
TSC22D1	0	131.1418261	0.33720547	OK	0
NUFIP1	0.00012626	3.659682413	0.00933429	OK	0.00025355
GTF2F2	1.76E-50	14.89574947	0.03823241	OK	6.64E-50
TPT1	0	159.5715427	0.41019697	OK	0
ZC3H13	5.64E-141	25.25016359	0.06486641	OK	3.05E-140
LCP1	0	253.7192628	0.6524776	OK	0
RUBCNL	1.37E-184	28.95071646	0.07433064	OK	8.24E-184
ESD	0	96.73829257	0.24873046	OK	0
SUCLA2	5.10E-108	22.04705153	0.056623	OK	2.50E-107
NUDT15	1.04E-18	8.752547974	0.02242798	OK	3.00E-18
MED4	1.59E-94	20.59286205	0.05288663	OK	7.42E-94
ITM2B	0	231.7341979	0.59587729	OK	0
RB1	0	120.2524454	0.30919594	OK	0
LPAR6	0	130.5373948	0.33563365	OK	0
RCBTB2	8.94E-101	21.27868825	0.05464107	OK	4.27E-100
CYSLTR2	3.37E-28	10.94869568	0.02802537	OK	1.08E-27
AL161421.1	6.17E-14	7.413001808	0.01897117	OK	1.64E-13
FNDC3A	1.44E-41	13.45511786	0.03452964	OK	5.15E-41
CAB39L	4.03E-73	18.04874841	0.04631795	OK	1.71E-72
SETDB2	3.60E-61	16.45929552	0.04224251	OK	1.44E-60
PHF11	3.18E-49	14.70086838	0.03773225	OK	1.20E-48
ARL11	2.09E-219	31.59606423	0.08115204	OK	1.35E-218
EBPL	1.59E-164	27.31047907	0.07015952	OK	9.12E-164
KPNA3	2.64E-41	13.40997292	0.03441171	OK	9.44E-41
SPRYD7	2.09E-26	10.56834542	0.02710128	OK	6.60E-26
DLEU2	9.99E-08	5.199598508	0.01329438	OK	2.28E-07
TRIM13	4.96E-05	3.892680106	0.00993566	OK	0.00010143
DLEU7	6.77E-32	11.69491043	0.02994784	OK	2.25E-31
RNASEH2B	1.81E-242	33.23274213	0.085394	OK	1.21E-241
INTS6	1.41E-31	11.6326108	0.029841	OK	4.66E-31
WDFY2	1.99E-07	5.07025879	0.0129623	OK	4.49E-07
DHRS12	5.51E-09	5.714114776	0.01461602	OK	1.31E-08
NEK3	3.57E-37	12.68533341	0.03253987	OK	1.23E-36
THSD1	0.00021066	3.526360518	0.0088107	OK	0.00041795
VPS36	8.00E-29	11.07821155	0.02841676	OK	2.59E-28
AL359513.1	9.75E-05	3.725430208	0.00938152	OK	0.00019683
CKAP2	0	45.0349619	0.11571757	OK	0
SUGT1	9.06E-292	36.48656654	0.09376623	OK	6.47E-291
OLFM4	2.49E-08	5.45205325	0.01381402	OK	5.80E-08
PCDH17	1.62E-71	17.84374073	0.04532026	OK	6.80E-71
DIAPH3	0	114.2622145	0.29227367	OK	0
TDRD3	1.54E-28	11.01950128	0.02825794	OK	4.97E-28

PCDH9	3.55E-46	14.21791102	0.03644117	OK	1.31E-45
DACH1	0.00021793	3.517360216	0.00889315	OK	0.00043204
MZT1	2.19E-91	20.23963366	0.05197442	OK	1.01E-90
DIS3	1.09E-06	4.735135532	0.01210245	OK	2.40E-06
PIBF1	2.22E-06	4.589333426	0.0117256	OK	4.83E-06
KLF5	9.43E-07	4.765278861	0.01214981	OK	2.08E-06
KLF12	5.40E-07	4.876635597	0.01245058	OK	1.20E-06
TBC1D4	1.86E-08	5.503417505	0.01406794	OK	4.35E-08
COMMD6	0	115.6489786	0.29736115	OK	0
UCHL3	0	56.94140006	0.14636661	OK	0
LMO7-AS1	3.01E-05	4.011867445	0.01015345	OK	6.23E-05
LMO7	0	110.4713193	0.28399934	OK	0
KCTD12	0	100.0700303	0.25728261	OK	0
CLN5	8.69E-28	10.86261152	0.02785993	OK	2.78E-27
FBXL3	2.63E-35	12.34391817	0.03166682	OK	8.97E-35
MYCBP2	2.10E-32	11.79368614	0.03025764	OK	7.03E-32
SLAIN1	3.05E-06	4.522895107	0.01151035	OK	6.59E-06
EDNRB	6.92E-129	24.12446787	0.06191703	OK	3.62E-128
RNF219	7.71E-08	5.247548881	0.01341638	OK	1.77E-07
NDFIP2	3.66E-18	8.609752788	0.02203823	OK	1.04E-17
SPRY2	9.15E-53	15.24310362	0.03911401	OK	3.49E-52
GPC6	0	42.53571193	0.1092744	OK	0
TGDS	9.87E-06	4.267709697	0.01089533	OK	2.09E-05
ABCC4	2.48E-12	6.906515112	0.01764636	OK	6.40E-12
DZIP1	1.66E-26	10.58979715	0.02708952	OK	5.26E-26
DNAJC3	2.02E-266	34.85155697	0.0895614	OK	1.39E-265
UGGT2	4.15E-11	6.495146957	0.01662316	OK	1.04E-10
MBNL2	1.43E-154	26.45917227	0.06796872	OK	8.02E-154
RAP2A	2.64E-124	23.68393035	0.06083103	OK	1.36E-123
IPO5	8.76E-18	8.509120769	0.02180513	OK	2.49E-17
FARP1	1.30E-201	30.27292743	0.07776569	OK	8.11E-201
STK24	3.87E-21	9.363054768	0.02400346	OK	1.15E-20
DOCK9	1.79E-22	9.682590019	0.02473638	OK	5.43E-22
UBAC2	0	45.48150996	0.1168933	OK	0
GPR18	1.67E-79	18.84314437	0.04824108	OK	7.33E-79
GPR183	0	241.8076303	0.62183283	OK	0
TM9SF2	5.60E-243	33.26803566	0.08548698	OK	3.75E-242
GGACT	1.65E-21	9.452545052	0.02420619	OK	4.95E-21
NALCN	5.35E-15	7.730730975	0.01969486	OK	1.45E-14
ITGBL1	0	151.4376853	0.38936023	OK	0
FGF14	2.44E-09	5.851100559	0.01487026	OK	5.85E-09
FGF14-AS2	1.18E-13	7.326454144	0.0187216	OK	3.13E-13
TPP2	5.25E-17	8.299078346	0.02126593	OK	1.47E-16
TEX30	4.24E-40	13.20251849	0.0338657	OK	1.50E-39
EFNB2	6.41E-93	20.41300668	0.05238313	OK	2.98E-92
ARGLU1	1.95E-09	5.888691981	0.01507132	OK	4.68E-09
LIG4	9.89E-08	5.201448417	0.01329252	OK	2.26E-07
ABHD13	9.37E-06	4.279511233	0.01092925	OK	1.98E-05
TNFSF13B	0	187.1169631	0.48116394	OK	0
IRS2	1.77E-91	20.25027379	0.05199897	OK	8.15E-91
AL355974.2	1.26E-78	18.73585915	0.04802392	OK	5.51E-78
COL4A1	0	81.67678168	0.20994413	OK	0
COL4A2	0	104.3007876	0.26814337	OK	0

RAB20	0	120.8841913	0.31082476	OK	0
NAXD	1.65E-05	4.15116571	0.01059445	OK	3.47E-05
CARS2	1.04E-19	9.009100727	0.02309008	OK	3.03E-19
ING1	8.29E-161	26.99573704	0.06934956	OK	4.70E-160
LINC00346	1.04E-06	4.745502902	0.01211066	OK	2.29E-06
ANKRD10	3.88E-60	16.3147498	0.0418842	OK	1.55E-59
AL442128.2	7.43E-06	4.330669208	0.01103099	OK	1.58E-05
ARHGEF7-AS2	4.45E-05	3.918675583	0.00989824	OK	9.13E-05
ARHGEF7	3.49E-16	8.071005626	0.02067634	OK	9.64E-16
AL139384.1	2.35E-31	11.58857887	0.02969279	OK	7.78E-31
ATP11A	1.19E-36	12.59032348	0.03228438	OK	4.11E-36
MCF2L	1.76E-06	4.637493352	0.01169022	OK	3.85E-06
F10	0	41.8836973	0.10757728	OK	0
PCID2	3.80E-42	13.55302165	0.03477751	OK	1.37E-41
CUL4A	4.05E-20	9.11184642	0.02335335	OK	1.19E-19
LAMP1	0	74.64589695	0.19191071	OK	0
GRTP1	1.75E-11	6.623628308	0.01687569	OK	4.43E-11
TMCO3	3.05E-34	12.1450708	0.03115565	OK	1.03E-33
TFDP1	8.26E-180	28.56856099	0.07338348	OK	4.90E-179
TMEM255B	8.99E-89	19.94091105	0.05118025	OK	4.10E-88
GAS6	0	157.0648358	0.40388514	OK	0
RASA3	2.85E-05	4.024668375	0.01027152	OK	5.91E-05
UPF3A	3.32E-56	15.75204142	0.040437	OK	1.30E-55
CHAMP1	0.00021752	3.517863929	0.00896975	OK	0.00043126
TTC5	4.09E-07	4.931056597	0.0126026	OK	9.14E-07
CCNB1IP1	1.04E-119	23.23358356	0.05966877	OK	5.28E-119
PARP2	9.28E-13	7.044932661	0.01803336	OK	2.41E-12
OSGEP	6.35E-83	19.25551084	0.04944464	OK	2.82E-82
APEX1	0	55.45591998	0.14255412	OK	0
PIP4P1	4.81E-144	25.52806642	0.06557638	OK	2.62E-143
PNP	0	86.45813911	0.22226895	OK	0
ANG	2.48E-79	18.82228052	0.04832845	OK	1.09E-78
RNASE6	0	188.9910292	0.4859846	OK	0
RNASE1	0	219.3098959	0.56395978	OK	0
RNASE2	2.35E-113	22.59642617	0.05799016	OK	1.17E-112
NDRG2	0	49.51467871	0.12724829	OK	0
ARHGEF40	8.31E-07	4.790794786	0.01224216	OK	1.83E-06
HNRNPC	0	58.30907359	0.14989347	OK	0
SUPT16H	1.14E-106	21.90585033	0.05626409	OK	5.57E-106
TOX4	6.81E-83	19.25185825	0.04943942	OK	3.03E-82
SALL2	2.59E-06	4.557057842	0.01156365	OK	5.62E-06
TRAV4	1.74E-08	5.515074246	0.0127625	OK	4.08E-08
TRAV13-2	9.30E-05	3.737207658	0.00849824	OK	0.0001881
TRAC	0	50.23102511	0.12901157	OK	0
DAD1	0	113.6167348	0.29213787	OK	0
ABHD4	4.53E-10	6.125251363	0.01565968	OK	1.11E-09
OXA1L	4.24E-70	17.66034763	0.04534319	OK	1.77E-69
SLC7A7	0	104.1540516	0.2677816	OK	0
MRPL52	0	54.74719384	0.1407319	OK	0
MMP14	1.39E-60	16.37723071	0.04203247	OK	5.57E-60
LRP10	0	42.70091345	0.10974565	OK	0
PRMT5	1.08E-05	4.247737152	0.01084118	OK	2.28E-05
HAUS4	1.52E-11	6.644235806	0.01699915	OK	3.86E-11

PSMB5	0	53.48305388	0.13748038	OK	0
ACIN1	3.52E-19	8.874408937	0.02274861	OK	1.02E-18
C14orf119	1.29E-182	28.79371622	0.07397823	OK	7.67E-182
CEBPE	4.83E-66	17.12494836	0.04388848	OK	1.99E-65
SLC7A8	0	41.88241638	0.10760955	OK	0
PABPN1	0.00031082	3.421992087	0.00872648	OK	0.00061069
SLC22A17	0	40.2534683	0.10338887	OK	0
EFS	6.11E-49	14.65657658	0.03754822	OK	2.29E-48
CMTM5	1.86E-21	9.44033256	0.02410324	OK	5.56E-21
NGDN	3.08E-21	9.38735782	0.02406443	OK	9.18E-21
THTPA	8.10E-12	6.736765907	0.01724398	OK	2.07E-11
AP1G2	2.39E-10	6.226444078	0.01591754	OK	5.87E-10
DHRS4-AS1	5.45E-24	10.03311054	0.02572119	OK	1.69E-23
DHRS4	2.33E-51	15.03000865	0.03857346	OK	8.87E-51
DHRS4L2	2.32E-176	28.2896376	0.07268189	OK	1.37E-175
PCK2	3.78E-166	27.44689899	0.07049826	OK	2.18E-165
DCAF11	5.19E-05	3.881302897	0.00990477	OK	0.00010625
PSME1	0	100.5702588	0.25858431	OK	0
EMC9	7.97E-28	10.87046952	0.0278737	OK	2.55E-27
PSME2	0	91.83847176	0.23612846	OK	0
AL136295.5	2.43E-05	4.062646876	0.01033695	OK	5.04E-05
REC8	4.77E-70	17.65362779	0.04519519	OK	1.99E-69
CHMP4A	1.84E-202	30.33738659	0.07795031	OK	1.15E-201
MDP1	1.66E-06	4.649342759	0.0118707	OK	3.63E-06
NEDD8	0	110.3248663	0.28367094	OK	0
GMPR2	6.25E-73	18.02454981	0.0462788	OK	2.65E-72
TINF2	2.33E-36	12.53737468	0.03216765	OK	8.02E-36
RABGGTA	4.66E-21	9.343419442	0.02394709	OK	1.39E-20
DHRS1	2.03E-14	7.559028487	0.01936259	OK	5.44E-14
CIDEB	3.86E-41	13.38178606	0.03430951	OK	1.38E-40
LTB4R	2.00E-37	12.73065743	0.03263308	OK	6.92E-37
ADCY4	4.25E-17	8.324082595	0.02112861	OK	1.19E-16
RIPK3	1.49E-23	9.933386659	0.02543792	OK	4.59E-23
NFATC4	2.26E-31	11.5920514	0.0296643	OK	7.47E-31
NYNRIN	0.00026858	3.461507986	0.00880264	OK	0.00052954
SDR39U1	8.33E-05	3.7648671	0.00960585	OK	0.00016882
CTSG	8.64E-07	4.782911112	0.01127752	OK	1.91E-06
GZMH	4.17E-46	14.20656361	0.03638428	OK	1.54E-45
GZMB	3.23E-14	7.498252182	0.01912797	OK	8.65E-14
STXBP6	1.57E-10	6.291562194	0.015843	OK	3.89E-10
NOVA1	5.94E-73	18.02741458	0.0462337	OK	2.51E-72
PRKD1	2.15E-08	5.477660427	0.01395443	OK	5.03E-08
G2E3	2.00E-07	5.068939372	0.01295793	OK	4.52E-07
SCFD1	9.58E-19	8.762162687	0.02245856	OK	2.75E-18
COCH	8.99E-15	7.664254612	0.0195156	OK	2.43E-14
STRN3	3.70E-29	11.14705041	0.02859136	OK	1.20E-28
HECTD1	9.50E-06	4.276212313	0.01092293	OK	2.01E-05
NUBPL	4.45E-09	5.750623851	0.01470167	OK	1.06E-08
ARHGAP5-AS1	3.04E-12	6.877567034	0.01758757	OK	7.83E-12
ARHGAP5	1.45E-18	8.71517718	0.02233804	OK	4.16E-18
AKAP6	2.89E-153	26.34549383	0.06764361	OK	1.61E-152
EGLN3	0	47.42918825	0.1218289	OK	0
SPTSSA	1.39E-109	22.20960686	0.0570441	OK	6.84E-109

EAPP	1.90E-203	30.41205319	0.07814044	OK	1.19E-202
SNX6	0	118.3453218	0.3043028	OK	0
CFL2	0	115.0790498	0.29586981	OK	0
BAZ1A	0	77.96889706	0.20044706	OK	0
SRP54	1.89E-29	11.20678937	0.02874722	OK	6.14E-29
FAM177A1	2.24E-203	30.40672837	0.0781289	OK	1.40E-202
PPP2R3C	1.31E-94	20.60220204	0.05290892	OK	6.13E-94
PSMA6	1.39E-115	22.82184004	0.05860406	OK	6.98E-115
NFKBIA	0	134.4756998	0.34578473	OK	0
RALGAPA1	3.76E-07	4.947355792	0.01264606	OK	8.42E-07
AL162311.3	3.93E-08	5.370126136	0.01333325	OK	9.10E-08
BRMS1L	4.20E-14	7.463846428	0.01910737	OK	1.12E-13
MBIP	1.12E-75	18.37127981	0.04716371	OK	4.79E-75
PAX9	4.45E-06	4.442268738	0.01124012	OK	9.54E-06
SLC25A21-AS1	1.35E-32	11.83112663	0.03029016	OK	4.51E-32
MIPOL1	1.11E-42	13.64277681	0.03497114	OK	4.02E-42
CLEC14A	0	102.7702079	0.26318183	OK	0
LINC00639	1.56E-16	8.168503027	0.01855645	OK	4.34E-16
SEC23A	4.03E-17	8.330466764	0.0213466	OK	1.13E-16
GEMIN2	0.00031355	3.41961625	0.00871145	OK	0.00061583
TRAPPC6B	4.88E-06	4.422577009	0.01129695	OK	1.04E-05
PNN	9.01E-50	14.78608401	0.03795466	OK	3.40E-49
MIA2	1.29E-35	12.40073328	0.03181446	OK	4.43E-35
FBXO33	1.13E-15	7.925917654	0.02030413	OK	3.10E-15
LINC02315	1.85E-06	4.62797543	0.01108003	OK	4.03E-06
LRFN5	3.63E-25	10.29712695	0.0244179	OK	1.13E-24
C14orf28	3.91E-18	8.602092381	0.02202465	OK	1.12E-17
FKBP3	3.18E-238	32.93764437	0.08463689	OK	2.12E-237
MIS18BP1	0	73.52439115	0.18901687	OK	0
RPS29	0	152.5416341	0.3922188	OK	0
LRR1	2.96E-103	21.54487114	0.05530575	OK	1.42E-102
RPL36AL	0	166.139364	0.42719216	OK	0
MGAT2	1.53E-115	22.817593	0.05859977	OK	7.68E-115
DNAAF2	3.51E-10	6.165675403	0.01577748	OK	8.61E-10
POLE2	1.18E-16	8.202067222	0.02078988	OK	3.29E-16
KLHDC2	1.69E-130	24.27752389	0.06235917	OK	8.88E-130
NEMF	1.43E-05	4.183806997	0.01068589	OK	3.01E-05
AL627171.1	5.71E-06	4.388399382	0.01119423	OK	1.22E-05
ARF6	0	58.21620841	0.1496509	OK	0
LINC01588	1.29E-14	7.617642714	0.01948154	OK	3.47E-14
VCPKMT	5.66E-05	3.860388202	0.00984676	OK	0.00011555
SOS2	9.50E-05	3.731931952	0.00952199	OK	0.00019201
ATP5S	1.51E-40	13.27992879	0.03407538	OK	5.37E-40
MAP4K5	1.89E-11	6.612841157	0.01692989	OK	4.76E-11
ATL1	3.26E-191	29.47231179	0.07567161	OK	1.99E-190
SAV1	1.07E-55	15.6781994	0.04024228	OK	4.14E-55
NIN	2.56E-29	11.17982955	0.0286741	OK	8.31E-29
PYGL	0	44.17910781	0.11353623	OK	0
TMX1	4.11E-282	35.87242551	0.09218352	OK	2.90E-281
FRMD6	1.21E-57	15.96012857	0.04092525	OK	4.77E-57
GNG2	1.03E-210	30.9567091	0.07951976	OK	6.56E-210
RTRAF	0	69.73495011	0.17928027	OK	0
NID2	1.87E-06	4.625268847	0.01174121	OK	4.08E-06

PTGDR	0.00014649	3.62142971	0.00917736	OK	0.00029329
PTGER2	0	75.94048807	0.19519711	OK	0
TXNDC16	1.93E-17	8.416964565	0.02156287	OK	5.44E-17
ERO1A	0	131.376874	0.33779164	OK	0
AL133453.1	0	56.23548745	0.14452666	OK	0
PSMC6	6.95E-77	18.52133481	0.04756029	OK	3.00E-76
STYX	6.51E-09	5.685731275	0.01454281	OK	1.54E-08
GNPNAT1	6.14E-13	7.10228146	0.01818321	OK	1.60E-12
FERMT2	0	151.915908	0.39062087	OK	0
DDHD1	7.12E-75	18.2704267	0.04690245	OK	3.04E-74
BMP4	1.52E-63	16.78694203	0.04302352	OK	6.17E-63
CDKN3	0	156.5136701	0.40169107	OK	0
CNIH1	3.17E-202	30.31947542	0.07790339	OK	1.98E-201
GMFB	4.09E-220	31.64760069	0.0813071	OK	2.64E-219
CGRRF1	7.49E-20	9.04488937	0.0231829	OK	2.19E-19
SAMD4A	5.60E-10	6.09121058	0.01557808	OK	1.37E-09
GCH1	1.54E-67	17.32424511	0.04443123	OK	6.38E-67
WDHD1	4.32E-31	11.53646059	0.02949249	OK	1.42E-30
SOCS4	5.46E-09	5.715879846	0.01462288	OK	1.30E-08
MAPK1IP1L	1.88E-169	27.72229308	0.07122319	OK	1.09E-168
LGALS3	0	164.4773678	0.42294115	OK	0
DLGAP5	0	191.3454841	0.4888614	OK	0
FBXO34	8.91E-66	17.08936019	0.04386767	OK	3.65E-65
ATG14	3.46E-05	3.978754086	0.01015591	OK	7.14E-05
KTN1	0	48.39019377	0.12438148	OK	0
LINC00520	5.58E-25	10.25561876	0.02591651	OK	1.74E-24
EXOC5	2.58E-08	5.445713534	0.01392909	OK	6.01E-08
ARMH4	3.54E-08	5.388929748	0.01373466	OK	8.21E-08
ACTR10	7.15E-203	30.36852269	0.07802864	OK	4.46E-202
PSMA3	0	51.31014201	0.13189202	OK	0
ARID4A	6.02E-107	21.93504908	0.05633877	OK	2.94E-106
TIMM9	3.23E-26	10.52745215	0.02699667	OK	1.02E-25
DACT1	6.84E-31	11.49691889	0.02945281	OK	2.25E-30
DAAM1	1.76E-153	26.36438596	0.067726	OK	9.79E-153
L3HYPDH	5.95E-10	6.081548674	0.01555644	OK	1.45E-09
JKAMP	2.39E-53	15.33041352	0.03935156	OK	9.18E-53
RTN1	7.26E-145	25.60190065	0.0657503	OK	3.96E-144
PCNX4	6.94E-08	5.266759108	0.01346842	OK	1.59E-07
DHRS7	0	73.98499152	0.19021085	OK	0
PPM1A	1.14E-06	4.727243169	0.01208127	OK	2.50E-06
SIX1	6.33E-31	11.50349785	0.02942042	OK	2.08E-30
MNAT1	1.01E-58	16.11431528	0.04136329	OK	4.01E-58
TRMT5	4.81E-08	5.333734736	0.01363341	OK	1.11E-07
SLC38A6	0	43.3488258	0.11139189	OK	0
PRKCH	1.19E-19	8.994049771	0.02303226	OK	3.47E-19
TMEM30B	1.23E-84	19.45850121	0.04990047	OK	5.53E-84
HIF1A-AS1	4.55E-06	4.437746584	0.01096825	OK	9.74E-06
HIF1A	0	115.9858465	0.2982332	OK	0
HIF1A-AS2	4.12E-251	33.82579061	0.08684279	OK	2.79E-250
SNAPC1	0	50.38670874	0.12950433	OK	0
RHOJ	5.03E-60	16.29881344	0.04177562	OK	2.01E-59
PPP2R5E	1.06E-22	9.735781961	0.02496291	OK	3.23E-22
SGPP1	1.07E-64	16.94354658	0.04347343	OK	4.38E-64

SYNE2	0	67.79845592	0.17427168	OK	0
MTHFD1	6.82E-18	8.538201921	0.02187414	OK	1.94E-17
AKAP5	2.04E-12	6.934446002	0.01766771	OK	5.27E-12
ZBTB1	3.20E-96	20.78119438	0.05336647	OK	1.50E-95
HSPA2	0	50.66947759	0.13020335	OK	0
SPTB	3.08E-06	4.521061178	0.0114554	OK	6.65E-06
CHURC1	0	60.93137403	0.15663621	OK	0
MAX	5.32E-162	27.09711504	0.06961255	OK	3.03E-161
FUT8	0.00042877	3.333523806	0.00849161	OK	0.00083608
MPP5	7.75E-07	4.804694541	0.0122752	OK	1.71E-06
ATP6V1D	0	44.18835392	0.11357186	OK	0
EIF2S1	4.16E-185	28.9919162	0.07448799	OK	2.50E-184
PLEK2	2.69E-196	29.86637176	0.07667774	OK	1.66E-195
PIGH	1.57E-17	8.441195226	0.02162867	OK	4.43E-17
ARG2	4.95E-30	11.32479794	0.02902374	OK	1.61E-29
VTI1B	0	49.01130527	0.12597802	OK	0
RDH11	1.17E-27	10.8350183	0.02779	OK	3.76E-27
ZFP36L1	0	95.1062374	0.24453035	OK	0
ACTN1	0	97.87867942	0.25165984	OK	0
GALNT16	1.87E-117	23.00964135	0.05887897	OK	9.40E-117
ERH	0	77.17934204	0.19842658	OK	0
SUSD6	2.29E-275	35.4371306	0.09105298	OK	1.60E-274
SRSF5	3.25E-107	21.9631077	0.0564135	OK	1.59E-106
SLC10A1	8.12E-12	6.736396403	0.01403135	OK	2.07E-11
SMOC1	1.00E-20	9.262307113	0.02347539	OK	2.97E-20
COX16	9.46E-08	5.209743336	0.01331805	OK	2.16E-07
SYNJ2BP	1.97E-27	10.78752413	0.02766483	OK	6.30E-27
MED6	1.21E-09	5.967085656	0.01526977	OK	2.92E-09
AC004816.1	3.77E-09	5.77839992	0.01388346	OK	9.00E-09
PCNX1	0.00020137	3.538286365	0.00902261	OK	0.00039992
AC004817.3	1.20E-165	27.40483843	0.07028984	OK	6.90E-165
SIPA1L1	1.36E-08	5.559014463	0.01421935	OK	3.18E-08
RGS6	1.28E-157	26.72268904	0.06827446	OK	7.23E-157
RBM25	1.52E-37	12.75187734	0.03272289	OK	5.28E-37
PSEN1	7.37E-34	12.07263048	0.03096631	OK	2.49E-33
PAPLN	1.42E-20	9.224557249	0.02341191	OK	4.22E-20
NUMB	6.40E-115	22.75501009	0.05844337	OK	3.20E-114
AC005280.2	5.63E-186	29.06071104	0.07457723	OK	3.39E-185
HEATR4	0.00019962	3.540582501	0.00854219	OK	0.00039667
RIOX1	3.29E-05	3.990750014	0.01017957	OK	6.80E-05
ACOT2	0.00026855	3.461540387	0.00880317	OK	0.00052952
ACOT4	4.34E-57	15.88031558	0.04067268	OK	1.70E-56
PNMA1	1.17E-60	16.38782437	0.04205852	OK	4.68E-60
ELMSAN1	1.24E-153	26.37743638	0.06775876	OK	6.94E-153
PTGR2	1.14E-06	4.726339615	0.01206844	OK	2.51E-06
ALDH6A1	9.03E-53	15.24395905	0.03911864	OK	3.45E-52
LIN52	0.00011516	3.68320917	0.009377	OK	0.00023174
ABCD4	1.95E-11	6.607972604	0.01691554	OK	4.92E-11
NPC2	0	250.9851325	0.64543227	OK	0
ISCA2	3.04E-175	28.19861208	0.07244054	OK	1.79E-174
LTBP2	0	114.5201925	0.29439733	OK	0
AC013451.1	1.69E-11	6.629042452	0.01552088	OK	4.27E-11
AREL1	3.35E-07	4.969808271	0.01268231	OK	7.51E-07

FCF1	7.19E-06	4.337850657	0.01107773	OK	1.53E-05
PGF	0	82.7194308	0.21259615	OK	0
EIF2B2	6.26E-20	9.064564323	0.02323268	OK	1.83E-19
MLH3	6.70E-09	5.68095182	0.01452982	OK	1.58E-08
ACYP1	1.18E-79	18.8617741	0.04842102	OK	5.16E-79
TMED10	0	61.31423792	0.15762261	OK	0
FOS	0	43.24047032	0.11113206	OK	0
LINC01220	0.00015599	3.605148178	0.00916591	OK	0.00031185
JDP2	0	60.06011734	0.15438755	OK	0
BATF	2.38E-152	26.26545485	0.06745176	OK	1.32E-151
FLVCR2	5.21E-71	17.77826178	0.04561167	OK	2.19E-70
ERG28	6.30E-94	20.52606553	0.05270512	OK	2.93E-93
IFT43	4.08E-96	20.76957725	0.05333441	OK	1.91E-95
TGFB3	7.10E-58	15.99340608	0.0410028	OK	2.80E-57
GPATCH2L	3.25E-06	4.509387872	0.01152188	OK	7.02E-06
VASH1	6.81E-31	11.49728841	0.02947389	OK	2.24E-30
IRF2BPL	3.87E-41	13.38158665	0.03433591	OK	1.38E-40
GSTZ1	2.28E-92	20.35090469	0.05225265	OK	1.06E-91
AHSA1	6.56E-88	19.8412249	0.05095372	OK	2.98E-87
SPTLC2	5.28E-165	27.3508101	0.07025429	OK	3.03E-164
SLIRP	0	48.7763364	0.12537475	OK	0
SNW1	2.70E-115	22.79289167	0.05854668	OK	1.35E-114
NRXN3	4.25E-25	10.281835	0.02616498	OK	1.33E-24
DIO2	4.54E-52	15.13806489	0.03875556	OK	1.73E-51
CEP128	3.98E-10	6.145886581	0.01570163	OK	9.74E-10
GTF2A1	5.09E-16	8.02461634	0.0205599	OK	1.40E-15
STON2	4.98E-37	12.65907411	0.03238652	OK	1.72E-36
SEL1L	1.27E-46	14.28961138	0.03667541	OK	4.70E-46
FLRT2	4.10E-184	28.9129812	0.07415372	OK	2.46E-183
LINC02328	2.75E-05	4.033560331	0.01023756	OK	5.69E-05
GALC	3.80E-80	18.9214116	0.04856939	OK	1.67E-79
GPR65	0	97.76526833	0.25135063	OK	0
SPATA7	3.89E-11	6.504694212	0.01664317	OK	9.77E-11
PTPN21	1.10E-32	11.8483942	0.03034649	OK	3.67E-32
ZC3H14	4.37E-09	5.753478742	0.01472041	OK	1.04E-08
TTC8	1.91E-22	9.675992299	0.02478839	OK	5.79E-22
FOXN3	2.96E-107	21.96736032	0.05642133	OK	1.44E-106
EFCAB11	1.05E-17	8.488377881	0.02173919	OK	2.97E-17
TDP1	2.45E-27	10.7674288	0.02759321	OK	7.83E-27
KCNK13	3.79E-45	14.05106712	0.03601979	OK	1.39E-44
PSMC1	4.32E-306	37.37876572	0.09606087	OK	3.15E-305
CALM1	0	150.9118722	0.38801213	OK	0
RPS6KA5	4.01E-06	4.464608188	0.01140022	OK	8.62E-06
DGLUCY	3.47E-35	12.32151713	0.03159671	OK	1.18E-34
CCDC88C	4.38E-42	13.5426695	0.03470699	OK	1.57E-41
PPP4R3A	4.69E-18	8.581242836	0.02199383	OK	1.34E-17
TC2N	2.29E-111	22.39316406	0.05746338	OK	1.14E-110
FBLN5	0	162.6752276	0.41826077	OK	0
TRIP11	1.02E-43	13.8160755	0.03545712	OK	3.70E-43
ATXN3	1.70E-06	4.644848805	0.0118704	OK	3.71E-06
NDUFB1	0	65.42820504	0.16820343	OK	0
CPSF2	0.00036899	3.375064147	0.00860366	OK	0.00072208
SLC24A4	3.13E-08	5.411253958	0.01379508	OK	7.26E-08

RIN3	0	45.94507021	0.11807309	OK	0
LGMN	0	184.2525284	0.47379995	OK	0
GOLGA5	6.08E-07	4.853072909	0.01240358	OK	1.35E-06
ITPK1	2.98E-32	11.76443125	0.03017262	OK	9.93E-32
MOAP1	1.46E-45	14.11831555	0.03622853	OK	5.37E-45
TMEM251	0	53.35274818	0.13713335	OK	0
GON7	1.62E-58	16.08503415	0.04128437	OK	6.43E-58
UBR7	4.40E-14	7.457721679	0.01909527	OK	1.17E-13
BTBD7	3.12E-12	6.874143867	0.01760289	OK	8.02E-12
ASB2	1.07E-86	19.70045772	0.05052233	OK	4.83E-86
DDX24	2.00E-196	29.87624181	0.07676513	OK	1.23E-195
IFI27L1	6.45E-22	9.55056091	0.02447867	OK	1.94E-21
IFI27	0	37.63319614	0.09666323	OK	0
IFI27L2	0	50.31399409	0.12932869	OK	0
SERPINA1	0	204.7488532	0.526513	OK	0
DICER1	3.68E-44	13.88924757	0.03564433	OK	1.34E-43
CLMN	0	40.47284126	0.10395738	OK	0
SYNE3	8.04E-08	5.239674734	0.0133888	OK	1.84E-07
SNHG10	6.93E-07	4.826990744	0.0123238	OK	1.53E-06
GLRX5	0	40.22920996	0.10339077	OK	0
C14orf132	2.64E-37	12.70881378	0.03254442	OK	9.14E-37
GSKIP	1.59E-09	5.922520577	0.01515097	OK	3.82E-09
PAPOLA	9.59E-258	34.2739423	0.08807565	OK	6.55E-257
VRK1	1.81E-104	21.67398322	0.05564261	OK	8.73E-104
LINC01550	2.15E-06	4.596752446	0.01170735	OK	4.67E-06
BCL11B	5.96E-22	9.558699834	0.02436239	OK	1.80E-21
SETD3	1.65E-56	15.79639928	0.04054824	OK	6.43E-56
CCNK	1.71E-59	16.22368393	0.04164551	OK	6.83E-59
EML1	7.77E-30	11.28513058	0.02891781	OK	2.53E-29
EVL	3.11E-191	29.47389848	0.07572322	OK	1.90E-190
DEGS2	0.00010858	3.698174239	0.00935006	OK	0.00021878
YY1	0	42.98827717	0.11048908	OK	0
SLC25A29	1.08E-23	9.965345754	0.02554065	OK	3.33E-23
WARS	5.47E-90	20.08049195	0.0515551	OK	2.50E-89
WDR25	6.88E-05	3.812423802	0.00972304	OK	0.00013996
MEG3	0	84.31818129	0.21638603	OK	0
AL117190.1	5.67E-17	8.289901969	0.01865142	OK	1.59E-16
MEG8	4.50E-10	6.126281825	0.01556507	OK	1.10E-09
LINC02285	9.84E-07	4.756639481	0.01211835	OK	2.17E-06
DIO3OS	2.47E-11	6.572598953	0.01651579	OK	6.23E-11
PPP2R5C	2.73E-30	11.37670241	0.02918426	OK	8.93E-30
AL118558.3	6.02E-09	5.699197342	0.01456311	OK	1.43E-08
DYNC1H1	2.78E-54	15.46971173	0.03971208	OK	1.07E-53
HSP90AA1	0	101.1407935	0.26002898	OK	0
WDR20	6.49E-06	4.360277172	0.01113579	OK	1.38E-05
CINP	1.45E-115	22.82002418	0.05861055	OK	7.27E-115
ANKRD9	2.62E-116	22.89480098	0.05879742	OK	1.32E-115
RCOR1	3.09E-227	32.16111429	0.08262971	OK	2.02E-226
TRAF3	1.94E-56	15.78603337	0.04051105	OK	7.58E-56
CDC42BPB	6.36E-05	3.831876342	0.00977858	OK	0.00012958
EXOC3L4	6.33E-07	4.844945204	0.01162738	OK	1.40E-06
TNFAIP2	0	99.16744074	0.25494828	OK	0
AL161669.3	7.71E-09	5.656771846	0.01362753	OK	1.82E-08

EIF5	0	47.80035595	0.12286545	OK	0
MARK3	7.17E-05	3.80207555	0.00970294	OK	0.00014584
CKB	2.76E-248	33.63303796	0.08639269	OK	1.86E-247
TRMT61A	2.98E-08	5.420087598	0.01385733	OK	6.92E-08
BAG5	2.70E-25	10.32552336	0.02647977	OK	8.46E-25
APOPT1	3.03E-91	20.22360929	0.05193529	OK	1.40E-90
XRCC3	1.87E-05	4.123172079	0.01045034	OK	3.91E-05
ZFYVE21	0	71.27068959	0.18321689	OK	0
PPP1R13B	8.59E-05	3.757112542	0.00955545	OK	0.00017401
ATP5MPL	0	67.42431036	0.17333717	OK	0
C14orf180	3.47E-30	11.3556982	0.02902523	OK	1.14E-29
INF2	1.20E-42	13.63767844	0.03498704	OK	4.31E-42
ADSSL1	5.49E-44	13.8605552	0.03555134	OK	2.00E-43
SIVA1	0	85.98305407	0.22106872	OK	0
AKT1	2.24E-51	15.0328675	0.03858697	OK	8.49E-51
PLD4	0	112.1621383	0.28825283	OK	0
AHNAK2	3.54E-31	11.55355912	0.029598	OK	1.17E-30
CDCA4	6.82E-26	10.45685241	0.02679937	OK	2.15E-25
GPR132	5.88E-205	30.52604729	0.07838921	OK	3.69E-204
LINC02298	1.50E-07	5.123334874	0.01300511	OK	3.41E-07
JAG2	1.49E-22	9.701322723	0.02439103	OK	4.52E-22
NUDT14	0	49.1864725	0.12641219	OK	0
BTBD6	6.36E-30	11.3027053	0.02899075	OK	2.07E-29
PACS2	0.00016199	3.595332556	0.00916926	OK	0.00032349
CRIP2	0	216.6041408	0.55699924	OK	0
CRIP1	0	77.49735266	0.19923479	OK	0
TEDC1	4.09E-48	14.52693366	0.03725427	OK	1.53E-47
TMEM121	7.65E-32	11.6844785	0.02989726	OK	2.54E-31
IGHA1	1.41E-21	9.46936495	0.02427138	OK	4.22E-21
IGHG3	9.23E-05	3.739114774	0.00952635	OK	0.0001867
FAM30A	1.99E-05	4.108419037	0.01011995	OK	4.16E-05
IGHV1-3	7.38E-151	26.13458113	0.05368642	OK	4.09E-150
NIPA2	4.10E-166	27.4439272	0.07049931	OK	2.36E-165
CYFIP1	0	54.12901299	0.13913816	OK	0
NDN	0	80.478233	0.206888	OK	0
SNRPN	0	96.0039715	0.24683494	OK	0
PWAR6	2.42E-21	9.412782137	0.02407595	OK	7.22E-21
UBE3A	5.26E-14	7.434234464	0.0190452	OK	1.40E-13
ATP10A	7.08E-17	8.263461413	0.02114773	OK	1.98E-16
HERC2	0.00011818	3.676604947	0.00938001	OK	0.00023767
NSMCE3	2.92E-91	20.22557377	0.05194038	OK	1.34E-90
TJP1	0	75.20036046	0.19328761	OK	0
ARHGAP11B	1.25E-06	4.707405682	0.01189758	OK	2.75E-06
AC091057.6	1.32E-15	7.906450794	0.01960341	OK	3.62E-15
MTMR10	2.80E-11	6.553989823	0.01677079	OK	7.05E-11
KLF13	1.40E-100	21.25751042	0.05459665	OK	6.70E-100
LINC02256	6.18E-14	7.412966982	0.01897488	OK	1.64E-13
ARHGAP11A	0	42.90005088	0.10973062	OK	0
SCG5	3.26E-73	18.06046323	0.046176	OK	1.38E-72
AC090877.2	5.68E-07	4.866597687	0.01133507	OK	1.26E-06
GREM1	1.69E-88	19.90930793	0.05070575	OK	7.69E-88
FMN1	0	42.69313488	0.10968821	OK	0
RYR3	1.96E-08	5.494625814	0.01390325	OK	4.57E-08

AVEN	2.84E-66	17.15589304	0.04403329	OK	1.17E-65
EMC7	2.65E-281	35.82046106	0.09205296	OK	1.87E-280
KATNBL1	2.17E-22	9.662672641	0.02477526	OK	6.59E-22
EMC4	0	38.92689079	0.10004253	OK	0
SLC12A6	1.53E-19	8.966507207	0.02297188	OK	4.45E-19
NOP10	0	133.9585182	0.3444589	OK	0
LPCAT4	0.00013111	3.65001758	0.00930503	OK	0.000263
GOLGA8A	1.80E-50	14.89428122	0.03820762	OK	6.79E-50
GOLGA8B	9.90E-87	19.70431059	0.05057259	OK	4.47E-86
ACTC1	0	80.84548528	0.20775163	OK	0
ZNF770	1.74E-24	10.14538233	0.02601069	OK	5.40E-24
MEIS2	7.02E-247	33.53676275	0.0861377	OK	4.72E-246
AC078909.1	9.63E-07	4.76113984	0.01212758	OK	2.12E-06
LINC02345	1.94E-152	26.27328429	0.06742702	OK	1.08E-151
SPRED1	0	60.26190081	0.1548974	OK	0
RASGRP1	3.53E-19	8.873979295	0.02264258	OK	1.02E-18
AC109630.1	3.54E-07	4.959179924	0.0126314	OK	7.93E-07
THBS1	0	50.57476255	0.12995929	OK	0
GPR176	2.09E-41	13.42729179	0.03438321	OK	7.48E-41
EIF2AK4	2.61E-76	18.45002616	0.04737393	OK	1.12E-75
SRP14	0	196.9535053	0.50642726	OK	0
SRP14-AS1	8.91E-06	4.29064776	0.01094751	OK	1.89E-05
BMF	1.53E-41	13.45041405	0.03447755	OK	5.48E-41
BUB1B	0	118.1158809	0.30114456	OK	0
PLCB2	3.24E-137	24.90551086	0.06394364	OK	1.74E-136
INAFM2	3.54E-24	10.07552138	0.02583263	OK	1.10E-23
KNSTRN	8.71E-51	14.94251383	0.03831529	OK	3.30E-50
IVD	6.20E-18	8.549230414	0.02190779	OK	1.76E-17
CHST14	1.25E-17	8.467994696	0.0216894	OK	3.53E-17
CCDC32	4.86E-21	9.339049792	0.02394044	OK	1.45E-20
RPUSD2	6.15E-05	3.839948695	0.00978803	OK	0.00012547
KNL1	0	116.0750021	0.29744447	OK	0
RMDN3	2.90E-53	15.31797948	0.03931271	OK	1.11E-52
GCHFR	0	106.6581687	0.27420306	OK	0
DNAJC17	8.77E-16	7.957599739	0.02038585	OK	2.41E-15
ZFYVE19	0.00026735	3.462738987	0.00882629	OK	0.00052726
SPINT1-AS1	1.21E-15	7.917418874	0.02021026	OK	3.32E-15
SPINT1	9.98E-123	23.53033967	0.06039762	OK	5.10E-122
VPS18	1.10E-08	5.596129914	0.01430699	OK	2.58E-08
INO80	3.88E-07	4.941180475	0.01263198	OK	8.68E-07
CHP1	1.48E-192	29.57696624	0.07599214	OK	9.03E-192
OIP5	0	44.88906099	0.11418549	OK	0
NUSAP1	0	162.5986723	0.41728408	OK	0
NDUFAF1	1.28E-11	6.669334407	0.01707189	OK	3.26E-11
RTF1	1.40E-122	23.51595422	0.06040664	OK	7.16E-122
LTK	3.09E-07	4.985216465	0.01229022	OK	6.95E-07
EHD4	0	43.59185585	0.11203186	OK	0
TMEM87A	1.12E-79	18.86453766	0.04844033	OK	4.90E-79
ZNF106	5.85E-66	17.113835	0.04394088	OK	2.40E-65
SNAP23	3.15E-166	27.4535503	0.07053123	OK	1.81E-165
HAUS2	1.05E-53	15.38366358	0.03948045	OK	4.05E-53
STARD9	1.06E-46	14.30214584	0.03665661	OK	3.92E-46
CCNDBP1	8.62E-100	21.1721967	0.05437701	OK	4.10E-99

MAP1A	4.85E-196	29.84665245	0.07663607	OK	2.99E-195
PDIA3	0	101.8202324	0.26179752	OK	0
SERF2	0	215.5698167	0.5542697	OK	0
MFAP1	1.08E-47	14.46044568	0.03711317	OK	4.01E-47
WDR76	6.22E-42	13.51680484	0.03463372	OK	2.23E-41
CASC4	2.19E-77	18.58345151	0.04771645	OK	9.47E-77
EIF3J-DT	5.40E-58	16.01044832	0.0410867	OK	2.13E-57
EIF3J	4.82E-200	30.15353583	0.07747757	OK	2.99E-199
SPG11	3.58E-05	3.970876389	0.01013505	OK	7.38E-05
B2M	0	219.7415669	0.56490583	OK	0
TRIM69	6.74E-20	9.056464293	0.02321235	OK	1.97E-19
SORD	9.47E-12	6.713943699	0.01718362	OK	2.41E-11
SHF	3.36E-12	6.863286738	0.01755465	OK	8.65E-12
GATM	0	80.25206257	0.20629407	OK	0
SPATA5L1	0.00018452	3.561281813	0.0090752	OK	0.00036733
C15orf48	0	238.2499758	0.61265122	OK	0
AC025580.2	1.36E-05	4.195554786	0.0106314	OK	2.86E-05
SLC30A4	1.03E-08	5.60649388	0.01431968	OK	2.43E-08
BLOC1S6	5.66E-218	31.49159149	0.08091709	OK	3.64E-217
SQOR	0	59.1490965	0.15204828	OK	0
AC073941.1	2.12E-09	5.874742941	0.0149207	OK	5.08E-09
MYEF2	1.27E-188	29.26942129	0.0751648	OK	7.70E-188
DUT	0	63.23746085	0.16256797	OK	0
FBN1	0	71.28170926	0.18317433	OK	0
CEP152	6.05E-11	6.437965542	0.01645717	OK	1.51E-10
SHC4	9.99E-23	9.74188525	0.02490243	OK	3.04E-22
EID1	0	164.519081	0.42305254	OK	0
SECISBP2L	4.04E-16	8.052861485	0.02063461	OK	1.12E-15
COPS2	2.09E-128	24.07871152	0.06185236	OK	1.09E-127
GALK2	0.00026458	3.465547763	0.00883466	OK	0.00052206
FGF7	0	43.47888771	0.11166615	OK	0
DTWD1	1.33E-59	16.23933359	0.04168566	OK	5.30E-59
ATP8B4	1.18E-289	36.35296332	0.09336973	OK	8.41E-289
SLC27A2	6.15E-07	4.850689444	0.01204119	OK	1.37E-06
HDC	2.17E-43	13.76154694	0.03364894	OK	7.86E-43
GABPB1	1.30E-18	8.727430589	0.02235924	OK	3.74E-18
GABPB1-AS1	1.26E-05	4.213394561	0.01076032	OK	2.65E-05
USP8	2.01E-30	11.40359787	0.02925378	OK	6.57E-30
SPPL2A	2.74E-155	26.52142773	0.06813569	OK	1.54E-154
TNFAIP8L3	8.36E-66	17.09305272	0.04386514	OK	3.43E-65
GLDN	0	47.1607669	0.1211597	OK	0
DMXL2	0	64.38827292	0.16549927	OK	0
LYSMD2	0	49.25862927	0.12661068	OK	0
TMOD2	3.05E-84	19.41204668	0.04983094	OK	1.37E-83
TMOD3	0	43.84751436	0.11269736	OK	0
LEO1	3.64E-21	9.36975426	0.02401874	OK	1.08E-20
MAPK6	3.98E-183	28.83434748	0.07407077	OK	2.38E-182
GNB5	1.15E-68	17.47309033	0.04483948	OK	4.77E-68
MYO5C	3.01E-14	7.507726633	0.01875329	OK	8.05E-14
MYO5A	0	56.12279916	0.14424155	OK	0
ARPP19	2.06E-78	18.7098925	0.04804421	OK	8.96E-78
UNC13C	1.86E-39	13.09078762	0.03350823	OK	6.54E-39
RSL24D1	0	74.29480981	0.19100736	OK	0

RAB27A	2.46E-33	11.97302588	0.03070878	OK	8.28E-33
PIGBOS1	1.91E-29	11.20574934	0.02874107	OK	6.21E-29
CCPG1	0	45.11422102	0.11595548	OK	0
DNAAF4	1.93E-07	5.075941189	0.0129696	OK	4.36E-07
PYGO1	9.20E-07	4.770219588	0.01215179	OK	2.03E-06
NEDD4	2.26E-41	13.42151515	0.0344224	OK	8.09E-41
TEX9	3.18E-15	7.796718555	0.0199319	OK	8.64E-15
MNS1	9.96E-24	9.973442054	0.02552864	OK	3.07E-23
TCF12	1.34E-29	11.23718668	0.02882106	OK	4.36E-29
CGNL1	4.83E-68	17.39101163	0.04458841	OK	2.00E-67
POLR2M	4.33E-08	5.352798153	0.01368641	OK	1.00E-07
ALDH1A2	1.16E-20	9.246116992	0.02366768	OK	3.45E-20
AQP9	0	194.7833263	0.5007552	OK	0
LIPC	5.15E-06	4.410975354	0.01084644	OK	1.10E-05
ADAM10	0	39.47326226	0.10143842	OK	0
MINDY2	2.38E-214	31.22578432	0.0802248	OK	1.52E-213
RNF111	2.73E-08	5.435594359	0.01390192	OK	6.35E-08
SLTM	4.64E-36	12.48265542	0.03203015	OK	1.59E-35
CCNB2	0	152.8570956	0.39184388	OK	0
MYO1E	0	41.89855763	0.10766683	OK	0
GTF2A2	0	49.52010532	0.12728832	OK	0
BNIP2	0	43.47061712	0.11172807	OK	0
FOXB1	5.13E-32	11.71837213	0.028205	OK	1.71E-31
ANXA2	0	137.8760514	0.35452366	OK	0
ICE2	1.39E-05	4.190680301	0.01070035	OK	2.92E-05
RORA	1.50E-147	25.84192938	0.06635669	OK	8.22E-147
VPS13C	8.48E-58	15.98234731	0.04103057	OK	3.34E-57
C2CD4B	1.97E-68	17.44218559	0.04422113	OK	8.19E-68
TLN2	2.38E-99	21.12420181	0.05422338	OK	1.13E-98
TPM1	0	271.6420685	0.69857448	OK	0
LACTB	0	126.8582237	0.32619236	OK	0
RPS27L	0	113.6518384	0.2922251	OK	0
RAB8B	0	56.26070495	0.14461971	OK	0
APH1B	1.08E-164	27.32464128	0.07019334	OK	6.20E-164
USP3	0	44.56121333	0.11452411	OK	0
USP3-AS1	2.28E-06	4.583857351	0.01164983	OK	4.96E-06
FBXL22	0	40.58145207	0.10422836	OK	0
FAM96A	0	127.3865412	0.32754962	OK	0
SNX1	1.24E-127	24.00488118	0.06166041	OK	6.43E-127
SNX22	4.19E-129	24.14518281	0.06119322	OK	2.19E-128
PPIB	0	133.8899682	0.34427052	OK	0
PCLAF	0	212.8458729	0.54624486	OK	0
TRIP4	4.07E-08	5.363822803	0.0137159	OK	9.42E-08
OAZ2	1.00E-214	31.25350925	0.08030347	OK	6.39E-214
AC100830.3	2.04E-13	7.253034691	0.01667911	OK	5.37E-13
AC100830.1	0.00010312	3.711239043	0.00926768	OK	0.00020801
RBPMS2	0	110.3159148	0.28317593	OK	0
PIF1	2.49E-12	6.906002816	0.01764639	OK	6.42E-12
PLEKHO2	0	47.65411091	0.12246132	OK	0
AC103691.2	1.03E-12	7.030504893	0.01537162	OK	2.68E-12
SPG21	0	42.05533802	0.10808241	OK	0
MTFMT	2.10E-06	4.601520703	0.01175569	OK	4.56E-06
RASL12	0	87.09259585	0.22383473	OK	0

PDCD7	2.22E-19	8.925248122	0.02287893	OK	6.45E-19
CLPX	0.00047983	3.302096195	0.00841646	OK	0.0009316
IGDCC4	1.99E-13	7.256357272	0.01800119	OK	5.24E-13
DPP8	0.00025103	3.479660249	0.00887263	OK	0.00049595
HACD3	3.37E-78	18.68352046	0.04797213	OK	1.47E-77
INTS14	3.10E-05	4.005279157	0.01021873	OK	6.40E-05
DENND4A	8.25E-41	13.32525405	0.03418486	OK	2.93E-40
RAB11A	1.88E-270	35.11665579	0.09024116	OK	1.31E-269
TIPIN	9.28E-22	9.512783621	0.02437545	OK	2.79E-21
MAP2K1	9.36E-116	22.83917536	0.05865445	OK	4.69E-115
SNAPC5	9.55E-52	15.08907685	0.03872776	OK	3.63E-51
RPL4	0	102.8627942	0.26447572	OK	0
ZWILCH	2.77E-17	8.374444522	0.02143049	OK	7.80E-17
SMAD6	7.14E-37	12.63081383	0.03235303	OK	2.46E-36
AC110048.2	3.13E-06	4.517357225	0.01114317	OK	6.76E-06
LINC02206	6.87E-99	21.07416332	0.05300567	OK	3.26E-98
SMAD3	4.39E-07	4.917294642	0.01256032	OK	9.79E-07
C15orf61	5.62E-37	12.64969353	0.03245562	OK	1.94E-36
PIAS1	4.74E-15	7.746023908	0.01984541	OK	1.29E-14
FEM1B	4.68E-19	8.842442199	0.02266092	OK	1.35E-18
ITGA11	2.98E-113	22.5859318	0.05796691	OK	1.48E-112
ANP32A	0	51.11147759	0.13138022	OK	0
SPESP1	9.53E-05	3.731178948	0.00948959	OK	0.00019254
GLCE	1.29E-09	5.956452142	0.01523337	OK	3.11E-09
PAQR5	0	43.19232351	0.11071345	OK	0
KIF23	0	44.21902962	0.1131619	OK	0
RPLP1	0	187.2768845	0.48141151	OK	0
TLE3	4.23E-162	27.1056042	0.06962517	OK	2.41E-161
UACA	0	62.69405861	0.16115381	OK	0
LARP6	0	69.00041456	0.17733053	OK	0
LRRC49	5.64E-18	8.560119931	0.02189152	OK	1.60E-17
THSD4	0	40.58660891	0.10422163	OK	0
MYO9A	1.74E-26	10.58563001	0.02714275	OK	5.49E-26
PKM	0	135.5860313	0.34864175	OK	0
HEXA	0	77.8316491	0.20010004	OK	0
ARIH1	6.37E-11	6.43027301	0.01646127	OK	1.59E-10
ADPGK	0	48.78526599	0.12538387	OK	0
NEO1	3.20E-201	30.24329843	0.07764684	OK	1.99E-200
NPTN	0	45.16987261	0.1160982	OK	0
CD276	1.40E-97	20.93106274	0.05373539	OK	6.59E-97
LOXL1-AS1	5.08E-08	5.323856956	0.01357117	OK	1.17E-07
LOXL1	0	60.05849157	0.15432639	OK	0
STOML1	6.50E-06	4.360245041	0.01113025	OK	1.38E-05
PML	1.18E-08	5.582713565	0.01427939	OK	2.78E-08
ISLR	0	71.85367019	0.1846354	OK	0
STRA6	1.04E-31	11.65836046	0.02771469	OK	3.45E-31
SEMA7A	2.35E-14	7.540098434	0.01913747	OK	6.29E-14
UBL7	1.21E-67	17.3380976	0.04450764	OK	5.02E-67
UBL7-AS1	2.92E-12	6.883645087	0.01761681	OK	7.51E-12
ARID3B	8.68E-14	7.367729442	0.01884995	OK	2.30E-13
CLK3	3.22E-07	4.97745987	0.0127241	OK	7.23E-07
CSK	0	71.64180946	0.18417213	OK	0
ULK3	1.23E-05	4.217623966	0.01076424	OK	2.60E-05

SCAMP2	0	115.6271188	0.29730866	OK	0
MPI	4.58E-05	3.912047594	0.00998113	OK	9.38E-05
FAM219B	4.84E-08	5.332602922	0.01363583	OK	1.12E-07
COX5A	0	138.3782957	0.35582736	OK	0
RPP25	1.58E-88	19.91274142	0.0511179	OK	7.19E-88
SCAMP5	1.18E-12	7.011671153	0.01778929	OK	3.06E-12
PPCDC	0.00010452	3.707824077	0.00945542	OK	0.00021078
C15orf39	5.23E-13	7.124347969	0.01823395	OK	1.37E-12
COMMD4	1.14E-166	27.49063082	0.07062592	OK	6.55E-166
AC068338.3	4.63E-15	7.748958541	0.01973169	OK	1.26E-14
NEIL1	1.38E-05	4.193068328	0.01068731	OK	2.89E-05
MAN2C1	0.00039103	3.359065066	0.00855861	OK	0.00076419
SNUPN	5.04E-26	10.48546966	0.02688755	OK	1.59E-25
IMP3	2.39E-300	37.0236315	0.09514721	OK	1.73E-299
CSPG4	1.17E-170	27.82215433	0.07143382	OK	6.82E-170
UBE2Q2	8.80E-29	11.06966011	0.02839365	OK	2.85E-28
FBXO22	9.16E-07	4.771156545	0.01219296	OK	2.02E-06
ETFA	1.24E-195	29.81523413	0.07660606	OK	7.63E-195
SCAPER	2.13E-08	5.47960371	0.01401469	OK	4.97E-08
RCN2	0	55.50222181	0.14266528	OK	0
PSTPIP1	0	41.73007204	0.10722275	OK	0
TSPAN3	5.51E-183	28.82308972	0.0740544	OK	3.29E-182
PEAK1	1.93E-20	9.191719857	0.02355372	OK	5.72E-20
LINGO1	0	68.12199345	0.17454608	OK	0
TBC1D2B	8.08E-38	12.80108951	0.03284246	OK	2.81E-37
CIB2	2.67E-90	20.11609552	0.0516168	OK	1.22E-89
IDH3A	3.56E-44	13.891557	0.0356457	OK	1.30E-43
DNAJA4	1.81E-07	5.087906755	0.01299265	OK	4.10E-07
WDR61	6.48E-26	10.46170648	0.02682994	OK	2.04E-25
CRABP1	3.01E-31	11.56749553	0.02926384	OK	9.94E-31
IREB2	1.13E-05	4.237571967	0.01082134	OK	2.38E-05
HYKK	1.25E-05	4.214051082	0.01073317	OK	2.64E-05
PSMA4	0	60.11719877	0.15454347	OK	0
CHRNB4	1.04E-18	8.752781757	0.01921791	OK	2.99E-18
ADAMTS7	4.69E-10	6.119688071	0.01555276	OK	1.15E-09
MORF4L1	0	109.3878132	0.28125617	OK	0
CTSH	0	233.5073879	0.60049416	OK	0
RASGRF1	0.00033443	3.402037811	0.00856429	OK	0.00065584
ANKRD34C	1.42E-08	5.55130693	0.01341981	OK	3.33E-08
TMED3	3.73E-190	29.38955201	0.07551007	OK	2.27E-189
MTHFS	9.03E-140	25.14027391	0.06456895	OK	4.87E-139
ST20	0	63.72617454	0.16374511	OK	0
ST20-AS1	1.43E-07	5.132402817	0.01310363	OK	3.25E-07
BCL2A1	0	194.6244298	0.50047372	OK	0
ZFAND6	0	39.47219796	0.10144388	OK	0
FAH	7.65E-265	34.747164	0.08927162	OK	5.27E-264
ABHD17C	1.80E-15	7.868073224	0.02007017	OK	4.91E-15
CEMIP	1.58E-12	6.970656645	0.01752839	OK	4.08E-12
MESD	0	41.92142028	0.10774433	OK	0
TLNRD1	1.20E-249	33.72611606	0.08665314	OK	8.08E-249
IL16	0	54.62253314	0.14036801	OK	0
STARD5	6.21E-06	4.370074747	0.01114838	OK	1.32E-05
MEX3B	2.75E-08	5.434134499	0.01387405	OK	6.40E-08

EFL1	1.39E-16	8.182467537	0.02095209	OK	3.87E-16
RPS17	3.37E-114	22.68197329	0.05825842	OK	1.68E-113
CPEB1	0.00028506	3.445443389	0.00871407	OK	0.00056108
WHAMM	1.88E-15	7.86296555	0.02014536	OK	5.12E-15
HOMER2	4.77E-34	12.10839204	0.03102029	OK	1.61E-33
RAMMET	4.12E-148	25.89182742	0.06651194	OK	2.26E-147
C15orf40	4.14E-44	13.88087041	0.03562142	OK	1.51E-43
BTBD1	1.27E-21	9.479848479	0.0242998	OK	3.82E-21
TM6SF1	0	90.60468513	0.23291686	OK	0
HDGFL3	0	47.58823235	0.12229923	OK	0
ADAMTSL3	1.46E-216	31.38827746	0.08058476	OK	9.37E-216
GOLGA6L4	2.20E-23	9.894230044	0.02528733	OK	6.77E-23
ZSCAN2	0.00014082	3.631625637	0.00924176	OK	0.00028209
WDR73	0.00050914	3.285424784	0.00837065	OK	0.00098696
NMB	5.58E-42	13.52485013	0.03469761	OK	2.00E-41
SEC11A	0	103.7071929	0.26665234	OK	0
ALPK3	3.65E-17	8.342024726	0.02135971	OK	1.02E-16
PDE8A	4.94E-19	8.836478835	0.0226412	OK	1.43E-18
AKAP13	0	57.37761261	0.1474967	OK	0
NTRK3	0	39.25044945	0.10083005	OK	0
MRPL46	1.10E-32	11.84829476	0.03039205	OK	3.68E-32
MRPS11	2.09E-56	15.78129796	0.04050906	OK	8.17E-56
ISG20	0	41.54716916	0.10674436	OK	0
ACAN	4.82E-80	18.90881996	0.04846126	OK	2.12E-79
HAPLN3	1.48E-48	14.59650582	0.03744185	OK	5.53E-48
MFGE8	0	270.0841457	0.69455664	OK	0
AC013565.1	2.08E-11	6.598295334	0.01677434	OK	5.25E-11
ABHD2	3.57E-96	20.77593814	0.0533533	OK	1.68E-95
FANCI	0	50.36430853	0.12891058	OK	0
TICRR	6.40E-05	3.830303381	0.00891816	OK	0.00013037
PEX11A	5.64E-22	9.56450387	0.02449521	OK	1.70E-21
MESP1	0.00029155	3.439355848	0.00874162	OK	0.00057339
ANPEP	0	181.9895865	0.46791637	OK	0
ARPIN	1.94E-78	18.71296304	0.04804364	OK	8.46E-78
ZNF710	1.54E-20	9.215988085	0.02360291	OK	4.57E-20
IDH2	0	72.4586516	0.18627247	OK	0
SEMA4B	0.00015247	3.611075292	0.00919184	OK	0.000305
CIB1	0	92.9483957	0.23898414	OK	0
NGRN	2.18E-06	4.593199125	0.01172559	OK	4.74E-06
ZNF774	0.00041764	3.340831126	0.00843589	OK	0.00081489
IQGAP1	0	43.85235941	0.1127113	OK	0
BLM	2.21E-91	20.23920134	0.05182041	OK	1.02E-90
FURIN	2.32E-17	8.395729123	0.02150744	OK	6.52E-17
FES	1.07E-241	33.17925333	0.08523634	OK	7.16E-241
HDDC3	7.79E-63	16.68968718	0.04283854	OK	3.15E-62
UNC45A	3.86E-24	10.06701886	0.02581076	OK	1.20E-23
RCCD1	4.00E-07	4.935411087	0.01260076	OK	8.94E-07
PRC1	0	50.24216055	0.12871098	OK	0
SLCO3A1	8.50E-10	6.024113785	0.01541746	OK	2.07E-09
FAM174B	1.44E-11	6.653006367	0.01696799	OK	3.64E-11
AC106028.4	4.87E-224	31.93155304	0.08197397	OK	3.17E-223
AC013394.1	1.61E-05	4.157757732	0.01061494	OK	3.37E-05
CHD2	9.15E-12	6.71903074	0.01720482	OK	2.33E-11

RGMA	2.25E-24	10.11992607	0.02590581	OK	6.99E-24
LINC02207	6.00E-45	14.01862476	0.03589349	OK	2.19E-44
LINC01197	5.39E-238	32.92163858	0.08450551	OK	3.58E-237
LINC00924	1.65E-10	6.283714599	0.01589563	OK	4.09E-10
NR2F2-AS1	3.26E-129	24.15566582	0.06199996	OK	1.70E-128
NR2F2	0	170.2093095	0.43766674	OK	0
ARRDC4	4.03E-138	24.98898853	0.06416122	OK	2.16E-137
IGF1R	9.41E-85	19.4723977	0.04998148	OK	4.22E-84
SYNM	0	95.96353557	0.24669069	OK	0
TTC23	3.30E-06	4.506348113	0.01149649	OK	7.12E-06
LRRC28	0.00049104	3.295609465	0.0083978	OK	0.00095304
AC015660.2	3.67E-07	4.952287833	0.01230316	OK	8.21E-07
MEF2A	6.17E-223	31.85201831	0.08184579	OK	4.00E-222
LRRK1	1.18E-49	14.76776487	0.03788379	OK	4.45E-49
CHSY1	1.30E-98	21.04381959	0.05403973	OK	6.17E-98
SELENOS	0	40.87552691	0.10505522	OK	0
SNRPA1	9.38E-148	25.86005656	0.06643125	OK	5.14E-147
PCSK6	1.54E-75	18.35367832	0.04691777	OK	6.62E-75
TM2D3	1.53E-41	13.45041067	0.03451589	OK	5.48E-41
TARSL2	1.45E-10	6.304158241	0.01613345	OK	3.59E-10
POLR3K	7.34E-103	21.50275938	0.05522085	OK	3.52E-102
SNRNP25	0	40.89481201	0.10509434	OK	0
RHBDF1	4.86E-56	15.72798563	0.04032293	OK	1.90E-55
MPG	0	46.0308399	0.11831304	OK	0
HBZ	2.77E-17	8.374818951	0.02106069	OK	7.77E-17
FAM234A	0.0001644	3.591481398	0.00915838	OK	0.00032819
MRPL28	3.56E-150	26.07435235	0.06698424	OK	1.97E-149
TMEM8A	2.45E-41	13.41563301	0.03441927	OK	8.75E-41
NME4	0	76.85993209	0.19759994	OK	0
CAPN15	2.68E-09	5.835767565	0.01491688	OK	6.40E-09
METTL26	0	43.48723474	0.11177165	OK	0
MCRIP2	0	81.87618316	0.2104788	OK	0
WDR90	0.0004624	3.312458064	0.00839351	OK	0.00089896
STUB1	0	48.45190867	0.12454083	OK	0
JMJD8	5.98E-168	27.59736195	0.07089255	OK	3.46E-167
METRN	2.05E-170	27.80198921	0.07141918	OK	1.20E-169
FAM173A	3.81E-241	33.14103149	0.08515903	OK	2.54E-240
HAGHL	1.84E-123	23.6019947	0.06059567	OK	9.41E-123
MSLN	1.80E-09	5.90120337	0.01470781	OK	4.34E-09
RPUSD1	4.54E-08	5.344150117	0.01366249	OK	1.05E-07
AC009041.2	1.62E-25	10.37431887	0.02655387	OK	5.09E-25
CACNA1H	1.49E-72	17.97631171	0.04594637	OK	6.31E-72
AC120498.10	4.62E-21	9.344523582	0.02186926	OK	1.38E-20
TPSB2	9.17E-26	10.42871163	0.02640602	OK	2.88E-25
TPSAB1	1.78E-33	12.00003999	0.03038344	OK	5.99E-33
TPSD1	1.48E-13	7.296159867	0.01713241	OK	3.91E-13
UBE2I	0	55.16826933	0.14181527	OK	0
TSR3	2.61E-96	20.79102422	0.05339659	OK	1.23E-95
GNPTG	0	56.73734761	0.14584725	OK	0
C16orf91	4.53E-31	11.53233776	0.02957887	OK	1.49E-30
CLCN7	6.60E-22	9.548188568	0.02446993	OK	1.99E-21
AL031600.3	1.29E-07	5.151315273	0.01243207	OK	2.94E-07
TMEM204	0	40.07744953	0.10294187	OK	0

JPT2	6.93E-31	11.49567971	0.02947289	OK	2.28E-30
NME3	0	54.87708867	0.14106542	OK	0
MRPS34	0	50.98772944	0.13106279	OK	0
SPSB3	6.34E-21	9.310909832	0.02387073	OK	1.88E-20
NUBP2	1.40E-57	15.95114094	0.04094637	OK	5.50E-57
HAGH	2.61E-105	21.76286185	0.05589656	OK	1.27E-104
FAHD1	2.04E-40	13.25747078	0.03401462	OK	7.24E-40
MSRB1	6.93E-23	9.778952009	0.02507042	OK	2.12E-22
NDUFB10	0	95.76921049	0.24623724	OK	0
RPS2	0	194.9382354	0.50121196	OK	0
SNHG9	2.44E-10	6.222819344	0.01592628	OK	6.01E-10
GFER	1.17E-31	11.64859301	0.02987976	OK	3.87E-31
SLC9A3R2	0	77.02600957	0.19798865	OK	0
NTHL1	4.08E-84	19.39718571	0.04979501	OK	1.82E-83
PKD1	1.55E-87	19.7978934	0.05082041	OK	7.04E-87
SNHG19	3.89E-11	6.50488588	0.01663233	OK	9.76E-11
TRAF7	2.22E-55	15.63155902	0.04011853	OK	8.60E-55
MLST8	1.66E-08	5.523300852	0.01412548	OK	3.89E-08
PGP	0	63.4187349	0.16303007	OK	0
ECI1	2.87E-206	30.62467767	0.07868704	OK	1.81E-205
RNPS1	2.97E-61	16.47095163	0.04228693	OK	1.19E-60
CCNF	2.59E-43	13.74882835	0.03499646	OK	9.37E-43
TEDC2	3.75E-32	11.74502245	0.02954802	OK	1.25E-31
AMDHD2	2.86E-219	31.58616579	0.08114149	OK	1.84E-218
CEMP1	1.48E-09	5.933446208	0.01380449	OK	3.58E-09
PDPK1	1.58E-42	13.61740197	0.0349429	OK	5.68E-42
KCTD5	1.66E-51	15.05277977	0.03862383	OK	6.29E-51
SRRM2	1.71E-19	8.954441722	0.02295578	OK	4.96E-19
ELOB	0	121.974643	0.31362727	OK	0
PRSS21	4.40E-52	15.14008826	0.03879299	OK	1.68E-51
ZG16B	3.03E-09	5.815100293	0.01482123	OK	7.24E-09
FLYWCH2	1.32E-47	14.44619861	0.03707582	OK	4.93E-47
PKMYT1	0	133.8084092	0.3418046	OK	0
PAQR4	1.55E-62	16.64851815	0.04270307	OK	6.27E-62
TNFRSF12A	0	47.46094189	0.12198218	OK	0
HCFC1R1	0	153.0008785	0.3934325	OK	0
THOC6	2.07E-52	15.18951595	0.038983	OK	7.91E-52
MMP25	6.86E-13	7.08687816	0.01802366	OK	1.79E-12
IL32	0	45.29949159	0.11639779	OK	0
AC108134.3	1.42E-20	9.225111967	0.02360265	OK	4.20E-20
ZNF205	3.15E-05	4.0012232	0.01020774	OK	6.51E-05
MEFV	9.27E-19	8.765892388	0.02239001	OK	2.66E-18
ZNF263	0.00037595	3.36991869	0.00858368	OK	0.00073537
ZNF174	0.00032394	3.410737249	0.00868698	OK	0.00063583
NAA60	4.69E-09	5.741682146	0.01468805	OK	1.11E-08
CLUAP1	1.82E-58	16.07803954	0.04126067	OK	7.19E-58
DNASE1	7.72E-11	6.401023814	0.01634279	OK	1.92E-10
TRAP1	3.56E-24	10.07495224	0.02583052	OK	1.10E-23
ADCY9	1.28E-07	5.153780188	0.0131573	OK	2.91E-07
SRL	2.42E-11	6.576017886	0.01651451	OK	6.09E-11
GLIS2	4.17E-75	18.29956446	0.04692922	OK	1.78E-74
CORO7	3.50E-188	29.2347985	0.07509016	OK	2.12E-187
VASN	0	104.2934848	0.26811049	OK	0

NMRAL1	9.94E-15	7.651423867	0.01960073	OK	2.68E-14
HMOX2	1.56E-81	19.08886045	0.04901424	OK	6.90E-81
CDIP1	2.69E-05	4.038263299	0.01030567	OK	5.58E-05
MGRN1	1.94E-09	5.889125989	0.01506545	OK	4.66E-09
NUDT16L1	5.31E-26	10.48044529	0.02687548	OK	1.67E-25
ANKS3	3.52E-14	7.48720344	0.01916417	OK	9.40E-14
ROGDI	9.64E-66	17.08472292	0.04385279	OK	3.95E-65
GLYR1	1.18E-11	6.681237824	0.01710548	OK	3.01E-11
UBN1	1.52E-55	15.6557801	0.04018625	OK	5.89E-55
PPL	7.31E-25	10.22956757	0.02615852	OK	2.28E-24
NAGPA	4.92E-158	26.75847349	0.06872964	OK	2.78E-157
ALG1	4.49E-09	5.748851386	0.01469816	OK	1.07E-08
C16orf89	4.93E-20	9.09042356	0.02320758	OK	1.45E-19
EEF2KMT	4.69E-05	3.906015489	0.00996448	OK	9.61E-05
METTL22	4.06E-07	4.932487553	0.01260226	OK	9.07E-07
TMEM186	3.62E-06	4.486573781	0.01145413	OK	7.79E-06
PMM2	2.00E-101	21.34882696	0.0548158	OK	9.56E-101
CARHSP1	1.57E-228	32.25359387	0.08287455	OK	1.03E-227
USP7	1.38E-12	6.989225056	0.01789999	OK	3.58E-12
C16orf72	3.45E-27	10.73598084	0.02753421	OK	1.10E-26
AC087190.1	0.00028784	3.442819757	0.00877363	OK	0.00056645
GRIN2A	1.29E-57	15.95598244	0.04082214	OK	5.09E-57
ATF7IP2	4.00E-19	8.860147895	0.02270238	OK	1.16E-18
EMP2	0	143.6889686	0.36947882	OK	0
NUBP1	7.11E-265	34.74927963	0.08929165	OK	4.90E-264
TVP23A	4.62E-231	32.43353082	0.08322978	OK	3.04E-230
CIITA	0	64.6514701	0.16615464	OK	0
DEXI	0	46.24466917	0.11884615	OK	0
RMI2	2.27E-195	29.79503131	0.07616843	OK	1.39E-194
SOCS1	2.30E-93	20.46296478	0.05253743	OK	1.07E-92
LITAF	0	147.6692257	0.37972143	OK	0
SNN	0	47.88264038	0.12306051	OK	0
TXNDC11	9.89E-12	6.707565214	0.01716949	OK	2.52E-11
AC007613.1	4.06E-06	4.462143421	0.01136413	OK	8.71E-06
ZC3H7A	2.49E-05	4.056364285	0.0103574	OK	5.18E-05
RSL1D1	0	64.52121953	0.16587088	OK	0
GSPT1	0	48.61757348	0.1249658	OK	0
TNFRSF17	6.73E-07	4.832826609	0.01154929	OK	1.49E-06
SNX29	0	45.70148217	0.11745365	OK	0
CPPED1	4.13E-241	33.13860763	0.08514624	OK	2.76E-240
ERCC4	6.90E-08	5.267924298	0.01346739	OK	1.58E-07
MKL2	3.49E-08	5.391851837	0.01378213	OK	8.08E-08
MIR193BHG	1.06E-46	14.3022	0.03664715	OK	3.92E-46
BFAR	1.20E-61	16.52550279	0.04242438	OK	4.84E-61
PDXDC1	1.19E-05	4.225585669	0.01079116	OK	2.51E-05
NTAN1	0	77.2324603	0.19855803	OK	0
C16orf45	0	60.04687526	0.15431152	OK	0
MYH11	0	282.1487582	0.72558856	OK	0
FOPNL	1.39E-10	6.310998918	0.01615252	OK	3.44E-10
XYLT1	4.64E-07	4.906526602	0.0125217	OK	1.03E-06
RPS15A	0	176.7573829	0.45446057	OK	0
ARL6IP1	0	65.34764903	0.16799318	OK	0
SMG1	3.81E-11	6.508019154	0.01666195	OK	9.56E-11

COQ7	1.49E-05	4.174817701	0.01065795	OK	3.13E-05
ITPRIPL2	5.35E-43	13.69622654	0.03514861	OK	1.93E-42
AC099518.5	1.37E-09	5.946843375	0.01521216	OK	3.30E-09
SYT17	2.83E-46	14.23371143	0.03648089	OK	1.04E-45
GDE1	1.30E-77	18.61116509	0.04778612	OK	5.66E-77
VPS35L	8.34E-09	5.643254613	0.01443269	OK	1.97E-08
AC002550.2	2.73E-06	4.546293725	0.01072896	OK	5.91E-06
KNOP1	2.06E-17	8.409323383	0.02154807	OK	5.81E-17
IQCK	3.19E-19	8.885152416	0.02275129	OK	9.24E-19
GPRC5B	5.34E-65	16.98457126	0.04349991	OK	2.18E-64
ACSM5	3.19E-27	10.7430835	0.02745945	OK	1.02E-26
ACSM3	5.40E-06	4.400541389	0.01119972	OK	1.15E-05
THUMPD1	4.94E-42	13.53381417	0.03473239	OK	1.77E-41
AC004381.1	1.51E-50	14.90564511	0.03095198	OK	5.73E-50
DCUN1D3	1.82E-07	5.086656579	0.01299983	OK	4.12E-07
LYRM1	4.30E-70	17.65955998	0.04533183	OK	1.80E-69
TMEM159	0	60.08363157	0.15444017	OK	0
METTL9	1.41E-204	30.49745831	0.07836103	OK	8.82E-204
IGSF6	0	241.318042	0.62057277	OK	0
OTOA	0	95.87555878	0.24642812	OK	0
UQCRC2	0	40.73343449	0.10468909	OK	0
MOSMO	1.55E-23	9.929716423	0.02545546	OK	4.76E-23
CDR2	8.35E-12	6.732382524	0.0172247	OK	2.13E-11
NPIPB5	1.22E-06	4.713813564	0.01204123	OK	2.66E-06
HS3ST2	7.20E-150	26.04741442	0.06673587	OK	3.97E-149
AC008915.2	9.25E-06	4.282280924	0.01091893	OK	1.96E-05
GGA2	7.18E-157	26.6582679	0.06847566	OK	4.04E-156
NDUFAB1	0	77.50319871	0.1992597	OK	0
DCTN5	0.00035387	3.386564399	0.00863085	OK	0.00069341
PLK1	0	111.1503713	0.28370509	OK	0
PRKCB	0	45.85870007	0.11783756	OK	0
RBBP6	3.41E-20	9.130390793	0.02340719	OK	1.01E-19
TNRC6A	5.36E-46	14.18897565	0.03641301	OK	1.97E-45
ARHGAP17	1.19E-12	7.009726317	0.01794753	OK	3.10E-12
LCMT1	3.36E-45	14.05974005	0.03608125	OK	1.23E-44
NSMCE1	8.84E-98	20.95285765	0.05381152	OK	4.17E-97
AC106739.2	4.20E-06	4.454935593	0.01135519	OK	9.00E-06
IL4R	2.12E-06	4.599323735	0.01175023	OK	4.61E-06
IL21R	4.47E-65	16.99500117	0.0435734	OK	1.83E-64
GTF3C1	0.00035628	3.384699276	0.00862635	OK	0.0006977
GSG1L	1.47E-58	16.0912562	0.04112166	OK	5.81E-58
XPO6	8.05E-11	6.394639854	0.01636304	OK	2.00E-10
CLN3	2.12E-06	4.599539218	0.0117253	OK	4.61E-06
APOBR	0	54.23694659	0.13934553	OK	0
IL27	7.67E-17	8.253814454	0.02104776	OK	2.14E-16
NUPR1	0	155.8690621	0.40080568	OK	0
SGF29	4.49E-19	8.847164675	0.02267326	OK	1.30E-18
SULT1A1	0	45.54468298	0.11703679	OK	0
ATXN2L	2.48E-06	4.566676764	0.01166599	OK	5.37E-06
TUFM	0	55.68444071	0.14314295	OK	0
RABEP2	0.00020779	3.529993113	0.00899634	OK	0.00041237
NFATC2IP	0.00018855	3.55561244	0.009068	OK	0.00037514
SPNS1	2.93E-208	30.77385233	0.07906111	OK	1.85E-207

LAT	2.86E-222	31.80392772	0.0816646	OK	1.85E-221
AC009093.2	9.94E-22	9.505650562	0.02429081	OK	2.99E-21
SPN	2.19E-237	32.87907713	0.08444076	OK	1.45E-236
QPRT	3.01E-98	21.00399251	0.05389785	OK	1.43E-97
C16orf54	6.85E-88	19.83904713	0.050921	OK	3.11E-87
KIF22	2.88E-123	23.5830254	0.06056884	OK	1.47E-122
MAZ	2.46E-186	29.08914068	0.07473726	OK	1.48E-185
PRRT2	3.10E-75	18.31573891	0.04698774	OK	1.33E-74
MVP	1.08E-86	19.69996385	0.05058914	OK	4.87E-86
CDIPT	1.38E-85	19.57039417	0.05025478	OK	6.22E-85
SEZ6L2	1.52E-07	5.121107661	0.01302321	OK	3.45E-07
ASPHD1	0	52.77375992	0.13525955	OK	0
TMEM219	0	78.17952025	0.20099917	OK	0
HIRIP3	1.54E-123	23.60958685	0.06062896	OK	7.87E-123
AC093512.2	6.09E-11	6.437175331	0.01558717	OK	1.52E-10
ALDOA	0	53.89252587	0.1385162	OK	0
PPP4C	0	94.19741396	0.24219379	OK	0
YPEL3	0	48.93675988	0.12578748	OK	0
MAPK3	3.27E-40	13.22200608	0.03392667	OK	1.16E-39
CORO1A	0	179.0014117	0.46029911	OK	0
AC012645.3	7.00E-05	3.808135192	0.00968582	OK	0.00014237
BOLA2B	2.87E-05	4.023483287	0.01025477	OK	5.93E-05
CD2BP2	8.82E-136	24.77272117	0.06363677	OK	4.70E-135
TBC1D10B	7.17E-06	4.338458629	0.01107824	OK	1.53E-05
MYLPF	2.18E-08	5.475545311	0.01393226	OK	5.09E-08
SEPT1	6.89E-50	14.80408462	0.03799009	OK	2.60E-49
ZNF771	3.43E-47	14.38042939	0.0368717	OK	1.27E-46
DCTPP1	1.22E-226	32.11843077	0.0825228	OK	7.96E-226
SEPHS2	3.25E-278	35.62153355	0.09153729	OK	2.28E-277
ITGAL	4.99E-65	16.98856106	0.04358469	OK	2.04E-64
ZNF688	1.86E-15	7.864023264	0.02014756	OK	5.07E-15
PRR14	0.00013639	3.639870174	0.00928272	OK	0.00027338
FBRS	0.00010115	3.71611791	0.00948016	OK	0.00020413
PHKG2	6.36E-64	16.83862844	0.04322352	OK	2.59E-63
CCDC189	1.93E-10	6.259975487	0.01597405	OK	4.75E-10
BCL7C	0	51.25004374	0.13173637	OK	0
CTF1	2.93E-49	14.70642273	0.03771085	OK	1.10E-48
ORAI3	5.57E-167	27.51653041	0.07068564	OK	3.21E-166
HSD3B7	0	60.24692841	0.15484187	OK	0
STX4	0	43.58283401	0.11201184	OK	0
ZNF668	6.91E-10	6.057570901	0.01549403	OK	1.68E-09
PRSS53	1.65E-08	5.525247219	0.01348587	OK	3.85E-08
VKORC1	0	78.28408036	0.20126774	OK	0
BCKDK	0	45.36811282	0.11660143	OK	0
KAT8	1.33E-11	6.664020531	0.01706259	OK	3.38E-11
PRSS36	3.09E-66	17.15091943	0.04398548	OK	1.27E-65
FUS	1.51E-68	17.45729715	0.04482466	OK	6.29E-68
PYCARD	0	225.3905558	0.57961885	OK	0
PYCARD-AS1	0.00031207	3.420900809	0.00869853	OK	0.00061298
ITGAM	0	57.97353494	0.14899477	OK	0
ITGAX	0	142.6036284	0.36666908	OK	0
ITGAD	4.98E-25	10.26661747	0.02609618	OK	1.56E-24
TGFB1I1	0	214.7809803	0.55230372	OK	0

ZNF720	1.05E-06	4.743115596	0.01211928	OK	2.31E-06
ZNF267	0	94.71750349	0.24351967	OK	0
SHCBP1	0	80.1450585	0.20549573	OK	0
VPS35	0	65.86802454	0.16933447	OK	0
ORC6	0	54.29416508	0.13936462	OK	0
C16orf87	1.19E-120	23.32647257	0.05991085	OK	6.07E-120
GPT2	9.43E-07	4.765371652	0.01209732	OK	2.08E-06
DNAJA2	2.81E-189	29.32092856	0.07533575	OK	1.70E-188
NETO2	3.22E-145	25.63360685	0.06581042	OK	1.76E-144
ITFG1	3.74E-27	10.72838995	0.02751477	OK	1.19E-26
SIAH1	6.24E-19	8.810282903	0.02258119	OK	1.80E-18
N4BP1	5.22E-27	10.69763315	0.02743577	OK	1.66E-26
CBLN1	0	39.38011008	0.10031989	OK	0
AC007614.1	5.29E-16	8.019947871	0.02001519	OK	1.46E-15
ZNF423	8.58E-08	5.227685171	0.01329531	OK	1.96E-07
CNEP1R1	2.03E-27	10.78493218	0.0276526	OK	6.48E-27
HEATR3	6.40E-37	12.63937137	0.03241139	OK	2.21E-36
TENT4B	5.36E-08	5.314227633	0.01358965	OK	1.23E-07
ADCY7	9.29E-154	26.38849722	0.06776112	OK	5.18E-153
BRD7	1.96E-167	27.55431408	0.07079237	OK	1.13E-166
AC007493.1	4.73E-05	3.904268111	0.00887687	OK	9.68E-05
NKD1	1.07E-15	7.932613447	0.02027828	OK	2.94E-15
SNX20	1.06E-187	29.19708441	0.07497481	OK	6.37E-187
NOD2	7.95E-24	9.995761491	0.02558126	OK	2.46E-23
CYLD	4.18E-35	12.3064179	0.0315732	OK	1.43E-34
HNRNPA1P48	4.86E-08	5.33181547	0.01362897	OK	1.12E-07
CHD9	4.66E-09	5.742607656	0.01469536	OK	1.11E-08
RBL2	4.97E-07	4.89287653	0.01250724	OK	1.11E-06
AKTIP	1.34E-189	29.34614625	0.07537284	OK	8.13E-189
RPGRIP1L	4.20E-05	3.932873262	0.01001218	OK	8.62E-05
FTO	2.51E-07	5.025571357	0.01284009	OK	5.65E-07
IRX3	8.69E-18	8.510069947	0.02172514	OK	2.47E-17
CRNDE	0	74.07694194	0.19040357	OK	0
IRX5	1.23E-07	5.161473885	0.01314021	OK	2.79E-07
MMP2	0	63.73810654	0.16376961	OK	0
MMP2-AS1	4.86E-26	10.48880574	0.02684307	OK	1.53E-25
LPCAT2	0	96.4975264	0.24809549	OK	0
AC007336.1	2.57E-22	9.645603886	0.02468284	OK	7.78E-22
CES1	1.36E-174	28.145434	0.07227108	OK	8.01E-174
GNAO1	3.86E-20	9.11701541	0.02316888	OK	1.14E-19
AMFR	6.98E-09	5.673932487	0.01451521	OK	1.65E-08
AC092140.1	5.47E-05	3.868804195	0.00983279	OK	0.00011175
NUDT21	1.87E-67	17.31306801	0.04444839	OK	7.74E-67
OGFOD1	7.24E-13	7.07946468	0.01812222	OK	1.89E-12
MT3	0.00012547	3.661305886	0.00903298	OK	0.00025197
MT2A	0	73.86364905	0.18989537	OK	0
MT1E	0	91.23957065	0.23456612	OK	0
MT1M	0	101.6050278	0.26119851	OK	0
MT1A	0	80.80066508	0.20752876	OK	0
MT1F	0	75.59033892	0.19429803	OK	0
MT1G	0	112.2433467	0.28839564	OK	0
MT1H	0	72.28087774	0.18555769	OK	0
MT1X	0	91.40302518	0.23499837	OK	0

NUP93	4.38E-11	6.486849851	0.01659855	OK	1.10E-10
HERPUD1	0	171.1669909	0.44015369	OK	0
CETP	1.24E-26	10.61716571	0.02698898	OK	3.93E-26
CPNE2	2.26E-76	18.45780194	0.04738593	OK	9.73E-76
FAM192A	9.02E-63	16.68090474	0.04282335	OK	3.65E-62
ARL2BP	5.78E-89	19.96300611	0.05126603	OK	2.64E-88
CCL22	6.01E-44	13.85406891	0.03396951	OK	2.18E-43
CX3CL1	2.99E-228	32.23351951	0.08277149	OK	1.96E-227
CCL17	2.18E-101	21.34470349	0.0540526	OK	1.04E-100
CIAPIN1	1.80E-21	9.44392238	0.02420678	OK	5.38E-21
COQ9	1.55E-05	4.165422154	0.01063481	OK	3.26E-05
POLR2C	1.47E-209	30.87082846	0.07931579	OK	9.33E-209
DOK4	3.96E-23	9.835540237	0.0251667	OK	1.21E-22
CCDC102A	5.13E-278	35.6087429	0.09145063	OK	3.60E-277
ADGRG1	1.52E-12	6.975863604	0.0175775	OK	3.94E-12
KIFC3	7.02E-156	26.5726923	0.06823957	OK	3.95E-155
USB1	1.21E-59	16.24500238	0.04169713	OK	4.83E-59
MMP15	7.61E-16	7.975170221	0.01998793	OK	2.09E-15
CFAP20	4.18E-18	8.594588656	0.0220249	OK	1.19E-17
CSNK2A2	6.57E-51	14.96131557	0.03840148	OK	2.49E-50
CCDC113	7.89E-05	3.778429285	0.00958501	OK	0.0001601
GINS3	3.60E-06	4.487840181	0.01140202	OK	7.75E-06
NDRG4	1.04E-07	5.191684908	0.012836	OK	2.38E-07
CNOT1	1.20E-27	10.83275707	0.02778171	OK	3.85E-27
SLC38A7	6.25E-44	13.85122256	0.03551562	OK	2.27E-43
GOT2	2.36E-07	5.037101045	0.01287244	OK	5.33E-07
CDH11	0	52.98547813	0.13612855	OK	0
CDH5	0	50.46020737	0.12851533	OK	0
LINC00920	0.0004215	3.338277266	0.0084737	OK	0.0008222
BEAN1-AS1	2.34E-08	5.462816447	0.01262602	OK	5.46E-08
TK2	4.93E-33	11.91516824	0.03056389	OK	1.66E-32
CKLF	0	142.8924713	0.36743344	OK	0
CMTM3	0	100.0124604	0.2571419	OK	0
DYNC1LI2	2.50E-223	31.88030629	0.08191634	OK	1.62E-222
NAE1	5.81E-43	13.69017015	0.03512807	OK	2.10E-42
RRAD	0	103.8689957	0.26702659	OK	0
FAM96B	0	77.72632006	0.1998336	OK	0
CES2	5.51E-05	3.866944526	0.00986647	OK	0.00011256
CBFB	1.15E-14	7.632346908	0.01954833	OK	3.10E-14
C16orf70	4.91E-12	6.809151407	0.01742155	OK	1.26E-11
B3GNT9	1.75E-10	6.274542183	0.01602496	OK	4.33E-10
TRADD	1.49E-77	18.60418096	0.04777039	OK	6.44E-77
HSF4	7.76E-05	3.782731659	0.00962422	OK	0.0001574
NOL3	1.79E-264	34.72272447	0.08920485	OK	1.23E-263
E2F4	5.13E-19	8.832284301	0.02263845	OK	1.48E-18
TMEM208	0	42.56873348	0.10940813	OK	0
FHOD1	4.33E-15	7.757433148	0.01985873	OK	1.18E-14
TPPP3	0	40.31731326	0.10350412	OK	0
ATP6V0D1	0	116.0519391	0.29840319	OK	0
AGRP	3.28E-195	29.78260462	0.07635442	OK	2.02E-194
AC027682.6	1.64E-11	6.633596688	0.01694006	OK	4.15E-11
RIPOR1	0.00015076	3.613998256	0.00921729	OK	0.00030166
AC027682.4	1.92E-09	5.891111728	0.01428357	OK	4.61E-09

CTCF	1.35E-17	8.458736351	0.0216786	OK	3.82E-17
ACD	8.59E-17	8.240255288	0.02111037	OK	2.40E-16
PARD6A	1.25E-27	10.82961312	0.02776615	OK	3.99E-27
ENKD1	2.96E-05	4.015654736	0.01023662	OK	6.13E-05
C16orf86	2.32E-07	5.040605788	0.01287883	OK	5.24E-07
THAP11	5.74E-71	17.7727742	0.04563027	OK	2.41E-70
NUTF2	0	41.83162781	0.10751234	OK	0
AC040162.1	3.79E-05	3.957089389	0.01008252	OK	7.80E-05
PSMB10	0	46.11231238	0.11851293	OK	0
LCAT	3.12E-11	6.53804482	0.01669095	OK	7.84E-11
SLC12A4	2.85E-18	8.638375896	0.02212304	OK	8.15E-18
DPEP3	5.65E-08	5.304422823	0.01272984	OK	1.30E-07
DPEP2	0	39.99092487	0.10273839	OK	0
DUS2	2.62E-05	4.045048938	0.01031066	OK	5.43E-05
DDX28	5.18E-08	5.320376729	0.01359189	OK	1.19E-07
PLA2G15	0	48.23468044	0.12395402	OK	0
SLC7A6OS	3.19E-08	5.407890761	0.01382878	OK	7.40E-08
SMPD3	5.84E-08	5.298546659	0.01319389	OK	1.34E-07
CDH1	1.35E-57	15.95342927	0.03784147	OK	5.30E-57
CHTF8	3.60E-11	6.516479804	0.0166816	OK	9.04E-11
SNTB2	8.80E-102	21.38716122	0.05491451	OK	4.21E-101
VPS4A	2.15E-24	10.12459918	0.02596211	OK	6.67E-24
NIP7	3.96E-28	10.93407972	0.02803944	OK	1.27E-27
TERF2	1.15E-12	7.015479974	0.01796349	OK	2.98E-12
CYB5B	6.70E-09	5.680952348	0.01453434	OK	1.58E-08
NQO1	1.97E-274	35.3764527	0.09087328	OK	1.37E-273
NOB1	2.41E-43	13.75398182	0.03529315	OK	8.72E-43
WWP2	2.30E-06	4.582291132	0.01170474	OK	4.99E-06
EXOSC6	6.49E-28	10.88915445	0.02792848	OK	2.08E-27
AARS	2.66E-05	4.040891008	0.01031191	OK	5.52E-05
DDX19B	1.81E-11	6.619245122	0.01694304	OK	4.56E-11
IL34	0	49.0027895	0.12583892	OK	0
MTSS1L	1.16E-06	4.723887684	0.01202659	OK	2.54E-06
VAC14	1.48E-224	31.96882723	0.08211075	OK	9.63E-224
CALB2	0.0001326	3.647119369	0.00848378	OK	0.00026589
AP1G1	9.68E-12	6.710749384	0.01718119	OK	2.47E-11
IST1	3.50E-13	7.179521322	0.01838907	OK	9.18E-13
PMFBP1	3.06E-78	18.68867449	0.04791169	OK	1.33E-77
ZFHX3	3.08E-73	18.06368188	0.04638259	OK	1.31E-72
HCCAT5	7.29E-09	5.666536958	0.01443349	OK	1.72E-08
AC116667.2	1.24E-05	4.21692219	0.01071376	OK	2.61E-05
AC140912.1	1.64E-91	20.25379041	0.05131706	OK	7.59E-91
PSMD7	0	56.85646123	0.146157	OK	0
GLG1	8.26E-101	21.28244298	0.05465945	OK	3.94E-100
RFWD3	0.00027893	3.451311982	0.00878127	OK	0.00054931
MLKL	7.46E-20	9.045413251	0.02317412	OK	2.18E-19
ZNRF1	7.71E-17	8.253162863	0.02113956	OK	2.15E-16
ZFP1	9.82E-08	5.202802406	0.0132864	OK	2.24E-07
BCAR1	1.88E-169	27.72234392	0.07117826	OK	1.09E-168
CFDP1	1.17E-98	21.04898602	0.05406115	OK	5.54E-98
TMEM170A	2.26E-60	16.3475449	0.04196418	OK	9.06E-60
GABARAPL2	0	95.40221677	0.24529341	OK	0
ADAT1	0.00023006	3.502965387	0.00892684	OK	0.00045542

KARS	3.96E-19	8.861064064	0.02271175	OK	1.15E-18
TERF2IP	0	42.40517236	0.10898926	OK	0
ADAMTS18	7.89E-234	32.62925905	0.07605329	OK	5.21E-233
NUDT7	3.49E-31	11.55475003	0.02962066	OK	1.15E-30
VAT1L	6.80E-24	10.01126409	0.02558252	OK	2.10E-23
WWOX	6.18E-06	4.371105367	0.01116071	OK	1.32E-05
AC084064.1	5.25E-05	3.878794661	0.00980993	OK	0.00010733
MAF	0	105.9273699	0.27234393	OK	0
CMC2	0	45.26398923	0.11633445	OK	0
CENPN	0	45.27127139	0.11622514	OK	0
ATMIN	8.17E-11	6.392325116	0.01636038	OK	2.03E-10
GCSH	0	38.77388054	0.09963494	OK	0
CMIP	0	48.75829461	0.12530506	OK	0
AC099524.1	0.00044528	3.322994404	0.00816638	OK	0.00086675
PLCG2	6.57E-21	9.307155027	0.02386173	OK	1.95E-20
MPHOSPH6	8.22E-87	19.71372248	0.05061897	OK	3.72E-86
CDH13	0	100.4734747	0.25828961	OK	0
AC009119.1	2.07E-06	4.604293854	0.01169187	OK	4.51E-06
HSBP1	0	91.3936502	0.23498519	OK	0
OSGIN1	1.20E-05	4.223290767	0.01077561	OK	2.54E-05
MBTPS1	1.07E-35	12.41618369	0.03185453	OK	3.66E-35
HSDL1	2.10E-05	4.096317455	0.01045296	OK	4.38E-05
DNAAF1	4.62E-16	8.036657869	0.02049819	OK	1.27E-15
WFDC1	0	79.49402589	0.20428145	OK	0
COTL1	0	259.5289945	0.6674193	OK	0
USP10	1.21E-22	9.722795249	0.02492757	OK	3.67E-22
CRISPLD2	0	69.98870252	0.17971881	OK	0
ZDHHC7	9.84E-22	9.506669551	0.0243668	OK	2.96E-21
KIAA0513	3.57E-07	4.957249191	0.01265606	OK	8.01E-07
LINC00311	0.00022068	3.514035844	0.00665993	OK	0.00043732
GINS2	0	69.93015119	0.17934394	OK	0
C16orf74	6.19E-172	27.92746636	0.07160686	OK	3.61E-171
EMC8	6.04E-56	15.71428133	0.04033575	OK	2.35E-55
COX4I1	0	181.7156187	0.46725784	OK	0
IRF8	0	112.0118334	0.28798948	OK	0
AC092723.3	3.17E-23	9.857716051	0.02182211	OK	9.73E-23
FOXF1	1.26E-27	10.8282348	0.02747688	OK	4.05E-27
FOXC2-AS1	3.86E-09	5.774642719	0.01439549	OK	9.19E-09
FOXC2	0	84.12500943	0.21624556	OK	0
FOXL1	1.78E-10	6.272115271	0.01601636	OK	4.40E-10
AC009154.2	0.00050163	3.289610625	0.00823264	OK	0.00097291
MAP1LC3B	0	117.3449104	0.30172995	OK	0
SLC7A5	1.17E-80	18.98321444	0.04871451	OK	5.16E-80
BANP	1.08E-11	6.695114324	0.01713717	OK	2.74E-11
AC134312.5	0.00038871	3.360709908	0.00830862	OK	0.00075986
ZFPM1	3.87E-31	11.5459323	0.02961702	OK	1.28E-30
ZC3H18	2.54E-11	6.568376286	0.01681473	OK	6.41E-11
CYBA	0	304.7270373	0.78366643	OK	0
MVD	1.19E-05	4.226760284	0.01079108	OK	2.50E-05
SNAI3	1.82E-25	10.36310944	0.02653668	OK	5.72E-25
RNF166	0	41.74780972	0.10728006	OK	0
CTU2	0.00046367	3.311692287	0.00843614	OK	0.00090134
PIEZO1	4.20E-39	13.02878162	0.03340264	OK	1.47E-38

AC138028.4	3.27E-17	8.354879992	0.01917356	OK	9.19E-17
CDT1	0	86.03479812	0.22043377	OK	0
APRT	0	103.9848922	0.26736925	OK	0
GALNS	2.03E-37	12.72951012	0.03264013	OK	7.02E-37
TRAPPC2L	1.29E-282	35.9047464	0.09226858	OK	9.09E-282
CBFA2T3	4.06E-73	18.04835466	0.04621847	OK	1.72E-72
AC092384.1	4.31E-05	3.926223054	0.0097771	OK	8.85E-05
AC135782.1	4.66E-08	5.339392725	0.0133057	OK	1.08E-07
ACSF3	3.23E-06	4.510886927	0.01152178	OK	6.97E-06
AC009113.1	0.00028194	3.448422167	0.00877409	OK	0.00055503
ANKRD11	2.74E-17	8.375827668	0.02146748	OK	7.71E-17
AC137932.3	2.45E-11	6.573784642	0.01674394	OK	6.18E-11
RPL13	0	167.3392445	0.43016439	OK	0
CPNE7	4.18E-14	7.464481255	0.01885975	OK	1.12E-13
CHMP1A	6.37E-12	6.771521096	0.01733845	OK	1.63E-11
SPATA33	7.03E-05	3.80713126	0.00971002	OK	0.00014293
SPATA2L	5.50E-80	18.90190292	0.04851945	OK	2.41E-79
FANCA	1.77E-27	10.7974055	0.02761448	OK	5.66E-27
TCF25	1.03E-56	15.82591097	0.04062912	OK	4.03E-56
DEF8	3.72E-120	23.27776476	0.05978733	OK	1.89E-119
GAS8	0.00035446	3.386104181	0.00861637	OK	0.00069439
DOC2B	5.01E-23	9.811721589	0.0239575	OK	1.53E-22
RFLNB	0	72.57054472	0.18650256	OK	0
VPS53	3.63E-05	3.96747066	0.01012604	OK	7.48E-05
GLOD4	2.25E-99	21.12693331	0.05426036	OK	1.07E-98
MRM3	0.00011318	3.687618325	0.00940397	OK	0.00022786
NXN	5.79E-14	7.421483676	0.0189673	OK	1.54E-13
TRARG1	1.69E-05	4.146627464	0.01051259	OK	3.53E-05
YWHAE	0	84.53911349	0.2173553	OK	0
CRK	2.74E-20	9.154090532	0.0234667	OK	8.08E-20
MYO1C	1.84E-123	23.60191729	0.06061599	OK	9.43E-123
PITPNA-AS1	1.84E-19	8.946318361	0.02292336	OK	5.34E-19
PITPNA	0	43.85772817	0.1127149	OK	0
SLC43A2	0	91.47094244	0.23515607	OK	0
SCARF1	8.86E-62	16.54389275	0.04241689	OK	3.57E-61
RILP	3.46E-162	27.11304016	0.06964494	OK	1.97E-161
PRPF8	3.32E-11	6.528565099	0.0167151	OK	8.34E-11
MIR22HG	5.96E-247	33.54165226	0.08618824	OK	4.00E-246
WDR81	2.98E-29	11.16626295	0.02861336	OK	9.68E-29
SERPINF2	1.18E-120	23.32700948	0.05980645	OK	6.00E-120
SERPINF1	0	85.5142944	0.21982932	OK	0
SMYD4	1.60E-05	4.158494256	0.01061149	OK	3.36E-05
RPA1	5.61E-10	6.091069573	0.0155828	OK	1.37E-09
HIC1	1.36E-193	29.65739809	0.07613972	OK	8.34E-193
SMG6	0.00025163	3.479018677	0.00887138	OK	0.00049705
TSR1	2.79E-11	6.554653977	0.01677748	OK	7.02E-11
MNT	2.85E-28	10.96399682	0.02810942	OK	9.16E-28
METTL16	1.93E-05	4.116300315	0.01050752	OK	4.02E-05
PAFAH1B1	1.48E-53	15.36180145	0.03943503	OK	5.67E-53
ASPA	1.94E-16	8.14224816	0.02059553	OK	5.38E-16
CTNS	9.61E-20	9.01762543	0.02310023	OK	2.80E-19
TAX1BP3	5.44E-77	18.53445444	0.04758824	OK	2.35E-76
EMC6	0	41.65552438	0.10705889	OK	0

ITGAE	2.84E-185	29.0050872	0.07451631	OK	1.70E-184
HASPIN	5.37E-06	4.401691453	0.01067841	OK	1.15E-05
NCBP3	3.03E-07	4.989559019	0.01275689	OK	6.80E-07
CAMKK1	0.00020133	3.538337658	0.00900615	OK	0.00039988
P2RX1	2.47E-13	7.226927098	0.0184599	OK	6.50E-13
ATP2A3	3.79E-11	6.508693059	0.01664341	OK	9.52E-11
ZZEF1	4.34E-07	4.919213453	0.01257127	OK	9.69E-07
CYB5D2	1.13E-13	7.332410293	0.0187766	OK	2.99E-13
UBE2G1	2.88E-34	12.1495552	0.03116815	OK	9.78E-34
SPNS3	0	40.62954496	0.10427293	OK	0
SPNS2	1.43E-77	18.60623937	0.04736556	OK	6.20E-77
ALOX15	1.26E-05	4.213224073	0.00984	OK	2.65E-05
ARRB2	0	189.9652194	0.48850075	OK	0
MED11	1.29E-37	12.76457656	0.0327517	OK	4.49E-37
CXCL16	0	212.3307922	0.54602457	OK	0
ZMYND15	0	40.00098265	0.10275769	OK	0
VMO1	0	143.5296001	0.36904025	OK	0
PSMB6	0	97.08880685	0.24963176	OK	0
PLD2	1.50E-05	4.172711267	0.0106461	OK	3.16E-05
MINK1	6.19E-05	3.838380151	0.0097866	OK	0.00012626
CHRNE	2.18E-201	30.25591234	0.07767649	OK	1.36E-200
C17orf107	4.76E-134	24.61144365	0.06317146	OK	2.53E-133
SLC25A11	2.78E-150	26.08390202	0.06700884	OK	1.53E-149
RNF167	1.05E-28	11.05422576	0.02835289	OK	3.38E-28
PFN1	0	210.903114	0.54231329	OK	0
SPAG7	4.45E-163	27.1884042	0.06985029	OK	2.54E-162
AC004771.5	0.00032442	3.410332744	0.00773011	OK	0.00063672
KIF1C	8.81E-103	21.49426031	0.05519007	OK	4.23E-102
AC087500.1	0.00028141	3.44892785	0.00876098	OK	0.00055409
SCIMP	0	50.31054204	0.12928893	OK	0
RABEP1	4.41E-17	8.319665473	0.02132054	OK	1.24E-16
RPAIN	6.34E-108	22.03720421	0.05660215	OK	3.10E-107
C1QBP	0	58.49598612	0.15037371	OK	0
DERL2	2.70E-90	20.11556843	0.05165917	OK	1.23E-89
NLRP1	7.30E-13	7.078297738	0.01812444	OK	1.90E-12
PIMREG	0	81.95890154	0.20826594	OK	0
TXNDC17	0	97.00712239	0.24942217	OK	0
MED31	2.72E-23	9.873075698	0.02531332	OK	8.36E-23
XAF1	5.89E-155	26.49266769	0.0680524	OK	3.30E-154
RNASEK	5.25E-132	24.41996511	0.06272243	OK	2.77E-131
C17orf49	2.80E-07	5.004284738	0.01278681	OK	6.30E-07
MIR497HG	0	40.75268755	0.10467699	OK	0
BCL6B	6.15E-103	21.51093803	0.05407372	OK	2.95E-102
SLC16A11	7.12E-08	5.262253816	0.01295488	OK	1.63E-07
CLEC10A	0	183.5785772	0.47197842	OK	0
ASGR2	1.77E-264	34.72309433	0.08909541	OK	1.22E-263
ASGR1	0	83.83229353	0.21549098	OK	0
ACADVL	0	59.89381822	0.15396625	OK	0
PHF23	6.13E-88	19.84461896	0.05095615	OK	2.79E-87
GABARAP	3.37E-19	8.879184211	0.0227559	OK	9.75E-19
CTDNEP1	0	55.89388516	0.14367995	OK	0
ELP5	1.91E-14	7.566891768	0.01938208	OK	5.13E-14
CLDN7	1.40E-65	17.06308299	0.04376136	OK	5.72E-65

SLC2A4	2.13E-138	25.01450266	0.0639376	OK	1.14E-137
EIF5A	0	93.42487136	0.24020909	OK	0
ACAP1	5.21E-303	37.18861162	0.09552463	OK	3.79E-302
TMEM256	9.53E-264	34.67454997	0.08910492	OK	6.56E-263
NLGN2	0.00039092	3.359147969	0.00854003	OK	0.00076403
TMEM102	9.84E-13	7.036802791	0.01800728	OK	2.56E-12
FGF11	4.90E-05	3.895363433	0.00985795	OK	0.00010033
ZBTB4	1.91E-09	5.891530321	0.01507428	OK	4.60E-09
POLR2A	1.40E-08	5.553987168	0.01420783	OK	3.28E-08
TNFSF12	0	101.0658985	0.25986148	OK	0
TNFSF13	3.54E-16	8.069084078	0.02062313	OK	9.79E-16
EIF4A1	0	61.64147663	0.15846345	OK	0
CD68	0	329.9610848	0.84857072	OK	0
AC016876.1	1.18E-10	6.335449498	0.01619761	OK	2.94E-10
MPDU1	4.21E-170	27.77614764	0.07134565	OK	2.45E-169
SOX15	2.99E-34	12.14669564	0.03111727	OK	1.01E-33
FXR2	2.11E-05	4.094844182	0.01044828	OK	4.40E-05
SAT2	0	47.19241288	0.12130123	OK	0
ATP1B2	0	38.65033024	0.09915319	OK	0
TP53	9.84E-22	9.506730518	0.02436272	OK	2.96E-21
WRAP53	2.91E-07	4.997188807	0.01275884	OK	6.54E-07
KDM6B	0	52.28102309	0.13437536	OK	0
TMEM88	1.57E-14	7.592277969	0.01935614	OK	4.22E-14
NAA38	0	48.05472796	0.12351881	OK	0
CHD3	1.94E-24	10.13434954	0.02598522	OK	6.04E-24
TRAPPC1	0	66.48486873	0.17092129	OK	0
ALOX15B	5.45E-41	13.35616063	0.03406224	OK	1.94E-40
PER1	1.64E-52	15.20485119	0.03902744	OK	6.26E-52
VAMP2	0	71.41251304	0.18359306	OK	0
TMEM107	3.03E-75	18.31701733	0.04702747	OK	1.30E-74
BORCS6	3.44E-20	9.129636743	0.02339945	OK	1.01E-19
AURKB	0	177.4776193	0.45309093	OK	0
LINC00324	9.96E-49	14.62342573	0.03751095	OK	3.73E-48
RANGRF	2.60E-24	10.10583725	0.02591341	OK	8.07E-24
ARHGEF15	2.24E-156	26.61557792	0.06655032	OK	1.26E-155
RPL26	0	180.1870961	0.46328534	OK	0
NDEL1	9.61E-60	16.2592107	0.04173637	OK	3.83E-59
MYH10	0	257.00023	0.66089412	OK	0
CCDC42	3.93E-15	7.769655624	0.01968426	OK	1.07E-14
PIK3R6	2.81E-07	5.003585878	0.0127385	OK	6.33E-07
PIK3R5	7.14E-209	30.81967336	0.07915099	OK	4.52E-208
AC002091.2	7.97E-05	3.775973272	0.00960029	OK	0.00016167
NTN1	2.15E-71	17.82768012	0.04569896	OK	9.06E-71
STX8	8.35E-107	21.92014604	0.05630007	OK	4.07E-106
GAS7	0	51.30703646	0.13185219	OK	0
SCO1	1.04E-07	5.191636665	0.01327365	OK	2.38E-07
TMEM220	1.47E-48	14.59670166	0.03745921	OK	5.51E-48
LINC00670	1.06E-06	4.742547497	0.01206414	OK	2.32E-06
MYOCD	0	39.80003234	0.1022275	OK	0
ARHGAP44	8.20E-09	5.646317151	0.01438177	OK	1.94E-08
ELAC2	8.15E-05	3.770497076	0.00961824	OK	0.00016512
COX10	3.25E-05	3.993979452	0.0101851	OK	6.71E-05
HS3ST3B1	1.71E-47	14.42842826	0.03696359	OK	6.38E-47

AC005224.3	8.37E-11	6.388625819	0.01629649	OK	2.08E-10
PMP22	0	61.81458304	0.15890614	OK	0
TRIM16	9.23E-08	5.214236376	0.01331421	OK	2.11E-07
AC015922.4	1.70E-08	5.519634264	0.01406055	OK	3.97E-08
LINC02087	0.00010505	3.706546907	0.00874218	OK	0.00021179
ADORA2B	6.39E-144	25.51696557	0.06548208	OK	3.48E-143
ZSWIM7	1.44E-112	22.5162378	0.05783259	OK	7.15E-112
TTC19	1.83E-28	11.00367282	0.02822135	OK	5.92E-28
NCOR1	5.31E-72	17.90586961	0.04597836	OK	2.24E-71
CENPV	1.47E-33	12.01547963	0.03080118	OK	4.97E-33
UBB	0	147.6603962	0.37967555	OK	0
AC093484.2	7.69E-05	3.784728564	0.00959456	OK	0.00015617
TRPV2	1.64E-142	25.38965889	0.06520516	OK	8.91E-142
LRRC75A	3.81E-82	19.16248879	0.04920361	OK	1.69E-81
ZNF287	0.00045593	3.316396627	0.00842648	OK	0.00088685
CCDC144A	1.91E-05	4.118091837	0.01050693	OK	3.99E-05
TNFRSF13B	1.48E-07	5.125916375	0.01263144	OK	3.36E-07
MPRIP	1.26E-178	28.47327419	0.07314146	OK	7.42E-178
FLCN	1.33E-05	4.200495543	0.01072232	OK	2.80E-05
COPS3	4.23E-212	31.05964275	0.07980398	OK	2.69E-211
NT5M	1.75E-12	6.95623914	0.01771779	OK	4.52E-12
RASD1	5.56E-138	24.97608834	0.06407773	OK	2.99E-137
PEMT	9.75E-11	6.36522665	0.01628822	OK	2.42E-10
SREBF1	7.61E-99	21.06931006	0.05409147	OK	3.61E-98
ATPAF2	9.59E-51	14.93615146	0.03832436	OK	3.63E-50
DRG2	3.04E-07	4.988584875	0.01275365	OK	6.83E-07
ALKBH5	7.40E-12	6.749782812	0.01728264	OK	1.89E-11
FLII	2.07E-115	22.80437211	0.05857036	OK	1.04E-114
MIEF2	6.84E-11	6.419427464	0.01640958	OK	1.71E-10
SMCR8	6.57E-10	6.065632312	0.01551139	OK	1.60E-09
SHMT1	4.80E-153	26.32623728	0.06759956	OK	2.67E-152
PRPSAP2	6.45E-06	4.361761676	0.01113982	OK	1.37E-05
GRAP	8.79E-12	6.724804537	0.01716212	OK	2.24E-11
AC007952.4	2.72E-63	16.75244445	0.04298962	OK	1.10E-62
EPN2	1.31E-239	33.03425765	0.08485971	OK	8.72E-239
B9D1	7.52E-07	4.810734145	0.01227472	OK	1.66E-06
MAPK7	2.29E-09	5.862106378	0.01498551	OK	5.48E-09
MFAP4	0	266.5110611	0.68534884	OK	0
SLC47A1	0.00033257	3.403555671	0.00864816	OK	0.00065238
ALDH3A2	1.68E-45	14.1088489	0.03620572	OK	6.14E-45
ALDH3A1	0.00044475	3.32332711	0.0082241	OK	0.0008658
ULK2	1.26E-05	4.213178183	0.01075097	OK	2.65E-05
AKAP10	0.00039276	3.357850981	0.00855631	OK	0.00076735
SPECC1	2.21E-103	21.55838877	0.05536616	OK	1.06E-102
CCDC144NL-AS1	2.91E-241	33.1492272	0.08500215	OK	1.94E-240
DHRS7B	3.74E-16	8.062534539	0.02065707	OK	1.03E-15
TMEM11	4.56E-22	9.586428064	0.02457588	OK	1.38E-21
MAP2K3	0	51.17403074	0.13152852	OK	0
KCNJ12	0.00018656	3.558395269	0.00870945	OK	0.00037129
WSB1	2.90E-70	17.68164966	0.04540155	OK	1.21E-69
LGALS9	0	175.7290442	0.45187858	OK	0
LYRM9	7.51E-40	13.1594309	0.03375456	OK	2.65E-39
TMEM97	1.66E-102	21.46478866	0.05506915	OK	7.96E-102

IFT20	1.51E-83	19.32958015	0.0496367	OK	6.75E-83
TNFAIP1	1.17E-05	4.228985168	0.01079494	OK	2.48E-05
POLDIP2	1.34E-31	11.6370347	0.02985016	OK	4.42E-31
TMEM199	1.63E-31	11.61996945	0.02980313	OK	5.40E-31
VTN	0.00014199	3.629497914	0.00914174	OK	0.00028435
SLC46A1	4.70E-19	8.841944377	0.02264287	OK	1.36E-18
UNC119	5.15E-65	16.98670035	0.04360579	OK	2.10E-64
PIGS	9.29E-08	5.213026029	0.01332755	OK	2.12E-07
ALDOC	4.16E-38	12.85265892	0.03296186	OK	1.45E-37
SPAG5	4.14E-259	34.36548144	0.0877809	OK	2.83E-258
KIAA0100	1.70E-20	9.205832827	0.02359701	OK	5.02E-20
SDF2	1.43E-74	18.23228881	0.04681563	OK	6.10E-74
SUPT6H	3.62E-13	7.174902661	0.01837654	OK	9.49E-13
RAB34	0	58.09708876	0.14934593	OK	0
RPL23A	0	178.7745659	0.45966779	OK	0
TRAF4	1.63E-113	22.61258198	0.05804167	OK	8.11E-113
ERAL1	2.93E-10	6.193957563	0.01584929	OK	7.21E-10
FLOT2	1.31E-08	5.565353097	0.01423192	OK	3.07E-08
PIPOX	6.66E-09	5.68178802	0.01448468	OK	1.58E-08
NUFIP2	0	46.69170658	0.12001014	OK	0
TAOK1	1.62E-14	7.588243957	0.01943999	OK	4.35E-14
ABHD15	6.88E-75	18.27226058	0.04688601	OK	2.94E-74
TP53I13	2.47E-57	15.9155147	0.04085497	OK	9.71E-57
GIT1	6.41E-05	3.829783169	0.00976813	OK	0.00013062
SSH2	1.49E-111	22.41241309	0.057557	OK	7.37E-111
NSRP1	3.76E-109	22.16470901	0.05693089	OK	1.85E-108
AC104984.3	6.09E-08	5.290651456	0.01339204	OK	1.40E-07
BLMH	1.90E-07	5.078527223	0.01298163	OK	4.30E-07
CPD	1.55E-86	19.68152068	0.0505223	OK	7.01E-86
GOSR1	1.91E-17	8.418565463	0.02157615	OK	5.37E-17
CRLF3	5.18E-112	22.4594235	0.05767175	OK	2.57E-111
ATAD5	4.50E-153	26.32874354	0.06756599	OK	2.50E-152
ADAP2	0	193.451964	0.49746118	OK	0
AC138207.5	0.00022056	3.514177256	0.0088971	OK	0.00043713
RNF135	0	38.99938973	0.10021669	OK	0
EVI2B	0	168.0367865	0.43209689	OK	0
EVI2A	0	104.3681994	0.26833135	OK	0
RAB11FIP4	1.08E-13	7.338629441	0.01872019	OK	2.86E-13
COPRS	0	87.06352522	0.2238406	OK	0
UTP6	6.63E-66	17.10654763	0.04391744	OK	2.72E-65
SUZ12	5.01E-127	23.94663149	0.06151049	OK	2.60E-126
RHOT1	1.12E-07	5.177974102	0.01324031	OK	2.56E-07
ZNF207	3.97E-28	10.9338787	0.02804607	OK	1.28E-27
PSMD11	8.19E-171	27.83494774	0.07151282	OK	4.78E-170
MYO1D	0	59.76895955	0.15360375	OK	0
TMEM98	0	92.44574131	0.23763217	OK	0
AC024610.2	1.31E-05	4.203448905	0.01056811	OK	2.77E-05
CCL2	0	117.8903438	0.30312244	OK	0
CCL7	0	164.5142113	0.42281222	OK	0
CCL8	0	92.33930531	0.23731796	OK	0
CCL13	0	67.51008022	0.17326943	OK	0
ZNF830	1.79E-07	5.090202689	0.0130129	OK	4.05E-07
NLE1	2.31E-06	4.581363955	0.01168949	OK	5.01E-06

SLFN5	2.96E-19	8.893597694	0.02279203	OK	8.57E-19
SLFN11	1.38E-28	11.02939625	0.0282834	OK	4.45E-28
SLFN12	0.00027583	3.454334261	0.00880476	OK	0.00054339
SLFN13	1.27E-40	13.29303555	0.03393976	OK	4.51E-40
SLFN12L	1.86E-07	5.082966261	0.01291824	OK	4.20E-07
LINC02001	6.81E-32	11.69438333	0.0299958	OK	2.26E-31
AP2B1	4.48E-20	9.100884509	0.02332907	OK	1.32E-19
TAF15	3.47E-41	13.3897937	0.03435954	OK	1.24E-40
MMP28	1.01E-32	11.85527441	0.03007378	OK	3.39E-32
CCL5	2.69E-224	31.95015559	0.0820733	OK	1.75E-223
RDM1	3.31E-34	12.13831766	0.02951937	OK	1.12E-33
CCL14	0	41.64957485	0.10595771	OK	0
CCL23	1.37E-133	24.56865877	0.0629377	OK	7.24E-133
CCL18	0	133.58052	0.34324526	OK	0
AC243829.4	3.30E-28	10.95050163	0.02800223	OK	1.06E-27
CCL3	0	229.3927433	0.58989869	OK	0
CCL4	0	172.2133837	0.44283068	OK	0
CCL3L1	0	169.088252	0.43477529	OK	0
CCL4L2	0	168.1351579	0.43232437	OK	0
ZNHIT3	5.66E-107	21.93781699	0.05634582	OK	2.76E-106
MYO19	1.51E-09	5.930373792	0.01515228	OK	3.64E-09
GGNBP2	1.27E-40	13.29309202	0.03411415	OK	4.51E-40
DHRS11	1.23E-11	6.675796522	0.01707438	OK	3.12E-11
AATF	3.33E-53	15.30889798	0.03929423	OK	1.28E-52
DUSP14	8.82E-54	15.39514377	0.03950702	OK	3.39E-53
DDX52	5.66E-07	4.867054635	0.01244149	OK	1.26E-06
MRPL45	5.05E-19	8.834018397	0.02264091	OK	1.46E-18
ARHGAP23	1.27E-19	8.987233109	0.02298778	OK	3.69E-19
EPOP	8.18E-09	5.646599065	0.01440539	OK	1.93E-08
AC006449.6	2.18E-08	5.47598074	0.01399349	OK	5.08E-08
CISD3	0	41.84144438	0.10753726	OK	0
PCGF2	4.56E-136	24.79928645	0.06366266	OK	2.43E-135
PSMB3	0	104.5609438	0.26884987	OK	0
CWC25	1.88E-10	6.263848444	0.01603437	OK	4.63E-10
C17orf98	2.68E-07	5.012924908	0.0117302	OK	6.03E-07
RPL23	0	144.1873207	0.3707403	OK	0
LASP1	8.92E-97	20.84244137	0.05352428	OK	4.20E-96
LINC00672	4.08E-16	8.051661027	0.02059918	OK	1.13E-15
PLXDC1	1.26E-234	32.68528651	0.08387733	OK	8.36E-234
RPL19	0	203.1415357	0.52225515	OK	0
CDK12	9.48E-08	5.209214559	0.01332208	OK	2.17E-07
STARD3	1.10E-65	17.07682797	0.043836	OK	4.52E-65
TCAP	3.20E-47	14.38528808	0.03677885	OK	1.19E-46
ERBB2	3.25E-82	19.17069592	0.04918512	OK	1.44E-81
MIEN1	0	47.31276024	0.12160844	OK	0
IKZF3	2.71E-28	10.96832885	0.02805517	OK	8.74E-28
GSDMA	2.28E-21	9.418915217	0.02407235	OK	6.82E-21
PSMD3	6.26E-115	22.75597579	0.05844952	OK	3.13E-114
THRA	0	48.87399684	0.12561273	OK	0
NR1D1	1.47E-15	7.893512221	0.0202078	OK	4.01E-15
CASC3	8.25E-08	5.234875723	0.0133855	OK	1.89E-07
CDC6	0	53.55061975	0.13655818	OK	0
RARA	2.41E-176	28.28819199	0.07266659	OK	1.42E-175

RARA-AS1	2.01E-11	6.603238857	0.01689986	OK	5.08E-11
TOP2A	0	205.2729542	0.52586259	OK	0
IGFBP4	0	80.1064184	0.20592196	OK	0
AC004585.1	9.42E-26	10.42614518	0.02642745	OK	2.96E-25
CCR7	0	43.63650438	0.11192613	OK	0
SMARCE1	2.33E-40	13.2477071	0.03399257	OK	8.24E-40
KRT222	4.75E-26	10.49096616	0.02534925	OK	1.50E-25
KRT25	6.49E-13	7.094611458	0.01688765	OK	1.69E-12
KRT10	0	63.60723079	0.1635198	OK	0
TMEM99	6.81E-51	14.95891981	0.03837038	OK	2.58E-50
KRT31	3.32E-13	7.186722908	0.01812474	OK	8.71E-13
KRT19	8.92E-41	13.31941698	0.03400376	OK	3.17E-40
KRT14	7.37E-29	11.08560253	0.02794607	OK	2.39E-28
KRT16	6.80E-121	23.35057743	0.0594832	OK	3.46E-120
KRT17	0	63.96185258	0.16425214	OK	0
EIF1	0	165.1920624	0.42469917	OK	0
JUP	1.19E-09	5.969830039	0.01526081	OK	2.87E-09
P3H4	4.16E-45	14.04453085	0.03599403	OK	1.52E-44
FKBP10	0	50.05523771	0.12860293	OK	0
NT5C3B	0	41.19817604	0.10586495	OK	0
ACLY	8.89E-07	4.777126354	0.01220683	OK	1.96E-06
CNP	7.34E-23	9.77313474	0.02505643	OK	2.24E-22
DNAJC7	8.78E-246	33.46142314	0.08598484	OK	5.89E-245
NKIRAS2	9.32E-40	13.14315055	0.0337233	OK	3.29E-39
RAB5C	0	119.6493946	0.30765786	OK	0
GHDC	1.73E-09	5.908361472	0.01511458	OK	4.16E-09
STAT5A	1.17E-35	12.4087981	0.03182922	OK	4.01E-35
STAT3	9.99E-33	11.85625764	0.03041916	OK	3.35E-32
CAVIN1	0	231.7256628	0.59589692	OK	0
ATP6V0A1	0	38.27180345	0.09834809	OK	0
NAGLU	1.45E-41	13.45450138	0.03452448	OK	5.19E-41
AC067852.2	6.02E-07	4.855113071	0.01239162	OK	1.34E-06
COASY	1.68E-14	7.583487276	0.01942305	OK	4.51E-14
MLX	4.05E-307	37.44202335	0.09621868	OK	2.95E-306
PSMC3IP	1.91E-16	8.144192213	0.02082636	OK	5.30E-16
TUBG1	3.69E-76	18.43120231	0.04731044	OK	1.59E-75
TUBG2	7.52E-61	16.41462475	0.04209465	OK	3.01E-60
PLEKHH3	2.73E-125	23.77937353	0.06105269	OK	1.41E-124
CCR10	2.50E-42	13.58381628	0.03478369	OK	8.99E-42
AC100793.2	1.38E-39	13.11347191	0.03361008	OK	4.86E-39
EZH1	2.96E-40	13.229722	0.03394358	OK	1.05E-39
RAMP2-AS1	3.87E-09	5.773963529	0.01455617	OK	9.23E-09
RAMP2	0	82.84976744	0.21261934	OK	0
VPS25	6.17E-116	22.85735822	0.05870844	OK	3.10E-115
COA3	0	39.15384078	0.10062487	OK	0
BECN1	1.94E-32	11.80045086	0.03027224	OK	6.49E-32
PSME3	7.89E-13	7.067539019	0.01809501	OK	2.05E-12
AOC3	0	79.63444179	0.20468274	OK	0
PTGES3L	4.06E-17	8.329518487	0.02112307	OK	1.14E-16
RPL27	0	155.266693	0.39923512	OK	0
IFI35	2.67E-154	26.43564555	0.06791015	OK	1.49E-153
VAT1	0	46.87200069	0.12046458	OK	0
RND2	4.05E-20	9.111729618	0.02329299	OK	1.19E-19

BRCA1	1.85E-173	28.05278765	0.0719536	OK	1.08E-172
NBR1	1.01E-39	13.13674712	0.03370565	OK	3.58E-39
TMEM106A	1.30E-157	26.72204909	0.06862709	OK	7.35E-157
ARL4D	1.51E-13	7.293123012	0.01867086	OK	4.00E-13
DHX8	2.19E-05	4.086918744	0.01043355	OK	4.56E-05
MEOX1	1.06E-156	26.64369838	0.06674768	OK	5.96E-156
SOST	0	78.9070864	0.20275209	OK	0
DUSP3	5.04E-88	19.85451833	0.05098572	OK	2.29E-87
CFAP97D1	3.92E-16	8.056602617	0.02043563	OK	1.08E-15
MPP2	7.73E-05	3.783515099	0.00958681	OK	0.00015692
AC007993.3	6.90E-38	12.81336586	0.03161744	OK	2.40E-37
PPY	0	98.84171874	0.2516599	OK	0
NAGS	3.68E-07	4.951383235	0.01262222	OK	8.25E-07
TMEM101	2.97E-13	7.2017458	0.01844401	OK	7.81E-13
LSM12	0	46.52767344	0.11958915	OK	0
G6PC3	6.46E-25	10.24151113	0.02625868	OK	2.01E-24
HDAC5	5.34E-15	7.730996602	0.01980431	OK	1.45E-14
C17orf53	2.58E-06	4.558193318	0.01144857	OK	5.59E-06
TMUB2	8.30E-05	3.765758326	0.00960919	OK	0.00016824
ATXN7L3	2.13E-09	5.873712169	0.01502433	OK	5.11E-09
UBTF	2.53E-32	11.77821464	0.0302151	OK	8.43E-32
SLC25A39	0	37.53389485	0.09645511	OK	0
GRN	0	242.5820361	0.62383394	OK	0
FZD2	6.73E-23	9.781935342	0.02507105	OK	2.06E-22
CCDC43	1.18E-35	12.4081394	0.03183274	OK	4.04E-35
AC005180.2	6.63E-12	6.765748074	0.01666185	OK	1.70E-11
GJC1	2.14E-55	15.6339259	0.04004469	OK	8.29E-55
HIGD1B	0	40.89352462	0.10383772	OK	0
EFTUD2	3.81E-78	18.67699728	0.04794419	OK	1.66E-77
KIF18B	0	40.07758412	0.10046919	OK	0
DCAKD	3.22E-19	8.884184889	0.02275315	OK	9.32E-19
NMT1	3.60E-18	8.611776018	0.0220726	OK	1.03E-17
PLCD3	3.45E-132	24.43705995	0.06270878	OK	1.82E-131
ACBD4	7.16E-20	9.049760852	0.02317839	OK	2.09E-19
HEXIM1	1.79E-12	6.953220739	0.01780562	OK	4.61E-12
HEXIM2	2.59E-12	6.900624064	0.01765569	OK	6.67E-12
AC008105.3	5.50E-104	21.62259888	0.05548364	OK	2.66E-103
FMNL1	0	69.19855066	0.17787556	OK	0
MAP3K14	4.37E-11	6.48733072	0.01658833	OK	1.10E-10
ARHGAP27	4.95E-91	20.19939871	0.05184348	OK	2.28E-90
PLEKHM1	1.28E-17	8.465502064	0.02168793	OK	3.61E-17
KANSL1	1.89E-09	5.893673546	0.01508291	OK	4.54E-09
KANSL1-AS1	6.81E-67	17.2386039	0.04425771	OK	2.81E-66
NSF	5.23E-26	10.48198154	0.02687512	OK	1.65E-25
GOSR2	7.17E-09	5.669352369	0.01450371	OK	1.70E-08
RPRML	1.80E-25	10.36433512	0.02616861	OK	5.65E-25
CDC27	1.85E-34	12.18559273	0.03126339	OK	6.30E-34
KPNB1	1.45E-95	20.70869038	0.05318553	OK	6.77E-95
TBX21	2.74E-09	5.831772285	0.01483259	OK	6.56E-09
MRPL10	3.24E-115	22.78490163	0.05851415	OK	1.62E-114
SCRN2	2.00E-06	4.611805101	0.01177993	OK	4.35E-06
SP2	0.00027679	3.453398949	0.00879843	OK	0.00054523
PNPO	1.07E-12	7.025400201	0.01798328	OK	2.77E-12

CDK5RAP3	0.00036283	3.379696865	0.00861716	OK	0.00071034
COPZ2	0	89.7536848	0.23073188	OK	0
NFE2L1	1.37E-34	12.21045978	0.03132364	OK	4.64E-34
CBX1	6.39E-126	23.84022789	0.06123664	OK	3.30E-125
SNX11	1.28E-07	5.153028245	0.01317013	OK	2.92E-07
SKAP1	3.97E-203	30.38787793	0.07793728	OK	2.48E-202
HOXB2	4.34E-162	27.10463034	0.06961724	OK	2.47E-161
HOXB-AS1	6.20E-152	26.22904887	0.06734165	OK	3.44E-151
HOXB3	8.55E-10	6.023203955	0.01536802	OK	2.08E-09
HOXB-AS3	1.12E-11	6.688891929	0.01631038	OK	2.86E-11
HOXB4	4.95E-18	8.575154688	0.02194279	OK	1.41E-17
HOXB6	4.29E-54	15.44164318	0.03947465	OK	1.65E-53
HOXB7	4.85E-79	18.78675271	0.04813501	OK	2.12E-78
CALCOCO2	1.87E-129	24.17863345	0.06211019	OK	9.77E-129
ATP5MC1	0	58.86784924	0.15132999	OK	0
UBE2Z	7.59E-12	6.746083446	0.01727412	OK	1.94E-11
SNF8	0	40.56395275	0.10425331	OK	0
GNGT2	4.19E-72	17.91900733	0.04596697	OK	1.77E-71
ABI3	0	77.72583243	0.19979025	OK	0
ZNF652	1.17E-14	7.629971571	0.01954662	OK	3.16E-14
PHB	0	49.95305537	0.12840155	OK	0
NGFR	4.70E-11	6.47630898	0.01645348	OK	1.18E-10
NXPH3	0	39.53862062	0.10155359	OK	0
SPOP	1.28E-95	20.71445527	0.05319848	OK	6.01E-95
SLC35B1	3.01E-146	25.72580795	0.06608123	OK	1.64E-145
FAM117A	9.97E-21	9.262709811	0.0237188	OK	2.96E-20
KAT7	7.58E-05	3.788305381	0.00966657	OK	0.00015398
TAC4	5.43E-06	4.399231366	0.01071889	OK	1.16E-05
DLX4	1.57E-15	7.885418229	0.02005838	OK	4.28E-15
DLX3	1.02E-17	8.491210829	0.02164939	OK	2.90E-17
ITGA3	3.74E-154	26.42287053	0.06784384	OK	2.09E-153
PDK2	8.50E-180	28.5675587	0.07338157	OK	5.04E-179
PPP1R9B	1.51E-17	8.446172383	0.02163737	OK	4.25E-17
SGCA	0	118.0666559	0.30353042	OK	0
COL1A1	0	84.41296208	0.21696818	OK	0
XYLT2	1.25E-05	4.214194263	0.01075054	OK	2.64E-05
MRPL27	3.00E-266	34.84015087	0.08952997	OK	2.08E-265
EME1	2.03E-20	9.186351788	0.02325864	OK	6.01E-20
LRRC59	0	39.90879168	0.10256076	OK	0
ACSF2	5.04E-16	8.025768764	0.020545	OK	1.39E-15
CACNA1G	1.60E-08	5.529700107	0.01402989	OK	3.76E-08
ABCC3	2.21E-187	29.17177002	0.07492896	OK	1.33E-186
ANKRD40	1.57E-14	7.592317022	0.01944906	OK	4.22E-14
AC005921.2	1.99E-21	9.433029551	0.02418095	OK	5.96E-21
LUC7L3	1.12E-47	14.45791818	0.0371101	OK	4.16E-47
TOB1	2.80E-207	30.70054605	0.07888221	OK	1.76E-206
SPAG9	8.60E-152	26.21657472	0.06735307	OK	4.77E-151
NME1	0	45.32437718	0.11649157	OK	0
NME2	2.50E-06	4.564958973	0.01165648	OK	5.41E-06
UTP18	0	46.10346778	0.11848509	OK	0
TOM1L1	2.19E-12	6.924584474	0.01770981	OK	5.64E-12
COX11	2.36E-90	20.12209949	0.05166726	OK	1.08E-89
STXBP4	9.51E-09	5.62065993	0.01435758	OK	2.24E-08

HLF	1.30E-21	9.477904291	0.02424197	OK	3.89E-21
MMD	4.49E-146	25.71021523	0.06602729	OK	2.45E-145
TMEM100	2.20E-17	8.401565394	0.02111323	OK	6.20E-17
PCTP	1.21E-09	5.96670132	0.01526053	OK	2.93E-09
TRIM25	5.08E-97	20.86940199	0.05358405	OK	2.39E-96
AC015912.3	3.51E-06	4.493122598	0.01146179	OK	7.57E-06
COIL	4.86E-19	8.838213549	0.02265099	OK	1.40E-18
SCPEP1	0	39.74774021	0.102152	OK	0
AKAP1	0	37.72932622	0.09693362	OK	0
AC007114.2	3.27E-08	5.403486152	0.01378741	OK	7.58E-08
MSI2	9.94E-08	5.200506677	0.01329334	OK	2.27E-07
MRPS23	1.21E-182	28.79576675	0.07398236	OK	7.23E-182
CUEDC1	9.28E-07	4.768405254	0.01218475	OK	2.05E-06
VEZF1	1.43E-27	10.81698551	0.02774294	OK	4.57E-27
SRSF1	1.66E-06	4.649731365	0.01188316	OK	3.63E-06
DYNLL2	3.31E-20	9.133706793	0.02341301	OK	9.75E-20
AC004687.1	0	51.72845886	0.13293084	OK	0
SUPT4H1	0	65.22589806	0.16768194	OK	0
SEPT4	0	95.05131795	0.24432292	OK	0
RAD51C	9.44E-74	18.12882265	0.04654527	OK	4.01E-73
SKA2	1.53E-209	30.86956329	0.07930856	OK	9.70E-209
PRR11	1.04E-182	28.80112396	0.07389971	OK	6.19E-182
GDPD1	2.01E-46	14.25770433	0.03654002	OK	7.42E-46
YPEL2	3.95E-18	8.600989667	0.02204439	OK	1.13E-17
DHX40	3.98E-14	7.470955849	0.01913562	OK	1.06E-13
AC091271.1	1.84E-09	5.897590648	0.01508561	OK	4.44E-09
CLTC	0	39.11752545	0.10053119	OK	0
PTRH2	5.26E-37	12.6548391	0.032466	OK	1.82E-36
VMP1	0	73.15373339	0.18806991	OK	0
RNFT1	2.56E-108	22.07815966	0.05669805	OK	1.26E-107
AC025048.4	4.06E-08	5.364415655	0.01361926	OK	9.39E-08
USP32	1.01E-22	9.740800506	0.02496426	OK	3.08E-22
APPBP2	6.54E-07	4.838509174	0.01236525	OK	1.45E-06
PPM1D	8.53E-32	11.67523171	0.02993921	OK	2.83E-31
BCAS3	1.53E-08	5.537805536	0.01415343	OK	3.59E-08
AC005884.1	7.38E-05	3.795047426	0.00947819	OK	0.00014992
AC005856.1	2.12E-16	8.131758278	0.01917147	OK	5.87E-16
AC005746.2	2.49E-15	7.827540514	0.01986077	OK	6.77E-15
TBX2-AS1	0	100.1302955	0.25728843	OK	0
TBX2	0	73.04911137	0.18771123	OK	0
C17orf82	2.54E-12	6.903191351	0.01756944	OK	6.55E-12
BRIP1	2.66E-90	20.11614189	0.05088476	OK	1.22E-89
METTL2A	2.29E-06	4.583383611	0.01170848	OK	4.97E-06
AC080038.1	4.19E-12	6.831834794	0.01745196	OK	1.08E-11
MRC2	0	40.36238114	0.1036863	OK	0
TANC2	4.77E-41	13.36603551	0.03428332	OK	1.70E-40
CYB561	1.55E-07	5.117429529	0.01306286	OK	3.51E-07
ACE	4.76E-62	16.58124199	0.04244118	OK	1.92E-61
DCAF7	3.14E-40	13.22524956	0.0339339	OK	1.11E-39
TACO1	1.88E-24	10.13746045	0.02598866	OK	5.85E-24
MAP3K3	8.04E-11	6.394795041	0.01636829	OK	2.00E-10
LIMD2	0	104.4197904	0.26845973	OK	0
CCDC47	0	47.84217741	0.12297214	OK	0

DDX42	1.18E-33	12.03346822	0.03087246	OK	4.00E-33
FTSJ3	8.39E-05	3.763211846	0.00960062	OK	0.0001699
PSMC5	0	50.81907073	0.13062942	OK	0
SMARCD2	8.46E-09	5.640920009	0.01442847	OK	2.00E-08
CD79B	1.06E-106	21.90929364	0.05607456	OK	5.16E-106
ICAM2	2.51E-135	24.73061436	0.06349903	OK	1.33E-134
ERN1	5.88E-117	22.95979171	0.05896293	OK	2.96E-116
SNHG25	4.01E-06	4.464902465	0.01139598	OK	8.61E-06
TEX2	3.48E-16	8.071364805	0.0206691	OK	9.62E-16
PECAM1	0	67.78129312	0.17422174	OK	0
MILR1	0	71.67564727	0.18423681	OK	0
DDX5	0	52.21407685	0.13421069	OK	0
SMURF2	6.54E-07	4.838547485	0.01236593	OK	1.45E-06
GNA13	0	70.73691627	0.18184433	OK	0
AC037487.2	3.03E-05	4.010133128	0.01016124	OK	6.27E-05
RGS9	8.53E-09	5.639492404	0.01441815	OK	2.01E-08
CEP112	1.29E-33	12.02664211	0.03081625	OK	4.34E-33
PRKCA	3.25E-16	8.079435044	0.02069078	OK	9.00E-16
HELZ	1.22E-29	11.24513437	0.02884285	OK	3.98E-29
AC007448.3	9.67E-10	6.003338764	0.01532744	OK	2.34E-09
PSMD12	7.75E-242	33.18902544	0.08528452	OK	5.18E-241
PITPNC1	1.16E-26	10.62337861	0.0272335	OK	3.68E-26
BPTF	4.50E-12	6.821795011	0.01747082	OK	1.15E-11
C17orf58	9.36E-28	10.85577423	0.02783108	OK	3.00E-27
KPNA2	0	80.42662202	0.20675599	OK	0
AC005332.5	6.74E-12	6.763309525	0.01728346	OK	1.72E-11
AC005332.1	3.72E-32	11.74567594	0.03008554	OK	1.24E-31
AMZ2	3.61E-43	13.7246626	0.03521914	OK	1.31E-42
ARSG	6.57E-05	3.823670677	0.00975115	OK	0.00013383
SLC16A6	2.94E-155	26.51883152	0.06804327	OK	1.65E-154
WIPI1	4.17E-43	13.71424191	0.03518698	OK	1.51E-42
PRKAR1A	3.60E-98	20.99561692	0.05392454	OK	1.70E-97
FAM20A	0	47.45252303	0.12191108	OK	0
ABCA8	0	84.6509203	0.21731843	OK	0
ABCA9	0	58.47930292	0.14989666	OK	0
ABCA6	0	68.61550954	0.17618024	OK	0
ABCA10	0	61.69491218	0.15762637	OK	0
ABCA5	5.68E-12	6.788127625	0.01736516	OK	1.46E-11
MAP2K6	6.44E-32	11.69910774	0.0299715	OK	2.14E-31
KCNJ2	7.65E-18	8.524895085	0.02181332	OK	2.17E-17
SOX9	1.38E-21	9.471639134	0.02401067	OK	4.13E-21
SLC39A11	2.00E-89	20.01604552	0.05138549	OK	9.12E-89
SSTR2	4.50E-07	4.912296022	0.01251856	OK	1.00E-06
FAM104A	7.75E-23	9.767639399	0.02504098	OK	2.37E-22
CDC42EP4	7.31E-41	13.33425987	0.0342005	OK	2.60E-40
RPL38	0	128.1278123	0.3294408	OK	0
TTYH2	1.11E-28	11.04857081	0.02829885	OK	3.60E-28
GPRC5C	0	65.69405195	0.16884575	OK	0
CD300A	0	134.0882936	0.34477502	OK	0
CD300LB	0	45.98201223	0.11811313	OK	0
CD300C	0	39.33562684	0.10105262	OK	0
AC064805.1	3.67E-55	15.59949794	0.03982959	OK	1.42E-54
CD300E	0	57.59984866	0.14789841	OK	0

CD300LF	3.81E-128	24.05375499	0.06174367	OK	1.99E-127
SLC9A3R1	7.22E-16	7.981560795	0.02044196	OK	1.98E-15
GRIN2C	0.00048342	3.300003083	0.00821281	OK	0.00093841
FDXR	4.13E-06	4.458264821	0.01138236	OK	8.87E-06
MRPL58	2.05E-45	14.09456192	0.0361691	OK	7.52E-45
ATP5PD	0	102.1650123	0.26268638	OK	0
SLC16A5	7.01E-09	5.67311097	0.0144914	OK	1.66E-08
NT5C	6.28E-103	21.50996221	0.0552466	OK	3.02E-102
JPT1	0	146.2172624	0.37598191	OK	0
SUMO2	0	158.5584678	0.40770697	OK	0
NUP85	5.03E-11	6.466131874	0.01654548	OK	1.26E-10
MRPS7	2.07E-212	31.08262453	0.07986558	OK	1.32E-211
MIF4GD	0	44.86939648	0.11532013	OK	0
SLC25A19	0	52.6424606	0.13529105	OK	0
GRB2	0	170.3060983	0.43794444	OK	0
CASKIN2	6.46E-09	5.687182004	0.01451448	OK	1.53E-08
TSEN54	1.11E-06	4.732384944	0.01208677	OK	2.44E-06
MYO15B	3.24E-05	3.994561797	0.01017074	OK	6.69E-05
SAP30BP	6.12E-33	11.89722324	0.03052211	OK	2.05E-32
ITGB4	1.48E-16	8.175229573	0.02078092	OK	4.11E-16
GALK1	0	47.53033175	0.12216016	OK	0
H3F3B	0	182.9337341	0.4703322	OK	0
UNC13D	4.64E-36	12.48268465	0.03200165	OK	1.59E-35
WBP2	8.90E-76	18.38353838	0.04720526	OK	3.83E-75
TRIM47	6.72E-170	27.75932567	0.07130587	OK	3.91E-169
TEN1	1.10E-32	11.84826447	0.03039049	OK	3.68E-32
SRP68	4.36E-13	7.149212662	0.01830692	OK	1.14E-12
GALR2	5.37E-31	11.51779912	0.02942861	OK	1.77E-30
EXOC7	2.57E-07	5.021384385	0.01283832	OK	5.78E-07
UBALD2	0	91.2634323	0.23464676	OK	0
PRPSAP1	1.89E-95	20.69568768	0.05314471	OK	8.87E-95
SPHK1	0	52.0435743	0.1337349	OK	0
RHBDF2	0	41.5401313	0.10673447	OK	0
CYGB	0.00013236	3.647579231	0.00925314	OK	0.00026546
ST6GALNAC1	6.92E-20	9.053508956	0.02183686	OK	2.02E-19
MXRA7	0	199.3551781	0.51262677	OK	0
JMJD6	1.57E-234	32.6786275	0.08396441	OK	1.04E-233
METTL23	9.53E-78	18.6279664	0.0478322	OK	4.14E-77
SRSF2	0	37.95548311	0.09754496	OK	0
MFSD11	3.74E-10	6.15557336	0.01575296	OK	9.16E-10
SEC14L1	0	53.76114881	0.13818869	OK	0
SEPT9	9.61E-83	19.23399832	0.04938641	OK	4.27E-82
TNRC6C	1.79E-89	20.02144054	0.05138917	OK	8.18E-89
TMC6	0	40.71622585	0.10462274	OK	0
TMC8	9.90E-33	11.85698378	0.03039148	OK	3.32E-32
SYNGR2	0	105.0511413	0.2701097	OK	0
TK1	0	152.5040697	0.39157608	OK	0
AFMID	7.81E-10	6.037818625	0.0154481	OK	1.90E-09
BIRC5	0	231.4827972	0.5935739	OK	0
SOCS3	0	93.82359723	0.24123229	OK	0
PGS1	1.45E-15	7.895251295	0.02022136	OK	3.96E-15
CYTH1	0	48.50150351	0.12464894	OK	0
USP36	7.58E-88	19.83396963	0.05092491	OK	3.44E-87

TIMP2	0	93.85763987	0.24131976	OK	0
LGALS3BP	0	62.31260064	0.16017795	OK	0
CANT1	5.28E-05	3.877393856	0.00989174	OK	0.00010794
C1QTNF1	0	103.2920065	0.26553995	OK	0
CBX4	2.64E-63	16.75423002	0.04300184	OK	1.07E-62
TBC1D16	0.00020125	3.538442144	0.0090086	OK	0.00039975
GAA	0	92.46564534	0.23773189	OK	0
EIF4A3	0	103.638884	0.26647124	OK	0
AC087741.1	8.18E-12	6.735377334	0.01718971	OK	2.09E-11
SGSH	6.76E-07	4.832076386	0.01234359	OK	1.50E-06
RNF213	1.65E-301	37.09558415	0.09533	OK	1.20E-300
CHMP6	3.97E-15	7.768374112	0.01989892	OK	1.08E-14
BAIAP2-DT	1.01E-12	7.033175414	0.01798788	OK	2.63E-12
BAIAP2	2.64E-174	28.12191428	0.07221536	OK	1.55E-173
NDUFAF8	9.93E-115	22.7357349	0.05839817	OK	4.96E-114
SLC38A10	1.78E-11	6.621264869	0.01695046	OK	4.50E-11
AC027601.4	0.00017966	3.568296366	0.00831916	OK	0.00035783
BAHCC1	0.00047287	3.306191095	0.00841039	OK	0.00091842
ACTG1	0	161.82081	0.41604779	OK	0
PDE6G	4.34E-42	13.54337605	0.03468879	OK	1.56E-41
OXLD1	2.70E-20	9.155575607	0.02346867	OK	7.97E-20
CCDC137	1.87E-14	7.56945278	0.01938826	OK	5.03E-14
ARL16	2.00E-46	14.25785789	0.03658923	OK	7.40E-46
HGS	0.00018894	3.555067465	0.00906569	OK	0.00037589
MRPL12	4.80E-258	34.29413757	0.08812101	OK	3.28E-257
SLC25A10	9.23E-10	6.010832864	0.01527337	OK	2.24E-09
MCRIP1	3.32E-248	33.62756572	0.08641235	OK	2.24E-247
P4HB	0	75.59081448	0.19434093	OK	0
ARHGDIA	0	65.55968984	0.16854127	OK	0
ALYREF	0	53.97727687	0.13874633	OK	0
ANAPC11	0	88.21141653	0.22680037	OK	0
NPB	7.99E-34	12.06590976	0.03088162	OK	2.70E-33
PCYT2	1.80E-29	11.21119257	0.02874289	OK	5.84E-29
SIRT7	3.29E-47	14.3833531	0.03691153	OK	1.22E-46
MAFG	1.79E-289	36.34153	0.09338807	OK	1.27E-288
PYCR1	1.99E-05	4.108375491	0.01038034	OK	4.16E-05
ASPSCR1	3.74E-13	7.170288394	0.01836138	OK	9.81E-13
CENPX	7.57E-296	36.74283638	0.09442349	OK	5.44E-295
LRRC45	0.00012713	3.657937312	0.00931598	OK	0.00025524
RAC3	2.03E-20	9.186339704	0.02341794	OK	6.01E-20
DCXR	0	41.87044544	0.10761209	OK	0
RFNG	4.54E-25	10.27549281	0.02634908	OK	1.42E-24
GPS1	1.19E-14	7.627934806	0.01954053	OK	3.21E-14
DUS1L	1.16E-49	14.76887141	0.03790528	OK	4.38E-49
CCDC57	1.82E-27	10.79469044	0.02767232	OK	5.83E-27
SLC16A3	0	131.6483501	0.33850372	OK	0
CSNK1D	1.54E-127	23.99581131	0.06163576	OK	7.99E-127
LINC01970	3.87E-16	8.058246845	0.02057061	OK	1.07E-15
CD7	2.05E-138	25.01597366	0.06419983	OK	1.10E-137
SECTM1	0	64.86139926	0.16669769	OK	0
OGFOD3	2.31E-41	13.4200788	0.03443587	OK	8.24E-41
HEXDC	1.95E-12	6.940870393	0.01777121	OK	5.03E-12
CYBC1	1.88E-304	37.27772608	0.09579394	OK	1.37E-303

NARF	1.54E-35	12.38704367	0.0317785	OK	5.25E-35
FOXK2	3.09E-17	8.361614704	0.02142157	OK	8.69E-17
WDR45B	2.72E-85	19.53581245	0.05016643	OK	1.22E-84
RAB40B	1.26E-52	15.22218424	0.03904917	OK	4.81E-52
FN3KRP	3.78E-33	11.93735897	0.03061892	OK	1.27E-32
FN3K	1.04E-09	5.990792388	0.0153075	OK	2.53E-09
TBCD	9.27E-07	4.768778659	0.01218081	OK	2.04E-06
B3GNTL1	2.38E-31	11.58762518	0.02968351	OK	7.86E-31
METRNL	0	187.208757	0.48141589	OK	0
AC144831.1	6.25E-07	4.847498583	0.01235677	OK	1.39E-06
USP14	3.14E-61	16.46744391	0.04227674	OK	1.26E-60
COLEC12	0	44.76737358	0.11502775	OK	0
TYMS	0	118.5411943	0.30450588	OK	0
ENOSF1	6.11E-20	9.067038156	0.02324004	OK	1.79E-19
YES1	6.66E-79	18.76986809	0.04815446	OK	2.91E-78
NDC80	0	48.30215787	0.12379442	OK	0
SMCHD1	4.74E-29	11.12499367	0.02853721	OK	1.54E-28
EMILIN2	0	134.3189186	0.34536991	OK	0
LPIN2	7.25E-78	18.64263431	0.04786869	OK	3.15E-77
MYOM1	0	40.73351244	0.10458264	OK	0
MYL12A	0	133.7853557	0.34399688	OK	0
MYL12B	0	148.5738482	0.38202739	OK	0
TGIF1	0	72.65165769	0.18677412	OK	0
GAPLINC	7.79E-147	25.77819305	0.06613318	OK	4.26E-146
DLGAP1-AS1	1.93E-11	6.609615199	0.0169191	OK	4.87E-11
LINC00667	7.63E-34	12.06977526	0.03096434	OK	2.58E-33
EPB41L3	0	133.9987234	0.34453964	OK	0
L3MBTL4	0.00036484	3.378177767	0.00859306	OK	0.00071411
L3MBTL4-AS1	1.68E-167	27.5600657	0.070744	OK	9.68E-167
PTPRM	8.24E-12	6.734183416	0.01722541	OK	2.10E-11
RAB12	1.79E-18	8.69169203	0.0222747	OK	5.12E-18
NDUFV2	0	94.44668477	0.24283698	OK	0
ANKRD12	4.84E-52	15.13390715	0.03884933	OK	1.84E-51
TWSG1	0	52.60879401	0.13520576	OK	0
RALBP1	8.10E-187	29.12729814	0.07483888	OK	4.88E-186
RAB31	0	65.54705017	0.16850755	OK	0
VAPA	0	67.82340648	0.17436241	OK	0
APCDD1	5.87E-21	9.319106961	0.02381263	OK	1.74E-20
NAPG	1.72E-52	15.20171914	0.03902024	OK	6.57E-52
PIEZO2	9.43E-27	10.64274003	0.02688718	OK	2.99E-26
GNAL	6.03E-75	18.27945693	0.04687828	OK	2.58E-74
CHMP1B	0	120.1907521	0.30904228	OK	0
MPPE1	8.26E-19	8.778790575	0.02249755	OK	2.38E-18
IMPA2	0	43.2342845	0.11109507	OK	0
TUBB6	0	38.83213826	0.09979632	OK	0
AFG3L2	3.71E-12	6.849400752	0.01753878	OK	9.52E-12
SPIRE1	4.43E-21	9.348880696	0.02396503	OK	1.32E-20
PSMG2	0	42.45440355	0.10911554	OK	0
PTPN2	0	82.79056016	0.2128524	OK	0
SEH1L	5.26E-19	8.829354879	0.02262345	OK	1.52E-18
LDLRAD4	4.71E-225	32.00456207	0.08222386	OK	3.07E-224
FAM210A	4.08E-124	23.66556442	0.06077974	OK	2.09E-123
RNMT	6.02E-50	14.8131675	0.03802368	OK	2.27E-49

ROCK1	8.42E-110	22.23201824	0.05710519	OK	4.15E-109
GREB1L	5.94E-10	6.081843943	0.0154616	OK	1.45E-09
SNRPD1	0	63.6674893	0.163674	OK	0
ABHD3	1.23E-102	21.47891435	0.05514662	OK	5.88E-102
MIB1	6.33E-19	8.808752692	0.02256988	OK	1.82E-18
GATA6-AS1	2.88E-18	8.637072309	0.02208386	OK	8.24E-18
GATA6	0	42.56452414	0.10934666	OK	0
RBBP8	2.23E-32	11.78883183	0.03022721	OK	7.44E-32
RIOK3	6.83E-151	26.13755861	0.06714736	OK	3.78E-150
RMC1	4.06E-09	5.76578562	0.01474655	OK	9.68E-09
NPC1	2.54E-103	21.55188488	0.05533046	OK	1.22E-102
ANKRD29	7.16E-142	25.33162082	0.06501213	OK	3.88E-141
LAMA3	1.13E-15	7.925771435	0.02020712	OK	3.10E-15
TTC39C	6.65E-140	25.15240918	0.06458824	OK	3.59E-139
TTC39C-AS1	1.91E-22	9.675581142	0.02299938	OK	5.81E-22
OSBPL1A	1.91E-279	35.70089917	0.09173806	OK	1.34E-278
IMPACT	1.45E-24	10.1631261	0.02605924	OK	4.50E-24
ZNF521	3.80E-20	9.118726916	0.02298598	OK	1.12E-19
SS18	0.00049335	3.294289851	0.00839678	OK	0.00095719
KCTD1	3.50E-24	10.07673023	0.02577107	OK	1.09E-23
CDH2	1.50E-229	32.32607961	0.08297225	OK	9.88E-229
DSC2	0	105.7175684	0.27179075	OK	0
RNF125	4.85E-162	27.10056184	0.0695925	OK	2.76E-161
RNF138	2.75E-78	18.69445415	0.0479894	OK	1.20E-77
AC011825.2	8.96E-08	5.219667347	0.01273383	OK	2.05E-07
GAREM1	4.40E-20	9.102876574	0.02329072	OK	1.29E-19
DTNA	2.57E-17	8.383325396	0.02147532	OK	7.24E-17
MAPRE2	2.83E-78	18.69284518	0.04799423	OK	1.23E-77
ZNF397	2.31E-07	5.041531918	0.01288617	OK	5.21E-07
ZNF24	2.48E-18	8.654378991	0.02218296	OK	7.09E-18
INO80C	1.60E-19	8.961853399	0.02296676	OK	4.64E-19
GALNT1	4.20E-150	26.06806322	0.06696535	OK	2.32E-149
C18orf21	2.96E-12	6.881697358	0.01761825	OK	7.61E-12
SLC39A6	7.95E-23	9.765079422	0.0250365	OK	2.43E-22
AC023043.1	4.04E-06	4.463058311	0.01137576	OK	8.68E-06
MOCOS	0.00026062	3.469599195	0.00873208	OK	0.00051444
FHOD3	6.72E-14	7.401710216	0.01889217	OK	1.79E-13
TPGS2	1.59E-66	17.18968209	0.0441309	OK	6.53E-66
AC016205.1	5.03E-07	4.890561218	0.0109846	OK	1.12E-06
LINC00907	3.12E-12	6.873941576	0.01524003	OK	8.03E-12
SETBP1	1.49E-76	18.48004726	0.04742266	OK	6.45E-76
SLC14A1	1.01E-136	24.85987611	0.06376148	OK	5.41E-136
SIGLEC15	3.23E-61	16.46576917	0.04210895	OK	1.30E-60
PSTPIP2	9.10E-251	33.80238508	0.08682077	OK	6.15E-250
ATP5F1A	0	63.51065273	0.16327145	OK	0
HAUS1	7.31E-66	17.10086335	0.0438999	OK	3.00E-65
PIAS2	7.38E-05	3.794947483	0.0096816	OK	0.00014997
HDHD2	5.47E-16	8.015758802	0.02053154	OK	1.51E-15
IER3IP1	1.36E-92	20.37606719	0.05232819	OK	6.33E-92
ZBTB7C	1.83E-36	12.55646769	0.0320842	OK	6.30E-36
SMAD7	6.10E-09	5.697001149	0.01456533	OK	1.44E-08
C18orf32	3.87E-11	6.505720657	0.01665256	OK	9.70E-11
RPL17	5.70E-182	28.74203146	0.07384598	OK	3.39E-181

ACAA2	0	57.12728055	0.14684756	OK	0
MYO5B	2.82E-08	5.429755166	0.01358949	OK	6.56E-08
SKA1	0	48.45194763	0.12269137	OK	0
MRO	1.49E-135	24.75154295	0.06352284	OK	7.94E-135
ME2	0	64.25564469	0.16517502	OK	0
MBD2	0	47.62241385	0.12240624	OK	0
POLI	1.75E-05	4.137923267	0.01056071	OK	3.67E-05
RAB27B	7.88E-15	7.681125099	0.01896039	OK	2.13E-14
CCDC68	4.33E-235	32.71804158	0.08394442	OK	2.87E-234
TCF4	0	39.20905585	0.10076432	OK	0
TXNL1	0	40.72871214	0.1046771	OK	0
FECH	8.70E-08	5.225121511	0.01335456	OK	1.99E-07
NARS	5.26E-124	23.65488443	0.06076331	OK	2.70E-123
ATP8B1	2.08E-100	21.23914897	0.05451006	OK	9.90E-100
NEDD4L	4.92E-44	13.86842225	0.03556549	OK	1.79E-43
MALT1	7.18E-171	27.83969515	0.07151254	OK	4.19E-170
ZNF532	9.77E-82	19.11336416	0.04905965	OK	4.32E-81
SEC11C	0	42.54738156	0.10935317	OK	0
LMAN1	2.04E-153	26.35871764	0.06771806	OK	1.14E-152
PMAIP1	0	80.82086966	0.2077507	OK	0
AC090409.1	1.24E-08	5.574537735	0.01415367	OK	2.92E-08
RNF152	1.27E-87	19.80797986	0.05081699	OK	5.76E-87
TNFRSF11A	9.44E-73	18.00172592	0.0461677	OK	3.99E-72
ZCCHC2	1.90E-75	18.34228482	0.04709205	OK	8.16E-75
BCL2	1.44E-90	20.14679571	0.05173252	OK	6.58E-90
KDSR	1.99E-47	14.41814842	0.03700545	OK	7.40E-47
AC036176.1	9.11E-06	4.285548526	0.01091723	OK	1.93E-05
VPS4B	2.14E-109	22.19012225	0.0569958	OK	1.05E-108
SERPINB2	0	64.18299885	0.16434683	OK	0
HMSD	9.33E-19	8.765063741	0.02211801	OK	2.68E-18
SERPINB8	0	42.52085978	0.10926493	OK	0
AC110597.1	3.12E-07	4.983712064	0.01218757	OK	7.00E-07
DSEL	0	40.96583745	0.1052354	OK	0
TMX3	3.15E-06	4.515928081	0.01153849	OK	6.81E-06
CCDC102B	0	51.623901	0.13258524	OK	0
DOK6	8.60E-11	6.384401807	0.01626536	OK	2.14E-10
CD226	2.59E-185	29.00824837	0.07440759	OK	1.55E-184
SOCS6	3.03E-19	8.891059846	0.02278114	OK	8.77E-19
CYB5A	5.19E-235	32.71249515	0.08405695	OK	3.44E-234
CNDP2	0	90.75703576	0.23333503	OK	0
ZADH2	5.22E-14	7.435288678	0.01904029	OK	1.39E-13
ZNF516	2.97E-05	4.015022588	0.01024146	OK	6.15E-05
AC018413.1	5.81E-06	4.384436896	0.01113111	OK	1.24E-05
C18orf65	8.62E-08	5.226907545	0.01332006	OK	1.97E-07
MBP	0	57.3637671	0.14745582	OK	0
AC018529.2	8.30E-17	8.244400754	0.02109274	OK	2.32E-16
ATP9B	5.14E-34	12.10229289	0.03104137	OK	1.74E-33
NFATC1	6.25E-24	10.01953291	0.02567893	OK	1.93E-23
CTDP1	5.75E-05	3.85673107	0.00983627	OK	0.00011724
PQLC1	0	44.80203124	0.11514625	OK	0
HSBP1L1	1.94E-62	16.63512066	0.04269675	OK	7.84E-62
TXNL4A	0	61.79427245	0.15885661	OK	0
RBFA	3.32E-08	5.400664231	0.01381328	OK	7.70E-08

ADNP2	0.00031133	3.421548053	0.00871839	OK	0.00061163
PARD6G	3.16E-27	10.74401128	0.02745746	OK	1.01E-26
C20orf96	1.55E-05	4.16552654	0.01062438	OK	3.26E-05
NRSN2	3.78E-20	9.119188908	0.02334419	OK	1.11E-19
TRIB3	4.43E-10	6.128523407	0.01565623	OK	1.08E-09
RBCK1	2.27E-90	20.12406127	0.05168107	OK	1.04E-89
TBC1D20	1.03E-05	4.257585385	0.01087217	OK	2.18E-05
CSNK2A1	1.12E-09	5.980082564	0.01530511	OK	2.70E-09
TCF15	0	48.37881304	0.1208181	OK	0
SLC52A3	1.01E-06	4.75180415	0.01092949	OK	2.22E-06
FAM110A	7.95E-224	31.91621818	0.08198574	OK	5.17E-223
ANGPT4	4.55E-37	12.66630034	0.03199592	OK	1.57E-36
PSMF1	1.16E-197	29.97130718	0.07700908	OK	7.18E-197
TMEM74B	1.59E-09	5.9216256	0.01484228	OK	3.84E-09
FKBP1A	0	88.29352798	0.22701193	OK	0
NSFL1C	6.40E-70	17.63706839	0.04528456	OK	2.67E-69
SIRPB2	3.69E-67	17.27405563	0.04430253	OK	1.52E-66
SIRPB1	0	64.49470568	0.16572205	OK	0
SIRPG	1.19E-18	8.73800976	0.02218251	OK	3.41E-18
AL117335.1	0.0002075	3.530358835	0.00898154	OK	0.00041184
SIRPA	0	89.9834607	0.23133501	OK	0
SNRPB	0	85.04141006	0.2186479	OK	0
NOP56	1.40E-33	12.01948057	0.03083658	OK	4.74E-33
IDH3B	9.89E-57	15.82850621	0.04063201	OK	3.87E-56
CPXM1	2.41E-05	4.06427721	0.01021345	OK	5.01E-05
VPS16	5.41E-19	8.826370182	0.02261657	OK	1.56E-18
PTPRA	1.28E-40	13.29268194	0.03411203	OK	4.53E-40
MRPS26	7.98E-219	31.55368125	0.08107532	OK	5.14E-218
OXT	4.69E-07	4.904160836	0.01189071	OK	1.05E-06
DDRGK1	5.89E-134	24.60285382	0.06320075	OK	3.12E-133
ITPA	2.61E-137	24.91426409	0.06399962	OK	1.40E-136
C20orf194	9.28E-21	9.270258142	0.02375625	OK	2.76E-20
ATRN	2.01E-22	9.67080832	0.0247812	OK	6.09E-22
ADAM33	5.67E-140	25.15873374	0.0645362	OK	3.06E-139
SIGLEC1	0	65.24063291	0.16764826	OK	0
HSPA12B	2.61E-37	12.70978077	0.03166374	OK	9.03E-37
C20orf27	0	60.39719705	0.15525336	OK	0
CENPB	1.51E-16	8.172786088	0.02094102	OK	4.19E-16
CDC25B	4.68E-20	9.096122447	0.02329266	OK	1.37E-19
PANK2	5.53E-33	11.90570691	0.03054071	OK	1.86E-32
RNF24	1.28E-41	13.46346882	0.03454379	OK	4.60E-41
SMOX	2.58E-37	12.71075095	0.03259122	OK	8.92E-37
ADRA1D	1.47E-21	9.464919657	0.02401572	OK	4.40E-21
PRNP	0	43.15968758	0.11092791	OK	0
RASSF2	0	45.50887508	0.11694282	OK	0
TMEM230	0	58.13860339	0.14945482	OK	0
PCNA	2.97E-189	29.31901145	0.07531866	OK	1.80E-188
CDS2	9.61E-44	13.82036058	0.035466	OK	3.49E-43
GPCPD1	0	63.88877576	0.16423351	OK	0
SHLD1	5.43E-35	12.28530908	0.03150044	OK	1.85E-34
TRMT6	0	39.76615089	0.10217929	OK	0
MCM8	6.04E-19	8.813909843	0.02248504	OK	1.74E-18
CRLS1	3.93E-22	9.601752335	0.02461548	OK	1.19E-21

BMP2	1.43E-84	19.45083759	0.04982664	OK	6.42E-84
TMX4	1.35E-164	27.31654774	0.07017792	OK	7.73E-164
PLCB1	2.16E-52	15.1867826	0.03888782	OK	8.24E-52
PLCB4	3.13E-122	23.48178386	0.06017686	OK	1.60E-121
LAMP5	1.29E-09	5.956033448	0.0151753	OK	3.12E-09
PARAL1	3.72E-06	4.48041371	0.01124999	OK	8.02E-06
MKKS	0.000448	3.321294667	0.00846412	OK	0.0008719
SLX4IP	2.36E-06	4.577233162	0.01168792	OK	5.11E-06
JAG1	0	85.05541583	0.21865294	OK	0
SPTLC3	8.79E-10	6.01874834	0.0153737	OK	2.13E-09
ISM1	9.56E-27	10.64143015	0.02714328	OK	3.03E-26
TASP1	1.09E-41	13.47523856	0.03456014	OK	3.92E-41
ESF1	1.21E-15	7.918066872	0.02028941	OK	3.30E-15
MACROD2	1.98E-09	5.885921279	0.01493697	OK	4.75E-09
KIF16B	5.01E-08	5.326322318	0.01361552	OK	1.16E-07
SNRPB2	0	50.82047189	0.13063279	OK	0
DSTN	0	261.5256302	0.67254854	OK	0
RRBP1	1.71E-127	23.99128599	0.06162922	OK	8.90E-127
SNX5	0	60.74255027	0.15614861	OK	0
MGME1	1.45E-66	17.19478742	0.04413452	OK	5.99E-66
PET117	3.43E-07	4.965292381	0.01268833	OK	7.69E-07
POLR3F	0.00026988	3.460209057	0.00882002	OK	0.000532
SEC23B	2.90E-46	14.23205438	0.03651608	OK	1.07E-45
SMIM26	0	46.80618702	0.12030803	OK	0
DTD1	5.99E-79	18.77550196	0.04820846	OK	2.62E-78
SLC24A3	8.49E-20	9.03122804	0.02303376	OK	2.48E-19
RIN2	5.48E-122	23.45794043	0.06025198	OK	2.80E-121
NAA20	0	62.41589212	0.16045495	OK	0
CRNKL1	2.47E-11	6.572916834	0.0168275	OK	6.22E-11
INSM1	5.95E-19	8.815690431	0.02177486	OK	1.71E-18
RALGAPA2	1.42E-05	4.186222684	0.01067891	OK	2.98E-05
KIZ	4.71E-19	8.841877462	0.02265458	OK	1.36E-18
XRN2	0	64.57331689	0.16600457	OK	0
THBD	0	135.6741884	0.34886314	OK	0
CD93	0	94.06253188	0.24182048	OK	0
NXT1	0	55.77483922	0.14337097	OK	0
GZF1	0	76.06559254	0.19552972	OK	0
CST3	0	241.1120339	0.61995976	OK	0
CST7	0	96.37592104	0.2476749	OK	0
APMAP	0	45.9651428	0.11813527	OK	0
ENTPD6	2.38E-06	4.575228742	0.011684	OK	5.16E-06
PYGB	1.01E-37	12.78362708	0.03279073	OK	3.51E-37
ABHD12	0	62.65629818	0.16106024	OK	0
GINS1	9.53E-81	18.99416972	0.04853803	OK	4.19E-80
NINL	2.41E-06	4.572686874	0.01164825	OK	5.22E-06
AL390198.1	7.34E-08	5.256498478	0.01330341	OK	1.68E-07
FAM182B	1.77E-06	4.63651473	0.01140525	OK	3.87E-06
REM1	6.39E-109	22.14084617	0.05679339	OK	3.14E-108
HM13	0	79.92269336	0.20548104	OK	0
ID1	0	74.33343097	0.19106066	OK	0
COX4I2	0	181.1764619	0.4652372	OK	0
BCL2L1	7.02E-29	11.08988751	0.02844185	OK	2.27E-28
TPX2	0	148.0986714	0.37985106	OK	0

FOXS1	7.23E-265	34.74877879	0.08921694	OK	4.99E-264
DUSP15	3.28E-07	4.974045799	0.01267482	OK	7.35E-07
PDRG1	9.29E-16	7.950412596	0.02036576	OK	2.55E-15
CCM2L	0	38.37211973	0.0964151	OK	0
HCK	0	131.2079438	0.33736356	OK	0
TM9SF4	8.75E-07	4.7804124	0.01221624	OK	1.93E-06
ASXL1	1.09E-09	5.984165199	0.01531481	OK	2.63E-09
NOL4L	5.70E-06	4.388582159	0.01119193	OK	1.22E-05
COMMD7	1.66E-249	33.71637359	0.08663936	OK	1.12E-248
MAPRE1	0	38.27720946	0.09836989	OK	0
CDK5RAP1	4.24E-06	4.452470365	0.01137189	OK	9.10E-06
SNTA1	0	92.18625603	0.23697369	OK	0
NECAB3	1.47E-07	5.127296644	0.01310115	OK	3.34E-07
E2F1	0	54.6711995	0.13979254	OK	0
PXMP4	3.05E-08	5.415623073	0.01384143	OK	7.09E-08
CHMP4B	5.50E-264	34.69040018	0.08914535	OK	3.79E-263
RALY	0	47.43798607	0.1219327	OK	0
EIF2S2	0	58.16928913	0.1495339	OK	0
ASIP	2.75E-14	7.519401882	0.01910562	OK	7.37E-14
AHCY	1.52E-125	23.80397051	0.0611392	OK	7.83E-125
ITCH	3.26E-05	3.993026375	0.01019274	OK	6.73E-05
DYNLRB1	0	80.61172724	0.20725405	OK	0
MAP1LC3A	0	51.41961778	0.13215114	OK	0
NCOA6	7.83E-07	4.802740595	0.01227019	OK	1.73E-06
GGT7	7.03E-15	7.695742544	0.01968939	OK	1.90E-14
GSS	1.97E-49	14.73331957	0.03781051	OK	7.41E-49
EDEM2	8.61E-227	32.1292315	0.08254477	OK	5.63E-226
PROCR	1.68E-36	12.56319306	0.03222149	OK	5.79E-36
MMP24OS	0	65.05575736	0.16724449	OK	0
EIF6	0	55.17196288	0.14182409	OK	0
CEP250	2.31E-05	4.074535739	0.01039361	OK	4.79E-05
FO393401.1	3.31E-85	19.52586112	0.05011146	OK	1.49E-84
ERGIC3	4.43E-105	21.73858879	0.05583437	OK	2.14E-104
SPAG4	5.97E-118	23.05906518	0.05914124	OK	3.01E-117
CPNE1	5.01E-21	9.335836823	0.02393539	OK	1.49E-20
ROMO1	0	49.30213824	0.12672739	OK	0
RBM39	5.60E-60	16.29226053	0.04182839	OK	2.24E-59
PHF20	4.45E-120	23.27010896	0.05977304	OK	2.26E-119
SCAND1	0	58.90203812	0.1514185	OK	0
CNBD2	2.32E-12	6.915965425	0.01770173	OK	5.99E-12
NORAD	0	47.06378176	0.12096884	OK	0
EPB41L1	2.19E-25	10.34583525	0.02651298	OK	6.85E-25
AAR2	1.43E-06	4.680471548	0.01195307	OK	3.13E-06
DLGAP4	1.86E-32	11.80384455	0.03028007	OK	6.23E-32
MYL9	0	332.7269502	0.85568223	OK	0
TGIF2	2.37E-10	6.227242512	0.01592689	OK	5.84E-10
RAB5IF	0	67.83780072	0.17439475	OK	0
SLA2	1.93E-06	4.618752903	0.01164911	OK	4.21E-06
NDRG3	1.72E-39	13.09663482	0.03359703	OK	6.06E-39
DSN1	4.53E-20	9.09978314	0.02330757	OK	1.33E-19
SAMHD1	0	139.7687799	0.35940051	OK	0
RBL1	3.04E-06	4.523712631	0.01154276	OK	6.57E-06
RPN2	0	62.42162506	0.16047029	OK	0

MANBAL	0	43.6348527	0.11212731	OK	0
SRC	4.10E-05	3.938401592	0.01004403	OK	8.43E-05
BLCAP	1.91E-08	5.498750026	0.01406426	OK	4.47E-08
NNAT	3.56E-32	11.74924633	0.02831935	OK	1.19E-31
CTNNBL1	4.66E-97	20.87353757	0.05360669	OK	2.19E-96
TGM2	0	61.61528708	0.15836005	OK	0
KIAA1755	3.17E-12	6.871599236	0.01749338	OK	8.16E-12
PPP1R16B	9.11E-07	4.772303455	0.01209174	OK	2.01E-06
MAFB	0	206.4356433	0.53086516	OK	0
TOP1	0	57.98701951	0.1490645	OK	0
PLCG1	2.57E-11	6.566536426	0.01675822	OK	6.48E-11
ZHX3	2.32E-41	13.4195082	0.03440868	OK	8.31E-41
LPIN3	2.85E-17	8.371424892	0.02141723	OK	8.00E-17
CHD6	5.28E-14	7.433782219	0.01904176	OK	1.41E-13
SRSF6	1.59E-09	5.922396538	0.01515643	OK	3.82E-09
IFT52	1.68E-39	13.09843354	0.03360643	OK	5.92E-39
MYBL2	0	148.1417581	0.37857955	OK	0
TOX2	2.76E-27	10.75659493	0.02753803	OK	8.80E-27
JPH2	1.85E-75	18.34369959	0.0470323	OK	7.95E-75
OSER1	0	37.70027379	0.0968836	OK	0
OSER1-DT	3.25E-14	7.497527363	0.01917915	OK	8.69E-14
SERINC3	6.96E-75	18.27165049	0.04691737	OK	2.97E-74
PKIG	0	124.939542	0.3212269	OK	0
ADA	3.10E-112	22.48213158	0.05770925	OK	1.54E-111
WISP2	0	86.74365071	0.22295673	OK	0
KCNK15	0	41.99796726	0.10783493	OK	0
RIMS4	6.15E-57	15.8584362	0.03963894	OK	2.41E-56
YWHAB	0	124.2395275	0.31944933	OK	0
TOMM34	2.30E-24	10.1178317	0.02592933	OK	7.14E-24
STK4	0	86.3526686	0.2220139	OK	0
SLPI	0	48.75722083	0.12499535	OK	0
MATN4	1.69E-09	5.911706646	0.01457265	OK	4.07E-09
SDC4	1.45E-230	32.39823696	0.083233	OK	9.56E-230
SYS1	6.12E-51	14.96603845	0.0384133	OK	2.32E-50
TP53TG5	1.53E-11	6.643381993	0.01679942	OK	3.88E-11
PIGT	4.06E-77	18.55028283	0.04763283	OK	1.75E-76
WFDC2	3.67E-235	32.72303775	0.08388361	OK	2.43E-234
DNTTIP1	1.51E-85	19.56581361	0.05024125	OK	6.80E-85
UBE2C	0	216.4436694	0.55397584	OK	0
TNNC2	1.91E-09	5.89186977	0.01498901	OK	4.59E-09
SNX21	4.21E-127	23.95387028	0.06149853	OK	2.18E-126
ACOT8	2.59E-07	5.019702865	0.01283147	OK	5.83E-07
NEURL2	2.72E-08	5.436438361	0.01388477	OK	6.33E-08
CTSA	0	86.66887942	0.22283039	OK	0
PLTP	0	242.1969383	0.62282051	OK	0
PCIF1	1.25E-08	5.572704914	0.01425352	OK	2.95E-08
MMP9	0	133.9185678	0.34416261	OK	0
SLC12A5	2.14E-149	26.00567695	0.06672764	OK	1.18E-148
CD40	1.33E-200	30.19608273	0.0775788	OK	8.28E-200
SLC35C2	4.25E-08	5.356102306	0.01369753	OK	9.82E-08
OCSTAMP	5.46E-06	4.398138961	0.01009536	OK	1.17E-05
TP53RK	4.92E-22	9.578485824	0.02455538	OK	1.49E-21
SLC2A10	5.06E-15	7.73761464	0.01975445	OK	1.37E-14

EYA2	7.78E-29	11.08071681	0.02835664	OK	2.52E-28
ZMYND8	1.20E-06	4.71647117	0.01205253	OK	2.63E-06
NCOA3	1.41E-09	5.941491195	0.01520389	OK	3.41E-09
SULF2	4.06E-188	29.22979062	0.0750742	OK	2.45E-187
PREX1	0	39.98392483	0.10275017	OK	0
AL133342.1	2.48E-31	11.58420466	0.02969773	OK	8.18E-31
CSE1L	2.38E-12	6.912799104	0.01769546	OK	6.13E-12
STAU1	4.26E-243	33.2762197	0.08550955	OK	2.85E-242
DDX27	7.14E-39	12.9881945	0.03332779	OK	2.50E-38
ZNFX1	2.77E-06	4.543015265	0.01160657	OK	6.00E-06
ZFAS1	0	64.03663594	0.16462432	OK	0
KCNB1	1.22E-06	4.712667721	0.01196394	OK	2.68E-06
PTGIS	0	122.8353174	0.31579375	OK	0
B4GALT5	9.83E-59	16.11610576	0.0413566	OK	3.90E-58
RNF114	1.05E-223	31.9074384	0.08198689	OK	6.84E-223
SNAI1	7.76E-08	5.246267898	0.01339679	OK	1.78E-07
TRERNA1	2.28E-05	4.077320174	0.00909159	OK	4.74E-05
UBE2V1	0.00018179	3.565205815	0.00909133	OK	0.00036194
TMEM189	1.30E-13	7.313999291	0.01872949	OK	3.43E-13
CEBPB-AS1	0.00016537	3.589950778	0.0091195	OK	0.00033007
CEBPB	0	173.2811612	0.44559108	OK	0
SMIM25	0	222.4591041	0.57200863	OK	0
PTPN1	0	66.19613274	0.17017436	OK	0
PARD6B	0.00026506	3.465054909	0.00880925	OK	0.00052293
BCAS4	3.64E-67	17.27486608	0.04430725	OK	1.50E-66
ADNP	2.87E-18	8.6375792	0.02213834	OK	8.20E-18
DPM1	3.53E-49	14.69375351	0.03771128	OK	1.33E-48
NFATC2	2.85E-111	22.38347949	0.05744513	OK	1.41E-110
ATP9A	1.93E-32	11.80081028	0.03022489	OK	6.46E-32
TSHZ2	0	75.44198981	0.19331655	OK	0
ZNF217	9.41E-113	22.53506563	0.05786948	OK	4.68E-112
PFDN4	1.06E-69	17.60856865	0.04521017	OK	4.42E-69
DOK5	3.05E-114	22.6864104	0.05822403	OK	1.52E-113
CBLN4	3.65E-17	8.341987114	0.02104908	OK	1.02E-16
FAM210B	4.42E-83	19.27426672	0.04948919	OK	1.97E-82
AURKA	1.29E-207	30.72581105	0.07885326	OK	8.12E-207
CSTF1	0.00015677	3.603854003	0.00918946	OK	0.00031338
CASS4	3.95E-42	13.55020608	0.03473892	OK	1.42E-41
RTF2	0	48.96002847	0.12584781	OK	0
TFAP2C	1.50E-39	13.10709426	0.03347822	OK	5.28E-39
RAE1	1.30E-16	8.190247388	0.02098464	OK	3.63E-16
RBM38	6.25E-82	19.13668892	0.04912689	OK	2.76E-81
PCK1	3.60E-08	5.386173519	0.01365627	OK	8.34E-08
PMEPA1	0	122.3433671	0.31456385	OK	0
RAB22A	3.75E-30	11.34893771	0.0291115	OK	1.23E-29
VAPB	1.93E-23	9.907364363	0.02539823	OK	5.95E-23
APCDD1L	2.24E-09	5.865398334	0.01472194	OK	5.37E-09
GNAS	0	174.3432128	0.44826795	OK	0
NELFCD	1.07E-07	5.186920185	0.0132637	OK	2.44E-07
CTSZ	0	234.3189657	0.60257328	OK	0
ATP5F1E	0	193.6597656	0.4979518	OK	0
PRELID3B	2.96E-210	30.92268972	0.07944735	OK	1.88E-209
ZNF831	1.07E-28	11.05205297	0.02812933	OK	3.46E-28

FAM217B	1.10E-14	7.638188423	0.01956289	OK	2.97E-14
PSMA7	0	153.7903982	0.39545005	OK	0
ADRM1	0	60.37997842	0.15521886	OK	0
LAMA5	1.72E-92	20.36480362	0.05224766	OK	7.96E-92
RPS21	0	165.4735358	0.42547863	OK	0
SLCO4A1	1.42E-63	16.79099601	0.04304348	OK	5.77E-63
MRGBP	2.53E-50	14.871443	0.03816209	OK	9.54E-50
OGFR	1.48E-159	26.88903675	0.06907439	OK	8.35E-159
TCFL5	2.23E-12	6.921887587	0.0176977	OK	5.75E-12
DIDO1	4.90E-15	7.741861469	0.01983	OK	1.33E-14
GID8	8.32E-43	13.66409895	0.03506526	OK	3.00E-42
SLC17A9	1.19E-12	7.010706678	0.01792498	OK	3.08E-12
YTHDF1	2.76E-17	8.374905502	0.02145872	OK	7.77E-17
BIRC7	5.24E-23	9.807196475	0.02497098	OK	1.60E-22
PPDPF	0	112.9009055	0.29029947	OK	0
STMN3	6.60E-38	12.81678621	0.03285107	OK	2.30E-37
ARFRP1	3.93E-18	8.601622665	0.02204441	OK	1.12E-17
SLC2A4RG	1.69E-300	37.03295431	0.09516348	OK	1.22E-299
ZBTB46	2.15E-12	6.926807535	0.01769298	OK	5.56E-12
TPD52L2	6.63E-237	32.84536976	0.08439849	OK	4.40E-236
DNAJC5	1.22E-148	25.93869511	0.06662266	OK	6.71E-148
UCKL1	2.05E-05	4.102232271	0.01047204	OK	4.27E-05
ZNF512B	4.38E-05	3.922603282	0.00999222	OK	8.98E-05
PRPF6	1.20E-50	14.9211094	0.03830009	OK	4.55E-50
SOX18	0	98.77959788	0.25268712	OK	0
TCEA2	7.13E-171	27.83995169	0.07152071	OK	4.16E-170
RGS19	0	131.3597481	0.33776509	OK	0
LKAAEAR1	9.87E-18	8.495266212	0.02162925	OK	2.80E-17
PCMTD2	7.99E-05	3.77529867	0.00963121	OK	0.00016209
PLPP2	7.08E-26	10.45320117	0.02607847	OK	2.23E-25
MIER2	3.95E-05	3.947624848	0.0100665	OK	8.11E-05
SHC2	4.03E-10	6.143846786	0.01563243	OK	9.86E-10
TPGS1	7.49E-107	21.9250623	0.05631301	OK	3.65E-106
CDC34	1.34E-40	13.28890567	0.03410005	OK	4.76E-40
GZMM	1.18E-82	19.22328936	0.04927686	OK	5.24E-82
AC009005.1	6.78E-27	10.67338773	0.02728632	OK	2.15E-26
BSG	0	77.49653744	0.19924172	OK	0
RNF126	4.29E-69	17.52911964	0.04500419	OK	1.79E-68
FSTL3	3.65E-86	19.63816839	0.05039927	OK	1.65E-85
PALM	3.79E-96	20.77309263	0.05287344	OK	1.78E-95
PTBP1	2.87E-26	10.53853979	0.02702727	OK	9.06E-26
AZU1	8.22E-06	4.308490204	0.01095654	OK	1.74E-05
ELANE	0.00016541	3.589891243	0.00900787	OK	0.00033011
CFD	0	224.447769	0.5771759	OK	0
R3HDM4	5.16E-44	13.86500523	0.03557469	OK	1.88E-43
KISS1R	9.04E-13	7.048549219	0.01741782	OK	2.35E-12
ARID3A	1.88E-182	28.78058878	0.0739179	OK	1.12E-181
WDR18	8.89E-35	12.24544064	0.03141389	OK	3.02E-34
TMEM259	2.22E-06	4.590053521	0.01173039	OK	4.82E-06
CNN2	1.00E-293	36.60970182	0.09407799	OK	7.18E-293
ABCA7	2.22E-11	6.588376565	0.01681401	OK	5.61E-11
ARHGAP45	0	51.83418587	0.1332109	OK	0
POLR2E	0	49.98645313	0.12848754	OK	0

GPX4	0	158.2604024	0.40694099	OK	0
SBNO2	2.40E-22	9.652451666	0.02473917	OK	7.28E-22
STK11	1.36E-09	5.947645889	0.01521887	OK	3.28E-09
CBARP	1.39E-06	4.685935124	0.0118872	OK	3.05E-06
ATP5F1D	0	94.49442795	0.24295718	OK	0
MIDN	0	66.80614513	0.17174191	OK	0
CIRBP	0	67.95639307	0.17470253	OK	0
C19orf24	0	59.4737361	0.15288364	OK	0
MUM1	1.87E-33	11.99557284	0.03076954	OK	6.32E-33
NDUFS7	0	59.40908652	0.15272263	OK	0
GAMT	0	46.73542563	0.12010278	OK	0
DAZAP1	1.06E-247	33.59305833	0.08631971	OK	7.13E-247
RPS15	0	195.4632906	0.50253654	OK	0
C19orf25	1.14E-12	7.016217577	0.01796794	OK	2.96E-12
UQCR11	0	104.9879042	0.26994563	OK	0
TCF3	1.80E-06	4.633757899	0.01183399	OK	3.92E-06
ATP8B3	4.86E-09	5.735485299	0.01461287	OK	1.16E-08
REXO1	1.66E-08	5.523161359	0.0141243	OK	3.90E-08
KLF16	3.99E-177	28.35171871	0.07282516	OK	2.35E-176
ABHD17A	2.24E-114	22.69998534	0.05830716	OK	1.12E-113
SCAMP4	1.94E-50	14.88895166	0.03820855	OK	7.35E-50
CSNK1G2	8.47E-39	12.97512698	0.03329023	OK	2.96E-38
MKNK2	9.73E-298	36.86103383	0.09471783	OK	7.02E-297
MOB3A	3.52E-70	17.67085484	0.04535553	OK	1.47E-69
IZUMO4	3.24E-07	4.976629812	0.01267404	OK	7.26E-07
AP3D1	5.10E-13	7.127715692	0.01825409	OK	1.33E-12
DOT1L	4.09E-112	22.46992472	0.05766404	OK	2.03E-111
PLEKHJ1	1.10E-78	18.74333048	0.04812834	OK	4.79E-78
SF3A2	2.22E-05	4.083723327	0.01042265	OK	4.61E-05
OAZ1	0	226.7534865	0.58307685	OK	0
LINGO3	4.06E-12	6.836555665	0.0173619	OK	1.04E-11
LSM7	0	62.56811182	0.1608474	OK	0
TIMM13	0	57.97424827	0.14902943	OK	0
LMNB2	5.88E-22	9.560111923	0.02449574	OK	1.78E-21
GADD45B	0	116.0176992	0.29831199	OK	0
GNG7	1.90E-137	24.92683761	0.06400336	OK	1.02E-136
SLC39A3	1.08E-72	17.99416139	0.04619905	OK	4.57E-72
THOP1	1.91E-09	5.89205166	0.01506496	OK	4.58E-09
TLE2	4.72E-77	18.54216364	0.04755688	OK	2.04E-76
AES	0	46.02029827	0.11828648	OK	0
GNA11	7.62E-49	14.64165918	0.03756101	OK	2.85E-48
GNA15	0	77.97313685	0.2004389	OK	0
AC005264.1	2.24E-05	4.081704267	0.01035599	OK	4.65E-05
S1PR4	8.59E-202	30.28664206	0.07776485	OK	5.35E-201
NCLN	9.16E-44	13.82377173	0.035461	OK	3.33E-43
NFIC	0	66.44842245	0.17082423	OK	0
DOHH	1.68E-05	4.147744886	0.01058881	OK	3.52E-05
MFSD12	0	91.35328697	0.23486289	OK	0
AC005786.2	8.87E-05	3.749064788	0.00878916	OK	0.00017956
HMG20B	4.07E-174	28.10654865	0.07221065	OK	2.39E-173
TBXA2R	2.41E-295	36.71133409	0.09427025	OK	1.73E-294
MRPL54	0	53.39088753	0.13724323	OK	0
MATK	0	100.4621902	0.25815211	OK	0

NMRK2	2.62E-25	10.32831235	0.02390319	OK	8.22E-25
DAPK3	5.14E-196	29.84473132	0.07666218	OK	3.16E-195
EEF2	0	85.00791193	0.21855806	OK	0
ZBTB7A	6.45E-25	10.24172032	0.02626606	OK	2.01E-24
MAP2K2	0	44.00136789	0.113094	OK	0
SIRT6	1.68E-07	5.102423161	0.01304306	OK	3.80E-07
EBI3	0	85.58823148	0.21994688	OK	0
YJU2	2.70E-06	4.5484405	0.0116195	OK	5.85E-06
TMIGD2	1.36E-06	4.691402041	0.01186537	OK	2.97E-06
FSD1	3.25E-33	11.94987614	0.02817415	OK	1.09E-32
MPND	3.36E-18	8.619664201	0.02208674	OK	9.58E-18
SH3GL1	4.68E-20	9.096131192	0.0233139	OK	1.37E-19
CHAF1A	4.95E-116	22.86696232	0.05867394	OK	2.49E-115
UBXN6	2.53E-09	5.845390051	0.01495729	OK	6.05E-09
HDGFL2	2.68E-23	9.874760266	0.02531912	OK	8.22E-23
PLIN5	0.00050972	3.285107284	0.00829456	OK	0.00098799
LRG1	7.93E-36	12.43994554	0.03183207	OK	2.72E-35
SEMA6B	1.57E-85	19.56406729	0.05018384	OK	7.04E-85
TNFAIP8L1	1.02E-31	11.66034229	0.02988677	OK	3.37E-31
MYDGF	0	102.9689827	0.26475638	OK	0
DPP9	1.25E-11	6.673722107	0.01708085	OK	3.17E-11
TICAM1	3.06E-19	8.889987864	0.02277674	OK	8.85E-19
PLIN3	0	72.38954307	0.18610494	OK	0
UHRF1	9.50E-126	23.82364428	0.06085179	OK	4.91E-125
PTPRS	5.81E-12	6.784858097	0.01729449	OK	1.49E-11
SAFB	1.68E-22	9.688814169	0.02484175	OK	5.11E-22
RPL36	0	150.6350138	0.38730949	OK	0
C19orf70	0	62.45004001	0.16054379	OK	0
HSD11B1L	4.57E-11	6.480623418	0.01657468	OK	1.14E-10
LONP1	2.28E-36	12.53926093	0.0321582	OK	7.83E-36
NDUFA11	0	72.06885452	0.18528224	OK	0
RANBP3	1.32E-05	4.202361586	0.0107299	OK	2.78E-05
RFX2	1.11E-12	7.020055626	0.01796727	OK	2.88E-12
CLPP	1.43E-152	26.2847033	0.06752509	OK	7.98E-152
ALKBH7	0	57.64733392	0.14819151	OK	0
GTF2F1	2.00E-75	18.33945159	0.04709097	OK	8.59E-75
KHSRP	4.63E-05	3.909298806	0.00997686	OK	9.48E-05
SLC25A23	2.78E-146	25.72889125	0.06603631	OK	1.52E-145
DENND1C	1.49E-26	10.60029778	0.02713852	OK	4.70E-26
TNFSF9	0	56.75531205	0.14584967	OK	0
TNFSF14	4.09E-192	29.54257261	0.07579514	OK	2.50E-191
C3	0	86.21309365	0.22154499	OK	0
GPR108	5.92E-120	23.25789261	0.05973921	OK	3.00E-119
TRIP10	6.22E-05	3.83727337	0.00979124	OK	0.0001268
VAV1	0	38.38808644	0.09862537	OK	0
ADGRE1	8.11E-30	11.28136727	0.0288577	OK	2.64E-29
INSR	8.37E-37	12.61831472	0.03237043	OK	2.89E-36
PEX11G	5.56E-14	7.426890217	0.01899441	OK	1.48E-13
ZNF358	1.14E-103	21.58888794	0.05544304	OK	5.51E-103
MCOLN1	0	69.21805169	0.17793583	OK	0
PNPLA6	3.71E-57	15.89012703	0.04077697	OK	1.45E-56
XAB2	1.15E-08	5.587890006	0.01428771	OK	2.70E-08
PET100	3.86E-250	33.75964513	0.08675214	OK	2.61E-249

STXBP2	0	124.6820437	0.32057891	OK	0
RETN	4.94E-197	29.92301037	0.07681623	OK	3.05E-196
MCEMP1	0	86.33123535	0.22175043	OK	0
FCER2	4.63E-20	9.097197976	0.02311329	OK	1.36E-19
CLEC4G	1.66E-119	23.2136795	0.05948923	OK	8.39E-119
CD209	0	80.05190798	0.20566486	OK	0
CLEC4M	0.00045417	3.31747614	0.0079138	OK	0.00088351
TGFBR3L	4.74E-14	7.447915547	0.01900012	OK	1.26E-13
SNAPC2	2.28E-36	12.53910687	0.03216925	OK	7.85E-36
CTXN1	8.32E-94	20.51251626	0.05235873	OK	3.87E-93
TIMM44	1.67E-13	7.280240919	0.01864464	OK	4.39E-13
ELAVL1	5.08E-32	11.71930034	0.03006437	OK	1.69E-31
CERS4	5.26E-07	4.881821816	0.01246707	OK	1.17E-06
CD320	0	49.05231766	0.12604875	OK	0
NDUFA7	2.85E-23	9.868569885	0.02528758	OK	8.74E-23
RPS28	0	194.0910844	0.49902486	OK	0
KANK3	6.07E-222	31.78022496	0.08129068	OK	3.93E-221
ANGPTL4	0	42.84237833	0.11005239	OK	0
RAB11B	1.09E-180	28.63917442	0.0735793	OK	6.50E-180
MARCH2	0	70.16079017	0.18037341	OK	0
HNRNPM	0	46.88708598	0.12051658	OK	0
PRAM1	3.74E-115	22.77858358	0.05843497	OK	1.87E-114
MYO1F	0	69.18626013	0.17784544	OK	0
ADAMTS10	5.56E-17	8.292196092	0.02120773	OK	1.56E-16
ZNF426	5.96E-10	6.081189285	0.01555475	OK	1.45E-09
ZNF846	4.49E-13	7.145368661	0.01829304	OK	1.17E-12
FBXL12	6.60E-16	7.992694944	0.02047628	OK	1.81E-15
UBL5	0	124.7927294	0.3208791	OK	0
PIN1	0	50.05850068	0.12867218	OK	0
OLFM2	3.31E-32	11.75535273	0.03003299	OK	1.11E-31
COL5A3	1.10E-05	4.243637417	0.01075131	OK	2.32E-05
C19orf66	8.32E-10	6.02754595	0.0154259	OK	2.02E-09
PPAN	5.09E-38	12.83699834	0.03292762	OK	1.77E-37
P2RY11	1.15E-85	19.57991636	0.05022644	OK	5.16E-85
EIF3G	0	86.49336826	0.22238143	OK	0
DNMT1	0	69.51499592	0.17870142	OK	0
S1PR2	2.98E-14	7.509036414	0.01920992	OK	7.97E-14
MRPL4	7.91E-159	26.82658357	0.06891856	OK	4.47E-158
ICAM1	0	59.51720195	0.15297241	OK	0
ICAM4	3.95E-05	3.947456714	0.01003673	OK	8.12E-05
ZGLP1	1.58E-283	35.96307691	0.09236427	OK	1.12E-282
FDX2	5.64E-06	4.391020535	0.01121098	OK	1.20E-05
ICAM3	0	63.33554559	0.16276239	OK	0
TYK2	2.13E-17	8.405365813	0.02152386	OK	6.01E-17
CDC37	0	40.88202088	0.10507184	OK	0
PDE4A	2.83E-30	11.37359874	0.02916477	OK	9.25E-30
KEAP1	1.30E-21	9.477367137	0.02429392	OK	3.91E-21
CDKN2D	1.96E-289	36.33903409	0.09337361	OK	1.39E-288
SLC44A2	4.10E-71	17.79171076	0.04564967	OK	1.72E-70
ILF3-DT	2.35E-22	9.6548026	0.02475406	OK	7.11E-22
ILF3	5.83E-29	11.10659651	0.0284901	OK	1.89E-28
DNM2	6.71E-60	16.28116693	0.04179201	OK	2.68E-59
TMED1	2.36E-46	14.24641768	0.03656098	OK	8.71E-46

C19orf38	0	51.11720781	0.13134263	OK	0
CARM1	3.44E-09	5.794062758	0.01482079	OK	8.20E-09
YIPF2	7.00E-47	14.33102682	0.03677728	OK	2.59E-46
TIMM29	5.59E-10	6.091459094	0.01558618	OK	1.36E-09
SMARCA4	1.69E-13	7.278708534	0.01864161	OK	4.44E-13
LDLR	2.31E-08	5.465039008	0.01396752	OK	5.40E-08
SPC24	0	81.08193558	0.20702647	OK	0
KANK2	0	115.5337575	0.29703639	OK	0
TMEM205	4.59E-132	24.42544785	0.06274359	OK	2.42E-131
PLPPR2	3.41E-11	6.524684789	0.01668496	OK	8.56E-11
EPOR	1.32E-08	5.563439123	0.01422335	OK	3.11E-08
RGL3	9.50E-22	9.5103911	0.02434278	OK	2.86E-21
CCDC151	1.01E-25	10.4194075	0.02670334	OK	3.18E-25
PRKCSH	1.85E-17	8.421831164	0.02158331	OK	5.23E-17
ECSIT	1.98E-21	9.433635643	0.0241808	OK	5.93E-21
CNN1	0	216.7779821	0.55742183	OK	0
ELOF1	3.94E-35	12.31129305	0.03158537	OK	1.34E-34
ACP5	0	161.695219	0.41576043	OK	0
ZNF441	8.56E-10	6.023099672	0.01539043	OK	2.08E-09
ZNF791	0.00011151	3.691410734	0.0094183	OK	0.00022455
MAN2B1	2.38E-71	17.82220153	0.04573522	OK	9.99E-71
WDR83	1.16E-06	4.72404643	0.01206918	OK	2.54E-06
WDR83OS	0	91.1392869	0.23432864	OK	0
DHPS	3.24E-85	19.52690346	0.05014336	OK	1.46E-84
TRIR	0	84.24814782	0.21660599	OK	0
ASNA1	6.76E-175	28.1703015	0.07237466	OK	3.97E-174
AC018761.3	1.40E-09	5.942720003	0.01481369	OK	3.38E-09
JUNB	0	109.140008	0.28061845	OK	0
PRDX2	0	127.7368651	0.32845343	OK	0
DNASE2	0	55.74967662	0.14330175	OK	0
GCDH	1.28E-09	5.957685655	0.01524019	OK	3.09E-09
SYCE2	5.25E-14	7.434580758	0.01873638	OK	1.40E-13
FARSA	1.36E-36	12.58004031	0.03227398	OK	4.68E-36
CALR	0	120.1068777	0.30882791	OK	0
AC092069.1	6.07E-20	9.067879229	0.02323885	OK	1.77E-19
RAD23A	0	53.62560683	0.13784775	OK	0
GADD45GIP1	0	62.37124642	0.16034108	OK	0
NFIX	0	97.58137767	0.25086321	OK	0
AC138474.1	5.88E-20	9.071290532	0.01886293	OK	1.72E-19
LYL1	0	66.20703534	0.17018679	OK	0
TRMT1	0	41.87693563	0.10761378	OK	0
NACC1	1.39E-37	12.75877854	0.03271741	OK	4.83E-37
STX10	2.34E-237	32.87703622	0.08447953	OK	1.55E-236
IER2	0	41.25442549	0.1060288	OK	0
CACNA1A	4.09E-15	7.764863775	0.01983686	OK	1.11E-14
CCDC130	0.00015221	3.611503413	0.00921267	OK	0.00030452
MRI1	4.73E-05	3.90415634	0.0099608	OK	9.68E-05
C19orf53	0	87.14136856	0.22404713	OK	0
AC020916.1	3.66E-150	26.07328887	0.06697694	OK	2.02E-149
PODNL1	3.61E-46	14.21662099	0.03638112	OK	1.33E-45
DCAF15	0.0002666	3.463502939	0.00882884	OK	0.00052586
IL27RA	3.53E-152	26.25047197	0.06740069	OK	1.96E-151
SAMD1	9.94E-76	18.37752209	0.04718239	OK	4.27E-75

PRKACA	1.99E-23	9.904310027	0.02539442	OK	6.13E-23
AC022098.3	1.04E-09	5.991018632	0.01514912	OK	2.53E-09
ASF1B	0	75.40375171	0.19310193	OK	0
AC022098.1	1.36E-15	7.903508449	0.02022615	OK	3.71E-15
ADGRE5	1.44E-34	12.20613481	0.03131669	OK	4.89E-34
DDX39A	0	47.32848515	0.12164099	OK	0
PKN1	2.35E-88	19.8927268	0.05108432	OK	1.07E-87
GIPC1	5.71E-78	18.65538523	0.04789675	OK	2.48E-77
DNAJB1	0	70.75607416	0.18190175	OK	0
TECR	3.59E-198	30.01041008	0.07710915	OK	2.22E-197
NDUFB7	0	87.99278633	0.22623731	OK	0
ADGRE3	1.59E-09	5.922474162	0.01434592	OK	3.82E-09
ADGRE2	0	56.23271477	0.14452605	OK	0
SYDE1	7.04E-157	26.65899913	0.06843526	OK	3.96E-156
ILVBL	1.11E-24	10.18865187	0.02612416	OK	3.47E-24
NOTCH3	0	93.16573823	0.23947878	OK	0
BRD4	3.76E-16	8.061917193	0.02065998	OK	1.04E-15
AKAP8	2.63E-09	5.838780133	0.01493671	OK	6.29E-09
AKAP8L	0.00011444	3.684797939	0.00940063	OK	0.00023034
RASAL3	1.04E-116	22.9350529	0.05887589	OK	5.22E-116
CYP4F22	1.32E-28	11.03345542	0.02496792	OK	4.26E-28
TPM4	0	90.87701727	0.23365054	OK	0
RAB8A	0	58.39931	0.15012305	OK	0
HSH2D	5.75E-157	26.66654535	0.0682986	OK	3.24E-156
FAM32A	4.11E-106	21.84744081	0.05611388	OK	2.00E-105
AP1M1	7.73E-06	4.322058875	0.01103793	OK	1.64E-05
KLF2	0	51.17713147	0.13154911	OK	0
C19orf44	2.79E-06	4.542035666	0.01156492	OK	6.02E-06
SLC35E1	1.22E-14	7.624586427	0.01953047	OK	3.29E-14
MED26	1.56E-06	4.662815231	0.01190493	OK	3.41E-06
SMIM7	4.38E-159	26.8485748	0.06897759	OK	2.48E-158
TMEM38A	3.29E-39	13.04739095	0.03341391	OK	1.15E-38
CPAMD8	7.79E-08	5.24558026	0.01336107	OK	1.79E-07
HAUS8	1.95E-87	19.78650971	0.05078964	OK	8.82E-87
MYO9B	0	39.3008625	0.10099779	OK	0
USE1	2.50E-38	12.89183291	0.03307877	OK	8.73E-38
OCEL1	3.90E-44	13.88516701	0.0356303	OK	1.42E-43
NR2F6	2.10E-154	26.44476441	0.06791128	OK	1.17E-153
USHBP1	3.43E-196	29.8582393	0.07452882	OK	2.11E-195
BABAM1	5.83E-126	23.84411511	0.0612487	OK	3.01E-125
ABHD8	1.73E-22	9.686135568	0.02482811	OK	5.25E-22
MRPL34	0	44.85807539	0.11529749	OK	0
DDA1	9.10E-31	11.47219759	0.02942786	OK	2.99E-30
GTPBP3	0.00047629	3.304170454	0.00841695	OK	0.0009249
PLVAP	0	86.21603565	0.22077865	OK	0
BST2	0	111.4513116	0.28657226	OK	0
BISPR	9.76E-18	8.496665213	0.02176111	OK	2.77E-17
MVB12A	2.46E-66	17.16419204	0.04406773	OK	1.01E-65
SLC27A1	1.50E-14	7.597955865	0.01945359	OK	4.04E-14
PGLS	0	112.9887482	0.29052718	OK	0
COLGALT1	0	41.76721159	0.10733716	OK	0
MAP1S	2.26E-23	9.891881318	0.02535846	OK	6.93E-23
FCHO1	7.72E-120	23.2464564	0.05965365	OK	3.91E-119

JAK3	3.75E-253	33.96429687	0.08723391	OK	2.55E-252
RPL18A	0	180.9094291	0.46512266	OK	0
CCDC124	1.50E-209	30.8702198	0.07932045	OK	9.51E-209
ARRDC2	2.52E-22	9.647244711	0.02473029	OK	7.66E-22
IL12RB1	7.22E-62	16.55619693	0.04245284	OK	2.91E-61
IFI30	0	199.1100094	0.51201303	OK	0
MPV17L2	9.96E-19	8.75772779	0.02243561	OK	2.86E-18
RAB3A	3.11E-14	7.503338629	0.01920426	OK	8.32E-14
JUND	0	85.83488874	0.22066941	OK	0
LSM4	0	59.58292034	0.1531678	OK	0
PGPEP1	5.72E-09	5.707967908	0.01460008	OK	1.36E-08
GDF15	6.30E-08	5.284475348	0.01350094	OK	1.45E-07
LRRC25	0	92.66252963	0.23822346	OK	0
SSBP4	1.86E-115	22.8090539	0.05858686	OK	9.34E-115
ISYNA1	0	109.6659574	0.28193341	OK	0
ELL	4.98E-53	15.28278558	0.03921903	OK	1.91E-52
FKBP8	0	55.30176082	0.14215852	OK	0
KXD1	9.86E-150	26.03533095	0.06688487	OK	5.44E-149
UBA52	0	180.350376	0.46371102	OK	0
CRLF1	0	53.71112321	0.13769642	OK	0
REX1BD	0	68.00076402	0.17482011	OK	0
TMEM59L	2.93E-23	9.865589135	0.02428504	OK	9.00E-23
CERS1	2.03E-84	19.43299681	0.04821779	OK	9.09E-84
COPE	0	114.1640413	0.29354931	OK	0
DDX49	2.84E-23	9.869033934	0.0253024	OK	8.70E-23
HOMER3	0	80.39199551	0.20667598	OK	0
SUGP2	1.50E-05	4.174047139	0.01065719	OK	3.14E-05
ARMC6	0.00034671	3.39216815	0.00864254	OK	0.00067968
SLC25A42	3.15E-16	8.083533391	0.02066937	OK	8.71E-16
MEF2B	1.01E-06	4.751513495	0.01102448	OK	2.22E-06
BORCS8	4.93E-41	13.36363045	0.03429072	OK	1.76E-40
RFXANK	2.95E-82	19.17572479	0.04923792	OK	1.31E-81
NR2C2AP	1.77E-11	6.622452648	0.01694946	OK	4.47E-11
GATAD2A	2.55E-14	7.5292991	0.01928503	OK	6.83E-14
NDUFA13	0	39.47786712	0.10145965	OK	0
PBX4	0.00025901	3.471259182	0.0087978	OK	0.00051145
LPAR2	2.41E-06	4.572299551	0.01166136	OK	5.23E-06
GMIP	4.50E-137	24.89240631	0.06391868	OK	2.41E-136
ATP13A1	5.41E-07	4.876191089	0.01245624	OK	1.20E-06
ZNF101	1.89E-09	5.893707225	0.01505808	OK	4.54E-09
ZNF93	6.23E-10	6.074259646	0.01552115	OK	1.52E-09
ZNF90	7.65E-07	4.807185573	0.0122745	OK	1.69E-06
AC011447.3	4.37E-10	6.130688945	0.0155461	OK	1.07E-09
ZNF626	5.31E-20	9.08249491	0.02326606	OK	1.55E-19
ZNF85	1.83E-26	10.5809511	0.02711441	OK	5.77E-26
ZNF430	4.18E-06	4.455725893	0.0113809	OK	8.97E-06
ZNF714	3.24E-11	6.532355219	0.01670412	OK	8.14E-11
ZNF738	1.21E-06	4.715119605	0.01203758	OK	2.65E-06
ZNF493	1.48E-06	4.673886866	0.01193715	OK	3.23E-06
ZNF100	2.79E-07	5.004984225	0.01279044	OK	6.28E-07
ZNF43	4.03E-09	5.767337316	0.01474862	OK	9.59E-09
ZNF208	4.14E-16	8.049952475	0.0205699	OK	1.14E-15
ZNF676	6.22E-06	4.369667893	0.01110941	OK	1.33E-05

ZNF98	2.07E-09	5.8787569	0.01472848	OK	4.96E-09
LINC01233	5.11E-10	6.105981605	0.01416249	OK	1.25E-09
ZNF730	9.99E-07	4.753559801	0.01179524	OK	2.20E-06
ZNF91	1.16E-05	4.231624103	0.01080613	OK	2.45E-05
ZNF675	1.53E-11	6.643891841	0.01700362	OK	3.87E-11
AC005616.1	4.78E-06	4.426869662	0.01119505	OK	1.02E-05
UQCRFS1	0	54.54321585	0.14020728	OK	0
POP4	1.17E-117	23.02986604	0.05915221	OK	5.90E-117
PLEKHF1	4.97E-30	11.32442385	0.02901946	OK	1.62E-29
C19orf12	1.25E-20	9.238846739	0.02367882	OK	3.69E-20
CCNE1	2.14E-39	13.08019002	0.03335355	OK	7.51E-39
URI1	1.12E-36	12.59541412	0.03231848	OK	3.86E-36
AC020912.1	3.64E-24	10.07288462	0.02572092	OK	1.13E-23
TSHZ3	3.94E-07	4.938257318	0.0126103	OK	8.81E-07
ZNF507	5.18E-36	12.47395886	0.03195376	OK	1.78E-35
DPY19L3	2.49E-05	4.056645185	0.01033497	OK	5.17E-05
PDCD5	0	61.74913749	0.15874082	OK	0
AC008736.1	4.24E-06	4.452449451	0.01132914	OK	9.10E-06
NUDT19	9.10E-10	6.013154435	0.01538155	OK	2.21E-09
CEP89	5.70E-05	3.85870305	0.00984546	OK	0.00011634
FAAP24	1.39E-05	4.190279701	0.01066867	OK	2.93E-05
GPATCH1	0.00019735	3.543600112	0.00903152	OK	0.0003923
LRP3	3.10E-06	4.519302288	0.01153831	OK	6.70E-06
CEBPA	0	43.23770231	0.11109582	OK	0
CEBPG	2.03E-54	15.48993353	0.03975893	OK	7.83E-54
PEPD	0	70.65646981	0.18164814	OK	0
CHST8	1.51E-17	8.445715963	0.02113346	OK	4.27E-17
LSM14A	3.31E-95	20.66880993	0.05308209	OK	1.55E-94
KIAA0355	1.71E-11	6.627585946	0.01696156	OK	4.31E-11
GPI	0	52.14243355	0.13402287	OK	0
UBA2	0	69.20898911	0.17791958	OK	0
WTIP	0	112.8895953	0.290215	OK	0
SCGB1B2P	6.62E-13	7.091764594	0.01737542	OK	1.73E-12
ZNF302	4.09E-37	12.67449362	0.0325133	OK	1.42E-36
ZNF181	1.13E-12	7.017172295	0.0179561	OK	2.94E-12
ZNF599	6.99E-08	5.265594231	0.01344434	OK	1.60E-07
GRAMD1A	1.14E-07	5.175449767	0.01323312	OK	2.59E-07
SCN1B	0	53.44674438	0.13733406	OK	0
LGI4	0	55.66385419	0.14290396	OK	0
FXYD1	0	250.497631	0.64417276	OK	0
FXYD5	0	188.216725	0.48400908	OK	0
LSR	3.07E-15	7.800842577	0.01997625	OK	8.36E-15
USF2	0	99.56050009	0.25598864	OK	0
HAMP	0	38.02935691	0.09765377	OK	0
FFAR3	1.19E-140	25.220585	0.0646864	OK	6.44E-140
GPR42	4.89E-28	10.91495169	0.02789759	OK	1.57E-27
FFAR2	3.13E-38	12.87463358	0.03296895	OK	1.09E-37
DMKN	0	66.4840293	0.17083825	OK	0
TMEM147	0	51.3930612	0.13210522	OK	0
RBM42	3.44E-96	20.77771268	0.05336231	OK	1.62E-95
COX6B1	0	142.9332494	0.36752852	OK	0
IGFLR1	0	41.91663325	0.10769349	OK	0
U2AF1L4	2.11E-20	9.182134228	0.02353155	OK	6.24E-20

PSENEN	0	56.58711072	0.14546304	OK	0
LIN37	0.00023684	3.495216815	0.00883829	OK	0.00046851
HSPB6	0	133.2082427	0.3424694	OK	0
PROSER3	0.00043076	3.332233951	0.00847594	OK	0.00083989
APLP1	1.18E-20	9.244337733	0.0236296	OK	3.51E-20
NFKBID	0	111.1914537	0.28588111	OK	0
HCST	0	257.8360867	0.66306588	OK	0
TYROBP	0	358.8932136	0.92298829	OK	0
SDHAF1	3.39E-13	7.183784246	0.01839546	OK	8.90E-13
ALKBH6	9.73E-08	5.204356521	0.01329873	OK	2.22E-07
CLIP3	8.86E-75	18.25846331	0.04680352	OK	3.78E-74
THAP8	5.62E-09	5.710746853	0.0145899	OK	1.33E-08
WDR62	1.62E-10	6.287269424	0.01586329	OK	4.00E-10
POLR2I	0	42.36535104	0.10888407	OK	0
TBCB	0	56.6387172	0.14559729	OK	0
CAPNS1	0	46.68641748	0.12000022	OK	0
COX7A1	0	198.8548315	0.51134141	OK	0
ZNF567	1.71E-07	5.098570428	0.0130299	OK	3.88E-07
ZNF568	1.56E-14	7.593268156	0.01943135	OK	4.19E-14
ZNF420	0.00026124	3.468958843	0.00883291	OK	0.00051562
ZNF570	6.64E-06	4.355460898	0.01111134	OK	1.41E-05
ZNF793	1.93E-06	4.61863435	0.01177198	OK	4.21E-06
ZNF540	4.19E-13	7.1548163	0.01828717	OK	1.10E-12
ZNF781	0.00012971	3.652772089	0.00928181	OK	0.00026026
SIPA1L3	3.50E-07	4.96125386	0.0126556	OK	7.85E-07
SPINT2	0	125.8007594	0.32347724	OK	0
PPP1R14A	0	288.3244623	0.74146656	OK	0
C19orf33	7.48E-11	6.405818453	0.01629471	OK	1.86E-10
YIF1B	0	51.97206235	0.13357416	OK	0
KCNK6	1.49E-64	16.92427821	0.04344075	OK	6.07E-64
PSMD8	0	87.37603058	0.22465142	OK	0
FAM98C	3.81E-09	5.776455501	0.01477716	OK	9.10E-09
RASGRP4	1.84E-28	11.00351891	0.02818354	OK	5.92E-28
RYR1	1.00E-65	17.08242391	0.04380179	OK	4.11E-65
MAP4K1	6.81E-92	20.29719133	0.0520319	OK	3.15E-91
EIF3K	0	139.1249729	0.35773723	OK	0
ACTN4	0	106.5670799	0.2740058	OK	0
ECH1	0	61.93009366	0.15920655	OK	0
HNRNPL	1.00E-14	7.650406826	0.01960053	OK	2.70E-14
RINL	2.19E-14	7.549110697	0.019325	OK	5.87E-14
SIRT2	9.67E-47	14.30856567	0.0367216	OK	3.58E-46
NFKBIB	7.70E-10	6.04012907	0.01545571	OK	1.87E-09
MRPS12	0	39.22004463	0.10079242	OK	0
FBXO17	9.31E-305	37.29662511	0.0957876	OK	6.77E-304
FBXO27	1.07E-172	27.99015642	0.07166644	OK	6.26E-172
NCCRP1	0	48.57897912	0.12086456	OK	0
GMFG	0	253.7589741	0.65258187	OK	0
PAF1	3.36E-18	8.619587262	0.02209031	OK	9.59E-18
MED29	1.70E-60	16.36484355	0.04201157	OK	6.82E-60
ZFP36	0	88.57031002	0.22771871	OK	0
PLEKHG2	0.00040881	3.346761426	0.00852605	OK	0.00079794
RPS16	0	175.7550791	0.45190037	OK	0
SUPT5H	9.36E-06	4.279613823	0.01093091	OK	1.98E-05

TIMM50	8.90E-41	13.31959839	0.03417681	OK	3.16E-40
EID2B	4.51E-07	4.911985675	0.0125467	OK	1.00E-06
EID2	9.05E-12	6.720518562	0.01720572	OK	2.31E-11
FBL	1.05E-82	19.22960475	0.04937813	OK	4.65E-82
FCGBP	0	56.3082928	0.14460971	OK	0
PSMC4	0	45.62812347	0.11727671	OK	0
ZNF780A	3.46E-07	4.963635957	0.01268296	OK	7.75E-07
PLD3	0	123.132467	0.31661361	OK	0
SERTAD1	0	55.03781419	0.14147769	OK	0
SERTAD3	2.55E-283	35.94979696	0.09238248	OK	1.80E-282
BLVRB	0	171.0337729	0.43981286	OK	0
SHKBP1	0	56.52920732	0.14530628	OK	0
LTBP4	0	115.2449685	0.29629765	OK	0
NUMBL	7.34E-31	11.4908181	0.02944229	OK	2.41E-30
C19orf54	3.38E-06	4.501377143	0.01147622	OK	7.28E-06
SNRPA	8.60E-88	19.82763507	0.05091652	OK	3.90E-87
CYP2S1	1.86E-230	32.39062164	0.08315225	OK	1.22E-229
AXL	0	52.24463043	0.13428594	OK	0
HNRNPUL1	4.41E-18	8.5884607	0.02201307	OK	1.25E-17
TGFB1	0	90.55115531	0.23281629	OK	0
TMEM91	1.26E-87	19.80824626	0.05085583	OK	5.73E-87
B9D2	2.12E-15	7.847433403	0.02009699	OK	5.79E-15
EXOSC5	6.90E-69	17.50210678	0.04493149	OK	2.87E-68
B3GNT8	5.37E-30	11.31753819	0.02900083	OK	1.75E-29
DMAC2	4.95E-08	5.32859411	0.01362434	OK	1.14E-07
PCAT19	0	53.71024841	0.13538007	OK	0
LINC01480	0	55.12352189	0.14160509	OK	0
AC243960.1	2.96E-173	28.0360446	0.07196498	OK	1.73E-172
CEACAM21	8.02E-51	14.94800224	0.03830094	OK	3.04E-50
CEACAM4	0	48.87287645	0.12554105	OK	0
CEACAM3	6.57E-05	3.823809406	0.00967262	OK	0.00013377
RPS19	0	179.4033062	0.46126063	OK	0
CD79A	2.48E-08	5.452435206	0.01382647	OK	5.79E-08
ARHGEF1	6.90E-132	24.40880142	0.06269078	OK	3.63E-131
RABAC1	0	114.6791344	0.29486983	OK	0
GRIK5	2.92E-09	5.82115384	0.01481947	OK	6.98E-09
POU2F2	0	89.84917681	0.23098195	OK	0
DEDD2	1.31E-90	20.1513889	0.05174335	OK	6.00E-90
GSK3A	7.96E-06	4.315571779	0.01102293	OK	1.69E-05
ERF	7.61E-38	12.80577019	0.03285257	OK	2.65E-37
PAFAH1B3	0	42.73166409	0.10979713	OK	0
TMEM145	5.50E-16	8.015197427	0.02040377	OK	1.51E-15
CNFN	1.18E-12	7.011224895	0.01790741	OK	3.07E-12
LYPD3	8.98E-10	6.015204056	0.01535339	OK	2.18E-09
ETHE1	0	44.40834238	0.11413737	OK	0
XRCC1	3.57E-12	6.854860246	0.01754734	OK	9.17E-12
IRGQ	0.00017734	3.571692948	0.00910353	OK	0.00035345
ZNF576	1.98E-18	8.679932611	0.02224074	OK	5.67E-18
ZNF428	0	46.26052162	0.11890141	OK	0
PLAUR	0	266.1947904	0.68456166	OK	0
KCNN4	0	64.90447244	0.16676257	OK	0
AC243964.2	1.95E-10	6.258097322	0.01564617	OK	4.80E-10
BCL3	0	49.87347043	0.1281791	OK	0

BCAM	0	138.0753901	0.35496936	OK	0
NECTIN2	0	57.50957993	0.14782697	OK	0
TOMM40	2.44E-263	34.6474301	0.08902806	OK	1.68E-262
APOE	0	177.4256011	0.4562415	OK	0
APOC1	0	217.0666588	0.55817567	OK	0
APOC2	4.80E-35	12.29524434	0.03146927	OK	1.64E-34
CLPTM1	1.23E-25	10.40062696	0.0266707	OK	3.87E-25
RELB	0	43.00673066	0.11052022	OK	0
ZNF296	1.23E-53	15.37337836	0.03942112	OK	4.74E-53
GEMIN7	3.08E-15	7.80080021	0.01997691	OK	8.36E-15
TRAPPC6A	7.60E-289	36.30171685	0.09328055	OK	5.40E-288
EXOC3L2	0	41.16771808	0.10298068	OK	0
CKM	2.00E-05	4.107027384	0.01043183	OK	4.18E-05
ERCC2	1.81E-08	5.508693916	0.01407617	OK	4.23E-08
PPP1R13L	5.73E-38	12.82771612	0.03287371	OK	2.00E-37
CD3EAP	4.22E-09	5.759491843	0.01472527	OK	1.00E-08
ERCC1	1.38E-281	35.83865549	0.09209752	OK	9.73E-281
FOSB	0	48.43873948	0.12450574	OK	0
RTN2	1.08E-15	7.932056307	0.02031177	OK	2.95E-15
PPM1N	7.61E-64	16.82800987	0.04315918	OK	3.09E-63
VASP	0	85.32758018	0.21938178	OK	0
OPA3	4.83E-09	5.736681103	0.01466995	OK	1.15E-08
GPR4	1.75E-197	29.95765118	0.07645078	OK	1.08E-196
EML2	2.35E-23	9.88793334	0.02534312	OK	7.21E-23
GIPR	9.04E-13	7.048477881	0.01765992	OK	2.35E-12
SNRPD2	0	82.57764089	0.21230999	OK	0
SIX5	4.27E-34	12.11747541	0.03103889	OK	1.44E-33
DMPK	7.87E-168	27.58744387	0.07081317	OK	4.55E-167
DMWD	2.86E-86	19.6505023	0.05042359	OK	1.29E-85
NOVA2	1.33E-58	16.09757972	0.03989311	OK	5.25E-58
CCDC61	3.43E-05	3.980849257	0.01015624	OK	7.08E-05
HIF3A	4.64E-20	9.096975667	0.02324538	OK	1.36E-19
PPP5C	4.42E-11	6.48540551	0.016592	OK	1.11E-10
CCDC8	5.29E-46	14.18989047	0.03634603	OK	1.95E-45
PNMA8A	2.31E-34	12.16758856	0.0311353	OK	7.84E-34
CALM3	0	93.34548025	0.24000489	OK	0
PTGIR	1.35E-257	34.26390384	0.08801698	OK	9.23E-257
DACT3	0	74.13226823	0.1905354	OK	0
DACT3-AS1	2.53E-07	5.023920353	0.01275485	OK	5.70E-07
SLC1A5	3.88E-57	15.88726358	0.04077296	OK	1.52E-56
AP2S1	0	171.5097428	0.44103941	OK	0
NPAS1	3.29E-22	9.620075041	0.02457089	OK	9.96E-22
TMEM160	0	41.53827622	0.10675875	OK	0
SAE1	1.49E-17	8.447156343	0.02164627	OK	4.22E-17
BBC3	4.08E-14	7.467646708	0.01912349	OK	1.09E-13
INAFM1	0	51.61691007	0.1326612	OK	0
C5AR1	0	293.6509667	0.75517792	OK	0
C5AR2	1.24E-263	34.66701777	0.08904125	OK	8.52E-263
DHX34	2.91E-12	6.883940776	0.01759748	OK	7.49E-12
MEIS3	1.47E-07	5.127648479	0.01305016	OK	3.33E-07
NAPA	2.35E-207	30.70618573	0.078898	OK	1.48E-206
EHD2	0	69.08351662	0.17754692	OK	0
NOP53	0	72.00322828	0.18511344	OK	0

SELENOW	0	170.3883334	0.43815049	OK	0
LIG1	1.25E-54	15.52105256	0.03979972	OK	4.83E-54
CARD8	0	39.56367328	0.1016616	OK	0
CARD8-AS1	9.77E-115	22.73644383	0.05836913	OK	4.88E-114
EMP3	0	154.9960183	0.39856257	OK	0
KDELR1	0	68.83196294	0.17695742	OK	0
GRWD1	1.00E-13	7.348853542	0.01881614	OK	2.65E-13
CYTH2	1.63E-34	12.19608414	0.03129054	OK	5.54E-34
SULT2B1	0.00024175	3.489738378	0.00883917	OK	0.00047805
RPL18	0	202.1530214	0.5197509	OK	0
DBP	2.21E-113	22.59917752	0.05803995	OK	1.10E-112
RASIP1	5.76E-179	28.50057338	0.07263715	OK	3.41E-178
IZUMO1	0.00028862	3.442088353	0.00848734	OK	0.00056793
BCAT2	1.65E-12	6.96418726	0.01782955	OK	4.27E-12
HSD17B14	1.06E-192	29.58823638	0.07599444	OK	6.48E-192
PLEKHA4	1.85E-267	34.91991806	0.08969537	OK	1.28E-266
PPP1R15A	0	65.47918653	0.16833343	OK	0
NUCB1	2.37E-280	35.75930939	0.09189588	OK	1.67E-279
DHDH	9.97E-30	11.2632107	0.0287744	OK	3.25E-29
BAX	0	72.63838571	0.18674707	OK	0
FTL	0	273.7034435	0.70376053	OK	0
RUVBL2	1.20E-31	11.64584618	0.02986953	OK	3.99E-31
LIN7B	2.74E-08	5.434924008	0.01389422	OK	6.38E-08
HRC	1.05E-203	30.43152825	0.07800642	OK	6.58E-203
TRPM4	2.99E-08	5.419505318	0.01385049	OK	6.94E-08
SLC6A16	2.97E-07	4.99301342	0.01269453	OK	6.68E-07
CD37	0	205.6639481	0.52887786	OK	0
TEAD2	1.35E-64	16.93027698	0.04340942	OK	5.49E-64
PIH1D1	1.76E-93	20.47599916	0.05258555	OK	8.19E-93
ALDH16A1	5.71E-89	19.96357621	0.0512559	OK	2.61E-88
FLT3LG	2.72E-24	10.10154206	0.02588043	OK	8.43E-24
RPL13A	0	142.0151425	0.36513326	OK	0
RPS11	0	145.9580409	0.37528694	OK	0
FCGRT	0	254.2050783	0.65372686	OK	0
RCN3	0	54.47569679	0.13998228	OK	0
NOSIP	1.64E-88	19.91092531	0.05113313	OK	7.45E-88
RRAS	0	158.8624557	0.40850872	OK	0
IRF3	9.52E-05	3.731328783	0.00952117	OK	0.00019244
BCL2L12	1.32E-94	20.60180559	0.05289317	OK	6.17E-94
PRMT1	0	44.10037021	0.11334841	OK	0
ADM5	5.74E-64	16.84467932	0.04242663	OK	2.34E-63
FUZ	9.00E-12	6.721448338	0.0172018	OK	2.29E-11
AC006942.1	1.05E-06	4.743529788	0.01208697	OK	2.31E-06
MED25	2.30E-10	6.232381988	0.01594676	OK	5.66E-10
PTOV1	1.74E-26	10.58572574	0.02714829	OK	5.49E-26
PNKP	4.03E-23	9.833699182	0.02521109	OK	1.23E-22
AKT1S1	4.18E-14	7.464651514	0.01911659	OK	1.11E-13
TBC1D17	6.62E-05	3.82182618	0.0097484	OK	0.00013481
IL4I1	0	99.62750582	0.25610865	OK	0
NUP62	2.56E-86	19.65612664	0.05047006	OK	1.16E-85
ATF5	0	83.6295604	0.21499699	OK	0
SIGLEC11	2.76E-06	4.543687086	0.01152568	OK	5.98E-06
VRK3	6.63E-05	3.821728472	0.00975086	OK	0.00013485

KCNC3	9.29E-08	5.213011577	0.01329449	OK	2.12E-07
NR1H2	8.37E-121	23.34169433	0.05995683	OK	4.26E-120
POLD1	4.90E-39	13.01700019	0.03334635	OK	1.72E-38
SPIB	0	109.2533048	0.28056001	OK	0
FAM71E1	5.13E-05	3.884545858	0.00979703	OK	0.00010487
EMC10	1.21E-49	14.76639739	0.03790199	OK	4.54E-49
AC020909.2	2.02E-21	9.431549846	0.02413214	OK	6.05E-21
JOSD2	0	40.60285595	0.10435058	OK	0
LRRC4B	8.10E-08	5.238407673	0.0132804	OK	1.85E-07
C19orf81	4.50E-07	4.912230983	0.0124866	OK	1.00E-06
CLEC11A	0	51.27799363	0.13178876	OK	0
C19orf48	4.43E-28	10.92383519	0.02800459	OK	1.42E-27
KLK1	5.36E-08	5.314201502	0.01347307	OK	1.23E-07
CTU1	2.52E-16	8.110738897	0.02077602	OK	6.97E-16
SIGLEC9	0	41.77226225	0.1073244	OK	0
SIGLEC7	0	46.54122693	0.1195886	OK	0
CD33	0	91.59204337	0.23546841	OK	0
IGLON5	7.82E-06	4.319555373	0.01096348	OK	1.66E-05
ETFB	0	61.94242834	0.15923768	OK	0
NKG7	7.55E-99	21.0696346	0.05407471	OK	3.58E-98
SIGLEC10	0	56.39650813	0.14491577	OK	0
SIGLEC8	3.01E-30	11.36815596	0.02885678	OK	9.85E-30
SIGLEC12	1.68E-07	5.102018736	0.01295917	OK	3.81E-07
SIGLEC6	5.30E-96	20.75702128	0.05018056	OK	2.49E-95
SIGLEC14	1.88E-42	13.60479055	0.03487304	OK	6.75E-42
HAS1	1.84E-23	9.912510263	0.02529434	OK	5.65E-23
FPR1	0	148.6658636	0.38225577	OK	0
FPR2	4.81E-48	14.51589005	0.03715254	OK	1.79E-47
FPR3	0	116.0315678	0.29832202	OK	0
PPP2R1A	2.30E-97	20.90728952	0.05369467	OK	1.08E-96
ZNF766	3.74E-10	6.15569332	0.01574877	OK	9.16E-10
ZNF480	2.31E-15	7.837082424	0.02007263	OK	6.28E-15
ZNF610	0.00012152	3.669478854	0.0092471	OK	0.00024421
ZNF880	2.44E-07	5.030623994	0.01285919	OK	5.51E-07
ZNF83	3.81E-28	10.93760616	0.02804637	OK	1.22E-27
ZNF415	1.07E-12	7.024890737	0.01793121	OK	2.78E-12
ZNF677	5.39E-11	6.45558724	0.01651806	OK	1.35E-10
ZNF331	0	160.266682	0.41211852	OK	0
NLRP12	4.74E-08	5.336504057	0.01359468	OK	1.09E-07
MYADM	6.83E-270	35.07989083	0.09014911	OK	4.74E-269
PRKCG	7.61E-10	6.042045351	0.01434642	OK	1.85E-09
VSTM1	2.61E-10	6.212500976	0.01549861	OK	6.41E-10
OSCAR	0	56.04315691	0.1440329	OK	0
NDUFA3	0	49.36973838	0.12690167	OK	0
TFPT	0	55.05448334	0.14150661	OK	0
PRPF31	8.98E-72	17.87657585	0.04590057	OK	3.78E-71
LENG1	8.33E-25	10.21692835	0.02619897	OK	2.60E-24
MBOAT7	4.67E-234	32.64534162	0.08387189	OK	3.08E-233
TSEN34	0	38.1445134	0.09802923	OK	0
RPS9	0	189.3974537	0.48697147	OK	0
LILRB3	0	90.07892757	0.23157838	OK	0
LILRA6	1.20E-172	27.98598988	0.07184696	OK	7.03E-172
LILRB5	0	122.2937908	0.31432531	OK	0

LILRB2	0	107.3245208	0.27593186	OK	0
LILRA5	1.49E-140	25.21166451	0.06472086	OK	8.06E-140
LILRA4	4.00E-06	4.465329661	0.01134086	OK	8.59E-06
LAIR1	0	185.3998985	0.47674955	OK	0
CDC42EP5	0	53.12575071	0.13648487	OK	0
LAIR2	0.00010787	3.699830743	0.00939607	OK	0.00021737
LILRA2	0	61.27883822	0.1574777	OK	0
LILRA1	1.88E-127	23.98747326	0.06156455	OK	9.75E-127
LILRB1	0	71.51893574	0.18383525	OK	0
LILRB4	0	211.5159177	0.54392295	OK	0
KIR3DL2	2.19E-05	4.086848068	0.01004199	OK	4.56E-05
FCAR	4.97E-181	28.66666945	0.07350449	OK	2.96E-180
AC245128.3	0	41.30778684	0.10602711	OK	0
PPP1R12C	1.29E-19	8.985345924	0.02302709	OK	3.75E-19
TNNT1	2.31E-223	31.88278517	0.08177197	OK	1.50E-222
PPP6R1	9.87E-44	13.81843476	0.03545395	OK	3.58E-43
HSPBP1	1.08E-15	7.931289426	0.02031696	OK	2.97E-15
TMEM150B	3.60E-05	3.969732214	0.01009052	OK	7.41E-05
TMEM238	3.51E-47	14.37892576	0.0368886	OK	1.30E-46
RPL28	0	206.6645813	0.53134202	OK	0
UBE2S	0	63.97770809	0.16446874	OK	0
ISOC2	1.96E-124	23.69645147	0.060865	OK	1.01E-123
NAT14	2.25E-58	16.06471304	0.04119431	OK	8.91E-58
SSC5D	6.08E-16	8.002894503	0.0204468	OK	1.67E-15
ZNF524	2.05E-59	16.21275829	0.04162066	OK	8.15E-59
ZNF580	5.12E-150	26.06049304	0.06694646	OK	2.82E-149
ZNF581	2.12E-48	14.57183802	0.03739532	OK	7.93E-48
CCDC106	6.86E-10	6.058697777	0.01550115	OK	1.67E-09
U2AF2	2.53E-14	7.530598656	0.01928853	OK	6.76E-14
EPN1	8.74E-118	23.04253597	0.05918296	OK	4.41E-117
ZNF787	4.02E-58	16.028761	0.04114483	OK	1.59E-57
ZNF582-AS1	1.51E-10	6.298153695	0.01608657	OK	3.73E-10
ZNF667-AS1	0	37.96205302	0.09752167	OK	0
ZNF471	4.92E-07	4.894883051	0.01247371	OK	1.09E-06
PEG3	2.47E-16	8.112785413	0.02073267	OK	6.86E-16
DUXA	2.54E-05	4.052301518	0.00973033	OK	5.27E-05
AURKC	2.32E-15	7.836290403	0.01993662	OK	6.32E-15
ZNF460	0.00028964	3.441136613	0.00876013	OK	0.00056978
TRAPPC2B	1.63E-31	11.62007392	0.02980737	OK	5.39E-31
ZNF135	6.97E-07	4.82586541	0.01226792	OK	1.54E-06
ZSCAN18	2.59E-159	26.86807669	0.06900391	OK	1.47E-158
AC020915.3	4.05E-06	4.462729579	0.01138939	OK	8.69E-06
AC010642.2	2.08E-27	10.78238939	0.02765078	OK	6.65E-27
A1BG	4.83E-103	21.52213278	0.055274	OK	2.32E-102
RPS5	0	184.4276432	0.47421459	OK	0
ZNF584	6.33E-05	3.832870533	0.00975771	OK	0.00012907
SLC27A5	2.03E-27	10.78496014	0.02764932	OK	6.47E-27
TRIM28	6.97E-54	15.41037782	0.03955634	OK	2.68E-53
CHMP2A	0	70.74848103	0.18188684	OK	0
UBE2M	0	67.79368107	0.17428735	OK	0
RPS4Y1	0	75.77867996	0.19482413	OK	0
TTTY15	3.82E-05	3.955298247	0.01009324	OK	7.86E-05
USP9Y	0.00032578	3.4091905	0.00867961	OK	0.00063922

DDX3Y	8.63E-25	10.21342671	0.02619246	OK	2.69E-24
NLGN4Y	1.26E-11	6.671973958	0.01701715	OK	3.20E-11
KDM5D	4.57E-14	7.452859685	0.01907723	OK	1.22E-13
EIF1AY	3.05E-92	20.33664347	0.05222673	OK	1.41E-91
IL17RA	1.19E-55	15.67118715	0.04021824	OK	4.63E-55
HDHD5	9.78E-122	23.43331953	0.06017932	OK	4.99E-121
ADA2	0	74.50996427	0.19153833	OK	0
ATP6V1E1	1.06E-219	31.61742194	0.08124247	OK	6.86E-219
BCL2L13	2.14E-17	8.405125461	0.02153473	OK	6.02E-17
BID	0	143.4765592	0.36892313	OK	0
LINC00528	3.98E-06	4.466148628	0.01132926	OK	8.56E-06
MICAL3	6.90E-17	8.266473551	0.02115284	OK	1.93E-16
TMEM191B	8.09E-07	4.796176811	0.01223888	OK	1.79E-06
DGCR2	5.20E-05	3.881076015	0.00990048	OK	0.00010634
SLC25A1	3.62E-36	12.50245102	0.03207671	OK	1.24E-35
C22orf39	2.82E-105	21.75926214	0.05588395	OK	1.37E-104
MRPL40	0	39.76122544	0.10218758	OK	0
UFD1	2.79E-245	33.42689513	0.085896	OK	1.87E-244
CDC45	0	81.61682214	0.20822363	OK	0
CLDN5	0	50.5330347	0.12897387	OK	0
TBX1	4.77E-10	6.116776828	0.0155982	OK	1.17E-09
GNB1L	3.28E-08	5.403039506	0.01380302	OK	7.60E-08
TXNRD2	6.52E-05	3.825535736	0.00975844	OK	0.00013285
COMT	0	84.03984152	0.21607197	OK	0
ARVCF	8.02E-40	13.15447932	0.03370555	OK	2.83E-39
TANGO2	4.06E-07	4.932251062	0.01260422	OK	9.08E-07
TRMT2A	1.51E-07	5.121723667	0.01309147	OK	3.44E-07
RANBP1	0	49.24837735	0.12658868	OK	0
RTN4R	6.51E-05	3.82601049	0.00971871	OK	0.0001326
DGCR6L	1.19E-120	23.32669863	0.05991664	OK	6.04E-120
AC007663.3	1.41E-06	4.683423616	0.01166984	OK	3.09E-06
SCARF2	3.68E-75	18.30642513	0.04693856	OK	1.57E-74
PI4KA	2.61E-05	4.04561045	0.01032374	OK	5.41E-05
SNAP29	1.47E-150	26.10813703	0.06706852	OK	8.15E-150
LINC01637	2.61E-109	22.18120545	0.05693816	OK	1.28E-108
THAP7	9.77E-24	9.975324445	0.02557493	OK	3.01E-23
HIC2	2.12E-09	5.874703564	0.0150104	OK	5.08E-09
TMEM191C	0.00010616	3.703894703	0.00943707	OK	0.00021398
UBE2L3	0	70.18217355	0.18043038	OK	0
YDJC	2.81E-135	24.72603719	0.06350452	OK	1.49E-134
SDF2L1	0	66.87077679	0.17190792	OK	0
PPIL2	4.31E-06	4.44904364	0.01136204	OK	9.25E-06
MAPK1	1.15E-64	16.93973461	0.04349023	OK	4.67E-64
PPM1F	1.36E-06	4.690476149	0.01197923	OK	2.98E-06
AC245060.5	2.92E-08	5.423598676	0.01384953	OK	6.79E-08
IGLC2	8.50E-53	15.24787126	0.03913665	OK	3.25E-52
RGL4	1.87E-35	12.37130798	0.03160747	OK	6.38E-35
VPREB3	2.65E-09	5.83761536	0.01465149	OK	6.34E-09
CHCHD10	0	88.61039764	0.22782689	OK	0
MMP11	1.60E-13	7.285818955	0.01853826	OK	4.22E-13
SMARCB1	1.99E-207	30.71156046	0.07890975	OK	1.26E-206
DERL3	2.50E-11	6.571215399	0.01676251	OK	6.29E-11
AP000350.5	0.00039868	3.353712318	0.00826509	OK	0.00077872

MIF	0	135.741896	0.34904537	OK	0
MIF-AS1	6.56E-19	8.804784331	0.02249831	OK	1.89E-18
GSTT2B	1.03E-192	29.5890354	0.07600407	OK	6.33E-192
DDT	0	77.01043292	0.19799243	OK	0
CABIN1	8.92E-17	8.235730814	0.02110185	OK	2.49E-16
SUSD2	1.06E-91	20.27561658	0.05187681	OK	4.88E-91
GGT5	5.40E-256	34.15622729	0.08765118	OK	3.68E-255
GUCD1	1.25E-16	8.195314906	0.02099721	OK	3.48E-16
SNRPD3	0	58.16597816	0.14952521	OK	0
GGT1	0.00025325	3.47729206	0.00881957	OK	0.00050021
LRRC75B	5.09E-20	9.086913501	0.02324404	OK	1.49E-19
SGSM1	5.62E-05	3.862261319	0.00981758	OK	0.00011468
KIAA1671	6.96E-68	17.37002287	0.04455319	OK	2.88E-67
GRK3	0	41.00383172	0.10535878	OK	0
SEZ6L	8.02E-05	3.77444407	0.00945624	OK	0.00016263
ASPHD2	5.70E-13	7.112463029	0.01815988	OK	1.49E-12
HPS4	0.00010466	3.707491741	0.00945619	OK	0.00021104
SRRD	1.44E-12	6.983898299	0.01787976	OK	3.72E-12
TFIP11	1.29E-05	4.207354851	0.01073884	OK	2.72E-05
TPST2	0	46.52254183	0.11956505	OK	0
CRYBB1	3.14E-278	35.62250216	0.09147805	OK	2.20E-277
MN1	2.00E-21	9.432745653	0.02412828	OK	5.98E-21
PITPNB	1.00E-182	28.80239542	0.07399986	OK	5.97E-182
TTC28	2.23E-20	9.176511785	0.02349351	OK	6.58E-20
CHEK2	3.86E-28	10.93649288	0.02800027	OK	1.24E-27
HSCB	6.09E-24	10.02212806	0.02569581	OK	1.88E-23
XBP1	0	57.08877029	0.146753	OK	0
ZNRF3	4.52E-06	4.439092061	0.01131563	OK	9.68E-06
KREMEN1	2.31E-08	5.465573184	0.01394451	OK	5.38E-08
RHBDD3	1.11E-07	5.180310411	0.01323594	OK	2.53E-07
EWSR1	2.76E-05	4.032238446	0.01029617	OK	5.72E-05
GAS2L1	6.34E-24	10.01823364	0.02568195	OK	1.96E-23
RASL10A	2.73E-15	7.815828258	0.01997089	OK	7.43E-15
AP1B1	0	74.1596519	0.1906328	OK	0
NEFH	1.44E-06	4.679654491	0.01191517	OK	3.14E-06
NIPSNAP1	7.69E-05	3.784937916	0.00965365	OK	0.00015605
ZMAT5	2.05E-72	17.95869191	0.04610869	OK	8.67E-72
UQCR10	0	112.1264003	0.28830685	OK	0
ASCC2	4.19E-08	5.358780776	0.01370363	OK	9.68E-08
LIF	9.97E-11	6.361830306	0.01602524	OK	2.48E-10
OSM	0	62.55468393	0.16076312	OK	0
TBC1D10A	4.56E-09	5.74632999	0.01469762	OK	1.08E-08
SF3A1	5.61E-15	7.72464262	0.01978868	OK	1.52E-14
RNF215	1.05E-05	4.253907347	0.01085144	OK	2.22E-05
SEC14L2	0.00027813	3.452095399	0.00872387	OK	0.00054782
MTFP1	3.76E-05	3.958914278	0.01005904	OK	7.75E-05
GAL3ST1	1.48E-11	6.648622121	0.0167888	OK	3.75E-11
PES1	1.19E-15	7.919874613	0.02028455	OK	3.25E-15
TCN2	0	41.30656632	0.10612025	OK	0
SLC35E4	7.98E-12	6.73881701	0.01724107	OK	2.04E-11
SMTN	0	51.77560929	0.13303358	OK	0
SELENOM	0	277.1658454	0.71277807	OK	0
LIMK2	8.60E-06	4.298511622	0.01096899	OK	1.82E-05

PIK3IP1	5.57E-252	33.88484916	0.08705724	OK	3.77E-251
DRG1	9.02E-22	9.515748578	0.02439404	OK	2.71E-21
PISD	1.76E-13	7.273173858	0.01862035	OK	4.63E-13
YWHAH	0	94.1997617	0.24220184	OK	0
RFPL3S	2.66E-09	5.836748814	0.01337401	OK	6.37E-09
RTCB	1.65E-168	27.64388628	0.07102058	OK	9.56E-168
FBXO7	1.65E-59	16.22588818	0.04165492	OK	6.59E-59
TIMP3	0	158.5978234	0.4077909	OK	0
HMGXB4	1.81E-14	7.573945596	0.01940272	OK	4.86E-14
AL008635.1	1.88E-06	4.623829412	0.01164678	OK	4.11E-06
TOM1	1.01E-77	18.62503977	0.04781716	OK	4.37E-77
Z82244.2	2.02E-14	7.559833916	0.01929954	OK	5.41E-14
HMOX1	0	213.3307024	0.54858632	OK	0
MCM5	0	41.11469404	0.10564076	OK	0
RASD2	1.12E-11	6.689444645	0.01674137	OK	2.85E-11
MB	2.27E-99	21.12647309	0.05263897	OK	1.08E-98
APOL6	4.11E-92	20.32194026	0.05218086	OK	1.90E-91
APOL5	1.60E-08	5.529916533	0.01301446	OK	3.75E-08
RBFOX2	0	64.817136	0.16658617	OK	0
APOL3	3.41E-28	10.947699	0.02806095	OK	1.10E-27
APOL4	6.46E-06	4.361593215	0.01110333	OK	1.37E-05
APOL2	1.26E-05	4.21246709	0.01075568	OK	2.66E-05
APOL1	1.62E-28	11.01504647	0.02822923	OK	5.22E-28
MYH9	0	80.98015514	0.20819879	OK	0
TXN2	3.95E-302	37.13417339	0.09543122	OK	2.86E-301
FOXRED2	1.02E-10	6.358923398	0.01624089	OK	2.53E-10
EIF3D	0	48.84398065	0.12554936	OK	0
IFT27	1.18E-21	9.487611899	0.02432051	OK	3.55E-21
NCF4	0	193.1027567	0.49656132	OK	0
CSF2RB	2.02E-142	25.38141046	0.06516965	OK	1.10E-141
TST	6.01E-84	19.37718705	0.04974578	OK	2.69E-83
MPST	5.21E-91	20.19691588	0.05186613	OK	2.40E-90
KCTD17	5.99E-07	4.856017264	0.01240879	OK	1.33E-06
IL2RB	3.89E-06	4.47097645	0.01135819	OK	8.37E-06
RAC2	0	130.9219544	0.33663268	OK	0
CYTH4	0	86.26031231	0.22175657	OK	0
MFNG	0	46.45110812	0.11936683	OK	0
CARD10	2.06E-91	20.24274161	0.050063	OK	9.49E-91
CDC42EP1	0	47.92334976	0.1231414	OK	0
LGALS2	0	179.8391695	0.46203349	OK	0
GGA1	0	39.38316026	0.10120521	OK	0
SH3BP1	0	39.31064384	0.10100187	OK	0
LGALS1	0	190.8333953	0.49070329	OK	0
TRIOBP	4.64E-20	9.096997177	0.02331858	OK	1.36E-19
H1F0	8.01E-74	18.1378143	0.04657087	OK	3.41E-73
GCAT	7.05E-14	7.395307708	0.01890129	OK	1.87E-13
EIF3L	0	69.21395742	0.17793962	OK	0
MICALL1	8.59E-09	5.638188492	0.01439794	OK	2.03E-08
POLR2F	0	40.35789225	0.10372224	OK	0
SLC16A8	2.29E-20	9.17368059	0.02343085	OK	6.75E-20
MAFF	0	37.88695224	0.09736247	OK	0
KDELR3	8.81E-200	30.13354163	0.07728923	OK	5.46E-199
DDX17	2.43E-33	11.97406926	0.0307221	OK	8.18E-33

FAM227A	1.35E-10	6.31516601	0.01580054	OK	3.35E-10
CBY1	0	42.56034008	0.10934925	OK	0
TOMM22	0	42.82175571	0.11005929	OK	0
JOSD1	7.56E-58	15.98950915	0.0410443	OK	2.98E-57
SUN2	0	43.75227376	0.11244151	OK	0
NPTXR	7.12E-17	8.262655938	0.02110486	OK	1.99E-16
CBX6	0	42.98566608	0.11047607	OK	0
APOBEC3A	5.53E-152	26.23337731	0.06719475	OK	3.07E-151
APOBEC3B	1.12E-217	31.46991255	0.07984135	OK	7.20E-217
APOBEC3C	8.22E-75	18.26258228	0.04688949	OK	3.51E-74
APOBEC3G	1.10E-192	29.58689913	0.07600528	OK	6.73E-192
APOBEC3H	2.58E-16	8.107500602	0.02071052	OK	7.16E-16
CBX7	4.20E-92	20.32094191	0.05216648	OK	1.94E-91
PDGFB	0	52.72979647	0.13548524	OK	0
RPL3	0	162.4088584	0.41757222	OK	0
SYNGR1	1.15E-85	19.57972991	0.05027151	OK	5.18E-85
MIEF1	5.68E-09	5.709164101	0.01459886	OK	1.35E-08
ATF4	7.47E-135	24.68648925	0.06341744	OK	3.97E-134
RPS19BP1	0	52.71084775	0.13549495	OK	0
ENTHD1	5.41E-06	4.399905941	0.01075307	OK	1.16E-05
GRAP2	5.59E-15	7.725148881	0.01968685	OK	1.51E-14
TNRC6B	5.54E-15	7.726228754	0.01979665	OK	1.50E-14
ADSL	4.26E-11	6.491216046	0.01660922	OK	1.07E-10
ST13	0	91.93315549	0.23637035	OK	0
RBX1	0	81.164213	0.20867543	OK	0
EP300	4.82E-20	9.092831707	0.0233083	OK	1.42E-19
L3MBTL2	0.00036523	3.377882162	0.00860876	OK	0.00071479
RANGAP1	5.93E-25	10.2498638	0.02627001	OK	1.85E-24
TEF	3.19E-19	8.885297078	0.02274398	OK	9.23E-19
TOB2	2.12E-22	9.665030742	0.02478091	OK	6.44E-22
PHF5A	1.00E-124	23.72462295	0.06094071	OK	5.17E-124
ACO2	5.95E-78	18.65321471	0.04789519	OK	2.58E-77
POLR3H	2.52E-09	5.845722309	0.01495451	OK	6.04E-09
CSDC2	0	46.45903448	0.11934745	OK	0
PMM1	1.84E-246	33.50811325	0.0860914	OK	1.23E-245
DESI1	7.64E-203	30.36635611	0.07801466	OK	4.77E-202
XRCC6	0	51.53372144	0.13246753	OK	0
SNU13	0	69.42010814	0.17847025	OK	0
MEI1	1.49E-79	18.84921963	0.04835424	OK	6.54E-79
SHISA8	1.68E-09	5.913079635	0.01462823	OK	4.04E-09
TNFRSF13C	6.12E-11	6.43628079	0.01628952	OK	1.53E-10
CENPM	0	213.8889886	0.5485723	OK	0
NAGA	0	56.33803137	0.14480643	OK	0
SMDT1	0	73.20567924	0.18820666	OK	0
NDUFA6	0	63.62436877	0.16356379	OK	0
LINC01315	1.96E-23	9.906245157	0.0253737	OK	6.01E-23
NFAM1	1.76E-120	23.30980983	0.0598321	OK	8.95E-120
RRP7A	0	53.93090723	0.13862715	OK	0
CYB5R3	0	134.3229504	0.34539436	OK	0
A4GALT	0	39.85499486	0.10237078	OK	0
ARFGAP3	5.98E-104	21.61875054	0.05552611	OK	2.89E-103
PACSIN2	5.43E-32	11.71350114	0.03004919	OK	1.81E-31
BIK	0	45.29434228	0.11577549	OK	0

MCAT	6.71E-13	7.089967675	0.0181506	OK	1.75E-12
TSPO	0	162.5707961	0.41803726	OK	0
TTLL12	0.00045687	3.315819482	0.0084352	OK	0.00088853
SAMM50	5.19E-39	13.01250503	0.03338671	OK	1.82E-38
PARVB	0	108.3162344	0.27848763	OK	0
PARVG	0	103.9132056	0.26716262	OK	0
AL031595.2	4.41E-08	5.349356263	0.01355701	OK	1.02E-07
PRR5	3.03E-166	27.45502923	0.07047006	OK	1.74E-165
NUP50-DT	0.00023386	3.498597709	0.00891159	OK	0.00046277
NUP50	7.70E-36	12.44229565	0.0319187	OK	2.64E-35
KIAA0930	0	43.72908807	0.11238211	OK	0
UPK3A	0	41.7293569	0.10701696	OK	0
FAM118A	5.19E-22	9.573055544	0.02454325	OK	1.57E-21
SMC1B	0.0001624	3.5946718	0.0085208	OK	0.00032429
RIBC2	4.67E-17	8.312861325	0.02100875	OK	1.31E-16
FBLN1	0	121.4041862	0.31196613	OK	0
ATXN10	3.53E-136	24.80961265	0.0637312	OK	1.88E-135
PRR34-AS1	3.25E-94	20.55821903	0.05278828	OK	1.52E-93
MIRLET7BHG	5.60E-11	6.449760643	0.01647774	OK	1.40E-10
GTSE1	0	210.0984925	0.53741278	OK	0
CELSR1	4.22E-75	18.29890846	0.0468276	OK	1.81E-74
GRAMD4	1.76E-28	11.00747616	0.02820524	OK	5.67E-28
CERK	1.38E-07	5.13956403	0.01313829	OK	3.13E-07
AL118516.1	3.51E-53	15.30550484	0.03928295	OK	1.34E-52
TBC1D22A	4.70E-167	27.52267991	0.07070044	OK	2.71E-166
FAM19A5	1.17E-14	7.629981893	0.01933688	OK	3.16E-14
BRD1	2.32E-05	4.07305641	0.0103995	OK	4.82E-05
CRELD2	1.12E-134	24.66995951	0.06337035	OK	5.97E-134
PIM3	0	130.1102959	0.33455703	OK	0
TRABD	5.99E-262	34.55501378	0.08878757	OK	4.11E-261
AL022328.4	0.00025129	3.479382368	0.00885816	OK	0.00049642
SELENOO	3.33E-07	4.970887115	0.01270222	OK	7.47E-07
HDAC10	1.34E-05	4.199574426	0.01071059	OK	2.81E-05
MAPK12	3.69E-06	4.482297353	0.01139259	OK	7.95E-06
MAPK11	0.00012029	3.672077226	0.00926713	OK	0.00024178
PLXNB2	1.90E-05	4.119234631	0.01051824	OK	3.97E-05
DENND6B	5.99E-191	29.45166947	0.07563842	OK	3.65E-190
LMF2	1.05E-14	7.644028985	0.01958179	OK	2.84E-14
NCAPH2	8.05E-05	3.77348907	0.00962549	OK	0.00016321
TYMP	0	283.7258811	0.72965657	OK	0
ODF3B	0	49.69587918	0.12772981	OK	0
U62317.2	1.88E-17	8.420337517	0.02151319	OK	5.29E-17
U62317.5	2.16E-133	24.55009823	0.06302282	OK	1.14E-132
KLHDC7B	5.80E-19	8.818422036	0.02256274	OK	1.67E-18
ARSA	1.38E-95	20.71101221	0.05318221	OK	6.46E-95
SHANK3	2.42E-26	10.55462898	0.02661939	OK	7.64E-26
GATD3B	2.73E-08	5.435825271	0.01386964	OK	6.35E-08
U2AF1L5	4.75E-07	4.901616147	0.01250661	OK	1.06E-06
HSPA13	6.30E-07	4.846038908	0.01238573	OK	1.40E-06
SAMSN1	0	127.4687343	0.32774481	OK	0
NRIP1	3.17E-193	29.62892021	0.07611841	OK	1.94E-192
USP25	4.22E-05	3.931389029	0.01003248	OK	8.67E-05
MIR99AHG	2.47E-77	18.57697581	0.04759592	OK	1.07E-76

CXADR	1.04E-20	9.257906583	0.02355561	OK	3.09E-20
BTG3	2.16E-189	29.32983586	0.07535773	OK	1.31E-188
C21orf91	0.00029211	3.438841311	0.0087647	OK	0.00057443
CHODL	2.27E-07	5.045183004	0.01263166	OK	5.11E-07
LINC01684	2.24E-05	4.080711574	0.01034757	OK	4.67E-05
MIR155HG	7.28E-121	23.34764524	0.05993806	OK	3.71E-120
MRPL39	7.62E-15	7.685526812	0.01968694	OK	2.06E-14
JAM2	2.96E-125	23.77598643	0.06097122	OK	1.52E-124
ATP5PF	0	116.9566734	0.30072596	OK	0
GABPA	0.00017832	3.570257602	0.00910468	OK	0.00035529
APP	0	63.04279751	0.16206188	OK	0
CYYR1	0	66.83064086	0.17098422	OK	0
ADAMTS1	0	107.3621002	0.27601594	OK	0
ADAMTS5	1.63E-18	8.701978885	0.02194566	OK	4.67E-18
N6AMT1	1.77E-07	5.092624538	0.01301032	OK	4.00E-07
LTN1	3.79E-08	5.37695301	0.01375248	OK	8.77E-08
RWDD2B	5.30E-10	6.100148333	0.01560081	OK	1.29E-09
USP16	1.43E-37	12.75697175	0.03273506	OK	4.94E-37
CCT8	0	52.02511936	0.13373128	OK	0
MAP3K7CL	0	61.95852369	0.1591812	OK	0
BACH1	0	51.22056259	0.1316552	OK	0
GRIK1	0.00013938	3.634274762	0.00915839	OK	0.00027923
TIAM1	3.79E-125	23.76564279	0.06101771	OK	1.95E-124
AP000251.1	2.40E-137	24.91753776	0.05960029	OK	1.29E-136
SOD1	0	139.0307307	0.35750149	OK	0
MIS18A	5.46E-20	9.079403941	0.02326286	OK	1.60E-19
URB1	3.54E-06	4.491395089	0.01145457	OK	7.62E-06
URB1-AS1	3.59E-06	4.488538504	0.01146104	OK	7.72E-06
EVA1C	4.34E-106	21.84493512	0.05606753	OK	2.11E-105
CFAP298	6.11E-33	11.89735112	0.03052137	OK	2.05E-32
SYNJ1	6.74E-05	3.817378947	0.00973608	OK	0.00013719
PAXBP1	0.00037359	3.371653768	0.00859638	OK	0.00073089
IFNAR2	1.48E-236	32.82089146	0.08432678	OK	9.84E-236
IL10RB-DT	0	52.15950222	0.13404212	OK	0
IL10RB	1.62E-06	4.65495487	0.01187114	OK	3.54E-06
IFNAR1	0	47.73668008	0.12269921	OK	0
IFNGR2	0	137.2200938	0.35284337	OK	0
TMEM50B	9.73E-44	13.81946436	0.03546521	OK	3.53E-43
GART	1.41E-09	5.94120993	0.01520178	OK	3.41E-09
SON	3.64E-10	6.160034338	0.01576871	OK	8.92E-10
DONSON	4.72E-05	3.90479512	0.00993786	OK	9.66E-05
CRYZL1	5.92E-16	8.006156481	0.02051327	OK	1.63E-15
ITSN1	9.68E-301	37.04797474	0.09519611	OK	7.00E-300
ATP5PO	0	98.12123884	0.25228643	OK	0
MRPS6	0	67.59436116	0.17377231	OK	0
SLC5A3	4.37E-07	4.918116992	0.01257172	OK	9.75E-07
LINC00310	7.39E-100	21.17940661	0.05430407	OK	3.52E-99
KCNE1	9.76E-64	16.81321636	0.04310964	OK	3.97E-63
RCAN1	1.94E-130	24.27198049	0.06233785	OK	1.02E-129
RUNX1	0	75.86115914	0.19501943	OK	0
CBR1	0	41.88966998	0.10766143	OK	0
CBR3	2.33E-118	23.0996515	0.05930152	OK	1.18E-117
DOPEY2	9.87E-55	15.53616321	0.03985612	OK	3.82E-54

MORC3	5.68E-27	10.68986466	0.02741675	OK	1.80E-26
CHAF1B	1.06E-17	8.486757197	0.0215674	OK	3.01E-17
AP000695.2	5.00E-25	10.26625888	0.02618004	OK	1.56E-24
PIGP	3.10E-84	19.41125817	0.0498472	OK	1.39E-83
TTC3	4.81E-45	14.03424429	0.03602022	OK	1.76E-44
AP001432.1	1.99E-12	6.938140383	0.01620039	OK	5.13E-12
VPS26C	4.07E-37	12.6749255	0.03251538	OK	1.41E-36
KCNJ15	7.29E-59	16.13456314	0.0412459	OK	2.89E-58
LINC01423	8.50E-06	4.301106285	0.01088376	OK	1.80E-05
ERG	0	40.23547829	0.10333927	OK	0
ETS2	0	87.64891501	0.22535296	OK	0
PSMG1	2.07E-50	14.88483722	0.03820301	OK	7.81E-50
HMGN1	0	99.94207608	0.25697139	OK	0
WRB	1.59E-88	19.91231323	0.05112896	OK	7.25E-88
SH3BGR	0	96.78603255	0.24879444	OK	0
PCP4	6.11E-22	9.556184776	0.02401685	OK	1.84E-21
BACE2	0	39.15827267	0.10059711	OK	0
FAM3B	1.28E-30	11.44280836	0.0291132	OK	4.19E-30
MX2	0	72.10713028	0.18535634	OK	0
MX1	1.82E-146	25.74520217	0.06613542	OK	9.98E-146
C2CD2	7.28E-27	10.66677622	0.02732692	OK	2.31E-26
ZBTB21	6.04E-06	4.376230247	0.01117624	OK	1.29E-05
ABCG1	0	47.09636512	0.12103092	OK	0
TFF3	1.33E-97	20.933361	0.05186647	OK	6.28E-97
AP001626.1	1.83E-15	7.86630668	0.01774244	OK	4.98E-15
WDR4	2.34E-09	5.858485583	0.01497076	OK	5.60E-09
NDUFV3	5.24E-146	25.70425557	0.06603367	OK	2.86E-145
SIK1	5.77E-09	5.7064891	0.01455622	OK	1.37E-08
RRP1B	8.57E-20	9.030223322	0.02314603	OK	2.50E-19
PDXK	0	153.8853102	0.39570295	OK	0
CSTB	0	245.7597324	0.63198651	OK	0
RRP1	2.35E-109	22.18593606	0.05696941	OK	1.16E-108
AGPAT3	2.42E-73	18.07687555	0.04640383	OK	1.03E-72
TRAPPC10	1.75E-99	21.13889014	0.05428673	OK	8.30E-99
LINC01678	0	62.67106289	0.16107037	OK	0
AP001056.2	0.00032569	3.409262324	0.00863515	OK	0.00063911
ICOSLG	2.67E-53	15.3234223	0.0392837	OK	1.02E-52
AP001059.3	4.62E-05	3.909860492	0.00994072	OK	9.46E-05
AP001059.2	2.89E-05	4.02173256	0.01017185	OK	5.98E-05
PFKL	6.35E-128	24.03255673	0.06173033	OK	3.30E-127
C21orf2	4.42E-20	9.102455288	0.0233328	OK	1.30E-19
TRPM2	0	38.41659206	0.09866393	OK	0
SUMO3	0	78.8752368	0.20278754	OK	0
PTTG1IP	0	61.40126634	0.1578462	OK	0
ITGB2	0	277.8807113	0.71462107	OK	0
ITGB2-AS1	9.78E-185	28.96246491	0.07435825	OK	5.86E-184
FAM207A	2.59E-139	25.09838024	0.06446856	OK	1.40E-138
ADARB1	1.03E-30	11.4615462	0.02938379	OK	3.38E-30
BX322562.1	4.02E-32	11.7390744	0.03002515	OK	1.34E-31
COL18A1	0	134.369392	0.34548484	OK	0
SLC19A1	1.33E-13	7.31068065	0.01870044	OK	3.51E-13
AJ011932.1	2.08E-46	14.25529101	0.03651676	OK	7.67E-46
COL6A1	0	115.2255137	0.29625466	OK	0

COL6A2	0	186.8427851	0.48045992	OK	0
SPATC1L	1.92E-08	5.497673367	0.0140588	OK	4.49E-08
MCM3AP-AS1	5.68E-06	4.389411452	0.01108238	OK	1.21E-05
YBEY	4.14E-08	5.360907598	0.0137087	OK	9.57E-08
C21orf58	2.35E-37	12.71797818	0.03250638	OK	8.14E-37
S100B	0	47.90021923	0.12293728	OK	0
PRMT2	1.62E-102	21.46601935	0.05513481	OK	7.76E-102
MT-ND1	0	71.94208425	0.18492086	OK	0
MT-ND2	0	81.94350147	0.21063884	OK	0
MT-CO1	0	59.63833105	0.15324029	OK	0
MT-CO2	0	65.25741909	0.16770614	OK	0
MT-ATP8	8.68E-71	17.74958615	0.04557374	OK	3.64E-70
MT-ATP6	0	85.80837975	0.22056642	OK	0
MT-CO3	0	76.19979216	0.19585072	OK	0
MT-ND3	0	94.29042498	0.24239862	OK	0
MT-ND4L	0	61.39536583	0.15782512	OK	0
MT-ND4	0	82.62598459	0.21237858	OK	0
MT-ND5	0	69.30306211	0.17814369	OK	0
MT-ND6	7.33E-31	11.49085191	0.02947615	OK	2.41E-30
MT-CYB	0	111.7805773	0.28734717	OK	0
AC240274.1	9.66E-06	4.272669524	0.01089859	OK	2.04E-05

Table S9. Significanly altered genes for each SMC subtype during atherosclerosis

gene	p_val	avg_log2FC	pct.1	pct.2	p_val_adj	subtype
FCER1G	2.42E-182	0.07483771	0.375	0.927	4.85E-179	SMC1
TMEM176B	6.52E-171	0.00333504	0.223	0.723	1.30E-167	SMC1
TYROBP	1.71E-166	0.12142118	0.362	0.92	3.41E-163	SMC1
C1QA	1.64E-159	0.08002003	0.442	0.947	3.28E-156	SMC1
IGHG4	4.83E-136	0.0031298	0.713	0.953	9.66E-133	SMC1
PTPRE	8.11E-136	0.03018274	0.366	0.879	1.62E-132	SMC1
NRN1	1.47E-134	0.13633284	0.423	0.988	2.94E-131	SMC1
CD300A	1.05E-129	0.01683862	0.063	0.242	2.10E-126	SMC1
PDE4B	6.54E-128	0.14560811	0.298	0.806	1.31E-124	SMC1
CYBB	2.29E-126	0.01868154	0.343	0.664	4.59E-123	SMC1
LINC00607	4.44E-126	-0.0088172	0.233	0.52	8.88E-123	SMC1
MS4A7	8.88E-126	0.01618735	0.248	0.597	1.78E-122	SMC1
KLRD1	4.83E-122	0.00052898	0.511	0.774	9.67E-119	SMC1
ALKAL2	3.68E-120	-0.017336	0.45	0.177	7.36E-117	SMC1
KIT	3.54E-119	0.01743394	0.71	0.301	7.09E-116	SMC1
PCDHGA5	6.82E-119	0.00150713	0.24	0.7	1.36E-115	SMC1
CLDN5	1.71E-117	-0.01402	0.143	0.352	3.42E-114	SMC1
APOC2	8.42E-117	-0.0046663	0.07	0.213	1.68E-113	SMC1
MARCH1	1.56E-116	0.01123503	0.136	0.312	3.12E-113	SMC1
LYZ	4.33E-111	0.13604449	0.324	0.741	8.66E-108	SMC1
SLC8A1	7.53E-110	0.00247125	0.352	0.863	1.51E-106	SMC1
CENPA	1.05E-109	-0.0017237	0.091	0.192	2.09E-106	SMC1
APOC1	1.69E-108	0.04630603	0.526	0.886	3.39E-105	SMC1
FCGR3A	8.47E-108	0.06191973	0.335	0.781	1.69E-104	SMC1
TPSAB1	3.77E-103	0.03888343	0.16	0.372	7.54E-100	SMC1
C1QB	7.89E-103	0.11550542	0.355	0.836	1.58E-99	SMC1
COTL1	8.65E-102	0.13953358	0.501	0.949	1.73E-98	SMC1
GZMA	1.18E-99	0.33840034	0.792	0.987	2.37E-96	SMC1
KANK3	1.90E-98	-0.0273031	0.61	0.3	3.81E-95	SMC1
HLA-DMB	1.55E-97	0.01635242	0.354	0.681	3.11E-94	SMC1
EMX2	3.17E-97	-0.0129203	0.093	0.453	6.34E-94	SMC1
TMEM176A	1.26E-96	0.00798303	0.396	0.709	2.52E-93	SMC1
PLCXD3	6.63E-96	0.01611356	0.199	0.556	1.33E-92	SMC1
LAPTM5	1.24E-93	0.19003124	0.757	0.969	2.47E-90	SMC1
DKK2	2.18E-93	0.00324694	0.073	0.13	4.36E-90	SMC1
LCP1	3.32E-93	0.14704695	0.593	0.936	6.63E-90	SMC1
LINC02185	4.05E-93	-0.0047665	0.517	0.872	8.09E-90	SMC1
ITLN1	9.20E-92	0.0764509	0.097	0.351	1.84E-88	SMC1
CPA3	2.95E-89	0.05377317	0.13	0.192	5.89E-86	SMC1
AC103591.3	1.30E-88	-0.0488824	0.576	0.294	2.61E-85	SMC1
C15orf48	2.56E-88	0.01141903	0.285	0.544	5.12E-85	SMC1
LINC01094	2.63E-88	0.00197471	0.296	0.474	5.25E-85	SMC1
B3GNT5	2.72E-88	0.00621473	0.178	0.326	5.44E-85	SMC1
RBP7	2.01E-86	0.01806097	0.404	0.797	4.02E-83	SMC1
CD48	1.24E-85	0.22839103	0.782	0.981	2.47E-82	SMC1
C1QC	1.26E-84	0.05721343	0.363	0.753	2.52E-81	SMC1
SHTN1	1.37E-84	0.0433208	0.443	0.828	2.75E-81	SMC1
PODN	1.78E-83	-0.0599639	0.571	0.294	3.56E-80	SMC1
LTB	2.59E-83	0.32000596	0.624	0.965	5.17E-80	SMC1
SULT1A1	8.64E-83	-0.0009077	0.424	0.179	1.73E-79	SMC1
CLEC4E	2.56E-82	0.00089944	0.122	0.191	5.11E-79	SMC1

ACP5	3.63E-81	0.01477941	0.634	0.772	7.26E-78	SMC1
GZMM	5.14E-81	0.05454475	0.758	0.922	1.03E-77	SMC1
ANKRD28	6.62E-81	0.02280383	0.549	0.862	1.32E-77	SMC1
BMP2	2.47E-79	-0.0442001	0.6	0.314	4.93E-76	SMC1
CD3E	8.56E-79	0.15452919	0.602	0.914	1.71E-75	SMC1
PLXNA4	1.23E-78	0.01445976	0.14	0.294	2.47E-75	SMC1
TSPAN15	1.50E-78	-0.0093245	0.521	0.69	3.00E-75	SMC1
EMILIN2	1.05E-77	-0.0178251	0.676	0.864	2.09E-74	SMC1
CXCL8	1.07E-76	0.08381733	0.405	0.741	2.15E-73	SMC1
CYYR1	7.94E-76	0.01929769	0.704	0.37	1.59E-72	SMC1
LOXL2	8.33E-76	-0.0232107	0.698	0.901	1.67E-72	SMC1
IGFBP3	8.09E-75	0.08727082	0.306	0.797	1.62E-71	SMC1
CCL3	1.87E-74	0.11706468	0.746	0.988	3.73E-71	SMC1
FMO3	2.74E-74	-0.0407844	0.681	0.969	5.48E-71	SMC1
CD83	2.50E-73	0.01474797	0.649	0.892	4.99E-70	SMC1
CCNA1	7.59E-73	-0.0176471	0.088	0.115	1.52E-69	SMC1
AC245014.3	6.18E-71	0.00680143	0.517	0.226	1.24E-67	SMC1
HTR2B	4.27E-70	0.01833674	0.404	0.69	8.54E-67	SMC1
LEFTY2	4.38E-70	0.07908016	0.269	0.614	8.77E-67	SMC1
CST7	5.25E-70	0.15097204	0.582	0.888	1.05E-66	SMC1
ALOX5AP	2.38E-69	0.13220626	0.715	0.938	4.76E-66	SMC1
BARX1	6.39E-69	-0.0357987	0.512	0.281	1.28E-65	SMC1
RHOH	9.38E-69	0.13919302	0.66	0.933	1.88E-65	SMC1
PTGER3	1.70E-68	0.18557399	0.31	0.833	3.40E-65	SMC1
PLAT	3.25E-68	-0.0460071	0.595	0.798	6.50E-65	SMC1
MEOX1	8.11E-68	0.00248243	0.641	0.286	1.62E-64	SMC1
TNFAIP3	8.86E-68	0.16918558	0.751	0.99	1.77E-64	SMC1
APOE	1.03E-67	0.11974089	0.7	0.991	2.07E-64	SMC1
GJB2	1.09E-67	-0.0027026	0.043	0.138	2.18E-64	SMC1
GOLM1	1.48E-67	0.00139775	0.36	0.72	2.97E-64	SMC1
NRP2	1.77E-65	0.14476479	0.352	0.83	3.54E-62	SMC1
NEGR1	3.06E-65	-0.0027096	0.217	0.443	6.12E-62	SMC1
GBP5	3.23E-65	0.06313635	0.747	0.866	6.46E-62	SMC1
KRT16	5.55E-65	-8.54E-05	0.083	0.194	1.11E-61	SMC1
POSTN	7.24E-65	0.1930385	0.559	0.931	1.45E-61	SMC1
IL1RN	9.03E-65	0.02235644	0.21	0.358	1.81E-61	SMC1
FCGBP	1.36E-64	0.00369169	0.094	0.149	2.72E-61	SMC1
DMXL2	1.43E-64	0.03268356	0.449	0.214	2.85E-61	SMC1
MS4A6A	2.92E-64	0.04356886	0.558	0.864	5.84E-61	SMC1
TREM1	1.24E-63	-0.0153634	0.118	0.22	2.49E-60	SMC1
ADM	4.35E-63	-0.0323405	0.582	0.863	8.70E-60	SMC1
CD4	4.53E-62	0.02870373	0.306	0.49	9.06E-59	SMC1
CENPF	1.12E-61	0.00753668	0.219	0.342	2.23E-58	SMC1
KCNK15	2.08E-61	0.15398966	0.392	0.801	4.15E-58	SMC1
EPSTI1	3.77E-61	0.015783	0.638	0.88	7.54E-58	SMC1
BCL11A	1.83E-60	0.00636141	0.104	0.244	3.66E-57	SMC1
S100A9	1.90E-60	0.03552335	0.604	0.838	3.80E-57	SMC1
TBX1	3.55E-60	-0.0085595	0.278	0.558	7.09E-57	SMC1
IFI27	4.13E-60	-0.2025773	0.764	0.934	8.26E-57	SMC1
CCL8	4.48E-60	-0.2590084	0.605	0.406	8.96E-57	SMC1
BEX1	1.46E-59	0.03464468	0.379	0.197	2.91E-56	SMC1
NCAPG	6.00E-59	0.00044527	0.132	0.19	1.20E-55	SMC1
VEGFD	1.06E-58	0.00240953	0.198	0.258	2.11E-55	SMC1

RNASE1	1.73E-58	0.07052223	0.654	0.916	3.46E-55	SMC1
F13A1	5.67E-58	0.01583541	0.448	0.612	1.13E-54	SMC1
CORO1A	1.02E-57	0.2686357	0.725	0.94	2.04E-54	SMC1
INHBB	2.63E-57	0.00385375	0.085	0.202	5.27E-54	SMC1
GDF15	3.10E-56	-0.2091943	0.671	0.44	6.21E-53	SMC1
EGFL6	1.22E-55	-0.0338319	0.806	0.902	2.45E-52	SMC1
TCEAL6	2.21E-54	-0.0213741	0.135	0.216	4.43E-51	SMC1
CLDN1	2.68E-54	-0.0088465	0.767	0.797	5.35E-51	SMC1
SLC1A3	1.72E-53	-0.0060503	0.631	0.727	3.44E-50	SMC1
LNP1	2.61E-53	-0.0328727	0.437	0.78	5.23E-50	SMC1
AC040970.1	3.18E-53	0.00155441	0.146	0.2	6.36E-50	SMC1
ECM1	3.41E-53	-0.0304414	0.777	0.892	6.81E-50	SMC1
CKAP2L	4.11E-53	0.00193511	0.533	0.248	8.22E-50	SMC1
FST	6.18E-52	0.03942219	0.756	0.547	1.24E-48	SMC1
GPBAR1	2.25E-51	-0.059666	0.536	0.377	4.49E-48	SMC1
ASGR1	2.69E-51	0.01061115	0.068	0.186	5.38E-48	SMC1
FCRLA	3.10E-51	-0.0049422	0.135	0.315	6.21E-48	SMC1
C3AR1	4.92E-51	0.01850778	0.365	0.513	9.84E-48	SMC1
CLSPN	7.82E-51	0.00348742	0.405	0.528	1.56E-47	SMC1
SMIM25	1.39E-50	0.02768955	0.619	0.348	2.78E-47	SMC1
SOX7	5.48E-50	0.00631148	0.579	0.295	1.10E-46	SMC1
CD93	5.86E-50	0.00839368	0.608	0.406	1.17E-46	SMC1
EDNRB	9.17E-50	-0.1079293	0.753	0.901	1.83E-46	SMC1
RGS13	9.69E-50	0.0078978	0.663	0.381	1.94E-46	SMC1
DHFR	1.36E-49	0.02409044	0.307	0.671	2.72E-46	SMC1
PART1	1.71E-49	0.05350687	0.238	0.442	3.42E-46	SMC1
MSR1	4.66E-49	0.02243168	0.216	0.251	9.31E-46	SMC1
PAMR1	1.21E-48	-0.0447994	0.635	0.312	2.41E-45	SMC1
CD69	1.66E-48	0.3321811	0.828	0.979	3.32E-45	SMC1
LINC00924	2.39E-48	-0.0177026	0.524	0.356	4.79E-45	SMC1
BCL2A1	2.66E-48	0.02564047	0.574	0.777	5.32E-45	SMC1
C1QTNF3	4.57E-48	0.00242212	0.542	0.186	9.14E-45	SMC1
SPTSSB	4.63E-48	-0.004126	0.585	0.421	9.26E-45	SMC1
NDC80	5.71E-48	0.01534938	0.577	0.75	1.14E-44	SMC1
DUSP23	1.05E-47	0.06098832	0.448	0.843	2.09E-44	SMC1
UCP2	1.29E-47	0.06561342	0.462	0.728	2.58E-44	SMC1
CENPW	1.30E-47	0.02231076	0.521	0.365	2.59E-44	SMC1
HCST	4.53E-47	0.22812954	0.696	0.977	9.05E-44	SMC1
PLAC8	6.00E-47	0.04394949	0.708	0.802	1.20E-43	SMC1
AREG	1.00E-46	0.27833279	0.489	0.808	2.01E-43	SMC1
EFNB2	1.61E-46	0.11012462	0.246	0.635	3.22E-43	SMC1
FBLN2	2.22E-46	-0.0508065	0.745	0.928	4.45E-43	SMC1
COL5A2	2.38E-46	0.06191347	0.352	0.702	4.76E-43	SMC1
HS3ST1	3.42E-46	-0.0037844	0.627	0.458	6.84E-43	SMC1
SLC16A10	3.70E-46	0.01216216	0.31	0.548	7.39E-43	SMC1
CXCL14	4.23E-46	-0.0049918	0.342	0.095	8.47E-43	SMC1
RND1	5.66E-46	0.00618054	0.406	0.238	1.13E-42	SMC1
AC092803.2	5.99E-46	-0.0099615	0.322	0.603	1.20E-42	SMC1
HCK	9.81E-46	-0.0006724	0.687	0.771	1.96E-42	SMC1
TTN	1.37E-45	0.01173603	0.328	0.493	2.75E-42	SMC1
CRABP2	1.72E-45	-0.0098428	0.768	0.872	3.44E-42	SMC1
PRSS35	2.52E-45	0.13985247	0.358	0.786	5.04E-42	SMC1
CCL3L1	2.70E-45	0.05273586	0.631	0.856	5.39E-42	SMC1

APLN	8.24E-45	0.00609387	0.698	0.438	1.65E-41	SMC1
NR2F1	1.03E-44	0.00198685	0.617	0.307	2.07E-41	SMC1
CRHBP	1.49E-44	0.00309746	0.101	0.158	2.98E-41	SMC1
RAPGEF5	1.66E-44	0.00854419	0.536	0.859	3.33E-41	SMC1
SGCG	1.73E-44	-0.0036956	0.567	0.281	3.45E-41	SMC1
SAMD11	1.79E-44	-0.0391412	0.692	0.473	3.57E-41	SMC1
HAND2	1.79E-44	0.03923134	0.735	0.488	3.57E-41	SMC1
TPD52L1	2.41E-44	0.10445766	0.33	0.766	4.81E-41	SMC1
FMOD	9.87E-44	0.11691865	0.253	0.624	1.97E-40	SMC1
PID1	2.12E-43	0.22112313	0.364	0.856	4.24E-40	SMC1
HHIP-AS1	5.28E-43	0.00787588	0.088	0.16	1.06E-39	SMC1
CCDC102B	5.46E-43	-0.1449201	0.85	0.959	1.09E-39	SMC1
JDP2	5.57E-43	-0.030045	0.563	0.819	1.11E-39	SMC1
FGF18	8.66E-43	-0.0068	0.48	0.262	1.73E-39	SMC1
PLA2G5	1.27E-42	-0.0094036	0.414	0.66	2.54E-39	SMC1
FAM111B	2.11E-42	0.00134251	0.098	0.203	4.23E-39	SMC1
HOPX	2.15E-42	-0.002256	0.723	0.892	4.29E-39	SMC1
ENTPD3	2.77E-42	-0.0710823	0.331	0.179	5.55E-39	SMC1
GUCY1A2	3.27E-42	-0.0262283	0.751	0.841	6.55E-39	SMC1
ADAMTS9	4.56E-42	-0.0170842	0.452	0.307	9.11E-39	SMC1
PLLP	5.04E-42	0.01043367	0.56	0.335	1.01E-38	SMC1
KRT18	5.64E-42	-0.0747194	0.782	0.852	1.13E-38	SMC1
VSIG4	7.27E-42	0.01259126	0.407	0.595	1.45E-38	SMC1
TPX2	7.33E-42	-0.0096807	0.256	0.433	1.47E-38	SMC1
IBSP	7.67E-42	0.00982479	0.229	0.303	1.53E-38	SMC1
GXYLT2	1.16E-41	0.12924041	0.405	0.748	2.33E-38	SMC1
CH25H	2.35E-41	-0.6762845	0.693	0.534	4.70E-38	SMC1
ASGR2	2.79E-41	0.00083896	0.036	0.192	5.57E-38	SMC1
FAM107A	3.17E-41	0.00819664	0.627	0.398	6.35E-38	SMC1
CREB5	4.18E-41	-0.0178396	0.522	0.352	8.37E-38	SMC1
CTSH	8.84E-41	0.00730341	0.227	0.634	1.77E-37	SMC1
ICOS	1.11E-40	0.05244827	0.79	0.891	2.22E-37	SMC1
C5AR1	1.14E-40	0.02374382	0.252	0.41	2.28E-37	SMC1
LAMB1	1.27E-40	0.05213262	0.623	0.862	2.54E-37	SMC1
MAP1A	3.47E-40	-0.0869989	0.53	0.808	6.94E-37	SMC1
DIAPH3	5.23E-40	-0.0074412	0.274	0.47	1.05E-36	SMC1
SOX9	6.78E-40	0.0014645	0.76	0.452	1.36E-36	SMC1
SIX1	8.22E-40	-0.017943	0.611	0.85	1.64E-36	SMC1
CD70	1.04E-39	0.01775835	0.622	0.685	2.07E-36	SMC1
MX2	1.05E-39	0.01080167	0.796	0.949	2.09E-36	SMC1
SNAI1	1.19E-39	-0.0164645	0.549	0.395	2.37E-36	SMC1
PLXDC1	1.27E-39	-0.0412864	0.775	0.841	2.55E-36	SMC1
TMEM158	1.73E-39	-0.0099631	0.72	0.716	3.46E-36	SMC1
CXCR6	1.98E-39	0.03468984	0.17	0.298	3.95E-36	SMC1
CXorf36	2.64E-39	-0.0148737	0.675	0.32	5.29E-36	SMC1
ERRFI1	4.14E-39	0.07578703	0.429	0.826	8.28E-36	SMC1
CYP26B1	4.49E-39	0.00600886	0.636	0.449	8.98E-36	SMC1
DNASE1L3	5.95E-39	0.01407557	0.687	0.416	1.19E-35	SMC1
CYFIP2	6.05E-39	0.13940252	0.405	0.873	1.21E-35	SMC1
CTLA4	7.85E-39	0.04321357	0.232	0.227	1.57E-35	SMC1
TSLP	1.12E-38	0.02697469	0.382	0.709	2.23E-35	SMC1
PTPRB	2.53E-38	0.00593467	0.173	0.222	5.06E-35	SMC1
CDCA3	4.62E-38	-0.0030432	0.727	0.379	9.24E-35	SMC1

AL121944.1	5.00E-38	-0.0098018	0.472	0.319	1.00E-34	SMC1
MMP11	1.35E-37	-0.0448004	0.789	0.912	2.70E-34	SMC1
CLEC10A	1.37E-37	0.00656614	0.126	0.148	2.74E-34	SMC1
HLA-DRA	1.62E-37	0.18953429	0.865	0.999	3.25E-34	SMC1
CDKN3	2.38E-37	-0.0026093	0.633	0.744	4.77E-34	SMC1
EFNA1	2.84E-37	-0.0692375	0.429	0.338	5.68E-34	SMC1
MAL	3.59E-37	-0.0395878	0.538	0.392	7.18E-34	SMC1
CD3G	4.93E-37	0.20325241	0.651	0.937	9.86E-34	SMC1
TNFSF9	5.33E-37	0.01139842	0.685	0.771	1.07E-33	SMC1
DSP	5.36E-37	-0.0079726	0.527	0.665	1.07E-33	SMC1
FAM133A	5.82E-37	0.02362445	0.323	0.58	1.16E-33	SMC1
PRDM1	1.13E-36	0.10870597	0.736	0.916	2.26E-33	SMC1
SEMA3B	3.95E-36	-0.0220903	0.254	0.559	7.89E-33	SMC1
BIRC3	5.77E-36	0.13344721	0.484	0.793	1.15E-32	SMC1
LEPR	6.49E-36	-0.0020787	0.202	0.455	1.30E-32	SMC1
PMAIP1	8.39E-36	0.01589013	0.719	0.888	1.68E-32	SMC1
OSR1	1.10E-35	0.10354286	0.274	0.578	2.21E-32	SMC1
ALDH1A2	1.12E-35	-0.0067076	0.456	0.762	2.24E-32	SMC1
RAD51AP1	1.31E-35	-0.0014828	0.329	0.258	2.63E-32	SMC1
PDGFB	1.32E-35	-0.0190751	0.426	0.291	2.63E-32	SMC1
SERPINE2	1.56E-35	-0.0014008	0.609	0.743	3.13E-32	SMC1
HLA-DOB	1.56E-35	-0.0085495	0.637	0.337	3.13E-32	SMC1
KPNA2	1.58E-35	0.00285609	0.583	0.764	3.16E-32	SMC1
PLD5	1.59E-35	0.03721284	0.179	0.242	3.19E-32	SMC1
GABRD	3.55E-35	-0.0324176	0.451	0.34	7.11E-32	SMC1
ADAMTS6	3.59E-35	0.02549547	0.357	0.597	7.17E-32	SMC1
SMPDL3A	6.57E-35	0.00510768	0.456	0.659	1.31E-31	SMC1
GRIA2	8.75E-35	0.336776	0.345	0.763	1.75E-31	SMC1
CENPU	1.87E-34	0.00807146	0.278	0.519	3.75E-31	SMC1
KRT14	3.14E-34	-0.0019723	0.077	0.101	6.28E-31	SMC1
HLA-DQA1	6.71E-34	0.04936005	0.47	0.75	1.34E-30	SMC1
OASL	7.24E-34	0.02554997	0.773	0.899	1.45E-30	SMC1
EGFL7	1.82E-33	0.00058252	0.367	0.597	3.64E-30	SMC1
EVI2B	1.82E-33	0.09062651	0.588	0.741	3.65E-30	SMC1
FYB1	2.61E-33	0.1761795	0.775	0.908	5.23E-30	SMC1
DSC2	2.65E-33	0.00769749	0.189	0.199	5.30E-30	SMC1
SFTA1P	3.37E-33	-0.0777397	0.8	0.867	6.74E-30	SMC1
LSAMP	3.56E-33	-0.0405034	0.616	0.351	7.12E-30	SMC1
THEMIS2	4.16E-33	0.01928889	0.366	0.42	8.31E-30	SMC1
IGF1	4.47E-33	0.02191087	0.238	0.262	8.94E-30	SMC1
FPR3	8.71E-33	0.00683778	0.039	0.147	1.74E-29	SMC1
MGP	9.57E-33	0.52470853	0.995	1	1.91E-29	SMC1
CD72	1.40E-32	0.00730138	0.537	0.219	2.80E-29	SMC1
ADGRF5	1.74E-32	-0.0071335	0.771	0.9	3.47E-29	SMC1
ALDH1A3	1.92E-32	0.00252396	0.254	0.301	3.83E-29	SMC1
CD163	1.99E-32	0.01216265	0.741	0.887	3.99E-29	SMC1
DKK1	2.21E-32	0.00123323	0.018	0.12	4.43E-29	SMC1
VWA5A	2.43E-32	0.02232324	0.414	0.731	4.86E-29	SMC1
SLC7A7	2.70E-32	0.01987056	0.213	0.355	5.41E-29	SMC1
AIF1	2.97E-32	0.11926459	0.465	0.729	5.94E-29	SMC1
FBXL22	3.67E-32	0.04397925	0.302	0.633	7.34E-29	SMC1
ITGA1	6.36E-32	0.12888723	0.576	0.942	1.27E-28	SMC1
PRSS23	6.87E-32	0.12403117	0.664	0.955	1.37E-28	SMC1

AP1S2	7.53E-32	0.08836342	0.522	0.855	1.51E-28	SMC1
PRG4	9.65E-32	-0.0083349	0.484	0.209	1.93E-28	SMC1
LAIR1	1.11E-31	0.01941543	0.261	0.364	2.22E-28	SMC1
CCL4L2	1.19E-31	0.26408493	0.762	0.964	2.38E-28	SMC1
GALNT5	1.25E-31	0.0240299	0.699	0.479	2.50E-28	SMC1
NCF4	1.79E-31	0.0223073	0.361	0.579	3.58E-28	SMC1
KYNU	2.53E-31	-0.00336	0.465	0.523	5.05E-28	SMC1
RASD1	2.87E-31	-0.309086	0.664	0.487	5.73E-28	SMC1
PTGS2	2.89E-31	-0.0116905	0.521	0.384	5.78E-28	SMC1
TNFSF18	2.91E-31	-0.000769	0.095	0.116	5.82E-28	SMC1
BICC1	3.01E-31	0.04301305	0.396	0.772	6.03E-28	SMC1
HELLS	3.36E-31	0.00650382	0.678	0.801	6.73E-28	SMC1
HIGD1B	3.57E-31	-0.0630431	0.797	0.831	7.13E-28	SMC1
SPON2	3.81E-31	-0.0022074	0.544	0.685	7.61E-28	SMC1
IL7R	4.07E-31	0.62855326	0.809	0.978	8.13E-28	SMC1
SGK1	4.78E-31	0.05099268	0.603	0.824	9.56E-28	SMC1
CFP	6.43E-31	0.01098143	0.189	0.106	1.29E-27	SMC1
GNA15	7.78E-31	0.01336516	0.231	0.224	1.56E-27	SMC1
THY1	8.69E-31	-0.0337071	0.822	0.983	1.74E-27	SMC1
AL355596.1	9.46E-31	0.00036596	0.179	0.148	1.89E-27	SMC1
ADH4	1.22E-30	0.00372672	0.516	0.257	2.44E-27	SMC1
CYTIP	1.63E-30	0.2435517	0.806	0.965	3.25E-27	SMC1
SPI1	1.66E-30	0.01246782	0.449	0.593	3.31E-27	SMC1
CACNA2D3	1.98E-30	0.01066841	0.188	0.287	3.96E-27	SMC1
CCR7	2.61E-30	0.08246981	0.762	0.863	5.22E-27	SMC1
CD2	2.87E-30	0.32920729	0.809	0.993	5.73E-27	SMC1
MTSS1	2.89E-30	-0.0161851	0.579	0.409	5.78E-27	SMC1
FPR2	4.39E-30	0.00050156	0.652	0.399	8.78E-27	SMC1
HHIP	5.24E-30	0.0038282	0.104	0.153	1.05E-26	SMC1
MAD2L1	6.49E-30	-0.0094938	0.615	0.874	1.30E-26	SMC1
GZMB	6.49E-30	0.09756063	0.699	0.883	1.30E-26	SMC1
NPW	7.24E-30	0.00235929	0.05	0.102	1.45E-26	SMC1
CERCAM	8.45E-30	0.01191718	0.359	0.647	1.69E-26	SMC1
IGKV3-20	8.63E-30	0.00302549	0.781	0.51	1.73E-26	SMC1
TRBC1	1.06E-29	0.19078234	0.521	0.77	2.13E-26	SMC1
TDO2	1.28E-29	-0.0030434	0.817	0.899	2.56E-26	SMC1
STXBP2	1.36E-29	0.01959282	0.496	0.587	2.73E-26	SMC1
CHCHD6	1.40E-29	-0.0211876	0.436	0.724	2.80E-26	SMC1
QPCT	2.47E-29	-0.0148682	0.545	0.744	4.94E-26	SMC1
KLRB1	2.65E-29	0.14200883	0.625	0.833	5.30E-26	SMC1
MYOZ2	5.00E-29	0.04558429	0.221	0.288	1.00E-25	SMC1
RNF144B	5.59E-29	0.02348335	0.433	0.362	1.12E-25	SMC1
PLIN2	5.81E-29	0.17878952	0.567	0.855	1.16E-25	SMC1
SERPINA1	9.04E-29	0.00848287	0.53	0.677	1.81E-25	SMC1
PRCP	9.67E-29	-0.0050111	0.435	0.693	1.93E-25	SMC1
PLAUR	1.27E-28	0.05236936	0.715	0.898	2.54E-25	SMC1
MARCO	1.54E-28	0.0032582	0.121	0.267	3.09E-25	SMC1
SPNS2	1.81E-28	-0.0076773	0.432	0.313	3.62E-25	SMC1
CDH2	1.83E-28	0.02357684	0.253	0.367	3.67E-25	SMC1
HMOX1	2.41E-28	0.05823078	0.412	0.663	4.83E-25	SMC1
OBP2A	2.77E-28	-0.0013931	0.79	0.551	5.54E-25	SMC1
CLU	3.74E-28	0.56400578	0.916	0.887	7.48E-25	SMC1
OLFML2B	4.11E-28	0.07042935	0.418	0.736	8.23E-25	SMC1

RASSF4	5.43E-28	-0.0253754	0.522	0.383	1.09E-24	SMC1
PTAFR	8.22E-28	0.00493523	0.398	0.434	1.64E-24	SMC1
JAM2	1.20E-27	0.03154883	0.669	0.457	2.40E-24	SMC1
ANGPTL5	1.46E-27	-0.0099677	0.73	0.483	2.92E-24	SMC1
RRM2	1.53E-27	0.00162548	0.507	0.253	3.06E-24	SMC1
SPOCK1	2.18E-27	0.01557767	0.299	0.349	4.35E-24	SMC1
MECOM	4.19E-27	-0.0160318	0.481	0.773	8.37E-24	SMC1
RBP1	4.46E-27	0.13923313	0.583	0.976	8.92E-24	SMC1
MME	5.38E-27	0.00215561	0.769	0.5	1.08E-23	SMC1
ARHGAP18	7.45E-27	0.04056948	0.743	0.908	1.49E-23	SMC1
HAPLN1	8.45E-27	0.0461221	0.773	0.735	1.69E-23	SMC1
C1QTNF4	1.03E-26	-0.0202541	0.72	0.609	2.06E-23	SMC1
STMN1	1.14E-26	0.19193106	0.324	0.745	2.28E-23	SMC1
GPHA2	1.20E-26	-0.0800854	0.724	0.46	2.39E-23	SMC1
THAP2	1.22E-26	0.03117209	0.556	0.436	2.45E-23	SMC1
ADAP2	1.25E-26	-0.0712849	0.786	0.934	2.50E-23	SMC1
HES5	2.31E-26	-0.075977	0.529	0.373	4.63E-23	SMC1
RBP4	2.79E-26	0.00886032	0.366	0.49	5.59E-23	SMC1
ACKR1	3.09E-26	-0.0199664	0.525	0.39	6.19E-23	SMC1
CD36	3.53E-26	0.02884058	0.643	0.749	7.06E-23	SMC1
MPZL2	3.67E-26	0.00106814	0.067	0.103	7.34E-23	SMC1
CXCL3	3.77E-26	0.05577883	0.136	0.384	7.54E-23	SMC1
KCNJ2	4.14E-26	0.00579741	0.43	0.228	8.29E-23	SMC1
OTOA	4.59E-26	-0.0006238	0.376	0.183	9.19E-23	SMC1
AQP9	4.65E-26	0.00243317	0.144	0.191	9.30E-23	SMC1
LYN	6.24E-26	0.01037101	0.606	0.801	1.25E-22	SMC1
EGFLAM	6.35E-26	-0.0005644	0.682	0.514	1.27E-22	SMC1
CD40LG	7.63E-26	0.04748843	0.725	0.716	1.53E-22	SMC1
HS3ST2	9.18E-26	0.0228703	0.375	0.127	1.84E-22	SMC1
FUCA1	1.13E-25	0.12142971	0.599	0.87	2.26E-22	SMC1
G0S2	1.31E-25	-0.0841836	0.572	0.816	2.61E-22	SMC1
ESM1	1.36E-25	0.00424477	0.702	0.424	2.72E-22	SMC1
KLF4	1.37E-25	-0.1317694	0.658	0.46	2.74E-22	SMC1
KRT7	1.67E-25	0.03054461	0.734	0.579	3.35E-22	SMC1
GPR34	1.99E-25	-0.0020865	0.691	0.688	3.98E-22	SMC1
CD247	2.13E-25	0.07724803	0.546	0.62	4.27E-22	SMC1
IGHV3-20	2.16E-25	0.00132937	0.63	0.451	4.32E-22	SMC1
MYCT1	2.86E-25	0.00497635	0.599	0.36	5.71E-22	SMC1
CD200	3.98E-25	0.05613328	0.734	0.969	7.95E-22	SMC1
LAT2	4.29E-25	0.01283468	0.269	0.392	8.58E-22	SMC1
DSE	5.37E-25	0.05832142	0.409	0.723	1.07E-21	SMC1
CD14	5.51E-25	0.0966988	0.443	0.659	1.10E-21	SMC1
TRAC	7.15E-25	0.25415191	0.812	0.977	1.43E-21	SMC1
DUSP5	9.37E-25	0.04417941	0.809	0.919	1.87E-21	SMC1
CSF2	1.11E-24	0.00103803	0.08	0.164	2.23E-21	SMC1
APOD	1.18E-24	-0.0048767	0.618	0.47	2.37E-21	SMC1
MRC1	1.61E-24	0.00012106	0.647	0.764	3.22E-21	SMC1
LILRB2	2.14E-24	0.00185296	0.431	0.47	4.28E-21	SMC1
C2	2.38E-24	-0.0059848	0.671	0.8	4.77E-21	SMC1
EDN1	2.44E-24	0.03916304	0.389	0.622	4.88E-21	SMC1
SPOCK2	2.80E-24	0.11874663	0.707	0.842	5.61E-21	SMC1
CSF3	5.94E-24	-0.0023448	0.659	0.417	1.19E-20	SMC1
GAP43	6.15E-24	-0.0168876	0.735	0.697	1.23E-20	SMC1

HES6	6.19E-24	-0.0438879	0.442	0.626	1.24E-20	SMC1
CLDN10	6.76E-24	0.00332609	0.323	0.169	1.35E-20	SMC1
ADAMTS4	6.81E-24	-0.2032899	0.571	0.44	1.36E-20	SMC1
SLCO2B1	6.89E-24	0.00859645	0.723	0.419	1.38E-20	SMC1
TACSTD2	9.56E-24	0.01117659	0.371	0.156	1.91E-20	SMC1
IL6	1.02E-23	-0.3566838	0.552	0.443	2.04E-20	SMC1
NKG7	1.20E-23	0.21184463	0.667	0.848	2.39E-20	SMC1
FAM13C	1.65E-23	0.15295635	0.496	0.844	3.31E-20	SMC1
AVPR1A	1.71E-23	-0.1335543	0.404	0.387	3.42E-20	SMC1
ANPEP	2.36E-23	-0.0004623	0.784	0.836	4.72E-20	SMC1
ABI3BP	2.52E-23	0.11070262	0.261	0.416	5.05E-20	SMC1
DRD4	2.75E-23	0.00047649	0.576	0.319	5.50E-20	SMC1
KIFC1	4.11E-23	0.00137818	0.708	0.457	8.23E-20	SMC1
GSTM5	4.25E-23	-0.0049642	0.498	0.336	8.50E-20	SMC1
DIO2	4.27E-23	0.00967976	0.585	0.765	8.54E-20	SMC1
TREML1	4.80E-23	0.00144162	0.736	0.537	9.59E-20	SMC1
BACE2	4.98E-23	0.16547856	0.746	0.94	9.96E-20	SMC1
GPC3	6.04E-23	-0.0239707	0.628	0.451	1.21E-19	SMC1
LRRN4CL	9.08E-23	0.0036598	0.679	0.519	1.82E-19	SMC1
IGF2.1	1.01E-22	-0.0131453	0.428	0.212	2.02E-19	SMC1
FLT1	1.01E-22	-0.0035103	0.246	0.217	2.02E-19	SMC1
TGFBI	1.10E-22	-0.1857549	0.616	0.824	2.20E-19	SMC1
RTKN2	1.80E-22	0.00101537	0.669	0.398	3.61E-19	SMC1
EZR	1.85E-22	0.23276741	0.503	0.809	3.69E-19	SMC1
CENPE	2.20E-22	0.00737655	0.412	0.547	4.40E-19	SMC1
CCL26	2.49E-22	0.00406706	0.8	0.916	4.99E-19	SMC1
LIFR	2.53E-22	0.04652698	0.257	0.491	5.06E-19	SMC1
PFKFB3	2.98E-22	0.06033111	0.489	0.758	5.97E-19	SMC1
PRPH	3.11E-22	-0.2671339	0.432	0.355	6.22E-19	SMC1
TYMP	3.14E-22	-0.1314848	0.851	0.98	6.29E-19	SMC1
MFAP5	3.19E-22	0.01066289	0.522	0.23	6.39E-19	SMC1
CHI3L1	3.52E-22	0.01255231	0.8	0.874	7.04E-19	SMC1
SAMSN1	5.11E-22	0.1765791	0.711	0.876	1.02E-18	SMC1
CXCL13	5.16E-22	0.01882472	0.342	0.097	1.03E-18	SMC1
COMP	5.33E-22	-0.0037332	0.299	0.356	1.07E-18	SMC1
SMAD6	5.67E-22	-0.0133085	0.492	0.353	1.13E-18	SMC1
JUN	6.01E-22	-0.4869464	0.961	0.98	1.20E-18	SMC1
PLEK	1.05E-21	0.03778373	0.472	0.499	2.11E-18	SMC1
KLRF1	1.51E-21	0.00180444	0.788	0.51	3.02E-18	SMC1
SELE	1.54E-21	0.00023491	0.696	0.495	3.08E-18	SMC1
TNFSF11	1.65E-21	-0.0007545	0.691	0.455	3.30E-18	SMC1
SCARA5	1.67E-21	-0.0173865	0.525	0.25	3.34E-18	SMC1
CLEC12A	1.88E-21	0.00663195	0.113	0.148	3.75E-18	SMC1
C2CD4B	1.89E-21	-0.0188322	0.435	0.279	3.79E-18	SMC1
USP2	2.01E-21	-0.1022698	0.214	0.222	4.02E-18	SMC1
DUSP2	2.22E-21	0.43337024	0.775	0.929	4.44E-18	SMC1
CDH19	2.30E-21	-0.0096623	0.48	0.27	4.59E-18	SMC1
CD84	2.35E-21	0.0163385	0.374	0.398	4.70E-18	SMC1
SUGCT	2.66E-21	0.27725969	0.648	0.988	5.32E-18	SMC1
C17orf58	2.81E-21	0.04808932	0.661	0.593	5.62E-18	SMC1
PLA2G2A	2.84E-21	-0.0182435	0.627	0.403	5.68E-18	SMC1
CEMIP	3.21E-21	0.01150133	0.773	0.515	6.42E-18	SMC1
SCG2	3.30E-21	0.02802298	0.724	0.429	6.60E-18	SMC1

NDUFAF6	3.44E-21	0.00885788	0.302	0.563	6.89E-18	SMC1
LYVE1	3.64E-21	0.01082452	0.625	0.402	7.28E-18	SMC1
COL1A1	4.41E-21	-0.0107941	0.556	0.772	8.82E-18	SMC1
ENC1	4.42E-21	0.0065923	0.757	0.734	8.83E-18	SMC1
CCDC144A	4.97E-21	0.02408062	0.612	0.54	9.95E-18	SMC1
ATF3	5.16E-21	-0.3512182	0.886	0.903	1.03E-17	SMC1
RAMP2	5.27E-21	0.04502627	0.666	0.583	1.05E-17	SMC1
LINC02362	6.61E-21	0.00150149	0.763	0.5	1.32E-17	SMC1
ITGB2	6.66E-21	0.12595544	0.785	0.974	1.33E-17	SMC1
PCK1	6.87E-21	0.02249511	0.765	0.577	1.37E-17	SMC1
ALPL	7.07E-21	-0.0028365	0.131	0.123	1.41E-17	SMC1
C3	7.41E-21	-0.0024588	0.586	0.374	1.48E-17	SMC1
GZMH	7.61E-21	0.13655675	0.656	0.77	1.52E-17	SMC1
AGT	1.01E-20	-0.2243473	0.834	0.873	2.02E-17	SMC1
ACOXL	1.02E-20	0.0015437	0.668	0.379	2.03E-17	SMC1
HSD3B7	1.04E-20	0.02098858	0.474	0.707	2.09E-17	SMC1
MND1	1.05E-20	0.00686819	0.567	0.44	2.10E-17	SMC1
MAFB	1.06E-20	0.08820115	0.454	0.749	2.12E-17	SMC1
MIR155HG	1.21E-20	0.00550638	0.391	0.564	2.43E-17	SMC1
DLX6	1.22E-20	-0.0773154	0.576	0.481	2.44E-17	SMC1
GJA1	1.23E-20	0.13411815	0.34	0.574	2.47E-17	SMC1
OLR1	2.06E-20	0.00457281	0.623	0.576	4.11E-17	SMC1
NR4A3	2.12E-20	-0.0887245	0.437	0.364	4.24E-17	SMC1
GAPT	2.45E-20	0.00588241	0.488	0.503	4.89E-17	SMC1
FCN1	2.53E-20	0.02720662	0.538	0.724	5.06E-17	SMC1
DERL3	3.59E-20	0.00197927	0.577	0.386	7.18E-17	SMC1
IFI30	4.07E-20	0.00461084	0.559	0.667	8.13E-17	SMC1
MXRA5	4.34E-20	-0.0422202	0.735	0.508	8.68E-17	SMC1
HPGDS	5.92E-20	0.00708772	0.713	0.517	1.18E-16	SMC1
TBXAS1	5.93E-20	-0.0033067	0.573	0.679	1.19E-16	SMC1
CD79A	6.41E-20	-0.0213044	0.774	0.769	1.28E-16	SMC1
HBB	6.80E-20	0.00232842	0.145	0.162	1.36E-16	SMC1
EMP1	7.04E-20	-0.0594604	0.701	0.783	1.41E-16	SMC1
GPM6B	7.29E-20	0.01882645	0.785	0.856	1.46E-16	SMC1
HP	8.36E-20	0.00423999	0.469	0.281	1.67E-16	SMC1
RBM47	9.10E-20	0.01317947	0.096	0.173	1.82E-16	SMC1
CD52	1.10E-19	0.44397274	0.806	0.943	2.20E-16	SMC1
HEG1	1.12E-19	0.03370619	0.505	0.747	2.24E-16	SMC1
NQO1	1.20E-19	0.22804474	0.654	0.928	2.40E-16	SMC1
MYH10	1.46E-19	0.48154775	0.803	0.877	2.92E-16	SMC1
AC104407.1	1.47E-19	-0.0077686	0.084	0.14	2.94E-16	SMC1
BASP1	1.59E-19	0.26507984	0.503	0.876	3.19E-16	SMC1
HIST1H1A	1.66E-19	0.04883984	0.6	0.522	3.32E-16	SMC1
CES1	3.34E-19	0.14751187	0.336	0.599	6.68E-16	SMC1
ARC	3.51E-19	-0.0405357	0.657	0.57	7.02E-16	SMC1
MSX1	3.93E-19	-0.0102385	0.399	0.624	7.87E-16	SMC1
SFRP1	4.22E-19	0.04981147	0.272	0.286	8.43E-16	SMC1
PLK2	4.90E-19	-0.0748983	0.575	0.799	9.80E-16	SMC1
P2RY14	5.21E-19	0.01242246	0.734	0.573	1.04E-15	SMC1
RGS1	5.39E-19	0.19878125	0.711	0.907	1.08E-15	SMC1
C1QTNF7	6.56E-19	0.0790018	0.741	0.574	1.31E-15	SMC1
ESAM	8.59E-19	-0.2464223	0.62	0.785	1.72E-15	SMC1
CCL4	9.27E-19	0.72287216	0.843	0.994	1.85E-15	SMC1

GZF1	1.22E-18	0.00276502	0.365	0.349	2.44E-15	SMC1
GRN	1.52E-18	0.0482851	0.646	0.872	3.04E-15	SMC1
KCNN4	1.66E-18	0.00597491	0.574	0.556	3.32E-15	SMC1
IL1B	2.38E-18	0.07627234	0.392	0.549	4.76E-15	SMC1
TCF21	2.75E-18	0.00988037	0.434	0.322	5.49E-15	SMC1
SLC26A4	2.76E-18	0.02220288	0.564	0.384	5.52E-15	SMC1
ACAN	2.80E-18	-0.1078623	0.457	0.377	5.61E-15	SMC1
ITGB4	3.15E-18	-0.0044628	0.763	0.534	6.29E-15	SMC1
AC233755.1	3.25E-18	0.00155638	0.591	0.414	6.50E-15	SMC1
CDCA5	3.31E-18	0.00415761	0.409	0.399	6.63E-15	SMC1
SLAMF1	3.50E-18	0.02727472	0.786	0.795	7.00E-15	SMC1
PAPPA	3.84E-18	0.02752629	0.489	0.673	7.68E-15	SMC1
ANXA3	4.96E-18	0.00617402	0.684	0.529	9.91E-15	SMC1
ANKRD1	5.00E-18	0.00228113	0.577	0.33	1.00E-14	SMC1
GADD45B	5.30E-18	-0.4342696	0.925	0.931	1.06E-14	SMC1
CD3D	5.33E-18	0.24769292	0.733	0.897	1.07E-14	SMC1
SELL	6.14E-18	0.03286449	0.315	0.398	1.23E-14	SMC1
TESC	6.21E-18	-0.033397	0.761	0.584	1.24E-14	SMC1
THSD7A	6.96E-18	0.00555442	0.779	0.567	1.39E-14	SMC1
PDCD1	8.98E-18	0.02680617	0.694	0.456	1.80E-14	SMC1
LST1	9.04E-18	0.02006766	0.591	0.706	1.81E-14	SMC1
JAML	9.46E-18	0.024139	0.293	0.33	1.89E-14	SMC1
FAM180B	1.16E-17	0.00064899	0.478	0.303	2.32E-14	SMC1
HLA-DQB1	1.19E-17	0.05135187	0.606	0.898	2.39E-14	SMC1
LRRC10B	1.25E-17	-0.1026252	0.519	0.427	2.50E-14	SMC1
TUBB2B	1.26E-17	-0.0074013	0.322	0.241	2.51E-14	SMC1
IFIT3	1.26E-17	-0.0152536	0.622	0.833	2.52E-14	SMC1
SFRP5	1.26E-17	0.00331741	0.123	0.17	2.53E-14	SMC1
IGFBP2	1.54E-17	0.54759682	0.922	0.998	3.08E-14	SMC1
SPP1	1.63E-17	0.10875421	0.683	0.941	3.25E-14	SMC1
CHEK1	1.89E-17	0.00905621	0.438	0.587	3.79E-14	SMC1
CSF1R	2.30E-17	0.00802477	0.514	0.513	4.60E-14	SMC1
COL18A1	2.60E-17	-0.2982454	0.692	0.865	5.20E-14	SMC1
IL33	2.73E-17	0.05602476	0.466	0.76	5.47E-14	SMC1
ADRA2A	2.77E-17	-0.1465117	0.583	0.477	5.53E-14	SMC1
LILRA4	3.29E-17	0.00512882	0.745	0.509	6.58E-14	SMC1
TCF15	3.45E-17	-0.0060663	0.615	0.417	6.90E-14	SMC1
MFSD1	4.18E-17	0.0217836	0.359	0.597	8.35E-14	SMC1
SEPT4	4.50E-17	-0.332424	0.887	0.985	8.99E-14	SMC1
EGR1	6.28E-17	-0.1424654	0.932	0.957	1.26E-13	SMC1
JCHAIN	6.48E-17	0.037507	0.827	0.997	1.30E-13	SMC1
MEG3	6.58E-17	0.03017331	0.297	0.381	1.32E-13	SMC1
NPR3	6.78E-17	-0.011642	0.639	0.556	1.36E-13	SMC1
RARRES1	7.29E-17	0.05505298	0.663	0.687	1.46E-13	SMC1
RASSF9	8.63E-17	0.04920596	0.175	0.364	1.73E-13	SMC1
USP53	1.02E-16	0.0351491	0.415	0.719	2.03E-13	SMC1
FBLN1	1.30E-16	0.09173959	0.329	0.59	2.60E-13	SMC1
LMO7	1.52E-16	0.2360684	0.485	0.903	3.03E-13	SMC1
CAVIN2	1.96E-16	0.00166714	0.573	0.474	3.92E-13	SMC1
MCAM	2.01E-16	-0.3322445	0.729	0.856	4.02E-13	SMC1
IGHG3	2.17E-16	0.0474955	0.822	0.952	4.35E-13	SMC1
CCNB2	2.29E-16	0.00136493	0.066	0.162	4.57E-13	SMC1
CXCR4	2.52E-16	0.60745703	0.805	0.943	5.04E-13	SMC1

RCN3	3.15E-16	0.07252415	0.68	0.859	6.29E-13	SMC1
HRCT1	3.71E-16	0.20712757	0.344	0.645	7.43E-13	SMC1
P2RY13	3.71E-16	0.00512022	0.552	0.319	7.43E-13	SMC1
SIGLEC15	4.30E-16	0.0050287	0.741	0.505	8.60E-13	SMC1
COL4A1	5.18E-16	-0.0737409	0.64	0.908	1.04E-12	SMC1
FPR1	6.11E-16	0.00880622	0.337	0.367	1.22E-12	SMC1
ST8SIA6	6.16E-16	0.00231377	0.165	0.174	1.23E-12	SMC1
HMGA1	6.79E-16	0.02906336	0.608	0.748	1.36E-12	SMC1
SDC2	6.93E-16	0.00456233	0.625	0.895	1.39E-12	SMC1
ITGA8	6.96E-16	0.35364083	0.855	0.872	1.39E-12	SMC1
FHL5	7.27E-16	-0.1441795	0.536	0.762	1.45E-12	SMC1
GADD45G	8.82E-16	0.06246249	0.353	0.628	1.76E-12	SMC1
ABCC9	8.85E-16	-0.0602544	0.421	0.648	1.77E-12	SMC1
APLNR	9.18E-16	-0.0095472	0.721	0.649	1.84E-12	SMC1
BATF	9.89E-16	0.0681802	0.742	0.805	1.98E-12	SMC1
RASGEF1B	1.05E-15	-0.0922101	0.666	0.552	2.11E-12	SMC1
PTCRA	1.18E-15	0.0050105	0.786	0.588	2.36E-12	SMC1
ARHGAP29	1.20E-15	-0.179648	0.766	0.923	2.41E-12	SMC1
C11orf96	1.37E-15	-0.3347348	0.953	0.99	2.75E-12	SMC1
ADIRF	1.63E-15	-0.1751154	0.985	0.995	3.27E-12	SMC1
HMGB3	2.33E-15	0.02646619	0.563	0.473	4.66E-12	SMC1
AC023157.3	2.89E-15	0.0326569	0.674	0.83	5.78E-12	SMC1
ALOX5	3.20E-15	0.01184562	0.104	0.207	6.40E-12	SMC1
CD33	3.44E-15	0.00462705	0.128	0.181	6.87E-12	SMC1
SLCO2A1	3.60E-15	0.00256401	0.607	0.426	7.20E-12	SMC1
CCDC144NL	3.75E-15	0.05813915	0.258	0.397	7.50E-12	SMC1
TNFSF15	4.01E-15	-0.0077481	0.702	0.631	8.01E-12	SMC1
TSKU	4.19E-15	-0.0082783	0.376	0.581	8.37E-12	SMC1
GPC6	4.48E-15	0.14461041	0.387	0.607	8.96E-12	SMC1
HTRA3	4.56E-15	-0.0068519	0.642	0.427	9.11E-12	SMC1
SNX10	4.66E-15	0.01363095	0.541	0.349	9.32E-12	SMC1
SMC4	4.76E-15	0.00514289	0.505	0.707	9.52E-12	SMC1
CCNA2	4.91E-15	0.00224443	0.534	0.329	9.83E-12	SMC1
UGDH	5.05E-15	0.07910508	0.573	0.513	1.01E-11	SMC1
MNDA	5.13E-15	0.03660402	0.38	0.497	1.03E-11	SMC1
NPPC	5.19E-15	-0.0058695	0.664	0.478	1.04E-11	SMC1
ANK2	5.45E-15	-0.001368	0.565	0.521	1.09E-11	SMC1
GFRA1	5.83E-15	0.00880379	0.647	0.513	1.17E-11	SMC1
MRC2	6.86E-15	0.08356885	0.548	0.812	1.37E-11	SMC1
PTP4A3	7.05E-15	-0.2035331	0.718	0.793	1.41E-11	SMC1
TSPAN8	7.77E-15	0.03011945	0.315	0.317	1.55E-11	SMC1
MFAP2	8.47E-15	0.14501391	0.482	0.81	1.69E-11	SMC1
RSPO3	8.75E-15	0.00489825	0.63	0.516	1.75E-11	SMC1
MFAP4	8.94E-15	0.3205344	0.844	0.836	1.79E-11	SMC1
MMRN1	9.32E-15	0.0089335	0.451	0.215	1.86E-11	SMC1
SSTR2	9.64E-15	-0.0219057	0.66	0.505	1.93E-11	SMC1
CALHM6	1.11E-14	0.01163986	0.365	0.398	2.21E-11	SMC1
BUB1	1.16E-14	0.00533244	0.801	0.648	2.33E-11	SMC1
FGFBP2	1.67E-14	0.02781664	0.244	0.21	3.35E-11	SMC1
NRXN1	1.68E-14	0.00556755	0.389	0.193	3.36E-11	SMC1
EPB41L3	1.85E-14	0.0065673	0.22	0.219	3.70E-11	SMC1
NPL	1.86E-14	0.0021093	0.702	0.848	3.73E-11	SMC1
RAB20	1.92E-14	-0.0350768	0.625	0.488	3.84E-11	SMC1

FSTL1	1.95E-14	0.17985202	0.507	0.865	3.89E-11	SMC1
MZB1	1.98E-14	0.02566584	0.809	0.952	3.96E-11	SMC1
CD7	2.43E-14	0.09883397	0.802	0.852	4.86E-11	SMC1
NPY1R	2.57E-14	-0.0879978	0.413	0.386	5.14E-11	SMC1
RASSF6	2.66E-14	-0.0052675	0.617	0.599	5.32E-11	SMC1
KCTD12	2.82E-14	0.03378713	0.563	0.82	5.64E-11	SMC1
AKR1C2	2.93E-14	0.08650613	0.413	0.71	5.86E-11	SMC1
SOCS1	3.26E-14	0.02922317	0.762	0.924	6.52E-11	SMC1
SCX	3.30E-14	0.09648662	0.322	0.55	6.60E-11	SMC1
LAIR2	3.36E-14	0.01454148	0.308	0.206	6.71E-11	SMC1
PAK1	3.54E-14	0.06921468	0.45	0.413	7.07E-11	SMC1
FAM241A	4.13E-14	-0.0701726	0.683	0.553	8.26E-11	SMC1
MATN4	4.18E-14	-0.0024108	0.208	0.141	8.36E-11	SMC1
S100A10	4.37E-14	0.44798386	0.899	0.984	8.74E-11	SMC1
GK	4.61E-14	0.04018027	0.507	0.702	9.22E-11	SMC1
IL2RA	5.15E-14	0.0172311	0.419	0.297	1.03E-10	SMC1
SPARCL1	5.49E-14	-0.487249	0.984	0.995	1.10E-10	SMC1
IGHV1-18	5.71E-14	0.00480815	0.452	0.323	1.14E-10	SMC1
TENT5C	5.74E-14	0.08777535	0.4	0.635	1.15E-10	SMC1
MX1	6.16E-14	0.11308727	0.728	0.912	1.23E-10	SMC1
RAB42	7.88E-14	-0.0027916	0.533	0.63	1.58E-10	SMC1
CADM3	8.79E-14	-0.0010652	0.726	0.526	1.76E-10	SMC1
PI15	1.14E-13	0.00258493	0.654	0.706	2.27E-10	SMC1
CTSS	1.32E-13	0.10653363	0.567	0.82	2.64E-10	SMC1
CP	1.40E-13	0.48073496	0.844	0.945	2.80E-10	SMC1
FAM69B	1.42E-13	-0.000885	0.786	0.595	2.84E-10	SMC1
F2R	1.43E-13	0.01292314	0.741	0.907	2.86E-10	SMC1
CD300E	1.53E-13	0.00973558	0.344	0.369	3.07E-10	SMC1
APOLD1	1.66E-13	-0.2170362	0.66	0.535	3.31E-10	SMC1
NTRK2	1.68E-13	-0.2757621	0.81	0.805	3.36E-10	SMC1
DIRC3	1.71E-13	0.00467878	0.567	0.348	3.41E-10	SMC1
CCL5	1.80E-13	0.56051643	0.829	0.994	3.59E-10	SMC1
PLAC9	1.95E-13	-0.1643222	0.935	0.972	3.91E-10	SMC1
HDC	2.06E-13	0.00964924	0.088	0.177	4.11E-10	SMC1
TMEM88	2.09E-13	0.00080399	0.316	0.207	4.18E-10	SMC1
SIRPB1	2.21E-13	0.00264688	0.77	0.541	4.43E-10	SMC1
INHBA	2.31E-13	-0.024212	0.497	0.728	4.63E-10	SMC1
KRT86	2.36E-13	-0.0022794	0.177	0.184	4.73E-10	SMC1
WFDC2	2.57E-13	-0.142064	0.619	0.543	5.15E-10	SMC1
OXTR	2.59E-13	-0.0106191	0.285	0.252	5.19E-10	SMC1
OAS1	3.05E-13	0.03235904	0.648	0.828	6.10E-10	SMC1
KRT8	3.32E-13	0.07889046	0.762	0.837	6.63E-10	SMC1
CBLN4	3.34E-13	-0.0067105	0.326	0.187	6.68E-10	SMC1
SHISA3	3.54E-13	-0.0041017	0.176	0.19	7.08E-10	SMC1
ISLR	3.83E-13	0.08702368	0.792	0.687	7.65E-10	SMC1
RGS7BP	4.86E-13	0.04461827	0.244	0.51	9.73E-10	SMC1
TMEM204	5.30E-13	0.03103663	0.735	0.869	1.06E-09	SMC1
LILRB4	5.60E-13	0.00479061	0.401	0.369	1.12E-09	SMC1
VMO1	5.64E-13	0.00782463	0.746	0.826	1.13E-09	SMC1
HES4	5.91E-13	-0.3599882	0.845	0.793	1.18E-09	SMC1
MARCKS	5.92E-13	0.17049676	0.883	0.955	1.18E-09	SMC1
CDH13	5.97E-13	0.1726625	0.455	0.81	1.19E-09	SMC1
SDS	6.55E-13	0.02873314	0.623	0.353	1.31E-09	SMC1

GAS1	7.64E-13	0.04465388	0.572	0.548	1.53E-09	SMC1
FSCN1	7.82E-13	-0.0144454	0.7	0.791	1.56E-09	SMC1
PCNA	7.99E-13	0.09247932	0.565	0.771	1.60E-09	SMC1
CCDC88A	8.05E-13	0.15066006	0.437	0.705	1.61E-09	SMC1
SLC39A8	8.44E-13	0.00384677	0.36	0.592	1.69E-09	SMC1
NRIP3	9.06E-13	0.01163489	0.437	0.581	1.81E-09	SMC1
ICAM1	9.51E-13	-0.0084233	0.681	0.644	1.90E-09	SMC1
TBX2-AS1	1.05E-12	-0.2842418	0.729	0.781	2.09E-09	SMC1
ABCA8	1.13E-12	0.05797269	0.683	0.763	2.26E-09	SMC1
IL1R2	1.16E-12	-0.0021063	0.443	0.22	2.32E-09	SMC1
SERPINH1	1.31E-12	-0.0559354	0.808	0.908	2.62E-09	SMC1
RERGL	1.54E-12	-0.1678089	0.814	0.855	3.08E-09	SMC1
NTM	1.58E-12	0.00127694	0.356	0.223	3.15E-09	SMC1
HSPH1	1.67E-12	0.07525203	0.586	0.794	3.34E-09	SMC1
RHEX	1.68E-12	0.00583316	0.086	0.178	3.36E-09	SMC1
ADAMTS1	1.84E-12	-0.0850339	0.756	0.884	3.67E-09	SMC1
SCN7A	1.89E-12	-0.0007518	0.082	0.135	3.78E-09	SMC1
PLTP	1.90E-12	-0.0713623	0.687	0.844	3.80E-09	SMC1
BTG2	1.95E-12	-0.1123435	0.784	0.86	3.91E-09	SMC1
CENPK	2.19E-12	0.01446476	0.66	0.527	4.38E-09	SMC1
CLIC3	2.30E-12	0.04181512	0.645	0.614	4.59E-09	SMC1
AQP3	2.53E-12	0.01308431	0.763	0.662	5.06E-09	SMC1
BHLHE41	2.68E-12	0.1010619	0.243	0.428	5.36E-09	SMC1
BEX2	3.46E-12	0.04128058	0.308	0.477	6.93E-09	SMC1
IGLV1-51	3.70E-12	-0.0004233	0.274	0.1	7.40E-09	SMC1
UBE2T	4.27E-12	-0.0007701	0.73	0.719	8.55E-09	SMC1
CDK1	4.33E-12	0.00264071	0.372	0.341	8.66E-09	SMC1
AC007952.4	4.59E-12	-0.0391932	0.668	0.593	9.19E-09	SMC1
GZMK	4.68E-12	0.39772378	0.805	0.902	9.36E-09	SMC1
PHGDH	4.78E-12	0.22146686	0.423	0.716	9.55E-09	SMC1
TRBC2	5.12E-12	0.35850121	0.807	0.962	1.02E-08	SMC1
SCIMP	5.20E-12	-0.0039262	0.72	0.695	1.04E-08	SMC1
CYP4B1	5.45E-12	-0.0018959	0.649	0.458	1.09E-08	SMC1
NCAPH	6.43E-12	-0.007511	0.594	0.673	1.29E-08	SMC1
MCM7	6.84E-12	0.00514224	0.488	0.708	1.37E-08	SMC1
TWISTNB	7.09E-12	0.03194311	0.571	0.751	1.42E-08	SMC1
RIPK2	7.10E-12	0.00342826	0.65	0.828	1.42E-08	SMC1
CTSB	8.03E-12	0.10301625	0.555	0.826	1.61E-08	SMC1
SLAMF8	8.60E-12	0.00274898	0.472	0.464	1.72E-08	SMC1
IGLV1-47	9.04E-12	-2.14E-05	0.044	0.113	1.81E-08	SMC1
VWF	9.64E-12	0.00687248	0.477	0.537	1.93E-08	SMC1
LRRC17	9.99E-12	0.24170163	0.523	0.83	2.00E-08	SMC1
PAPPA2	1.01E-11	-0.0027856	0.431	0.22	2.02E-08	SMC1
MRAS	1.03E-11	0.06130817	0.734	0.688	2.05E-08	SMC1
WNT5A	1.15E-11	-0.0031485	0.743	0.54	2.30E-08	SMC1
CHRDL1	1.23E-11	-0.0070592	0.315	0.203	2.45E-08	SMC1
DNAAF1	1.31E-11	0.00264185	0.249	0.293	2.63E-08	SMC1
OSM	1.33E-11	0.00234365	0.647	0.573	2.65E-08	SMC1
PAEP	1.49E-11	0.00148566	0.786	0.592	2.99E-08	SMC1
CD8A	1.73E-11	0.05377839	0.731	0.807	3.47E-08	SMC1
AL138899.1	1.81E-11	-0.0035747	0.608	0.427	3.63E-08	SMC1
TFEC	1.84E-11	0.007442	0.236	0.221	3.68E-08	SMC1
PCDHB4	1.88E-11	-0.018885	0.729	0.641	3.77E-08	SMC1

CD160	1.89E-11	0.0018926	0.133	0.19	3.78E-08	SMC1
NCF1	1.90E-11	0.03646039	0.747	0.863	3.80E-08	SMC1
IGLC7	2.12E-11	0.00056653	0.584	0.387	4.24E-08	SMC1
MCM10	2.43E-11	0.00222237	0.649	0.445	4.87E-08	SMC1
TFPI2	2.52E-11	0.21916245	0.367	0.56	5.03E-08	SMC1
TSPAN2	2.70E-11	0.20302055	0.444	0.741	5.39E-08	SMC1
TSPAN7	3.07E-11	0.04307198	0.405	0.621	6.15E-08	SMC1
NUSAP1	3.15E-11	0.0082364	0.596	0.365	6.31E-08	SMC1
PALMD	3.20E-11	-0.028097	0.683	0.574	6.40E-08	SMC1
ANKH	3.21E-11	0.14493401	0.44	0.683	6.42E-08	SMC1
GPRC5A	3.86E-11	0.0746871	0.43	0.358	7.72E-08	SMC1
TMEM37	4.27E-11	-0.0176582	0.587	0.695	8.54E-08	SMC1
RGS4	4.38E-11	0.11378401	0.571	0.798	8.76E-08	SMC1
AKR1C3	5.05E-11	0.25534681	0.76	0.971	1.01E-07	SMC1
IFI6	5.91E-11	0.26902095	0.621	0.921	1.18E-07	SMC1
ORC6	6.56E-11	-0.0059225	0.219	0.323	1.31E-07	SMC1
ATAD2	6.61E-11	0.08396402	0.502	0.664	1.32E-07	SMC1
GDF6	6.65E-11	0.00120678	0.229	0.115	1.33E-07	SMC1
ITGA6	6.70E-11	0.04762782	0.303	0.292	1.34E-07	SMC1
MEDAG	6.85E-11	0.04988117	0.572	0.793	1.37E-07	SMC1
SERPINF1	6.89E-11	-0.4296991	0.825	0.876	1.38E-07	SMC1
CCL18	7.65E-11	0.0223065	0.545	0.319	1.53E-07	SMC1
CDKN1A	8.08E-11	-0.2276788	0.799	0.807	1.62E-07	SMC1
HLA-DMA	8.88E-11	0.03648559	0.676	0.879	1.78E-07	SMC1
VCAN	9.49E-11	0.45017216	0.934	0.995	1.90E-07	SMC1
NDUFA4L2	9.78E-11	-0.7657052	0.81	0.79	1.96E-07	SMC1
CCL13	9.88E-11	-0.0015964	0.772	0.544	1.98E-07	SMC1
SPRY1	1.05E-10	0.0134675	0.808	0.95	2.09E-07	SMC1
FKBP10	1.08E-10	0.06274422	0.559	0.751	2.15E-07	SMC1
RDH10	1.10E-10	0.0244642	0.655	0.597	2.20E-07	SMC1
FCGR2A	1.17E-10	-0.043485	0.287	0.497	2.34E-07	SMC1
PDLIM1	1.17E-10	-0.1356297	0.823	0.942	2.35E-07	SMC1
TKTL1	1.24E-10	-0.0026727	0.505	0.415	2.49E-07	SMC1
BIRC5	1.25E-10	0.00277874	0.116	0.171	2.49E-07	SMC1
CSRP2	1.31E-10	-0.2161581	0.8	0.929	2.62E-07	SMC1
PCOLCE	1.33E-10	-0.0793717	0.904	0.994	2.67E-07	SMC1
ANGPT2	1.59E-10	-0.0135005	0.796	0.929	3.17E-07	SMC1
UBD	1.61E-10	-0.0001258	0.116	0.081	3.21E-07	SMC1
SELP	1.64E-10	0.00592485	0.454	0.288	3.29E-07	SMC1
IL1RL1	1.75E-10	0.01161157	0.312	0.305	3.50E-07	SMC1
MT1F	1.77E-10	-0.0117045	0.774	0.806	3.54E-07	SMC1
GFPT2	1.83E-10	0.00842579	0.449	0.343	3.67E-07	SMC1
SPIB	1.88E-10	0.01112746	0.724	0.495	3.76E-07	SMC1
SDC1	1.95E-10	0.00328745	0.084	0.185	3.89E-07	SMC1
SAP30	2.30E-10	0.04713656	0.514	0.736	4.61E-07	SMC1
CST6	2.35E-10	0.18932229	0.649	0.686	4.70E-07	SMC1
FJX1	2.40E-10	-0.021912	0.678	0.607	4.79E-07	SMC1
IGHV1-69D	2.52E-10	-0.0004127	0.052	0.115	5.04E-07	SMC1
SLC2A5	2.61E-10	0.00255315	0.275	0.184	5.22E-07	SMC1
TMEFF2	2.67E-10	-0.0044361	0.763	0.621	5.34E-07	SMC1
ADCYAP1	2.85E-10	0.00420056	0.533	0.374	5.70E-07	SMC1
C19orf38	2.88E-10	0.00709241	0.787	0.642	5.76E-07	SMC1
FAM162B	2.89E-10	-0.10591	0.658	0.535	5.77E-07	SMC1

CD38	2.94E-10	-0.0002404	0.722	0.685	5.89E-07	SMC1
SAA2	3.25E-10	0.00130145	0.492	0.321	6.49E-07	SMC1
CA2	3.25E-10	-0.0009173	0.353	0.422	6.50E-07	SMC1
BCAM	3.32E-10	-0.2776821	0.755	0.731	6.65E-07	SMC1
NOTCH3	3.34E-10	-0.2037487	0.78	0.798	6.68E-07	SMC1
ITM2A	3.54E-10	0.16418682	0.658	0.927	7.08E-07	SMC1
IL18	3.64E-10	0.01057802	0.453	0.431	7.28E-07	SMC1
LXN	3.96E-10	0.03328762	0.743	0.891	7.93E-07	SMC1
KRT19	4.13E-10	0.00202352	0.701	0.67	8.26E-07	SMC1
TNXB	4.66E-10	0.04068747	0.352	0.456	9.33E-07	SMC1
GALNT15	4.80E-10	0.02980378	0.423	0.201	9.59E-07	SMC1
TRGC2	4.88E-10	0.01990336	0.782	0.691	9.77E-07	SMC1
SVIL	5.00E-10	0.2495137	0.811	0.785	1.00E-06	SMC1
DUSP6	5.04E-10	0.07999907	0.725	0.884	1.01E-06	SMC1
FTL	5.12E-10	0.20870386	0.999	1	1.02E-06	SMC1
GGH	5.41E-10	0.02560635	0.411	0.629	1.08E-06	SMC1
NRGN	5.69E-10	0.03614344	0.83	0.897	1.14E-06	SMC1
NCKAP5	7.34E-10	-0.0249004	0.745	0.816	1.47E-06	SMC1
PCLAF	7.98E-10	0.00750526	0.67	0.451	1.60E-06	SMC1
CLEC3B	8.87E-10	-0.0127184	0.746	0.584	1.77E-06	SMC1
CD68	9.27E-10	0.04122781	0.592	0.762	1.85E-06	SMC1
C5AR2	9.28E-10	-0.0015984	0.703	0.524	1.86E-06	SMC1
HPGD	1.08E-09	0.01554068	0.717	0.684	2.16E-06	SMC1
CCL17	1.23E-09	0.00142278	0.726	0.559	2.46E-06	SMC1
LINC02544	1.53E-09	-0.0204175	0.756	0.705	3.06E-06	SMC1
CEBPD	1.53E-09	-0.3360534	0.867	0.917	3.07E-06	SMC1
RAMP1	1.59E-09	0.32662589	0.805	0.845	3.18E-06	SMC1
NLRP3	1.64E-09	0.0056528	0.732	0.549	3.27E-06	SMC1
LGALS9	1.64E-09	0.01547386	0.622	0.499	3.28E-06	SMC1
ROBO4	1.65E-09	0.00255932	0.526	0.547	3.30E-06	SMC1
MAP3K7CL	1.66E-09	-0.4275225	0.706	0.755	3.32E-06	SMC1
CAMK2N1	1.69E-09	0.00282934	0.721	0.601	3.38E-06	SMC1
NAF1	1.92E-09	-0.0054209	0.357	0.372	3.84E-06	SMC1
MMP9	1.93E-09	0.00990279	0.148	0.238	3.86E-06	SMC1
ADAMTS5	1.94E-09	-0.0068453	0.563	0.508	3.88E-06	SMC1
F10	1.99E-09	-0.1691555	0.602	0.513	3.98E-06	SMC1
TNFSF10	2.02E-09	0.08230719	0.831	0.995	4.04E-06	SMC1
PLVAP	2.14E-09	0.00826979	0.58	0.653	4.28E-06	SMC1
HCAR3	2.17E-09	-0.0002918	0.068	0.117	4.34E-06	SMC1
OR51E1	2.36E-09	-0.1583942	0.737	0.792	4.71E-06	SMC1
THBS4	2.46E-09	-0.002448	0.394	0.329	4.92E-06	SMC1
NES	2.48E-09	-0.0583041	0.427	0.45	4.95E-06	SMC1
TMEM70	2.61E-09	0.11757852	0.623	0.581	5.21E-06	SMC1
MAMDC2	2.63E-09	-0.0031342	0.499	0.476	5.25E-06	SMC1
KDR	2.65E-09	0.01343386	0.534	0.335	5.30E-06	SMC1
IGKV1-39	2.69E-09	0.0031618	0.51	0.405	5.38E-06	SMC1
FEN1	2.73E-09	0.02221184	0.582	0.545	5.46E-06	SMC1
MT1A	2.80E-09	-0.587254	0.797	0.62	5.60E-06	SMC1
CLEC14A	2.86E-09	0.00289721	0.522	0.324	5.73E-06	SMC1
ALCAM	2.96E-09	0.27877248	0.625	0.941	5.93E-06	SMC1
PROX1	3.13E-09	-0.0001397	0.487	0.299	6.26E-06	SMC1
TOR3A	3.28E-09	0.07904225	0.522	0.505	6.57E-06	SMC1
PTPRZ1	3.74E-09	0.02425662	0.514	0.483	7.47E-06	SMC1

CYP27A1	3.81E-09	0.0954221	0.463	0.663	7.63E-06	SMC1
ACTA2	3.85E-09	-0.2199099	0.989	0.998	7.70E-06	SMC1
ANGPTL4	3.86E-09	-0.2739064	0.767	0.657	7.73E-06	SMC1
PLA2G7	3.93E-09	-0.0028758	0.596	0.345	7.86E-06	SMC1
NPDC1	3.98E-09	0.1573476	0.592	0.824	7.97E-06	SMC1
PCSK1N	4.03E-09	-0.0130299	0.687	0.676	8.07E-06	SMC1
SRGN	4.41E-09	0.30609266	0.848	0.973	8.83E-06	SMC1
FEZ1	4.47E-09	0.18924049	0.534	0.805	8.94E-06	SMC1
TWIST1	4.53E-09	0.13174342	0.556	0.795	9.06E-06	SMC1
COL3A1	4.57E-09	0.05524393	0.635	0.9	9.14E-06	SMC1
PDGFRB	4.69E-09	-0.1453295	0.761	0.83	9.38E-06	SMC1
HIST1H1D	4.86E-09	0.07136528	0.46	0.663	9.73E-06	SMC1
TFPI	5.04E-09	-0.071345	0.884	0.959	1.01E-05	SMC1
ACE	5.04E-09	0.00368294	0.346	0.265	1.01E-05	SMC1
LAP3	5.09E-09	0.01676777	0.694	0.82	1.02E-05	SMC1
NUAK1	5.23E-09	0.06410317	0.285	0.355	1.05E-05	SMC1
C12orf75	5.71E-09	0.28235509	0.911	0.937	1.14E-05	SMC1
LAMA2	5.87E-09	0.01058477	0.363	0.55	1.17E-05	SMC1
AC007406.3	5.95E-09	-0.0378032	0.778	0.738	1.19E-05	SMC1
MIA	6.18E-09	-2.22E-05	0.162	0.108	1.24E-05	SMC1
PTTG1	6.37E-09	0.0132641	0.456	0.586	1.27E-05	SMC1
ITGA10	6.79E-09	0.21435971	0.57	0.845	1.36E-05	SMC1
AEBP1	6.80E-09	0.39738508	0.898	0.994	1.36E-05	SMC1
NGFR	7.94E-09	-0.0100129	0.469	0.37	1.59E-05	SMC1
KRT17	9.76E-09	0.09401826	0.767	0.972	1.95E-05	SMC1
CCR1	9.90E-09	0.0175312	0.542	0.394	1.98E-05	SMC1
S100A12	1.04E-08	-0.0035271	0.42	0.219	2.08E-05	SMC1
IGKV1-17	1.08E-08	1.34E-05	0.085	0.155	2.16E-05	SMC1
TIMP3	1.14E-08	-0.0034426	0.627	0.83	2.27E-05	SMC1
RGCC	1.14E-08	0.3520887	0.676	0.786	2.28E-05	SMC1
ELL2	1.18E-08	0.03687289	0.577	0.517	2.35E-05	SMC1
IL32	1.18E-08	0.47386589	0.821	0.962	2.35E-05	SMC1
TNFSF14	1.24E-08	0.01950694	0.679	0.49	2.48E-05	SMC1
ACKR4	1.25E-08	0.02639412	0.64	0.627	2.50E-05	SMC1
HLA-DPB1	1.29E-08	0.122083	0.786	0.959	2.59E-05	SMC1
HMCN1	1.31E-08	0.1518548	0.629	0.857	2.62E-05	SMC1
GRHL2	1.37E-08	0.00255658	0.225	0.191	2.75E-05	SMC1
SMC2	1.45E-08	0.09126563	0.492	0.673	2.91E-05	SMC1
TNFRSF9	1.49E-08	0.00465742	0.735	0.617	2.99E-05	SMC1
DPEP1	1.55E-08	3.44E-06	0.071	0.135	3.11E-05	SMC1
MRGPRF	1.56E-08	0.06711226	0.487	0.709	3.13E-05	SMC1
CBR3	1.72E-08	0.09928374	0.655	0.823	3.44E-05	SMC1
LMNB1	1.84E-08	0.00527453	0.423	0.58	3.68E-05	SMC1
DAPL1	1.87E-08	-0.0442603	0.475	0.478	3.74E-05	SMC1
GCA	2.02E-08	0.01639916	0.522	0.671	4.05E-05	SMC1
SLIT3	2.10E-08	-0.2610729	0.602	0.678	4.20E-05	SMC1
ALDH3A1	2.35E-08	0.00483903	0.298	0.224	4.69E-05	SMC1
IGFBP1	2.54E-08	-0.0026385	0.065	0.126	5.07E-05	SMC1
SFRP4	2.61E-08	0.24048217	0.585	0.821	5.21E-05	SMC1
P2RY6	2.65E-08	-0.0031306	0.561	0.353	5.30E-05	SMC1
CXCL16	2.70E-08	0.05013188	0.657	0.809	5.40E-05	SMC1
ACTG2	2.71E-08	-0.1894658	0.512	0.688	5.42E-05	SMC1
IGLL5	2.90E-08	0.00251649	0.256	0.223	5.81E-05	SMC1

PDGFA	4.19E-08	-0.2113946	0.836	0.851	8.38E-05	SMC1
OGN	4.20E-08	0.25862537	0.84	0.933	8.40E-05	SMC1
EZH2	4.36E-08	0.00349066	0.764	0.74	8.73E-05	SMC1
ENPP1	4.39E-08	0.24323434	0.466	0.744	8.78E-05	SMC1
MTUS1	4.40E-08	0.19219807	0.449	0.672	8.79E-05	SMC1
CYGB	4.64E-08	-0.0189037	0.637	0.656	9.27E-05	SMC1
TUBA1A	4.75E-08	-0.1671669	0.948	0.983	9.51E-05	SMC1
FGR	5.17E-08	0.01009039	0.418	0.384	0.00010331	SMC1
LAMP5	5.28E-08	-0.0050465	0.632	0.615	0.0001056	SMC1
PI16	5.89E-08	-0.0111203	0.457	0.215	0.00011774	SMC1
FAP	6.13E-08	0.10109016	0.784	0.84	0.00012255	SMC1
CRYM	6.73E-08	-0.0003824	0.069	0.145	0.00013459	SMC1
IGHGP	6.78E-08	0.00320725	0.156	0.198	0.00013564	SMC1
PARD6G-AS	6.82E-08	0.00712195	0.478	0.305	0.00013639	SMC1
MS4A1	6.93E-08	0.00580581	0.806	0.843	0.00013858	SMC1
GUCY1B1	7.43E-08	-0.0941048	0.718	0.808	0.0001486	SMC1
IL2	7.73E-08	-0.0059093	0.392	0.279	0.00015451	SMC1
IGFBP4	8.43E-08	-0.4356873	0.793	0.759	0.00016851	SMC1
DAB2	8.52E-08	0.13750394	0.649	0.636	0.00017044	SMC1
CX3CL1	9.40E-08	0.04016355	0.645	0.597	0.00018795	SMC1
PKMYT1	9.63E-08	0.00238742	0.104	0.167	0.00019258	SMC1
LDB2	1.11E-07	0.00621785	0.604	0.716	0.00022114	SMC1
CAV2	1.14E-07	-0.1363525	0.787	0.915	0.00022776	SMC1
CTSW	1.20E-07	0.04336072	0.473	0.506	0.00024057	SMC1
IGHA1	1.22E-07	0.09298903	0.888	1	0.00024421	SMC1
STEAP4	1.30E-07	0.07021209	0.847	0.978	0.00025956	SMC1
C16orf89	1.40E-07	-0.024093	0.563	0.522	0.00028021	SMC1
CD151	1.41E-07	0.23677285	0.934	0.972	0.00028215	SMC1
GBP1	1.43E-07	0.05010987	0.645	0.779	0.00028699	SMC1
HAS2	1.52E-07	0.0031131	0.694	0.748	0.00030351	SMC1
COL10A1	1.68E-07	0.0038091	0.191	0.206	0.00033666	SMC1
TRDC	1.70E-07	0.01306607	0.729	0.503	0.00033948	SMC1
CERS1	1.77E-07	3.44E-05	0.057	0.116	0.00035417	SMC1
CDH11	1.81E-07	0.18951924	0.756	0.979	0.00036122	SMC1
IFNG	2.02E-07	0.07548449	0.8	0.927	0.00040454	SMC1
FCGR2B	2.10E-07	0.00591622	0.662	0.626	0.00041903	SMC1
ADM5	2.26E-07	0.00765721	0.355	0.236	0.00045254	SMC1
CXCL11	2.27E-07	-0.0429896	0.152	0.078	0.00045373	SMC1
CD40	2.31E-07	0.01502918	0.493	0.653	0.00046108	SMC1
NME1	2.63E-07	0.08330712	0.513	0.692	0.00052526	SMC1
CCL21	2.67E-07	0.06458006	0.819	0.84	0.0005343	SMC1
DEPDC1B	3.17E-07	0.00251434	0.156	0.207	0.00063491	SMC1
IGSF6	3.26E-07	0.0040596	0.482	0.433	0.0006517	SMC1
ANGPTL7	3.54E-07	0.01020265	0.45	0.237	0.00070805	SMC1
ATF5	3.66E-07	0.0443644	0.476	0.435	0.00073111	SMC1
PYCR1	3.67E-07	-0.0014857	0.75	0.572	0.00073383	SMC1
RETN	3.80E-07	-0.0019536	0.147	0.199	0.00075979	SMC1
IL4I1	3.82E-07	0.00641676	0.233	0.228	0.00076484	SMC1
SOD3	3.86E-07	-0.1075111	0.973	0.997	0.00077265	SMC1
MT1H	3.87E-07	0.00044981	0.648	0.413	0.0007746	SMC1
CTSL	3.97E-07	0.08315159	0.587	0.848	0.00079405	SMC1
ICAM2	4.22E-07	0.02501313	0.793	0.914	0.00084439	SMC1
SRPX	4.27E-07	-0.0458643	0.787	0.652	0.00085374	SMC1

COL5A1	4.46E-07	0.01864001	0.423	0.591	0.00089203	SMC1
RHOB	4.76E-07	-0.1014409	0.685	0.844	0.00095155	SMC1
ISYNA1	4.88E-07	-0.1781601	0.777	0.774	0.00097691	SMC1
NRP1	4.90E-07	-0.0291196	0.797	0.9	0.00097925	SMC1
SOX4	5.39E-07	0.00219005	0.835	0.919	0.00107741	SMC1
CBLN1	5.56E-07	-0.0367259	0.75	0.64	0.00111138	SMC1
MGLL	5.60E-07	-0.1343838	0.727	0.755	0.0011205	SMC1
BTNL9	6.58E-07	0.00062355	0.181	0.206	0.0013155	SMC1
FGF7	6.66E-07	-0.0418652	0.861	0.837	0.00133282	SMC1
IFIT1	6.86E-07	0.10825054	0.604	0.786	0.00137186	SMC1
SLAMF7	7.06E-07	0.00132037	0.19	0.264	0.0014126	SMC1
CLEC5A	7.58E-07	0.00483358	0.12	0.238	0.0015161	SMC1
LCN6	7.66E-07	0.00059584	0.432	0.323	0.00153117	SMC1
SLC14A1	8.20E-07	0.10946051	0.714	0.727	0.0016407	SMC1
DHRS9	8.26E-07	0.00398201	0.034	0.116	0.00165104	SMC1
CNTN4	8.53E-07	0.13007431	0.257	0.327	0.00170563	SMC1
BGN	8.80E-07	0.2289755	0.975	0.998	0.00175963	SMC1
IGLV3-21	8.88E-07	-0.0079286	0.105	0.178	0.00177699	SMC1
IGLC2	8.91E-07	0.10789234	0.946	1	0.00178282	SMC1
CCDC80	8.93E-07	0.19920422	0.889	0.878	0.00178674	SMC1
ABCA9	9.00E-07	0.01094842	0.362	0.291	0.00179999	SMC1
ENPP2	9.49E-07	0.07311624	0.676	0.869	0.0018987	SMC1
PTGER1	9.64E-07	0.0070889	0.306	0.283	0.00192849	SMC1
ADAM15	9.73E-07	0.04282917	0.559	0.59	0.00194595	SMC1
FHL1	9.76E-07	0.24818176	0.901	0.943	0.00195225	SMC1
COPZ2	1.17E-06	0.2576379	0.665	0.652	0.00233621	SMC1
PAIP2B	1.25E-06	0.06523566	0.407	0.576	0.00249142	SMC1
RND3	1.28E-06	0.00733135	0.732	0.813	0.00255338	SMC1
CTSK	1.28E-06	0.16053304	0.815	0.93	0.00256224	SMC1
SLPI	1.30E-06	0.14831661	0.814	0.894	0.00259781	SMC1
LAMA4	1.36E-06	0.04626423	0.79	0.949	0.00272276	SMC1
AQP2	1.41E-06	-6.12E-05	0.07	0.112	0.00281464	SMC1
NET1	1.55E-06	-0.0118127	0.817	0.907	0.00309229	SMC1
IL10	1.66E-06	-2.32E-05	0.754	0.733	0.00331114	SMC1
TNFRSF17	1.66E-06	0.00741759	0.48	0.359	0.00332416	SMC1
TINAGL1	1.68E-06	-0.0610532	0.934	0.972	0.00335548	SMC1
TSPAN13	1.70E-06	0.04334795	0.745	0.716	0.0033952	SMC1
RHOJ	1.74E-06	0.0372425	0.53	0.698	0.00347313	SMC1
HIST1H1C	1.75E-06	0.13453402	0.589	0.558	0.00350127	SMC1
OTUD1	1.79E-06	0.07348229	0.344	0.359	0.00358578	SMC1
PLA1A	1.88E-06	0.00075385	0.79	0.6	0.00376219	SMC1
SPINK2	1.88E-06	0.02103774	0.822	0.909	0.00376231	SMC1
MMP7	1.95E-06	-8.30E-05	0.15	0.186	0.0038907	SMC1
NUPR1	2.17E-06	0.29832744	0.901	0.95	0.00433122	SMC1
PHACTR1	2.18E-06	0.05769595	0.257	0.316	0.00435161	SMC1
OMD	2.34E-06	0.23861547	0.63	0.894	0.00468791	SMC1
CTSC	2.47E-06	0.28047197	0.869	0.976	0.00494003	SMC1
TNFAIP6	2.53E-06	-0.0063602	0.75	0.653	0.00506171	SMC1
PRDM6	2.80E-06	0.21554126	0.452	0.714	0.00559367	SMC1
CKB	2.88E-06	-0.088676	0.488	0.448	0.00576651	SMC1
PTGIS	2.97E-06	0.19648808	0.492	0.744	0.00593631	SMC1
IER3	3.08E-06	-0.29723	0.814	0.936	0.00615657	SMC1
GPR42	3.12E-06	0.00111215	0.089	0.119	0.00624797	SMC1

SLC16A3	3.17E-06	0.01324995	0.473	0.552	0.00634202	SMC1
TUBB6	3.23E-06	0.0193636	0.615	0.774	0.00645952	SMC1
PGM5-AS1	3.24E-06	0.01036394	0.342	0.357	0.00648643	SMC1
CALD1	3.34E-06	0.14931528	0.998	0.999	0.00668793	SMC1
HNMT	3.43E-06	0.12419174	0.789	0.915	0.00685027	SMC1
ARL4D	3.48E-06	-0.07172	0.428	0.441	0.00695029	SMC1
IGHV4-34	3.74E-06	1.85E-05	0.103	0.162	0.00747673	SMC1
LINC01133	3.75E-06	-0.0072356	0.248	0.21	0.00749499	SMC1
BMP3	3.93E-06	-0.0015471	0.375	0.273	0.00785636	SMC1
LILRB3	3.95E-06	0.01039241	0.19	0.22	0.00790975	SMC1
IGLV2-8	4.09E-06	-0.0009103	0.466	0.477	0.00817553	SMC1
TMEM98	4.26E-06	0.15351799	0.644	0.87	0.00852643	SMC1
IGLC3	4.62E-06	-0.0299388	0.857	0.948	0.0092346	SMC1
THBS2	4.88E-06	0.25582555	0.836	0.962	0.00976134	SMC1
NCF2	4.93E-06	-0.0200021	0.601	0.535	0.00985385	SMC1
SCRG1	4.94E-06	0.19222287	0.445	0.64	0.00988569	SMC1
DES	5.09E-06	-0.3204904	0.329	0.483	0.0101814	SMC1
TIMD4	5.12E-06	0.00080645	0.105	0.16	0.01023201	SMC1
GREM1	5.39E-06	0.00194193	0.313	0.281	0.01078556	SMC1
LINC01235	5.49E-06	0.00063664	0.273	0.343	0.01098528	SMC1
PTX3	5.80E-06	-0.0068189	0.511	0.584	0.01160918	SMC1
RSAD2	5.84E-06	0.02600638	0.486	0.572	0.01167733	SMC1
IGHA2	5.90E-06	-0.000803	0.732	0.608	0.01180686	SMC1
CAV1	6.66E-06	-0.119678	0.942	0.981	0.01331126	SMC1
AK5	6.75E-06	-0.0245681	0.429	0.435	0.01349507	SMC1
CPXM2	7.33E-06	0.18133143	0.517	0.763	0.01465919	SMC1
IGHG2	7.41E-06	0.00561616	0.822	0.995	0.01482658	SMC1
RGS18	7.58E-06	0.00914003	0.188	0.223	0.01516789	SMC1
COL21A1	7.68E-06	0.20997207	0.446	0.658	0.01535174	SMC1
TIMP1	7.76E-06	0.26319455	0.994	0.999	0.01551694	SMC1
ITM2C	7.92E-06	0.06293509	0.547	0.78	0.01583733	SMC1
ABRA	8.19E-06	-0.0107536	0.357	0.488	0.01638057	SMC1
FNDC1	8.25E-06	0.01431795	0.586	0.623	0.01649306	SMC1
CEBPB	8.33E-06	-0.0881699	0.712	0.847	0.01666323	SMC1
MXRA8	8.38E-06	0.18581611	0.608	0.869	0.01675521	SMC1
CTSD	8.69E-06	0.29864928	0.882	0.984	0.01738337	SMC1
S100A4	8.92E-06	-0.3138642	0.991	0.998	0.01784764	SMC1
KLRC1	9.11E-06	0.00590639	0.782	0.633	0.01821456	SMC1
CCL23	9.17E-06	-0.0021824	0.133	0.065	0.0183428	SMC1
CYTOR	1.11E-05	-0.1173065	0.877	0.914	0.02216052	SMC1
PHLDA1	1.11E-05	-0.3700147	0.836	0.87	0.02228438	SMC1
TYMS	1.15E-05	-0.0157523	0.351	0.369	0.02294035	SMC1
LGALSL	1.19E-05	-0.1452561	0.813	0.845	0.02389823	SMC1
FABP3	1.20E-05	0.10185606	0.33	0.342	0.02402912	SMC1
CX3CR1	1.20E-05	0.00371073	0.092	0.133	0.0240706	SMC1
CR1	1.22E-05	0.00705485	0.094	0.116	0.02437022	SMC1
CD37	1.23E-05	0.15992808	0.781	0.886	0.02459477	SMC1
TMEM100	1.30E-05	0.01425502	0.192	0.227	0.02592596	SMC1
PAICS	1.33E-05	0.01451835	0.429	0.609	0.02655474	SMC1
BID	1.36E-05	0.02515919	0.709	0.83	0.02723432	SMC1
DEPP1	1.37E-05	-0.1747265	0.631	0.723	0.02745417	SMC1
CRLF1	1.42E-05	-0.0052639	0.388	0.348	0.02835986	SMC1
STAB1	1.50E-05	0.01479055	0.461	0.434	0.02993117	SMC1

IGFBP5	1.54E-05	-0.0592395	0.928	0.987	0.03088641	SMC1
PHLDA3	1.57E-05	-0.000722	0.786	0.864	0.03148648	SMC1
NPC2	1.70E-05	0.37267103	0.9	0.985	0.03399594	SMC1
KCNK17	1.71E-05	-0.0236772	0.736	0.774	0.03420334	SMC1
UCHL1	1.73E-05	0.1286879	0.287	0.483	0.03461699	SMC1
IGKV3-15	1.77E-05	-0.0001109	0.102	0.157	0.03531966	SMC1
RGS2	1.77E-05	0.13132637	0.574	0.71	0.03545399	SMC1
HSPG2	1.79E-05	0.02531135	0.313	0.388	0.03572369	SMC1
CPVL	1.86E-05	0.02270977	0.514	0.512	0.03715335	SMC1
HMMR	1.90E-05	-0.0007401	0.435	0.333	0.03798614	SMC1
SAA1	1.93E-05	0.00081065	0.114	0.126	0.03860306	SMC1
FAT1	1.97E-05	-0.0759205	0.486	0.587	0.03934139	SMC1
AL157895.1	2.07E-05	0.00109959	0.29	0.212	0.04149526	SMC1
WWTR1	2.09E-05	0.22555608	0.589	0.823	0.04181636	SMC1
ECEL1	2.14E-05	-0.0011357	0.474	0.324	0.0428383	SMC1
CTSG	2.38E-05	0.00944972	0.786	0.735	0.04759563	SMC1
TNFAIP2	2.38E-05	0.02098856	0.743	0.724	0.04763648	SMC1
RGS16	2.39E-05	-0.5551877	0.881	0.931	0.04772601	SMC1
EPB41L2	2.47E-05	0.16094504	0.682	0.873	0.04942628	SMC1
DUSP2	9.62E-177	-0.0478717	0.688	0.999	1.92E-173	SMC2
IL1B	3.24E-167	0.13064525	0.051	0.709	6.49E-164	SMC2
FCER1G	1.36E-159	0.2002007	0.239	0.892	2.72E-156	SMC2
COTL1	2.18E-157	0.28990917	0.367	0.932	4.36E-154	SMC2
CCL3	1.65E-141	0.41714228	0.349	0.905	3.30E-138	SMC2
CCR7	1.97E-141	0.05262407	0.501	0.953	3.94E-138	SMC2
IL1RN	7.70E-129	0.05185588	0.057	0.537	1.54E-125	SMC2
GLRX	4.35E-128	0.18151761	0.287	0.894	8.70E-125	SMC2
IL7R	1.21E-127	0.15552336	0.271	0.841	2.42E-124	SMC2
VCAM1	2.31E-122	0.26660413	0.589	0.967	4.63E-119	SMC2
PAPPA	6.54E-122	0.10211214	0.23	0.832	1.31E-118	SMC2
CCL5	2.22E-120	0.66294966	0.543	0.973	4.44E-117	SMC2
ITGB2	2.63E-118	0.06685819	0.456	0.848	5.26E-115	SMC2
APOC1	1.23E-117	0.356561	0.115	0.707	2.47E-114	SMC2
BIRC3	3.65E-117	0.14540939	0.564	0.951	7.31E-114	SMC2
ATP2B1-AS1	3.14E-113	-0.1138973	0.499	0.913	6.28E-110	SMC2
CXCR6	8.25E-112	-0.0175688	0.478	0.81	1.65E-108	SMC2
TSPAN2	4.88E-111	0.08229449	0.368	0.874	9.76E-108	SMC2
HMOX1	3.14E-110	0.14963336	0.498	0.888	6.27E-107	SMC2
LAPTM5	5.16E-102	0.40613239	0.39	0.905	1.03E-98	SMC2
GPR183	6.37E-101	0.27755272	0.219	0.77	1.27E-97	SMC2
CCL3L1	4.75E-100	0.25676939	0.185	0.753	9.51E-97	SMC2
TFPI2	7.74E-99	0.28070898	0.156	0.696	1.55E-95	SMC2
AIF1	7.02E-97	0.34209084	0.155	0.715	1.40E-93	SMC2
MGST1	9.45E-97	-0.0778197	0.413	0.133	1.89E-93	SMC2
PART1	4.47E-96	0.06834799	0.095	0.523	8.94E-93	SMC2
CTSW	1.49E-95	-0.0206951	0.167	0.637	2.98E-92	SMC2
IGSF6	2.30E-95	0.01928456	0.434	0.814	4.59E-92	SMC2
LCP1	1.50E-94	0.14901428	0.524	0.901	3.00E-91	SMC2
TNF	2.66E-94	-0.0440686	0.582	0.888	5.33E-91	SMC2
CSF2RA	1.11E-92	-0.0024357	0.157	0.1	2.22E-89	SMC2
FYB1	1.34E-92	0.06481182	0.158	0.669	2.67E-89	SMC2
DUSP5	2.36E-92	0.06060825	0.647	0.959	4.73E-89	SMC2
LGALS2	6.48E-92	-0.0050046	0.585	0.822	1.30E-88	SMC2

CD52	1.03E-88	0.39945064	0.592	0.93	2.06E-85	SMC2
CD69	5.71E-87	0.27274555	0.27	0.782	1.14E-83	SMC2
DUSP4	2.12E-84	-0.0829275	0.609	0.896	4.24E-81	SMC2
CYTIP	3.34E-83	0.10382715	0.545	0.885	6.67E-80	SMC2
CSF1R	7.69E-83	0.06947959	0.39	0.751	1.54E-79	SMC2
IFI30	2.97E-82	0.002782	0.193	0.668	5.94E-79	SMC2
MMP7	3.83E-82	0.01891774	0.144	0.549	7.66E-79	SMC2
AC243960.1	5.34E-82	-0.0163751	0.312	0.749	1.07E-78	SMC2
PTPRE	7.65E-82	0.17378379	0.161	0.621	1.53E-78	SMC2
HLA-DMB	8.02E-82	0.10056104	0.207	0.734	1.60E-78	SMC2
MZB1	9.98E-82	0.00900824	0.54	0.936	2.00E-78	SMC2
LMO3	2.33E-81	0.04642376	0.5	0.222	4.65E-78	SMC2
PARD6G-AS	1.07E-80	-0.0121361	0.082	0.444	2.14E-77	SMC2
CRTAC1	2.19E-80	0.40206568	0.336	0.821	4.37E-77	SMC2
PDE4B	3.29E-80	0.10814306	0.371	0.782	6.58E-77	SMC2
LTB	7.83E-80	0.07497327	0.614	0.898	1.57E-76	SMC2
GBP5	1.80E-79	-0.0041782	0.509	0.841	3.60E-76	SMC2
CD3D	7.93E-79	0.13112897	0.614	0.86	1.59E-75	SMC2
TPX2	2.27E-78	0.02574996	0.082	0.411	4.54E-75	SMC2
ENPP1	3.21E-78	0.31596927	0.307	0.829	6.41E-75	SMC2
C5AR1	2.25E-76	0.0601152	0.586	0.886	4.49E-73	SMC2
NRN1	3.33E-76	0.03837824	0.144	0.581	6.65E-73	SMC2
BATF	3.82E-76	0.01340147	0.639	0.911	7.64E-73	SMC2
G0S2	5.62E-75	0.01908461	0.378	0.793	1.12E-71	SMC2
KCNN4	6.60E-75	0.01260672	0.207	0.611	1.32E-71	SMC2
CD84	7.45E-75	0.05372043	0.126	0.598	1.49E-71	SMC2
COLEC11	2.78E-74	0.03967508	0.134	0.499	5.56E-71	SMC2
PTGER3	1.39E-73	0.27639863	0.359	0.841	2.77E-70	SMC2
ARHGAP18	1.59E-73	0.14589306	0.48	0.869	3.18E-70	SMC2
RAMP2	1.62E-73	-0.2394886	0.335	0.221	3.23E-70	SMC2
ALDH1A2	3.34E-73	0.18512534	0.636	0.889	6.68E-70	SMC2
HLA-DQA1	3.05E-72	0.19890467	0.2	0.669	6.10E-69	SMC2
RNASET2	6.52E-72	0.18817741	0.621	0.908	1.30E-68	SMC2
LIMCH1	2.19E-71	0.10443391	0.215	0.728	4.37E-68	SMC2
SNX10	4.83E-71	-0.0089512	0.237	0.636	9.66E-68	SMC2
HLA-DPB1	4.89E-71	0.26266852	0.427	0.848	9.78E-68	SMC2
CACNA2D3	1.17E-70	-0.0293611	0.104	0.423	2.34E-67	SMC2
LILRB4	2.88E-70	0.00802377	0.519	0.905	5.76E-67	SMC2
AC025164.1	5.55E-70	-0.0731207	0.586	0.897	1.11E-66	SMC2
SCIMP	4.96E-69	0.03490833	0.237	0.153	9.92E-66	SMC2
CITED2	6.93E-69	0.1541738	0.596	0.869	1.39E-65	SMC2
EDIL3	7.66E-69	0.49312213	0.324	0.935	1.53E-65	SMC2
CXCR4	1.49E-68	0.22441209	0.589	0.869	2.98E-65	SMC2
SPOCK2	2.79E-67	0.03981799	0.079	0.558	5.57E-64	SMC2
RHOH	5.40E-67	0.04925608	0.613	0.888	1.08E-63	SMC2
CXCL10	1.47E-66	0.19640519	0.665	0.938	2.95E-63	SMC2
SPC24	2.05E-66	-0.0153277	0.118	0.42	4.10E-63	SMC2
RGS3	3.46E-66	0.1133482	0.296	0.737	6.92E-63	SMC2
IER3	7.15E-66	0.02457109	0.76	0.996	1.43E-62	SMC2
CREB5	8.62E-66	-0.3478234	0.234	0.171	1.72E-62	SMC2
PPIF	1.66E-65	0.027866	0.26	0.665	3.32E-62	SMC2
THSD4	1.85E-65	-0.1395682	0.345	0.161	3.71E-62	SMC2
KIF23	2.10E-65	0.02377188	0.26	0.579	4.19E-62	SMC2

DIO2	2.11E-65	0.0177911	0.382	0.832	4.22E-62	SMC2
PLD5	5.26E-65	-0.0230233	0.434	0.234	1.05E-61	SMC2
CCDC144NL	1.82E-64	0.15913308	0.563	0.89	3.65E-61	SMC2
CHST1	2.21E-64	0.13556025	0.454	0.798	4.42E-61	SMC2
NRIP3	2.37E-64	0.02080635	0.251	0.593	4.75E-61	SMC2
ANKRD28	4.53E-64	0.04189162	0.68	0.962	9.06E-61	SMC2
LINC00996	6.87E-64	-0.0127455	0.336	0.6	1.37E-60	SMC2
NPR3	8.40E-64	-0.0113988	0.536	0.297	1.68E-60	SMC2
VSIG4	1.15E-63	0.0820271	0.662	0.908	2.30E-60	SMC2
LOX	1.45E-63	-0.1184882	0.335	0.19	2.91E-60	SMC2
CXCR3	1.53E-63	0.01556386	0.705	0.935	3.06E-60	SMC2
EZH2	2.19E-63	0.02067316	0.146	0.535	4.38E-60	SMC2
ITLN1	4.88E-63	0.30372364	0.036	0.419	9.76E-60	SMC2
AC023157.3	6.71E-62	0.06845069	0.636	0.938	1.34E-58	SMC2
DLX6-AS1	1.34E-61	-0.1229596	0.514	0.225	2.67E-58	SMC2
ENPP2	1.59E-61	-0.0344608	0.296	0.607	3.18E-58	SMC2
CLEC7A	3.37E-61	0.05825067	0.577	0.848	6.74E-58	SMC2
HLA-DPA1	3.58E-61	0.53447523	0.399	0.892	7.16E-58	SMC2
SVEP1	4.14E-61	-0.3970625	0.611	0.324	8.27E-58	SMC2
HCK	3.64E-60	-0.0051539	0.115	0.381	7.28E-57	SMC2
FHL2	5.42E-60	0.1251483	0.291	0.737	1.08E-56	SMC2
MS4A4A	8.32E-60	0.06924278	0.147	0.504	1.66E-56	SMC2
MSR1	1.07E-59	0.07350761	0.051	0.42	2.15E-56	SMC2
CRISPLD2	2.56E-59	-0.4581963	0.4	0.238	5.11E-56	SMC2
TRBC2	2.97E-59	0.18061495	0.706	0.954	5.94E-56	SMC2
RNF144B	4.97E-59	-0.0065915	0.55	0.859	9.94E-56	SMC2
NCF4	6.44E-59	-0.0007293	0.614	0.924	1.29E-55	SMC2
AP000692.2	1.47E-58	-0.0631923	0.654	0.935	2.95E-55	SMC2
CTSZ	1.58E-57	0.32863812	0.33	0.828	3.16E-54	SMC2
PRG4	4.02E-57	-0.2495825	0.319	0.167	8.05E-54	SMC2
RGS1	4.49E-57	0.22108688	0.581	0.856	8.98E-54	SMC2
TOR3A	7.51E-57	0.05633702	0.583	0.912	1.50E-53	SMC2
C1orf162	1.40E-56	-0.003144	0.489	0.818	2.80E-53	SMC2
ADAMTS9	5.27E-56	-0.1500923	0.496	0.324	1.05E-52	SMC2
PNP	6.08E-56	0.06941609	0.677	0.938	1.22E-52	SMC2
C1QA	1.12E-55	0.53355375	0.275	0.741	2.23E-52	SMC2
IL2RA	1.30E-55	0.02071237	0.732	0.94	2.59E-52	SMC2
UCHL1	1.70E-55	-0.0208747	0.29	0.178	3.40E-52	SMC2
LAIR1	2.72E-55	0.0884492	0.109	0.542	5.43E-52	SMC2
AURKA	5.80E-55	-0.0037877	0.652	0.893	1.16E-51	SMC2
APOE	7.03E-55	1.48073917	0.346	0.886	1.41E-51	SMC2
NRP2	1.23E-54	0.17416096	0.372	0.813	2.46E-51	SMC2
APOD	1.31E-54	-2.2409794	0.579	0.327	2.62E-51	SMC2
HSPA6	1.33E-54	-0.1395926	0.548	0.786	2.66E-51	SMC2
UPP1	1.60E-54	0.07875832	0.339	0.748	3.19E-51	SMC2
PLPP5	1.69E-54	0.04187469	0.685	0.959	3.39E-51	SMC2
AREG	2.45E-54	0.12525782	0.2	0.645	4.89E-51	SMC2
FABP3	3.50E-54	-0.0434583	0.535	0.31	6.99E-51	SMC2
MCEMP1	5.03E-54	0.0127208	0.002	0.316	1.01E-50	SMC2
INSIG1	5.24E-54	-0.0740421	0.611	0.886	1.05E-50	SMC2
MT1M	5.67E-54	0.20086402	0.289	0.213	1.13E-50	SMC2
COCH	6.09E-54	-0.0366816	0.485	0.226	1.22E-50	SMC2
JCHAIN	8.20E-54	0.18063758	0.665	0.9	1.64E-50	SMC2

FCER1A	1.05E-53	-0.0127483	0.703	0.879	2.10E-50	SMC2
CTSL	1.84E-53	0.25513913	0.282	0.774	3.67E-50	SMC2
CRTAM	4.64E-53	0.00183759	0.092	0.431	9.29E-50	SMC2
LYZ	6.89E-53	0.29190017	0.391	0.747	1.38E-49	SMC2
GPR34	1.61E-52	-0.0182699	0.224	0.604	3.21E-49	SMC2
RHOJ	2.71E-52	0.11891655	0.655	0.875	5.42E-49	SMC2
PDGFRA	1.06E-51	-0.0499779	0.408	0.274	2.13E-48	SMC2
MALL	3.85E-51	0.0064626	0.608	0.844	7.70E-48	SMC2
IGLL1	6.28E-51	-0.0002003	0.256	0.58	1.26E-47	SMC2
HCST	8.07E-51	0.19721135	0.263	0.664	1.61E-47	SMC2
F2R	9.25E-51	0.27432887	0.6	0.907	1.85E-47	SMC2
GMDS	1.25E-50	0.22511884	0.356	0.766	2.49E-47	SMC2
CCR1	1.49E-50	0.01746492	0.211	0.518	2.98E-47	SMC2
HLA-DRA	1.69E-50	0.82036739	0.786	0.967	3.37E-47	SMC2
ATP6V1B2	2.50E-50	0.1326715	0.523	0.359	5.00E-47	SMC2
RGS18	2.54E-50	0.01858055	0.157	0.507	5.09E-47	SMC2
FAM13C	2.97E-50	0.15973925	0.364	0.768	5.95E-47	SMC2
ACOT7	5.73E-50	0.11033655	0.261	0.644	1.15E-46	SMC2
ACAN	6.80E-50	-0.0023295	0.228	0.146	1.36E-46	SMC2
ASGR1	8.38E-50	-0.0006256	0.078	0.461	1.68E-46	SMC2
F5	1.01E-49	0.00069272	0.618	0.835	2.02E-46	SMC2
IFIT2	1.17E-49	0.14100025	0.524	0.836	2.34E-46	SMC2
FOLR3	1.54E-49	-0.0163723	0.504	0.301	3.07E-46	SMC2
FSCN1	2.17E-49	-0.0219509	0.297	0.721	4.35E-46	SMC2
FBP1	5.99E-49	0.00963834	0.696	0.917	1.20E-45	SMC2
FMOD	7.19E-49	0.15354983	0.086	0.511	1.44E-45	SMC2
FKBP10	1.09E-48	-0.1261899	0.461	0.791	2.19E-45	SMC2
SLC11A1	3.55E-48	-0.0415151	0.416	0.736	7.09E-45	SMC2
C1QC	3.97E-48	0.31985478	0.556	0.863	7.94E-45	SMC2
STAB1	6.10E-48	0.05879558	0.341	0.748	1.22E-44	SMC2
SFRP4	1.03E-47	-0.6621842	0.501	0.341	2.07E-44	SMC2
PGAM2	1.22E-47	0.01496606	0.109	0.366	2.44E-44	SMC2
LST1	1.35E-47	-0.0778487	0.483	0.766	2.71E-44	SMC2
CTSH	1.53E-47	-0.1050231	0.359	0.743	3.06E-44	SMC2
NLRP3	2.09E-47	-0.0004516	0.438	0.671	4.19E-44	SMC2
ITM2C	3.76E-47	0.23424068	0.278	0.699	7.52E-44	SMC2
FAM92B	5.10E-47	0.00146438	0.269	0.589	1.02E-43	SMC2
ACOXL	6.04E-47	-0.0028783	0.395	0.659	1.21E-43	SMC2
AXL	6.87E-47	0.03580072	0.193	0.188	1.37E-43	SMC2
C1QB	9.58E-47	0.73475885	0.615	0.961	1.92E-43	SMC2
SMOC1	1.27E-46	0.10114009	0.391	0.711	2.53E-43	SMC2
TGFB2	2.31E-46	0.05051233	0.4	0.257	4.61E-43	SMC2
CD68	3.52E-46	0.045995	0.142	0.533	7.04E-43	SMC2
RASGEF1B	5.01E-46	0.15134775	0.549	0.832	1.00E-42	SMC2
LILRB1	5.60E-46	-0.0005379	0.725	0.893	1.12E-42	SMC2
PDGFB	7.20E-46	0.03610084	0.512	0.725	1.44E-42	SMC2
TLR2	8.36E-46	0.12264149	0.198	0.136	1.67E-42	SMC2
FCGBP	1.68E-45	0.0114371	0.052	0.289	3.36E-42	SMC2
TYMP	2.04E-45	0.14364492	0.631	0.932	4.09E-42	SMC2
SMPD3	2.34E-45	0.00660077	0.437	0.638	4.68E-42	SMC2
PTPRB	8.54E-45	0.02545137	0.67	0.86	1.71E-41	SMC2
SGCE	1.65E-44	0.07968181	0.421	0.298	3.30E-41	SMC2
EDN1	2.76E-44	0.11373535	0.236	0.633	5.53E-41	SMC2

CD247	3.79E-44	0.07094857	0.698	0.936	7.58E-41	SMC2
PLEK	4.57E-44	0.13918841	0.437	0.763	9.13E-41	SMC2
EMILIN1	5.98E-44	0.40976714	0.338	0.786	1.20E-40	SMC2
CD14	7.31E-44	0.25319884	0.468	0.797	1.46E-40	SMC2
CXorf21	7.75E-44	0.03044302	0.426	0.645	1.55E-40	SMC2
SPNS2	1.60E-43	0.01770224	0.487	0.367	3.19E-40	SMC2
OR51E1	1.91E-43	0.00937938	0.517	0.257	3.81E-40	SMC2
RETN	2.27E-43	0.0177177	0.035	0.354	4.53E-40	SMC2
SPRY1	2.27E-43	-0.0415	0.615	0.919	4.54E-40	SMC2
GALNT5	2.38E-43	0.04809192	0.427	0.71	4.76E-40	SMC2
AKR1C2	9.01E-43	0.20388709	0.259	0.129	1.80E-39	SMC2
NRP1	9.15E-43	-0.2167444	0.262	0.236	1.83E-39	SMC2
PDGFRL	9.66E-43	-0.1001723	0.639	0.44	1.93E-39	SMC2
CYP1B1	1.95E-42	0.00517095	0.397	0.706	3.90E-39	SMC2
GOLM1	2.29E-42	-0.0221068	0.192	0.486	4.59E-39	SMC2
SUGCT	2.86E-42	0.33501652	0.525	0.818	5.72E-39	SMC2
AQP2	2.98E-42	-0.0077713	0.196	0.164	5.97E-39	SMC2
HAND2	3.45E-42	-0.1941822	0.56	0.434	6.91E-39	SMC2
ACKR3	3.81E-42	-0.3648304	0.527	0.382	7.61E-39	SMC2
PNOC	4.14E-42	0.00345227	0.329	0.641	8.27E-39	SMC2
SDC1	5.00E-42	0.00828512	0.362	0.673	1.00E-38	SMC2
IL32	5.27E-42	0.23956052	0.567	0.843	1.05E-38	SMC2
E2F1	8.17E-42	-0.0134937	0.104	0.341	1.63E-38	SMC2
DIAPH3	8.48E-42	-0.0006344	0.639	0.904	1.70E-38	SMC2
SIRPB1	9.73E-42	-0.0362282	0.731	0.913	1.95E-38	SMC2
SIGLEC1	1.41E-41	0.0123184	0.363	0.619	2.82E-38	SMC2
ACKR1	1.45E-41	-0.2416752	0.681	0.557	2.90E-38	SMC2
TDO2	1.64E-41	0.10678679	0.747	0.976	3.28E-38	SMC2
MS4A6A	2.02E-41	0.2975319	0.171	0.558	4.04E-38	SMC2
NME1	2.24E-41	0.10427524	0.618	0.931	4.47E-38	SMC2
IGHV3-43	2.56E-41	-0.0024331	0.299	0.607	5.12E-38	SMC2
IGLV2-11	3.18E-41	-0.0046862	0.275	0.595	6.35E-38	SMC2
TRBC1	3.89E-41	0.02505444	0.544	0.832	7.77E-38	SMC2
BMX	5.03E-41	0.00354601	0.047	0.309	1.01E-37	SMC2
OLR1	5.94E-41	0.01908911	0.23	0.535	1.19E-37	SMC2
TAC1	8.18E-41	-0.1226991	0.071	0.363	1.64E-37	SMC2
NBL1	1.20E-40	0.18829971	0.747	0.542	2.40E-37	SMC2
CD83	1.34E-40	0.078433	0.267	0.653	2.67E-37	SMC2
DNASE1L3	2.56E-40	-0.1338503	0.532	0.435	5.12E-37	SMC2
SGO1	2.76E-40	-0.004409	0.18	0.457	5.52E-37	SMC2
NEFM	4.97E-40	-0.0541224	0.481	0.732	9.95E-37	SMC2
NDUFAF6	6.65E-40	-0.0341531	0.303	0.637	1.33E-36	SMC2
MAFF	7.20E-40	-0.1152178	0.5	0.364	1.44E-36	SMC2
DPEP1	1.02E-39	-0.0006328	0.347	0.678	2.04E-36	SMC2
CERS1	1.02E-39	-0.1778787	0.15	0.117	2.04E-36	SMC2
IGHG1	1.09E-39	0.02441768	0.758	0.958	2.18E-36	SMC2
C3AR1	1.17E-39	-0.0008289	0.089	0.287	2.33E-36	SMC2
CLEC4A	1.20E-39	0.03564393	0.646	0.85	2.39E-36	SMC2
DUSP23	2.21E-39	0.1613349	0.518	0.828	4.42E-36	SMC2
SLC8A1	2.35E-39	0.11510167	0.653	0.911	4.69E-36	SMC2
POSTN	2.54E-39	0.66570472	0.691	0.986	5.09E-36	SMC2
AGT	2.97E-39	0.40588514	0.519	0.916	5.94E-36	SMC2
AC233755.2	3.28E-39	-0.001684	0.299	0.608	6.57E-36	SMC2

CD4	3.84E-39	0.00110839	0.274	0.194	7.69E-36	SMC2
TNC	3.86E-39	0.17048183	0.782	0.988	7.72E-36	SMC2
DUSP6	4.18E-39	0.108752	0.724	0.981	8.36E-36	SMC2
NGFR	4.53E-39	-0.0134577	0.341	0.157	9.06E-36	SMC2
HP	7.79E-39	-0.1020201	0.465	0.244	1.56E-35	SMC2
SIGLEC15	1.11E-38	-0.0309291	0.341	0.205	2.22E-35	SMC2
RAB27B	1.17E-38	0.03030643	0.681	0.883	2.34E-35	SMC2
LOXL1	1.47E-38	0.09726877	0.297	0.672	2.93E-35	SMC2
GCA	1.73E-38	-0.0025945	0.434	0.722	3.45E-35	SMC2
TYROBP	1.82E-38	0.3312328	0.64	0.919	3.64E-35	SMC2
ITGA1	1.97E-38	-0.0346086	0.355	0.802	3.94E-35	SMC2
FBLN1	2.07E-38	-1.2190996	0.29	0.65	4.15E-35	SMC2
PODN	2.24E-38	-0.321191	0.242	0.262	4.48E-35	SMC2
IGHGP	3.22E-38	-0.0362507	0.355	0.682	6.44E-35	SMC2
VWF	3.33E-38	0.01553189	0.578	0.859	6.67E-35	SMC2
CCL4L2	5.05E-38	0.39359112	0.711	0.919	1.01E-34	SMC2
TREM1	5.43E-38	0.02689696	0.561	0.703	1.09E-34	SMC2
RGCC	5.45E-38	0.15673421	0.663	0.882	1.09E-34	SMC2
LINC00924	9.64E-38	-0.0221238	0.491	0.262	1.93E-34	SMC2
TCL1A	1.23E-37	-0.0030158	0.734	0.874	2.46E-34	SMC2
ZNF503	1.26E-37	0.04876489	0.229	0.214	2.53E-34	SMC2
TNFRSF4	1.33E-37	0.02103867	0.737	0.974	2.67E-34	SMC2
STEAP1	1.68E-37	0.11017402	0.631	0.9	3.36E-34	SMC2
SCX	2.27E-37	0.16649833	0.144	0.512	4.55E-34	SMC2
GZMB	2.78E-37	0.07305618	0.658	0.949	5.57E-34	SMC2
AC012236.1	3.26E-37	-0.0006801	0.27	0.581	6.51E-34	SMC2
LINC01781	4.51E-37	0.00780047	0.706	0.862	9.02E-34	SMC2
MT1A	6.33E-37	-0.1615125	0.25	0.257	1.27E-33	SMC2
MARCH1	6.93E-37	0.01342462	0.708	0.961	1.39E-33	SMC2
RNASE1	7.40E-37	-0.0225373	0.659	0.919	1.48E-33	SMC2
CRIM1	8.21E-37	0.41485466	0.359	0.272	1.64E-33	SMC2
FAM111B	8.84E-37	-0.0012398	0.142	0.453	1.77E-33	SMC2
ARL4D	9.81E-37	0.10293665	0.386	0.27	1.96E-33	SMC2
FOLR2	1.05E-36	0.06048795	0.551	0.835	2.10E-33	SMC2
CST7	1.38E-36	0.08574552	0.587	0.79	2.77E-33	SMC2
IGHV3-30	2.26E-36	0.00532126	0.343	0.64	4.52E-33	SMC2
GZMH	2.38E-36	0.17770854	0.302	0.661	4.76E-33	SMC2
SCN7A	2.40E-36	-0.0647385	0.108	0.469	4.80E-33	SMC2
TMEFF2	2.56E-36	-0.0301925	0.393	0.302	5.12E-33	SMC2
PLTP	2.60E-36	-0.0354934	0.215	0.568	5.20E-33	SMC2
FAP	2.63E-36	0.22290743	0.679	0.957	5.25E-33	SMC2
ARL15	3.90E-36	-0.0030214	0.118	0.122	7.80E-33	SMC2
MSRB3	4.23E-36	0.18064394	0.549	0.869	8.46E-33	SMC2
CORO1A	5.90E-36	0.20695171	0.361	0.707	1.18E-32	SMC2
RGS4	6.99E-36	0.09581461	0.532	0.839	1.40E-32	SMC2
TMEM158	8.31E-36	0.00475818	0.69	0.875	1.66E-32	SMC2
KRT14	8.70E-36	0.00512177	0.571	0.795	1.74E-32	SMC2
FAM241A	9.44E-36	0.16820802	0.466	0.339	1.89E-32	SMC2
LGALSL	1.06E-35	-0.039278	0.621	0.489	2.12E-32	SMC2
ABRA	1.08E-35	0.03208429	0.062	0.313	2.15E-32	SMC2
ALKAL2	1.15E-35	-0.0322054	0.199	0.15	2.30E-32	SMC2
AC245014.3	2.33E-35	-0.0563331	0.276	0.237	4.66E-32	SMC2
GPC3	3.61E-35	-0.0356772	0.241	0.173	7.22E-32	SMC2

EZR	4.08E-35	0.1482402	0.408	0.726	8.16E-32	SMC2
CDCA8	7.58E-35	-0.0027463	0.618	0.795	1.52E-31	SMC2
GAP43	7.68E-35	0.02385076	0.643	0.856	1.54E-31	SMC2
CD300A	1.01E-34	0.01144381	0.587	0.756	2.02E-31	SMC2
PLIN2	1.29E-34	0.12853803	0.306	0.694	2.59E-31	SMC2
CRLF1	2.52E-34	0.07929256	0.514	0.308	5.04E-31	SMC2
MS4A2	2.98E-34	-0.0003814	0.097	0.336	5.96E-31	SMC2
FHL5	3.67E-34	0.09042689	0.192	0.57	7.34E-31	SMC2
CYP27A1	3.82E-34	0.02232403	0.434	0.726	7.63E-31	SMC2
CCDC102B	4.53E-34	0.51301434	0.804	0.992	9.05E-31	SMC2
FCGR2B	5.10E-34	0.01436381	0.684	0.949	1.02E-30	SMC2
HELLS	5.75E-34	0.05477936	0.229	0.545	1.15E-30	SMC2
TNFSF13B	7.22E-34	0.01700752	0.381	0.774	1.44E-30	SMC2
MARCKSL1	1.37E-33	0.04699543	0.488	0.776	2.74E-30	SMC2
TNFRSF9	2.12E-33	-0.0050121	0.689	0.852	4.25E-30	SMC2
DLX5	2.36E-33	-0.1272253	0.608	0.459	4.71E-30	SMC2
IGHV1-3	3.09E-33	-0.0071245	0.071	0.427	6.18E-30	SMC2
SMC4	4.66E-33	0.23714368	0.529	0.806	9.32E-30	SMC2
CLEC11A	4.72E-33	0.08629436	0.608	0.772	9.44E-30	SMC2
HLA-DQB1	5.33E-33	0.08443007	0.538	0.916	1.07E-29	SMC2
CD163	6.01E-33	0.05562029	0.263	0.569	1.20E-29	SMC2
APOLD1	6.50E-33	0.01083536	0.586	0.53	1.30E-29	SMC2
ADGRL4	8.06E-33	0.02701998	0.672	0.866	1.61E-29	SMC2
VEGFD	8.19E-33	-0.0117134	0.239	0.205	1.64E-29	SMC2
EMCN	1.12E-32	0.0227	0.724	0.974	2.24E-29	SMC2
SGO2	1.53E-32	0.01466815	0.259	0.569	3.06E-29	SMC2
MT1G	1.77E-32	-0.0172546	0.579	0.755	3.54E-29	SMC2
HIST1H1A	2.50E-32	0.02047952	0.71	0.603	5.00E-29	SMC2
SCRG1	2.51E-32	0.1180874	0.216	0.209	5.03E-29	SMC2
FMO1	3.12E-32	-0.0237674	0.249	0.203	6.24E-29	SMC2
LINC01480	3.31E-32	-0.0107813	0.5	0.705	6.62E-29	SMC2
ANKRD22	4.55E-32	0.01111808	0.027	0.217	9.09E-29	SMC2
PMAIP1	5.62E-32	-0.0054573	0.196	0.549	1.12E-28	SMC2
IGHV1-2	6.63E-32	-0.0002985	0.21	0.484	1.33E-28	SMC2
CCL4	6.66E-32	0.82898972	0.771	0.995	1.33E-28	SMC2
PTX3	7.10E-32	-0.0077802	0.238	0.228	1.42E-28	SMC2
LTC4S	7.22E-32	0.10122842	0.102	0.48	1.44E-28	SMC2
NTRK2	7.44E-32	-0.0528733	0.373	0.264	1.49E-28	SMC2
VEGFA	1.14E-31	-0.0473089	0.45	0.358	2.29E-28	SMC2
IGFBP3	1.20E-31	0.6226647	0.12	0.462	2.41E-28	SMC2
FCRL5	1.62E-31	-0.0021921	0.477	0.729	3.24E-28	SMC2
OSM	2.27E-31	0.0012925	0.597	0.776	4.54E-28	SMC2
MKI67	2.55E-31	0.01620704	0.061	0.379	5.11E-28	SMC2
WWTR1	3.10E-31	0.2900322	0.562	0.839	6.19E-28	SMC2
ITPKC	3.35E-31	0.06364333	0.337	0.271	6.70E-28	SMC2
GZMK	3.46E-31	0.23917847	0.604	0.84	6.93E-28	SMC2
IL10	3.80E-31	0.04675238	0.714	0.828	7.60E-28	SMC2
CSRP1	4.09E-31	0.0287953	0.387	0.782	8.19E-28	SMC2
CTSC	4.23E-31	0.44503676	0.721	0.958	8.45E-28	SMC2
ECM1	4.40E-31	-0.1465706	0.479	0.35	8.81E-28	SMC2
SCIN	4.70E-31	0.02779416	0.262	0.539	9.40E-28	SMC2
KRT18	7.93E-31	-0.0267606	0.258	0.64	1.59E-27	SMC2
TSLP	9.06E-31	0.06575256	0.289	0.577	1.81E-27	SMC2

PKMYT1	9.54E-31	-0.0003172	0.18	0.415	1.91E-27	SMC2
RBP4	1.10E-30	-0.0337372	0.438	0.221	2.20E-27	SMC2
HIST1H1B	1.25E-30	0.00840309	0.636	0.794	2.51E-27	SMC2
PLXNA4	1.90E-30	0.01831441	0.208	0.547	3.81E-27	SMC2
CPA3	2.01E-30	-0.0909364	0.036	0.275	4.02E-27	SMC2
NMUR2	2.14E-30	-0.041213	0.11	0.122	4.28E-27	SMC2
NGF	3.44E-30	-0.0619585	0.301	0.237	6.89E-27	SMC2
LGALS3BP	3.79E-30	0.09284088	0.646	0.957	7.59E-27	SMC2
MGST2	4.21E-30	0.11017183	0.216	0.557	8.42E-27	SMC2
ABCA9	4.43E-30	-0.452569	0.179	0.575	8.86E-27	SMC2
MMP23B	5.07E-30	0.02188039	0.631	0.473	1.01E-26	SMC2
HSPB7	6.19E-30	0.02928537	0.534	0.461	1.24E-26	SMC2
APOBEC3A	6.49E-30	-0.0427674	0.121	0.411	1.30E-26	SMC2
CD3E	6.70E-30	0.1250135	0.62	0.893	1.34E-26	SMC2
KLRD1	7.02E-30	-0.0051798	0.672	0.889	1.40E-26	SMC2
IER5L	7.07E-30	0.11252835	0.377	0.707	1.41E-26	SMC2
KLHL23	1.02E-29	-0.0371218	0.53	0.385	2.04E-26	SMC2
HNMT	1.03E-29	0.10025372	0.486	0.822	2.05E-26	SMC2
SPP1	1.14E-29	0.6970323	0.665	0.935	2.28E-26	SMC2
RARRES1	1.21E-29	-0.309777	0.391	0.332	2.43E-26	SMC2
TTN	2.17E-29	0.08113737	0.267	0.629	4.34E-26	SMC2
LINC00520	2.20E-29	-0.0015219	0.668	0.846	4.40E-26	SMC2
EVI2B	2.35E-29	0.12094131	0.677	0.921	4.71E-26	SMC2
MXRA5	3.35E-29	0.05257466	0.748	0.958	6.69E-26	SMC2
SRGN	3.64E-29	0.44407913	0.238	0.575	7.29E-26	SMC2
ALDH1A1	5.44E-29	-0.2302702	0.346	0.243	1.09E-25	SMC2
AL078590.2	5.79E-29	-0.0137685	0.729	0.944	1.16E-25	SMC2
MAD2L1	6.42E-29	-0.017407	0.684	0.917	1.28E-25	SMC2
HYAL2	8.23E-29	-0.114261	0.6	0.898	1.65E-25	SMC2
BICC1	9.18E-29	-0.0985405	0.437	0.352	1.84E-25	SMC2
DNAH17	1.03E-28	0.0148131	0.001	0.358	2.07E-25	SMC2
SGK1	1.50E-28	0.03511585	0.62	0.843	3.00E-25	SMC2
UCP2	1.83E-28	0.02051399	0.71	0.878	3.66E-25	SMC2
MYCT1	2.73E-28	0.02119116	0.706	0.911	5.46E-25	SMC2
HSPB8	2.87E-28	-0.2373762	0.209	0.257	5.74E-25	SMC2
STC1	4.21E-28	-0.0308373	0.612	0.44	8.41E-25	SMC2
THEMIS2	4.69E-28	0.02250133	0.328	0.572	9.38E-25	SMC2
RDH10	6.37E-28	-0.1415863	0.186	0.237	1.27E-24	SMC2
SORBS2	6.54E-28	-0.0465016	0.284	0.629	1.31E-24	SMC2
FSTL3	6.63E-28	-0.2610452	0.146	0.152	1.33E-24	SMC2
MATN4	7.26E-28	-0.0969401	0.514	0.408	1.45E-24	SMC2
DNAJB4	9.57E-28	0.13479628	0.35	0.278	1.91E-24	SMC2
RAPGEF5	9.61E-28	0.23651158	0.434	0.738	1.92E-24	SMC2
ANKRD37	9.69E-28	0.10264596	0.546	0.848	1.94E-24	SMC2
POU3F4	9.91E-28	-0.0510658	0.146	0.356	1.98E-24	SMC2
HTRA3	1.10E-27	-0.2089694	0.398	0.314	2.20E-24	SMC2
AOX1	1.22E-27	-0.1150087	0.177	0.125	2.43E-24	SMC2
ART4	1.22E-27	0.27015251	0.53	0.393	2.44E-24	SMC2
LAMA4	1.27E-27	-0.0681869	0.722	0.97	2.53E-24	SMC2
IGLV1-51	1.32E-27	-0.0267974	0.145	0.049	2.65E-24	SMC2
IQCA1	1.38E-27	-0.0566559	0.68	0.806	2.76E-24	SMC2
POU2AF1	1.39E-27	-0.0087018	0.481	0.683	2.79E-24	SMC2
SERPINE1	1.67E-27	0.57795254	0.47	0.804	3.35E-24	SMC2

DIRC3	1.68E-27	-0.0284543	0.659	0.864	3.37E-24	SMC2
HIST1H1D	2.59E-27	0.00055248	0.406	0.687	5.17E-24	SMC2
TCEAL2	3.07E-27	0.14959236	0.123	0.104	6.14E-24	SMC2
PAIP2B	3.46E-27	0.01459322	0.588	0.768	6.92E-24	SMC2
IGHD	3.60E-27	0.0076884	0.353	0.622	7.19E-24	SMC2
ABCA8	4.81E-27	-0.3146716	0.392	0.676	9.62E-24	SMC2
RASSF6	5.39E-27	0.00335461	0.407	0.654	1.08E-23	SMC2
GSTM5	5.58E-27	-0.1723556	0.162	0.117	1.12E-23	SMC2
ACP5	6.31E-27	0.14821224	0.582	0.748	1.26E-23	SMC2
TNFRSF17	7.26E-27	-0.0169374	0.529	0.709	1.45E-23	SMC2
TROAP	7.52E-27	-0.0017831	0.213	0.199	1.50E-23	SMC2
B3GNT5	8.31E-27	0.0114692	0.551	0.778	1.66E-23	SMC2
C1QTNF4	8.87E-27	-0.0319445	0.171	0.136	1.77E-23	SMC2
FCRLA	1.05E-26	0.00534508	0.369	0.619	2.09E-23	SMC2
RRM2	1.15E-26	-0.0031376	0.132	0.402	2.30E-23	SMC2
VAMP8	1.24E-26	0.15816954	0.621	0.867	2.47E-23	SMC2
GALR2	1.24E-26	-0.0005689	0.246	0.495	2.48E-23	SMC2
RTKN2	1.45E-26	0.0175868	0.086	0.104	2.89E-23	SMC2
BASP1	1.64E-26	0.08559157	0.252	0.602	3.27E-23	SMC2
HAVCR2	1.76E-26	0.01396183	0.234	0.519	3.51E-23	SMC2
CLC	1.82E-26	0.01140265	0.18	0.413	3.65E-23	SMC2
DSP	1.93E-26	0.00068731	0.213	0.191	3.87E-23	SMC2
TPPP3	2.33E-26	0.06644451	0.463	0.367	4.66E-23	SMC2
TNFAIP6	3.04E-26	0.03363181	0.542	0.392	6.07E-23	SMC2
PLCXD3	3.72E-26	-0.0343063	0.421	0.702	7.43E-23	SMC2
IGHV2-5	4.34E-26	-0.0004398	0.48	0.695	8.68E-23	SMC2
PMCH	5.25E-26	-3.40E-05	0.243	0.476	1.05E-22	SMC2
KLRC1	5.51E-26	-0.0213563	0.731	0.904	1.10E-22	SMC2
IGHG3	8.01E-26	0.17072018	0.764	0.949	1.60E-22	SMC2
HOPX	8.23E-26	0.03825471	0.35	0.713	1.65E-22	SMC2
TNFRSF12A	8.47E-26	0.14081427	0.348	0.31	1.69E-22	SMC2
BCL2A1	1.05E-25	0.07373726	0.146	0.491	2.11E-22	SMC2
CD38	1.65E-25	0.00840223	0.25	0.221	3.29E-22	SMC2
MFSD1	1.69E-25	-0.0520277	0.152	0.444	3.38E-22	SMC2
PGF	2.40E-25	-0.0121476	0.726	0.503	4.81E-22	SMC2
CD2	2.74E-25	0.21173698	0.656	0.859	5.48E-22	SMC2
HIST1H2AJ	3.03E-25	1.14E-06	0.011	0.119	6.06E-22	SMC2
ACE	3.18E-25	-0.0785969	0.287	0.584	6.36E-22	SMC2
CEMIP	3.36E-25	0.00185126	0.315	0.565	6.71E-22	SMC2
PRRX2	3.48E-25	0.09384849	0.389	0.374	6.96E-22	SMC2
IL4I1	3.89E-25	0.00021197	0.038	0.285	7.78E-22	SMC2
KLRB1	4.50E-25	0.14953906	0.589	0.87	9.01E-22	SMC2
ROBO4	4.92E-25	-0.0227009	0.627	0.481	9.83E-22	SMC2
KPNA2	5.27E-25	0.00160695	0.514	0.817	1.05E-21	SMC2
SLAMF1	5.83E-25	0.04815548	0.65	0.831	1.17E-21	SMC2
CCR6	6.75E-25	0.01821107	0.624	0.744	1.35E-21	SMC2
TMEM47	7.84E-25	0.31847106	0.155	0.44	1.57E-21	SMC2
SERPINA1	8.16E-25	0.02825631	0.387	0.595	1.63E-21	SMC2
MRC1	8.92E-25	0.02244093	0.713	0.879	1.78E-21	SMC2
TRBV28	1.00E-24	0.00404679	0.065	0.117	2.01E-21	SMC2
TENT5C	1.46E-24	0.00725628	0.341	0.652	2.92E-21	SMC2
GAPT	1.69E-24	0.0128335	0.108	0.356	3.38E-21	SMC2
HLA-DRB5	2.05E-24	0.21740357	0.671	0.856	4.10E-21	SMC2

CLMP	2.06E-24	-0.0449334	0.373	0.329	4.11E-21	SMC2
PLAC9	2.22E-24	0.38773388	0.53	0.397	4.44E-21	SMC2
AL157895.1	2.23E-24	-0.0126027	0.533	0.309	4.45E-21	SMC2
MGLL	2.70E-24	-0.0767244	0.308	0.28	5.40E-21	SMC2
LRRC25	3.26E-24	-0.0047123	0.39	0.627	6.51E-21	SMC2
ITIH5	3.77E-24	0.08021862	0.424	0.386	7.53E-21	SMC2
ANGPTL4	4.00E-24	-0.1066246	0.729	0.602	8.00E-21	SMC2
LGALS9	4.50E-24	0.06563572	0.716	0.869	8.99E-21	SMC2
STMN1	4.58E-24	0.10959368	0.274	0.566	9.16E-21	SMC2
KDELR3	4.80E-24	0.11499206	0.656	0.882	9.59E-21	SMC2
BCAM	6.22E-24	0.03116257	0.346	0.344	1.24E-20	SMC2
GRIA2	6.83E-24	0.23828504	0.141	0.428	1.37E-20	SMC2
BEX2	9.72E-24	-0.0053487	0.338	0.272	1.94E-20	SMC2
PRDX4	1.06E-23	0.10001465	0.62	0.894	2.11E-20	SMC2
ICAM1	1.15E-23	-0.0472635	0.494	0.336	2.30E-20	SMC2
CLDN5	1.16E-23	0.04065343	0.577	0.744	2.32E-20	SMC2
GREM1	1.36E-23	-0.0102274	0.509	0.301	2.72E-20	SMC2
IGHV6-1	1.43E-23	0.00145608	0.272	0.528	2.85E-20	SMC2
SPARCL1	1.52E-23	0.77650713	0.694	0.512	3.03E-20	SMC2
BMP2	1.58E-23	0.03184456	0.424	0.356	3.15E-20	SMC2
CNTNAP3B	1.92E-23	-0.0467537	0.154	0.207	3.84E-20	SMC2
S100P	2.22E-23	-0.0672931	0.341	0.603	4.43E-20	SMC2
KIR2DL4	2.93E-23	-1.10E-07	0.013	0.111	5.87E-20	SMC2
PLAT	3.16E-23	-0.2945088	0.575	0.472	6.32E-20	SMC2
PAPSS2	3.50E-23	0.03550771	0.471	0.329	6.99E-20	SMC2
ZNF385D	4.66E-23	0.29940916	0.445	0.752	9.33E-20	SMC2
CD40	4.94E-23	0.00093535	0.207	0.519	9.89E-20	SMC2
TFEC	5.79E-23	0.0275305	0.582	0.73	1.16E-19	SMC2
NCAPH	5.90E-23	-0.0133216	0.53	0.759	1.18E-19	SMC2
FPR1	6.00E-23	-0.0172561	0.053	0.251	1.20E-19	SMC2
SOX17	6.81E-23	-0.0073419	0.697	0.893	1.36E-19	SMC2
GAL	7.51E-23	-0.001294	0.245	0.459	1.50E-19	SMC2
IGKV3-20	7.89E-23	-0.0042874	0.735	0.957	1.58E-19	SMC2
TGFBI	7.97E-23	0.31113752	0.651	0.909	1.59E-19	SMC2
NRXN1	1.08E-22	-0.1231168	0.251	0.493	2.15E-19	SMC2
MND1	1.23E-22	0.00739485	0.363	0.331	2.47E-19	SMC2
KCNAB1	1.42E-22	0.02038817	0.372	0.678	2.84E-19	SMC2
FCGRT	1.80E-22	0.09883044	0.726	0.871	3.59E-19	SMC2
CA2	2.43E-22	0.01758105	0.669	0.798	4.86E-19	SMC2
CD33	2.54E-22	0.00434346	0.107	0.392	5.09E-19	SMC2
PODXL	2.78E-22	0.0307167	0.584	0.757	5.57E-19	SMC2
SDF2L1	3.18E-22	0.12615692	0.613	0.871	6.35E-19	SMC2
LGMN	3.24E-22	0.13546743	0.299	0.634	6.49E-19	SMC2
NLRP7	3.25E-22	0.00073533	0.245	0.484	6.50E-19	SMC2
KLRF1	3.40E-22	-0.0042918	0.732	0.939	6.79E-19	SMC2
MYOC	4.10E-22	-0.0161472	0.232	0.195	8.20E-19	SMC2
DKK2	4.38E-22	0.02548335	0.018	0.28	8.75E-19	SMC2
RASL11A	4.40E-22	0.02876511	0.49	0.472	8.80E-19	SMC2
COL10A1	5.33E-22	0.00117644	0.219	0.457	1.07E-18	SMC2
LTBP2	5.65E-22	0.39432768	0.437	0.806	1.13E-18	SMC2
CXCL14	6.88E-22	-0.7722705	0.382	0.335	1.38E-18	SMC2
ORC6	7.65E-22	-0.0152753	0.547	0.711	1.53E-18	SMC2
CCDC3	9.30E-22	0.28535804	0.348	0.691	1.86E-18	SMC2

RERG	9.56E-22	0.22117818	0.39	0.692	1.91E-18	SMC2
KIF14	9.73E-22	-0.0174507	0.1	0.339	1.95E-18	SMC2
IGKV1D-8	1.05E-21	0.00220281	0.245	0.484	2.10E-18	SMC2
PDCD1	1.10E-21	0.00950238	0.688	0.814	2.20E-18	SMC2
PCOLCE2	1.12E-21	-0.0203169	0.214	0.641	2.24E-18	SMC2
JSRP1	1.17E-21	-0.0036619	0.574	0.741	2.35E-18	SMC2
ANPEP	1.23E-21	-0.0309494	0.618	0.576	2.46E-18	SMC2
STMN2	1.37E-21	-0.0016338	0.31	0.569	2.75E-18	SMC2
HES5	1.38E-21	-0.0216112	0.224	0.245	2.75E-18	SMC2
FMO2	1.38E-21	0.42290524	0.608	0.928	2.76E-18	SMC2
IGHV3-20	1.42E-21	-0.0017497	0.494	0.652	2.84E-18	SMC2
GRASP	1.48E-21	0.02613605	0.608	0.74	2.95E-18	SMC2
FAM43A	1.60E-21	-0.0183801	0.38	0.374	3.20E-18	SMC2
GATA2	1.64E-21	-0.0193192	0.605	0.881	3.29E-18	SMC2
IRF4	1.71E-21	-0.0176679	0.669	0.767	3.42E-18	SMC2
PPP1R14A	1.80E-21	0.16401409	0.303	0.63	3.59E-18	SMC2
IGHV1-69	2.16E-21	-5.82E-06	0.242	0.477	4.31E-18	SMC2
UBE2T	2.25E-21	0.02455857	0.445	0.646	4.50E-18	SMC2
DNAJB1	2.29E-21	-0.4134627	0.665	0.873	4.59E-18	SMC2
LINC01094	2.47E-21	0.00084641	0.092	0.339	4.93E-18	SMC2
DERL3	2.63E-21	0.05687962	0.685	0.871	5.27E-18	SMC2
IGHV3-21	2.72E-21	-1.98E-05	0.247	0.482	5.43E-18	SMC2
IGHV3-48	2.96E-21	-7.47E-06	0.242	0.478	5.93E-18	SMC2
IGKV3D-11	3.09E-21	-5.24E-06	0.242	0.478	6.19E-18	SMC2
THSD7A	3.47E-21	0.00608553	0.509	0.713	6.94E-18	SMC2
NKG7	3.89E-21	0.19358456	0.216	0.558	7.78E-18	SMC2
TUBA1C	4.10E-21	0.26981578	0.664	0.867	8.19E-18	SMC2
TSPAN15	4.38E-21	0.00657817	0.263	0.489	8.77E-18	SMC2
TNFRSF18	4.48E-21	0.0099472	0.733	0.963	8.96E-18	SMC2
IGKV1-17	4.56E-21	-0.0001898	0.347	0.606	9.13E-18	SMC2
IGLV5-45	4.80E-21	-3.88E-06	0.242	0.478	9.59E-18	SMC2
UAP1	4.91E-21	-0.2570435	0.574	0.722	9.83E-18	SMC2
RAB31	5.09E-21	-0.0398734	0.502	0.779	1.02E-17	SMC2
IGHV4-31	5.12E-21	-1.05E-05	0.242	0.478	1.02E-17	SMC2
LAMA2	5.14E-21	-0.1023138	0.605	0.859	1.03E-17	SMC2
QPCT	5.47E-21	0.00358321	0.501	0.688	1.09E-17	SMC2
IGLV7-43	5.78E-21	-4.27E-07	0.242	0.478	1.16E-17	SMC2
MRAS	6.28E-21	-0.003874	0.276	0.274	1.26E-17	SMC2
S100A12	6.82E-21	-0.0781232	0.136	0.076	1.36E-17	SMC2
IGHV4-4	6.83E-21	0.0166647	0.241	0.476	1.37E-17	SMC2
ISG20	7.19E-21	0.12199928	0.675	0.923	1.44E-17	SMC2
HPGDS	7.68E-21	-0.0266828	0.692	0.858	1.54E-17	SMC2
TSPAN13	7.73E-21	-0.0313433	0.4	0.304	1.55E-17	SMC2
FFAR3	7.92E-21	0.00211919	0.732	0.925	1.58E-17	SMC2
IGKV1-39	8.52E-21	-9.30E-05	0.408	0.673	1.70E-17	SMC2
PTTG1	9.12E-21	0.04225768	0.612	0.812	1.82E-17	SMC2
SMPDL3A	9.68E-21	-0.0825333	0.182	0.481	1.94E-17	SMC2
IGLV2-23	1.00E-20	-4.24E-05	0.238	0.474	2.01E-17	SMC2
IGLV5-48	1.20E-20	-1.89E-06	0.242	0.477	2.40E-17	SMC2
IGKV1-27	1.31E-20	-7.65E-06	0.242	0.476	2.63E-17	SMC2
TSC22D1	1.51E-20	0.46108368	0.613	0.927	3.02E-17	SMC2
FAM180B	1.53E-20	-0.1901523	0.227	0.247	3.06E-17	SMC2
PTGS1	1.69E-20	-0.002467	0.646	0.737	3.39E-17	SMC2

DKK3	2.19E-20	0.43264221	0.47	0.744	4.39E-17	SMC2
OVOL3	2.24E-20	-1.83E-06	0.242	0.477	4.47E-17	SMC2
ENTPD3	2.48E-20	-0.0164001	0.604	0.808	4.96E-17	SMC2
CD248	2.51E-20	-0.381463	0.38	0.29	5.03E-17	SMC2
TCHH	2.64E-20	-0.0183458	0.478	0.602	5.29E-17	SMC2
PYCR1	2.80E-20	0.03126965	0.373	0.573	5.61E-17	SMC2
NINJ1	2.97E-20	0.21735699	0.193	0.488	5.94E-17	SMC2
CD74	3.12E-20	0.29574182	0.693	0.9	6.25E-17	SMC2
IGHV1-18	3.63E-20	1.37E-05	0.287	0.505	7.26E-17	SMC2
TOP2A	3.82E-20	0.0004909	0.701	0.851	7.64E-17	SMC2
SSPN	3.87E-20	0.17934832	0.42	0.753	7.75E-17	SMC2
AC104024.1	3.94E-20	0.00269855	0.263	0.495	7.89E-17	SMC2
FBLN2	3.97E-20	-0.308182	0.35	0.669	7.93E-17	SMC2
GRHL2	4.06E-20	0.03722346	0.11	0.362	8.12E-17	SMC2
CALB2	4.58E-20	-0.0025511	0.034	0.182	9.16E-17	SMC2
NRG1	4.74E-20	-0.0270806	0.111	0.359	9.48E-17	SMC2
NQO1	5.68E-20	-0.0864834	0.718	0.93	1.14E-16	SMC2
HBEGF	6.09E-20	0.03235833	0.414	0.717	1.22E-16	SMC2
PTGER2	6.68E-20	0.01805018	0.592	0.755	1.34E-16	SMC2
IFI6	7.98E-20	0.26100639	0.683	0.867	1.60E-16	SMC2
ELL2	8.29E-20	0.19615935	0.248	0.279	1.66E-16	SMC2
IL17B	9.95E-20	0.00182558	0.274	0.516	1.99E-16	SMC2
TSPAN33	1.25E-19	0.00209966	0.645	0.829	2.49E-16	SMC2
AC026369.3	1.37E-19	-8.91E-06	0.224	0.458	2.75E-16	SMC2
MYL2	1.42E-19	0.00140086	0.241	0.473	2.84E-16	SMC2
XCL2	1.45E-19	0.05836491	0.574	0.533	2.90E-16	SMC2
CLEC14A	1.67E-19	-0.0225984	0.58	0.813	3.35E-16	SMC2
FCGR1A	1.73E-19	0.00377151	0.396	0.631	3.46E-16	SMC2
BST1	1.80E-19	-0.0808394	0.301	0.201	3.59E-16	SMC2
FOXS1	1.96E-19	-0.0039229	0.597	0.476	3.91E-16	SMC2
NFKBIA	1.99E-19	0.0603095	0.797	0.846	3.98E-16	SMC2
IGHV3-7	2.48E-19	0.00586617	0.262	0.495	4.95E-16	SMC2
ALCAM	3.21E-19	0.38844255	0.239	0.556	6.42E-16	SMC2
TMEM88	3.25E-19	0.0067981	0.718	0.797	6.50E-16	SMC2
AVPR1A	3.69E-19	-0.1284826	0.556	0.74	7.38E-16	SMC2
TMEM70	4.13E-19	0.16941762	0.584	0.488	8.26E-16	SMC2
IGFBP1	4.26E-19	-0.0001244	0.152	0.32	8.52E-16	SMC2
BCHE	5.28E-19	-0.0926837	0.206	0.248	1.06E-15	SMC2
ADGRF5	6.24E-19	0.19608482	0.745	0.991	1.25E-15	SMC2
AC133644.2	6.32E-19	-0.001703	0.722	0.809	1.26E-15	SMC2
CKB	6.56E-19	-0.4571745	0.537	0.485	1.31E-15	SMC2
EPB41L3	7.86E-19	-0.1609935	0.105	0.454	1.57E-15	SMC2
CXCL16	8.02E-19	0.06425552	0.253	0.524	1.60E-15	SMC2
SPINK5	8.97E-19	0.00213081	0.124	0.298	1.79E-15	SMC2
CD37	9.29E-19	0.23637418	0.343	0.626	1.86E-15	SMC2
SLC25A4	9.64E-19	0.11764066	0.549	0.808	1.93E-15	SMC2
DPYSL3	9.95E-19	-0.0687061	0.344	0.606	1.99E-15	SMC2
NUAK1	1.02E-18	0.06166049	0.25	0.583	2.05E-15	SMC2
OSR1	1.09E-18	0.02320731	0.221	0.241	2.18E-15	SMC2
HES4	1.14E-18	0.08670147	0.49	0.377	2.27E-15	SMC2
ANGPTL1	1.24E-18	0.05638467	0.324	0.626	2.48E-15	SMC2
ABCC9	1.25E-18	-0.0765672	0.25	0.215	2.50E-15	SMC2
SLC31A2	1.41E-18	-0.0059454	0.175	0.519	2.81E-15	SMC2

COX4I2	1.45E-18	0.05036284	0.497	0.436	2.90E-15	SMC2
SLC16A10	1.52E-18	0.02196065	0.493	0.347	3.05E-15	SMC2
IGHV3-74	1.67E-18	0.0008396	0.26	0.463	3.34E-15	SMC2
WFDC2	1.83E-18	-0.0042567	0.293	0.211	3.66E-15	SMC2
RAB32	2.04E-18	0.10145204	0.182	0.472	4.09E-15	SMC2
FBXL22	2.15E-18	0.11848072	0.429	0.744	4.30E-15	SMC2
SLPI	2.21E-18	0.16043756	0.694	0.561	4.43E-15	SMC2
F13A1	2.43E-18	0.15019442	0.488	0.646	4.86E-15	SMC2
KCNK17	2.50E-18	-0.035099	0.401	0.356	5.00E-15	SMC2
SOCS1	2.82E-18	-0.0231476	0.693	0.905	5.64E-15	SMC2
SFRP1	2.83E-18	-0.6609426	0.524	0.415	5.67E-15	SMC2
IGHV1-46	3.14E-18	-0.0001447	0.305	0.519	6.28E-15	SMC2
AK6	3.21E-18	0.16595203	0.411	0.366	6.42E-15	SMC2
C2	3.41E-18	-0.0027088	0.583	0.835	6.82E-15	SMC2
FUCA1	3.90E-18	0.07675934	0.422	0.603	7.81E-15	SMC2
TNFAIP2	3.99E-18	-0.1204919	0.596	0.558	7.98E-15	SMC2
FLT3	4.37E-18	-8.75E-05	0.282	0.524	8.74E-15	SMC2
CXCL3	4.52E-18	0.13005222	0.058	0.397	9.03E-15	SMC2
RGS2	7.24E-18	0.26706937	0.418	0.711	1.45E-14	SMC2
BCL2L14	7.47E-18	-3.50E-05	0.253	0.491	1.49E-14	SMC2
NES	8.39E-18	-0.016798	0.487	0.763	1.68E-14	SMC2
HS3ST1	9.05E-18	0.00869617	0.231	0.176	1.81E-14	SMC2
ASS1	1.01E-17	0.07205342	0.589	0.499	2.03E-14	SMC2
CHIT1	1.02E-17	-1.07E-06	0.24	0.465	2.05E-14	SMC2
GPRC5C	1.04E-17	0.27218838	0.473	0.789	2.08E-14	SMC2
SMPX	1.07E-17	-0.0138952	0.439	0.328	2.14E-14	SMC2
SLC40A1	1.19E-17	0.18016695	0.377	0.706	2.38E-14	SMC2
IGHV4-28	1.20E-17	2.42E-05	0.259	0.488	2.40E-14	SMC2
FBXO32	1.39E-17	0.34941581	0.363	0.705	2.77E-14	SMC2
CCDC144A	1.42E-17	-0.0885895	0.365	0.348	2.84E-14	SMC2
MDK	1.48E-17	0.16455211	0.667	0.855	2.96E-14	SMC2
CHI3L1	1.59E-17	-0.0051259	0.485	0.71	3.18E-14	SMC2
KCTD12	1.69E-17	0.11427064	0.681	0.762	3.37E-14	SMC2
CCL26	1.69E-17	0.07926142	0.721	0.89	3.38E-14	SMC2
OLFM1	1.76E-17	-0.0194781	0.442	0.333	3.52E-14	SMC2
PHLDA1	1.82E-17	0.23717306	0.613	0.779	3.64E-14	SMC2
MX1	2.18E-17	0.23312776	0.703	0.898	4.37E-14	SMC2
IGHV4-34	2.19E-17	-0.0114292	0.144	0.4	4.37E-14	SMC2
ERRFI1	2.55E-17	0.03977589	0.452	0.43	5.10E-14	SMC2
GNA15	2.97E-17	0.0209812	0.032	0.102	5.94E-14	SMC2
IGLC3	3.46E-17	0.18989147	0.799	0.911	6.92E-14	SMC2
POU2F2	3.51E-17	-0.0887834	0.326	0.619	7.01E-14	SMC2
AGR2	3.51E-17	-0.002744	0.379	0.598	7.03E-14	SMC2
HES1	3.72E-17	0.03820414	0.609	0.523	7.44E-14	SMC2
ARC	3.80E-17	0.00993146	0.694	0.947	7.61E-14	SMC2
IGLV2-8	4.07E-17	-0.0004272	0.315	0.515	8.13E-14	SMC2
FMO3	4.16E-17	0.03066148	0.637	0.821	8.32E-14	SMC2
PLPP3	4.34E-17	-0.2663499	0.297	0.256	8.67E-14	SMC2
RASD1	4.42E-17	-0.0757171	0.553	0.489	8.84E-14	SMC2
SLC39A8	4.76E-17	-0.0477841	0.509	0.633	9.52E-14	SMC2
GABRD	4.80E-17	-0.0117791	0.245	0.206	9.60E-14	SMC2
CCL21	4.81E-17	0.81932941	0.739	0.946	9.62E-14	SMC2
VIT	4.98E-17	-0.3323952	0.256	0.255	9.97E-14	SMC2

PPA1	6.07E-17	0.12537907	0.7	0.877	1.21E-13	SMC2
TK1	6.30E-17	0.01468487	0.374	0.584	1.26E-13	SMC2
AL590226.1	6.75E-17	-0.0003774	0.652	0.799	1.35E-13	SMC2
FCRL2	7.17E-17	-0.0001937	0.35	0.557	1.43E-13	SMC2
PLAC8	9.06E-17	0.02225335	0.372	0.623	1.81E-13	SMC2
TIMP4	9.39E-17	0.01868051	0.701	0.539	1.88E-13	SMC2
CLEC5A	9.83E-17	-0.0050935	0.084	0.369	1.97E-13	SMC2
KIR2DL1	1.14E-16	-0.0005132	0.19	0.393	2.28E-13	SMC2
GALNT15	1.68E-16	-0.2825834	0.136	0.129	3.37E-13	SMC2
PLAUR	1.90E-16	0.0014697	0.223	0.535	3.81E-13	SMC2
MGP	2.11E-16	0.87858395	0.942	0.951	4.22E-13	SMC2
THBD	2.15E-16	0.24336168	0.471	0.738	4.30E-13	SMC2
TNFAIP3	2.31E-16	0.15289514	0.724	0.84	4.61E-13	SMC2
FAM180A	2.43E-16	0.04954021	0.488	0.378	4.85E-13	SMC2
FABP5	2.48E-16	0.33324243	0.59	0.804	4.95E-13	SMC2
CHRDL1	2.66E-16	-0.3158569	0.212	0.214	5.32E-13	SMC2
INSR	2.79E-16	0.02256697	0.191	0.206	5.59E-13	SMC2
NPW	3.42E-16	-0.0062879	0.138	0.16	6.84E-13	SMC2
CYTOR	3.56E-16	-0.1363822	0.709	0.9	7.12E-13	SMC2
TNFRSF13C	3.72E-16	0.01373963	0.438	0.575	7.43E-13	SMC2
ALDH1A3	3.93E-16	-0.0102678	0.489	0.608	7.85E-13	SMC2
CDA	3.98E-16	-0.0086757	0.606	0.709	7.97E-13	SMC2
FFAR2	4.11E-16	-0.0106342	0.119	0.169	8.23E-13	SMC2
SLIT3	4.45E-16	-0.4659643	0.514	0.459	8.91E-13	SMC2
HAMP	4.48E-16	0.00314174	0.17	0.382	8.96E-13	SMC2
CHCHD6	4.87E-16	0.00387873	0.255	0.484	9.73E-13	SMC2
MAMDC2	5.24E-16	0.14325353	0.537	0.463	1.05E-12	SMC2
CAMK2N1	6.04E-16	0.06660023	0.481	0.503	1.21E-12	SMC2
LRRC39	7.04E-16	0.01115022	0.229	0.287	1.41E-12	SMC2
S100A8	7.08E-16	-0.4773399	0.466	0.659	1.42E-12	SMC2
IGFBP2	7.75E-16	0.82762778	0.422	0.799	1.55E-12	SMC2
BCL11A	8.21E-16	0.01414565	0.646	0.748	1.64E-12	SMC2
LPL	8.27E-16	0.05891081	0.55	0.73	1.65E-12	SMC2
LINC01615	1.05E-15	-0.0138695	0.744	0.999	2.10E-12	SMC2
GK	1.12E-15	-0.0979615	0.574	0.717	2.25E-12	SMC2
CD40LG	1.22E-15	-0.0144057	0.185	0.474	2.45E-12	SMC2
AKR1C1	1.26E-15	0.0231058	0.576	0.523	2.51E-12	SMC2
XCR1	1.34E-15	-1.72E-05	0.226	0.454	2.68E-12	SMC2
ADAMTS6	1.44E-15	-0.023431	0.411	0.614	2.89E-12	SMC2
PTN	1.45E-15	0.09820732	0.633	0.79	2.91E-12	SMC2
KNL1	1.52E-15	-0.0105231	0.315	0.523	3.03E-12	SMC2
TACSTD2	1.54E-15	0.00828027	0.12	0.115	3.08E-12	SMC2
NUCB2	1.68E-15	0.23258705	0.429	0.722	3.36E-12	SMC2
NDNF	1.68E-15	-0.0324251	0.421	0.385	3.36E-12	SMC2
USP2	1.73E-15	-0.0007662	0.153	0.199	3.45E-12	SMC2
ID3	1.76E-15	0.28065383	0.481	0.45	3.51E-12	SMC2
IGFBP7	1.79E-15	0.83202966	0.916	0.879	3.58E-12	SMC2
NUDT4	1.83E-15	0.13062067	0.574	0.768	3.66E-12	SMC2
ATP1B1	1.84E-15	-0.0468874	0.52	0.729	3.68E-12	SMC2
VWA1	1.84E-15	-0.0034546	0.554	0.728	3.69E-12	SMC2
RHEX	1.95E-15	-0.0211084	0.403	0.671	3.90E-12	SMC2
RGS10	2.08E-15	0.07315514	0.291	0.599	4.16E-12	SMC2
AC103591.3	2.13E-15	0.02927919	0.289	0.282	4.26E-12	SMC2

FNDC1	2.22E-15	0.01302151	0.364	0.528	4.43E-12	SMC2
CLIC3	2.22E-15	0.11707915	0.424	0.364	4.45E-12	SMC2
IGFBP4	2.27E-15	-0.0142999	0.626	0.791	4.55E-12	SMC2
FEN1	2.32E-15	0.02616627	0.544	0.747	4.64E-12	SMC2
TRGC1	2.65E-15	0.00077115	0.229	0.447	5.30E-12	SMC2
AC097375.1	2.72E-15	1.13E-06	0.234	0.455	5.43E-12	SMC2
IDO1	3.38E-15	0.00882738	0.303	0.558	6.75E-12	SMC2
SPON2	3.49E-15	-0.1179912	0.278	0.298	6.99E-12	SMC2
DLX6	3.69E-15	-0.0454204	0.278	0.156	7.39E-12	SMC2
RAB20	3.94E-15	0.08263573	0.439	0.626	7.89E-12	SMC2
SUCNR1	4.05E-15	-0.1710261	0.315	0.267	8.09E-12	SMC2
SGCA	4.14E-15	0.23813378	0.657	0.919	8.28E-12	SMC2
BGN	4.36E-15	0.83298615	0.61	0.53	8.72E-12	SMC2
CCDC80	4.98E-15	-0.2290961	0.708	0.843	9.97E-12	SMC2
IL3RA	5.07E-15	0.01345629	0.23	0.443	1.01E-11	SMC2
ITGAX	5.26E-15	0.0259119	0.066	0.383	1.05E-11	SMC2
SHTN1	5.36E-15	-0.0590909	0.343	0.619	1.07E-11	SMC2
CD200	5.37E-15	0.15840371	0.393	0.673	1.07E-11	SMC2
TRAC	5.99E-15	0.21044294	0.548	0.87	1.20E-11	SMC2
RND1	6.24E-15	0.01204699	0.317	0.335	1.25E-11	SMC2
IGLL5	6.35E-15	-0.0206124	0.328	0.329	1.27E-11	SMC2
CCL20	6.35E-15	0.0614267	0.364	0.584	1.27E-11	SMC2
LYPD2	6.36E-15	-0.0011771	0.03	0.274	1.27E-11	SMC2
IGHA1	6.75E-15	0.27012605	0.827	0.988	1.35E-11	SMC2
CDKN2A	7.16E-15	0.08174909	0.673	0.829	1.43E-11	SMC2
PAK1	7.70E-15	0.07286011	0.445	0.375	1.54E-11	SMC2
TM4SF18	7.95E-15	0.00332005	0.54	0.718	1.59E-11	SMC2
LINC02345	8.08E-15	0.002465	0.113	0.252	1.62E-11	SMC2
CLEC3B	8.18E-15	-0.1264678	0.609	0.518	1.64E-11	SMC2
SOD2	8.51E-15	0.19816029	0.731	0.84	1.70E-11	SMC2
CFP	8.85E-15	0.01654875	0.718	0.776	1.77E-11	SMC2
IFNG	9.01E-15	-0.0220728	0.699	0.87	1.80E-11	SMC2
SFTA1P	9.92E-15	0.03766279	0.574	0.47	1.98E-11	SMC2
EPB41L2	1.02E-14	0.05367021	0.567	0.478	2.05E-11	SMC2
BID	1.10E-14	0.05204557	0.295	0.495	2.19E-11	SMC2
MELK	1.12E-14	-0.0016373	0.24	0.461	2.24E-11	SMC2
AP003481.1	1.13E-14	0.010029	0.413	0.301	2.26E-11	SMC2
IL2RB	1.19E-14	0.01002886	0.243	0.518	2.38E-11	SMC2
FXYD6	1.30E-14	0.07104928	0.494	0.44	2.60E-11	SMC2
GUCY1A2	1.72E-14	0.02860211	0.733	0.883	3.44E-11	SMC2
NT5DC2	1.75E-14	-0.0044885	0.613	0.894	3.50E-11	SMC2
HMGB2	1.96E-14	0.11080112	0.312	0.581	3.92E-11	SMC2
LINC01857	2.15E-14	0.00404017	0.307	0.497	4.31E-11	SMC2
CALCRL	2.26E-14	0.20730585	0.755	0.989	4.52E-11	SMC2
EDNRA	2.27E-14	0.12810534	0.605	0.642	4.54E-11	SMC2
FCGR2A	2.35E-14	0.05837733	0.115	0.379	4.69E-11	SMC2
FKBP11	2.37E-14	-0.0980395	0.571	0.793	4.73E-11	SMC2
CSTB	2.44E-14	0.17503539	0.409	0.691	4.89E-11	SMC2
TM4SF1	2.59E-14	0.28241868	0.501	0.709	5.17E-11	SMC2
LINC02544	2.64E-14	0.02369093	0.318	0.27	5.28E-11	SMC2
BUB1	2.84E-14	4.21E-05	0.372	0.332	5.67E-11	SMC2
CD36	3.09E-14	0.11991507	0.555	0.698	6.18E-11	SMC2
ANXA3	3.94E-14	-0.0243785	0.236	0.26	7.87E-11	SMC2

JAML	4.09E-14	0.01232573	0.066	0.275	8.18E-11	SMC2
PLPP1	4.09E-14	0.27545225	0.444	0.398	8.18E-11	SMC2
P4HA2	4.40E-14	-0.0280521	0.594	0.537	8.80E-11	SMC2
IL34	5.02E-14	0.03630847	0.497	0.752	1.00E-10	SMC2
IL6	6.25E-14	-0.0247041	0.604	0.561	1.25E-10	SMC2
TIGIT	6.51E-14	-0.0065156	0.569	0.743	1.30E-10	SMC2
CD300LG	7.10E-14	-0.0001414	0.123	0.26	1.42E-10	SMC2
BMP3	7.94E-14	-0.0066062	0.38	0.375	1.59E-10	SMC2
CD1E	8.28E-14	-8.50E-05	0.258	0.446	1.66E-10	SMC2
KRT5	9.22E-14	0.00432539	0.108	0.238	1.84E-10	SMC2
SCT	9.57E-14	-0.0071943	0.452	0.34	1.91E-10	SMC2
C5AR2	1.03E-13	-0.0283108	0.624	0.48	2.06E-10	SMC2
MMP13	1.13E-13	0.09577029	0.252	0.423	2.25E-10	SMC2
FAM110D	1.18E-13	0.00647601	0.657	0.79	2.37E-10	SMC2
ACSL1	1.24E-13	0.01239068	0.147	0.398	2.47E-10	SMC2
PCK1	1.28E-13	-0.0304663	0.398	0.367	2.56E-10	SMC2
PLVAP	1.38E-13	-0.0725336	0.558	0.795	2.76E-10	SMC2
TPSAB1	1.44E-13	-0.1124646	0.443	0.602	2.88E-10	SMC2
MIR4435-2H	1.44E-13	0.03271062	0.722	0.653	2.89E-10	SMC2
ACTB	1.78E-13	0.60285329	0.75	0.718	3.56E-10	SMC2
C3	1.82E-13	-1.0172874	0.554	0.412	3.65E-10	SMC2
EMP1	1.83E-13	-0.3229967	0.551	0.721	3.65E-10	SMC2
SPI1	2.15E-13	0.09858784	0.162	0.333	4.31E-10	SMC2
DEPDC1B	2.31E-13	-0.0015836	0.178	0.424	4.62E-10	SMC2
GJA5	2.60E-13	-0.0241711	0.321	0.243	5.21E-10	SMC2
IRF1	2.61E-13	-0.1705943	0.589	0.744	5.22E-10	SMC2
CSF3R	2.71E-13	-0.0715364	0.206	0.187	5.42E-10	SMC2
LYVE1	2.73E-13	0.00163173	0.446	0.335	5.46E-10	SMC2
IGHV5-51	2.82E-13	-0.0096521	0.164	0.369	5.64E-10	SMC2
TNFSF10	2.98E-13	-0.0864406	0.498	0.753	5.97E-10	SMC2
ANGPT2	3.01E-13	0.43353437	0.452	0.706	6.02E-10	SMC2
BCAT1	3.02E-13	0.04669551	0.605	0.854	6.05E-10	SMC2
MYO1B	3.13E-13	0.19686852	0.604	0.831	6.26E-10	SMC2
ANXA2	3.31E-13	0.32505959	0.53	0.497	6.63E-10	SMC2
LAP3	3.68E-13	0.26708188	0.506	0.47	7.37E-10	SMC2
HIST1H2AL	3.95E-13	0.00043846	0.499	0.652	7.89E-10	SMC2
CXCL1	4.13E-13	0.08205905	0.632	0.467	8.26E-10	SMC2
TINAGL1	6.34E-13	0.45473282	0.575	0.528	1.27E-09	SMC2
VMO1	6.49E-13	0.05504723	0.641	0.726	1.30E-09	SMC2
ERN1	6.97E-13	0.05982201	0.215	0.317	1.39E-09	SMC2
LINC00309	7.91E-13	0.01071573	0.695	0.89	1.58E-09	SMC2
PPARG	8.19E-13	0.02892014	0.242	0.29	1.64E-09	SMC2
TESC	8.25E-13	-0.0075242	0.278	0.264	1.65E-09	SMC2
ANGPT1	8.30E-13	0.5759805	0.43	0.724	1.66E-09	SMC2
EPSTI1	8.98E-13	0.08065591	0.3	0.56	1.80E-09	SMC2
PLD3	9.34E-13	0.08457847	0.316	0.614	1.87E-09	SMC2
CLSPN	9.88E-13	0.00465081	0.471	0.598	1.98E-09	SMC2
PLXDC1	9.96E-13	0.04457526	0.745	0.947	1.99E-09	SMC2
AQP3	1.20E-12	-0.0301889	0.597	0.744	2.40E-09	SMC2
SSTR2	1.22E-12	-0.0119589	0.677	0.847	2.44E-09	SMC2
HERPUD1	1.25E-12	-0.0536719	0.543	0.78	2.50E-09	SMC2
LSAMP	1.26E-12	-0.1104285	0.375	0.358	2.51E-09	SMC2
CCL17	1.49E-12	1.18E-05	0.172	0.336	2.97E-09	SMC2

C2CD4B	1.54E-12	-0.0332105	0.545	0.449	3.09E-09	SMC2
ADAMTS5	1.75E-12	-0.2276248	0.325	0.317	3.49E-09	SMC2
ANGPTL7	1.92E-12	-0.0675437	0.117	0.072	3.85E-09	SMC2
NDC80	1.93E-12	0.0090247	0.665	0.866	3.86E-09	SMC2
MIR181A1H	2.02E-12	0.04599532	0.726	0.919	4.04E-09	SMC2
C1QTNF1	2.11E-12	0.43257851	0.429	0.687	4.22E-09	SMC2
SLC16A3	2.22E-12	0.04138113	0.635	0.79	4.44E-09	SMC2
BUB1B	2.37E-12	4.99E-05	0.13	0.316	4.74E-09	SMC2
MTUS1	2.47E-12	0.00394362	0.278	0.565	4.93E-09	SMC2
CD59	2.82E-12	0.25669491	0.412	0.699	5.64E-09	SMC2
TUBA1A	3.04E-12	0.41643737	0.853	0.839	6.08E-09	SMC2
HTR2B	3.32E-12	0.06458361	0.211	0.411	6.64E-09	SMC2
C17orf58	3.78E-12	-0.1230963	0.354	0.331	7.55E-09	SMC2
NPTX2	4.57E-12	0.06378728	0.154	0.23	9.14E-09	SMC2
MMRN2	4.73E-12	-0.0263229	0.313	0.535	9.46E-09	SMC2
BMP6	4.87E-12	0.01037518	0.095	0.285	9.74E-09	SMC2
CTHRC1	4.90E-12	-0.1108768	0.712	0.572	9.81E-09	SMC2
MAFB	5.60E-12	0.14892865	0.405	0.642	1.12E-08	SMC2
LEFTY2	5.70E-12	0.04475546	0.557	0.767	1.14E-08	SMC2
COL5A2	6.03E-12	0.35456083	0.277	0.495	1.21E-08	SMC2
DAB2	6.54E-12	0.02391599	0.664	0.804	1.31E-08	SMC2
NAF1	6.62E-12	0.04377556	0.453	0.744	1.32E-08	SMC2
ID4	6.67E-12	0.4226333	0.633	0.554	1.33E-08	SMC2
ECSCR	6.90E-12	-0.0084719	0.361	0.328	1.38E-08	SMC2
SCNN1B	8.05E-12	1.76E-05	0.275	0.469	1.61E-08	SMC2
CRIP2	8.37E-12	0.45773255	0.579	0.522	1.67E-08	SMC2
SRPX	8.49E-12	-0.0058364	0.636	0.63	1.70E-08	SMC2
GSTM3	9.02E-12	0.04957956	0.339	0.641	1.80E-08	SMC2
RNASE6	9.12E-12	0.02550877	0.669	0.904	1.82E-08	SMC2
CYFIP2	9.62E-12	0.19260389	0.394	0.663	1.92E-08	SMC2
OBP2A	9.74E-12	-0.0026833	0.519	0.66	1.95E-08	SMC2
LILRB3	9.96E-12	-0.0009452	0.337	0.341	1.99E-08	SMC2
MRGPRF	1.01E-11	0.01637694	0.362	0.28	2.03E-08	SMC2
CENPK	1.04E-11	-0.001853	0.361	0.32	2.08E-08	SMC2
PAEP	1.11E-11	-0.0001209	0.364	0.519	2.21E-08	SMC2
CCL18	1.13E-11	0.0866124	0.332	0.52	2.26E-08	SMC2
ECEL1	1.15E-11	-0.0016709	0.154	0.15	2.31E-08	SMC2
LINC01485	1.17E-11	0.00014164	0.37	0.57	2.33E-08	SMC2
SULF1	1.22E-11	0.42557607	0.442	0.726	2.43E-08	SMC2
MXRA8	1.23E-11	0.12601458	0.324	0.287	2.46E-08	SMC2
EMX2	1.34E-11	0.03306969	0.489	0.73	2.68E-08	SMC2
SIX1	1.47E-11	-0.0300818	0.286	0.507	2.94E-08	SMC2
ST8SIA6	1.52E-11	-0.0041513	0.1	0.32	3.04E-08	SMC2
GGT5	1.52E-11	0.15026483	0.682	0.894	3.05E-08	SMC2
CD72	1.78E-11	0.00503064	0.63	0.705	3.56E-08	SMC2
LIF	1.81E-11	-0.0257045	0.297	0.509	3.63E-08	SMC2
LYN	1.84E-11	-0.0545248	0.18	0.447	3.67E-08	SMC2
C16orf89	2.03E-11	-0.1708876	0.437	0.415	4.07E-08	SMC2
CD93	2.15E-11	0.04374107	0.545	0.669	4.29E-08	SMC2
RBPMS2	2.17E-11	-0.0207131	0.445	0.442	4.34E-08	SMC2
MYC	2.40E-11	-0.0144091	0.434	0.482	4.80E-08	SMC2
PCLAF	2.48E-11	0.01461015	0.034	0.232	4.97E-08	SMC2
MIA	2.63E-11	-0.0105352	0.089	0.1	5.25E-08	SMC2

ELN	2.81E-11	-0.2430763	0.483	0.461	5.61E-08	SMC2
SMC2	3.52E-11	0.01450003	0.668	0.664	7.05E-08	SMC2
HBB	3.64E-11	1.0848986	0.139	0.057	7.28E-08	SMC2
GPR146	3.78E-11	0.00039494	0.175	0.206	7.56E-08	SMC2
PLA2G2A	3.98E-11	-1.1444039	0.372	0.366	7.95E-08	SMC2
TMEM100	4.04E-11	-0.0189872	0.136	0.178	8.07E-08	SMC2
LBH	6.28E-11	0.23082371	0.533	0.698	1.26E-07	SMC2
LMCD1	6.67E-11	-0.0690416	0.613	0.553	1.33E-07	SMC2
SLCO2A1	6.89E-11	-0.0020809	0.661	0.822	1.38E-07	SMC2
IBSP	7.02E-11	0.2210392	0.672	0.82	1.40E-07	SMC2
HEY1	7.10E-11	0.03787793	0.669	0.86	1.42E-07	SMC2
ANGPTL5	7.21E-11	0.00164579	0.448	0.423	1.44E-07	SMC2
RAB3C	7.38E-11	0.01590717	0.643	0.522	1.48E-07	SMC2
OTOA	7.38E-11	0.00823992	0.25	0.453	1.48E-07	SMC2
DES	7.71E-11	-0.0345739	0.136	0.115	1.54E-07	SMC2
GLUL	7.85E-11	-0.0257029	0.355	0.6	1.57E-07	SMC2
CKS2	8.30E-11	0.16484484	0.708	0.89	1.66E-07	SMC2
SEMA3F	8.84E-11	0.03284826	0.624	0.817	1.77E-07	SMC2
CD3G	1.01E-10	0.08006102	0.68	0.893	2.01E-07	SMC2
ADM	1.06E-10	-0.1398976	0.473	0.47	2.11E-07	SMC2
SDC3	1.08E-10	-0.0565404	0.2	0.377	2.16E-07	SMC2
FCN3	1.14E-10	-7.87E-06	0.116	0.247	2.29E-07	SMC2
NR1H3	1.16E-10	-0.1801325	0.673	0.612	2.33E-07	SMC2
AP1S2	1.18E-10	0.15271554	0.62	0.789	2.36E-07	SMC2
NFIL3	1.21E-10	-0.0859909	0.307	0.617	2.42E-07	SMC2
AC106028.4	1.24E-10	-0.0031133	0.273	0.486	2.48E-07	SMC2
EGLN3	1.25E-10	-0.1117578	0.542	0.732	2.49E-07	SMC2
ALOX5	1.39E-10	-0.0074907	0.268	0.474	2.79E-07	SMC2
IGLV3-19	1.41E-10	-0.0012531	0.082	0.244	2.82E-07	SMC2
HSPA1A	1.43E-10	-0.4174073	0.834	0.946	2.86E-07	SMC2
SPIB	1.43E-10	0.01844884	0.208	0.469	2.86E-07	SMC2
GPNMB	1.45E-10	-0.3251584	0.485	0.661	2.90E-07	SMC2
GPR84	1.46E-10	-0.0001254	0.106	0.228	2.92E-07	SMC2
MT1F	1.78E-10	0.04137798	0.643	0.839	3.56E-07	SMC2
ASAH1	1.92E-10	0.16477398	0.634	0.568	3.84E-07	SMC2
SLAMF7	2.13E-10	0.00207704	0.311	0.541	4.26E-07	SMC2
CD48	2.14E-10	0.2253915	0.722	0.869	4.28E-07	SMC2
MXD1	2.68E-10	-0.0373927	0.451	0.636	5.36E-07	SMC2
CXCL12	2.82E-10	0.3049788	0.737	0.711	5.64E-07	SMC2
PRF1	2.92E-10	0.00112564	0.194	0.207	5.84E-07	SMC2
CLLU1OS	2.96E-10	-8.23E-05	0.474	0.631	5.91E-07	SMC2
IRF8	3.23E-10	0.05377135	0.582	0.767	6.46E-07	SMC2
S100A16	3.38E-10	0.1549866	0.576	0.763	6.77E-07	SMC2
CXCL8	4.15E-10	0.24629188	0.365	0.603	8.30E-07	SMC2
SMPDL3B	4.28E-10	-0.0003116	0.474	0.644	8.55E-07	SMC2
MYOZ2	4.86E-10	0.01377811	0.505	0.78	9.73E-07	SMC2
CDH19	4.99E-10	-0.077898	0.113	0.312	9.98E-07	SMC2
PCDH10	5.12E-10	0.19905359	0.387	0.626	1.02E-06	SMC2
PSAP	5.42E-10	0.02954946	0.329	0.524	1.08E-06	SMC2
IFITM3	6.46E-10	0.82213805	0.765	0.733	1.29E-06	SMC2
STX11	6.77E-10	0.0185874	0.377	0.58	1.35E-06	SMC2
CTSS	6.87E-10	0.20474899	0.595	0.801	1.37E-06	SMC2
ERLEC1	6.89E-10	0.19337715	0.473	0.71	1.38E-06	SMC2

CRHBP	6.99E-10	0.02369541	0.068	0.248	1.40E-06	SMC2
TRIB1	7.25E-10	-0.0543804	0.643	0.782	1.45E-06	SMC2
ZNF331	7.95E-10	-0.1158096	0.254	0.491	1.59E-06	SMC2
XBP1	8.43E-10	-0.3138779	0.516	0.499	1.69E-06	SMC2
SERPINB2	8.69E-10	-0.0054462	0.19	0.381	1.74E-06	SMC2
CCNA2	9.70E-10	0.01027597	0.581	0.511	1.94E-06	SMC2
PELI1	1.00E-09	0.26409528	0.312	0.58	2.01E-06	SMC2
CLEC10A	1.11E-09	0.03999905	0.447	0.6	2.22E-06	SMC2
IGLV3-1	1.14E-09	-0.007612	0.452	0.649	2.28E-06	SMC2
FPR3	1.26E-09	-1.77E-05	0.306	0.527	2.52E-06	SMC2
CFD	1.34E-09	-1.3708965	0.257	0.331	2.69E-06	SMC2
FTH1	1.37E-09	0.40238942	0.859	0.944	2.74E-06	SMC2
NTM	1.43E-09	-0.1574144	0.213	0.255	2.86E-06	SMC2
LINC02207	1.55E-09	-0.0001874	0.063	0.171	3.09E-06	SMC2
CAVIN1	1.55E-09	0.1118756	0.548	0.52	3.09E-06	SMC2
SAMSN1	1.67E-09	0.08406905	0.098	0.332	3.33E-06	SMC2
EBI3	1.92E-09	0.01191074	0.185	0.159	3.84E-06	SMC2
PLN	1.95E-09	0.70821751	0.209	0.404	3.90E-06	SMC2
LHFPL6	2.21E-09	0.69341249	0.423	0.419	4.43E-06	SMC2
CDH6	2.23E-09	-0.0089203	0.677	0.874	4.46E-06	SMC2
NPY1R	2.35E-09	-0.3035765	0.291	0.348	4.71E-06	SMC2
NNMT	2.36E-09	0.11810732	0.724	0.902	4.71E-06	SMC2
TPM2	2.77E-09	0.85643869	0.656	0.667	5.55E-06	SMC2
PID1	2.79E-09	0.16208269	0.431	0.478	5.58E-06	SMC2
PRSS35	2.90E-09	0.06449034	0.197	0.249	5.79E-06	SMC2
S100A6	3.02E-09	0.67428922	0.757	0.804	6.04E-06	SMC2
SYAP1	3.27E-09	-0.0477406	0.214	0.484	6.53E-06	SMC2
SOD3	3.99E-09	0.69413501	0.416	0.344	7.99E-06	SMC2
IGLV2-14	4.33E-09	-0.0097168	0.348	0.581	8.65E-06	SMC2
BTNL9	4.41E-09	-0.0016108	0.668	0.763	8.81E-06	SMC2
MOXD1	4.74E-09	0.02587191	0.377	0.558	9.48E-06	SMC2
CSF2	4.83E-09	-0.003433	0.1	0.309	9.67E-06	SMC2
SLC9A3R2	5.33E-09	0.21101441	0.559	0.743	1.07E-05	SMC2
CBR3	5.87E-09	0.04489523	0.483	0.539	1.17E-05	SMC2
ENAH	6.39E-09	0.11573328	0.706	0.909	1.28E-05	SMC2
APLN	6.45E-09	-0.0010645	0.285	0.412	1.29E-05	SMC2
HS6ST3	6.67E-09	0.01480085	0.079	0.195	1.33E-05	SMC2
WNT5A	7.96E-09	-0.0240836	0.445	0.453	1.59E-05	SMC2
CRYM	8.01E-09	-0.0003214	0.074	0.195	1.60E-05	SMC2
GSN	8.13E-09	-0.176947	0.542	0.782	1.63E-05	SMC2
PDK4	8.88E-09	0.0638912	0.234	0.507	1.78E-05	SMC2
EFNA1	9.28E-09	-0.0767312	0.618	0.592	1.86E-05	SMC2
CCNB1	1.12E-08	0.02755735	0.274	0.446	2.23E-05	SMC2
CYP4B1	1.16E-08	-0.2818784	0.315	0.327	2.32E-05	SMC2
GPM6A	1.35E-08	-0.0306592	0.206	0.156	2.70E-05	SMC2
COL4A1	1.36E-08	0.56635984	0.622	0.804	2.72E-05	SMC2
AL356417.2	1.43E-08	-0.0009066	0.214	0.4	2.86E-05	SMC2
GZF1	1.44E-08	0.04117146	0.575	0.546	2.89E-05	SMC2
SERPINH1	1.45E-08	-0.0685424	0.642	0.795	2.90E-05	SMC2
LINC02446	1.53E-08	0.00067483	0.083	0.103	3.06E-05	SMC2
FAM133A	1.59E-08	-0.0058031	0.172	0.31	3.19E-05	SMC2
RGS16	1.63E-08	0.39899276	0.799	0.896	3.27E-05	SMC2
PILRA	1.65E-08	-0.0023748	0.581	0.427	3.31E-05	SMC2

CYTL1	1.69E-08	0.09435013	0.641	0.771	3.39E-05	SMC2
CD70	1.72E-08	0.0241931	0.403	0.355	3.44E-05	SMC2
FJX1	1.74E-08	0.0194928	0.463	0.631	3.48E-05	SMC2
ACTA2	1.74E-08	0.75846884	0.756	0.892	3.49E-05	SMC2
MID1IP1	1.82E-08	0.05563383	0.549	0.564	3.64E-05	SMC2
FAM107A	1.85E-08	-0.0194429	0.392	0.587	3.71E-05	SMC2
CYBB	1.87E-08	0.07879439	0.434	0.566	3.73E-05	SMC2
CARMN	1.89E-08	0.56880782	0.561	0.569	3.77E-05	SMC2
RCN3	1.90E-08	0.06956605	0.409	0.652	3.79E-05	SMC2
PTHLH	1.90E-08	0.08928163	0.512	0.687	3.81E-05	SMC2
RRAD	2.01E-08	-0.0646423	0.453	0.622	4.01E-05	SMC2
PRDM6	2.09E-08	-0.10567	0.255	0.481	4.17E-05	SMC2
IGHG2	2.21E-08	0.11938649	0.747	0.911	4.42E-05	SMC2
TBX2	2.29E-08	-0.1634182	0.239	0.488	4.58E-05	SMC2
TMEM215	2.52E-08	-6.84E-05	0.044	0.126	5.03E-05	SMC2
LDB2	2.65E-08	0.02442579	0.623	0.545	5.30E-05	SMC2
MARCO	2.79E-08	0.06109841	0.411	0.55	5.57E-05	SMC2
DHRS3	2.94E-08	-0.3855252	0.242	0.264	5.88E-05	SMC2
PRPH	2.99E-08	-1.40E-05	0.101	0.118	5.98E-05	SMC2
CPXM2	3.04E-08	0.11601757	0.662	0.654	6.08E-05	SMC2
MFAP2	3.12E-08	0.09204709	0.222	0.253	6.24E-05	SMC2
GPBAR1	3.13E-08	-0.0093181	0.122	0.145	6.26E-05	SMC2
KRT86	3.27E-08	0.00426199	0.106	0.234	6.55E-05	SMC2
BMPR2	3.35E-08	0.15994852	0.276	0.474	6.69E-05	SMC2
QPRT	3.54E-08	-0.0141628	0.591	0.713	7.09E-05	SMC2
TPD52L1	3.85E-08	0.04786077	0.664	0.839	7.69E-05	SMC2
ADAP2	3.94E-08	-0.0823758	0.735	0.944	7.89E-05	SMC2
CH25H	3.97E-08	0.00135976	0.209	0.285	7.94E-05	SMC2
GFPT2	4.18E-08	-0.294668	0.718	0.764	8.35E-05	SMC2
TGFB1I1	4.18E-08	0.56829223	0.433	0.447	8.37E-05	SMC2
SMIM5	4.33E-08	-0.0410721	0.192	0.23	8.67E-05	SMC2
GNG11	4.35E-08	0.20987685	0.5	0.763	8.70E-05	SMC2
SHISA3	4.77E-08	-0.0219329	0.192	0.224	9.54E-05	SMC2
E2F8	5.07E-08	0.00127073	0.092	0.238	0.0001014	SMC2
FKBP1A	5.24E-08	0.253639	0.27	0.515	0.00010478	SMC2
FBLN5	5.24E-08	-0.063348	0.227	0.472	0.0001048	SMC2
RDX	5.47E-08	0.09255511	0.247	0.463	0.00010933	SMC2
PLK1	5.49E-08	0.01939566	0.62	0.614	0.00010976	SMC2
OLFML3	5.99E-08	-0.0807676	0.639	0.598	0.00011978	SMC2
C19orf38	6.20E-08	-0.0159802	0.298	0.472	0.00012396	SMC2
SLC7A2	6.24E-08	0.26051742	0.425	0.676	0.00012489	SMC2
C1S	6.58E-08	-0.0878925	0.567	0.526	0.0001316	SMC2
KRT17	6.90E-08	0.2168062	0.696	0.844	0.00013798	SMC2
DEPP1	7.23E-08	0.15471067	0.349	0.6	0.00014458	SMC2
LRRC10B	7.72E-08	-0.0024301	0.652	0.812	0.00015444	SMC2
TBX1	7.74E-08	-0.0715208	0.314	0.309	0.00015487	SMC2
CA4	7.89E-08	-0.0220859	0.03	0.173	0.00015781	SMC2
FCN1	8.00E-08	0.03885814	0.276	0.436	0.00015996	SMC2
HPGD	8.50E-08	-0.0060214	0.305	0.466	0.00016995	SMC2
IFI44L	8.57E-08	0.15445831	0.703	0.928	0.00017142	SMC2
SLC1A3	8.59E-08	-0.0887061	0.463	0.401	0.00017188	SMC2
FCGR3A	8.60E-08	0.17855082	0.308	0.538	0.00017196	SMC2
PLA1A	9.05E-08	-0.006614	0.224	0.382	0.00018096	SMC2

GRP	9.46E-08	0.02772783	0.204	0.325	0.00018922	SMC2
WNT2	1.01E-07	-0.0217802	0.201	0.374	0.00020193	SMC2
AK5	1.02E-07	0.02773939	0.246	0.432	0.00020317	SMC2
P2RY13	1.06E-07	0.02157275	0.183	0.234	0.00021113	SMC2
PROCR	1.07E-07	0.19244762	0.173	0.363	0.00021404	SMC2
GPX8	1.17E-07	0.08690716	0.733	0.724	0.00023432	SMC2
TNFRSF13B	1.19E-07	0.00340518	0.286	0.438	0.00023723	SMC2
TNNI2	1.20E-07	0.00050328	0.21	0.214	0.00024012	SMC2
HCAR3	1.25E-07	0.00015011	0.12	0.308	0.00025015	SMC2
CTSD	1.26E-07	0.28476241	0.299	0.542	0.00025198	SMC2
SEMA3G	1.42E-07	0.02359363	0.164	0.314	0.00028328	SMC2
PDXK	1.59E-07	0.05228734	0.541	0.669	0.00031808	SMC2
OGFRL1	1.66E-07	0.04349446	0.667	0.711	0.00033224	SMC2
STXBP2	1.74E-07	-0.0188757	0.27	0.305	0.00034806	SMC2
HMMR	1.80E-07	0.03879135	0.21	0.366	0.00035915	SMC2
BACE2	1.80E-07	0.0908807	0.253	0.397	0.0003593	SMC2
DSE	1.81E-07	-0.2005318	0.339	0.411	0.00036294	SMC2
LY86	1.89E-07	0.02074887	0.436	0.645	0.00037742	SMC2
PAICS	1.99E-07	0.01457577	0.371	0.603	0.0003987	SMC2
ALPL	2.01E-07	-0.0134755	0.234	0.407	0.00040299	SMC2
ODC1	2.06E-07	0.02323145	0.422	0.599	0.00041151	SMC2
SYT1	2.16E-07	-0.0116106	0.025	0.138	0.0004318	SMC2
SERPINA5	2.22E-07	0.00649639	0.219	0.297	0.00044314	SMC2
IGSF21	2.30E-07	0.0091737	0.21	0.413	0.00045998	SMC2
NCR3	2.32E-07	0.0045453	0.295	0.462	0.00046386	SMC2
RGS13	2.41E-07	-0.0025492	0.437	0.634	0.0004813	SMC2
RASL12	2.48E-07	0.05056087	0.248	0.444	0.0004966	SMC2
MPZL2	2.53E-07	0.0879865	0.092	0.192	0.00050695	SMC2
TWISTNB	2.55E-07	0.14644598	0.224	0.289	0.0005098	SMC2
IL1R1	2.58E-07	-0.0535414	0.305	0.37	0.00051537	SMC2
CDCA3	2.79E-07	0.01496622	0.592	0.766	0.00055708	SMC2
LINC02185	3.16E-07	0.03783114	0.313	0.317	0.00063292	SMC2
EFEMP2	3.21E-07	0.06554176	0.649	0.846	0.00064241	SMC2
PCDHA12	3.36E-07	-0.0024295	0.095	0.274	0.00067124	SMC2
GRN	3.37E-07	0.15293228	0.671	0.779	0.00067438	SMC2
RBM47	3.40E-07	-0.0592257	0.206	0.401	0.00067922	SMC2
NEGR1	3.43E-07	-0.0203036	0.506	0.691	0.00068545	SMC2
KCNMB1	3.45E-07	0.09203144	0.268	0.531	0.00068966	SMC2
CASQ2	3.81E-07	-0.0092971	0.167	0.35	0.0007621	SMC2
GZMA	3.95E-07	0.30367172	0.732	0.944	0.00078906	SMC2
BAMBI	4.15E-07	0.03351824	0.313	0.499	0.00082945	SMC2
CD1C	4.22E-07	0.02217671	0.111	0.297	0.00084331	SMC2
ID1	4.29E-07	0.3548914	0.618	0.774	0.00085768	SMC2
CX3CL1	4.71E-07	0.2840529	0.424	0.627	0.00094185	SMC2
SLC7A7	4.72E-07	0.06616019	0.311	0.473	0.00094358	SMC2
FLT1	4.82E-07	-0.0365781	0.646	0.772	0.00096452	SMC2
HIST1H1E	5.05E-07	-0.0546343	0.46	0.659	0.00100933	SMC2
GFRA1	5.20E-07	-0.0917717	0.273	0.31	0.00103953	SMC2
CENPM	5.48E-07	0.00345793	0.603	0.695	0.0010962	SMC2
EGR2	5.86E-07	0.104838	0.707	0.836	0.00117299	SMC2
EAF2	5.92E-07	0.00633632	0.627	0.527	0.00118324	SMC2
ASPN	5.99E-07	0.3942024	0.333	0.587	0.00119856	SMC2
SYNM	7.06E-07	0.14339302	0.351	0.392	0.00141245	SMC2

GPM6B	7.11E-07	0.16333797	0.418	0.389	0.00142218	SMC2
CCL8	7.17E-07	-0.0225246	0.656	0.817	0.00143426	SMC2
CRISPLD1	7.38E-07	0.27575956	0.357	0.545	0.00147506	SMC2
ISYNA1	7.83E-07	0.12928789	0.457	0.478	0.00156634	SMC2
DRD4	8.59E-07	0.04256811	0.695	0.848	0.00171741	SMC2
TNFSF9	9.01E-07	0.00927449	0.585	0.495	0.00180232	SMC2
ADH4	9.07E-07	0.02442297	0.328	0.332	0.0018131	SMC2
SAMD11	9.69E-07	-0.3518074	0.329	0.359	0.00193735	SMC2
ISLR	9.75E-07	0.05774104	0.595	0.648	0.00195082	SMC2
NPDC1	9.79E-07	0.01942659	0.341	0.394	0.0019589	SMC2
COL4A2	9.95E-07	0.42854684	0.531	0.762	0.00199054	SMC2
AQP1	1.09E-06	0.68329651	0.344	0.663	0.0021839	SMC2
EGFLAM	1.10E-06	-0.0098898	0.211	0.455	0.00219821	SMC2
DCN	1.12E-06	-0.3526936	0.763	0.593	0.00223899	SMC2
MMP1	1.21E-06	0.03217922	0.108	0.173	0.0024203	SMC2
TNFSF18	1.26E-06	0.01281024	0.155	0.335	0.00252991	SMC2
CD55	1.35E-06	-0.0208458	0.5	0.558	0.00270624	SMC2
PPT1	1.64E-06	0.10616886	0.17	0.262	0.00327195	SMC2
COPZ2	1.67E-06	0.1980152	0.506	0.545	0.00334229	SMC2
DEFB1	1.68E-06	-0.0378672	0.108	0.215	0.00336042	SMC2
KIF11	1.72E-06	0.0179092	0.421	0.522	0.00344636	SMC2
SLC2A5	1.73E-06	-0.0003048	0.33	0.343	0.00345117	SMC2
DEPDC1	1.73E-06	0.00255704	0.082	0.225	0.00347	SMC2
HLA-DMA	1.81E-06	-0.0559739	0.605	0.768	0.00361139	SMC2
CD86	1.92E-06	0.01300824	0.6	0.707	0.00383251	SMC2
ICOS	1.94E-06	0.01148293	0.178	0.252	0.0038836	SMC2
PDLIM7	1.98E-06	0.12906208	0.195	0.411	0.00396987	SMC2
SHCBP1	2.30E-06	-0.0002968	0.165	0.256	0.00459638	SMC2
CDT1	2.34E-06	0.0027178	0.346	0.332	0.00467116	SMC2
SLAMF8	2.40E-06	0.02070653	0.289	0.461	0.00480251	SMC2
FHL1	2.42E-06	0.18285592	0.543	0.778	0.00483106	SMC2
P2RY6	2.44E-06	-0.0243196	0.276	0.444	0.00488683	SMC2
GADD45G	2.46E-06	0.23587676	0.277	0.488	0.00491748	SMC2
MEG3	2.60E-06	-0.4885539	0.579	0.561	0.00519258	SMC2
CCL23	2.67E-06	0.00059154	0.089	0.202	0.00533394	SMC2
SPIC	2.84E-06	-0.0056332	0.064	0.138	0.00567587	SMC2
MAP1B	2.95E-06	0.36529335	0.578	0.602	0.00589156	SMC2
MARCKS	2.99E-06	-0.0644235	0.801	0.972	0.00598193	SMC2
ARX	3.06E-06	-7.19E-06	0.042	0.123	0.00611541	SMC2
CDH5	3.06E-06	0.00436868	0.271	0.428	0.00611705	SMC2
SERPINB9	3.26E-06	-0.0853309	0.182	0.417	0.00651641	SMC2
ICAM2	3.32E-06	0.01285892	0.654	0.775	0.00663513	SMC2
C15orf48	3.33E-06	0.14793901	0.625	0.603	0.00666426	SMC2
ADAMTS4	3.50E-06	-0.2476544	0.665	0.683	0.00700317	SMC2
SLC26A4	3.80E-06	0.04502842	0.354	0.356	0.00760926	SMC2
IGHE	3.88E-06	-1.55E-06	0.227	0.4	0.00775894	SMC2
LRRN4CL	3.89E-06	-0.1940834	0.29	0.367	0.00777686	SMC2
IFIT3	3.90E-06	0.10859404	0.196	0.259	0.00779103	SMC2
THY1	4.11E-06	0.26067648	0.51	0.734	0.00822011	SMC2
CDC20	4.14E-06	0.00591437	0.624	0.709	0.00827847	SMC2
MAL	4.17E-06	-0.0394885	0.209	0.271	0.00834027	SMC2
GJB2	4.30E-06	-0.0048815	0.222	0.4	0.0085958	SMC2
HLA-DQA2	4.45E-06	0.06241102	0.462	0.634	0.00890014	SMC2

C5orf46	4.45E-06	0.1132577	0.726	0.874	0.00890523	SMC2
COMP	4.50E-06	-0.1960688	0.09	0.125	0.00900404	SMC2
PYCARD	4.69E-06	0.14236584	0.603	0.718	0.00938987	SMC2
PLA2G5	4.71E-06	0.04330289	0.312	0.48	0.00942299	SMC2
TGM2	4.86E-06	0.62774644	0.275	0.484	0.00972829	SMC2
ZNF683	4.88E-06	0.00557569	0.321	0.385	0.00976611	SMC2
LPCAT2	4.97E-06	0.13091737	0.362	0.565	0.00993742	SMC2
HAS1	5.10E-06	-0.0320227	0.182	0.352	0.01019323	SMC2
DNAAF1	5.16E-06	0.00934064	0.189	0.244	0.01032636	SMC2
SPAG4	5.18E-06	0.01157463	0.449	0.637	0.01035196	SMC2
SPARC	5.38E-06	0.49318806	0.53	0.791	0.01076738	SMC2
MBNL1-AS1	5.45E-06	0.19549455	0.605	0.668	0.01089902	SMC2
IER5	5.46E-06	0.2053827	0.689	0.928	0.01092467	SMC2
SNAI1	5.49E-06	0.02979614	0.399	0.509	0.01098369	SMC2
GABRA2	5.53E-06	-0.0192116	0.052	0.142	0.01105294	SMC2
PLOD2	5.57E-06	0.15338538	0.366	0.602	0.01114623	SMC2
ASF1B	5.58E-06	-0.0001529	0.226	0.425	0.01116923	SMC2
FAM167B	5.61E-06	0.01846714	0.111	0.32	0.01122531	SMC2
ADIRF	5.62E-06	0.81198924	0.566	0.522	0.01124117	SMC2
CHI3L2	6.39E-06	0.05491669	0.475	0.625	0.01278325	SMC2
NCF1	6.40E-06	0.05388452	0.321	0.542	0.01280064	SMC2
SBSPON	6.69E-06	0.08442712	0.128	0.305	0.01337799	SMC2
CNKSR3	6.78E-06	0.08391951	0.421	0.48	0.01356516	SMC2
SOX18	6.85E-06	0.02287731	0.501	0.672	0.01369244	SMC2
KCNJ2	6.92E-06	-0.0032905	0.206	0.442	0.01383438	SMC2
FN1	6.95E-06	0.72755084	0.687	0.86	0.01389495	SMC2
PRSS23	6.97E-06	0.29770519	0.553	0.817	0.01394461	SMC2
GDF10	7.04E-06	-0.1072978	0.649	0.623	0.01407917	SMC2
MEOX1	7.29E-06	0.01829092	0.232	0.425	0.01457716	SMC2
APLNR	7.31E-06	-0.0059401	0.214	0.373	0.01461885	SMC2
CNTN4	7.46E-06	-0.0299742	0.462	0.644	0.0149181	SMC2
IL13	7.83E-06	0.00646478	0.178	0.405	0.01566224	SMC2
DKK1	7.84E-06	-0.0027054	0.24	0.444	0.01568903	SMC2
EFHD1	7.92E-06	0.09260137	0.424	0.43	0.0158404	SMC2
TFF3	8.45E-06	-0.0025418	0.696	0.78	0.01690704	SMC2
RBP1	8.46E-06	0.12953059	0.302	0.48	0.01692758	SMC2
MFAP5	8.94E-06	-0.7483667	0.405	0.428	0.01787447	SMC2
MYH11	9.06E-06	0.37831356	0.567	0.546	0.01811673	SMC2
HES6	9.07E-06	-0.0433382	0.159	0.393	0.01813622	SMC2
SAT1	9.20E-06	0.21180624	0.666	0.657	0.01839159	SMC2
CCL19	9.36E-06	1.28795276	0.791	0.996	0.0187235	SMC2
LAMB1	9.47E-06	0.43568162	0.286	0.541	0.01893564	SMC2
TPM1	9.54E-06	0.51243369	0.784	0.817	0.01907425	SMC2
GINS2	9.55E-06	-0.006173	0.714	0.847	0.0190919	SMC2
DTL	9.56E-06	3.66E-05	0.157	0.268	0.01912696	SMC2
FILIP1L	9.60E-06	0.03732836	0.486	0.678	0.01919034	SMC2
DMXL2	9.67E-06	-0.017649	0.246	0.299	0.0193371	SMC2
CD8B	9.71E-06	0.04874333	0.256	0.294	0.01942606	SMC2
TRDC	1.03E-05	0.01228357	0.271	0.488	0.02057008	SMC2
S100A9	1.06E-05	-0.05061	0.606	0.657	0.02117911	SMC2
BDNF	1.08E-05	0.00289866	0.166	0.255	0.02160259	SMC2
LGALS3	1.10E-05	0.55466182	0.602	0.614	0.02192673	SMC2
MS4A1	1.10E-05	0.03602644	0.309	0.492	0.02200124	SMC2

KLK1	1.12E-05	-0.0009012	0.222	0.416	0.02236933	SMC2
SAP30	1.16E-05	0.03839714	0.6	0.802	0.02315251	SMC2
SELENOP	1.18E-05	0.12482836	0.667	0.808	0.02363653	SMC2
PECAM1	1.24E-05	-0.0162994	0.141	0.362	0.02485796	SMC2
MCM7	1.26E-05	-0.0014303	0.512	0.785	0.02514122	SMC2
RSPO3	1.33E-05	-0.1405422	0.204	0.278	0.0266165	SMC2
IGHV3-11	1.33E-05	0.00036505	0.239	0.473	0.02669341	SMC2
TAGLN	1.35E-05	0.19996206	0.822	0.841	0.0269943	SMC2
MX2	1.35E-05	0.08095703	0.525	0.577	0.02704983	SMC2
KRT16	1.36E-05	0.00986371	0.183	0.332	0.02729575	SMC2
C2orf40	1.40E-05	0.71520954	0.797	0.759	0.02795454	SMC2
JDP2	1.44E-05	-0.0568679	0.596	0.576	0.02889086	SMC2
MYLK	1.49E-05	0.358874	0.487	0.729	0.02988617	SMC2
INHBB	1.52E-05	0.0346115	0.152	0.217	0.03037569	SMC2
PTAFR	1.57E-05	0.01669779	0.366	0.486	0.03142138	SMC2
HHIP	1.58E-05	0.02201282	0.133	0.199	0.03156507	SMC2
VPREB3	1.63E-05	0.02222948	0.272	0.44	0.03269547	SMC2
TBXAS1	1.65E-05	0.03873403	0.506	0.453	0.0329208	SMC2
MTSS1	1.97E-05	-0.147258	0.629	0.705	0.03945785	SMC2
IGHV5-10-1	2.12E-05	0.00248923	0.298	0.369	0.04242142	SMC2
IFI27	2.16E-05	0.18991337	0.558	0.76	0.04322308	SMC2
THBS4	2.30E-05	-0.0019973	0.312	0.488	0.04600012	SMC2
RCAN2	2.32E-05	0.37203182	0.29	0.388	0.04631268	SMC2
PALMD	2.38E-05	0.03078635	0.263	0.301	0.04764932	SMC2
ANKRD1	2.41E-05	-0.0004487	0.702	0.851	0.04813117	SMC2
JUN	2.42E-05	-0.181085	0.831	0.892	0.04842712	SMC2
AIF1	3.78E-56	0.016805	0.218	0.953	7.57E-53	SMC3
ALOX5AP	1.02E-53	0.0267396	0.456	0.986	2.04E-50	SMC3
FCN1	3.89E-49	-0.0013444	0.147	0.858	7.79E-46	SMC3
FCGR2B	5.40E-46	0.00808249	0.215	0.873	1.08E-42	SMC3
CXCR3	2.43E-45	-0.0136391	0.329	0.901	4.86E-42	SMC3
TRAC	4.59E-44	0.05894988	0.668	0.995	9.19E-41	SMC3
SAMSN1	2.63E-42	-0.0223467	0.668	1	5.25E-39	SMC3
SPI1	5.09E-42	0.00910031	0.251	0.877	1.02E-38	SMC3
SOX18	8.71E-42	-0.0067371	0.137	0.807	1.74E-38	SMC3
CD37	1.98E-40	0.00884306	0.713	0.995	3.96E-37	SMC3
LGALS9	2.18E-40	-0.0134044	0.189	0.821	4.37E-37	SMC3
LCP1	1.03E-39	0.02564408	0.586	0.981	2.06E-36	SMC3
IGHG1	2.74E-39	-0.000596	0.668	1	5.47E-36	SMC3
SDS	6.17E-39	0.0018268	0.088	0.722	1.23E-35	SMC3
NCF4	2.96E-38	-0.0067841	0.414	0.906	5.92E-35	SMC3
EPSTI1	4.39E-37	0.0372798	0.541	0.948	8.78E-34	SMC3
TYROBP	1.39E-36	0.09774629	0.202	0.877	2.77E-33	SMC3
C1orf162	2.38E-36	-0.003722	0.472	0.91	4.75E-33	SMC3
C15orf48	8.00E-36	-0.0396502	0.023	0.613	1.60E-32	SMC3
FABP4	2.49E-35	0.14815979	0.322	0.948	4.98E-32	SMC3
TBX1	4.26E-35	0.06970159	0.283	0.854	8.51E-32	SMC3
CD52	5.32E-35	0.09999473	0.541	0.986	1.06E-31	SMC3
EVI2B	1.02E-34	0.02533058	0.648	0.976	2.03E-31	SMC3
MARCO	1.71E-34	0.05721489	0.117	0.693	3.42E-31	SMC3
MS4A7	3.90E-33	0.02917029	0.107	0.741	7.79E-30	SMC3
MS4A6A	5.49E-33	0.0487426	0.283	0.825	1.10E-29	SMC3
KYNU	1.89E-32	-0.0116369	0.15	0.75	3.78E-29	SMC3

CD3D	3.02E-32	0.10535394	0.645	0.981	6.05E-29	SMC3
RNASE1	3.97E-32	0.16341658	0.306	0.892	7.94E-29	SMC3
CD2	8.17E-32	0.03796919	0.642	0.995	1.63E-28	SMC3
HTR2B	1.08E-31	0.04647274	0.14	0.788	2.17E-28	SMC3
TRBC1	1.20E-31	0.01412497	0.658	1	2.40E-28	SMC3
C1QC	3.78E-31	0.12261812	0.267	0.863	7.57E-28	SMC3
CD83	5.19E-31	0.05988806	0.59	0.962	1.04E-27	SMC3
SLC1A3	5.68E-31	0.01117847	0.244	0.788	1.14E-27	SMC3
GFRA2	9.44E-31	0.01522977	0.202	0.788	1.89E-27	SMC3
FCER1G	1.64E-30	0.07830421	0.13	0.797	3.29E-27	SMC3
JCHAIN	4.42E-30	0.00048815	0.73	1	8.85E-27	SMC3
ACP5	4.85E-30	0.00100661	0.547	0.972	9.71E-27	SMC3
IL7R	8.44E-30	0.13129496	0.723	1	1.69E-26	SMC3
CST7	1.03E-29	0.09293847	0.664	0.967	2.05E-26	SMC3
RHOH	5.70E-29	0.01037114	0.642	0.986	1.14E-25	SMC3
CD86	6.80E-29	-0.0007986	0.554	0.939	1.36E-25	SMC3
LTB	1.03E-28	0.14576789	0.664	0.972	2.07E-25	SMC3
POU2F2	2.19E-28	0.01712178	0.495	0.877	4.38E-25	SMC3
GBP5	3.86E-28	0.01307304	0.554	0.939	7.73E-25	SMC3
LYZ	4.47E-28	0.1992046	0.14	0.745	8.93E-25	SMC3
CD3E	6.72E-28	0.05308939	0.707	0.991	1.34E-24	SMC3
KLRB1	7.73E-28	0.00849191	0.378	0.854	1.55E-24	SMC3
CD27	7.73E-28	-0.0011163	0.674	0.995	1.55E-24	SMC3
MCTP1	1.38E-27	-0.0060859	0.186	0.717	2.76E-24	SMC3
CXorf21	3.36E-27	0.00021193	0.661	0.967	6.72E-24	SMC3
KLRD1	4.14E-27	-0.0345009	0.55	0.939	8.29E-24	SMC3
PMAIP1	4.62E-27	0.00531265	0.446	0.858	9.23E-24	SMC3
CCR6	5.35E-27	0.00133834	0.472	0.925	1.07E-23	SMC3
CD200	7.36E-27	-0.1039926	0.619	0.962	1.47E-23	SMC3
CD163	7.92E-27	-0.0043366	0.339	0.821	1.58E-23	SMC3
CD36	8.43E-27	-0.0093241	0.427	0.887	1.69E-23	SMC3
MMP9	1.27E-26	0.00148609	0.329	0.835	2.53E-23	SMC3
MIR155HG	1.96E-26	0.00107588	0.537	0.929	3.92E-23	SMC3
HLA-DQB1	3.05E-26	0.04724435	0.681	0.972	6.09E-23	SMC3
PTGER3	6.81E-26	-0.0732457	0.564	0.981	1.36E-22	SMC3
FABP5	7.61E-26	-0.0253076	0.192	0.825	1.52E-22	SMC3
DUSP2	9.16E-26	0.13007543	0.726	0.995	1.83E-22	SMC3
LAIR1	9.51E-26	0.0070679	0.322	0.83	1.90E-22	SMC3
CYTIP	1.21E-25	0.08022366	0.73	1	2.42E-22	SMC3
IGHG4	1.82E-25	-0.0027681	0.577	0.972	3.64E-22	SMC3
PALMD	2.34E-25	0.02787967	0.169	0.774	4.68E-22	SMC3
PI16	2.68E-25	0.04610006	0.433	0.849	5.36E-22	SMC3
TREM1	3.45E-25	0.00361916	0.111	0.646	6.89E-22	SMC3
MNDA	3.46E-25	-0.0110859	0.085	0.604	6.92E-22	SMC3
ISG20	3.80E-25	0.0426197	0.609	0.896	7.60E-22	SMC3
HCST	7.59E-25	0.08818308	0.72	0.995	1.52E-21	SMC3
OBP2A	9.48E-25	-0.0026508	0.691	0.995	1.90E-21	SMC3
CTSW	1.22E-24	-0.0120535	0.466	0.882	2.44E-21	SMC3
EPCAM	1.54E-24	0.00501236	0.088	0.59	3.09E-21	SMC3
IL10	1.83E-24	-0.0038952	0.616	0.958	3.66E-21	SMC3
TESC	2.21E-24	-0.0091574	0.394	0.854	4.42E-21	SMC3
CCDC102B	2.64E-24	-0.1571914	0.707	0.986	5.29E-21	SMC3
GZMM	2.71E-24	0.00546126	0.694	1	5.42E-21	SMC3

CCL3	2.78E-24	0.10349406	0.498	0.953	5.56E-21	SMC3
MMP11	2.79E-24	-0.0222795	0.622	0.967	5.57E-21	SMC3
PTCRA	4.49E-24	-0.0026811	0.684	0.986	8.98E-21	SMC3
OASL	6.83E-24	0.0082659	0.661	0.967	1.37E-20	SMC3
MSR1	7.67E-24	0.03315186	0.156	0.726	1.53E-20	SMC3
TRGC2	1.85E-23	0.00496009	0.368	0.816	3.70E-20	SMC3
CCR1	2.05E-23	-0.003886	0.091	0.642	4.09E-20	SMC3
CPVL	2.98E-23	0.00669672	0.518	0.887	5.95E-20	SMC3
GPBAR1	3.75E-23	-0.0091935	0.339	0.792	7.50E-20	SMC3
DHFR	4.84E-23	-0.0348209	0.469	0.892	9.67E-20	SMC3
TNFAIP2	5.00E-23	0.03759148	0.326	0.83	1.00E-19	SMC3
ITGB2	5.80E-23	0.04541357	0.707	1	1.16E-19	SMC3
KLRF1	7.16E-23	-0.0014821	0.691	0.995	1.43E-19	SMC3
IGF2.1	7.52E-23	-0.0012155	0.521	0.925	1.50E-19	SMC3
EDNRA	1.38E-22	0.021455	0.632	0.967	2.76E-19	SMC3
PLAUR	1.58E-22	0.0384091	0.436	0.92	3.16E-19	SMC3
C5AR2	1.73E-22	0.00430653	0.681	0.986	3.46E-19	SMC3
C1QB	2.04E-22	0.21295223	0.215	0.755	4.08E-19	SMC3
TNFSF10	2.79E-22	-0.1041585	0.678	0.991	5.59E-19	SMC3
CD79A	4.31E-22	0.01972444	0.678	0.995	8.61E-19	SMC3
IGHA2	5.28E-22	0.00486195	0.7	1	1.06E-18	SMC3
RASL12	6.13E-22	0.00217663	0.515	0.92	1.23E-18	SMC3
NKG7	6.60E-22	0.0290593	0.505	0.835	1.32E-18	SMC3
C1QA	6.84E-22	0.10725862	0.606	0.972	1.37E-18	SMC3
CLEC12A	7.01E-22	-0.018473	0.016	0.58	1.40E-18	SMC3
RASIP1	7.29E-22	-0.0011528	0.557	0.91	1.46E-18	SMC3
IL1RN	7.80E-22	-0.0080799	0.078	0.571	1.56E-18	SMC3
LTA	9.00E-22	0.00239193	0.619	0.967	1.80E-18	SMC3
FGR	1.20E-21	0.00812629	0.498	0.896	2.39E-18	SMC3
GNLY	1.40E-21	0.13126188	0.202	0.741	2.79E-18	SMC3
S100A9	1.49E-21	0.03312466	0.319	0.802	2.98E-18	SMC3
GREM1	2.09E-21	0.00308872	0.01	0.509	4.19E-18	SMC3
VSIG4	2.24E-21	-0.0073932	0.371	0.835	4.47E-18	SMC3
PTPRE	2.24E-21	0.01027192	0.228	0.774	4.48E-18	SMC3
CD84	3.00E-21	-0.0011908	0.482	0.84	6.00E-18	SMC3
HCK	3.14E-21	-0.0037699	0.554	0.948	6.28E-18	SMC3
EFHD2	3.17E-21	0.01740279	0.456	0.849	6.35E-18	SMC3
RGCC	3.64E-21	0.31966307	0.456	0.986	7.28E-18	SMC3
LST1	4.12E-21	0.01603879	0.329	0.769	8.24E-18	SMC3
IRF7	4.35E-21	0.01621625	0.485	0.896	8.70E-18	SMC3
SLAMF1	5.90E-21	0.00196302	0.632	0.953	1.18E-17	SMC3
KCNJ6	1.08E-20	0.09843514	0.088	0.557	2.17E-17	SMC3
TCIM	1.16E-20	0.06234656	0.264	0.769	2.31E-17	SMC3
SCGB3A2	1.21E-20	-0.0146936	0.648	0.991	2.42E-17	SMC3
HIST1H1B	1.44E-20	-0.0026627	0.049	0.533	2.88E-17	SMC3
NRP2	1.85E-20	0.1178703	0.547	0.967	3.70E-17	SMC3
PLD4	2.13E-20	0.00055612	0.521	0.33	4.27E-17	SMC3
FAM13C	3.48E-20	-0.03774	0.537	0.934	6.97E-17	SMC3
NLRP3	4.33E-20	0.02119735	0.697	1	8.66E-17	SMC3
BEX1	5.04E-20	0.07295709	0.199	0.717	1.01E-16	SMC3
DRD4	5.17E-20	-0.0047429	0.267	0.708	1.03E-16	SMC3
APOE	5.22E-20	0.30137307	0.505	1	1.04E-16	SMC3
TCL1A	5.92E-20	-0.001551	0.684	0.981	1.18E-16	SMC3

DKK1	6.37E-20	0.01980108	0.016	0.547	1.27E-16	SMC3
CXCL1	7.10E-20	0.0412154	0.687	0.995	1.42E-16	SMC3
EZH2	7.59E-20	0.00699818	0.505	0.887	1.52E-16	SMC3
MATK	9.38E-20	0.00071334	0.358	0.792	1.88E-16	SMC3
HES5	9.65E-20	0.02982429	0.544	0.297	1.93E-16	SMC3
CYGB	9.83E-20	0.01122922	0.221	0.731	1.97E-16	SMC3
ENHO	1.32E-19	0.01304638	0.264	0.764	2.64E-16	SMC3
AURKA	1.53E-19	-0.0080873	0.394	0.811	3.06E-16	SMC3
CD48	2.10E-19	0.34822276	0.733	1	4.20E-16	SMC3
DUSP5	2.15E-19	-0.0058955	0.717	1	4.30E-16	SMC3
GZMK	3.52E-19	0.17714378	0.73	0.995	7.04E-16	SMC3
AOAH	3.63E-19	-0.0058203	0.577	0.953	7.25E-16	SMC3
MPEG1	3.64E-19	0.00392003	0.098	0.613	7.28E-16	SMC3
RERGL	4.92E-19	-0.0841589	0.619	0.991	9.84E-16	SMC3
TLR2	4.94E-19	-0.0053496	0.609	0.958	9.88E-16	SMC3
HSPA6	5.41E-19	-0.0616056	0.274	0.731	1.08E-15	SMC3
FNDC1	6.96E-19	-0.0064023	0.655	0.995	1.39E-15	SMC3
SLC39A8	7.10E-19	0.01904777	0.502	0.854	1.42E-15	SMC3
THEMIS2	1.08E-18	0.00256145	0.625	0.967	2.16E-15	SMC3
MARCKSL1	1.65E-18	0.02807672	0.58	0.92	3.29E-15	SMC3
FPR2	1.66E-18	0.00418167	0.668	0.972	3.32E-15	SMC3
CXCL8	1.67E-18	0.0769011	0.15	0.66	3.34E-15	SMC3
HLA-DRB5	2.09E-18	0.09355732	0.713	1	4.18E-15	SMC3
HPGDS	2.24E-18	0.00525417	0.619	0.972	4.48E-15	SMC3
FMO3	2.77E-18	-0.0054556	0.603	0.981	5.54E-15	SMC3
BIRC3	3.30E-18	0.03064891	0.485	0.868	6.60E-15	SMC3
SERPINE1	3.37E-18	0.04470712	0.72	0.991	6.74E-15	SMC3
CDCA5	3.42E-18	-0.0012555	0.642	0.972	6.84E-15	SMC3
KANK3	3.43E-18	0.03457638	0.45	0.283	6.85E-15	SMC3
PAIP2B	3.62E-18	0.01746262	0.463	0.83	7.23E-15	SMC3
GPR34	3.87E-18	-0.0009997	0.661	0.948	7.73E-15	SMC3
SLCO2B1	3.95E-18	0.006674	0.674	0.991	7.91E-15	SMC3
MZB1	4.61E-18	-0.0083563	0.694	1	9.22E-15	SMC3
CXCL3	4.89E-18	0.20676146	0.261	0.731	9.77E-15	SMC3
HLA-DMB	5.11E-18	0.01192037	0.404	0.863	1.02E-14	SMC3
CLSPN	5.31E-18	-0.0029909	0.664	0.972	1.06E-14	SMC3
CCL4L2	5.58E-18	0.20758219	0.671	0.972	1.12E-14	SMC3
LILRB3	8.66E-18	-0.0375172	0.221	0.646	1.73E-14	SMC3
SCIMP	9.59E-18	-0.0040192	0.515	0.915	1.92E-14	SMC3
GZMA	1.09E-17	0.1182549	0.73	1	2.18E-14	SMC3
MT1G	1.11E-17	0.05984186	0.479	0.816	2.23E-14	SMC3
HBB	1.25E-17	-0.0009085	0.101	0.547	2.49E-14	SMC3
CSRP2	1.28E-17	-0.1093206	0.648	0.939	2.56E-14	SMC3
TNF	1.35E-17	0.08323816	0.668	0.991	2.69E-14	SMC3
LILRB4	1.37E-17	0.01290537	0.039	0.538	2.74E-14	SMC3
CSF2RA	1.43E-17	-0.0109894	0.091	0.585	2.85E-14	SMC3
IFI44L	1.53E-17	-0.0402256	0.697	0.995	3.06E-14	SMC3
PECAM1	1.54E-17	-0.0087386	0.163	0.632	3.09E-14	SMC3
MS4A4A	1.58E-17	0.0451145	0.537	0.892	3.16E-14	SMC3
IL32	1.62E-17	0.29267105	0.625	0.939	3.24E-14	SMC3
CD4	1.90E-17	-0.0138698	0.248	0.708	3.81E-14	SMC3
PLEK	2.05E-17	0.01060461	0.296	0.726	4.10E-14	SMC3
IGSF6	2.25E-17	0.01814647	0.635	0.967	4.49E-14	SMC3

CD69	2.26E-17	0.16849988	0.72	1	4.52E-14	SMC3
CASQ2	2.38E-17	-0.0280933	0.534	0.925	4.76E-14	SMC3
TNFRSF18	2.73E-17	-0.0373157	0.593	0.953	5.47E-14	SMC3
COLEC11	3.38E-17	-0.0116655	0.55	0.877	6.77E-14	SMC3
KLHL23	3.44E-17	-0.0318203	0.567	0.887	6.88E-14	SMC3
SERPINA1	3.78E-17	0.01212409	0.616	0.92	7.56E-14	SMC3
WFDC1	4.06E-17	0.0375321	0.28	0.774	8.12E-14	SMC3
GAPT	5.01E-17	0.01427932	0.583	0.953	1.00E-13	SMC3
RBP5	5.25E-17	-0.0137711	0.531	0.929	1.05E-13	SMC3
HGF	5.37E-17	-0.0834036	0.645	0.991	1.07E-13	SMC3
TIGIT	5.58E-17	0.00861773	0.355	0.755	1.12E-13	SMC3
ISG15	5.82E-17	0.05654068	0.629	0.981	1.16E-13	SMC3
OAS1	6.33E-17	-0.0552726	0.443	0.844	1.27E-13	SMC3
IGHV3-20	6.50E-17	-0.0003523	0.651	0.953	1.30E-13	SMC3
APLN	6.61E-17	-0.0012493	0.655	0.943	1.32E-13	SMC3
TRBC2	8.77E-17	0.15799276	0.687	0.991	1.75E-13	SMC3
PILRA	8.77E-17	-0.0156142	0.127	0.58	1.75E-13	SMC3
RASGEF1B	1.25E-16	0.0029466	0.554	0.906	2.51E-13	SMC3
OR51E1	1.37E-16	-0.0218801	0.593	0.929	2.74E-13	SMC3
MS4A1	1.39E-16	0.00994733	0.704	1	2.78E-13	SMC3
AREG	1.46E-16	0.00664059	0.541	0.906	2.91E-13	SMC3
FCRL2	1.57E-16	0.00198329	0.687	0.986	3.14E-13	SMC3
IGLC3	1.62E-16	0.15905361	0.749	1	3.24E-13	SMC3
SLCO2A1	1.69E-16	-0.0044747	0.544	0.953	3.38E-13	SMC3
NPY2R	1.77E-16	-0.0675219	0.277	0.231	3.54E-13	SMC3
SIRPB1	2.10E-16	0.00694655	0.378	0.778	4.20E-13	SMC3
CSTA	2.65E-16	-0.0046808	0.127	0.599	5.29E-13	SMC3
ZNF385D	2.72E-16	-0.0064271	0.329	0.882	5.44E-13	SMC3
C19orf38	2.82E-16	-0.0092254	0.577	0.873	5.65E-13	SMC3
FYB1	2.92E-16	0.02436488	0.619	0.91	5.83E-13	SMC3
AC007406.3	3.07E-16	-0.0062034	0.684	0.986	6.14E-13	SMC3
PDE4B	3.16E-16	-0.0027028	0.362	0.797	6.31E-13	SMC3
KRT17	3.33E-16	0.01421922	0.599	0.915	6.66E-13	SMC3
IFNG	3.44E-16	0.04343043	0.7	1	6.88E-13	SMC3
MMP12	4.05E-16	-0.0061815	0.01	0.486	8.10E-13	SMC3
TDO2	4.30E-16	-0.0483596	0.697	1	8.61E-13	SMC3
ZNF683	5.69E-16	0.00373273	0.651	0.962	1.14E-12	SMC3
PLIN5	6.56E-16	0.02623584	0.179	0.618	1.31E-12	SMC3
GADD45G	6.72E-16	0.16840045	0.583	0.934	1.34E-12	SMC3
DIRC3	6.75E-16	-0.0061246	0.505	0.816	1.35E-12	SMC3
LPL	7.48E-16	-0.0008525	0.554	0.892	1.50E-12	SMC3
TK1	7.56E-16	-0.0066809	0.505	0.887	1.51E-12	SMC3
KLRC1	7.75E-16	-0.0005412	0.697	0.995	1.55E-12	SMC3
CDT1	8.43E-16	0.00437518	0.508	0.84	1.69E-12	SMC3
GPR183	8.67E-16	0.03975927	0.678	0.953	1.73E-12	SMC3
CD1A	8.80E-16	-0.0008667	0.684	0.986	1.76E-12	SMC3
DGKI	1.38E-15	0.27120398	0.192	0.623	2.76E-12	SMC3
LYN	1.40E-15	-0.0079958	0.635	0.953	2.80E-12	SMC3
ANGPT2	1.45E-15	-0.1989779	0.658	1	2.90E-12	SMC3
WFDC2	1.52E-15	0.13231664	0.583	0.906	3.03E-12	SMC3
TGFBI	2.28E-15	-0.0732112	0.606	0.925	4.56E-12	SMC3
SLC26A4	2.57E-15	-0.0061773	0.472	0.858	5.14E-12	SMC3
FAM107A	2.74E-15	0.17903389	0.306	0.825	5.48E-12	SMC3

SPINK1	3.21E-15	-0.0016286	0.622	0.929	6.41E-12	SMC3
GZMB	3.41E-15	-0.0191397	0.681	0.986	6.82E-12	SMC3
CDH5	3.84E-15	-0.016643	0.661	0.976	7.69E-12	SMC3
RAD51AP1	3.85E-15	0.00289149	0.283	0.693	7.70E-12	SMC3
NCF1	3.89E-15	0.01122726	0.606	0.962	7.79E-12	SMC3
POSTN	3.99E-15	-0.0367918	0.619	1	7.98E-12	SMC3
IGHM	5.12E-15	0.00479178	0.632	0.906	1.02E-11	SMC3
IGKV3-20	5.29E-15	0.00178904	0.684	0.995	1.06E-11	SMC3
LAPTM5	5.79E-15	0.1035779	0.45	0.83	1.16E-11	SMC3
PLAU	6.36E-15	0.00904691	0.59	0.882	1.27E-11	SMC3
FAM241A	7.37E-15	0.06450001	0.423	0.821	1.47E-11	SMC3
TMEM158	7.43E-15	0.01497184	0.228	0.675	1.49E-11	SMC3
SPC24	8.32E-15	0.00884347	0.065	0.542	1.66E-11	SMC3
C7	8.40E-15	-0.0652924	0.694	0.995	1.68E-11	SMC3
RNASE6	9.02E-15	0.00219384	0.368	0.802	1.80E-11	SMC3
COLEC12	1.30E-14	0.01639643	0.632	0.92	2.60E-11	SMC3
TNFRSF9	1.53E-14	0.02039238	0.583	0.887	3.05E-11	SMC3
VMO1	1.63E-14	0.01070689	0.485	0.854	3.27E-11	SMC3
ACKR1	1.65E-14	-0.0413757	0.296	0.203	3.30E-11	SMC3
MCM10	1.74E-14	-0.0010803	0.661	0.943	3.47E-11	SMC3
TREM2	1.77E-14	-0.00042	0.41	0.769	3.54E-11	SMC3
NCAPH	1.77E-14	0.00782967	0.7	1	3.55E-11	SMC3
XCL2	1.80E-14	0.01895141	0.684	0.991	3.60E-11	SMC3
MARCH1	1.88E-14	0.01316963	0.658	0.943	3.75E-11	SMC3
CRTAM	2.00E-14	-0.0032557	0.697	1	4.00E-11	SMC3
SLIT2	2.04E-14	0.07615683	0.599	0.358	4.08E-11	SMC3
CFD	2.06E-14	0.00219389	0.306	0.184	4.13E-11	SMC3
BCHE	2.27E-14	0.01253435	0.306	0.75	4.54E-11	SMC3
AC025164.1	2.52E-14	0.05397245	0.635	0.943	5.03E-11	SMC3
FAM30A	3.50E-14	0.00162822	0.697	0.981	7.00E-11	SMC3
HIGD1B	3.68E-14	-0.0239459	0.691	0.986	7.36E-11	SMC3
CCL4	3.99E-14	0.67162557	0.788	1	7.98E-11	SMC3
NRN1	4.20E-14	-0.0292761	0.554	0.892	8.40E-11	SMC3
TRDC	4.48E-14	-0.0251134	0.664	0.991	8.96E-11	SMC3
FST	5.12E-14	0.05382985	0.583	0.415	1.02E-10	SMC3
SPINK2	5.17E-14	-0.0088765	0.697	0.991	1.03E-10	SMC3
AK5	6.05E-14	0.00546816	0.166	0.108	1.21E-10	SMC3
RGS1	6.81E-14	0.04386192	0.629	0.976	1.36E-10	SMC3
IL13RA2	6.94E-14	0.16691144	0.521	0.892	1.39E-10	SMC3
CREB5	7.35E-14	-0.0635854	0.586	0.406	1.47E-10	SMC3
FHL2	8.12E-14	0.0321129	0.414	0.962	1.62E-10	SMC3
CCL26	9.11E-14	-0.0118933	0.645	0.995	1.82E-10	SMC3
OSM	9.43E-14	0.01786604	0.345	0.698	1.89E-10	SMC3
ADAMDEC1	9.78E-14	0.00050628	0.55	0.901	1.96E-10	SMC3
PTGER2	9.96E-14	0.04082714	0.189	0.637	1.99E-10	SMC3
PAEP	1.07E-13	-0.0003977	0.635	0.962	2.13E-10	SMC3
BATF3	1.29E-13	0.02617508	0.534	0.84	2.58E-10	SMC3
NR1H3	1.55E-13	0.00107865	0.502	0.821	3.10E-10	SMC3
FMO2	1.58E-13	-0.3173315	0.638	0.981	3.16E-10	SMC3
CPM	1.60E-13	0.0002263	0.681	0.995	3.19E-10	SMC3
ADAP2	1.71E-13	0.02209996	0.638	0.939	3.43E-10	SMC3
CTSC	1.97E-13	0.06963874	0.73	1	3.93E-10	SMC3
STEAP4	2.06E-13	-0.1330672	0.697	1	4.11E-10	SMC3

ALOX5	2.06E-13	0.00338816	0.055	0.495	4.13E-10	SMC3
FCGBP	2.10E-13	0.00740664	0.16	0.585	4.21E-10	SMC3
SPOCK2	2.38E-13	0.01522579	0.681	0.972	4.75E-10	SMC3
AGR2	2.55E-13	0.15051335	0.537	0.915	5.11E-10	SMC3
ENC1	2.76E-13	-0.0232924	0.704	0.995	5.53E-10	SMC3
MFAP2	3.45E-13	-0.0233657	0.528	0.877	6.89E-10	SMC3
VAMP8	3.85E-13	0.02728987	0.43	0.736	7.71E-10	SMC3
CTHRC1	4.15E-13	-0.0385719	0.681	0.991	8.30E-10	SMC3
PKIB	4.15E-13	0.01380016	0.378	0.712	8.30E-10	SMC3
ICOS	4.30E-13	0.00937232	0.651	0.986	8.60E-10	SMC3
SFRP5	4.49E-13	-0.0046106	0.098	0.533	8.97E-10	SMC3
ABRA	4.51E-13	0.00159139	0.075	0.528	9.01E-10	SMC3
BATF	4.57E-13	-0.0117804	0.423	0.811	9.13E-10	SMC3
PLA2G5	4.74E-13	0.02556771	0.169	0.59	9.48E-10	SMC3
AL583785.1	4.78E-13	-0.0023032	0.638	0.991	9.55E-10	SMC3
CFH	4.93E-13	-0.1517475	0.71	1	9.86E-10	SMC3
MAP3K7CL	5.10E-13	-0.0627	0.668	0.953	1.02E-09	SMC3
FCGR3A	5.26E-13	0.06755283	0.14	0.623	1.05E-09	SMC3
ADGRF5	5.87E-13	-0.0281732	0.687	0.995	1.17E-09	SMC3
THSD7A	5.87E-13	-0.0019836	0.687	0.976	1.17E-09	SMC3
CTSG	7.73E-13	-0.0027324	0.671	0.981	1.55E-09	SMC3
ESM1	7.94E-13	-0.0019499	0.645	0.943	1.59E-09	SMC3
CLDN1	8.73E-13	-0.0047678	0.606	0.877	1.75E-09	SMC3
LOXL2	9.08E-13	0.00123537	0.515	0.83	1.82E-09	SMC3
CD40LG	9.40E-13	-0.0019419	0.537	0.887	1.88E-09	SMC3
DUSP23	9.44E-13	-0.0647003	0.362	0.811	1.89E-09	SMC3
EGFL7	9.52E-13	-0.03059	0.528	0.892	1.90E-09	SMC3
HOPX	9.96E-13	-0.0009599	0.7	0.995	1.99E-09	SMC3
CNGA1	1.00E-12	0.02946749	0.57	0.901	2.01E-09	SMC3
CCL17	1.24E-12	0.00095639	0.622	0.958	2.48E-09	SMC3
LCN6	1.29E-12	-0.001018	0.557	0.882	2.58E-09	SMC3
FAM162B	1.32E-12	-0.0369488	0.658	0.967	2.65E-09	SMC3
CXCL10	1.40E-12	0.00892936	0.678	0.991	2.80E-09	SMC3
KRT16	1.46E-12	-0.003509	0.635	0.953	2.91E-09	SMC3
MX2	1.57E-12	-0.002053	0.59	0.882	3.13E-09	SMC3
EGFL6	1.62E-12	-0.0082675	0.697	0.991	3.24E-09	SMC3
PTAFR	1.83E-12	-0.0045875	0.616	0.915	3.65E-09	SMC3
CCL20	2.01E-12	0.01126376	0.02	0.5	4.02E-09	SMC3
IGLC7	2.15E-12	-0.0005536	0.583	0.873	4.29E-09	SMC3
EDNRB	2.15E-12	-0.0251542	0.681	0.953	4.30E-09	SMC3
CDH19	2.25E-12	0.00274927	0.068	0.542	4.51E-09	SMC3
LTC4S	2.42E-12	0.14294974	0.293	0.783	4.85E-09	SMC3
ANKRD28	2.99E-12	-0.0102121	0.694	0.953	5.98E-09	SMC3
UBE2C	3.20E-12	-0.0038718	0.691	0.995	6.40E-09	SMC3
NR4A3	3.53E-12	0.00710953	0.616	0.976	7.05E-09	SMC3
MIA	3.61E-12	-0.0033596	0.029	0.462	7.22E-09	SMC3
BANK1	4.19E-12	0.01807107	0.684	0.967	8.38E-09	SMC3
SERPINI1	4.30E-12	-0.0414534	0.518	0.844	8.60E-09	SMC3
TFPI2	4.32E-12	0.01796267	0.635	0.906	8.64E-09	SMC3
TNFSF11	4.37E-12	0.00863285	0.541	0.934	8.74E-09	SMC3
IL23A	4.58E-12	0.00126932	0.531	0.868	9.15E-09	SMC3
STEAP1	4.74E-12	-0.0209672	0.697	0.991	9.48E-09	SMC3
LINC02362	5.01E-12	-0.0021321	0.684	0.991	1.00E-08	SMC3

CD68	5.09E-12	0.09581272	0.599	0.896	1.02E-08	SMC3
TOP2A	5.15E-12	-0.0004007	0.55	0.858	1.03E-08	SMC3
GGT5	5.29E-12	-0.0915458	0.704	1	1.06E-08	SMC3
CCDC3	5.44E-12	-0.2117927	0.7	0.967	1.09E-08	SMC3
C3AR1	5.47E-12	0.00983705	0.638	0.948	1.09E-08	SMC3
AQP3	6.35E-12	0.0258234	0.655	0.915	1.27E-08	SMC3
PRDM1	6.66E-12	-0.0105681	0.603	0.981	1.33E-08	SMC3
CXCR4	6.71E-12	0.22525911	0.765	0.995	1.34E-08	SMC3
CD163L1	6.77E-12	-0.0027869	0.603	0.915	1.35E-08	SMC3
IL17B	7.09E-12	0.0484875	0.521	0.84	1.42E-08	SMC3
ACOXL	7.77E-12	-0.0004113	0.658	0.962	1.55E-08	SMC3
FFAR3	7.95E-12	-0.00019	0.625	0.934	1.59E-08	SMC3
TNFRSF4	8.84E-12	-0.0001491	0.397	0.736	1.77E-08	SMC3
CTSS	9.37E-12	0.02125572	0.544	0.854	1.87E-08	SMC3
SCGN	9.56E-12	-0.0147394	0.678	0.986	1.91E-08	SMC3
IL2RB	1.01E-11	0.00803468	0.664	0.972	2.02E-08	SMC3
ADCYAP1	1.02E-11	0.04639458	0.612	0.915	2.04E-08	SMC3
CCL21	1.09E-11	-0.0670414	0.681	0.967	2.18E-08	SMC3
CYTL1	1.13E-11	0.19791505	0.332	0.882	2.26E-08	SMC3
ABCC9	1.21E-11	-0.0519361	0.537	0.844	2.41E-08	SMC3
LINC02185	1.27E-11	0.00871314	0.469	0.382	2.53E-08	SMC3
KRT19	1.28E-11	0.36506517	0.362	0.825	2.57E-08	SMC3
LINC01094	1.34E-11	0.00766555	0.537	0.849	2.69E-08	SMC3
ANPEP	1.51E-11	-0.0049077	0.648	0.953	3.01E-08	SMC3
CYP1B1	1.64E-11	0.14846241	0.743	0.958	3.28E-08	SMC3
NUF2	1.67E-11	0.0153243	0.107	0.08	3.34E-08	SMC3
CHODL	1.73E-11	-0.0045184	0.674	0.934	3.46E-08	SMC3
FAM133A	1.73E-11	-0.0127341	0.531	0.854	3.46E-08	SMC3
GUCY1A2	2.63E-11	-0.0070305	0.664	0.976	5.26E-08	SMC3
LINC01857	2.76E-11	-0.0001474	0.681	0.995	5.53E-08	SMC3
SUGCT	2.80E-11	-0.0497097	0.648	0.967	5.61E-08	SMC3
FABP3	2.81E-11	0.04781556	0.173	0.627	5.61E-08	SMC3
BCL2A1	3.07E-11	0.00274602	0.14	0.542	6.13E-08	SMC3
CDH2	3.63E-11	-0.0004311	0.41	0.335	7.27E-08	SMC3
LILRB2	4.02E-11	0.00971979	0.606	0.943	8.04E-08	SMC3
FILIP1	4.39E-11	-0.153991	0.619	0.906	8.79E-08	SMC3
SELP	4.42E-11	-0.0059091	0.358	0.679	8.84E-08	SMC3
PHLDA1	4.67E-11	-0.0069526	0.573	0.892	9.35E-08	SMC3
SLC18A2	4.84E-11	-0.0002044	0.651	0.925	9.69E-08	SMC3
SMPX	4.97E-11	0.16649619	0.381	0.849	9.94E-08	SMC3
COL9A3	5.95E-11	0.01161378	0.443	0.354	1.19E-07	SMC3
TNFAIP3	6.10E-11	0.09329325	0.664	0.925	1.22E-07	SMC3
TNFAIP6	6.71E-11	-0.0125728	0.668	1	1.34E-07	SMC3
JAM2	7.56E-11	0.06319117	0.505	0.392	1.51E-07	SMC3
APOD	8.39E-11	0.01111342	0.508	0.429	1.68E-07	SMC3
SCG3	8.39E-11	-8.28E-05	0.586	0.887	1.68E-07	SMC3
SLC8A1	8.69E-11	0.02215278	0.524	0.934	1.74E-07	SMC3
SMC2	9.12E-11	-0.0550891	0.42	0.802	1.82E-07	SMC3
FHL5	9.83E-11	-0.0612182	0.463	0.816	1.97E-07	SMC3
HLA-DMA	1.00E-10	0.12401709	0.391	0.816	2.00E-07	SMC3
ADM5	1.02E-10	0.00636905	0.068	0.505	2.04E-07	SMC3
MGST1	1.05E-10	-0.0295767	0.423	0.802	2.09E-07	SMC3
TUBB2B	1.17E-10	-0.0167171	0.388	0.302	2.35E-07	SMC3

IGLV2-8	1.23E-10	-7.34E-05	0.557	0.858	2.47E-07	SMC3
NFIL3	1.27E-10	0.03870458	0.622	0.887	2.53E-07	SMC3
HMGB3	1.28E-10	0.002095	0.202	0.599	2.57E-07	SMC3
RGS13	1.38E-10	-0.0004731	0.518	0.792	2.77E-07	SMC3
SLC7A2	1.46E-10	-0.0998117	0.629	0.892	2.93E-07	SMC3
RGS16	1.47E-10	0.03157928	0.726	1	2.94E-07	SMC3
CD7	1.54E-10	0.00326279	0.684	1	3.09E-07	SMC3
CYFIP2	1.68E-10	-0.0790509	0.489	0.816	3.37E-07	SMC3
GALNT15	1.77E-10	0.00612539	0.476	0.425	3.54E-07	SMC3
HLA-DRB1	1.90E-10	0.21075843	0.616	0.976	3.81E-07	SMC3
PIM2	2.10E-10	0.02652764	0.231	0.632	4.20E-07	SMC3
APOC1	2.12E-10	0.10308136	0.173	0.613	4.25E-07	SMC3
PCDHB4	2.23E-10	0.06686561	0.717	0.929	4.45E-07	SMC3
HLA-DQA2	3.45E-10	0.0238296	0.691	0.995	6.89E-07	SMC3
TNNI2	3.76E-10	0.00601549	0.58	0.849	7.53E-07	SMC3
BACE2	3.83E-10	0.0592173	0.671	0.953	7.67E-07	SMC3
PROCR	3.98E-10	0.03055358	0.407	0.783	7.96E-07	SMC3
CXCL9	4.17E-10	-0.0297947	0.691	1	8.35E-07	SMC3
AL138899.1	4.49E-10	-0.0009046	0.668	0.962	8.98E-07	SMC3
DEPDC1B	5.68E-10	0.0002071	0.358	0.278	1.14E-06	SMC3
CALHM6	5.95E-10	0.00194761	0.609	0.906	1.19E-06	SMC3
OXTR	6.13E-10	-0.0021297	0.629	0.972	1.23E-06	SMC3
NAMPT	6.42E-10	0.02673938	0.518	0.887	1.28E-06	SMC3
CBLN4	6.51E-10	-0.0337997	0.101	0.486	1.30E-06	SMC3
LRG1	6.71E-10	0.00616317	0.583	0.896	1.34E-06	SMC3
HMGA1	6.73E-10	0.03861239	0.55	0.854	1.35E-06	SMC3
SLC9B2	6.86E-10	-0.0002358	0.466	0.83	1.37E-06	SMC3
SNX10	6.87E-10	0.01469366	0.505	0.788	1.37E-06	SMC3
SMIM25	6.89E-10	0.01237344	0.13	0.557	1.38E-06	SMC3
SRGN	6.98E-10	0.12083078	0.779	0.995	1.40E-06	SMC3
DAB2	7.20E-10	-0.0903766	0.505	0.84	1.44E-06	SMC3
SERPINB9	7.30E-10	0.0230499	0.622	0.467	1.46E-06	SMC3
AL078590.2	8.05E-10	-0.0001304	0.625	0.934	1.61E-06	SMC3
CD8A	8.25E-10	0.01830026	0.697	0.995	1.65E-06	SMC3
TSPAN15	9.78E-10	0.00388918	0.495	0.797	1.96E-06	SMC3
TFEC	1.00E-09	-0.0001588	0.078	0.495	2.00E-06	SMC3
NCF2	1.03E-09	0.03073193	0.397	0.75	2.06E-06	SMC3
CXCL2	1.05E-09	-0.0719291	0.606	0.868	2.11E-06	SMC3
NPPC	1.07E-09	-0.0040266	0.511	0.774	2.14E-06	SMC3
HSPB8	1.09E-09	0.09485814	0.668	0.906	2.19E-06	SMC3
HAVCR2	1.37E-09	0.00331664	0.622	0.887	2.73E-06	SMC3
HOXA5	1.38E-09	0.00079303	0.309	0.274	2.75E-06	SMC3
CCL19	1.39E-09	-0.0863296	0.707	1	2.77E-06	SMC3
CD3G	1.46E-09	0.05562398	0.704	0.995	2.91E-06	SMC3
GINS2	1.48E-09	0.00792956	0.632	0.934	2.97E-06	SMC3
CD1E	1.49E-09	0.00409171	0.564	0.873	2.98E-06	SMC3
GXYLT2	1.65E-09	-0.0197778	0.472	0.816	3.30E-06	SMC3
FBXO32	1.68E-09	0.0155891	0.671	0.943	3.35E-06	SMC3
AC133644.2	1.84E-09	0.00261351	0.691	0.995	3.69E-06	SMC3
SBSPON	1.97E-09	0.13634638	0.554	0.873	3.93E-06	SMC3
LXN	2.12E-09	-0.0346098	0.59	0.901	4.23E-06	SMC3
HAS1	2.18E-09	-6.09E-05	0.651	0.948	4.35E-06	SMC3
ACAN	2.22E-09	-0.0894954	0.169	0.255	4.43E-06	SMC3

CYYR1	2.24E-09	0.00180651	0.674	0.939	4.48E-06	SMC3
SPTSSB	2.30E-09	-0.0010481	0.521	0.877	4.60E-06	SMC3
GPX3	2.39E-09	0.06534684	0.684	0.934	4.78E-06	SMC3
CP	2.55E-09	0.22034167	0.805	0.995	5.10E-06	SMC3
OLR1	2.77E-09	0.00441132	0.134	0.491	5.54E-06	SMC3
S100A8	2.84E-09	0.04087914	0.212	0.608	5.68E-06	SMC3
QPCT	2.97E-09	-0.0032677	0.655	0.967	5.93E-06	SMC3
CRISPLD1	3.02E-09	0.05083211	0.671	0.991	6.04E-06	SMC3
HLA-DQA1	3.06E-09	0.09720644	0.433	0.731	6.13E-06	SMC3
FAM167B	3.22E-09	0.00666549	0.691	0.981	6.43E-06	SMC3
PRDM8	3.32E-09	-0.0038596	0.521	0.868	6.65E-06	SMC3
HDC	3.38E-09	0.00665738	0.622	0.91	6.77E-06	SMC3
AC104024.1	3.82E-09	-1.38E-05	0.417	0.689	7.65E-06	SMC3
GBP4	3.85E-09	-0.0476515	0.645	0.91	7.70E-06	SMC3
IGHG2	4.08E-09	0.07018145	0.739	1	8.15E-06	SMC3
TNFSF14	4.21E-09	-0.0310465	0.332	0.656	8.41E-06	SMC3
EGR3	4.50E-09	0.03592572	0.707	0.976	9.00E-06	SMC3
CD72	4.82E-09	0.01630083	0.436	0.769	9.65E-06	SMC3
CD300E	4.84E-09	-0.0033167	0.182	0.538	9.68E-06	SMC3
CHI3L1	4.94E-09	-0.0055066	0.694	1	9.89E-06	SMC3
ANGPT1	5.12E-09	-0.081046	0.655	0.948	1.02E-05	SMC3
HLA-DPA1	5.74E-09	0.19428679	0.759	0.995	1.15E-05	SMC3
NMB	5.83E-09	-0.0064266	0.674	0.896	1.17E-05	SMC3
PCLAF	6.32E-09	-0.0030386	0.358	0.693	1.26E-05	SMC3
CD247	6.44E-09	-0.0034984	0.697	0.995	1.29E-05	SMC3
CD33	7.02E-09	0.02073161	0.42	0.75	1.40E-05	SMC3
FUT7	7.05E-09	0.00385346	0.537	0.849	1.41E-05	SMC3
IGHG3	7.54E-09	0.07134414	0.645	0.976	1.51E-05	SMC3
CD38	7.61E-09	0.0026935	0.694	0.995	1.52E-05	SMC3
IGLV3-1	7.62E-09	-0.0016451	0.603	0.91	1.52E-05	SMC3
FCGR1A	7.66E-09	0.09859231	0.065	0.443	1.53E-05	SMC3
SMIM5	8.02E-09	0.00085924	0.14	0.132	1.60E-05	SMC3
SPP1	8.14E-09	0.20436354	0.368	0.849	1.63E-05	SMC3
MND1	8.32E-09	0.03037049	0.218	0.585	1.66E-05	SMC3
ESCO2	8.79E-09	-0.0009238	0.59	0.873	1.76E-05	SMC3
ARHGAP29	8.87E-09	-0.2724007	0.697	0.939	1.77E-05	SMC3
IFI27	9.54E-09	0.02924085	0.619	0.892	1.91E-05	SMC3
CD300A	9.79E-09	0.00788898	0.049	0.467	1.96E-05	SMC3
INHBB	1.01E-08	0.00396473	0.182	0.514	2.02E-05	SMC3
VCAM1	1.01E-08	0.16888785	0.492	0.967	2.03E-05	SMC3
TRIB1	1.02E-08	-0.0051152	0.534	0.83	2.03E-05	SMC3
CCL5	1.04E-08	0.30151037	0.752	1	2.08E-05	SMC3
ACE	1.13E-08	0.00646063	0.531	0.844	2.26E-05	SMC3
ADM	1.14E-08	0.02566071	0.593	0.887	2.27E-05	SMC3
CEMIP	1.15E-08	-0.0066667	0.681	0.991	2.29E-05	SMC3
MT1H	1.21E-08	0.00103703	0.397	0.693	2.43E-05	SMC3
C5orf46	1.33E-08	-0.0256343	0.697	1	2.66E-05	SMC3
PLA1A	1.37E-08	-0.0015635	0.694	1	2.73E-05	SMC3
MFAP5	1.49E-08	-0.0131008	0.322	0.632	2.97E-05	SMC3
ARC	1.56E-08	0.00966986	0.612	0.92	3.11E-05	SMC3
FOLR2	1.56E-08	0.00296003	0.635	0.906	3.12E-05	SMC3
ITM2A	1.62E-08	0.15968936	0.606	0.906	3.25E-05	SMC3
PDCD1	1.67E-08	0.00792126	0.264	0.575	3.33E-05	SMC3

IGLL5	1.76E-08	0.00601623	0.407	0.712	3.51E-05	SMC3
KCNMB1	1.79E-08	-0.0753452	0.765	0.972	3.58E-05	SMC3
CD70	1.83E-08	0.01056431	0.261	0.58	3.65E-05	SMC3
PDXK	2.03E-08	0.04773718	0.518	0.854	4.07E-05	SMC3
NPW	2.12E-08	0.00301612	0.023	0.439	4.23E-05	SMC3
SMOC2	2.20E-08	-0.251128	0.642	0.925	4.40E-05	SMC3
CDKN1A	2.24E-08	0.05404947	0.733	0.948	4.49E-05	SMC3
P2RY13	2.26E-08	0.00945442	0.599	0.835	4.51E-05	SMC3
CXCR6	2.26E-08	0.00236585	0.678	0.991	4.53E-05	SMC3
HMMR	2.28E-08	-0.0037996	0.42	0.684	4.57E-05	SMC3
AQP9	2.39E-08	0.00530695	0.221	0.58	4.77E-05	SMC3
AC004540.2	2.46E-08	-0.0060124	0.388	0.67	4.92E-05	SMC3
MOXD1	2.59E-08	0.05127276	0.492	0.764	5.17E-05	SMC3
THBS2	2.59E-08	-0.0553112	0.752	0.986	5.18E-05	SMC3
PDK4	2.67E-08	0.02195621	0.349	0.33	5.34E-05	SMC3
TACSTD2	2.88E-08	0.00486323	0.215	0.184	5.76E-05	SMC3
IGKV1-39	3.02E-08	-0.0010203	0.678	0.986	6.04E-05	SMC3
CDKN2A	3.33E-08	0.04142308	0.57	0.439	6.66E-05	SMC3
PODN	3.49E-08	-0.056173	0.423	0.363	6.98E-05	SMC3
CADM3	3.63E-08	-0.0159611	0.59	0.887	7.26E-05	SMC3
GATA2	3.78E-08	0.0413056	0.482	0.821	7.57E-05	SMC3
SPON2	3.80E-08	-0.0092238	0.606	0.92	7.61E-05	SMC3
IGLC2	4.15E-08	0.18060618	0.899	1	8.29E-05	SMC3
SCG2	4.15E-08	-0.0100401	0.691	1	8.30E-05	SMC3
LAMP5	4.23E-08	-0.0002426	0.375	0.726	8.45E-05	SMC3
POU2AF1	4.26E-08	-0.0001013	0.371	0.665	8.52E-05	SMC3
UCHL1	4.32E-08	-0.0379251	0.515	0.406	8.65E-05	SMC3
TRBV28	4.67E-08	-0.0001871	0.625	0.892	9.35E-05	SMC3
AC243960.1	4.86E-08	0.00070508	0.658	0.962	9.73E-05	SMC3
THY1	5.20E-08	-0.1312426	0.707	1	0.0001039	SMC3
LINC00924	5.33E-08	-0.0062891	0.453	0.769	0.00010661	SMC3
RAMP1	5.44E-08	0.63177736	0.954	0.962	0.00010883	SMC3
OLFM1	5.47E-08	-0.0258183	0.537	0.821	0.00010933	SMC3
ASGR2	5.70E-08	0.00025864	0.104	0.132	0.00011391	SMC3
HLA-DRA	5.82E-08	0.41160749	0.655	1	0.00011643	SMC3
CBR3	5.84E-08	0.02848334	0.642	0.906	0.00011678	SMC3
IL3RA	6.01E-08	-0.0004749	0.616	0.892	0.0001202	SMC3
LILRB1	6.23E-08	0.00559475	0.309	0.627	0.00012454	SMC3
PCDH10	6.26E-08	-0.0414938	0.674	0.939	0.00012511	SMC3
S100A3	6.56E-08	0.03509198	0.573	0.821	0.00013119	SMC3
TM4SF1	6.57E-08	-0.4000026	0.837	0.986	0.00013142	SMC3
FCER2	7.02E-08	0.00163597	0.02	0.41	0.00014039	SMC3
PCOLCE2	7.33E-08	-0.1129045	0.511	0.41	0.0001465	SMC3
GDF10	7.64E-08	0.17488183	0.479	0.892	0.00015281	SMC3
AC093895.1	7.69E-08	-3.65E-05	0.375	0.623	0.00015385	SMC3
HELLS	8.24E-08	-0.0187617	0.564	0.821	0.0001648	SMC3
CBLN1	8.56E-08	-0.0016426	0.518	0.792	0.00017121	SMC3
TMEM100	8.58E-08	-0.0586199	0.375	0.745	0.00017165	SMC3
IL1B	8.68E-08	0.04056439	0.3	0.627	0.00017367	SMC3
IBSP	8.81E-08	-0.0069736	0.394	0.736	0.00017616	SMC3
TMEM176B	9.67E-08	0.02398954	0.557	0.849	0.00019349	SMC3
BUB1	9.98E-08	-0.001411	0.694	0.995	0.00019965	SMC3
TSPAN8	1.11E-07	0.0101658	0.326	0.712	0.00022248	SMC3

TCHH	1.14E-07	-0.0005996	0.56	0.825	0.00022874	SMC3
LUM	1.17E-07	0.11160367	0.723	1	0.00023437	SMC3
INSIG1	1.18E-07	0.06104452	0.531	0.783	0.00023598	SMC3
IGHV3-74	1.21E-07	0.00066783	0.114	0.094	0.00024144	SMC3
PTP4A3	1.22E-07	-0.0193845	0.661	0.892	0.00024401	SMC3
EMILIN2	1.23E-07	-0.0113203	0.336	0.637	0.00024608	SMC3
PTGER1	1.25E-07	-0.0016415	0.554	0.882	0.00025039	SMC3
SMPDL3B	1.25E-07	-0.0013665	0.684	0.981	0.00025079	SMC3
IGHV3-23	1.35E-07	-0.0017105	0.052	0.198	0.00027038	SMC3
GABRD	1.39E-07	-0.0011438	0.528	0.858	0.0002773	SMC3
ECM1	1.57E-07	-0.0158697	0.674	0.929	0.00031341	SMC3
IRF8	1.61E-07	0.01477937	0.479	0.726	0.00032209	SMC3
CALCRL	1.62E-07	-0.0448735	0.684	1	0.00032396	SMC3
TFRC	1.65E-07	-0.0051037	0.124	0.462	0.00032924	SMC3
XCL1	1.65E-07	-0.0283373	0.684	0.887	0.00032966	SMC3
EMCN	1.69E-07	-0.0114981	0.697	1	0.00033721	SMC3
BMP3	1.75E-07	-0.0003424	0.642	0.962	0.0003493	SMC3
HCAR2	1.82E-07	-0.0007917	0.179	0.557	0.0003636	SMC3
PTTG1	1.83E-07	-0.010963	0.625	0.934	0.00036537	SMC3
TNFSF13B	1.83E-07	0.00662262	0.707	1	0.00036537	SMC3
MMP13	1.85E-07	-7.82E-05	0.557	0.811	0.00037004	SMC3
LINC01485	1.85E-07	-0.0007667	0.671	0.953	0.00037062	SMC3
PTHLH	2.03E-07	0.05223588	0.583	0.896	0.00040682	SMC3
KRT14	2.15E-07	0.00318368	0.645	0.962	0.00042925	SMC3
NCKAP5	2.27E-07	0.00868599	0.606	0.868	0.00045326	SMC3
PLAC8	2.46E-07	0.00853639	0.593	0.906	0.00049252	SMC3
ZNF331	2.55E-07	0.09952998	0.417	0.75	0.00050991	SMC3
PID1	2.82E-07	-0.0629026	0.622	0.887	0.00056333	SMC3
CDK1	2.87E-07	-0.0021775	0.267	0.566	0.00057413	SMC3
FAP	2.95E-07	-0.0274762	0.71	0.981	0.00059053	SMC3
GUCY1B1	2.98E-07	-0.1376013	0.73	0.972	0.00059568	SMC3
TROAP	3.00E-07	0.00436794	0.596	0.906	0.00059999	SMC3
DAPL1	3.00E-07	0.17056754	0.583	0.453	0.00060039	SMC3
GCA	3.05E-07	-0.0294155	0.603	0.877	0.000609	SMC3
IGFBP3	3.21E-07	0.34301052	0.485	0.877	0.00064188	SMC3
SGCA	3.21E-07	-0.0201029	0.697	0.995	0.00064191	SMC3
ALDH1A1	3.23E-07	-0.0050644	0.622	0.901	0.00064552	SMC3
CLEC4A	3.28E-07	-0.0036754	0.453	0.759	0.0006554	SMC3
PAPPA	3.31E-07	0.01347496	0.567	0.877	0.00066281	SMC3
ANK2	3.31E-07	0.03136449	0.534	0.83	0.00066281	SMC3
LAIR2	3.33E-07	0.0001957	0.635	0.925	0.00066592	SMC3
ECEL1	3.42E-07	-0.0010718	0.619	0.896	0.00068315	SMC3
MME	3.42E-07	-0.1140397	0.645	0.542	0.00068321	SMC3
AGT	3.74E-07	-0.1616671	0.71	1	0.00074826	SMC3
RBM47	3.89E-07	-0.0044182	0.091	0.439	0.00077701	SMC3
LINC02446	4.05E-07	-7.79E-05	0.59	0.868	0.00080983	SMC3
SLC16A3	4.06E-07	0.03121612	0.313	0.66	0.00081256	SMC3
KCNQ1OT1	4.09E-07	0.06112875	0.629	0.538	0.00081734	SMC3
LMNB1	4.25E-07	0.01287792	0.564	0.825	0.00085028	SMC3
RBP7	4.61E-07	0.03179255	0.524	0.783	0.00092226	SMC3
FAM111B	4.92E-07	-0.0002988	0.381	0.689	0.00098456	SMC3
PDE5A	5.09E-07	0.49905379	0.987	0.981	0.00101744	SMC3
EDN1	5.13E-07	-0.0512924	0.505	0.783	0.00102512	SMC3

ADH4	5.18E-07	-0.002471	0.15	0.123	0.0010363	SMC3
IGHV1-69D	5.53E-07	0.00152705	0.668	0.962	0.00110576	SMC3
TBXAS1	5.65E-07	-0.0166763	0.365	0.627	0.00112967	SMC3
LIF	5.70E-07	-0.0036807	0.42	0.66	0.00114018	SMC3
C19orf33	5.87E-07	-0.0017041	0.397	0.368	0.0011732	SMC3
SLC16A10	6.01E-07	-0.0238773	0.316	0.656	0.00120181	SMC3
CYTOR	6.06E-07	-0.0148596	0.762	1	0.00121246	SMC3
KCNK15	6.17E-07	0.09531515	0.687	0.958	0.00123453	SMC3
AC233755.1	6.22E-07	-3.51E-05	0.684	0.972	0.00124467	SMC3
ALPL	6.70E-07	0.00064541	0.117	0.127	0.00134062	SMC3
CKAP2L	7.07E-07	0.00035116	0.404	0.726	0.00141406	SMC3
TFF3	7.52E-07	0.00219302	0.622	0.925	0.00150334	SMC3
C2	7.57E-07	0.00030067	0.436	0.717	0.00151401	SMC3
NGFR	7.71E-07	0.17437497	0.195	0.509	0.00154192	SMC3
VEGFA	7.71E-07	-0.0361285	0.596	0.91	0.00154216	SMC3
TNFRSF13C	8.15E-07	0.00358625	0.681	0.986	0.00162947	SMC3
LYVE1	8.61E-07	-0.0129043	0.642	0.929	0.00172274	SMC3
HOXB5	8.70E-07	-0.0040995	0.606	0.887	0.00173978	SMC3
SFRP2	9.14E-07	0.68442508	0.717	1	0.00182741	SMC3
WNT2	9.25E-07	-8.70E-05	0.443	0.717	0.00184964	SMC3
CXCL14	9.50E-07	-0.0224168	0.195	0.175	0.00190035	SMC3
KCTD12	9.55E-07	0.09835199	0.505	0.797	0.00190972	SMC3
NR2F1	9.89E-07	0.01029761	0.319	0.637	0.00197729	SMC3
LINC01781	9.99E-07	0.00367365	0.684	0.967	0.00199873	SMC3
CSF3R	1.01E-06	-0.04893	0.342	0.637	0.00201198	SMC3
GGH	1.04E-06	-0.0188763	0.564	0.575	0.00208189	SMC3
QPRT	1.05E-06	-0.0682663	0.375	0.311	0.00210153	SMC3
RAB3C	1.11E-06	0.00022309	0.671	0.943	0.00222096	SMC3
FGF18	1.11E-06	0.00209111	0.098	0.434	0.00222346	SMC3
IGKV3-11	1.15E-06	-0.0003445	0.674	0.953	0.00230159	SMC3
CDCA3	1.16E-06	-0.0026548	0.687	0.981	0.002316	SMC3
LY86	1.24E-06	0.00602063	0.459	0.731	0.00247229	SMC3
ICAM2	1.27E-06	0.00607368	0.322	0.627	0.00254081	SMC3
FAM69B	1.37E-06	-0.005203	0.384	0.717	0.0027369	SMC3
GRIA2	1.40E-06	-0.0206904	0.515	0.797	0.00280533	SMC3
FFAR2	1.45E-06	0.00027914	0.068	0.123	0.00289659	SMC3
TMEM70	1.51E-06	0.05380439	0.554	0.821	0.00302392	SMC3
IL34	1.54E-06	-0.0353513	0.681	0.925	0.00307813	SMC3
ULBP1	1.55E-06	-0.0001004	0.629	0.925	0.00310667	SMC3
KCNMA1	1.56E-06	0.01579777	0.606	0.906	0.00311477	SMC3
SDF2L1	1.60E-06	-0.022817	0.573	0.929	0.00319055	SMC3
MMRN2	1.66E-06	-0.063564	0.505	0.741	0.00331863	SMC3
RASSF9	1.68E-06	0.063407	0.205	0.571	0.00335836	SMC3
SLPI	1.69E-06	0.25940331	0.681	0.991	0.00338347	SMC3
DPEP1	1.75E-06	-2.33E-05	0.456	0.712	0.00349076	SMC3
CX3CL1	1.84E-06	0.08609357	0.485	0.792	0.00368443	SMC3
CYP27A1	1.89E-06	0.00625013	0.195	0.557	0.00377183	SMC3
LINC01480	1.89E-06	0.0040311	0.694	0.995	0.00377348	SMC3
TM4SF18	2.03E-06	-0.0031444	0.694	1	0.00406606	SMC3
CD8B	2.10E-06	0.01862729	0.681	0.995	0.00419514	SMC3
NEFM	2.12E-06	-0.000237	0.668	0.953	0.0042333	SMC3
C1QTNF3	2.28E-06	-0.0020493	0.199	0.542	0.00456124	SMC3
CRISPLD2	2.30E-06	0.01375164	0.55	0.533	0.00459458	SMC3

IL2	2.39E-06	0.00800281	0.678	0.967	0.00477652	SMC3
AC007952.4	2.42E-06	0.00379618	0.593	0.835	0.00483552	SMC3
IGSF21	2.45E-06	-0.0019209	0.036	0.373	0.0049009	SMC3
NCR3	2.46E-06	-0.0061527	0.482	0.778	0.00491204	SMC3
IGHV4-34	2.47E-06	-1.10E-05	0.616	0.91	0.00494393	SMC3
KNL1	2.77E-06	-6.67E-05	0.515	0.783	0.00553081	SMC3
CLDN10	3.36E-06	-0.0014746	0.554	0.486	0.00672379	SMC3
CD93	3.46E-06	0.01636512	0.485	0.453	0.00691525	SMC3
EGLN3	3.93E-06	-0.0118848	0.489	0.745	0.00785057	SMC3
HRH1	4.02E-06	0.15644952	0.375	0.722	0.00804796	SMC3
RSPO2	4.27E-06	0.28811854	0.495	0.807	0.00853944	SMC3
MAFF	4.58E-06	0.21120179	0.749	0.948	0.00916668	SMC3
FN1	4.64E-06	-0.0766583	0.837	0.976	0.00927811	SMC3
CA4	4.83E-06	9.85E-05	0.107	0.104	0.00966391	SMC3
CNKSR3	4.93E-06	-0.0311618	0.573	0.476	0.0098599	SMC3
SCX	4.95E-06	0.35299614	0.371	0.783	0.00989955	SMC3
CDH11	5.03E-06	-0.0583874	0.749	0.995	0.01005626	SMC3
KCNN4	5.24E-06	0.00060564	0.59	0.873	0.01048623	SMC3
CRTAC1	5.40E-06	-0.03991	0.697	0.995	0.01079161	SMC3
IGFBP1	5.57E-06	-3.80E-06	0.446	0.708	0.01113152	SMC3
CHI3L2	5.68E-06	0.00983932	0.658	0.934	0.01135296	SMC3
PCK1	5.68E-06	0.29923688	0.704	0.637	0.01135826	SMC3
PLIN2	6.03E-06	0.11646297	0.756	0.962	0.0120505	SMC3
CH25H	6.13E-06	0.03050602	0.635	0.948	0.01226796	SMC3
COMP	6.16E-06	-0.0009999	0.492	0.764	0.01232274	SMC3
HSPG2	6.20E-06	0.08608907	0.332	0.368	0.01239737	SMC3
LOX	6.23E-06	-5.42E-05	0.42	0.41	0.01246595	SMC3
NRARP	6.28E-06	-0.0109518	0.436	0.741	0.01256844	SMC3
IGKC	6.29E-06	0.25563251	0.984	1	0.01258825	SMC3
AL356417.2	6.93E-06	0.00124581	0.684	0.986	0.01385761	SMC3
SIGLEC15	6.95E-06	-0.0091302	0.358	0.344	0.01389656	SMC3
TNFSF15	7.12E-06	0.02578389	0.671	0.972	0.01423452	SMC3
FSTL3	7.15E-06	0.0169209	0.476	0.453	0.01429336	SMC3
TNFRSF17	7.34E-06	-0.000504	0.681	0.972	0.01467072	SMC3
IL2RA	7.36E-06	-0.0001966	0.651	0.953	0.01471601	SMC3
PLA2G2D	7.95E-06	-0.000101	0.401	0.585	0.01590259	SMC3
AC104407.1	7.96E-06	-0.0511187	0.339	0.377	0.0159106	SMC3
PLPP3	8.39E-06	-0.0302649	0.358	0.358	0.01678269	SMC3
ALCAM	8.44E-06	-0.020585	0.658	0.925	0.01687631	SMC3
TPX2	8.45E-06	-0.0016997	0.427	0.689	0.01689916	SMC3
RGS18	8.73E-06	-5.19E-05	0.534	0.816	0.01745544	SMC3
LUZP2	9.33E-06	-0.0257584	0.414	0.434	0.01865882	SMC3
PAMR1	9.33E-06	-0.1266152	0.645	0.505	0.0186627	SMC3
THBS4	9.88E-06	-0.0016289	0.378	0.717	0.01975462	SMC3
GBP1	9.96E-06	-0.0593113	0.622	0.906	0.01991623	SMC3
SLC2A5	1.00E-05	-0.0007526	0.241	0.542	0.02000155	SMC3
SLC7A7	1.03E-05	-0.0070045	0.254	0.557	0.02068653	SMC3
CYP26B1	1.04E-05	0.05391394	0.29	0.618	0.02087838	SMC3
MAD2L1	1.09E-05	0.16283004	0.72	0.967	0.02173791	SMC3
SPARCL1	1.11E-05	-0.2377358	0.827	0.995	0.02226784	SMC3
RSPO3	1.17E-05	-0.0023479	0.407	0.675	0.02330312	SMC3
CCR7	1.17E-05	0.02152683	0.707	0.995	0.02334676	SMC3
CCL3L1	1.17E-05	-0.0105365	0.489	0.774	0.02341154	SMC3

GPRC5A	1.20E-05	0.11210727	0.508	0.467	0.02392697	SMC3
AC040970.1	1.21E-05	0.0101402	0.173	0.509	0.0241484	SMC3
SSTR2	1.21E-05	0.00327763	0.436	0.722	0.02419416	SMC3
SOX4	1.23E-05	0.15837596	0.645	0.943	0.02463507	SMC3
PLXNA4	1.24E-05	0.05046421	0.143	0.524	0.02481844	SMC3
GNG11	1.28E-05	0.0077169	0.772	0.972	0.02565377	SMC3
IGHV2-5	1.30E-05	-2.02E-07	0.127	0.288	0.02597083	SMC3
COL14A1	1.36E-05	-0.0760114	0.808	0.976	0.02714997	SMC3
APOC2	1.43E-05	-0.0003605	0.147	0.226	0.02858676	SMC3
RND1	1.46E-05	0.02011162	0.42	0.698	0.02914832	SMC3
ATP2B1-AS1	1.53E-05	0.03495826	0.498	0.741	0.03064548	SMC3
PLD5	1.54E-05	0.09877558	0.479	0.387	0.03074975	SMC3
DIO2	1.55E-05	-0.0114539	0.612	0.873	0.03091236	SMC3
RAMP2	1.55E-05	0.14279578	0.293	0.627	0.03104701	SMC3
SRPX	1.61E-05	0.02710566	0.687	0.915	0.03227418	SMC3
HSD11B1	1.66E-05	-0.0123949	0.476	0.741	0.03325249	SMC3
NOSTRIN	1.69E-05	0.00492469	0.508	0.448	0.03379246	SMC3
ASGR1	1.69E-05	0.00811635	0.104	0.434	0.03385563	SMC3
TREML1	1.76E-05	0.00704681	0.684	0.981	0.03510827	SMC3
AC023157.3	1.81E-05	-0.0444692	0.717	0.892	0.0361509	SMC3
FJX1	1.81E-05	0.05450023	0.635	0.825	0.03620249	SMC3
LMO2	1.92E-05	0.66077887	0.857	0.906	0.03840974	SMC3
PMCH	1.96E-05	-5.18E-05	0.134	0.283	0.03926025	SMC3
JAML	1.98E-05	0.04724094	0.195	0.519	0.03952685	SMC3
TGM2	2.00E-05	-0.1460338	0.72	0.981	0.04002124	SMC3
ZCCHC12	2.09E-05	-0.0024488	0.586	0.892	0.04175211	SMC3
CCL7	2.12E-05	-0.0153677	0	0.292	0.04241933	SMC3
PLCXD3	2.12E-05	-0.0021571	0.326	0.637	0.04248359	SMC3
MMP7	2.13E-05	0.00651511	0.593	0.906	0.04250314	SMC3
GSTM5	2.14E-05	-0.1270889	0.218	0.325	0.04282482	SMC3
RCAN1	2.18E-05	0.26627529	0.573	0.863	0.04358015	SMC3
STMN2	2.20E-05	-0.0006098	0.681	0.981	0.0439686	SMC3
SIGLEC1	2.21E-05	-0.0122784	0.573	0.802	0.04420352	SMC3
ERN1	2.28E-05	0.03730148	0.326	0.637	0.04552438	SMC3
MMP1	2.40E-05	-5.57E-05	0.567	0.797	0.04795603	SMC3
HHIP	2.42E-05	0.00527753	0.433	0.731	0.04835484	SMC3
CPXM2	2.45E-05	0.04510359	0.55	0.783	0.04898534	SMC3
CRYM	2.46E-05	0.01267043	0.648	0.604	0.04920306	SMC3
MMP23B	2.48E-05	-0.0480428	0.567	0.481	0.04960446	SMC3
SPI1	4.59E-26	0.02874405	0.238	0.877	9.18E-23	SMC4
HIST1H2AL	6.70E-26	-0.0118429	0.659	0.986	1.34E-22	SMC4
DPEP1	7.03E-25	-2.53E-05	0.139	0.74	1.41E-21	SMC4
CD84	7.11E-25	0.00730769	0.245	0.918	1.42E-21	SMC4
SLCO2B1	2.13E-24	-0.0027081	0.626	0.986	4.25E-21	SMC4
S100A12	5.37E-24	0.00121592	0.242	0.877	1.07E-20	SMC4
MMP13	6.81E-23	-0.0018385	0.222	0.863	1.36E-19	SMC4
TIMD4	8.68E-23	-5.18E-05	0.278	0.945	1.74E-19	SMC4
FCER1A	1.03E-22	-0.0137078	0.457	0.89	2.06E-19	SMC4
NMUR2	1.03E-22	-0.0678328	0.159	0.795	2.07E-19	SMC4
LYZ	1.68E-22	0.030863	0.089	0.795	3.37E-19	SMC4
IGFBP1	3.70E-22	-1.31E-05	0.113	0.671	7.40E-19	SMC4
FOLR2	4.16E-22	0.00930376	0.517	0.932	8.32E-19	SMC4
GBP5	5.15E-22	-0.0028006	0.715	1	1.03E-18	SMC4

LAPTM5	6.65E-22	0.07483301	0.47	0.986	1.33E-18	SMC4
CCL17	1.24E-21	-5.37E-05	0.209	0.836	2.49E-18	SMC4
EMILIN2	1.49E-21	-0.0119434	0.44	0.945	2.99E-18	SMC4
AC233755.1	2.24E-21	-0.0019463	0.722	0.959	4.49E-18	SMC4
OBP2A	2.69E-21	-7.06E-05	0.272	0.959	5.38E-18	SMC4
NCAPG	3.03E-21	-0.0035677	0.036	0.753	6.07E-18	SMC4
AURKB	4.04E-21	-0.000224	0.139	0.836	8.09E-18	SMC4
EVI2B	5.13E-21	0.05367034	0.735	1	1.03E-17	SMC4
SCT	6.78E-21	-9.33E-06	0.175	0.795	1.36E-17	SMC4
IGHV4-28	6.79E-21	2.36E-07	0.149	0.726	1.36E-17	SMC4
LILRB4	8.79E-21	0.01070779	0.262	0.822	1.76E-17	SMC4
TLR2	1.01E-20	-0.0013025	0.245	0.849	2.02E-17	SMC4
P2RY6	1.64E-20	-0.0398246	0.344	0.932	3.28E-17	SMC4
ACP5	2.83E-20	0.00764589	0.437	0.932	5.66E-17	SMC4
CD36	2.93E-20	-0.0031641	0.394	0.904	5.85E-17	SMC4
SIX1	3.99E-20	-0.0125704	0.371	0.986	7.99E-17	SMC4
KIR2DL1	7.87E-20	-5.20E-06	0.109	0.603	1.57E-16	SMC4
EREG	8.32E-20	0.03240235	0.5	0.849	1.66E-16	SMC4
C1QA	8.62E-20	-0.0479392	0.54	1	1.72E-16	SMC4
GINS2	8.85E-20	-0.0095554	0.742	1	1.77E-16	SMC4
DNAAF1	1.30E-19	0.02218071	0.156	0.822	2.60E-16	SMC4
LINC02362	1.52E-19	-0.0065435	0.699	1	3.03E-16	SMC4
AC023157.3	1.81E-19	-0.053269	0.49	0.986	3.61E-16	SMC4
CYP1B1	3.28E-19	0.00840187	0.361	0.877	6.57E-16	SMC4
HS6ST3	3.40E-19	0.00191348	0.255	0.164	6.81E-16	SMC4
TYROBP	4.24E-19	0.05499738	0.666	0.973	8.47E-16	SMC4
LINC00520	5.43E-19	-1.40E-05	0.278	0.945	1.09E-15	SMC4
CD52	7.29E-19	0.16580457	0.762	1	1.46E-15	SMC4
SSUH2	7.51E-19	-6.19E-05	0.063	0.466	1.50E-15	SMC4
GOLM1	1.43E-18	-0.0467852	0.649	0.986	2.85E-15	SMC4
SERPINA1	1.61E-18	0.01675937	0.48	0.836	3.22E-15	SMC4
MSR1	1.68E-18	-0.0107386	0.02	0.712	3.35E-15	SMC4
AREG	1.78E-18	0.13374179	0.288	0.877	3.55E-15	SMC4
VAMP8	1.85E-18	0.04802437	0.666	0.973	3.70E-15	SMC4
FYB1	1.90E-18	0.05021328	0.791	1	3.80E-15	SMC4
SGK1	2.29E-18	0.00807931	0.53	0.973	4.57E-15	SMC4
ACKR1	4.22E-18	-0.3294945	0.583	0.205	8.44E-15	SMC4
CLMP	5.44E-18	-0.0699413	0.725	0.959	1.09E-14	SMC4
FCGR2B	7.09E-18	-0.0015026	0.662	0.959	1.42E-14	SMC4
KIF23	7.87E-18	0.00291296	0.278	0.918	1.57E-14	SMC4
PRDM8	8.54E-18	-0.0014691	0.43	0.945	1.71E-14	SMC4
AC091271.1	1.02E-17	-0.2098826	0.49	0.945	2.04E-14	SMC4
CD2	1.10E-17	0.11406718	0.781	1	2.20E-14	SMC4
PRDM1	1.11E-17	0.04478957	0.609	1	2.22E-14	SMC4
NPTX2	1.18E-17	0.08154432	0.341	0.918	2.35E-14	SMC4
LINC01485	1.42E-17	-0.0002526	0.272	0.849	2.84E-14	SMC4
IGHV4-59	1.44E-17	6.73E-07	0.275	0.945	2.88E-14	SMC4
IGKV1-17	1.63E-17	-7.59E-07	0.265	0.945	3.26E-14	SMC4
PLAC8	2.06E-17	0.00513739	0.666	1	4.11E-14	SMC4
SNX10	2.41E-17	0.01336307	0.371	0.836	4.83E-14	SMC4
CD1E	2.52E-17	-5.88E-06	0.291	0.959	5.05E-14	SMC4
KIF11	2.78E-17	-0.0104117	0.775	0.986	5.56E-14	SMC4
CD48	2.79E-17	0.08614439	0.791	1	5.58E-14	SMC4

IGKV3-15	3.14E-17	7.60E-05	0.275	0.945	6.29E-14	SMC4
PTAFR	4.71E-17	-0.0107469	0.752	1	9.42E-14	SMC4
CCL18	4.81E-17	0.0041156	0.348	0.795	9.62E-14	SMC4
CCL20	5.60E-17	0.00798718	0.046	0.726	1.12E-13	SMC4
C1QB	7.06E-17	0.09564372	0.262	0.836	1.41E-13	SMC4
MCM10	9.06E-17	-0.0003157	0.334	0.863	1.81E-13	SMC4
IGHV4-34	9.68E-17	-9.87E-05	0.298	0.932	1.94E-13	SMC4
ANPEP	1.11E-16	-0.0387914	0.705	1	2.21E-13	SMC4
ICOS	1.84E-16	0.02392184	0.636	1	3.67E-13	SMC4
PLIN5	2.02E-16	-0.0004765	0.258	0.904	4.05E-13	SMC4
AP003481.1	2.17E-16	-1.56E-05	0.291	0.945	4.35E-13	SMC4
GALNT5	2.35E-16	-0.0388838	0.675	0.973	4.70E-13	SMC4
AC004817.3	2.74E-16	-0.0002519	0.315	0.877	5.49E-13	SMC4
BUB1B	2.79E-16	0.00021739	0.252	0.877	5.59E-13	SMC4
PGM5-AS1	2.97E-16	0.0484347	0.391	0.11	5.94E-13	SMC4
IL3RA	3.30E-16	-0.0044835	0.46	0.89	6.59E-13	SMC4
ACTC1	4.20E-16	0.06447139	0.493	0.904	8.40E-13	SMC4
MIR181A1H	4.95E-16	-0.0029628	0.434	0.959	9.90E-13	SMC4
CTSG	4.96E-16	6.32E-05	0.487	0.959	9.91E-13	SMC4
CXCR4	5.15E-16	0.34644359	0.815	1	1.03E-12	SMC4
SHTN1	5.21E-16	-0.0049183	0.387	0.973	1.04E-12	SMC4
FAM167B	6.07E-16	-0.0245417	0.748	1	1.21E-12	SMC4
CTSW	8.65E-16	0.02525136	0.662	1	1.73E-12	SMC4
IL7R	9.17E-16	0.39739499	0.801	1	1.83E-12	SMC4
IL4I1	9.43E-16	-0.0023679	0.517	0.808	1.89E-12	SMC4
S100A3	9.50E-16	0.01590901	0.414	0.918	1.90E-12	SMC4
KIFC1	9.87E-16	-0.002066	0.762	0.986	1.97E-12	SMC4
IGLV1-47	1.06E-15	-0.0001569	0.235	0.904	2.12E-12	SMC4
BCAT1	1.23E-15	-0.0226405	0.623	1	2.47E-12	SMC4
CD83	1.27E-15	-0.0253155	0.513	1	2.54E-12	SMC4
EGR3	1.73E-15	-0.0871427	0.626	0.986	3.45E-12	SMC4
HMMR	2.26E-15	-0.0013859	0.325	0.945	4.53E-12	SMC4
AQP2	2.50E-15	-5.06E-05	0.083	0.452	5.00E-12	SMC4
FUT7	2.78E-15	0.0008129	0.308	0.877	5.56E-12	SMC4
CSF1R	3.92E-15	0.01884444	0.318	0	7.84E-12	SMC4
MS4A7	4.22E-15	-0.0076836	0.222	0.712	8.44E-12	SMC4
CPVL	4.39E-15	0.04961667	0.596	1	8.78E-12	SMC4
BCL2L14	5.51E-15	0.00025776	0.248	0.863	1.10E-11	SMC4
C15orf48	6.00E-15	0.00299265	0.397	0.767	1.20E-11	SMC4
LRRC39	6.28E-15	0.00271948	0.119	0.082	1.26E-11	SMC4
CD247	6.31E-15	0.02289313	0.762	1	1.26E-11	SMC4
IGHV1-24	6.36E-15	0.00011525	0.136	0.589	1.27E-11	SMC4
ABCA10	9.72E-15	-0.0064557	0.338	0.082	1.94E-11	SMC4
SLC1A7	1.28E-14	0.00175183	0.52	0.205	2.56E-11	SMC4
PLEK	1.38E-14	0.0196473	0.252	0.699	2.75E-11	SMC4
LY86	1.50E-14	0.00757518	0.487	0.973	3.00E-11	SMC4
CDC45	2.01E-14	0.00675846	0.252	0.863	4.02E-11	SMC4
RNASE1	2.02E-14	-0.1123582	0.384	0.904	4.04E-11	SMC4
ZNF385D	2.43E-14	-0.0857466	0.533	0.986	4.86E-11	SMC4
IL1R1	2.44E-14	-0.1984057	0.798	0.986	4.87E-11	SMC4
GFPT2	2.53E-14	0.01921394	0.341	0.89	5.06E-11	SMC4
GSTM5	3.06E-14	-0.0857634	0.702	0.247	6.11E-11	SMC4
TSPAN15	3.50E-14	-0.0017536	0.573	0.918	6.99E-11	SMC4

PPY	3.83E-14	-1.25E-06	0.063	0.397	7.66E-11	SMC4
ESCO2	4.38E-14	0.00311272	0.536	0.959	8.76E-11	SMC4
TNFAIP3	4.41E-14	0.08695427	0.798	0.986	8.83E-11	SMC4
TRAC	4.77E-14	0.04185031	0.808	1	9.54E-11	SMC4
GPC3	6.66E-14	-0.2504531	0.54	0.205	1.33E-10	SMC4
GZMB	6.68E-14	0.02417632	0.735	1	1.34E-10	SMC4
CPM	8.89E-14	0.00049117	0.649	0.986	1.78E-10	SMC4
CRYM	9.05E-14	-0.0016961	0.07	0.479	1.81E-10	SMC4
PTX3	9.17E-14	0.12342725	0.699	0.973	1.83E-10	SMC4
CD3D	9.69E-14	0.08916958	0.798	1	1.94E-10	SMC4
CXCL14	9.87E-14	0.00197227	0.142	0.055	1.97E-10	SMC4
FAM69B	1.05E-13	-0.0097095	0.649	1	2.10E-10	SMC4
PAIP2B	1.47E-13	0.0122445	0.232	0.726	2.94E-10	SMC4
MCEMP1	1.62E-13	3.14E-05	0.003	0.671	3.25E-10	SMC4
ALDH1A2	2.03E-13	0.07435258	0.414	0.918	4.05E-10	SMC4
PMAIP1	2.40E-13	0.01762254	0.762	1	4.79E-10	SMC4
TESC	2.74E-13	-0.031272	0.619	0.877	5.47E-10	SMC4
FPR1	2.79E-13	0.01852794	0.427	0.849	5.58E-10	SMC4
LDB2	3.51E-13	0.02378906	0.715	0.973	7.01E-10	SMC4
IL10	3.78E-13	0.01146735	0.457	0.808	7.55E-10	SMC4
IGHV1-46	4.32E-13	-0.0003959	0.427	0.959	8.63E-10	SMC4
MGST1	4.51E-13	0.07993843	0.675	0.329	9.02E-10	SMC4
HCST	4.66E-13	0.1086883	0.778	1	9.32E-10	SMC4
FPR2	4.69E-13	-0.0006045	0.341	0.904	9.38E-10	SMC4
AC100858.3	6.25E-13	-3.00E-05	0.036	0.301	1.25E-09	SMC4
MMP11	6.41E-13	-0.0056367	0.685	0.986	1.28E-09	SMC4
RETN	6.60E-13	-0.0051122	0.033	0.699	1.32E-09	SMC4
IGHV3-20	6.94E-13	8.71E-06	0.308	0.959	1.39E-09	SMC4
CD37	7.19E-13	0.10414906	0.781	0.986	1.44E-09	SMC4
PIM2	9.19E-13	0.06784275	0.305	0.849	1.84E-09	SMC4
OLFM1	9.78E-13	-0.0126753	0.705	0.411	1.96E-09	SMC4
PCDH10	1.05E-12	-0.044468	0.841	0.986	2.09E-09	SMC4
DUSP2	1.06E-12	0.20383421	0.785	1	2.12E-09	SMC4
IGLV2-8	1.10E-12	1.72E-06	0.374	0.959	2.20E-09	SMC4
SLC39A8	1.24E-12	0.00526062	0.652	0.986	2.49E-09	SMC4
C1QTNF4	1.39E-12	-0.0116806	0.596	0.959	2.77E-09	SMC4
TNF	1.40E-12	0.08216072	0.778	1	2.79E-09	SMC4
DHFR	1.60E-12	-0.0968018	0.209	0.767	3.20E-09	SMC4
S100A9	1.61E-12	0.07976228	0.387	0.877	3.22E-09	SMC4
KLRC1	1.67E-12	-0.0036038	0.781	1	3.34E-09	SMC4
VWA5A	1.74E-12	0.01184002	0.503	0.918	3.47E-09	SMC4
KRT19	1.79E-12	0.00377182	0.159	0.63	3.59E-09	SMC4
MRC1	1.80E-12	0.00611473	0.513	0.849	3.60E-09	SMC4
IGHA1	2.21E-12	-0.0937707	0.884	1	4.42E-09	SMC4
SLN	2.26E-12	0.00344288	0.334	0.767	4.51E-09	SMC4
MS4A4A	2.28E-12	0.00880802	0.672	0.959	4.56E-09	SMC4
LINC02446	2.46E-12	0.00507098	0.321	0.932	4.92E-09	SMC4
FCER1G	3.06E-12	0.08590696	0.682	0.973	6.11E-09	SMC4
CENPF	4.10E-12	-0.0046627	0.503	0.808	8.21E-09	SMC4
MLPH	4.34E-12	-0.0112824	0.394	0.877	8.69E-09	SMC4
AQP3	4.70E-12	-0.0359329	0.546	0.89	9.39E-09	SMC4
ENPP1	4.74E-12	-0.1242126	0.599	1	9.48E-09	SMC4
TFEC	4.75E-12	0.00486549	0.536	0.849	9.51E-09	SMC4

CETP	5.33E-12	0.00535068	0.199	0.068	1.07E-08	SMC4
ITLN1	5.50E-12	-0.0343245	0.119	0.658	1.10E-08	SMC4
NRXN1	6.86E-12	4.36E-05	0.321	0.932	1.37E-08	SMC4
BATF	7.19E-12	0.04293807	0.712	1	1.44E-08	SMC4
PHLDA1	7.46E-12	-0.0611205	0.758	0.973	1.49E-08	SMC4
CRTAC1	8.50E-12	-0.1151123	0.815	1	1.70E-08	SMC4
ALPL	1.00E-11	-0.0039622	0.113	0.671	2.00E-08	SMC4
CSF3	1.01E-11	0.00714679	0.354	0.164	2.03E-08	SMC4
HCK	1.11E-11	-0.002846	0.457	0.74	2.23E-08	SMC4
CDT1	1.14E-11	-0.0006344	0.576	0.959	2.29E-08	SMC4
BUB1	1.22E-11	-0.0009332	0.748	0.959	2.44E-08	SMC4
AL138899.1	1.25E-11	0.00352864	0.533	0.945	2.51E-08	SMC4
GGH	1.30E-11	-0.050868	0.613	0.315	2.59E-08	SMC4
RGS2	1.37E-11	0.08463345	0.55	0.959	2.74E-08	SMC4
DSP	1.40E-11	0.03596049	0.404	0.836	2.80E-08	SMC4
DIRAS3	1.45E-11	0.01466695	0.185	0.671	2.89E-08	SMC4
HAS1	1.49E-11	-7.92E-05	0.397	0.959	2.98E-08	SMC4
PTHLH	1.60E-11	0.01213109	0.51	0.932	3.20E-08	SMC4
NMB	1.62E-11	-0.0242859	0.781	0.986	3.25E-08	SMC4
ELL2	1.65E-11	0.13321134	0.361	0.932	3.31E-08	SMC4
VMO1	1.80E-11	-0.008774	0.646	1	3.60E-08	SMC4
KLRC2	1.81E-11	-0.0003997	0.033	0.137	3.62E-08	SMC4
RBM47	1.82E-11	-0.0098081	0.467	0.863	3.64E-08	SMC4
DEPDC1	2.45E-11	-0.0004047	0.073	0.397	4.89E-08	SMC4
ITGB2	2.51E-11	0.06889597	0.781	1	5.02E-08	SMC4
CXCL11	2.64E-11	0.0062807	0.129	0.014	5.28E-08	SMC4
C7	2.65E-11	-0.0230342	0.56	0.973	5.30E-08	SMC4
GDF10	2.66E-11	-0.0357599	0.785	1	5.32E-08	SMC4
FFAR3	2.73E-11	-0.0006193	0.474	0.959	5.45E-08	SMC4
CD3G	2.95E-11	0.10402885	0.795	1	5.89E-08	SMC4
IGF1	3.07E-11	0.00370135	0.434	0.178	6.14E-08	SMC4
TREM2	3.12E-11	-0.001175	0.626	0.808	6.24E-08	SMC4
PHACTR1	3.16E-11	0.02228795	0.48	0.932	6.32E-08	SMC4
RASGEF1B	4.00E-11	-0.0427623	0.758	0.959	8.00E-08	SMC4
PLLP	4.38E-11	0.01772786	0.619	0.37	8.76E-08	SMC4
NEFL	5.26E-11	-1.31E-05	0.063	0.342	1.05E-07	SMC4
ALDH1A3	6.08E-11	0.01280545	0.546	0.89	1.22E-07	SMC4
GABRA2	6.31E-11	6.21E-05	0.046	0.137	1.26E-07	SMC4
SLPI	6.37E-11	0.05174785	0.781	1	1.27E-07	SMC4
CD163	7.26E-11	-0.013461	0.57	0.767	1.45E-07	SMC4
BATF3	7.46E-11	-0.0269446	0.775	0.986	1.49E-07	SMC4
GFRA1	7.76E-11	0.00297831	0.344	0.096	1.55E-07	SMC4
APOC1	7.95E-11	0.03621503	0.536	0.849	1.59E-07	SMC4
TRBV14	8.35E-11	-7.10E-05	0.053	0.315	1.67E-07	SMC4
SLC16A10	8.87E-11	0.00341378	0.623	0.863	1.77E-07	SMC4
CD4	8.94E-11	0.02660076	0.623	0.932	1.79E-07	SMC4
KCNK15	9.47E-11	-0.0581808	0.705	0.959	1.89E-07	SMC4
CD69	9.76E-11	0.37429725	0.811	1	1.95E-07	SMC4
BMP7	9.79E-11	5.32E-06	0.063	0.329	1.96E-07	SMC4
MAD2L1	1.09E-10	0.02941356	0.652	0.452	2.18E-07	SMC4
IGKV3-20	1.16E-10	-0.0010645	0.775	0.973	2.32E-07	SMC4
SPOCK2	1.24E-10	0.04929868	0.795	1	2.48E-07	SMC4
MND1	1.28E-10	-0.0099438	0.305	0.712	2.57E-07	SMC4

AC104024.1	1.45E-10	-0.0003756	0.354	0.959	2.91E-07	SMC4
RBP7	1.60E-10	-0.022262	0.745	0.973	3.20E-07	SMC4
ADAP2	1.63E-10	0.018238	0.752	0.959	3.25E-07	SMC4
F5	1.70E-10	-0.0159547	0.01	0.644	3.40E-07	SMC4
FNDC1	1.72E-10	-0.0960471	0.788	1	3.44E-07	SMC4
CLLU1OS	1.85E-10	-2.20E-05	0.043	0.288	3.69E-07	SMC4
SLIT2	1.90E-10	0.10940236	0.619	0.288	3.79E-07	SMC4
MAFB	2.05E-10	-0.0056063	0.351	0.849	4.10E-07	SMC4
KPNA2	2.14E-10	0.01733995	0.566	0.274	4.28E-07	SMC4
PTPRE	2.19E-10	-0.0742523	0.695	0.959	4.38E-07	SMC4
RASD1	2.48E-10	0.02183403	0.238	0.63	4.95E-07	SMC4
TIGIT	2.52E-10	0.01291448	0.493	0.918	5.03E-07	SMC4
DSE	2.56E-10	-0.0302827	0.748	0.959	5.11E-07	SMC4
APOE	2.62E-10	1.01457693	0.579	0.986	5.23E-07	SMC4
SLC2A5	2.64E-10	-0.0019202	0.325	0.781	5.28E-07	SMC4
CKAP2L	2.78E-10	-1.48E-05	0.103	0.671	5.56E-07	SMC4
NGFR	2.81E-10	0.0128391	0.454	0.849	5.62E-07	SMC4
CCR6	3.04E-10	0.00645668	0.675	0.959	6.07E-07	SMC4
PROCR	3.08E-10	0.02120424	0.795	1	6.16E-07	SMC4
FCGR3A	3.25E-10	0.03264949	0.371	0.836	6.50E-07	SMC4
ITPKC	3.25E-10	0.01541765	0.656	0.973	6.50E-07	SMC4
SPINK1	3.44E-10	0.04141194	0.315	0.753	6.88E-07	SMC4
IBSP	3.60E-10	-0.0460165	0.742	0.973	7.19E-07	SMC4
NKG7	3.67E-10	0.14040765	0.791	0.986	7.33E-07	SMC4
NRN1	3.84E-10	0.12087301	0.265	0.849	7.68E-07	SMC4
EPCAM	4.45E-10	-3.10E-05	0.033	0.274	8.90E-07	SMC4
PLAUR	4.66E-10	-0.1553946	0.632	0.945	9.31E-07	SMC4
FCN3	4.76E-10	-7.37E-05	0.036	0.301	9.51E-07	SMC4
NCF4	5.55E-10	0.03042618	0.46	0.904	1.11E-06	SMC4
SMPX	6.41E-10	-0.0103884	0.679	0.877	1.28E-06	SMC4
PCLAF	6.51E-10	-0.0066801	0.467	0.753	1.30E-06	SMC4
HLA-DMA	7.12E-10	-0.1027619	0.599	0.932	1.42E-06	SMC4
CYYR1	7.12E-10	-0.0387414	0.692	0.945	1.42E-06	SMC4
PILRA	7.17E-10	-0.0003525	0.219	0.521	1.43E-06	SMC4
EDN1	7.55E-10	0.09542472	0.517	0.274	1.51E-06	SMC4
KLRB1	7.56E-10	0.04475585	0.619	0.986	1.51E-06	SMC4
HAMP	7.70E-10	-0.0002209	0.089	0.589	1.54E-06	SMC4
CLEC5A	7.85E-10	0.00549245	0.017	0.644	1.57E-06	SMC4
THEMIS2	7.89E-10	-0.0036779	0.659	0.945	1.58E-06	SMC4
DIRC3	7.95E-10	0.00130837	0.252	0.123	1.59E-06	SMC4
FJX1	9.08E-10	0.03395513	0.735	0.63	1.82E-06	SMC4
DUSP5	9.19E-10	-0.008077	0.798	1	1.84E-06	SMC4
TCF21	9.25E-10	0.04998223	0.656	0.356	1.85E-06	SMC4
TNFSF9	9.25E-10	-0.000469	0.695	0.986	1.85E-06	SMC4
C1orf162	9.54E-10	0.0785521	0.692	0.918	1.91E-06	SMC4
GZMA	9.54E-10	0.09968356	0.788	1	1.91E-06	SMC4
MTUS1	9.76E-10	-0.0801107	0.291	0.945	1.95E-06	SMC4
GRP	1.01E-09	-0.0020993	0.666	0.959	2.02E-06	SMC4
IGHG2	1.01E-09	-0.0115325	0.805	1	2.02E-06	SMC4
CD3E	1.05E-09	0.06002079	0.785	1	2.10E-06	SMC4
GJB2	1.26E-09	0.00015925	0.046	0.123	2.51E-06	SMC4
ACE	1.29E-09	-0.0058813	0.162	0.301	2.59E-06	SMC4
CDCA3	1.34E-09	0.0140547	0.712	1	2.68E-06	SMC4

CYBB	1.37E-09	0.02479224	0.493	0.712	2.74E-06	SMC4
RSPO2	1.42E-09	-0.0206656	0.646	0.945	2.84E-06	SMC4
ADAMDEC1	1.42E-09	-0.0004673	0.692	0.945	2.85E-06	SMC4
RGS1	1.55E-09	0.13258116	0.762	1	3.10E-06	SMC4
SLAMF1	1.57E-09	0.00015558	0.781	1	3.15E-06	SMC4
CD163L1	1.60E-09	-0.0577833	0.248	0.685	3.20E-06	SMC4
BCL2A1	1.78E-09	-0.0094061	0.483	0.822	3.55E-06	SMC4
AIF1	1.80E-09	0.11919317	0.689	0.959	3.61E-06	SMC4
EFNB2	1.85E-09	-0.0560106	0.228	0.753	3.71E-06	SMC4
ESM1	2.27E-09	0.01023756	0.566	0.959	4.55E-06	SMC4
CXCL13	2.49E-09	0.00225558	0.166	0.247	4.98E-06	SMC4
DTL	2.61E-09	5.36E-07	0.06	0.301	5.23E-06	SMC4
COLEC12	2.73E-09	0.04479225	0.742	0.986	5.46E-06	SMC4
TCL1A	3.08E-09	0.00017804	0.619	0.959	6.16E-06	SMC4
HIST1H1B	3.14E-09	-0.000218	0.172	0.74	6.28E-06	SMC4
SOX18	3.19E-09	-0.0288476	0.57	0.89	6.38E-06	SMC4
SERPINE2	3.43E-09	0.11324687	0.788	0.973	6.86E-06	SMC4
GZMK	3.48E-09	0.08775107	0.808	1	6.96E-06	SMC4
BMP2	3.60E-09	-0.0028078	0.341	0.767	7.21E-06	SMC4
LCP1	3.76E-09	0.1391505	0.788	1	7.52E-06	SMC4
EZH2	3.99E-09	0.00249708	0.656	0.904	7.99E-06	SMC4
HLA-DQB1	4.30E-09	-0.1278263	0.772	0.973	8.60E-06	SMC4
NRG1	4.33E-09	0.00698017	0.136	0.644	8.65E-06	SMC4
TNFSF14	4.54E-09	0.0178736	0.758	1	9.07E-06	SMC4
IGKV1-5	4.83E-09	-0.0001927	0.046	0.329	9.66E-06	SMC4
PLK1	5.31E-09	-0.0016644	0.205	0.233	1.06E-05	SMC4
LINC01094	5.78E-09	-0.0005122	0.566	0.685	1.16E-05	SMC4
STMN2	5.78E-09	-0.0001813	0.702	0.959	1.16E-05	SMC4
EMX2	5.84E-09	0.11080295	0.255	0.753	1.17E-05	SMC4
GLRX	6.00E-09	0.01803021	0.682	1	1.20E-05	SMC4
TNFSF10	6.13E-09	-0.0602949	0.798	1	1.23E-05	SMC4
LTC4S	6.49E-09	-0.0151528	0.629	0.904	1.30E-05	SMC4
HTR2B	7.00E-09	0.08615785	0.305	0.753	1.40E-05	SMC4
HTRA3	7.02E-09	0.01401459	0.692	0.644	1.40E-05	SMC4
EMCN	7.40E-09	-0.0185299	0.646	0.849	1.48E-05	SMC4
PKMYT1	7.59E-09	0.000932	0.381	0.945	1.52E-05	SMC4
HLA-DMB	7.76E-09	0.04338974	0.573	0.973	1.55E-05	SMC4
CYP26B1	7.95E-09	-0.0969747	0.745	0.616	1.59E-05	SMC4
AL078590.2	8.18E-09	-0.0002355	0.47	0.959	1.64E-05	SMC4
JAML	8.66E-09	0.00843957	0.318	0.699	1.73E-05	SMC4
ADH1C	9.71E-09	0.00137981	0.656	0.836	1.94E-05	SMC4
PART1	1.05E-08	0.02157398	0.175	0.507	2.11E-05	SMC4
CYP4B1	1.18E-08	0.00013824	0.407	0.932	2.36E-05	SMC4
CFP	1.18E-08	-0.0114872	0.225	0.274	2.36E-05	SMC4
TGFBI	1.19E-08	-0.068284	0.394	0.849	2.37E-05	SMC4
NR4A2	1.19E-08	0.03772784	0.705	0.986	2.37E-05	SMC4
RAB3C	1.19E-08	0.00135822	0.434	0.959	2.39E-05	SMC4
EGFL7	1.25E-08	-0.0508504	0.454	0.671	2.50E-05	SMC4
MIR155HG	1.44E-08	0.00112232	0.679	0.986	2.89E-05	SMC4
PPP1R1A	1.62E-08	0.04861722	0.096	0.37	3.24E-05	SMC4
EDNRA	1.64E-08	-0.0738579	0.768	0.959	3.28E-05	SMC4
LGALS9	1.65E-08	-0.0171994	0.646	0.932	3.29E-05	SMC4
B3GNT5	1.69E-08	0.02669486	0.268	0.644	3.38E-05	SMC4

PDGFB	1.76E-08	-0.0027272	0.566	0.877	3.52E-05	SMC4
CD7	1.80E-08	0.02040147	0.778	1	3.61E-05	SMC4
SLC7A2	1.87E-08	0.04896804	0.811	0.973	3.75E-05	SMC4
KCNN4	1.95E-08	0.01679062	0.765	0.945	3.89E-05	SMC4
MFAP5	2.01E-08	0.3024697	0.556	0.315	4.02E-05	SMC4
HS3ST1	2.09E-08	-0.0041644	0.295	0.658	4.17E-05	SMC4
TNNI2	2.15E-08	-0.0005944	0.752	0.986	4.30E-05	SMC4
TCF15	2.18E-08	-0.0005489	0.629	0.945	4.35E-05	SMC4
RNASE6	2.19E-08	0.02380513	0.464	0.822	4.39E-05	SMC4
TNC	2.39E-08	-0.1921291	0.798	1	4.78E-05	SMC4
RASSF9	2.47E-08	-0.0392805	0.5	0.849	4.95E-05	SMC4
PELI1	2.69E-08	0.00626007	0.52	0.822	5.38E-05	SMC4
CENPK	2.76E-08	0.00727225	0.457	0.192	5.52E-05	SMC4
SLC9B2	2.95E-08	0.02602053	0.212	0.63	5.91E-05	SMC4
CCL5	3.16E-08	0.27566758	0.828	1	6.31E-05	SMC4
MX2	3.16E-08	-0.0043358	0.685	0.959	6.33E-05	SMC4
GRASP	3.26E-08	-0.0424986	0.536	0.808	6.52E-05	SMC4
LINC01781	3.31E-08	-0.0008992	0.765	0.973	6.61E-05	SMC4
FCGR2A	3.36E-08	0.02736571	0.397	0.918	6.72E-05	SMC4
ALOX5AP	3.48E-08	0.07463366	0.758	1	6.97E-05	SMC4
LINC00996	3.61E-08	-1.32E-05	0.06	0.151	7.22E-05	SMC4
SERPINB2	3.69E-08	-0.0042604	0.132	0.63	7.38E-05	SMC4
LNP1	4.04E-08	0.1988824	0.719	0.438	8.07E-05	SMC4
PLA2G5	4.54E-08	0.08259639	0.43	0.89	9.08E-05	SMC4
EGR2	4.73E-08	-0.0063405	0.652	0.959	9.46E-05	SMC4
KRT14	4.85E-08	0.29519686	0.526	0.342	9.69E-05	SMC4
ICAM2	4.96E-08	0.10929572	0.728	0.616	9.92E-05	SMC4
HCAR2	5.29E-08	0.0140799	0.126	0.288	0.00010589	SMC4
CHI3L1	5.35E-08	-0.0026868	0.778	0.973	0.0001071	SMC4
HIST1H1A	5.74E-08	0.07738346	0.576	0.315	0.00011483	SMC4
PTGER2	5.77E-08	0.01943284	0.288	0.671	0.00011537	SMC4
CYTIP	6.02E-08	0.1612818	0.778	1	0.00012036	SMC4
MARCO	6.09E-08	0.01357239	0.447	0.644	0.00012179	SMC4
TNFSF11	6.30E-08	-0.0061762	0.758	0.973	0.00012602	SMC4
SMOC1	6.42E-08	-0.0066345	0.788	1	0.00012838	SMC4
SELP	6.51E-08	-0.0099563	0.662	0.932	0.00013026	SMC4
VWA1	6.61E-08	0.08396895	0.755	0.658	0.00013225	SMC4
IFI44L	6.92E-08	0.00426039	0.798	1	0.00013831	SMC4
IL1B	8.14E-08	-0.0030807	0.175	0.507	0.00016274	SMC4
CHCHD6	8.53E-08	0.04392133	0.464	0.89	0.0001706	SMC4
IGLC7	8.83E-08	-0.0011148	0.457	0.822	0.00017651	SMC4
S100A8	9.36E-08	0.0501101	0.285	0.63	0.00018711	SMC4
HOPX	9.64E-08	0.00830113	0.825	1	0.00019276	SMC4
CORO1A	9.75E-08	0.08680038	0.712	0.973	0.00019496	SMC4
G0S2	1.09E-07	0.07373914	0.54	0.863	0.00021726	SMC4
POU2F2	1.09E-07	0.03272426	0.576	0.37	0.00021744	SMC4
SOX17	1.20E-07	-0.0259281	0.772	0.959	0.00023912	SMC4
IRF4	1.22E-07	0.00751984	0.768	0.986	0.00024339	SMC4
MMP1	1.25E-07	0.00144691	0.364	0.918	0.00024947	SMC4
NCR3	1.29E-07	0.00269116	0.652	0.945	0.00025777	SMC4
TSPAN8	1.41E-07	0.0972764	0.728	0.479	0.00028127	SMC4
NLRP3	1.44E-07	0.00808625	0.609	0.945	0.00028854	SMC4
SFRP5	1.52E-07	-0.0198188	0.318	0.712	0.00030402	SMC4

CXCL8	1.62E-07	0.05581541	0.43	0.808	0.0003248	SMC4
CHI3L2	1.81E-07	0.00258798	0.589	0.973	0.00036127	SMC4
ADM5	1.84E-07	-0.0099781	0.162	0.644	0.00036733	SMC4
DDIT4	1.85E-07	0.21426948	0.788	0.986	0.00037095	SMC4
TBXAS1	1.89E-07	-0.0168305	0.636	0.836	0.00037846	SMC4
PLD4	1.92E-07	0.00838418	0.01	0.137	0.00038467	SMC4
AC106028.4	1.98E-07	-9.08E-05	0.036	0.164	0.00039641	SMC4
HS3ST2	2.03E-07	0.00037126	0.172	0.014	0.00040572	SMC4
RHEX	2.10E-07	-0.0004258	0.45	0.89	0.0004202	SMC4
STEAP1	2.12E-07	-0.0188217	0.781	0.959	0.000424	SMC4
MAL	2.17E-07	-0.0023159	0.43	0.89	0.00043332	SMC4
ERN1	2.26E-07	0.00091596	0.291	0.671	0.00045109	SMC4
VWF	2.31E-07	-0.1483878	0.272	0.589	0.00046268	SMC4
ENHO	2.46E-07	0.00427187	0.487	0.877	0.00049287	SMC4
CP	2.54E-07	-0.2771215	0.821	1	0.00050809	SMC4
LEFTY2	2.74E-07	0.10761187	0.252	0.658	0.00054724	SMC4
GAPT	2.88E-07	0.00564361	0.099	0.616	0.00057605	SMC4
BIRC3	2.99E-07	0.17333317	0.758	0.945	0.00059866	SMC4
IGKC	3.01E-07	-0.0295825	0.99	1	0.00060285	SMC4
TSPAN7	3.03E-07	-0.1292063	0.331	0.685	0.00060612	SMC4
SEMA3B	3.07E-07	-0.050182	0.364	0.726	0.00061413	SMC4
DKK2	3.12E-07	-0.0069071	0.275	0.534	0.00062307	SMC4
ANGPTL5	3.14E-07	-0.0016333	0.656	0.945	0.00062893	SMC4
DERL3	3.30E-07	0.00328719	0.666	0.932	0.00066098	SMC4
SPTSSB	3.39E-07	-0.0058984	0.719	0.945	0.00067866	SMC4
MATN4	3.45E-07	0.00631316	0.752	0.986	0.00068997	SMC4
CLEC4C	4.05E-07	0.00409766	0.017	0.151	0.00081072	SMC4
CLEC11A	4.20E-07	-0.0504295	0.457	0.836	0.00084049	SMC4
TMIGD3	4.21E-07	0.00695236	0.129	0.329	0.0008418	SMC4
FAP	4.39E-07	0.03594298	0.864	1	0.00087783	SMC4
CTSC	4.52E-07	-0.0607393	0.858	0.986	0.00090353	SMC4
CDCA5	4.73E-07	-0.0004139	0.659	0.959	0.00094614	SMC4
SLCO2A1	4.84E-07	0.00136827	0.702	0.945	0.00096845	SMC4
PECAM1	4.85E-07	-0.1283338	0.583	0.863	0.00096955	SMC4
LINC02207	4.95E-07	-4.58E-05	0.03	0.219	0.00099011	SMC4
HMGA1	4.99E-07	0.03749632	0.305	0.699	0.00099756	SMC4
SPIB	5.67E-07	0.01519945	0.517	0.356	0.00113311	SMC4
CEMIP	6.23E-07	-0.0733408	0.775	0.986	0.00124603	SMC4
CCR7	6.39E-07	0.04168622	0.675	1	0.00127749	SMC4
CXCL3	6.92E-07	0.03487325	0.242	0.616	0.00138357	SMC4
TCIM	7.12E-07	0.12500378	0.669	0.932	0.00142371	SMC4
FCRLA	7.39E-07	-0.0001287	0.613	0.945	0.00147713	SMC4
IL2	7.46E-07	0.00561186	0.685	0.973	0.001492	SMC4
C5AR1	7.69E-07	-0.1029791	0.52	0.822	0.00153896	SMC4
TPX2	7.74E-07	0.04051641	0.351	0.699	0.00154819	SMC4
EGLN3	7.77E-07	-0.0381396	0.781	0.986	0.00155349	SMC4
KANK3	8.38E-07	0.01531426	0.384	0.699	0.00167575	SMC4
PCDHA12	8.57E-07	-0.0004949	0.715	0.945	0.00171402	SMC4
MCTP1	9.31E-07	0.00282575	0.447	0.74	0.00186247	SMC4
MSMP	9.55E-07	0.01374343	0.732	0.438	0.00190971	SMC4
ADM	1.03E-06	0.02687973	0.619	0.945	0.00206628	SMC4
CD27	1.08E-06	-0.0047141	0.334	0.616	0.00216548	SMC4
IQCA1	1.09E-06	-9.28E-05	0.593	0.849	0.00217027	SMC4

IL2RB	1.10E-06	0.00491266	0.765	1	0.00219597	SMC4
IGKV3-11	1.12E-06	-0.0003484	0.285	0.671	0.00223684	SMC4
AC084871.2	1.15E-06	-0.0009952	0.017	0.151	0.00230906	SMC4
SLAMF8	1.19E-06	0.02560651	0.765	0.986	0.00237921	SMC4
COL15A1	1.22E-06	-0.2841417	0.848	1	0.00243107	SMC4
AK5	1.22E-06	-0.0248196	0.526	0.452	0.00243884	SMC4
PLA2G7	1.26E-06	-0.0103349	0.301	0.699	0.00251525	SMC4
CFD	1.26E-06	0.01440949	0.493	0.329	0.00252949	SMC4
NRARP	1.33E-06	-0.0322266	0.384	0.753	0.00265502	SMC4
CD300LG	1.33E-06	-0.0012626	0.02	0.151	0.00266266	SMC4
CREB5	1.35E-06	-0.1393595	0.623	0.329	0.00269164	SMC4
PLTP	1.44E-06	0.12390895	0.381	0.863	0.00288978	SMC4
SFRP1	1.45E-06	0.30657612	0.768	0.74	0.00289073	SMC4
MEOX1	1.52E-06	-0.0225324	0.358	0.329	0.00304191	SMC4
OGN	1.54E-06	0.62248383	0.983	1	0.00307778	SMC4
ASPN	1.55E-06	0.74417147	0.96	0.959	0.00309624	SMC4
CLEC4A	1.56E-06	-0.001928	0.692	0.918	0.00312161	SMC4
DPT	1.62E-06	-0.1028129	0.841	1	0.00324534	SMC4
AC007406.3	1.63E-06	-0.0023998	0.656	0.959	0.00325752	SMC4
RHOH	1.64E-06	0.05405437	0.778	1	0.00327912	SMC4
SELE	1.66E-06	-0.2243218	0.533	0.904	0.00332515	SMC4
ZNF683	1.77E-06	-0.003575	0.553	0.699	0.00354593	SMC4
OLFML3	1.83E-06	0.09791517	0.844	0.671	0.00366967	SMC4
JSRP1	1.92E-06	-0.0023845	0.589	0.863	0.00383344	SMC4
HIST1H1C	2.05E-06	0.16294954	0.652	0.37	0.00409498	SMC4
MMP7	2.06E-06	-0.0033428	0.265	0.63	0.00411452	SMC4
AL157895.1	2.07E-06	-0.0009698	0.755	0.959	0.00414778	SMC4
CXCL10	2.19E-06	-0.0103452	0.523	0.74	0.00437219	SMC4
ACOXL	2.31E-06	-0.0002794	0.738	0.959	0.00462907	SMC4
ULBP1	2.46E-06	-0.0001929	0.639	0.959	0.00492836	SMC4
SAMD11	2.49E-06	-0.0382207	0.417	0.301	0.00497861	SMC4
LYVE1	2.49E-06	-0.1830892	0.583	0.74	0.00498098	SMC4
LMNB1	2.51E-06	0.01766942	0.341	0.151	0.00502617	SMC4
PLAU	2.52E-06	0.0068655	0.649	0.863	0.00503909	SMC4
LMCD1	2.66E-06	0.69460722	0.894	0.863	0.00531997	SMC4
LYN	2.71E-06	-0.1792363	0.56	0.753	0.00541063	SMC4
BMP3	2.77E-06	-0.0062765	0.765	1	0.00553694	SMC4
CCDC102B	2.86E-06	0.04804429	0.818	0.959	0.00572702	SMC4
IFNG	3.04E-06	0.01540413	0.781	1	0.00608631	SMC4
SGCG	3.34E-06	-0.0225642	0.725	0.521	0.00668125	SMC4
SCIN	3.41E-06	0.0613997	0.715	0.562	0.0068209	SMC4
CD8A	3.49E-06	0.03044771	0.762	1	0.00697691	SMC4
NRP1	3.63E-06	-0.1998033	0.811	0.959	0.00725356	SMC4
IL18	3.65E-06	0.00847556	0.172	0.329	0.00729726	SMC4
SMIM25	3.68E-06	-0.0023201	0.05	0.589	0.00736196	SMC4
SCG3	3.71E-06	-0.0001221	0.609	0.959	0.00742458	SMC4
POU3F4	3.82E-06	8.97E-05	0.073	0.192	0.00764179	SMC4
GAL	3.83E-06	-3.23E-05	0.202	0.685	0.00766899	SMC4
FAIM2	3.88E-06	-0.0043462	0.48	0.877	0.00776135	SMC4
UCP2	3.94E-06	0.02078538	0.48	0.822	0.00788399	SMC4
CA2	4.16E-06	-0.0003012	0.576	0.849	0.00831273	SMC4
APOBEC3A	4.16E-06	-0.0020011	0.192	0.274	0.00832466	SMC4
APOD	4.17E-06	0.0093725	0.748	0.603	0.00833923	SMC4

ECSCR	4.19E-06	-0.0240942	0.348	0.356	0.00837269	SMC4
CASQ2	4.28E-06	0.0131798	0.725	0.89	0.00856101	SMC4
EPB41L3	4.33E-06	-0.0212242	0.106	0.616	0.00865197	SMC4
TRBC1	4.43E-06	0.16091853	0.692	0.945	0.00885223	SMC4
FAM162B	4.52E-06	0.01056176	0.609	0.904	0.00904511	SMC4
THBD	4.53E-06	-0.1934423	0.583	0.89	0.00905529	SMC4
MIA	4.54E-06	-0.0002535	0.033	0.178	0.00907001	SMC4
IGHV3-23	4.55E-06	2.81E-06	0.043	0.205	0.00909926	SMC4
NUF2	4.58E-06	0.00183516	0.219	0.205	0.00915203	SMC4
GATA2	4.71E-06	0.00551037	0.268	0.233	0.0094183	SMC4
CD24	4.76E-06	0.00554797	0.623	0.863	0.00952751	SMC4
NRGN	4.77E-06	-0.080584	0.748	0.986	0.00953723	SMC4
IGHA2	5.22E-06	-0.0050242	0.748	0.986	0.01044604	SMC4
LST1	5.34E-06	0.04259701	0.51	0.74	0.01068852	SMC4
HLA-DOB	5.34E-06	0.00500629	0.758	0.973	0.01068864	SMC4
TOP2A	5.38E-06	0.00144949	0.467	0.959	0.01076334	SMC4
CENPE	5.41E-06	-0.0039602	0.656	0.767	0.01081196	SMC4
GPR183	5.45E-06	0.13600308	0.738	1	0.01090049	SMC4
MNDA	5.66E-06	0.13961947	0.126	0.493	0.01131768	SMC4
FHL1	6.66E-06	0.53487968	0.993	0.986	0.01332991	SMC4
COL11A1	6.86E-06	-0.0108136	0.523	0.781	0.01372129	SMC4
HIGD1B	7.12E-06	0.00408285	0.689	0.959	0.01423138	SMC4
TNFAIP2	7.28E-06	-0.000512	0.672	0.863	0.01456936	SMC4
AL583785.1	7.43E-06	0.00023399	0.732	0.877	0.01486111	SMC4
BMPER	7.44E-06	-0.0491271	0.692	0.89	0.01487174	SMC4
PDLIM4	7.49E-06	-0.24646	0.772	0.945	0.01497274	SMC4
AOAH	7.58E-06	0.06787534	0.526	0.822	0.01516621	SMC4
ARC	7.82E-06	0.02290065	0.497	0.397	0.01564722	SMC4
CYGB	8.13E-06	0.01169565	0.715	0.548	0.01626974	SMC4
HLA-DQA1	8.28E-06	-0.1515799	0.732	0.959	0.01655276	SMC4
JCHAIN	8.37E-06	0.0797596	0.791	1	0.01674778	SMC4
LINC02345	8.41E-06	0.00036232	0.182	0.671	0.01681298	SMC4
ISG20	8.47E-06	0.07927296	0.738	0.959	0.01693457	SMC4
CR1	8.69E-06	-4.96E-06	0.219	0.712	0.01738479	SMC4
CD79A	8.85E-06	-0.0042312	0.755	0.986	0.01770892	SMC4
PTP4A3	9.00E-06	-0.0310906	0.735	0.932	0.01800102	SMC4
WNT2	9.27E-06	0.00489185	0.149	0.562	0.01854039	SMC4
AC026369.3	9.52E-06	-5.34E-05	0.053	0.219	0.01904284	SMC4
DCN	9.56E-06	-0.1524078	0.689	1	0.01912472	SMC4
TSPAN33	9.74E-06	0.01396756	0.775	0.781	0.01947729	SMC4
SLC8A1	9.87E-06	-0.2118805	0.679	0.452	0.01973244	SMC4
NPPC	9.98E-06	-0.0018404	0.609	0.959	0.01995572	SMC4
LINC00924	1.00E-05	-0.0319913	0.368	0.74	0.02008723	SMC4
CCL4L2	1.06E-05	0.08670008	0.712	0.932	0.02112569	SMC4
CLEC4E	1.07E-05	0.003423	0.026	0.534	0.02149151	SMC4
AL954642.1	1.09E-05	0.00042752	0.129	0.014	0.02174002	SMC4
BHLHE41	1.09E-05	-0.0174733	0.606	0.329	0.02185508	SMC4
CDC20	1.11E-05	0.00122856	0.222	0.301	0.02223403	SMC4
ANKRD22	1.13E-05	0.01484174	0.043	0.151	0.02266286	SMC4
CDA	1.14E-05	0.00591563	0.493	0.699	0.02281007	SMC4
PDPN	1.14E-05	-0.0074057	0.768	0.986	0.02283067	SMC4
TTK	1.14E-05	-0.0002571	0.669	0.904	0.02284127	SMC4
SPC24	1.15E-05	-0.006832	0.192	0.548	0.02306375	SMC4

CAPG	1.16E-05	0.12746645	0.629	0.959	0.02312201	SMC4
CD300E	1.18E-05	-0.0016558	0.086	0.562	0.02357427	SMC4
AC073352.2	1.22E-05	0.00052019	0.394	0.781	0.02434614	SMC4
KNL1	1.27E-05	-0.0060604	0.603	0.959	0.0253458	SMC4
CDH5	1.27E-05	-0.0081196	0.636	0.767	0.02542708	SMC4
AKR1C1	1.28E-05	0.08950563	0.503	0.37	0.02555909	SMC4
BTNL9	1.34E-05	-0.0001334	0.311	0.658	0.02679673	SMC4
STEAP4	1.34E-05	-0.0838672	0.818	1	0.02681209	SMC4
AC093895.1	1.38E-05	0.00058815	0.023	0.562	0.02757774	SMC4
GPX3	1.39E-05	0.00830995	0.868	1	0.02785819	SMC4
CCL4	1.40E-05	0.66517436	0.861	1	0.02791544	SMC4
EBI3	1.42E-05	-0.0017421	0.755	1	0.02839318	SMC4
DSC2	1.45E-05	-0.0026716	0.705	0.877	0.02900433	SMC4
ABRA	1.48E-05	0.0036501	0.142	0.096	0.0295038	SMC4
RBP4	1.51E-05	0.01313018	0.437	0.315	0.03015768	SMC4
QPCT	1.64E-05	0.02696427	0.639	0.411	0.03272015	SMC4
C1QC	1.70E-05	0.00473411	0.43	0.74	0.03390016	SMC4
MS4A6A	1.78E-05	0.05951267	0.513	0.836	0.03560113	SMC4
IL13RA2	1.79E-05	0.08284831	0.732	0.479	0.03579022	SMC4
CDH6	1.80E-05	0.00831821	0.497	0.356	0.03590939	SMC4
ADAMTS5	1.81E-05	0.01219381	0.46	0.753	0.03610498	SMC4
SPP1	1.82E-05	0.06233813	0.682	0.932	0.03643588	SMC4
C3AR1	1.85E-05	0.00852023	0.526	0.658	0.03690411	SMC4
RAB20	1.86E-05	0.0687692	0.46	0.315	0.03723485	SMC4
AP1S2	1.91E-05	-0.3082101	0.47	0.904	0.03816658	SMC4
CD40LG	2.08E-05	0.02819011	0.742	0.986	0.04155238	SMC4
CXCL1	2.21E-05	0.0112806	0.51	0.781	0.04410871	SMC4
CRISPLD1	2.21E-05	0.0891863	0.725	0.959	0.04418622	SMC4
CLDN14	2.34E-05	-0.001803	0.103	0.452	0.04687261	SMC4
MRAS	2.37E-05	0.11161406	0.606	0.397	0.04736457	SMC4
PAPPA	2.41E-05	0.07122832	0.268	0.644	0.04816177	SMC4
CXCR6	9.55E-07	-0.0210872	0.431	1	0.00190912	SMC5
NRG1	4.37E-06	-0.2440156	0.411	1	0.00873509	SMC5
FCN1	5.20E-06	-1.6914941	0.569	1	0.01040065	SMC5
S100A9	6.89E-06	-1.9816492	0.792	1	0.01377365	SMC5
LINC02185	7.84E-06	-0.2514739	0.396	1	0.01568157	SMC5
AL954642.1	9.41E-06	-0.0531743	0.457	1	0.01881007	SMC5
CTSS	1.20E-05	-1.1984707	0.985	1	0.02393761	SMC5
CSTA	1.41E-05	-1.0262689	0.553	1	0.02827468	SMC5
LILRA2	1.60E-05	-0.7930458	0.289	0.917	0.0319417	SMC5
S100A8	1.60E-05	-2.1954682	0.503	0.917	0.03202921	SMC5
MELK	1.62E-05	-0.010686	0.152	0.75	0.03231037	SMC5
LINC01857	1.68E-05	-0.0147583	0.264	0.917	0.03359835	SMC5
S100A12	1.85E-05	-1.3505592	0.35	0.917	0.03698381	SMC5

Table S10. Predicted transcription factors activated in smooth muscle cell (SMC) 5 subtype

ARID3A	ELK4	HLTF	MTF1	RFX5	TFAP2C	ZNF354C
ARNT	EMX2	HMGA1	MXD1	RFXANK	TFDP2	ZNF419
ARNTL	EOMES	HOXA1	MXI1	RFXAP	TFE3	ZNF43
ATF1	EP300	HOXA2	MYB	RUNX1	THAP11	ZNF441
ATF3	ERF	HOXB2	MYBL2	RUNX2	THRA	ZNF444
ATF5	ERG	HOXB3	MYC	RUNX3	THRB	ZNF449
BACH1	ETS1	HSF1	MZF1	RXRA	UBE2K	ZNF502
BACH2	ETS2	IKZF2	NFATC1	RXRB	USF1	ZNF548
BCL11A	ETV1	ILF2	NFATC2	RXRG	VDR	ZNF567
BCLAF1	ETV2	ING4	NFATC3	SHOX2	XBP1	ZNF57
BHLHE40	ETV3	IRF1	NFE2	SMAD4	YY1	ZNF571
BHLHE41	ETV5	IRF4	NFE2L1	SOX15	ZBTB11	ZNF577
BPTF	ETV6	IRF6	NFE2L2	SOX17	ZBTB14	ZNF580
CEBPA	ETV7	IRF7	NFIA	SOX4	ZBTB20	ZNF582
CEBPB	EVX1	IRF8	NFIC	SOX8	ZBTB46	ZNF594
CEBPD	FLI1	IRF9	NFKB1	SOX9	ZBTB7A	ZNF610
CHURC1	FOS	JUN	NFKB2	SP1	ZEB1	ZNF627
CLOCK	FOSB	JUNB	NKX3-1	SP3	ZFP1	ZNF641
CREB1	FOSL1	JUND	NR2C2	SP4	ZFP14	ZNF667
CREB3	FOSL2	KLF10	NR2F1	SPI1	ZFP37	ZNF672
CREB3L1	FOXC1	KLF12	NR2F6	SPIB	ZFP90	ZNF682
CREB3L2	FOXJ2	KLF13	NR3C1	SREBF1	ZFP91	ZNF708
CREB5	FOXJ3	KLF4	PAX6	SREBF2	ZFX	ZNF718
CREM	FOXO1	KLF5	PBX1	STAT1	ZKSCAN8	ZNF74
CTCF	FOXO3	KLF6	PBX4	STAT2	ZNF121	ZNF740
CUX1	GABPA	KLF9	PDLIM5	STAT3	ZNF148	ZNF770
DBP	GABPB1	LTF	PLAGL1	STAT5A	ZNF160	ZNF787
DLX2	GATA2	MAF	POU2F1	STAT6	ZNF18	ZNF813
DLX3	GATA3	MAFB	POU2F2	TAL1	ZNF195	ZNF84
DLX5	GATA6	MAFF	POU3F1	TBP	ZNF200	ZNF846
E2F1	GFI1	MAFG	PPARG	TBX18	ZNF202	ZNF880
E2F3	GLI3	MAFK	PRDM1	TBX21	ZNF205	ZSCAN12
E2F6	GLIS3	MAZ	PRDM5	TBX3	ZNF230	ZSCAN31
EBF1	GRHL2	MBD2	PRRX2	TCF12	ZNF236	ZXDB
EGR1	GTF2IRD1	MEIS1	PURA	TCF3	ZNF253	
ELF1	HAND2	MEOX1	RARA	TCF4	ZNF287	
ELF2	HDAC2	MESP1	RARB	TCF7L1	ZNF296	
ELF4	HDX	MGA	RARG	TEAD2	ZNF329	
ELK1	HEYL	MITF	RBAK	TEAD4	ZNF343	
ELK3	HIVEP2	MLX	RELA	TFAP2A	ZNF354B	
